# Global, regional, and national burden of congenital heart disease, 1990–2017: a systematic analysis for the Global Burden of Disease Study 2017

**DOI:** 10.1016/S2352-4642(19)30402-X

**Published:** 2020-03

**Authors:** GBD 2017 Congenital Heart Disease Collaborators, Meghan S Zimmerman, Alison Grace Carswell Smith, Craig A Sable, Michelle Marie Echko, Lauren B Wilner, Helen Elizabeth Olsen, Hagos Tasew Atalay, Ashish Awasthi, Zulfiqar A Bhutta, Jackie LeeAnne Boucher, Franz Castro, Paolo Angelo Cortesi, Manisha Dubey, Florian Fischer, Samer Hamidi, Simon I Hay, Chi Linh Hoang, Christopher Hugo-Hamman, Kathy J Jenkins, Anita Kar, Ibrahim A Khalil, Raman Krishna Kumar, Gene F Kwan, Desalegn Tadese Mengistu, Ali H Mokdad, Mohsen Naghavi, Lemma Negesa, Ionut Negoi, Ruxandra Irina Negoi, Cuong Tat Nguyen, Huong Lan Thi Nguyen, Long Hoang Nguyen, Son Hoang Nguyen, Trang Huyen Nguyen, Molly R Nixon, Jean Jacques Noubiap, Shanti Patel, Emmanuel K Peprah, Robert C Reiner, Gregory A Roth, Mohamad-Hani Temsah, Marcos Roberto Tovani-Palone, Jeffrey A Towbin, Bach Xuan Tran, Tung Thanh Tran, Nu Thi Truong, Theo Vos, Kia Vosoughi, Robert G Weintraub, Kidu Gidey Weldegwergs, Zoubida Zaidi, Bistra Zheleva, Liesl Zuhlke, Christopher J L Murray, Gerard R Martin, Nicholas J Kassebaum

## Abstract

**Background:**

Previous congenital heart disease estimates came from few data sources, were geographically narrow, and did not evaluate congenital heart disease throughout the life course. Completed as part of the Global Burden of Diseases, Injuries, and Risk Factors Study 2017, this study aimed to provide comprehensive estimates of congenital heart disease mortality, prevalence, and disability by age for 195 countries and territories from 1990 to 2017.

**Methods:**

Mortality estimates were generated for aggregate congenital heart disease and non-fatal estimates for five subcategories (single ventricle and single ventricle pathway congenital heart anomalies; severe congenital heart anomalies excluding single ventricle heart defects; critical malformations of great vessels, congenital valvular heart disease, and patent ductus arteriosus; ventricular septal defect and atrial septal defect; and other congenital heart anomalies), for 1990 through to 2017. All available global data were systematically analysed to generate congenital heart disease mortality estimates (using Cause of Death Ensemble modelling) and prevalence estimates (DisMod-MR 2·1). Systematic literature reviews of all types of congenital anomalies to capture information on prevalence, associated mortality, and long-term health outcomes on congenital heart disease informed subsequent disability estimates.

**Findings:**

Congenital heart disease caused 261 247 deaths (95% uncertainty interval 216 567–308 159) globally in 2017, a 34·5% decline from 1990, with 180 624 deaths (146 825–214 178) being among infants (aged <1 years). Congenital heart disease mortality rates declined with increasing Socio-demographic Index (SDI); most deaths occurred in countries in the low and low-middle SDI quintiles. The prevalence rates of congenital heart disease at birth changed little temporally or by SDI, resulting in 11 998 283 (10 958 658–13 123 888) people living with congenital heart disease globally, an 18·7% increase from 1990 to 2017, and causing a total of 589 479 (287 200–973 359) years lived with disability.

**Interpretation:**

Congenital heart disease is a large, rapidly emerging global problem in child health. Without the ability to substantially alter the prevalence of congenital heart disease, interventions and resources must be used to improve survival and quality of life. Our findings highlight the large global inequities in congenital heart disease and can serve as a starting point for policy changes to improve screening, treatment, and data collection.

**Funding:**

Bill & Melinda Gates Foundation.

## Introduction

Diagnostic and treatment capabilities for congenital heart disease have dramatically improved over the past 80 years. In the Metropolitan Atlanta Congenital Defects Program, infant survival with critical congenital heart disease improved from 67·4% for the 1979–93 birth cohort to 82·5% for the 1994–2005 cohort.^1^ A Finnish registry study similarly showed a decrease in early and late post-operative congenital heart disease mortality in 1990–2009 compared with 1953–89.^2^ Reports from Belgium and Sweden found that 90–95% of children with congenital heart disease born between 1972 and the early 1990s survived into adulthood.^3^, ^4^ These studies show substantial improvement in survival in developed regions of the world, but the same success rates are not yet seen in developing regions.

The Sustainable Development Goals (SDGs) were adopted by the UN in 2016. SDG 3.2 aims to reduce the mortality of neonates to less than 12 deaths per 1000 live births and the mortality of children to less than 25 deaths per 1000 live births. SDG 3.4 aims to reduce premature mortality due to non-communicable diseases (NCDs) by one-third by 2030. Congenital heart disease accounts for nearly one-third of all congenital birth defects^5^ and, therefore, the focus on congenital heart disease is integral to eliminating preventable child deaths and NCDs in the SDG era.^6^ Nine-tenths of the world's children born with congenital heart disease live in locations with little to no care and where mortality remains high.^7^ Despite some improvements in the past two decades, neonates in low-income and middle-income countries (LMICs) with severe forms of congenital heart disease and without access to surgical treatment are more likely to die before their fifth birthday than are those in high income countries (HICs).^8^ Additional premature mortality might occur secondary to cardiac and pulmonary complications.^8^

**Research in context**

**Evidence before this study**

Previous work to describe the global burden of congenital heart disease was generated from a small number of sources, which were geographically restricted and included only broad categories of congenital heart disease. Previous studies did not comprehensively evaluate congenital heart disease throughout the lifecourse, instead relying on strong assumptions about their distribution and natural history. We did a systematic literature review for prevalence, excess mortality, with-condition mortality, congenital birth defects registries, and hospital administrative data. The literature was also reviewed for outcomes related to congenital heart disease, most notably intellectual disability. No previous studies incorporated morbidity or provided years lived with disability (YLDs). Both measures are informative to policy makers when assessing lifelong implications and the loss of healthy years in comparison with other childhood diseases. We searched PubMed for English-language research articles describing the global burden of congenital heart disease published between Jan 1, 2010, and June 30, 2019, using the terms “pediatric or childhood or child” AND “congenital heart disease or congenital heart defect” AND “global or international or worldwide or world” AND “burden OR metrics OR incidence OR mortality OR prevalence OR survival” but did not find additional applicable work.

**Added value of this study**

To our knowledge, this is the first report of congenital heart disease burden using Global Burden of Diseases, Injuries, and Risk Factors Study 2017 results, with prevalence, YLDs, and mortality as the outcome measures. This study incorporates all available data sources and previous publications making it the most comprehensive study on congenital heart disease burden to date. Estimates of prevalence were more accurate than in previous studies because remission rates for specific subtypes of congenital heart disease were taken into account and congenital heart disease was divided into five subcategories based on anatomic and clinical similarities for improved clarity of disease burden by lesion. This study not only gives a more accurate assessment of the global burden of congenital heart disease, but also highlights the disproportionate disease burden in low Socio-demographic Index (SDI) regions compared to higher SDI regions.

**Implications of all the available evidence**

With the use of GBD 2017 data, this research provides a comprehensive overview of the global burden of congenital heart disease and shows a substantial burden of disease throughout the life cycle. Congenital heart disease burden is especially notable in low SDI regions, where a greater variety of diseases exist and where data is especially needed to better inform priorities for policymakers. As countries and international agencies implement, monitor, and evaluate the UN's Sustainable Development Goals, refined and updated estimates of congenital heart disease burden is crucially important to identify areas in need. This research provides vital information to governments, stakeholders, policymakers, and the global health community worldwide, for the present and the future, as GBD data continues to be refined and improved.

Optimal allocation of resources for screening, clinical care, and development of cost-effective treatment strategies requires accurate assessment of the absolute and relative burden of congenital heart disease across the world. Previous publications have estimated 250 000 annual congenital heart disease deaths globally, 1·35 million annual births of neonates with congenital heart disease globally,^5^ and 2·4 million people living with congenital heart disease in the USA alone.^9^ However, even the global studies were generated from a small number of geographically limited sources, included only broad categories of congenital heart disease, and did not comprehensively evaluate congenital heart disease throughout the lifecourse,^10^ instead relying on strong assumptions about their distribution and natural history.

The Global Burden of Diseases, Injuries, and Risk Factors Study (GBD) is an international collaboration of researchers who annually produce internally consistent estimates of death and disability throughout the world. The 2017 update of GBD generated the most up-to-date estimates of congenital heart disease prevalence and mortality throughout the world. This analysis placed particular emphasis on developing data-driven estimates for the entire lifecourse. Since 2016, GBD has generated estimates of congenital heart disease burden by five different anatomic subtypes, including consideration of remission of some subtypes of congenital heart disease. It incorporated many different data sources on mortality and prevalence and employed a variety of statistical approaches to maximise the robustness of the final results. We report here on the GBD 2017 approach and results, to describe the geographical, temporal, and sociodemographic trends of congenital heart disease from 1990 to 2017.

## Methods

### Overview

Detailed methodology for congenital heart disease estimates is provided in the appendix. Detailed methods for each analytic step in GBD 2017 are described elsewhere.^11^, ^12^, ^13^, ^14^ Reporting is compliant with the Guidelines for Accurate and Transparent Health Estimates Reporting (GATHER).^15^ All input data are available online via the GBD Input Data Sources Tool of the Global Health Data Exchange.

Datasets for estimating congenital heart disease epidemiology were informed by three mechanisms: systematic literature review for prevalence, excess mortality, and with-condition mortality; congenital birth defects registries; and hospital administrative data. The volume of data included in the final models is summarised in the appendix (pp 12–14 and 20). Each model's data and fit can be examined in the GBD online Epi Visualization tool.

### Data availability

Availability of data for fatal and non-fatal cases of congenital heart disease varied widely across countries and regions (appendix p 13). Temporal coverage of data by GBD region for each of mortality and non-fatal analyses are shown in the appendix (p 14). Cause-specific mortality data were identified from most countries globally, but from few countries in Africa, southeast Asia, or Oceania. A flowchart illustrating the estimation process is shown in the appendix (p 15). Deaths due to congenital heart disease were estimated using a common cause-specific mortality framework developed for GBD 2017. Each death was assigned to a single underlying cause. Deaths assigned to ill-defined or non-specific causes (eg, “heart disease, unspecified”, International Classification of Diseases, 10th revision [ICD-10] code I51.9) or intermediate causes (eg, “heart failure”, ICD-10 code I50) were reassigned to probable causes of death, including congenital heart disease, with the use of statistical redistribution algorithms. Illustrations of redistribution for ICD-9 and ICD-10 in the USA are shown in the appendix (p 15, 16). Non-fatal data were identified from a few additional countries with a low Socio-demographic Index (SDI), although the temporal coverage of non-fatal data was categorically lower than was that of mortality data. A flowchart illustrating the estimation process is shown in the appendix (p 18).

### Mortality estimates

Congenital heart disease mortality was estimated using the Cause of Death Ensemble model (CODEm), a tool that runs many different models on the same data and chooses an ensemble of models that best reflects all the available input data. The covariates used in CODEm models for total congenital birth defects and congenital heart disease are listed in the appendix (p 17). Covariates having a prespecified positive correlation with congenital heart disease mortality were maternal alcohol consumption, proportion of live births in women aged 35 years or older, age-standardised diabetes prevalence, indoor air pollution, and reproductive age-standardised smoking prevalence. Covariates having a prespecified negative correlation with congenital heart disease mortality were measles vaccine coverage, education (years per capita), and legality of abortion.

### Non-fatal disease estimates

Starting with GBD 2016, in consultation with GBD experts and paediatric cardiology experts, we have reorganised cause categories for non-fatal estimates to be more anatomically oriented, clinically relevant, and structured in a way to facilitate incorporation of literature, registry, and administrative data to inform levels and trends. These five congenital heart disease categories are named descriptively as: single ventricle and single ventricle pathway congenital heart anomalies; severe congenital heart anomalies excluding single ventricle heart defects; critical malformations of great vessels, congenital valvular heart disease, and patent ductus arteriosus; ventricular septal defect and atrial septal defect; and other congenital heart anomalies.

ICD-9 and ICD-10 codes mapped to each category are shown in the appendix (p 11).

Five separate DisMod-MR 2.1 models were developed for each of the congenital heart disease categories. DisMod-MR 2.1 is a compartmental model consisting of three states—susceptible, diseased, and dead—with state transitions determined by the incidence, remission rate, excess mortality, and other-cause mortality.^16^ DisMod-MR 2.1 uses a geographical cascade to borrow strength across geography and time, balancing a set of differential equations to find an internally consistent numerical solution for each age, sex, location, and year that best reflects the input data and any specified constraints on values of the calculated rates. Incidence was set to 0 for all congenital models, as congenital conditions occur at the time of birth and by definition there are no incident cases after birth. Remission was bounded to 0% for all subcause models except ventricular and atrial septal defect, for which it was constrained to be between 0% and 20% per year until age 10 years on the basis of the values that corresponded to observed spontaneous closure in longitudinal studies.^17^, ^18^ For most of the severe congenital conditions, the mortality associated with the condition is highly dependent on access to adequate surgical interventions and other medical care during the first hours, weeks, and years of life. Therefore, a decreasing slope on excess mortality was applied to capture the highest risk of mortality from congenital conditions in the neonatal age groups and a subsequent decreasing risk of mortality from congenital conditions later in life. Model covariates and calculated coefficients for each are shown in the appendix (pp 21, 22).

The proportions of each type of congenital heart disease with each type of sequela were derived using available information to calculate years lived with disability (YLDs). To assess the distribution of health outcomes associated with the congenital causes, we extracted data from the same studies identified in the systematic review done for the estimation of the prevalence of fatal and non-fatal congenital heart disease, this time focused on the long-term health outcomes of survivors in cohorts born with each type of congenital malformation. For conditions requiring surgical intervention shortly after birth to ensure survival, the health states included in the disability weight calculations correspond to the post-surgery outcomes reported in cohorts of individuals born with these life-threatening congenital conditions.

Heart failure distribution was derived from the updated GBD 2017 heart failure analysis using the same approach of proportional allocation based on cause-specific mortality as previously described.^19^ Several scientific literature sources reported on the prevalence and severity of intellectual disability in populations with congenital heart defect;^20^, ^21^, ^22^ the asymptomatic proportion of individuals with ventricular and atrial septal defects (including those who had undergone repair) was derived from literature sources on the long-term outcomes of patients diagnosed with and, in some cases, treated for, septal defects at birth.^23^ All except those with asymptomatic ventricular and atrial septal defects were also assigned a health state of congenital heart disease. Evidence for congenital heart disease-related disability universally was presented separately for intellectual disability and heart failure. We therefore assumed independence of the probability of each in calculating the proportion of cases of congenital heart disease in each combination of health states (eg, mild heart failure and profound intellectual disability).

To assess the distribution of health outcomes associated with the most common causes of heart disease that have origins in childhood, we compared YLDs and years of life lost (YLLs) from congenital heart disease with YLDs and YLLs from rheumatic heart disease.

### Role of the funding source

The funder of the study played no role in study design, data collection, data analysis, data interpretation, or writing of the report. All authors had full access to all the data in the study and had final responsibility for the decision to submit for publication.

## Results

**Figure 1:**
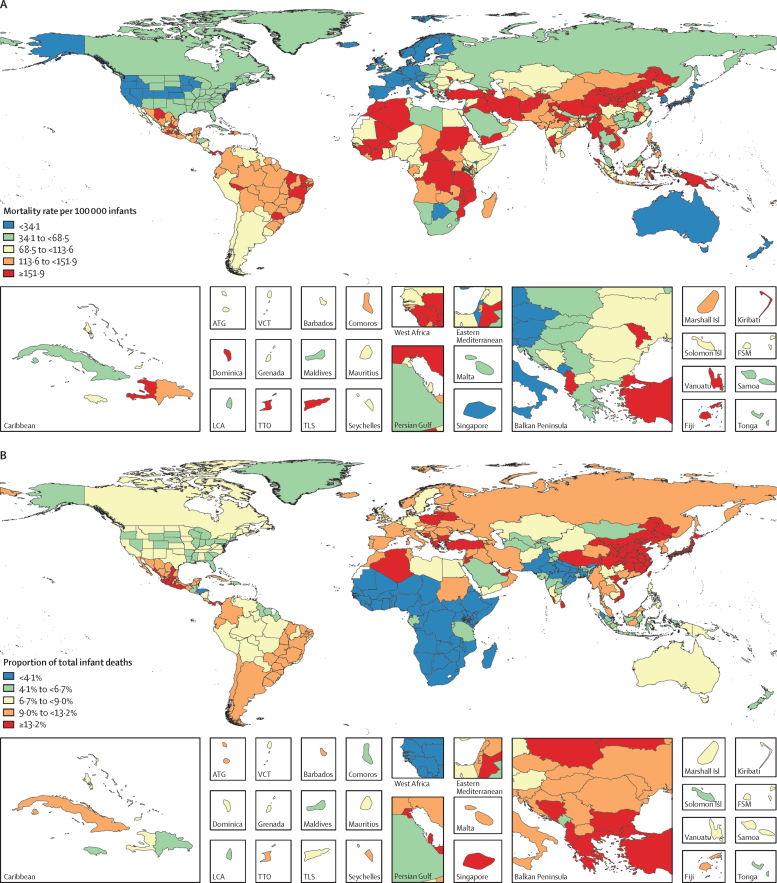

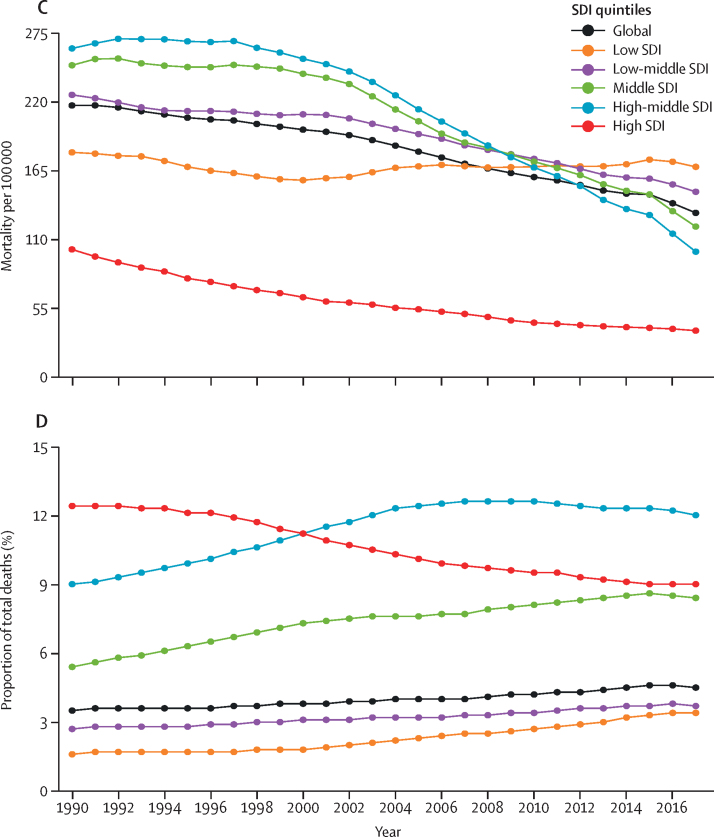
Mortality of congenital heart disease in children under 1 year of age, 1990–2017 (A) Mortality per 100 000 infants by country in 2017 and (C) by SDI from 1990 to 2017. (B) Proportion of infant deaths by country in 2017 and (D) by SDI from 1990 to 2017. Subnational data are available for Brazil, China, India, and the USA. ATG=Antigua and Barbuda. FSM=Federated States of Micronesia. LCA=Saint Lucia. SDI=Socio-demographic Index. TLS=Timor-Leste. TTO=Trinidad and Tobago. VCT=Saint Vincent and the Grenadines.

Congenital heart disease was the underlying cause of an estimated 261 247 (95% uncertainty interval [UI] 216 567–308 159) deaths globally in 2017, a 34·5% (19·8–44·6) decline from 1990, when the number of deaths was 398 580 (268 422–506 469; table 1). Of all congenital heart disease deaths in 2017, 180 624 (146 825–214 178; 69%) occurred in infants younger than 1 year (appendix pp 30–66). Congenital heart disease deaths and mortality were highest in the low and low-middle SDI quintiles. In 2017, regions with the highest infant mortality due to congenital heart disease were, in descending order, Oceania, North Africa and the Middle East, the Caribbean, central sub-Saharan Africa, and southeast Asia (table 1). The regions with the highest mortality rates also had the highest uncertainty intervals (table 1; figure 1A; appendix pp 13, 14). Rates of improvement tracked with development status, with more locations with higher SDIs generally progressing faster than those with lower SDI. The fastest declines in infant mortality due to congenital heart disease occurred in central Europe, high-income Asia Pacific, western Europe, tropical Latin America, Australasia, and eastern Europe, all of which have experienced declines of at least 60% since 1990. Infant mortality rates due to congenital heart disease have declined across all SDI quintiles except low SDI (figure 1C). However, improvement in congenital heart disease mortality has lagged behind that of other causes. Therefore, the proportion of all infant deaths caused by congenital heart disease increased in all quintiles except the high-SDI quintile (figures 1B, 1D).

**Table 1:** Global Burden of Diseases, Injuries, and Risk Factors Study 2017 congenital heart disease mortality, 1990–2017

	**Number of deaths (all ages)**			**Mortality per 100 000 children younger than 1 year**			**Age-standardised mortality per 100 000 individuals**		
	1990	2017	Percentage change, 1990–2017	1990	2017	Percentage change, 1990–2017	1990	2017	Percentage change, 1990–2017
**Global**	**398 580 (268 422 to 506 469)**	**261 247 (216 567 to 308 159)**	−34·5% (−44·6 to −19·8)*	**217·1 (146·8 to 277·5)**	**131·0 (106·5 to 155·3)**	−39·7% (−49·1 to −27·1)*	**6·3 (4·3 to 8·0)**	**3·9 (3·2 to 4·6)**	−39·0% (−48·2 to −26·0)*
Low SDI	67 340 (25 251 to 109 078)	83 873 (56 785 to 110 218)	24·6% (−13·1 to 130·6)	179·5 (71·7 to 287·6)	167·9 (115·7 to 221·1)	−6·5% (−35·0 to 63·1)	5·5 (2·1 to 8·9)	4·9 (3·4 to 6·4)	−10·6% (−37·9 to 61·8)
Low-middle SDI	103 086 (60 630 to 140 879)	82 667 (64 667 to 104 233)	−19·8% (−37·2 to 8·1)	225·6 (135·8 to 310·2)	147·9 (110·5 to 189·4)	−34·5% (−48·3 to −14·6)*	6·8 (4·0 to 9·2)	4·4 (3·5 to 5·6)	−34·9% (−49·0 to −12·9)*
Middle SDI	127 734 (98 849 to 154 111)	58 512 (50 989 to 63 052)	−54·2% (−64·8 to −41·8)*	249·3 (190·1 to 300·6)	120·0 (102·8 to 132·4)	−51·8% (−63·5 to −38·5)*	7·0 (5·5 to 8·5)	3·5 (3·1 to 3·8)	−49·6% (−61·3 to −36·2)*
High-middle SDI	76 987 (61 961 to 89 280)	26 948 (24 814 to 29 373)	−65·0% (−71·0 to −54·8)*	262·8 (206·5 to 308·3)	99·9 (89·2 to 109·1)	−62·0% (−69·3 to −49·2)*	7·3 (5·8 to 8·4)	3·0 (2·7 to 3·2)	−59·3% (−66·5 to −47·4)*
High SDI	21 607 (17 568 to 23 083)	8500 (7653 to 9992)	−60·7% (−64·1 to −52·7)*	101·7 (78·2 to 110·8)	36·6 (32·8 to 41·7)	−64·0% (−68·0 to −54·5)*	3·1 (2·5 to 3·4)	1·2 (1·1 to 1·4)	−61·7% (−65·5 to −52·9)*
**Central Europe, eastern Europe, and central Asia**									
Central Asia	3834 (3183 to 4356)	3155 (2491 to 3815)	−17·7% (−34·2 to 0·7)	150·0 (125·3 to 170·0)	119·1 (94·2 to 148·1)	−20·6% (−36·2 to −2·7)*	4·2 (3·5 to 4·8)	3·5 (2·7 to 4·2)	−17·2% (−34·0 to 1·2)
Central Europe	4969 (3898 to 5568)	1111 (970 to 1247)	−77·6% (−80·8 to −70·1)*	212·6 (162·0 to 242·9)	59·4 (51·9 to 68·4)	−72·1% (−77·2 to −60·9)*	5·7 (4·4 to 6·4)	1·8 (1·6 to 2·0)	−68·9% (−73·7 to −57·5)*
Eastern Europe	7409 (6242 to 8297)	2751 (2390 to 3530)	−62·9% (−68·3 to −51·8)*	161·8 (136·7 to 182·1)	60·7 (52·0 to 77·7)	−62·5% (−68·8 to −50·0)*	4·7 (3·9 to 5·3)	2·0 (1·7 to 2·6)	−56·8% (−63·6 to −42·7)*
**High income**									
Australasia	374 (321 to 429)	197 (159 to 236)	−47·5% (−56·1 to −37·2)*	70·5 (60·3 to 81·6)	26·3 (19·8 to 33·9)	−62·8% (−72·3 to −51·0)*	2·3 (2·0 to 2·6)	0·9 (0·7 to 1·1)	−59·8% (−67·0 to −50·2)*
High-income Asia Pacific	3608 (2624 to 4060)	897 (792 to 1121)	−75·2% (−79·1 to −63·2)*	104·1 (73·3 to 120·3)	29·3 (25·4 to 35·1)	−71·9% (−77·0 to −57·4)*	3·3 (2·4 to 3·8)	1·0 (0·8 to 1·2)	−71·2% (−76·1 to −57·0)*
High-income North America	6430 (5446 to 7079)	3522 (3137 to 4311)	−45·2% (−49·3 to −36·0)*	84·1 (70·0 to 91·9)	37·8 (33·2 to 45·8)	−55·0% (−59·9 to −46·5)*	2·8 (2·3 to 3·0)	1·4 (1·2 to 1·7)	−51·0% (−55·0 to −42·4)*
Southern Latin America	2129 (1766 to 2450)	1259 (946 to 1509)	−40·9% (−54·6 to −24·7)*	159·3 (131·0 to 185·2)	85·6 (63·1 to 106·0)	−46·3% (−60·7 to −29·0)*	4·3 (3·6 to 5·0)	2·4 (1·8 to 2·9)	−43·8% (−57·2 to −28·2)*
Western Europe	7630 (5736 to 8323)	2539 (2138 to 2 848)	−66·7% (−70·4 to −59·1)*	99·4 (64·5 to 113·0)	29·2 (22·3 to 33·2)	−70·6% (−74·5 to −61·4)*	3·0 (2·2 to 3·3)	1·0 (0·8 to 1·1)	−68·5% (−72·2 to −60·0)*
**Latin America and Caribbean**									
Andean Latin America	1891 (1359 to 2596)	2026 (1338 to 2593)	7·1% (−44·3 to 55·6)	115·0 (81·2 to 161·8)	108·1 (69·5 to 143·4)	−6·0% (−53·5 to 41·5)	3·4 (2·4 to 4·6)	3·1 (2·1 to 4·0)	−6·9% (−51·2 to 34·3)
Caribbean	2744 (1692 to 3 463)	2120 (1442 to 2825)	−22·7% (−41·9 to −3·0)*	218·6 (138·6 to 281·3)	193·4 (129·5 to 270·3)	−11·5% (−35·5 to 14·4)	6·6 (4·1 to 8·3)	5·4 (3·7 to 7·2)	−17·9% (−38·6 to 3·2)
Central Latin America	10 767 (9594 to 14 534)	8965 (6154 to 10 275)	−16·7% (−52·9 to 1·9)	165·6 (146·8 to 222·6)	129·2 (85·3 to 150·7)	−22·0% (−58·5 to −1·9)*	4·8 (4·3 to 6·4)	3·7 (2·6 to 4·3)	−21·8% (−55·9 to −4·2)*
Tropical Latin America	19 295 (9542 to 26 614)	5962 (5095 to 6896)	−69·1% (−78·5 to −38·6)*	449·3 (209·9 to 625·3)	144·5 (118·1 to 165·1)	−67·8% (−78·1 to −32·1)*	11·3 (5·6 to 15·5)	3·7 (3·1 to 4·3)	−67·1% (−77·2 to −35·0)*
**North Africa and Middle East**									
North Africa and Middle East	60 031 (30 316 to 84 703)	35 710 (29 606 to 44 359)	−40·5% (−54·5% to −0·3%)*	450·8 (221·5 to 653·2)	211·7 (168·5 to 269·5)	−53·0% (−64·0% to −22·2%)*	11·8 (6·0 to 16·6)	5·8 (4·8 to 7·2)	−51·0% (−62·6% to −18·6%)*
**South Asia**									
South Asia	75 091 (38 351 to 109 094)	66 984 (50 484 to 81 590)	−10·8% (−38·2 to 34·5)	154·8 (76·6 to 223·5)	138·5 (95·5 to 176·6)	−10·6% (−38·3 to 29·4)	4·8 (2·5 to 7·0)	4·0 (3·0 to 4·9)	−17·0% (−42·1 to 21·0)
**Southeast Asia, east Asia, and Oceania**									
East Asia	93 878 (80 522 to 113 589)	31 885 (29 266 to 35 408)	−66·0% (−72·5 to −58·2)*	254·8 (217·6 to 312·0)	112·5 (103·0 to 125·7)	−55·9% (−66·0 to −46·2)*	7·4 (6·3 to 8·9)	3·4 (3·1 to 3·7)	−54·5% (−63·6 to −44·1)*
Oceania	775 (440 to 1071)	1 307 (790 to 1793)	68·8% (23·8 to 126·7)*	244·2 (137·7 to 348·0)	226·4 (126·0 to 327·7)	−7·3% (−35·6 to 32·8)	8·3 (4·9 to 11·2)	7·7 (4·8 to 10·5)	−6·7% (−30·5 to 23·6)
Southeast Asia	42 680 (25 044 to 55 932)	23 976 (19 721 to 27 605)	−43·8% (−56·5 to −19·3)*	268·3 (151·0 to 352·8)	153·1 (120·2 to 181·0)	−42·9% (−55·3 to −17·6)*	7·3 (4·3 to 9·6)	4·4 (3·6 to 5·1)	−40·4% (−53·8 to −15·3)*
**Sub-Saharan Africa**									
Central sub-Saharan Africa	8152 (3377 to 14 002)	11 257 (7101 to 17 318)	38·1% (−0·3 to 128·0)	231·0 (112·1 to 388·3)	189·5 (121·2 to 288·3)	−17·9% (−43·0 to 30·0)	7·7 (3·2 to 13·1)	5·9 (3·8 to 9·0)	−23·4% (−44·2 to 26·7)
Eastern sub-Saharan Africa	25 818 (11 083 to 44 514)	26 998 (19 197 to 38 981)	4·6% (−21·1 to 97·0)	208·4 (100·9 to 344·1)	141·5 (99·7 to 203·7)	−32·1% (−47·8 to 16·3)	6·9 (3·1 to 11·9)	4·4 (3·1 to 6·3)	−36·9% (−52·6 to 17·2)
Southern sub-Saharan Africa	1568 (1301 to 1894)	1252 (1084 to 1444)	−20·1% (−36·4 to 4·2)	62·7 (51·0 to 78·2)	42·1 (35·0 to 50·5)	−32·8% (−48·2 to −10·3)*	2·4 (2·0 to 2·8)	1·5 (1·3 to 1·8)	−34·8% (−47·8 to −16·2)*
Western sub-Saharan Africa	19 507 (7079 to 30 605)	27 373 (15 756 to 39 149)	40·3% (7·1 to 121·7)*	164·6 (68·0 to 261·0)	121·1 (76·8 to 169·4)	−26·4% (−46·0 to 12·2)	5·5 (2·1 to 8·6)	4·0 (2·4 to 5·6)	−27·5% (−44·6 to 14·0)

Data in parentheses are 95% uncertainty intervals. SDI=Socio-demographic Index.

* Significant increase or decrease.

**Figure 2:**
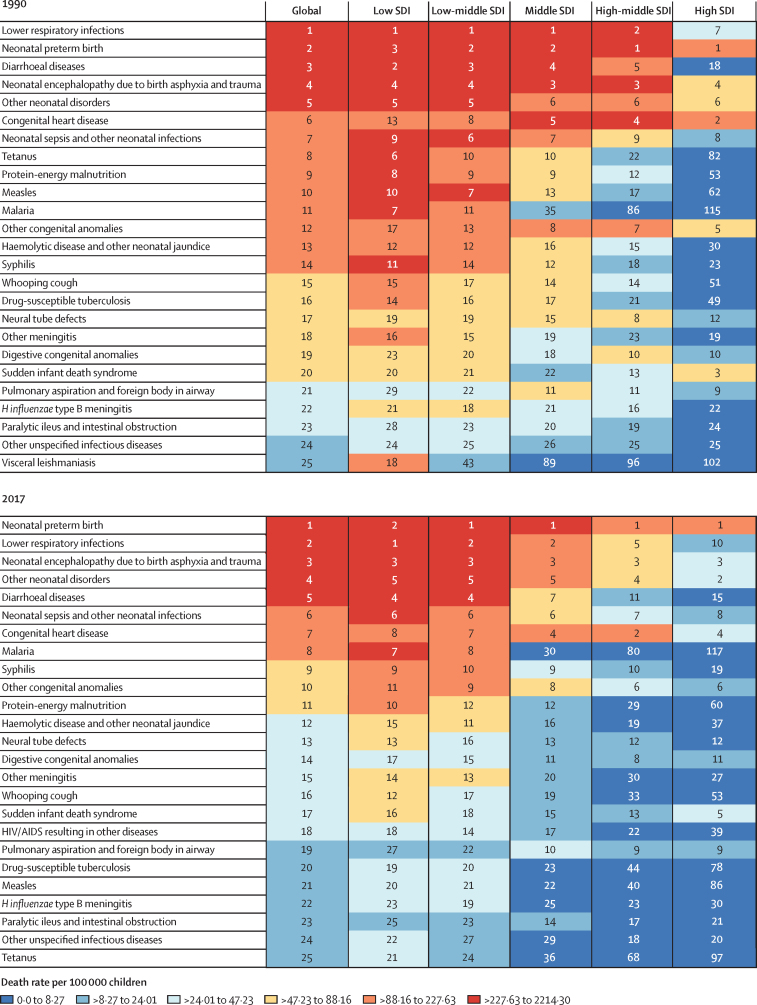
Leading causes of death in children younger than 1 year The heat map depicts rank order of causes of death (by death rate per 100 000) in children younger than 1 year in 1990 and 2017. The rank of congenital heart disease increased from ninth to seventh globally and 17th to 11th in low SDI countries from 1990 to 2017. SDI=Socio-demographic Index.

In the high-SDI quintile in 2017, neonatal preterm birth, other neonatal disorders, neonatal encephalopathy, congenital heart disease, and sudden infant death syndrome were the top five causes of infant mortality (figure 2; appendix p 21). Congenital heart disease rose to be among the top eight causes of infant mortality by 2017 in all SDI quintiles. Two conditions closely related to congenital heart disease were also highly ranked: lower respiratory infections (ie, pneumonia), which was ranked in the top five in all except the high-SDI quintile, and neonatal preterm birth, which was ranked either first or second in all strata of SDI.

**Figure 3:**
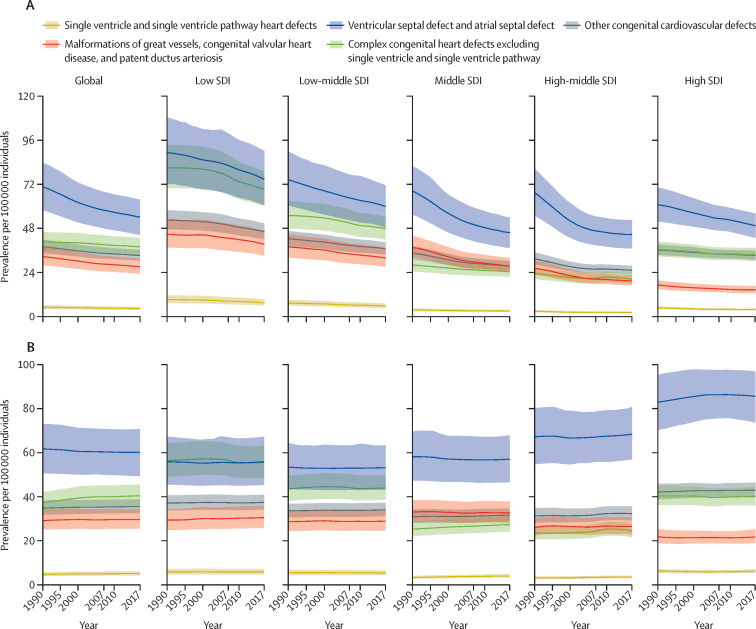
Trends in prevalence of congenital heart disease from 1990 to 2017 Data are prevalence (95% UI). (A) all-age prevalence and (B) age-standardised prevalence by Socio-demographic Index and congenital heart defect sub-category with 95% uncertainty intervals. SDI=Socio-demographic Index.

A total of 11 998 283 (95% UI 10 958 658–13 123 888) people were estimated to be living with congenital heart disease globally in 2017, an 18·7% increase from 10 105 235 (9 159 197–11 150 207) prevalent cases in 1990. The increase is related to improved survival and population growth, partially offset by ageing. There was an estimated prevalence of 466 566 (429 140–505 806) patients with congenital heart disease in the USA, of whom 279 320 (266 461–331 437) are younger than 20 years. The birth prevalence of all congenital heart disease was 1787·6 cases (1587·0–1999·2) per 100 000 babies in 2017, which has increased by 4·2% (2·8–5·6) since 1990 (table 2). The all-ages global prevalence of most subcategories of congenital heart disease remained unchanged from 1990 to 2017 (figure 3). The exception was the ventricular and atrial septal defect subcategory, which decreased from 1990 to 2017 at the global level. This effect was more pronounced in the middle and high-middle SDI quintiles. Estimates of congenital heart disease prevalence (for all congenital heart disease and congenital heart disease subtypes) and number of deaths (for all congenital heart disease), by age group and GBD region are shown in the appendix (pp 30–66).

**Table 2:** Global Burden of Diseases, Injuries, and Risk Factors Study 2017 prevalence of congenital heart disease, 1990–2017

	**Prevalence at birth per 100 000 babies**			**Prevalence per 100 000 children younger than 1 year**			**Prevalence per 100 000 individuals (all ages)**			**Age-standardised prevalence rate per 100 000**		
	1990	2017	Percent change, 1990–2017	1990	2017	Percent change, 1990–2017	1990	2017	Percent change, 1990–2017	1990	2017	Percent change, 1990–2017
**Global**	**1716·1 (1519·6 to 1925·8)**	**1787·6 (1587·0 to 1999·2)**	4·2% (2·8 to 5·6)*	**1192·4 (1051·2 to 1353·9)**	**1233·1 (1092·3 to 1392·7)**	3·4% (1·9 to 4·9)*	**187·3 (169·8 to 206·7)**	**157·0 (143·4 to 171·8)**	−16·2% (−17·4 to −14·9)*	**167·5 (152·1 to 184·8)**	**170·6 (155·6 to 186·9)**	1·9% (0·8 to 2·9)*
Low SDI	2517·0 (2212·7 to 2823·4)	2501·1 (2200·5 to 2807·6)	−0·6% (−2·6 to 1·2)	1613·0 (1425·4 to 1809·7)	1611·3 (1424·7 to 1812·2)	−0·1% (−2·0 to 1·7)	276·8 (250·4 to 305·1)	237·5 (215·5 to 260·9)	−14·2% (−15·5 to −12·9)*	184·3 (167·7 to 202·4)	185·1 (168·5 to 202·4)	0·4% (−1·0 to 1·9)
Low-middle SDI	1897·8 (1669·9 to 2128·2)	1867·1 (1648·8 to 2089·7)	−1·6% (−3·7 to 0·7)	1278·5 (1131·7 to 1442·7)	1273·8 (1126·3 to 1438·8)	−0·4% (−2·5 to 1·9)	217·3 (196·8 to 239·9)	182·7 (166·6 to 200·1)	−15·9% (−17·2 to −14·5)*	164·5 (149·2 to 180·5)	165·4 (151·0 to 180·9)	0·6% (−0·8 to 2·0)
Middle SDI	1384·5 (1217·9 to 1567·3)	1350·7 (1191·8 to 1531·5)	−2·4% (−4·3 to −0·8)*	1027·9 (894·1 to 1179·4)	1001·6 (879·9 to 1137·5)	−2·5% (−4·8 to −0·8)*	172·4 (155·2 to 191·5)	128·7 (116·8 to 141·5)	−25·4% (−26·9 to −23·9)*	150·3 (135·7 to 166·6)	152·4 (137·8 to 168·1)	1·4% (−0·1 to 2·7)
High-middle SDI	1207·1 (1052·7 to 1383·7)	1184·2 (1038·4 to 1342·2)	−1·9% (−4·2 to 0·5)	949·3 (816·3 to 1102·6)	937·4 (817·3 to 1076·3)	−1·3% (−3·8 to 1·3)	151·8 (136·1 to 169·9)	112·4 (102·1 to 123·6)	−25·9% (−27·9 to −24·1)*	151·0 (135·3 to 169·0)	154·9 (139·3 to 171·6)	2·6% (0·7 to 4·2)*
High SDI	1235·3 (1106·3 to 1381·0)	1255·5 (1129·6 to 1393·2)	1·6% (−0·2 to 3·6)	995·4 (878·7 to 1122·7)	1020·3 (910·6 to 1135·9)	2·5% (0·5 to 4·6)*	156·0 (144·0 to 170·0)	135·0 (125·1 to 145·4)	−13·5% (−14·8 to −12·0)*	193·3 (177·1 to 211·7)	196·4 (180·9 to 212·6)	1·6% (0·2 to 2·9)*
**Central Europe, eastern Europe, and central Asia**												
Central Asia	1 582·5 (1 387·5 to 1 805·6)	1 542·4 (1 361·2 to 1 751·5)	−2·5% (−7·0 to 2·2)	1 215·2 (1 057·5 to 1 392·5)	1 180·1 (1 026·7 to 1 362·9)	−2·9% (−7·6 to 2·1)	226·8 (202·5 to 252·7)	188·7 (168·9 to 210·4)	−16·8% (−19·8 to −14·0)*	187·1 (167·5 to 207·1)	187·2 (167·6 to 208·7)	0·1% (−3·4 to 3·5)
Central Europe	1 221·5 (1 053·9 to 1 404·5)	1 216·5 (1 096·0 to 1 350·8)	−0·4% (−6·0 to 5·4)	1 007·2 (861·0 to 1 167·5)	1 014·6 (904·7 to 1 130·5)	0·7% (−5·2 to 7·0)	148·7 (133·0 to 165·1)	118·1 (108·8 to 127·7)	−20·6% (−24·0 to −16·6)*	177·3 (157·5 to 198·3)	184·2 (168·4 to 200·4)	3·9% (−0·4 to 8·6)
Eastern Europe	1 341·2 (1 167·3 to 1 542·7)	1 314·2 (1 146·0 to 1 513·7)	−2·0% (−4·6 to 0·3)	1 067·5 (918·4 to 1 242·3)	1 053·7 (907·5 to 1 220·8)	−1·3% (−4·0 to 1·2)	146·0 (131·2 to 162·8)	122·4 (110·8 to 136·1)	−16·2% (−17·7 to −14·7)*	176·6 (157·8 to 197·1)	178·5 (158·8 to 200·0)	1·1% (−0·8 to 2·9)
**High income**												
Australasia	974·9 (854·8 to 1127·8)	1023·6 (891·6 to 1165·2)	5·0% (−3·1 to 14·1)	784·7 (680·8 to 910·5)	824·8 (715·0 to 949·8)	5·1% (−3·7 to 14·7)	132·5 (119·5 to 145·6)	121·9 (110·6 to 133·8)	−7·9% (−12·1 to −3·0)*	152·6 (136·9 to 168·9)	158·2 (142·2 to 174·6)	3·7% (−1·3 to 9·6)
High-income Asia Pacific	2116·9 (1853·4 to 2425·1)	2066·2 (1792·0 to 2378·9)	−2·4% (−5·5 to 1·1)	1700·2 (1461·8 to 1966·5)	1690·3 (1440·5 to 1954·9)	−0·6% (−4·0 to 3·1)	224·2 (202·6 to 247·1)	170·3 (154·9 to 186·5)	−24·1% (−25·7 to −22·4)*	290·4 (259·3 to 323·6)	294·0 (261·0 to 326·5)	1·2% (−1·1 to 3·8)
High-income North America	1232·2 (1091·7 to 1389·6)	1229·0 (1093·0 to 1382·1)	−0·3% (−3·5 to 3·3)	995·7 (872·4 to 1132·3)	995·8 (877·9 to 1128·3)	0·0% (−3·3 to 3·7)	163·2 (148·9 to 179·3)	144·6 (133·2 to 156·8)	−11·4% (−13·5 to −9·2)*	191·9 (174·3 to 211·4)	191·7 (175·7 to 209·7)	−0·1% (−2·3 to 2·4)
Southern Latin America	1711·8 (1501·9 to 1935·9)	1595·1 (1401·4 to 1806·9)	−6·8% (−12·9 to 0·0)	1226·0 (1080·5 to 1385·3)	1165·5 (1023·4 to 1328·5)	−4·9% (−11·2 to 2·0)	203·8 (185·7 to 223·9)	161·6 (146·9 to 177·9)	−20·7% (−24·3 to −17·1)*	199·5 (181·9 to 219·1)	187·5 (169·9 to 206·5)	−6·0% (−10·4 to −1·6)*
Western Europe	963·9 (891·7 to 1043·1)	1139·3 (1055·8 to 1226·5)	18·2% (15·8 to 20·6)*	773·9 (713·3 to 841·9)	920·9 (853·8 to 990·7)	19·0% (16·5 to 21·6)*	127·6 (119·8 to 136·3)	126·1 (118·5 to 134·5)	−1·1% (−2·8 to 0·6)	163·1 (153·3 to 174·0)	181·8 (170·7 to 193·6)	11·5% (9·6 to 13·5)*
**Latin America and Caribbean**												
Andean Latin America	1370·9 (1211·6 to 1536·3)	1326·8 (1166·8 to 1506·3)	−3·2% (−8·0 to 1·6)	964·0 (849·6 to 1083·3)	936·9 (831·4 to 1061·3)	−2·8% (−7·7 to 2·1)	187·5 (171·8 to 204·7)	152·0 (139·9 to 165·7)	−19·0% (−21·9 to −16·0)*	147·5 (135·3 to 160·9)	146·5 (135·0 to 159·6)	−0·7% (−4·2 to 2·8)
Caribbean	1445·5 (1271·8 to 1638·0)	1638·0 (1450·9 to 1854·8)	13·3% (7·8 to 19·2)*	1039·7 (917·2 to 1180·8)	1138·8 (1007·0 to 1299·6)	9·5% (4·4 to 14·9)*	180·1 (163·3 to 198·5)	150·1 (137·6 to 164·2)	−16·6% (−19·1 to −14·0)*	161·9 (147·3 to 178·2)	167·2 (152·8 to 183·1)	3·2% (0·3 to 6·3)*
Central Latin America	913·5 (800·1 to 1030·3)	894·1 (785·7 to 1003·6)	−2·1% (−4·4 to 0·2)	645·2 (564·1 to 735·9)	628·2 (554·4 to 709·4)	−2·6% (−5·0 to −0·2)*	135·6 (123·6 to 149·7)	106·0 (97·4 to 115·3)	−21·9% (−23·6 to −20·2)*	109·5 (100·1 to 119·9)	108·6 (99·7 to 118·2)	−0·8% (−2·5 to 0·8)
Tropical Latin America	1077·2 (939·7 to 1222·7)	983·0 (868·7 to 1 103·0)	−8·8% (−11·0 to −6·1)*	752·2 (657·1 to 860·7)	688·5 (609·3 to 783·1)	−8·5% (−10·8 to −5·8)*	140·7 (127·1 to 155·7)	105·6 (96·9 to 114·7)	−25·0% (−27·1 to −22·7)*	123·6 (111·9 to 136·5)	122·8 (112·4 to 134·0)	−0·6% (−2·8 to 1·6)
**North Africa and Middle East**												
North Africa and Middle East	1251·3 (1105·6 to 1414·0)	1303·1 (1159·3 to 1464·2)	4·1% (1·0 to 7·1)*	903·2 (794·1 to 1032·7)	915·4 (805·9 to 1045·6)	1·4% (−1·8 to 4·4)	175·7 (157·8 to 195·4)	141·1 (127·6 to 155·9)	−19·7% (−21·7 to −17·9)*	132·1 (119·4 to 146·3)	135·0 (122·2 to 149·2)	2·2% (0·1 to 4·3)*
**South Asia**												
South Asia	2047·8 (1805·0 to 2295·6)	1987·3 (1751·0 to 2228·6)	−3·0% (−5·2 to −0·6)*	1299·6 (1152·0 to 1463·1)	1257·8 (1117·7 to 1414·8)	−3·2% (−5·2 to −1·1)*	215·8 (195·6 to 237·5)	165·4 (151·2 to 181·1)	−23·4% (−24·6 to −22·0)*	165·7 (150·7 to 181·3)	163·9 (149·8 to 179·3)	−1·1% (−2·5 to 0·4)
**Southeast Asia, east Asia, and Oceania**												
East Asia	1266·9 (1088·2 to 1473·5)	1243·7 (1077·8 to 1430·2)	−1·8% (−5·5 to 1·2)	979·1 (831·3 to 1156·5)	973·3 (838·8 to 1122·7)	−0·6% (−4·6 to 2·8)	145·9 (129·5 to 166·3)	100·7 (91·1 to 111·5)	−31·0% (−33·7 to −28·7)*	142·1 (125·9 to 161·8)	150·6 (134·9 to 168·0)	6·0% (2·7 to 8·8)*
Oceania	1905·4 (1664·4 to 2168·9)	2088·4 (1844·1 to 2380·3)	9·6% (2·3 to 16·8)*	1407·4 (1228·5 to 1622·1)	1521·0 (1331·0 to 1737·7)	8·1% (1·3 to 15·6)*	265·7 (237·6 to 297·7)	260·4 (234·6 to 290·4)	−2·0% (−6·6 to 3·2)	195·2 (175·4 to 217·3)	206·3 (186·3 to 229·2)	5·7% (1·0 to 11·0)*
Southeast Asia	1714·5 (1504·1 to 1944·3)	1686·7 (1477·1 to 1917·9)	−1·6% (−4·3 to 1·3)	1296·8 (1127·2 to 1475·9)	1280·8 (1120·3 to 1465·5)	−1·2% (−4·1 to 1·9)	224·1 (201·4 to 249·0)	168·8 (152·5 to 186·3)	−24·7% (−26·3 to −23·0)*	184·3 (165·9 to 204·4)	186·4 (168·0 to 206·4)	1·1% (−0·8 to 3·2)
**Sub-Saharan Africa**												
Central sub-Saharan Africa	2512·8 (2205·7 to 2833·2)	2659·8 (2299·4 to 3034·0)	5·8% (−0·0 to 12·9)	1708·1 (1497·0 to 1924·2)	1760·9 (1529·4 to 1994·2)	3·1% (−2·7 to 9·9)	307·3 (276·3 to 340·3)	284·0 (255·7 to 314·3)	−7·6% (−11·7 to −2·9)*	193·9 (175·4 to 213·2)	197·2 (178·2 to 217·8)	1·7% (−2·5 to 6·8)
Eastern sub-Saharan Africa	2855·5 (2504·8 to 3218·7)	2669·5 (2350·6 to 3010·3)	−6·5% (−8·6 to −4·4)*	1883·2 (1655·3 to 2127·7)	1788·0 (1577·6 to 2016·8)	−5·0% (−7·0 to −2·9)*	338·2 (304·7 to 373·4)	291·7 (263·1 to 320·9)	−13·7% (−15·2 to −12·2)*	208·6 (188·8 to 229·4)	202·2 (183·0 to 221·9)	−3·1% (−4·6 to −1·5)*
Southern sub-Saharan Africa	2005·5 (1764·9 to 2249·6)	2010·9 (1764·8 to 2257·8)	0·3% (−2·8 to 3·2)	1426·6 (1254·7 to 1615·7)	1429·2 (1257·8 to 1619·2)	0·2% (−2·9 to 3·1)	239·1 (215·5 to 264·2)	199·6 (180·3 to 220·4)	−16·5% (−18·5 to −14·7)*	187·1 (169·2 to 205·8)	187·3 (169·3 to 206·7)	0·1% (−2·1 to 2·3)
Western sub-Saharan Africa	2405·2 (2105·2 to 2721·1)	2321·3 (2033·9 to 2619·2)	−3·5% (−7·1 to 0·4)	1641·9 (1441·1 to 1866·0)	1595·4 (1401·8 to 1807·5)	−2·8% (−6·7 to 1·4)	288·8 (259·7 to 319·8)	266·5 (241·1 to 294·2)	−7·7% (−10·8 to −4·7)*	187·2 (169·2 to 205·9)	183·6 (166·9 to 201·5)	−1·9% (−5·0 to 1·0)

Data are estimate (95% uncertainty interval). SDI=Socio-demographic Index.

* Significant increase or decrease.

Since 1990, the rate of YLDs has remained around 7 YLDs per 100 000 individuals in high-SDI countries and around 14 YLDs per 100 000 individuals in low-SDI countries. In 2017, the estimated global number of all-ages YLDs due to congenital heart disease was 589 479 YLDs (95% UI 287 200–973 359); YLD rates were highest in infants younger than 1 year. Infant YLD rates varied significantly throughout the world and within the USA, with the highest rates estimated for western, central, and eastern sub-Saharan Africa, central and southeast Asia, and some internal provinces of China and India (appendix p 27). From 1990 to 2017, there was no significant increase in infant YLD rates (appendix p 27).

Rates of congenital heart disease at birth varied by a factor of about two across all countries and territories in 2017 (Figure S9), with the highest rates observed in western, central, and eastern sub-Saharan Africa, central and southeast Asia, and some internal provinces of China and India, mirroring the YLD rates. The lowest rates for congenital heart disease at birth were concentrated in the Americas, western Europe, north Africa, Australia, and some northern provinces of China. Mapping age-standardised prevalence revealed a different pattern, with many HICs, along with some countries within sub-Saharan Africa, having the highest age-standardised prevalence rates.

When evaluating YLDs for all heart disease in individuals younger than 20 years, congenital heart disease and rheumatic heart disease are the most common causes, but their epidemiology and aggregate burden are quite different (appendix p 29). The aggregate YLDs caused by rheumatic heart disease were estimated to be substantially larger in 2017 than the number caused by congenital heart disease (1 900 974 YLDs [95% UI 1 232 815–2 765 992] for rheumatic heart disease versus 589 479 [287 200–973 359] for congenital heart disease) but the total number of years of life lost (YLLs) caused by congenital heart disease was more than double that of rheumatic heart disease (21 634 418 [17 779 611–25 604 823] for congenital heart disease versus 7 492 586 [6 926 660–8 046 713] for rheumatic heart disease). Although rheumatic heart disease often has its origins in childhood, most of both the fatal and non-fatal burden of rheumatic heart disease occurs in adults older than 20 years. By contrast, the fatal and non-fatal burden of congenital heart disease is highly concentrated in children, especially infants younger than 1 year.

## Discussion

The global prevalence of congenital heart disease at birth, in 2017, is estimated to be nearly 1·8 cases per 100 live births, a 4·2% (95% UI 2·8–5·6) increase since 1990. In comparison with previously determined prevalence rates, the birth prevalence is likely to be higher because of the inclusion of data for low and middle SDI regions, which have not been previously accounted for, as well as because of biological and social factors that warrant additional investigation.^5^ There was a decreasing trend in prevalence for the ventricular and atrial septal defect subcategory, probably related to an increased proportion of lesions being repaired or having spontaneously regressed, in conjunction with an ageing population. When the population is comprised of a greater proportion of older generations than was the population in 1990, a larger proportion of ventricular and atrial septal defect lesions are either repaired or have regressed. Ageing probably also accounts for the observed increase in the estimated proportion of all congenital heart disease cases in patients aged 15 years and older, which increased from nearly 23% in 1990, to over 28% in 2017. Nearly 12·0 million people were estimated to be living with congenital heart disease in 2017 across all age groups, an 18·7% increase from the estimated 10·1 million in 1990. The total number of YLDs from congenital heart disease in 2017 is estimated to be 589 479 (287 200–973 359), which is on par with attention deficit/hyperactivity disorder, peptic ulcer disease, lower respiratory infections, and haemolytic disease and other neonatal jaundice. Cardiac, pulmonary, nutritional, haematological, and gastrointestinal complications all contribute to YLD, but there are insufficient data available to quantify the effect of specific causes of morbidity.^8^ There is also suggestion of increased developmental problems in some children with congenital heart disease—such as sensory impairments, intellectual disability, and learning disabilities—independent of the severity of the congenital heart disease lesion (appendix pp 23, 24).^21^

The annual global mortality of congenital heart disease in 2017 is estimated to be 261 247 (95% UI 216 567–308 159). The rate of decline in congenital heart disease prevalence markedly differs by SDI quintile in the first year of life. In the low SDI quintile, the prevalence of congenital heart disease mortality for infants has declined by only 6% since 1990, compared with declines of more than 50% in the middle, high-middle, and high SDI subgroups. Although the prevalence in middle and high-middle SDI quintiles has decreased gradually over the past three decades, mortality is still more than double that of HICs. This finding is consistent with published evidence showing very low survival of patients with complex and critical congenital heart disease who are older than 1 year and do not have access to advanced treatment and diagnostics.^24^ Studies in HICs also show substantially higher long-term morbidity and mortality in patients with all forms of congenital heart disease than in the general population.^25^, ^26^

The relative importance of congenital heart disease as a cause of child mortality is rapidly increasing, as evidenced by the increase in the proportion of deaths due to congenital heart disease from 1990 to 2017, for all but the high SDI quintile. Other common diseases, such as respiratory disease and malnutrition, still rank as leading causes of death in low SDI countries, but have fallen in rank in higher SDI countries. This observation is consistent with commonly seen shifts from communicable to non-communicable diseases in countries with improving economies and public health systems. In middle and high-middle SDI quintiles, mortality from other common childhood killers has declined to a greater degree than that from congenital heart disease, and therefore mortality from congenital heart disease is accounting for a larger percentage of infant deaths. Although these trends would suggest a shift in investments and priority toward congenital heart disease, it is important to recognise that the cost and complexity of treating congenital heart disease is much greater than for many other childhood illnesses.

In many countries, prevalence and mortality from rheumatic heart disease parallel lack of access to cardiac surgery services for congenital heart disease. Although the age distributions of congenital heart disease and rheumatic heart disease mortality and YLDs differ, many emerging cardiac surgery programmes in LMICs treat children with congenital heart disease as well as children and young adults with rheumatic heart disease. A recent report provided country-level data for burden of rheumatic heart disease with an estimated annual mortality of over 300 000 deaths.^27^ Insufficient availability of cardiac surgery services in sub-Saharan Africa results in well under 3% of children in need of surgery actually receiving it.^28^ Pooling resources to care for both of these diseases makes a stronger case for investment in surgical services than for either disease alone.

The ability to accurately estimate both fatal and non-fatal indicators for congenital heart disease has historically been, and remains, complicated for many reasons. Despite GBD 2017 mortality estimates being similar to previous publications, designating congenital heart disease as the underlying cause of death risks underestimating the full disease burden because of the association between congenital heart disease and other diseases, including genetic syndromes and pulmonary and cardiac complications.^8^ The remission of specific types of congenital heart disease such as ventricular and atrial septal defects also complicates the estimation of congenital heart disease prevalence. As with many diseases, there are variations in data gathering and reporting strategies within regions, countries, and hospitals, which include but are not limited to coding systems, reporting of multiple congenital heart defects, and accounting for termination of pregnancies. Strickland and colleagues demonstrated that ICD-9 Clinical Modification coding can have relatively low sensitivity and high false-positive fraction for some congenital heart diseases compared with clinical nomenclature.^29^ There continues to be debate surrounding the so-called unnatural history of patients after uncomplicated repair of ventricular and atrial septal defects in childhood, and whether or not these individuals do or do not continue to be living with congenital heart disease.^8^, ^22^ This question also applies to lesions with no haemodynamic consequences, such as patent ductus arteriosus and some pulmonary stenosis, because they are less likely to come to attention and therefore remission rates of these defects is unknown. Although many patients with moderate or large ventricular and atrial septal defects have substantial morbidity and mortality (if not repaired), most people born with small ventricular septal defects, partially closed atrial septal defects, or patent foramen ovales, have no increased morbidity or mortality from this diagnosis. Ventricular septal defects are the most common form of congenital heart disease and at least 25% of all ventricular septal defects spontaneously close,^30^ with some studies reporting up to 20% closure per year. The actual rate of closure varies between studies, in large part because of different methodology.^17^, ^18^, ^31^

Previous studies have used varying techniques to account for the aforementioned issues. The US Centers for Disease Control and Prevention reported higher congenital heart disease prevalence than did GBD 2017 for the USA, at 1·4 million adults and 1 million children in 2010,^9^ but these estimates were based on 2010 data from Quebec, Canada, that extrapolated the probability of survival of all congenital heart disease types to the entirety of births in the USA.^32^ This approach, in addition to not using primary data from the USA, assumes that every person born with any type of congenital heart disease, regardless of severity, continues to have disease throughout their life. Hoffman and colleagues generated global prevalence estimates of structural heart disease at birth of 1–1·2 cases per 1000 births, but excluded most ventricular and atrial septal defects.^33^ Liu and colleagues estimated a global birth prevalence of 941 cases per 100 000 births.^34^ Despite not having any adjustments for compositional bias, temporal changes in detection, or definitional differences in input data, Liu and colleagues' estimates were also close to those of GBD in locations with abundant data (eg, western Europe, east Asia, and USA). By contrast, large differences in global estimates emerged in Liu and colleagues' study compared with GBD because of the inclusion of two studies from sub-Saharan Africa that reported congenital heart disease nearly an order of magnitude lower than other studies and the use of a simple meta-regression, in which all data for children younger than 6 years was considered as equivalent to birth data and the only predictor was region and World Bank income category. The approach used in the Modell Global Database on Birth Defects has been to assume a uniform baseline prevalence in the absence of prevention and treatment efforts and to then adjust the baseline on the basis of estimates of intervention coverage for selected groups of congenital malformations.^35^

Our analysis has several notable advances compared with previous GBD estimates and other estimates of congenital heart disease. First, we evolved the prevalence analysis to focus on specific anatomic groupings of congenital heart disease rather than categorically classifying each case of congenital heart disease as moderate, severe, or critical, as was done for GBD 2010, 2013, and 2015. Second, we incorporated many new sources of data. Before GBD 2016, survival estimates were mechanistically tied to neonatal mortality rates, which is unlikely to be accurate, especially in locations where neonatal survival has improved but no congenital heart disease screening or treatment exists. The estimates are now driven by the combined power of data from screening programmes, congenital registries, administrative data, and data sources on mortality and survival. Third, we removed the assumption that congenital heart disease never remits. Failing to account for these lesions in remission modelling could result in substantial overestimation of congenital heart disease prevalence, which probably occurred in other analyses. Fourth, we included all individuals with congenital heart disease in GBD 2017 estimates. Previous GBD estimates excluded any individual with chromosomal or genetic syndromes, meaning that a child with trisomy 21 and atrioventricular septal defect would not have been counted. All of this came together in making GBD 2017 the only effort to comprehensively estimate congenital heart disease prevalence and mortality globally across all ages and over time, accounting for alcohol consumption in women of reproductive age by country, inclusion or exclusions of genetic syndromes and pregnancy terminations in reported data, and quantifying variable excess mortality and remission. This analysis unsurprisingly generated lower predicted long-term survival, higher remission, and lower prevalence than global or regional extrapolations based on studies from HICs,^36^, ^37^ but also generated estimates that are empirical and poised to be highly actionable.

The present analysis also has several limitations. First, it shares all the limitations of the overall GBD 2017 study, which includes little or no direct data on congenital heart disease outcomes from many countries.^11^, ^12^, ^13^, ^14^ As with all GBD modelling, the methodology provided in the appendix (pp 12–14, 17–22) addresses gaps in knowledge, highlighting what we don't know and providing uncertainty intervals for each data point. Second, even after adjustment, potential underreporting might persist in administrative datasets from locations with low diagnostic capacity and in older age groups. Third, and relatedly, although subtyping of congenital heart disease is expanded in our dataset, it has yet to capture the full spectrum of disease. This limitation is particularly true for defects that have a wide variety of presentations, severity, and outcomes, such as ventricular septal defects, atrial septal defects, Ebstein's anomaly, patent ductus arteriosus, and double outlet right ventricle. Within each of the five subtypes, there is a mix of diseases, especially in the group called critical malformations of great vessels, congenital valvular heart disease, and patent ductus arteriosus. Some lesions remit spontaneously or are considered to be functionally cured after surgical or catheter-based intervention, whereas others can never remit spontaneously or by intervention. Although these issues could theoretically lead to underestimates of prevalence after the neonatal period, this possibility is tempered by the fact that the model results closely reflect most data sources that show rapidly decreasing prevalence rates with age. Fourth, also as described above, the GBD cause-specific mortality framework assigns each death to a single underlying cause, which is unlikely to capture the full burden of congenital heart disease, especially in older age groups and in high-mortality settings where diagnostics and treatment are most limited. Fifth, we have not comprehensively quantified individuals with multiple congenital heart disease lesions, instead constraining the sum of each subtype to match the total. Sixth, cross-sectional data (at a given time point) cannot be extrapolated to make conclusions about longitudinal outcomes. For example, comparison of birth and all-age prevalence of congenital heart disease in 2017 does not allow for direct quantification of the magnitude of death or remission of congenital heart disease over time. Those in older age groups were born in years with lower prevalence rates of congenital heart disease at birth and lower total global population. Using cross-sectional data to predict death and remission over time would result in a substantial overestimation in the decline of congenital heart disease prevalence rates with age. Seventh, little data are available on some aspects of congenital heart disease. The most notable data gaps include: the distribution of disabling sequelae (including developmental disabilities) for which the only information we have identified is from high SDI settings; fetal, newborn, and population prevalence from many lower-resource settings, including nearly all of sub-Saharan Africa, Oceania, Southeast Asia, and parts of North Africa and the Middle East; and cause-specific mortality data from sub-Saharan Africa, southeast Asia, and Oceania, ideally in conjunction with programmes to ascertain prevalence in neonates and young children. Finally, this analysis did not include any explicit consideration of coverage of access to cardiac surgery, catheter-based interventions, or supportive medical care, which could be helpful determinants of degree of disability and long-term survival.

The landscape of health—especially child health—is changing across the world. Accurate and systematically updated disease burden data are essential to track progress and encourage sustainable solutions for providing high-quality treatment for all children in need. GBD estimates of congenital heart disease burden provide a critically important advance in global population health, helping provide a clearer roadmap for resource use and allocation to increase access to cardiovascular and NCD care, especially in LMICs where progress on congenital heart disease has been largely stagnant. The UN has prioritised reduction of premature deaths from NCDs (including congenital and acquired heart disease), but to meet the SDG target of “ending preventable deaths of newborns and children under 5 years of age”, health policy makers will need to develop specific accountability measures that address barriers and improve access to paediatric cardiac diagnosis and treatment.

For the **SDGs** see http://www.un.org/sustainabledevelopment/blog/2015/12/sustainabledevelopment-goals-kick-offwith-start-of-new-year/

For the **Global Health Data Exchange** see http://ghdx.healthdata.org

For the **Epi Visualization tool** see http://vizhub.healthdata.org/epi

**This online publication has been corrected. The corrected version first appeared at thelancet.com/child-adolescent on February 7, 2020**
