# Correction to *Lancet Child Adolesc Health* 2020; 4: 185–200

**DOI:** 10.1016/S2352-4642(20)30031-6

**Published:** 2020-03

*GBD 2017 Congenital Heart Disease Collaborators. Global, regional, and national burden of congenital heart disease, 1990–2017: a systematic analysis for the Global Burden of Disease Study 2017*. Lancet Child Adolesc Health *2020; **4:** 185–200.* The first sentence of the discussion has been corrected to read “The global prevalence of congenital heart disease at birth, in 2017, is estimated to be nearly 1·8 cases per 100 live births […]”. This correction has been made to the online version as of Feb 7, 2020, and the printed version is correct.

