# Supplementary material for: Global, regional, and national burden of congenital heart disease, 1990–2017: a systematic analysis for the Global Burden of Disease Study 2017
Source: Lancet Child Adolesc Health. 2020 Mar;4(3):185–200. doi: 10.1016/S2352-4642(19)30402-X (PMC7645774; doi:10.1016/S2352-4642(19)30402-X)
Supplement: Supplementary appendix [file mmc1.pdf]

THE LANCET
Child & Adolescent Health

Supplementary appendix

This appendix formed part of the original submission and has been peer reviewed. We post it as supplied by the authors.

Supplement to: GBD 2017 Congenital Heart Disease Collaborators. Global, regional, and national burden of congenital heart disease, 1990–2017: a systematic analysis for the Global Burden of Disease Study 2017. *Lancet Child Adolesc Health* 2020; published online Jan 21. https://doi.org/10.1016/S2352-4642(19)30402-X.

Supplementary appendix to Global, regional, and national burden of congenital heart disease, 1990–2017: a systematic analysis for the Global Burden of Disease Study 2017

Table of Contents

Investigators 3

Affiliations 4

Figure S1. Socio-demographic Index quintiles by GBD administrative Level 1 geography, 2017 8

Table S1: GATHER checklist of information that should be included in reports of global health estimates, with description of compliance and location of information for GBD 2017 8

Congenital heart disease: overview, definitions, and cause classification 10

Cause-specific mortality overview 10

Non-fatal health outcomes overview 10

Table S2: International Classification of Diseases version 9 (ICD-9) and 10 (ICD-10) mapping to categories of congenital heart disease 11

Overview of data availability for congenital heart disease 12

Figure S2: Classification of countries according to the availability of data on mortality and non-fatal cases of congenital heart disease, 1990–2017 13

Figure S3: Data representativeness by region and year, 1980–2017, mortality and non-fatal data 14

Supplemental methods: Cause-specific mortality of congenital heart disease 15

Figure S4: Estimation flowchart for cause-specific mortality due to congenital heart disease 15

Input data 15

Figure S5: ICD-9 (left) and ICD-10 (right) redistribution of ill-defined deaths to congenital heart disease for USA, all years, all ages, both sexes (original on left, final after redistribution on right) 15

Data evaluation and model specification 16

Table S3: Covariates included in CODEm model of overall congenital birth defects 17

Table S4: Covariates included in CODEm model of congenital heart disease 17

CoDCorrect and calculation of years of life lost (YLLs) 17

Supplemental methods: Non-fatal health loss due to congenital heart disease 18

Figure S6: Estimation flowchart for estimation of prevalence and YLDs due to congenital cardiovascular birth defects 18
Input data 18
Table S5: Prevalence dataset contents for each of the congenital heart defects non-fatal models 20
Data evaluation and model specification 20
Table S6: Covariates and model performance for DisMod-MR 2.1 of total congenital heart anomalies 21
Table S7: Covariates and model performance for DisMod-MR 2.1 of single ventricle and single ventricle pathway defects 21
Table S8: Covariates and model performance for DisMod-MR 2.1 of complex congenital heart defects excluding single ventricle and single ventricle pathway defects 22
Table S9: Covariates and model performance for DisMod-MR 2.1 of malformations of great vessels, congenital valvular heart disease, and patent ductus arteriosus 22
Table S10: Covariates and model performance for DisMod-MR 2.1 of ventricular septal defect and atrial septal defect 22
Assigning disease sequelae and calculation of years of life lived with disability (YLDs) 22
Table S11: Severity splits: Proportion of each type of CHD with each disabling sequela by age group. 23
References for descriptions presented in supplement 24
Supplemental Figures and Tables 26
Figure S7: Leading causes of death in children under 1 year of age. Figure S7 shows arrow diagrams depicting rank order and mean percentage change of causes of death (by death rate per 100,000) in children under 1 year of age in 1990 and 2017 for each Socio-demographic Index quintile. 26
Figure S8: Year lived with disability due to congenital heart disease in children under 1 year of age showing years of life lived with disability per 100,000 by country in 2016 (top) and by Socio-demographic Index from 1990 to 2017 (bottom). 27
Figure S9: Prevalence (top) and age-standardised rate (bottom) of congenital heart disease at birth by GBD location in 2017 28
Figure S10: Years lived with disability and years of life lost due to congenital heart disease and rheumatic heart disease 29
Table S12. CHD deaths, prevalence by age (<1, 1-4, 5-9, 10-14, 15-19, 20-49, 50+), GBD regions & CHD sub-categories for 1990 and 2017 30
Table S13: Alphabetical listing of all data sources used in estimating cause-specific mortality due to congenital birth defects overall for GBD 2017 67
Table S14: Alphabetical listing of all data sources used in estimating cause-specific mortality due to congenital heart anomalies for GBD 2017 248
Table S15: Alphabetical listing of all data sources used in estimating nonfatal health outcomes (incidence, prevalence, YLDs) due to congenital heart anomalies for GBD 2017 400

Investigators

Meghan S Zimmerman, MD[1,2,*]
Alison Smith, BA[3,*]
Craig A Sable, MD[1 §]
Michelle Marie Echko, BS[4]
Lauren B Wilner, MPH[4]
Helen Elizabeth Olsen, MA[5]
Hagos Tasew Atalay, MSc [6]
Ashish Awasth, PhD[7,8]
Zulfiqar A Bhutta, PhD[9,10]
Jackie LeeAnne Boucher, MSc[11]
Franz Castro, MD[12]
Paolo Angelo Cortesi, PHD[13]
Manisha Dubey, PhD[14]
Florian Fischer, PhD[15]
Samer Hamidi, DrPH[16]
Simon I. Hay, D.Sc[4,17]
Chi Linh Hoang, B.Med.Sc.[18]
Christopher Thomas Hugo-Hamman, BEP[19,20]
Kathy J Jenkins, MD[21,22]
Anita Kar, PhD[23,24]
Ibrahim A. Khalil, MD[4,17]
Raman Krishna Kumar, MD[8,25]
Gene F Kwan, MD[26,27]
Desalegn Tadese Mengistu, MSc[28]
Ali H Mokdad, PhD[4,17]
Mohsen Naghavi, MD[4,17]
Lemma Negesa, MSc[29]
Ionut Negoi, PhD[30]
Ruxandra Irina Negoi, PhD[31,32]
Cuong Tat Nguyen, MPH[33]
Huong Lan Thi Nguyen, MPH[33]
Long Hoang Nguyen, PhD[18]
Son Hoang Nguyen, BS[18]
Trang Huyen Nguyen, MSc[18]
Molly R Nixon, PhD[4]
Jean Jacques Noubiap, MD[34]
Shanti Patel, MD[35]
Emmanuel K Peprah, PhD[36]
Robert C Reiner, PhD[4,17]
Gregory A Roth, MD[4,37]
Mohamad-Hani Temsah, MRCPCH[38,39]
Marcos Roberto Tovani-Palone, MSc[40]
Jeffrey A. Towbin, MD[41]
Bach Xuan Tran, PhD[42]
Tung Thanh Tran, B.Med.Sc.[33]
Nu Thi Truong, B.Hlth.Sci[18]
Theo Vos, PhD[4,17]
Kia Vosoughi, MD[43,44]
Robert G Weintraub, MB[45,46]
Kidu Gidey Weldegwergs, MSc[47]
Zoubida Zaidi, DrPH[48]

Bistra Zheleva, MBA[49]
Liesl J Zuhlke, PhD[19,34]
Christopher J L Murray, DPhil[4,17]
Gerard R Martin, MD[1]
Nicholas J Kassebaum, MD[4,50,]

Affiliations

1. Department of Cardiology, Children's National Health System, Washington, DC, United States.
2. Milken Institute School of Public Health, George Washington University, Washington, DC, United States.
3. School of Medicine, Emory University, Atlanta, GA, USA.
4. Institute for Health Metrics and Evaluation, University of Washington, Seattle, WA, United States.
5. Department of Global Health, Bill & Melinda Gates Foundation, Seattle, , United States.
6. Department of Nursing, Aksum University, Aksum, Tigray, Ethiopia.
7. Indian Institute of Public Health, Gandhinagar, Gujarat, India.
8. Public Health Foundation of India, Gurugram, Haryana, India.
9. The Centre for Global Child Health, Hospital for Sick Children, University of Toronto, Toronto, Ontario, Canada.
10. Center of Excellence in Women and Child Health, Aga Khan University, Karachi, Sindh, Pakistan +.
11. Department of Administration, Children's HeartLink, Edina, MN, United States.
12. Department of Research and Health Technology Assessment, Gorgas Memorial Institute for Health Studies, Panama City, Panama.
13. School of Medicine and Surgery, University of Milan Bicocca, Monza, MB, Italy.
14. United Nations World Food Programme, New Delhi, India.
15. Department of Public Health Medicine, Bielefeld University, Bielefeld, North Rhine-Westphalia, Germany.
16. School of Health and Environmental Studies, Hamdan Bin Mohammed Smart University, Dubai, United Arab Emirates.
17. Department of Health Metrics Sciences, University of Washington, Seattle, WA, United States.
18. Center of Excellence in Behavioral Medicine, Nguyen Tat Thanh University, Ho Chi Minh, Vietnam.
19. Department of Pediatrics and Child Health, University of Cape Town, Cape Town, Western Cape, South Africa.
20. Pediatric Cardiology, Windhoek, Namibia.
21. Center for Applied Pediatric Quality Analytics, Boston Children's Hospital, Boston, MA, United States.
22. Pediatrics, Boston, MA, United States.
23. School of Health Sciences, Savitribai Phule Pune University, Pune, Maharashtra, India.
24. Birth Defects and Public Health Education Group, Maharashtra Association of Anthropological Sciences, Pune, Maharashtra, India.
25. Department of Pediatric Cardiology, Amrita Institute of Medical Sciences, Ernakulam, Kerala, India.
26. School of Medicine, Boston University, Boston, MA, United States.
27. Partners In Health, Boston, MA, United States.
28. School of Medicine, Mekelle University, Mekelle, Tigrai, Ethiopia.
29. Haramaya University, Harar, Ethiopia.
30. Emergency Hospital of Bucharest, Carol Davila University of Medicine and Pharmacy, Bucharest, Romania.
31. Anatomy and Embryology Department, Carol Davila University of Medicine and Pharmacy, Bucharest, Romania.
32. Department of Cardiology, Cardio-Aid, Bucharest, Bucharest, Romania.
33. Institute for Global Health Innovations, Duy Tan University, Hanoi, Vietnam.
34. Department of Medicine, University of Cape Town, Cape Town, Western Cape, South Africa.
35. Department of Medicine, Maimonides Medical Center, Brooklyn, NY, United States.
36. Department of Social and Behavioral Sciences, New York University, New York, NY, United States.
37. Division of Cardiology, University of Washington, Seattle, WA, United States.
38. Department of Pediatrics, King Saud University, Riyadh, Saudi Arabia.
39. College of Medicine, Alfaisal University, Riyadh, Riyadh, Saudi Arabia.
40. Department of Pathology and Legal Medicine, University of São Paulo, Ribeirão Preto, São Paulo, Brazil.
41. Department of Pediatrics, University of Tennessee, Memphis, Tennessee, United States.
42. Department of Health Economics, Hanoi Medical University, Hanoi, Vietnam.
43. Department of Gastroenterology and Hepatology, Johns Hopkins University, Baltimore, MD, United States.
44. Preventive Medicine and Public Health Research Center, Iran University of Medical Sciences, Tehran, Iran.

45. Cardiology Department, Royal Children's Hospital, Melbourne, VIC, Australia.
46. Murdoch Childrens Research Institute, Melbourne, VIC, Australia.
47. Clinical Pharmacy Unit, Mekelle University, Mekelle, Ethiopia.
48. A.C.S. Medical College and Hospital, Algiers, Algeria.
49. Children's HeartLink, Edina, MN, United States.
50. Department of Anesthesiology & Pain Medicine, University of Washington, Seattle, WA, United States.

Summary of general Global Burden of Disease study methods

The text in this section is a reproduction of text used in the supplementary appendices of other manuscripts based on GBD 2017 results. Subsequent sections are written to describe specifics of estimation of the burden of disease due to congenital heart disease. This publication complies with the Guidelines for Accurate and Transparent Health Estimates Reporting (GATHER). A GATHER checklist is in Table S1 below.

The Institute for Health Metrics and Evaluation, with a growing collaboration of scientists, produces annual updates of the Global Burden of Diseases, Injuries, and Risk Factors (GBD) study. Estimates span the period from 1990 to the most recent completed year. By the time of the release of GBD 2017 in November 2018, there were nearly 3,100 collaborators in 140 countries and two territories who contributed to this global public good. Annual updates allow incorporation of new data and method improvements to ensure that the most up-to-date information is available to policy makers in a timely fashion to help make resource allocation decisions.

The guiding principle of GBD is to assess health loss due to mortality and disability comprehensively, where we define disability as any departure from full health. In GBD 2017, estimates were made for 195 countries and territories, and 823 subnational locations, for 28 years starting from 1990, for 23 age groups and both sexes. Deaths were estimated for 282 diseases and injuries, while prevalence and incidence were estimated for 354 diseases and injuries, and exposure and attributable burden estimated for 84 risk factors and clusters of risks. In order to allow meaningful comparisons between deaths and non-fatal disease outcomes as well as between diseases, the data on deaths and prevalence are summarised in a single indicator, the disability-adjusted life-year (DALY). DALYs are the sum of years of life lost (YLLs) and years lived with disability (YLDs). YLLs are estimated as the multiplication of counts of death and a standard, "ideal," remaining life expectancy at the age of death. The standard life expectancy is derived from the lowest observed mortality rates in any population in the world greater than 5 million.[1] YLDs are estimated as the product of prevalence of individual consequences of disease (or "sequelae") times a disability weight that quantifies the relative severity of a sequela as a number between zero (representing "full health") and 1 (representing death). Disability weights have been estimated in nine population surveys and an open-access internet survey in which respondents are asked to choose the "healthier"[2] between random pairs of health states that are presented with a short description of the main features.

All-cause mortality rates are estimated from vital registration data in countries with complete coverage.[1] For other countries, the probabilities of death before age 5 and between ages 15 and 60 are estimated from censuses and surveys asking mothers to provide a history of children ever born and those still alive, and surveys asking adults about siblings who are alive or have passed away. Using model life tables, these probabilities of death are transformed into age-specific death rates by location, year, and sex. GBD has collated a large database of cause of death data from vital registrations and verbal autopsy surveys in which relatives are asked a standard set of questions to ascertain the likely cause of death, supplemented with police and mortuary data for injury deaths in countries with no other data. For countries with vital registration data, the completeness is assessed with demographic methods based on comparing recorded deaths with population counts between two successive censuses.

The cause of death information is provided in a large number of different classification systems based on versions of the International Classification of Diseases or bespoke classifications in some countries.[3] All data are mapped into the disease and injury categories of GBD. All classification systems contain codes that are less informative because they lack a specific diagnosis (eg, unspecified cancer) or refer to codes that cannot be underlying cause of death (eg, low back pain or senility) or are intermediate causes (eg, heart failure or sepsis). Such deaths are redistributed to more precise underlying causes of death. After these redistributions and corrections for under-registration, the data are analyzed using cause of death ensemble modeling (CODEm), a highly systematised tool that runs many different models on the same data and chooses an ensemble of models that best reflects all the available input data. Models are chosen with variations in the statistical approach ("mixed effects" of spatiotemporal Gaussian process regression), in the unit of analysis (rates or cause fractions), and the choice of predictive covariates. The statistical performance of all models is tested by holding out 30% of the data and checking how well a model covers the data that were held out. To enforce consistency from CODEm, the sum of all cause-specific mortality rates is scaled to that of the all-cause mortality rates in each age, sex, location, and year category.

Non-fatal estimates are based on systematic reviews of published papers and unpublished documents, survey microdata, administrative records of health encounters, registries, and disease surveillance systems.[4] These are cataloged in the Global Health Data Exchange (GHDx, http://ghdx.healthdata.org/), the largest repository of health data globally. We first set a reference case definition and/or study method that best quantifies each disease or injury or consequence thereof. If there is evidence of a systematic bias in data that used different case definitions or methods compared to reference data, we adjust those data points to reflect what their value would have been if measured as the reference. This is a necessary step if one wants to use all data pertaining to a particular quantity of interest rather than choosing a small subset of data of the highest quality only. DisMod-MR 2.1, a Bayesian meta-regression tool, is our main method of analysing non-fatal data. It is designed as a geographical cascade where a first model is run on all the world's data, which produces an initial global fit and estimates coefficients for predictor variables and the adjustments for alternative study characteristics. The global fit adjusted by the values of random effects for each of seven GBD super-regions, the coefficients on sex and country predictors, are passed down as data to a model for each super-region together with the input data for that geography. The same steps are repeated going from super-region to 21 region fits and then to 195 fits by country and, where applicable, a further level down to subnational units. Below the global fit, all models are run separately by sex and for six time periods: 1990, 1995, 2000, 2005, 2010, and 2017. During each fit, all data on prevalence, incidence, remission (ie, cure rate), and mortality are forced to be internally consistent. For most diseases, the bulk of data on prevalence or incidence is at the disease level, with fewer studies providing data on the proportions of cases of disease in each of the sequelae defined for the disease. The proportions in each sequela are pooled using DisMod-MR 2.1 or meta-analysis, or derived from analyses of patient-level datasets. The multiplication of prevalent cases for each disease sequela and the appropriate disability weight produces YLD estimates that do not yet take into account comorbidity. To correct for comorbidity, these data are used in a simulation to create hypothetical individuals in each age, sex, location, and year combination who experience no, one, or multiple sequelae simultaneously. We assume that disability weights are multiplicative rather than additive as this avoids assigning a combined disability weight value in any individual to exceed 1, (ie, be worse than a "year lost due to death"). This comorbidity adjustment leads to an average scaling down of disease-specific YLDs ranging from about 2% in young children up to 17% in oldest ages.

All our estimates of causes of death are categorical: each death is assigned to a single underlying cause. This has the attractive property that all estimates add to 100%. For risks, we use a different, "counterfactual" approach,

answering the question: “what would the burden have been if the population had been exposed to a theoretical minimum level of exposure to a risk?” Thus, we need to define what level of exposure to a risk factor leads to the lowest amount of disease. We then analyse data on the prevalence of exposure to a risk and derive relative risks for any risk-outcome pair for which we find sufficient evidence of a causal relationship.[5] Prevalence of exposure is estimated in DisMod-MR 2.1, using spatiotemporal Gaussian process regression, or from satellite imagery in the case of ambient air pollution. Relative risk data are pooled using meta-analysis of cohort, case-control and/or intervention studies. For each risk and outcome pair, we evaluate the evidence and judge if the evidence falls into the categories of “convincing” or “probable” as defined by the World Cancer Research Fund.[6] From the prevalence and relative risk results, population attributable fractions are estimated relative to the theoretical minimum risk exposure level (TMREL). When we aggregate estimates for clusters of risks (eg, metabolic or behavioural risks), we use a multiplicative function rather than simple addition and take into account how much of each risk is mediated through another risk. For instance, some of the risk of high body-mass index is directly onto stroke as an outcome, but much of its impact is mediated through high blood pressure, high cholesterol, or high fasting plasma glucose, and we would not want to double count the mediated effects when we estimate aggregates across risk factors.

Uncertainty is propagated throughout all these calculations by creating 1,000 values for each prevalence, death, YLL, YLD, or DALY estimate and performing aggregations across causes and locations at the level of each of the 1,000 values for all intermediate steps in the calculation. The lower and upper bounds of the 95% uncertainty interval are the 25th and 975th values of the ordered 1,000 values. For all age-standardised rates, GBD uses a standard population calculated as the non-weighted average across all countries of the percentage of the population in each five-year age group.[7]

GBD uses a composite indicator of sociodemographic development to evaluate trends by development status and epidemiological transition. The composite indicator is termed the Socio-demographic Index (SDI) and reflects the principal components analysis (PCA) of normalised values of a location’s income per capita, the average years of schooling in the population 15 and over, and the total fertility rate under 25 years. Countries and territories are grouped into five quintiles of high, high-middle, middle, low-middle, and low SDI based on their 2017 values.[1] A map of each GBD location according to its SDI quintile is shown in Figure S1.

Figure S1. Socio-demographic Index quintiles by GBD administrative Level 1 geography, 2017

All locations are color-coded according to their Socio-demographic Index (SDI) quintile in the year 2017. Subnational locations that are estimated separately for GBD 2017 can belong to different SDI quintiles. SDI is a composite metric of total fertility under 25 years, years of education, and per capita income. SDI=Socio-demographic Index.

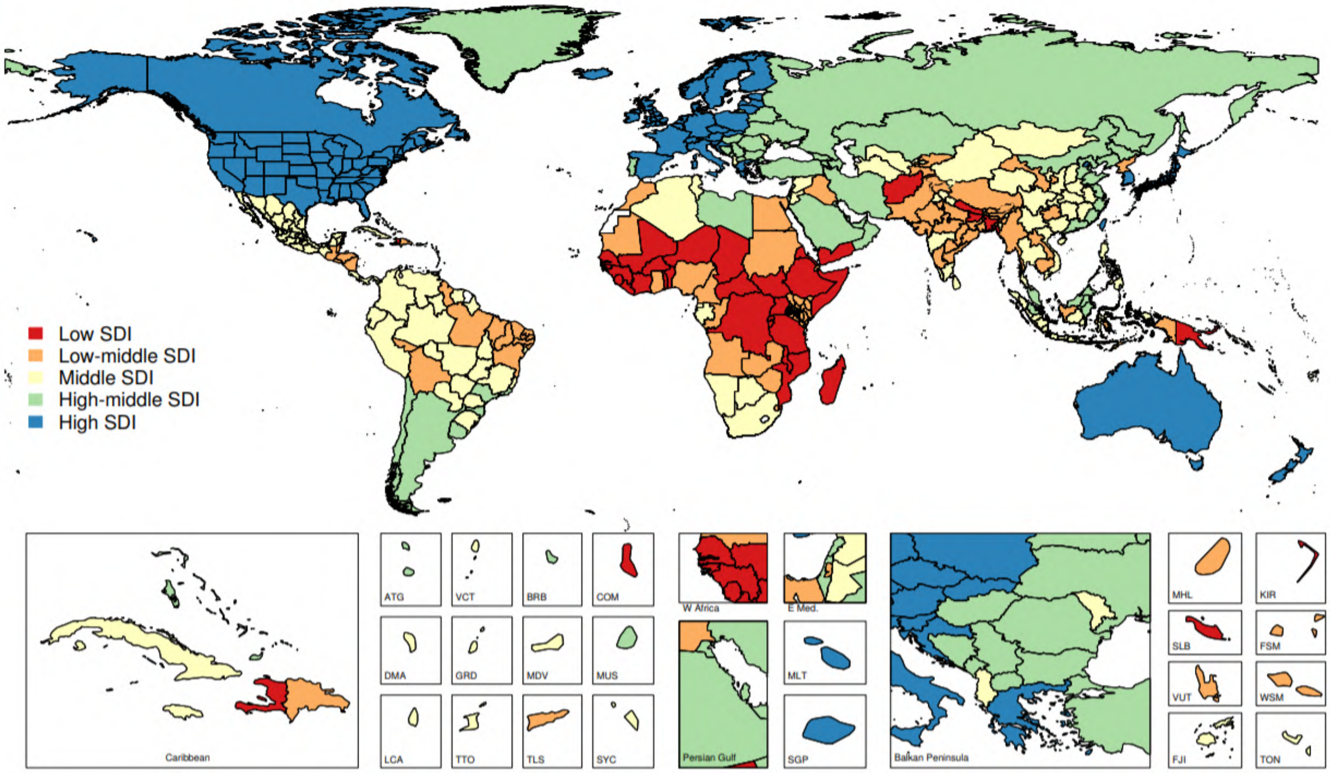

Table S1: GATHER checklist of information that should be included in reports of global health estimates, with description of compliance and location of information for GBD 2017

This table delineates each component of the Guidelines for Accurate and Transparent Health Estimates Reporting (GATHER) statement for how documentation of estimation studies should be completed.

#	GATHER checklist item	Description of compliance	Reference
Objectives and funding			
1	Define the indicators, populations, and time periods for which estimates were made.	Narrative provided in paper and appendix describing indicators, definitions, and populations	Main text (Methods) and appendix
2	List the funding sources for the work.	Funding sources listed in paper	Summary (Funding)
Data Inputs			
For all data inputs from multiple sources that are synthesized as part of the study:			
3	Describe how data were identified and how data were accessed.	Narrative description of data seeking methods provided	Main text (Methods) and appendix
4	Specify inclusion and exclusion criteria. Identify all ad-hoc exclusions.	Narrative about inclusion and exclusion criteria by data type provided; ad hoc exclusions in cause-specific write-ups	Main text (Methods) and appendix
5	Provide information on all included data sources and their main characteristics. For each data source used, report reference	An interactive, online data source tool that provides metadata for data sources	Online data citation tools: http://ghdx.healthdata.org/gb

	information or contact name/institution, population represented, data collection method, year(s) of data collection, sex and age range, diagnostic criteria or measurement method, and sample size, as relevant.	by component, geography, cause, risk, or impairment has been developed	d-2016
6	Identify and describe categories of input data with potentially important biases (e.g. based on characteristics listed in item 5).	Summary of known biases by cause included in appendix	Appendix
For data inputs that contribute to the analysis but were not synthesized as part of the study:			
7	Describe and give sources for any other data inputs.	Included in online data source tool	http://ghdx.healthdata.org/gbd-2017
For all data inputs:			
8	Provide all data inputs in a file format from which data can be efficiently extracted (e.g., a spreadsheet as opposed to a PDF), including all relevant meta-data listed in item 5. For any data inputs that cannot be shared due to ethical or legal reasons, such as third-party ownership, provide a contact name or the name of the institution that retains the right to the data.	Downloads of input data available through online tools, including data visualization tools and data query tools; input data not available in tools will be made available upon request	Online data visualisation tools, data query tools, and the Global Health Data Exchange
**Data analysis**			
9	Provide a conceptual overview of the data analysis method. A diagram may be helpful.	Flow diagrams of the overall methodological processes, as well as cause-specific modelling processes, have been provided	Main text (Methods) and appendix
10	Provide a detailed description of all steps of the analysis, including mathematical formulae. This description should cover, as relevant, data cleaning, data pre-processing, data adjustments and weighting of data sources, and mathematical or statistical model(s).	Flow diagrams and corresponding methodological write-ups for each cause, as well as the databases and modelling processes, have been provided	Main text (Methods) and appendix
11	Describe how candidate models were evaluated and how the final model(s) were selected.	Provided in the methodological write-ups	Appendix
12	Provide the results of an evaluation of model performance, if done, as well as the results of any relevant sensitivity analysis.	Provided in the methodological write-ups	Appendix
13	Describe methods for calculating uncertainty of the estimates. State which sources of uncertainty were, and were not, accounted for in the uncertainty analysis.	Appendix	Appendix
14	State how analytic or statistical source code used to generate estimates can be accessed.	Appendix	http://ghdx.healthdata.org/gbd-2017-code
**Results and Discussion**			
15	Provide published estimates in a file format from which data can be efficiently extracted.	GBD 2017 results are available through online data visualization tools, the Global Health Data Exchange, and the online data query tool	Main text, and online data tools (data visualisation tools, data query tools, and the Global Health Data Exchange)
16	Report a quantitative measure of the uncertainty of the estimates (e.g., uncertainty intervals).	Uncertainty intervals are provided with all results	Main text, appendix, and online data tools (data visualisation tools, data query tools, and the Global Health Data Exchange)
17	Interpret results in light of existing evidence. If updating a previous set of estimates, describe the reasons for changes in estimates.	Discussion of methodological changes between GBD rounds provided in the narrative of the manuscript and appendix	Main text (Methods and Discussion) and appendix
18	Discuss limitations of the estimates. Include a discussion of any modelling assumptions or data limitations that affect interpretation of the estimates.	Discussion of limitations provided in the narrative of the main paper, as well as in the methodological write-ups in the appendix	Main text (Limitations) and appendix

Congenital heart disease: overview, definitions, and cause classification

Cause-specific mortality overview

No significant changes were made to the cause-specific mortality estimation process for congenital heart disease in GBD 2017. Each death in GBD is assigned to a single underlying cause. Mortality was limited to the age range from birth up to 70 years for all types of congenital birth defects. We started by estimating total congenital birth defects mortality using CODEm (described above). Nine subtypes of congenital birth defects were then estimated separately using CODEm, with the sum of deaths from each scaled to match the total for all congenital birth defects and total congenital birth defects summed with all other specific causes of death to equal all-cause mortality for each age group, sex, location, and year. Subcauses of congenital birth defects included 1) neural tube defects, 2) ***congenital heart anomalies***, 3) orofacial clefts, 4) Down syndrome, 5) other chromosomal anomalies, 6) congenital musculoskeletal anomalies, 7) urogenital congenital anomalies, 8) digestive congenital anomalies, and 9) other congenital birth defects. Two additional types of congenital birth defects – Klinefelter syndrome and Turner syndrome – were estimated as causing non-fatal disease burden only.

Non-fatal health outcomes overview

We substantially revised the estimation strategy for non-fatal health outcomes across all types of congenital birth defects, with important ramifications for the assessment of non-fatal CHD burden in particular.

First, in GBD 2010, GBD 2013, and GBD 2015, only *isolated* birth defects were included in the case definition of each model, so all those individuals with coexistent chromosomal or genetic disorders were not captured, which may have substantially underestimated the number of prevalent cases of CHD in particular. This was revised starting in GBD 2016 so that the reference case definition includes all persons with each defect.

Second, prior to GBD 2016, CHD were all subdivided into one of three broad categories – critical, severe, and less-severe – and prevalence was estimated based solely on congenital birth registry data. Long-term CHD prevalence was assumed to vary as a function of access to three tiers of medical facilities: “no” care, “supportive” care, or “modern” care. Survival probabilities in each group were derived from expert opinion (no care group) or via global application of standardised mortality ratio (SMR) observations from high-income locations,[8-11] with the added assumption that treatment availability (and therefore survival) for CHD tracks linearly with overall neonatal mortality ratio. While this mechanistic modelling approach produced consistent results that were easily interpretable, they lacked a true empirical basis. Starting with GBD 2016, in consultation with GBD experts and paediatric cardiology experts, we have reorganised cause categories for non-fatal estimates to be more anatomically similar, clinically relevant, and structured in a way to facilitate incorporation of literature, registry, and administrative data to inform levels and trends. It was also developed with two additional constraints, namely to minimise how many 4-digit and 5-digit ICD-9 and ICD-10 codes were split between different groups and to also maximise the matching with reporting groups in major congenital birth defect registry systems (eg, EUROCAT). These five CHD categories are named descriptively as the following, and ICD-9 and ICD-10 codes mapped to each are shown in Table S2:

1. Single ventricle and single ventricle pathway defects
2. Complex congenital heart defects excluding single ventricle and single ventricle pathway defects
3. Malformations of great vessels, congenital valvular heart disease, and patent ductus arteriosus
4. Ventricular septal defect and atrial septal defect
5. Other congenital cardiovascular anomalies

Third, disabling sequelae for CHD prior to GBD 2016 were limited to heart failure and a generic health state referred to as “congenital heart disease” that was derived from analysis of survey responses to the Medical Expenditure Panel Survey in the United States as described previously. We completed systematic literature reviews as described below for GBD 2016 to facilitate quantification of additional common outcomes that occur as a result of CHD, most notably developmental intellectual disability.

Table S2: International Classification of Diseases version 9 (ICD-9) and 10 (ICD-10) mapping to categories of congenital heart disease

ICD-10	ICD-10 name	ICD-9	ICD-9 name
**Malformations of great vessels, congenital valvular heart disease and patent ductus arteriosus**			
**Q22.1**	Congenital pulmonary valve stenosis	**746.0**	Anomalies of pulmonary valve
**Q22.2**	Congenital pulmonary valve insufficiency	**746.00**	Pulmonary valve anomaly, unspecified
**Q22.3**	Other congenital malformations of pulmonary valve	**746.02**	Stenosis, congenital
**Q22.5**	Ebstein’s anomaly	**746.09**	Other
**Q22.8**	Other congenital malformations of tricuspid valve	**746.2**	Ebstein’s anomaly
**Q22.9**	Congenital malformation of tricuspid valve, unspecified	**746.5**	Congenital mitral stenosis
**Q23.2**	Congenital mitral stenosis	**746.6**	Congenital mitral insufficiency
**Q23.3**	Congenital mitral insufficiency	**746.8**	Other specified anomalies of heart
**Q23.8**	Other congenital malformations of aortic and mitral valves	**746.81**	Subaortic stenosis
**Q23.9**	Congenital malformation of aortic and mitral valves, unspecified	**746.83**	Infundibular pulmonic stenosis
**Q25.0**	Patent ductus arteriosus	**746.84**	Obstructive anomalies of heart, NEC
**Q25.1**	Coarctation of aorta	**747.0**	Patent ductus arteriosus
**Q25.2**	Atresia of aorta	**747.1**	Coarctation of aorta
**Q25.3**	Supravalvular aortic stenosis	**747.10**	Coarctation of aorta (preductal) (postductal)
**Q25.4**	Other congenital malformations of aorta	**747.11**	Interruption of aortic arch
**Q25.5**	Atresia of pulmonary artery	**747.2**	Other anomalies of aorta
		**747.20**	Anomaly of aorta, unspecified
		**747.21**	Anomalies of aortic arch
		**747.22**	Atresia and stenosis of aorta
**Complex congenital heart defects excluding single ventricle and single ventricle pathway**			
**Q20.0**	Common arterial trunk	**745.0**	Common truncus
**Q20.1**	Double outlet right ventricle	**745.1**	Transposition of great vessels
**Q20.2**	Double outlet left ventricle	**745.10**	Complete transposition of great vessels
**Q20.3**	Discordant ventriculoarterial connection	**745.11**	Double outlet right ventricle
**Q21.3**	Tetralogy of Fallot	**745.12**	Corrected transposition of great vessels
**Q22.0**	Pulmonary valve atresia	**745.19**	Other
**Q23.0**	Congenital stenosis of aortic valve	**745.2**	Tetralogy of Fallot
**Q23.1**	Congenital insufficiency of aortic valve	**746.01**	Atresia, congenital
**Q26.2**	Total anomalous pulmonary venous connection	**746.3**	Congenital stenosis of aortic valve
		**746.4**	Congenital insufficiency of aortic valve
		**747.41**	Total anomalous pulmonary venous connection
**Single ventricle and single ventricle pathway heart defects**			
**Q20.4**	Double inlet ventricle	**745.3**	Common ventricle
**Q22.4**	Congenital tricuspid stenosis	**745.7**	Cor biloculare
**Q22.6**	Hypoplastic right heart syndrome	**746.1**	Tricuspid atresia and stenosis, congenital
**Q23.4**	Hypoplastic left heart syndrome	**746.7**	Hypoplastic left heart syndrome
**Ventricular septal defect and atrial septal defect**			
**Q21.0**	Ventricular septal defect	**745.4**	Ventricular septal defect
**Q21.1**	Atrial septal defect	**745.5**	Ostium secundum type atrial septal defect
**Q21.2**	Atrioventricular septal defect	**745.6**	Endocardial cushion defects
**Q21.4**	Aortopulmonary septal defect	**745.60**	Endocardial cushion defect, unspecified type
**Q21.8**	Other congenital malformations of cardiac septa	**745.61**	Ostium primum defect
**Q21.9**	Congenital malformation of cardiac septum, unspecified	**745.69**	Other
**Q26.3**	Partial anomalous pulmonary venous connection	**745.8**	Other
		**745.9**	Unspecified defect of septal closure
		**747.42**	Partial anomalous pulmonary venous connection
**Other congenital heart and cardiovascular anomalies**			
**Q20.5**	Discordant atrioventricular connection	**746.82**	Cor triatriatum
**Q20.6**	Isomerism of atrial appendages	**746.85**	Coronary artery anomaly
**Q20.8**	Other congenital malformations of cardiac chambers and connections	**746.86**	Congenital heart block

**Q20.9**	Congenital malformation of cardiac chambers and connections, unspecified	**746.87**	Malposition of heart and cardiac apex
**Q24**	Other congenital malformations of heart	**746.89**	Other
**Q24.0**	Dextrocardia	**746.9**	Unspecified anomaly of heart
**Q24.1**	Levocardia	**747**	Other congenital anomalies of circulatory system
**Q24.2**	Cor triatriatum	**747.3**	Anomalies of pulmonary artery
**Q24.3**	Pulmonary infundibular stenosis	**747.4**	Anomalies of great veins
**Q24.4**	Congenital subaortic stenosis	**747.40**	Anomaly of great veins, unspecified
**Q24.5**	Malformation of coronary vessels	**747.49**	Other anomalies of great veins
**Q24.6**	Congenital heart block	**747.5**	Absence or hypoplasia of umbilical artery
**Q24.8**	Other specified congenital malformations of heart	**747.6**	Other anomalies of peripheral vascular system
**Q24.9**	Congenital malformation of heart, unspecified	**747.60**	Anomaly of the peripheral vascular system, unspecified site
**Q25.6**	Stenosis of pulmonary artery	**747.61**	Gastro-intestinal vessel anomaly
**Q25.7**	Other congenital malformations of pulmonary artery	**747.62**	Renal vessel anomaly
**Q25.71**	Coarctation of pulmonary artery	**747.63**	Upper limb vessel anomaly
**Q25.72**	Congenital pulmonary arteriovenous malformation	**747.64**	Lower limb vessel anomaly
**Q25.79**	Other congenital malformations of pulmonary artery	**747.69**	Anomalies of other specified sites of peripheral vascular system
**Q25.8**	Other congenital malformations of other great arteries	**747.8**	Other specified anomalies of circulatory system
**Q25.9**	Congenital malformation of great arteries, unspecified	**747.81**	Anomalies of cerebrovascular system
**Q26.0**	Congenital stenosis of vena cava	**747.82**	Spinal vessel anomaly
**Q26.1**	Persistent left superior vena cava	**747.83**	Spinal vessel anomaly
**Q26.4**	Anomalous pulmonary venous connection, unspecified	**747.89**	Other
**Q26.5**	Anomalous portal venous connection	**747.9**	Unspecified anomaly of circulatory system
**Q26.6**	Portal vein-hepatic artery fistula		
**Q26.8**	Other congenital malformations of great veins		
**Q26.9**	Congenital malformation of great vein, unspecified		
**Q27**	Other congenital malformations of peripheral vascular system		
**Q27.1**	Congenital renal artery stenosis		
**Q27.2**	Other congenital malformations of renal artery		
**Q27.3**	Arteriovenous malformation (peripheral)		
**Q27.30**	Arteriovenous malformation, site unspecified		
**Q27.31**	Arteriovenous malformation of vessel of upper limb		
**Q27.32**	Arteriovenous malformation of vessel of lower limb		
**Q27.33**	Arteriovenous malformation of digestive system vessel		
**Q27.34**	Arteriovenous malformation of renal vessel		
**Q27.39**	Arteriovenous malformation, other site		
**Q27.4**	Congenital phlebectasia		
**Q27.8**	Other specified congenital malformations of peripheral vascular system		
**Q27.9**	Congenital malformation of peripheral vascular system, unspecified		
**Q28**	Other congenital malformations of circulatory system		
**Q28.0**	Arteriovenous malformation of precerebral vessels		
**Q28.1**	Other malformations of precerebral vessels		
**Q28.2**	Arteriovenous malformation of cerebral vessels		
**Q28.3**	Other malformations of cerebral vessels		
**Q28.8**	Other specified congenital malformations of circulatory system		
**Q28.9**	Congenital malformation of circulatory system, unspecified		

Overview of data availability for congenital heart disease

The subsequent sections describe the sources and processing of data sources for estimation of CHD burden. Availability of data on fatal and non-fatal cases of CHD varied widely across countries and regions as can be seen in Figure S2. Temporal coverage of data by GBD region for each of mortality and non-fatal analyses are shown in Figure S3. Cause-specific mortality data were identified from the majority of countries globally, but from few countries in Africa, southeast Asia, or Oceania. Non-fatal data were identified from a few additional lower SDI countries, although the temporal coverage of non-fatal data was categorically lower than that of mortality data.

Figure S2: Classification of countries according to the availability of data on mortality and non-fatal cases of congenital heart disease, 1990–2017

This map shows availability of data at the country level according to presence of data for cause-specific mortality only (purple), non-fatal prevalence data only (orange), or both (green). Countries with no mortality data or non-fatal data from 1990 to 2017, inclusive, are coloured in yellow.

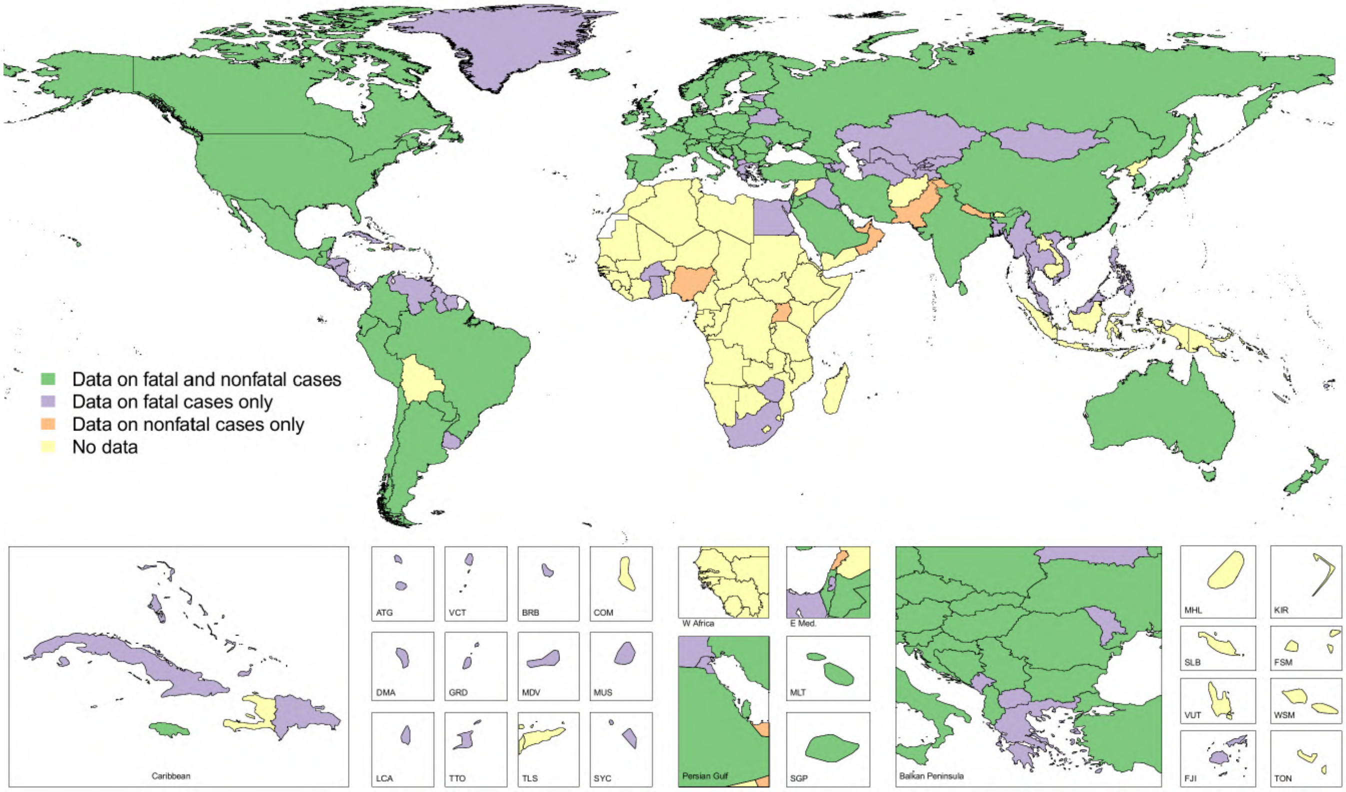

Figure S3: Data representativeness by region and year, 1980–2017, mortality and non-fatal data

*Marker size corresponds to the number of site-years of data available in a given year for cause-specific mortality (top panel) and non-fatal models (bottom panel) from 1980 through 2017. Site-years of data for mortality and non-fatal modelling are shown. Marker size corresponds to the number of site-years of data available. GBD 2017 used the term "site-year" to refer to an individual geographical dataset (ie, community-based, subnational, or nationally representative datasets) in a given year. Marker size may differ from legend because data are continuous. *Non-fatal models contain data representative of the Latin America and Caribbean super-region, but site-years are not shown in this figure.*

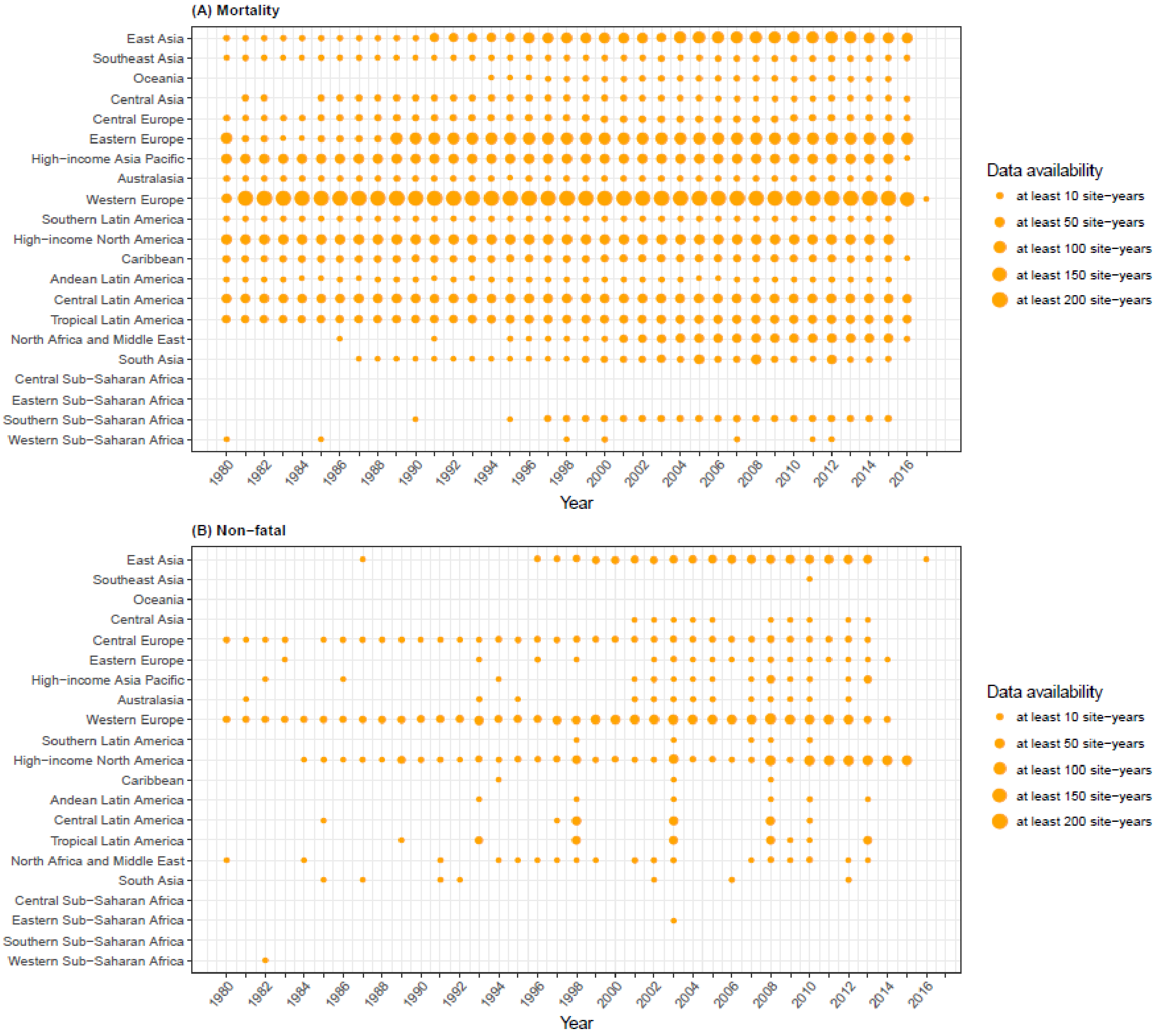

Supplemental methods: Cause-specific mortality of congenital heart disease

A flowchart illustrating the estimation process is shown in Figure S4

Figure S4: Estimation flowchart for cause-specific mortality due to congenital heart disease

The flowchart indicates the input data, databases, results, and processes used to estimate cause-specific mortality due to congenital heart disease. Colour-coding indicates the domain of GBD estimation in which each item is classified.

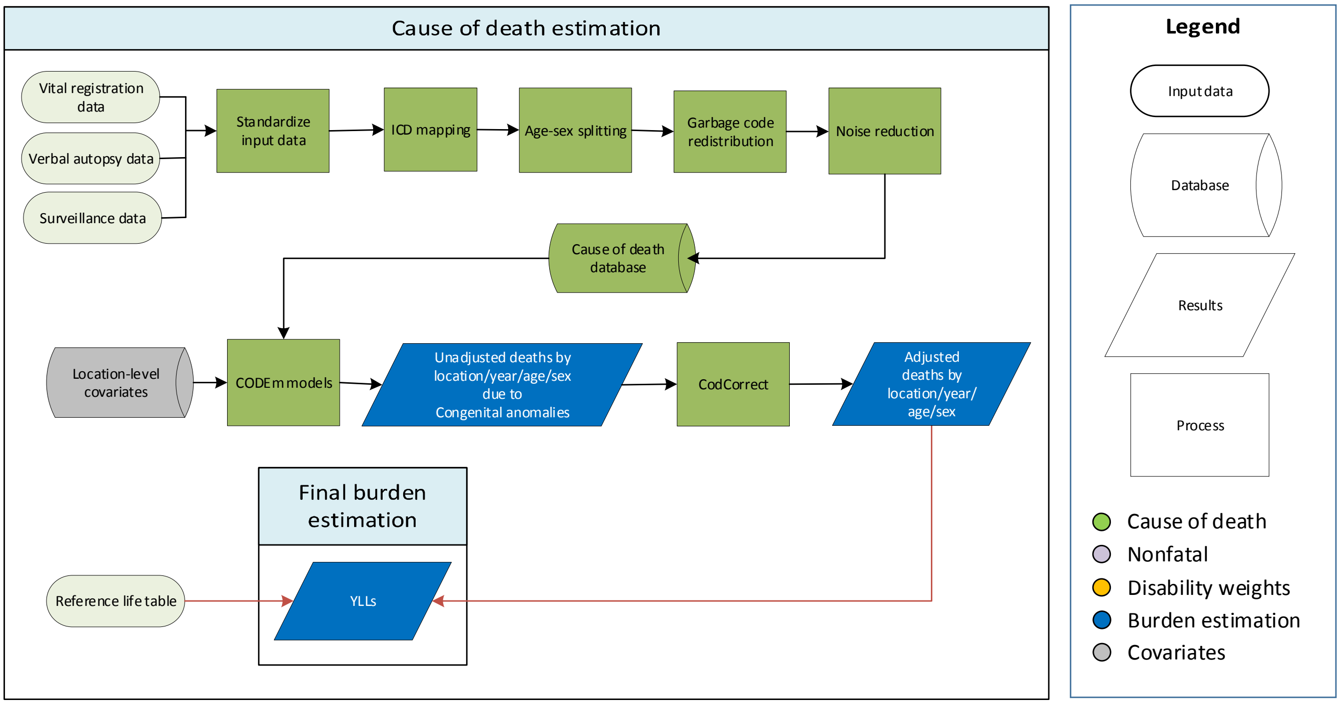

Input data

Deaths due to CHD were estimated using a common cause-specific mortality framework developed for GBD 2017. Each death was assigned to a single underlying cause. All data were centrally extracted, mapped to specific GBD causes, processed, and stored in causes of death (CoD) database. Those CoD data sources that specified the sub-cause of birth defect were included in estimation of both the parent congenital anomalies model as well as in sub-type-specific models. Vital registration (VR) was the dominant data type, followed by verbal autopsy (VA) and surveillance systems. Deaths assigned to ill-defined or nonspecific causes (eg, “heart disease, unspecified” [ICD-10 code I51.9]) or intermediate causes (eg, “heart failure” [ICD-10 code I50]) were reassigned to likely causes of death, including CHD, with the use of statistical redistribution algorithms. Illustrations of redistribution for ICD-9 and ICD-10 in the United States are shown in Figure S5.

Figure S5: ICD-9 (left) and ICD-10 (right) redistribution of ill-defined deaths to congenital heart disease for USA, all years, all ages, both sexes (original on left, final after redistribution on right)

The Sankey diagram illustrates the quantitative effects of redistribution of ill-defined deaths in the GBD cause of death data processing algorithms. The left panel shows ICD-9 and the right panel shows ICD-10 for all years in the USA. The left side of each panel shows the original cause of death as coded, while the right shows the final GBD cause to which those deaths were assigned. The relative size of each band corresponds to the number of deaths. (D=disease. S&S=signs and symptoms. HF=heart failure. RHF=right heart failure; Gi=gastrointestinal.)

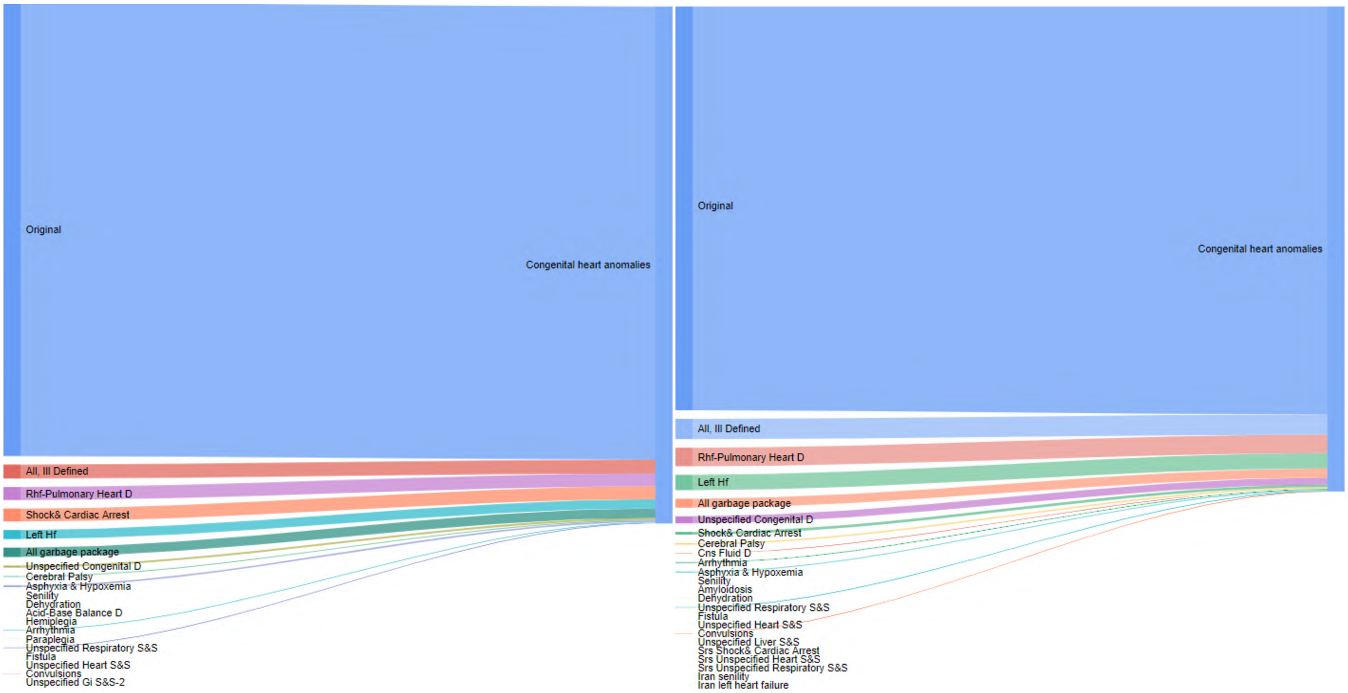

Data evaluation and model specification

For GBD 2017, data exclusions were limited. We outliered all VA data in those over 5 years old as the age patterns were unreliable and led to poor model performance in the under-5 age groups. We also excluded some data sources from the parent model where only a subset of sub-causes were specified (eg, congenital heart disease, neural tube defects, and other congenital anomalies) and the sum of the sub-causes clearly represented systematic underreporting of one of the sub-causes. Systematic underreporting was suspected when sex- and age-specific rates were more than an order of magnitude lower than neighbouring or comparable locations. Data sources for those locations were still included by default for sub-cause-specific models because underreporting of the total was not assumed to necessarily be associated with underreporting of all of the component conditions.

Tables S3 and S4, below, list the covariates used in CODEm models for total congenital birth defects and CHD, respectively. Covariates having a specified positive correlation with CHD mortality were maternal alcohol consumption, proportion of live births in women aged 35+, age-standardised diabetes prevalence, indoor air pollution, and reproductive age-standardised smoking prevalence. Covariates having a specified negative correlation with CHD mortality were measles vaccine coverage, education (years per capita), and legality of abortion. Directionality with log-transformed lag distributed income per capita (LN-LDI) and SDI was not pre-specified. CODEm uses a train-test-test approach to model development. Only those covariates where the relationship with observed data (in-sample) was statistically significant at the p<0.05 level and in the specified direction were retained. All combinations of covariates were tested in separate candidate regression models, retaining only those models where effect sizes on all covariates were significant and in the specified direction. All retained models were ranked based on out-of-sample predictive validity for a first set of held-out data. Rankings were used to construct weighted ensembles of component models. Ensembles and component models were then ranked on a second set of held-out data. The top-ranked model was selected to generate the final estimates. There is no *a priori* requirement that the final model is an ensemble, but generally ensembles performed better than component models. This process was then repeated 30 times with different structured

holdout patterns to ensure stability of final estimates. Input data and model results are publicly available in the Cause of Death Visualization Tool (http://vizhub.healthdata.org/cod).

Table S3: Covariates included in CODEm model of overall congenital birth defects

Covariate	Transformation	Level	Direction
Maternal alcohol consumption during pregnancy (proportion)	None	1	Positive
In-facility delivery (proportion)	None	1	Negative
Live births 35+ (proportion)	None	1	Positive
Folic acid unadjusted (µg)	None	1	Negative
Legality of abortion	None	2	Negative
Antenatal care (1 visit) coverage (proportion)	None	2	Not specified
Smoking prevalence (reproductive age-standardized)	None	2	Positive
Antenatal care (4 visits) coverage (proportion)	None	2	Negative
Healthcare access and quality index	None	2	Negative
Education (years per capita)	None	2	Negative
Alcohol (liters per capita)	None	3	Positive
Fruits unadjusted (g)	None	3	Positive
Outdoor air pollution ($PM_{2.5}$)	None	3	Positive
Indoor air pollution (all cooking fuels)	None	3	Positive
Socio-demographic Index	None	3	Negative
Vegetables unadjusted (g)	None	3	Positive

Table S4: Covariates included in CODEm model of congenital heart disease

Covariate	Transformation	Level	Direction
Maternal alcohol consumption during pregnancy (proportion)	None	1	Positive
Socio-demographic Index	None	2	Negative
Smoking prevalence (reproductive age-standardized)	None	2	Positive
Diabetes age-standardized prevalence (proportion)	None	2	Positive
Healthcare access and quality index	None	2	Negative
Legality of abortion	None	2	Negative
Antenatal care (1 visit) coverage (proportion)	None	2	Negative
In-facility delivery (proportion)	None	2	Negative
Education (years per capita)	None	2	Negative
Alcohol (liters per capita)	None	3	Positive
Antenatal care (4 visits) coverage (proportion)	None	3	Negative
Skilled birth attendance (proportion)	None	3	Negative
Live births 35+ (proportion)	None	3	Positive

CoDCorrect and calculation of years of life lost (YLLs)

As described above, after completion of all cause-specific mortality models, the results are summed and scaled to match total all-cause mortality for each age group, sex, location, and year. Scaling is performed hierarchically so the sum of congenital subcauses equals the total for congenital birth defects after the latter is scaled to the all-cause mortality envelope. YLLs are calculated as the number of deaths at a specific age multiplied by remaining life expectancy at the age of death. Remaining life expectancy is based on a global standard as described above.

Supplemental methods: Non-fatal health loss due to congenital heart disease

A flowchart illustrating the estimation process is shown in Figure S6.

Figure S6: Estimation flowchart for estimation of prevalence and YLDs due to congenital cardiovascular birth defects

This flowchart illustrates the input data, databases, results, and processes used to estimate non-fatal health loss due to congenital heart disease. Colour-coding corresponds to the domain of GBD estimation in which each component is classified. ASD=atrial septal defect. CSMR=cause-specific mortality rate. DALY=disability-adjusted life-year. EUROCAT=European surveillance of congenital anomalies. HAQ=Healthcare access and quality. ICBDSR=International clearinghouse for birth defects surveillance and research. LM-LDI=natural log-transformed lag-distributed income. NBDPN=National birth defects prevention network. VSD=ventricular septal defect. YLD=year of life lived with disability. YLL=year of life lost.

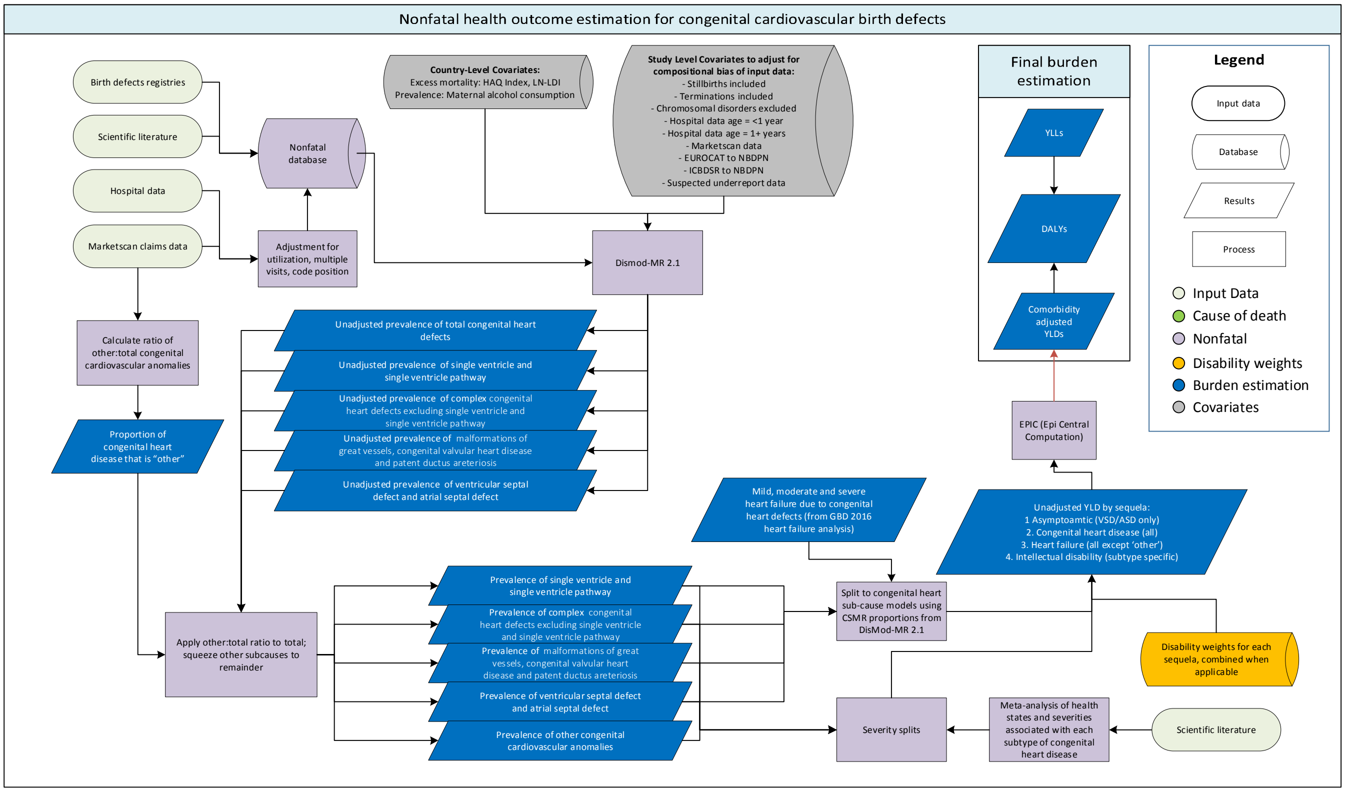

Input data

The GBD case definition of congenital anomalies includes any condition present at birth that is a result of abnormalities of embryonic development, excluding those that are directly the result of infections or substance abuse (eg, fetal alcohol syndrome, congenital syphilis) and excludes minor anomalies as they are defined by EUROCAT. Further, our GBD case definition includes only live births and excludes all terminations of pregnancy following prenatal diagnosis and stillbirths. Datasets for estimating CHD epidemiology were informed by three mechanisms: 1) systematic literature review for prevalence, excess mortality, and with-condition mortality, 2) congenital birth defects registries, and 3) hospital administrative data.

First, we conducted a systematic review of the available literature for all types of congenital anomalies by constructing search strings designed to capture information on the prevalence, associated mortality, and long-term health outcomes associated with each sub-category of congenital anomalies. Searches were limited to PubMed. The search was performed on September 13, 2016, with the following search string:

> ("Heart Defects, Congenital"[MeSH] OR “patent ductus arteriosus”[Title/Abstract] OR "Ebstein's anomaly"[Title/Abstract] OR "Ebstein anomaly"[Title/Abstract] OR "tetralogy of fallot"[Title/Abstract] OR "total anomalous pulmonary venous connection"[Title/Abstract] OR “total veins”[Title/Abstract] OR (“congenital”[Title/Abstract] AND (aorta[Title/Abstract] OR aortic[Title/Abstract] OR valve[Title/Abstract] OR conotruncal[Title/Abstract] OR “cono-truncal”[Title/Abstract] OR “great vessels”[Title/Abstract] OR “great arteries”[Title/Abstract] OR “truncus”[Title/Abstract] OR ventricle[Title/Abstract] OR ventricular[Title/Abstract] OR septal[Title/Abstract] OR septum[Title/Abstract] OR tricuspid[Title/Abstract] OR mitral[Title/Abstract] OR pulmonary[Title/Abstract] OR endocardial[Title/Abstract] OR heart[Title/Abstract]) AND (transposition[Title/Abstract] OR atresia[Title/Abstract] OR stenosis[Title/Abstract] OR stenotic[Title/Abstract] OR stenosis[Title/Abstract] OR defect[Title/Abstract] OR insufficiency[Title/Abstract] OR single[Title/Abstract] OR hypoplastic[Title/Abstract]))) AND (mortality[Title/Abstract] OR survival[Title/Abstract] OR prevalen*[Title/Abstract] OR inciden*[Title/Abstract]) AND (2013/11/01[PDAT]:2016[PDAT]) NOT (hydroceph*[Title/Abstract] OR "Case Reports"[Publication Type] OR “Review”[Publication Type] OR “case report”[Title/Abstract] OR transplant*[Title/Abstract] OR polymorphism[Title/Abstract] OR “cat”[Title/Abstract] OR “dog” [Title/Abstract] OR “rabbit”[Title/Abstract] OR “mouse”[Title/Abstract] OR “mice”[Title/Abstract] OR “rat”[Title/Abstract] OR “porcine” [Title/Abstract] OR “novel”[Title] OR “single institution”[Title/Abstract])

A total of 2,083 titles and abstracts were screened, of which 88 were selected for full-text review to ensure the availability of required information and the representativeness of the reported population, and the exclusion of duplicate data also reported as part of the birth registry data inputs. A total of 27 sources were extracted. Data were identified primarily from scientific publications based on community-based cross-sectional and cohort studies and population-representative administrative datasets.

Second, we supplemented with birth prevalence data from congenital birth defects registries, including the European Surveillance of Congenital Anomalies (EUROCAT), International Clearinghouse for Birth Defects Surveillance and Research (ICBDSR), The World Atlas of Birth Defects which largely covers earlier years for countries reporting to ICBDSR, Birth Defects Registry of India (BDRI) from participating facilities, China Maternal and Child Health Surveillance (MCHS), and the ICD-coded National Birth Defects Prevention Network (NBDPN) for participating states in the USA. The Birth Defects Registry of India (BDRI) reports congenital anomalies from participating hospitals within India. Registry data from countries where GBD subnational analyses were completed were assigned to the most detailed location available in the dataset; if only national data were available, prevalence values were copied to each of the subnationals and sample sizes were weighted proportional to population.

Third, GBD 2017 incorporated administrative data from ICD-coded hospital discharges, outpatient visits, and insurance claims from USA MarketScan. These data were adjusted for overall inpatient utilisation as well as readmission rates, primary versus non-primary discharge code position, and inpatient-to-outpatient ratio as previously described.[4] All adjustment factors were based on analysis of claims data sources.

Table S5 below, along with Figures S2 and S3 above, summarise the volume of data included in the final models. Each model's data and fit can be examined in the GBD online epi visualization tool (http://vizhub.healthdata.org/epi).

Table S5: Prevalence dataset contents for each of the congenital heart defects non-fatal models

Model	Site-years (total)	Number of countries with data	Number of GBD regions with data (out of 21 regions)	Number of GBD super-regions with data (out of 7 super-regions)
**Congenital heart (parent model)**	1618	64	16	7
**Single ventricle and single ventricle pathway heart defects**	1332	48	16	7
**Malformations of great vessels, valvular heart disease, and patent ductus arteriosus**	921	40	14	6
**Ventricular septal defect and atrial septal defect**	1142	44	16	7
**Complex congenital heart defects excluding single ventricle and single ventricle pathway**	1344	45	15	6

Data evaluation and model specification

Five separate DisMod-MR 2.1 models were developed for each of the CHD categories including 1) total congenital cardiovascular anomalies, 2) atrial septal defect and ventricular septal defect (ASD/VSD), 3) single ventricle and single ventricle pathway, 4) complex CHD excluding single ventricle defects, and 5) malformations of the great vessels including patent ductus arteriosus (PDA). DisMod-MR 2.1 is a compartmental model consisting of three states – susceptible, diseased, and dead – with state transitions determined by the rates of incidence, remission, excess mortality, and other-cause mortality.[12] DisMod-MR 2.1 synthesises all available data and covariates using differential equations with appropriate boundary conditions to generate internally consistent results for all disease parameters. The tool uses an offset log-normal model with nested random effects on GBD location and fixed effects on both location-specific covariates and study covariates denoting study characteristics causing deviation from a reference category (ie, crosswalks as described below).

The reference category for each model was ICD-coded registry data and literature data based on the judgement that the reporting in these sources was most likely to be complete. China MCHS and USA NBDPN were the only registries where data were ICD-coded. All other registries report only a subset of CHD in their statistical reports, although all specify which ICD codes or CHD lesions are included. To facilitate adjustment for this compositional bias, we generated a series of "dummy" extractions using NBDPN data that matched the subset of codes used in each of the other registries. MCHS data were tabulated by GBD collaborators in country according to the map in Table S2, so we did not have access to individual-level data to make similar extracts for it. All hospital and claims data for 20 years and older were excluded based on the observation from a number of different sources of an up to ten-fold decline in prevalence when transitioning from 15-19 years to 20-24 years, a difference we considered implausible. All other data were evaluated visually and, if considered implausible, were outliered. All data with a prevalence value of zero at birth were outliered; all those with prevalence of <3 per 1,000 for total congenital cardiovascular anomalies were identified with a study covariate to denote suspected underreporting; these data were identified with a second study covariate to denote suspected sampling error as well. Hospital data for under 1 year of age and for over 1 were identified with separate study covariates to allow for differential calculation of crosswalk coefficients. Claims data from MarketScan were crosswalked for all models except total congenital cardiovascular anomalies and single ventricle and single ventricle pathway – these crosswalks were tested but had no effect in the models.

For most of the severe congenital conditions, the mortality associated with the condition is highly dependent on access to adequate surgical interventions and other medical care during the first hours, weeks, and years of life. Location-level covariates were the same for all models – log-transformed lag-distributed income (LDI) per capita and Healthcare Access and Quality (HAQ) Index[13] on excess mortality and maternal alcohol consumption on prevalence for all models. Incidence was set to zero for all congenital models, as congenital conditions occur at the time of birth and by definition there are no incident cases after birth. Remission was bounded to zero for all subcause models except ASD/VSD, where it was constrained to be between 0% and 20% per year until age 10 years based on the values that corresponded to observed spontaneous closure in longitudinal studies.[14,15] Cause-specific priors and slope priors were used to guide biologically plausible DisMod-MR 2.1 estimates of excess mortality and remission where applicable. Random effects limits were bounded to +/- 0.5 for all models except VSD/ASD, which was set to +/- 0.3. For a subset of conditions, a decreasing slope prior on excess mortality rate was applied to capture the highest risk of mortality from congenital conditions in the neonatal age groups and a subsequent decreasing risk of mortality from congenital conditions later in life. Excess mortality was set to zero after age 70 for all models in keeping with the GBD cause of death estimates for all congenital causes. Specific study-level covariates (i.e., crosswalks), value prior settings, and model performance statistics for each of the five models are described below.

We then used age- and sex-specific prevalence ratios derived from the MarketScan inpatient claims data to derive the proportion of the total CHD that is "other congenital cardiovascular disease." This proportion was applied to the total CHD model results and the sum of prevalence by age group, sex, year, and location of the other four categories was scaled with it to match the total prevalence of CHD. Model covariates and calculated coefficients for each are shown in Tables S6 through S10.

Table S6: Covariates and model performance for DisMod-MR 2.1 of total congenital heart anomalies

Covariate name	Type	Measure	Beta value	Exponentiated value
Healthcare Access and Quality index	Country	Prevalence	0.015 (0.013 - 0.017)	1.015 (1.013 - 1.017)
LDI (I$ per capita)	Country	Excess mort rate	-0.999 (-1.250 - -0.750)	0.368 (0.287 - 0.472)
Maternal alcohol consumption during pregnancy (proportion)	Country	Prevalence	0.299 (0.007 - 0.493)	1.349 (1.007 - 1.637)
Chromosomal diagnoses excluded	Study	Prevalence	-0.075 (-0.108 - -0.042)	0.927 (0.897 - 0.959)
EUROCAT to NBDPN registry case composition adjustment	Study	Prevalence	-0.003 (-0.008 - -0.000)	0.997 (0.992 - 1.000)
Hospital data for ages over 1 year only	Study	Prevalence	-1.618 (-1.828 - -0.626)	0.198 (0.161 - 0.535)
Hospital data for the under-1 year age group	Study	Prevalence	0.013 (-0.124 - 0.200)	1.013 (0.883 - 1.221)
ICDBSR to NBDPN registry case composition adjustment	Study	Prevalence	-1.400 (-1.554 - -1.318)	0.247 (0.211 - 0.268)
MarketScan	Study	Prevalence	-0.700 (-0.824 - -0.042)	0.496 (0.439 - 0.959)
Stillbirths included as cases	Study	Prevalence	0.084 (0.037 - 0.099)	1.088 (1.038 - 1.105)
Terminations of pregnancy included as cases	Study	Prevalence	0.097 (0.089 - 0.100)	1.102 (1.093 - 1.105)

Table S7: Covariates and model performance for DisMod-MR 2.1 of single ventricle and single ventricle pathway defects

Covariate name	Type	Measure	Beta value	Exponentiated value
LDI (I$ per capita)	Country	Excess mortality rate	-0.747 (-0.998 - -0.500)	0.474 (0.369 - 0.607)
Maternal alcohol consumption during pregnancy (proportion)	Country	Prevalence	0.139 (0.005 - 0.436)	1.149 (1.005 - 1.547)
Chromosomal diagnoses excluded	Study	Prevalence	-0.046 (-0.089 - -0.008)	0.955 (0.915 - 0.992)
EUROCAT to NBDPN registry case composition adjustment	Study	Prevalence	-0.035 (-0.100 - -0.001)	0.966 (0.904 - 0.999)
Hospital data for ages over 1 year only	Study	Prevalence	-0.014 (-0.049 - -0.000)	0.986 (0.953 - 1.000)
Hospital data for the under-1 year age group	Study	Prevalence	-0.014 (-0.045 - -0.000)	0.986 (0.956 - 1.000)
MarketScan	Study	Prevalence	-0.001 (-0.003 - -0.000)	0.999 (0.997 - 1.000)
Stillbirths included as cases	Study	Prevalence	0.002 (0.000 - 0.009)	1.002 (1.000 - 1.009)
Terminations of pregnancy included as cases	Study	Prevalence	0.012 (0.000 - 0.046)	1.012 (1.000 - 1.047)
Underreported	Study	Prevalence	-0.317 (-0.412 - -0.222)	0.728 (0.662 - 0.801)

Table S8: Covariates and model performance for DisMod-MR 2.1 of complex congenital heart defects excluding single ventricle and single ventricle pathway defects

Covariate name	Type	Measure	Beta value	Exponentiated value
LDI (I$ per capita)	Country	Excess mort rate	-0.621 (-1.000 - -0.250)	0.537 (0.368 - 0.779)
Maternal alcohol consumption during pregnancy (proportion)	Country	Prevalence	0.365 (0.094 - 0.497)	1.441 (1.099 - 1.644)
Chromosomal diagnoses excluded	Study	Prevalence	-0.204 (-0.231 - -0.180)	0.815 (0.794 - 0.835)
EUROCAT to NBDPN registry case composition adjustment	Study	Prevalence	-0.309 (-0.544 - -0.145)	0.734 (0.580 - 0.865)
Hospital data for ages over 1 year only	Study	Prevalence	-0.068 (-0.235 - -0.002)	0.935 (0.790 - 0.998)
Hospital data for the under-1 year age group	Study	Prevalence	-0.137 (-0.287 - -0.020)	0.872 (0.751 - 0.980)
ICDBSR to NBDPN registry case composition adjustment	Study	Prevalence	-0.781 (-0.827 - -0.729)	0.458 (0.437 - 0.483)
Stillbirths included as cases	Study	Prevalence	0.004 (0.001 - 0.013)	1.004 (1.001 - 1.013)
Terminations of pregnancy included as cases	Study	Prevalence	0.072 (0.009 - 0.135)	1.074 (1.009 - 1.145)
Underreported	Study	Prevalence	-0.174 (-0.344 - -0.044)	0.840 (0.709 - 0.957)
World Atlas to NBDPN registry case composition adjustment	Study	Prevalence	-0.826 (-0.881 - -0.766)	0.438 (0.414 - 0.465)

Table S9: Covariates and model performance for DisMod-MR 2.1 of malformations of great vessels, congenital valvular heart disease, and patent ductus arteriosus

Covariate name	Type	Measure	Beta value	Exponentiated value
LDI (I$ per capita)	Country	Excess mort rate	-0.275 (-0.500 - -0.056)	0.760 (0.607 - 0.946)
Maternal alcohol consumption during pregnancy	Country	Prevalence	0.130 (0.004 - 0.453)	1.139 (1.004 - 1.573)
Chromosomal diagnoses excluded	Study	Prevalence	-0.068 (-0.100 - -0.032)	0.934 (0.905 - 0.969)
EUROCAT to NBDPN registry case composition adjustment	Study	Prevalence	-0.169 (-0.386 - -0.021)	0.845 (0.680 - 0.979)
Hospital data for ages over 1 year only	Study	Prevalence	-0.274 (-0.670 - -0.002)	0.760 (0.512 - 0.998)
Hospital data for the under-1 year age group	Study	Prevalence	-0.090 (-0.387 - -0.003)	0.914 (0.679 - 0.997)
ICDBSR to NBDPN registry case composition adjustment	Study	Prevalence	-0.504 (-0.752 - -0.396)	0.604 (0.472 - 0.673)
MarketScan	Study	Prevalence	-0.059 (-0.225 - -0.001)	0.942 (0.799 - 0.999)
Stillbirths included as cases	Study	Prevalence	0.009 (0.000 - 0.033)	1.009 (1.000 - 1.034)
Terminations of pregnancy included as cases	Study	Prevalence	0.150 (0.000 - 0.296)	1.162 (1.000 - 1.345)
Underreported	Study	Prevalence	-0.315 (-0.423 - -0.187)	0.730 (0.655 - 0.830)

Table S10: Covariates and model performance for DisMod-MR 2.1 of ventricular septal defect and atrial septal defect

Covariate name	Type	Measure	Beta value	Exponentiated value
Healthcare access and quality index	Country	With-cond mort rate	-0.089 (-0.154 - -0.045)	0.915 (0.858 - 0.956)
LDI (I$ per capita)	Country	Excess mort rate	-0.025 (-0.050 - -0.000)	0.975 (0.952 - 1.000)
Maternal alcohol consumption during pregnancy (proportion)	Country	Prevalence	0.229 (0.039 - 0.442)	1.257 (1.040 - 1.556)
Hospital data for ages over 1 year only	Study	Prevalence	-1.558 (-1.684 - -1.440)	0.211 (0.186 - 0.237)
Hospital data for the under-1 year age group	Study	Prevalence	-0.118 (-0.265 - -0.010)	0.889 (0.767 - 0.991)
MarketScan	Study	Prevalence	-1.052 (-1.197 - -0.948)	0.349 (0.302 - 0.387)
Stillbirths included as cases	Study	Prevalence	0.041 (0.002 - 0.113)	1.042 (1.002 - 1.120)
Terminations of pregnancy included as cases	Study	Prevalence	0.128 (0.007 - 0.281)	1.137 (1.007 - 1.325)
Underreported	Study	Prevalence	-0.536 (-0.669 - -0.400)	0.585 (0.512 - 0.670)

Assigning disease sequelae and calculation of years of life lived with disability (YLDs)

After scaling to match the total congenital cardiovascular anomalies, each model was paired with a set of mutually exclusive and collectively exhaustive sequelae which quantify the main outcomes of each. YLDs were calculated by multiplying the sequela-specific prevalence by corresponding GBD disability weights (DW) derived from population surveys of over 60,000 respondents.[2,16] The proportions of each type of CHD with each type of sequela were derived using available information. To determine the distribution of health outcomes associated with the congenital causes, we extracted data from the same studies identified in the systematic review described above, this time focused on the long-term health outcomes of survivors in cohorts born with each type of congenital malformation. For conditions requiring surgical intervention shortly after birth to ensure survival, the health states included in the disability weight calculations correspond to the post-surgery outcomes

reported in cohorts of individuals born with these life-threatening congenital conditions. Where data were available on multiple cohorts, we pooled these cohorts together to calculate the proportion of individuals with each health state. Where data on the joint distribution of the long-term health outcomes were not available, we assumed independence of each long-term health outcome. Combined disability weights were calculated for all necessary combinations of existing disability weights.

Heart failure distribution was derived from the updated GBD 2017 heart failure analysis using the same approach of proportional allocation based on cause-specific mortality rate as previously described.[17] Several scientific literature sources reported on the prevalence and severity of intellectual disability in congenital heart defect populations;[18-20] the asymptomatic proportion of ASD/VSD was derived from literature sources on the long-term outcomes of patients diagnosed with septal defects at birth.[21-23] All except those asymptomatic ASD/VSDs were also assigned a health state of "congenital heart disease," the DW for which was derived from analysis of the Medical Expenditure Panel Survey in the United States as previously described.[4] Evidence for CHD-related disability universally was presented separately for each of intellectual disability and heart failure. We therefore assumed independence of the probability of each in calculating the proportion of CHD cases in each combination of health states (eg, mild heart failure + profound intellectual disability). Due to limited data availability, global pooled estimates of disability were applied to all locations and years, although the distribution was different for under 5 years and those older than 5 years. Combined disability weights were calculated in a multiplicative fashion as described above. Each unique combination of health states, including asymptomatic, represented a sequela of YLD calculation. YLDs were calculated as prevalence multiplied by DW for each sequela. YLDs for each sequela were then processed through a microsimulation framework to adjust for comorbidity across all GBD causes by location, age group, year, and sex.

Table S11: Severity splits: Proportion of each type of CHD with each disabling sequela by age group.

Sequela	**<5 years proportion**	**≥ 5 years proportion**
Congenital heart disease due to malformations of great vessels, congenital valvular heart disease and PDA	0.646 (0.629 - 0.659)	0.972 (0.968 - 0.974)
Congenital heart disease and borderline intellectual disability due to malformations of great vessels, congenital valvular heart disease and PDA	0.209 (0.197 - 0.222)	0.005 (0.004 - 0.007)
Congenital heart disease and mild intellectual disability due to malformations of great vessels, congenital valvular heart disease and PDA	0.056 (0.049 - 0.063)	0.014 (0.012 - 0.016)
Congenital heart disease and moderate intellectual disability due to malformations of great vessels, congenital valvular heart disease and PDA	0.045 (0.039 - 0.052)	0.004 (0.004 - 0.006)
Congenital heart disease and severe intellectual disability due to malformations of great vessels, congenital valvular heart disease and PDA	0.029 (0.025 - 0.035)	0.003 (0.002 - 0.004)
Congenital heart disease and profound intellectual disability due to malformations of great vessels, congenital valvular heart disease and PDA	0.015 (0.012 - 0.02)	0.002 (0.001 - 0.002)
Congenital heart disease due to single ventricle and single ventricle pathway heart defects	0.63 (0.611 - 0.645)	0.972 (0.968 - 0.974)
Congenital heart disease and borderline intellectual disability due to single ventricle and single ventricle pathway heart defects	0.207 (0.193 - 0.221)	0.005 (0.004 - 0.007)
Congenital heart disease and mild intellectual disability due to single ventricle and single ventricle pathway heart defects	0.062 (0.055 - 0.072)	0.014 (0.012 - 0.016)
Congenital heart disease and moderate intellectual disability due to single ventricle and single ventricle pathway heart defects	0.051 (0.044 - 0.06)	0.004 (0.004 - 0.006)
Congenital heart disease and severe intellectual disability due to single ventricle and single ventricle pathway heart defects	0.033 (0.028 - 0.04)	0.003 (0.002 - 0.004)
Congenital heart disease and profound intellectual disability due to single ventricle and single ventricle pathway heart defects	0.017 (0.014 - 0.023)	0.002 (0.001 - 0.002)
Congenital heart disease due to severe congenital heart anomalies excluding single ventricle heart defects	0.596 (0.541 - 0.629)	0.972 (0.968 - 0.974)
Congenital heart disease and borderline intellectual disability due to complex congenital heart anomalies excluding single ventricle heart defects	0.234 (0.196 - 0.273)	0.005 (0.004 - 0.007)
Congenital heart disease and mild intellectual disability due to complex congenital heart anomalies excluding single ventricle heart defects	0.065 (0.048 - 0.094)	0.014 (0.012 - 0.016)

Congenital heart disease and moderate intellectual disability due to complex congenital heart anomalies excluding single ventricle heart defects	0.053 (0.039 - 0.08)	0.004 (0.004 - 0.006)
Congenital heart disease and severe intellectual disability due to complex congenital heart anomalies excluding single ventricle heart defects	0.034 (0.024 - 0.058)	0.003 (0.002 - 0.004)
Congenital heart disease and profound intellectual disability due to complex congenital heart anomalies excluding single ventricle heart defects	0.018 (0.012 - 0.039)	0.002 (0.001 - 0.002)
Asymptomatic VSD/ASD	0.45 (0.433 - 0.466)	0.45 (0.433 - 0.466)
Congenital heart disease due to VSD/ASD	0.359 (0.342 - 0.374)	0.534 (0.516 - 0.55)
Congenital heart disease and borderline intellectual disability due to VSD/ASD	0.116 (0.106 - 0.127)	0.003 (0.002 - 0.006)
Congenital heart disease and mild intellectual disability due to VSD/ASD	0.029 (0.024 - 0.036)	0.008 (0.006 - 0.012)
Congenital heart disease and moderate intellectual disability due to VSD/ASD	0.024 (0.02 - 0.03)	0.002 (0.002 - 0.005)
Congenital heart disease and severe intellectual disability due to VSD/ASD	0.015 (0.012 - 0.02)	0.002 (0.001 - 0.004)
Congenital heart disease and profound intellectual disability due to VSD/ASD	0.008 (0.006 - 0.012)	0.001 (0.001 - 0.003)
Congenital heart disease due to other congenital cardiovascular anomalies	0.646 (0.629 - 0.659)	0.972 (0.968 - 0.974)
Congenital heart disease and borderline intellectual disability due to other congenital cardiovascular anomalies	0.209 (0.197 - 0.222)	0.005 (0.004 - 0.007)
Congenital heart disease and mild intellectual disability due to other congenital cardiovascular anomalies	0.056 (0.049 - 0.063)	0.014 (0.012 - 0.016)
Congenital heart disease and moderate intellectual disability due to other congenital cardiovascular anomalies	0.045 (0.039 - 0.052)	0.004 (0.004 - 0.006)
Congenital heart disease and severe intellectual disability due to other congenital cardiovascular anomalies	0.029 (0.025 - 0.035)	0.003 (0.002 - 0.004)
Congenital heart disease and profound intellectual disability due to other congenital cardiovascular anomalies	0.015 (0.012 - 0.02)	0.002 (0.001 - 0.002)

References for descriptions presented in supplement

1. Dicker D, Nguyen G, Abate D, GBD-Collaborators. Global, regional, and national age-sex-specific mortality and life expectancy, 1950-2017: a systematic analysis for the Global Burden of Disease Study 2017. *Lancet* 2018; **392**(10159): 1684-735.
2. Salomon JA, Haagsma JA, Davis A, et al. Disability weights for the Global Burden of Disease 2013 study. *Lancet Glob Health* 2015; **3**(11): e712-23.
3. Roth GA, Abate D, Abate KH, GBD-Collaborators. Global, regional, and national age-sex-specific mortality for 282 causes of death in 195 countries and territories, 1980-2017: a systematic analysis for the Global Burden of Disease Study 2017. *Lancet* 2018; **392**(10159): 1736-88.
4. James SL, Abate D, Abate KH, GBD-Collaborators. Global, regional, and national incidence, prevalence, and years lived with disability for 354 diseases and injuries for 195 countries and territories, 1990-2017: a systematic analysis for the Global Burden of Disease Study 2017. *Lancet* 2018; **392**(10159): 1789-858.
5. Stanaway JD, Afshin A, Gakidou E, GBD-Collaborators. Global, regional, and national comparative risk assessment of 84 behavioural, environmental and occupational, and metabolic risks or clusters of risks for 195 countries and territories, 1990-2017: a systematic analysis for the Global Burden of Disease Study 2017. *Lancet* 2018; **392**(10159): 1923-94.
6. American Institute for Cancer Research. Food, nutrition, physical activity, and the prevention of cancer: a global perspective. *Washington, DC: American Institute for Cancer Research* 2007.
7. Murray CJL, Callender CSKH, Kulikoff XR, GBD-Collaborators. Population and fertility by age and sex for 195 countries and territories, 1950-2017: a systematic analysis for the Global Burden of Disease Study 2017. *Lancet* 2018; **392**(10159): 1995-2051.
8. Mandalenakis Z, Rosengren A, Skoglund K, Lappas G, Eriksson P, Dellborg M. Survivorship in Children and Young Adults With Congenital Heart Disease in Sweden. *JAMA Intern Med* 2017; **177**(2): 224-30.

9. Moons P, Bovijn L, Budts W, Belmans A, Gewillig M. Temporal trends in survival to adulthood among patients born with congenital heart disease from 1970 to 1992 in Belgium. *Circulation* 2010; **122**(22): 2264-72.
10. Oster ME, Lee KA, Honein MA, Riehle-Colarusso T, Shin M, Correa A. Temporal trends in survival among infants with critical congenital heart defects. *Pediatrics* 2013; **131**(5): e1502-8.
11. Tennant PW, Pearce MS, Bythell M, Rankin J. 20-year survival of children born with congenital anomalies: a population-based study. *Lancet* 2010; **375**(9715): 649-56.
12. Flaxman AD, Vos T, Murray C. Integrated Meta-Regression Framework fo Descriptive Epidemiology: UW Press; 2012.
13. Fullman N, Yearwood J, Abay SM, GBD-Collaborators. Measuring performance on the Healthcare Access and Quality Index for 195 countries and territories and selected subnational locations: a systematic analysis from the Global Burden of Disease Study 2016. *Lancet* 2018; **391**(10136): 2236-71.
14. Miyake T, Shinohara T, Nakamura Y, et al. Spontaneous closure of ventricular septal defects followed up from <3 months of age. *Pediatr Int* 2004; **46**(2): 135-40.
15. Zhang J, Ko JM, Guileyardo JM, Roberts WC. A review of spontaneous closure of ventricular septal defect. *Proc (Bayl Univ Med Cent)* 2015; **28**(4): 516-20.
16. Salomon JA, Vos T, Hogan DR, et al. Common values in assessing health outcomes from disease and injury: disability weights measurement study for the Global Burden of Disease Study 2010. *Lancet* 2012; **380**(9859): 2129-43.
17. Roth GA, Johnson C, Abajobir A, et al. Global, Regional, and National Burden of Cardiovascular Diseases for 10 Causes, 1990 to 2015. *J Am Coll Cardiol* 2017; **70**(1): 1-25.
18. Gaynor JW, Stopp C, Wypij D, et al. Neurodevelopmental outcomes after cardiac surgery in infancy. *Pediatrics* 2015; **135**(5): 816-25.
19. Menting ME, Cuypers JA, Opic P, et al. The unnatural history of the ventricular septal defect: outcome up to 40 years after surgical closure. *J Am Coll Cardiol* 2015; **65**(18): 1941-51.
20. Riehle-Colarusso T, Autry A, Razzaghi H, et al. Congenital Heart Defects and Receipt of Special Education Services. *Pediatrics* 2015; **136**(3): 496-504.
21. Gabriel HM, Heger M, Innerhofer P, et al. Long-term outcome of patients with ventricular septal defect considered not to require surgical closure during childhood. *J Am Coll Cardiol* 2002; **39**(6): 1066-71.
22. Neumayer U, Stone S, Somerville J. Small ventricular septal defects in adults. *Eur Heart J* 1998; **19**(10): 1573-82.
23. Wren C, O'Sullivan J. Survival with congenital heart disease and need for follow up in adult life. *Heart* 2001; **85**(4): 438-43.

Supplemental Figures and Tables

Figure S7: Leading causes of death in children under 1 year of age. Figure S7 shows arrow diagrams depicting rank order and mean percentage change of causes of death (by death rate per 100,000) in children under 1 year of age in 1990 and 2017 for each Socio-demographic Index quintile.

HIV/AIDS=human immunodeficiency virus/acquired immunodeficiency syndrome. SIDS=sudden infant death syndrome. SDI=Socio-demographic Index.

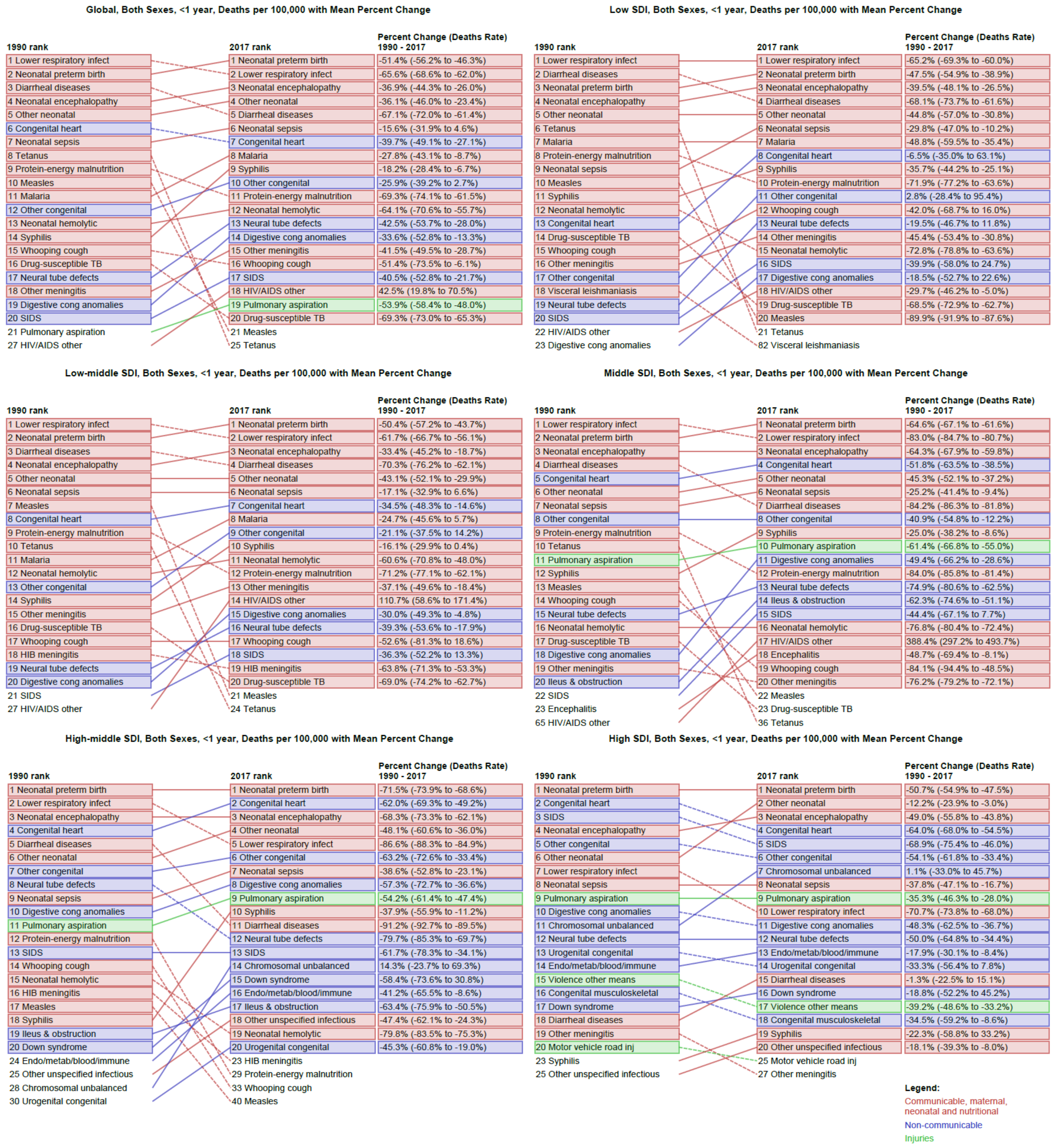

Figure S8: Year lived with disability due to congenital heart disease in children under 1 year of age showing years of life lived with disability per 100,000 by country in 2016 (top) and by Socio-demographic Index from 1990 to 2017 (bottom).

Note that subnational estimates were completed for 15 countries: Brazil, China, England, Ethiopia, Indonesia, India, Iran, Japan, Kenya, Mexico, New Zealand, Russia, South Africa, Ukraine, and the United States. SDI=Socio-demographic Index. YLDs=years lived with disability.

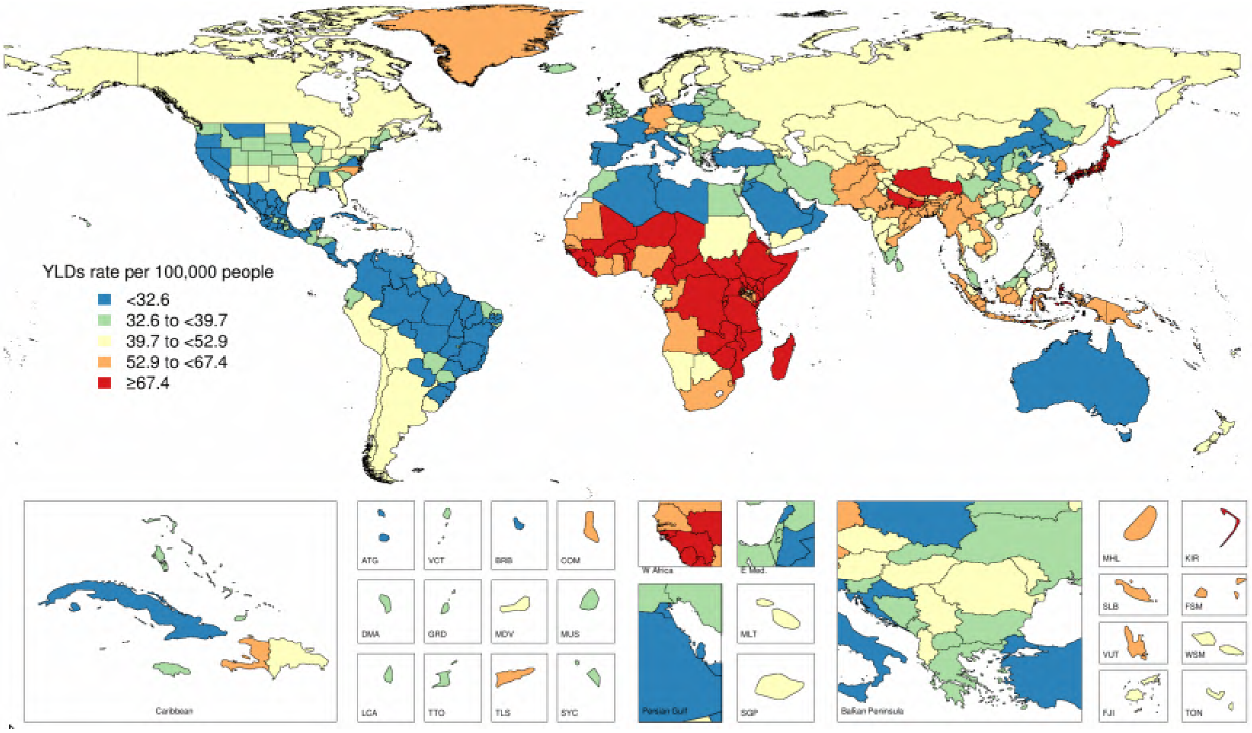

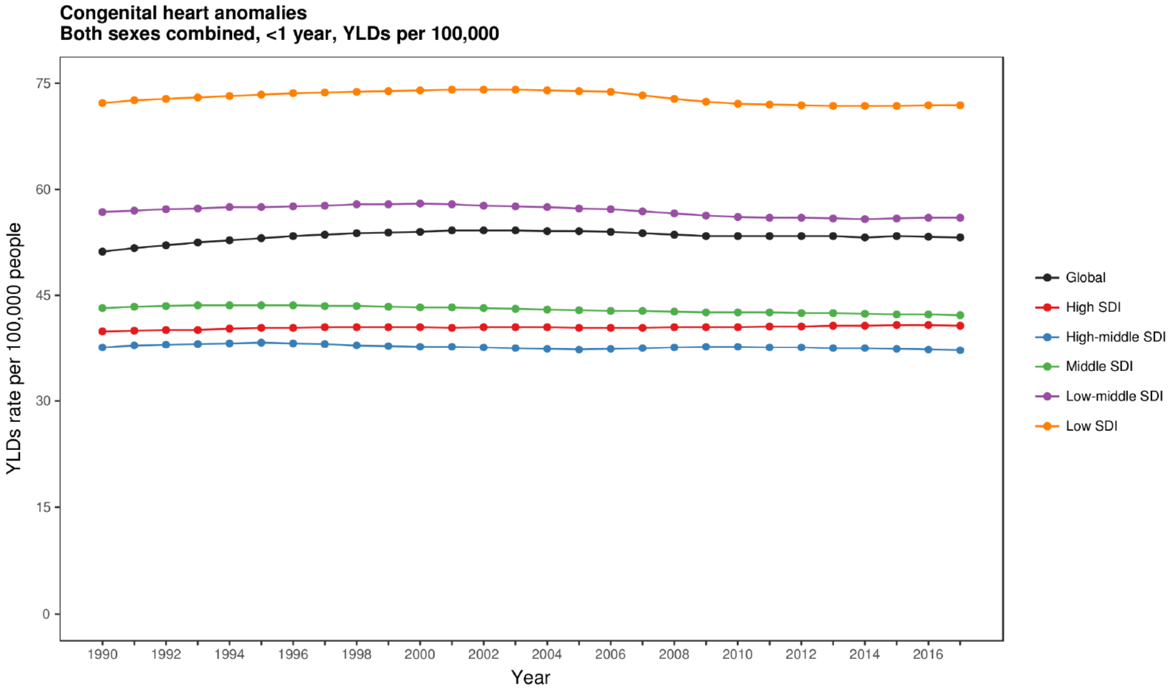

Figure S9: Prevalence (top) and age-standardised rate (bottom) of congenital heart disease at birth by GBD location in 2017

Top panel shows all-age prevalence rate (per 100,000) and bottom panel shows age-standardised prevalence rate by location in 2016. Note that subnational estimates were completed for 15 countries: Brazil, China, England, Ethiopia, Indonesia, India, Iran, Japan, Kenya, Mexico, New Zealand, Russia, South Africa, Ukraine, and the United States.

Appendix Figure S9A. Global prevalence of congenital heart disease for 2017, birth, both sexes combined.

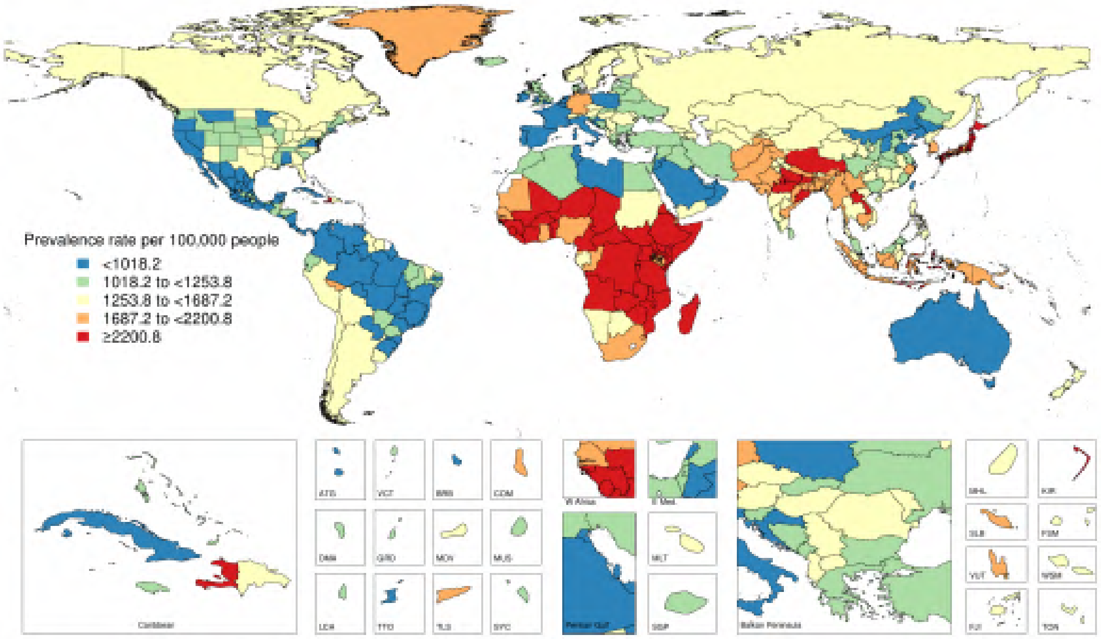

Appendix Figure S9B. Global prevalence of congenital heart disease for 2017, age-standardized, both sexes combined.

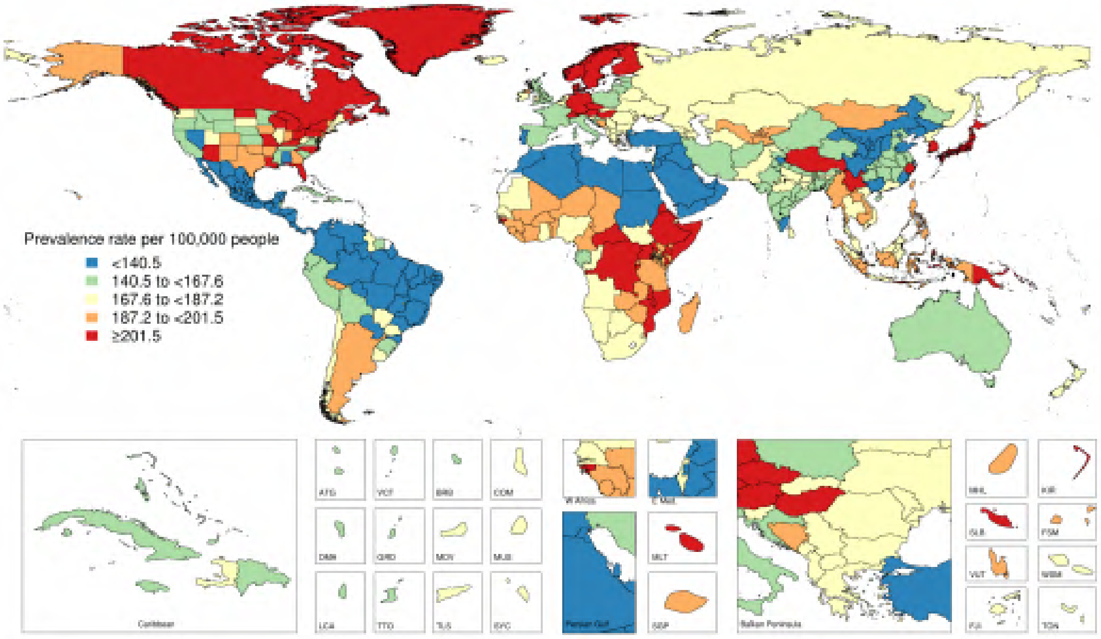

Figure S10: Years lived with disability and years of life lost due to congenital heart disease and rheumatic heart disease

This figure shows the relati ve global impact on years lived with disability and years of life lost from congenital and rheumatic heart disease by age. YLDs=years lived with disability. YLLs=years of life lost.

Appendix Figure S10. Global YLDs and YLLs by Age, Rheumatic heart disease and Congenital heart anomalies, 2017

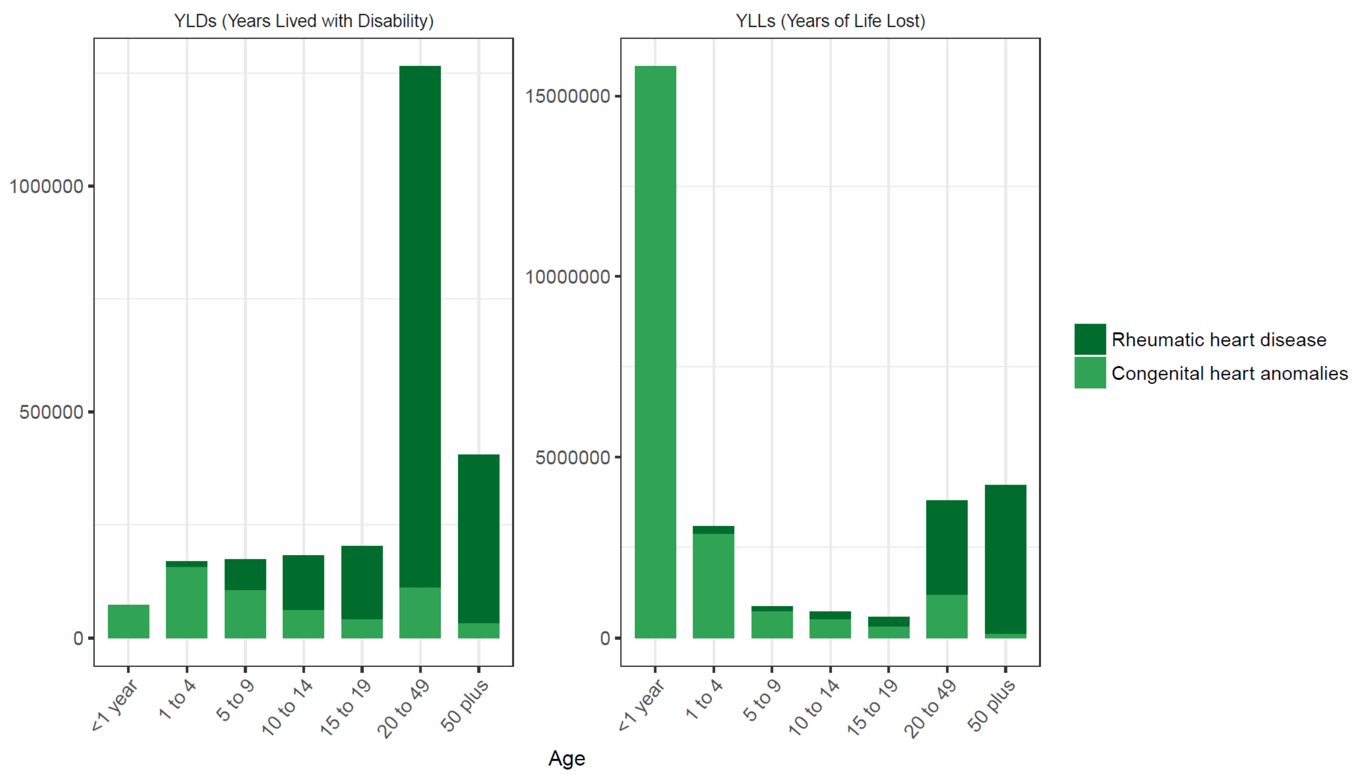

Table S12. CHD deaths, prevalence by age (<1, 1-4, 5-9, 10-14, 15-19, 20-49, 50+), GBD regions & CHD sub-categories for 1990 and 2017

			ALL CHD		ASD/VSD		Single ventricle defects		Complex CHD (not single-ventricle)		Malformations of great vessels		Other CHD	
**Location**	**Age**	**Metric**	**1990**	**2017**	**1990**	**2017**	**1990**	**2017**	**1990**	**2017**	**1990**	**2017**	**1990**	**2017**
**Global**	<1 year	Deaths Number	288,283 (194,852 to 368,488)	180,624 (146,825 to 214,178)										
**Global**	<1 year	Prevalence Number	1,583,134 (1,395,695 to 1,797,473)	1,700,786 (1,506,602 to 1,920,926)	636,969 (508,509 to 796,758)	653,080 (523,251 to 810,644)	63,916 (49,669 to 81,038)	67,116 (52,692 to 84,276)	318,356 (258,698 to 387,557)	363,721 (296,008 to 443,954)	311,014 (247,845 to 387,526)	345,042 (274,182 to 429,191)	252,880 (223,162 to 287,239)	271,828 (240,525 to 307,247)
**Global**	<1 year	Prevalence Rate	1,192.4 (1,051.2 to 1,353.9)	1,233.1 (1,092.3 to 1,392.7)	479.8 (383.0 to 600.1)	473.5 (379.4 to 587.7)	48.1 (37.4 to 61.0)	48.7 (38.2 to 61.1)	239.8 (194.9 to 291.9)	263.7 (214.6 to 321.9)	234.3 (186.7 to 291.9)	250.2 (198.8 to 311.2)	190.5 (168.1 to 216.4)	197.1 (174.4 to 222.8)
**Global**	1 to 4	Deaths Number	60,986 (32,267 to 85,719)	33,590 (23,176 to 44,492)										
**Global**	1 to 4	Prevalence Number	3,301,779 (2,919,088 to 3,730,912)	3,575,440 (3,189,910 to 4,005,223)	1,539,528 (1,238,215 to 1,885,288)	1,602,064 (1,288,943 to 1,939,366)	91,607 (73,164 to 115,636)	104,567 (83,140 to 130,587)	534,035 (453,875 to 628,759)	631,946 (537,705 to 740,649)	565,325 (466,213 to 681,255)	617,338 (508,770 to 743,384)	571,286 (503,543 to 646,969)	619,525 (550,543 to 695,350)
**Global**	1 to 4	Prevalence Rate	645.3 (570.5 to 729.1)	658.8 (587.8 to 738.0)	300.9 (242.0 to 368.4)	295.2 (237.5 to 357.3)	17.9 (14.3 to 22.6)	19.3 (15.3 to 24.1)	104.4 (88.7 to 122.9)	116.4 (99.1 to 136.5)	110.5 (91.1 to 133.1)	113.7 (93.7 to 137.0)	111.6 (98.4 to 126.4)	114.2 (101.4 to 128.1)
**Global**	5 to 9	Deaths Number	14,980 (10,981 to 19,113)	9,321 (8,186 to 10,702)										
**Global**	5 to 9	Prevalence Number	1,884,480 (1,689,484 to 2,101,525)	2,147,652 (1,938,243 to 2,389,038)	814,126 (665,566 to 972,651)	897,031 (739,274 to 1,067,759)	54,397 (43,965 to 67,153)	66,555 (54,249 to 81,474)	343,294 (294,999 to 394,122)	420,002 (361,942 to 483,130)	314,107 (264,676 to 369,521)	355,049 (297,586 to 418,228)	358,556 (319,895 to 400,549)	409,015 (366,369 to 455,752)
**Global**	5 to 9	Prevalence Rate	322.3 (288.9 to 359.4)	324.8 (293.1 to 361.3)	139.2 (113.8 to 166.3)	135.7 (111.8 to 161.5)	9.3 (7.5 to 11.5)	10.1 (8.2 to 12.3)	58.7 (50.4 to 67.4)	63.5 (54.7 to 73.1)	53.7 (45.3 to 63.2)	53.7 (45.0 to 63.3)	61.3 (54.7 to 68.5)	61.9 (55.4 to 68.9)
**Global**	10 to 14	Deaths Number	8,253 (6,876 to 9,982)	7,123 (6,491 to 7,792)										
**Global**	10 to 14	Prevalence Number	1,013,588 (922,776 to 1,116,185)	1,206,347 (1,098,941 to 1,325,679)	369,077 (308,891 to 436,717)	419,566 (351,562 to 496,291)	27,758 (22,790 to 33,634)	36,514 (30,018 to 44,060)	230,288 (198,727 to 262,811)	295,305 (255,760 to 336,837)	174,018 (147,916 to 204,012)	202,032 (171,392 to 236,307)	212,447 (193,114 to 234,173)	252,931 (230,321 to 278,591)
**Global**	10 to 14	Prevalence Rate	188.2 (171.4 to 207.3)	189.6 (172.7 to 208.4)	68.5 (57.4 to 81.1)	65.9 (55.3 to 78.0)	5.2 (4.2 to 6.2)	5.7 (4.7 to 6.9)	42.8 (36.9 to 48.8)	46.4 (40.2 to 52.9)	32.3 (27.5 to 37.9)	31.8 (26.9 to 37.1)	39.4 (35.9 to 43.5)	39.8 (36.2 to 43.8)
**Global**	15 to 19	Deaths Number	5,444 (4,881 to 6,378)	4,581 (4,261 to 4,922)										
**Global**	15 to	Prevalence Number	657,810 (602,278 to	783,644 (718,648 to	198,636 (166,339 to	222,629 (186,478 to	16,133 (13,285 to	21,887 (18,104 to	173,529 (150,362 to	225,694 (196,155 to	115,491 (99,425 to	129,802 (110,951 to	154,021 (140,732 to	183,631 (168,101 to

	19		718,685)	854,251)	235,232)	262,374)	19,271)	26,189)	197,260)	256,512)	134,565)	151,643)	168,893)	200,753)
**Global**	15 to 19	Prevalence Rate	124.9 (114.4 to 136.5)	127.1 (116.6 to 138.6)	37.7 (31.6 to 44.7)	36.1 (30.2 to 42.6)	3.1 (2.5 to 3.7)	3.6 (2.9 to 4.2)	32.9 (28.5 to 37.5)	36.6 (31.8 to 41.6)	21.9 (18.9 to 25.6)	21.1 (18.0 to 24.6)	29.2 (26.7 to 32.1)	29.8 (27.3 to 32.6)
**Global**	20 to 49	Deaths Number	17,844 (16,233 to 20,488)	21,743 (20,537 to 23,305)										
**Global**	20 to 49	Prevalence Number	1,369,509 (1,259,738 to 1,485,730)	1,986,168 (1,827,974 to 2,152,188)	246,390 (205,360 to 293,799)	324,494 (268,729 to 387,514)	26,956 (22,565 to 32,012)	41,484 (34,804 to 49,434)	474,379 (418,604 to 533,392)	723,856 (641,449 to 813,293)	234,213 (202,548 to 269,703)	329,340 (285,264 to 378,868)	387,572 (356,170 to 421,810)	566,994 (521,883 to 615,790)
**Global**	20 to 49	Prevalence Rate	62.0 (57.0 to 67.2)	60.3 (55.5 to 65.3)	11.1 (9.3 to 13.3)	9.8 (8.2 to 11.8)	1.2 (1.0 to 1.4)	1.3 (1.1 to 1.5)	21.5 (18.9 to 24.1)	22.0 (19.5 to 24.7)	10.6 (9.2 to 12.2)	10.0 (8.7 to 11.5)	17.5 (16.1 to 19.1)	17.2 (15.8 to 18.7)
**Global**	50 plus	Deaths Number	2,790 (2,576 to 3,434)	4,266 (3,906 to 4,726)										
**Global**	50 plus	Prevalence Number	283,060 (259,116 to 310,425)	570,825 (521,823 to 626,336)	10,042 (8,074 to 12,188)	18,815 (15,191 to 22,711)	2,986 (2,476 to 3,577)	5,623 (4,678 to 6,680)	115,343 (102,207 to 128,804)	228,213 (202,678 to 255,455)	43,948 (37,959 to 51,027)	92,152 (79,680 to 106,711)	110,741 (101,103 to 121,733)	226,023 (206,270 to 248,588)
**Global**	50 plus	Prevalence Rate	33.1 (30.3 to 36.3)	34.2 (31.2 to 37.5)	1.2 (0.9 to 1.4)	1.1 (0.9 to 1.4)	0.3 (0.3 to 0.4)	0.3 (0.3 to 0.4)	13.5 (12.0 to 15.1)	13.7 (12.1 to 15.3)	5.1 (4.4 to 6.0)	5.5 (4.8 to 6.4)	13.0 (11.8 to 14.2)	13.5 (12.3 to 14.9)
**High SDI**	<1 year	Deaths Number	13,117 (10,096 to 14,292)	4,248 (3,804 to 4,835)										
**High SDI**	<1 year	Prevalence Number	128,424 (113,370 to 144,856)	118,287 (105,569 to 131,691)	69,713 (57,873 to 83,240)	65,692 (55,598 to 77,049)	3,468 (2,945 to 4,077)	3,099 (2,641 to 3,637)	18,691 (15,961 to 22,238)	16,257 (14,119 to 19,111)	16,085 (13,101 to 19,880)	14,375 (11,789 to 17,762)	20,467 (18,052 to 23,117)	18,865 (16,828 to 20,989)
**High SDI**	<1 year	Prevalence Rate	995.4 (878.7 to 1,122.7)	1,020.3 (910.6 to 1,135.9)	540.3 (448.5 to 645.1)	566.6 (479.6 to 664.6)	26.9 (22.8 to 31.6)	26.7 (22.8 to 31.4)	144.9 (123.7 to 172.4)	140.2 (121.8 to 164.8)	124.7 (101.5 to 154.1)	124.0 (101.7 to 153.2)	158.6 (139.9 to 179.2)	162.7 (145.1 to 181.0)
**High SDI**	1 to 4	Deaths Number	2,204 (1,678 to 2,423)	597 (530 to 736)										
**High SDI**	1 to 4	Prevalence Number	332,813 (295,052 to 372,955)	312,347 (280,030 to 346,263)	187,874 (157,515 to 219,923)	179,142 (152,884 to 206,252)	9,451 (8,065 to 11,107)	8,689 (7,486 to 10,160)	39,917 (35,262 to 45,173)	35,580 (31,728 to 39,894)	37,973 (31,626 to 45,806)	34,610 (29,119 to 41,689)	57,598 (50,764 to 64,818)	54,326 (48,488 to 60,315)
**High SDI**	1 to 4	Prevalence Rate	647.8 (574.3 to 725.9)	664.4 (595.7 to 736.6)	365.7 (306.6 to 428.1)	381.1 (325.2 to 438.7)	18.4 (15.7 to 21.6)	18.5 (15.9 to 21.6)	77.7 (68.6 to 87.9)	75.7 (67.5 to 84.9)	73.9 (61.6 to 89.2)	73.6 (61.9 to 88.7)	112.1 (98.8 to 126.2)	115.6 (103.1 to 128.3)
**High SDI**	5 to 9	Deaths Number	740 (545 to 819)	216 (189 to 262)										
**High SDI**	5 to 9	Prevalence Number	248,627 (223,234 to 276,471)	233,189 (211,144 to 256,779)	129,376 (109,071 to 149,914)	122,537 (105,610 to 139,540)	8,643 (7,393 to 10,078)	7,972 (6,858 to 9,230)	35,065 (30,982 to 39,438)	32,090 (28,463 to 35,917)	27,883 (23,652 to 33,018)	25,723 (21,917 to 30,411)	47,659 (42,712 to 53,096)	44,868 (40,587 to 49,300)
**High SDI**	5 to 9	Prevalence Rate	375.8 (337.4 to 417.8)	380.6 (344.7 to 419.2)	195.5 (164.8 to 226.6)	200.0 (172.4 to 227.8)	13.1 (11.2 to 15.2)	13.0 (11.2 to 15.1)	53.0 (46.8 to 59.6)	52.4 (46.5 to 58.6)	42.1 (35.7 to 49.9)	42.0 (35.8 to 49.6)	72.0 (64.6 to 80.2)	73.2 (66.3 to 80.5)

**High SDI**	10 to 14	Deaths Number	603 (469 to 658)	221 (193 to 267)										
**High SDI**	10 to 14	Prevalence Number	162,226 (147,817 to 178,211)	150,108 (137,356 to 163,994)	72,552 (62,117 to 83,619)	67,579 (58,420 to 77,164)	6,183 (5,311 to 7,185)	5,643 (4,876 to 6,531)	31,023 (27,331 to 34,918)	28,482 (25,179 to 31,808)	17,984 (15,361 to 20,893)	16,377 (13,985 to 19,002)	34,483 (31,366 to 37,899)	32,027 (29,327 to 35,097)
**High SDI**	10 to 14	Prevalence Rate	242.8 (221.2 to 266.7)	244.4 (223.7 to 267.1)	108.6 (93.0 to 125.1)	110.1 (95.1 to 125.7)	9.3 (7.9 to 10.8)	9.2 (7.9 to 10.6)	46.4 (40.9 to 52.3)	46.4 (41.0 to 51.8)	26.9 (23.0 to 31.3)	26.7 (22.8 to 30.9)	51.6 (46.9 to 56.7)	52.2 (47.8 to 57.2)
**High SDI**	15 to 19	Deaths Number	646 (526 to 747)	252 (221 to 324)										
**High SDI**	15 to 19	Prevalence Number	124,337 (114,304 to 134,947)	110,857 (101,952 to 120,170)	46,197 (39,679 to 53,454)	41,546 (35,926 to 47,957)	4,838 (4,124 to 5,613)	4,213 (3,616 to 4,873)	30,201 (26,673 to 33,878)	26,765 (23,732 to 29,803)	13,525 (11,611 to 15,740)	11,890 (10,227 to 13,781)	29,574 (27,103 to 32,161)	26,443 (24,267 to 28,718)
**High SDI**	15 to 19	Prevalence Rate	172.4 (158.5 to 187.1)	174.5 (160.5 to 189.1)	64.1 (55.0 to 74.1)	65.4 (56.5 to 75.5)	6.7 (5.7 to 7.8)	6.6 (5.7 to 7.7)	41.9 (37.0 to 47.0)	42.1 (37.4 to 46.9)	18.8 (16.1 to 21.8)	18.7 (16.1 to 21.7)	41.0 (37.6 to 44.6)	41.6 (38.2 to 45.2)
**High SDI**	20 to 49	Deaths Number	2,964 (2,649 to 3,677)	1,897 (1,722 to 2,387)										
**High SDI**	20 to 49	Prevalence Number	376,349 (347,519 to 404,992)	389,614 (360,309 to 419,079)	77,084 (66,168 to 89,437)	77,514 (66,741 to 89,312)	12,688 (10,822 to 14,687)	12,222 (10,489 to 14,152)	138,498 (122,998 to 155,039)	145,242 (129,469 to 161,660)	40,452 (35,136 to 46,934)	42,163 (36,538 to 48,998)	107,627 (99,239 to 116,182)	112,474 (103,916 to 121,186)
**High SDI**	20 to 49	Prevalence Rate	87.4 (80.7 to 94.0)	84.6 (78.2 to 91.0)	17.9 (15.4 to 20.8)	16.8 (14.5 to 19.4)	2.9 (2.5 to 3.4)	2.7 (2.3 to 3.1)	32.2 (28.6 to 36.0)	31.5 (28.1 to 35.1)	9.4 (8.2 to 10.9)	9.2 (7.9 to 10.6)	25.0 (23.0 to 27.0)	24.4 (22.6 to 26.3)
**High SDI**	50 plus	Deaths Number	1,333 (1,210 to 1,822)	1,069 (890 to 1,387)										
**High SDI**	50 plus	Prevalence Number	126,474 (115,413 to 138,271)	210,061 (191,767 to 229,116)	5,053 (4,227 to 5,968)	8,042 (6,762 to 9,472)	2,151 (1,791 to 2,585)	3,132 (2,663 to 3,697)	56,754 (50,562 to 63,124)	93,581 (83,618 to 104,026)	12,728 (10,936 to 14,756)	20,671 (17,854 to 24,064)	49,786 (45,354 to 54,627)	84,635 (77,080 to 92,535)
**High SDI**	50 plus	Prevalence Rate	50.6 (46.1 to 55.3)	51.7 (47.2 to 56.4)	2.0 (1.7 to 2.4)	2.0 (1.7 to 2.3)	0.9 (0.7 to 1.0)	0.8 (0.7 to 0.9)	22.7 (20.2 to 25.2)	23.0 (20.6 to 25.6)	5.1 (4.4 to 5.9)	5.1 (4.4 to 5.9)	19.9 (18.1 to 21.8)	20.8 (19.0 to 22.8)
**High-middle SDI**	<1 year	Deaths Number	56,869 (44,689 to 66,735)	17,767 (15,871 to 19,405)										
**High-middle SDI**	<1 year	Prevalence Number	205,462 (176,680 to 238,643)	166,756 (145,395 to 191,476)	109,842 (87,660 to 138,538)	88,875 (71,922 to 109,065)	4,590 (3,649 to 5,694)	3,685 (2,956 to 4,501)	22,461 (18,456 to 27,267)	17,651 (14,583 to 21,311)	35,817 (28,860 to 44,787)	29,955 (24,043 to 37,453)	32,752 (28,159 to 38,088)	26,591 (23,188 to 30,549)
**High-middle SDI**	<1 year	Prevalence Rate	949.3 (816.3 to 1,102.6)	937.4 (817.3 to 1,076.3)	507.5 (405.0 to 640.1)	499.6 (404.3 to 613.1)	21.2 (16.9 to 26.3)	20.7 (16.6 to 25.3)	103.8 (85.3 to 126.0)	99.2 (82.0 to 119.8)	165.5 (133.3 to 206.9)	168.4 (135.2 to 210.5)	151.3 (130.1 to 176.0)	149.5 (130.3 to 171.7)
**High-middle SDI**	1 to 4	Deaths Number	9,647 (7,448 to 11,509)	2,736 (2,425 to 3,170)										
**High-middle SDI**	1 to 4	Prevalence Number	536,784 (463,466 to 619,728)	401,911 (350,133 to 456,575)	299,019 (241,301 to 367,759)	222,931 (183,559 to 270,034)	9,731 (7,875 to 12,142)	7,793 (6,352 to 9,533)	50,010 (42,739 to 57,975)	38,335 (33,019 to 44,214)	85,785 (71,263 to 103,255)	63,790 (52,810 to 76,852)	92,240 (79,262 to 106,819)	69,061 (60,154 to 78,633)

**High-middle SDI**	1 to 4	Prevalence Rate	584.3 (504.5 to 674.5)	586.4 (510.9 to 666.2)	325.5 (262.6 to 400.3)	325.3 (267.8 to 394.0)	10.6 (8.6 to 13.2)	11.4 (9.3 to 13.9)	54.4 (46.5 to 63.1)	55.9 (48.2 to 64.5)	93.4 (77.6 to 112.4)	93.1 (77.1 to 112.1)	100.4 (86.3 to 116.3)	100.8 (87.8 to 114.7)
**High-middle SDI**	5 to 9	Deaths Number	3,230 (2,707 to 3,702)	1,171 (1,069 to 1,295)										
**High-middle SDI**	5 to 9	Prevalence Number	320,973 (283,638 to 362,786)	258,973 (230,162 to 291,593)	163,191 (132,541 to 195,568)	132,111 (109,045 to 156,902)	7,126 (5,840 to 8,774)	6,150 (5,120 to 7,402)	38,236 (32,924 to 44,015)	30,588 (26,580 to 35,071)	51,676 (43,753 to 61,168)	41,124 (34,852 to 48,426)	60,744 (53,481 to 68,667)	49,001 (43,454 to 55,330)
**High-middle SDI**	5 to 9	Prevalence Rate	306.0 (270.4 to 345.8)	313.1 (278.3 to 352.5)	155.6 (126.3 to 186.4)	159.7 (131.8 to 189.7)	6.8 (5.6 to 8.4)	7.4 (6.2 to 8.9)	36.4 (31.4 to 42.0)	37.0 (32.1 to 42.4)	49.3 (41.7 to 58.3)	49.7 (42.1 to 58.5)	57.9 (51.0 to 65.5)	59.2 (52.5 to 66.9)
**High-middle SDI**	10 to 14	Deaths Number	1,778 (1,580 to 1,982)	867 (811 to 944)										
**High-middle SDI**	10 to 14	Prevalence Number	177,504 (159,243 to 197,605)	147,388 (132,597 to 162,785)	76,715 (63,916 to 91,232)	63,858 (53,567 to 75,362)	4,279 (3,490 to 5,198)	3,864 (3,200 to 4,605)	28,863 (24,832 to 33,035)	23,814 (20,601 to 27,103)	30,656 (26,183 to 35,932)	25,167 (21,384 to 29,467)	36,992 (33,243 to 41,233)	30,685 (27,626 to 33,970)
**High-middle SDI**	10 to 14	Prevalence Rate	180.0 (161.5 to 200.4)	185.7 (167.1 to 205.1)	77.8 (64.8 to 92.5)	80.5 (67.5 to 95.0)	4.3 (3.5 to 5.3)	4.9 (4.0 to 5.8)	29.3 (25.2 to 33.5)	30.0 (26.0 to 34.1)	31.1 (26.6 to 36.4)	31.7 (26.9 to 37.1)	37.5 (33.7 to 41.8)	38.7 (34.8 to 42.8)
**High-middle SDI**	15 to 19	Deaths Number	1,206 (1,109 to 1,326)	567 (523 to 631)										
**High-middle SDI**	15 to 19	Prevalence Number	120,205 (108,867 to 132,177)	101,287 (92,117 to 111,290)	43,058 (35,665 to 51,491)	36,523 (30,545 to 43,219)	2,740 (2,247 to 3,330)	2,669 (2,234 to 3,183)	23,890 (20,631 to 27,287)	20,691 (17,924 to 23,446)	22,615 (19,512 to 26,244)	17,866 (15,393 to 20,946)	27,902 (25,238 to 30,790)	23,539 (21,358 to 25,901)
**High-middle SDI**	15 to 19	Prevalence Rate	118.2 (107.0 to 129.9)	124.8 (113.5 to 137.1)	42.3 (35.1 to 50.6)	45.0 (37.6 to 53.3)	2.7 (2.2 to 3.3)	3.3 (2.8 to 3.9)	23.5 (20.3 to 26.8)	25.5 (22.1 to 28.9)	22.2 (19.2 to 25.8)	22.0 (19.0 to 25.8)	27.4 (24.8 to 30.3)	29.0 (26.3 to 31.9)
**High-middle SDI**	20 to 49	Deaths Number	3,753 (3,502 to 4,157)	3,015 (2,786 to 3,401)										
**High-middle SDI**	20 to 49	Prevalence Number	268,622 (246,167 to 292,128)	364,444 (335,562 to 395,053)	56,695 (46,937 to 67,982)	72,346 (60,070 to 86,635)	4,995 (4,119 to 6,088)	7,861 (6,579 to 9,494)	77,548 (67,437 to 88,307)	110,659 (96,551 to 125,852)	53,593 (46,617 to 61,817)	69,499 (60,360 to 80,278)	75,791 (69,641 to 82,439)	104,079 (95,670 to 113,063)
**High-middle SDI**	20 to 49	Prevalence Rate	55.6 (51.0 to 60.5)	55.5 (51.1 to 60.1)	11.7 (9.7 to 14.1)	11.0 (9.1 to 13.2)	1.0 (0.9 to 1.3)	1.2 (1.0 to 1.4)	16.1 (14.0 to 18.3)	16.8 (14.7 to 19.1)	11.1 (9.7 to 12.8)	10.6 (9.2 to 12.2)	15.7 (14.4 to 17.1)	15.8 (14.6 to 17.2)
**High-middle SDI**	50 plus	Deaths Number	503 (461 to 609)	825 (733 to 915)										
**High-middle SDI**	50 plus	Prevalence Number	56,235 (51,283 to 62,014)	113,912 (103,940 to 125,581)	2,204 (1,723 to 2,724)	4,233 (3,337 to 5,200)	489 (399 to 593)	1,109 (907 to 1,357)	21,069 (18,505 to 23,783)	40,159 (35,241 to 45,383)	10,525 (9,097 to 12,206)	23,643 (20,459 to 27,351)	21,948 (19,970 to 24,215)	44,768 (40,793 to 49,370)
**High-middle SDI**	50 plus	Prevalence Rate	27.8 (25.4 to 30.7)	29.8 (27.2 to 32.8)	1.1 (0.9 to 1.3)	1.1 (0.9 to 1.4)	0.2 (0.2 to 0.3)	0.3 (0.2 to 0.4)	10.4 (9.1 to 11.8)	10.5 (9.2 to 11.9)	5.2 (4.5 to 6.0)	6.2 (5.3 to 7.1)	10.9 (9.9 to 12.0)	11.7 (10.7 to 12.9)
**Middle SDI**	<1 year	Deaths Number	94,594 (72,132 to 114,065)	39,221 (33,572 to 43,261)										

**Middle SDI**	<1 year	Prevalence Number	390,055 (339,290 to 447,571)	327,266 (287,486 to 371,647)	173,793 (137,027 to 220,655)	142,630 (114,175 to 178,284)	11,863 (9,186 to 15,012)	10,394 (8,220 to 12,993)	49,549 (40,410 to 60,164)	42,673 (34,869 to 52,007)	92,632 (74,495 to 115,939)	79,346 (63,354 to 98,864)	62,218 (54,101 to 71,424)	52,223 (45,895 to 59,360)
**Middle SDI**	<1 year	Prevalence Rate	1,027.9 (894.1 to 1,179.4)	1,001.6 (879.9 to 1,137.5)	458.0 (361.1 to 581.5)	436.5 (349.4 to 545.7)	31.3 (24.2 to 39.6)	31.8 (25.2 to 39.8)	130.6 (106.5 to 158.5)	130.6 (106.7 to 159.2)	244.1 (196.3 to 305.5)	242.8 (193.9 to 302.6)	164.0 (142.6 to 188.2)	159.8 (140.5 to 181.7)
**Middle SDI**	1 to 4	Deaths Number	16,950 (12,168 to 21,628)	5,422 (4,599 to 6,062)										
**Middle SDI**	1 to 4	Prevalence Number	891,411 (777,755 to 1,020,148)	749,864 (659,413 to 846,204)	433,946 (346,449 to 536,166)	356,911 (286,056 to 434,305)	19,006 (15,079 to 24,184)	18,447 (14,836 to 22,973)	99,244 (84,591 to 116,251)	88,458 (75,965 to 102,803)	185,452 (152,936 to 223,674)	156,921 (129,683 to 189,637)	153,762 (134,232 to 176,335)	129,127 (113,420 to 146,273)
**Middle SDI**	1 to 4	Prevalence Rate	594.5 (518.7 to 680.4)	584.6 (514.1 to 659.7)	289.4 (231.1 to 357.6)	278.3 (223.0 to 338.6)	12.7 (10.1 to 16.1)	14.4 (11.6 to 17.9)	66.2 (56.4 to 77.5)	69.0 (59.2 to 80.2)	123.7 (102.0 to 149.2)	122.3 (101.1 to 147.9)	102.6 (89.5 to 117.6)	100.7 (88.4 to 114.0)
**Middle SDI**	5 to 9	Deaths Number	5,215 (4,074 to 6,345)	2,502 (2,237 to 2,726)										
**Middle SDI**	5 to 9	Prevalence Number	511,820 (457,278 to 573,155)	481,136 (430,466 to 537,131)	226,180 (183,352 to 272,883)	210,183 (171,128 to 250,900)	11,992 (9,655 to 14,898)	13,219 (10,827 to 16,155)	71,067 (60,897 to 81,789)	68,888 (59,284 to 78,861)	105,518 (89,208 to 124,296)	97,789 (82,510 to 115,542)	97,062 (85,981 to 108,859)	91,058 (80,983 to 101,897)
**Middle SDI**	5 to 9	Prevalence Rate	302.3 (270.1 to 338.5)	302.8 (270.9 to 338.1)	133.6 (108.3 to 161.2)	132.3 (107.7 to 157.9)	7.1 (5.7 to 8.8)	8.3 (6.8 to 10.2)	42.0 (36.0 to 48.3)	43.4 (37.3 to 49.6)	62.3 (52.7 to 73.4)	61.6 (51.9 to 72.7)	57.3 (50.8 to 64.3)	57.3 (51.0 to 64.1)
**Middle SDI**	10 to 14	Deaths Number	2,941 (2,551 to 3,434)	2,127 (1,934 to 2,275)										
**Middle SDI**	10 to 14	Prevalence Number	284,801 (257,876 to 315,241)	284,302 (257,699 to 313,588)	104,847 (86,967 to 125,269)	103,745 (86,439 to 123,313)	6,443 (5,214 to 7,960)	8,026 (6,552 to 9,720)	52,143 (44,761 to 59,742)	53,927 (46,527 to 61,386)	62,033 (52,889 to 72,468)	59,576 (50,494 to 69,872)	59,335 (53,735 to 65,933)	59,028 (53,490 to 65,316)
**Middle SDI**	10 to 14	Prevalence Rate	174.9 (158.3 to 193.6)	178.7 (162.0 to 197.1)	64.4 (53.4 to 76.9)	65.2 (54.3 to 77.5)	4.0 (3.2 to 4.9)	5.0 (4.1 to 6.1)	32.0 (27.5 to 36.7)	33.9 (29.2 to 38.6)	38.1 (32.5 to 44.5)	37.4 (31.7 to 43.9)	36.4 (33.0 to 40.5)	37.1 (33.6 to 41.1)
**Middle SDI**	15 to 19	Deaths Number	1,921 (1,744 to 2,239)	1,494 (1,350 to 1,602)										
**Middle SDI**	15 to 19	Prevalence Number	191,322 (174,393 to 209,894)	190,627 (174,351 to 208,581)	57,355 (47,628 to 68,655)	56,120 (46,532 to 66,658)	3,708 (3,007 to 4,536)	5,121 (4,234 to 6,173)	42,235 (36,383 to 48,264)	44,978 (38,885 to 51,184)	43,535 (37,543 to 50,404)	40,174 (34,662 to 46,631)	44,489 (40,487 to 49,101)	44,233 (40,359 to 48,476)
**Middle SDI**	15 to 19	Prevalence Rate	113.2 (103.2 to 124.2)	118.9 (108.8 to 130.1)	33.9 (28.2 to 40.6)	35.0 (29.0 to 41.6)	2.2 (1.8 to 2.7)	3.2 (2.6 to 3.9)	25.0 (21.5 to 28.6)	28.1 (24.3 to 31.9)	25.8 (22.2 to 29.8)	25.1 (21.6 to 29.1)	26.3 (24.0 to 29.1)	27.6 (25.2 to 30.2)
**Middle SDI**	20 to 49	Deaths Number	5,679 (5,282 to 6,409)	6,683 (6,181 to 7,077)										
**Middle SDI**	20 to 49	Prevalence Number	352,402 (322,136 to 384,207)	515,329 (473,599 to 560,318)	62,596 (51,358 to 75,699)	82,139 (67,090 to 99,477)	4,758 (3,808 to 5,865)	9,793 (8,076 to 11,924)	104,119 (90,616 to 117,916)	166,566 (145,493 to 188,339)	81,964 (70,916 to 94,296)	110,131 (95,889 to 126,731)	98,966 (90,584 to 108,078)	146,700 (134,829 to 159,766)
**Middle SDI**	20 to 49	Prevalence Rate	54.2 (49.6 to 59.1)	54.0 (49.6 to 58.7)	9.6 (7.9 to 11.6)	8.6 (7.0 to 10.4)	0.7 (0.6 to 0.9)	1.0 (0.8 to 1.2)	16.0 (13.9 to 18.1)	17.5 (15.2 to 19.7)	12.6 (10.9 to 14.5)	11.5 (10.0 to 13.3)	15.2 (13.9 to 16.6)	15.4 (14.1 to 16.7)

**Middle SDI**	50 plus	Deaths Number	434 (398 to 485)	1,063 (905 to 1,169)										
**Middle SDI**	50 plus	Prevalence Number	50,599 (46,136 to 55,947)	135,223 (122,970 to 149,456)	1,576 (1,217 to 1,981)	3,972 (3,062 to 4,968)	198 (155 to 250)	879 (704 to 1,088)	16,535 (14,529 to 18,821)	46,717 (41,094 to 52,680)	12,648 (10,929 to 14,630)	30,784 (26,563 to 35,567)	19,643 (17,865 to 21,789)	52,871 (47,919 to 58,440)
**Middle SDI**	50 plus	Prevalence Rate	24.6 (22.4 to 27.2)	28.3 (25.8 to 31.3)	0.8 (0.6 to 1.0)	0.8 (0.6 to 1.0)	0.1 (0.1 to 0.1)	0.2 (0.1 to 0.2)	8.0 (7.1 to 9.2)	9.8 (8.6 to 11.0)	6.2 (5.3 to 7.1)	6.4 (5.6 to 7.5)	9.6 (8.7 to 10.6)	11.1 (10.0 to 12.2)
**Low-middle SDI**	<1 year	Deaths Number	74,129 (44,629 to 101,945)	57,718 (43,140 to 73,955)										
**Low-middle SDI**	<1 year	Prevalence Number	420,166 (371,901 to 474,118)	497,266 (439,690 to 561,664)	148,019 (117,771 to 184,994)	174,146 (139,349 to 218,641)	21,380 (16,602 to 27,365)	22,129 (17,160 to 28,037)	100,187 (80,868 to 122,106)	118,211 (95,476 to 143,907)	83,426 (65,466 to 103,920)	103,279 (80,959 to 128,609)	67,154 (59,423 to 75,846)	79,500 (70,309 to 89,824)
**Low-middle SDI**	<1 year	Prevalence Rate	1,278.5 (1,131.7 to 1,442.7)	1,273.8 (1,126.3 to 1,438.8)	450.4 (358.4 to 562.9)	446.1 (357.0 to 560.1)	65.1 (50.5 to 83.3)	56.7 (44.0 to 71.8)	304.9 (246.1 to 371.6)	302.8 (244.6 to 368.6)	253.9 (199.2 to 316.2)	264.6 (207.4 to 329.5)	204.3 (180.8 to 230.8)	203.7 (180.1 to 230.1)
**Low-middle SDI**	1 to 4	Deaths Number	17,328 (7,098 to 26,824)	10,703 (7,056 to 14,940)										
**Low-middle SDI**	1 to 4	Prevalence Number	799,349 (706,932 to 894,862)	1,012,850 (902,100 to 1,131,639)	332,923 (267,926 to 413,358)	421,078 (337,970 to 514,461)	28,372 (22,155 to 36,043)	33,608 (26,382 to 42,057)	162,623 (137,556 to 192,896)	204,763 (173,016 to 240,364)	136,871 (112,271 to 166,060)	177,683 (146,038 to 214,853)	138,559 (122,480 to 155,752)	175,718 (155,925 to 196,962)
**Low-middle SDI**	1 to 4	Prevalence Rate	657.9 (581.8 to 736.5)	652.0 (580.7 to 728.5)	274.0 (220.5 to 340.2)	271.1 (217.6 to 331.2)	23.4 (18.2 to 29.7)	21.6 (17.0 to 27.1)	133.8 (113.2 to 158.8)	131.8 (111.4 to 154.7)	112.6 (92.4 to 136.7)	114.4 (94.0 to 138.3)	114.0 (100.8 to 128.2)	113.1 (100.4 to 126.8)
**Low-middle SDI**	5 to 9	Deaths Number	3,963 (2,430 to 5,512)	3,038 (2,497 to 3,729)										
**Low-middle SDI**	5 to 9	Prevalence Number	438,636 (395,236 to 487,494)	586,062 (529,640 to 652,261)	165,789 (134,944 to 199,086)	223,106 (182,842 to 267,588)	15,415 (12,225 to 19,292)	20,421 (16,375 to 25,217)	100,677 (86,248 to 116,685)	132,891 (113,623 to 153,347)	73,275 (61,254 to 87,109)	97,983 (81,795 to 117,075)	83,479 (75,091 to 93,021)	111,661 (100,735 to 124,328)
**Low-middle SDI**	5 to 9	Prevalence Rate	315.9 (284.6 to 351.1)	312.2 (282.1 to 347.4)	119.4 (97.2 to 143.4)	118.8 (97.4 to 142.5)	11.1 (8.8 to 13.9)	10.9 (8.7 to 13.4)	72.5 (62.1 to 84.0)	70.8 (60.5 to 81.7)	52.8 (44.1 to 62.7)	52.2 (43.6 to 62.4)	60.1 (54.1 to 67.0)	59.5 (53.7 to 66.2)
**Low-middle SDI**	10 to 14	Deaths Number	2,101 (1,546 to 2,728)	2,370 (2,072 to 2,725)										
**Low-middle SDI**	10 to 14	Prevalence Number	222,506 (202,128 to 245,595)	322,456 (293,553 to 355,866)	67,558 (55,710 to 81,230)	98,433 (81,739 to 117,382)	6,811 (5,433 to 8,473)	10,590 (8,485 to 12,940)	63,558 (54,642 to 73,354)	91,481 (78,668 to 105,371)	37,973 (31,937 to 44,847)	54,359 (45,895 to 63,972)	46,605 (42,219 to 51,397)	67,593 (61,239 to 74,652)
**Low-middle SDI**	10 to 14	Prevalence Rate	181.0 (164.4 to 199.8)	181.0 (164.8 to 199.7)	55.0 (45.3 to 66.1)	55.2 (45.9 to 65.9)	5.5 (4.4 to 6.9)	5.9 (4.8 to 7.3)	51.7 (44.4 to 59.7)	51.3 (44.2 to 59.1)	30.9 (26.0 to 36.5)	30.5 (25.8 to 35.9)	37.9 (34.3 to 41.8)	37.9 (34.4 to 41.9)
**Low-middle SDI**	15 to 19	Deaths Number	1,230 (988 to 1,586)	1,428 (1,273 to 1,598)										
**Low-middle SDI**	15 to 19	Prevalence Number	131,401 (119,828 to 143,990)	204,700 (187,224 to 223,701)	31,611 (25,986 to 38,156)	48,874 (40,402 to 58,902)	3,217 (2,579 to 3,960)	5,918 (4,836 to 7,214)	43,458 (37,370 to 49,692)	68,137 (58,835 to 77,598)	22,333 (19,031 to 26,215)	33,792 (28,689 to 39,584)	30,782 (28,030 to 33,776)	47,979 (43,827 to 52,465)

**Low-middle SDI**	15 to 19	Prevalence Rate	119.2 (108.7 to 130.6)	120.9 (110.5 to 132.1)	28.7 (23.6 to 34.6)	28.9 (23.9 to 34.8)	2.9 (2.3 to 3.6)	3.5 (2.9 to 4.3)	39.4 (33.9 to 45.1)	40.2 (34.7 to 45.8)	20.3 (17.3 to 23.8)	20.0 (16.9 to 23.4)	27.9 (25.4 to 30.6)	28.3 (25.9 to 31.0)
**Low-middle SDI**	20 to 49	Deaths Number	3,987 (3,309 to 5,142)	6,585 (6,036 to 7,117)										
**Low-middle SDI**	20 to 49	Prevalence Number	225,292 (205,323 to 246,838)	418,503 (382,218 to 459,153)	30,887 (24,797 to 37,541)	54,365 (43,854 to 66,002)	3,183 (2,512 to 3,930)	7,803 (6,305 to 9,493)	90,687 (79,046 to 102,615)	171,857 (151,005 to 194,179)	36,971 (31,706 to 42,958)	65,555 (56,148 to 76,051)	63,564 (57,940 to 69,837)	118,923 (108,622 to 130,936)
**Low-middle SDI**	20 to 49	Prevalence Rate	57.4 (52.3 to 62.9)	58.5 (53.4 to 64.1)	7.9 (6.3 to 9.6)	7.6 (6.1 to 9.2)	0.8 (0.6 to 1.0)	1.1 (0.9 to 1.3)	23.1 (20.1 to 26.1)	24.0 (21.1 to 27.1)	9.4 (8.1 to 10.9)	9.2 (7.8 to 10.6)	16.2 (14.8 to 17.8)	16.6 (15.2 to 18.3)
**Low-middle SDI**	50 plus	Deaths Number	348 (303 to 434)	824 (742 to 897)										
**Low-middle SDI**	50 plus	Prevalence Number	30,779 (27,824 to 34,191)	70,131 (63,407 to 77,700)	729 (548 to 928)	1,580 (1,199 to 2,015)	86 (65 to 109)	345 (265 to 434)	12,765 (11,230 to 14,463)	29,655 (26,044 to 33,598)	5,230 (4,475 to 6,137)	11,144 (9,519 to 12,887)	11,968 (10,802 to 13,271)	27,406 (24,758 to 30,383)
**Low-middle SDI**	50 plus	Prevalence Rate	25.3 (22.9 to 28.1)	28.0 (25.3 to 31.1)	0.6 (0.5 to 0.8)	0.6 (0.5 to 0.8)	0.1 (0.1 to 0.1)	0.1 (0.1 to 0.2)	10.5 (9.2 to 11.9)	11.9 (10.4 to 13.4)	4.3 (3.7 to 5.1)	4.5 (3.8 to 5.2)	9.9 (8.9 to 10.9)	11.0 (9.9 to 12.1)
**Low SDI**	<1 year	Deaths Number	48,277 (19,296 to 77,367)	61,159 (42,156 to 80,552)										
**Low SDI**	<1 year	Prevalence Number	433,864 (383,409 to 486,759)	587,072 (519,077 to 660,291)	133,103 (106,368 to 167,064)	179,794 (142,823 to 226,334)	22,530 (17,151 to 28,997)	27,738 (21,217 to 35,399)	127,017 (102,029 to 157,104)	168,535 (135,446 to 207,581)	81,747 (64,495 to 102,597)	117,017 (90,877 to 146,552)	69,466 (61,366 to 77,939)	93,987 (82,996 to 105,787)
**Low SDI**	<1 year	Prevalence Rate	1,613.0 (1,425.4 to 1,809.7)	1,611.3 (1,424.7 to 1,812.2)	494.9 (395.5 to 621.1)	493.5 (392.0 to 621.2)	83.8 (63.8 to 107.8)	76.1 (58.2 to 97.2)	472.2 (379.3 to 584.1)	462.6 (371.7 to 569.7)	303.9 (239.8 to 381.4)	321.2 (249.4 to 402.2)	258.3 (228.1 to 289.8)	258.0 (227.8 to 290.3)
**Low SDI**	1 to 4	Deaths Number	14,586 (2,847 to 25,588)	14,056 (6,954 to 21,259)										
**Low SDI**	1 to 4	Prevalence Number	729,073 (646,100 to 818,763)	1,089,865 (969,030 to 1,220,518)	279,303 (225,072 to 342,954)	417,618 (333,718 to 515,880)	24,911 (19,344 to 32,222)	35,919 (27,831 to 45,570)	181,325 (152,501 to 215,354)	264,089 (223,299 to 311,525)	116,537 (95,303 to 142,222)	182,428 (149,291 to 223,525)	126,997 (112,371 to 143,030)	189,810 (167,792 to 213,011)
**Low SDI**	1 to 4	Prevalence Rate	768.0 (680.6 to 862.5)	766.6 (681.6 to 858.5)	294.2 (237.1 to 361.3)	293.7 (234.7 to 362.9)	26.2 (20.4 to 33.9)	25.3 (19.6 to 32.1)	191.0 (160.6 to 226.9)	185.8 (157.1 to 219.1)	122.8 (100.4 to 149.8)	128.3 (105.0 to 157.2)	133.8 (118.4 to 150.7)	133.5 (118.0 to 149.8)
**Low SDI**	5 to 9	Deaths Number	1,749 (808 to 3,233)	2,361 (1,791 to 3,083)										
**Low SDI**	5 to 9	Prevalence Number	357,930 (321,852 to 396,114)	583,101 (524,823 to 646,649)	126,439 (103,302 to 152,554)	206,613 (168,625 to 247,949)	11,145 (8,607 to 14,166)	18,719 (14,708 to 23,712)	97,637 (83,290 to 113,298)	155,010 (132,591 to 179,288)	54,327 (44,885 to 65,334)	91,314 (75,662 to 109,793)	68,381 (61,328 to 75,921)	111,444 (100,113 to 124,022)
**Low SDI**	5 to 9	Prevalence Rate	346.3 (311.4 to 383.2)	345.1 (310.6 to 382.7)	122.3 (99.9 to 147.6)	122.3 (99.8 to 146.7)	10.8 (8.3 to 13.7)	11.1 (8.7 to 14.0)	94.5 (80.6 to 109.6)	91.7 (78.5 to 106.1)	52.6 (43.4 to 63.2)	54.0 (44.8 to 65.0)	66.2 (59.3 to 73.5)	66.0 (59.2 to 73.4)
**Low SDI**	10 to 14	Deaths Number	784 (475 to 1,362)	1,511 (1,235 to 1,825)										

**Low SDI**	10 to 14	Prevalence Number	162,960 (147,141 to 180,384)	299,066 (270,623 to 329,901)	45,914 (37,708 to 55,245)	84,698 (69,797 to 101,367)	4,000 (3,089 to 5,072)	8,346 (6,566 to 10,446)	54,237 (46,440 to 62,615)	97,179 (83,068 to 111,850)	24,522 (20,558 to 28,873)	45,873 (38,433 to 54,213)	34,286 (30,932 to 37,996)	62,970 (56,814 to 69,499)
**Low SDI**	10 to 14	Prevalence Rate	191.3 (172.7 to 211.7)	191.0 (172.8 to 210.7)	53.9 (44.3 to 64.9)	54.1 (44.6 to 64.7)	4.7 (3.6 to 6.0)	5.3 (4.2 to 6.7)	63.7 (54.5 to 73.5)	62.1 (53.1 to 71.4)	28.8 (24.1 to 33.9)	29.3 (24.5 to 34.6)	40.2 (36.3 to 44.6)	40.2 (36.3 to 44.4)
**Low SDI**	15 to 19	Deaths Number	408 (270 to 712)	825 (712 to 985)										
**Low SDI**	15 to 19	Prevalence Number	87,819 (79,526 to 96,342)	174,106 (157,804 to 191,127)	19,479 (16,023 to 23,398)	38,844 (31,793 to 46,654)	1,601 (1,250 to 2,010)	3,936 (3,111 to 4,861)	33,303 (28,675 to 38,203)	64,761 (55,573 to 74,348)	12,791 (10,800 to 15,094)	25,605 (21,566 to 30,057)	20,645 (18,683 to 22,689)	40,961 (37,079 to 44,955)
**Low SDI**	15 to 19	Prevalence Rate	123.6 (112.0 to 135.6)	124.0 (112.4 to 136.1)	27.4 (22.6 to 32.9)	27.7 (22.6 to 33.2)	2.3 (1.8 to 2.8)	2.8 (2.2 to 3.5)	46.9 (40.4 to 53.8)	46.1 (39.6 to 53.0)	18.0 (15.2 to 21.3)	18.2 (15.4 to 21.4)	29.1 (26.3 to 31.9)	29.2 (26.4 to 32.0)
**Low SDI**	20 to 49	Deaths Number	1,371 (1,013 to 2,297)	3,492 (3,171 to 3,915)										
**Low SDI**	20 to 49	Prevalence Number	141,524 (127,946 to 155,708)	291,582 (264,032 to 319,570)	17,991 (14,469 to 21,735)	36,773 (29,627 to 44,480)	1,287 (991 to 1,631)	3,723 (2,916 to 4,644)	62,325 (54,089 to 71,260)	127,850 (111,310 to 145,527)	19,778 (16,814 to 23,224)	40,317 (34,240 to 47,303)	40,143 (36,297 to 44,216)	82,920 (75,011 to 91,099)
**Low SDI**	20 to 49	Prevalence Rate	58.2 (52.7 to 64.1)	59.0 (53.4 to 64.7)	7.4 (6.0 to 8.9)	7.4 (6.0 to 9.0)	0.5 (0.4 to 0.7)	0.8 (0.6 to 0.9)	25.7 (22.3 to 29.3)	25.9 (22.5 to 29.4)	8.1 (6.9 to 9.6)	8.2 (6.9 to 9.6)	16.5 (14.9 to 18.2)	16.8 (15.2 to 18.4)
**Low SDI**	50 plus	Deaths Number	165 (131 to 250)	470 (403 to 542)										
**Low SDI**	50 plus	Prevalence Number	16,844 (15,148 to 18,834)	37,291 (33,502 to 41,683)	381 (282 to 493)	800 (589 to 1,037)	19 (13 to 26)	93 (66 to 121)	7,323 (6,411 to 8,334)	16,431 (14,373 to 18,772)	2,566 (2,173 to 2,996)	5,325 (4,530 to 6,247)	6,555 (5,891 to 7,322)	14,642 (13,149 to 16,363)
**Low SDI**	50 plus	Prevalence Rate	23.6 (21.2 to 26.4)	25.5 (22.9 to 28.5)	0.5 (0.4 to 0.7)	0.5 (0.4 to 0.7)	0.0 (0.0 to 0.0)	0.1 (0.0 to 0.1)	10.3 (9.0 to 11.7)	11.2 (9.8 to 12.8)	3.6 (3.0 to 4.2)	3.6 (3.1 to 4.3)	9.2 (8.3 to 10.3)	10.0 (9.0 to 11.2)
**Andean Latin America**	<1 year	Deaths Number	1,402 (989 to 1,973)	1,471 (946 to 1,951)										
**Andean Latin America**	<1 year	Prevalence Number	11,751 (10,357 to 13,206)	12,750 (11,314 to 14,442)	3,626 (2,889 to 4,556)	3,907 (3,122 to 4,840)	397 (311 to 497)	417 (331 to 524)	2,750 (2,238 to 3,346)	2,904 (2,354 to 3,555)	3,096 (2,444 to 3,909)	3,482 (2,760 to 4,378)	1,881 (1,657 to 2,116)	2,041 (1,811 to 2,311)
**Andean Latin America**	<1 year	Prevalence Rate	964.0 (849.6 to 1,083.3)	936.9 (831.4 to 1,061.3)	297.5 (237.0 to 373.7)	287.1 (229.4 to 355.7)	32.6 (25.5 to 40.7)	30.6 (24.3 to 38.5)	225.6 (183.6 to 274.5)	213.4 (173.0 to 261.2)	254.0 (200.5 to 320.7)	255.9 (202.8 to 321.7)	154.3 (135.9 to 173.5)	150.0 (133.1 to 169.8)
**Andean Latin America**	1 to 4	Deaths Number	296 (172 to 439)	192 (106 to 266)										
**Andean Latin America**	1 to 4	Prevalence Number	22,859 (20,487 to 25,463)	27,156 (24,361 to 30,650)	7,904 (6,406 to 9,759)	9,294 (7,540 to 11,312)	644 (501 to 805)	759 (599 to 938)	4,642 (3,952 to 5,421)	5,403 (4,605 to 6,306)	5,695 (4,686 to 6,869)	6,979 (5,786 to 8,479)	3,975 (3,561 to 4,437)	4,720 (4,231 to 5,330)
**Andean Latin America**	1 to 4	Prevalence Rate	521.5 (467.4 to 580.9)	508.5 (456.2 to 573.9)	180.3 (146.1 to 222.6)	174.0 (141.2 to 211.8)	14.7 (11.4 to 18.4)	14.2 (11.2 to 17.6)	105.9 (90.1 to 123.7)	101.2 (86.2 to 118.1)	129.9 (106.9 to 156.7)	130.7 (108.3 to 158.8)	90.7 (81.2 to 101.2)	88.4 (79.2 to 99.8)

**Andean Latin America**	5 to 9	Deaths Number	58 (46 to 73)	72 (51 to 91)										
**Andean Latin America**	5 to 9	Prevalence Number	13,323 (12,081 to 14,734)	16,146 (14,633 to 17,742)	3,858 (3,169 to 4,661)	4,662 (3,833 to 5,552)	407 (325 to 504)	500 (403 to 615)	3,171 (2,736 to 3,655)	3,771 (3,259 to 4,300)	3,345 (2,793 to 4,000)	4,138 (3,451 to 4,953)	2,541 (2,298 to 2,812)	3,075 (2,782 to 3,396)
**Andean Latin America**	5 to 9	Prevalence Rate	268.6 (243.6 to 297.1)	264.4 (239.7 to 290.6)	77.8 (63.9 to 94.0)	76.3 (62.8 to 90.9)	8.2 (6.5 to 10.2)	8.2 (6.6 to 10.1)	63.9 (55.2 to 73.7)	61.8 (53.4 to 70.4)	67.5 (56.3 to 80.7)	67.8 (56.5 to 81.1)	51.2 (46.3 to 56.7)	50.4 (45.6 to 55.6)
**Andean Latin America**	10 to 14	Deaths Number	43 (37 to 52)	58 (46 to 68)										
**Andean Latin America**	10 to 14	Prevalence Number	7,733 (7,081 to 8,470)	9,034 (8,287 to 9,903)	1,714 (1,418 to 2,078)	2,024 (1,671 to 2,405)	224 (180 to 273)	271 (220 to 330)	2,291 (1,979 to 2,612)	2,631 (2,284 to 2,989)	1,880 (1,591 to 2,204)	2,218 (1,880 to 2,605)	1,625 (1,481 to 1,784)	1,890 (1,728 to 2,078)
**Andean Latin America**	10 to 14	Prevalence Rate	166.4 (152.3 to 182.2)	166.4 (152.6 to 182.4)	36.9 (30.5 to 44.7)	37.3 (30.8 to 44.3)	4.8 (3.9 to 5.9)	5.0 (4.0 to 6.1)	49.3 (42.6 to 56.2)	48.4 (42.1 to 55.0)	40.4 (34.2 to 47.4)	40.9 (34.6 to 48.0)	35.0 (31.9 to 38.4)	34.8 (31.8 to 38.3)
**Andean Latin America**	15 to 19	Deaths Number	23 (19 to 27)	41 (31 to 49)										
**Andean Latin America**	15 to 19	Prevalence Number	4,844 (4,434 to 5,294)	6,511 (5,990 to 7,117)	833 (680 to 1,018)	1,139 (936 to 1,358)	121 (98 to 148)	173 (142 to 211)	1,651 (1,431 to 1,886)	2,198 (1,904 to 2,496)	1,097 (934 to 1,281)	1,475 (1,253 to 1,726)	1,142 (1,043 to 1,250)	1,526 (1,402 to 1,666)
**Andean Latin America**	15 to 19	Prevalence Rate	118.3 (108.3 to 129.3)	119.0 (109.5 to 130.1)	20.4 (16.6 to 24.9)	20.8 (17.1 to 24.8)	3.0 (2.4 to 3.6)	3.2 (2.6 to 3.9)	40.3 (35.0 to 46.1)	40.2 (34.8 to 45.6)	26.8 (22.8 to 31.3)	27.0 (22.9 to 31.5)	27.9 (25.5 to 30.5)	27.9 (25.6 to 30.4)
**Andean Latin America**	20 to 49	Deaths Number	63 (56 to 72)	155 (112 to 181)										
**Andean Latin America**	20 to 49	Prevalence Number	9,716 (8,868 to 10,665)	17,165 (15,706 to 18,764)	924 (737 to 1,127)	1,572 (1,263 to 1,925)	155 (125 to 190)	295 (241 to 361)	3,941 (3,450 to 4,484)	7,055 (6,199 to 7,996)	1,919 (1,651 to 2,229)	3,313 (2,857 to 3,846)	2,777 (2,531 to 3,050)	4,930 (4,511 to 5,405)
**Andean Latin America**	20 to 49	Prevalence Rate	66.8 (60.9 to 73.3)	65.6 (60.0 to 71.7)	6.3 (5.1 to 7.7)	6.0 (4.8 to 7.4)	1.1 (0.9 to 1.3)	1.1 (0.9 to 1.4)	27.1 (23.7 to 30.8)	27.0 (23.7 to 30.6)	13.2 (11.3 to 15.3)	12.7 (10.9 to 14.7)	19.1 (17.4 to 21.0)	18.8 (17.2 to 20.7)
**Andean Latin America**	50 plus	Deaths Number	6 (5 to 8)	37 (23 to 47)										
**Andean Latin America**	50 plus	Prevalence Number	1,629 (1,480 to 1,811)	4,397 (3,977 to 4,848)	27 (21 to 35)	69 (53 to 89)	8 (6 to 10)	26 (20 to 32)	673 (589 to 766)	1,829 (1,611 to 2,078)	285 (245 to 332)	735 (633 to 852)	637 (577 to 709)	1,739 (1,568 to 1,921)
**Andean Latin America**	50 plus	Prevalence Rate	37.7 (34.2 to 41.9)	39.9 (36.1 to 44.0)	0.6 (0.5 to 0.8)	0.6 (0.5 to 0.8)	0.2 (0.1 to 0.2)	0.2 (0.2 to 0.3)	15.6 (13.6 to 17.7)	16.6 (14.6 to 18.8)	6.6 (5.7 to 7.7)	6.7 (5.7 to 7.7)	14.7 (13.3 to 16.4)	15.8 (14.2 to 17.4)
**Australasia**	<1 year	Deaths Number	220 (188 to 255)	96 (72 to 124)										
**Australasia**	<1 year	Prevalence Number	2,453 (2,129 to 2,847)	3,008 (2,608 to 3,464)	1,224 (959 to 1,550)	1,545 (1,227 to 1,902)	48 (39 to 59)	59 (47 to 72)	389 (332 to 466)	459 (377 to 563)	401 (320 to 503)	464 (365 to 586)	392 (339 to 455)	481 (417 to 554)

**Australasia**	<1 year	Prevalence Rate	784.7 (680.8 to 910.5)	824.8 (715.0 to 949.8)	391.4 (306.8 to 495.8)	423.7 (336.5 to 521.6)	15.3 (12.4 to 18.9)	16.2 (12.9 to 19.8)	124.3 (106.3 to 149.1)	126.0 (103.4 to 154.3)	128.4 (102.5 to 161.0)	127.1 (100.1 to 160.7)	125.3 (108.6 to 145.5)	131.8 (114.3 to 151.8)
**Australasia**	1 to 4	Deaths Number	34 (25 to 41)	9 (6 to 13)										
**Australasia**	1 to 4	Prevalence Number	6,154 (5,340 to 7,105)	7,593 (6,600 to 8,696)	3,230 (2,570 to 3,959)	4,102 (3,277 to 5,011)	122 (100 to 150)	149 (121 to 183)	821 (726 to 932)	962 (839 to 1,109)	916 (748 to 1,118)	1,058 (866 to 1,293)	1,065 (921 to 1,236)	1,322 (1,144 to 1,517)
**Australasia**	1 to 4	Prevalence Rate	501.3 (435.0 to 578.8)	526.0 (457.2 to 602.4)	263.1 (209.3 to 322.5)	284.2 (227.0 to 347.1)	10.0 (8.2 to 12.2)	10.3 (8.4 to 12.7)	66.9 (59.2 to 75.9)	66.6 (58.1 to 76.8)	74.6 (60.9 to 91.1)	73.3 (60.0 to 89.6)	86.7 (75.0 to 100.6)	91.6 (79.2 to 105.1)
**Australasia**	5 to 9	Deaths Number	10 (7 to 12)	5 (4 to 6)										
**Australasia**	5 to 9	Prevalence Number	4,396 (3,859 to 4,955)	5,571 (4,879 to 6,294)	2,138 (1,744 to 2,542)	2,779 (2,244 to 3,318)	104 (86 to 125)	131 (107 to 160)	692 (608 to 781)	848 (740 to 965)	619 (517 to 744)	741 (615 to 898)	843 (742 to 953)	1,072 (937 to 1,213)
**Australasia**	5 to 9	Prevalence Rate	287.8 (252.7 to 324.5)	301.4 (264.0 to 340.6)	140.0 (114.2 to 166.5)	150.3 (121.4 to 179.5)	6.8 (5.6 to 8.2)	7.1 (5.8 to 8.7)	45.3 (39.8 to 51.1)	45.9 (40.1 to 52.2)	40.5 (33.9 to 48.7)	40.1 (33.3 to 48.6)	55.2 (48.6 to 62.4)	58.0 (50.7 to 65.6)
**Australasia**	10 to 14	Deaths Number	9 (7 to 11)	5 (4 to 7)										
**Australasia**	10 to 14	Prevalence Number	2,834 (2,533 to 3,133)	3,383 (3,017 to 3,750)	1,176 (962 to 1,386)	1,438 (1,175 to 1,702)	72 (59 to 86)	86 (70 to 104)	605 (531 to 684)	706 (615 to 801)	382 (320 to 453)	434 (362 to 515)	600 (536 to 664)	719 (641 to 799)
**Australasia**	10 to 14	Prevalence Rate	187.0 (167.1 to 206.7)	193.6 (172.7 to 214.6)	77.6 (63.5 to 91.4)	82.3 (67.3 to 97.4)	4.7 (3.9 to 5.6)	4.9 (4.0 to 6.0)	39.9 (35.0 to 45.2)	40.4 (35.2 to 45.8)	25.2 (21.1 to 29.9)	24.9 (20.7 to 29.5)	39.6 (35.4 to 43.8)	41.2 (36.7 to 45.7)
**Australasia**	15 to 19	Deaths Number	14 (10 to 16)	6 (4 to 8)										
**Australasia**	15 to 19	Prevalence Number	2,267 (2,045 to 2,483)	2,390 (2,169 to 2,612)	776 (640 to 914)	834 (684 to 988)	57 (47 to 67)	60 (50 to 73)	608 (534 to 687)	634 (553 to 717)	291 (245 to 344)	294 (249 to 348)	536 (482 to 588)	568 (514 to 621)
**Australasia**	15 to 19	Prevalence Rate	134.3 (121.1 to 147.1)	138.0 (125.2 to 150.8)	45.9 (37.9 to 54.1)	48.1 (39.5 to 57.0)	3.4 (2.8 to 4.0)	3.5 (2.9 to 4.2)	36.0 (31.6 to 40.7)	36.6 (31.9 to 41.4)	17.2 (14.5 to 20.4)	17.0 (14.4 to 20.1)	31.8 (28.6 to 34.8)	32.8 (29.7 to 35.9)
**Australasia**	20 to 49	Deaths Number	59 (50 to 71)	49 (40 to 59)										
**Australasia**	20 to 49	Prevalence Number	6,560 (6,002 to 7,107)	8,298 (7,614 to 9,029)	1,202 (991 to 1,434)	1,507 (1,220 to 1,786)	140 (116 to 168)	177 (146 to 213)	2,577 (2,270 to 2,901)	3,285 (2,888 to 3,710)	776 (664 to 914)	941 (803 to 1,107)	1,864 (1,698 to 2,026)	2,388 (2,186 to 2,604)
**Australasia**	20 to 49	Prevalence Rate	72.1 (65.9 to 78.1)	71.5 (65.6 to 77.8)	13.2 (10.9 to 15.8)	13.0 (10.5 to 15.4)	1.5 (1.3 to 1.8)	1.5 (1.3 to 1.8)	28.3 (24.9 to 31.9)	28.3 (24.9 to 32.0)	8.5 (7.3 to 10.0)	8.1 (6.9 to 9.5)	20.5 (18.7 to 22.3)	20.6 (18.8 to 22.4)
**Australasia**	50 plus	Deaths Number	28 (24 to 39)	26 (21 to 33)										
**Australasia**	50 plus	Prevalence Number	2,069 (1,867 to 2,273)	4,128 (3,728 to 4,555)	64 (49 to 81)	124 (96 to 157)	23 (18 to 30)	43 (34 to 53)	961 (853 to 1,078)	1,906 (1,689 to 2,147)	212 (180 to 250)	405 (342 to 481)	809 (729 to 893)	1,649 (1,486 to 1,828)

**Australasia**	50 plus	Prevalence Rate	44.6 (40.2 to 49.0)	45.4 (41.0 to 50.1)	1.4 (1.1 to 1.7)	1.4 (1.1 to 1.7)	0.5 (0.4 to 0.6)	0.5 (0.4 to 0.6)	20.7 (18.4 to 23.3)	21.0 (18.6 to 23.6)	4.6 (3.9 to 5.4)	4.5 (3.8 to 5.3)	17.4 (15.7 to 19.3)	18.1 (16.3 to 20.1)
**Caribbean**	<1 year	Deaths Number	1,947 (1,234 to 2,504)	1,537 (1,029 to 2,147)										
**Caribbean**	<1 year	Prevalence Number	9,257 (8,166 to 10,513)	9,048 (8,000 to 10,325)	3,786 (2,997 to 4,634)	3,458 (2,762 to 4,228)	153 (120 to 191)	150 (118 to 190)	1,715 (1,406 to 2,062)	1,797 (1,471 to 2,171)	2,129 (1,716 to 2,640)	2,199 (1,776 to 2,743)	1,475 (1,299 to 1,674)	1,442 (1,275 to 1,648)
**Caribbean**	<1 year	Prevalence Rate	1,039.7 (917.2 to 1,180.8)	1,138.8 (1,007.0 to 1,299.6)	425.2 (336.6 to 520.5)	435.3 (347.7 to 532.1)	17.2 (13.4 to 21.4)	18.9 (14.8 to 23.9)	192.6 (157.9 to 231.6)	226.2 (185.1 to 273.2)	239.1 (192.7 to 296.5)	276.8 (223.6 to 345.3)	165.6 (146.0 to 188.0)	181.6 (160.5 to 207.5)
**Caribbean**	1 to 4	Deaths Number	458 (163 to 737)	247 (120 to 375)										
**Caribbean**	1 to 4	Prevalence Number	19,359 (17,153 to 21,843)	19,139 (17,057 to 21,470)	8,615 (6,896 to 10,401)	8,188 (6,610 to 9,873)	271 (215 to 343)	269 (212 to 342)	3,012 (2,574 to 3,506)	3,152 (2,696 to 3,661)	4,108 (3,441 to 4,919)	4,207 (3,475 to 5,131)	3,353 (2,961 to 3,789)	3,322 (2,957 to 3,740)
**Caribbean**	1 to 4	Prevalence Rate	589.0 (521.9 to 664.5)	613.9 (547.1 to 688.7)	262.1 (209.8 to 316.5)	262.7 (212.0 to 316.7)	8.2 (6.6 to 10.4)	8.6 (6.8 to 11.0)	91.6 (78.3 to 106.7)	101.1 (86.5 to 117.4)	125.0 (104.7 to 149.7)	135.0 (111.5 to 164.6)	102.0 (90.1 to 115.3)	106.6 (94.8 to 120.0)
**Caribbean**	5 to 9	Deaths Number	89 (62 to 114)	64 (47 to 83)										
**Caribbean**	5 to 9	Prevalence Number	11,393 (10,282 to 12,692)	11,626 (10,491 to 12,901)	4,325 (3,524 to 5,160)	4,256 (3,525 to 5,031)	174 (138 to 217)	176 (139 to 221)	2,142 (1,855 to 2,457)	2,294 (1,980 to 2,633)	2,574 (2,169 to 3,036)	2,675 (2,254 to 3,191)	2,179 (1,961 to 2,431)	2,225 (2,006 to 2,477)
**Caribbean**	5 to 9	Prevalence Rate	305.2 (275.4 to 340.0)	310.6 (280.3 to 344.7)	115.9 (94.4 to 138.2)	113.7 (94.2 to 134.4)	4.7 (3.7 to 5.8)	4.7 (3.7 to 5.9)	57.4 (49.7 to 65.8)	61.3 (52.9 to 70.4)	68.9 (58.1 to 81.3)	71.5 (60.2 to 85.2)	58.4 (52.5 to 65.1)	59.5 (53.6 to 66.2)
**Caribbean**	10 to 14	Deaths Number	45 (37 to 55)	41 (33 to 49)										
**Caribbean**	10 to 14	Prevalence Number	6,586 (6,027 to 7,231)	7,021 (6,425 to 7,681)	1,974 (1,642 to 2,346)	2,032 (1,699 to 2,394)	100 (80 to 124)	104 (83 to 130)	1,590 (1,380 to 1,808)	1,768 (1,533 to 2,023)	1,526 (1,302 to 1,756)	1,629 (1,400 to 1,912)	1,395 (1,274 to 1,539)	1,487 (1,360 to 1,631)
**Caribbean**	10 to 14	Prevalence Rate	185.3 (169.6 to 203.5)	187.5 (171.5 to 205.1)	55.5 (46.2 to 66.0)	54.2 (45.4 to 63.9)	2.8 (2.3 to 3.5)	2.8 (2.2 to 3.5)	44.7 (38.8 to 50.9)	47.2 (40.9 to 54.0)	43.0 (36.6 to 49.4)	43.5 (37.4 to 51.0)	39.3 (35.9 to 43.3)	39.7 (36.3 to 43.6)
**Caribbean**	15 to 19	Deaths Number	41 (34 to 50)	35 (29 to 41)										
**Caribbean**	15 to 19	Prevalence Number	4,793 (4,406 to 5,218)	5,064 (4,643 to 5,530)	1,145 (954 to 1,372)	1,168 (966 to 1,377)	69 (55 to 84)	70 (56 to 86)	1,360 (1,183 to 1,542)	1,495 (1,298 to 1,703)	1,082 (934 to 1,253)	1,130 (974 to 1,311)	1,137 (1,043 to 1,246)	1,201 (1,099 to 1,313)
**Caribbean**	15 to 19	Prevalence Rate	130.0 (119.5 to 141.5)	130.7 (119.9 to 142.8)	31.1 (25.9 to 37.2)	30.1 (24.9 to 35.5)	1.9 (1.5 to 2.3)	1.8 (1.4 to 2.2)	36.9 (32.1 to 41.8)	38.6 (33.5 to 44.0)	29.3 (25.3 to 34.0)	29.2 (25.2 to 33.8)	30.8 (28.3 to 33.8)	31.0 (28.4 to 33.9)
**Caribbean**	20 to 49	Deaths Number	138 (119 to 158)	156 (135 to 178)										

**Caribbean**	20 to 49	Prevalence Number	10,114 (9,307 to 10,995)	13,229 (12,166 to 14,396)	1,390 (1,143 to 1,677)	1,669 (1,359 to 1,982)	107 (86 to 130)	132 (107 to 161)	3,628 (3,199 to 4,116)	4,971 (4,364 to 5,630)	2,108 (1,834 to 2,432)	2,649 (2,308 to 3,046)	2,882 (2,646 to 3,140)	3,807 (3,502 to 4,142)
**Caribbean**	20 to 49	Prevalence Rate	69.5 (63.9 to 75.5)	66.5 (61.1 to 72.3)	9.6 (7.8 to 11.5)	8.4 (6.8 to 10.0)	0.7 (0.6 to 0.9)	0.7 (0.5 to 0.8)	24.9 (22.0 to 28.3)	25.0 (21.9 to 28.3)	14.5 (12.6 to 16.7)	13.3 (11.6 to 15.3)	19.8 (18.2 to 21.6)	19.1 (17.6 to 20.8)
**Caribbean**	50 plus	Deaths Number	25 (20 to 28)	42 (33 to 49)										
**Caribbean**	50 plus	Prevalence Number	1,992 (1,821 to 2,186)	4,116 (3,768 to 4,514)	48 (37 to 60)	97 (76 to 118)	8 (6 to 10)	18 (14 to 23)	761 (670 to 853)	1,607 (1,416 to 1,811)	384 (335 to 443)	760 (661 to 873)	792 (722 to 870)	1,634 (1,494 to 1,790)
**Caribbean**	50 plus	Prevalence Rate	37.3 (34.1 to 40.9)	39.0 (35.7 to 42.7)	0.9 (0.7 to 1.1)	0.9 (0.7 to 1.1)	0.1 (0.1 to 0.2)	0.2 (0.1 to 0.2)	14.3 (12.5 to 16.0)	15.2 (13.4 to 17.1)	7.2 (6.3 to 8.3)	7.2 (6.3 to 8.3)	14.8 (13.5 to 16.3)	15.5 (14.1 to 16.9)
**Central Asia**	<1 year	Deaths Number	2,922 (2,441 to 3,311)	2,251 (1,781 to 2,799)										
**Central Asia**	<1 year	Prevalence Number	23,675 (20,601 to 27,127)	22,299 (19,400 to 25,754)	12,311 (9,876 to 15,213)	11,412 (9,121 to 14,189)	466 (368 to 582)	445 (352 to 555)	4,296 (3,517 to 5,211)	4,183 (3,473 to 5,073)	2,828 (2,230 to 3,555)	2,698 (2,144 to 3,359)	3,775 (3,282 to 4,330)	3,560 (3,095 to 4,120)
**Central Asia**	<1 year	Prevalence Rate	1,215.2 (1,057.5 to 1,392.5)	1,180.1 (1,026.7 to 1,362.9)	631.9 (506.9 to 780.9)	603.9 (482.7 to 750.9)	23.9 (18.9 to 29.9)	23.6 (18.6 to 29.4)	220.5 (180.6 to 267.5)	221.4 (183.8 to 268.5)	145.2 (114.5 to 182.5)	142.8 (113.4 to 177.8)	193.8 (168.5 to 222.3)	188.4 (163.8 to 218.0)
**Central Asia**	1 to 4	Deaths Number	506 (335 to 654)	356 (244 to 463)										
**Central Asia**	1 to 4	Prevalence Number	54,181 (47,034 to 62,106)	53,815 (46,738 to 61,674)	30,434 (24,745 to 36,810)	30,058 (24,206 to 36,591)	931 (747 to 1,167)	931 (742 to 1,156)	7,775 (6,628 to 9,039)	7,876 (6,768 to 9,146)	5,653 (4,614 to 6,843)	5,635 (4,645 to 6,874)	9,388 (8,129 to 10,766)	9,316 (8,057 to 10,701)
**Central Asia**	1 to 4	Prevalence Rate	709.7 (616.1 to 813.5)	699.1 (607.2 to 801.2)	398.6 (324.1 to 482.2)	390.5 (314.5 to 475.3)	12.2 (9.8 to 15.3)	12.1 (9.6 to 15.0)	101.8 (86.8 to 118.4)	102.3 (87.9 to 118.8)	74.0 (60.4 to 89.6)	73.2 (60.3 to 89.3)	123.0 (106.5 to 141.0)	121.0 (104.7 to 139.0)
**Central Asia**	5 to 9	Deaths Number	126 (105 to 146)	119 (101 to 139)										
**Central Asia**	5 to 9	Prevalence Number	30,425 (26,810 to 34,268)	32,451 (28,475 to 36,583)	15,657 (12,847 to 18,568)	16,849 (13,812 to 19,939)	599 (488 to 739)	629 (509 to 772)	5,152 (4,468 to 5,894)	5,428 (4,686 to 6,209)	3,208 (2,685 to 3,841)	3,360 (2,813 to 4,018)	5,809 (5,124 to 6,552)	6,185 (5,416 to 6,978)
**Central Asia**	5 to 9	Prevalence Rate	366.5 (322.9 to 412.8)	370.6 (325.2 to 417.8)	188.6 (154.7 to 223.6)	192.4 (157.7 to 227.7)	7.2 (5.9 to 8.9)	7.2 (5.8 to 8.8)	62.1 (53.8 to 71.0)	62.0 (53.5 to 70.9)	38.6 (32.3 to 46.3)	38.4 (32.1 to 45.9)	70.0 (61.7 to 78.9)	70.6 (61.9 to 79.7)
**Central Asia**	10 to 14	Deaths Number	81 (68 to 92)	81 (70 to 93)										
**Central Asia**	10 to 14	Prevalence Number	15,997 (14,348 to 17,837)	16,385 (14,639 to 18,289)	6,935 (5,771 to 8,259)	7,234 (6,103 to 8,520)	331 (269 to 404)	332 (269 to 405)	3,610 (3,135 to 4,110)	3,633 (3,151 to 4,123)	1,760 (1,480 to 2,080)	1,753 (1,467 to 2,059)	3,361 (3,007 to 3,752)	3,433 (3,055 to 3,837)
**Central Asia**	10 to 14	Prevalence Rate	219.4 (196.8 to 244.6)	223.7 (199.9 to 249.7)	95.1 (79.2 to 113.3)	98.8 (83.3 to 116.3)	4.5 (3.7 to 5.5)	4.5 (3.7 to 5.5)	49.5 (43.0 to 56.4)	49.6 (43.0 to 56.3)	24.1 (20.3 to 28.5)	23.9 (20.0 to 28.1)	46.1 (41.2 to 51.5)	46.9 (41.7 to 52.4)

**Central Asia**	15 to 19	Deaths Number	52 (42 to 58)	59 (48 to 68)										
**Central Asia**	15 to 19	Prevalence Number	9,826 (8,854 to 10,862)	10,570 (9,522 to 11,599)	3,498 (2,913 to 4,175)	3,842 (3,231 to 4,508)	193 (158 to 234)	205 (167 to 248)	2,743 (2,388 to 3,116)	2,912 (2,537 to 3,297)	1,083 (918 to 1,278)	1,132 (957 to 1,332)	2,309 (2,076 to 2,559)	2,479 (2,230 to 2,727)
**Central Asia**	15 to 19	Prevalence Rate	148.3 (133.6 to 163.9)	151.0 (136.1 to 165.7)	52.8 (44.0 to 63.0)	54.9 (46.2 to 64.4)	2.9 (2.4 to 3.5)	2.9 (2.4 to 3.5)	41.4 (36.0 to 47.0)	41.6 (36.2 to 47.1)	16.3 (13.9 to 19.3)	16.2 (13.7 to 19.0)	34.8 (31.3 to 38.6)	35.4 (31.9 to 39.0)
**Central Asia**	20 to 49	Deaths Number	128 (116 to 167)	244 (195 to 270)										
**Central Asia**	20 to 49	Prevalence Number	20,212 (18,532 to 22,105)	29,702 (27,063 to 32,403)	4,257 (3,509 to 5,114)	6,090 (5,007 to 7,156)	299 (244 to 365)	428 (349 to 521)	7,650 (6,776 to 8,626)	11,453 (10,099 to 12,913)	2,226 (1,908 to 2,600)	3,185 (2,709 to 3,753)	5,780 (5,294 to 6,327)	8,547 (7,781 to 9,336)
**Central Asia**	20 to 49	Prevalence Rate	74.9 (68.7 to 81.9)	72.4 (66.0 to 79.0)	15.8 (13.0 to 19.0)	14.8 (12.2 to 17.4)	1.1 (0.9 to 1.4)	1.0 (0.9 to 1.3)	28.4 (25.1 to 32.0)	27.9 (24.6 to 31.5)	8.3 (7.1 to 9.6)	7.8 (6.6 to 9.1)	21.4 (19.6 to 23.5)	20.8 (19.0 to 22.8)
**Central Asia**	50 plus	Deaths Number	19 (16 to 27)	44 (34 to 50)										
**Central Asia**	50 plus	Prevalence Number	3,773 (3,418 to 4,188)	6,177 (5,612 to 6,822)	144 (110 to 179)	240 (185 to 296)	20 (16 to 25)	35 (28 to 43)	1,701 (1,498 to 1,921)	2,826 (2,498 to 3,186)	444 (380 to 523)	691 (592 to 812)	1,463 (1,326 to 1,625)	2,384 (2,164 to 2,634)
**Central Asia**	50 plus	Prevalence Rate	35.7 (32.4 to 39.6)	37.1 (33.7 to 41.0)	1.4 (1.0 to 1.7)	1.4 (1.1 to 1.8)	0.2 (0.2 to 0.2)	0.2 (0.2 to 0.3)	16.1 (14.2 to 18.2)	17.0 (15.0 to 19.1)	4.2 (3.6 to 5.0)	4.2 (3.6 to 4.9)	13.9 (12.5 to 15.4)	14.3 (13.0 to 15.8)
**Central Europe**	<1 year	Deaths Number	3,654 (2,785 to 4,174)	647 (566 to 746)										
**Central Europe**	<1 year	Prevalence Number	17,309 (14,797 to 20,063)	11,060 (9,861 to 12,323)	10,547 (8,437 to 12,833)	6,823 (5,869 to 7,808)	376 (308 to 458)	219 (181 to 264)	1,931 (1,604 to 2,328)	1,186 (1,002 to 1,413)	1,701 (1,385 to 2,086)	1,073 (884 to 1,295)	2,753 (2,352 to 3,195)	1,760 (1,566 to 1,960)
**Central Europe**	<1 year	Prevalence Rate	1,007.2 (861.0 to 1,167.5)	1,014.6 (904.7 to 1,130.5)	613.7 (491.0 to 746.8)	625.9 (538.4 to 716.3)	21.9 (17.9 to 26.6)	20.1 (16.6 to 24.2)	112.4 (93.3 to 135.5)	108.8 (91.9 to 129.7)	99.0 (80.6 to 121.4)	98.4 (81.1 to 118.8)	160.2 (136.9 to 185.9)	161.4 (143.7 to 179.8)
**Central Europe**	1 to 4	Deaths Number	475 (350 to 570)	79 (62 to 93)										
**Central Europe**	1 to 4	Prevalence Number	48,668 (41,672 to 55,808)	31,074 (27,541 to 34,293)	30,279 (24,500 to 36,177)	19,509 (16,758 to 22,345)	1,051 (867 to 1,281)	616 (514 to 740)	4,478 (3,861 to 5,165)	2,756 (2,405 to 3,151)	4,502 (3,751 to 5,418)	2,849 (2,405 to 3,385)	8,358 (7,125 to 9,647)	5,345 (4,736 to 5,921)
**Central Europe**	1 to 4	Prevalence Rate	661.6 (566.5 to 758.6)	681.7 (604.2 to 752.3)	411.6 (333.0 to 491.8)	428.0 (367.7 to 490.2)	14.3 (11.8 to 17.4)	13.5 (11.3 to 16.2)	60.9 (52.5 to 70.2)	60.5 (52.8 to 69.1)	61.2 (51.0 to 73.6)	62.5 (52.8 to 74.3)	113.6 (96.9 to 131.1)	117.3 (103.9 to 129.9)
**Central Europe**	5 to 9	Deaths Number	141 (107 to 165)	32 (25 to 38)										
**Central Europe**	5 to 9	Prevalence Number	35,846 (31,306 to 40,698)	23,601 (21,221 to 26,118)	20,769 (16,988 to 24,232)	13,877 (11,965 to 15,850)	949 (786 to 1,143)	578 (484 to 694)	3,836 (3,333 to 4,380)	2,397 (2,097 to 2,721)	3,475 (2,928 to 4,118)	2,250 (1,918 to 2,637)	6,817 (5,920 to 7,734)	4,499 (4,029 to 4,991)

**Central Europe**	5 to 9	Prevalence Rate	365.4 (319.1 to 414.8)	383.5 (344.8 to 424.4)	211.7 (173.1 to 247.0)	225.5 (194.4 to 257.5)	9.7 (8.0 to 11.7)	9.4 (7.9 to 11.3)	39.1 (34.0 to 44.6)	38.9 (34.1 to 44.2)	35.4 (29.8 to 42.0)	36.6 (31.2 to 42.9)	69.5 (60.3 to 78.8)	73.1 (65.5 to 81.1)
**Central Europe**	10 to 14	Deaths Number	125 (98 to 141)	32 (26 to 36)										
**Central Europe**	10 to 14	Prevalence Number	23,206 (20,661 to 25,934)	13,589 (12,318 to 15,008)	11,786 (9,803 to 13,804)	7,039 (6,061 to 8,081)	690 (575 to 821)	384 (321 to 457)	3,434 (3,000 to 3,887)	1,922 (1,682 to 2,172)	2,412 (2,055 to 2,809)	1,372 (1,174 to 1,595)	4,885 (4,336 to 5,463)	2,872 (2,600 to 3,171)
**Central Europe**	10 to 14	Prevalence Rate	221.5 (197.2 to 247.5)	232.3 (210.5 to 256.5)	112.5 (93.6 to 131.8)	120.3 (103.6 to 138.1)	6.6 (5.5 to 7.8)	6.6 (5.5 to 7.8)	32.8 (28.6 to 37.1)	32.8 (28.8 to 37.1)	23.0 (19.6 to 26.8)	23.5 (20.1 to 27.3)	46.6 (41.4 to 52.1)	49.1 (44.4 to 54.2)
**Central Europe**	15 to 19	Deaths Number	107 (87 to 121)	35 (29 to 41)										
**Central Europe**	15 to 19	Prevalence Number	14,351 (12,922 to 15,880)	9,100 (8,274 to 9,987)	6,173 (5,189 to 7,244)	3,994 (3,445 to 4,615)	450 (374 to 532)	279 (233 to 329)	2,778 (2,435 to 3,143)	1,704 (1,491 to 1,922)	1,576 (1,356 to 1,836)	971 (837 to 1,134)	3,374 (3,037 to 3,735)	2,153 (1,952 to 2,366)
**Central Europe**	15 to 19	Prevalence Rate	148.0 (133.3 to 163.8)	154.0 (140.0 to 169.0)	63.7 (53.5 to 74.7)	67.6 (58.3 to 78.1)	4.6 (3.9 to 5.5)	4.7 (3.9 to 5.6)	28.6 (25.1 to 32.4)	28.8 (25.2 to 32.5)	16.3 (14.0 to 18.9)	16.4 (14.2 to 19.2)	34.8 (31.3 to 38.5)	36.4 (33.0 to 40.0)
**Central Europe**	20 to 49	Deaths Number	405 (365 to 517)	234 (193 to 262)										
**Central Europe**	20 to 49	Prevalence Number	34,212 (31,420 to 37,062)	31,807 (29,374 to 34,336)	8,386 (6,956 to 9,891)	7,470 (6,322 to 8,622)	986 (825 to 1,170)	924 (778 to 1,093)	10,707 (9,462 to 12,029)	10,181 (8,957 to 11,468)	4,353 (3,757 to 5,089)	4,042 (3,511 to 4,703)	9,780 (8,968 to 10,614)	9,190 (8,469 to 9,945)
**Central Europe**	20 to 49	Prevalence Rate	65.9 (60.6 to 71.4)	66.1 (61.0 to 71.3)	16.2 (13.4 to 19.1)	15.5 (13.1 to 17.9)	1.9 (1.6 to 2.3)	1.9 (1.6 to 2.3)	20.6 (18.2 to 23.2)	21.1 (18.6 to 23.8)	8.4 (7.2 to 9.8)	8.4 (7.3 to 9.8)	18.9 (17.3 to 20.5)	19.1 (17.6 to 20.7)
**Central Europe**	50 plus	Deaths Number	62 (54 to 74)	53 (45 to 59)										
**Central Europe**	50 plus	Prevalence Number	10,410 (9,525 to 11,426)	14,400 (13,096 to 15,862)	482 (375 to 593)	572 (452 to 701)	138 (113 to 166)	199 (166 to 237)	4,159 (3,675 to 4,683)	5,751 (5,061 to 6,468)	1,508 (1,300 to 1,767)	2,034 (1,751 to 2,376)	4,124 (3,768 to 4,536)	5,844 (5,307 to 6,445)
**Central Europe**	50 plus	Prevalence Rate	33.0 (30.2 to 36.2)	35.7 (32.4 to 39.3)	1.5 (1.2 to 1.9)	1.4 (1.1 to 1.7)	0.4 (0.4 to 0.5)	0.5 (0.4 to 0.6)	13.2 (11.6 to 14.8)	14.2 (12.5 to 16.0)	4.8 (4.1 to 5.6)	5.0 (4.3 to 5.9)	13.1 (11.9 to 14.4)	14.5 (13.1 to 16.0)
**Central Latin America**	<1 year	Deaths Number	8,090 (7,172 to 10,875)	6,366 (4,202 to 7,423)										
**Central Latin America**	<1 year	Prevalence Number	31,520 (27,556 to 35,951)	30,948 (27,313 to 34,947)	9,356 (7,205 to 12,063)	8,757 (6,864 to 10,983)	1,460 (1,171 to 1,807)	1,459 (1,167 to 1,798)	6,181 (5,040 to 7,424)	6,202 (5,105 to 7,420)	9,520 (7,673 to 11,790)	9,619 (7,694 to 11,956)	5,002 (4,369 to 5,702)	4,911 (4,329 to 5,554)
**Central Latin America**	<1 year	Prevalence Rate	645.2 (564.1 to 735.9)	628.2 (554.4 to 709.4)	191.5 (147.5 to 246.9)	177.7 (139.3 to 222.9)	29.9 (24.0 to 37.0)	29.6 (23.7 to 36.5)	126.5 (103.2 to 152.0)	125.9 (103.6 to 150.6)	194.9 (157.1 to 241.3)	195.3 (156.2 to 242.7)	102.4 (89.4 to 116.7)	99.7 (87.9 to 112.7)
**Central Latin America**	1 to 4	Deaths Number	1,303 (1,092 to 1,960)	859 (552 to 1,031)										

**Central Latin America**	1 to 4	Prevalence Number	67,536 (60,001 to 75,894)	69,816 (62,507 to 77,920)	21,234 (16,537 to 26,840)	21,058 (16,744 to 26,089)	2,740 (2,208 to 3,384)	2,922 (2,371 to 3,598)	12,081 (10,320 to 14,008)	12,813 (11,032 to 14,814)	20,018 (16,576 to 24,097)	21,181 (17,558 to 25,601)	11,463 (10,143 to 12,965)	11,843 (10,551 to 13,282)
**Central Latin America**	1 to 4	Prevalence Rate	372.2 (330.7 to 418.3)	362.3 (324.3 to 404.3)	117.0 (91.1 to 147.9)	109.3 (86.9 to 135.4)	15.1 (12.2 to 18.7)	15.2 (12.3 to 18.7)	66.6 (56.9 to 77.2)	66.5 (57.2 to 76.9)	110.3 (91.4 to 132.8)	109.9 (91.1 to 132.8)	63.2 (55.9 to 71.5)	61.5 (54.7 to 68.9)
**Central Latin America**	5 to 9	Deaths Number	392 (331 to 512)	271 (198 to 309)										
**Central Latin America**	5 to 9	Prevalence Number	42,067 (38,053 to 46,637)	45,903 (41,710 to 50,752)	10,519 (8,364 to 13,228)	11,118 (8,889 to 13,788)	1,921 (1,571 to 2,338)	2,158 (1,772 to 2,627)	8,920 (7,685 to 10,223)	9,928 (8,552 to 11,367)	12,792 (10,800 to 15,190)	14,067 (11,920 to 16,661)	7,915 (7,140 to 8,799)	8,633 (7,822 to 9,545)
**Central Latin America**	5 to 9	Prevalence Rate	200.1 (181.0 to 221.9)	197.2 (179.2 to 218.0)	50.0 (39.8 to 62.9)	47.8 (38.2 to 59.2)	9.1 (7.5 to 11.1)	9.3 (7.6 to 11.3)	42.4 (36.6 to 48.6)	42.6 (36.7 to 48.8)	60.9 (51.4 to 72.3)	60.4 (51.2 to 71.6)	37.7 (34.0 to 41.9)	37.1 (33.6 to 41.0)
**Central Latin America**	10 to 14	Deaths Number	256 (219 to 312)	240 (182 to 272)										
**Central Latin America**	10 to 14	Prevalence Number	25,622 (23,475 to 27,945)	29,453 (27,022 to 32,139)	4,666 (3,708 to 5,848)	5,239 (4,225 to 6,499)	1,162 (955 to 1,404)	1,370 (1,128 to 1,660)	6,777 (5,842 to 7,758)	7,935 (6,838 to 9,038)	7,683 (6,536 to 9,010)	8,785 (7,498 to 10,305)	5,335 (4,877 to 5,825)	6,125 (5,614 to 6,692)
**Central Latin America**	10 to 14	Prevalence Rate	127.7 (117.0 to 139.2)	127.1 (116.6 to 138.7)	23.2 (18.5 to 29.1)	22.6 (18.2 to 28.1)	5.8 (4.8 to 7.0)	5.9 (4.9 to 7.2)	33.8 (29.1 to 38.7)	34.2 (29.5 to 39.0)	38.3 (32.6 to 44.9)	37.9 (32.4 to 44.5)	26.6 (24.3 to 29.0)	26.4 (24.2 to 28.9)
**Central Latin America**	15 to 19	Deaths Number	166 (146 to 217)	216 (169 to 255)										
**Central Latin America**	15 to 19	Prevalence Number	16,770 (15,388 to 18,263)	21,182 (19,423 to 23,097)	2,258 (1,770 to 2,828)	2,788 (2,234 to 3,466)	698 (575 to 838)	902 (745 to 1,085)	5,126 (4,431 to 5,827)	6,620 (5,705 to 7,508)	4,760 (4,064 to 5,527)	5,926 (5,089 to 6,906)	3,928 (3,595 to 4,283)	4,946 (4,534 to 5,404)
**Central Latin America**	15 to 19	Prevalence Rate	92.2 (84.6 to 100.5)	92.3 (84.6 to 100.7)	12.4 (9.7 to 15.6)	12.1 (9.7 to 15.1)	3.8 (3.2 to 4.6)	3.9 (3.2 to 4.7)	28.2 (24.4 to 32.1)	28.8 (24.9 to 32.7)	26.2 (22.4 to 30.4)	25.8 (22.2 to 30.1)	21.6 (19.8 to 23.6)	21.6 (19.8 to 23.6)
**Central Latin America**	20 to 49	Deaths Number	495 (428 to 618)	818 (653 to 962)										
**Central Latin America**	20 to 49	Prevalence Number	33,462 (30,582 to 36,487)	57,155 (52,152 to 62,324)	2,326 (1,811 to 2,924)	3,616 (2,852 to 4,531)	963 (794 to 1,161)	1,690 (1,400 to 2,040)	12,399 (10,797 to 14,036)	21,912 (19,135 to 24,840)	8,304 (7,159 to 9,554)	13,681 (11,863 to 15,806)	9,469 (8,649 to 10,341)	16,257 (14,846 to 17,724)
**Central Latin America**	20 to 49	Prevalence Rate	52.8 (48.3 to 57.6)	51.4 (46.9 to 56.1)	3.7 (2.9 to 4.6)	3.3 (2.6 to 4.1)	1.5 (1.3 to 1.8)	1.5 (1.3 to 1.8)	19.6 (17.0 to 22.2)	19.7 (17.2 to 22.4)	13.1 (11.3 to 15.1)	12.3 (10.7 to 14.2)	15.0 (13.7 to 16.3)	14.6 (13.4 to 16.0)
**Central Latin America**	50 plus	Deaths Number	65 (57 to 84)	196 (142 to 221)										
**Central Latin America**	50 plus	Prevalence Number	5,447 (4,965 to 6,040)	15,566 (14,130 to 17,150)	55 (40 to 73)	153 (111 to 199)	56 (45 to 70)	178 (143 to 222)	2,085 (1,827 to 2,359)	6,064 (5,307 to 6,873)	1,155 (998 to 1,344)	3,153 (2,717 to 3,659)	2,097 (1,903 to 2,328)	6,018 (5,466 to 6,650)
**Central Latin America**	50 plus	Prevalence Rate	30.7 (28.0 to 34.0)	32.1 (29.1 to 35.3)	0.3 (0.2 to 0.4)	0.3 (0.2 to 0.4)	0.3 (0.3 to 0.4)	0.4 (0.3 to 0.5)	11.7 (10.3 to 13.3)	12.5 (10.9 to 14.2)	6.5 (5.6 to 7.6)	6.5 (5.6 to 7.5)	11.8 (10.7 to 13.1)	12.4 (11.3 to 13.7)

**Central sub-Saharan Africa**	<1 year	Deaths Number	5,500 (2,670 to 9,248)	7,803 (4,992 to 11,872)										
**Central sub-Saharan Africa**	<1 year	Prevalence Number	40,681 (35,653 to 45,828)	72,506 (62,971 to 82,112)	12,160 (9,724 to 15,333)	21,253 (16,857 to 27,237)	1,303 (980 to 1,699)	2,277 (1,709 to 2,982)	11,978 (9,524 to 14,986)	22,119 (17,415 to 27,167)	8,727 (6,717 to 11,038)	15,248 (11,678 to 19,294)	6,514 (5,706 to 7,342)	11,609 (10,077 to 13,166)
**Central sub-Saharan Africa**	<1 year	Prevalence Rate	1,708.1 (1,497.0 to 1,924.2)	1,760.9 (1,529.4 to 1,994.2)	510.6 (408.3 to 643.8)	516.2 (409.4 to 661.5)	54.7 (41.1 to 71.3)	55.3 (41.5 to 72.4)	502.9 (399.9 to 629.2)	537.2 (422.9 to 659.8)	366.4 (282.0 to 463.5)	370.3 (283.6 to 468.6)	273.5 (239.6 to 308.3)	281.9 (244.7 to 319.8)
**Central sub-Saharan Africa**	1 to 4	Deaths Number	2,276 (402 to 4,614)	2,729 (1,049 to 5,011)										
**Central sub-Saharan Africa**	1 to 4	Prevalence Number	65,023 (57,287 to 73,153)	128,841 (113,551 to 145,411)	24,607 (19,868 to 30,676)	48,388 (38,629 to 60,638)	1,237 (936 to 1,611)	2,467 (1,870 to 3,224)	16,402 (13,648 to 19,896)	32,974 (27,340 to 39,804)	11,432 (9,156 to 14,032)	22,518 (18,066 to 27,564)	11,345 (9,983 to 12,801)	22,494 (19,814 to 25,433)
**Central sub-Saharan Africa**	1 to 4	Prevalence Rate	810.0 (713.6 to 911.3)	823.8 (726.0 to 929.7)	306.5 (247.5 to 382.1)	309.4 (247.0 to 387.7)	15.4 (11.7 to 20.1)	15.8 (12.0 to 20.6)	204.3 (170.0 to 247.8)	210.8 (174.8 to 254.5)	142.4 (114.1 to 174.8)	144.0 (115.5 to 176.2)	141.3 (124.4 to 159.5)	143.8 (126.7 to 162.6)
**Central sub-Saharan Africa**	5 to 9	Deaths Number	122 (55 to 213)	216 (137 to 315)										
**Central sub-Saharan Africa**	5 to 9	Prevalence Number	29,514 (26,428 to 32,881)	66,070 (59,040 to 74,190)	10,606 (8,676 to 12,799)	23,452 (19,163 to 28,517)	464 (346 to 608)	1,066 (787 to 1,383)	8,067 (6,787 to 9,540)	18,289 (15,446 to 21,698)	4,713 (3,800 to 5,684)	10,585 (8,706 to 12,955)	5,664 (5,067 to 6,317)	12,678 (11,305 to 14,264)
**Central sub-Saharan Africa**	5 to 9	Prevalence Rate	360.8 (323.1 to 401.9)	365.8 (326.9 to 410.8)	129.6 (106.1 to 156.5)	129.8 (106.1 to 157.9)	5.7 (4.2 to 7.4)	5.9 (4.4 to 7.7)	98.6 (83.0 to 116.6)	101.3 (85.5 to 120.1)	57.6 (46.5 to 69.5)	58.6 (48.2 to 71.7)	69.2 (61.9 to 77.2)	70.2 (62.6 to 79.0)
**Central sub-Saharan Africa**	10 to 14	Deaths Number	63 (32 to 105)	134 (89 to 191)										
**Central sub-Saharan Africa**	10 to 14	Prevalence Number	13,465 (12,098 to 14,927)	31,000 (27,797 to 34,252)	3,886 (3,185 to 4,653)	8,759 (7,148 to 10,558)	153 (115 to 202)	367 (267 to 476)	4,470 (3,808 to 5,275)	10,442 (8,850 to 12,201)	2,102 (1,734 to 2,519)	4,868 (4,073 to 5,795)	2,854 (2,567 to 3,169)	6,565 (5,867 to 7,266)
**Central sub-Saharan Africa**	10 to 14	Prevalence Rate	199.0 (178.8 to 220.6)	201.9 (181.0 to 223.1)	57.4 (47.1 to 68.8)	57.0 (46.5 to 68.8)	2.3 (1.7 to 3.0)	2.4 (1.7 to 3.1)	66.1 (56.3 to 78.0)	68.0 (57.6 to 79.5)	31.1 (25.6 to 37.2)	31.7 (26.5 to 37.7)	42.2 (37.9 to 46.8)	42.8 (38.2 to 47.3)
**Central sub-Saharan**	15 to 19	Deaths Number	38 (22 to 59)	73 (51 to 101)										

**Africa**														
**Central sub-Saharan Africa**	15 to 19	Prevalence Number	7,376 (6,637 to 8,186)	16,735 (15,032 to 18,535)	1,669 (1,375 to 2,012)	3,687 (2,989 to 4,486)	57 (41 to 76)	135 (99 to 178)	2,796 (2,383 to 3,267)	6,429 (5,502 to 7,430)	1,110 (920 to 1,314)	2,531 (2,125 to 3,024)	1,744 (1,569 to 1,935)	3,953 (3,544 to 4,376)
**Central sub-Saharan Africa**	15 to 19	Prevalence Rate	129.2 (116.2 to 143.3)	131.1 (117.8 to 145.2)	29.2 (24.1 to 35.2)	28.9 (23.4 to 35.1)	1.0 (0.7 to 1.3)	1.1 (0.8 to 1.4)	49.0 (41.7 to 57.2)	50.4 (43.1 to 58.2)	19.4 (16.1 to 23.0)	19.8 (16.6 to 23.7)	30.5 (27.5 to 33.9)	31.0 (27.8 to 34.3)
**Central sub-Saharan Africa**	20 to 49	Deaths Number	133 (93 to 195)	266 (210 to 335)										
**Central sub-Saharan Africa**	20 to 49	Prevalence Number	11,711 (10,542 to 13,024)	27,528 (24,818 to 30,456)	1,532 (1,229 to 1,860)	3,437 (2,697 to 4,173)	40 (28 to 54)	100 (71 to 133)	5,114 (4,413 to 5,897)	12,165 (10,521 to 13,953)	1,698 (1,422 to 1,999)	3,994 (3,371 to 4,731)	3,327 (2,995 to 3,699)	7,832 (7,054 to 8,669)
**Central sub-Saharan Africa**	20 to 49	Prevalence Rate	62.5 (56.3 to 69.5)	62.1 (56.0 to 68.7)	8.2 (6.6 to 9.9)	7.8 (6.1 to 9.4)	0.2 (0.2 to 0.3)	0.2 (0.2 to 0.3)	27.3 (23.6 to 31.5)	27.5 (23.7 to 31.5)	9.1 (7.6 to 10.7)	9.0 (7.6 to 10.7)	17.8 (16.0 to 19.8)	17.7 (15.9 to 19.6)
**Central sub-Saharan Africa**	50 plus	Deaths Number	18 (13 to 26)	36 (26 to 50)										
**Central sub-Saharan Africa**	50 plus	Prevalence Number	1,319 (1,181 to 1,474)	2,839 (2,535 to 3,186)	31 (22 to 40)	66 (46 to 89)	0 (0 to 1)	1 (1 to 2)	564 (485 to 646)	1,226 (1,056 to 1,412)	213 (180 to 251)	460 (387 to 542)	510 (456 to 570)	1,086 (969 to 1,220)
**Central sub-Saharan Africa**	50 plus	Prevalence Rate	25.6 (22.9 to 28.6)	25.5 (22.7 to 28.6)	0.6 (0.4 to 0.8)	0.6 (0.4 to 0.8)	0.0 (0.0 to 0.0)	0.0 (0.0 to 0.0)	11.0 (9.4 to 12.5)	11.0 (9.5 to 12.7)	4.1 (3.5 to 4.9)	4.1 (3.5 to 4.9)	9.9 (8.9 to 11.1)	9.7 (8.7 to 10.9)
**East Asia**	<1 year	Deaths Number	66,508 (56,814 to 81,453)	21,566 (19,744 to 24,101)										
**East Asia**	<1 year	Prevalence Number	255,612 (217,033 to 301,930)	186,660 (160,870 to 215,313)	126,883 (98,570 to 166,217)	92,218 (73,716 to 115,217)	3,823 (2,840 to 4,987)	2,598 (2,025 to 3,287)	18,400 (14,813 to 22,488)	12,356 (10,123 to 14,956)	65,725 (52,618 to 81,915)	49,689 (39,712 to 61,897)	40,780 (34,618 to 48,163)	29,799 (25,665 to 34,406)
**East Asia**	<1 year	Prevalence Rate	979.1 (831.3 to 1,156.5)	973.3 (838.8 to 1,122.7)	486.0 (377.6 to 636.7)	480.9 (384.4 to 600.8)	14.6 (10.9 to 19.1)	13.5 (10.6 to 17.1)	70.5 (56.7 to 86.1)	64.4 (52.8 to 78.0)	251.8 (201.5 to 313.8)	259.1 (207.1 to 322.7)	156.2 (132.6 to 184.5)	155.4 (133.8 to 179.4)
**East Asia**	1 to 4	Deaths Number	13,757 (10,446 to 16,651)	2,877 (2,500 to 3,330)										
**East Asia**	1 to 4	Prevalence Number	627,188 (536,209 to 738,895)	384,505 (332,825 to 440,220)	334,321 (265,860 to 421,456)	203,845 (166,027 to 247,315)	6,152 (4,632 to 8,162)	4,014 (3,131 to 5,126)	39,720 (33,133 to 47,003)	23,290 (19,773 to 27,115)	138,998 (115,276 to 166,608)	87,260 (72,187 to 105,107)	107,997 (91,991 to 127,529)	66,095 (57,086 to 75,707)
**East Asia**	1 to 4	Prevalence Rate	578.4 (494.5 to	591.7 (512.1 to	308.3 (245.2 to	313.7 (255.5 to	5.7 (4.3 to 7.5)	6.2 (4.8 to 7.9)	36.6 (30.6 to	35.8 (30.4 to	128.2 (106.3 to	134.3 (111.1 to	99.6 (84.8 to	101.7 (87.8 to

			681.4)	677.4)	388.7)	380.6)			43.3)	41.7)	153.7)	161.7)	117.6)	116.5)
**East Asia**	5 to 9	Deaths Number	4,482 (3,748 to 5,292)	1,521 (1,402 to 1,661)										
**East Asia**	5 to 9	Prevalence Number	322,733 (282,110 to 370,492)	234,025 (207,119 to 265,270)	160,731 (129,274 to 197,323)	117,552 (96,189 to 141,165)	3,257 (2,436 to 4,250)	2,761 (2,184 to 3,483)	26,023 (22,090 to 30,372)	18,365 (15,770 to 21,397)	71,685 (60,846 to 84,455)	51,211 (43,561 to 60,331)	61,037 (53,323 to 70,454)	44,135 (38,987 to 50,256)
**East Asia**	5 to 9	Prevalence Rate	291.9 (255.1 to 335.1)	308.4 (273.0 to 349.6)	145.4 (116.9 to 178.4)	154.9 (126.8 to 186.0)	2.9 (2.2 to 3.8)	3.6 (2.9 to 4.6)	23.5 (20.0 to 27.5)	24.2 (20.8 to 28.2)	64.8 (55.0 to 76.4)	67.5 (57.4 to 79.5)	55.2 (48.2 to 63.7)	58.2 (51.4 to 66.2)
**East Asia**	10 to 14	Deaths Number	2,401 (2,136 to 2,775)	1,225 (1,146 to 1,307)										
**East Asia**	10 to 14	Prevalence Number	178,607 (159,531 to 200,979)	138,529 (123,907 to 154,842)	76,633 (62,663 to 92,988)	60,801 (50,649 to 72,787)	1,799 (1,347 to 2,359)	1,796 (1,418 to 2,276)	20,087 (16,945 to 23,363)	15,393 (13,182 to 17,737)	43,060 (36,852 to 50,133)	31,981 (27,299 to 37,234)	37,028 (33,052 to 41,828)	28,558 (25,587 to 32,051)
**East Asia**	10 to 14	Prevalence Rate	165.4 (147.7 to 186.1)	178.6 (159.7 to 199.6)	71.0 (58.0 to 86.1)	78.4 (65.3 to 93.8)	1.7 (1.2 to 2.2)	2.3 (1.8 to 2.9)	18.6 (15.7 to 21.6)	19.8 (17.0 to 22.9)	39.9 (34.1 to 46.4)	41.2 (35.2 to 48.0)	34.3 (30.6 to 38.7)	36.8 (33.0 to 41.3)
**East Asia**	15 to 19	Deaths Number	1,771 (1,572 to 2,083)	712 (646 to 788)										
**East Asia**	15 to 19	Prevalence Number	139,748 (125,543 to 155,572)	95,853 (86,498 to 106,287)	49,353 (40,501 to 59,901)	35,759 (29,714 to 42,769)	1,342 (1,005 to 1,751)	1,293 (1,030 to 1,605)	20,489 (17,328 to 23,689)	13,958 (11,960 to 16,072)	36,438 (31,470 to 42,387)	22,919 (19,825 to 26,722)	32,126 (28,877 to 35,986)	21,923 (19,757 to 24,346)
**East Asia**	15 to 19	Prevalence Rate	105.2 (94.5 to 117.1)	117.5 (106.0 to 130.3)	37.1 (30.5 to 45.1)	43.8 (36.4 to 52.4)	1.0 (0.8 to 1.3)	1.6 (1.3 to 2.0)	15.4 (13.0 to 17.8)	17.1 (14.7 to 19.7)	27.4 (23.7 to 31.9)	28.1 (24.3 to 32.8)	24.2 (21.7 to 27.1)	26.9 (24.2 to 29.8)
**East Asia**	20 to 49	Deaths Number	4,691 (4,192 to 5,554)	3,294 (2,841 to 3,766)										
**East Asia**	20 to 49	Prevalence Number	270,843 (246,887 to 296,216)	338,523 (310,124 to 367,070)	60,031 (49,163 to 72,845)	71,777 (58,789 to 87,062)	1,968 (1,481 to 2,569)	4,021 (3,215 to 5,042)	55,940 (47,741 to 64,694)	77,390 (66,935 to 88,487)	77,789 (67,697 to 89,404)	89,619 (78,003 to 102,727)	75,115 (68,368 to 82,539)	95,716 (87,727 to 104,401)
**East Asia**	20 to 49	Prevalence Rate	47.6 (43.4 to 52.1)	48.3 (44.2 to 52.4)	10.5 (8.6 to 12.8)	10.2 (8.4 to 12.4)	0.3 (0.3 to 0.5)	0.6 (0.5 to 0.7)	9.8 (8.4 to 11.4)	11.0 (9.5 to 12.6)	13.7 (11.9 to 15.7)	12.8 (11.1 to 14.7)	13.2 (12.0 to 14.5)	13.7 (12.5 to 14.9)
**East Asia**	50 plus	Deaths Number	267 (204 to 362)	691 (519 to 849)										
**East Asia**	50 plus	Prevalence Number	40,828 (37,049 to 45,302)	113,286 (103,506 to 125,182)	1,679 (1,296 to 2,120)	4,374 (3,408 to 5,429)	96 (71 to 126)	642 (510 to 812)	9,997 (8,576 to 11,475)	30,969 (26,871 to 35,417)	13,195 (11,487 to 15,172)	32,908 (28,519 to 37,790)	15,861 (14,368 to 17,643)	44,393 (40,502 to 49,104)
**East Asia**	50 plus	Prevalence Rate	20.7 (18.8 to 22.9)	25.4 (23.2 to 28.1)	0.9 (0.7 to 1.1)	1.0 (0.8 to 1.2)	0.0 (0.0 to 0.1)	0.1 (0.1 to 0.2)	5.1 (4.3 to 5.8)	6.9 (6.0 to 7.9)	6.7 (5.8 to 7.7)	7.4 (6.4 to 8.5)	8.0 (7.3 to 8.9)	9.9 (9.1 to 11.0)
**Eastern Europe**	<1 year	Deaths Number	5,016 (4,237 to 5,647)	1,427 (1,222 to 1,827)										

**Eastern Europe**	<1 year	Prevalence Number	33,099 (28,477 to 38,521)	24,766 (21,331 to 28,695)	19,078 (15,274 to 23,597)	14,327 (11,557 to 17,654)	930 (752 to 1,136)	685 (554 to 835)	4,630 (3,820 to 5,612)	3,415 (2,825 to 4,134)	3,186 (2,548 to 3,966)	2,396 (1,924 to 2,986)	5,275 (4,538 to 6,140)	3,943 (3,395 to 4,573)
**Eastern Europe**	<1 year	Prevalence Rate	1,067.5 (918.4 to 1,242.3)	1,053.7 (907.5 to 1,220.8)	615.3 (492.6 to 761.0)	609.5 (491.7 to 751.1)	30.0 (24.2 to 36.6)	29.1 (23.6 to 35.5)	149.3 (123.2 to 181.0)	145.3 (120.2 to 175.9)	102.8 (82.2 to 127.9)	102.0 (81.9 to 127.1)	170.1 (146.3 to 198.0)	167.8 (144.4 to 194.5)
**Eastern Europe**	1 to 4	Deaths Number	837 (666 to 999)	223 (180 to 311)										
**Eastern Europe**	1 to 4	Prevalence Number	97,687 (83,897 to 111,952)	71,380 (61,442 to 82,808)	58,152 (46,986 to 70,389)	42,623 (34,580 to 51,428)	2,518 (2,065 to 3,073)	1,811 (1,485 to 2,221)	11,470 (9,844 to 13,296)	8,260 (7,101 to 9,563)	8,734 (7,192 to 10,650)	6,408 (5,236 to 7,799)	16,811 (14,401 to 19,321)	12,277 (10,546 to 14,209)
**Eastern Europe**	1 to 4	Prevalence Rate	681.8 (585.6 to 781.4)	684.8 (589.5 to 794.5)	405.9 (328.0 to 491.3)	408.9 (331.8 to 493.4)	17.6 (14.4 to 21.4)	17.4 (14.2 to 21.3)	80.1 (68.7 to 92.8)	79.2 (68.1 to 91.8)	61.0 (50.2 to 74.3)	61.5 (50.2 to 74.8)	117.3 (100.5 to 134.9)	117.8 (101.2 to 136.3)
**Eastern Europe**	5 to 9	Deaths Number	319 (260 to 382)	86 (69 to 129)										
**Eastern Europe**	5 to 9	Prevalence Number	65,346 (57,374 to 73,839)	47,286 (41,623 to 53,666)	35,755 (29,306 to 42,271)	26,107 (21,535 to 30,848)	2,028 (1,678 to 2,442)	1,441 (1,196 to 1,740)	9,010 (7,775 to 10,316)	6,332 (5,474 to 7,226)	6,160 (5,160 to 7,365)	4,434 (3,719 to 5,308)	12,392 (10,837 to 14,049)	8,973 (7,873 to 10,185)
**Eastern Europe**	5 to 9	Prevalence Rate	368.6 (323.6 to 416.5)	375.6 (330.6 to 426.2)	201.7 (165.3 to 238.4)	207.3 (171.0 to 245.0)	11.4 (9.5 to 13.8)	11.4 (9.5 to 13.8)	50.8 (43.9 to 58.2)	50.3 (43.5 to 57.4)	34.7 (29.1 to 41.5)	35.2 (29.5 to 42.2)	69.9 (61.1 to 79.2)	71.3 (62.5 to 80.9)
**Eastern Europe**	10 to 14	Deaths Number	192 (161 to 232)	78 (66 to 110)										
**Eastern Europe**	10 to 14	Prevalence Number	35,828 (31,959 to 39,864)	24,178 (21,613 to 26,958)	16,835 (14,073 to 19,787)	11,530 (9,650 to 13,521)	1,228 (1,020 to 1,461)	814 (674 to 967)	6,671 (5,762 to 7,586)	4,365 (3,797 to 4,965)	3,572 (3,011 to 4,214)	2,384 (2,012 to 2,809)	7,522 (6,707 to 8,396)	5,086 (4,532 to 5,670)
**Eastern Europe**	10 to 14	Prevalence Rate	216.8 (193.4 to 241.2)	221.7 (198.2 to 247.2)	101.9 (85.2 to 119.7)	105.7 (88.5 to 124.0)	7.4 (6.2 to 8.8)	7.5 (6.2 to 8.9)	40.4 (34.9 to 45.9)	40.0 (34.8 to 45.5)	21.6 (18.2 to 25.5)	21.9 (18.4 to 25.8)	45.5 (40.6 to 50.8)	46.6 (41.6 to 52.0)
**Eastern Europe**	15 to 19	Deaths Number	160 (131 to 188)	73 (60 to 105)										
**Eastern Europe**	15 to 19	Prevalence Number	22,476 (20,244 to 24,734)	14,334 (12,904 to 15,822)	8,803 (7,365 to 10,327)	5,717 (4,768 to 6,722)	780 (649 to 930)	491 (409 to 584)	5,320 (4,611 to 6,015)	3,301 (2,869 to 3,737)	2,279 (1,923 to 2,674)	1,435 (1,213 to 1,696)	5,294 (4,762 to 5,838)	3,390 (3,050 to 3,744)
**Eastern Europe**	15 to 19	Prevalence Rate	141.6 (127.5 to 155.8)	144.5 (130.1 to 159.5)	55.5 (46.4 to 65.1)	57.6 (48.1 to 67.7)	4.9 (4.1 to 5.9)	4.9 (4.1 to 5.9)	33.5 (29.1 to 37.9)	33.3 (28.9 to 37.7)	14.4 (12.1 to 16.8)	14.5 (12.2 to 17.1)	33.4 (30.0 to 36.8)	34.2 (30.7 to 37.7)
**Eastern Europe**	20 to 49	Deaths Number	659 (505 to 735)	612 (542 to 823)										
**Eastern Europe**	20 to 49	Prevalence Number	57,863 (52,860 to 63,099)	53,111 (48,539 to 58,038)	12,720 (10,465 to 15,082)	11,308 (9,273 to 13,364)	1,684 (1,409 to 2,011)	1,525 (1,277 to 1,828)	20,695 (18,191 to 23,374)	19,254 (16,972 to 21,742)	6,004 (5,109 to 7,089)	5,499 (4,691 to 6,446)	16,759 (15,291 to 18,304)	15,525 (14,172 to 16,978)
**Eastern Europe**	20 to 49	Prevalence Rate	61.2 (55.9 to 66.8)	59.3 (54.2 to 64.7)	13.5 (11.1 to 16.0)	12.6 (10.3 to 14.9)	1.8 (1.5 to 2.1)	1.7 (1.4 to 2.0)	21.9 (19.2 to 24.7)	21.5 (18.9 to 24.3)	6.4 (5.4 to 7.5)	6.1 (5.2 to 7.2)	17.7 (16.2 to 19.4)	17.3 (15.8 to 18.9)

**Eastern Europe**	50 plus	Deaths Number	226 (188 to 263)	252 (198 to 284)										
**Eastern Europe**	50 plus	Prevalence Number	18,224 (16,534 to 20,187)	21,067 (19,100 to 23,379)	726 (567 to 898)	792 (621 to 980)	245 (201 to 295)	275 (225 to 335)	8,077 (7,107 to 9,134)	9,293 (8,185 to 10,488)	2,065 (1,742 to 2,434)	2,352 (1,986 to 2,793)	7,111 (6,444 to 7,894)	8,355 (7,569 to 9,291)
**Eastern Europe**	50 plus	Prevalence Rate	29.4 (26.7 to 32.6)	30.0 (27.2 to 33.3)	1.2 (0.9 to 1.5)	1.1 (0.9 to 1.4)	0.4 (0.3 to 0.5)	0.4 (0.3 to 0.5)	13.0 (11.5 to 14.8)	13.2 (11.6 to 14.9)	3.3 (2.8 to 3.9)	3.3 (2.8 to 4.0)	11.5 (10.4 to 12.8)	11.9 (10.8 to 13.2)
**Eastern sub-Saharan Africa**	<1 year	Deaths Number	17,433 (8,443 to 28,779)	18,850 (13,292 to 27,140)										
**Eastern sub-Saharan Africa**	<1 year	Prevalence Number	157,502 (138,444 to 177,952)	238,267 (210,233 to 268,762)	44,291 (35,050 to 55,691)	69,128 (54,586 to 86,035)	5,217 (3,938 to 6,778)	7,848 (5,835 to 10,196)	50,454 (40,444 to 61,965)	73,256 (58,628 to 90,988)	32,319 (25,100 to 40,553)	49,889 (38,881 to 62,498)	25,221 (22,171 to 28,497)	38,146 (33,661 to 43,007)
**Eastern sub-Saharan Africa**	<1 year	Prevalence Rate	1,883.2 (1,655.3 to 2,127.7)	1,788.0 (1,577.6 to 2,016.8)	529.6 (419.1 to 665.9)	518.8 (409.6 to 645.6)	62.4 (47.1 to 81.0)	58.9 (43.8 to 76.5)	603.3 (483.6 to 740.9)	549.7 (440.0 to 682.8)	386.4 (300.1 to 484.9)	374.4 (291.8 to 469.0)	301.6 (265.1 to 340.7)	286.3 (252.6 to 322.7)
**Eastern sub-Saharan Africa**	1 to 4	Deaths Number	6,988 (1,438 to 14,111)	5,962 (3,450 to 9,358)										
**Eastern sub-Saharan Africa**	1 to 4	Prevalence Number	247,407 (218,969 to 278,548)	424,133 (376,576 to 475,130)	88,594 (70,726 to 109,475)	154,932 (123,372 to 189,172)	4,544 (3,433 to 6,020)	8,174 (6,195 to 10,726)	68,328 (57,283 to 82,190)	112,557 (94,178 to 134,033)	42,774 (34,965 to 52,709)	74,500 (61,027 to 91,666)	43,167 (38,195 to 48,708)	73,970 (65,595 to 83,109)
**Eastern sub-Saharan Africa**	1 to 4	Prevalence Rate	880.1 (779.0 to 990.9)	848.6 (753.4 to 950.6)	315.2 (251.6 to 389.5)	310.0 (246.8 to 378.5)	16.2 (12.2 to 21.4)	16.4 (12.4 to 21.5)	243.1 (203.8 to 292.4)	225.2 (188.4 to 268.2)	152.2 (124.4 to 187.5)	149.1 (122.1 to 183.4)	153.6 (135.9 to 173.3)	148.0 (131.2 to 166.3)
**Eastern sub-Saharan Africa**	5 to 9	Deaths Number	496 (249 to 890)	672 (472 to 920)										
**Eastern sub-Saharan Africa**	5 to 9	Prevalence Number	115,497 (103,567 to 128,403)	216,952 (194,556 to 240,874)	38,626 (31,404 to 46,617)	74,133 (60,025 to 89,423)	1,632 (1,221 to 2,134)	3,456 (2,599 to 4,499)	34,572 (29,477 to 40,524)	62,568 (53,334 to 73,010)	18,526 (15,236 to 22,231)	35,231 (28,969 to 42,621)	22,142 (19,862 to 24,642)	41,565 (37,183 to 46,132)
**Eastern sub-Saharan Africa**	5 to 9	Prevalence Rate	392.6 (352.1 to 436.5)	379.9 (340.7 to 421.8)	131.3 (106.8 to 158.5)	129.8 (105.1 to 156.6)	5.5 (4.2 to 7.3)	6.1 (4.6 to 7.9)	117.5 (100.2 to 137.8)	109.6 (93.4 to 127.8)	63.0 (51.8 to 75.6)	61.7 (50.7 to 74.6)	75.3 (67.5 to 83.8)	72.8 (65.1 to 80.8)
**Eastern sub-Saharan Africa**	10 to 14	Deaths Number	256 (147 to 439)	432 (322 to 570)										
**Eastern sub-**	10 to	Prevalence Number	54,243 (49,009 to	108,507 (98,070 to	14,410 (11,874 to	29,529 (24,177 to	509 (375 to	1,231 (915 to	19,361 (16,571 to	37,586 (32,087 to	8,485 (7,076 to	17,220 (14,440 to	11,478 (10,367 to	22,941 (20,745 to

**Saharan Africa**	14		59,856)	120,357)	17,304)	35,183)	673)	1,609)	22,474)	43,661)	10,011)	20,392)	12,684)	25,444)
**Eastern sub-Saharan Africa**	10 to 14	Prevalence Rate	215.3 (194.5 to 237.6)	209.4 (189.3 to 232.3)	57.2 (47.1 to 68.7)	57.0 (46.7 to 67.9)	2.0 (1.5 to 2.7)	2.4 (1.8 to 3.1)	76.8 (65.8 to 89.2)	72.5 (61.9 to 84.3)	33.7 (28.1 to 39.7)	33.2 (27.9 to 39.4)	45.6 (41.2 to 50.3)	44.3 (40.0 to 49.1)
**Eastern sub-Saharan Africa**	15 to 19	Deaths Number	147 (92 to 240)	250 (194 to 327)										
**Eastern sub-Saharan Africa**	15 to 19	Prevalence Number	28,346 (25,623 to 31,081)	60,155 (54,390 to 66,223)	5,883 (4,804 to 7,064)	12,799 (10,474 to 15,291)	168 (123 to 220)	457 (338 to 590)	11,316 (9,721 to 13,012)	23,496 (20,057 to 27,162)	4,279 (3,600 to 5,060)	9,217 (7,767 to 10,786)	6,700 (6,053 to 7,355)	14,186 (12,814 to 15,632)
**Eastern sub-Saharan Africa**	15 to 19	Prevalence Rate	138.4 (125.1 to 151.7)	135.2 (122.2 to 148.8)	28.7 (23.5 to 34.5)	28.8 (23.5 to 34.4)	0.8 (0.6 to 1.1)	1.0 (0.8 to 1.3)	55.2 (47.4 to 63.5)	52.8 (45.1 to 61.0)	20.9 (17.6 to 24.7)	20.7 (17.5 to 24.2)	32.7 (29.5 to 35.9)	31.9 (28.8 to 35.1)
**Eastern sub-Saharan Africa**	20 to 49	Deaths Number	447 (318 to 693)	754 (613 to 941)										
**Eastern sub-Saharan Africa**	20 to 49	Prevalence Number	40,829 (36,909 to 44,870)	90,463 (81,781 to 99,915)	4,968 (3,985 to 6,037)	11,140 (8,924 to 13,535)	96 (68 to 127)	298 (213 to 391)	18,277 (15,828 to 20,966)	40,072 (34,725 to 45,822)	5,884 (5,011 to 6,878)	13,230 (11,249 to 15,449)	11,603 (10,494 to 12,741)	25,722 (23,239 to 28,408)
**Eastern sub-Saharan Africa**	20 to 49	Prevalence Rate	64.5 (58.3 to 70.9)	63.4 (57.3 to 70.0)	7.9 (6.3 to 9.5)	7.8 (6.3 to 9.5)	0.2 (0.1 to 0.2)	0.2 (0.1 to 0.3)	28.9 (25.0 to 33.1)	28.1 (24.3 to 32.1)	9.3 (7.9 to 10.9)	9.3 (7.9 to 10.8)	18.3 (16.6 to 20.1)	18.0 (16.3 to 19.9)
**Eastern sub-Saharan Africa**	50 plus	Deaths Number	51 (38 to 79)	78 (56 to 112)										
**Eastern sub-Saharan Africa**	50 plus	Prevalence Number	3,858 (3,477 to 4,302)	8,091 (7,268 to 9,008)	86 (62 to 111)	178 (128 to 229)	1 (0 to 1)	2 (2 to 3)	1,638 (1,429 to 1,863)	3,457 (3,010 to 3,957)	634 (538 to 741)	1,315 (1,117 to 1,543)	1,499 (1,350 to 1,673)	3,138 (2,819 to 3,501)
**Eastern sub-Saharan Africa**	50 plus	Prevalence Rate	23.6 (21.3 to 26.4)	24.8 (22.2 to 27.6)	0.5 (0.4 to 0.7)	0.5 (0.4 to 0.7)	0.0 (0.0 to 0.0)	0.0 (0.0 to 0.0)	10.0 (8.8 to 11.4)	10.6 (9.2 to 12.1)	3.9 (3.3 to 4.5)	4.0 (3.4 to 4.7)	9.2 (8.3 to 10.2)	9.6 (8.6 to 10.7)
**High-income Asia Pacific**	<1 year	Deaths Number	2,025 (1,425 to 2,339)	420 (366 to 505)										
**High-income Asia Pacific**	<1 year	Prevalence Number	33,062 (28,426 to 38,240)	24,277 (20,691 to 28,078)	19,583 (15,744 to 23,861)	14,837 (12,023 to 17,920)	864 (717 to 1,029)	596 (494 to 710)	4,374 (3,606 to 5,289)	2,892 (2,383 to 3,505)	2,957 (2,367 to 3,699)	2,069 (1,668 to 2,579)	5,284 (4,541 to 6,119)	3,883 (3,306 to 4,487)

**High-income Asia Pacific**	<1 year	Prevalence Rate	1,700.2 (1,461.8 to 1,966.5)	1,690.3 (1,440.5 to 1,954.9)	1,007.0 (809.6 to 1,227.0)	1,033.0 (837.1 to 1,247.6)	44.4 (36.9 to 52.9)	41.5 (34.4 to 49.4)	224.9 (185.4 to 272.0)	201.4 (165.9 to 244.1)	152.1 (121.7 to 190.2)	144.1 (116.1 to 179.6)	271.7 (233.5 to 314.7)	270.3 (230.2 to 312.4)
**High-income Asia Pacific**	1 to 4	Deaths Number	490 (363 to 564)	86 (73 to 111)										
**High-income Asia Pacific**	1 to 4	Prevalence Number	88,643 (75,971 to 101,361)	66,080 (56,422 to 75,589)	54,754 (44,334 to 65,426)	41,894 (34,008 to 49,911)	2,379 (1,989 to 2,844)	1,676 (1,414 to 1,995)	8,944 (7,790 to 10,275)	5,967 (5,201 to 6,848)	7,126 (5,907 to 8,610)	4,996 (4,127 to 5,983)	15,439 (13,214 to 17,694)	11,548 (9,829 to 13,226)
**High-income Asia Pacific**	1 to 4	Prevalence Rate	1,064.1 (912.0 to 1,216.8)	1,081.2 (923.2 to 1,236.8)	657.3 (532.2 to 785.4)	685.5 (556.4 to 816.7)	28.6 (23.9 to 34.1)	27.4 (23.1 to 32.6)	107.4 (93.5 to 123.3)	97.6 (85.1 to 112.1)	85.5 (70.9 to 103.4)	81.7 (67.5 to 97.9)	185.3 (158.6 to 212.4)	189.0 (160.8 to 216.4)
**High-income Asia Pacific**	5 to 9	Deaths Number	213 (125 to 262)	27 (22 to 38)										
**High-income Asia Pacific**	5 to 9	Prevalence Number	68,552 (60,165 to 77,385)	48,354 (41,985 to 54,551)	39,355 (32,504 to 46,079)	28,665 (23,734 to 33,615)	2,327 (1,984 to 2,753)	1,566 (1,334 to 1,839)	8,124 (7,094 to 9,234)	5,116 (4,459 to 5,804)	5,568 (4,708 to 6,598)	3,694 (3,128 to 4,348)	13,178 (11,547 to 14,906)	9,311 (8,091 to 10,520)
**High-income Asia Pacific**	5 to 9	Prevalence Rate	576.0 (505.5 to 650.2)	595.5 (517.1 to 671.8)	330.7 (273.1 to 387.2)	353.0 (292.3 to 414.0)	19.6 (16.7 to 23.1)	19.3 (16.4 to 22.7)	68.3 (59.6 to 77.6)	63.0 (54.9 to 71.5)	46.8 (39.6 to 55.4)	45.5 (38.5 to 53.5)	110.7 (97.0 to 125.2)	114.7 (99.6 to 129.6)
**High-income Asia Pacific**	10 to 14	Deaths Number	156 (100 to 183)	24 (21 to 34)										
**High-income Asia Pacific**	10 to 14	Prevalence Number	46,072 (41,405 to 51,263)	29,815 (26,546 to 33,225)	23,092 (19,482 to 26,795)	15,512 (13,087 to 18,081)	1,765 (1,506 to 2,065)	1,121 (958 to 1,307)	7,592 (6,651 to 8,591)	4,474 (3,904 to 5,050)	3,801 (3,227 to 4,441)	2,341 (1,990 to 2,749)	9,823 (8,810 to 10,930)	6,366 (5,658 to 7,105)
**High-income Asia Pacific**	10 to 14	Prevalence Rate	352.1 (316.4 to 391.7)	362.5 (322.7 to 403.9)	176.5 (148.9 to 204.7)	188.6 (159.1 to 219.8)	13.5 (11.5 to 15.8)	13.6 (11.6 to 15.9)	58.0 (50.8 to 65.6)	54.4 (47.5 to 61.4)	29.0 (24.7 to 33.9)	28.5 (24.2 to 33.4)	75.1 (67.3 to 83.5)	77.4 (68.8 to 86.4)
**High-income Asia Pacific**	15 to 19	Deaths Number	131 (89 to 149)	24 (21 to 34)										
**High-income Asia Pacific**	15 to 19	Prevalence Number	36,223 (32,722 to 39,879)	23,067 (20,807 to 25,415)	15,297 (13,065 to 17,773)	10,094 (8,527 to 11,788)	1,436 (1,232 to 1,664)	920 (792 to 1,071)	7,817 (6,881 to 8,822)	4,694 (4,111 to 5,284)	3,017 (2,576 to 3,517)	1,841 (1,580 to 2,156)	8,656 (7,818 to 9,520)	5,518 (4,973 to 6,086)
**High-income Asia**	15 to 19	Prevalence Rate	239.1 (216.0 to 263.2)	241.9 (218.2 to 266.5)	101.0 (86.2 to 117.3)	105.8 (89.4 to 123.6)	9.5 (8.1 to 11.0)	9.6 (8.3 to 11.2)	51.6 (45.4 to 58.2)	49.2 (43.1 to 55.4)	19.9 (17.0 to 23.2)	19.3 (16.6 to 22.6)	57.1 (51.6 to 62.8)	57.9 (52.1 to 63.8)

**Pacific**														
**High-income Asia Pacific**	20 to 49	Deaths Number	396 (327 to 453)	178 (158 to 234)										
**High-income Asia Pacific**	20 to 49	Prevalence Number	87,819 (80,685 to 95,079)	78,128 (71,564 to 84,498)	20,781 (17,651 to 24,110)	17,910 (15,177 to 20,892)	3,248 (2,790 to 3,780)	2,928 (2,522 to 3,399)	30,902 (27,376 to 34,801)	28,021 (24,819 to 31,451)	7,751 (6,690 to 8,988)	6,659 (5,732 to 7,745)	25,137 (23,054 to 27,249)	22,610 (20,656 to 24,495)
**High-income Asia Pacific**	20 to 49	Prevalence Rate	112.9 (103.7 to 122.2)	105.8 (96.9 to 114.5)	26.7 (22.7 to 31.0)	24.3 (20.6 to 28.3)	4.2 (3.6 to 4.9)	4.0 (3.4 to 4.6)	39.7 (35.2 to 44.7)	38.0 (33.6 to 42.6)	10.0 (8.6 to 11.6)	9.0 (7.8 to 10.5)	32.3 (29.6 to 35.0)	30.6 (28.0 to 33.2)
**High-income Asia Pacific**	50 plus	Deaths Number	197 (160 to 222)	137 (119 to 191)										
**High-income Asia Pacific**	50 plus	Prevalence Number	27,469 (25,054 to 30,178)	44,955 (40,848 to 49,451)	1,311 (1,087 to 1,553)	1,795 (1,470 to 2,119)	670 (550 to 834)	929 (780 to 1,102)	12,422 (11,026 to 13,854)	20,006 (17,736 to 22,384)	2,591 (2,232 to 2,992)	3,990 (3,438 to 4,636)	10,475 (9,529 to 11,520)	18,236 (16,540 to 20,054)
**High-income Asia Pacific**	50 plus	Prevalence Rate	63.4 (57.9 to 69.7)	61.2 (55.6 to 67.3)	3.0 (2.5 to 3.6)	2.4 (2.0 to 2.9)	1.5 (1.3 to 1.9)	1.3 (1.1 to 1.5)	28.7 (25.5 to 32.0)	27.2 (24.1 to 30.5)	6.0 (5.2 to 6.9)	5.4 (4.7 to 6.3)	24.2 (22.0 to 26.6)	24.8 (22.5 to 27.3)
**High-income North America**	<1 year	Deaths Number	3,799 (3,164 to 4,155)	1,617 (1,419 to 1,961)										
**High-income North America**	<1 year	Prevalence Number	45,011 (39,434 to 51,184)	42,604 (37,560 to 48,273)	24,157 (19,867 to 28,945)	23,291 (19,080 to 27,882)	1,333 (1,119 to 1,584)	1,300 (1,122 to 1,509)	6,941 (5,840 to 8,396)	6,341 (5,536 to 7,428)	5,408 (4,302 to 6,854)	4,881 (3,969 to 6,067)	7,171 (6,277 to 8,160)	6,791 (5,987 to 7,691)
**High-income North America**	<1 year	Prevalence Rate	995.7 (872.4 to 1,132.3)	995.8 (877.9 to 1,128.3)	534.4 (439.5 to 640.3)	544.4 (446.0 to 651.7)	29.5 (24.8 to 35.0)	30.4 (26.2 to 35.3)	153.6 (129.2 to 185.7)	148.2 (129.4 to 173.6)	119.6 (95.2 to 151.6)	114.1 (92.8 to 141.8)	158.6 (138.9 to 180.5)	158.7 (139.9 to 179.8)
**High-income North America**	1 to 4	Deaths Number	574 (429 to 636)	202 (175 to 258)										
**High-income North America**	1 to 4	Prevalence Number	108,047 (94,704 to 121,986)	109,356 (96,394 to 123,221)	60,659 (50,192 to 71,451)	61,551 (51,305 to 72,447)	3,481 (2,949 to 4,111)	3,631 (3,161 to 4,194)	13,731 (12,028 to 15,705)	13,598 (12,253 to 15,141)	11,368 (9,230 to 13,972)	11,484 (9,545 to 14,013)	18,808 (16,499 to 21,283)	19,092 (16,810 to 21,527)
**High-income North America**	1 to 4	Prevalence Rate	639.8 (560.8 to 722.3)	636.2 (560.8 to 716.9)	359.2 (297.2 to 423.1)	358.1 (298.5 to 421.5)	20.6 (17.5 to 24.3)	21.1 (18.4 to 24.4)	81.3 (71.2 to 93.0)	79.1 (71.3 to 88.1)	67.3 (54.7 to 82.7)	66.8 (55.5 to 81.5)	111.4 (97.7 to 126.0)	111.1 (97.8 to 125.2)

**High-income North America**	5 to 9	Deaths Number	172 (132 to 191)	83 (71 to 104)										
**High-income North America**	5 to 9	Prevalence Number	75,860 (67,741 to 85,086)	81,619 (73,095 to 90,746)	39,203 (32,629 to 46,274)	41,648 (35,258 to 48,980)	3,012 (2,545 to 3,530)	3,244 (2,814 to 3,704)	11,408 (10,033 to 12,901)	12,352 (11,034 to 13,736)	7,608 (6,336 to 9,168)	8,605 (7,243 to 10,239)	14,629 (13,039 to 16,435)	15,770 (14,114 to 17,546)
**High-income North America**	5 to 9	Prevalence Rate	370.1 (330.5 to 415.1)	363.1 (325.1 to 403.7)	191.3 (159.2 to 225.8)	185.3 (156.8 to 217.9)	14.7 (12.4 to 17.2)	14.4 (12.5 to 16.5)	55.7 (49.0 to 62.9)	54.9 (49.1 to 61.1)	37.1 (30.9 to 44.7)	38.3 (32.2 to 45.5)	71.4 (63.6 to 80.2)	70.2 (62.8 to 78.0)
**High-income North America**	10 to 14	Deaths Number	155 (127 to 179)	98 (84 to 120)										
**High-income North America**	10 to 14	Prevalence Number	46,564 (42,122 to 51,590)	55,540 (50,679 to 60,959)	20,596 (17,447 to 24,217)	24,184 (20,670 to 28,152)	2,035 (1,730 to 2,382)	2,338 (2,034 to 2,666)	9,462 (8,286 to 10,691)	11,424 (10,174 to 12,715)	4,528 (3,799 to 5,384)	5,715 (4,836 to 6,697)	9,943 (8,972 to 11,042)	11,879 (10,813 to 13,068)
**High-income North America**	10 to 14	Prevalence Rate	239.9 (217.0 to 265.8)	236.0 (215.4 to 259.0)	106.1 (89.9 to 124.8)	102.8 (87.8 to 119.6)	10.5 (8.9 to 12.3)	9.9 (8.6 to 11.3)	48.8 (42.7 to 55.1)	48.5 (43.2 to 54.0)	23.3 (19.6 to 27.7)	24.3 (20.5 to 28.5)	51.2 (46.2 to 56.9)	50.5 (46.0 to 55.5)
**High-income North America**	15 to 19	Deaths Number	179 (150 to 217)	116 (102 to 149)										
**High-income North America**	15 to 19	Prevalence Number	34,401 (31,379 to 37,615)	40,295 (37,007 to 43,866)	12,649 (10,751 to 14,899)	14,663 (12,619 to 17,073)	1,533 (1,295 to 1,790)	1,666 (1,445 to 1,896)	8,799 (7,743 to 9,920)	10,357 (9,238 to 11,519)	3,211 (2,712 to 3,776)	3,983 (3,375 to 4,653)	8,210 (7,464 to 9,009)	9,627 (8,810 to 10,497)
**High-income North America**	15 to 19	Prevalence Rate	171.5 (156.4 to 187.5)	171.0 (157.1 to 186.2)	63.0 (53.6 to 74.3)	62.2 (53.6 to 72.5)	7.6 (6.5 to 8.9)	7.1 (6.1 to 8.0)	43.9 (38.6 to 49.4)	44.0 (39.2 to 48.9)	16.0 (13.5 to 18.8)	16.9 (14.3 to 19.7)	40.9 (37.2 to 44.9)	40.9 (37.4 to 44.5)
**High-income North America**	20 to 49	Deaths Number	1,069 (930 to 1,279)	900 (786 to 1,161)										
**High-income North America**	20 to 49	Prevalence Number	113,125 (103,877 to 122,970)	126,298 (116,964 to 135,852)	23,529 (19,920 to 27,867)	26,540 (22,701 to 30,865)	4,301 (3,642 to 5,003)	4,105 (3,572 to 4,723)	43,040 (38,082 to 48,484)	47,913 (42,889 to 53,128)	9,726 (8,323 to 11,393)	11,253 (9,617 to 13,142)	32,529 (29,855 to 35,464)	36,486 (33,733 to 39,266)
**High-income North America**	20 to 49	Prevalence Rate	88.0 (80.8 to 95.6)	88.7 (82.2 to 95.4)	18.3 (15.5 to 21.7)	18.6 (15.9 to 21.7)	3.3 (2.8 to 3.9)	2.9 (2.5 to 3.3)	33.5 (29.6 to 37.7)	33.7 (30.1 to 37.3)	7.6 (6.5 to 8.9)	7.9 (6.8 to 9.2)	25.3 (23.2 to 27.6)	25.6 (23.7 to 27.6)
**High-income North**	50 plus	Deaths Number	482 (432 to 606)	506 (416 to 664)										

**America**														
**High-income North America**	50 plus	Prevalence Number	33,019 (29,976 to 36,493)	62,688 (57,451 to 68,286)	1,365 (1,118 to 1,632)	2,946 (2,470 to 3,475)	592 (493 to 710)	903 (761 to 1,060)	15,325 (13,584 to 17,186)	28,806 (25,861 to 31,984)	2,552 (2,157 to 3,018)	5,057 (4,321 to 5,931)	13,187 (11,906 to 14,629)	24,975 (22,812 to 27,284)
**High-income North America**	50 plus	Prevalence Rate	49.7 (45.1 to 54.9)	51.8 (47.5 to 56.5)	2.1 (1.7 to 2.5)	2.4 (2.0 to 2.9)	0.9 (0.7 to 1.1)	0.7 (0.6 to 0.9)	23.1 (20.4 to 25.9)	23.8 (21.4 to 26.5)	3.8 (3.2 to 4.5)	4.2 (3.6 to 4.9)	19.8 (17.9 to 22.0)	20.7 (18.9 to 22.6)
**North Africa and Middle East**	<1 year	Deaths Number	48,201 (23,682 to 69,838)	27,007 (21,496 to 34,384)										
**North Africa and Middle East**	<1 year	Prevalence Number	96,573 (84,910 to 110,425)	116,785 (102,812 to 133,387)	46,856 (37,591 to 57,794)	53,627 (43,000 to 66,877)	4,099 (3,269 to 5,071)	5,323 (4,305 to 6,576)	16,151 (13,308 to 19,658)	20,823 (17,161 to 25,306)	14,080 (11,120 to 17,553)	18,403 (14,545 to 23,275)	15,386 (13,505 to 17,591)	18,608 (16,364 to 21,245)
**North Africa and Middle East**	<1 year	Prevalence Rate	903.2 (794.1 to 1,032.7)	915.4 (805.9 to 1,045.6)	438.2 (351.6 to 540.5)	420.4 (337.1 to 524.2)	38.3 (30.6 to 47.4)	41.7 (33.7 to 51.5)	151.1 (124.5 to 183.8)	163.2 (134.5 to 198.4)	131.7 (104.0 to 164.2)	144.3 (114.0 to 182.4)	143.9 (126.3 to 164.5)	145.9 (128.3 to 166.5)
**North Africa and Middle East**	1 to 4	Deaths Number	8,143 (3,955 to 12,335)	4,300 (3,494 to 5,288)										
**North Africa and Middle East**	1 to 4	Prevalence Number	207,754 (181,694 to 235,879)	262,789 (230,511 to 299,462)	109,483 (88,376 to 134,788)	134,114 (107,872 to 163,036)	7,418 (6,024 to 9,096)	10,164 (8,267 to 12,488)	29,735 (25,620 to 34,424)	39,069 (33,517 to 45,364)	25,333 (20,750 to 30,622)	34,192 (28,012 to 41,521)	35,786 (31,209 to 40,802)	45,250 (39,612 to 51,690)
**North Africa and Middle East**	1 to 4	Prevalence Rate	509.4 (445.5 to 578.4)	509.3 (446.7 to 580.4)	268.5 (216.7 to 330.5)	259.9 (209.1 to 316.0)	18.2 (14.8 to 22.3)	19.7 (16.0 to 24.2)	72.9 (62.8 to 84.4)	75.7 (65.0 to 87.9)	62.1 (50.9 to 75.1)	66.3 (54.3 to 80.5)	87.8 (76.5 to 100.1)	87.7 (76.8 to 100.2)
**North Africa and Middle East**	5 to 9	Deaths Number	1,727 (1,198 to 2,228)	1,346 (1,127 to 1,696)										
**North Africa and Middle East**	5 to 9	Prevalence Number	120,555 (107,285 to 134,713)	158,582 (141,403 to 177,734)	56,420 (45,768 to 67,475)	73,071 (59,752 to 87,754)	5,083 (4,161 to 6,180)	7,101 (5,839 to 8,567)	21,458 (18,620 to 24,547)	28,265 (24,469 to 32,274)	14,752 (12,413 to 17,617)	20,091 (16,747 to 24,074)	22,842 (20,332 to 25,585)	30,054 (26,723 to 33,840)
**North Africa and Middle East**	5 to 9	Prevalence Rate	253.8 (225.9 to 283.6)	256.7 (228.9 to 287.7)	118.8 (96.4 to 142.1)	118.3 (96.7 to 142.1)	10.7 (8.8 to 13.0)	11.5 (9.5 to 13.9)	45.2 (39.2 to 51.7)	45.8 (39.6 to 52.2)	31.1 (26.1 to 37.1)	32.5 (27.1 to 39.0)	48.1 (42.8 to 53.9)	48.7 (43.3 to 54.8)
**North Africa and Middle East**	10 to 14	Deaths Number	865 (675 to 1,050)	892 (768 to 1,053)										

**North Africa and Middle East**	10 to 14	Prevalence Number	62,901 (56,824 to 69,327)	86,561 (78,400 to 95,312)	23,675 (19,481 to 28,170)	32,518 (26,998 to 38,939)	2,857 (2,346 to 3,431)	4,100 (3,385 to 4,955)	15,057 (13,075 to 17,138)	20,595 (17,878 to 23,464)	8,214 (6,924 to 9,752)	11,327 (9,504 to 13,406)	13,097 (11,805 to 14,487)	18,022 (16,291 to 19,984)
**North Africa and Middle East**	10 to 14	Prevalence Rate	148.8 (134.4 to 164.0)	153.0 (138.6 to 168.5)	56.0 (46.1 to 66.6)	57.5 (47.7 to 68.8)	6.8 (5.5 to 8.1)	7.2 (6.0 to 8.8)	35.6 (30.9 to 40.5)	36.4 (31.6 to 41.5)	19.4 (16.4 to 23.1)	20.0 (16.8 to 23.7)	31.0 (27.9 to 34.3)	31.9 (28.8 to 35.3)
**North Africa and Middle East**	15 to 19	Deaths Number	321 (261 to 390)	473 (409 to 549)										
**North Africa and Middle East**	15 to 19	Prevalence Number	36,441 (33,120 to 39,885)	54,389 (49,515 to 59,582)	10,723 (8,805 to 12,824)	16,267 (13,415 to 19,396)	1,603 (1,323 to 1,913)	2,475 (2,052 to 2,950)	10,784 (9,351 to 12,234)	15,926 (13,852 to 18,058)	4,853 (4,119 to 5,738)	7,077 (5,944 to 8,384)	8,478 (7,689 to 9,319)	12,644 (11,465 to 13,876)
**North Africa and Middle East**	15 to 19	Prevalence Rate	100.1 (91.0 to 109.6)	103.6 (94.3 to 113.5)	29.5 (24.2 to 35.2)	31.0 (25.5 to 36.9)	4.4 (3.6 to 5.3)	4.7 (3.9 to 5.6)	29.6 (25.7 to 33.6)	30.3 (26.4 to 34.4)	13.3 (11.3 to 15.8)	13.5 (11.3 to 16.0)	23.3 (21.1 to 25.6)	24.1 (21.8 to 26.4)
**North Africa and Middle East**	20 to 49	Deaths Number	663 (537 to 823)	1,363 (1,168 to 1,540)										
**North Africa and Middle East**	20 to 49	Prevalence Number	64,668 (59,062 to 70,480)	141,163 (129,201 to 154,531)	10,242 (8,203 to 12,323)	21,004 (17,073 to 25,371)	2,200 (1,840 to 2,632)	4,916 (4,139 to 5,847)	25,467 (22,387 to 28,738)	57,514 (50,511 to 65,025)	8,544 (7,272 to 10,065)	17,553 (14,922 to 20,705)	18,216 (16,602 to 19,883)	40,176 (36,726 to 44,062)
**North Africa and Middle East**	20 to 49	Prevalence Rate	51.8 (47.4 to 56.5)	51.8 (47.4 to 56.7)	8.2 (6.6 to 9.9)	7.7 (6.3 to 9.3)	1.8 (1.5 to 2.1)	1.8 (1.5 to 2.1)	20.4 (17.9 to 23.0)	21.1 (18.5 to 23.9)	6.9 (5.8 to 8.1)	6.4 (5.5 to 7.6)	14.6 (13.3 to 15.9)	14.8 (13.5 to 16.2)
**North Africa and Middle East**	50 plus	Deaths Number	112 (96 to 134)	329 (268 to 387)										
**North Africa and Middle East**	50 plus	Prevalence Number	9,811 (8,904 to 10,927)	25,651 (23,170 to 28,431)	224 (165 to 285)	582 (427 to 745)	123 (101 to 149)	385 (317 to 464)	4,389 (3,878 to 4,949)	11,671 (10,303 to 13,131)	1,278 (1,082 to 1,506)	3,104 (2,629 to 3,667)	3,796 (3,439 to 4,243)	9,909 (8,955 to 11,015)
**North Africa and Middle East**	50 plus	Prevalence Rate	26.2 (23.8 to 29.2)	28.7 (25.9 to 31.8)	0.6 (0.4 to 0.8)	0.7 (0.5 to 0.8)	0.3 (0.3 to 0.4)	0.4 (0.4 to 0.5)	11.7 (10.4 to 13.2)	13.1 (11.5 to 14.7)	3.4 (2.9 to 4.0)	3.5 (2.9 to 4.1)	10.1 (9.2 to 11.3)	11.1 (10.0 to 12.3)
**Oceania**	<1 year	Deaths Number	516 (291 to 735)	861 (479 to 1,247)										
**Oceania**	<1 year	Prevalence Number	2,973 (2,595 to 3,426)	5,786 (5,063 to 6,610)	1,292 (1,021 to 1,613)	2,394 (1,916 to 2,962)	64 (50 to 83)	139 (107 to 178)	326 (265 to 398)	716 (579 to 881)	816 (649 to 1,014)	1,612 (1,285 to 2,011)	475 (414 to 547)	925 (808 to 1,058)

**Oceania**	<1 year	Prevalence Rate	1,407.4 (1,228.5 to 1,622.1)	1,521.0 (1,331.0 to 1,737.7)	611.5 (483.4 to 763.6)	629.3 (503.7 to 778.7)	30.5 (23.5 to 39.3)	36.6 (28.2 to 46.8)	154.2 (125.4 to 188.5)	188.2 (152.3 to 231.6)	386.4 (307.0 to 479.8)	423.8 (337.7 to 528.6)	224.8 (196.0 to 258.9)	243.1 (212.5 to 278.1)
**Oceania**	1 to 4	Deaths Number	136 (46 to 224)	199 (84 to 315)										
**Oceania**	1 to 4	Prevalence Number	6,179 (5,407 to 7,043)	11,867 (10,394 to 13,503)	2,915 (2,336 to 3,573)	5,384 (4,373 to 6,501)	101 (78 to 131)	222 (171 to 287)	611 (519 to 723)	1,304 (1,084 to 1,540)	1,477 (1,215 to 1,798)	2,890 (2,363 to 3,518)	1,075 (941 to 1,225)	2,067 (1,810 to 2,358)
**Oceania**	1 to 4	Prevalence Rate	799.8 (699.8 to 911.6)	848.6 (743.3 to 965.6)	377.3 (302.4 to 462.4)	385.0 (312.7 to 464.9)	13.1 (10.1 to 17.0)	15.9 (12.2 to 20.5)	79.0 (67.2 to 93.5)	93.2 (77.6 to 110.2)	191.1 (157.2 to 232.8)	206.6 (169.0 to 251.6)	139.2 (121.8 to 158.6)	147.8 (129.4 to 168.7)
**Oceania**	5 to 9	Deaths Number	31 (22 to 41)	58 (42 to 74)										
**Oceania**	5 to 9	Prevalence Number	3,479 (3,099 to 3,901)	6,284 (5,598 to 7,048)	1,479 (1,209 to 1,762)	2,568 (2,125 to 3,068)	55 (42 to 69)	116 (89 to 148)	438 (376 to 510)	867 (736 to 1,015)	842 (696 to 1,006)	1,532 (1,262 to 1,844)	665 (591 to 746)	1,201 (1,069 to 1,350)
**Oceania**	5 to 9	Prevalence Rate	400.3 (356.6 to 448.7)	419.6 (373.8 to 470.6)	170.2 (139.1 to 202.7)	171.5 (141.9 to 204.9)	6.3 (4.9 to 8.0)	7.7 (5.9 to 9.9)	50.4 (43.2 to 58.7)	57.9 (49.1 to 67.8)	96.9 (80.1 to 115.8)	102.3 (84.2 to 123.1)	76.5 (68.0 to 85.9)	80.2 (71.4 to 90.2)
**Oceania**	10 to 14	Deaths Number	22 (17 to 28)	39 (31 to 48)										
**Oceania**	10 to 14	Prevalence Number	1,781 (1,604 to 1,976)	3,166 (2,865 to 3,532)	645 (532 to 764)	1,109 (930 to 1,330)	24 (18 to 30)	51 (39 to 65)	296 (255 to 342)	568 (482 to 662)	440 (370 to 520)	772 (642 to 920)	375 (338 to 417)	666 (601 to 745)
**Oceania**	10 to 14	Prevalence Rate	227.7 (205.1 to 252.7)	238.7 (216.1 to 266.3)	82.5 (68.0 to 97.7)	83.6 (70.1 to 100.3)	3.1 (2.3 to 3.9)	3.8 (2.9 to 4.9)	37.8 (32.6 to 43.7)	42.9 (36.4 to 50.0)	56.3 (47.3 to 66.5)	58.2 (48.4 to 69.4)	48.0 (43.2 to 53.3)	50.3 (45.3 to 56.2)
**Oceania**	15 to 19	Deaths Number	7 (5 to 10)	13 (9 to 17)										
**Oceania**	15 to 19	Prevalence Number	994 (902 to 1,096)	1,927 (1,747 to 2,131)	307 (255 to 367)	580 (479 to 696)	11 (8 to 13)	25 (19 to 31)	200 (174 to 230)	416 (356 to 483)	240 (204 to 281)	451 (381 to 535)	236 (214 to 260)	455 (412 to 504)
**Oceania**	15 to 19	Prevalence Rate	145.3 (131.8 to 160.1)	152.7 (138.4 to 168.9)	44.9 (37.3 to 53.6)	46.0 (38.0 to 55.1)	1.5 (1.2 to 2.0)	1.9 (1.5 to 2.5)	29.3 (25.4 to 33.6)	33.0 (28.2 to 38.3)	35.1 (29.8 to 41.1)	35.8 (30.2 to 42.4)	34.4 (31.3 to 38.0)	36.1 (32.6 to 39.9)
**Oceania**	20 to 49	Deaths Number	56 (44 to 71)	122 (96 to 154)										
**Oceania**	20 to 49	Prevalence Number	1,590 (1,455 to 1,731)	3,404 (3,104 to 3,712)	334 (275 to 398)	700 (576 to 838)	10 (8 to 13)	25 (19 to 32)	426 (373 to 484)	969 (839 to 1,108)	369 (316 to 429)	742 (625 to 866)	451 (412 to 492)	969 (882 to 1,057)
**Oceania**	20 to 49	Prevalence Rate	64.1 (58.7 to 69.8)	64.8 (59.1 to 70.6)	13.5 (11.1 to 16.1)	13.3 (11.0 to 15.9)	0.4 (0.3 to 0.5)	0.5 (0.4 to 0.6)	17.2 (15.0 to 19.5)	18.4 (16.0 to 21.1)	14.9 (12.7 to 17.3)	14.1 (11.9 to 16.5)	18.2 (16.6 to 19.8)	18.4 (16.8 to 20.1)
**Oceania**	50 plus	Deaths Number	7 (6 to 9)	16 (13 to 19)										
**Oceania**	50 plus	Prevalence Number	158 (143 to 174)	375 (342 to 415)	8 (6 to 10)	21 (15 to 25)	0 (0 to 0)	1 (1 to 1)	51 (44 to 58)	127 (110 to 144)	38 (32 to 44)	84 (72 to 98)	61 (55 to 67)	143 (130 to 158)

**Oceania**	50 plus	Prevalence Rate	24.4 (22.2 to 26.9)	25.7 (23.5 to 28.5)	1.3 (1.0 to 1.6)	1.4 (1.1 to 1.7)	0.0 (0.0 to 0.1)	0.1 (0.0 to 0.1)	7.9 (6.9 to 9.0)	8.7 (7.6 to 9.9)	5.9 (5.0 to 6.8)	5.8 (4.9 to 6.7)	9.4 (8.5 to 10.3)	9.8 (8.9 to 10.9)
**South Asia**	<1 year	Deaths Number	51,903 (25,680 to 74,913)	46,103 (31,794 to 58,795)										
**South Asia**	<1 year	Prevalence Number	435,599 (386,116 to 490,398)	418,734 (372,088 to 471,009)	147,925 (117,965 to 185,690)	142,517 (112,915 to 179,240)	32,624 (25,159 to 41,769)	30,112 (23,388 to 37,931)	114,667 (93,077 to 141,557)	104,520 (85,301 to 127,370)	70,668 (56,124 to 88,414)	74,558 (58,786 to 93,125)	69,716 (61,777 to 78,517)	67,027 (59,578 to 75,422)
**South Asia**	<1 year	Prevalence Rate	1,299.6 (1,152.0 to 1,463.1)	1,257.8 (1,117.7 to 1,414.8)	441.3 (351.9 to 554.0)	428.1 (339.2 to 538.4)	97.3 (75.1 to 124.6)	90.4 (70.2 to 113.9)	342.1 (277.7 to 422.3)	313.9 (256.2 to 382.6)	210.8 (167.4 to 263.8)	224.0 (176.6 to 279.7)	208.0 (184.3 to 234.3)	201.3 (179.0 to 226.5)
**South Asia**	1 to 4	Deaths Number	11,386 (4,513 to 17,584)	6,080 (4,524 to 7,834)										
**South Asia**	1 to 4	Prevalence Number	843,158 (746,730 to 945,271)	909,460 (811,298 to 1,017,385)	339,765 (271,289 to 419,474)	367,338 (293,397 to 454,129)	42,170 (33,090 to 53,831)	46,960 (36,882 to 58,396)	191,339 (161,844 to 226,832)	193,839 (164,957 to 228,162)	124,098 (101,544 to 150,753)	144,172 (118,559 to 175,753)	145,786 (129,044 to 163,925)	157,150 (139,936 to 176,158)
**South Asia**	1 to 4	Prevalence Rate	664.5 (588.5 to 745.0)	644.5 (575.0 to 721.0)	267.8 (213.8 to 330.6)	260.3 (207.9 to 321.8)	33.2 (26.1 to 42.4)	33.3 (26.1 to 41.4)	150.8 (127.5 to 178.8)	137.4 (116.9 to 161.7)	97.8 (80.0 to 118.8)	102.2 (84.0 to 124.6)	114.9 (101.7 to 129.2)	111.4 (99.2 to 124.8)
**South Asia**	5 to 9	Deaths Number	4,117 (2,231 to 6,282)	2,734 (2,038 to 3,429)										
**South Asia**	5 to 9	Prevalence Number	472,785 (426,462 to 523,091)	556,946 (504,383 to 617,065)	171,446 (139,971 to 206,499)	201,708 (165,152 to 243,970)	22,232 (17,618 to 27,817)	29,037 (23,280 to 35,836)	120,129 (102,832 to 139,268)	134,738 (115,329 to 155,254)	69,509 (58,003 to 82,749)	86,013 (71,619 to 102,513)	89,470 (80,333 to 99,150)	105,449 (95,019 to 117,156)
**South Asia**	5 to 9	Prevalence Rate	319.0 (287.7 to 352.9)	310.9 (281.6 to 344.5)	115.7 (94.4 to 139.3)	112.6 (92.2 to 136.2)	15.0 (11.9 to 18.8)	16.2 (13.0 to 20.0)	81.1 (69.4 to 94.0)	75.2 (64.4 to 86.7)	46.9 (39.1 to 55.8)	48.0 (40.0 to 57.2)	60.4 (54.2 to 66.9)	58.9 (53.0 to 65.4)
**South Asia**	10 to 14	Deaths Number	1,945 (1,333 to 2,720)	2,110 (1,706 to 2,502)										
**South Asia**	10 to 14	Prevalence Number	231,241 (209,786 to 255,139)	319,059 (290,358 to 351,039)	66,688 (54,640 to 79,545)	91,454 (75,743 to 110,030)	9,161 (7,275 to 11,393)	15,159 (12,298 to 18,634)	72,708 (62,657 to 83,827)	96,824 (83,106 to 111,140)	34,669 (29,149 to 40,965)	49,276 (41,314 to 58,294)	48,014 (43,518 to 53,088)	66,346 (60,225 to 73,245)
**South Asia**	10 to 14	Prevalence Rate	181.8 (165.0 to 200.6)	179.4 (163.3 to 197.4)	52.4 (43.0 to 62.5)	51.4 (42.6 to 61.9)	7.2 (5.7 to 9.0)	8.5 (6.9 to 10.5)	57.2 (49.3 to 65.9)	54.4 (46.7 to 62.5)	27.3 (22.9 to 32.2)	27.7 (23.2 to 32.8)	37.8 (34.2 to 41.7)	37.3 (33.9 to 41.2)
**South Asia**	15 to 19	Deaths Number	1,145 (848 to 1,591)	1,250 (1,038 to 1,462)										
**South Asia**	15 to 19	Prevalence Number	134,733 (122,682 to 147,678)	211,757 (193,215 to 231,776)	30,312 (24,875 to 36,376)	47,089 (38,486 to 56,866)	4,150 (3,335 to 5,104)	8,465 (6,946 to 10,289)	49,113 (42,378 to 56,386)	75,285 (65,222 to 85,658)	19,917 (17,033 to 23,491)	31,638 (26,757 to 37,208)	31,241 (28,430 to 34,250)	49,281 (44,933 to 53,875)
**South Asia**	15 to 19	Prevalence Rate	119.0 (108.4 to 130.5)	119.0 (108.6 to 130.3)	26.8 (22.0 to 32.1)	26.5 (21.6 to 32.0)	3.7 (2.9 to 4.5)	4.8 (3.9 to 5.8)	43.4 (37.4 to 49.8)	42.3 (36.7 to 48.1)	17.6 (15.0 to 20.8)	17.8 (15.0 to 20.9)	27.6 (25.1 to 30.3)	27.7 (25.3 to 30.3)
**South Asia**	20 to 49	Deaths Number	4,307 (3,478 to 5,850)	7,924 (7,079 to 8,685)										

**South Asia**	20 to 49	Prevalence Number	242,437 (220,491 to 265,963)	449,290 (410,193 to 492,482)	29,518 (23,631 to 35,802)	51,416 (41,235 to 62,295)	4,018 (3,166 to 4,939)	11,063 (9,055 to 13,410)	106,049 (92,885 to 120,226)	196,799 (172,904 to 222,320)	34,939 (30,065 to 40,765)	63,065 (54,022 to 73,462)	67,913 (61,656 to 74,781)	126,947 (115,618 to 139,629)
**South Asia**	20 to 49	Prevalence Rate	56.7 (51.6 to 62.3)	57.7 (52.7 to 63.3)	6.9 (5.5 to 8.4)	6.6 (5.3 to 8.0)	0.9 (0.7 to 1.2)	1.4 (1.2 to 1.7)	24.8 (21.7 to 28.1)	25.3 (22.2 to 28.6)	8.2 (7.0 to 9.5)	8.1 (6.9 to 9.4)	15.9 (14.4 to 17.5)	16.3 (14.9 to 17.9)
**South Asia**	50 plus	Deaths Number	290 (225 to 389)	783 (634 to 925)										
**South Asia**	50 plus	Prevalence Number	32,221 (29,032 to 35,940)	80,184 (72,242 to 89,011)	625 (457 to 813)	1,372 (1,000 to 1,780)	80 (57 to 103)	478 (373 to 594)	14,153 (12,427 to 16,121)	35,780 (31,372 to 40,650)	4,866 (4,142 to 5,718)	11,025 (9,400 to 12,898)	12,497 (11,244 to 13,954)	31,529 (28,352 to 35,051)
**South Asia**	50 plus	Prevalence Rate	25.0 (22.5 to 27.9)	28.2 (25.4 to 31.3)	0.5 (0.4 to 0.6)	0.5 (0.4 to 0.6)	0.1 (0.0 to 0.1)	0.2 (0.1 to 0.2)	11.0 (9.6 to 12.5)	12.6 (11.0 to 14.3)	3.8 (3.2 to 4.4)	3.9 (3.3 to 4.5)	9.7 (8.7 to 10.8)	11.1 (10.0 to 12.3)
**Southeast Asia**	<1 year	Deaths Number	32,756 (18,440 to 43,083)	16,647 (13,066 to 19,682)										
**Southeast Asia**	<1 year	Prevalence Number	158,351 (137,636 to 180,222)	139,263 (121,810 to 159,349)	74,395 (59,449 to 91,368)	64,865 (51,810 to 81,154)	3,358 (2,597 to 4,245)	2,979 (2,321 to 3,732)	15,471 (12,700 to 18,746)	13,646 (11,213 to 16,436)	39,877 (31,932 to 50,007)	35,553 (28,291 to 44,086)	25,250 (21,952 to 28,754)	22,219 (19,429 to 25,436)
**Southeast Asia**	<1 year	Prevalence Rate	1,296.8 (1,127.2 to 1,475.9)	1,280.8 (1,120.3 to 1,465.5)	609.3 (486.9 to 748.3)	596.6 (476.5 to 746.4)	27.5 (21.3 to 34.8)	27.4 (21.3 to 34.3)	126.7 (104.0 to 153.5)	125.5 (103.1 to 151.2)	326.6 (261.5 to 409.5)	327.0 (260.2 to 405.5)	206.8 (179.8 to 235.5)	204.3 (178.7 to 233.9)
**Southeast Asia**	1 to 4	Deaths Number	5,333 (2,928 to 7,959)	2,370 (1,916 to 2,857)										
**Southeast Asia**	1 to 4	Prevalence Number	355,251 (311,007 to 402,664)	332,897 (291,491 to 379,470)	181,936 (147,090 to 219,624)	169,391 (135,530 to 207,063)	5,233 (4,099 to 6,664)	5,215 (4,135 to 6,523)	30,892 (26,457 to 35,997)	28,945 (24,864 to 33,696)	75,588 (62,412 to 91,035)	71,664 (59,318 to 86,433)	61,602 (53,703 to 69,957)	57,682 (50,434 to 65,512)
**Southeast Asia**	1 to 4	Prevalence Rate	749.6 (656.3 to 849.7)	743.8 (651.3 to 847.8)	383.9 (310.4 to 463.4)	378.5 (302.8 to 462.6)	11.0 (8.6 to 14.1)	11.7 (9.2 to 14.6)	65.2 (55.8 to 76.0)	64.7 (55.6 to 75.3)	159.5 (131.7 to 192.1)	160.1 (132.5 to 193.1)	130.0 (113.3 to 147.6)	128.9 (112.7 to 146.4)
**Southeast Asia**	5 to 9	Deaths Number	1,244 (804 to 1,820)	944 (804 to 1,098)										
**Southeast Asia**	5 to 9	Prevalence Number	217,087 (192,652 to 242,312)	216,035 (191,594 to 241,786)	102,854 (84,329 to 122,220)	102,565 (83,979 to 122,011)	3,314 (2,628 to 4,145)	3,702 (2,978 to 4,581)	24,093 (20,757 to 27,640)	23,833 (20,540 to 27,276)	45,404 (38,261 to 53,473)	44,751 (37,286 to 52,767)	41,422 (36,685 to 46,397)	41,185 (36,462 to 46,210)
**Southeast Asia**	5 to 9	Prevalence Rate	374.1 (332.0 to 417.6)	375.4 (332.9 to 420.1)	177.2 (145.3 to 210.6)	178.2 (145.9 to 212.0)	5.7 (4.5 to 7.1)	6.4 (5.2 to 8.0)	41.5 (35.8 to 47.6)	41.4 (35.7 to 47.4)	78.2 (65.9 to 92.1)	77.8 (64.8 to 91.7)	71.4 (63.2 to 80.0)	71.6 (63.4 to 80.3)
**Southeast Asia**	10 to 14	Deaths Number	878 (674 to 1,138)	839 (730 to 939)										
**Southeast Asia**	10 to 14	Prevalence Number	115,825 (104,574 to 128,477)	124,305 (112,155 to 137,530)	46,717 (38,891 to 55,080)	50,666 (42,249 to 59,762)	1,726 (1,374 to 2,168)	2,188 (1,766 to 2,702)	17,550 (15,108 to 20,125)	18,747 (16,137 to 21,360)	25,584 (21,917 to 29,690)	26,721 (22,686 to 31,131)	24,248 (21,785 to 27,059)	25,984 (23,425 to 28,837)
**Southeast Asia**	10 to 14	Prevalence Rate	212.3 (191.7 to 235.5)	216.2 (195.1 to 239.2)	85.6 (71.3 to 101.0)	88.1 (73.5 to 103.9)	3.2 (2.5 to 4.0)	3.8 (3.1 to 4.7)	32.2 (27.7 to 36.9)	32.6 (28.1 to 37.2)	46.9 (40.2 to 54.4)	46.5 (39.5 to 54.1)	44.4 (39.9 to 49.6)	45.2 (40.7 to 50.2)

**Southeast Asia**	15 to 19	Deaths Number	624 (511 to 801)	737 (634 to 832)										
**Southeast Asia**	15 to 19	Prevalence Number	67,182 (61,194 to 73,949)	79,296 (72,067 to 86,937)	22,567 (18,672 to 26,697)	27,090 (22,578 to 32,048)	906 (725 to 1,128)	1,329 (1,074 to 1,632)	12,766 (11,068 to 14,564)	15,111 (13,081 to 17,200)	15,201 (13,080 to 17,574)	17,240 (14,840 to 20,008)	15,742 (14,302 to 17,400)	18,527 (16,790 to 20,374)
**Southeast Asia**	15 to 19	Prevalence Rate	136.7 (124.5 to 150.5)	140.7 (127.9 to 154.3)	45.9 (38.0 to 54.3)	48.1 (40.1 to 56.9)	1.8 (1.5 to 2.3)	2.4 (1.9 to 2.9)	26.0 (22.5 to 29.6)	26.8 (23.2 to 30.5)	30.9 (26.6 to 35.8)	30.6 (26.3 to 35.5)	32.0 (29.1 to 35.4)	32.9 (29.8 to 36.2)
**Southeast Asia**	20 to 49	Deaths Number	1,729 (1,488 to 2,121)	2,168 (1,994 to 2,339)										
**Southeast Asia**	20 to 49	Prevalence Number	117,014 (107,437 to 127,469)	182,877 (168,500 to 198,966)	24,578 (20,221 to 29,366)	36,601 (30,034 to 43,569)	1,113 (886 to 1,381)	2,309 (1,882 to 2,836)	30,786 (26,884 to 34,901)	50,976 (44,748 to 57,549)	27,431 (23,787 to 31,560)	40,785 (35,365 to 46,661)	33,106 (30,385 to 36,089)	52,205 (48,031 to 56,914)
**Southeast Asia**	20 to 49	Prevalence Rate	62.4 (57.3 to 68.0)	61.1 (56.3 to 66.5)	13.1 (10.8 to 15.7)	12.2 (10.0 to 14.6)	0.6 (0.5 to 0.7)	0.8 (0.6 to 0.9)	16.4 (14.3 to 18.6)	17.0 (15.0 to 19.2)	14.6 (12.7 to 16.8)	13.6 (11.8 to 15.6)	17.7 (16.2 to 19.2)	17.4 (16.0 to 19.0)
**Southeast Asia**	50 plus	Deaths Number	116 (98 to 142)	271 (215 to 309)										
**Southeast Asia**	50 plus	Prevalence Number	15,030 (13,695 to 16,567)	38,846 (35,591 to 42,695)	618 (473 to 767)	1,547 (1,184 to 1,915)	42 (32 to 54)	193 (154 to 237)	4,648 (4,086 to 5,292)	12,731 (11,208 to 14,328)	3,887 (3,365 to 4,471)	9,263 (8,043 to 10,652)	5,834 (5,312 to 6,440)	15,112 (13,822 to 16,630)
**Southeast Asia**	50 plus	Prevalence Rate	26.7 (24.3 to 29.5)	30.0 (27.5 to 33.0)	1.1 (0.8 to 1.4)	1.2 (0.9 to 1.5)	0.1 (0.1 to 0.1)	0.1 (0.1 to 0.2)	8.3 (7.3 to 9.4)	9.8 (8.7 to 11.1)	6.9 (6.0 to 7.9)	7.2 (6.2 to 8.2)	10.4 (9.4 to 11.4)	11.7 (10.7 to 12.8)
**Southern Latin America**	<1 year	Deaths Number	1,629 (1,340 to 1,893)	881 (649 to 1,092)										
**Southern Latin America**	<1 year	Prevalence Number	12,534 (11,046 to 14,163)	12,001 (10,538 to 13,679)	4,824 (3,826 to 6,001)	4,905 (3,875 to 6,150)	253 (201 to 316)	242 (193 to 304)	3,443 (2,800 to 4,244)	2,535 (2,054 to 3,086)	2,007 (1,564 to 2,535)	2,399 (1,869 to 2,988)	2,007 (1,766 to 2,269)	1,920 (1,685 to 2,190)
**Southern Latin America**	<1 year	Prevalence Rate	1,226.0 (1,080.5 to 1,385.3)	1,165.5 (1,023.4 to 1,328.5)	471.9 (374.3 to 587.0)	476.4 (376.3 to 597.3)	24.7 (19.6 to 31.0)	23.5 (18.7 to 29.6)	336.7 (273.9 to 415.1)	246.2 (199.4 to 299.7)	196.3 (153.0 to 248.0)	233.0 (181.5 to 290.2)	196.3 (172.8 to 222.0)	186.4 (163.7 to 212.7)
**Southern Latin America**	1 to 4	Deaths Number	202 (155 to 253)	100 (71 to 129)										
**Southern Latin America**	1 to 4	Prevalence Number	27,295 (24,117 to 30,844)	26,303 (23,139 to 29,855)	12,190 (9,757 to 14,866)	12,291 (9,830 to 14,875)	564 (451 to 698)	542 (434 to 673)	5,868 (5,049 to 6,776)	4,266 (3,682 to 4,943)	3,924 (3,179 to 4,824)	4,612 (3,697 to 5,755)	4,749 (4,191 to 5,378)	4,591 (4,031 to 5,219)
**Southern Latin America**	1 to 4	Prevalence Rate	661.4 (584.4 to 747.4)	642.8 (565.4 to 729.6)	295.4 (236.4 to 360.2)	300.4 (240.2 to 363.5)	13.7 (10.9 to 16.9)	13.3 (10.6 to 16.4)	142.2 (122.4 to 164.2)	104.3 (90.0 to 120.8)	95.1 (77.0 to 116.9)	112.7 (90.3 to 140.6)	115.1 (101.6 to 130.3)	112.2 (98.5 to 127.5)
**Southern Latin America**	5 to 9	Deaths Number	55 (43 to 66)	31 (22 to 40)										
**Southern Latin America**	5 to 9	Prevalence Number	17,839 (15,923 to 19,920)	17,139 (15,279 to 19,164)	7,256 (5,936 to 8,705)	7,412 (6,085 to 8,833)	412 (327 to 506)	409 (329 to 503)	4,368 (3,769 to 4,967)	3,210 (2,771 to 3,668)	2,384 (1,940 to 2,901)	2,813 (2,310 to 3,451)	3,419 (3,049 to 3,825)	3,294 (2,936 to 3,683)

**Southern Latin America**	5 to 9	Prevalence Rate	355.9 (317.7 to 397.4)	344.5 (307.1 to 385.2)	144.7 (118.4 to 173.7)	149.0 (122.3 to 177.5)	8.2 (6.5 to 10.1)	8.2 (6.6 to 10.1)	87.1 (75.2 to 99.1)	64.5 (55.7 to 73.7)	47.6 (38.7 to 57.9)	56.5 (46.4 to 69.4)	68.2 (60.8 to 76.3)	66.2 (59.0 to 74.0)
**Southern Latin America**	10 to 14	Deaths Number	43 (33 to 51)	31 (22 to 39)										
**Southern Latin America**	10 to 14	Prevalence Number	11,066 (10,012 to 12,244)	10,711 (9,707 to 11,829)	3,663 (3,036 to 4,434)	3,881 (3,210 to 4,618)	238 (193 to 291)	250 (203 to 305)	3,489 (3,021 to 3,970)	2,676 (2,319 to 3,066)	1,334 (1,119 to 1,575)	1,630 (1,361 to 1,947)	2,342 (2,116 to 2,593)	2,275 (2,061 to 2,513)
**Southern Latin America**	10 to 14	Prevalence Rate	232.0 (209.9 to 256.7)	219.5 (198.9 to 242.4)	76.8 (63.7 to 93.0)	79.5 (65.8 to 94.6)	5.0 (4.0 to 6.1)	5.1 (4.2 to 6.3)	73.1 (63.3 to 83.2)	54.8 (47.5 to 62.8)	28.0 (23.5 to 33.0)	33.4 (27.9 to 39.9)	49.1 (44.4 to 54.4)	46.6 (42.2 to 51.5)
**Southern Latin America**	15 to 19	Deaths Number	31 (25 to 37)	30 (24 to 37)										
**Southern Latin America**	15 to 19	Prevalence Number	7,562 (6,853 to 8,282)	8,057 (7,345 to 8,829)	1,985 (1,629 to 2,403)	2,371 (1,969 to 2,822)	143 (117 to 175)	172 (141 to 208)	2,831 (2,470 to 3,220)	2,477 (2,151 to 2,827)	810 (687 to 962)	1,121 (944 to 1,333)	1,792 (1,623 to 1,964)	1,916 (1,746 to 2,100)
**Southern Latin America**	15 to 19	Prevalence Rate	169.8 (153.9 to 185.9)	156.8 (142.9 to 171.8)	44.6 (36.6 to 53.9)	46.1 (38.3 to 54.9)	3.2 (2.6 to 3.9)	3.3 (2.7 to 4.0)	63.6 (55.4 to 72.3)	48.2 (41.8 to 55.0)	18.2 (15.4 to 21.6)	21.8 (18.4 to 25.9)	40.2 (36.4 to 44.1)	37.3 (34.0 to 40.9)
**Southern Latin America**	20 to 49	Deaths Number	145 (121 to 168)	153 (122 to 180)										
**Southern Latin America**	20 to 49	Prevalence Number	19,094 (17,337 to 20,925)	23,518 (21,559 to 25,811)	2,641 (2,147 to 3,134)	3,697 (3,033 to 4,465)	220 (180 to 269)	340 (282 to 414)	9,095 (7,994 to 10,319)	9,955 (8,714 to 11,249)	1,609 (1,360 to 1,893)	2,698 (2,282 to 3,172)	5,529 (5,016 to 6,049)	6,828 (6,253 to 7,491)
**Southern Latin America**	20 to 49	Prevalence Rate	95.2 (86.5 to 104.4)	83.0 (76.0 to 91.0)	13.2 (10.7 to 15.6)	13.0 (10.7 to 15.8)	1.1 (0.9 to 1.3)	1.2 (1.0 to 1.5)	45.4 (39.9 to 51.5)	35.1 (30.7 to 39.7)	8.0 (6.8 to 9.4)	9.5 (8.1 to 11.2)	27.6 (25.0 to 30.2)	24.1 (22.1 to 26.4)
**Southern Latin America**	50 plus	Deaths Number	24 (20 to 35)	32 (26 to 39)										
**Southern Latin America**	50 plus	Prevalence Number	5,311 (4,774 to 5,940)	7,868 (7,085 to 8,751)	135 (101 to 169)	222 (174 to 278)	15 (12 to 19)	36 (29 to 45)	2,680 (2,362 to 3,030)	3,691 (3,241 to 4,160)	365 (306 to 431)	758 (641 to 890)	2,117 (1,902 to 2,371)	3,160 (2,840 to 3,517)
**Southern Latin America**	50 plus	Prevalence Rate	55.0 (49.5 to 61.6)	48.5 (43.7 to 54.0)	1.4 (1.0 to 1.7)	1.4 (1.1 to 1.7)	0.2 (0.1 to 0.2)	0.2 (0.2 to 0.3)	27.8 (24.5 to 31.4)	22.8 (20.0 to 25.7)	3.8 (3.2 to 4.5)	4.7 (4.0 to 5.5)	21.9 (19.7 to 24.6)	19.5 (17.5 to 21.7)
**Southern sub-Saharan Africa**	<1 year	Deaths Number	931 (757 to 1,160)	720 (598 to 862)										
**Southern sub-Saharan Africa**	<1 year	Prevalence Number	21,179 (18,627 to 23,986)	24,397 (21,473 to 27,641)	7,414 (5,871 to 9,193)	8,690 (6,884 to 10,798)	630 (480 to 812)	713 (548 to 913)	5,421 (4,367 to 6,638)	6,187 (4,983 to 7,575)	4,325 (3,406 to 5,347)	4,904 (3,842 to 6,146)	3,388 (2,981 to 3,837)	3,902 (3,433 to 4,421)
**Southern sub-Saharan Africa**	<1 year	Prevalence Rate	1,426.6 (1,254.7 to 1,615.7)	1,429.2 (1,257.8 to 1,619.2)	499.4 (395.5 to 619.2)	509.1 (403.2 to 632.5)	42.4 (32.3 to 54.7)	41.8 (32.1 to 53.5)	365.2 (294.1 to 447.1)	362.4 (291.9 to 443.7)	291.4 (229.4 to 360.2)	287.3 (225.0 to 360.0)	228.2 (200.8 to 258.5)	228.6 (201.1 to 259.0)

**Southern sub-Saharan Africa**	1 to 4	Deaths Number	288 (200 to 387)	176 (134 to 228)										
**Southern sub-Saharan Africa**	1 to 4	Prevalence Number	43,278 (38,147 to 48,721)	51,437 (45,348 to 57,842)	17,682 (14,159 to 21,569)	21,193 (16,807 to 25,808)	821 (633 to 1,064)	989 (764 to 1,285)	9,532 (8,053 to 11,275)	11,222 (9,488 to 13,346)	7,683 (6,308 to 9,392)	9,044 (7,401 to 11,020)	7,560 (6,664 to 8,515)	8,989 (7,910 to 10,105)
**Southern sub-Saharan Africa**	1 to 4	Prevalence Rate	752.4 (663.1 to 847.0)	752.9 (663.8 to 846.7)	307.4 (246.1 to 375.0)	310.2 (246.0 to 377.8)	14.3 (11.0 to 18.5)	14.5 (11.2 to 18.8)	165.7 (140.0 to 196.0)	164.3 (138.9 to 195.4)	133.6 (109.7 to 163.3)	132.4 (108.3 to 161.3)	131.4 (115.9 to 148.0)	131.6 (115.8 to 147.9)
**Southern sub-Saharan Africa**	5 to 9	Deaths Number	79 (65 to 93)	59 (49 to 71)										
**Southern sub-Saharan Africa**	5 to 9	Prevalence Number	25,062 (22,453 to 27,894)	29,115 (26,052 to 32,552)	9,366 (7,607 to 11,235)	10,807 (8,797 to 12,924)	420 (321 to 538)	514 (393 to 655)	6,213 (5,263 to 7,246)	7,257 (6,171 to 8,488)	4,249 (3,504 to 5,149)	4,948 (4,064 to 5,940)	4,813 (4,302 to 5,367)	5,589 (5,000 to 6,243)
**Southern sub-Saharan Africa**	5 to 9	Prevalence Rate	368.1 (329.8 to 409.7)	366.7 (328.1 to 410.0)	137.6 (111.7 to 165.0)	136.1 (110.8 to 162.8)	6.2 (4.7 to 7.9)	6.5 (5.0 to 8.3)	91.3 (77.3 to 106.4)	91.4 (77.7 to 106.9)	62.4 (51.5 to 75.6)	62.3 (51.2 to 74.8)	70.7 (63.2 to 78.8)	70.4 (63.0 to 78.6)
**Southern sub-Saharan Africa**	10 to 14	Deaths Number	53 (43 to 63)	47 (37 to 59)										
**Southern sub-Saharan Africa**	10 to 14	Prevalence Number	13,400 (12,113 to 14,801)	15,340 (13,825 to 17,003)	4,031 (3,326 to 4,800)	4,522 (3,709 to 5,419)	177 (135 to 225)	220 (170 to 278)	4,083 (3,476 to 4,732)	4,737 (4,054 to 5,496)	2,266 (1,904 to 2,692)	2,615 (2,183 to 3,109)	2,843 (2,575 to 3,133)	3,247 (2,931 to 3,601)
**Southern sub-Saharan Africa**	10 to 14	Prevalence Rate	210.3 (190.1 to 232.3)	209.5 (188.8 to 232.2)	63.3 (52.2 to 75.3)	61.8 (50.7 to 74.0)	2.8 (2.1 to 3.5)	3.0 (2.3 to 3.8)	64.1 (54.6 to 74.3)	64.7 (55.4 to 75.1)	35.6 (29.9 to 42.2)	35.7 (29.8 to 42.5)	44.6 (40.4 to 49.2)	44.3 (40.0 to 49.2)
**Southern sub-Saharan Africa**	15 to 19	Deaths Number	23 (19 to 27)	24 (19 to 29)										
**Southern sub-Saharan Africa**	15 to 19	Prevalence Number	7,930 (7,203 to 8,674)	9,389 (8,498 to 10,305)	1,866 (1,531 to 2,232)	2,148 (1,754 to 2,572)	78 (59 to 99)	102 (80 to 128)	2,791 (2,386 to 3,204)	3,356 (2,898 to 3,872)	1,307 (1,099 to 1,541)	1,560 (1,311 to 1,845)	1,888 (1,716 to 2,064)	2,223 (2,017 to 2,438)
**Southern sub-Saharan Africa**	15 to 19	Prevalence Rate	135.9 (123.5 to 148.7)	136.0 (123.1 to 149.3)	32.0 (26.2 to 38.3)	31.1 (25.4 to 37.3)	1.3 (1.0 to 1.7)	1.5 (1.2 to 1.9)	47.8 (40.9 to 54.9)	48.6 (42.0 to 56.1)	22.4 (18.8 to 26.4)	22.6 (19.0 to 26.7)	32.4 (29.4 to 35.4)	32.2 (29.2 to 35.3)
**Southern sub-Saharan**	20 to 49	Deaths Number	172 (154 to 190)	190 (168 to 211)										

**Africa**														
**Southern sub-Saharan Africa**	20 to 49	Prevalence Number	12,919 (11,765 to 14,194)	21,262 (19,375 to 23,460)	1,675 (1,347 to 2,035)	2,565 (2,052 to 3,101)	69 (53 to 88)	127 (98 to 161)	5,474 (4,743 to 6,230)	9,151 (7,991 to 10,443)	2,008 (1,714 to 2,366)	3,306 (2,814 to 3,889)	3,693 (3,361 to 4,060)	6,113 (5,570 to 6,748)
**Southern sub-Saharan Africa**	20 to 49	Prevalence Rate	63.9 (58.2 to 70.2)	61.5 (56.1 to 67.9)	8.3 (6.7 to 10.1)	7.4 (5.9 to 9.0)	0.3 (0.3 to 0.4)	0.4 (0.3 to 0.5)	27.1 (23.5 to 30.8)	26.5 (23.1 to 30.2)	9.9 (8.5 to 11.7)	9.6 (8.1 to 11.3)	18.3 (16.6 to 20.1)	17.7 (16.1 to 19.5)
**Southern sub-Saharan Africa**	50 plus	Deaths Number	21 (17 to 27)	36 (32 to 42)										
**Southern sub-Saharan Africa**	50 plus	Prevalence Number	1,634 (1,474 to 1,818)	3,374 (3,047 to 3,768)	28 (20 to 36)	57 (40 to 75)	2 (1 to 2)	4 (3 to 6)	704 (617 to 801)	1,460 (1,272 to 1,664)	257 (218 to 301)	530 (452 to 621)	644 (580 to 717)	1,322 (1,192 to 1,476)
**Southern sub-Saharan Africa**	50 plus	Prevalence Rate	28.2 (25.4 to 31.4)	28.9 (26.1 to 32.3)	0.5 (0.3 to 0.6)	0.5 (0.3 to 0.6)	0.0 (0.0 to 0.0)	0.0 (0.0 to 0.0)	12.2 (10.6 to 13.8)	12.5 (10.9 to 14.2)	4.4 (3.8 to 5.2)	4.5 (3.9 to 5.3)	11.1 (10.0 to 12.4)	11.3 (10.2 to 12.6)
**Tropical Latin America**	<1 year	Deaths Number	15,958 (7,454 to 22,209)	4,557 (3,724 to 5,207)										
**Tropical Latin America**	<1 year	Prevalence Number	26,719 (23,338 to 30,573)	21,719 (19,222 to 24,703)	9,521 (7,503 to 11,932)	7,480 (6,033 to 9,319)	1,408 (1,101 to 1,767)	1,115 (888 to 1,385)	4,808 (3,837 to 5,962)	4,098 (3,327 to 5,009)	6,745 (5,214 to 8,738)	5,578 (4,448 to 7,047)	4,237 (3,697 to 4,852)	3,447 (3,051 to 3,919)
**Tropical Latin America**	<1 year	Prevalence Rate	752.2 (657.1 to 860.7)	688.5 (609.3 to 783.1)	268.0 (211.2 to 335.9)	237.1 (191.3 to 295.4)	39.6 (31.0 to 49.7)	35.4 (28.2 to 43.9)	135.4 (108.0 to 167.9)	129.9 (105.5 to 158.8)	189.9 (146.8 to 246.0)	176.8 (141.0 to 223.4)	119.3 (104.1 to 136.6)	109.3 (96.7 to 124.2)
**Tropical Latin America**	1 to 4	Deaths Number	1,854 (809 to 2,692)	302 (245 to 380)										
**Tropical Latin America**	1 to 4	Prevalence Number	62,480 (55,060 to 70,541)	52,166 (46,867 to 58,126)	24,037 (19,090 to 29,986)	19,156 (15,628 to 23,153)	2,485 (1,985 to 3,108)	2,104 (1,701 to 2,601)	9,851 (8,230 to 11,610)	8,694 (7,382 to 10,126)	15,381 (12,430 to 19,136)	13,266 (10,932 to 16,179)	10,726 (9,410 to 12,162)	8,945 (8,022 to 10,010)
**Tropical Latin America**	1 to 4	Prevalence Rate	433.6 (382.1 to 489.5)	403.1 (362.2 to 449.2)	166.8 (132.5 to 208.1)	148.0 (120.8 to 178.9)	17.2 (13.8 to 21.6)	16.3 (13.1 to 20.1)	68.4 (57.1 to 80.6)	67.2 (57.1 to 78.3)	106.7 (86.3 to 132.8)	102.5 (84.5 to 125.0)	74.4 (65.3 to 84.4)	69.1 (62.0 to 77.4)
**Tropical Latin America**	5 to 9	Deaths Number	467 (300 to 632)	166 (141 to 214)										
**Tropical Latin America**	5 to 9	Prevalence Number	43,298 (38,745 to 48,353)	36,622 (33,250 to 40,424)	13,958 (11,278 to 16,859)	11,082 (9,131 to 13,194)	1,850 (1,487 to 2,294)	1,591 (1,303 to 1,940)	8,060 (6,821 to 9,437)	7,116 (6,096 to 8,256)	11,169 (9,285 to 13,533)	9,855 (8,307 to 11,818)	8,262 (7,393 to 9,255)	6,978 (6,330 to 7,703)
**Tropical Latin America**	5 to 9	Prevalence Rate	231.6 (207.3 to 258.7)	224.4 (203.7 to 247.7)	74.7 (60.3 to 90.2)	67.9 (56.0 to 80.8)	9.9 (8.0 to 12.3)	9.7 (8.0 to 11.9)	43.1 (36.5 to 50.5)	43.6 (37.4 to 50.6)	59.8 (49.7 to 72.4)	60.4 (50.9 to 72.4)	44.2 (39.6 to 49.5)	42.8 (38.8 to 47.2)
**Tropical Latin**	10 to	Deaths Number	242 (182 to	134 (116 to										

**America**	14		302)	177)									
**Tropical Latin America**	10 to 14	Prevalence Number	25,832 (23,555 to 28,402)	24,777 (22,711 to 27,092)	6,527 (5,346 to 7,853)	5,899 (4,920 to 7,063)	1,060 (861 to 1,306)	1,033 (853 to 1,254)	6,046 (5,113 to 7,027)	5,927 (5,071 to 6,798)	6,682 (5,610 to 7,943)	6,641 (5,635 to 7,816)	5,518 (5,012 to 6,091)	5,277 (4,826 to 5,790)
**Tropical Latin America**	10 to 14	Prevalence Rate	144.8 (132.1 to 159.2)	146.4 (134.2 to 160.1)	36.6 (30.0 to 44.0)	34.9 (29.1 to 41.7)	5.9 (4.8 to 7.3)	6.1 (5.0 to 7.4)	33.9 (28.7 to 39.4)	35.0 (30.0 to 40.2)	37.5 (31.5 to 44.5)	39.2 (33.3 to 46.2)	30.9 (28.1 to 34.1)	31.2 (28.5 to 34.2)
**Tropical Latin America**	15 to 19	Deaths Number	121 (85 to 172)	97 (85 to 131)										
**Tropical Latin America**	15 to 19	Prevalence Number	16,149 (14,724 to 17,704)	18,975 (17,413 to 20,644)	3,244 (2,687 to 3,924)	3,640 (3,043 to 4,356)	582 (474 to 711)	702 (582 to 850)	4,391 (3,723 to 5,077)	5,209 (4,474 to 5,980)	4,020 (3,381 to 4,781)	4,846 (4,131 to 5,711)	3,912 (3,565 to 4,301)	4,577 (4,186 to 4,985)
**Tropical Latin America**	15 to 19	Prevalence Rate	103.0 (94.0 to 113.0)	107.5 (98.6 to 116.9)	20.7 (17.1 to 25.0)	20.6 (17.2 to 24.7)	3.7 (3.0 to 4.5)	4.0 (3.3 to 4.8)	28.0 (23.8 to 32.4)	29.5 (25.3 to 33.9)	25.6 (21.6 to 30.5)	27.4 (23.4 to 32.3)	25.0 (22.7 to 27.4)	25.9 (23.7 to 28.2)
**Tropical Latin America**	20 to 49	Deaths Number	538 (425 to 644)	539 (475 to 739)										
**Tropical Latin America**	20 to 49	Prevalence Number	35,311 (32,123 to 38,787)	58,449 (53,413 to 63,743)	4,138 (3,359 to 5,012)	6,440 (5,377 to 7,670)	845 (682 to 1,041)	1,428 (1,173 to 1,733)	11,822 (10,060 to 13,723)	19,996 (17,313 to 23,028)	8,261 (7,046 to 9,727)	13,485 (11,666 to 15,622)	10,245 (9,306 to 11,268)	17,100 (15,623 to 18,689)
**Tropical Latin America**	20 to 49	Prevalence Rate	56.2 (51.1 to 61.7)	58.2 (53.2 to 63.5)	6.6 (5.3 to 8.0)	6.4 (5.4 to 7.6)	1.3 (1.1 to 1.7)	1.4 (1.2 to 1.7)	18.8 (16.0 to 21.8)	19.9 (17.3 to 22.9)	13.1 (11.2 to 15.5)	13.4 (11.6 to 15.6)	16.3 (14.8 to 17.9)	17.0 (15.6 to 18.6)
**Tropical Latin America**	50 plus	Deaths Number	116 (85 to 142)	167 (122 to 202)										
**Tropical Latin America**	50 plus	Prevalence Number	5,959 (5,357 to 6,662)	17,448 (15,803 to 19,314)	146 (114 to 181)	476 (378 to 575)	45 (36 to 56)	150 (120 to 185)	2,155 (1,845 to 2,484)	6,223 (5,402 to 7,105)	1,296 (1,104 to 1,530)	3,747 (3,253 to 4,356)	2,316 (2,079 to 2,593)	6,851 (6,198 to 7,599)
**Tropical Latin America**	50 plus	Prevalence Rate	30.2 (27.2 to 33.8)	35.4 (32.1 to 39.2)	0.7 (0.6 to 0.9)	1.0 (0.8 to 1.2)	0.2 (0.2 to 0.3)	0.3 (0.2 to 0.4)	10.9 (9.4 to 12.6)	12.6 (11.0 to 14.4)	6.6 (5.6 to 7.8)	7.6 (6.6 to 8.8)	11.8 (10.6 to 13.2)	13.9 (12.6 to 15.4)
**Western Europe**	<1 year	Deaths Number	4,579 (2,971 to 5,204)	1,261 (963 to 1,433)										
**Western Europe**	<1 year	Prevalence Number	35,632 (32,843 to 38,764)	39,766 (36,868 to 42,780)	17,131 (15,200 to 19,265)	20,720 (18,624 to 22,771)	1,001 (868 to 1,157)	1,012 (851 to 1,201)	5,962 (5,265 to 6,866)	5,957 (5,167 to 6,970)	5,869 (4,892 to 7,111)	5,738 (4,759 to 6,997)	5,670 (5,226 to 6,168)	6,339 (5,877 to 6,823)
**Western Europe**	<1 year	Prevalence Rate	773.9 (713.3 to 841.9)	920.9 (853.8 to 990.7)	372.1 (330.1 to 418.4)	479.8 (431.3 to 527.3)	21.7 (18.9 to 25.1)	23.4 (19.7 to 27.8)	129.5 (114.4 to 149.1)	138.0 (119.7 to 161.4)	127.5 (106.2 to 154.4)	132.9 (110.2 to 162.0)	123.1 (113.5 to 133.9)	146.8 (136.1 to 158.0)
**Western Europe**	1 to 4	Deaths Number	739 (507 to 857)	164 (137 to 200)										
**Western Europe**	1 to 4	Prevalence Number	93,372 (86,472 to 100,630)	105,284 (97,714 to 112,841)	46,979 (41,703 to 52,520)	57,167 (51,218 to 62,797)	2,880 (2,473 to 3,365)	2,910 (2,456 to 3,468)	13,392 (12,059 to 14,955)	13,189 (11,784 to 14,790)	14,036 (11,970 to 16,723)	13,713 (11,586 to 16,628)	16,086 (14,879 to 17,359)	18,305 (16,960 to 19,666)

**Western Europe**	1 to 4	Prevalence Rate	507.1 (469.6 to 546.5)	592.8 (550.2 to 635.3)	255.1 (226.5 to 285.2)	321.9 (288.4 to 353.6)	15.6 (13.4 to 18.3)	16.4 (13.8 to 19.5)	72.7 (65.5 to 81.2)	74.3 (66.3 to 83.3)	76.2 (65.0 to 90.8)	77.2 (65.2 to 93.6)	87.4 (80.8 to 94.3)	103.1 (95.5 to 110.7)
**Western Europe**	5 to 9	Deaths Number	223 (156 to 252)	66 (55 to 78)										
**Western Europe**	5 to 9	Prevalence Number	71,678 (66,533 to 77,401)	80,705 (74,805 to 87,039)	33,166 (29,347 to 37,076)	40,052 (35,865 to 44,659)	2,623 (2,257 to 3,030)	2,724 (2,324 to 3,211)	11,860 (10,545 to 13,272)	12,089 (10,677 to 13,551)	10,303 (8,813 to 12,111)	10,302 (8,814 to 12,148)	13,727 (12,734 to 14,848)	15,537 (14,398 to 16,776)
**Western Europe**	5 to 9	Prevalence Rate	303.3 (281.5 to 327.5)	343.2 (318.1 to 370.1)	140.3 (124.2 to 156.9)	170.3 (152.5 to 189.9)	11.1 (9.6 to 12.8)	11.6 (9.9 to 13.7)	50.2 (44.6 to 56.2)	51.4 (45.4 to 57.6)	43.6 (37.3 to 51.2)	43.8 (37.5 to 51.7)	58.1 (53.9 to 62.8)	66.1 (61.2 to 71.3)
**Western Europe**	10 to 14	Deaths Number	193 (140 to 216)	63 (52 to 74)										
**Western Europe**	10 to 14	Prevalence Number	50,035 (46,488 to 53,844)	52,093 (48,155 to 56,319)	19,776 (17,536 to 22,258)	22,057 (19,627 to 24,892)	1,920 (1,651 to 2,213)	1,898 (1,609 to 2,237)	10,874 (9,639 to 12,136)	10,558 (9,301 to 11,864)	6,823 (5,831 to 7,914)	6,439 (5,547 to 7,473)	10,642 (9,873 to 11,473)	11,140 (10,295 to 12,080)
**Western Europe**	10 to 14	Prevalence Rate	202.8 (188.5 to 218.3)	224.2 (207.2 to 242.3)	80.2 (71.1 to 90.2)	94.9 (84.5 to 107.1)	7.8 (6.7 to 9.0)	8.2 (6.9 to 9.6)	44.1 (39.1 to 49.2)	45.4 (40.0 to 51.0)	27.7 (23.6 to 32.1)	27.7 (23.9 to 32.2)	43.1 (40.0 to 46.5)	47.9 (44.3 to 52.0)
**Western Europe**	15 to 19	Deaths Number	238 (193 to 281)	77 (66 to 99)										
**Western Europe**	15 to 19	Prevalence Number	41,029 (38,060 to 44,037)	38,133 (35,158 to 41,155)	13,437 (11,878 to 15,159)	13,316 (11,811 to 15,132)	1,554 (1,335 to 1,789)	1,398 (1,184 to 1,635)	10,957 (9,717 to 12,194)	9,732 (8,589 to 10,895)	5,317 (4,581 to 6,175)	4,566 (3,929 to 5,281)	9,764 (9,045 to 10,484)	9,122 (8,402 to 9,865)
**Western Europe**	15 to 19	Prevalence Rate	149.3 (138.5 to 160.3)	162.6 (149.9 to 175.5)	48.9 (43.2 to 55.2)	56.8 (50.4 to 64.5)	5.7 (4.9 to 6.5)	6.0 (5.0 to 7.0)	39.9 (35.4 to 44.4)	41.5 (36.6 to 46.5)	19.3 (16.7 to 22.5)	19.5 (16.8 to 22.5)	35.5 (32.9 to 38.2)	38.9 (35.8 to 42.1)
**Western Europe**	20 to 49	Deaths Number	1,092 (977 to 1,462)	557 (482 to 718)										
**Western Europe**	20 to 49	Prevalence Number	139,870 (129,701 to 149,759)	141,267 (130,748 to 151,778)	25,837 (22,511 to 29,338)	25,604 (22,337 to 29,185)	4,333 (3,728 to 5,010)	4,165 (3,529 to 4,894)	53,132 (47,340 to 59,068)	54,831 (48,803 to 61,291)	16,642 (14,439 to 19,270)	15,765 (13,685 to 18,269)	39,925 (36,903 to 42,898)	40,901 (37,808 to 44,011)
**Western Europe**	20 to 49	Prevalence Rate	83.9 (77.8 to 89.9)	83.4 (77.2 to 89.6)	15.5 (13.5 to 17.6)	15.1 (13.2 to 17.2)	2.6 (2.2 to 3.0)	2.5 (2.1 to 2.9)	31.9 (28.4 to 35.4)	32.4 (28.8 to 36.2)	10.0 (8.7 to 11.6)	9.3 (8.1 to 10.8)	24.0 (22.1 to 25.7)	24.1 (22.3 to 26.0)
**Western Europe**	50 plus	Deaths Number	568 (476 to 931)	350 (282 to 469)										
**Western Europe**	50 plus	Prevalence Number	56,381 (51,563 to 61,480)	82,322 (74,996 to 89,990)	2,072 (1,724 to 2,441)	2,789 (2,347 to 3,261)	783 (650 to 929)	1,054 (883 to 1,259)	25,270 (22,562 to 28,149)	37,007 (32,946 to 41,287)	5,917 (5,094 to 6,864)	8,076 (6,977 to 9,395)	22,338 (20,360 to 24,412)	33,396 (30,366 to 36,618)
**Western Europe**	50 plus	Prevalence Rate	50.2 (45.9 to 54.8)	51.9 (47.3 to 56.7)	1.8 (1.5 to 2.2)	1.8 (1.5 to 2.1)	0.7 (0.6 to 0.8)	0.7 (0.6 to 0.8)	22.5 (20.1 to 25.1)	23.3 (20.8 to 26.0)	5.3 (4.5 to 6.1)	5.1 (4.4 to 5.9)	19.9 (18.1 to 21.7)	21.0 (19.1 to 23.1)
**Western sub-Saharan**	<1 year	Deaths Number	13,295 (5,492 to 21,085)	18,536 (11,756 to 25,923)										

**Africa**														
**Western sub-Saharan Africa**	<1 year	Prevalence Number	132,640 (116,414 to 150,744)	244,143 (214,510 to 276,595)	40,610 (32,315 to 50,711)	76,926 (60,797 to 96,459)	4,108 (3,058 to 5,318)	7,425 (5,553 to 9,726)	38,067 (30,162 to 47,485)	68,127 (54,288 to 83,819)	28,628 (22,106 to 35,985)	52,588 (41,116 to 66,318)	21,228 (18,628 to 24,116)	39,077 (34,320 to 44,290)
**Western sub-Saharan Africa**	<1 year	Prevalence Rate	1,641.9 (1,441.1 to 1,866.0)	1,595.4 (1,401.8 to 1,807.5)	502.7 (400.0 to 627.7)	502.7 (397.3 to 630.4)	50.8 (37.9 to 65.8)	48.5 (36.3 to 63.6)	471.2 (373.4 to 587.8)	445.2 (354.8 to 547.7)	354.4 (273.7 to 445.5)	343.7 (268.7 to 433.4)	262.8 (230.6 to 298.5)	255.4 (224.3 to 289.4)
**Western sub-Saharan Africa**	1 to 4	Deaths Number	4,912 (639 to 9,255)	6,077 (1,521 to 10,712)										
**Western sub-Saharan Africa**	1 to 4	Prevalence Number	210,262 (185,421 to 237,290)	430,348 (382,040 to 483,756)	81,757 (66,231 to 100,984)	170,587 (135,700 to 209,695)	3,865 (2,916 to 5,070)	8,041 (6,062 to 10,452)	51,410 (43,082 to 62,194)	101,811 (84,828 to 121,847)	36,482 (29,483 to 44,865)	74,710 (60,185 to 91,046)	36,748 (32,390 to 41,486)	75,200 (66,565 to 84,644)
**Western sub-Saharan Africa**	1 to 4	Prevalence Rate	778.2 (686.2 to 878.2)	762.0 (676.5 to 856.6)	302.6 (245.1 to 373.7)	302.1 (240.3 to 371.3)	14.3 (10.8 to 18.8)	14.2 (10.7 to 18.5)	190.3 (159.4 to 230.2)	180.3 (150.2 to 215.8)	135.0 (109.1 to 166.0)	132.3 (106.6 to 161.2)	136.0 (119.9 to 153.5)	133.2 (117.9 to 149.9)
**Western sub-Saharan Africa**	5 to 9	Deaths Number	415 (186 to 739)	748 (485 to 1,112)										
**Western sub-Saharan Africa**	5 to 9	Prevalence Number	97,743 (87,016 to 108,705)	216,620 (193,781 to 240,861)	36,641 (30,032 to 44,147)	82,669 (67,003 to 99,092)	1,535 (1,146 to 1,995)	3,653 (2,785 to 4,685)	25,556 (21,576 to 29,911)	54,939 (46,776 to 64,209)	15,222 (12,523 to 18,391)	33,755 (27,939 to 40,865)	18,790 (16,715 to 20,927)	41,604 (37,239 to 46,302)
**Western sub-Saharan Africa**	5 to 9	Prevalence Rate	344.4 (306.6 to 383.1)	336.6 (301.1 to 374.3)	129.1 (105.8 to 155.6)	128.5 (104.1 to 154.0)	5.4 (4.0 to 7.0)	5.7 (4.3 to 7.3)	90.1 (76.0 to 105.4)	85.4 (72.7 to 99.8)	53.6 (44.1 to 64.8)	52.5 (43.4 to 63.5)	66.2 (58.9 to 73.7)	64.6 (57.9 to 71.9)
**Western sub-Saharan Africa**	10 to 14	Deaths Number	230 (145 to 409)	519 (366 to 738)										
**Western sub-Saharan Africa**	10 to 14	Prevalence Number	44,750 (40,319 to 49,630)	103,899 (93,758 to 115,312)	13,653 (11,234 to 16,357)	32,141 (26,507 to 38,692)	529 (394 to 693)	1,402 (1,063 to 1,790)	14,236 (12,031 to 16,425)	32,393 (27,635 to 37,653)	6,813 (5,693 to 8,044)	15,912 (13,345 to 18,881)	9,520 (8,556 to 10,582)	22,051 (19,871 to 24,470)
**Western sub-Saharan Africa**	10 to 14	Prevalence Rate	189.8 (171.0 to 210.5)	185.6 (167.5 to 206.0)	57.9 (47.7 to 69.4)	57.4 (47.3 to 69.1)	2.2 (1.7 to 2.9)	2.5 (1.9 to 3.2)	60.4 (51.0 to 69.7)	57.9 (49.4 to 67.3)	28.9 (24.2 to 34.1)	28.4 (23.8 to 33.7)	40.4 (36.3 to 44.9)	39.4 (35.5 to 43.7)
**Western sub-Saharan Africa**	15 to 19	Deaths Number	106 (67 to 173)	241 (168 to 326)										

**Western sub-Saharan Africa**	15 to 19	Prevalence Number	24,368 (21,985 to 26,848)	56,465 (51,284 to 62,252)	5,857 (4,785 to 7,051)	13,646 (11,182 to 16,672)	203 (151 to 266)	570 (431 to 729)	8,893 (7,607 to 10,272)	20,384 (17,343 to 23,480)	3,602 (3,031 to 4,225)	8,449 (7,117 to 9,986)	5,814 (5,245 to 6,415)	13,417 (12,164 to 14,820)
**Western sub-Saharan Africa**	15 to 19	Prevalence Rate	123.8 (111.7 to 136.4)	121.6 (110.4 to 134.0)	29.8 (24.3 to 35.8)	29.4 (24.1 to 35.9)	1.0 (0.8 to 1.4)	1.2 (0.9 to 1.6)	45.2 (38.7 to 52.2)	43.9 (37.3 to 50.5)	18.3 (15.4 to 21.5)	18.2 (15.3 to 21.5)	29.5 (26.7 to 32.6)	28.9 (26.2 to 31.9)
**Western sub-Saharan Africa**	20 to 49	Deaths Number	459 (334 to 743)	1,069 (856 to 1,417)										
**Western sub-Saharan Africa**	20 to 49	Prevalence Number	40,143 (36,355 to 44,397)	93,532 (84,726 to 103,198)	5,378 (4,305 to 6,544)	12,430 (10,062 to 15,295)	160 (116 to 214)	488 (367 to 637)	17,260 (14,998 to 19,773)	39,992 (34,721 to 45,950)	5,873 (4,966 to 6,828)	13,877 (11,796 to 16,211)	11,472 (10,382 to 12,679)	26,744 (24,216 to 29,545)
**Western sub-Saharan Africa**	20 to 49	Prevalence Rate	61.0 (55.2 to 67.4)	60.2 (54.6 to 66.5)	8.2 (6.5 to 9.9)	8.0 (6.5 to 9.8)	0.2 (0.2 to 0.3)	0.3 (0.2 to 0.4)	26.2 (22.8 to 30.0)	25.8 (22.4 to 29.6)	8.9 (7.5 to 10.4)	8.9 (7.6 to 10.4)	17.4 (15.8 to 19.3)	17.2 (15.6 to 19.0)
**Western sub-Saharan Africa**	50 plus	Deaths Number	90 (57 to 134)	181 (120 to 253)										
**Western sub-Saharan Africa**	50 plus	Prevalence Number	5,145 (4,619 to 5,731)	10,760 (9,671 to 11,955)	105 (76 to 137)	228 (168 to 296)	2 (2 to 4)	9 (6 to 13)	2,186 (1,907 to 2,490)	4,605 (3,988 to 5,260)	833 (710 to 978)	1,732 (1,470 to 2,030)	2,019 (1,809 to 2,249)	4,186 (3,753 to 4,655)
**Western sub-Saharan Africa**	50 plus	Prevalence Rate	27.0 (24.3 to 30.1)	27.8 (25.0 to 30.9)	0.6 (0.4 to 0.7)	0.6 (0.4 to 0.8)	0.0 (0.0 to 0.0)	0.0 (0.0 to 0.0)	11.5 (10.0 to 13.1)	11.9 (10.3 to 13.6)	4.4 (3.7 to 5.1)	4.5 (3.8 to 5.2)	10.6 (9.5 to 11.8)	10.8 (9.7 to 12.0)

Table S13: Alphabetical listing of all data sources used in estimating cause-specific mortality due to congenital birth defects overall for GBD 2017

Citation from Global Health Data Exchange (http://ghdx.healthdata.org/gbd-2017/data-input-sources)
Abu-Rashid N, Al-Jirf S, Bashour H. Causes of death among Syrian children using verbal autopsy. East Mediterr Health J. 1996; 2(3): 440-8
Agency for Health Research and Development (Indonesia). Indonesia Basic Health Research 2007-2008
Agency for Health Research and Development (Indonesia). Indonesia Sample Registration System - Deaths 2015
Albania Vital Registration - Deaths 1987 ICD9 as it appears in World Health Organization (WHO). WHO Mortality Database Version November 2015. Geneva, Switzerland: World Health Organization (WHO), 2015
Albania Vital Registration - Deaths 1988 ICD9 as it appears in World Health Organization (WHO). WHO Mortality Database Version November 2015. Geneva, Switzerland: World Health Organization (WHO), 2015
Albania Vital Registration - Deaths 1989 ICD9 as it appears in World Health Organization (WHO). WHO Mortality Database Version November 2015. Geneva, Switzerland: World Health Organization (WHO), 2015
Albania Vital Registration - Deaths 1992 ICD9 as it appears in World Health Organization (WHO). WHO Mortality Database Version November 2015. Geneva, Switzerland: World Health Organization (WHO), 2015
Albania Vital Registration - Deaths 1993 ICD9 as it appears in World Health Organization (WHO). WHO Mortality Database Version November 2015. Geneva, Switzerland: World Health Organization (WHO), 2015
Albania Vital Registration - Deaths 1994 ICD9 as it appears in World Health Organization (WHO). WHO Mortality Database Version November 2015. Geneva, Switzerland: World Health Organization (WHO), 2015
Albania Vital Registration - Deaths 1995 ICD9 as it appears in World Health Organization (WHO). WHO Mortality Database Version November 2015. Geneva, Switzerland: World Health Organization (WHO), 2015
Albania Vital Registration - Deaths 1996 ICD9 as it appears in World Health Organization (WHO). WHO Mortality Database Version November 2015. Geneva, Switzerland: World Health Organization (WHO), 2015
Albania Vital Registration - Deaths 1997 ICD9 as it appears in World Health Organization (WHO). WHO Mortality Database Version November 2015. Geneva, Switzerland: World Health Organization (WHO), 2015
Albania Vital Registration - Deaths 1998 ICD9 as it appears in World Health Organization (WHO). WHO Mortality Database Version November 2015. Geneva, Switzerland: World Health Organization (WHO), 2015
Albania Vital Registration - Deaths 1999 ICD9 as it appears in World Health Organization (WHO). WHO Mortality Database Version November 2015. Geneva, Switzerland: World Health Organization (WHO), 2015
Albania Vital Registration - Deaths 2000 ICD9 as it appears in World Health Organization (WHO). WHO Mortality Database Version November 2015. Geneva, Switzerland: World Health Organization (WHO), 2015
Albania Vital Registration - Deaths 2001 ICD9 as it appears in World Health Organization (WHO). WHO Mortality Database Version November 2015. Geneva, Switzerland: World Health Organization (WHO), 2015
Albania Vital Registration - Deaths 2002 ICD9 as it appears in World Health Organization (WHO). WHO Mortality Database Version November 2015. Geneva, Switzerland: World Health Organization (WHO), 2015

Albania Vital Registration - Deaths 2003 ICD9 as it appears in World Health Organization (WHO). WHO Mortality Database Version November 2015. Geneva, Switzerland: World Health Organization (WHO), 2015

Albania Vital Registration - Deaths 2004 ICD9 as it appears in World Health Organization (WHO). WHO Mortality Database Version November 2015. Geneva, Switzerland: World Health Organization (WHO), 2015

Albania Vital Registration - Deaths 2005 ICD9 as it appears in World Health Organization (WHO). WHO Mortality Database Version September 2016. Geneva, Switzerland: World Health Organization (WHO), 2016

Albania Vital Registration - Deaths 2006 ICD9 as it appears in World Health Organization (WHO). WHO Mortality Database Version September 2016. Geneva, Switzerland: World Health Organization (WHO), 2016

Albania Vital Registration - Deaths 2007 ICD9 as it appears in World Health Organization (WHO). WHO Mortality Database Version September 2016. Geneva, Switzerland: World Health Organization (WHO), 2016

Albania Vital Registration - Deaths 2008 ICD9 as it appears in World Health Organization (WHO). WHO Mortality Database Version September 2016. Geneva, Switzerland: World Health Organization (WHO), 2016

Albania Vital Registration - Deaths 2009 ICD9 as it appears in World Health Organization (WHO). WHO Mortality Database Version September 2016. Geneva, Switzerland: World Health Organization (WHO), 2016

Albania Vital Registration - Deaths 2010 ICD9 as it appears in World Health Organization (WHO). WHO Mortality Database Version March 2017. Geneva, Switzerland: World Health Organization (WHO), 2017

Anand K, Kant S, Kumar G, Kapoor SK. "Development" is not essential to reduce infant mortality rate in India: experience from the Ballabgarh project. J Epidemiol Community Health. 2000; 54(4): 247-53

Anand K, Patro BK, Paul E, Kapoor SK. Management of Sick Children by Health Workers in Ballabgarh: Lessons for Implementation of IMCI in India. J Trop Pediatr. 2004; 50(1): 41-7

Antigua and Barbuda Vital Registration - Deaths 1983 ICD9 as it appears in World Health Organization (WHO). WHO Mortality Database Version September 2016. Geneva, Switzerland: World Health Organization (WHO), 2016

Antigua and Barbuda Vital Registration - Deaths 1986 ICD9 as it appears in World Health Organization (WHO). WHO Mortality Database Version September 2016. Geneva, Switzerland: World Health Organization (WHO), 2016

Antigua and Barbuda Vital Registration - Deaths 1987 ICD9 as it appears in World Health Organization (WHO). WHO Mortality Database Version September 2016. Geneva, Switzerland: World Health Organization (WHO), 2016

Antigua and Barbuda Vital Registration - Deaths 1988 ICD9 as it appears in World Health Organization (WHO). WHO Mortality Database Version September 2016. Geneva, Switzerland: World Health Organization (WHO), 2016

Antigua and Barbuda Vital Registration - Deaths 1989 ICD9 as it appears in World Health Organization (WHO). WHO Mortality Database Version September 2016. Geneva, Switzerland: World Health Organization (WHO), 2016

Antigua and Barbuda Vital Registration - Deaths 1990 ICD9 as it appears in World Health Organization (WHO). WHO Mortality Database Version September 2016. Geneva, Switzerland: World Health Organization (WHO), 2016

Antigua and Barbuda Vital Registration - Deaths 1991 ICD9 as it appears in World Health Organization (WHO). WHO Mortality Database Version September 2016. Geneva, Switzerland: World Health Organization (WHO), 2016

Antigua and Barbuda Vital Registration - Deaths 1992 ICD9 as it appears in World Health Organization (WHO). WHO Mortality Database Version September 2016. Geneva, Switzerland: World Health Organization (WHO), 2016

Antigua and Barbuda Vital Registration - Deaths 1993 ICD9 as it appears in World Health Organization (WHO). WHO Mortality Database Version September 2016. Geneva, Switzerland: World Health Organization (WHO), 2016
Antigua and Barbuda Vital Registration - Deaths 1994 ICD9 as it appears in World Health Organization (WHO). WHO Mortality Database Version September 2016. Geneva, Switzerland: World Health Organization (WHO), 2016
Antigua and Barbuda Vital Registration - Deaths 1995 ICD9 as it appears in World Health Organization (WHO). WHO Mortality Database Version September 2016. Geneva, Switzerland: World Health Organization (WHO), 2016
Antigua and Barbuda Vital Registration - Deaths 2000 ICD10 as it appears in World Health Organization (WHO). WHO Mortality Database Version October 2017. Geneva, Switzerland: World Health Organization (WHO), 2017
Antigua and Barbuda Vital Registration - Deaths 2001 ICD10 as it appears in World Health Organization (WHO). WHO Mortality Database Version October 2017. Geneva, Switzerland: World Health Organization (WHO), 2017
Antigua and Barbuda Vital Registration - Deaths 2002 ICD10 as it appears in World Health Organization (WHO). WHO Mortality Database Version October 2017. Geneva, Switzerland: World Health Organization (WHO), 2017
Antigua and Barbuda Vital Registration - Deaths 2003 ICD10 as it appears in World Health Organization (WHO). WHO Mortality Database Version October 2017. Geneva, Switzerland: World Health Organization (WHO), 2017
Antigua and Barbuda Vital Registration - Deaths 2004 ICD10 as it appears in World Health Organization (WHO). WHO Mortality Database Version October 2017. Geneva, Switzerland: World Health Organization (WHO), 2017
Antigua and Barbuda Vital Registration - Deaths 2005 ICD10 as it appears in World Health Organization (WHO). WHO Mortality Database Version October 2017. Geneva, Switzerland: World Health Organization (WHO), 2017
Antigua and Barbuda Vital Registration - Deaths 2006 ICD10 as it appears in World Health Organization (WHO). WHO Mortality Database Version October 2017. Geneva, Switzerland: World Health Organization (WHO), 2017
Antigua and Barbuda Vital Registration - Deaths 1985 ICD9 as it appears in World Health Organization (WHO). WHO Mortality Database Version September 2016. Geneva, Switzerland: World Health Organization (WHO), 2016
Antigua and Barbuda Vital Registration - Deaths 2007 ICD10 as it appears in World Health Organization (WHO). WHO Mortality Database Version October 2017. Geneva, Switzerland: World Health Organization (WHO), 2017
Antigua and Barbuda Vital Registration - Deaths 2008 ICD10 as it appears in World Health Organization (WHO). WHO Mortality Database Version October 2017. Geneva, Switzerland: World Health Organization (WHO), 2017
Antigua and Barbuda Vital Registration - Deaths 2009 ICD10 as it appears in World Health Organization (WHO). WHO Mortality Database Version October 2017. Geneva, Switzerland: World Health Organization (WHO), 2017
Antigua and Barbuda Vital Registration - Deaths 2012 ICD10 as it appears in World Health Organization (WHO). WHO Mortality Database Version October 2017. Geneva, Switzerland: World Health Organization (WHO), 2017
Antigua and Barbuda Vital Registration - Deaths 2013 ICD10 as it appears in World Health Organization (WHO). WHO Mortality Database Version October 2017. Geneva, Switzerland: World Health Organization (WHO), 2017
Antigua and Barbuda Vital Registration - Deaths 2014 ICD10 as it appears in World Health Organization (WHO). WHO Mortality Database Version October 2017. Geneva, Switzerland: World Health Organization (WHO), 2017
Antigua and Barbuda Vital Registration - Deaths 2015 ICD10 as it appears in World Health Organization (WHO). WHO Mortality Database Version October 2017. Geneva, Switzerland: World Health Organization (WHO), 2017

Argentina Vital Registration - Deaths 1980 ICD9 as it appears in World Health Organization (WHO). WHO Mortality Database Version September 2016. Geneva, Switzerland: World Health Organization (WHO), 2016
Argentina Vital Registration - Deaths 1981 ICD9 as it appears in World Health Organization (WHO). WHO Mortality Database Version September 2016. Geneva, Switzerland: World Health Organization (WHO), 2016
Argentina Vital Registration - Deaths 1982 ICD9 as it appears in World Health Organization (WHO). WHO Mortality Database Version September 2016. Geneva, Switzerland: World Health Organization (WHO), 2016
Argentina Vital Registration - Deaths 1983 ICD9 as it appears in World Health Organization (WHO). WHO Mortality Database Version September 2016. Geneva, Switzerland: World Health Organization (WHO), 2016
Argentina Vital Registration - Deaths 1984 ICD9 as it appears in World Health Organization (WHO). WHO Mortality Database Version September 2016. Geneva, Switzerland: World Health Organization (WHO), 2016
Argentina Vital Registration - Deaths 1985 ICD9 as it appears in World Health Organization (WHO). WHO Mortality Database Version September 2016. Geneva, Switzerland: World Health Organization (WHO), 2016
Argentina Vital Registration - Deaths 1986 ICD9 as it appears in World Health Organization (WHO). WHO Mortality Database Version September 2016. Geneva, Switzerland: World Health Organization (WHO), 2016
Argentina Vital Registration - Deaths 1987 ICD9 as it appears in World Health Organization (WHO). WHO Mortality Database Version September 2016. Geneva, Switzerland: World Health Organization (WHO), 2016
Argentina Vital Registration - Deaths 1988 ICD9 as it appears in World Health Organization (WHO). WHO Mortality Database Version September 2016. Geneva, Switzerland: World Health Organization (WHO), 2016
Argentina Vital Registration - Deaths 1989 ICD9 as it appears in World Health Organization (WHO). WHO Mortality Database Version September 2016. Geneva, Switzerland: World Health Organization (WHO), 2016
Argentina Vital Registration - Deaths 1990 ICD9 as it appears in World Health Organization (WHO). WHO Mortality Database Version September 2016. Geneva, Switzerland: World Health Organization (WHO), 2016
Argentina Vital Registration - Deaths 1991 ICD9 as it appears in World Health Organization (WHO). WHO Mortality Database Version September 2016. Geneva, Switzerland: World Health Organization (WHO), 2016
Argentina Vital Registration - Deaths 1992 ICD9 as it appears in World Health Organization (WHO). WHO Mortality Database Version September 2016. Geneva, Switzerland: World Health Organization (WHO), 2016
Argentina Vital Registration - Deaths 1993 ICD9 as it appears in World Health Organization (WHO). WHO Mortality Database Version September 2016. Geneva, Switzerland: World Health Organization (WHO), 2016
Argentina Vital Registration - Deaths 1994 ICD9 as it appears in World Health Organization (WHO). WHO Mortality Database Version September 2016. Geneva, Switzerland: World Health Organization (WHO), 2016
Argentina Vital Registration - Deaths 1995 ICD9 as it appears in World Health Organization (WHO). WHO Mortality Database Version September 2016. Geneva, Switzerland: World Health Organization (WHO), 2016
Argentina Vital Registration - Deaths 1996 ICD9 as it appears in World Health Organization (WHO). WHO Mortality Database Version September 2016. Geneva, Switzerland: World Health Organization (WHO), 2016
Argentina Vital Registration - Deaths 1997 ICD10 as it appears in World Health Organization (WHO). WHO Mortality Database Version October 2017. Geneva, Switzerland: World Health Organization (WHO), 2017

Argentina Vital Registration - Deaths 1998 ICD10 as it appears in World Health Organization (WHO). WHO Mortality Database Version October 2017. Geneva, Switzerland: World Health Organization (WHO), 2017
Argentina Vital Registration - Deaths 1999 ICD10 as it appears in World Health Organization (WHO). WHO Mortality Database Version October 2017. Geneva, Switzerland: World Health Organization (WHO), 2017
Argentina Vital Registration - Deaths 2000 ICD10 as it appears in World Health Organization (WHO). WHO Mortality Database Version October 2017. Geneva, Switzerland: World Health Organization (WHO), 2017
Argentina Vital Registration - Deaths 2001 ICD10 as it appears in World Health Organization (WHO). WHO Mortality Database Version October 2017. Geneva, Switzerland: World Health Organization (WHO), 2017
Argentina Vital Registration - Deaths 2002 ICD10 as it appears in World Health Organization (WHO). WHO Mortality Database Version October 2017. Geneva, Switzerland: World Health Organization (WHO), 2017
Argentina Vital Registration - Deaths 2003 ICD10 as it appears in World Health Organization (WHO). WHO Mortality Database Version October 2017. Geneva, Switzerland: World Health Organization (WHO), 2017
Argentina Vital Registration - Deaths 2004 ICD10 as it appears in World Health Organization (WHO). WHO Mortality Database Version October 2017. Geneva, Switzerland: World Health Organization (WHO), 2017
Argentina Vital Registration - Deaths 2005 ICD10 as it appears in World Health Organization (WHO). WHO Mortality Database Version October 2017. Geneva, Switzerland: World Health Organization (WHO), 2017
Argentina Vital Registration - Deaths 2006 ICD10 as it appears in World Health Organization (WHO). WHO Mortality Database Version October 2017. Geneva, Switzerland: World Health Organization (WHO), 2017
Argentina Vital Registration - Deaths 2007 ICD10 as it appears in World Health Organization (WHO). WHO Mortality Database Version October 2017. Geneva, Switzerland: World Health Organization (WHO), 2017
Argentina Vital Registration - Deaths 2008 ICD10 as it appears in World Health Organization (WHO). WHO Mortality Database Version October 2017. Geneva, Switzerland: World Health Organization (WHO), 2017
Argentina Vital Registration - Deaths 2009 ICD10 as it appears in World Health Organization (WHO). WHO Mortality Database Version October 2017. Geneva, Switzerland: World Health Organization (WHO), 2017
Argentina Vital Registration - Deaths 2010 ICD10 as it appears in World Health Organization (WHO). WHO Mortality Database Version October 2017. Geneva, Switzerland: World Health Organization (WHO), 2017
Argentina Vital Registration - Deaths 2011 ICD10 as it appears in World Health Organization (WHO). WHO Mortality Database Version October 2017. Geneva, Switzerland: World Health Organization (WHO), 2017
Argentina Vital Registration - Deaths 2012 ICD10 as it appears in World Health Organization (WHO). WHO Mortality Database Version October 2017. Geneva, Switzerland: World Health Organization (WHO), 2017
Argentina Vital Registration - Deaths 2013 ICD10 as it appears in World Health Organization (WHO). WHO Mortality Database Version October 2017. Geneva, Switzerland: World Health Organization (WHO), 2017
Argentina Vital Registration - Deaths 2014 ICD10 as it appears in World Health Organization (WHO). WHO Mortality Database Version October 2017. Geneva, Switzerland: World Health Organization (WHO), 2017
Argentina Vital Registration - Deaths 2015 ICD10 as it appears in World Health Organization (WHO). WHO Mortality Database Version April 2018. Geneva, Switzerland: World Health Organization (WHO), 2018

Armenia Vital Registration - Deaths 1981 ICD9 as it appears in World Health Organization (WHO). WHO Mortality Database Version September 2016. Geneva, Switzerland: World Health Organization (WHO), 2016
Armenia Vital Registration - Deaths 1982 ICD9 as it appears in World Health Organization (WHO). WHO Mortality Database Version September 2016. Geneva, Switzerland: World Health Organization (WHO), 2016
Armenia Vital Registration - Deaths 1985 ICD9 as it appears in World Health Organization (WHO). WHO Mortality Database Version September 2016. Geneva, Switzerland: World Health Organization (WHO), 2016
Armenia Vital Registration - Deaths 1986 ICD9 as it appears in World Health Organization (WHO). WHO Mortality Database Version September 2016. Geneva, Switzerland: World Health Organization (WHO), 2016
Armenia Vital Registration - Deaths 1987 ICD9 as it appears in World Health Organization (WHO). WHO Mortality Database Version September 2016. Geneva, Switzerland: World Health Organization (WHO), 2016
Armenia Vital Registration - Deaths 1989 ICD9 as it appears in World Health Organization (WHO). WHO Mortality Database Version September 2016. Geneva, Switzerland: World Health Organization (WHO), 2016
Armenia Vital Registration - Deaths 1990 ICD9 as it appears in World Health Organization (WHO). WHO Mortality Database Version September 2016. Geneva, Switzerland: World Health Organization (WHO), 2016
Armenia Vital Registration - Deaths 1991 ICD9 as it appears in World Health Organization (WHO). WHO Mortality Database Version September 2016. Geneva, Switzerland: World Health Organization (WHO), 2016
Armenia Vital Registration - Deaths 1992 ICD9 as it appears in World Health Organization (WHO). WHO Mortality Database Version September 2016. Geneva, Switzerland: World Health Organization (WHO), 2016
Armenia Vital Registration - Deaths 1993 ICD9 as it appears in World Health Organization (WHO). WHO Mortality Database Version September 2016. Geneva, Switzerland: World Health Organization (WHO), 2016
Armenia Vital Registration - Deaths 1994 ICD9 as it appears in World Health Organization (WHO). WHO Mortality Database Version September 2016. Geneva, Switzerland: World Health Organization (WHO), 2016
Armenia Vital Registration - Deaths 1995 ICD9 as it appears in World Health Organization (WHO). WHO Mortality Database Version September 2016. Geneva, Switzerland: World Health Organization (WHO), 2016
Armenia Vital Registration - Deaths 1996 ICD9 as it appears in World Health Organization (WHO). WHO Mortality Database Version September 2016. Geneva, Switzerland: World Health Organization (WHO), 2016
Armenia Vital Registration - Deaths 1997 ICD9 as it appears in World Health Organization (WHO). WHO Mortality Database Version September 2016. Geneva, Switzerland: World Health Organization (WHO), 2016
Armenia Vital Registration - Deaths 1998 ICD9 as it appears in World Health Organization (WHO). WHO Mortality Database Version September 2016. Geneva, Switzerland: World Health Organization (WHO), 2016
Armenia Vital Registration - Deaths 1999 ICD9 as it appears in World Health Organization (WHO). WHO Mortality Database Version September 2016. Geneva, Switzerland: World Health Organization (WHO), 2016
Armenia Vital Registration - Deaths 2000 ICD9 as it appears in World Health Organization (WHO). WHO Mortality Database Version September 2016. Geneva, Switzerland: World Health Organization (WHO), 2016
Armenia Vital Registration - Deaths 2001 ICD9 as it appears in World Health Organization (WHO). WHO Mortality Database Version September 2016. Geneva, Switzerland: World Health Organization (WHO), 2016

Armenia Vital Registration - Deaths 2002 ICD9 as it appears in World Health Organization (WHO). WHO Mortality Database Version September 2016. Geneva, Switzerland: World Health Organization (WHO), 2016
Armenia Vital Registration - Deaths 2003 ICD9 as it appears in World Health Organization (WHO). WHO Mortality Database Version September 2016. Geneva, Switzerland: World Health Organization (WHO), 2016
Armenia Vital Registration - Deaths 2006 ICD10 as it appears in World Health Organization (WHO). WHO Mortality Database Version November 2015. Geneva, Switzerland: World Health Organization (WHO), 2015
Armenia Vital Registration - Deaths 2008 ICD10 as it appears in World Health Organization (WHO). WHO Mortality Database Version November 2015. Geneva, Switzerland: World Health Organization (WHO), 2015
Armenia Vital Registration - Deaths 2009 ICD10 as it appears in World Health Organization (WHO). WHO Mortality Database Version November 2015. Geneva, Switzerland: World Health Organization (WHO), 2015
Armenia Vital Registration - Deaths 2010 ICD10 as it appears in World Health Organization (WHO). WHO Mortality Database Version November 2015. Geneva, Switzerland: World Health Organization (WHO), 2015
Armenia Vital Registration - Deaths 2011 ICD10 as it appears in World Health Organization (WHO). WHO Mortality Database Version November 2015. Geneva, Switzerland: World Health Organization (WHO), 2015
Armenia Vital Registration - Deaths 2012 ICD10 as it appears in World Health Organization (WHO). WHO Mortality Database Version October 2017. Geneva, Switzerland: World Health Organization (WHO), 2017
Armenia Vital Registration - Deaths 2013 ICD10 as it appears in World Health Organization (WHO). WHO Mortality Database Version March 2017. Geneva, Switzerland: World Health Organization (WHO), 2017
Armenia Vital Registration - Deaths 2014 ICD10 as it appears in World Health Organization (WHO). WHO Mortality Database Version October 2017. Geneva, Switzerland: World Health Organization (WHO), 2017
Armenia Vital Registration - Deaths 2015 ICD10 as it appears in World Health Organization (WHO). WHO Mortality Database Version March 2017. Geneva, Switzerland: World Health Organization (WHO), 2017
Armenia Vital Registration - Deaths 2016 ICD10 as it appears in World Health Organization (WHO). WHO Mortality Database Version April 2018. Geneva, Switzerland: World Health Organization (WHO), 2018
Australia Vital Registration - Deaths 1980 ICD9 as it appears in World Health Organization (WHO). WHO Mortality Database Version November 2015. Geneva, Switzerland: World Health Organization (WHO), 2015
Australia Vital Registration - Deaths 1981 ICD9 as it appears in World Health Organization (WHO). WHO Mortality Database Version November 2015. Geneva, Switzerland: World Health Organization (WHO), 2015
Australia Vital Registration - Deaths 1982 ICD9 as it appears in World Health Organization (WHO). WHO Mortality Database Version November 2015. Geneva, Switzerland: World Health Organization (WHO), 2015
Australia Vital Registration - Deaths 1983 ICD9 as it appears in World Health Organization (WHO). WHO Mortality Database Version November 2015. Geneva, Switzerland: World Health Organization (WHO), 2015
Australia Vital Registration - Deaths 1984 ICD9 as it appears in World Health Organization (WHO). WHO Mortality Database Version November 2015. Geneva, Switzerland: World Health Organization (WHO), 2015
Australia Vital Registration - Deaths 1985 ICD9 as it appears in World Health Organization (WHO). WHO Mortality Database Version November 2015. Geneva, Switzerland: World Health Organization (WHO), 2015

Australia Vital Registration - Deaths 1986 ICD9 as it appears in World Health Organization (WHO). WHO Mortality Database Version November 2015. Geneva, Switzerland: World Health Organization (WHO), 2015

Australia Vital Registration - Deaths 1987 ICD9 as it appears in World Health Organization (WHO). WHO Mortality Database Version November 2015. Geneva, Switzerland: World Health Organization (WHO), 2015

Australia Vital Registration - Deaths 1988 ICD9 as it appears in World Health Organization (WHO). WHO Mortality Database Version November 2015. Geneva, Switzerland: World Health Organization (WHO), 2015

Australia Vital Registration - Deaths 1989 ICD9 as it appears in World Health Organization (WHO). WHO Mortality Database Version November 2015. Geneva, Switzerland: World Health Organization (WHO), 2015

Australia Vital Registration - Deaths 1990 ICD9 as it appears in World Health Organization (WHO). WHO Mortality Database Version November 2015. Geneva, Switzerland: World Health Organization (WHO), 2015

Australia Vital Registration - Deaths 1991 ICD9 as it appears in World Health Organization (WHO). WHO Mortality Database Version November 2015. Geneva, Switzerland: World Health Organization (WHO), 2015

Australia Vital Registration - Deaths 1992 ICD9 as it appears in World Health Organization (WHO). WHO Mortality Database Version November 2015. Geneva, Switzerland: World Health Organization (WHO), 2015

Australia Vital Registration - Deaths 1993 ICD9 as it appears in World Health Organization (WHO). WHO Mortality Database Version November 2015. Geneva, Switzerland: World Health Organization (WHO), 2015

Australia Vital Registration - Deaths 1994 ICD9 as it appears in World Health Organization (WHO). WHO Mortality Database Version November 2015. Geneva, Switzerland: World Health Organization (WHO), 2015

Australia Vital Registration - Deaths 1995 ICD9 as it appears in World Health Organization (WHO). WHO Mortality Database Version November 2015. Geneva, Switzerland: World Health Organization (WHO), 2015

Australia Vital Registration - Deaths 1996 ICD9 as it appears in World Health Organization (WHO). WHO Mortality Database Version November 2015. Geneva, Switzerland: World Health Organization (WHO), 2015

Australia Vital Registration - Deaths 1997 ICD9 as it appears in World Health Organization (WHO). WHO Mortality Database Version November 2015. Geneva, Switzerland: World Health Organization (WHO), 2015

Australia Vital Registration - Deaths 1998 ICD10 as it appears in World Health Organization (WHO). WHO Mortality Database Version October 2017. Geneva, Switzerland: World Health Organization (WHO), 2017

Australia Vital Registration - Deaths 1999 ICD10 as it appears in World Health Organization (WHO). WHO Mortality Database Version October 2017. Geneva, Switzerland: World Health Organization (WHO), 2017

Australia Vital Registration - Deaths 2000 ICD10 as it appears in World Health Organization (WHO). WHO Mortality Database Version October 2017. Geneva, Switzerland: World Health Organization (WHO), 2017

Australia Vital Registration - Deaths 2001 ICD10 as it appears in World Health Organization (WHO). WHO Mortality Database Version October 2017. Geneva, Switzerland: World Health Organization (WHO), 2017

Australia Vital Registration - Deaths 2002 ICD10 as it appears in World Health Organization (WHO). WHO Mortality Database Version October 2017. Geneva, Switzerland: World Health Organization (WHO), 2017

Australia Vital Registration - Deaths 2003 ICD10 as it appears in World Health Organization (WHO). WHO Mortality Database Version October 2017. Geneva, Switzerland: World Health Organization (WHO), 2017

Australia Vital Registration - Deaths 2004 ICD10 as it appears in World Health Organization (WHO). WHO Mortality Database Version October 2017. Geneva, Switzerland: World Health Organization (WHO), 2017

Australia Vital Registration - Deaths 2006 ICD10 as it appears in World Health Organization (WHO). WHO Mortality Database Version October 2017. Geneva, Switzerland: World Health Organization (WHO), 2017

Australia Vital Registration - Deaths 2007 ICD10 as it appears in World Health Organization (WHO). WHO Mortality Database Version October 2017. Geneva, Switzerland: World Health Organization (WHO), 2017

Australia Vital Registration - Deaths 2008 ICD10 as it appears in World Health Organization (WHO). WHO Mortality Database Version October 2017. Geneva, Switzerland: World Health Organization (WHO), 2017

Australia Vital Registration - Deaths 2009 ICD10 as it appears in World Health Organization (WHO). WHO Mortality Database Version October 2017. Geneva, Switzerland: World Health Organization (WHO), 2017

Australia Vital Registration - Deaths 2010 ICD10 as it appears in World Health Organization (WHO). WHO Mortality Database Version October 2017. Geneva, Switzerland: World Health Organization (WHO), 2017

Australia Vital Registration - Deaths 2011 ICD10 as it appears in World Health Organization (WHO). WHO Mortality Database Version October 2017. Geneva, Switzerland: World Health Organization (WHO), 2017

Australia Vital Registration - Deaths 2012 ICD10 as it appears in World Health Organization (WHO). WHO Mortality Database Version October 2017. Geneva, Switzerland: World Health Organization (WHO), 2017

Australia Vital Registration - Deaths 2013 ICD10 as it appears in World Health Organization (WHO). WHO Mortality Database Version October 2017. Geneva, Switzerland: World Health Organization (WHO), 2017

Australia Vital Registration - Deaths 2014 ICD10 as it appears in World Health Organization (WHO). WHO Mortality Database Version October 2017. Geneva, Switzerland: World Health Organization (WHO), 2017

Australia Vital Registration - Deaths 2015 ICD10 as it appears in World Health Organization (WHO). WHO Mortality Database Version October 2017. Geneva, Switzerland: World Health Organization (WHO), 2017

Australian Bureau of Statistics. Australia Vital Registration - Deaths 2005

Austria Vital Registration - Deaths 1980 ICD9 as it appears in World Health Organization (WHO). WHO Mortality Database Version November 2015. Geneva, Switzerland: World Health Organization (WHO), 2015

Austria Vital Registration - Deaths 1981 ICD9 as it appears in World Health Organization (WHO). WHO Mortality Database Version November 2015. Geneva, Switzerland: World Health Organization (WHO), 2015

Austria Vital Registration - Deaths 1982 ICD9 as it appears in World Health Organization (WHO). WHO Mortality Database Version November 2015. Geneva, Switzerland: World Health Organization (WHO), 2015

Austria Vital Registration - Deaths 1983 ICD9 as it appears in World Health Organization (WHO). WHO Mortality Database Version September 2016. Geneva, Switzerland: World Health Organization (WHO), 2016

Austria Vital Registration - Deaths 1984 ICD9 as it appears in World Health Organization (WHO). WHO Mortality Database Version November 2015. Geneva, Switzerland: World Health Organization (WHO), 2015

Austria Vital Registration - Deaths 1985 ICD9 as it appears in World Health Organization (WHO). WHO Mortality Database Version November 2015. Geneva, Switzerland: World Health Organization (WHO), 2015

Austria Vital Registration - Deaths 1986 ICD9 as it appears in World Health Organization (WHO). WHO Mortality Database Version November 2015. Geneva, Switzerland: World Health Organization (WHO), 2015
Austria Vital Registration - Deaths 1987 ICD9 as it appears in World Health Organization (WHO). WHO Mortality Database Version November 2015. Geneva, Switzerland: World Health Organization (WHO), 2015
Austria Vital Registration - Deaths 1988 ICD9 as it appears in World Health Organization (WHO). WHO Mortality Database Version November 2015. Geneva, Switzerland: World Health Organization (WHO), 2015
Austria Vital Registration - Deaths 1989 ICD9 as it appears in World Health Organization (WHO). WHO Mortality Database Version November 2015. Geneva, Switzerland: World Health Organization (WHO), 2015
Austria Vital Registration - Deaths 1990 ICD9 as it appears in World Health Organization (WHO). WHO Mortality Database Version November 2015. Geneva, Switzerland: World Health Organization (WHO), 2015
Austria Vital Registration - Deaths 1991 ICD9 as it appears in World Health Organization (WHO). WHO Mortality Database Version November 2015. Geneva, Switzerland: World Health Organization (WHO), 2015
Austria Vital Registration - Deaths 1992 ICD9 as it appears in World Health Organization (WHO). WHO Mortality Database Version November 2015. Geneva, Switzerland: World Health Organization (WHO), 2015
Austria Vital Registration - Deaths 1993 ICD9 as it appears in World Health Organization (WHO). WHO Mortality Database Version November 2015. Geneva, Switzerland: World Health Organization (WHO), 2015
Austria Vital Registration - Deaths 1994 ICD9 as it appears in World Health Organization (WHO). WHO Mortality Database Version November 2015. Geneva, Switzerland: World Health Organization (WHO), 2015
Austria Vital Registration - Deaths 1995 ICD9 as it appears in World Health Organization (WHO). WHO Mortality Database Version November 2015. Geneva, Switzerland: World Health Organization (WHO), 2015
Austria Vital Registration - Deaths 1996 ICD9 as it appears in World Health Organization (WHO). WHO Mortality Database Version November 2015. Geneva, Switzerland: World Health Organization (WHO), 2015
Austria Vital Registration - Deaths 2002 ICD10 as it appears in World Health Organization (WHO). WHO Mortality Database Version October 2017. Geneva, Switzerland: World Health Organization (WHO), 2017
Austria Vital Registration - Deaths 2003 ICD10 as it appears in World Health Organization (WHO). WHO Mortality Database Version October 2017. Geneva, Switzerland: World Health Organization (WHO), 2017
Austria Vital Registration - Deaths 2004 ICD10 as it appears in World Health Organization (WHO). WHO Mortality Database Version October 2017. Geneva, Switzerland: World Health Organization (WHO), 2017
Austria Vital Registration - Deaths 2005 ICD10 as it appears in World Health Organization (WHO). WHO Mortality Database Version October 2017. Geneva, Switzerland: World Health Organization (WHO), 2017
Austria Vital Registration - Deaths 2006 ICD10 as it appears in World Health Organization (WHO). WHO Mortality Database Version October 2017. Geneva, Switzerland: World Health Organization (WHO), 2017
Austria Vital Registration - Deaths 2007 ICD10 as it appears in World Health Organization (WHO). WHO Mortality Database Version October 2017. Geneva, Switzerland: World Health Organization (WHO), 2017
Austria Vital Registration - Deaths 2008 ICD10 as it appears in World Health Organization (WHO). WHO Mortality Database Version October 2017. Geneva, Switzerland: World Health Organization (WHO), 2017

Austria Vital Registration - Deaths 1997 ICD9 as it appears in World Health Organization (WHO). WHO Mortality Database Version November 2015. Geneva, Switzerland: World Health Organization (WHO), 2015
Austria Vital Registration - Deaths 1998 ICD9 as it appears in World Health Organization (WHO). WHO Mortality Database Version November 2015. Geneva, Switzerland: World Health Organization (WHO), 2015
Austria Vital Registration - Deaths 1999 ICD9 as it appears in World Health Organization (WHO). WHO Mortality Database Version November 2015. Geneva, Switzerland: World Health Organization (WHO), 2015
Austria Vital Registration - Deaths 2000 ICD9 as it appears in World Health Organization (WHO). WHO Mortality Database Version November 2015. Geneva, Switzerland: World Health Organization (WHO), 2015
Austria Vital Registration - Deaths 2001 ICD9 as it appears in World Health Organization (WHO). WHO Mortality Database Version November 2015. Geneva, Switzerland: World Health Organization (WHO), 2015
Austria Vital Registration - Deaths 2009 ICD10 as it appears in World Health Organization (WHO). WHO Mortality Database Version October 2017. Geneva, Switzerland: World Health Organization (WHO), 2017
Austria Vital Registration - Deaths 2010 ICD10 as it appears in World Health Organization (WHO). WHO Mortality Database Version October 2017. Geneva, Switzerland: World Health Organization (WHO), 2017
Austria Vital Registration - Deaths 2011 ICD10 as it appears in World Health Organization (WHO). WHO Mortality Database Version October 2017. Geneva, Switzerland: World Health Organization (WHO), 2017
Austria Vital Registration - Deaths 2012 ICD10 as it appears in World Health Organization (WHO). WHO Mortality Database Version October 2017. Geneva, Switzerland: World Health Organization (WHO), 2017
Austria Vital Registration - Deaths 2013 ICD10 as it appears in World Health Organization (WHO). WHO Mortality Database Version October 2017. Geneva, Switzerland: World Health Organization (WHO), 2017
Austria Vital Registration - Deaths 2014 ICD10 as it appears in World Health Organization (WHO). WHO Mortality Database Version October 2017. Geneva, Switzerland: World Health Organization (WHO), 2017
Austria Vital Registration - Deaths 2015 ICD10 as it appears in World Health Organization (WHO). WHO Mortality Database Version October 2017. Geneva, Switzerland: World Health Organization (WHO), 2017
Austria Vital Registration - Deaths 2016 ICD10 as it appears in World Health Organization (WHO). WHO Mortality Database Version October 2017. Geneva, Switzerland: World Health Organization (WHO), 2017
Azerbaijan Vital Registration - Deaths 1992 ICD9 as it appears in World Health Organization (WHO). WHO Mortality Database Version September 2016. Geneva, Switzerland: World Health Organization (WHO), 2016
Azerbaijan Vital Registration - Deaths 1993 ICD9 as it appears in World Health Organization (WHO). WHO Mortality Database Version September 2016. Geneva, Switzerland: World Health Organization (WHO), 2016
Azerbaijan Vital Registration - Deaths 1994 ICD9 as it appears in World Health Organization (WHO). WHO Mortality Database Version September 2016. Geneva, Switzerland: World Health Organization (WHO), 2016
Azerbaijan Vital Registration - Deaths 1995 ICD9 as it appears in World Health Organization (WHO). WHO Mortality Database Version September 2016. Geneva, Switzerland: World Health Organization (WHO), 2016
Azerbaijan Vital Registration - Deaths 1996 ICD9 as it appears in World Health Organization (WHO). WHO Mortality Database Version September 2016. Geneva, Switzerland: World Health Organization (WHO), 2016

Azerbaijan Vital Registration - Deaths 1997 ICD9 as it appears in World Health Organization (WHO). WHO Mortality Database Version September 2016. Geneva, Switzerland: World Health Organization (WHO), 2016
Azerbaijan Vital Registration - Deaths 1998 ICD9 as it appears in World Health Organization (WHO). WHO Mortality Database Version September 2016. Geneva, Switzerland: World Health Organization (WHO), 2016
Azerbaijan Vital Registration - Deaths 1999 ICD9 as it appears in World Health Organization (WHO). WHO Mortality Database Version September 2016. Geneva, Switzerland: World Health Organization (WHO), 2016
Azerbaijan Vital Registration - Deaths 2000 ICD9 as it appears in World Health Organization (WHO). WHO Mortality Database Version September 2016. Geneva, Switzerland: World Health Organization (WHO), 2016
Azerbaijan Vital Registration - Deaths 2001 ICD10 as it appears in World Health Organization (WHO). WHO Mortality Database Version November 2015. Geneva, Switzerland: World Health Organization (WHO), 2015
Azerbaijan Vital Registration - Deaths 2002 ICD10 as it appears in World Health Organization (WHO). WHO Mortality Database Version November 2015. Geneva, Switzerland: World Health Organization (WHO), 2015
Azerbaijan Vital Registration - Deaths 2003 ICD10 as it appears in World Health Organization (WHO). WHO Mortality Database Version November 2015. Geneva, Switzerland: World Health Organization (WHO), 2015
Azerbaijan Vital Registration - Deaths 2004 ICD10 as it appears in World Health Organization (WHO). WHO Mortality Database Version November 2015. Geneva, Switzerland: World Health Organization (WHO), 2015
Azerbaijan Vital Registration - Deaths 2007 ICD10 as it appears in World Health Organization (WHO). WHO Mortality Database Version October 2017. Geneva, Switzerland: World Health Organization (WHO), 2017
Azerbaijan Vital Registration - Deaths 1981 ICD9 as it appears in World Health Organization (WHO). WHO Mortality Database Version September 2016. Geneva, Switzerland: World Health Organization (WHO), 2016
Azerbaijan Vital Registration - Deaths 1982 ICD9 as it appears in World Health Organization (WHO). WHO Mortality Database Version September 2016. Geneva, Switzerland: World Health Organization (WHO), 2016
Azerbaijan Vital Registration - Deaths 1985 ICD9 as it appears in World Health Organization (WHO). WHO Mortality Database Version September 2016. Geneva, Switzerland: World Health Organization (WHO), 2016
Azerbaijan Vital Registration - Deaths 1986 ICD9 as it appears in World Health Organization (WHO). WHO Mortality Database Version September 2016. Geneva, Switzerland: World Health Organization (WHO), 2016
Azerbaijan Vital Registration - Deaths 1987 ICD9 as it appears in World Health Organization (WHO). WHO Mortality Database Version September 2016. Geneva, Switzerland: World Health Organization (WHO), 2016
Azerbaijan Vital Registration - Deaths 1988 ICD9 as it appears in World Health Organization (WHO). WHO Mortality Database Version September 2016. Geneva, Switzerland: World Health Organization (WHO), 2016
Azerbaijan Vital Registration - Deaths 1989 ICD9 as it appears in World Health Organization (WHO). WHO Mortality Database Version September 2016. Geneva, Switzerland: World Health Organization (WHO), 2016
Azerbaijan Vital Registration - Deaths 1990 ICD9 as it appears in World Health Organization (WHO). WHO Mortality Database Version September 2016. Geneva, Switzerland: World Health Organization (WHO), 2016
Azerbaijan Vital Registration - Deaths 1991 ICD9 as it appears in World Health Organization (WHO). WHO Mortality Database Version September 2016. Geneva, Switzerland: World Health Organization (WHO), 2016

Bahamas Vital Registration - Deaths 1980 ICD9 as it appears in World Health Organization (WHO). WHO Mortality Database Version September 2016. Geneva, Switzerland: World Health Organization (WHO), 2016
Bahamas Vital Registration - Deaths 1981 ICD9 as it appears in World Health Organization (WHO). WHO Mortality Database Version September 2016. Geneva, Switzerland: World Health Organization (WHO), 2016
Bahamas Vital Registration - Deaths 1984 ICD9 as it appears in World Health Organization (WHO). WHO Mortality Database Version September 2016. Geneva, Switzerland: World Health Organization (WHO), 2016
Bahamas Vital Registration - Deaths 1985 ICD9 as it appears in World Health Organization (WHO). WHO Mortality Database Version September 2016. Geneva, Switzerland: World Health Organization (WHO), 2016
Bahamas Vital Registration - Deaths 1987 ICD9 as it appears in World Health Organization (WHO). WHO Mortality Database Version September 2016. Geneva, Switzerland: World Health Organization (WHO), 2016
Bahamas Vital Registration - Deaths 1993 ICD9 as it appears in World Health Organization (WHO). WHO Mortality Database Version September 2016. Geneva, Switzerland: World Health Organization (WHO), 2016
Bahamas Vital Registration - Deaths 1994 ICD9 as it appears in World Health Organization (WHO). WHO Mortality Database Version September 2016. Geneva, Switzerland: World Health Organization (WHO), 2016
Bahamas Vital Registration - Deaths 1995 ICD9 as it appears in World Health Organization (WHO). WHO Mortality Database Version September 2016. Geneva, Switzerland: World Health Organization (WHO), 2016
Bahamas Vital Registration - Deaths 1996 ICD9 as it appears in World Health Organization (WHO). WHO Mortality Database Version September 2016. Geneva, Switzerland: World Health Organization (WHO), 2016
Bahamas Vital Registration - Deaths 1997 ICD9 as it appears in World Health Organization (WHO). WHO Mortality Database Version September 2016. Geneva, Switzerland: World Health Organization (WHO), 2016
Bahamas Vital Registration - Deaths 1998 ICD9 as it appears in World Health Organization (WHO). WHO Mortality Database Version September 2016. Geneva, Switzerland: World Health Organization (WHO), 2016
Bahamas Vital Registration - Deaths 1999 ICD10 as it appears in World Health Organization (WHO). WHO Mortality Database Version October 2017. Geneva, Switzerland: World Health Organization (WHO), 2017
Bahamas Vital Registration - Deaths 2000 ICD10 as it appears in World Health Organization (WHO). WHO Mortality Database Version October 2017. Geneva, Switzerland: World Health Organization (WHO), 2017
Bahamas Vital Registration - Deaths 2001 ICD10 as it appears in World Health Organization (WHO). WHO Mortality Database Version October 2017. Geneva, Switzerland: World Health Organization (WHO), 2017
Bahamas Vital Registration - Deaths 2002 ICD10 as it appears in World Health Organization (WHO). WHO Mortality Database Version October 2017. Geneva, Switzerland: World Health Organization (WHO), 2017
Bahamas Vital Registration - Deaths 2003 ICD10 as it appears in World Health Organization (WHO). WHO Mortality Database Version October 2017. Geneva, Switzerland: World Health Organization (WHO), 2017
Bahamas Vital Registration - Deaths 2005 ICD10 as it appears in World Health Organization (WHO). WHO Mortality Database Version October 2017. Geneva, Switzerland: World Health Organization (WHO), 2017
Bahamas Vital Registration - Deaths 2004 ICD10 as it appears in World Health Organization (WHO). WHO Mortality Database Version October 2017. Geneva, Switzerland: World Health Organization (WHO), 2017

Bahamas Vital Registration - Deaths 2006 ICD10 as it appears in World Health Organization (WHO). WHO Mortality Database Version October 2017. Geneva, Switzerland: World Health Organization (WHO), 2017
Bahamas Vital Registration - Deaths 2007 ICD10 as it appears in World Health Organization (WHO). WHO Mortality Database Version October 2017. Geneva, Switzerland: World Health Organization (WHO), 2017
Bahamas Vital Registration - Deaths 2008 ICD10 as it appears in World Health Organization (WHO). WHO Mortality Database Version October 2017. Geneva, Switzerland: World Health Organization (WHO), 2017
Bahamas Vital Registration - Deaths 2009 ICD10 as it appears in World Health Organization (WHO). WHO Mortality Database Version October 2017. Geneva, Switzerland: World Health Organization (WHO), 2017
Bahamas Vital Registration - Deaths 2010 ICD10 as it appears in World Health Organization (WHO). WHO Mortality Database Version October 2017. Geneva, Switzerland: World Health Organization (WHO), 2017
Bahamas Vital Registration - Deaths 2011 ICD10 as it appears in World Health Organization (WHO). WHO Mortality Database Version October 2017. Geneva, Switzerland: World Health Organization (WHO), 2017
Bahamas Vital Registration - Deaths 2012 ICD10 as it appears in World Health Organization (WHO). WHO Mortality Database Version October 2017. Geneva, Switzerland: World Health Organization (WHO), 2017
Bahamas Vital Registration - Deaths 2013 ICD10 as it appears in World Health Organization (WHO). WHO Mortality Database Version October 2017. Geneva, Switzerland: World Health Organization (WHO), 2017
Bahrain Vital Registration - Deaths 1997 ICD10 as it appears in World Health Organization (WHO). WHO Mortality Database Version April 2018. Geneva, Switzerland: World Health Organization (WHO), 2018
Bahrain Vital Registration - Deaths 1998 ICD10 as it appears in World Health Organization (WHO). WHO Mortality Database Version April 2018. Geneva, Switzerland: World Health Organization (WHO), 2018
Bahrain Vital Registration - Deaths 1999 ICD10 as it appears in World Health Organization (WHO). WHO Mortality Database Version April 2018. Geneva, Switzerland: World Health Organization (WHO), 2018
Bahrain Vital Registration - Deaths 2000 ICD10 as it appears in World Health Organization (WHO). WHO Mortality Database Version October 2017. Geneva, Switzerland: World Health Organization (WHO), 2017
Bahrain Vital Registration - Deaths 2001 ICD10 as it appears in World Health Organization (WHO). WHO Mortality Database Version October 2017. Geneva, Switzerland: World Health Organization (WHO), 2017
Bahrain Vital Registration - Deaths 1986 as it appears in World Health Organization (WHO). WHO Mortality Database Version September 2016. Geneva, Switzerland: World Health Organization (WHO), 2016
Bahrain Vital Registration - Deaths 2002 ICD10 as it appears in World Health Organization (WHO). WHO Mortality Database Version October 2017. Geneva, Switzerland: World Health Organization (WHO), 2017
Bahrain Vital Registration - Deaths 2003 ICD10 as it appears in World Health Organization (WHO). WHO Mortality Database Version October 2017. Geneva, Switzerland: World Health Organization (WHO), 2017
Bahrain Vital Registration - Deaths 2004 ICD10 as it appears in World Health Organization (WHO). WHO Mortality Database Version October 2017. Geneva, Switzerland: World Health Organization (WHO), 2017
Bahrain Vital Registration - Deaths 2005 ICD10 as it appears in World Health Organization (WHO). WHO Mortality Database Version October 2017. Geneva, Switzerland: World Health Organization (WHO), 2017

Bahrain Vital Registration - Deaths 2006 ICD10 as it appears in World Health Organization (WHO). WHO Mortality Database Version October 2017. Geneva, Switzerland: World Health Organization (WHO), 2017

Bahrain Vital Registration - Deaths 2007 ICD10 as it appears in World Health Organization (WHO). WHO Mortality Database Version October 2017. Geneva, Switzerland: World Health Organization (WHO), 2017

Bahrain Vital Registration - Deaths 2008 ICD10 as it appears in World Health Organization (WHO). WHO Mortality Database Version October 2017. Geneva, Switzerland: World Health Organization (WHO), 2017

Bahrain Vital Registration - Deaths 2009 ICD10 as it appears in World Health Organization (WHO). WHO Mortality Database Version October 2017. Geneva, Switzerland: World Health Organization (WHO), 2017

Bahrain Vital Registration - Deaths 2010 ICD10 as it appears in World Health Organization (WHO). WHO Mortality Database Version October 2017. Geneva, Switzerland: World Health Organization (WHO), 2017

Bahrain Vital Registration - Deaths 2011 ICD10 as it appears in World Health Organization (WHO). WHO Mortality Database Version October 2017. Geneva, Switzerland: World Health Organization (WHO), 2017

Bahrain Vital Registration - Deaths 2012 ICD10 as it appears in World Health Organization (WHO). WHO Mortality Database Version October 2017. Geneva, Switzerland: World Health Organization (WHO), 2017

Bahrain Vital Registration - Deaths 2013 ICD10 as it appears in World Health Organization (WHO). WHO Mortality Database Version October 2017. Geneva, Switzerland: World Health Organization (WHO), 2017

Bahrain Vital Registration - Deaths 2014 ICD10 as it appears in World Health Organization (WHO). WHO Mortality Database Version October 2017. Geneva, Switzerland: World Health Organization (WHO), 2017

Bapat U, Alcock G, More NS, Das S, Joshi W, Osrin D. Stillbirths and newborn deaths in slum settlements in Mumbai, India: a prospective verbal autopsy study. BMC Pregnancy Childbirth. 2012; 12(39)

Baqui AH, Black RE, Arifeen SE, Hill K, Mitra SN, al Sabir A. Causes of childhood deaths in Bangladesh: results of a nationwide verbal autopsy study. Bull World Health Organ. 1998; 76(2): 161-71

Baqui AH, Darmstadt GL, Williams EK, Kumar V, Kiran TU, Panwar D, Srivastava VK, Ahuja R, Black RE, Santosham M. Rates, timing and causes of neonatal deaths in rural India: implications for neonatal health programmes. Bull World Health Organ. 2006; 84(9): 706-13

Barbados Vital Registration - Deaths 1980 ICD9 as it appears in World Health Organization (WHO). WHO Mortality Database Version September 2016. Geneva, Switzerland: World Health Organization (WHO), 2016

Barbados Vital Registration - Deaths 1981 ICD9 as it appears in World Health Organization (WHO). WHO Mortality Database Version September 2016. Geneva, Switzerland: World Health Organization (WHO), 2016

Barbados Vital Registration - Deaths 1982 ICD9 as it appears in World Health Organization (WHO). WHO Mortality Database Version September 2016. Geneva, Switzerland: World Health Organization (WHO), 2016

Barbados Vital Registration - Deaths 1983 ICD9 as it appears in World Health Organization (WHO). WHO Mortality Database Version September 2016. Geneva, Switzerland: World Health Organization (WHO), 2016

Barbados Vital Registration - Deaths 1984 ICD9 as it appears in World Health Organization (WHO). WHO Mortality Database Version September 2016. Geneva, Switzerland: World Health Organization (WHO), 2016

Barbados Vital Registration - Deaths 1985 ICD9 as it appears in World Health Organization (WHO). WHO Mortality Database Version September 2016. Geneva, Switzerland: World Health Organization (WHO), 2016

Barbados Vital Registration - Deaths 1986 ICD9 as it appears in World Health Organization (WHO). WHO Mortality Database Version September 2016. Geneva, Switzerland: World Health Organization (WHO), 2016
Barbados Vital Registration - Deaths 1987 ICD9 as it appears in World Health Organization (WHO). WHO Mortality Database Version September 2016. Geneva, Switzerland: World Health Organization (WHO), 2016
Barbados Vital Registration - Deaths 1988 ICD9 as it appears in World Health Organization (WHO). WHO Mortality Database Version September 2016. Geneva, Switzerland: World Health Organization (WHO), 2016
Barbados Vital Registration - Deaths 1989 ICD9 as it appears in World Health Organization (WHO). WHO Mortality Database Version September 2016. Geneva, Switzerland: World Health Organization (WHO), 2016
Barbados Vital Registration - Deaths 1990 ICD9 as it appears in World Health Organization (WHO). WHO Mortality Database Version September 2016. Geneva, Switzerland: World Health Organization (WHO), 2016
Barbados Vital Registration - Deaths 1991 ICD9 as it appears in World Health Organization (WHO). WHO Mortality Database Version September 2016. Geneva, Switzerland: World Health Organization (WHO), 2016
Barbados Vital Registration - Deaths 1992 ICD9 as it appears in World Health Organization (WHO). WHO Mortality Database Version September 2016. Geneva, Switzerland: World Health Organization (WHO), 2016
Barbados Vital Registration - Deaths 1993 ICD9 as it appears in World Health Organization (WHO). WHO Mortality Database Version September 2016. Geneva, Switzerland: World Health Organization (WHO), 2016
Barbados Vital Registration - Deaths 1994 ICD9 as it appears in World Health Organization (WHO). WHO Mortality Database Version September 2016. Geneva, Switzerland: World Health Organization (WHO), 2016
Barbados Vital Registration - Deaths 1995 ICD9 as it appears in World Health Organization (WHO). WHO Mortality Database Version September 2016. Geneva, Switzerland: World Health Organization (WHO), 2016
Barbados Vital Registration - Deaths 2000 ICD10 as it appears in World Health Organization (WHO). WHO Mortality Database Version October 2017. Geneva, Switzerland: World Health Organization (WHO), 2017
Barbados Vital Registration - Deaths 2001 ICD10 as it appears in World Health Organization (WHO). WHO Mortality Database Version October 2017. Geneva, Switzerland: World Health Organization (WHO), 2017
Barbados Vital Registration - Deaths 2002 ICD10 as it appears in World Health Organization (WHO). WHO Mortality Database Version October 2017. Geneva, Switzerland: World Health Organization (WHO), 2017
Barbados Vital Registration - Deaths 2003 ICD10 as it appears in World Health Organization (WHO). WHO Mortality Database Version October 2017. Geneva, Switzerland: World Health Organization (WHO), 2017
Barbados Vital Registration - Deaths 2004 ICD10 as it appears in World Health Organization (WHO). WHO Mortality Database Version October 2017. Geneva, Switzerland: World Health Organization (WHO), 2017
Barbados Vital Registration - Deaths 2005 ICD10 as it appears in World Health Organization (WHO). WHO Mortality Database Version October 2017. Geneva, Switzerland: World Health Organization (WHO), 2017
Barbados Vital Registration - Deaths 2006 ICD10 as it appears in World Health Organization (WHO). WHO Mortality Database Version October 2017. Geneva, Switzerland: World Health Organization (WHO), 2017
Barbados Vital Registration - Deaths 2007 ICD10 as it appears in World Health Organization (WHO). WHO Mortality Database Version October 2017. Geneva, Switzerland: World Health Organization (WHO), 2017

Barbados Vital Registration - Deaths 2008 ICD10 as it appears in World Health Organization (WHO). WHO Mortality Database Version October 2017. Geneva, Switzerland: World Health Organization (WHO), 2017

Barbados Vital Registration - Deaths 2009 ICD10 as it appears in World Health Organization (WHO). WHO Mortality Database Version October 2017. Geneva, Switzerland: World Health Organization (WHO), 2017

Barbados Vital Registration - Deaths 2010 ICD10 as it appears in World Health Organization (WHO). WHO Mortality Database Version October 2017. Geneva, Switzerland: World Health Organization (WHO), 2017

Barbados Vital Registration - Deaths 2011 ICD10 as it appears in World Health Organization (WHO). WHO Mortality Database Version October 2017. Geneva, Switzerland: World Health Organization (WHO), 2017

Barbados Vital Registration - Deaths 2012 ICD10 as it appears in World Health Organization (WHO). WHO Mortality Database Version October 2017. Geneva, Switzerland: World Health Organization (WHO), 2017

Barbados Vital Registration - Deaths 2013 ICD10 as it appears in World Health Organization (WHO). WHO Mortality Database Version October 2017. Geneva, Switzerland: World Health Organization (WHO), 2017

Barrêto IC, Kerr Pontes L, Corrêa L. Surveillance of infant deaths in local health systems: assessment of verbal autopsy reports and of information gathered from health agents. Rev Panam Salud Publica. 2000; 79(5): 303-12

Baskent University, Ministry of Health (Turkey), State Institute of Statistics (Turkey). Turkey Verbal Autopsy Survey 2003

Batieha AM, Khader YS, Berdzuli N, Chua-Oon C, Badran EF, Al-sheyab NA, Basha AS, Obaidat A, Ra'eda J. Level, Causes and Risk Factors of Neonatal Mortality, in Jordan: Results of a National Prospective Study. Matern Child Health J. 2016; 20(5): 1061–1071

Belarus Vital Registration - Deaths 1981 ICD9 as it appears in World Health Organization (WHO). WHO Mortality Database Version September 2016. Geneva, Switzerland: World Health Organization (WHO), 2016

Belarus Vital Registration - Deaths 1982 ICD9 as it appears in World Health Organization (WHO). WHO Mortality Database Version September 2016. Geneva, Switzerland: World Health Organization (WHO), 2016

Belarus Vital Registration - Deaths 1985 ICD9 as it appears in World Health Organization (WHO). WHO Mortality Database Version September 2016. Geneva, Switzerland: World Health Organization (WHO), 2016

Belarus Vital Registration - Deaths 1986 ICD9 as it appears in World Health Organization (WHO). WHO Mortality Database Version September 2016. Geneva, Switzerland: World Health Organization (WHO), 2016

Belarus Vital Registration - Deaths 1987 ICD9 as it appears in World Health Organization (WHO). WHO Mortality Database Version September 2016. Geneva, Switzerland: World Health Organization (WHO), 2016

Belarus Vital Registration - Deaths 1988 ICD9 as it appears in World Health Organization (WHO). WHO Mortality Database Version September 2016. Geneva, Switzerland: World Health Organization (WHO), 2016

Belarus Vital Registration - Deaths 1989 ICD9 as it appears in World Health Organization (WHO). WHO Mortality Database Version September 2016. Geneva, Switzerland: World Health Organization (WHO), 2016

Belarus Vital Registration - Deaths 1990 ICD9 as it appears in World Health Organization (WHO). WHO Mortality Database Version September 2016. Geneva, Switzerland: World Health Organization (WHO), 2016

Belarus Vital Registration - Deaths 1991 ICD9 as it appears in World Health Organization (WHO). WHO Mortality Database Version September 2016. Geneva, Switzerland: World Health Organization (WHO), 2016

Belarus Vital Registration - Deaths 1992 ICD9 as it appears in World Health Organization (WHO). WHO Mortality Database Version September 2016. Geneva, Switzerland: World Health Organization (WHO), 2016
Belarus Vital Registration - Deaths 1993 ICD9 as it appears in World Health Organization (WHO). WHO Mortality Database Version September 2016. Geneva, Switzerland: World Health Organization (WHO), 2016
Belarus Vital Registration - Deaths 1994 ICD9 as it appears in World Health Organization (WHO). WHO Mortality Database Version September 2016. Geneva, Switzerland: World Health Organization (WHO), 2016
Belarus Vital Registration - Deaths 1995 ICD9 as it appears in World Health Organization (WHO). WHO Mortality Database Version September 2016. Geneva, Switzerland: World Health Organization (WHO), 2016
Belarus Vital Registration - Deaths 1997 ICD9 as it appears in World Health Organization (WHO). WHO Mortality Database Version September 2016. Geneva, Switzerland: World Health Organization (WHO), 2016
Belarus Vital Registration - Deaths 1998 ICD9 as it appears in World Health Organization (WHO). WHO Mortality Database Version September 2016. Geneva, Switzerland: World Health Organization (WHO), 2016
Belarus Vital Registration - Deaths 1999 ICD9 as it appears in World Health Organization (WHO). WHO Mortality Database Version September 2016. Geneva, Switzerland: World Health Organization (WHO), 2016
Belarus Vital Registration - Deaths 2000 ICD9 as it appears in World Health Organization (WHO). WHO Mortality Database Version September 2016. Geneva, Switzerland: World Health Organization (WHO), 2016
Belarus Vital Registration - Deaths 2001 ICD9 as it appears in World Health Organization (WHO). WHO Mortality Database Version September 2016. Geneva, Switzerland: World Health Organization (WHO), 2016
Belarus Vital Registration - Deaths 2002 ICD10 as it appears in World Health Organization (WHO). WHO Mortality Database Version November 2015. Geneva, Switzerland: World Health Organization (WHO), 2015
Belarus Vital Registration - Deaths 2003 ICD10 as it appears in World Health Organization (WHO). WHO Mortality Database Version November 2015. Geneva, Switzerland: World Health Organization (WHO), 2015
Belarus Vital Registration - Deaths 2007 ICD10 as it appears in World Health Organization (WHO). WHO Mortality Database Version November 2015. Geneva, Switzerland: World Health Organization (WHO), 2015
Belarus Vital Registration - Deaths 2008 ICD10 as it appears in World Health Organization (WHO). WHO Mortality Database Version November 2015. Geneva, Switzerland: World Health Organization (WHO), 2015
Belarus Vital Registration - Deaths 2009 ICD10 as it appears in World Health Organization (WHO). WHO Mortality Database Version November 2015. Geneva, Switzerland: World Health Organization (WHO), 2015
Belarus Vital Registration - Deaths 2010 ICD10 as it appears in World Health Organization (WHO). WHO Mortality Database Version November 2015. Geneva, Switzerland: World Health Organization (WHO), 2015
Belarus Vital Registration - Deaths 2011 ICD10 as it appears in World Health Organization (WHO). WHO Mortality Database Version November 2015. Geneva, Switzerland: World Health Organization (WHO), 2015
Belarus Vital Registration - Deaths 2013 ICD10 as it appears in World Health Organization (WHO). WHO Mortality Database Version November 2015. Geneva, Switzerland: World Health Organization (WHO), 2015
Belarus Vital Registration - Deaths 2014 ICD10 as it appears in World Health Organization (WHO). WHO Mortality Database Version November 2015. Geneva, Switzerland: World Health Organization (WHO), 2015

Belgium Vital Registration - Deaths 1980 ICD9 as it appears in World Health Organization (WHO). WHO Mortality Database Version November 2015. Geneva, Switzerland: World Health Organization (WHO), 2015
Belgium Vital Registration - Deaths 1981 ICD9 as it appears in World Health Organization (WHO). WHO Mortality Database Version November 2015. Geneva, Switzerland: World Health Organization (WHO), 2015
Belgium Vital Registration - Deaths 1982 ICD9 as it appears in World Health Organization (WHO). WHO Mortality Database Version November 2015. Geneva, Switzerland: World Health Organization (WHO), 2015
Belgium Vital Registration - Deaths 1983 ICD9 as it appears in World Health Organization (WHO). WHO Mortality Database Version November 2015. Geneva, Switzerland: World Health Organization (WHO), 2015
Belgium Vital Registration - Deaths 1984 ICD9 as it appears in World Health Organization (WHO). WHO Mortality Database Version November 2015. Geneva, Switzerland: World Health Organization (WHO), 2015
Belgium Vital Registration - Deaths 1985 ICD9 as it appears in World Health Organization (WHO). WHO Mortality Database Version September 2016. Geneva, Switzerland: World Health Organization (WHO), 2016
Belgium Vital Registration - Deaths 1986 ICD9 as it appears in World Health Organization (WHO). WHO Mortality Database Version November 2015. Geneva, Switzerland: World Health Organization (WHO), 2015
Belgium Vital Registration - Deaths 1987 ICD9 as it appears in World Health Organization (WHO). WHO Mortality Database Version November 2015. Geneva, Switzerland: World Health Organization (WHO), 2015
Belgium Vital Registration - Deaths 1988 ICD9 as it appears in World Health Organization (WHO). WHO Mortality Database Version September 2016. Geneva, Switzerland: World Health Organization (WHO), 2016
Belgium Vital Registration - Deaths 1989 ICD9 as it appears in World Health Organization (WHO). WHO Mortality Database Version September 2016. Geneva, Switzerland: World Health Organization (WHO), 2016
Belgium Vital Registration - Deaths 1990 ICD9 as it appears in World Health Organization (WHO). WHO Mortality Database Version September 2016. Geneva, Switzerland: World Health Organization (WHO), 2016
Belgium Vital Registration - Deaths 1991 ICD9 as it appears in World Health Organization (WHO). WHO Mortality Database Version September 2016. Geneva, Switzerland: World Health Organization (WHO), 2016
Belgium Vital Registration - Deaths 1992 ICD9 as it appears in World Health Organization (WHO). WHO Mortality Database Version November 2015. Geneva, Switzerland: World Health Organization (WHO), 2015
Belgium Vital Registration - Deaths 1993 ICD9 as it appears in World Health Organization (WHO). WHO Mortality Database Version November 2015. Geneva, Switzerland: World Health Organization (WHO), 2015
Belgium Vital Registration - Deaths 1994 ICD9 as it appears in World Health Organization (WHO). WHO Mortality Database Version November 2015. Geneva, Switzerland: World Health Organization (WHO), 2015
Belgium Vital Registration - Deaths 1995 ICD9 as it appears in World Health Organization (WHO). WHO Mortality Database Version November 2015. Geneva, Switzerland: World Health Organization (WHO), 2015
Belgium Vital Registration - Deaths 1996 ICD9 as it appears in World Health Organization (WHO). WHO Mortality Database Version November 2015. Geneva, Switzerland: World Health Organization (WHO), 2015
Belgium Vital Registration - Deaths 1997 ICD9 as it appears in World Health Organization (WHO). WHO Mortality Database Version November 2015. Geneva, Switzerland: World Health Organization (WHO), 2015

Belgium Vital Registration - Deaths 1998 ICD10 as it appears in World Health Organization (WHO). WHO Mortality Database Version October 2017. Geneva, Switzerland: World Health Organization (WHO), 2017
Belgium Vital Registration - Deaths 1999 ICD10 as it appears in World Health Organization (WHO). WHO Mortality Database Version October 2017. Geneva, Switzerland: World Health Organization (WHO), 2017
Belgium Vital Registration - Deaths 2004 ICD10 as it appears in World Health Organization (WHO). WHO Mortality Database Version October 2017. Geneva, Switzerland: World Health Organization (WHO), 2017
Belgium Vital Registration - Deaths 2000 ICD10 as it appears in World Health Organization (WHO). WHO Mortality Database Version October 2017. Geneva, Switzerland: World Health Organization (WHO), 2017
Belgium Vital Registration - Deaths 2001 ICD10 as it appears in World Health Organization (WHO). WHO Mortality Database Version October 2017. Geneva, Switzerland: World Health Organization (WHO), 2017
Belgium Vital Registration - Deaths 2002 ICD10 as it appears in World Health Organization (WHO). WHO Mortality Database Version October 2017. Geneva, Switzerland: World Health Organization (WHO), 2017
Belgium Vital Registration - Deaths 2003 ICD10 as it appears in World Health Organization (WHO). WHO Mortality Database Version October 2017. Geneva, Switzerland: World Health Organization (WHO), 2017
Belgium Vital Registration - Deaths 2005 ICD10 as it appears in World Health Organization (WHO). WHO Mortality Database Version October 2017. Geneva, Switzerland: World Health Organization (WHO), 2017
Belgium Vital Registration - Deaths 2006 ICD10 as it appears in World Health Organization (WHO). WHO Mortality Database Version October 2017. Geneva, Switzerland: World Health Organization (WHO), 2017
Belgium Vital Registration - Deaths 2007 ICD10 as it appears in World Health Organization (WHO). WHO Mortality Database Version October 2017. Geneva, Switzerland: World Health Organization (WHO), 2017
Belgium Vital Registration - Deaths 2008 ICD10 as it appears in World Health Organization (WHO). WHO Mortality Database Version October 2017. Geneva, Switzerland: World Health Organization (WHO), 2017
Belgium Vital Registration - Deaths 2009 ICD10 as it appears in World Health Organization (WHO). WHO Mortality Database Version October 2017. Geneva, Switzerland: World Health Organization (WHO), 2017
Belgium Vital Registration - Deaths 2010 ICD10 as it appears in World Health Organization (WHO). WHO Mortality Database Version October 2017. Geneva, Switzerland: World Health Organization (WHO), 2017
Belgium Vital Registration - Deaths 2011 ICD10 as it appears in World Health Organization (WHO). WHO Mortality Database Version October 2017. Geneva, Switzerland: World Health Organization (WHO), 2017
Belgium Vital Registration - Deaths 2012 ICD10 as it appears in World Health Organization (WHO). WHO Mortality Database Version October 2017. Geneva, Switzerland: World Health Organization (WHO), 2017
Belgium Vital Registration - Deaths 2013 ICD10 as it appears in World Health Organization (WHO). WHO Mortality Database Version October 2017. Geneva, Switzerland: World Health Organization (WHO), 2017
Belgium Vital Registration - Deaths 2014 ICD10 as it appears in World Health Organization (WHO). WHO Mortality Database Version March 2017. Geneva, Switzerland: World Health Organization (WHO), 2017
Belgium Vital Registration - Deaths 2015 ICD10 as it appears in World Health Organization (WHO). WHO Mortality Database Version April 2018. Geneva, Switzerland: World Health Organization (WHO), 2018

Belize Vital Registration - Deaths 1980 ICD9 as it appears in World Health Organization (WHO). WHO Mortality Database Version September 2016. Geneva, Switzerland: World Health Organization (WHO), 2016
Belize Vital Registration - Deaths 1981 ICD9 as it appears in World Health Organization (WHO). WHO Mortality Database Version September 2016. Geneva, Switzerland: World Health Organization (WHO), 2016
Belize Vital Registration - Deaths 1982 ICD9 as it appears in World Health Organization (WHO). WHO Mortality Database Version September 2016. Geneva, Switzerland: World Health Organization (WHO), 2016
Belize Vital Registration - Deaths 1983 ICD9 as it appears in World Health Organization (WHO). WHO Mortality Database Version September 2016. Geneva, Switzerland: World Health Organization (WHO), 2016
Belize Vital Registration - Deaths 1984 ICD9 as it appears in World Health Organization (WHO). WHO Mortality Database Version September 2016. Geneva, Switzerland: World Health Organization (WHO), 2016
Belize Vital Registration - Deaths 1986 ICD9 as it appears in World Health Organization (WHO). WHO Mortality Database Version September 2016. Geneva, Switzerland: World Health Organization (WHO), 2016
Belize Vital Registration - Deaths 1987 ICD9 as it appears in World Health Organization (WHO). WHO Mortality Database Version September 2016. Geneva, Switzerland: World Health Organization (WHO), 2016
Belize Vital Registration - Deaths 1989 ICD9 as it appears in World Health Organization (WHO). WHO Mortality Database Version September 2016. Geneva, Switzerland: World Health Organization (WHO), 2016
Belize Vital Registration - Deaths 1997 ICD10 as it appears in World Health Organization (WHO). WHO Mortality Database Version October 2017. Geneva, Switzerland: World Health Organization (WHO), 2017
Belize Vital Registration - Deaths 1998 ICD10 as it appears in World Health Organization (WHO). WHO Mortality Database Version October 2017. Geneva, Switzerland: World Health Organization (WHO), 2017
Belize Vital Registration - Deaths 1999 ICD10 as it appears in World Health Organization (WHO). WHO Mortality Database Version October 2017. Geneva, Switzerland: World Health Organization (WHO), 2017
Belize Vital Registration - Deaths 2000 ICD10 as it appears in World Health Organization (WHO). WHO Mortality Database Version October 2017. Geneva, Switzerland: World Health Organization (WHO), 2017
Belize Vital Registration - Deaths 2001 ICD10 as it appears in World Health Organization (WHO). WHO Mortality Database Version October 2017. Geneva, Switzerland: World Health Organization (WHO), 2017
Belize Vital Registration - Deaths 2002 ICD10 as it appears in World Health Organization (WHO). WHO Mortality Database Version October 2017. Geneva, Switzerland: World Health Organization (WHO), 2017
Belize Vital Registration - Deaths 2003 ICD10 as it appears in World Health Organization (WHO). WHO Mortality Database Version October 2017. Geneva, Switzerland: World Health Organization (WHO), 2017
Belize Vital Registration - Deaths 2004 ICD10 as it appears in World Health Organization (WHO). WHO Mortality Database Version October 2017. Geneva, Switzerland: World Health Organization (WHO), 2017
Belize Vital Registration - Deaths 2005 ICD10 as it appears in World Health Organization (WHO). WHO Mortality Database Version October 2017. Geneva, Switzerland: World Health Organization (WHO), 2017
Belize Vital Registration - Deaths 1990 ICD9 as it appears in World Health Organization (WHO). WHO Mortality Database Version September 2016. Geneva, Switzerland: World Health Organization (WHO), 2016

Belize Vital Registration - Deaths 1991 ICD9 as it appears in World Health Organization (WHO). WHO Mortality Database Version September 2016. Geneva, Switzerland: World Health Organization (WHO), 2016

Belize Vital Registration - Deaths 1993 ICD9 as it appears in World Health Organization (WHO). WHO Mortality Database Version September 2016. Geneva, Switzerland: World Health Organization (WHO), 2016

Belize Vital Registration - Deaths 1994 ICD9 as it appears in World Health Organization (WHO). WHO Mortality Database Version September 2016. Geneva, Switzerland: World Health Organization (WHO), 2016

Belize Vital Registration - Deaths 1995 ICD9 as it appears in World Health Organization (WHO). WHO Mortality Database Version September 2016. Geneva, Switzerland: World Health Organization (WHO), 2016

Belize Vital Registration - Deaths 1996 ICD9 as it appears in World Health Organization (WHO). WHO Mortality Database Version September 2016. Geneva, Switzerland: World Health Organization (WHO), 2016

Belize Vital Registration - Deaths 2006 ICD10 as it appears in World Health Organization (WHO). WHO Mortality Database Version October 2017. Geneva, Switzerland: World Health Organization (WHO), 2017

Belize Vital Registration - Deaths 2007 ICD10 as it appears in World Health Organization (WHO). WHO Mortality Database Version October 2017. Geneva, Switzerland: World Health Organization (WHO), 2017

Belize Vital Registration - Deaths 2008 ICD10 as it appears in World Health Organization (WHO). WHO Mortality Database Version October 2017. Geneva, Switzerland: World Health Organization (WHO), 2017

Belize Vital Registration - Deaths 2009 ICD10 as it appears in World Health Organization (WHO). WHO Mortality Database Version October 2017. Geneva, Switzerland: World Health Organization (WHO), 2017

Belize Vital Registration - Deaths 2010 ICD10 as it appears in World Health Organization (WHO). WHO Mortality Database Version October 2017. Geneva, Switzerland: World Health Organization (WHO), 2017

Belize Vital Registration - Deaths 2011 ICD10 as it appears in World Health Organization (WHO). WHO Mortality Database Version October 2017. Geneva, Switzerland: World Health Organization (WHO), 2017

Belize Vital Registration - Deaths 2012 ICD10 as it appears in World Health Organization (WHO). WHO Mortality Database Version October 2017. Geneva, Switzerland: World Health Organization (WHO), 2017

Belize Vital Registration - Deaths 2013 ICD10 as it appears in World Health Organization (WHO). WHO Mortality Database Version October 2017. Geneva, Switzerland: World Health Organization (WHO), 2017

Belize Vital Registration - Deaths 2014 ICD10 as it appears in World Health Organization (WHO). WHO Mortality Database Version October 2017. Geneva, Switzerland: World Health Organization (WHO), 2017

Belize Vital Registration - Deaths 2015 ICD10 as it appears in World Health Organization (WHO). WHO Mortality Database Version October 2017. Geneva, Switzerland: World Health Organization (WHO), 2017

Benara SK, Singh P. Validity of causes of infant death by verbal autopsy. Indian Pediatr. 1999; 66(5): 647-50

Bermuda Vital Registration - Deaths 1980 ICD9 as it appears in World Health Organization (WHO). WHO Mortality Database Version September 2016. Geneva, Switzerland: World Health Organization (WHO), 2016

Bermuda Vital Registration - Deaths 1983 ICD9 as it appears in World Health Organization (WHO). WHO Mortality Database Version September 2016. Geneva, Switzerland: World Health Organization (WHO), 2016

Bermuda Vital Registration - Deaths 1984 ICD9 as it appears in World Health Organization (WHO). WHO Mortality Database Version September 2016. Geneva, Switzerland: World Health Organization (WHO), 2016
Bermuda Vital Registration - Deaths 1985 ICD9 as it appears in World Health Organization (WHO). WHO Mortality Database Version September 2016. Geneva, Switzerland: World Health Organization (WHO), 2016
Bermuda Vital Registration - Deaths 1986 ICD9 as it appears in World Health Organization (WHO). WHO Mortality Database Version September 2016. Geneva, Switzerland: World Health Organization (WHO), 2016
Bermuda Vital Registration - Deaths 1987 ICD9 as it appears in World Health Organization (WHO). WHO Mortality Database Version September 2016. Geneva, Switzerland: World Health Organization (WHO), 2016
Bermuda Vital Registration - Deaths 1988 ICD9 as it appears in World Health Organization (WHO). WHO Mortality Database Version September 2016. Geneva, Switzerland: World Health Organization (WHO), 2016
Bermuda Vital Registration - Deaths 1989 ICD9 as it appears in World Health Organization (WHO). WHO Mortality Database Version September 2016. Geneva, Switzerland: World Health Organization (WHO), 2016
Bermuda Vital Registration - Deaths 1990 ICD9 as it appears in World Health Organization (WHO). WHO Mortality Database Version September 2016. Geneva, Switzerland: World Health Organization (WHO), 2016
Bermuda Vital Registration - Deaths 1991 ICD9 as it appears in World Health Organization (WHO). WHO Mortality Database Version September 2016. Geneva, Switzerland: World Health Organization (WHO), 2016
Bermuda Vital Registration - Deaths 1992 ICD9 as it appears in World Health Organization (WHO). WHO Mortality Database Version September 2016. Geneva, Switzerland: World Health Organization (WHO), 2016
Bermuda Vital Registration - Deaths 1993 ICD9 as it appears in World Health Organization (WHO). WHO Mortality Database Version September 2016. Geneva, Switzerland: World Health Organization (WHO), 2016
Bermuda Vital Registration - Deaths 1994 ICD9 as it appears in World Health Organization (WHO). WHO Mortality Database Version September 2016. Geneva, Switzerland: World Health Organization (WHO), 2016
Bermuda Vital Registration - Deaths 1995 ICD9 as it appears in World Health Organization (WHO). WHO Mortality Database Version September 2016. Geneva, Switzerland: World Health Organization (WHO), 2016
Bermuda Vital Registration - Deaths 1996 ICD10 as it appears in World Health Organization (WHO). WHO Mortality Database Version October 2017. Geneva, Switzerland: World Health Organization (WHO), 2017
Bermuda Vital Registration - Deaths 1997 ICD10 as it appears in World Health Organization (WHO). WHO Mortality Database Version October 2017. Geneva, Switzerland: World Health Organization (WHO), 2017
Bermuda Vital Registration - Deaths 1998 ICD10 as it appears in World Health Organization (WHO). WHO Mortality Database Version October 2017. Geneva, Switzerland: World Health Organization (WHO), 2017
Bermuda Vital Registration - Deaths 1999 ICD10 as it appears in World Health Organization (WHO). WHO Mortality Database Version October 2017. Geneva, Switzerland: World Health Organization (WHO), 2017
Bermuda Vital Registration - Deaths 2000 ICD10 as it appears in World Health Organization (WHO). WHO Mortality Database Version October 2017. Geneva, Switzerland: World Health Organization (WHO), 2017
Bermuda Vital Registration - Deaths 2001 ICD10 as it appears in World Health Organization (WHO). WHO Mortality Database Version October 2017. Geneva, Switzerland: World Health Organization (WHO), 2017

Bermuda Vital Registration - Deaths 2002 ICD10 as it appears in World Health Organization (WHO). WHO Mortality Database Version October 2017. Geneva, Switzerland: World Health Organization (WHO), 2017

Bermuda Vital Registration - Deaths 2003 ICD10 as it appears in World Health Organization (WHO). WHO Mortality Database Version October 2017. Geneva, Switzerland: World Health Organization (WHO), 2017

Bermuda Vital Registration - Deaths 2004 ICD10 as it appears in World Health Organization (WHO). WHO Mortality Database Version October 2017. Geneva, Switzerland: World Health Organization (WHO), 2017

Bermuda Vital Registration - Deaths 2005 ICD10 as it appears in World Health Organization (WHO). WHO Mortality Database Version October 2017. Geneva, Switzerland: World Health Organization (WHO), 2017

Bermuda Vital Registration - Deaths 2006 ICD10 as it appears in World Health Organization (WHO). WHO Mortality Database Version October 2017. Geneva, Switzerland: World Health Organization (WHO), 2017

Bermuda Vital Registration - Deaths 2007 ICD10 as it appears in World Health Organization (WHO). WHO Mortality Database Version October 2017. Geneva, Switzerland: World Health Organization (WHO), 2017

Bermuda Vital Registration - Deaths 2008 ICD10 as it appears in World Health Organization (WHO). WHO Mortality Database Version October 2017. Geneva, Switzerland: World Health Organization (WHO), 2017

Bermuda Vital Registration - Deaths 2009 ICD10 as it appears in World Health Organization (WHO). WHO Mortality Database Version October 2017. Geneva, Switzerland: World Health Organization (WHO), 2017

Bermuda Vital Registration - Deaths 2010 ICD10 as it appears in World Health Organization (WHO). WHO Mortality Database Version October 2017. Geneva, Switzerland: World Health Organization (WHO), 2017

Bermuda Vital Registration - Deaths 2011 ICD10 as it appears in World Health Organization (WHO). WHO Mortality Database Version October 2017. Geneva, Switzerland: World Health Organization (WHO), 2017

Bermuda Vital Registration - Deaths 2012 ICD10 as it appears in World Health Organization (WHO). WHO Mortality Database Version October 2017. Geneva, Switzerland: World Health Organization (WHO), 2017

Bermuda Vital Registration - Deaths 2013 ICD10 as it appears in World Health Organization (WHO). WHO Mortality Database Version October 2017. Geneva, Switzerland: World Health Organization (WHO), 2017

Bermuda Vital Registration - Deaths 2014 ICD10 as it appears in World Health Organization (WHO). WHO Mortality Database Version October 2017. Geneva, Switzerland: World Health Organization (WHO), 2017

Bermuda Vital Registration - Deaths 2015 ICD10 as it appears in World Health Organization (WHO). WHO Mortality Database Version October 2017. Geneva, Switzerland: World Health Organization (WHO), 2017

Bhandari N, Bahl R, Taneja S, Martines J, Bhan MK. Pathways to infant mortality in urban slums of Delhi, India: implications for improving the quality of community- and hospital-based programmes. J Health Popul Nutr. 2002; 20(2): 148-55

Births and Deaths Registry (Ghana). Ghana - Accra Births and Deaths Registry - Deaths 2000-2007

Bosnia and Herzegovina Vital Registration - Deaths 1991 ICD9 as it appears in World Health Organization (WHO). WHO Mortality Database Version September 2016. Geneva, Switzerland: World Health Organization (WHO), 2016

Bosnia and Herzegovina Vital Registration - Deaths 2011 ICD10 as it appears in World Health Organization (WHO). WHO Mortality Database Version October 2017. Geneva, Switzerland: World Health Organization (WHO), 2017

Bosnia and Herzegovina Vital Registration - Deaths 2014 ICD10 as it appears in World Health Organization (WHO). WHO Mortality Database Version October 2017. Geneva, Switzerland: World Health Organization (WHO), 2017

BRAC, International Centre for Diarrhoeal Disease Research, Bangladesh (ICDDR,B). Bangladesh - Dhaka Causes of Maternal, Neonatal and Child Deaths: An Exploratory Study of Dhaka's Slum Dwellers. Dhaka, Bangladesh: International Centre for Diarrhoeal Disease Research, Bangladesh (ICDDR,B), 2009

Brunei Vital Registration - Deaths 1996 ICD10 as it appears in World Health Organization (WHO). WHO Mortality Database Version November 2015. Geneva, Switzerland: World Health Organization (WHO), 2015

Brunei Vital Registration - Deaths 1997 ICD10 as it appears in World Health Organization (WHO). WHO Mortality Database Version November 2015. Geneva, Switzerland: World Health Organization (WHO), 2015

Brunei Vital Registration - Deaths 1998 ICD10 as it appears in World Health Organization (WHO). WHO Mortality Database Version November 2015. Geneva, Switzerland: World Health Organization (WHO), 2015

Brunei Vital Registration - Deaths 1999 ICD10 as it appears in World Health Organization (WHO). WHO Mortality Database Version November 2015. Geneva, Switzerland: World Health Organization (WHO), 2015

Brunei Vital Registration - Deaths 2000 ICD10 as it appears in World Health Organization (WHO). WHO Mortality Database Version November 2015. Geneva, Switzerland: World Health Organization (WHO), 2015

Brunei Vital Registration - Deaths 2001 ICD10 as it appears in World Health Organization (WHO). WHO Mortality Database Version November 2015. Geneva, Switzerland: World Health Organization (WHO), 2015

Brunei Vital Registration - Deaths 2002 ICD10 as it appears in World Health Organization (WHO). WHO Mortality Database Version November 2015. Geneva, Switzerland: World Health Organization (WHO), 2015

Brunei Vital Registration - Deaths 2003 ICD10 as it appears in World Health Organization (WHO). WHO Mortality Database Version November 2015. Geneva, Switzerland: World Health Organization (WHO), 2015

Brunei Vital Registration - Deaths 2004 ICD10 as it appears in World Health Organization (WHO). WHO Mortality Database Version November 2015. Geneva, Switzerland: World Health Organization (WHO), 2015

Brunei Vital Registration - Deaths 2005 ICD10 as it appears in World Health Organization (WHO). WHO Mortality Database Version November 2015. Geneva, Switzerland: World Health Organization (WHO), 2015

Brunei Vital Registration - Deaths 2006 ICD10 as it appears in World Health Organization (WHO). WHO Mortality Database Version November 2015. Geneva, Switzerland: World Health Organization (WHO), 2015

Brunei Vital Registration - Deaths 2007 ICD10 as it appears in World Health Organization (WHO). WHO Mortality Database Version November 2015. Geneva, Switzerland: World Health Organization (WHO), 2015

Brunei Vital Registration - Deaths 2008 ICD10 as it appears in World Health Organization (WHO). WHO Mortality Database Version November 2015. Geneva, Switzerland: World Health Organization (WHO), 2015

Brunei Vital Registration - Deaths 2009 ICD10 as it appears in World Health Organization (WHO). WHO Mortality Database Version November 2015. Geneva, Switzerland: World Health Organization (WHO), 2015

Brunei Vital Registration - Deaths 2010 ICD10 as it appears in World Health Organization (WHO). WHO Mortality Database Version November 2015. Geneva, Switzerland: World Health Organization (WHO), 2015

Brunei Vital Registration - Deaths 2011 ICD10 as it appears in World Health Organization (WHO). WHO Mortality Database Version October 2017. Geneva, Switzerland: World Health Organization (WHO), 2017
Brunei Vital Registration - Deaths 2012 ICD10 as it appears in World Health Organization (WHO). WHO Mortality Database Version October 2017. Geneva, Switzerland: World Health Organization (WHO), 2017
Brunei Vital Registration - Deaths 2013 ICD10 as it appears in World Health Organization (WHO). WHO Mortality Database Version October 2017. Geneva, Switzerland: World Health Organization (WHO), 2017
Brunei Vital Registration - Deaths 2014 ICD10 as it appears in World Health Organization (WHO). WHO Mortality Database Version October 2017. Geneva, Switzerland: World Health Organization (WHO), 2017
Brunei Vital Registration - Deaths 2015 ICD10 as it appears in World Health Organization (WHO). WHO Mortality Database Version October 2017. Geneva, Switzerland: World Health Organization (WHO), 2017
Bulgaria Vital Registration - Deaths 1980 ICD9 as it appears in World Health Organization (WHO). WHO Mortality Database Version September 2016. Geneva, Switzerland: World Health Organization (WHO), 2016
Bulgaria Vital Registration - Deaths 1981 ICD9 as it appears in World Health Organization (WHO). WHO Mortality Database Version September 2016. Geneva, Switzerland: World Health Organization (WHO), 2016
Bulgaria Vital Registration - Deaths 1982 ICD9 as it appears in World Health Organization (WHO). WHO Mortality Database Version September 2016. Geneva, Switzerland: World Health Organization (WHO), 2016
Bulgaria Vital Registration - Deaths 1983 ICD9 as it appears in World Health Organization (WHO). WHO Mortality Database Version September 2016. Geneva, Switzerland: World Health Organization (WHO), 2016
Bulgaria Vital Registration - Deaths 1984 ICD9 as it appears in World Health Organization (WHO). WHO Mortality Database Version September 2016. Geneva, Switzerland: World Health Organization (WHO), 2016
Bulgaria Vital Registration - Deaths 1985 ICD9 as it appears in World Health Organization (WHO). WHO Mortality Database Version September 2016. Geneva, Switzerland: World Health Organization (WHO), 2016
Bulgaria Vital Registration - Deaths 1986 ICD9 as it appears in World Health Organization (WHO). WHO Mortality Database Version September 2016. Geneva, Switzerland: World Health Organization (WHO), 2016
Bulgaria Vital Registration - Deaths 1987 ICD9 as it appears in World Health Organization (WHO). WHO Mortality Database Version September 2016. Geneva, Switzerland: World Health Organization (WHO), 2016
Bulgaria Vital Registration - Deaths 1988 ICD9 as it appears in World Health Organization (WHO). WHO Mortality Database Version September 2016. Geneva, Switzerland: World Health Organization (WHO), 2016
Bulgaria Vital Registration - Deaths 1989 ICD9 as it appears in World Health Organization (WHO). WHO Mortality Database Version September 2016. Geneva, Switzerland: World Health Organization (WHO), 2016
Bulgaria Vital Registration - Deaths 1990 ICD9 as it appears in World Health Organization (WHO). WHO Mortality Database Version November 2015. Geneva, Switzerland: World Health Organization (WHO), 2015
Bulgaria Vital Registration - Deaths 1991 ICD9 as it appears in World Health Organization (WHO). WHO Mortality Database Version November 2015. Geneva, Switzerland: World Health Organization (WHO), 2015
Bulgaria Vital Registration - Deaths 1992 ICD9 as it appears in World Health Organization (WHO). WHO Mortality Database Version November 2015. Geneva, Switzerland: World Health Organization (WHO), 2015

Bulgaria Vital Registration - Deaths 1993 ICD9 as it appears in World Health Organization (WHO). WHO Mortality Database Version November 2015. Geneva, Switzerland: World Health Organization (WHO), 2015

Bulgaria Vital Registration - Deaths 1994 ICD9 as it appears in World Health Organization (WHO). WHO Mortality Database Version November 2015. Geneva, Switzerland: World Health Organization (WHO), 2015

Bulgaria Vital Registration - Deaths 1995 ICD9 as it appears in World Health Organization (WHO). WHO Mortality Database Version November 2015. Geneva, Switzerland: World Health Organization (WHO), 2015

Bulgaria Vital Registration - Deaths 1996 ICD9 as it appears in World Health Organization (WHO). WHO Mortality Database Version November 2015. Geneva, Switzerland: World Health Organization (WHO), 2015

Bulgaria Vital Registration - Deaths 1997 ICD9 as it appears in World Health Organization (WHO). WHO Mortality Database Version November 2015. Geneva, Switzerland: World Health Organization (WHO), 2015

Bulgaria Vital Registration - Deaths 1998 ICD9 as it appears in World Health Organization (WHO). WHO Mortality Database Version November 2015. Geneva, Switzerland: World Health Organization (WHO), 2015

Bulgaria Vital Registration - Deaths 1999 ICD9 as it appears in World Health Organization (WHO). WHO Mortality Database Version November 2015. Geneva, Switzerland: World Health Organization (WHO), 2015

Bulgaria Vital Registration - Deaths 2000 ICD9 as it appears in World Health Organization (WHO). WHO Mortality Database Version November 2015. Geneva, Switzerland: World Health Organization (WHO), 2015

Bulgaria Vital Registration - Deaths 2001 ICD9 as it appears in World Health Organization (WHO). WHO Mortality Database Version November 2015. Geneva, Switzerland: World Health Organization (WHO), 2015

Bulgaria Vital Registration - Deaths 2002 ICD9 as it appears in World Health Organization (WHO). WHO Mortality Database Version November 2015. Geneva, Switzerland: World Health Organization (WHO), 2015

Bulgaria Vital Registration - Deaths 2003 ICD9 as it appears in World Health Organization (WHO). WHO Mortality Database Version November 2015. Geneva, Switzerland: World Health Organization (WHO), 2015

Bulgaria Vital Registration - Deaths 2004 ICD9 as it appears in World Health Organization (WHO). WHO Mortality Database Version November 2015. Geneva, Switzerland: World Health Organization (WHO), 2015

Bulgaria Vital Registration - Deaths 2005 ICD10 as it appears in World Health Organization (WHO). WHO Mortality Database Version October 2017. Geneva, Switzerland: World Health Organization (WHO), 2017

Bulgaria Vital Registration - Deaths 2006 ICD10 as it appears in World Health Organization (WHO). WHO Mortality Database Version October 2017. Geneva, Switzerland: World Health Organization (WHO), 2017

Bulgaria Vital Registration - Deaths 2007 ICD10 as it appears in World Health Organization (WHO). WHO Mortality Database Version October 2017. Geneva, Switzerland: World Health Organization (WHO), 2017

Bulgaria Vital Registration - Deaths 2008 ICD10 as it appears in World Health Organization (WHO). WHO Mortality Database Version October 2017. Geneva, Switzerland: World Health Organization (WHO), 2017

Bulgaria Vital Registration - Deaths 2009 ICD10 as it appears in World Health Organization (WHO). WHO Mortality Database Version October 2017. Geneva, Switzerland: World Health Organization (WHO), 2017

Bulgaria Vital Registration - Deaths 2010 ICD10 as it appears in World Health Organization (WHO). WHO Mortality Database Version October 2017. Geneva, Switzerland: World Health Organization (WHO), 2017

Bulgaria Vital Registration - Deaths 2011 ICD10 as it appears in World Health Organization (WHO). WHO Mortality Database Version October 2017. Geneva, Switzerland: World Health Organization (WHO), 2017
Bulgaria Vital Registration - Deaths 2012 ICD10 as it appears in World Health Organization (WHO). WHO Mortality Database Version October 2017. Geneva, Switzerland: World Health Organization (WHO), 2017
Bulgaria Vital Registration - Deaths 2013 ICD10 as it appears in World Health Organization (WHO). WHO Mortality Database Version October 2017. Geneva, Switzerland: World Health Organization (WHO), 2017
Bulgaria Vital Registration - Deaths 2014 ICD10 as it appears in World Health Organization (WHO). WHO Mortality Database Version October 2017. Geneva, Switzerland: World Health Organization (WHO), 2017
Canada Vital Registration - Deaths 1980 ICD9 as it appears in World Health Organization (WHO). WHO Mortality Database Version September 2016. Geneva, Switzerland: World Health Organization (WHO), 2016
Canada Vital Registration - Deaths 1981 ICD9 as it appears in World Health Organization (WHO). WHO Mortality Database Version September 2016. Geneva, Switzerland: World Health Organization (WHO), 2016
Canada Vital Registration - Deaths 1982 ICD9 as it appears in World Health Organization (WHO). WHO Mortality Database Version September 2016. Geneva, Switzerland: World Health Organization (WHO), 2016
Canada Vital Registration - Deaths 1983 ICD9 as it appears in World Health Organization (WHO). WHO Mortality Database Version September 2016. Geneva, Switzerland: World Health Organization (WHO), 2016
Canada Vital Registration - Deaths 1984 ICD9 as it appears in World Health Organization (WHO). WHO Mortality Database Version September 2016. Geneva, Switzerland: World Health Organization (WHO), 2016
Canada Vital Registration - Deaths 1985 ICD9 as it appears in World Health Organization (WHO). WHO Mortality Database Version September 2016. Geneva, Switzerland: World Health Organization (WHO), 2016
Canada Vital Registration - Deaths 1986 ICD9 as it appears in World Health Organization (WHO). WHO Mortality Database Version November 2015. Geneva, Switzerland: World Health Organization (WHO), 2015
Canada Vital Registration - Deaths 1987 ICD9 as it appears in World Health Organization (WHO). WHO Mortality Database Version November 2015. Geneva, Switzerland: World Health Organization (WHO), 2015
Canada Vital Registration - Deaths 1988 ICD9 as it appears in World Health Organization (WHO). WHO Mortality Database Version November 2015. Geneva, Switzerland: World Health Organization (WHO), 2015
Canada Vital Registration - Deaths 1989 ICD9 as it appears in World Health Organization (WHO). WHO Mortality Database Version November 2015. Geneva, Switzerland: World Health Organization (WHO), 2015
Canada Vital Registration - Deaths 1990 ICD9 as it appears in World Health Organization (WHO). WHO Mortality Database Version November 2015. Geneva, Switzerland: World Health Organization (WHO), 2015
Canada Vital Registration - Deaths 1991 ICD9 as it appears in World Health Organization (WHO). WHO Mortality Database Version November 2015. Geneva, Switzerland: World Health Organization (WHO), 2015
Canada Vital Registration - Deaths 1992 ICD9 as it appears in World Health Organization (WHO). WHO Mortality Database Version November 2015. Geneva, Switzerland: World Health Organization (WHO), 2015
Canada Vital Registration - Deaths 1993 ICD9 as it appears in World Health Organization (WHO). WHO Mortality Database Version November 2015. Geneva, Switzerland: World Health Organization (WHO), 2015

Canada Vital Registration - Deaths 1994 ICD9 as it appears in World Health Organization (WHO). WHO Mortality Database Version November 2015. Geneva, Switzerland: World Health Organization (WHO), 2015
Canada Vital Registration - Deaths 1995 ICD9 as it appears in World Health Organization (WHO). WHO Mortality Database Version November 2015. Geneva, Switzerland: World Health Organization (WHO), 2015
Canada Vital Registration - Deaths 1996 ICD9 as it appears in World Health Organization (WHO). WHO Mortality Database Version November 2015. Geneva, Switzerland: World Health Organization (WHO), 2015
Canada Vital Registration - Deaths 1997 ICD9 as it appears in World Health Organization (WHO). WHO Mortality Database Version November 2015. Geneva, Switzerland: World Health Organization (WHO), 2015
Canada Vital Registration - Deaths 1998 ICD9 as it appears in World Health Organization (WHO). WHO Mortality Database Version November 2015. Geneva, Switzerland: World Health Organization (WHO), 2015
Canada Vital Registration - Deaths 1999 ICD9 as it appears in World Health Organization (WHO). WHO Mortality Database Version November 2015. Geneva, Switzerland: World Health Organization (WHO), 2015
Canada Vital Registration - Deaths 2000 ICD10 as it appears in World Health Organization (WHO). WHO Mortality Database Version October 2017. Geneva, Switzerland: World Health Organization (WHO), 2017
Canada Vital Registration - Deaths 2001 ICD10 as it appears in World Health Organization (WHO). WHO Mortality Database Version October 2017. Geneva, Switzerland: World Health Organization (WHO), 2017
Canada Vital Registration - Deaths 2002 ICD10 as it appears in World Health Organization (WHO). WHO Mortality Database Version October 2017. Geneva, Switzerland: World Health Organization (WHO), 2017
Canada Vital Registration - Deaths 2003 ICD10 as it appears in World Health Organization (WHO). WHO Mortality Database Version October 2017. Geneva, Switzerland: World Health Organization (WHO), 2017
Canada Vital Registration - Deaths 2004 ICD10 as it appears in World Health Organization (WHO). WHO Mortality Database Version October 2017. Geneva, Switzerland: World Health Organization (WHO), 2017
Canada Vital Registration - Deaths 2005 ICD10 as it appears in World Health Organization (WHO). WHO Mortality Database Version October 2017. Geneva, Switzerland: World Health Organization (WHO), 2017
Canada Vital Registration - Deaths 2006 ICD10 as it appears in World Health Organization (WHO). WHO Mortality Database Version October 2017. Geneva, Switzerland: World Health Organization (WHO), 2017
Canada Vital Registration - Deaths 2007 ICD10 as it appears in World Health Organization (WHO). WHO Mortality Database Version October 2017. Geneva, Switzerland: World Health Organization (WHO), 2017
Canada Vital Registration - Deaths 2008 ICD10 as it appears in World Health Organization (WHO). WHO Mortality Database Version October 2017. Geneva, Switzerland: World Health Organization (WHO), 2017
Canada Vital Registration - Deaths 2009 ICD10 as it appears in World Health Organization (WHO). WHO Mortality Database Version October 2017. Geneva, Switzerland: World Health Organization (WHO), 2017
Canada Vital Registration - Deaths 2010 ICD10 as it appears in World Health Organization (WHO). WHO Mortality Database Version October 2017. Geneva, Switzerland: World Health Organization (WHO), 2017
Canada Vital Registration - Deaths 2011 ICD10 as it appears in World Health Organization (WHO). WHO Mortality Database Version October 2017. Geneva, Switzerland: World Health Organization (WHO), 2017

Canada Vital Registration - Deaths 2012 ICD10 as it appears in World Health Organization (WHO). WHO Mortality Database Version March 2017. Geneva, Switzerland: World Health Organization (WHO), 2017

Canada Vital Registration - Deaths 2013 ICD10 as it appears in World Health Organization (WHO). WHO Mortality Database Version October 2017. Geneva, Switzerland: World Health Organization (WHO), 2017

Cape Verde Vital Registration - Deaths 1980 ICD8 as it appears in World Health Organization (WHO). WHO Mortality Database Version July 2012. Geneva, Switzerland: World Health Organization (WHO), 2012

Cape Verde Vital Registration - Deaths 2011 ICD10 as it appears in World Health Organization (WHO). WHO Mortality Database Version October 2017. Geneva, Switzerland: World Health Organization (WHO), 2017

Cape Verde Vital Registration - Deaths 2012 ICD10 as it appears in World Health Organization (WHO). WHO Mortality Database Version October 2017. Geneva, Switzerland: World Health Organization (WHO), 2017

Centers for Disease Control and Prevention (CDC), INDEPTH, International Vaccine Institute. Bangladesh - Abhoynagar, Mirsarai, and Kamalapur Health and Demographic Surveillance System

Central Statistical Organization (Qatar), Ministry of Public Health (Qatar). Qatar Vital Statistics Annual Bulletin 1984. Doha, Qatar: Central Statistical Organization (Qatar)

Central Statistical Organization (Qatar), Ministry of Public Health (Qatar). Qatar Vital Statistics Annual Bulletin 1985. Doha, Qatar: Central Statistical Organization (Qatar)

Central Statistics Organization (Afghanistan), ICF Macro, Indian Institute of Health Management Research (IIHMR), Ministry of Public Health (Afghanistan), World Health Organization Regional Office for the Eastern Mediterranean (EMRO-WHO). Afghanistan Special Demographic and Health Survey 2010. Fairfax, United States: ICF International

Chief Medical Office of Greenland. Greenland Vital Registration - Deaths 1995

Chief Medical Office of Greenland. Greenland Vital Registration - Deaths 1996

Chief Medical Office of Greenland. Greenland Vital Registration - Deaths 1997

Chief Medical Office of Greenland. Greenland Vital Registration - Deaths 1998

Chief Medical Office of Greenland. Greenland Vital Registration - Deaths 1999

Chief Medical Office of Greenland. Greenland Vital Registration - Deaths 2000

Chief Medical Office of Greenland. Greenland Vital Registration - Deaths 2001

Chief Medical Office of Greenland. Greenland Vital Registration - Deaths 2002

Chief Medical Office of Greenland. Greenland Vital Registration - Deaths 2003

Chief Medical Office of Greenland. Greenland Vital Registration - Deaths 2004

Chief Medical Office of Greenland. Greenland Vital Registration - Deaths 2005

Chief Medical Office of Greenland. Greenland Vital Registration - Deaths 2006

Chief Medical Office of Greenland. Greenland Vital Registration - Deaths 2007

Chief Medical Office of Greenland. Greenland Vital Registration - Deaths 2008

Chief Medical Office of Greenland. Greenland Vital Registration - Deaths 2009

Chief Medical Office of Greenland. Greenland Vital Registration - Deaths 2010

Chief Medical Office of Greenland. Greenland Vital Registration - Deaths 2011
Chief Medical Office of Greenland. Greenland Vital Registration - Deaths 2012
Chief Medical Office of Greenland. Greenland Vital Registration - Deaths 2013
Chief Medical Office of Greenland. Greenland Vital Registration - Deaths 2014
Chief Medical Office of Greenland. Greenland Vital Registration - Deaths 2015
Chile Vital Registration - Deaths 1980 ICD9 as it appears in World Health Organization (WHO). WHO Mortality Database Version September 2016. Geneva, Switzerland: World Health Organization (WHO), 2016
Chile Vital Registration - Deaths 1981 ICD9 as it appears in World Health Organization (WHO). WHO Mortality Database Version September 2016. Geneva, Switzerland: World Health Organization (WHO), 2016
Chile Vital Registration - Deaths 1982 ICD9 as it appears in World Health Organization (WHO). WHO Mortality Database Version September 2016. Geneva, Switzerland: World Health Organization (WHO), 2016
Chile Vital Registration - Deaths 1983 ICD9 as it appears in World Health Organization (WHO). WHO Mortality Database Version September 2016. Geneva, Switzerland: World Health Organization (WHO), 2016
Chile Vital Registration - Deaths 1984 ICD9 as it appears in World Health Organization (WHO). WHO Mortality Database Version September 2016. Geneva, Switzerland: World Health Organization (WHO), 2016
Chile Vital Registration - Deaths 1986 ICD9 as it appears in World Health Organization (WHO). WHO Mortality Database Version September 2016. Geneva, Switzerland: World Health Organization (WHO), 2016
Chile Vital Registration - Deaths 1987 ICD9 as it appears in World Health Organization (WHO). WHO Mortality Database Version September 2016. Geneva, Switzerland: World Health Organization (WHO), 2016
Chile Vital Registration - Deaths 1988 ICD9 as it appears in World Health Organization (WHO). WHO Mortality Database Version September 2016. Geneva, Switzerland: World Health Organization (WHO), 2016
Chile Vital Registration - Deaths 1989 ICD9 as it appears in World Health Organization (WHO). WHO Mortality Database Version September 2016. Geneva, Switzerland: World Health Organization (WHO), 2016
Chile Vital Registration - Deaths 1997 ICD10 as it appears in World Health Organization (WHO). WHO Mortality Database Version October 2017. Geneva, Switzerland: World Health Organization (WHO), 2017
Chile Vital Registration - Deaths 1998 ICD10 as it appears in World Health Organization (WHO). WHO Mortality Database Version October 2017. Geneva, Switzerland: World Health Organization (WHO), 2017
Chile Vital Registration - Deaths 1999 ICD10 as it appears in World Health Organization (WHO). WHO Mortality Database Version October 2017. Geneva, Switzerland: World Health Organization (WHO), 2017
Chile Vital Registration - Deaths 2000 ICD10 as it appears in World Health Organization (WHO). WHO Mortality Database Version October 2017. Geneva, Switzerland: World Health Organization (WHO), 2017
Chile Vital Registration - Deaths 2001 ICD10 as it appears in World Health Organization (WHO). WHO Mortality Database Version October 2017. Geneva, Switzerland: World Health Organization (WHO), 2017
Chile Vital Registration - Deaths 2002 ICD10 as it appears in World Health Organization (WHO). WHO Mortality Database Version October 2017. Geneva, Switzerland: World Health Organization (WHO), 2017

Chile Vital Registration - Deaths 2003 ICD10 as it appears in World Health Organization (WHO). WHO Mortality Database Version October 2017. Geneva, Switzerland: World Health Organization (WHO), 2017
Chile Vital Registration - Deaths 2004 ICD10 as it appears in World Health Organization (WHO). WHO Mortality Database Version October 2017. Geneva, Switzerland: World Health Organization (WHO), 2017
Chile Vital Registration - Deaths 2005 ICD10 as it appears in World Health Organization (WHO). WHO Mortality Database Version October 2017. Geneva, Switzerland: World Health Organization (WHO), 2017
Chile Vital Registration - Deaths 2006 ICD10 as it appears in World Health Organization (WHO). WHO Mortality Database Version October 2017. Geneva, Switzerland: World Health Organization (WHO), 2017
Chile Vital Registration - Deaths 2007 ICD10 as it appears in World Health Organization (WHO). WHO Mortality Database Version October 2017. Geneva, Switzerland: World Health Organization (WHO), 2017
Chile Vital Registration - Deaths 2008 ICD10 as it appears in World Health Organization (WHO). WHO Mortality Database Version October 2017. Geneva, Switzerland: World Health Organization (WHO), 2017
Chile Vital Registration - Deaths 2009 ICD10 as it appears in World Health Organization (WHO). WHO Mortality Database Version October 2017. Geneva, Switzerland: World Health Organization (WHO), 2017
Chile Vital Registration - Deaths 2010 ICD10 as it appears in World Health Organization (WHO). WHO Mortality Database Version October 2017. Geneva, Switzerland: World Health Organization (WHO), 2017
Chile Vital Registration - Deaths 2011 ICD10 as it appears in World Health Organization (WHO). WHO Mortality Database Version October 2017. Geneva, Switzerland: World Health Organization (WHO), 2017
Chile Vital Registration - Deaths 2012 ICD10 as it appears in World Health Organization (WHO). WHO Mortality Database Version October 2017. Geneva, Switzerland: World Health Organization (WHO), 2017
Chile Vital Registration - Deaths 2013 ICD10 as it appears in World Health Organization (WHO). WHO Mortality Database Version October 2017. Geneva, Switzerland: World Health Organization (WHO), 2017
Chile Vital Registration - Deaths 2014 ICD10 as it appears in World Health Organization (WHO). WHO Mortality Database Version October 2017. Geneva, Switzerland: World Health Organization (WHO), 2017
Chile Vital Registration - Deaths 2015 ICD10 as it appears in World Health Organization (WHO). WHO Mortality Database Version October 2017. Geneva, Switzerland: World Health Organization (WHO), 2017
Chinese Center for Disease Control and Prevention (CCDC). China Disease Surveillance Points 1991 - China CDC
Chinese Center for Disease Control and Prevention (CCDC). China Disease Surveillance Points 1992 - China CDC
Chinese Center for Disease Control and Prevention (CCDC). China Disease Surveillance Points 1993 - China CDC
Chinese Center for Disease Control and Prevention (CCDC). China Disease Surveillance Points 1994 - China CDC
Chinese Center for Disease Control and Prevention (CCDC). China Disease Surveillance Points 1995 - China CDC
Chinese Center for Disease Control and Prevention (CCDC). China Disease Surveillance Points 1996 - China CDC
Chinese Center for Disease Control and Prevention (CCDC). China Disease Surveillance Points 1997 - China CDC
Chinese Center for Disease Control and Prevention (CCDC). China Disease Surveillance Points 1998 - China CDC
Chinese Center for Disease Control and Prevention (CCDC). China Disease Surveillance Points 1999 - China CDC
Chinese Center for Disease Control and Prevention (CCDC). China Disease Surveillance Points 2000 - China CDC

Chinese Center for Disease Control and Prevention (CCDC). China Disease Surveillance Points 2001 - China CDC
Chinese Center for Disease Control and Prevention (CCDC). China Disease Surveillance Points 2002 - China CDC
Chinese Center for Disease Control and Prevention (CCDC). China Disease Surveillance Points 2004 - China CDC
Chinese Center for Disease Control and Prevention (CCDC). China Disease Surveillance Points 2005 - China CDC
Chinese Center for Disease Control and Prevention (CCDC). China Disease Surveillance Points 2006 - China CDC
Chinese Center for Disease Control and Prevention (CCDC). China Disease Surveillance Points 2007 - China CDC
Chinese Center for Disease Control and Prevention (CCDC). China Disease Surveillance Points 2013 - China CDC
Chinese Center for Disease Control and Prevention (CCDC). China Disease Surveillance Points 2014 - China CDC
Chinese Center for Disease Control and Prevention (CCDC). China Disease Surveillance Points 2015 - China CDC
Chinese Center for Disease Control and Prevention (CCDC). China Disease Surveillance Points 2016 - China CDC
Chinese Center for Disease Control and Prevention (CCDC). China Disease Surveillance Points and Death Registration 2008 - China CDC
Chinese Center for Disease Control and Prevention (CCDC). China Disease Surveillance Points and Death Registration 2009 - China CDC
Chinese Center for Disease Control and Prevention (CCDC). China Disease Surveillance Points and Death Registration 2010 - China CDC
Chinese Center for Disease Control and Prevention (CCDC). China Disease Surveillance Points and Death Registration 2011 - China CDC
Chinese Center for Disease Control and Prevention (CCDC). China Disease Surveillance Points and Death Registration 2012 - China CDC
Colombia Vital Registration - Deaths 1997 ICD10 as it appears in World Health Organization (WHO). WHO Mortality Database Version October 2017. Geneva, Switzerland: World Health Organization (WHO), 2017
Colombia Vital Registration - Deaths 1998 ICD10 as it appears in World Health Organization (WHO). WHO Mortality Database Version October 2017. Geneva, Switzerland: World Health Organization (WHO), 2017
Colombia Vital Registration - Deaths 1999 ICD10 as it appears in World Health Organization (WHO). WHO Mortality Database Version October 2017. Geneva, Switzerland: World Health Organization (WHO), 2017
Colombia Vital Registration - Deaths 2000 ICD10 as it appears in World Health Organization (WHO). WHO Mortality Database Version October 2017. Geneva, Switzerland: World Health Organization (WHO), 2017
Colombia Vital Registration - Deaths 2001 ICD10 as it appears in World Health Organization (WHO). WHO Mortality Database Version October 2017. Geneva, Switzerland: World Health Organization (WHO), 2017
Colombia Vital Registration - Deaths 2002 ICD10 as it appears in World Health Organization (WHO). WHO Mortality Database Version October 2017. Geneva, Switzerland: World Health Organization (WHO), 2017
Colombia Vital Registration - Deaths 2003 ICD10 as it appears in World Health Organization (WHO). WHO Mortality Database Version April 2018. Geneva, Switzerland: World Health Organization (WHO), 2018
Colombia Vital Registration - Deaths 2004 ICD10 as it appears in World Health Organization (WHO). WHO Mortality Database Version October 2017. Geneva, Switzerland: World Health Organization (WHO), 2017
Colombia Vital Registration - Deaths 2005 ICD10 as it appears in World Health Organization (WHO). WHO Mortality Database Version October 2017. Geneva, Switzerland: World Health Organization (WHO), 2017
Colombia Vital Registration - Deaths 2006 ICD10 as it appears in World Health Organization (WHO). WHO Mortality Database Version October 2017. Geneva, Switzerland: World Health Organization (WHO), 2017

Colombia Vital Registration - Deaths 2007 ICD10 as it appears in World Health Organization (WHO). WHO Mortality Database Version October 2017. Geneva, Switzerland: World Health Organization (WHO), 2017
Colombia Vital Registration - Deaths 2008 ICD10 as it appears in World Health Organization (WHO). WHO Mortality Database Version October 2017. Geneva, Switzerland: World Health Organization (WHO), 2017
Colombia Vital Registration - Deaths 2009 ICD10 as it appears in World Health Organization (WHO). WHO Mortality Database Version October 2017. Geneva, Switzerland: World Health Organization (WHO), 2017
Colombia Vital Registration - Deaths 2010 ICD10 as it appears in World Health Organization (WHO). WHO Mortality Database Version October 2017. Geneva, Switzerland: World Health Organization (WHO), 2017
Colombia Vital Registration - Deaths 2011 ICD10 as it appears in World Health Organization (WHO). WHO Mortality Database Version October 2017. Geneva, Switzerland: World Health Organization (WHO), 2017
Colombia Vital Registration - Deaths 2012 ICD10 as it appears in World Health Organization (WHO). WHO Mortality Database Version October 2017. Geneva, Switzerland: World Health Organization (WHO), 2017
Colombia Vital Registration - Deaths 2013 ICD10 as it appears in World Health Organization (WHO). WHO Mortality Database Version October 2017. Geneva, Switzerland: World Health Organization (WHO), 2017
Colombia Vital Registration - Deaths 2014 ICD10 as it appears in World Health Organization (WHO). WHO Mortality Database Version April 2018. Geneva, Switzerland: World Health Organization (WHO), 2018
Colombia Vital Registration - Deaths 2015 ICD10 as it appears in World Health Organization (WHO). WHO Mortality Database Version April 2018. Geneva, Switzerland: World Health Organization (WHO), 2018
Costa Rica Vital Registration - Deaths 1980 ICD9 as it appears in World Health Organization (WHO). WHO Mortality Database Version September 2016. Geneva, Switzerland: World Health Organization (WHO), 2016
Costa Rica Vital Registration - Deaths 1981 ICD9 as it appears in World Health Organization (WHO). WHO Mortality Database Version September 2016. Geneva, Switzerland: World Health Organization (WHO), 2016
Costa Rica Vital Registration - Deaths 1982 ICD9 as it appears in World Health Organization (WHO). WHO Mortality Database Version September 2016. Geneva, Switzerland: World Health Organization (WHO), 2016
Costa Rica Vital Registration - Deaths 1983 ICD9 as it appears in World Health Organization (WHO). WHO Mortality Database Version September 2016. Geneva, Switzerland: World Health Organization (WHO), 2016
Costa Rica Vital Registration - Deaths 1984 ICD9 as it appears in World Health Organization (WHO). WHO Mortality Database Version September 2016. Geneva, Switzerland: World Health Organization (WHO), 2016
Costa Rica Vital Registration - Deaths 1985 ICD9 as it appears in World Health Organization (WHO). WHO Mortality Database Version September 2016. Geneva, Switzerland: World Health Organization (WHO), 2016
Costa Rica Vital Registration - Deaths 1986 ICD9 as it appears in World Health Organization (WHO). WHO Mortality Database Version September 2016. Geneva, Switzerland: World Health Organization (WHO), 2016
Costa Rica Vital Registration - Deaths 1987 ICD9 as it appears in World Health Organization (WHO). WHO Mortality Database Version September 2016. Geneva, Switzerland: World Health Organization (WHO), 2016
Costa Rica Vital Registration - Deaths 1988 ICD9 as it appears in World Health Organization (WHO). WHO Mortality Database Version September 2016. Geneva, Switzerland: World Health Organization (WHO), 2016

Costa Rica Vital Registration - Deaths 1989 ICD9 as it appears in World Health Organization (WHO). WHO Mortality Database Version September 2016. Geneva, Switzerland: World Health Organization (WHO), 2016
Costa Rica Vital Registration - Deaths 1990 ICD9 as it appears in World Health Organization (WHO). WHO Mortality Database Version September 2016. Geneva, Switzerland: World Health Organization (WHO), 2016
Costa Rica Vital Registration - Deaths 1991 ICD9 as it appears in World Health Organization (WHO). WHO Mortality Database Version September 2016. Geneva, Switzerland: World Health Organization (WHO), 2016
Costa Rica Vital Registration - Deaths 1992 ICD9 as it appears in World Health Organization (WHO). WHO Mortality Database Version September 2016. Geneva, Switzerland: World Health Organization (WHO), 2016
Costa Rica Vital Registration - Deaths 1993 ICD9 as it appears in World Health Organization (WHO). WHO Mortality Database Version September 2016. Geneva, Switzerland: World Health Organization (WHO), 2016
Costa Rica Vital Registration - Deaths 1994 ICD9 as it appears in World Health Organization (WHO). WHO Mortality Database Version September 2016. Geneva, Switzerland: World Health Organization (WHO), 2016
Costa Rica Vital Registration - Deaths 1995 ICD9 as it appears in World Health Organization (WHO). WHO Mortality Database Version September 2016. Geneva, Switzerland: World Health Organization (WHO), 2016
Costa Rica Vital Registration - Deaths 1996 ICD9 as it appears in World Health Organization (WHO). WHO Mortality Database Version September 2016. Geneva, Switzerland: World Health Organization (WHO), 2016
Costa Rica Vital Registration - Deaths 1997 ICD10 as it appears in World Health Organization (WHO). WHO Mortality Database Version October 2017. Geneva, Switzerland: World Health Organization (WHO), 2017
Costa Rica Vital Registration - Deaths 1998 ICD10 as it appears in World Health Organization (WHO). WHO Mortality Database Version October 2017. Geneva, Switzerland: World Health Organization (WHO), 2017
Costa Rica Vital Registration - Deaths 1999 ICD10 as it appears in World Health Organization (WHO). WHO Mortality Database Version October 2017. Geneva, Switzerland: World Health Organization (WHO), 2017
Costa Rica Vital Registration - Deaths 2000 ICD10 as it appears in World Health Organization (WHO). WHO Mortality Database Version October 2017. Geneva, Switzerland: World Health Organization (WHO), 2017
Costa Rica Vital Registration - Deaths 2001 ICD10 as it appears in World Health Organization (WHO). WHO Mortality Database Version October 2017. Geneva, Switzerland: World Health Organization (WHO), 2017
Costa Rica Vital Registration - Deaths 2002 ICD10 as it appears in World Health Organization (WHO). WHO Mortality Database Version October 2017. Geneva, Switzerland: World Health Organization (WHO), 2017
Costa Rica Vital Registration - Deaths 2003 ICD10 as it appears in World Health Organization (WHO). WHO Mortality Database Version October 2017. Geneva, Switzerland: World Health Organization (WHO), 2017
Costa Rica Vital Registration - Deaths 2004 ICD10 as it appears in World Health Organization (WHO). WHO Mortality Database Version October 2017. Geneva, Switzerland: World Health Organization (WHO), 2017
Costa Rica Vital Registration - Deaths 2005 ICD10 as it appears in World Health Organization (WHO). WHO Mortality Database Version October 2017. Geneva, Switzerland: World Health Organization (WHO), 2017
Costa Rica Vital Registration - Deaths 2006 ICD10 as it appears in World Health Organization (WHO). WHO Mortality Database Version October 2017. Geneva, Switzerland: World Health Organization (WHO), 2017

Costa Rica Vital Registration - Deaths 2007 ICD10 as it appears in World Health Organization (WHO). WHO Mortality Database Version October 2017. Geneva, Switzerland: World Health Organization (WHO), 2017
Costa Rica Vital Registration - Deaths 2008 ICD10 as it appears in World Health Organization (WHO). WHO Mortality Database Version October 2017. Geneva, Switzerland: World Health Organization (WHO), 2017
Costa Rica Vital Registration - Deaths 2009 ICD10 as it appears in World Health Organization (WHO). WHO Mortality Database Version October 2017. Geneva, Switzerland: World Health Organization (WHO), 2017
Costa Rica Vital Registration - Deaths 2010 ICD10 as it appears in World Health Organization (WHO). WHO Mortality Database Version October 2017. Geneva, Switzerland: World Health Organization (WHO), 2017
Costa Rica Vital Registration - Deaths 2011 ICD10 as it appears in World Health Organization (WHO). WHO Mortality Database Version October 2017. Geneva, Switzerland: World Health Organization (WHO), 2017
Costa Rica Vital Registration - Deaths 2012 ICD10 as it appears in World Health Organization (WHO). WHO Mortality Database Version October 2017. Geneva, Switzerland: World Health Organization (WHO), 2017
Costa Rica Vital Registration - Deaths 2013 ICD10 as it appears in World Health Organization (WHO). WHO Mortality Database Version October 2017. Geneva, Switzerland: World Health Organization (WHO), 2017
Costa Rica Vital Registration - Deaths 2014 ICD10 as it appears in World Health Organization (WHO). WHO Mortality Database Version October 2017. Geneva, Switzerland: World Health Organization (WHO), 2017
Croatia Vital Registration - Deaths 1991 ICD9 as it appears in World Health Organization (WHO). WHO Mortality Database Version November 2015. Geneva, Switzerland: World Health Organization (WHO), 2015
Croatia Vital Registration - Deaths 1992 ICD9 as it appears in World Health Organization (WHO). WHO Mortality Database Version November 2015. Geneva, Switzerland: World Health Organization (WHO), 2015
Croatia Vital Registration - Deaths 1993 ICD9 as it appears in World Health Organization (WHO). WHO Mortality Database Version November 2015. Geneva, Switzerland: World Health Organization (WHO), 2015
Croatia Vital Registration - Deaths 1994 ICD9 as it appears in World Health Organization (WHO). WHO Mortality Database Version November 2015. Geneva, Switzerland: World Health Organization (WHO), 2015
Croatia Vital Registration - Deaths 1995 ICD10 as it appears in World Health Organization (WHO). WHO Mortality Database Version October 2017. Geneva, Switzerland: World Health Organization (WHO), 2017
Croatia Vital Registration - Deaths 1996 ICD10 as it appears in World Health Organization (WHO). WHO Mortality Database Version October 2017. Geneva, Switzerland: World Health Organization (WHO), 2017
Croatia Vital Registration - Deaths 1997 ICD10 as it appears in World Health Organization (WHO). WHO Mortality Database Version October 2017. Geneva, Switzerland: World Health Organization (WHO), 2017
Croatia Vital Registration - Deaths 1998 ICD10 as it appears in World Health Organization (WHO). WHO Mortality Database Version October 2017. Geneva, Switzerland: World Health Organization (WHO), 2017
Croatia Vital Registration - Deaths 1999 ICD10 as it appears in World Health Organization (WHO). WHO Mortality Database Version October 2017. Geneva, Switzerland: World Health Organization (WHO), 2017
Croatia Vital Registration - Deaths 2000 ICD10 as it appears in World Health Organization (WHO). WHO Mortality Database Version October 2017. Geneva, Switzerland: World Health Organization (WHO), 2017

Croatia Vital Registration - Deaths 2001 ICD10 as it appears in World Health Organization (WHO). WHO Mortality Database Version October 2017. Geneva, Switzerland: World Health Organization (WHO), 2017
Croatia Vital Registration - Deaths 2002 ICD10 as it appears in World Health Organization (WHO). WHO Mortality Database Version October 2017. Geneva, Switzerland: World Health Organization (WHO), 2017
Croatia Vital Registration - Deaths 2003 ICD10 as it appears in World Health Organization (WHO). WHO Mortality Database Version October 2017. Geneva, Switzerland: World Health Organization (WHO), 2017
Croatia Vital Registration - Deaths 2004 ICD10 as it appears in World Health Organization (WHO). WHO Mortality Database Version October 2017. Geneva, Switzerland: World Health Organization (WHO), 2017
Croatia Vital Registration - Deaths 2005 ICD10 as it appears in World Health Organization (WHO). WHO Mortality Database Version October 2017. Geneva, Switzerland: World Health Organization (WHO), 2017
Croatia Vital Registration - Deaths 2006 ICD10 as it appears in World Health Organization (WHO). WHO Mortality Database Version October 2017. Geneva, Switzerland: World Health Organization (WHO), 2017
Croatia Vital Registration - Deaths 2007 ICD10 as it appears in World Health Organization (WHO). WHO Mortality Database Version October 2017. Geneva, Switzerland: World Health Organization (WHO), 2017
Croatia Vital Registration - Deaths 2008 ICD10 as it appears in World Health Organization (WHO). WHO Mortality Database Version October 2017. Geneva, Switzerland: World Health Organization (WHO), 2017
Croatia Vital Registration - Deaths 2009 ICD10 as it appears in World Health Organization (WHO). WHO Mortality Database Version October 2017. Geneva, Switzerland: World Health Organization (WHO), 2017
Croatia Vital Registration - Deaths 2010 ICD10 as it appears in World Health Organization (WHO). WHO Mortality Database Version October 2017. Geneva, Switzerland: World Health Organization (WHO), 2017
Croatia Vital Registration - Deaths 2011 ICD10 as it appears in World Health Organization (WHO). WHO Mortality Database Version October 2017. Geneva, Switzerland: World Health Organization (WHO), 2017
Croatia Vital Registration - Deaths 2012 ICD10 as it appears in World Health Organization (WHO). WHO Mortality Database Version October 2017. Geneva, Switzerland: World Health Organization (WHO), 2017
Croatia Vital Registration - Deaths 2013 ICD10 as it appears in World Health Organization (WHO). WHO Mortality Database Version October 2017. Geneva, Switzerland: World Health Organization (WHO), 2017
Croatia Vital Registration - Deaths 2014 ICD10 as it appears in World Health Organization (WHO). WHO Mortality Database Version October 2017. Geneva, Switzerland: World Health Organization (WHO), 2017
Croatia Vital Registration - Deaths 2015 ICD10 as it appears in World Health Organization (WHO). WHO Mortality Database Version April 2018. Geneva, Switzerland: World Health Organization (WHO), 2018
Croatia Vital Registration - Deaths 2016 ICD10 as it appears in World Health Organization (WHO). WHO Mortality Database Version April 2018. Geneva, Switzerland: World Health Organization (WHO), 2018
Cuba Vital Registration - Deaths 1980 ICD9 as it appears in World Health Organization (WHO). WHO Mortality Database Version September 2016. Geneva, Switzerland: World Health Organization (WHO), 2016
Cuba Vital Registration - Deaths 1981 ICD9 as it appears in World Health Organization (WHO). WHO Mortality Database Version September 2016. Geneva, Switzerland: World Health Organization (WHO), 2016

Cuba Vital Registration - Deaths 1982 ICD9 as it appears in World Health Organization (WHO). WHO Mortality Database Version September 2016. Geneva, Switzerland: World Health Organization (WHO), 2016
Cuba Vital Registration - Deaths 1983 ICD9 as it appears in World Health Organization (WHO). WHO Mortality Database Version September 2016. Geneva, Switzerland: World Health Organization (WHO), 2016
Cuba Vital Registration - Deaths 1984 ICD9 as it appears in World Health Organization (WHO). WHO Mortality Database Version September 2016. Geneva, Switzerland: World Health Organization (WHO), 2016
Cuba Vital Registration - Deaths 1985 ICD9 as it appears in World Health Organization (WHO). WHO Mortality Database Version September 2016. Geneva, Switzerland: World Health Organization (WHO), 2016
Cuba Vital Registration - Deaths 1986 ICD9 as it appears in World Health Organization (WHO). WHO Mortality Database Version September 2016. Geneva, Switzerland: World Health Organization (WHO), 2016
Cuba Vital Registration - Deaths 1987 ICD9 as it appears in World Health Organization (WHO). WHO Mortality Database Version September 2016. Geneva, Switzerland: World Health Organization (WHO), 2016
Cuba Vital Registration - Deaths 1988 ICD9 as it appears in World Health Organization (WHO). WHO Mortality Database Version September 2016. Geneva, Switzerland: World Health Organization (WHO), 2016
Cuba Vital Registration - Deaths 1989 ICD9 as it appears in World Health Organization (WHO). WHO Mortality Database Version September 2016. Geneva, Switzerland: World Health Organization (WHO), 2016
Cuba Vital Registration - Deaths 1990 ICD9 as it appears in World Health Organization (WHO). WHO Mortality Database Version September 2016. Geneva, Switzerland: World Health Organization (WHO), 2016
Cuba Vital Registration - Deaths 1991 ICD9 as it appears in World Health Organization (WHO). WHO Mortality Database Version September 2016. Geneva, Switzerland: World Health Organization (WHO), 2016
Cuba Vital Registration - Deaths 1992 ICD9 as it appears in World Health Organization (WHO). WHO Mortality Database Version September 2016. Geneva, Switzerland: World Health Organization (WHO), 2016
Cuba Vital Registration - Deaths 1993 ICD9 as it appears in World Health Organization (WHO). WHO Mortality Database Version September 2016. Geneva, Switzerland: World Health Organization (WHO), 2016
Cuba Vital Registration - Deaths 1994 ICD9 as it appears in World Health Organization (WHO). WHO Mortality Database Version September 2016. Geneva, Switzerland: World Health Organization (WHO), 2016
Cuba Vital Registration - Deaths 1995 ICD9 as it appears in World Health Organization (WHO). WHO Mortality Database Version September 2016. Geneva, Switzerland: World Health Organization (WHO), 2016
Cuba Vital Registration - Deaths 1996 ICD9 as it appears in World Health Organization (WHO). WHO Mortality Database Version September 2016. Geneva, Switzerland: World Health Organization (WHO), 2016
Cuba Vital Registration - Deaths 1997 ICD9 as it appears in World Health Organization (WHO). WHO Mortality Database Version November 2015. Geneva, Switzerland: World Health Organization (WHO), 2015
Cuba Vital Registration - Deaths 1998 ICD9 as it appears in World Health Organization (WHO). WHO Mortality Database Version November 2015. Geneva, Switzerland: World Health Organization (WHO), 2015
Cuba Vital Registration - Deaths 1999 ICD9 as it appears in World Health Organization (WHO). WHO Mortality Database Version November 2015. Geneva, Switzerland: World Health Organization (WHO), 2015

Cuba Vital Registration - Deaths 2000 ICD9 as it appears in World Health Organization (WHO). WHO Mortality Database Version November 2015. Geneva, Switzerland: World Health Organization (WHO), 2015
Cuba Vital Registration - Deaths 2001 ICD10 as it appears in World Health Organization (WHO). WHO Mortality Database Version October 2017. Geneva, Switzerland: World Health Organization (WHO), 2017
Cuba Vital Registration - Deaths 2002 ICD10 as it appears in World Health Organization (WHO). WHO Mortality Database Version October 2017. Geneva, Switzerland: World Health Organization (WHO), 2017
Cuba Vital Registration - Deaths 2003 ICD10 as it appears in World Health Organization (WHO). WHO Mortality Database Version October 2017. Geneva, Switzerland: World Health Organization (WHO), 2017
Cuba Vital Registration - Deaths 2004 ICD10 as it appears in World Health Organization (WHO). WHO Mortality Database Version October 2017. Geneva, Switzerland: World Health Organization (WHO), 2017
Cuba Vital Registration - Deaths 2005 ICD10 as it appears in World Health Organization (WHO). WHO Mortality Database Version October 2017. Geneva, Switzerland: World Health Organization (WHO), 2017
Cuba Vital Registration - Deaths 2006 ICD10 as it appears in World Health Organization (WHO). WHO Mortality Database Version October 2017. Geneva, Switzerland: World Health Organization (WHO), 2017
Cuba Vital Registration - Deaths 2007 ICD10 as it appears in World Health Organization (WHO). WHO Mortality Database Version October 2017. Geneva, Switzerland: World Health Organization (WHO), 2017
Cuba Vital Registration - Deaths 2008 ICD10 as it appears in World Health Organization (WHO). WHO Mortality Database Version October 2017. Geneva, Switzerland: World Health Organization (WHO), 2017
Cuba Vital Registration - Deaths 2009 ICD10 as it appears in World Health Organization (WHO). WHO Mortality Database Version October 2017. Geneva, Switzerland: World Health Organization (WHO), 2017
Cuba Vital Registration - Deaths 2010 ICD10 as it appears in World Health Organization (WHO). WHO Mortality Database Version October 2017. Geneva, Switzerland: World Health Organization (WHO), 2017
Cuba Vital Registration - Deaths 2011 ICD10 as it appears in World Health Organization (WHO). WHO Mortality Database Version October 2017. Geneva, Switzerland: World Health Organization (WHO), 2017
Cuba Vital Registration - Deaths 2012 ICD10 as it appears in World Health Organization (WHO). WHO Mortality Database Version October 2017. Geneva, Switzerland: World Health Organization (WHO), 2017
Cuba Vital Registration - Deaths 2013 ICD10 as it appears in World Health Organization (WHO). WHO Mortality Database Version October 2017. Geneva, Switzerland: World Health Organization (WHO), 2017
Cuba Vital Registration - Deaths 2014 ICD10 as it appears in World Health Organization (WHO). WHO Mortality Database Version October 2017. Geneva, Switzerland: World Health Organization (WHO), 2017
Cuba Vital Registration - Deaths 2015 ICD10 as it appears in World Health Organization (WHO). WHO Mortality Database Version October 2017. Geneva, Switzerland: World Health Organization (WHO), 2017
Cyprus Vital Registration - Deaths 2004 ICD10 as it appears in World Health Organization (WHO). WHO Mortality Database Version October 2017. Geneva, Switzerland: World Health Organization (WHO), 2017
Cyprus Vital Registration - Deaths 2005 ICD10 as it appears in World Health Organization (WHO). WHO Mortality Database Version October 2017. Geneva, Switzerland: World Health Organization (WHO), 2017

Cyprus Vital Registration - Deaths 2006 ICD10 as it appears in World Health Organization (WHO). WHO Mortality Database Version October 2017. Geneva, Switzerland: World Health Organization (WHO), 2017
Cyprus Vital Registration - Deaths 2007 ICD10 as it appears in World Health Organization (WHO). WHO Mortality Database Version October 2017. Geneva, Switzerland: World Health Organization (WHO), 2017
Cyprus Vital Registration - Deaths 2008 ICD10 as it appears in World Health Organization (WHO). WHO Mortality Database Version October 2017. Geneva, Switzerland: World Health Organization (WHO), 2017
Cyprus Vital Registration - Deaths 2009 ICD10 as it appears in World Health Organization (WHO). WHO Mortality Database Version October 2017. Geneva, Switzerland: World Health Organization (WHO), 2017
Cyprus Vital Registration - Deaths 2010 ICD10 as it appears in World Health Organization (WHO). WHO Mortality Database Version October 2017. Geneva, Switzerland: World Health Organization (WHO), 2017
Cyprus Vital Registration - Deaths 2011 ICD10 as it appears in World Health Organization (WHO). WHO Mortality Database Version October 2017. Geneva, Switzerland: World Health Organization (WHO), 2017
Cyprus Vital Registration - Deaths 2012 ICD10 as it appears in World Health Organization (WHO). WHO Mortality Database Version October 2017. Geneva, Switzerland: World Health Organization (WHO), 2017
Cyprus Vital Registration - Deaths 2013 ICD10 as it appears in World Health Organization (WHO). WHO Mortality Database Version October 2017. Geneva, Switzerland: World Health Organization (WHO), 2017
Cyprus Vital Registration - Deaths 2014 ICD10 as it appears in World Health Organization (WHO). WHO Mortality Database Version March 2017. Geneva, Switzerland: World Health Organization (WHO), 2017
Cyprus Vital Registration - Deaths 2015 ICD10 as it appears in World Health Organization (WHO). WHO Mortality Database Version October 2017. Geneva, Switzerland: World Health Organization (WHO), 2017
Cyprus Vital Registration - Deaths 2016 ICD10 as it appears in World Health Organization (WHO). WHO Mortality Database Version April 2018. Geneva, Switzerland: World Health Organization (WHO), 2018
Czech Republic Vital Registration - Deaths 1993 ICD9 as it appears in World Health Organization (WHO). WHO Mortality Database Version November 2015. Geneva, Switzerland: World Health Organization (WHO), 2015
Czech Republic Vital Registration - Deaths 1994 ICD10 as it appears in World Health Organization (WHO). WHO Mortality Database Version October 2017. Geneva, Switzerland: World Health Organization (WHO), 2017
Czech Republic Vital Registration - Deaths 1995 ICD10 as it appears in World Health Organization (WHO). WHO Mortality Database Version October 2017. Geneva, Switzerland: World Health Organization (WHO), 2017
Czech Republic Vital Registration - Deaths 1996 ICD10 as it appears in World Health Organization (WHO). WHO Mortality Database Version October 2017. Geneva, Switzerland: World Health Organization (WHO), 2017
Czech Republic Vital Registration - Deaths 1997 ICD10 as it appears in World Health Organization (WHO). WHO Mortality Database Version October 2017. Geneva, Switzerland: World Health Organization (WHO), 2017
Czech Republic Vital Registration - Deaths 1998 ICD10 as it appears in World Health Organization (WHO). WHO Mortality Database Version October 2017. Geneva, Switzerland: World Health Organization (WHO), 2017
Czech Republic Vital Registration - Deaths 1999 ICD10 as it appears in World Health Organization (WHO). WHO Mortality Database Version October 2017. Geneva, Switzerland: World Health Organization (WHO), 2017

Czech Republic Vital Registration - Deaths 2000 ICD10 as it appears in World Health Organization (WHO). WHO Mortality Database Version October 2017. Geneva, Switzerland: World Health Organization (WHO), 2017
Czech Republic Vital Registration - Deaths 2001 ICD10 as it appears in World Health Organization (WHO). WHO Mortality Database Version October 2017. Geneva, Switzerland: World Health Organization (WHO), 2017
Czech Republic Vital Registration - Deaths 2002 ICD10 as it appears in World Health Organization (WHO). WHO Mortality Database Version October 2017. Geneva, Switzerland: World Health Organization (WHO), 2017
Czech Republic Vital Registration - Deaths 2003 ICD10 as it appears in World Health Organization (WHO). WHO Mortality Database Version October 2017. Geneva, Switzerland: World Health Organization (WHO), 2017
Czech Republic Vital Registration - Deaths 2004 ICD10 as it appears in World Health Organization (WHO). WHO Mortality Database Version October 2017. Geneva, Switzerland: World Health Organization (WHO), 2017
Czech Republic Vital Registration - Deaths 2005 ICD10 as it appears in World Health Organization (WHO). WHO Mortality Database Version October 2017. Geneva, Switzerland: World Health Organization (WHO), 2017
Czech Republic Vital Registration - Deaths 2006 ICD10 as it appears in World Health Organization (WHO). WHO Mortality Database Version October 2017. Geneva, Switzerland: World Health Organization (WHO), 2017
Czech Republic Vital Registration - Deaths 2007 ICD10 as it appears in World Health Organization (WHO). WHO Mortality Database Version October 2017. Geneva, Switzerland: World Health Organization (WHO), 2017
Czech Republic Vital Registration - Deaths 2008 ICD10 as it appears in World Health Organization (WHO). WHO Mortality Database Version October 2017. Geneva, Switzerland: World Health Organization (WHO), 2017
Czech Republic Vital Registration - Deaths 2009 ICD10 as it appears in World Health Organization (WHO). WHO Mortality Database Version October 2017. Geneva, Switzerland: World Health Organization (WHO), 2017
Czech Republic Vital Registration - Deaths 2010 ICD10 as it appears in World Health Organization (WHO). WHO Mortality Database Version October 2017. Geneva, Switzerland: World Health Organization (WHO), 2017
Czech Republic Vital Registration - Deaths 2011 ICD10 as it appears in World Health Organization (WHO). WHO Mortality Database Version October 2017. Geneva, Switzerland: World Health Organization (WHO), 2017
Czech Republic Vital Registration - Deaths 2012 ICD10 as it appears in World Health Organization (WHO). WHO Mortality Database Version October 2017. Geneva, Switzerland: World Health Organization (WHO), 2017
Czech Republic Vital Registration - Deaths 2013 ICD10 as it appears in World Health Organization (WHO). WHO Mortality Database Version October 2017. Geneva, Switzerland: World Health Organization (WHO), 2017
Czech Republic Vital Registration - Deaths 2014 ICD10 as it appears in World Health Organization (WHO). WHO Mortality Database Version October 2017. Geneva, Switzerland: World Health Organization (WHO), 2017
Czech Republic Vital Registration - Deaths 2015 ICD10 as it appears in World Health Organization (WHO). WHO Mortality Database Version March 2017. Geneva, Switzerland: World Health Organization (WHO), 2017
Czech Republic Vital Registration - Deaths 2016 ICD10 as it appears in World Health Organization (WHO). WHO Mortality Database Version April 2018. Geneva, Switzerland: World Health Organization (WHO), 2018
Czechoslovakia - Czech Republic Vital Registration - Deaths 1986 ICD9 as it appears in World Health Organization (WHO). WHO Mortality Database Version November 2015. Geneva, Switzerland: World Health Organization (WHO), 2015

Czechoslovakia - Czech Republic Vital Registration - Deaths 1987 ICD9 as it appears in World Health Organization (WHO). WHO Mortality Database Version November 2015. Geneva, Switzerland: World Health Organization (WHO), 2015

Czechoslovakia - Czech Republic Vital Registration - Deaths 1988 ICD9 as it appears in World Health Organization (WHO). WHO Mortality Database Version November 2015. Geneva, Switzerland: World Health Organization (WHO), 2015

Czechoslovakia - Czech Republic Vital Registration - Deaths 1989 ICD9 as it appears in World Health Organization (WHO). WHO Mortality Database Version November 2015. Geneva, Switzerland: World Health Organization (WHO), 2015

Czechoslovakia - Czech Republic Vital Registration - Deaths 1990 ICD9 as it appears in World Health Organization (WHO). WHO Mortality Database Version November 2015. Geneva, Switzerland: World Health Organization (WHO), 2015

Czechoslovakia - Czech Republic Vital Registration - Deaths 1991 ICD9 as it appears in World Health Organization (WHO). WHO Mortality Database Version November 2015. Geneva, Switzerland: World Health Organization (WHO), 2015

Czechoslovakia - Czech Republic Vital Registration - Deaths 1992 ICD9 as it appears in World Health Organization (WHO). WHO Mortality Database Version November 2015. Geneva, Switzerland: World Health Organization (WHO), 2015

Delacollette C, Van der Stuyft P, Molima K, Delacollette-Lebrun C, Wery M. Etude de la mortalité globale et de la mortalité liée au paludisme dans le Kivu montagneux, Zaïre. Rev Epidemiol Sante Publique. 1989; 37(2): 161-6

Denmark Vital Registration - Deaths 1980 ICD8 as it appears in World Health Organization (WHO). WHO Mortality Database Version July 2012. Geneva, Switzerland: World Health Organization (WHO), 2012

Denmark Vital Registration - Deaths 1981 ICD8 as it appears in World Health Organization (WHO). WHO Mortality Database Version July 2012. Geneva, Switzerland: World Health Organization (WHO), 2012

Denmark Vital Registration - Deaths 1982 ICD8 as it appears in World Health Organization (WHO). WHO Mortality Database Version July 2012. Geneva, Switzerland: World Health Organization (WHO), 2012

Denmark Vital Registration - Deaths 1983 ICD8 as it appears in World Health Organization (WHO). WHO Mortality Database Version July 2012. Geneva, Switzerland: World Health Organization (WHO), 2012

Denmark Vital Registration - Deaths 1984 ICD8 as it appears in World Health Organization (WHO). WHO Mortality Database Version July 2012. Geneva, Switzerland: World Health Organization (WHO), 2012

Denmark Vital Registration - Deaths 1985 ICD8 as it appears in World Health Organization (WHO). WHO Mortality Database Version July 2012. Geneva, Switzerland: World Health Organization (WHO), 2012

Denmark Vital Registration - Deaths 1986 ICD8 as it appears in World Health Organization (WHO). WHO Mortality Database Version July 2012. Geneva, Switzerland: World Health Organization (WHO), 2012

Denmark Vital Registration - Deaths 1987 ICD8 as it appears in World Health Organization (WHO). WHO Mortality Database Version July 2012. Geneva, Switzerland: World Health Organization (WHO), 2012

Denmark Vital Registration - Deaths 1988 ICD8 as it appears in World Health Organization (WHO). WHO Mortality Database Version July 2012. Geneva, Switzerland: World Health Organization (WHO), 2012

Denmark Vital Registration - Deaths 1989 ICD8 as it appears in World Health Organization (WHO). WHO Mortality Database Version July 2012. Geneva, Switzerland: World Health Organization (WHO), 2012

Denmark Vital Registration - Deaths 1990 ICD8 as it appears in World Health Organization (WHO). WHO Mortality Database Version July 2012. Geneva, Switzerland: World Health Organization (WHO), 2012

Denmark Vital Registration - Deaths 1991 ICD8 as it appears in World Health Organization (WHO). WHO Mortality Database Version July 2012. Geneva, Switzerland: World Health Organization (WHO), 2012
Denmark Vital Registration - Deaths 1992 ICD8 as it appears in World Health Organization (WHO). WHO Mortality Database Version July 2012. Geneva, Switzerland: World Health Organization (WHO), 2012
Denmark Vital Registration - Deaths 1993 ICD8 as it appears in World Health Organization (WHO). WHO Mortality Database Version July 2012. Geneva, Switzerland: World Health Organization (WHO), 2012
Denmark Vital Registration - Deaths 1994 ICD10 as it appears in World Health Organization (WHO). WHO Mortality Database Version October 2017. Geneva, Switzerland: World Health Organization (WHO), 2017
Denmark Vital Registration - Deaths 1995 ICD10 as it appears in World Health Organization (WHO). WHO Mortality Database Version October 2017. Geneva, Switzerland: World Health Organization (WHO), 2017
Denmark Vital Registration - Deaths 1996 ICD10 as it appears in World Health Organization (WHO). WHO Mortality Database Version October 2017. Geneva, Switzerland: World Health Organization (WHO), 2017
Denmark Vital Registration - Deaths 1997 ICD10 as it appears in World Health Organization (WHO). WHO Mortality Database Version October 2017. Geneva, Switzerland: World Health Organization (WHO), 2017
Denmark Vital Registration - Deaths 1998 ICD10 as it appears in World Health Organization (WHO). WHO Mortality Database Version October 2017. Geneva, Switzerland: World Health Organization (WHO), 2017
Denmark Vital Registration - Deaths 1999 ICD10 as it appears in World Health Organization (WHO). WHO Mortality Database Version October 2017. Geneva, Switzerland: World Health Organization (WHO), 2017
Denmark Vital Registration - Deaths 2000 ICD10 as it appears in World Health Organization (WHO). WHO Mortality Database Version October 2017. Geneva, Switzerland: World Health Organization (WHO), 2017
Denmark Vital Registration - Deaths 2001 ICD10 as it appears in World Health Organization (WHO). WHO Mortality Database Version October 2017. Geneva, Switzerland: World Health Organization (WHO), 2017
Denmark Vital Registration - Deaths 2002 ICD10 as it appears in World Health Organization (WHO). WHO Mortality Database Version October 2017. Geneva, Switzerland: World Health Organization (WHO), 2017
Denmark Vital Registration - Deaths 2003 ICD10 as it appears in World Health Organization (WHO). WHO Mortality Database Version October 2017. Geneva, Switzerland: World Health Organization (WHO), 2017
Denmark Vital Registration - Deaths 2004 ICD10 as it appears in World Health Organization (WHO). WHO Mortality Database Version October 2017. Geneva, Switzerland: World Health Organization (WHO), 2017
Denmark Vital Registration - Deaths 2005 ICD10 as it appears in World Health Organization (WHO). WHO Mortality Database Version October 2017. Geneva, Switzerland: World Health Organization (WHO), 2017
Denmark Vital Registration - Deaths 2006 ICD10 as it appears in World Health Organization (WHO). WHO Mortality Database Version October 2017. Geneva, Switzerland: World Health Organization (WHO), 2017
Denmark Vital Registration - Deaths 2007 ICD10 as it appears in World Health Organization (WHO). WHO Mortality Database Version October 2017. Geneva, Switzerland: World Health Organization (WHO), 2017
Denmark Vital Registration - Deaths 2008 ICD10 as it appears in World Health Organization (WHO). WHO Mortality Database Version October 2017. Geneva, Switzerland: World Health Organization (WHO), 2017

Denmark Vital Registration - Deaths 2009 ICD10 as it appears in World Health Organization (WHO). WHO Mortality Database Version October 2017. Geneva, Switzerland: World Health Organization (WHO), 2017
Denmark Vital Registration - Deaths 2010 ICD10 as it appears in World Health Organization (WHO). WHO Mortality Database Version October 2017. Geneva, Switzerland: World Health Organization (WHO), 2017
Denmark Vital Registration - Deaths 2011 ICD10 as it appears in World Health Organization (WHO). WHO Mortality Database Version October 2017. Geneva, Switzerland: World Health Organization (WHO), 2017
Denmark Vital Registration - Deaths 2012 ICD10 as it appears in World Health Organization (WHO). WHO Mortality Database Version October 2017. Geneva, Switzerland: World Health Organization (WHO), 2017
Denmark Vital Registration - Deaths 2013 ICD10 as it appears in World Health Organization (WHO). WHO Mortality Database Version March 2017. Geneva, Switzerland: World Health Organization (WHO), 2017
Denmark Vital Registration - Deaths 2014 ICD10 as it appears in World Health Organization (WHO). WHO Mortality Database Version March 2017. Geneva, Switzerland: World Health Organization (WHO), 2017
Denmark Vital Registration - Deaths 2015 ICD10 as it appears in World Health Organization (WHO). WHO Mortality Database Version October 2017. Geneva, Switzerland: World Health Organization (WHO), 2017
Department of Census and Statistics (Sri Lanka). Sri Lanka Vital Statistics - Deaths 2007. Colombo, Sri Lanka: Department of Census and Statistics (Sri Lanka)
Department of Census and Statistics (Sri Lanka). Sri Lanka Vital Statistics - Deaths 2013. Colombo, Sri Lanka: Department of Census and Statistics (Sri Lanka)
Department of Health (Philippines). Philippines Vital Statistics - Deaths 1979-2000
Department of Health (Philippines). Philippines Vital Statistics - Deaths 2001-2005
Department of Health (Taiwan). Taiwan Vital Registration - Deaths 1980
Department of Health (Taiwan). Taiwan Vital Registration - Deaths 1981
Department of Health (Taiwan). Taiwan Vital Registration - Deaths 1982
Department of Health (Taiwan). Taiwan Vital Registration - Deaths 1983
Department of Health (Taiwan). Taiwan Vital Registration - Deaths 1984
Department of Health (Taiwan). Taiwan Vital Registration - Deaths 1985
Department of Health (Taiwan). Taiwan Vital Registration - Deaths 1986
Department of Health (Taiwan). Taiwan Vital Registration - Deaths 1987
Department of Health (Taiwan). Taiwan Vital Registration - Deaths 1988
Department of Health (Taiwan). Taiwan Vital Registration - Deaths 1989
Department of Health (Taiwan). Taiwan Vital Registration - Deaths 1990
Department of Health (Taiwan). Taiwan Vital Registration - Deaths 1991
Department of Health (Taiwan). Taiwan Vital Registration - Deaths 1992. Taipei City, Taiwan: Ministry of Health and Welfare (Taiwan)
Department of Health (Taiwan). Taiwan Vital Registration - Deaths 1993. Taipei City, Taiwan: Ministry of Health and Welfare (Taiwan)
Department of Health (Taiwan). Taiwan Vital Registration - Deaths 1994. Taipei City, Taiwan: Ministry of Health and Welfare (Taiwan)

Department of Health (Taiwan). Taiwan Vital Registration - Deaths 1995. Taipei City, Taiwan: Ministry of Health and Welfare (Taiwan)
Department of Health (Taiwan). Taiwan Vital Registration - Deaths 1996. Taipei City, Taiwan: Ministry of Health and Welfare (Taiwan)
Department of Health (Taiwan). Taiwan Vital Registration - Deaths 1997. Taipei City, Taiwan: Ministry of Health and Welfare (Taiwan)
Department of Health (Taiwan). Taiwan Vital Registration - Deaths 1998. Taipei City, Taiwan: Ministry of Health and Welfare (Taiwan)
Department of Health (Taiwan). Taiwan Vital Registration - Deaths 1999. Taipei City, Taiwan: Ministry of Health and Welfare (Taiwan)
Department of Health (Taiwan). Taiwan Vital Registration - Deaths 2000. Taipei City, Taiwan: Ministry of Health and Welfare (Taiwan)
Department of Health (Taiwan). Taiwan Vital Registration - Deaths 2001. Taipei City, Taiwan: Ministry of Health and Welfare (Taiwan)
Department of Health (Taiwan). Taiwan Vital Registration - Deaths 2002. Taipei City, Taiwan: Ministry of Health and Welfare (Taiwan)
Department of Health (Taiwan). Taiwan Vital Registration - Deaths 2003. Taipei City, Taiwan: Ministry of Health and Welfare (Taiwan)
Department of Health (Taiwan). Taiwan Vital Registration - Deaths 2004. Taipei City, Taiwan: Ministry of Health and Welfare (Taiwan)
Department of Health (Taiwan). Taiwan Vital Registration - Deaths 2005. Taipei City, Taiwan: Ministry of Health and Welfare (Taiwan)
Department of Health (Taiwan). Taiwan Vital Registration - Deaths 2006. Taipei City, Taiwan: Ministry of Health and Welfare (Taiwan)
Department of Health (Taiwan). Taiwan Vital Registration - Deaths 2007. Taipei City, Taiwan: Ministry of Health and Welfare (Taiwan)
Department of Health (Taiwan). Taiwan Vital Registration - Deaths 2008. Taipei City, Taiwan: Ministry of Health and Welfare (Taiwan)
Department of Health (Taiwan). Taiwan Vital Registration - Deaths 2009. Taipei City, Taiwan: Ministry of Health and Welfare (Taiwan)
Department of Health (Taiwan). Taiwan Vital Registration - Deaths 2010. Taipei City, Taiwan: Ministry of Health and Welfare (Taiwan)
Department of Health (Taiwan). Taiwan Vital Registration - Deaths 2011. Taipei City, Taiwan: Ministry of Health and Welfare (Taiwan)
Department of Health (Taiwan). Taiwan Vital Registration - Deaths 2012. Taipei City, Taiwan: Ministry of Health and Welfare (Taiwan)
Department of Home Affairs (South Africa), Statistics South Africa. South Africa Vital Registration - Causes of Death 1997-2005. Pretoria, South Africa: Statistics South Africa
Department of Home Affairs (South Africa), Statistics South Africa. South Africa Vital Registration - Causes of Death 2006. Pretoria, South Africa: Statistics South Africa
Department of Home Affairs (South Africa), Statistics South Africa. South Africa Vital Registration - Causes of Death 2007. Pretoria, South Africa: Statistics South Africa
Department of Home Affairs (South Africa), Statistics South Africa. South Africa Vital Registration - Causes of Death 2008. Pretoria, South Africa: Statistics South Africa
Department of Home Affairs (South Africa), Statistics South Africa. South Africa Vital Registration - Causes of Death 2009. Pretoria, South Africa: Statistics South Africa
Department of Home Affairs (South Africa), Statistics South Africa. South Africa Vital Registration - Causes of Death 2010. Pretoria, South Africa: Statistics South Africa
Department of Home Affairs (South Africa), Statistics South Africa. South Africa Vital Registration - Causes of Death 2011. Pretoria, South Africa: Statistics South Africa
Department of Home Affairs (South Africa), Statistics South Africa. South Africa Vital Registration - Causes of Death 2012. Pretoria, South Africa: Statistics South Africa

Department of Home Affairs (South Africa), Statistics South Africa. South Africa Vital Registration - Causes of Death 2013. Pretoria, South Africa: Statistics South Africa

Department of Home Affairs (South Africa), Statistics South Africa. South Africa Vital Registration - Causes of Death 2014. Pretoria, South Africa: Statistics South Africa

Department of Home Affairs (South Africa), Statistics South Africa. South Africa Vital Registration - Causes of Death 2015. Pretoria, South Africa: Statistics South Africa

Department of Statistics (Malaysia). Vital Statistics: Peninsular Malaysia 1980. Kuala Lumpur, Malaysia: Department of Statistics (Malaysia), 1983

Department of Statistics (Malaysia). Vital Statistics: Peninsular Malaysia 1981. Kuala Lumpur, Malaysia: Department of Statistics (Malaysia), 1983

Department of Statistics (Malaysia). Vital Statistics: Peninsular Malaysia 1982. Kuala Lumpur, Malaysia: Department of Statistics (Malaysia), 1984

Department of Statistics and Health Information, Ministry of Health (Chile), National Institute of Statistics (Chile). Chile Vital Statistics - Deaths 1990. Santiago, Chile: Department of Statistics and Health Information, Ministry of Health (Chile)

Department of Statistics and Health Information, Ministry of Health (Chile), National Institute of Statistics (Chile). Chile Vital Statistics - Deaths 1991. Santiago, Chile: Department of Statistics and Health Information, Ministry of Health (Chile)

Department of Statistics and Health Information, Ministry of Health (Chile), National Institute of Statistics (Chile). Chile Vital Statistics - Deaths 1992. Santiago, Chile: Department of Statistics and Health Information, Ministry of Health (Chile)

Department of Statistics and Health Information, Ministry of Health (Chile), National Institute of Statistics (Chile). Chile Vital Statistics - Deaths 1993. Santiago, Chile: Department of Statistics and Health Information, Ministry of Health (Chile)

Department of Statistics and Health Information, Ministry of Health (Chile), National Institute of Statistics (Chile). Chile Vital Statistics - Deaths 1994. Santiago, Chile: Department of Statistics and Health Information, Ministry of Health (Chile)

Department of Statistics and Health Information, Ministry of Health (Chile), National Institute of Statistics (Chile). Chile Vital Statistics - Deaths 1995. Santiago, Chile: Department of Statistics and Health Information, Ministry of Health (Chile)

Department of Statistics and Health Information, Ministry of Health (Chile), National Institute of Statistics (Chile). Chile Vital Statistics - Deaths 1996. Santiago, Chile: Department of Statistics and Health Information, Ministry of Health (Chile)

Deshmukh V, Lahariya C, Krishnamurthy S, Das MK, Pandey RM, Arora NK. Taken to Health Care Provider or Not, Under-Five Children Die of Preventable Causes: Findings from Cross-Sectional Survey and Social Autopsy in Rural India. Indian J Community Med. 2016; 41(2): 108–19

Directorate of Economics and Statistics and Office of Chief Registrar Births and Deaths (Delhi Territory, India). India - Delhi Medical Certification of Cause of Death Report 2013

Directorate of Economics and Statistics, Government of Karnataka, Office of the Chief Registrar of Births and Deaths, Government of Karnataka. India - Karnataka Medical Certification of Cause of Death Report 2014. Bangalore, India: Directorate of Economics and Statistics, Government of Karnataka

Directorate of Economics and Statistics, Government of Karnataka, Office of the Chief Registrar of Births and Deaths, Government of Karnataka. India - Karnataka Medical Certification of Cause of Death Report 2015. Bangalore, India: Directorate of Economics and Statistics, Government of Karnataka

Directorate of Health (Iceland). Iceland Causes of Death Register 2017

Dogra V, Khanna R, Jain A, Kumar AMV, Shewade HD, Majumdar SS. Neonatal mortality in India's rural poor: Findings of a household survey and verbal autopsy study in Rajasthan, Bihar and Odisha. J Trop Pediatr. 2015; 61(3): 210–4

Dominica Vital Registration - Deaths 1980 ICD9 as it appears in World Health Organization (WHO). WHO Mortality Database Version September 2016. Geneva, Switzerland: World Health Organization (WHO), 2016

Dominica Vital Registration - Deaths 1981 ICD9 as it appears in World Health Organization (WHO). WHO Mortality Database Version September 2016. Geneva, Switzerland: World Health Organization (WHO), 2016

Dominica Vital Registration - Deaths 1982 ICD9 as it appears in World Health Organization (WHO). WHO Mortality Database Version September 2016. Geneva, Switzerland: World Health Organization (WHO), 2016

Dominica Vital Registration - Deaths 1983 ICD9 as it appears in World Health Organization (WHO). WHO Mortality Database Version September 2016. Geneva, Switzerland: World Health Organization (WHO), 2016

Dominica Vital Registration - Deaths 1984 ICD9 as it appears in World Health Organization (WHO). WHO Mortality Database Version September 2016. Geneva, Switzerland: World Health Organization (WHO), 2016

Dominica Vital Registration - Deaths 1985 ICD9 as it appears in World Health Organization (WHO). WHO Mortality Database Version September 2016. Geneva, Switzerland: World Health Organization (WHO), 2016

Dominica Vital Registration - Deaths 1986 ICD9 as it appears in World Health Organization (WHO). WHO Mortality Database Version September 2016. Geneva, Switzerland: World Health Organization (WHO), 2016

Dominica Vital Registration - Deaths 1987 ICD9 as it appears in World Health Organization (WHO). WHO Mortality Database Version September 2016. Geneva, Switzerland: World Health Organization (WHO), 2016

Dominica Vital Registration - Deaths 1988 ICD9 as it appears in World Health Organization (WHO). WHO Mortality Database Version September 2016. Geneva, Switzerland: World Health Organization (WHO), 2016

Dominica Vital Registration - Deaths 1989 ICD9 as it appears in World Health Organization (WHO). WHO Mortality Database Version September 2016. Geneva, Switzerland: World Health Organization (WHO), 2016

Dominica Vital Registration - Deaths 1990 ICD9 as it appears in World Health Organization (WHO). WHO Mortality Database Version September 2016. Geneva, Switzerland: World Health Organization (WHO), 2016

Dominica Vital Registration - Deaths 1991 ICD9 as it appears in World Health Organization (WHO). WHO Mortality Database Version September 2016. Geneva, Switzerland: World Health Organization (WHO), 2016

Dominica Vital Registration - Deaths 1992 ICD9 as it appears in World Health Organization (WHO). WHO Mortality Database Version September 2016. Geneva, Switzerland: World Health Organization (WHO), 2016

Dominica Vital Registration - Deaths 1993 ICD9 as it appears in World Health Organization (WHO). WHO Mortality Database Version September 2016. Geneva, Switzerland: World Health Organization (WHO), 2016

Dominica Vital Registration - Deaths 1994 ICD9 as it appears in World Health Organization (WHO). WHO Mortality Database Version September 2016. Geneva, Switzerland: World Health Organization (WHO), 2016

Dominica Vital Registration - Deaths 1995 ICD9 as it appears in World Health Organization (WHO). WHO Mortality Database Version September 2016. Geneva, Switzerland: World Health Organization (WHO), 2016
Dominica Vital Registration - Deaths 1996 ICD9 as it appears in World Health Organization (WHO). WHO Mortality Database Version September 2016. Geneva, Switzerland: World Health Organization (WHO), 2016
Dominica Vital Registration - Deaths 1997 ICD9 as it appears in World Health Organization (WHO). WHO Mortality Database Version September 2016. Geneva, Switzerland: World Health Organization (WHO), 2016
Dominica Vital Registration - Deaths 1998 ICD9 as it appears in World Health Organization (WHO). WHO Mortality Database Version September 2016. Geneva, Switzerland: World Health Organization (WHO), 2016
Dominica Vital Registration - Deaths 1999 ICD9 as it appears in World Health Organization (WHO). WHO Mortality Database Version September 2016. Geneva, Switzerland: World Health Organization (WHO), 2016
Dominica Vital Registration - Deaths 2000 ICD9 as it appears in World Health Organization (WHO). WHO Mortality Database Version November 2015. Geneva, Switzerland: World Health Organization (WHO), 2015
Dominica Vital Registration - Deaths 2001 ICD10 as it appears in World Health Organization (WHO). WHO Mortality Database Version October 2017. Geneva, Switzerland: World Health Organization (WHO), 2017
Dominica Vital Registration - Deaths 2002 ICD10 as it appears in World Health Organization (WHO). WHO Mortality Database Version October 2017. Geneva, Switzerland: World Health Organization (WHO), 2017
Dominica Vital Registration - Deaths 2003 ICD10 as it appears in World Health Organization (WHO). WHO Mortality Database Version October 2017. Geneva, Switzerland: World Health Organization (WHO), 2017
Dominica Vital Registration - Deaths 2004 ICD10 as it appears in World Health Organization (WHO). WHO Mortality Database Version October 2017. Geneva, Switzerland: World Health Organization (WHO), 2017
Dominica Vital Registration - Deaths 2005 ICD10 as it appears in World Health Organization (WHO). WHO Mortality Database Version October 2017. Geneva, Switzerland: World Health Organization (WHO), 2017
Dominica Vital Registration - Deaths 2006 ICD10 as it appears in World Health Organization (WHO). WHO Mortality Database Version October 2017. Geneva, Switzerland: World Health Organization (WHO), 2017
Dominica Vital Registration - Deaths 2007 ICD10 as it appears in World Health Organization (WHO). WHO Mortality Database Version October 2017. Geneva, Switzerland: World Health Organization (WHO), 2017
Dominica Vital Registration - Deaths 2008 ICD10 as it appears in World Health Organization (WHO). WHO Mortality Database Version October 2017. Geneva, Switzerland: World Health Organization (WHO), 2017
Dominica Vital Registration - Deaths 2009 ICD10 as it appears in World Health Organization (WHO). WHO Mortality Database Version October 2017. Geneva, Switzerland: World Health Organization (WHO), 2017
Dominica Vital Registration - Deaths 2010 ICD10 as it appears in World Health Organization (WHO). WHO Mortality Database Version October 2017. Geneva, Switzerland: World Health Organization (WHO), 2017
Dominica Vital Registration - Deaths 2011 ICD10 as it appears in World Health Organization (WHO). WHO Mortality Database Version October 2017. Geneva, Switzerland: World Health Organization (WHO), 2017
Dominica Vital Registration - Deaths 2012 ICD10 as it appears in World Health Organization (WHO). WHO Mortality Database Version October 2017. Geneva, Switzerland: World Health Organization (WHO), 2017

Dominica Vital Registration - Deaths 2013 ICD10 as it appears in World Health Organization (WHO). WHO Mortality Database Version October 2017. Geneva, Switzerland: World Health Organization (WHO), 2017
Dominica Vital Registration - Deaths 2014 ICD10 as it appears in World Health Organization (WHO). WHO Mortality Database Version October 2017. Geneva, Switzerland: World Health Organization (WHO), 2017
Dominica Vital Registration - Deaths 2015 ICD10 as it appears in World Health Organization (WHO). WHO Mortality Database Version October 2017. Geneva, Switzerland: World Health Organization (WHO), 2017
Dominican Republic Vital Registration - Deaths 1980 ICD9 as it appears in World Health Organization (WHO). WHO Mortality Database Version September 2016. Geneva, Switzerland: World Health Organization (WHO), 2016
Dominican Republic Vital Registration - Deaths 1981 ICD9 as it appears in World Health Organization (WHO). WHO Mortality Database Version September 2016. Geneva, Switzerland: World Health Organization (WHO), 2016
Dominican Republic Vital Registration - Deaths 1982 ICD9 as it appears in World Health Organization (WHO). WHO Mortality Database Version September 2016. Geneva, Switzerland: World Health Organization (WHO), 2016
Dominican Republic Vital Registration - Deaths 1983 ICD9 as it appears in World Health Organization (WHO). WHO Mortality Database Version September 2016. Geneva, Switzerland: World Health Organization (WHO), 2016
Dominican Republic Vital Registration - Deaths 1984 ICD9 as it appears in World Health Organization (WHO). WHO Mortality Database Version September 2016. Geneva, Switzerland: World Health Organization (WHO), 2016
Dominican Republic Vital Registration - Deaths 1985 ICD9 as it appears in World Health Organization (WHO). WHO Mortality Database Version September 2016. Geneva, Switzerland: World Health Organization (WHO), 2016
Dominican Republic Vital Registration - Deaths 1986 ICD9 as it appears in World Health Organization (WHO). WHO Mortality Database Version September 2016. Geneva, Switzerland: World Health Organization (WHO), 2016
Dominican Republic Vital Registration - Deaths 1987 ICD9 as it appears in World Health Organization (WHO). WHO Mortality Database Version September 2016. Geneva, Switzerland: World Health Organization (WHO), 2016
Dominican Republic Vital Registration - Deaths 1988 ICD9 as it appears in World Health Organization (WHO). WHO Mortality Database Version September 2016. Geneva, Switzerland: World Health Organization (WHO), 2016
Dominican Republic Vital Registration - Deaths 1989 ICD9 as it appears in World Health Organization (WHO). WHO Mortality Database Version September 2016. Geneva, Switzerland: World Health Organization (WHO), 2016
Dominican Republic Vital Registration - Deaths 1990 ICD9 as it appears in World Health Organization (WHO). WHO Mortality Database Version November 2015. Geneva, Switzerland: World Health Organization (WHO), 2015
Dominican Republic Vital Registration - Deaths 1991 ICD9 as it appears in World Health Organization (WHO). WHO Mortality Database Version September 2016. Geneva, Switzerland: World Health Organization (WHO), 2016
Dominican Republic Vital Registration - Deaths 1992 ICD9 as it appears in World Health Organization (WHO). WHO Mortality Database Version September 2016. Geneva, Switzerland: World Health Organization (WHO), 2016
Dominican Republic Vital Registration - Deaths 1994 ICD9 as it appears in World Health Organization (WHO). WHO Mortality Database Version September 2016. Geneva, Switzerland: World Health Organization (WHO), 2016
Dominican Republic Vital Registration - Deaths 1995 ICD9 as it appears in World Health Organization (WHO). WHO Mortality Database Version September 2016. Geneva, Switzerland: World Health Organization (WHO), 2016

Dominican Republic Vital Registration - Deaths 1996 ICD10 as it appears in World Health Organization (WHO). WHO Mortality Database Version October 2017. Geneva, Switzerland: World Health Organization (WHO), 2017
Dominican Republic Vital Registration - Deaths 1997 ICD10 as it appears in World Health Organization (WHO). WHO Mortality Database Version October 2017. Geneva, Switzerland: World Health Organization (WHO), 2017
Dominican Republic Vital Registration - Deaths 1998 ICD10 as it appears in World Health Organization (WHO). WHO Mortality Database Version October 2017. Geneva, Switzerland: World Health Organization (WHO), 2017
Dominican Republic Vital Registration - Deaths 1999 ICD10 as it appears in World Health Organization (WHO). WHO Mortality Database Version October 2017. Geneva, Switzerland: World Health Organization (WHO), 2017
Dominican Republic Vital Registration - Deaths 2000 ICD10 as it appears in World Health Organization (WHO). WHO Mortality Database Version October 2017. Geneva, Switzerland: World Health Organization (WHO), 2017
Dominican Republic Vital Registration - Deaths 2001 ICD10 as it appears in World Health Organization (WHO). WHO Mortality Database Version October 2017. Geneva, Switzerland: World Health Organization (WHO), 2017
Dominican Republic Vital Registration - Deaths 2003 ICD10 as it appears in World Health Organization (WHO). WHO Mortality Database Version October 2017. Geneva, Switzerland: World Health Organization (WHO), 2017
Dominican Republic Vital Registration - Deaths 2004 ICD10 as it appears in World Health Organization (WHO). WHO Mortality Database Version October 2017. Geneva, Switzerland: World Health Organization (WHO), 2017
Dominican Republic Vital Registration - Deaths 2005 ICD10 as it appears in World Health Organization (WHO). WHO Mortality Database Version October 2017. Geneva, Switzerland: World Health Organization (WHO), 2017
Dominican Republic Vital Registration - Deaths 2006 ICD10 as it appears in World Health Organization (WHO). WHO Mortality Database Version October 2017. Geneva, Switzerland: World Health Organization (WHO), 2017
Dominican Republic Vital Registration - Deaths 2007 ICD10 as it appears in World Health Organization (WHO). WHO Mortality Database Version October 2017. Geneva, Switzerland: World Health Organization (WHO), 2017
Dominican Republic Vital Registration - Deaths 2008 ICD10 as it appears in World Health Organization (WHO). WHO Mortality Database Version October 2017. Geneva, Switzerland: World Health Organization (WHO), 2017
Dominican Republic Vital Registration - Deaths 2009 ICD10 as it appears in World Health Organization (WHO). WHO Mortality Database Version October 2017. Geneva, Switzerland: World Health Organization (WHO), 2017
Dominican Republic Vital Registration - Deaths 2010 ICD10 as it appears in World Health Organization (WHO). WHO Mortality Database Version October 2017. Geneva, Switzerland: World Health Organization (WHO), 2017
Dominican Republic Vital Registration - Deaths 2011 ICD10 as it appears in World Health Organization (WHO). WHO Mortality Database Version October 2017. Geneva, Switzerland: World Health Organization (WHO), 2017
Dominican Republic Vital Registration - Deaths 2012 ICD10 as it appears in World Health Organization (WHO). WHO Mortality Database Version October 2017. Geneva, Switzerland: World Health Organization (WHO), 2017
Dominican Republic Vital Registration - Deaths 2013 ICD10 as it appears in World Health Organization (WHO). WHO Mortality Database Version October 2017. Geneva, Switzerland: World Health Organization (WHO), 2017
Ecuador Vital Registration - Deaths 1980 ICD9 as it appears in World Health Organization (WHO). WHO Mortality Database Version September 2016. Geneva, Switzerland: World Health Organization (WHO), 2016

Ecuador Vital Registration - Deaths 1981 ICD9 as it appears in World Health Organization (WHO). WHO Mortality Database Version September 2016. Geneva, Switzerland: World Health Organization (WHO), 2016
Ecuador Vital Registration - Deaths 1982 ICD9 as it appears in World Health Organization (WHO). WHO Mortality Database Version September 2016. Geneva, Switzerland: World Health Organization (WHO), 2016
Ecuador Vital Registration - Deaths 1983 ICD9 as it appears in World Health Organization (WHO). WHO Mortality Database Version September 2016. Geneva, Switzerland: World Health Organization (WHO), 2016
Ecuador Vital Registration - Deaths 1984 ICD9 as it appears in World Health Organization (WHO). WHO Mortality Database Version September 2016. Geneva, Switzerland: World Health Organization (WHO), 2016
Ecuador Vital Registration - Deaths 1985 ICD9 as it appears in World Health Organization (WHO). WHO Mortality Database Version September 2016. Geneva, Switzerland: World Health Organization (WHO), 2016
Ecuador Vital Registration - Deaths 1986 ICD9 as it appears in World Health Organization (WHO). WHO Mortality Database Version September 2016. Geneva, Switzerland: World Health Organization (WHO), 2016
Ecuador Vital Registration - Deaths 1988 ICD9 as it appears in World Health Organization (WHO). WHO Mortality Database Version September 2016. Geneva, Switzerland: World Health Organization (WHO), 2016
Ecuador Vital Registration - Deaths 1989 ICD9 as it appears in World Health Organization (WHO). WHO Mortality Database Version September 2016. Geneva, Switzerland: World Health Organization (WHO), 2016
Ecuador Vital Registration - Deaths 1990 ICD9 as it appears in World Health Organization (WHO). WHO Mortality Database Version September 2016. Geneva, Switzerland: World Health Organization (WHO), 2016
Ecuador Vital Registration - Deaths 1991 ICD9 as it appears in World Health Organization (WHO). WHO Mortality Database Version September 2016. Geneva, Switzerland: World Health Organization (WHO), 2016
Ecuador Vital Registration - Deaths 1992 ICD9 as it appears in World Health Organization (WHO). WHO Mortality Database Version September 2016. Geneva, Switzerland: World Health Organization (WHO), 2016
Ecuador Vital Registration - Deaths 1993 ICD9 as it appears in World Health Organization (WHO). WHO Mortality Database Version September 2016. Geneva, Switzerland: World Health Organization (WHO), 2016
Ecuador Vital Registration - Deaths 1994 ICD9 as it appears in World Health Organization (WHO). WHO Mortality Database Version September 2016. Geneva, Switzerland: World Health Organization (WHO), 2016
Ecuador Vital Registration - Deaths 1995 ICD9 as it appears in World Health Organization (WHO). WHO Mortality Database Version September 2016. Geneva, Switzerland: World Health Organization (WHO), 2016
Ecuador Vital Registration - Deaths 1996 ICD9 as it appears in World Health Organization (WHO). WHO Mortality Database Version November 2015. Geneva, Switzerland: World Health Organization (WHO), 2015
Ecuador Vital Registration - Deaths 1997 ICD10 as it appears in World Health Organization (WHO). WHO Mortality Database Version October 2017. Geneva, Switzerland: World Health Organization (WHO), 2017
Ecuador Vital Registration - Deaths 1998 ICD10 as it appears in World Health Organization (WHO). WHO Mortality Database Version October 2017. Geneva, Switzerland: World Health Organization (WHO), 2017
Ecuador Vital Registration - Deaths 1999 ICD10 as it appears in World Health Organization (WHO). WHO Mortality Database Version October 2017. Geneva, Switzerland: World Health Organization (WHO), 2017

Ecuador Vital Registration - Deaths 2000 ICD10 as it appears in World Health Organization (WHO). WHO Mortality Database Version October 2017. Geneva, Switzerland: World Health Organization (WHO), 2017
Ecuador Vital Registration - Deaths 2001 ICD10 as it appears in World Health Organization (WHO). WHO Mortality Database Version October 2017. Geneva, Switzerland: World Health Organization (WHO), 2017
Ecuador Vital Registration - Deaths 2002 ICD10 as it appears in World Health Organization (WHO). WHO Mortality Database Version October 2017. Geneva, Switzerland: World Health Organization (WHO), 2017
Ecuador Vital Registration - Deaths 2003 ICD10 as it appears in World Health Organization (WHO). WHO Mortality Database Version October 2017. Geneva, Switzerland: World Health Organization (WHO), 2017
Ecuador Vital Registration - Deaths 2004 ICD10 as it appears in World Health Organization (WHO). WHO Mortality Database Version October 2017. Geneva, Switzerland: World Health Organization (WHO), 2017
Ecuador Vital Registration - Deaths 2005 ICD10 as it appears in World Health Organization (WHO). WHO Mortality Database Version October 2017. Geneva, Switzerland: World Health Organization (WHO), 2017
Ecuador Vital Registration - Deaths 2006 ICD10 as it appears in World Health Organization (WHO). WHO Mortality Database Version October 2017. Geneva, Switzerland: World Health Organization (WHO), 2017
Ecuador Vital Registration - Deaths 1987 ICD9 as it appears in World Health Organization (WHO). WHO Mortality Database Version September 2016. Geneva, Switzerland: World Health Organization (WHO), 2016
Ecuador Vital Registration - Deaths 2007 ICD10 as it appears in World Health Organization (WHO). WHO Mortality Database Version October 2017. Geneva, Switzerland: World Health Organization (WHO), 2017
Ecuador Vital Registration - Deaths 2008 ICD10 as it appears in World Health Organization (WHO). WHO Mortality Database Version October 2017. Geneva, Switzerland: World Health Organization (WHO), 2017
Ecuador Vital Registration - Deaths 2009 ICD10 as it appears in World Health Organization (WHO). WHO Mortality Database Version October 2017. Geneva, Switzerland: World Health Organization (WHO), 2017
Ecuador Vital Registration - Deaths 2010 ICD10 as it appears in World Health Organization (WHO). WHO Mortality Database Version October 2017. Geneva, Switzerland: World Health Organization (WHO), 2017
Ecuador Vital Registration - Deaths 2011 ICD10 as it appears in World Health Organization (WHO). WHO Mortality Database Version October 2017. Geneva, Switzerland: World Health Organization (WHO), 2017
Ecuador Vital Registration - Deaths 2012 ICD10 as it appears in World Health Organization (WHO). WHO Mortality Database Version October 2017. Geneva, Switzerland: World Health Organization (WHO), 2017
Ecuador Vital Registration - Deaths 2013 ICD10 as it appears in World Health Organization (WHO). WHO Mortality Database Version October 2017. Geneva, Switzerland: World Health Organization (WHO), 2017
Ecuador Vital Registration - Deaths 2014 ICD10 as it appears in World Health Organization (WHO). WHO Mortality Database Version October 2017. Geneva, Switzerland: World Health Organization (WHO), 2017
Ecuador Vital Registration - Deaths 2015 ICD10 as it appears in World Health Organization (WHO). WHO Mortality Database Version October 2017. Geneva, Switzerland: World Health Organization (WHO), 2017
Edmond KM, Kirkwood BR, Amenga-Etego S, Owusu-Agyei S, Hurt LS. Effect of early infant feeding practices on infection-specific neonatal mortality: an investigation of the causal links with observational data from rural Ghana. Am J Clin Nutr. 2007; 86(4): 1126-31

Edmond KM, Quigley MA, Zandoh C, Danso S, Hurt C, Owusu Agyei S, Kirkwood BR. Aetiology of stillbirths and neonatal deaths in rural Ghana: implications for health programming in developing countries. Paediatr Perinat Epidemiol. 2008; 22(5): 430-7

Eduardo Mondlane University (Mozambique), Manhica Health Research Center (CISM), Ministry of Health (Mozambique). Mozambique Main Causes of Reported Death Study 2001

Egypt Vital Registration - Deaths 1991 ICD9 as it appears in World Health Organization (WHO). WHO Mortality Database Version September 2016. Geneva, Switzerland: World Health Organization (WHO), 2016

El Salvador Vital Registration - Deaths 1981 ICD9 as it appears in World Health Organization (WHO). WHO Mortality Database Version September 2016. Geneva, Switzerland: World Health Organization (WHO), 2016

El Salvador Vital Registration - Deaths 1982 ICD9 as it appears in World Health Organization (WHO). WHO Mortality Database Version September 2016. Geneva, Switzerland: World Health Organization (WHO), 2016

El Salvador Vital Registration - Deaths 1983 ICD9 as it appears in World Health Organization (WHO). WHO Mortality Database Version September 2016. Geneva, Switzerland: World Health Organization (WHO), 2016

El Salvador Vital Registration - Deaths 1984 ICD9 as it appears in World Health Organization (WHO). WHO Mortality Database Version September 2016. Geneva, Switzerland: World Health Organization (WHO), 2016

El Salvador Vital Registration - Deaths 1990 ICD9 as it appears in World Health Organization (WHO). WHO Mortality Database Version September 2016. Geneva, Switzerland: World Health Organization (WHO), 2016

El Salvador Vital Registration - Deaths 1991 ICD9 as it appears in World Health Organization (WHO). WHO Mortality Database Version September 2016. Geneva, Switzerland: World Health Organization (WHO), 2016

El Salvador Vital Registration - Deaths 1992 ICD9 as it appears in World Health Organization (WHO). WHO Mortality Database Version September 2016. Geneva, Switzerland: World Health Organization (WHO), 2016

El Salvador Vital Registration - Deaths 1993 ICD9 as it appears in World Health Organization (WHO). WHO Mortality Database Version September 2016. Geneva, Switzerland: World Health Organization (WHO), 2016

El Salvador Vital Registration - Deaths 1995 ICD9 as it appears in World Health Organization (WHO). WHO Mortality Database Version September 2016. Geneva, Switzerland: World Health Organization (WHO), 2016

El Salvador Vital Registration - Deaths 1996 ICD9 as it appears in World Health Organization (WHO). WHO Mortality Database Version September 2016. Geneva, Switzerland: World Health Organization (WHO), 2016

El Salvador Vital Registration - Deaths 1997 ICD10 as it appears in World Health Organization (WHO). WHO Mortality Database Version October 2017. Geneva, Switzerland: World Health Organization (WHO), 2017

El Salvador Vital Registration - Deaths 1998 ICD10 as it appears in World Health Organization (WHO). WHO Mortality Database Version October 2017. Geneva, Switzerland: World Health Organization (WHO), 2017

El Salvador Vital Registration - Deaths 1999 ICD10 as it appears in World Health Organization (WHO). WHO Mortality Database Version October 2017. Geneva, Switzerland: World Health Organization (WHO), 2017

El Salvador Vital Registration - Deaths 2000 ICD10 as it appears in World Health Organization (WHO). WHO Mortality Database Version October 2017. Geneva, Switzerland: World Health Organization (WHO), 2017

El Salvador Vital Registration - Deaths 2001 ICD10 as it appears in World Health Organization (WHO). WHO Mortality Database Version October 2017. Geneva, Switzerland: World Health Organization (WHO), 2017

El Salvador Vital Registration - Deaths 2002 ICD10 as it appears in World Health Organization (WHO). WHO Mortality Database Version October 2017. Geneva, Switzerland: World Health Organization (WHO), 2017
El Salvador Vital Registration - Deaths 2003 ICD10 as it appears in World Health Organization (WHO). WHO Mortality Database Version October 2017. Geneva, Switzerland: World Health Organization (WHO), 2017
El Salvador Vital Registration - Deaths 2004 ICD10 as it appears in World Health Organization (WHO). WHO Mortality Database Version October 2017. Geneva, Switzerland: World Health Organization (WHO), 2017
El Salvador Vital Registration - Deaths 2005 ICD10 as it appears in World Health Organization (WHO). WHO Mortality Database Version October 2017. Geneva, Switzerland: World Health Organization (WHO), 2017
El Salvador Vital Registration - Deaths 2006 ICD10 as it appears in World Health Organization (WHO). WHO Mortality Database Version October 2017. Geneva, Switzerland: World Health Organization (WHO), 2017
El Salvador Vital Registration - Deaths 2007 ICD10 as it appears in World Health Organization (WHO). WHO Mortality Database Version October 2017. Geneva, Switzerland: World Health Organization (WHO), 2017
El Salvador Vital Registration - Deaths 2008 ICD10 as it appears in World Health Organization (WHO). WHO Mortality Database Version October 2017. Geneva, Switzerland: World Health Organization (WHO), 2017
El Salvador Vital Registration - Deaths 2009 ICD10 as it appears in World Health Organization (WHO). WHO Mortality Database Version October 2017. Geneva, Switzerland: World Health Organization (WHO), 2017
El Salvador Vital Registration - Deaths 2010 ICD10 as it appears in World Health Organization (WHO). WHO Mortality Database Version October 2017. Geneva, Switzerland: World Health Organization (WHO), 2017
El Salvador Vital Registration - Deaths 2011 ICD10 as it appears in World Health Organization (WHO). WHO Mortality Database Version October 2017. Geneva, Switzerland: World Health Organization (WHO), 2017
El Salvador Vital Registration - Deaths 2012 ICD10 as it appears in World Health Organization (WHO). WHO Mortality Database Version October 2017. Geneva, Switzerland: World Health Organization (WHO), 2017
El Salvador Vital Registration - Deaths 2013 ICD10 as it appears in World Health Organization (WHO). WHO Mortality Database Version October 2017. Geneva, Switzerland: World Health Organization (WHO), 2017
El Salvador Vital Registration - Deaths 2014 ICD10 as it appears in World Health Organization (WHO). WHO Mortality Database Version October 2017. Geneva, Switzerland: World Health Organization (WHO), 2017
Estonia Vital Registration - Deaths 1981 ICD9 as it appears in World Health Organization (WHO). WHO Mortality Database Version September 2016. Geneva, Switzerland: World Health Organization (WHO), 2016
Estonia Vital Registration - Deaths 1982 ICD9 as it appears in World Health Organization (WHO). WHO Mortality Database Version September 2016. Geneva, Switzerland: World Health Organization (WHO), 2016
Estonia Vital Registration - Deaths 1985 ICD9 as it appears in World Health Organization (WHO). WHO Mortality Database Version September 2016. Geneva, Switzerland: World Health Organization (WHO), 2016
Estonia Vital Registration - Deaths 1986 ICD9 as it appears in World Health Organization (WHO). WHO Mortality Database Version September 2016. Geneva, Switzerland: World Health Organization (WHO), 2016
Estonia Vital Registration - Deaths 1987 ICD9 as it appears in World Health Organization (WHO). WHO Mortality Database Version September 2016. Geneva, Switzerland: World Health Organization (WHO), 2016

Estonia Vital Registration - Deaths 1988 ICD9 as it appears in World Health Organization (WHO). WHO Mortality Database Version September 2016. Geneva, Switzerland: World Health Organization (WHO), 2016
Estonia Vital Registration - Deaths 1989 ICD9 as it appears in World Health Organization (WHO). WHO Mortality Database Version September 2016. Geneva, Switzerland: World Health Organization (WHO), 2016
Estonia Vital Registration - Deaths 1990 ICD9 as it appears in World Health Organization (WHO). WHO Mortality Database Version September 2016. Geneva, Switzerland: World Health Organization (WHO), 2016
Estonia Vital Registration - Deaths 1991 ICD9 as it appears in World Health Organization (WHO). WHO Mortality Database Version September 2016. Geneva, Switzerland: World Health Organization (WHO), 2016
Estonia Vital Registration - Deaths 1992 ICD9 as it appears in World Health Organization (WHO). WHO Mortality Database Version September 2016. Geneva, Switzerland: World Health Organization (WHO), 2016
Estonia Vital Registration - Deaths 1993 ICD9 as it appears in World Health Organization (WHO). WHO Mortality Database Version September 2016. Geneva, Switzerland: World Health Organization (WHO), 2016
Estonia Vital Registration - Deaths 1994 ICD9 as it appears in World Health Organization (WHO). WHO Mortality Database Version November 2015. Geneva, Switzerland: World Health Organization (WHO), 2015
Estonia Vital Registration - Deaths 1995 ICD9 as it appears in World Health Organization (WHO). WHO Mortality Database Version November 2015. Geneva, Switzerland: World Health Organization (WHO), 2015
Estonia Vital Registration - Deaths 1996 ICD9 as it appears in World Health Organization (WHO). WHO Mortality Database Version November 2015. Geneva, Switzerland: World Health Organization (WHO), 2015
Estonia Vital Registration - Deaths 1997 ICD10 as it appears in World Health Organization (WHO). WHO Mortality Database Version October 2017. Geneva, Switzerland: World Health Organization (WHO), 2017
Estonia Vital Registration - Deaths 1998 ICD10 as it appears in World Health Organization (WHO). WHO Mortality Database Version October 2017. Geneva, Switzerland: World Health Organization (WHO), 2017
Estonia Vital Registration - Deaths 1999 ICD10 as it appears in World Health Organization (WHO). WHO Mortality Database Version October 2017. Geneva, Switzerland: World Health Organization (WHO), 2017
Estonia Vital Registration - Deaths 2000 ICD10 as it appears in World Health Organization (WHO). WHO Mortality Database Version October 2017. Geneva, Switzerland: World Health Organization (WHO), 2017
Estonia Vital Registration - Deaths 2001 ICD10 as it appears in World Health Organization (WHO). WHO Mortality Database Version October 2017. Geneva, Switzerland: World Health Organization (WHO), 2017
Estonia Vital Registration - Deaths 2002 ICD10 as it appears in World Health Organization (WHO). WHO Mortality Database Version October 2017. Geneva, Switzerland: World Health Organization (WHO), 2017
Estonia Vital Registration - Deaths 2003 ICD10 as it appears in World Health Organization (WHO). WHO Mortality Database Version October 2017. Geneva, Switzerland: World Health Organization (WHO), 2017
Estonia Vital Registration - Deaths 2004 ICD10 as it appears in World Health Organization (WHO). WHO Mortality Database Version October 2017. Geneva, Switzerland: World Health Organization (WHO), 2017
Estonia Vital Registration - Deaths 2005 ICD10 as it appears in World Health Organization (WHO). WHO Mortality Database Version October 2017. Geneva, Switzerland: World Health Organization (WHO), 2017

Estonia Vital Registration - Deaths 2006 ICD10 as it appears in World Health Organization (WHO). WHO Mortality Database Version October 2017. Geneva, Switzerland: World Health Organization (WHO), 2017

Estonia Vital Registration - Deaths 2007 ICD10 as it appears in World Health Organization (WHO). WHO Mortality Database Version October 2017. Geneva, Switzerland: World Health Organization (WHO), 2017

Estonia Vital Registration - Deaths 2008 ICD10 as it appears in World Health Organization (WHO). WHO Mortality Database Version October 2017. Geneva, Switzerland: World Health Organization (WHO), 2017

Estonia Vital Registration - Deaths 2009 ICD10 as it appears in World Health Organization (WHO). WHO Mortality Database Version October 2017. Geneva, Switzerland: World Health Organization (WHO), 2017

Estonia Vital Registration - Deaths 2010 ICD10 as it appears in World Health Organization (WHO). WHO Mortality Database Version October 2017. Geneva, Switzerland: World Health Organization (WHO), 2017

Estonia Vital Registration - Deaths 2011 ICD10 as it appears in World Health Organization (WHO). WHO Mortality Database Version October 2017. Geneva, Switzerland: World Health Organization (WHO), 2017

Estonia Vital Registration - Deaths 2012 ICD10 as it appears in World Health Organization (WHO). WHO Mortality Database Version October 2017. Geneva, Switzerland: World Health Organization (WHO), 2017

Estonia Vital Registration - Deaths 2013 ICD10 as it appears in World Health Organization (WHO). WHO Mortality Database Version October 2017. Geneva, Switzerland: World Health Organization (WHO), 2017

Estonia Vital Registration - Deaths 2014 ICD10 as it appears in World Health Organization (WHO). WHO Mortality Database Version October 2017. Geneva, Switzerland: World Health Organization (WHO), 2017

Estonia Vital Registration - Deaths 2015 ICD10 as it appears in World Health Organization (WHO). WHO Mortality Database Version October 2017. Geneva, Switzerland: World Health Organization (WHO), 2017

Fargues P, Nassour O, National Institute for Demographic Studies (France), Sahel Institute. Twelve Years of Urban Mortality in the Sahel. Levels, Trends, Seasons, and Causes of Mortality in Bamako, 1974-1985. Paris, France: National Institute for Demographic Studies (France), 1988

Fiji Vital Registration - Deaths 1999 ICD9 as it appears in World Health Organization (WHO). WHO Mortality Database Version September 2016. Geneva, Switzerland: World Health Organization (WHO), 2016

Fiji Vital Registration - Deaths 2002 ICD10 as it appears in World Health Organization (WHO). WHO Mortality Database Version October 2017. Geneva, Switzerland: World Health Organization (WHO), 2017

Fiji Vital Registration - Deaths 2003 ICD10 as it appears in World Health Organization (WHO). WHO Mortality Database Version October 2017. Geneva, Switzerland: World Health Organization (WHO), 2017

Fiji Vital Registration - Deaths 2004 ICD10 as it appears in World Health Organization (WHO). WHO Mortality Database Version October 2017. Geneva, Switzerland: World Health Organization (WHO), 2017

Fiji Vital Registration - Deaths 2005 ICD10 as it appears in World Health Organization (WHO). WHO Mortality Database Version October 2017. Geneva, Switzerland: World Health Organization (WHO), 2017

Fiji Vital Registration - Deaths 2006 ICD10 as it appears in World Health Organization (WHO). WHO Mortality Database Version October 2017. Geneva, Switzerland: World Health Organization (WHO), 2017

Fiji Vital Registration - Deaths 2007 ICD10 as it appears in World Health Organization (WHO). WHO Mortality Database Version October 2017. Geneva, Switzerland: World Health Organization (WHO), 2017

Fiji Vital Registration - Deaths 2008 ICD10 as it appears in World Health Organization (WHO). WHO Mortality Database Version October 2017. Geneva, Switzerland: World Health Organization (WHO), 2017

Fiji Vital Registration - Deaths 2009 ICD10 as it appears in World Health Organization (WHO). WHO Mortality Database Version October 2017. Geneva, Switzerland: World Health Organization (WHO), 2017

Fiji Vital Registration - Deaths 2011 ICD10 as it appears in World Health Organization (WHO). WHO Mortality Database Version October 2017. Geneva, Switzerland: World Health Organization (WHO), 2017

Fiji Vital Registration - Deaths 2012 ICD10 as it appears in World Health Organization (WHO). WHO Mortality Database Version October 2017. Geneva, Switzerland: World Health Organization (WHO), 2017

Fikree FF, Azam SI, Berendes HW. Time to focus child survival programmes on the newborn: assessment of levels and causes of infant mortality in rural Pakistan. Bull World Health Organ. 2002; 80(4): 271-6

Finland Vital Registration - Deaths 1980 ICD8 as it appears in World Health Organization (WHO). WHO Mortality Database Version July 2012. Geneva, Switzerland: World Health Organization (WHO), 2012

Finland Vital Registration - Deaths 1982 ICD8 as it appears in World Health Organization (WHO). WHO Mortality Database Version July 2012. Geneva, Switzerland: World Health Organization (WHO), 2012

Finland Vital Registration - Deaths 1983 ICD8 as it appears in World Health Organization (WHO). WHO Mortality Database Version July 2012. Geneva, Switzerland: World Health Organization (WHO), 2012

Finland Vital Registration - Deaths 1984 ICD8 as it appears in World Health Organization (WHO). WHO Mortality Database Version July 2012. Geneva, Switzerland: World Health Organization (WHO), 2012

Finland Vital Registration - Deaths 1985 ICD8 as it appears in World Health Organization (WHO). WHO Mortality Database Version July 2012. Geneva, Switzerland: World Health Organization (WHO), 2012

Finland Vital Registration - Deaths 1986 ICD8 as it appears in World Health Organization (WHO). WHO Mortality Database Version July 2012. Geneva, Switzerland: World Health Organization (WHO), 2012

Finland Vital Registration - Deaths 1987 ICD9 as it appears in World Health Organization (WHO). WHO Mortality Database Version September 2016. Geneva, Switzerland: World Health Organization (WHO), 2016

Finland Vital Registration - Deaths 1988 ICD9 as it appears in World Health Organization (WHO). WHO Mortality Database Version September 2016. Geneva, Switzerland: World Health Organization (WHO), 2016

Finland Vital Registration - Deaths 1989 ICD9 as it appears in World Health Organization (WHO). WHO Mortality Database Version September 2016. Geneva, Switzerland: World Health Organization (WHO), 2016

Finland Vital Registration - Deaths 1990 ICD9 as it appears in World Health Organization (WHO). WHO Mortality Database Version September 2016. Geneva, Switzerland: World Health Organization (WHO), 2016

Finland Vital Registration - Deaths 1991 ICD9 as it appears in World Health Organization (WHO). WHO Mortality Database Version September 2016. Geneva, Switzerland: World Health Organization (WHO), 2016

Finland Vital Registration - Deaths 1992 ICD9 as it appears in World Health Organization (WHO). WHO Mortality Database Version September 2016. Geneva, Switzerland: World Health Organization (WHO), 2016

Finland Vital Registration - Deaths 1993 ICD9 as it appears in World Health Organization (WHO). WHO Mortality Database Version September 2016. Geneva, Switzerland: World Health Organization (WHO), 2016

Finland Vital Registration - Deaths 1994 ICD9 as it appears in World Health Organization (WHO). WHO Mortality Database Version September 2016. Geneva, Switzerland: World Health Organization (WHO), 2016

Finland Vital Registration - Deaths 1995 ICD9 as it appears in World Health Organization (WHO). WHO Mortality Database Version September 2016. Geneva, Switzerland: World Health Organization (WHO), 2016

Finland Vital Registration - Deaths 1996 ICD10 as it appears in World Health Organization (WHO). WHO Mortality Database Version October 2017. Geneva, Switzerland: World Health Organization (WHO), 2017

Finland Vital Registration - Deaths 1997 ICD10 as it appears in World Health Organization (WHO). WHO Mortality Database Version October 2017. Geneva, Switzerland: World Health Organization (WHO), 2017

Finland Vital Registration - Deaths 1998 ICD10 as it appears in World Health Organization (WHO). WHO Mortality Database Version October 2017. Geneva, Switzerland: World Health Organization (WHO), 2017

Finland Vital Registration - Deaths 1999 ICD10 as it appears in World Health Organization (WHO). WHO Mortality Database Version October 2017. Geneva, Switzerland: World Health Organization (WHO), 2017

Finland Vital Registration - Deaths 2000 ICD10 as it appears in World Health Organization (WHO). WHO Mortality Database Version October 2017. Geneva, Switzerland: World Health Organization (WHO), 2017

Finland Vital Registration - Deaths 2001 ICD10 as it appears in World Health Organization (WHO). WHO Mortality Database Version October 2017. Geneva, Switzerland: World Health Organization (WHO), 2017

Finland Vital Registration - Deaths 2002 ICD10 as it appears in World Health Organization (WHO). WHO Mortality Database Version October 2017. Geneva, Switzerland: World Health Organization (WHO), 2017

Finland Vital Registration - Deaths 2003 ICD10 as it appears in World Health Organization (WHO). WHO Mortality Database Version October 2017. Geneva, Switzerland: World Health Organization (WHO), 2017

Finland Vital Registration - Deaths 2004 ICD10 as it appears in World Health Organization (WHO). WHO Mortality Database Version October 2017. Geneva, Switzerland: World Health Organization (WHO), 2017

Finland Vital Registration - Deaths 2005 ICD10 as it appears in World Health Organization (WHO). WHO Mortality Database Version October 2017. Geneva, Switzerland: World Health Organization (WHO), 2017

Finland Vital Registration - Deaths 2006 ICD10 as it appears in World Health Organization (WHO). WHO Mortality Database Version October 2017. Geneva, Switzerland: World Health Organization (WHO), 2017

Finland Vital Registration - Deaths 2007 ICD10 as it appears in World Health Organization (WHO). WHO Mortality Database Version October 2017. Geneva, Switzerland: World Health Organization (WHO), 2017

Finland Vital Registration - Deaths 2008 ICD10 as it appears in World Health Organization (WHO). WHO Mortality Database Version October 2017. Geneva, Switzerland: World Health Organization (WHO), 2017

Finland Vital Registration - Deaths 1981 ICD8 as it appears in World Health Organization (WHO). WHO Mortality Database Version July 2012. Geneva, Switzerland: World Health Organization (WHO), 2012

Finland Vital Registration - Deaths 2009 ICD10 as it appears in World Health Organization (WHO). WHO Mortality Database Version October 2017. Geneva, Switzerland: World Health Organization (WHO), 2017

Finland Vital Registration - Deaths 2010 ICD10 as it appears in World Health Organization (WHO). WHO Mortality Database Version October 2017. Geneva, Switzerland: World Health Organization (WHO), 2017
Finland Vital Registration - Deaths 2011 ICD10 as it appears in World Health Organization (WHO). WHO Mortality Database Version October 2017. Geneva, Switzerland: World Health Organization (WHO), 2017
Finland Vital Registration - Deaths 2012 ICD10 as it appears in World Health Organization (WHO). WHO Mortality Database Version October 2017. Geneva, Switzerland: World Health Organization (WHO), 2017
Finland Vital Registration - Deaths 2013 ICD10 as it appears in World Health Organization (WHO). WHO Mortality Database Version October 2017. Geneva, Switzerland: World Health Organization (WHO), 2017
Finland Vital Registration - Deaths 2014 ICD10 as it appears in World Health Organization (WHO). WHO Mortality Database Version October 2017. Geneva, Switzerland: World Health Organization (WHO), 2017
Finland Vital Registration - Deaths 2015 ICD10 as it appears in World Health Organization (WHO). WHO Mortality Database Version October 2017. Geneva, Switzerland: World Health Organization (WHO), 2017
France Vital Registration - Deaths 1980 ICD9 as it appears in World Health Organization (WHO). WHO Mortality Database Version November 2015. Geneva, Switzerland: World Health Organization (WHO), 2015
France Vital Registration - Deaths 1981 ICD9 as it appears in World Health Organization (WHO). WHO Mortality Database Version November 2015. Geneva, Switzerland: World Health Organization (WHO), 2015
France Vital Registration - Deaths 1982 ICD9 as it appears in World Health Organization (WHO). WHO Mortality Database Version November 2015. Geneva, Switzerland: World Health Organization (WHO), 2015
France Vital Registration - Deaths 1983 ICD9 as it appears in World Health Organization (WHO). WHO Mortality Database Version November 2015. Geneva, Switzerland: World Health Organization (WHO), 2015
France Vital Registration - Deaths 1984 ICD9 as it appears in World Health Organization (WHO). WHO Mortality Database Version November 2015. Geneva, Switzerland: World Health Organization (WHO), 2015
France Vital Registration - Deaths 1985 ICD9 as it appears in World Health Organization (WHO). WHO Mortality Database Version November 2015. Geneva, Switzerland: World Health Organization (WHO), 2015
France Vital Registration - Deaths 1986 ICD9 as it appears in World Health Organization (WHO). WHO Mortality Database Version November 2015. Geneva, Switzerland: World Health Organization (WHO), 2015
France Vital Registration - Deaths 1987 ICD9 as it appears in World Health Organization (WHO). WHO Mortality Database Version November 2015. Geneva, Switzerland: World Health Organization (WHO), 2015
France Vital Registration - Deaths 1988 ICD9 as it appears in World Health Organization (WHO). WHO Mortality Database Version November 2015. Geneva, Switzerland: World Health Organization (WHO), 2015
France Vital Registration - Deaths 1989 ICD9 as it appears in World Health Organization (WHO). WHO Mortality Database Version November 2015. Geneva, Switzerland: World Health Organization (WHO), 2015
France Vital Registration - Deaths 1990 ICD9 as it appears in World Health Organization (WHO). WHO Mortality Database Version November 2015. Geneva, Switzerland: World Health Organization (WHO), 2015
France Vital Registration - Deaths 1991 ICD9 as it appears in World Health Organization (WHO). WHO Mortality Database Version November 2015. Geneva, Switzerland: World Health Organization (WHO), 2015

France Vital Registration - Deaths 1992 ICD9 as it appears in World Health Organization (WHO). WHO Mortality Database Version November 2015. Geneva, Switzerland: World Health Organization (WHO), 2015
France Vital Registration - Deaths 1993 ICD9 as it appears in World Health Organization (WHO). WHO Mortality Database Version November 2015. Geneva, Switzerland: World Health Organization (WHO), 2015
France Vital Registration - Deaths 1994 ICD9 as it appears in World Health Organization (WHO). WHO Mortality Database Version November 2015. Geneva, Switzerland: World Health Organization (WHO), 2015
France Vital Registration - Deaths 1995 ICD9 as it appears in World Health Organization (WHO). WHO Mortality Database Version November 2015. Geneva, Switzerland: World Health Organization (WHO), 2015
France Vital Registration - Deaths 1996 ICD9 as it appears in World Health Organization (WHO). WHO Mortality Database Version November 2015. Geneva, Switzerland: World Health Organization (WHO), 2015
France Vital Registration - Deaths 1997 ICD9 as it appears in World Health Organization (WHO). WHO Mortality Database Version November 2015. Geneva, Switzerland: World Health Organization (WHO), 2015
France Vital Registration - Deaths 1998 ICD9 as it appears in World Health Organization (WHO). WHO Mortality Database Version November 2015. Geneva, Switzerland: World Health Organization (WHO), 2015
France Vital Registration - Deaths 1999 ICD9 as it appears in World Health Organization (WHO). WHO Mortality Database Version November 2015. Geneva, Switzerland: World Health Organization (WHO), 2015
France Vital Registration - Deaths 2000 ICD10 as it appears in World Health Organization (WHO). WHO Mortality Database Version October 2017. Geneva, Switzerland: World Health Organization (WHO), 2017
France Vital Registration - Deaths 2001 ICD10 as it appears in World Health Organization (WHO). WHO Mortality Database Version October 2017. Geneva, Switzerland: World Health Organization (WHO), 2017
France Vital Registration - Deaths 2002 ICD10 as it appears in World Health Organization (WHO). WHO Mortality Database Version October 2017. Geneva, Switzerland: World Health Organization (WHO), 2017
France Vital Registration - Deaths 2003 ICD10 as it appears in World Health Organization (WHO). WHO Mortality Database Version October 2017. Geneva, Switzerland: World Health Organization (WHO), 2017
France Vital Registration - Deaths 2004 ICD10 as it appears in World Health Organization (WHO). WHO Mortality Database Version October 2017. Geneva, Switzerland: World Health Organization (WHO), 2017
France Vital Registration - Deaths 2005 ICD10 as it appears in World Health Organization (WHO). WHO Mortality Database Version October 2017. Geneva, Switzerland: World Health Organization (WHO), 2017
France Vital Registration - Deaths 2006 ICD10 as it appears in World Health Organization (WHO). WHO Mortality Database Version October 2017. Geneva, Switzerland: World Health Organization (WHO), 2017
France Vital Registration - Deaths 2007 ICD10 as it appears in World Health Organization (WHO). WHO Mortality Database Version October 2017. Geneva, Switzerland: World Health Organization (WHO), 2017
France Vital Registration - Deaths 2008 ICD10 as it appears in World Health Organization (WHO). WHO Mortality Database Version October 2017. Geneva, Switzerland: World Health Organization (WHO), 2017
France Vital Registration - Deaths 2009 ICD10 as it appears in World Health Organization (WHO). WHO Mortality Database Version October 2017. Geneva, Switzerland: World Health Organization (WHO), 2017

France Vital Registration - Deaths 2010 ICD10 as it appears in World Health Organization (WHO). WHO Mortality Database Version October 2017. Geneva, Switzerland: World Health Organization (WHO), 2017

France Vital Registration - Deaths 2011 ICD10 as it appears in World Health Organization (WHO). WHO Mortality Database Version October 2017. Geneva, Switzerland: World Health Organization (WHO), 2017

France Vital Registration - Deaths 2012 ICD10 as it appears in World Health Organization (WHO). WHO Mortality Database Version October 2017. Geneva, Switzerland: World Health Organization (WHO), 2017

France Vital Registration - Deaths 2013 ICD10 as it appears in World Health Organization (WHO). WHO Mortality Database Version October 2017. Geneva, Switzerland: World Health Organization (WHO), 2017

France Vital Registration - Deaths 2014 ICD10 as it appears in World Health Organization (WHO). WHO Mortality Database Version October 2017. Geneva, Switzerland: World Health Organization (WHO), 2017

Freeman JV, Christian P, Khatry SK, Adhikari RK, LeClerq SC, Katz J, Darmstadt GL. Evaluation of neonatal verbal autopsy using physician review versus algorithm-based cause-of-death assignment in rural Nepal. Paediatr Perinat Epidemiol. 2005; 19(4): 323-31

Geetha T, Chenoy R, Stevens D, Johanson RB. A multicentre study of perinatal mortality in Nepal. Paediatr Perinat Epidemiol. 1995; 9(1): 74-89

General Information Authority (Libya). Libya Vital Statistics 2006. Tripoli, Libya: General Information Authority (Libya)

General Information Authority (Libya). Libya Vital Statistics 2007. Tripoli, Libya: General Information Authority (Libya)

General Information Authority (Libya). Libya Vital Statistics 2008. Tripoli, Libya: General Information Authority (Libya)

Georgia Vital Registration - Deaths 1981 ICD9 as it appears in World Health Organization (WHO). WHO Mortality Database Version September 2016. Geneva, Switzerland: World Health Organization (WHO), 2016

Georgia Vital Registration - Deaths 1982 ICD9 as it appears in World Health Organization (WHO). WHO Mortality Database Version September 2016. Geneva, Switzerland: World Health Organization (WHO), 2016

Georgia Vital Registration - Deaths 1985 ICD9 as it appears in World Health Organization (WHO). WHO Mortality Database Version September 2016. Geneva, Switzerland: World Health Organization (WHO), 2016

Georgia Vital Registration - Deaths 1986 ICD9 as it appears in World Health Organization (WHO). WHO Mortality Database Version September 2016. Geneva, Switzerland: World Health Organization (WHO), 2016

Georgia Vital Registration - Deaths 1987 ICD9 as it appears in World Health Organization (WHO). WHO Mortality Database Version September 2016. Geneva, Switzerland: World Health Organization (WHO), 2016

Georgia Vital Registration - Deaths 1988 ICD9 as it appears in World Health Organization (WHO). WHO Mortality Database Version September 2016. Geneva, Switzerland: World Health Organization (WHO), 2016

Georgia Vital Registration - Deaths 1989 ICD9 as it appears in World Health Organization (WHO). WHO Mortality Database Version September 2016. Geneva, Switzerland: World Health Organization (WHO), 2016

Georgia Vital Registration - Deaths 1990 ICD9 as it appears in World Health Organization (WHO). WHO Mortality Database Version September 2016. Geneva, Switzerland: World Health Organization (WHO), 2016

Georgia Vital Registration - Deaths 1991 ICD9 as it appears in World Health Organization (WHO). WHO Mortality Database Version September 2016. Geneva, Switzerland: World Health Organization (WHO), 2016

Georgia Vital Registration - Deaths 1992 ICD9 as it appears in World Health Organization (WHO). WHO Mortality Database Version September 2016. Geneva, Switzerland: World Health Organization (WHO), 2016
Georgia Vital Registration - Deaths 1994 ICD9 as it appears in World Health Organization (WHO). WHO Mortality Database Version September 2016. Geneva, Switzerland: World Health Organization (WHO), 2016
Georgia Vital Registration - Deaths 1995 ICD9 as it appears in World Health Organization (WHO). WHO Mortality Database Version September 2016. Geneva, Switzerland: World Health Organization (WHO), 2016
Georgia Vital Registration - Deaths 1996 ICD9 as it appears in World Health Organization (WHO). WHO Mortality Database Version September 2016. Geneva, Switzerland: World Health Organization (WHO), 2016
Georgia Vital Registration - Deaths 1997 ICD9 as it appears in World Health Organization (WHO). WHO Mortality Database Version September 2016. Geneva, Switzerland: World Health Organization (WHO), 2016
Georgia Vital Registration - Deaths 1998 ICD10 as it appears in World Health Organization (WHO). WHO Mortality Database Version October 2017. Geneva, Switzerland: World Health Organization (WHO), 2017
Georgia Vital Registration - Deaths 1999 ICD10 as it appears in World Health Organization (WHO). WHO Mortality Database Version October 2017. Geneva, Switzerland: World Health Organization (WHO), 2017
Georgia Vital Registration - Deaths 2000 ICD10 as it appears in World Health Organization (WHO). WHO Mortality Database Version October 2017. Geneva, Switzerland: World Health Organization (WHO), 2017
Georgia Vital Registration - Deaths 2001 ICD10 as it appears in World Health Organization (WHO). WHO Mortality Database Version October 2017. Geneva, Switzerland: World Health Organization (WHO), 2017
Georgia Vital Registration - Deaths 2004 ICD10 as it appears in World Health Organization (WHO). WHO Mortality Database Version October 2017. Geneva, Switzerland: World Health Organization (WHO), 2017
Georgia Vital Registration - Deaths 2005 ICD10 as it appears in World Health Organization (WHO). WHO Mortality Database Version October 2017. Geneva, Switzerland: World Health Organization (WHO), 2017
Georgia Vital Registration - Deaths 2006 ICD10 as it appears in World Health Organization (WHO). WHO Mortality Database Version October 2017. Geneva, Switzerland: World Health Organization (WHO), 2017
Georgia Vital Registration - Deaths 2007 ICD10 as it appears in World Health Organization (WHO). WHO Mortality Database Version October 2017. Geneva, Switzerland: World Health Organization (WHO), 2017
Georgia Vital Registration - Deaths 2009 ICD10 as it appears in World Health Organization (WHO). WHO Mortality Database Version October 2017. Geneva, Switzerland: World Health Organization (WHO), 2017
Georgia Vital Registration - Deaths 2012 ICD10 as it appears in World Health Organization (WHO). WHO Mortality Database Version October 2017. Geneva, Switzerland: World Health Organization (WHO), 2017
Georgia Vital Registration - Deaths 2014 ICD10 as it appears in World Health Organization (WHO). WHO Mortality Database Version October 2017. Geneva, Switzerland: World Health Organization (WHO), 2017
Georgia Vital Registration - Deaths 2015 ICD10 as it appears in World Health Organization (WHO). WHO Mortality Database Version October 2017. Geneva, Switzerland: World Health Organization (WHO), 2017
Germany Vital Registration - Deaths 1991 ICD9 as it appears in World Health Organization (WHO). WHO Mortality Database Version September 2016. Geneva, Switzerland: World Health Organization (WHO), 2016

Germany Vital Registration - Deaths 1992 ICD9 as it appears in World Health Organization (WHO). WHO Mortality Database Version September 2016. Geneva, Switzerland: World Health Organization (WHO), 2016
Germany Vital Registration - Deaths 1993 ICD9 as it appears in World Health Organization (WHO). WHO Mortality Database Version September 2016. Geneva, Switzerland: World Health Organization (WHO), 2016
Germany Vital Registration - Deaths 1994 ICD9 as it appears in World Health Organization (WHO). WHO Mortality Database Version September 2016. Geneva, Switzerland: World Health Organization (WHO), 2016
Germany Vital Registration - Deaths 1995 ICD9 as it appears in World Health Organization (WHO). WHO Mortality Database Version November 2015. Geneva, Switzerland: World Health Organization (WHO), 2015
Germany Vital Registration - Deaths 1996 ICD9 as it appears in World Health Organization (WHO). WHO Mortality Database Version November 2015. Geneva, Switzerland: World Health Organization (WHO), 2015
Germany Vital Registration - Deaths 1997 ICD9 as it appears in World Health Organization (WHO). WHO Mortality Database Version November 2015. Geneva, Switzerland: World Health Organization (WHO), 2015
Germany Vital Registration - Deaths 1998 ICD10 as it appears in World Health Organization (WHO). WHO Mortality Database Version October 2017. Geneva, Switzerland: World Health Organization (WHO), 2017
Germany Vital Registration - Deaths 1999 ICD10 as it appears in World Health Organization (WHO). WHO Mortality Database Version October 2017. Geneva, Switzerland: World Health Organization (WHO), 2017
Germany Vital Registration - Deaths 2000 ICD10 as it appears in World Health Organization (WHO). WHO Mortality Database Version October 2017. Geneva, Switzerland: World Health Organization (WHO), 2017
Germany Vital Registration - Deaths 2001 ICD10 as it appears in World Health Organization (WHO). WHO Mortality Database Version October 2017. Geneva, Switzerland: World Health Organization (WHO), 2017
Germany Vital Registration - Deaths 2002 ICD10 as it appears in World Health Organization (WHO). WHO Mortality Database Version October 2017. Geneva, Switzerland: World Health Organization (WHO), 2017
Germany Vital Registration - Deaths 2003 ICD10 as it appears in World Health Organization (WHO). WHO Mortality Database Version October 2017. Geneva, Switzerland: World Health Organization (WHO), 2017
Germany Vital Registration - Deaths 2004 ICD10 as it appears in World Health Organization (WHO). WHO Mortality Database Version October 2017. Geneva, Switzerland: World Health Organization (WHO), 2017
Germany Vital Registration - Deaths 2005 ICD10 as it appears in World Health Organization (WHO). WHO Mortality Database Version October 2017. Geneva, Switzerland: World Health Organization (WHO), 2017
Germany Vital Registration - Deaths 2006 ICD10 as it appears in World Health Organization (WHO). WHO Mortality Database Version October 2017. Geneva, Switzerland: World Health Organization (WHO), 2017
Germany Vital Registration - Deaths 1980 ICD9 as it appears in World Health Organization (WHO). WHO Mortality Database Version September 2016. Geneva, Switzerland: World Health Organization (WHO), 2016
Germany Vital Registration - Deaths 1981 ICD9 as it appears in World Health Organization (WHO). WHO Mortality Database Version September 2016. Geneva, Switzerland: World Health Organization (WHO), 2016
Germany Vital Registration - Deaths 1982 ICD9 as it appears in World Health Organization (WHO). WHO Mortality Database Version September 2016. Geneva, Switzerland: World Health Organization (WHO), 2016

Germany Vital Registration - Deaths 1983 ICD9 as it appears in World Health Organization (WHO). WHO Mortality Database Version September 2016. Geneva, Switzerland: World Health Organization (WHO), 2016

Germany Vital Registration - Deaths 1984 ICD9 as it appears in World Health Organization (WHO). WHO Mortality Database Version September 2016. Geneva, Switzerland: World Health Organization (WHO), 2016

Germany Vital Registration - Deaths 1985 ICD9 as it appears in World Health Organization (WHO). WHO Mortality Database Version September 2016. Geneva, Switzerland: World Health Organization (WHO), 2016

Germany Vital Registration - Deaths 1986 ICD9 as it appears in World Health Organization (WHO). WHO Mortality Database Version September 2016. Geneva, Switzerland: World Health Organization (WHO), 2016

Germany Vital Registration - Deaths 1987 ICD9 as it appears in World Health Organization (WHO). WHO Mortality Database Version September 2016. Geneva, Switzerland: World Health Organization (WHO), 2016

Germany Vital Registration - Deaths 1988 ICD9 as it appears in World Health Organization (WHO). WHO Mortality Database Version September 2016. Geneva, Switzerland: World Health Organization (WHO), 2016

Germany Vital Registration - Deaths 1989 ICD9 as it appears in World Health Organization (WHO). WHO Mortality Database Version September 2016. Geneva, Switzerland: World Health Organization (WHO), 2016

Germany Vital Registration - Deaths 1990 ICD9 as it appears in World Health Organization (WHO). WHO Mortality Database Version September 2016. Geneva, Switzerland: World Health Organization (WHO), 2016

Germany Vital Registration - Deaths 2007 ICD10 as it appears in World Health Organization (WHO). WHO Mortality Database Version October 2017. Geneva, Switzerland: World Health Organization (WHO), 2017

Germany Vital Registration - Deaths 2008 ICD10 as it appears in World Health Organization (WHO). WHO Mortality Database Version October 2017. Geneva, Switzerland: World Health Organization (WHO), 2017

Germany Vital Registration - Deaths 2009 ICD10 as it appears in World Health Organization (WHO). WHO Mortality Database Version October 2017. Geneva, Switzerland: World Health Organization (WHO), 2017

Germany Vital Registration - Deaths 2010 ICD10 as it appears in World Health Organization (WHO). WHO Mortality Database Version October 2017. Geneva, Switzerland: World Health Organization (WHO), 2017

Germany Vital Registration - Deaths 2011 ICD10 as it appears in World Health Organization (WHO). WHO Mortality Database Version October 2017. Geneva, Switzerland: World Health Organization (WHO), 2017

Germany Vital Registration - Deaths 2012 ICD10 as it appears in World Health Organization (WHO). WHO Mortality Database Version October 2017. Geneva, Switzerland: World Health Organization (WHO), 2017

Germany Vital Registration - Deaths 2013 ICD10 as it appears in World Health Organization (WHO). WHO Mortality Database Version October 2017. Geneva, Switzerland: World Health Organization (WHO), 2017

Germany Vital Registration - Deaths 2014 ICD10 as it appears in World Health Organization (WHO). WHO Mortality Database Version October 2017. Geneva, Switzerland: World Health Organization (WHO), 2017

Germany Vital Registration - Deaths 2015 ICD10 as it appears in World Health Organization (WHO). WHO Mortality Database Version October 2017. Geneva, Switzerland: World Health Organization (WHO), 2017

Ghana Health Service, Ghana Statistical Service, ICF Macro, MEASURE Evaluation Project, Carolina Population Center, University of North Carolina. Ghana Child Verbal Autopsy Study 2008

Ghana Health Service, Ghana Statistical Service, Macro International, Inc. Ghana Special Demographic and Health Survey 2007-2008. Fairfax, United States: ICF International

Ghosh R, Sharma AK. Determinants of tetanus and sepsis among the last neonatal deaths at household level in a peri-urban area of India. Postgrad Med J. 2011; 87(1026): 257-63

Goyet S, Rammaert B, McCarron M, Khieu V, Fournier I, Kitsutani P, Ly S, Mounts A, Letson WG, Buchy P, Vong S. Mortality in Cambodia An 18-Month Prospective Community-based Surveillance of All-age Deaths Using Verbal Autopsies. Asia Pac J Public Health. 2013; 1010539513

Greece Vital Registration - Deaths 1980 ICD9 as it appears in World Health Organization (WHO). WHO Mortality Database Version September 2016. Geneva, Switzerland: World Health Organization (WHO), 2016

Greece Vital Registration - Deaths 1981 ICD9 as it appears in World Health Organization (WHO). WHO Mortality Database Version September 2016. Geneva, Switzerland: World Health Organization (WHO), 2016

Greece Vital Registration - Deaths 1982 ICD9 as it appears in World Health Organization (WHO). WHO Mortality Database Version September 2016. Geneva, Switzerland: World Health Organization (WHO), 2016

Greece Vital Registration - Deaths 1983 ICD9 as it appears in World Health Organization (WHO). WHO Mortality Database Version September 2016. Geneva, Switzerland: World Health Organization (WHO), 2016

Greece Vital Registration - Deaths 1984 ICD9 as it appears in World Health Organization (WHO). WHO Mortality Database Version September 2016. Geneva, Switzerland: World Health Organization (WHO), 2016

Greece Vital Registration - Deaths 1985 ICD9 as it appears in World Health Organization (WHO). WHO Mortality Database Version September 2016. Geneva, Switzerland: World Health Organization (WHO), 2016

Greece Vital Registration - Deaths 1986 ICD9 as it appears in World Health Organization (WHO). WHO Mortality Database Version September 2016. Geneva, Switzerland: World Health Organization (WHO), 2016

Greece Vital Registration - Deaths 1987 ICD9 as it appears in World Health Organization (WHO). WHO Mortality Database Version November 2015. Geneva, Switzerland: World Health Organization (WHO), 2015

Greece Vital Registration - Deaths 1988 ICD9 as it appears in World Health Organization (WHO). WHO Mortality Database Version November 2015. Geneva, Switzerland: World Health Organization (WHO), 2015

Greece Vital Registration - Deaths 1989 ICD9 as it appears in World Health Organization (WHO). WHO Mortality Database Version November 2015. Geneva, Switzerland: World Health Organization (WHO), 2015

Greece Vital Registration - Deaths 1990 ICD9 as it appears in World Health Organization (WHO). WHO Mortality Database Version November 2015. Geneva, Switzerland: World Health Organization (WHO), 2015

Greece Vital Registration - Deaths 1991 ICD9 as it appears in World Health Organization (WHO). WHO Mortality Database Version November 2015. Geneva, Switzerland: World Health Organization (WHO), 2015

Greece Vital Registration - Deaths 1992 ICD9 as it appears in World Health Organization (WHO). WHO Mortality Database Version November 2015. Geneva, Switzerland: World Health Organization (WHO), 2015

Greece Vital Registration - Deaths 1993 ICD9 as it appears in World Health Organization (WHO). WHO Mortality Database Version November 2015. Geneva, Switzerland: World Health Organization (WHO), 2015

Greece Vital Registration - Deaths 1994 ICD9 as it appears in World Health Organization (WHO). WHO Mortality Database Version November 2015. Geneva, Switzerland: World Health Organization (WHO), 2015
Greece Vital Registration - Deaths 1995 ICD9 as it appears in World Health Organization (WHO). WHO Mortality Database Version November 2015. Geneva, Switzerland: World Health Organization (WHO), 2015
Greece Vital Registration - Deaths 1996 ICD9 as it appears in World Health Organization (WHO). WHO Mortality Database Version November 2015. Geneva, Switzerland: World Health Organization (WHO), 2015
Greece Vital Registration - Deaths 1997 ICD9 as it appears in World Health Organization (WHO). WHO Mortality Database Version November 2015. Geneva, Switzerland: World Health Organization (WHO), 2015
Greece Vital Registration - Deaths 1998 ICD9 as it appears in World Health Organization (WHO). WHO Mortality Database Version November 2015. Geneva, Switzerland: World Health Organization (WHO), 2015
Greece Vital Registration - Deaths 1999 ICD9 as it appears in World Health Organization (WHO). WHO Mortality Database Version November 2015. Geneva, Switzerland: World Health Organization (WHO), 2015
Greece Vital Registration - Deaths 2000 ICD9 as it appears in World Health Organization (WHO). WHO Mortality Database Version November 2015. Geneva, Switzerland: World Health Organization (WHO), 2015
Greece Vital Registration - Deaths 2001 ICD9 as it appears in World Health Organization (WHO). WHO Mortality Database Version November 2015. Geneva, Switzerland: World Health Organization (WHO), 2015
Greece Vital Registration - Deaths 2002 ICD9 as it appears in World Health Organization (WHO). WHO Mortality Database Version November 2015. Geneva, Switzerland: World Health Organization (WHO), 2015
Greece Vital Registration - Deaths 2003 ICD9 as it appears in World Health Organization (WHO). WHO Mortality Database Version November 2015. Geneva, Switzerland: World Health Organization (WHO), 2015
Greece Vital Registration - Deaths 2004 ICD9 as it appears in World Health Organization (WHO). WHO Mortality Database Version November 2015. Geneva, Switzerland: World Health Organization (WHO), 2015
Greece Vital Registration - Deaths 2005 ICD9 as it appears in World Health Organization (WHO). WHO Mortality Database Version November 2015. Geneva, Switzerland: World Health Organization (WHO), 2015
Greece Vital Registration - Deaths 2006 ICD9 as it appears in World Health Organization (WHO). WHO Mortality Database Version November 2015. Geneva, Switzerland: World Health Organization (WHO), 2015
Greece Vital Registration - Deaths 2007 ICD9 as it appears in World Health Organization (WHO). WHO Mortality Database Version September 2016. Geneva, Switzerland: World Health Organization (WHO), 2016
Greece Vital Registration - Deaths 2008 ICD9 as it appears in World Health Organization (WHO). WHO Mortality Database Version November 2015. Geneva, Switzerland: World Health Organization (WHO), 2015
Greece Vital Registration - Deaths 2009 ICD9 as it appears in World Health Organization (WHO). WHO Mortality Database Version September 2016. Geneva, Switzerland: World Health Organization (WHO), 2016
Greece Vital Registration - Deaths 2010 ICD9 as it appears in World Health Organization (WHO). WHO Mortality Database Version September 2016. Geneva, Switzerland: World Health Organization (WHO), 2016
Greece Vital Registration - Deaths 2011 ICD9 as it appears in World Health Organization (WHO). WHO Mortality Database Version September 2016. Geneva, Switzerland: World Health Organization (WHO), 2016

Greece Vital Registration - Deaths 2012 ICD9 as it appears in World Health Organization (WHO). WHO Mortality Database Version September 2016. Geneva, Switzerland: World Health Organization (WHO), 2016

Greece Vital Registration - Deaths 2013 ICD9 as it appears in World Health Organization (WHO). WHO Mortality Database Version September 2016. Geneva, Switzerland: World Health Organization (WHO), 2016

Greece Vital Registration - Deaths 2014 ICD10 as it appears in World Health Organization (WHO). WHO Mortality Database Version March 2017. Geneva, Switzerland: World Health Organization (WHO), 2017

Greece Vital Registration - Deaths 2015 ICD10 as it appears in World Health Organization (WHO). WHO Mortality Database Version April 2018. Geneva, Switzerland: World Health Organization (WHO), 2018

Greenwood AM, Greenwood BM, Bradley AK, Williams K, Shenton FC, Tulloch S, Bypass P, Oldfield FS. A prospective survey of the outcome of pregnancy in a rural area of the Gambia. Bull World Health Organ. 1987; 65(5): 635-43

Grenada Vital Registration - Deaths 1984 ICD9 as it appears in World Health Organization (WHO). WHO Mortality Database Version September 2016. Geneva, Switzerland: World Health Organization (WHO), 2016

Grenada Vital Registration - Deaths 1985 ICD9 as it appears in World Health Organization (WHO). WHO Mortality Database Version September 2016. Geneva, Switzerland: World Health Organization (WHO), 2016

Grenada Vital Registration - Deaths 1988 ICD9 as it appears in World Health Organization (WHO). WHO Mortality Database Version September 2016. Geneva, Switzerland: World Health Organization (WHO), 2016

Grenada Vital Registration - Deaths 1989 ICD9 as it appears in World Health Organization (WHO). WHO Mortality Database Version September 2016. Geneva, Switzerland: World Health Organization (WHO), 2016

Grenada Vital Registration - Deaths 1990 ICD9 as it appears in World Health Organization (WHO). WHO Mortality Database Version September 2016. Geneva, Switzerland: World Health Organization (WHO), 2016

Grenada Vital Registration - Deaths 1991 ICD9 as it appears in World Health Organization (WHO). WHO Mortality Database Version September 2016. Geneva, Switzerland: World Health Organization (WHO), 2016

Grenada Vital Registration - Deaths 1992 ICD9 as it appears in World Health Organization (WHO). WHO Mortality Database Version September 2016. Geneva, Switzerland: World Health Organization (WHO), 2016

Grenada Vital Registration - Deaths 1993 ICD9 as it appears in World Health Organization (WHO). WHO Mortality Database Version September 2016. Geneva, Switzerland: World Health Organization (WHO), 2016

Grenada Vital Registration - Deaths 1994 ICD9 as it appears in World Health Organization (WHO). WHO Mortality Database Version September 2016. Geneva, Switzerland: World Health Organization (WHO), 2016

Grenada Vital Registration - Deaths 1995 ICD9 as it appears in World Health Organization (WHO). WHO Mortality Database Version September 2016. Geneva, Switzerland: World Health Organization (WHO), 2016

Grenada Vital Registration - Deaths 1996 ICD9 as it appears in World Health Organization (WHO). WHO Mortality Database Version September 2016. Geneva, Switzerland: World Health Organization (WHO), 2016

Grenada Vital Registration - Deaths 2001 ICD10 as it appears in World Health Organization (WHO). WHO Mortality Database Version October 2017. Geneva, Switzerland: World Health Organization (WHO), 2017

Grenada Vital Registration - Deaths 2002 ICD10 as it appears in World Health Organization (WHO). WHO Mortality Database Version October 2017. Geneva, Switzerland: World Health Organization (WHO), 2017

Grenada Vital Registration - Deaths 2003 ICD10 as it appears in World Health Organization (WHO). WHO Mortality Database Version October 2017. Geneva, Switzerland: World Health Organization (WHO), 2017
Grenada Vital Registration - Deaths 2004 ICD10 as it appears in World Health Organization (WHO). WHO Mortality Database Version October 2017. Geneva, Switzerland: World Health Organization (WHO), 2017
Grenada Vital Registration - Deaths 2005 ICD10 as it appears in World Health Organization (WHO). WHO Mortality Database Version October 2017. Geneva, Switzerland: World Health Organization (WHO), 2017
Grenada Vital Registration - Deaths 2007 ICD10 as it appears in World Health Organization (WHO). WHO Mortality Database Version October 2017. Geneva, Switzerland: World Health Organization (WHO), 2017
Grenada Vital Registration - Deaths 2006 ICD10 as it appears in World Health Organization (WHO). WHO Mortality Database Version October 2017. Geneva, Switzerland: World Health Organization (WHO), 2017
Grenada Vital Registration - Deaths 2008 ICD10 as it appears in World Health Organization (WHO). WHO Mortality Database Version October 2017. Geneva, Switzerland: World Health Organization (WHO), 2017
Grenada Vital Registration - Deaths 2009 ICD10 as it appears in World Health Organization (WHO). WHO Mortality Database Version October 2017. Geneva, Switzerland: World Health Organization (WHO), 2017
Grenada Vital Registration - Deaths 2010 ICD10 as it appears in World Health Organization (WHO). WHO Mortality Database Version October 2017. Geneva, Switzerland: World Health Organization (WHO), 2017
Grenada Vital Registration - Deaths 2011 ICD10 as it appears in World Health Organization (WHO). WHO Mortality Database Version October 2017. Geneva, Switzerland: World Health Organization (WHO), 2017
Grenada Vital Registration - Deaths 2012 ICD10 as it appears in World Health Organization (WHO). WHO Mortality Database Version October 2017. Geneva, Switzerland: World Health Organization (WHO), 2017
Grenada Vital Registration - Deaths 2013 ICD10 as it appears in World Health Organization (WHO). WHO Mortality Database Version October 2017. Geneva, Switzerland: World Health Organization (WHO), 2017
Grenada Vital Registration - Deaths 2014 ICD10 as it appears in World Health Organization (WHO). WHO Mortality Database Version October 2017. Geneva, Switzerland: World Health Organization (WHO), 2017
Grenada Vital Registration - Deaths 2015 ICD10 as it appears in World Health Organization (WHO). WHO Mortality Database Version October 2017. Geneva, Switzerland: World Health Organization (WHO), 2017
Grenada Vital Registration - Deaths 2016 ICD10 as it appears in World Health Organization (WHO). WHO Mortality Database Version October 2017. Geneva, Switzerland: World Health Organization (WHO), 2017
Guatemala Vital Registration - Deaths 1980 ICD9 as it appears in World Health Organization (WHO). WHO Mortality Database Version September 2016. Geneva, Switzerland: World Health Organization (WHO), 2016
Guatemala Vital Registration - Deaths 1981 ICD9 as it appears in World Health Organization (WHO). WHO Mortality Database Version September 2016. Geneva, Switzerland: World Health Organization (WHO), 2016
Guatemala Vital Registration - Deaths 1984 ICD9 as it appears in World Health Organization (WHO). WHO Mortality Database Version September 2016. Geneva, Switzerland: World Health Organization (WHO), 2016
Guatemala Vital Registration - Deaths 1986 ICD9 as it appears in World Health Organization (WHO). WHO Mortality Database Version September 2016. Geneva, Switzerland: World Health Organization (WHO), 2016

Guatemala Vital Registration - Deaths 1987 ICD9 as it appears in World Health Organization (WHO). WHO Mortality Database Version September 2016. Geneva, Switzerland: World Health Organization (WHO), 2016
Guatemala Vital Registration - Deaths 1988 ICD9 as it appears in World Health Organization (WHO). WHO Mortality Database Version September 2016. Geneva, Switzerland: World Health Organization (WHO), 2016
Guatemala Vital Registration - Deaths 1989 ICD9 as it appears in World Health Organization (WHO). WHO Mortality Database Version September 2016. Geneva, Switzerland: World Health Organization (WHO), 2016
Guatemala Vital Registration - Deaths 1990 ICD9 as it appears in World Health Organization (WHO). WHO Mortality Database Version September 2016. Geneva, Switzerland: World Health Organization (WHO), 2016
Guatemala Vital Registration - Deaths 1991 ICD9 as it appears in World Health Organization (WHO). WHO Mortality Database Version September 2016. Geneva, Switzerland: World Health Organization (WHO), 2016
Guatemala Vital Registration - Deaths 1992 ICD9 as it appears in World Health Organization (WHO). WHO Mortality Database Version September 2016. Geneva, Switzerland: World Health Organization (WHO), 2016
Guatemala Vital Registration - Deaths 1993 ICD9 as it appears in World Health Organization (WHO). WHO Mortality Database Version September 2016. Geneva, Switzerland: World Health Organization (WHO), 2016
Guatemala Vital Registration - Deaths 1994 ICD9 as it appears in World Health Organization (WHO). WHO Mortality Database Version September 2016. Geneva, Switzerland: World Health Organization (WHO), 2016
Guatemala Vital Registration - Deaths 1995 ICD9 as it appears in World Health Organization (WHO). WHO Mortality Database Version September 2016. Geneva, Switzerland: World Health Organization (WHO), 2016
Guatemala Vital Registration - Deaths 1996 ICD9 as it appears in World Health Organization (WHO). WHO Mortality Database Version September 2016. Geneva, Switzerland: World Health Organization (WHO), 2016
Guatemala Vital Registration - Deaths 1997 ICD9 as it appears in World Health Organization (WHO). WHO Mortality Database Version September 2016. Geneva, Switzerland: World Health Organization (WHO), 2016
Guatemala Vital Registration - Deaths 1998 ICD9 as it appears in World Health Organization (WHO). WHO Mortality Database Version September 2016. Geneva, Switzerland: World Health Organization (WHO), 2016
Guatemala Vital Registration - Deaths 1999 ICD9 as it appears in World Health Organization (WHO). WHO Mortality Database Version September 2016. Geneva, Switzerland: World Health Organization (WHO), 2016
Guatemala Vital Registration - Deaths 2000 ICD9 as it appears in World Health Organization (WHO). WHO Mortality Database Version November 2015. Geneva, Switzerland: World Health Organization (WHO), 2015
Guatemala Vital Registration - Deaths 2001 ICD9 as it appears in World Health Organization (WHO). WHO Mortality Database Version November 2015. Geneva, Switzerland: World Health Organization (WHO), 2015
Guatemala Vital Registration - Deaths 2002 ICD9 as it appears in World Health Organization (WHO). WHO Mortality Database Version November 2015. Geneva, Switzerland: World Health Organization (WHO), 2015
Guatemala Vital Registration - Deaths 2003 ICD9 as it appears in World Health Organization (WHO). WHO Mortality Database Version November 2015. Geneva, Switzerland: World Health Organization (WHO), 2015
Guatemala Vital Registration - Deaths 2004 ICD9 as it appears in World Health Organization (WHO). WHO Mortality Database Version November 2015. Geneva, Switzerland: World Health Organization (WHO), 2015

Guatemala Vital Registration - Deaths 2005 ICD10 as it appears in World Health Organization (WHO). WHO Mortality Database Version October 2017. Geneva, Switzerland: World Health Organization (WHO), 2017

Guatemala Vital Registration - Deaths 2006 ICD10 as it appears in World Health Organization (WHO). WHO Mortality Database Version October 2017. Geneva, Switzerland: World Health Organization (WHO), 2017

Guatemala Vital Registration - Deaths 2007 ICD10 as it appears in World Health Organization (WHO). WHO Mortality Database Version October 2017. Geneva, Switzerland: World Health Organization (WHO), 2017

Guatemala Vital Registration - Deaths 2008 ICD10 as it appears in World Health Organization (WHO). WHO Mortality Database Version October 2017. Geneva, Switzerland: World Health Organization (WHO), 2017

Guatemala Vital Registration - Deaths 2009 ICD10 as it appears in World Health Organization (WHO). WHO Mortality Database Version October 2017. Geneva, Switzerland: World Health Organization (WHO), 2017

Guatemala Vital Registration - Deaths 2010 ICD10 as it appears in World Health Organization (WHO). WHO Mortality Database Version October 2017. Geneva, Switzerland: World Health Organization (WHO), 2017

Guatemala Vital Registration - Deaths 2011 ICD10 as it appears in World Health Organization (WHO). WHO Mortality Database Version October 2017. Geneva, Switzerland: World Health Organization (WHO), 2017

Guatemala Vital Registration - Deaths 2012 ICD10 as it appears in World Health Organization (WHO). WHO Mortality Database Version October 2017. Geneva, Switzerland: World Health Organization (WHO), 2017

Guatemala Vital Registration - Deaths 2013 ICD10 as it appears in World Health Organization (WHO). WHO Mortality Database Version October 2017. Geneva, Switzerland: World Health Organization (WHO), 2017

Guatemala Vital Registration - Deaths 2014 ICD10 as it appears in World Health Organization (WHO). WHO Mortality Database Version October 2017. Geneva, Switzerland: World Health Organization (WHO), 2017

Guatemala Vital Registration - Deaths 2015 ICD10 as it appears in World Health Organization (WHO). WHO Mortality Database Version October 2017. Geneva, Switzerland: World Health Organization (WHO), 2017

Gunay T, Kilic B, Keskinoglu P, Kayser Konakci S, Pabuccuoglu O. Infant Mortality Rates in Narlidere District, Turkey (1999 to 2001): Trends in Rates and Risk Factors. Turk J Public Health. 2009; 6(1): 9-18

Gunduz O, Bakar C, Simsek C, Baba A, Elci A, Gurleyuk H, Mutlu M, Cakir A. Statistical Analysis of Death Causes (2005-2010) in Villages with High Arsenic Levels in Drinking Water Supplies of Simav Plain, Turkey. Arch Environ Occup Health. 2014

Guyana Vital Registration - Deaths 1984 ICD9 as it appears in World Health Organization (WHO). WHO Mortality Database Version September 2016. Geneva, Switzerland: World Health Organization (WHO), 2016

Guyana Vital Registration - Deaths 1988 ICD9 as it appears in World Health Organization (WHO). WHO Mortality Database Version September 2016. Geneva, Switzerland: World Health Organization (WHO), 2016

Guyana Vital Registration - Deaths 1989 ICD9 as it appears in World Health Organization (WHO). WHO Mortality Database Version September 2016. Geneva, Switzerland: World Health Organization (WHO), 2016

Guyana Vital Registration - Deaths 1990 ICD9 as it appears in World Health Organization (WHO). WHO Mortality Database Version September 2016. Geneva, Switzerland: World Health Organization (WHO), 2016

Guyana Vital Registration - Deaths 1993 ICD9 as it appears in World Health Organization (WHO). WHO Mortality Database Version September 2016. Geneva, Switzerland: World Health Organization (WHO), 2016

Guyana Vital Registration - Deaths 1994 ICD9 as it appears in World Health Organization (WHO). WHO Mortality Database Version September 2016. Geneva, Switzerland: World Health Organization (WHO), 2016
Guyana Vital Registration - Deaths 1995 ICD9 as it appears in World Health Organization (WHO). WHO Mortality Database Version September 2016. Geneva, Switzerland: World Health Organization (WHO), 2016
Guyana Vital Registration - Deaths 1996 ICD9 as it appears in World Health Organization (WHO). WHO Mortality Database Version September 2016. Geneva, Switzerland: World Health Organization (WHO), 2016
Guyana Vital Registration - Deaths 1997 ICD9 as it appears in World Health Organization (WHO). WHO Mortality Database Version September 2016. Geneva, Switzerland: World Health Organization (WHO), 2016
Guyana Vital Registration - Deaths 1998 ICD9 as it appears in World Health Organization (WHO). WHO Mortality Database Version November 2015. Geneva, Switzerland: World Health Organization (WHO), 2015
Guyana Vital Registration - Deaths 1999 ICD9 as it appears in World Health Organization (WHO). WHO Mortality Database Version November 2015. Geneva, Switzerland: World Health Organization (WHO), 2015
Guyana Vital Registration - Deaths 2001 ICD10 as it appears in World Health Organization (WHO). WHO Mortality Database Version October 2017. Geneva, Switzerland: World Health Organization (WHO), 2017
Guyana Vital Registration - Deaths 2002 ICD10 as it appears in World Health Organization (WHO). WHO Mortality Database Version October 2017. Geneva, Switzerland: World Health Organization (WHO), 2017
Guyana Vital Registration - Deaths 2003 ICD10 as it appears in World Health Organization (WHO). WHO Mortality Database Version October 2017. Geneva, Switzerland: World Health Organization (WHO), 2017
Guyana Vital Registration - Deaths 2004 ICD10 as it appears in World Health Organization (WHO). WHO Mortality Database Version October 2017. Geneva, Switzerland: World Health Organization (WHO), 2017
Guyana Vital Registration - Deaths 2005 ICD10 as it appears in World Health Organization (WHO). WHO Mortality Database Version October 2017. Geneva, Switzerland: World Health Organization (WHO), 2017
Guyana Vital Registration - Deaths 2000 ICD10 - PAHO
Guyana Vital Registration - Deaths 2006 ICD10 as it appears in World Health Organization (WHO). WHO Mortality Database Version October 2017. Geneva, Switzerland: World Health Organization (WHO), 2017
Guyana Vital Registration - Deaths 2007 ICD10 as it appears in World Health Organization (WHO). WHO Mortality Database Version October 2017. Geneva, Switzerland: World Health Organization (WHO), 2017
Guyana Vital Registration - Deaths 2008 ICD10 as it appears in World Health Organization (WHO). WHO Mortality Database Version October 2017. Geneva, Switzerland: World Health Organization (WHO), 2017
Guyana Vital Registration - Deaths 2009 ICD10 as it appears in World Health Organization (WHO). WHO Mortality Database Version October 2017. Geneva, Switzerland: World Health Organization (WHO), 2017
Guyana Vital Registration - Deaths 2010 ICD10 as it appears in World Health Organization (WHO). WHO Mortality Database Version October 2017. Geneva, Switzerland: World Health Organization (WHO), 2017
Guyana Vital Registration - Deaths 2011 ICD10 as it appears in World Health Organization (WHO). WHO Mortality Database Version October 2017. Geneva, Switzerland: World Health Organization (WHO), 2017

Guyana Vital Registration - Deaths 2012 ICD10 as it appears in World Health Organization (WHO). WHO Mortality Database Version October 2017. Geneva, Switzerland: World Health Organization (WHO), 2017

Guyana Vital Registration - Deaths 2013 ICD10 as it appears in World Health Organization (WHO). WHO Mortality Database Version October 2017. Geneva, Switzerland: World Health Organization (WHO), 2017

Halder AK, Gurley ES, Naheed A, Saha SK, Brooks WA, Arifeen SE, Sazzad HMS, Kenah E, Luby SP. Causes of early childhood deaths in urban Dhaka, Bangladesh. PLoS One. 2009; 4(12): e8145

Hanoi School of Public Health, Ministry of Health (Vietnam), School of Population Health, University of Queensland (Australia). Vietnam Burden of Disease and Injury Study 2008

Health Research Centre of Angola. Angola - Dande Health and Demographic Surveillance System

Hieu DT, Hanenberg R, Vach TH, Vinh DQ, Sokal D. Maternal mortality in Vietnam in 1994-95. Stud Fam Plann. 1999; 30(4): 329-38

Hirve S, Ganatra B. A prospective cohort study on the survival experience of under five children in rural western India. Indian Pediatr. 1997; 34(11): 995-1001

Hoa NP, Rao C, Hoy DG, Hinh ND, Chuc NT, Ngo DA. Mortality measures from sample-based surveillance: evidence of the epidemiological transition in Viet Nam. Bull World Health Organ. 2012; 90(10): 764-72

Hoa NP, Rao C, Hoy DG, Hinh ND, Chuc NT, Ngo DA. Mortality measures from sample-based surveillance: evidence of the epidemiological transition in Viet Nam. Bull World Health Organ. 2012; 90(10): 764-72. [Unpublished data]

Honduras Vital Registration - Deaths 1981 ICD9 as it appears in World Health Organization (WHO). WHO Mortality Database Version September 2016. Geneva, Switzerland: World Health Organization (WHO), 2016

Honduras Vital Registration - Deaths 1982 ICD9 as it appears in World Health Organization (WHO). WHO Mortality Database Version September 2016. Geneva, Switzerland: World Health Organization (WHO), 2016

Honduras Vital Registration - Deaths 1987 ICD9 as it appears in World Health Organization (WHO). WHO Mortality Database Version September 2016. Geneva, Switzerland: World Health Organization (WHO), 2016

Honduras Vital Registration - Deaths 1988 ICD9 as it appears in World Health Organization (WHO). WHO Mortality Database Version September 2016. Geneva, Switzerland: World Health Organization (WHO), 2016

Honduras Vital Registration - Deaths 1990 ICD9 as it appears in World Health Organization (WHO). WHO Mortality Database Version September 2016. Geneva, Switzerland: World Health Organization (WHO), 2016

Hong Kong Vital Registration - Deaths 1980 ICD9 as it appears in World Health Organization (WHO). WHO Mortality Database Version September 2016. Geneva, Switzerland: World Health Organization (WHO), 2016

Hong Kong Vital Registration - Deaths 1981 ICD9 as it appears in World Health Organization (WHO). WHO Mortality Database Version September 2016. Geneva, Switzerland: World Health Organization (WHO), 2016

Hong Kong Vital Registration - Deaths 1982 ICD9 as it appears in World Health Organization (WHO). WHO Mortality Database Version September 2016. Geneva, Switzerland: World Health Organization (WHO), 2016

Hong Kong Vital Registration - Deaths 1983 ICD9 as it appears in World Health Organization (WHO). WHO Mortality Database Version September 2016. Geneva, Switzerland: World Health Organization (WHO), 2016

Hong Kong Vital Registration - Deaths 1984 ICD9 as it appears in World Health Organization (WHO). WHO Mortality Database Version September 2016. Geneva, Switzerland: World Health Organization (WHO), 2016

Hong Kong Vital Registration - Deaths 1985 ICD9 as it appears in World Health Organization (WHO). WHO Mortality Database Version September 2016. Geneva, Switzerland: World Health Organization (WHO), 2016
Hong Kong Vital Registration - Deaths 1986 ICD9 as it appears in World Health Organization (WHO). WHO Mortality Database Version September 2016. Geneva, Switzerland: World Health Organization (WHO), 2016
Hong Kong Vital Registration - Deaths 1987 ICD9 as it appears in World Health Organization (WHO). WHO Mortality Database Version September 2016. Geneva, Switzerland: World Health Organization (WHO), 2016
Hong Kong Vital Registration - Deaths 1988 ICD9 as it appears in World Health Organization (WHO). WHO Mortality Database Version September 2016. Geneva, Switzerland: World Health Organization (WHO), 2016
Hong Kong Vital Registration - Deaths 1989 ICD9 as it appears in World Health Organization (WHO). WHO Mortality Database Version September 2016. Geneva, Switzerland: World Health Organization (WHO), 2016
Hong Kong Vital Registration - Deaths 1990 ICD9 as it appears in World Health Organization (WHO). WHO Mortality Database Version September 2016. Geneva, Switzerland: World Health Organization (WHO), 2016
Hong Kong Vital Registration - Deaths 1991 ICD9 as it appears in World Health Organization (WHO). WHO Mortality Database Version September 2016. Geneva, Switzerland: World Health Organization (WHO), 2016
Hong Kong Vital Registration - Deaths 1992 ICD9 as it appears in World Health Organization (WHO). WHO Mortality Database Version September 2016. Geneva, Switzerland: World Health Organization (WHO), 2016
Hong Kong Vital Registration - Deaths 1993 ICD9 as it appears in World Health Organization (WHO). WHO Mortality Database Version September 2016. Geneva, Switzerland: World Health Organization (WHO), 2016
Hong Kong Vital Registration - Deaths 1994 ICD9 as it appears in World Health Organization (WHO). WHO Mortality Database Version November 2015. Geneva, Switzerland: World Health Organization (WHO), 2015
Hong Kong Vital Registration - Deaths 1995 ICD9 as it appears in World Health Organization (WHO). WHO Mortality Database Version November 2015. Geneva, Switzerland: World Health Organization (WHO), 2015
Hong Kong Vital Registration - Deaths 1996 ICD9 as it appears in World Health Organization (WHO). WHO Mortality Database Version November 2015. Geneva, Switzerland: World Health Organization (WHO), 2015
Hong Kong Vital Registration - Deaths 1997 ICD9 as it appears in World Health Organization (WHO). WHO Mortality Database Version November 2015. Geneva, Switzerland: World Health Organization (WHO), 2015
Hong Kong Vital Registration - Deaths 1998 ICD9 as it appears in World Health Organization (WHO). WHO Mortality Database Version November 2015. Geneva, Switzerland: World Health Organization (WHO), 2015
Hong Kong Vital Registration - Deaths 1999 ICD9 as it appears in World Health Organization (WHO). WHO Mortality Database Version November 2015. Geneva, Switzerland: World Health Organization (WHO), 2015
Hong Kong Vital Registration - Deaths 2000 ICD9 as it appears in World Health Organization (WHO). WHO Mortality Database Version November 2015. Geneva, Switzerland: World Health Organization (WHO), 2015
Hong Kong Vital Registration - Deaths 2001 ICD10 as it appears in World Health Organization (WHO). WHO Mortality Database Version October 2017. Geneva, Switzerland: World Health Organization (WHO), 2017
Hong Kong Vital Registration - Deaths 2002 ICD10 as it appears in World Health Organization (WHO). WHO Mortality Database Version October 2017. Geneva, Switzerland: World Health Organization (WHO), 2017

Hong Kong Vital Registration - Deaths 2003 ICD10 as it appears in World Health Organization (WHO). WHO Mortality Database Version October 2017. Geneva, Switzerland: World Health Organization (WHO), 2017

Hong Kong Vital Registration - Deaths 2004 ICD10 as it appears in World Health Organization (WHO). WHO Mortality Database Version October 2017. Geneva, Switzerland: World Health Organization (WHO), 2017

Hong Kong Vital Registration - Deaths 2005 ICD10 as it appears in World Health Organization (WHO). WHO Mortality Database Version October 2017. Geneva, Switzerland: World Health Organization (WHO), 2017

Hong Kong Vital Registration - Deaths 2006 ICD10 as it appears in World Health Organization (WHO). WHO Mortality Database Version October 2017. Geneva, Switzerland: World Health Organization (WHO), 2017

Hong Kong Vital Registration - Deaths 2007 ICD10 as it appears in World Health Organization (WHO). WHO Mortality Database Version October 2017. Geneva, Switzerland: World Health Organization (WHO), 2017

Hong Kong Vital Registration - Deaths 2008 ICD10 as it appears in World Health Organization (WHO). WHO Mortality Database Version October 2017. Geneva, Switzerland: World Health Organization (WHO), 2017

Hong Kong Vital Registration - Deaths 2009 ICD10 as it appears in World Health Organization (WHO). WHO Mortality Database Version October 2017. Geneva, Switzerland: World Health Organization (WHO), 2017

Hong Kong Vital Registration - Deaths 2010 ICD10 as it appears in World Health Organization (WHO). WHO Mortality Database Version October 2017. Geneva, Switzerland: World Health Organization (WHO), 2017

Hong Kong Vital Registration - Deaths 2011 ICD10 as it appears in World Health Organization (WHO). WHO Mortality Database Version October 2017. Geneva, Switzerland: World Health Organization (WHO), 2017

Hong Kong Vital Registration - Deaths 2012 ICD10 as it appears in World Health Organization (WHO). WHO Mortality Database Version October 2017. Geneva, Switzerland: World Health Organization (WHO), 2017

Hong Kong Vital Registration - Deaths 2013 ICD10 as it appears in World Health Organization (WHO). WHO Mortality Database Version October 2017. Geneva, Switzerland: World Health Organization (WHO), 2017

Hong Kong Vital Registration - Deaths 2014 ICD10 as it appears in World Health Organization (WHO). WHO Mortality Database Version April 2018. Geneva, Switzerland: World Health Organization (WHO), 2018

Hong Kong Vital Registration - Deaths 2015 ICD10 as it appears in World Health Organization (WHO). WHO Mortality Database Version April 2018. Geneva, Switzerland: World Health Organization (WHO), 2018

Huang W, Yu H, Wang F, Li G. Infant mortality among various nationalities in the middle part of Guizhou, China. Soc Sci Med. 1997; 45(7): 1031-40

Hungary Vital Registration - Deaths 1980 ICD9 as it appears in World Health Organization (WHO). WHO Mortality Database Version November 2015. Geneva, Switzerland: World Health Organization (WHO), 2015

Hungary Vital Registration - Deaths 1981 ICD9 as it appears in World Health Organization (WHO). WHO Mortality Database Version November 2015. Geneva, Switzerland: World Health Organization (WHO), 2015

Hungary Vital Registration - Deaths 1982 ICD9 as it appears in World Health Organization (WHO). WHO Mortality Database Version November 2015. Geneva, Switzerland: World Health Organization (WHO), 2015

Hungary Vital Registration - Deaths 1983 ICD9 as it appears in World Health Organization (WHO). WHO Mortality Database Version November 2015. Geneva, Switzerland: World Health Organization (WHO), 2015

Hungary Vital Registration - Deaths 1984 ICD9 as it appears in World Health Organization (WHO). WHO Mortality Database Version November 2015. Geneva, Switzerland: World Health Organization (WHO), 2015
Hungary Vital Registration - Deaths 1985 ICD9 as it appears in World Health Organization (WHO). WHO Mortality Database Version November 2015. Geneva, Switzerland: World Health Organization (WHO), 2015
Hungary Vital Registration - Deaths 1986 ICD9 as it appears in World Health Organization (WHO). WHO Mortality Database Version November 2015. Geneva, Switzerland: World Health Organization (WHO), 2015
Hungary Vital Registration - Deaths 1987 ICD9 as it appears in World Health Organization (WHO). WHO Mortality Database Version November 2015. Geneva, Switzerland: World Health Organization (WHO), 2015
Hungary Vital Registration - Deaths 1988 ICD9 as it appears in World Health Organization (WHO). WHO Mortality Database Version November 2015. Geneva, Switzerland: World Health Organization (WHO), 2015
Hungary Vital Registration - Deaths 1989 ICD9 as it appears in World Health Organization (WHO). WHO Mortality Database Version November 2015. Geneva, Switzerland: World Health Organization (WHO), 2015
Hungary Vital Registration - Deaths 1990 ICD9 as it appears in World Health Organization (WHO). WHO Mortality Database Version November 2015. Geneva, Switzerland: World Health Organization (WHO), 2015
Hungary Vital Registration - Deaths 1991 ICD9 as it appears in World Health Organization (WHO). WHO Mortality Database Version November 2015. Geneva, Switzerland: World Health Organization (WHO), 2015
Hungary Vital Registration - Deaths 1992 ICD9 as it appears in World Health Organization (WHO). WHO Mortality Database Version November 2015. Geneva, Switzerland: World Health Organization (WHO), 2015
Hungary Vital Registration - Deaths 1993 ICD9 as it appears in World Health Organization (WHO). WHO Mortality Database Version November 2015. Geneva, Switzerland: World Health Organization (WHO), 2015
Hungary Vital Registration - Deaths 1994 ICD9 as it appears in World Health Organization (WHO). WHO Mortality Database Version November 2015. Geneva, Switzerland: World Health Organization (WHO), 2015
Hungary Vital Registration - Deaths 1995 ICD9 as it appears in World Health Organization (WHO). WHO Mortality Database Version November 2015. Geneva, Switzerland: World Health Organization (WHO), 2015
Hungary Vital Registration - Deaths 1996 ICD10 as it appears in World Health Organization (WHO). WHO Mortality Database Version October 2017. Geneva, Switzerland: World Health Organization (WHO), 2017
Hungary Vital Registration - Deaths 1997 ICD10 as it appears in World Health Organization (WHO). WHO Mortality Database Version October 2017. Geneva, Switzerland: World Health Organization (WHO), 2017
Hungary Vital Registration - Deaths 1998 ICD10 as it appears in World Health Organization (WHO). WHO Mortality Database Version October 2017. Geneva, Switzerland: World Health Organization (WHO), 2017
Hungary Vital Registration - Deaths 1999 ICD10 as it appears in World Health Organization (WHO). WHO Mortality Database Version October 2017. Geneva, Switzerland: World Health Organization (WHO), 2017
Hungary Vital Registration - Deaths 2000 ICD10 as it appears in World Health Organization (WHO). WHO Mortality Database Version October 2017. Geneva, Switzerland: World Health Organization (WHO), 2017
Hungary Vital Registration - Deaths 2001 ICD10 as it appears in World Health Organization (WHO). WHO Mortality Database Version October 2017. Geneva, Switzerland: World Health Organization (WHO), 2017

Hungary Vital Registration - Deaths 2002 ICD10 as it appears in World Health Organization (WHO). WHO Mortality Database Version October 2017. Geneva, Switzerland: World Health Organization (WHO), 2017
Hungary Vital Registration - Deaths 2003 ICD10 as it appears in World Health Organization (WHO). WHO Mortality Database Version October 2017. Geneva, Switzerland: World Health Organization (WHO), 2017
Hungary Vital Registration - Deaths 2004 ICD10 as it appears in World Health Organization (WHO). WHO Mortality Database Version October 2017. Geneva, Switzerland: World Health Organization (WHO), 2017
Hungary Vital Registration - Deaths 2005 ICD10 as it appears in World Health Organization (WHO). WHO Mortality Database Version October 2017. Geneva, Switzerland: World Health Organization (WHO), 2017
Hungary Vital Registration - Deaths 2006 ICD10 as it appears in World Health Organization (WHO). WHO Mortality Database Version October 2017. Geneva, Switzerland: World Health Organization (WHO), 2017
Hungary Vital Registration - Deaths 2007 ICD10 as it appears in World Health Organization (WHO). WHO Mortality Database Version October 2017. Geneva, Switzerland: World Health Organization (WHO), 2017
Hungary Vital Registration - Deaths 2008 ICD10 as it appears in World Health Organization (WHO). WHO Mortality Database Version October 2017. Geneva, Switzerland: World Health Organization (WHO), 2017
Hungary Vital Registration - Deaths 2009 ICD10 as it appears in World Health Organization (WHO). WHO Mortality Database Version October 2017. Geneva, Switzerland: World Health Organization (WHO), 2017
Hungary Vital Registration - Deaths 2010 ICD10 as it appears in World Health Organization (WHO). WHO Mortality Database Version October 2017. Geneva, Switzerland: World Health Organization (WHO), 2017
Hungary Vital Registration - Deaths 2011 ICD10 as it appears in World Health Organization (WHO). WHO Mortality Database Version October 2017. Geneva, Switzerland: World Health Organization (WHO), 2017
Hungary Vital Registration - Deaths 2012 ICD10 as it appears in World Health Organization (WHO). WHO Mortality Database Version October 2017. Geneva, Switzerland: World Health Organization (WHO), 2017
Hungary Vital Registration - Deaths 2013 ICD10 as it appears in World Health Organization (WHO). WHO Mortality Database Version October 2017. Geneva, Switzerland: World Health Organization (WHO), 2017
Hungary Vital Registration - Deaths 2014 ICD10 as it appears in World Health Organization (WHO). WHO Mortality Database Version October 2017. Geneva, Switzerland: World Health Organization (WHO), 2017
Hungary Vital Registration - Deaths 2015 ICD10 as it appears in World Health Organization (WHO). WHO Mortality Database Version March 2017. Geneva, Switzerland: World Health Organization (WHO), 2017
Hungary Vital Registration - Deaths 2016 ICD10 as it appears in World Health Organization (WHO). WHO Mortality Database Version April 2018. Geneva, Switzerland: World Health Organization (WHO), 2018
Iceland Vital Registration - Deaths 1980 ICD8 as it appears in World Health Organization (WHO). WHO Mortality Database Version July 2012. Geneva, Switzerland: World Health Organization (WHO), 2012
Iceland Vital Registration - Deaths 1981 ICD9 as it appears in World Health Organization (WHO). WHO Mortality Database Version September 2016. Geneva, Switzerland: World Health Organization (WHO), 2016
Iceland Vital Registration - Deaths 1982 ICD9 as it appears in World Health Organization (WHO). WHO Mortality Database Version September 2016. Geneva, Switzerland: World Health Organization (WHO), 2016

Iceland Vital Registration - Deaths 1983 ICD9 as it appears in World Health Organization (WHO). WHO Mortality Database Version September 2016. Geneva, Switzerland: World Health Organization (WHO), 2016
Iceland Vital Registration - Deaths 1984 ICD9 as it appears in World Health Organization (WHO). WHO Mortality Database Version September 2016. Geneva, Switzerland: World Health Organization (WHO), 2016
Iceland Vital Registration - Deaths 1985 ICD9 as it appears in World Health Organization (WHO). WHO Mortality Database Version September 2016. Geneva, Switzerland: World Health Organization (WHO), 2016
Iceland Vital Registration - Deaths 1986 ICD9 as it appears in World Health Organization (WHO). WHO Mortality Database Version September 2016. Geneva, Switzerland: World Health Organization (WHO), 2016
Iceland Vital Registration - Deaths 1987 ICD9 as it appears in World Health Organization (WHO). WHO Mortality Database Version September 2016. Geneva, Switzerland: World Health Organization (WHO), 2016
Iceland Vital Registration - Deaths 1988 ICD9 as it appears in World Health Organization (WHO). WHO Mortality Database Version September 2016. Geneva, Switzerland: World Health Organization (WHO), 2016
Iceland Vital Registration - Deaths 1989 ICD9 as it appears in World Health Organization (WHO). WHO Mortality Database Version November 2015. Geneva, Switzerland: World Health Organization (WHO), 2015
Iceland Vital Registration - Deaths 1990 ICD9 as it appears in World Health Organization (WHO). WHO Mortality Database Version November 2015. Geneva, Switzerland: World Health Organization (WHO), 2015
Iceland Vital Registration - Deaths 1991 ICD9 as it appears in World Health Organization (WHO). WHO Mortality Database Version November 2015. Geneva, Switzerland: World Health Organization (WHO), 2015
Iceland Vital Registration - Deaths 1992 ICD9 as it appears in World Health Organization (WHO). WHO Mortality Database Version November 2015. Geneva, Switzerland: World Health Organization (WHO), 2015
Iceland Vital Registration - Deaths 1993 ICD9 as it appears in World Health Organization (WHO). WHO Mortality Database Version November 2015. Geneva, Switzerland: World Health Organization (WHO), 2015
Iceland Vital Registration - Deaths 1994 ICD9 as it appears in World Health Organization (WHO). WHO Mortality Database Version November 2015. Geneva, Switzerland: World Health Organization (WHO), 2015
Iceland Vital Registration - Deaths 1995 ICD9 as it appears in World Health Organization (WHO). WHO Mortality Database Version November 2015. Geneva, Switzerland: World Health Organization (WHO), 2015
Iceland Vital Registration - Deaths 1996 ICD10 as it appears in World Health Organization (WHO). WHO Mortality Database Version October 2017. Geneva, Switzerland: World Health Organization (WHO), 2017
Iceland Vital Registration - Deaths 1997 ICD10 as it appears in World Health Organization (WHO). WHO Mortality Database Version October 2017. Geneva, Switzerland: World Health Organization (WHO), 2017
Iceland Vital Registration - Deaths 1998 ICD10 as it appears in World Health Organization (WHO). WHO Mortality Database Version October 2017. Geneva, Switzerland: World Health Organization (WHO), 2017
Iceland Vital Registration - Deaths 1999 ICD10 as it appears in World Health Organization (WHO). WHO Mortality Database Version October 2017. Geneva, Switzerland: World Health Organization (WHO), 2017
Iceland Vital Registration - Deaths 2000 ICD10 as it appears in World Health Organization (WHO). WHO Mortality Database Version October 2017. Geneva, Switzerland: World Health Organization (WHO), 2017

Iceland Vital Registration - Deaths 2001 ICD10 as it appears in World Health Organization (WHO). WHO Mortality Database Version October 2017. Geneva, Switzerland: World Health Organization (WHO), 2017
Iceland Vital Registration - Deaths 2002 ICD10 as it appears in World Health Organization (WHO). WHO Mortality Database Version October 2017. Geneva, Switzerland: World Health Organization (WHO), 2017
Iceland Vital Registration - Deaths 2003 ICD10 as it appears in World Health Organization (WHO). WHO Mortality Database Version October 2017. Geneva, Switzerland: World Health Organization (WHO), 2017
Iceland Vital Registration - Deaths 2004 ICD10 as it appears in World Health Organization (WHO). WHO Mortality Database Version October 2017. Geneva, Switzerland: World Health Organization (WHO), 2017
Iceland Vital Registration - Deaths 2005 ICD10 as it appears in World Health Organization (WHO). WHO Mortality Database Version October 2017. Geneva, Switzerland: World Health Organization (WHO), 2017
Iceland Vital Registration - Deaths 2006 ICD10 as it appears in World Health Organization (WHO). WHO Mortality Database Version October 2017. Geneva, Switzerland: World Health Organization (WHO), 2017
Iceland Vital Registration - Deaths 2007 ICD10 as it appears in World Health Organization (WHO). WHO Mortality Database Version October 2017. Geneva, Switzerland: World Health Organization (WHO), 2017
Iceland Vital Registration - Deaths 2008 ICD10 as it appears in World Health Organization (WHO). WHO Mortality Database Version October 2017. Geneva, Switzerland: World Health Organization (WHO), 2017
Iceland Vital Registration - Deaths 2009 ICD10 as it appears in World Health Organization (WHO). WHO Mortality Database Version October 2017. Geneva, Switzerland: World Health Organization (WHO), 2017
Iceland Vital Registration - Deaths 2010 ICD10 as it appears in World Health Organization (WHO). WHO Mortality Database Version March 2017. Geneva, Switzerland: World Health Organization (WHO), 2017
Iceland Vital Registration - Deaths 2011 ICD10 as it appears in World Health Organization (WHO). WHO Mortality Database Version March 2017. Geneva, Switzerland: World Health Organization (WHO), 2017
Iceland Vital Registration - Deaths 2012 ICD10 as it appears in World Health Organization (WHO). WHO Mortality Database Version March 2017. Geneva, Switzerland: World Health Organization (WHO), 2017
Iceland Vital Registration - Deaths 2013 ICD10 as it appears in World Health Organization (WHO). WHO Mortality Database Version March 2017. Geneva, Switzerland: World Health Organization (WHO), 2017
Iceland Vital Registration - Deaths 2014 ICD10 as it appears in World Health Organization (WHO). WHO Mortality Database Version March 2017. Geneva, Switzerland: World Health Organization (WHO), 2017
Iceland Vital Registration - Deaths 2015 ICD10 as it appears in World Health Organization (WHO). WHO Mortality Database Version March 2017. Geneva, Switzerland: World Health Organization (WHO), 2017
Iceland Vital Registration - Deaths 2016 ICD10 as it appears in World Health Organization (WHO). WHO Mortality Database Version October 2017. Geneva, Switzerland: World Health Organization (WHO), 2017
INDEPTH, International Centre for Diarrhoeal Disease Research, Bangladesh (ICDDR,B). Bangladesh - Matlab Health and Demographic Surveillance System
INDEPTH, Nouna Health Research Center (CRSN)(Burkina Faso). Burkina Faso - Nouna Health and Demographic Surveillance System
Indian Council of Medical Research (ICMR). India Study on Causes of Death by Verbal Autopsy 2003

International Centre for Diarrhoeal Disease Research, Bangladesh (ICDDR,B). Bangladesh - Chandpur and Comilla District Verbal Autopsy Study 2011-2014

Iraq Vital Registration - Deaths 2008 ICD10 as it appears in World Health Organization (WHO). WHO Mortality Database Version October 2017. Geneva, Switzerland: World Health Organization (WHO), 2017

Ireland Vital Registration - Deaths 1980 ICD9 as it appears in World Health Organization (WHO). WHO Mortality Database Version November 2015. Geneva, Switzerland: World Health Organization (WHO), 2015

Ireland Vital Registration - Deaths 1981 ICD9 as it appears in World Health Organization (WHO). WHO Mortality Database Version November 2015. Geneva, Switzerland: World Health Organization (WHO), 2015

Ireland Vital Registration - Deaths 1982 ICD9 as it appears in World Health Organization (WHO). WHO Mortality Database Version November 2015. Geneva, Switzerland: World Health Organization (WHO), 2015

Ireland Vital Registration - Deaths 1983 ICD9 as it appears in World Health Organization (WHO). WHO Mortality Database Version November 2015. Geneva, Switzerland: World Health Organization (WHO), 2015

Ireland Vital Registration - Deaths 1984 ICD9 as it appears in World Health Organization (WHO). WHO Mortality Database Version November 2015. Geneva, Switzerland: World Health Organization (WHO), 2015

Ireland Vital Registration - Deaths 1985 ICD9 as it appears in World Health Organization (WHO). WHO Mortality Database Version November 2015. Geneva, Switzerland: World Health Organization (WHO), 2015

Ireland Vital Registration - Deaths 1986 ICD9 as it appears in World Health Organization (WHO). WHO Mortality Database Version November 2015. Geneva, Switzerland: World Health Organization (WHO), 2015

Ireland Vital Registration - Deaths 1987 ICD9 as it appears in World Health Organization (WHO). WHO Mortality Database Version November 2015. Geneva, Switzerland: World Health Organization (WHO), 2015

Ireland Vital Registration - Deaths 1988 ICD9 as it appears in World Health Organization (WHO). WHO Mortality Database Version November 2015. Geneva, Switzerland: World Health Organization (WHO), 2015

Ireland Vital Registration - Deaths 1989 ICD9 as it appears in World Health Organization (WHO). WHO Mortality Database Version November 2015. Geneva, Switzerland: World Health Organization (WHO), 2015

Ireland Vital Registration - Deaths 1990 ICD9 as it appears in World Health Organization (WHO). WHO Mortality Database Version November 2015. Geneva, Switzerland: World Health Organization (WHO), 2015

Ireland Vital Registration - Deaths 1991 ICD9 as it appears in World Health Organization (WHO). WHO Mortality Database Version November 2015. Geneva, Switzerland: World Health Organization (WHO), 2015

Ireland Vital Registration - Deaths 1992 ICD9 as it appears in World Health Organization (WHO). WHO Mortality Database Version November 2015. Geneva, Switzerland: World Health Organization (WHO), 2015

Ireland Vital Registration - Deaths 1993 ICD9 as it appears in World Health Organization (WHO). WHO Mortality Database Version November 2015. Geneva, Switzerland: World Health Organization (WHO), 2015

Ireland Vital Registration - Deaths 1994 ICD9 as it appears in World Health Organization (WHO). WHO Mortality Database Version November 2015. Geneva, Switzerland: World Health Organization (WHO), 2015

Ireland Vital Registration - Deaths 1995 ICD9 as it appears in World Health Organization (WHO). WHO Mortality Database Version November 2015. Geneva, Switzerland: World Health Organization (WHO), 2015

Ireland Vital Registration - Deaths 1996 ICD9 as it appears in World Health Organization (WHO). WHO Mortality Database Version November 2015. Geneva, Switzerland: World Health Organization (WHO), 2015
Ireland Vital Registration - Deaths 1997 ICD9 as it appears in World Health Organization (WHO). WHO Mortality Database Version November 2015. Geneva, Switzerland: World Health Organization (WHO), 2015
Ireland Vital Registration - Deaths 1998 ICD9 as it appears in World Health Organization (WHO). WHO Mortality Database Version November 2015. Geneva, Switzerland: World Health Organization (WHO), 2015
Ireland Vital Registration - Deaths 1999 ICD9 as it appears in World Health Organization (WHO). WHO Mortality Database Version November 2015. Geneva, Switzerland: World Health Organization (WHO), 2015
Ireland Vital Registration - Deaths 2000 ICD9 as it appears in World Health Organization (WHO). WHO Mortality Database Version November 2015. Geneva, Switzerland: World Health Organization (WHO), 2015
Ireland Vital Registration - Deaths 2001 ICD9 as it appears in World Health Organization (WHO). WHO Mortality Database Version November 2015. Geneva, Switzerland: World Health Organization (WHO), 2015
Ireland Vital Registration - Deaths 2002 ICD9 as it appears in World Health Organization (WHO). WHO Mortality Database Version November 2015. Geneva, Switzerland: World Health Organization (WHO), 2015
Ireland Vital Registration - Deaths 2003 ICD9 as it appears in World Health Organization (WHO). WHO Mortality Database Version November 2015. Geneva, Switzerland: World Health Organization (WHO), 2015
Ireland Vital Registration - Deaths 2004 ICD9 as it appears in World Health Organization (WHO). WHO Mortality Database Version November 2015. Geneva, Switzerland: World Health Organization (WHO), 2015
Ireland Vital Registration - Deaths 2005 ICD9 as it appears in World Health Organization (WHO). WHO Mortality Database Version November 2015. Geneva, Switzerland: World Health Organization (WHO), 2015
Ireland Vital Registration - Deaths 2006 ICD9 as it appears in World Health Organization (WHO). WHO Mortality Database Version September 2016. Geneva, Switzerland: World Health Organization (WHO), 2016
Ireland Vital Registration - Deaths 2007 ICD10 as it appears in World Health Organization (WHO). WHO Mortality Database Version October 2017. Geneva, Switzerland: World Health Organization (WHO), 2017
Ireland Vital Registration - Deaths 2008 ICD10 as it appears in World Health Organization (WHO). WHO Mortality Database Version October 2017. Geneva, Switzerland: World Health Organization (WHO), 2017
Ireland Vital Registration - Deaths 2009 ICD10 as it appears in World Health Organization (WHO). WHO Mortality Database Version October 2017. Geneva, Switzerland: World Health Organization (WHO), 2017
Ireland Vital Registration - Deaths 2010 ICD10 as it appears in World Health Organization (WHO). WHO Mortality Database Version October 2017. Geneva, Switzerland: World Health Organization (WHO), 2017
Ireland Vital Registration - Deaths 2011 ICD10 as it appears in World Health Organization (WHO). WHO Mortality Database Version October 2017. Geneva, Switzerland: World Health Organization (WHO), 2017
Ireland Vital Registration - Deaths 2012 ICD10 as it appears in World Health Organization (WHO). WHO Mortality Database Version October 2017. Geneva, Switzerland: World Health Organization (WHO), 2017
Ireland Vital Registration - Deaths 2013 ICD10 as it appears in World Health Organization (WHO). WHO Mortality Database Version October 2017. Geneva, Switzerland: World Health Organization (WHO), 2017

Ireland Vital Registration - Deaths 2014 ICD10 as it appears in World Health Organization (WHO). WHO Mortality Database Version October 2017. Geneva, Switzerland: World Health Organization (WHO), 2017
Israel Vital Registration - Deaths 1980 ICD9 as it appears in World Health Organization (WHO). WHO Mortality Database Version September 2016. Geneva, Switzerland: World Health Organization (WHO), 2016
Israel Vital Registration - Deaths 1981 ICD9 as it appears in World Health Organization (WHO). WHO Mortality Database Version September 2016. Geneva, Switzerland: World Health Organization (WHO), 2016
Israel Vital Registration - Deaths 1982 ICD9 as it appears in World Health Organization (WHO). WHO Mortality Database Version September 2016. Geneva, Switzerland: World Health Organization (WHO), 2016
Israel Vital Registration - Deaths 1983 ICD9 as it appears in World Health Organization (WHO). WHO Mortality Database Version September 2016. Geneva, Switzerland: World Health Organization (WHO), 2016
Israel Vital Registration - Deaths 1984 ICD9 as it appears in World Health Organization (WHO). WHO Mortality Database Version September 2016. Geneva, Switzerland: World Health Organization (WHO), 2016
Israel Vital Registration - Deaths 1985 ICD9 as it appears in World Health Organization (WHO). WHO Mortality Database Version September 2016. Geneva, Switzerland: World Health Organization (WHO), 2016
Israel Vital Registration - Deaths 1986 ICD9 as it appears in World Health Organization (WHO). WHO Mortality Database Version September 2016. Geneva, Switzerland: World Health Organization (WHO), 2016
Israel Vital Registration - Deaths 1987 ICD9 as it appears in World Health Organization (WHO). WHO Mortality Database Version September 2016. Geneva, Switzerland: World Health Organization (WHO), 2016
Israel Vital Registration - Deaths 1988 ICD9 as it appears in World Health Organization (WHO). WHO Mortality Database Version September 2016. Geneva, Switzerland: World Health Organization (WHO), 2016
Israel Vital Registration - Deaths 1989 ICD9 as it appears in World Health Organization (WHO). WHO Mortality Database Version September 2016. Geneva, Switzerland: World Health Organization (WHO), 2016
Israel Vital Registration - Deaths 1990 ICD9 as it appears in World Health Organization (WHO). WHO Mortality Database Version September 2016. Geneva, Switzerland: World Health Organization (WHO), 2016
Israel Vital Registration - Deaths 1991 ICD9 as it appears in World Health Organization (WHO). WHO Mortality Database Version September 2016. Geneva, Switzerland: World Health Organization (WHO), 2016
Israel Vital Registration - Deaths 1992 ICD9 as it appears in World Health Organization (WHO). WHO Mortality Database Version September 2016. Geneva, Switzerland: World Health Organization (WHO), 2016
Israel Vital Registration - Deaths 1993 ICD9 as it appears in World Health Organization (WHO). WHO Mortality Database Version September 2016. Geneva, Switzerland: World Health Organization (WHO), 2016
Israel Vital Registration - Deaths 1994 ICD9 as it appears in World Health Organization (WHO). WHO Mortality Database Version September 2016. Geneva, Switzerland: World Health Organization (WHO), 2016
Israel Vital Registration - Deaths 1995 ICD9 as it appears in World Health Organization (WHO). WHO Mortality Database Version November 2015. Geneva, Switzerland: World Health Organization (WHO), 2015
Israel Vital Registration - Deaths 1996 ICD9 as it appears in World Health Organization (WHO). WHO Mortality Database Version November 2015. Geneva, Switzerland: World Health Organization (WHO), 2015

Israel Vital Registration - Deaths 1997 ICD9 as it appears in World Health Organization (WHO). WHO Mortality Database Version November 2015. Geneva, Switzerland: World Health Organization (WHO), 2015
Israel Vital Registration - Deaths 1998 ICD10 as it appears in World Health Organization (WHO). WHO Mortality Database Version October 2017. Geneva, Switzerland: World Health Organization (WHO), 2017
Israel Vital Registration - Deaths 1999 ICD10 as it appears in World Health Organization (WHO). WHO Mortality Database Version October 2017. Geneva, Switzerland: World Health Organization (WHO), 2017
Israel Vital Registration - Deaths 2000 ICD10 as it appears in World Health Organization (WHO). WHO Mortality Database Version October 2017. Geneva, Switzerland: World Health Organization (WHO), 2017
Israel Vital Registration - Deaths 2001 ICD10 as it appears in World Health Organization (WHO). WHO Mortality Database Version October 2017. Geneva, Switzerland: World Health Organization (WHO), 2017
Israel Vital Registration - Deaths 2002 ICD10 as it appears in World Health Organization (WHO). WHO Mortality Database Version October 2017. Geneva, Switzerland: World Health Organization (WHO), 2017
Israel Vital Registration - Deaths 2003 ICD10 as it appears in World Health Organization (WHO). WHO Mortality Database Version October 2017. Geneva, Switzerland: World Health Organization (WHO), 2017
Israel Vital Registration - Deaths 2004 ICD10 as it appears in World Health Organization (WHO). WHO Mortality Database Version October 2017. Geneva, Switzerland: World Health Organization (WHO), 2017
Israel Vital Registration - Deaths 2005 ICD10 as it appears in World Health Organization (WHO). WHO Mortality Database Version October 2017. Geneva, Switzerland: World Health Organization (WHO), 2017
Israel Vital Registration - Deaths 2006 ICD10 as it appears in World Health Organization (WHO). WHO Mortality Database Version October 2017. Geneva, Switzerland: World Health Organization (WHO), 2017
Israel Vital Registration - Deaths 2007 ICD10 as it appears in World Health Organization (WHO). WHO Mortality Database Version October 2017. Geneva, Switzerland: World Health Organization (WHO), 2017
Israel Vital Registration - Deaths 2008 ICD10 as it appears in World Health Organization (WHO). WHO Mortality Database Version October 2017. Geneva, Switzerland: World Health Organization (WHO), 2017
Israel Vital Registration - Deaths 2009 ICD10 as it appears in World Health Organization (WHO). WHO Mortality Database Version October 2017. Geneva, Switzerland: World Health Organization (WHO), 2017
Israel Vital Registration - Deaths 2010 ICD10 as it appears in World Health Organization (WHO). WHO Mortality Database Version October 2017. Geneva, Switzerland: World Health Organization (WHO), 2017
Israel Vital Registration - Deaths 2011 ICD10 as it appears in World Health Organization (WHO). WHO Mortality Database Version October 2017. Geneva, Switzerland: World Health Organization (WHO), 2017
Israel Vital Registration - Deaths 2012 ICD10 as it appears in World Health Organization (WHO). WHO Mortality Database Version October 2017. Geneva, Switzerland: World Health Organization (WHO), 2017
Israel Vital Registration - Deaths 2013 ICD10 as it appears in World Health Organization (WHO). WHO Mortality Database Version October 2017. Geneva, Switzerland: World Health Organization (WHO), 2017
Israel Vital Registration - Deaths 2014 ICD10 as it appears in World Health Organization (WHO). WHO Mortality Database Version March 2017. Geneva, Switzerland: World Health Organization (WHO), 2017

Israel Vital Registration - Deaths 2015 ICD10 as it appears in World Health Organization (WHO). WHO Mortality Database Version April 2018. Geneva, Switzerland: World Health Organization (WHO), 2018
Italy Vital Registration - Deaths 1980 ICD9 as it appears in World Health Organization (WHO). WHO Mortality Database Version November 2015. Geneva, Switzerland: World Health Organization (WHO), 2015
Italy Vital Registration - Deaths 1981 ICD9 as it appears in World Health Organization (WHO). WHO Mortality Database Version November 2015. Geneva, Switzerland: World Health Organization (WHO), 2015
Italy Vital Registration - Deaths 1982 ICD9 as it appears in World Health Organization (WHO). WHO Mortality Database Version November 2015. Geneva, Switzerland: World Health Organization (WHO), 2015
Italy Vital Registration - Deaths 1983 ICD9 as it appears in World Health Organization (WHO). WHO Mortality Database Version November 2015. Geneva, Switzerland: World Health Organization (WHO), 2015
Italy Vital Registration - Deaths 1984 ICD9 as it appears in World Health Organization (WHO). WHO Mortality Database Version November 2015. Geneva, Switzerland: World Health Organization (WHO), 2015
Italy Vital Registration - Deaths 1985 ICD9 as it appears in World Health Organization (WHO). WHO Mortality Database Version November 2015. Geneva, Switzerland: World Health Organization (WHO), 2015
Italy Vital Registration - Deaths 1986 ICD9 as it appears in World Health Organization (WHO). WHO Mortality Database Version November 2015. Geneva, Switzerland: World Health Organization (WHO), 2015
Italy Vital Registration - Deaths 1987 ICD9 as it appears in World Health Organization (WHO). WHO Mortality Database Version November 2015. Geneva, Switzerland: World Health Organization (WHO), 2015
Italy Vital Registration - Deaths 1988 ICD9 as it appears in World Health Organization (WHO). WHO Mortality Database Version November 2015. Geneva, Switzerland: World Health Organization (WHO), 2015
Italy Vital Registration - Deaths 1989 ICD9 as it appears in World Health Organization (WHO). WHO Mortality Database Version November 2015. Geneva, Switzerland: World Health Organization (WHO), 2015
Italy Vital Registration - Deaths 1990 ICD9 as it appears in World Health Organization (WHO). WHO Mortality Database Version November 2015. Geneva, Switzerland: World Health Organization (WHO), 2015
Italy Vital Registration - Deaths 1991 ICD9 as it appears in World Health Organization (WHO). WHO Mortality Database Version November 2015. Geneva, Switzerland: World Health Organization (WHO), 2015
Italy Vital Registration - Deaths 1992 ICD9 as it appears in World Health Organization (WHO). WHO Mortality Database Version November 2015. Geneva, Switzerland: World Health Organization (WHO), 2015
Italy Vital Registration - Deaths 1993 ICD9 as it appears in World Health Organization (WHO). WHO Mortality Database Version November 2015. Geneva, Switzerland: World Health Organization (WHO), 2015
Italy Vital Registration - Deaths 1994 ICD9 as it appears in World Health Organization (WHO). WHO Mortality Database Version November 2015. Geneva, Switzerland: World Health Organization (WHO), 2015
Italy Vital Registration - Deaths 1995 ICD9 as it appears in World Health Organization (WHO). WHO Mortality Database Version November 2015. Geneva, Switzerland: World Health Organization (WHO), 2015
Italy Vital Registration - Deaths 1996 ICD9 as it appears in World Health Organization (WHO). WHO Mortality Database Version November 2015. Geneva, Switzerland: World Health Organization (WHO), 2015

Italy Vital Registration - Deaths 1997 ICD9 as it appears in World Health Organization (WHO). WHO Mortality Database Version November 2015. Geneva, Switzerland: World Health Organization (WHO), 2015
Italy Vital Registration - Deaths 1998 ICD9 as it appears in World Health Organization (WHO). WHO Mortality Database Version November 2015. Geneva, Switzerland: World Health Organization (WHO), 2015
Italy Vital Registration - Deaths 1999 ICD9 as it appears in World Health Organization (WHO). WHO Mortality Database Version November 2015. Geneva, Switzerland: World Health Organization (WHO), 2015
Italy Vital Registration - Deaths 2000 ICD9 as it appears in World Health Organization (WHO). WHO Mortality Database Version November 2015. Geneva, Switzerland: World Health Organization (WHO), 2015
Italy Vital Registration - Deaths 2001 ICD9 as it appears in World Health Organization (WHO). WHO Mortality Database Version November 2015. Geneva, Switzerland: World Health Organization (WHO), 2015
Italy Vital Registration - Deaths 2002 ICD9 as it appears in World Health Organization (WHO). WHO Mortality Database Version November 2015. Geneva, Switzerland: World Health Organization (WHO), 2015
Italy Vital Registration - Deaths 2003 ICD10 as it appears in World Health Organization (WHO). WHO Mortality Database Version October 2017. Geneva, Switzerland: World Health Organization (WHO), 2017
Italy Vital Registration - Deaths 2006 ICD10 as it appears in World Health Organization (WHO). WHO Mortality Database Version October 2017. Geneva, Switzerland: World Health Organization (WHO), 2017
Italy Vital Registration - Deaths 2007 ICD10 as it appears in World Health Organization (WHO). WHO Mortality Database Version October 2017. Geneva, Switzerland: World Health Organization (WHO), 2017
Italy Vital Registration - Deaths 2004 ICD10 as it appears in World Health Organization (WHO). WHO Mortality Database Version October 2017. Geneva, Switzerland: World Health Organization (WHO), 2017
Italy Vital Registration - Deaths 2005 ICD10 as it appears in World Health Organization (WHO). WHO Mortality Database Version October 2017. Geneva, Switzerland: World Health Organization (WHO), 2017
Italy Vital Registration - Deaths 2008 ICD10 as it appears in World Health Organization (WHO). WHO Mortality Database Version October 2017. Geneva, Switzerland: World Health Organization (WHO), 2017
Italy Vital Registration - Deaths 2009 ICD10 as it appears in World Health Organization (WHO). WHO Mortality Database Version October 2017. Geneva, Switzerland: World Health Organization (WHO), 2017
Italy Vital Registration - Deaths 2010 ICD10 as it appears in World Health Organization (WHO). WHO Mortality Database Version October 2017. Geneva, Switzerland: World Health Organization (WHO), 2017
Italy Vital Registration - Deaths 2011 ICD10 as it appears in World Health Organization (WHO). WHO Mortality Database Version October 2017. Geneva, Switzerland: World Health Organization (WHO), 2017
Italy Vital Registration - Deaths 2012 ICD10 as it appears in World Health Organization (WHO). WHO Mortality Database Version October 2017. Geneva, Switzerland: World Health Organization (WHO), 2017
Italy Vital Registration - Deaths 2013 ICD10 as it appears in World Health Organization (WHO). WHO Mortality Database Version October 2017. Geneva, Switzerland: World Health Organization (WHO), 2017
Italy Vital Registration - Deaths 2014 ICD10 as it appears in World Health Organization (WHO). WHO Mortality Database Version October 2017. Geneva, Switzerland: World Health Organization (WHO), 2017

Jain Y, Bansal M, Tiwari R, Kasar PK. Causes of Neonatal Mortality: A community based study using verbal autopsy tool. Natl J Community Med. 2013; 4(3)

Jamaica Vital Registration - Deaths 1980 ICD9 as it appears in World Health Organization (WHO). WHO Mortality Database Version September 2016. Geneva, Switzerland: World Health Organization (WHO), 2016

Jamaica Vital Registration - Deaths 1981 ICD9 as it appears in World Health Organization (WHO). WHO Mortality Database Version September 2016. Geneva, Switzerland: World Health Organization (WHO), 2016

Jamaica Vital Registration - Deaths 1982 ICD9 as it appears in World Health Organization (WHO). WHO Mortality Database Version September 2016. Geneva, Switzerland: World Health Organization (WHO), 2016

Jamaica Vital Registration - Deaths 1983 ICD9 as it appears in World Health Organization (WHO). WHO Mortality Database Version September 2016. Geneva, Switzerland: World Health Organization (WHO), 2016

Jamaica Vital Registration - Deaths 1984 ICD9 as it appears in World Health Organization (WHO). WHO Mortality Database Version September 2016. Geneva, Switzerland: World Health Organization (WHO), 2016

Jamaica Vital Registration - Deaths 1985 ICD9 as it appears in World Health Organization (WHO). WHO Mortality Database Version September 2016. Geneva, Switzerland: World Health Organization (WHO), 2016

Jamaica Vital Registration - Deaths 1986 ICD9 as it appears in World Health Organization (WHO). WHO Mortality Database Version September 2016. Geneva, Switzerland: World Health Organization (WHO), 2016

Jamaica Vital Registration - Deaths 1987 ICD9 as it appears in World Health Organization (WHO). WHO Mortality Database Version September 2016. Geneva, Switzerland: World Health Organization (WHO), 2016

Jamaica Vital Registration - Deaths 1988 ICD9 as it appears in World Health Organization (WHO). WHO Mortality Database Version September 2016. Geneva, Switzerland: World Health Organization (WHO), 2016

Jamaica Vital Registration - Deaths 1989 ICD9 as it appears in World Health Organization (WHO). WHO Mortality Database Version September 2016. Geneva, Switzerland: World Health Organization (WHO), 2016

Jamaica Vital Registration - Deaths 1990 ICD9 as it appears in World Health Organization (WHO). WHO Mortality Database Version September 2016. Geneva, Switzerland: World Health Organization (WHO), 2016

Jamaica Vital Registration - Deaths 1991 ICD9 as it appears in World Health Organization (WHO). WHO Mortality Database Version September 2016. Geneva, Switzerland: World Health Organization (WHO), 2016

Jamaica Vital Registration - Deaths 2000 ICD10 as it appears in World Health Organization (WHO). WHO Mortality Database Version October 2017. Geneva, Switzerland: World Health Organization (WHO), 2017

Jamaica Vital Registration - Deaths 2001 ICD10 as it appears in World Health Organization (WHO). WHO Mortality Database Version October 2017. Geneva, Switzerland: World Health Organization (WHO), 2017

Jamaica Vital Registration - Deaths 2002 ICD10 as it appears in World Health Organization (WHO). WHO Mortality Database Version October 2017. Geneva, Switzerland: World Health Organization (WHO), 2017

Jamaica Vital Registration - Deaths 2003 ICD10 as it appears in World Health Organization (WHO). WHO Mortality Database Version October 2017. Geneva, Switzerland: World Health Organization (WHO), 2017

Jamaica Vital Registration - Deaths 2004 ICD10 as it appears in World Health Organization (WHO). WHO Mortality Database Version October 2017. Geneva, Switzerland: World Health Organization (WHO), 2017

Jamaica Vital Registration - Deaths 2005 ICD10 as it appears in World Health Organization (WHO). WHO Mortality Database Version October 2017. Geneva, Switzerland: World Health Organization (WHO), 2017

Jamaica Vital Registration - Deaths 2006 ICD10 as it appears in World Health Organization (WHO). WHO Mortality Database Version October 2017. Geneva, Switzerland: World Health Organization (WHO), 2017

Jamaica Vital Registration - Deaths 2009 ICD10 as it appears in World Health Organization (WHO). WHO Mortality Database Version October 2017. Geneva, Switzerland: World Health Organization (WHO), 2017

Jamaica Vital Registration - Deaths 2010 ICD10 as it appears in World Health Organization (WHO). WHO Mortality Database Version October 2017. Geneva, Switzerland: World Health Organization (WHO), 2017

Jamaica Vital Registration - Deaths 2011 ICD10 as it appears in World Health Organization (WHO). WHO Mortality Database Version October 2017. Geneva, Switzerland: World Health Organization (WHO), 2017

Jasseh M, Webb EL, Jaffar S, Howie S, Townend J, Smith PG, Greenwood BM, Corrah T. Reaching millennium development goal 4 - the Gambia. Trop Med Int Health. 2011; 16(10): 1314-25

Jehan I, Harris H, Salat S, Zeb A, Mobeen N, Pasha O, McClure EM, Moore J, Wright LL, Goldenberg RL. Neonatal mortality, risk factors and causes: a prospective population-based cohort study in urban Pakistan. Bull World Health Organ. 2009; 87(2): 130-8

Jordan Vital Registration - Deaths 2008 ICD10 as it appears in World Health Organization (WHO). WHO Mortality Database Version October 2017. Geneva, Switzerland: World Health Organization (WHO), 2017

Jordan Vital Registration - Deaths 2009 ICD10 as it appears in World Health Organization (WHO). WHO Mortality Database Version October 2017. Geneva, Switzerland: World Health Organization (WHO), 2017

Jordan Vital Registration - Deaths 2010 ICD10 as it appears in World Health Organization (WHO). WHO Mortality Database Version October 2017. Geneva, Switzerland: World Health Organization (WHO), 2017

Jordan Vital Registration - Deaths 2011 ICD10 as it appears in World Health Organization (WHO). WHO Mortality Database Version October 2017. Geneva, Switzerland: World Health Organization (WHO), 2017

Jordan Vital Registration - Deaths 2012 ICD10 as it appears in World Health Organization (WHO). WHO Mortality Database Version March 2017. Geneva, Switzerland: World Health Organization (WHO), 2017

Joshi R, Cardona M, Iyengar S, Sukumar A, Raju CR, Raju KR, Raju K, Reddy KS, Lopez A, Neal B. Chronic diseases now a leading cause of death in rural India – mortality data from the Andhra Pradesh Rural Health Initiative. Int J Epidemiol. 2006; 35(6): 1522-9

Kakazo M, Lehmann D, Coakley K, Gratten H, Saleu G, Taime J, Riley ID, Alphers MP. Mortality rates and the utilization of health services during terminal illness in the Asaro Valley, Eastern Highlands Province, Papua New Guinea. P N G Med J. 1999; 42(2-Jan): 13-26

Kalter HD, Roubanatou AM, Koffi A, Black RE. Direct estimates of national neonatal and child cause-specific mortality proportions in Niger by expert algorithm and physician-coded analysis of verbal autopsy interviews. J Glob Health. 2015; 5(1): 010415

Kapoor RK, Srivastava AK, Misra PK, Sharma B, Thakur S, Srivastava KI, Singh GK. Perinatal mortality in urban slums in Lucknow. Indian Pediatr. 1996; 33(1): 19-23

Kazakhstan Vital Registration - Deaths 1981 ICD9 as it appears in World Health Organization (WHO). WHO Mortality Database Version September 2016. Geneva, Switzerland: World Health Organization (WHO), 2016

Kazakhstan Vital Registration - Deaths 1982 ICD9 as it appears in World Health Organization (WHO). WHO Mortality Database Version September 2016. Geneva, Switzerland: World Health Organization (WHO), 2016
Kazakhstan Vital Registration - Deaths 1985 ICD9 as it appears in World Health Organization (WHO). WHO Mortality Database Version September 2016. Geneva, Switzerland: World Health Organization (WHO), 2016
Kazakhstan Vital Registration - Deaths 1986 ICD9 as it appears in World Health Organization (WHO). WHO Mortality Database Version September 2016. Geneva, Switzerland: World Health Organization (WHO), 2016
Kazakhstan Vital Registration - Deaths 1987 ICD9 as it appears in World Health Organization (WHO). WHO Mortality Database Version September 2016. Geneva, Switzerland: World Health Organization (WHO), 2016
Kazakhstan Vital Registration - Deaths 1988 ICD9 as it appears in World Health Organization (WHO). WHO Mortality Database Version September 2016. Geneva, Switzerland: World Health Organization (WHO), 2016
Kazakhstan Vital Registration - Deaths 1989 ICD9 as it appears in World Health Organization (WHO). WHO Mortality Database Version September 2016. Geneva, Switzerland: World Health Organization (WHO), 2016
Kazakhstan Vital Registration - Deaths 1990 ICD9 as it appears in World Health Organization (WHO). WHO Mortality Database Version September 2016. Geneva, Switzerland: World Health Organization (WHO), 2016
Kazakhstan Vital Registration - Deaths 1991 ICD9 as it appears in World Health Organization (WHO). WHO Mortality Database Version September 2016. Geneva, Switzerland: World Health Organization (WHO), 2016
Kazakhstan Vital Registration - Deaths 1992 ICD9 as it appears in World Health Organization (WHO). WHO Mortality Database Version September 2016. Geneva, Switzerland: World Health Organization (WHO), 2016
Kazakhstan Vital Registration - Deaths 1993 ICD9 as it appears in World Health Organization (WHO). WHO Mortality Database Version September 2016. Geneva, Switzerland: World Health Organization (WHO), 2016
Kazakhstan Vital Registration - Deaths 1994 ICD9 as it appears in World Health Organization (WHO). WHO Mortality Database Version September 2016. Geneva, Switzerland: World Health Organization (WHO), 2016
Kazakhstan Vital Registration - Deaths 1995 ICD9 as it appears in World Health Organization (WHO). WHO Mortality Database Version September 2016. Geneva, Switzerland: World Health Organization (WHO), 2016
Kazakhstan Vital Registration - Deaths 1996 ICD9 as it appears in World Health Organization (WHO). WHO Mortality Database Version September 2016. Geneva, Switzerland: World Health Organization (WHO), 2016
Kazakhstan Vital Registration - Deaths 1997 ICD9 as it appears in World Health Organization (WHO). WHO Mortality Database Version September 2016. Geneva, Switzerland: World Health Organization (WHO), 2016
Kazakhstan Vital Registration - Deaths 1998 ICD9 as it appears in World Health Organization (WHO). WHO Mortality Database Version September 2016. Geneva, Switzerland: World Health Organization (WHO), 2016
Kazakhstan Vital Registration - Deaths 1999 ICD9 as it appears in World Health Organization (WHO). WHO Mortality Database Version September 2016. Geneva, Switzerland: World Health Organization (WHO), 2016
Kazakhstan Vital Registration - Deaths 2000 ICD9 as it appears in World Health Organization (WHO). WHO Mortality Database Version September 2016. Geneva, Switzerland: World Health Organization (WHO), 2016
Kazakhstan Vital Registration - Deaths 2001 ICD9 as it appears in World Health Organization (WHO). WHO Mortality Database Version September 2016. Geneva, Switzerland: World Health Organization (WHO), 2016

Kazakhstan Vital Registration - Deaths 2002 ICD9 as it appears in World Health Organization (WHO). WHO Mortality Database Version September 2016. Geneva, Switzerland: World Health Organization (WHO), 2016

Kazakhstan Vital Registration - Deaths 2003 ICD9 as it appears in World Health Organization (WHO). WHO Mortality Database Version September 2016. Geneva, Switzerland: World Health Organization (WHO), 2016

Kazakhstan Vital Registration - Deaths 2004 ICD10 as it appears in World Health Organization (WHO). WHO Mortality Database Version November 2015. Geneva, Switzerland: World Health Organization (WHO), 2015

Kazakhstan Vital Registration - Deaths 2005 ICD10 as it appears in World Health Organization (WHO). WHO Mortality Database Version November 2015. Geneva, Switzerland: World Health Organization (WHO), 2015

Kazakhstan Vital Registration - Deaths 2006 ICD10 as it appears in World Health Organization (WHO). WHO Mortality Database Version November 2015. Geneva, Switzerland: World Health Organization (WHO), 2015

Kazakhstan Vital Registration - Deaths 2007 ICD10 as it appears in World Health Organization (WHO). WHO Mortality Database Version April 2018. Geneva, Switzerland: World Health Organization (WHO), 2018

Kazakhstan Vital Registration - Deaths 2008 ICD10 as it appears in World Health Organization (WHO). WHO Mortality Database Version November 2015. Geneva, Switzerland: World Health Organization (WHO), 2015

Kazakhstan Vital Registration - Deaths 2009 ICD10 as it appears in World Health Organization (WHO). WHO Mortality Database Version November 2015. Geneva, Switzerland: World Health Organization (WHO), 2015

Kazakhstan Vital Registration - Deaths 2010 ICD10 as it appears in World Health Organization (WHO). WHO Mortality Database Version November 2015. Geneva, Switzerland: World Health Organization (WHO), 2015

Kazakhstan Vital Registration - Deaths 2011 ICD10 as it appears in World Health Organization (WHO). WHO Mortality Database Version November 2015. Geneva, Switzerland: World Health Organization (WHO), 2015

Kazakhstan Vital Registration - Deaths 2012 ICD10 as it appears in World Health Organization (WHO). WHO Mortality Database Version November 2015. Geneva, Switzerland: World Health Organization (WHO), 2015

Kazakhstan Vital Registration - Deaths 2013 ICD10 as it appears in World Health Organization (WHO). WHO Mortality Database Version October 2017. Geneva, Switzerland: World Health Organization (WHO), 2017

Kazakhstan Vital Registration - Deaths 2014 ICD10 as it appears in World Health Organization (WHO). WHO Mortality Database Version October 2017. Geneva, Switzerland: World Health Organization (WHO), 2017

Kazakhstan Vital Registration - Deaths 2015 ICD10 as it appears in World Health Organization (WHO). WHO Mortality Database Version October 2017. Geneva, Switzerland: World Health Organization (WHO), 2017

Khanal S, Gc VS, Dawson P, Houston R. Verbal autopsy to ascertain causes of neonatal deaths in a community setting: a study from Morang, Nepal. JNMA J Nepal Med Assoc. 2011; 51(181): 21-7

Khanna R, Kumar A, Vaghela JF, Sreenivas V, Puliyel JM. Community based retrospective study of sex in infant mortality in India. BMJ. 2003; 327(7407): 126

Khoury SA, Massad D, Fardous T. Mortality and causes of death in Jordan 1995-96: assessment by verbal autopsy. Bull World Health Organ. 1999; 77(8): 641-50

Kumar R, Kapoor SK, Krishnan A. Performance of cause-specific childhood mortality surveillance by health workers using a short verbal autopsy tool. Southeast Asia J Public Health. 2012; 1(2): 151-158

Kumar V, Datta N, Wadhwa SS, Singhi S. Morbidity and mortality in diarrhea in rural Haryana Indian. Indian J Pediatr. 1985; 52(418): 455-61
Kumar V, Garg BS. Global health and infant mortality: application of verbal autopsy tool to categorize infant deaths, ascertain their causes and identify the gaps in health management information system in India. Int J Curr Res Rev. 2014; 5(15): 87-94
Kuwait Vital Registration - Deaths 1995 ICD10 as it appears in World Health Organization (WHO). WHO Mortality Database Version October 2017. Geneva, Switzerland: World Health Organization (WHO), 2017
Kuwait Vital Registration - Deaths 1996 ICD10 as it appears in World Health Organization (WHO). WHO Mortality Database Version October 2017. Geneva, Switzerland: World Health Organization (WHO), 2017
Kuwait Vital Registration - Deaths 1997 ICD10 as it appears in World Health Organization (WHO). WHO Mortality Database Version October 2017. Geneva, Switzerland: World Health Organization (WHO), 2017
Kuwait Vital Registration - Deaths 1999 ICD10 as it appears in World Health Organization (WHO). WHO Mortality Database Version October 2017. Geneva, Switzerland: World Health Organization (WHO), 2017
Kuwait Vital Registration - Deaths 2000 ICD10 as it appears in World Health Organization (WHO). WHO Mortality Database Version October 2017. Geneva, Switzerland: World Health Organization (WHO), 2017
Kuwait Vital Registration - Deaths 2001 ICD10 as it appears in World Health Organization (WHO). WHO Mortality Database Version October 2017. Geneva, Switzerland: World Health Organization (WHO), 2017
Kuwait Vital Registration - Deaths 2002 ICD10 as it appears in World Health Organization (WHO). WHO Mortality Database Version October 2017. Geneva, Switzerland: World Health Organization (WHO), 2017
Kuwait Vital Registration - Deaths 2008 ICD10 as it appears in World Health Organization (WHO). WHO Mortality Database Version October 2017. Geneva, Switzerland: World Health Organization (WHO), 2017
Kuwait Vital Registration - Deaths 1998 ICD10 as it appears in World Health Organization (WHO). WHO Mortality Database Version October 2017. Geneva, Switzerland: World Health Organization (WHO), 2017
Kuwait Vital Registration - Deaths 2003 ICD10 as it appears in World Health Organization (WHO). WHO Mortality Database Version October 2017. Geneva, Switzerland: World Health Organization (WHO), 2017
Kuwait Vital Registration - Deaths 2004 ICD10 as it appears in World Health Organization (WHO). WHO Mortality Database Version October 2017. Geneva, Switzerland: World Health Organization (WHO), 2017
Kuwait Vital Registration - Deaths 2005 ICD10 as it appears in World Health Organization (WHO). WHO Mortality Database Version October 2017. Geneva, Switzerland: World Health Organization (WHO), 2017
Kuwait Vital Registration - Deaths 2006 ICD10 as it appears in World Health Organization (WHO). WHO Mortality Database Version October 2017. Geneva, Switzerland: World Health Organization (WHO), 2017
Kuwait Vital Registration - Deaths 2007 ICD10 as it appears in World Health Organization (WHO). WHO Mortality Database Version October 2017. Geneva, Switzerland: World Health Organization (WHO), 2017
Kuwait Vital Registration - Deaths 2009 ICD10 as it appears in World Health Organization (WHO). WHO Mortality Database Version October 2017. Geneva, Switzerland: World Health Organization (WHO), 2017
Kuwait Vital Registration - Deaths 2010 ICD10 as it appears in World Health Organization (WHO). WHO Mortality Database Version October 2017. Geneva, Switzerland: World Health Organization (WHO), 2017

Kuwait Vital Registration - Deaths 2011 ICD10 as it appears in World Health Organization (WHO). WHO Mortality Database Version October 2017. Geneva, Switzerland: World Health Organization (WHO), 2017
Kuwait Vital Registration - Deaths 2012 ICD10 as it appears in World Health Organization (WHO). WHO Mortality Database Version October 2017. Geneva, Switzerland: World Health Organization (WHO), 2017
Kuwait Vital Registration - Deaths 2013 ICD10 as it appears in World Health Organization (WHO). WHO Mortality Database Version October 2017. Geneva, Switzerland: World Health Organization (WHO), 2017
Kuwait Vital Registration - Deaths 2014 ICD10 as it appears in World Health Organization (WHO). WHO Mortality Database Version October 2017. Geneva, Switzerland: World Health Organization (WHO), 2017
Kyrgyzstan Vital Registration - Deaths 1981 ICD9 as it appears in World Health Organization (WHO). WHO Mortality Database Version September 2016. Geneva, Switzerland: World Health Organization (WHO), 2016
Kyrgyzstan Vital Registration - Deaths 1982 ICD9 as it appears in World Health Organization (WHO). WHO Mortality Database Version September 2016. Geneva, Switzerland: World Health Organization (WHO), 2016
Kyrgyzstan Vital Registration - Deaths 1985 ICD9 as it appears in World Health Organization (WHO). WHO Mortality Database Version September 2016. Geneva, Switzerland: World Health Organization (WHO), 2016
Kyrgyzstan Vital Registration - Deaths 1986 ICD9 as it appears in World Health Organization (WHO). WHO Mortality Database Version September 2016. Geneva, Switzerland: World Health Organization (WHO), 2016
Kyrgyzstan Vital Registration - Deaths 1987 ICD9 as it appears in World Health Organization (WHO). WHO Mortality Database Version September 2016. Geneva, Switzerland: World Health Organization (WHO), 2016
Kyrgyzstan Vital Registration - Deaths 1988 ICD9 as it appears in World Health Organization (WHO). WHO Mortality Database Version September 2016. Geneva, Switzerland: World Health Organization (WHO), 2016
Kyrgyzstan Vital Registration - Deaths 1989 ICD9 as it appears in World Health Organization (WHO). WHO Mortality Database Version September 2016. Geneva, Switzerland: World Health Organization (WHO), 2016
Kyrgyzstan Vital Registration - Deaths 1990 ICD9 as it appears in World Health Organization (WHO). WHO Mortality Database Version September 2016. Geneva, Switzerland: World Health Organization (WHO), 2016
Kyrgyzstan Vital Registration - Deaths 1991 ICD9 as it appears in World Health Organization (WHO). WHO Mortality Database Version September 2016. Geneva, Switzerland: World Health Organization (WHO), 2016
Kyrgyzstan Vital Registration - Deaths 1992 ICD9 as it appears in World Health Organization (WHO). WHO Mortality Database Version September 2016. Geneva, Switzerland: World Health Organization (WHO), 2016
Kyrgyzstan Vital Registration - Deaths 1993 ICD9 as it appears in World Health Organization (WHO). WHO Mortality Database Version September 2016. Geneva, Switzerland: World Health Organization (WHO), 2016
Kyrgyzstan Vital Registration - Deaths 1994 ICD9 as it appears in World Health Organization (WHO). WHO Mortality Database Version September 2016. Geneva, Switzerland: World Health Organization (WHO), 2016
Kyrgyzstan Vital Registration - Deaths 1995 ICD9 as it appears in World Health Organization (WHO). WHO Mortality Database Version September 2016. Geneva, Switzerland: World Health Organization (WHO), 2016
Kyrgyzstan Vital Registration - Deaths 1996 ICD9 as it appears in World Health Organization (WHO). WHO Mortality Database Version September 2016. Geneva, Switzerland: World Health Organization (WHO), 2016

Kyrgyzstan Vital Registration - Deaths 1997 ICD9 as it appears in World Health Organization (WHO). WHO Mortality Database Version September 2016. Geneva, Switzerland: World Health Organization (WHO), 2016
Kyrgyzstan Vital Registration - Deaths 1998 ICD9 as it appears in World Health Organization (WHO). WHO Mortality Database Version September 2016. Geneva, Switzerland: World Health Organization (WHO), 2016
Kyrgyzstan Vital Registration - Deaths 1999 ICD9 as it appears in World Health Organization (WHO). WHO Mortality Database Version September 2016. Geneva, Switzerland: World Health Organization (WHO), 2016
Kyrgyzstan Vital Registration - Deaths 2000 ICD10 as it appears in World Health Organization (WHO). WHO Mortality Database Version October 2017. Geneva, Switzerland: World Health Organization (WHO), 2017
Kyrgyzstan Vital Registration - Deaths 2001 ICD10 as it appears in World Health Organization (WHO). WHO Mortality Database Version October 2017. Geneva, Switzerland: World Health Organization (WHO), 2017
Kyrgyzstan Vital Registration - Deaths 2002 ICD10 as it appears in World Health Organization (WHO). WHO Mortality Database Version October 2017. Geneva, Switzerland: World Health Organization (WHO), 2017
Kyrgyzstan Vital Registration - Deaths 2003 ICD10 as it appears in World Health Organization (WHO). WHO Mortality Database Version October 2017. Geneva, Switzerland: World Health Organization (WHO), 2017
Kyrgyzstan Vital Registration - Deaths 2004 ICD10 as it appears in World Health Organization (WHO). WHO Mortality Database Version October 2017. Geneva, Switzerland: World Health Organization (WHO), 2017
Kyrgyzstan Vital Registration - Deaths 2005 ICD10 as it appears in World Health Organization (WHO). WHO Mortality Database Version October 2017. Geneva, Switzerland: World Health Organization (WHO), 2017
Kyrgyzstan Vital Registration - Deaths 2006 ICD10 as it appears in World Health Organization (WHO). WHO Mortality Database Version October 2017. Geneva, Switzerland: World Health Organization (WHO), 2017
Kyrgyzstan Vital Registration - Deaths 2007 ICD10 as it appears in World Health Organization (WHO). WHO Mortality Database Version October 2017. Geneva, Switzerland: World Health Organization (WHO), 2017
Kyrgyzstan Vital Registration - Deaths 2008 ICD10 as it appears in World Health Organization (WHO). WHO Mortality Database Version October 2017. Geneva, Switzerland: World Health Organization (WHO), 2017
Kyrgyzstan Vital Registration - Deaths 2009 ICD10 as it appears in World Health Organization (WHO). WHO Mortality Database Version October 2017. Geneva, Switzerland: World Health Organization (WHO), 2017
Kyrgyzstan Vital Registration - Deaths 2010 ICD10 as it appears in World Health Organization (WHO). WHO Mortality Database Version October 2017. Geneva, Switzerland: World Health Organization (WHO), 2017
Kyrgyzstan Vital Registration - Deaths 2011 ICD10 as it appears in World Health Organization (WHO). WHO Mortality Database Version October 2017. Geneva, Switzerland: World Health Organization (WHO), 2017
Kyrgyzstan Vital Registration - Deaths 2012 ICD10 as it appears in World Health Organization (WHO). WHO Mortality Database Version October 2017. Geneva, Switzerland: World Health Organization (WHO), 2017
Kyrgyzstan Vital Registration - Deaths 2013 ICD10 as it appears in World Health Organization (WHO). WHO Mortality Database Version October 2017. Geneva, Switzerland: World Health Organization (WHO), 2017
Kyrgyzstan Vital Registration - Deaths 2014 ICD10 as it appears in World Health Organization (WHO). WHO Mortality Database Version March 2017. Geneva, Switzerland: World Health Organization (WHO), 2017

Kyrgyzstan Vital Registration - Deaths 2015 ICD10 as it appears in World Health Organization (WHO). WHO Mortality Database Version March 2017. Geneva, Switzerland: World Health Organization (WHO), 2017
Latvia Vital Registration - Deaths 1980 ICD9 as it appears in World Health Organization (WHO). WHO Mortality Database Version September 2016. Geneva, Switzerland: World Health Organization (WHO), 2016
Latvia Vital Registration - Deaths 1981 ICD9 as it appears in World Health Organization (WHO). WHO Mortality Database Version September 2016. Geneva, Switzerland: World Health Organization (WHO), 2016
Latvia Vital Registration - Deaths 1982 ICD9 as it appears in World Health Organization (WHO). WHO Mortality Database Version September 2016. Geneva, Switzerland: World Health Organization (WHO), 2016
Latvia Vital Registration - Deaths 1983 ICD9 as it appears in World Health Organization (WHO). WHO Mortality Database Version September 2016. Geneva, Switzerland: World Health Organization (WHO), 2016
Latvia Vital Registration - Deaths 1984 ICD9 as it appears in World Health Organization (WHO). WHO Mortality Database Version September 2016. Geneva, Switzerland: World Health Organization (WHO), 2016
Latvia Vital Registration - Deaths 1985 ICD9 as it appears in World Health Organization (WHO). WHO Mortality Database Version September 2016. Geneva, Switzerland: World Health Organization (WHO), 2016
Latvia Vital Registration - Deaths 1986 ICD9 as it appears in World Health Organization (WHO). WHO Mortality Database Version September 2016. Geneva, Switzerland: World Health Organization (WHO), 2016
Latvia Vital Registration - Deaths 1987 ICD9 as it appears in World Health Organization (WHO). WHO Mortality Database Version September 2016. Geneva, Switzerland: World Health Organization (WHO), 2016
Latvia Vital Registration - Deaths 1988 ICD9 as it appears in World Health Organization (WHO). WHO Mortality Database Version September 2016. Geneva, Switzerland: World Health Organization (WHO), 2016
Latvia Vital Registration - Deaths 1989 ICD9 as it appears in World Health Organization (WHO). WHO Mortality Database Version September 2016. Geneva, Switzerland: World Health Organization (WHO), 2016
Latvia Vital Registration - Deaths 1990 ICD9 as it appears in World Health Organization (WHO). WHO Mortality Database Version September 2016. Geneva, Switzerland: World Health Organization (WHO), 2016
Latvia Vital Registration - Deaths 1991 ICD9 as it appears in World Health Organization (WHO). WHO Mortality Database Version September 2016. Geneva, Switzerland: World Health Organization (WHO), 2016
Latvia Vital Registration - Deaths 1992 ICD9 as it appears in World Health Organization (WHO). WHO Mortality Database Version September 2016. Geneva, Switzerland: World Health Organization (WHO), 2016
Latvia Vital Registration - Deaths 1993 ICD9 as it appears in World Health Organization (WHO). WHO Mortality Database Version September 2016. Geneva, Switzerland: World Health Organization (WHO), 2016
Latvia Vital Registration - Deaths 1994 ICD9 as it appears in World Health Organization (WHO). WHO Mortality Database Version September 2016. Geneva, Switzerland: World Health Organization (WHO), 2016
Latvia Vital Registration - Deaths 1995 ICD9 as it appears in World Health Organization (WHO). WHO Mortality Database Version September 2016. Geneva, Switzerland: World Health Organization (WHO), 2016
Latvia Vital Registration - Deaths 1996 ICD10 as it appears in World Health Organization (WHO). WHO Mortality Database Version October 2017. Geneva, Switzerland: World Health Organization (WHO), 2017

Latvia Vital Registration - Deaths 1997 ICD10 as it appears in World Health Organization (WHO). WHO Mortality Database Version October 2017. Geneva, Switzerland: World Health Organization (WHO), 2017
Latvia Vital Registration - Deaths 1998 ICD10 as it appears in World Health Organization (WHO). WHO Mortality Database Version October 2017. Geneva, Switzerland: World Health Organization (WHO), 2017
Latvia Vital Registration - Deaths 1999 ICD10 as it appears in World Health Organization (WHO). WHO Mortality Database Version October 2017. Geneva, Switzerland: World Health Organization (WHO), 2017
Latvia Vital Registration - Deaths 2000 ICD10 as it appears in World Health Organization (WHO). WHO Mortality Database Version October 2017. Geneva, Switzerland: World Health Organization (WHO), 2017
Latvia Vital Registration - Deaths 2001 ICD10 as it appears in World Health Organization (WHO). WHO Mortality Database Version October 2017. Geneva, Switzerland: World Health Organization (WHO), 2017
Latvia Vital Registration - Deaths 2002 ICD10 as it appears in World Health Organization (WHO). WHO Mortality Database Version October 2017. Geneva, Switzerland: World Health Organization (WHO), 2017
Latvia Vital Registration - Deaths 2003 ICD10 as it appears in World Health Organization (WHO). WHO Mortality Database Version October 2017. Geneva, Switzerland: World Health Organization (WHO), 2017
Latvia Vital Registration - Deaths 2004 ICD10 as it appears in World Health Organization (WHO). WHO Mortality Database Version October 2017. Geneva, Switzerland: World Health Organization (WHO), 2017
Latvia Vital Registration - Deaths 2005 ICD10 as it appears in World Health Organization (WHO). WHO Mortality Database Version October 2017. Geneva, Switzerland: World Health Organization (WHO), 2017
Latvia Vital Registration - Deaths 2006 ICD10 as it appears in World Health Organization (WHO). WHO Mortality Database Version October 2017. Geneva, Switzerland: World Health Organization (WHO), 2017
Latvia Vital Registration - Deaths 2007 ICD10 as it appears in World Health Organization (WHO). WHO Mortality Database Version October 2017. Geneva, Switzerland: World Health Organization (WHO), 2017
Latvia Vital Registration - Deaths 2008 ICD10 as it appears in World Health Organization (WHO). WHO Mortality Database Version October 2017. Geneva, Switzerland: World Health Organization (WHO), 2017
Latvia Vital Registration - Deaths 2009 ICD10 as it appears in World Health Organization (WHO). WHO Mortality Database Version October 2017. Geneva, Switzerland: World Health Organization (WHO), 2017
Latvia Vital Registration - Deaths 2010 ICD10 as it appears in World Health Organization (WHO). WHO Mortality Database Version October 2017. Geneva, Switzerland: World Health Organization (WHO), 2017
Latvia Vital Registration - Deaths 2011 ICD10 as it appears in World Health Organization (WHO). WHO Mortality Database Version October 2017. Geneva, Switzerland: World Health Organization (WHO), 2017
Latvia Vital Registration - Deaths 2012 ICD10 as it appears in World Health Organization (WHO). WHO Mortality Database Version October 2017. Geneva, Switzerland: World Health Organization (WHO), 2017
Latvia Vital Registration - Deaths 2013 ICD10 as it appears in World Health Organization (WHO). WHO Mortality Database Version October 2017. Geneva, Switzerland: World Health Organization (WHO), 2017
Latvia Vital Registration - Deaths 2014 ICD10 as it appears in World Health Organization (WHO). WHO Mortality Database Version October 2017. Geneva, Switzerland: World Health Organization (WHO), 2017

Latvia Vital Registration - Deaths 2015 ICD10 as it appears in World Health Organization (WHO). WHO Mortality Database Version October 2017. Geneva, Switzerland: World Health Organization (WHO), 2017
Lithuania Vital Registration - Deaths 1981 ICD9 as it appears in World Health Organization (WHO). WHO Mortality Database Version September 2016. Geneva, Switzerland: World Health Organization (WHO), 2016
Lithuania Vital Registration - Deaths 1982 ICD9 as it appears in World Health Organization (WHO). WHO Mortality Database Version September 2016. Geneva, Switzerland: World Health Organization (WHO), 2016
Lithuania Vital Registration - Deaths 1985 ICD9 as it appears in World Health Organization (WHO). WHO Mortality Database Version September 2016. Geneva, Switzerland: World Health Organization (WHO), 2016
Lithuania Vital Registration - Deaths 1986 ICD9 as it appears in World Health Organization (WHO). WHO Mortality Database Version September 2016. Geneva, Switzerland: World Health Organization (WHO), 2016
Lithuania Vital Registration - Deaths 1987 ICD9 as it appears in World Health Organization (WHO). WHO Mortality Database Version September 2016. Geneva, Switzerland: World Health Organization (WHO), 2016
Lithuania Vital Registration - Deaths 1988 ICD9 as it appears in World Health Organization (WHO). WHO Mortality Database Version September 2016. Geneva, Switzerland: World Health Organization (WHO), 2016
Lithuania Vital Registration - Deaths 1989 ICD9 as it appears in World Health Organization (WHO). WHO Mortality Database Version September 2016. Geneva, Switzerland: World Health Organization (WHO), 2016
Lithuania Vital Registration - Deaths 1990 ICD9 as it appears in World Health Organization (WHO). WHO Mortality Database Version September 2016. Geneva, Switzerland: World Health Organization (WHO), 2016
Lithuania Vital Registration - Deaths 1991 ICD9 as it appears in World Health Organization (WHO). WHO Mortality Database Version September 2016. Geneva, Switzerland: World Health Organization (WHO), 2016
Lithuania Vital Registration - Deaths 1992 ICD9 as it appears in World Health Organization (WHO). WHO Mortality Database Version September 2016. Geneva, Switzerland: World Health Organization (WHO), 2016
Lithuania Vital Registration - Deaths 1993 ICD9 as it appears in World Health Organization (WHO). WHO Mortality Database Version September 2016. Geneva, Switzerland: World Health Organization (WHO), 2016
Lithuania Vital Registration - Deaths 1994 ICD9 as it appears in World Health Organization (WHO). WHO Mortality Database Version November 2015. Geneva, Switzerland: World Health Organization (WHO), 2015
Lithuania Vital Registration - Deaths 1995 ICD9 as it appears in World Health Organization (WHO). WHO Mortality Database Version November 2015. Geneva, Switzerland: World Health Organization (WHO), 2015
Lithuania Vital Registration - Deaths 1996 ICD9 as it appears in World Health Organization (WHO). WHO Mortality Database Version November 2015. Geneva, Switzerland: World Health Organization (WHO), 2015
Lithuania Vital Registration - Deaths 1998 ICD10 as it appears in World Health Organization (WHO). WHO Mortality Database Version October 2017. Geneva, Switzerland: World Health Organization (WHO), 2017
Lithuania Vital Registration - Deaths 1999 ICD10 as it appears in World Health Organization (WHO). WHO Mortality Database Version October 2017. Geneva, Switzerland: World Health Organization (WHO), 2017
Lithuania Vital Registration - Deaths 2000 ICD10 as it appears in World Health Organization (WHO). WHO Mortality Database Version October 2017. Geneva, Switzerland: World Health Organization (WHO), 2017

Lithuania Vital Registration - Deaths 2001 ICD10 as it appears in World Health Organization (WHO). WHO Mortality Database Version October 2017. Geneva, Switzerland: World Health Organization (WHO), 2017
Lithuania Vital Registration - Deaths 2002 ICD10 as it appears in World Health Organization (WHO). WHO Mortality Database Version October 2017. Geneva, Switzerland: World Health Organization (WHO), 2017
Lithuania Vital Registration - Deaths 2003 ICD10 as it appears in World Health Organization (WHO). WHO Mortality Database Version October 2017. Geneva, Switzerland: World Health Organization (WHO), 2017
Lithuania Vital Registration - Deaths 2004 ICD10 as it appears in World Health Organization (WHO). WHO Mortality Database Version October 2017. Geneva, Switzerland: World Health Organization (WHO), 2017
Lithuania Vital Registration - Deaths 2005 ICD10 as it appears in World Health Organization (WHO). WHO Mortality Database Version October 2017. Geneva, Switzerland: World Health Organization (WHO), 2017
Lithuania Vital Registration - Deaths 2006 ICD10 as it appears in World Health Organization (WHO). WHO Mortality Database Version October 2017. Geneva, Switzerland: World Health Organization (WHO), 2017
Lithuania Vital Registration - Deaths 2007 ICD10 as it appears in World Health Organization (WHO). WHO Mortality Database Version October 2017. Geneva, Switzerland: World Health Organization (WHO), 2017
Lithuania Vital Registration - Deaths 2008 ICD10 as it appears in World Health Organization (WHO). WHO Mortality Database Version October 2017. Geneva, Switzerland: World Health Organization (WHO), 2017
Lithuania Vital Registration - Deaths 1997 ICD9 as it appears in World Health Organization (WHO). WHO Mortality Database Version November 2015. Geneva, Switzerland: World Health Organization (WHO), 2015
Lithuania Vital Registration - Deaths 2009 ICD10 as it appears in World Health Organization (WHO). WHO Mortality Database Version October 2017. Geneva, Switzerland: World Health Organization (WHO), 2017
Lithuania Vital Registration - Deaths 2010 ICD10 as it appears in World Health Organization (WHO). WHO Mortality Database Version October 2017. Geneva, Switzerland: World Health Organization (WHO), 2017
Lithuania Vital Registration - Deaths 2011 ICD10 as it appears in World Health Organization (WHO). WHO Mortality Database Version October 2017. Geneva, Switzerland: World Health Organization (WHO), 2017
Lithuania Vital Registration - Deaths 2012 ICD10 as it appears in World Health Organization (WHO). WHO Mortality Database Version October 2017. Geneva, Switzerland: World Health Organization (WHO), 2017
Lithuania Vital Registration - Deaths 2013 ICD10 as it appears in World Health Organization (WHO). WHO Mortality Database Version October 2017. Geneva, Switzerland: World Health Organization (WHO), 2017
Lithuania Vital Registration - Deaths 2014 ICD10 as it appears in World Health Organization (WHO). WHO Mortality Database Version October 2017. Geneva, Switzerland: World Health Organization (WHO), 2017
Lithuania Vital Registration - Deaths 2015 ICD10 as it appears in World Health Organization (WHO). WHO Mortality Database Version March 2017. Geneva, Switzerland: World Health Organization (WHO), 2017
Lithuania Vital Registration - Deaths 2016 ICD10 as it appears in World Health Organization (WHO). WHO Mortality Database Version April 2018. Geneva, Switzerland: World Health Organization (WHO), 2018
Luxembourg Vital Registration - Deaths 1980 ICD9 as it appears in World Health Organization (WHO). WHO Mortality Database Version November 2015. Geneva, Switzerland: World Health Organization (WHO), 2015

Luxembourg Vital Registration - Deaths 1981 ICD9 as it appears in World Health Organization (WHO). WHO Mortality Database Version November 2015. Geneva, Switzerland: World Health Organization (WHO), 2015
Luxembourg Vital Registration - Deaths 1982 ICD9 as it appears in World Health Organization (WHO). WHO Mortality Database Version November 2015. Geneva, Switzerland: World Health Organization (WHO), 2015
Luxembourg Vital Registration - Deaths 1983 ICD9 as it appears in World Health Organization (WHO). WHO Mortality Database Version November 2015. Geneva, Switzerland: World Health Organization (WHO), 2015
Luxembourg Vital Registration - Deaths 1984 ICD9 as it appears in World Health Organization (WHO). WHO Mortality Database Version November 2015. Geneva, Switzerland: World Health Organization (WHO), 2015
Luxembourg Vital Registration - Deaths 1985 ICD9 as it appears in World Health Organization (WHO). WHO Mortality Database Version November 2015. Geneva, Switzerland: World Health Organization (WHO), 2015
Luxembourg Vital Registration - Deaths 1986 ICD9 as it appears in World Health Organization (WHO). WHO Mortality Database Version November 2015. Geneva, Switzerland: World Health Organization (WHO), 2015
Luxembourg Vital Registration - Deaths 1987 ICD9 as it appears in World Health Organization (WHO). WHO Mortality Database Version November 2015. Geneva, Switzerland: World Health Organization (WHO), 2015
Luxembourg Vital Registration - Deaths 1988 ICD9 as it appears in World Health Organization (WHO). WHO Mortality Database Version November 2015. Geneva, Switzerland: World Health Organization (WHO), 2015
Luxembourg Vital Registration - Deaths 1989 ICD9 as it appears in World Health Organization (WHO). WHO Mortality Database Version November 2015. Geneva, Switzerland: World Health Organization (WHO), 2015
Luxembourg Vital Registration - Deaths 1990 ICD9 as it appears in World Health Organization (WHO). WHO Mortality Database Version November 2015. Geneva, Switzerland: World Health Organization (WHO), 2015
Luxembourg Vital Registration - Deaths 1991 ICD9 as it appears in World Health Organization (WHO). WHO Mortality Database Version September 2016. Geneva, Switzerland: World Health Organization (WHO), 2016
Luxembourg Vital Registration - Deaths 1992 ICD9 as it appears in World Health Organization (WHO). WHO Mortality Database Version November 2015. Geneva, Switzerland: World Health Organization (WHO), 2015
Luxembourg Vital Registration - Deaths 1993 ICD9 as it appears in World Health Organization (WHO). WHO Mortality Database Version November 2015. Geneva, Switzerland: World Health Organization (WHO), 2015
Luxembourg Vital Registration - Deaths 1994 ICD9 as it appears in World Health Organization (WHO). WHO Mortality Database Version November 2015. Geneva, Switzerland: World Health Organization (WHO), 2015
Luxembourg Vital Registration - Deaths 1995 ICD9 as it appears in World Health Organization (WHO). WHO Mortality Database Version November 2015. Geneva, Switzerland: World Health Organization (WHO), 2015
Luxembourg Vital Registration - Deaths 1996 ICD9 as it appears in World Health Organization (WHO). WHO Mortality Database Version November 2015. Geneva, Switzerland: World Health Organization (WHO), 2015
Luxembourg Vital Registration - Deaths 1997 ICD9 as it appears in World Health Organization (WHO). WHO Mortality Database Version November 2015. Geneva, Switzerland: World Health Organization (WHO), 2015
Luxembourg Vital Registration - Deaths 1998 ICD10 as it appears in World Health Organization (WHO). WHO Mortality Database Version October 2017. Geneva, Switzerland: World Health Organization (WHO), 2017

Luxembourg Vital Registration - Deaths 1999 ICD10 as it appears in World Health Organization (WHO). WHO Mortality Database Version October 2017. Geneva, Switzerland: World Health Organization (WHO), 2017
Luxembourg Vital Registration - Deaths 2000 ICD10 as it appears in World Health Organization (WHO). WHO Mortality Database Version October 2017. Geneva, Switzerland: World Health Organization (WHO), 2017
Luxembourg Vital Registration - Deaths 2001 ICD10 as it appears in World Health Organization (WHO). WHO Mortality Database Version October 2017. Geneva, Switzerland: World Health Organization (WHO), 2017
Luxembourg Vital Registration - Deaths 2002 ICD10 as it appears in World Health Organization (WHO). WHO Mortality Database Version October 2017. Geneva, Switzerland: World Health Organization (WHO), 2017
Luxembourg Vital Registration - Deaths 2003 ICD10 as it appears in World Health Organization (WHO). WHO Mortality Database Version October 2017. Geneva, Switzerland: World Health Organization (WHO), 2017
Luxembourg Vital Registration - Deaths 2004 ICD10 as it appears in World Health Organization (WHO). WHO Mortality Database Version October 2017. Geneva, Switzerland: World Health Organization (WHO), 2017
Luxembourg Vital Registration - Deaths 2005 ICD10 as it appears in World Health Organization (WHO). WHO Mortality Database Version October 2017. Geneva, Switzerland: World Health Organization (WHO), 2017
Luxembourg Vital Registration - Deaths 2006 ICD10 as it appears in World Health Organization (WHO). WHO Mortality Database Version October 2017. Geneva, Switzerland: World Health Organization (WHO), 2017
Luxembourg Vital Registration - Deaths 2007 ICD10 as it appears in World Health Organization (WHO). WHO Mortality Database Version October 2017. Geneva, Switzerland: World Health Organization (WHO), 2017
Luxembourg Vital Registration - Deaths 2008 ICD10 as it appears in World Health Organization (WHO). WHO Mortality Database Version October 2017. Geneva, Switzerland: World Health Organization (WHO), 2017
Luxembourg Vital Registration - Deaths 2009 ICD10 as it appears in World Health Organization (WHO). WHO Mortality Database Version October 2017. Geneva, Switzerland: World Health Organization (WHO), 2017
Luxembourg Vital Registration - Deaths 2010 ICD10 as it appears in World Health Organization (WHO). WHO Mortality Database Version October 2017. Geneva, Switzerland: World Health Organization (WHO), 2017
Luxembourg Vital Registration - Deaths 2011 ICD10 as it appears in World Health Organization (WHO). WHO Mortality Database Version October 2017. Geneva, Switzerland: World Health Organization (WHO), 2017
Luxembourg Vital Registration - Deaths 2012 ICD10 as it appears in World Health Organization (WHO). WHO Mortality Database Version October 2017. Geneva, Switzerland: World Health Organization (WHO), 2017
Luxembourg Vital Registration - Deaths 2013 ICD10 as it appears in World Health Organization (WHO). WHO Mortality Database Version October 2017. Geneva, Switzerland: World Health Organization (WHO), 2017
Luxembourg Vital Registration - Deaths 2014 ICD10 as it appears in World Health Organization (WHO). WHO Mortality Database Version October 2017. Geneva, Switzerland: World Health Organization (WHO), 2017
Luxembourg Vital Registration - Deaths 2015 ICD10 as it appears in World Health Organization (WHO). WHO Mortality Database Version October 2017. Geneva, Switzerland: World Health Organization (WHO), 2017
Macau Vital Registration - Deaths 1994 ICD9 as it appears in World Health Organization (WHO). WHO Mortality Database Version November 2015. Geneva, Switzerland: World Health Organization (WHO), 2015

Macedonia Vital Registration - Deaths 1991 ICD9 as it appears in World Health Organization (WHO). WHO Mortality Database Version November 2015. Geneva, Switzerland: World Health Organization (WHO), 2015
Macedonia Vital Registration - Deaths 1992 ICD9 as it appears in World Health Organization (WHO). WHO Mortality Database Version November 2015. Geneva, Switzerland: World Health Organization (WHO), 2015
Macedonia Vital Registration - Deaths 1993 ICD9 as it appears in World Health Organization (WHO). WHO Mortality Database Version November 2015. Geneva, Switzerland: World Health Organization (WHO), 2015
Macedonia Vital Registration - Deaths 1994 ICD9 as it appears in World Health Organization (WHO). WHO Mortality Database Version November 2015. Geneva, Switzerland: World Health Organization (WHO), 2015
Macedonia Vital Registration - Deaths 1995 ICD9 as it appears in World Health Organization (WHO). WHO Mortality Database Version November 2015. Geneva, Switzerland: World Health Organization (WHO), 2015
Macedonia Vital Registration - Deaths 1996 ICD9 as it appears in World Health Organization (WHO). WHO Mortality Database Version November 2015. Geneva, Switzerland: World Health Organization (WHO), 2015
Macedonia Vital Registration - Deaths 1997 ICD9 as it appears in World Health Organization (WHO). WHO Mortality Database Version November 2015. Geneva, Switzerland: World Health Organization (WHO), 2015
Macedonia Vital Registration - Deaths 1998 ICD9 as it appears in World Health Organization (WHO). WHO Mortality Database Version November 2015. Geneva, Switzerland: World Health Organization (WHO), 2015
Macedonia Vital Registration - Deaths 1999 ICD9 as it appears in World Health Organization (WHO). WHO Mortality Database Version November 2015. Geneva, Switzerland: World Health Organization (WHO), 2015
Macedonia Vital Registration - Deaths 2000 ICD9 as it appears in World Health Organization (WHO). WHO Mortality Database Version November 2015. Geneva, Switzerland: World Health Organization (WHO), 2015
Macedonia Vital Registration - Deaths 2001 ICD9 as it appears in World Health Organization (WHO). WHO Mortality Database Version November 2015. Geneva, Switzerland: World Health Organization (WHO), 2015
Macedonia Vital Registration - Deaths 2002 ICD9 as it appears in World Health Organization (WHO). WHO Mortality Database Version November 2015. Geneva, Switzerland: World Health Organization (WHO), 2015
Macedonia Vital Registration - Deaths 2003 ICD9 as it appears in World Health Organization (WHO). WHO Mortality Database Version November 2015. Geneva, Switzerland: World Health Organization (WHO), 2015
Macedonia Vital Registration - Deaths 2004 ICD9 as it appears in World Health Organization (WHO). WHO Mortality Database Version September 2016. Geneva, Switzerland: World Health Organization (WHO), 2016
Macedonia Vital Registration - Deaths 2005 ICD9 as it appears in World Health Organization (WHO). WHO Mortality Database Version September 2016. Geneva, Switzerland: World Health Organization (WHO), 2016
Macedonia Vital Registration - Deaths 2006 ICD10 as it appears in World Health Organization (WHO). WHO Mortality Database Version October 2017. Geneva, Switzerland: World Health Organization (WHO), 2017
Macedonia Vital Registration - Deaths 2007 ICD10 as it appears in World Health Organization (WHO). WHO Mortality Database Version October 2017. Geneva, Switzerland: World Health Organization (WHO), 2017
Macedonia Vital Registration - Deaths 2008 ICD10 as it appears in World Health Organization (WHO). WHO Mortality Database Version October 2017. Geneva, Switzerland: World Health Organization (WHO), 2017

Macedonia Vital Registration - Deaths 2009 ICD10 as it appears in World Health Organization (WHO). WHO Mortality Database Version October 2017. Geneva, Switzerland: World Health Organization (WHO), 2017

Macedonia Vital Registration - Deaths 2010 ICD10 as it appears in World Health Organization (WHO). WHO Mortality Database Version October 2017. Geneva, Switzerland: World Health Organization (WHO), 2017

Macedonia Vital Registration - Deaths 2011 ICD10 as it appears in World Health Organization (WHO). WHO Mortality Database Version March 2017. Geneva, Switzerland: World Health Organization (WHO), 2017

Macedonia Vital Registration - Deaths 2012 ICD10 as it appears in World Health Organization (WHO). WHO Mortality Database Version March 2017. Geneva, Switzerland: World Health Organization (WHO), 2017

Macedonia Vital Registration - Deaths 2013 ICD10 as it appears in World Health Organization (WHO). WHO Mortality Database Version March 2017. Geneva, Switzerland: World Health Organization (WHO), 2017

Macro International, Inc, Ministry of Health and Population (Nepal), New ERA. Nepal Demographic and Health Survey 2006. Fairfax, United States: ICF International

Macro International, Inc, National Institute of Population Studies (Pakistan). Pakistan Demographic and Health Survey 2006-2007. Fairfax, United States: ICF International

Malaysia Vital Registration - Deaths 2000 ICD10 as it appears in World Health Organization (WHO). WHO Mortality Database Version October 2017. Geneva, Switzerland: World Health Organization (WHO), 2017

Malaysia Vital Registration - Deaths 2001 ICD10 as it appears in World Health Organization (WHO). WHO Mortality Database Version October 2017. Geneva, Switzerland: World Health Organization (WHO), 2017

Malaysia Vital Registration - Deaths 2002 ICD10 as it appears in World Health Organization (WHO). WHO Mortality Database Version October 2017. Geneva, Switzerland: World Health Organization (WHO), 2017

Malaysia Vital Registration - Deaths 2003 ICD10 as it appears in World Health Organization (WHO). WHO Mortality Database Version October 2017. Geneva, Switzerland: World Health Organization (WHO), 2017

Malaysia Vital Registration - Deaths 2004 ICD10 as it appears in World Health Organization (WHO). WHO Mortality Database Version October 2017. Geneva, Switzerland: World Health Organization (WHO), 2017

Malaysia Vital Registration - Deaths 2005 ICD10 as it appears in World Health Organization (WHO). WHO Mortality Database Version October 2017. Geneva, Switzerland: World Health Organization (WHO), 2017

Malaysia Vital Registration - Deaths 2006 ICD10 as it appears in World Health Organization (WHO). WHO Mortality Database Version October 2017. Geneva, Switzerland: World Health Organization (WHO), 2017

Malaysia Vital Registration - Deaths 2007 ICD10 as it appears in World Health Organization (WHO). WHO Mortality Database Version October 2017. Geneva, Switzerland: World Health Organization (WHO), 2017

Malaysia Vital Registration - Deaths 2008 ICD10 as it appears in World Health Organization (WHO). WHO Mortality Database Version October 2017. Geneva, Switzerland: World Health Organization (WHO), 2017

Malaysia Vital Registration - Deaths 2009 ICD10 as it appears in World Health Organization (WHO). WHO Mortality Database Version October 2017. Geneva, Switzerland: World Health Organization (WHO), 2017

Malaysia Vital Registration - Deaths 2010 ICD10 as it appears in World Health Organization (WHO). WHO Mortality Database Version October 2017. Geneva, Switzerland: World Health Organization (WHO), 2017

Malaysia Vital Registration - Deaths 2011 ICD10 as it appears in World Health Organization (WHO). WHO Mortality Database Version October 2017. Geneva, Switzerland: World Health Organization (WHO), 2017
Malaysia Vital Registration - Deaths 2012 ICD10 as it appears in World Health Organization (WHO). WHO Mortality Database Version October 2017. Geneva, Switzerland: World Health Organization (WHO), 2017
Malaysia Vital Registration - Deaths 2013 ICD10 as it appears in World Health Organization (WHO). WHO Mortality Database Version October 2017. Geneva, Switzerland: World Health Organization (WHO), 2017
Malaysia Vital Registration - Deaths 2014 ICD10 as it appears in World Health Organization (WHO). WHO Mortality Database Version October 2017. Geneva, Switzerland: World Health Organization (WHO), 2017
Maldives Vital Registration - Deaths 2010 ICD10 as it appears in World Health Organization (WHO). WHO Mortality Database Version October 2017. Geneva, Switzerland: World Health Organization (WHO), 2017
Maldives Vital Registration - Deaths 2011 ICD10 as it appears in World Health Organization (WHO). WHO Mortality Database Version October 2017. Geneva, Switzerland: World Health Organization (WHO), 2017
Malta Vital Registration - Deaths 1980 ICD9 as it appears in World Health Organization (WHO). WHO Mortality Database Version November 2015. Geneva, Switzerland: World Health Organization (WHO), 2015
Malta Vital Registration - Deaths 1981 ICD9 as it appears in World Health Organization (WHO). WHO Mortality Database Version November 2015. Geneva, Switzerland: World Health Organization (WHO), 2015
Malta Vital Registration - Deaths 1982 ICD9 as it appears in World Health Organization (WHO). WHO Mortality Database Version November 2015. Geneva, Switzerland: World Health Organization (WHO), 2015
Malta Vital Registration - Deaths 1983 ICD9 as it appears in World Health Organization (WHO). WHO Mortality Database Version November 2015. Geneva, Switzerland: World Health Organization (WHO), 2015
Malta Vital Registration - Deaths 1984 ICD9 as it appears in World Health Organization (WHO). WHO Mortality Database Version November 2015. Geneva, Switzerland: World Health Organization (WHO), 2015
Malta Vital Registration - Deaths 1985 ICD9 as it appears in World Health Organization (WHO). WHO Mortality Database Version November 2015. Geneva, Switzerland: World Health Organization (WHO), 2015
Malta Vital Registration - Deaths 1986 ICD9 as it appears in World Health Organization (WHO). WHO Mortality Database Version November 2015. Geneva, Switzerland: World Health Organization (WHO), 2015
Malta Vital Registration - Deaths 1987 ICD9 as it appears in World Health Organization (WHO). WHO Mortality Database Version November 2015. Geneva, Switzerland: World Health Organization (WHO), 2015
Malta Vital Registration - Deaths 1988 ICD9 as it appears in World Health Organization (WHO). WHO Mortality Database Version November 2015. Geneva, Switzerland: World Health Organization (WHO), 2015
Malta Vital Registration - Deaths 1989 ICD9 as it appears in World Health Organization (WHO). WHO Mortality Database Version November 2015. Geneva, Switzerland: World Health Organization (WHO), 2015
Malta Vital Registration - Deaths 1990 ICD9 as it appears in World Health Organization (WHO). WHO Mortality Database Version November 2015. Geneva, Switzerland: World Health Organization (WHO), 2015
Malta Vital Registration - Deaths 1991 ICD9 as it appears in World Health Organization (WHO). WHO Mortality Database Version November 2015. Geneva, Switzerland: World Health Organization (WHO), 2015

Malta Vital Registration - Deaths 1992 ICD9 as it appears in World Health Organization (WHO). WHO Mortality Database Version November 2015. Geneva, Switzerland: World Health Organization (WHO), 2015
Malta Vital Registration - Deaths 1994 ICD9 as it appears in World Health Organization (WHO). WHO Mortality Database Version November 2015. Geneva, Switzerland: World Health Organization (WHO), 2015
Malta Vital Registration - Deaths 1995 ICD10 as it appears in World Health Organization (WHO). WHO Mortality Database Version October 2017. Geneva, Switzerland: World Health Organization (WHO), 2017
Malta Vital Registration - Deaths 1996 ICD10 as it appears in World Health Organization (WHO). WHO Mortality Database Version October 2017. Geneva, Switzerland: World Health Organization (WHO), 2017
Malta Vital Registration - Deaths 1998 ICD10 as it appears in World Health Organization (WHO). WHO Mortality Database Version October 2017. Geneva, Switzerland: World Health Organization (WHO), 2017
Malta Vital Registration - Deaths 1999 ICD10 as it appears in World Health Organization (WHO). WHO Mortality Database Version October 2017. Geneva, Switzerland: World Health Organization (WHO), 2017
Malta Vital Registration - Deaths 2000 ICD10 as it appears in World Health Organization (WHO). WHO Mortality Database Version October 2017. Geneva, Switzerland: World Health Organization (WHO), 2017
Malta Vital Registration - Deaths 2001 ICD10 as it appears in World Health Organization (WHO). WHO Mortality Database Version October 2017. Geneva, Switzerland: World Health Organization (WHO), 2017
Malta Vital Registration - Deaths 2002 ICD10 as it appears in World Health Organization (WHO). WHO Mortality Database Version October 2017. Geneva, Switzerland: World Health Organization (WHO), 2017
Malta Vital Registration - Deaths 2004 ICD10 as it appears in World Health Organization (WHO). WHO Mortality Database Version October 2017. Geneva, Switzerland: World Health Organization (WHO), 2017
Malta Vital Registration - Deaths 2005 ICD10 as it appears in World Health Organization (WHO). WHO Mortality Database Version October 2017. Geneva, Switzerland: World Health Organization (WHO), 2017
Malta Vital Registration - Deaths 2006 ICD10 as it appears in World Health Organization (WHO). WHO Mortality Database Version October 2017. Geneva, Switzerland: World Health Organization (WHO), 2017
Malta Vital Registration - Deaths 2007 ICD10 as it appears in World Health Organization (WHO). WHO Mortality Database Version October 2017. Geneva, Switzerland: World Health Organization (WHO), 2017
Malta Vital Registration - Deaths 2008 ICD10 as it appears in World Health Organization (WHO). WHO Mortality Database Version October 2017. Geneva, Switzerland: World Health Organization (WHO), 2017
Malta Vital Registration - Deaths 1993 ICD9 as it appears in World Health Organization (WHO). WHO Mortality Database Version November 2015. Geneva, Switzerland: World Health Organization (WHO), 2015
Malta Vital Registration - Deaths 1997 ICD10 as it appears in World Health Organization (WHO). WHO Mortality Database Version October 2017. Geneva, Switzerland: World Health Organization (WHO), 2017
Malta Vital Registration - Deaths 2003 ICD10 as it appears in World Health Organization (WHO). WHO Mortality Database Version October 2017. Geneva, Switzerland: World Health Organization (WHO), 2017
Malta Vital Registration - Deaths 2009 ICD10 as it appears in World Health Organization (WHO). WHO Mortality Database Version October 2017. Geneva, Switzerland: World Health Organization (WHO), 2017

Malta Vital Registration - Deaths 2010 ICD10 as it appears in World Health Organization (WHO). WHO Mortality Database Version October 2017. Geneva, Switzerland: World Health Organization (WHO), 2017
Malta Vital Registration - Deaths 2011 ICD10 as it appears in World Health Organization (WHO). WHO Mortality Database Version October 2017. Geneva, Switzerland: World Health Organization (WHO), 2017
Malta Vital Registration - Deaths 2012 ICD10 as it appears in World Health Organization (WHO). WHO Mortality Database Version October 2017. Geneva, Switzerland: World Health Organization (WHO), 2017
Malta Vital Registration - Deaths 2013 ICD10 as it appears in World Health Organization (WHO). WHO Mortality Database Version October 2017. Geneva, Switzerland: World Health Organization (WHO), 2017
Malta Vital Registration - Deaths 2014 ICD10 as it appears in World Health Organization (WHO). WHO Mortality Database Version October 2017. Geneva, Switzerland: World Health Organization (WHO), 2017
Malta Vital Registration - Deaths 2015 ICD10 as it appears in World Health Organization (WHO). WHO Mortality Database Version April 2018. Geneva, Switzerland: World Health Organization (WHO), 2018
Manortney S, Carey A, Ansong D, Harvey R, Good B, Boaheng J, Crookston B, Dickerson T. Verbal Autopsy: an Analysis of the Common Causes of Childhood Death in the Barekese Sub-district of Ghana. J Public Health Afr. 2011; 2(e18): 73-7
Mauritius Vital Registration - Deaths 1980 ICD8 as it appears in World Health Organization (WHO). WHO Mortality Database Version July 2012. Geneva, Switzerland: World Health Organization (WHO), 2012
Mauritius Vital Registration - Deaths 1981 ICD9 as it appears in World Health Organization (WHO). WHO Mortality Database Version September 2016. Geneva, Switzerland: World Health Organization (WHO), 2016
Mauritius Vital Registration - Deaths 1982 ICD9 as it appears in World Health Organization (WHO). WHO Mortality Database Version September 2016. Geneva, Switzerland: World Health Organization (WHO), 2016
Mauritius Vital Registration - Deaths 1983 ICD9 as it appears in World Health Organization (WHO). WHO Mortality Database Version September 2016. Geneva, Switzerland: World Health Organization (WHO), 2016
Mauritius Vital Registration - Deaths 1984 ICD9 as it appears in World Health Organization (WHO). WHO Mortality Database Version September 2016. Geneva, Switzerland: World Health Organization (WHO), 2016
Mauritius Vital Registration - Deaths 1985 ICD9 as it appears in World Health Organization (WHO). WHO Mortality Database Version September 2016. Geneva, Switzerland: World Health Organization (WHO), 2016
Mauritius Vital Registration - Deaths 1986 ICD9 as it appears in World Health Organization (WHO). WHO Mortality Database Version September 2016. Geneva, Switzerland: World Health Organization (WHO), 2016
Mauritius Vital Registration - Deaths 1987 ICD9 as it appears in World Health Organization (WHO). WHO Mortality Database Version September 2016. Geneva, Switzerland: World Health Organization (WHO), 2016
Mauritius Vital Registration - Deaths 1988 ICD9 as it appears in World Health Organization (WHO). WHO Mortality Database Version September 2016. Geneva, Switzerland: World Health Organization (WHO), 2016
Mauritius Vital Registration - Deaths 1989 ICD9 as it appears in World Health Organization (WHO). WHO Mortality Database Version September 2016. Geneva, Switzerland: World Health Organization (WHO), 2016
Mauritius Vital Registration - Deaths 1990 ICD9 as it appears in World Health Organization (WHO). WHO Mortality Database Version September 2016. Geneva, Switzerland: World Health Organization (WHO), 2016

Mauritius Vital Registration - Deaths 1991 ICD9 as it appears in World Health Organization (WHO). WHO Mortality Database Version November 2015. Geneva, Switzerland: World Health Organization (WHO), 2015

Mauritius Vital Registration - Deaths 1992 ICD9 as it appears in World Health Organization (WHO). WHO Mortality Database Version November 2015. Geneva, Switzerland: World Health Organization (WHO), 2015

Mauritius Vital Registration - Deaths 1993 ICD9 as it appears in World Health Organization (WHO). WHO Mortality Database Version November 2015. Geneva, Switzerland: World Health Organization (WHO), 2015

Mauritius Vital Registration - Deaths 1994 ICD9 as it appears in World Health Organization (WHO). WHO Mortality Database Version November 2015. Geneva, Switzerland: World Health Organization (WHO), 2015

Mauritius Vital Registration - Deaths 1995 ICD9 as it appears in World Health Organization (WHO). WHO Mortality Database Version November 2015. Geneva, Switzerland: World Health Organization (WHO), 2015

Mauritius Vital Registration - Deaths 1996 ICD9 as it appears in World Health Organization (WHO). WHO Mortality Database Version November 2015. Geneva, Switzerland: World Health Organization (WHO), 2015

Mauritius Vital Registration - Deaths 1997 ICD9 as it appears in World Health Organization (WHO). WHO Mortality Database Version November 2015. Geneva, Switzerland: World Health Organization (WHO), 2015

Mauritius Vital Registration - Deaths 1998 ICD9 as it appears in World Health Organization (WHO). WHO Mortality Database Version November 2015. Geneva, Switzerland: World Health Organization (WHO), 2015

Mauritius Vital Registration - Deaths 1999 ICD9 as it appears in World Health Organization (WHO). WHO Mortality Database Version November 2015. Geneva, Switzerland: World Health Organization (WHO), 2015

Mauritius Vital Registration - Deaths 2000 ICD9 as it appears in World Health Organization (WHO). WHO Mortality Database Version November 2015. Geneva, Switzerland: World Health Organization (WHO), 2015

Mauritius Vital Registration - Deaths 2001 ICD9 as it appears in World Health Organization (WHO). WHO Mortality Database Version November 2015. Geneva, Switzerland: World Health Organization (WHO), 2015

Mauritius Vital Registration - Deaths 2003 ICD9 as it appears in World Health Organization (WHO). WHO Mortality Database Version November 2015. Geneva, Switzerland: World Health Organization (WHO), 2015

Mauritius Vital Registration - Deaths 2004 ICD9 as it appears in World Health Organization (WHO). WHO Mortality Database Version November 2015. Geneva, Switzerland: World Health Organization (WHO), 2015

Mauritius Vital Registration - Deaths 2005 ICD10 as it appears in World Health Organization (WHO). WHO Mortality Database Version October 2017. Geneva, Switzerland: World Health Organization (WHO), 2017

Mauritius Vital Registration - Deaths 2006 ICD10 as it appears in World Health Organization (WHO). WHO Mortality Database Version October 2017. Geneva, Switzerland: World Health Organization (WHO), 2017

Mauritius Vital Registration - Deaths 2007 ICD10 as it appears in World Health Organization (WHO). WHO Mortality Database Version October 2017. Geneva, Switzerland: World Health Organization (WHO), 2017

Mauritius Vital Registration - Deaths 2008 ICD10 as it appears in World Health Organization (WHO). WHO Mortality Database Version October 2017. Geneva, Switzerland: World Health Organization (WHO), 2017

Mauritius Vital Registration - Deaths 2002 ICD9 as it appears in World Health Organization (WHO). WHO Mortality Database Version November 2015. Geneva, Switzerland: World Health Organization (WHO), 2015

Mauritius Vital Registration - Deaths 2009 ICD10 as it appears in World Health Organization (WHO). WHO Mortality Database Version October 2017. Geneva, Switzerland: World Health Organization (WHO), 2017

Mauritius Vital Registration - Deaths 2010 ICD10 as it appears in World Health Organization (WHO). WHO Mortality Database Version October 2017. Geneva, Switzerland: World Health Organization (WHO), 2017

Mauritius Vital Registration - Deaths 2011 ICD10 as it appears in World Health Organization (WHO). WHO Mortality Database Version October 2017. Geneva, Switzerland: World Health Organization (WHO), 2017

Mauritius Vital Registration - Deaths 2012 ICD10 as it appears in World Health Organization (WHO). WHO Mortality Database Version October 2017. Geneva, Switzerland: World Health Organization (WHO), 2017

Mauritius Vital Registration - Deaths 2013 ICD10 as it appears in World Health Organization (WHO). WHO Mortality Database Version October 2017. Geneva, Switzerland: World Health Organization (WHO), 2017

Mauritius Vital Registration - Deaths 2014 ICD10 as it appears in World Health Organization (WHO). WHO Mortality Database Version October 2017. Geneva, Switzerland: World Health Organization (WHO), 2017

Mauritius Vital Registration - Deaths 2015 ICD10 as it appears in World Health Organization (WHO). WHO Mortality Database Version October 2017. Geneva, Switzerland: World Health Organization (WHO), 2017

Mauritius Vital Registration - Deaths 2016 ICD10 as it appears in World Health Organization (WHO). WHO Mortality Database Version October 2017. Geneva, Switzerland: World Health Organization (WHO), 2017

Memon ZA, Khan GN, Soofi SB. Impact of a community-based perinatal and newborn preventive care package on perinatal and neonatal mortality in a remote mountainous district in Northern Pakistan. BMC Pregnancy Childbirth. 2015; 15(106)

Menezes AMB, Victora CG, Barros FC, Albernaz E, Menezes FS, Jannke HA, Alves C, Rocha C. Infant mortality in two population-based cohorts in southern Brazil: trends and differentials. Cad Saude Publica. 1996; 12(Supp 1): 79-86

Ministry of Health (Brazil). Brazil Mortality Information System - Deaths 1980. Rio de Janeiro, Brazil: Ministry of Health (Brazil)

Ministry of Health (Brazil). Brazil Mortality Information System - Deaths 1981. Rio de Janeiro, Brazil: Ministry of Health (Brazil)

Ministry of Health (Brazil). Brazil Mortality Information System - Deaths 1982. Rio de Janeiro, Brazil: Ministry of Health (Brazil)

Ministry of Health (Brazil). Brazil Mortality Information System - Deaths 1983. Rio de Janeiro, Brazil: Ministry of Health (Brazil)

Ministry of Health (Brazil). Brazil Mortality Information System - Deaths 1984. Rio de Janeiro, Brazil: Ministry of Health (Brazil)

Ministry of Health (Brazil). Brazil Mortality Information System - Deaths 1985. Rio de Janeiro, Brazil: Ministry of Health (Brazil)

Ministry of Health (Brazil). Brazil Mortality Information System - Deaths 1986. Rio de Janeiro, Brazil: Ministry of Health (Brazil)

Ministry of Health (Brazil). Brazil Mortality Information System - Deaths 1987. Rio de Janeiro, Brazil: Ministry of Health (Brazil)

Ministry of Health (Brazil). Brazil Mortality Information System - Deaths 1988. Rio de Janeiro, Brazil: Ministry of Health (Brazil)

Ministry of Health (Brazil). Brazil Mortality Information System - Deaths 1989. Rio de Janeiro, Brazil: Ministry of Health (Brazil)

Ministry of Health (Brazil). Brazil Mortality Information System - Deaths 1990. Rio de Janeiro, Brazil: Ministry of Health (Brazil)

Ministry of Health (Brazil). Brazil Mortality Information System - Deaths 1991. Rio de Janeiro, Brazil: Ministry of Health (Brazil)

Ministry of Health (Brazil). Brazil Mortality Information System - Deaths 1992. Rio de Janeiro, Brazil: Ministry of Health (Brazil)

Ministry of Health (Brazil). Brazil Mortality Information System - Deaths 1993. Rio de Janeiro, Brazil: Ministry of Health (Brazil)

Ministry of Health (Brazil). Brazil Mortality Information System - Deaths 1994. Rio de Janeiro, Brazil: Ministry of Health (Brazil)

Ministry of Health (Brazil). Brazil Mortality Information System - Deaths 1995. Rio de Janeiro, Brazil: Ministry of Health (Brazil)
Ministry of Health (Brazil). Brazil Mortality Information System - Deaths 1996. Rio de Janeiro, Brazil: Ministry of Health (Brazil)
Ministry of Health (Brazil). Brazil Mortality Information System - Deaths 1997. Rio de Janeiro, Brazil: Ministry of Health (Brazil)
Ministry of Health (Brazil). Brazil Mortality Information System - Deaths 1998. Rio de Janeiro, Brazil: Ministry of Health (Brazil)
Ministry of Health (Brazil). Brazil Mortality Information System - Deaths 1999. Rio de Janeiro, Brazil: Ministry of Health (Brazil)
Ministry of Health (Brazil). Brazil Mortality Information System - Deaths 2000. Rio de Janeiro, Brazil: Ministry of Health (Brazil)
Ministry of Health (Brazil). Brazil Mortality Information System - Deaths 2001. Rio de Janeiro, Brazil: Ministry of Health (Brazil)
Ministry of Health (Brazil). Brazil Mortality Information System - Deaths 2002. Rio de Janeiro, Brazil: Ministry of Health (Brazil)
Ministry of Health (Brazil). Brazil Mortality Information System - Deaths 2003. Rio de Janeiro, Brazil: Ministry of Health (Brazil)
Ministry of Health (Brazil). Brazil Mortality Information System - Deaths 2004. Rio de Janeiro, Brazil: Ministry of Health (Brazil)
Ministry of Health (Brazil). Brazil Mortality Information System - Deaths 2005. Rio de Janeiro, Brazil: Ministry of Health (Brazil)
Ministry of Health (Brazil). Brazil Mortality Information System - Deaths 2006. Rio de Janeiro, Brazil: Ministry of Health (Brazil)
Ministry of Health (Brazil). Brazil Mortality Information System - Deaths 2007. Rio de Janeiro, Brazil: Ministry of Health (Brazil)
Ministry of Health (Brazil). Brazil Mortality Information System - Deaths 2008. Rio de Janeiro, Brazil: Ministry of Health (Brazil)
Ministry of Health (Brazil). Brazil Mortality Information System - Deaths 2009. Rio de Janeiro, Brazil: Ministry of Health (Brazil)
Ministry of Health (Brazil). Brazil Mortality Information System - Deaths 2010. Rio de Janeiro, Brazil: Ministry of Health (Brazil)
Ministry of Health (Brazil). Brazil Mortality Information System - Deaths 2011. Rio de Janeiro, Brazil: Ministry of Health (Brazil)
Ministry of Health (Brazil). Brazil Mortality Information System - Deaths 2012. Rio de Janeiro, Brazil: Ministry of Health (Brazil)
Ministry of Health (Brazil). Brazil Mortality Information System - Deaths 2013. Rio de Janeiro, Brazil: Ministry of Health (Brazil)
Ministry of Health (Brazil). Brazil Mortality Information System - Deaths 2014. Rio de Janeiro, Brazil: Ministry of Health (Brazil)
Ministry of Health (Brazil). Brazil Mortality Information System - Deaths 2015. Rio de Janeiro, Brazil: Ministry of Health (Brazil)
Ministry of Health (Brazil). Brazil Mortality Information System - Deaths 2016. Rio de Janeiro, Brazil: Ministry of Health (Brazil)
Ministry of Health (China). China National Maternal and Child Health Surveillance System Child Mortality By Cause 2013 - MCHS
Ministry of Health (China). China National Maternal and Child Health Surveillance System Child Mortality Data By Cause 1996-2012 - MCHS
Ministry of Health (Honduras). Honduras Maternal Mortality Ratio Update 2010. Honduras: Ministry of Health (Honduras), 2013
Ministry of Health (Indonesia). Indonesia Cause of Death Survey 2010-2011
Ministry of Health (Indonesia). Indonesia Sample Registration System - Deaths 2012
Ministry of Health (Indonesia). Indonesia Sample Registration System - Deaths 2013
Ministry of Health (Indonesia). Indonesia Sample Registration System - Deaths 2014
Ministry of Health (Jordan). Jordan Causes of Death and Population 2013
Ministry of Health (Jordan). Jordan Vital Registration - Deaths 2004-2006
Ministry of Health (Myanmar). Myanmar National Mortality Survey 2016
Ministry of Health (New Zealand). New Zealand Mortality Collection 1988. Wellington, New Zealand: Ministry of Health (New Zealand)
Ministry of Health (New Zealand). New Zealand Mortality Collection 1989. Wellington, New Zealand: Ministry of Health (New Zealand)

Ministry of Health (New Zealand). New Zealand Mortality Collection 1990. Wellington, New Zealand: Ministry of Health (New Zealand)

Ministry of Health (New Zealand). New Zealand Mortality Collection 1991. Wellington, New Zealand: Ministry of Health (New Zealand)

Ministry of Health (New Zealand). New Zealand Mortality Collection 1992. Wellington, New Zealand: Ministry of Health (New Zealand)

Ministry of Health (New Zealand). New Zealand Mortality Collection 1993. Wellington, New Zealand: Ministry of Health (New Zealand)

Ministry of Health (New Zealand). New Zealand Mortality Collection 1994. Wellington, New Zealand: Ministry of Health (New Zealand)

Ministry of Health (New Zealand). New Zealand Mortality Collection 1996. Wellington, New Zealand: Ministry of Health (New Zealand)

Ministry of Health (New Zealand). New Zealand Mortality Collection 1997. Wellington, New Zealand: Ministry of Health (New Zealand)

Ministry of Health (New Zealand). New Zealand Mortality Collection 1998. Wellington, New Zealand: Ministry of Health (New Zealand)

Ministry of Health (New Zealand). New Zealand Mortality Collection 1999. Wellington, New Zealand: Ministry of Health (New Zealand)

Ministry of Health (New Zealand). New Zealand Mortality Collection 2000. Wellington, New Zealand: Ministry of Health (New Zealand)

Ministry of Health (New Zealand). New Zealand Mortality Collection 2001. Wellington, New Zealand: Ministry of Health (New Zealand)

Ministry of Health (New Zealand). New Zealand Mortality Collection 2002. Wellington, New Zealand: Ministry of Health (New Zealand)

Ministry of Health (New Zealand). New Zealand Mortality Collection 2003. Wellington, New Zealand: Ministry of Health (New Zealand)

Ministry of Health (New Zealand). New Zealand Mortality Collection 2004. Wellington, New Zealand: Ministry of Health (New Zealand)

Ministry of Health (New Zealand). New Zealand Mortality Collection 2005. Wellington, New Zealand: Ministry of Health (New Zealand)

Ministry of Health (New Zealand). New Zealand Mortality Collection 2006. Wellington, New Zealand: Ministry of Health (New Zealand)

Ministry of Health (New Zealand). New Zealand Mortality Collection 2007. Wellington, New Zealand: Ministry of Health (New Zealand)

Ministry of Health (New Zealand). New Zealand Mortality Collection 2008. Wellington, New Zealand: Ministry of Health (New Zealand)

Ministry of Health (New Zealand). New Zealand Mortality Collection 2009. Wellington, New Zealand: Ministry of Health (New Zealand)

Ministry of Health (New Zealand). New Zealand Mortality Collection 2010. Wellington, New Zealand: Ministry of Health (New Zealand)

Ministry of Health (New Zealand). New Zealand Mortality Collection 2011. Wellington, New Zealand: Ministry of Health (New Zealand)

Ministry of Health (New Zealand). New Zealand Mortality Collection 2012. Wellington, New Zealand: Ministry of Health (New Zealand)

Ministry of Health (New Zealand). New Zealand Mortality Collection 2013. Wellington, New Zealand: Ministry of Health (New Zealand)

Ministry of Health (New Zealand). New Zealand Mortality Collection 2014. Wellington, New Zealand: Ministry of Health (New Zealand)

Ministry of Health (New Zealand). New Zealand Mortality Collection 2015. Wellington, New Zealand: Ministry of Health (New Zealand)

Ministry of Health (New Zealand). New Zealand Mortality Data 1980. Wellington, New Zealand: Ministry of Health (New Zealand)

Ministry of Health (New Zealand). New Zealand Mortality Data 1981. Wellington, New Zealand: Ministry of Health (New Zealand)

Ministry of Health (New Zealand). New Zealand Mortality Data 1982. Wellington, New Zealand: Ministry of Health (New Zealand)

Ministry of Health (New Zealand). New Zealand Mortality Data 1983. Wellington, New Zealand: Ministry of Health (New Zealand)

Ministry of Health (New Zealand). New Zealand Mortality Data 1984. Wellington, New Zealand: Ministry of Health (New Zealand)

Ministry of Health (New Zealand). New Zealand Mortality Data 1985. Wellington, New Zealand: Ministry of Health (New Zealand)

Ministry of Health (New Zealand). New Zealand Mortality Data 1986. Wellington, New Zealand: Ministry of Health (New Zealand)

Ministry of Health (New Zealand). New Zealand Mortality Data 1987. Wellington, New Zealand: Ministry of Health (New Zealand)

Ministry of Health (Palestine). Palestine - Gaza Strip and West Bank Mortality by Sex, Age, and Cause 2012

Ministry of Health (Palestine). Palestine - Gaza Strip and West Bank Mortality by Sex, Age, and Cause 2013

Ministry of Health (Palestine). Palestine - Gaza Strip and West Bank Mortality by Sex, Age, and Cause 2014
Ministry of Health (Palestine). Palestine - Gaza Strip and West Bank Mortality by Sex, Age, and Cause 2015
Ministry of Health (Palestine). Palestine - Gaza Strip and West Bank Mortality by Sex, Age, and Cause 2016
Ministry of Health (Saudi Arabia). Saudi Arabia Vital Registration - Deaths 1996-2011
Ministry of Health (Singapore). Singapore Causes of Death 1980
Ministry of Health (Singapore). Singapore Causes of Death 1981
Ministry of Health (Singapore). Singapore Causes of Death 1982
Ministry of Health (Singapore). Singapore Causes of Death 1983
Ministry of Health (Singapore). Singapore Causes of Death 1984
Ministry of Health (Singapore). Singapore Causes of Death 1985
Ministry of Health (Singapore). Singapore Causes of Death 1986
Ministry of Health (Singapore). Singapore Causes of Death 1987
Ministry of Health (Singapore). Singapore Causes of Death 1988
Ministry of Health (Singapore). Singapore Causes of Death 1989
Ministry of Health (Singapore). Singapore Causes of Death 1990
Ministry of Health (Singapore). Singapore Causes of Death 1991
Ministry of Health (Singapore). Singapore Causes of Death 1992
Ministry of Health (Singapore). Singapore Causes of Death 1993
Ministry of Health (Singapore). Singapore Causes of Death 1994
Ministry of Health (Singapore). Singapore Causes of Death 1995
Ministry of Health (Singapore). Singapore Causes of Death 1996
Ministry of Health (Singapore). Singapore Causes of Death 1997
Ministry of Health (Singapore). Singapore Causes of Death 1998
Ministry of Health (Singapore). Singapore Causes of Death 1999
Ministry of Health (Singapore). Singapore Causes of Death 2000
Ministry of Health (Singapore). Singapore Causes of Death 2001
Ministry of Health (Singapore). Singapore Causes of Death 2002
Ministry of Health (Singapore). Singapore Causes of Death 2003
Ministry of Health (Singapore). Singapore Causes of Death 2004
Ministry of Health (Singapore). Singapore Causes of Death 2005
Ministry of Health (Singapore). Singapore Causes of Death 2006
Ministry of Health (Singapore). Singapore Causes of Death 2007
Ministry of Health (Singapore). Singapore Causes of Death 2008
Ministry of Health (Singapore). Singapore Causes of Death 2009
Ministry of Health (Singapore). Singapore Causes of Death 2010

Ministry of Health (Singapore). Singapore Causes of Death 2011
Ministry of Health (Singapore). Singapore Causes of Death 2012
Ministry of Health (Singapore). Singapore Causes of Death 2013
Ministry of Health (Singapore). Singapore Causes of Death 2014
Ministry of Health (Singapore). Singapore Causes of Death 2015
Ministry of Health (Singapore). Singapore Causes of Death 2016
Ministry of Health (Tonga). Tonga Vital Statistics - Deaths 2003
Ministry of Health (United Arab Emirates). United Arab Emirates Annual Statistical Report 2006. Abu Dhabi, United Arab Emirates: Ministry of Health (United Arab Emirates)
Ministry of Health (United Arab Emirates). United Arab Emirates Annual Statistical Report 2007. Abu Dhabi, United Arab Emirates: Ministry of Health (United Arab Emirates)
Ministry of Health and Medical Education (Iran). Iran Death Registration System 2001
Ministry of Health and Medical Education (Iran). Iran Death Registration System 2002
Ministry of Health and Medical Education (Iran). Iran Death Registration System 2003
Ministry of Health and Medical Education (Iran). Iran Death Registration System 2004
Ministry of Health and Medical Education (Iran). Iran Death Registration System 2005
Ministry of Health and Medical Education (Iran). Iran Death Registration System 2006
Ministry of Health and Medical Education (Iran). Iran Death Registration System 2007
Ministry of Health and Medical Education (Iran). Iran Death Registration System 2008
Ministry of Health and Medical Education (Iran). Iran Death Registration System 2009
Ministry of Health and Medical Education (Iran). Iran Death Registration System 2010
Ministry of Health and Medical Education (Iran). Iran Death Registration System 2011
Ministry of Health and Medical Education (Iran). Iran Death Registration System 2012
Ministry of Health and Medical Education (Iran). Iran Death Registration System 2013
Ministry of Health and Medical Education (Iran). Iran Death Registration System 2014
Ministry of Health and Medical Education (Iran). Iran Death Registration System 2015
Ministry of Health and Welfare (Taiwan). Taiwan Vital Registration - Deaths 2013. Taipei City, Taiwan: Ministry of Health and Welfare (Taiwan)
Ministry of Health and Welfare (Taiwan). Taiwan Vital Registration - Deaths 2014. Taipei City, Taiwan: Ministry of Health and Welfare (Taiwan)
Ministry of Health and Welfare (Taiwan). Taiwan Vital Registration - Deaths 2015. Taipei City, Taiwan: Ministry of Health and Welfare (Taiwan)
Ministry of Health and Welfare (Taiwan). Taiwan Vital Registration - Deaths 2016. Taipei City, Taiwan: Ministry of Health and Welfare (Taiwan)
Ministry of Health, Labour and Welfare (Japan). Japan Vital Registration - Deaths 1980

Ministry of Health, Labour and Welfare (Japan). Japan Vital Registration - Deaths 1981
Ministry of Health, Labour and Welfare (Japan). Japan Vital Registration - Deaths 1982
Ministry of Health, Labour and Welfare (Japan). Japan Vital Registration - Deaths 1983
Ministry of Health, Labour and Welfare (Japan). Japan Vital Registration - Deaths 1984
Ministry of Health, Labour and Welfare (Japan). Japan Vital Registration - Deaths 1985
Ministry of Health, Labour and Welfare (Japan). Japan Vital Registration - Deaths 1986
Ministry of Health, Labour and Welfare (Japan). Japan Vital Registration - Deaths 1987
Ministry of Health, Labour and Welfare (Japan). Japan Vital Registration - Deaths 1988
Ministry of Health, Labour and Welfare (Japan). Japan Vital Registration - Deaths 1989
Ministry of Health, Labour and Welfare (Japan). Japan Vital Registration - Deaths 1990
Ministry of Health, Labour and Welfare (Japan). Japan Vital Registration - Deaths 1991
Ministry of Health, Labour and Welfare (Japan). Japan Vital Registration - Deaths 1992
Ministry of Health, Labour and Welfare (Japan). Japan Vital Registration - Deaths 1993
Ministry of Health, Labour and Welfare (Japan). Japan Vital Registration - Deaths 1994
Ministry of Health, Labour and Welfare (Japan). Japan Vital Registration - Deaths 1995
Ministry of Health, Labour and Welfare (Japan). Japan Vital Registration - Deaths 1996
Ministry of Health, Labour and Welfare (Japan). Japan Vital Registration - Deaths 1997
Ministry of Health, Labour and Welfare (Japan). Japan Vital Registration - Deaths 1998
Ministry of Health, Labour and Welfare (Japan). Japan Vital Registration - Deaths 1999
Ministry of Health, Labour and Welfare (Japan). Japan Vital Registration - Deaths 2000
Ministry of Health, Labour and Welfare (Japan). Japan Vital Registration - Deaths 2001
Ministry of Health, Labour and Welfare (Japan). Japan Vital Registration - Deaths 2002
Ministry of Health, Labour and Welfare (Japan). Japan Vital Registration - Deaths 2003
Ministry of Health, Labour and Welfare (Japan). Japan Vital Registration - Deaths 2004
Ministry of Health, Labour and Welfare (Japan). Japan Vital Registration - Deaths 2005
Ministry of Health, Labour and Welfare (Japan). Japan Vital Registration - Deaths 2006
Ministry of Health, Labour and Welfare (Japan). Japan Vital Registration - Deaths 2007
Ministry of Health, Labour and Welfare (Japan). Japan Vital Registration - Deaths 2008
Ministry of Health, Labour and Welfare (Japan). Japan Vital Registration - Deaths 2009
Ministry of Health, Labour and Welfare (Japan). Japan Vital Registration - Deaths 2010
Ministry of Health, Labour and Welfare (Japan). Japan Vital Registration - Deaths 2011
Ministry of Health, Labour and Welfare (Japan). Japan Vital Registration - Deaths 2012
Ministry of Health, Labour and Welfare (Japan). Japan Vital Registration - Deaths 2013
Ministry of Health, Labour and Welfare (Japan). Japan Vital Registration - Deaths 2014
Ministry of Health, Labour and Welfare (Japan). Japan Vital Registration - Deaths 2015

Ministry of Public Health (Morocco). Morocco National Survey on Causes and Circumstances of Infant and Child Deaths 1988-1989

Ministry of Public Health (Thailand). Thailand Burden of Disease and Injuries 1998-1999

Ministry of Public Health (Uruguay). Uruguay Vital Registration - Deaths 1991

Mitra and Associates, ORC Macro. Bangladesh Demographic and Health Survey 2004. Fairfax, United States: ICF International

Moir JS, Garner PA, Heywood PF, Alpers MP. Mortality in a rural area of Madang Province, Papua New Guinea. Ann Trop Med Parasitol. 1989; 83(3): 305-19

Moldova Vital Registration - Deaths 2010 ICD10 as it appears in World Health Organization (WHO). WHO Mortality Database Version October 2017. Geneva, Switzerland: World Health Organization (WHO), 2017

Moldova Vital Registration - Deaths 2011 ICD10 as it appears in World Health Organization (WHO). WHO Mortality Database Version October 2017. Geneva, Switzerland: World Health Organization (WHO), 2017

Moldova Vital Registration - Deaths 2012 ICD10 as it appears in World Health Organization (WHO). WHO Mortality Database Version October 2017. Geneva, Switzerland: World Health Organization (WHO), 2017

Moldova Vital Registration - Deaths 2013 ICD10 as it appears in World Health Organization (WHO). WHO Mortality Database Version October 2017. Geneva, Switzerland: World Health Organization (WHO), 2017

Moldova Vital Registration - Deaths 2014 ICD10 as it appears in World Health Organization (WHO). WHO Mortality Database Version October 2017. Geneva, Switzerland: World Health Organization (WHO), 2017

Moldova Vital Registration - Deaths 2015 ICD10 as it appears in World Health Organization (WHO). WHO Mortality Database Version March 2017. Geneva, Switzerland: World Health Organization (WHO), 2017

Moldova Vital Registration - Deaths 2016 ICD10 as it appears in World Health Organization (WHO). WHO Mortality Database Version April 2018. Geneva, Switzerland: World Health Organization (WHO), 2018

Mongolia Vital Registration - Deaths 1994 ICD9 as it appears in World Health Organization (WHO). WHO Mortality Database Version September 2016. Geneva, Switzerland: World Health Organization (WHO), 2016

Mongolia Vital Registration - Deaths 2016 ICD10 as it appears in World Health Organization (WHO). WHO Mortality Database Version April 2018. Geneva, Switzerland: World Health Organization (WHO), 2018

Montenegro Vital Registration - Deaths 2001 ICD10 as it appears in World Health Organization (WHO). WHO Mortality Database Version October 2017. Geneva, Switzerland: World Health Organization (WHO), 2017

Montenegro Vital Registration - Deaths 2002 ICD10 as it appears in World Health Organization (WHO). WHO Mortality Database Version October 2017. Geneva, Switzerland: World Health Organization (WHO), 2017

Montenegro Vital Registration - Deaths 2005 ICD10 as it appears in World Health Organization (WHO). WHO Mortality Database Version April 2018. Geneva, Switzerland: World Health Organization (WHO), 2018

Montenegro Vital Registration - Deaths 2006 ICD10 as it appears in World Health Organization (WHO). WHO Mortality Database Version April 2018. Geneva, Switzerland: World Health Organization (WHO), 2018

Montenegro Vital Registration - Deaths 2007 ICD10 as it appears in World Health Organization (WHO). WHO Mortality Database Version April 2018. Geneva, Switzerland: World Health Organization (WHO), 2018

Montenegro Vital Registration - Deaths 2008 ICD10 as it appears in World Health Organization (WHO). WHO Mortality Database Version April 2018. Geneva, Switzerland: World Health Organization (WHO), 2018

Montenegro Vital Registration - Deaths 2009 ICD10 as it appears in World Health Organization (WHO). WHO Mortality Database Version October 2017. Geneva, Switzerland: World Health Organization (WHO), 2017

Mo-suwan L, Isaranurug S, Chanvitan P, Techasena W, Sutra S, Supakunpinyo C, Choprapawon C. Perinatal death pattern in the four districts of Thailand: findings from the Prospective Cohort Study of Thai Children (PCTC). J Med Assoc Thai. 2009; 92(5): 660-6

Myint, S, Ministry of Health (Myanmar). Cause of Death Verification Study in Myanmar. Presentation at: World Health Organization Regional Office for South East Asia. Regional Consultation on Mortality Statistics; 2007; New Delhi, India

Nahar S, Mostafa OA, Farheen A. Verbal autopsy for neonatal mortality: a community-based study. Med J Cairo Univ. 2012; 80(2): 25-9

National Administrative Department of Statistics (DANE) (Colombia). Colombia Vital Statistics - Deaths 2008. Bogotá, Colombia: National Administrative Department of Statistics (DANE) (Colombia)

National Board of Health and Welfare (Sweden). Sweden - Stockholm County Vital Registration - Deaths 1980

National Board of Health and Welfare (Sweden). Sweden - Stockholm County Vital Registration - Deaths 1981

National Board of Health and Welfare (Sweden). Sweden - Stockholm County Vital Registration - Deaths 1982

National Board of Health and Welfare (Sweden). Sweden - Stockholm County Vital Registration - Deaths 1983

National Board of Health and Welfare (Sweden). Sweden - Stockholm County Vital Registration - Deaths 1984

National Board of Health and Welfare (Sweden). Sweden - Stockholm County Vital Registration - Deaths 1985

National Board of Health and Welfare (Sweden). Sweden - Stockholm County Vital Registration - Deaths 1986

National Board of Health and Welfare (Sweden). Sweden Cause of Death Register 1987

National Board of Health and Welfare (Sweden). Sweden Cause of Death Register 1988

National Board of Health and Welfare (Sweden). Sweden Cause of Death Register 1989

National Board of Health and Welfare (Sweden). Sweden Cause of Death Register 1990

National Board of Health and Welfare (Sweden). Sweden Cause of Death Register 1991

National Board of Health and Welfare (Sweden). Sweden Cause of Death Register 1992

National Board of Health and Welfare (Sweden). Sweden Cause of Death Register 1993

National Board of Health and Welfare (Sweden). Sweden Cause of Death Register 1994

National Board of Health and Welfare (Sweden). Sweden Cause of Death Register 1995

National Board of Health and Welfare (Sweden). Sweden Cause of Death Register 1996

National Board of Health and Welfare (Sweden). Sweden Cause of Death Register 1997

National Board of Health and Welfare (Sweden). Sweden Cause of Death Register 1998

National Board of Health and Welfare (Sweden). Sweden Cause of Death Register 1999

National Board of Health and Welfare (Sweden). Sweden Cause of Death Register 2000

National Board of Health and Welfare (Sweden). Sweden Cause of Death Register 2001

National Board of Health and Welfare (Sweden). Sweden Cause of Death Register 2002

National Board of Health and Welfare (Sweden). Sweden Cause of Death Register 2003

National Board of Health and Welfare (Sweden). Sweden Cause of Death Register 2004

National Board of Health and Welfare (Sweden). Sweden Cause of Death Register 2005
National Board of Health and Welfare (Sweden). Sweden Cause of Death Register 2006
National Board of Health and Welfare (Sweden). Sweden Cause of Death Register 2007
National Board of Health and Welfare (Sweden). Sweden Cause of Death Register 2008
National Board of Health and Welfare (Sweden). Sweden Cause of Death Register 2009
National Board of Health and Welfare (Sweden). Sweden Cause of Death Register 2010
National Board of Health and Welfare (Sweden). Sweden Cause of Death Register 2011
National Board of Health and Welfare (Sweden). Sweden Cause of Death Register 2012. Stockholm, Sweden: National Board of Health and Welfare (Sweden)
National Board of Health and Welfare (Sweden). Sweden Cause of Death Register 2013. Stockholm, Sweden: National Board of Health and Welfare (Sweden)
National Board of Health and Welfare (Sweden). Sweden Cause of Death Register 2014. Stockholm, Sweden: National Board of Health and Welfare (Sweden)
National Board of Health and Welfare (Sweden). Sweden Cause of Death Register 2015. Stockholm, Sweden: National Board of Health and Welfare (Sweden)
National Board of Health and Welfare (Sweden). Sweden Cause of Death Register 2016. Stockholm, Sweden: National Board of Health and Welfare (Sweden)
National Board of Health and Welfare (Sweden). Sweden Vital Registration - Deaths 1980
National Board of Health and Welfare (Sweden). Sweden Vital Registration - Deaths 1981
National Board of Health and Welfare (Sweden). Sweden Vital Registration - Deaths 1982
National Board of Health and Welfare (Sweden). Sweden Vital Registration - Deaths 1983
National Board of Health and Welfare (Sweden). Sweden Vital Registration - Deaths 1984
National Board of Health and Welfare (Sweden). Sweden Vital Registration - Deaths 1985
National Board of Health and Welfare (Sweden). Sweden Vital Registration - Deaths 1986
National Center for Health Statistics (NCHS), Centers for Disease Control and Prevention (CDC), U.S. Department of Defense. United States NVSS Custom Mortality Data 2005 and United States Military Deaths 1980-2014
National Center for Health Statistics (NCHS), Centers for Disease Control and Prevention (CDC), U.S. Department of Defense. United States NVSS Custom Mortality Data 2006 and United States Military Deaths 1980-2014
National Center for Health Statistics (NCHS), Centers for Disease Control and Prevention (CDC), U.S. Department of Defense. United States NVSS Custom Mortality Data 2007 and United States Military Deaths 1980-2014
National Center for Health Statistics (NCHS), Centers for Disease Control and Prevention (CDC), U.S. Department of Defense. United States NVSS Custom Mortality Data 2008 and United States Military Deaths 1980-2014
National Center for Health Statistics (NCHS), Centers for Disease Control and Prevention (CDC), U.S. Department of Defense. United States NVSS Custom Mortality Data 2009 and United States Military Deaths 1980-2014
National Center for Health Statistics (NCHS), Centers for Disease Control and Prevention (CDC), U.S. Department of Defense. United States NVSS Custom Mortality Data 2010 and United States Military Deaths 1980-2014

National Center for Health Statistics (NCHS), Centers for Disease Control and Prevention (CDC), U.S. Department of Defense. United States NVSS Custom Mortality Data 2011 and United States Military Deaths 1980-2014
National Center for Health Statistics (NCHS), Centers for Disease Control and Prevention (CDC), U.S. Department of Defense. United States NVSS Custom Mortality Data 2012 and United States Military Deaths 1980-2014
National Center for Health Statistics (NCHS), Centers for Disease Control and Prevention (CDC), U.S. Department of Defense. United States NVSS Custom Mortality Data 2013 and United States Military Deaths 1980-2014
National Center for Health Statistics (NCHS), Centers for Disease Control and Prevention (CDC), U.S. Department of Defense. United States NVSS Custom Mortality Data 2014 and United States Military Deaths 1980-2014
National Center for Health Statistics (NCHS), Centers for Disease Control and Prevention (CDC), U.S. Department of Defense. United States NVSS Mortality Data 1980 - NBER and United States Military Deaths 1980-2014
National Center for Health Statistics (NCHS), Centers for Disease Control and Prevention (CDC), U.S. Department of Defense. United States NVSS Mortality Data 1981 - NBER and United States Military Deaths 1980-2014
National Center for Health Statistics (NCHS), Centers for Disease Control and Prevention (CDC), U.S. Department of Defense. United States NVSS Mortality Data 1982 - NBER and United States Military Deaths 1980-2014
National Center for Health Statistics (NCHS), Centers for Disease Control and Prevention (CDC), U.S. Department of Defense. United States NVSS Mortality Data 1983 - NBER and United States Military Deaths 1980-2014
National Center for Health Statistics (NCHS), Centers for Disease Control and Prevention (CDC), U.S. Department of Defense. United States NVSS Mortality Data 1984 - NBER and United States Military Deaths 1980-2014
National Center for Health Statistics (NCHS), Centers for Disease Control and Prevention (CDC), U.S. Department of Defense. United States NVSS Mortality Data 1985 - NBER and United States Military Deaths 1980-2014
National Center for Health Statistics (NCHS), Centers for Disease Control and Prevention (CDC), U.S. Department of Defense. United States NVSS Mortality Data 1986 - NBER and United States Military Deaths 1980-2014
National Center for Health Statistics (NCHS), Centers for Disease Control and Prevention (CDC), U.S. Department of Defense. United States NVSS Mortality Data 1987 - NBER and United States Military Deaths 1980-2014
National Center for Health Statistics (NCHS), Centers for Disease Control and Prevention (CDC), U.S. Department of Defense. United States NVSS Mortality Data 1988 - NBER and United States Military Deaths 1980-2014
National Center for Health Statistics (NCHS), Centers for Disease Control and Prevention (CDC), U.S. Department of Defense. United States NVSS Mortality Data 1989 - NBER and United States Military Deaths 1980-2014
National Center for Health Statistics (NCHS), Centers for Disease Control and Prevention (CDC), U.S. Department of Defense. United States NVSS Mortality Data 1990 - NBER and United States Military Deaths 1980-2014
National Center for Health Statistics (NCHS), Centers for Disease Control and Prevention (CDC), U.S. Department of Defense. United States NVSS Mortality Data 1991 - NBER and United States Military Deaths 1980-2014
National Center for Health Statistics (NCHS), Centers for Disease Control and Prevention (CDC), U.S. Department of Defense. United States NVSS Mortality Data 1992 - NBER and United States Military Deaths 1980-2014
National Center for Health Statistics (NCHS), Centers for Disease Control and Prevention (CDC), U.S. Department of Defense. United States NVSS Mortality Data 1993 - NBER and United States Military Deaths 1980-2014

National Center for Health Statistics (NCHS), Centers for Disease Control and Prevention (CDC), U.S. Department of Defense. United States NVSS Mortality Data 1994 - NBER and United States Military Deaths 1980-2014
National Center for Health Statistics (NCHS), Centers for Disease Control and Prevention (CDC), U.S. Department of Defense. United States NVSS Mortality Data 1995 - NBER and United States Military Deaths 1980-2014
National Center for Health Statistics (NCHS), Centers for Disease Control and Prevention (CDC), U.S. Department of Defense. United States NVSS Mortality Data 1996 - NBER and United States Military Deaths 1980-2014
National Center for Health Statistics (NCHS), Centers for Disease Control and Prevention (CDC), U.S. Department of Defense. United States NVSS Mortality Data 1997 - NBER and United States Military Deaths 1980-2014
National Center for Health Statistics (NCHS), Centers for Disease Control and Prevention (CDC), U.S. Department of Defense. United States NVSS Mortality Data 1998 - NBER and United States Military Deaths 1980-2014
National Center for Health Statistics (NCHS), Centers for Disease Control and Prevention (CDC), U.S. Department of Defense. United States NVSS Mortality Data 1999 - NBER and United States Military Deaths 1980-2014
National Center for Health Statistics (NCHS), Centers for Disease Control and Prevention (CDC), U.S. Department of Defense. United States NVSS Mortality Data 2000 - NBER and United States Military Deaths 1980-2014
National Center for Health Statistics (NCHS), Centers for Disease Control and Prevention (CDC), U.S. Department of Defense. United States NVSS Mortality Data 2001 - NBER and United States Military Deaths 1980-2014
National Center for Health Statistics (NCHS), Centers for Disease Control and Prevention (CDC), U.S. Department of Defense. United States NVSS Mortality Data 2002 - NBER and United States Military Deaths 1980-2014
National Center for Health Statistics (NCHS), Centers for Disease Control and Prevention (CDC), U.S. Department of Defense. United States NVSS Mortality Data 2003 - NBER and United States Military Deaths 1980-2014
National Center for Health Statistics (NCHS), Centers for Disease Control and Prevention (CDC), U.S. Department of Defense. United States NVSS Mortality Data 2004 - NBER and United States Military Deaths 1980-2014
National Center for Health Statistics, Centers for Disease Control and Prevention. American Samoa NVSS Custom Mortality Data 2005. Hyattsville, United States: National Center for Health Statistics, Centers for Disease Control and Prevention
National Center for Health Statistics, Centers for Disease Control and Prevention. American Samoa NVSS Custom Mortality Data 2006. Hyattsville, United States: National Center for Health Statistics, Centers for Disease Control and Prevention
National Center for Health Statistics, Centers for Disease Control and Prevention. American Samoa NVSS Custom Mortality Data 2007. Hyattsville, United States: National Center for Health Statistics, Centers for Disease Control and Prevention
National Center for Health Statistics, Centers for Disease Control and Prevention. American Samoa NVSS Custom Mortality Data 2008. Hyattsville, United States: National Center for Health Statistics, Centers for Disease Control and Prevention
National Center for Health Statistics, Centers for Disease Control and Prevention. American Samoa NVSS Custom Mortality Data 2009. Hyattsville, United States: National Center for Health Statistics, Centers for Disease Control and Prevention
National Center for Health Statistics, Centers for Disease Control and Prevention. American Samoa NVSS Custom Mortality Data 2010. Hyattsville, United States: National Center for Health Statistics, Centers for Disease Control and Prevention
National Center for Health Statistics, Centers for Disease Control and Prevention. American Samoa NVSS Custom Mortality Data 2011. Hyattsville, United States: National Center for Health Statistics, Centers for Disease Control and Prevention

National Center for Health Statistics, Centers for Disease Control and Prevention. American Samoa NVSS Custom Mortality Data 2012. Hyattsville, United States: National Center for Health Statistics, Centers for Disease Control and Prevention
National Center for Health Statistics, Centers for Disease Control and Prevention. American Samoa NVSS Custom Mortality Data 2013. Hyattsville, United States: National Center for Health Statistics, Centers for Disease Control and Prevention
National Center for Health Statistics, Centers for Disease Control and Prevention. American Samoa NVSS Custom Mortality Data 2014. Hyattsville, United States: National Center for Health Statistics, Centers for Disease Control and Prevention
National Center for Health Statistics, Centers for Disease Control and Prevention. American Samoa NVSS Custom Mortality Data 2015. Hyattsville, United States: National Center for Health Statistics, Centers for Disease Control and Prevention
National Center for Health Statistics, Centers for Disease Control and Prevention. American Samoa NVSS Mortality Data 1997 - NBER. Hyattsville, United States: National Center for Health Statistics, Centers for Disease Control and Prevention
National Center for Health Statistics, Centers for Disease Control and Prevention. American Samoa NVSS Mortality Data 1998 - NBER. Hyattsville, United States: National Center for Health Statistics, Centers for Disease Control and Prevention
National Center for Health Statistics, Centers for Disease Control and Prevention. American Samoa NVSS Mortality Data 1999 - NBER. Hyattsville, United States: National Center for Health Statistics, Centers for Disease Control and Prevention
National Center for Health Statistics, Centers for Disease Control and Prevention. American Samoa NVSS Mortality Data 2000 - NBER. Hyattsville, United States: National Center for Health Statistics, Centers for Disease Control and Prevention
National Center for Health Statistics, Centers for Disease Control and Prevention. American Samoa NVSS Mortality Data 2001 - NBER. Hyattsville, United States: National Center for Health Statistics, Centers for Disease Control and Prevention
National Center for Health Statistics, Centers for Disease Control and Prevention. American Samoa NVSS Mortality Data 2002 - NBER. Hyattsville, United States: National Center for Health Statistics, Centers for Disease Control and Prevention
National Center for Health Statistics, Centers for Disease Control and Prevention. American Samoa NVSS Mortality Data 2003 - NBER. Hyattsville, United States: National Center for Health Statistics, Centers for Disease Control and Prevention
National Center for Health Statistics, Centers for Disease Control and Prevention. American Samoa NVSS Mortality Data 2004 - NBER. Hyattsville, United States: National Center for Health Statistics, Centers for Disease Control and Prevention
National Center for Health Statistics, Centers for Disease Control and Prevention. Guam NVSS Custom Mortality Data 2005. Hyattsville, United States: National Center for Health Statistics, Centers for Disease Control and Prevention
National Center for Health Statistics, Centers for Disease Control and Prevention. Guam NVSS Custom Mortality Data 2006. Hyattsville, United States: National Center for Health Statistics, Centers for Disease Control and Prevention
National Center for Health Statistics, Centers for Disease Control and Prevention. Guam NVSS Custom Mortality Data 2007. Hyattsville, United States: National Center for Health Statistics, Centers for Disease Control and Prevention
National Center for Health Statistics, Centers for Disease Control and Prevention. Guam NVSS Custom Mortality Data 2008. Hyattsville, United States: National Center for Health Statistics, Centers for Disease Control and Prevention
National Center for Health Statistics, Centers for Disease Control and Prevention. Guam NVSS Custom Mortality Data 2009. Hyattsville, United States: National Center for Health Statistics, Centers for Disease Control and Prevention
National Center for Health Statistics, Centers for Disease Control and Prevention. Guam NVSS Custom Mortality Data 2010. Hyattsville, United States: National Center for Health Statistics, Centers for Disease Control and Prevention

National Center for Health Statistics, Centers for Disease Control and Prevention. Guam NVSS Custom Mortality Data 2011. Hyattsville, United States: National Center for Health Statistics, Centers for Disease Control and Prevention

National Center for Health Statistics, Centers for Disease Control and Prevention. Guam NVSS Custom Mortality Data 2012. Hyattsville, United States: National Center for Health Statistics, Centers for Disease Control and Prevention

National Center for Health Statistics, Centers for Disease Control and Prevention. Guam NVSS Custom Mortality Data 2013. Hyattsville, United States: National Center for Health Statistics, Centers for Disease Control and Prevention

National Center for Health Statistics, Centers for Disease Control and Prevention. Guam NVSS Custom Mortality Data 2014. Hyattsville, United States: National Center for Health Statistics, Centers for Disease Control and Prevention

National Center for Health Statistics, Centers for Disease Control and Prevention. Guam NVSS Custom Mortality Data 2015. Hyattsville, United States: National Center for Health Statistics, Centers for Disease Control and Prevention

National Center for Health Statistics, Centers for Disease Control and Prevention. Guam NVSS Mortality Data 1994 - NBER. Hyattsville, United States: National Center for Health Statistics, Centers for Disease Control and Prevention

National Center for Health Statistics, Centers for Disease Control and Prevention. Guam NVSS Mortality Data 1995 - NBER. Hyattsville, United States: National Center for Health Statistics, Centers for Disease Control and Prevention

National Center for Health Statistics, Centers for Disease Control and Prevention. Guam NVSS Mortality Data 1996 - NBER. Hyattsville, United States: National Center for Health Statistics, Centers for Disease Control and Prevention

National Center for Health Statistics, Centers for Disease Control and Prevention. Guam NVSS Mortality Data 1997 - NBER. Hyattsville, United States: National Center for Health Statistics, Centers for Disease Control and Prevention

National Center for Health Statistics, Centers for Disease Control and Prevention. Guam NVSS Mortality Data 1998 - NBER. Hyattsville, United States: National Center for Health Statistics, Centers for Disease Control and Prevention

National Center for Health Statistics, Centers for Disease Control and Prevention. Guam NVSS Mortality Data 1999 - NBER. Hyattsville, United States: National Center for Health Statistics, Centers for Disease Control and Prevention

National Center for Health Statistics, Centers for Disease Control and Prevention. Guam NVSS Mortality Data 2000 - NBER. Hyattsville, United States: National Center for Health Statistics, Centers for Disease Control and Prevention

National Center for Health Statistics, Centers for Disease Control and Prevention. Guam NVSS Mortality Data 2001 - NBER. Hyattsville, United States: National Center for Health Statistics, Centers for Disease Control and Prevention

National Center for Health Statistics, Centers for Disease Control and Prevention. Guam NVSS Mortality Data 2002 - NBER. Hyattsville, United States: National Center for Health Statistics, Centers for Disease Control and Prevention

National Center for Health Statistics, Centers for Disease Control and Prevention. Guam NVSS Mortality Data 2003 - NBER. Hyattsville, United States: National Center for Health Statistics, Centers for Disease Control and Prevention

National Center for Health Statistics, Centers for Disease Control and Prevention. Guam NVSS Mortality Data 2004 - NBER. Hyattsville, United States: National Center for Health Statistics, Centers for Disease Control and Prevention

National Center for Health Statistics, Centers for Disease Control and Prevention. Northern Mariana Islands NVSS Custom Mortality Data 2005. Hyattsville, United States: National Center for Health Statistics, Centers for Disease Control and Prevention

National Center for Health Statistics, Centers for Disease Control and Prevention. Northern Mariana Islands NVSS Custom Mortality Data 2006. Hyattsville, United States: National Center for Health Statistics, Centers for Disease Control and Prevention

National Center for Health Statistics, Centers for Disease Control and Prevention. Northern Mariana Islands NVSS Custom Mortality Data 2007. Hyattsville, United States: National Center for Health Statistics, Centers for Disease Control and Prevention

National Center for Health Statistics, Centers for Disease Control and Prevention. Northern Mariana Islands NVSS Custom Mortality Data 2008. Hyattsville, United States: National Center for Health Statistics, Centers for Disease Control and Prevention

National Center for Health Statistics, Centers for Disease Control and Prevention. Northern Mariana Islands NVSS Custom Mortality Data 2009. Hyattsville, United States: National Center for Health Statistics, Centers for Disease Control and Prevention

National Center for Health Statistics, Centers for Disease Control and Prevention. Northern Mariana Islands NVSS Custom Mortality Data 2010. Hyattsville, United States: National Center for Health Statistics, Centers for Disease Control and Prevention

National Center for Health Statistics, Centers for Disease Control and Prevention. Northern Mariana Islands NVSS Custom Mortality Data 2011. Hyattsville, United States: National Center for Health Statistics, Centers for Disease Control and Prevention

National Center for Health Statistics, Centers for Disease Control and Prevention. Northern Mariana Islands NVSS Custom Mortality Data 2012. Hyattsville, United States: National Center for Health Statistics, Centers for Disease Control and Prevention

National Center for Health Statistics, Centers for Disease Control and Prevention. Northern Mariana Islands NVSS Custom Mortality Data 2013. Hyattsville, United States: National Center for Health Statistics, Centers for Disease Control and Prevention

National Center for Health Statistics, Centers for Disease Control and Prevention. Northern Mariana Islands NVSS Custom Mortality Data 2014. Hyattsville, United States: National Center for Health Statistics, Centers for Disease Control and Prevention

National Center for Health Statistics, Centers for Disease Control and Prevention. Northern Mariana Islands NVSS Custom Mortality Data 2015. Hyattsville, United States: National Center for Health Statistics, Centers for Disease Control and Prevention

National Center for Health Statistics, Centers for Disease Control and Prevention. Northern Mariana Islands NVSS Mortality Data 1998 - NBER. Hyattsville, United States: National Center for Health Statistics, Centers for Disease Control and Prevention

National Center for Health Statistics, Centers for Disease Control and Prevention. Northern Mariana Islands NVSS Mortality Data 1999 - NBER. Hyattsville, United States: National Center for Health Statistics, Centers for Disease Control and Prevention

National Center for Health Statistics, Centers for Disease Control and Prevention. Northern Mariana Islands NVSS Mortality Data 2000 - NBER. Hyattsville, United States: National Center for Health Statistics, Centers for Disease Control and Prevention

National Center for Health Statistics, Centers for Disease Control and Prevention. Northern Mariana Islands NVSS Mortality Data 2001 - NBER. Hyattsville, United States: National Center for Health Statistics, Centers for Disease Control and Prevention

National Center for Health Statistics, Centers for Disease Control and Prevention. Northern Mariana Islands NVSS Mortality Data 2002 - NBER. Hyattsville, United States: National Center for Health Statistics, Centers for Disease Control and Prevention

National Center for Health Statistics, Centers for Disease Control and Prevention. Northern Mariana Islands NVSS Mortality Data 2003 - NBER. Hyattsville, United States: National Center for Health Statistics, Centers for Disease Control and Prevention

National Center for Health Statistics, Centers for Disease Control and Prevention. Northern Mariana Islands NVSS Mortality Data 2004 - NBER. Hyattsville, United States: National Center for Health Statistics, Centers for Disease Control and Prevention

National Center for Health Statistics, Centers for Disease Control and Prevention. Puerto Rico NVSS Custom Mortality Data 2005. Hyattsville, United States: National Center for Health Statistics, Centers for Disease Control and Prevention

National Center for Health Statistics, Centers for Disease Control and Prevention. Puerto Rico NVSS Custom Mortality Data 2006. Hyattsville, United States: National Center for Health Statistics, Centers for Disease Control and Prevention

National Center for Health Statistics, Centers for Disease Control and Prevention. Puerto Rico NVSS Custom Mortality Data 2007. Hyattsville, United States: National Center for Health Statistics, Centers for Disease Control and Prevention
National Center for Health Statistics, Centers for Disease Control and Prevention. Puerto Rico NVSS Custom Mortality Data 2008. Hyattsville, United States: National Center for Health Statistics, Centers for Disease Control and Prevention
National Center for Health Statistics, Centers for Disease Control and Prevention. Puerto Rico NVSS Custom Mortality Data 2009. Hyattsville, United States: National Center for Health Statistics, Centers for Disease Control and Prevention
National Center for Health Statistics, Centers for Disease Control and Prevention. Puerto Rico NVSS Custom Mortality Data 2010. Hyattsville, United States: National Center for Health Statistics, Centers for Disease Control and Prevention
National Center for Health Statistics, Centers for Disease Control and Prevention. Puerto Rico NVSS Custom Mortality Data 2011. Hyattsville, United States: National Center for Health Statistics, Centers for Disease Control and Prevention
National Center for Health Statistics, Centers for Disease Control and Prevention. Puerto Rico NVSS Custom Mortality Data 2012. Hyattsville, United States: National Center for Health Statistics, Centers for Disease Control and Prevention
National Center for Health Statistics, Centers for Disease Control and Prevention. Puerto Rico NVSS Custom Mortality Data 2013. Hyattsville, United States: National Center for Health Statistics, Centers for Disease Control and Prevention
National Center for Health Statistics, Centers for Disease Control and Prevention. Puerto Rico NVSS Custom Mortality Data 2014. Hyattsville, United States: National Center for Health Statistics, Centers for Disease Control and Prevention
National Center for Health Statistics, Centers for Disease Control and Prevention. Puerto Rico NVSS Custom Mortality Data 2015. Hyattsville, United States: National Center for Health Statistics, Centers for Disease Control and Prevention
National Center for Health Statistics, Centers for Disease Control and Prevention. Puerto Rico NVSS Mortality Data 1994 - NBER. Hyattsville, United States: National Center for Health Statistics, Centers for Disease Control and Prevention
National Center for Health Statistics, Centers for Disease Control and Prevention. Puerto Rico NVSS Mortality Data 1995 - NBER. Hyattsville, United States: National Center for Health Statistics, Centers for Disease Control and Prevention
National Center for Health Statistics, Centers for Disease Control and Prevention. Puerto Rico NVSS Mortality Data 1996 - NBER. Hyattsville, United States: National Center for Health Statistics, Centers for Disease Control and Prevention
National Center for Health Statistics, Centers for Disease Control and Prevention. Puerto Rico NVSS Mortality Data 1997 - NBER. Hyattsville, United States: National Center for Health Statistics, Centers for Disease Control and Prevention
National Center for Health Statistics, Centers for Disease Control and Prevention. Puerto Rico NVSS Mortality Data 1998 - NBER. Hyattsville, United States: National Center for Health Statistics, Centers for Disease Control and Prevention
National Center for Health Statistics, Centers for Disease Control and Prevention. Puerto Rico NVSS Mortality Data 1999 - NBER. Hyattsville, United States: National Center for Health Statistics, Centers for Disease Control and Prevention
National Center for Health Statistics, Centers for Disease Control and Prevention. Puerto Rico NVSS Mortality Data 2000 - NBER. Hyattsville, United States: National Center for Health Statistics, Centers for Disease Control and Prevention
National Center for Health Statistics, Centers for Disease Control and Prevention. Puerto Rico NVSS Mortality Data 2001 - NBER. Hyattsville, United States: National Center for Health Statistics, Centers for Disease Control and Prevention
National Center for Health Statistics, Centers for Disease Control and Prevention. Puerto Rico NVSS Mortality Data 2002 - NBER. Hyattsville, United States: National Center for Health Statistics, Centers for Disease Control and Prevention

National Center for Health Statistics, Centers for Disease Control and Prevention. Puerto Rico NVSS Mortality Data 2003 - NBER. Hyattsville, United States: National Center for Health Statistics, Centers for Disease Control and Prevention
National Center for Health Statistics, Centers for Disease Control and Prevention. Puerto Rico NVSS Mortality Data 2004 - NBER. Hyattsville, United States: National Center for Health Statistics, Centers for Disease Control and Prevention
National Center for Health Statistics, Centers for Disease Control and Prevention. United States NVSS Custom Mortality Data 2015. Hyattsville, United States: National Center for Health Statistics, Centers for Disease Control and Prevention
National Center for Health Statistics, Centers for Disease Control and Prevention. United States Virgin Islands NVSS Custom Mortality Data 2005. Hyattsville, United States: National Center for Health Statistics, Centers for Disease Control and Prevention
National Center for Health Statistics, Centers for Disease Control and Prevention. United States Virgin Islands NVSS Custom Mortality Data 2006. Hyattsville, United States: National Center for Health Statistics, Centers for Disease Control and Prevention
National Center for Health Statistics, Centers for Disease Control and Prevention. United States Virgin Islands NVSS Custom Mortality Data 2007. Hyattsville, United States: National Center for Health Statistics, Centers for Disease Control and Prevention
National Center for Health Statistics, Centers for Disease Control and Prevention. United States Virgin Islands NVSS Custom Mortality Data 2008. Hyattsville, United States: National Center for Health Statistics, Centers for Disease Control and Prevention
National Center for Health Statistics, Centers for Disease Control and Prevention. United States Virgin Islands NVSS Custom Mortality Data 2009. Hyattsville, United States: National Center for Health Statistics, Centers for Disease Control and Prevention
National Center for Health Statistics, Centers for Disease Control and Prevention. United States Virgin Islands NVSS Custom Mortality Data 2010. Hyattsville, United States: National Center for Health Statistics, Centers for Disease Control and Prevention
National Center for Health Statistics, Centers for Disease Control and Prevention. United States Virgin Islands NVSS Custom Mortality Data 2011. Hyattsville, United States: National Center for Health Statistics, Centers for Disease Control and Prevention
National Center for Health Statistics, Centers for Disease Control and Prevention. United States Virgin Islands NVSS Custom Mortality Data 2012. Hyattsville, United States: National Center for Health Statistics, Centers for Disease Control and Prevention
National Center for Health Statistics, Centers for Disease Control and Prevention. United States Virgin Islands NVSS Custom Mortality Data 2015. Hyattsville, United States: National Center for Health Statistics, Centers for Disease Control and Prevention
National Center for Health Statistics, Centers for Disease Control and Prevention. United States Virgin Islands NVSS Mortality Data 1994 - NBER. Hyattsville, United States: National Center for Health Statistics, Centers for Disease Control and Prevention
National Center for Health Statistics, Centers for Disease Control and Prevention. United States Virgin Islands NVSS Mortality Data 1995 - NBER. Hyattsville, United States: National Center for Health Statistics, Centers for Disease Control and Prevention
National Center for Health Statistics, Centers for Disease Control and Prevention. United States Virgin Islands NVSS Mortality Data 1996 - NBER. Hyattsville, United States: National Center for Health Statistics, Centers for Disease Control and Prevention
National Center for Health Statistics, Centers for Disease Control and Prevention. United States Virgin Islands NVSS Mortality Data 1997 - NBER. Hyattsville, United States: National Center for Health Statistics, Centers for Disease Control and Prevention
National Center for Health Statistics, Centers for Disease Control and Prevention. United States Virgin Islands NVSS Mortality Data 1998 - NBER. Hyattsville, United States: National Center for Health Statistics, Centers for Disease Control and Prevention
National Center for Health Statistics, Centers for Disease Control and Prevention. United States Virgin Islands NVSS Mortality Data 1999 - NBER. Hyattsville, United States: National Center for Health Statistics, Centers for Disease Control and Prevention

National Center for Health Statistics, Centers for Disease Control and Prevention. United States Virgin Islands NVSS Mortality Data 2000 - NBER. Hyattsville, United States: National Center for Health Statistics, Centers for Disease Control and Prevention
National Center for Health Statistics, Centers for Disease Control and Prevention. United States Virgin Islands NVSS Mortality Data 2001 - NBER. Hyattsville, United States: National Center for Health Statistics, Centers for Disease Control and Prevention
National Center for Health Statistics, Centers for Disease Control and Prevention. United States Virgin Islands NVSS Mortality Data 2002 - NBER. Hyattsville, United States: National Center for Health Statistics, Centers for Disease Control and Prevention
National Center for Health Statistics, Centers for Disease Control and Prevention. United States Virgin Islands NVSS Mortality Data 2003 - NBER. Hyattsville, United States: National Center for Health Statistics, Centers for Disease Control and Prevention
National Center for Health Statistics, Centers for Disease Control and Prevention. United States Virgin Islands NVSS Mortality Data 2004 - NBER. Hyattsville, United States: National Center for Health Statistics, Centers for Disease Control and Prevention
National Institute of Health Research and Development (NIHRD), Ministry of Health (Indonesia). Indonesia Mortality Registration System Strengthening Project
National Institute of Statistics (Italy). Italy Vital Registration - Deaths 2015. Rome, Italy: National Institute of Statistics (Italy)
National Institute of Statistics and Geography (INEGI) (Mexico), Secretariat of Health (Mexico). Mexico Vital Registration - Deaths 1980
National Institute of Statistics and Geography (INEGI) (Mexico), Secretariat of Health (Mexico). Mexico Vital Registration - Deaths 1981
National Institute of Statistics and Geography (INEGI) (Mexico), Secretariat of Health (Mexico). Mexico Vital Registration - Deaths 1982
National Institute of Statistics and Geography (INEGI) (Mexico), Secretariat of Health (Mexico). Mexico Vital Registration - Deaths 1983
National Institute of Statistics and Geography (INEGI) (Mexico), Secretariat of Health (Mexico). Mexico Vital Registration - Deaths 1984
National Institute of Statistics and Geography (INEGI) (Mexico), Secretariat of Health (Mexico). Mexico Vital Registration - Deaths 1985
National Institute of Statistics and Geography (INEGI) (Mexico), Secretariat of Health (Mexico). Mexico Vital Registration - Deaths 1986
National Institute of Statistics and Geography (INEGI) (Mexico), Secretariat of Health (Mexico). Mexico Vital Registration - Deaths 1987
National Institute of Statistics and Geography (INEGI) (Mexico), Secretariat of Health (Mexico). Mexico Vital Registration - Deaths 1988
National Institute of Statistics and Geography (INEGI) (Mexico), Secretariat of Health (Mexico). Mexico Vital Registration - Deaths 1989
National Institute of Statistics and Geography (INEGI) (Mexico), Secretariat of Health (Mexico). Mexico Vital Registration - Deaths 1990
National Institute of Statistics and Geography (INEGI) (Mexico), Secretariat of Health (Mexico). Mexico Vital Registration - Deaths 1991
National Institute of Statistics and Geography (INEGI) (Mexico), Secretariat of Health (Mexico). Mexico Vital Registration - Deaths 1992
National Institute of Statistics and Geography (INEGI) (Mexico), Secretariat of Health (Mexico). Mexico Vital Registration - Deaths 1993
National Institute of Statistics and Geography (INEGI) (Mexico), Secretariat of Health (Mexico). Mexico Vital Registration - Deaths 1994
National Institute of Statistics and Geography (INEGI) (Mexico), Secretariat of Health (Mexico). Mexico Vital Registration - Deaths 1995
National Institute of Statistics and Geography (INEGI) (Mexico), Secretariat of Health (Mexico). Mexico Vital Registration - Deaths 1996
National Institute of Statistics and Geography (INEGI) (Mexico), Secretariat of Health (Mexico). Mexico Vital Registration - Deaths 1997
National Institute of Statistics and Geography (INEGI) (Mexico), Secretariat of Health (Mexico). Mexico Vital Registration - Deaths 1998
National Institute of Statistics and Geography (INEGI) (Mexico), Secretariat of Health (Mexico). Mexico Vital Registration - Deaths 1999
National Institute of Statistics and Geography (INEGI) (Mexico), Secretariat of Health (Mexico). Mexico Vital Registration - Deaths 2000
National Institute of Statistics and Geography (INEGI) (Mexico), Secretariat of Health (Mexico). Mexico Vital Registration - Deaths 2001

National Institute of Statistics and Geography (INEGI) (Mexico), Secretariat of Health (Mexico). Mexico Vital Registration - Deaths 2002

National Institute of Statistics and Geography (INEGI) (Mexico), Secretariat of Health (Mexico). Mexico Vital Registration - Deaths 2003

National Institute of Statistics and Geography (INEGI) (Mexico), Secretariat of Health (Mexico). Mexico Vital Registration - Deaths 2004

National Institute of Statistics and Geography (INEGI) (Mexico), Secretariat of Health (Mexico). Mexico Vital Registration - Deaths 2005

National Institute of Statistics and Geography (INEGI) (Mexico), Secretariat of Health (Mexico). Mexico Vital Registration - Deaths 2006

National Institute of Statistics and Geography (INEGI) (Mexico), Secretariat of Health (Mexico). Mexico Vital Registration - Deaths 2007

National Institute of Statistics and Geography (INEGI) (Mexico), Secretariat of Health (Mexico). Mexico Vital Registration - Deaths 2008

National Institute of Statistics and Geography (INEGI) (Mexico), Secretariat of Health (Mexico). Mexico Vital Registration - Deaths 2010

National Institute of Statistics and Geography (INEGI) (Mexico), Secretariat of Health (Mexico). Mexico Vital Registration - Deaths 2011

National Institute of Statistics and Geography (INEGI) (Mexico). Mexico Vital Registration - Deaths 2012

National Institute of Statistics and Geography (INEGI) (Mexico). Mexico Vital Registration - Deaths 2013

National Institute of Statistics and Geography (INEGI) (Mexico). Mexico Vital Registration - Deaths 2014. Mexico City, Mexico: National Institute of Statistics and Geography (INEGI) (Mexico)

National Institute of Statistics and Geography (INEGI) (Mexico). Mexico Vital Registration - Deaths 2015. Mexico City, Mexico: National Institute of Statistics and Geography (INEGI) (Mexico)

National Institute of Statistics and Geography (INEGI) (Mexico). Mexico Vital Registration - Deaths 2016. Mexico City, Mexico: National Institute of Statistics and Geography (INEGI) (Mexico)

National Institute of Statistics and Geography (INEGI) (Mexico). Mexico Vital Statistics - Deaths 2009

National Records of Scotland. United Kingdom - Scotland Vital Events Reference Tables 2015. Edinburgh, Scotland: National Records of Scotland, 2016

National Records of Scotland. United Kingdom - Scotland Vital Events Reference Tables 2016. Edinburgh, Scotland: National Records of Scotland, 2017

National Statistics Office (Philippines). Philippines Vital Registration - Deaths 2012

Navrongo Health Research Centre. Ghana - Navrongo Health and Demographic Surveillance System

Netherlands Vital Registration - Deaths 1980 ICD9 as it appears in World Health Organization (WHO). WHO Mortality Database Version November 2015. Geneva, Switzerland: World Health Organization (WHO), 2015

Netherlands Vital Registration - Deaths 1981 ICD9 as it appears in World Health Organization (WHO). WHO Mortality Database Version November 2015. Geneva, Switzerland: World Health Organization (WHO), 2015

Netherlands Vital Registration - Deaths 1982 ICD9 as it appears in World Health Organization (WHO). WHO Mortality Database Version November 2015. Geneva, Switzerland: World Health Organization (WHO), 2015

Netherlands Vital Registration - Deaths 1983 ICD9 as it appears in World Health Organization (WHO). WHO Mortality Database Version November 2015. Geneva, Switzerland: World Health Organization (WHO), 2015

Netherlands Vital Registration - Deaths 1984 ICD9 as it appears in World Health Organization (WHO). WHO Mortality Database Version November 2015. Geneva, Switzerland: World Health Organization (WHO), 2015

Netherlands Vital Registration - Deaths 1986 ICD9 as it appears in World Health Organization (WHO). WHO Mortality Database Version November 2015. Geneva, Switzerland: World Health Organization (WHO), 2015

Netherlands Vital Registration - Deaths 1987 ICD9 as it appears in World Health Organization (WHO). WHO Mortality Database Version November 2015. Geneva, Switzerland: World Health Organization (WHO), 2015
Netherlands Vital Registration - Deaths 1989 ICD9 as it appears in World Health Organization (WHO). WHO Mortality Database Version November 2015. Geneva, Switzerland: World Health Organization (WHO), 2015
Netherlands Vital Registration - Deaths 1990 ICD9 as it appears in World Health Organization (WHO). WHO Mortality Database Version November 2015. Geneva, Switzerland: World Health Organization (WHO), 2015
Netherlands Vital Registration - Deaths 1991 ICD9 as it appears in World Health Organization (WHO). WHO Mortality Database Version November 2015. Geneva, Switzerland: World Health Organization (WHO), 2015
Netherlands Vital Registration - Deaths 1992 ICD9 as it appears in World Health Organization (WHO). WHO Mortality Database Version November 2015. Geneva, Switzerland: World Health Organization (WHO), 2015
Netherlands Vital Registration - Deaths 1993 ICD9 as it appears in World Health Organization (WHO). WHO Mortality Database Version November 2015. Geneva, Switzerland: World Health Organization (WHO), 2015
Netherlands Vital Registration - Deaths 1994 ICD9 as it appears in World Health Organization (WHO). WHO Mortality Database Version November 2015. Geneva, Switzerland: World Health Organization (WHO), 2015
Netherlands Vital Registration - Deaths 1995 ICD9 as it appears in World Health Organization (WHO). WHO Mortality Database Version November 2015. Geneva, Switzerland: World Health Organization (WHO), 2015
Netherlands Vital Registration - Deaths 1996 ICD10 as it appears in World Health Organization (WHO). WHO Mortality Database Version October 2017. Geneva, Switzerland: World Health Organization (WHO), 2017
Netherlands Vital Registration - Deaths 1997 ICD10 as it appears in World Health Organization (WHO). WHO Mortality Database Version October 2017. Geneva, Switzerland: World Health Organization (WHO), 2017
Netherlands Vital Registration - Deaths 1998 ICD10 as it appears in World Health Organization (WHO). WHO Mortality Database Version October 2017. Geneva, Switzerland: World Health Organization (WHO), 2017
Netherlands Vital Registration - Deaths 1999 ICD10 as it appears in World Health Organization (WHO). WHO Mortality Database Version October 2017. Geneva, Switzerland: World Health Organization (WHO), 2017
Netherlands Vital Registration - Deaths 2000 ICD10 as it appears in World Health Organization (WHO). WHO Mortality Database Version October 2017. Geneva, Switzerland: World Health Organization (WHO), 2017
Netherlands Vital Registration - Deaths 2001 ICD10 as it appears in World Health Organization (WHO). WHO Mortality Database Version October 2017. Geneva, Switzerland: World Health Organization (WHO), 2017
Netherlands Vital Registration - Deaths 2002 ICD10 as it appears in World Health Organization (WHO). WHO Mortality Database Version October 2017. Geneva, Switzerland: World Health Organization (WHO), 2017
Netherlands Vital Registration - Deaths 2003 ICD10 as it appears in World Health Organization (WHO). WHO Mortality Database Version October 2017. Geneva, Switzerland: World Health Organization (WHO), 2017
Netherlands Vital Registration - Deaths 2004 ICD10 as it appears in World Health Organization (WHO). WHO Mortality Database Version October 2017. Geneva, Switzerland: World Health Organization (WHO), 2017
Netherlands Vital Registration - Deaths 2005 ICD10 as it appears in World Health Organization (WHO). WHO Mortality Database Version October 2017. Geneva, Switzerland: World Health Organization (WHO), 2017

Netherlands Vital Registration - Deaths 2006 ICD10 as it appears in World Health Organization (WHO). WHO Mortality Database Version October 2017. Geneva, Switzerland: World Health Organization (WHO), 2017

Netherlands Vital Registration - Deaths 2007 ICD10 as it appears in World Health Organization (WHO). WHO Mortality Database Version October 2017. Geneva, Switzerland: World Health Organization (WHO), 2017

Netherlands Vital Registration - Deaths 2008 ICD10 as it appears in World Health Organization (WHO). WHO Mortality Database Version October 2017. Geneva, Switzerland: World Health Organization (WHO), 2017

Netherlands Vital Registration - Deaths 1985 ICD9 as it appears in World Health Organization (WHO). WHO Mortality Database Version November 2015. Geneva, Switzerland: World Health Organization (WHO), 2015

Netherlands Vital Registration - Deaths 1988 ICD9 as it appears in World Health Organization (WHO). WHO Mortality Database Version November 2015. Geneva, Switzerland: World Health Organization (WHO), 2015

Netherlands Vital Registration - Deaths 2009 ICD10 as it appears in World Health Organization (WHO). WHO Mortality Database Version October 2017. Geneva, Switzerland: World Health Organization (WHO), 2017

Netherlands Vital Registration - Deaths 2010 ICD10 as it appears in World Health Organization (WHO). WHO Mortality Database Version October 2017. Geneva, Switzerland: World Health Organization (WHO), 2017

Netherlands Vital Registration - Deaths 2011 ICD10 as it appears in World Health Organization (WHO). WHO Mortality Database Version October 2017. Geneva, Switzerland: World Health Organization (WHO), 2017

Netherlands Vital Registration - Deaths 2012 ICD10 as it appears in World Health Organization (WHO). WHO Mortality Database Version October 2017. Geneva, Switzerland: World Health Organization (WHO), 2017

Netherlands Vital Registration - Deaths 2013 ICD10 as it appears in World Health Organization (WHO). WHO Mortality Database Version October 2017. Geneva, Switzerland: World Health Organization (WHO), 2017

Netherlands Vital Registration - Deaths 2014 ICD10 as it appears in World Health Organization (WHO). WHO Mortality Database Version March 2017. Geneva, Switzerland: World Health Organization (WHO), 2017

Netherlands Vital Registration - Deaths 2015 ICD10 as it appears in World Health Organization (WHO). WHO Mortality Database Version March 2017. Geneva, Switzerland: World Health Organization (WHO), 2017

Netherlands Vital Registration - Deaths 2016 ICD10 as it appears in World Health Organization (WHO). WHO Mortality Database Version April 2018. Geneva, Switzerland: World Health Organization (WHO), 2018

Nga NT, Hoa DTP, Målqvist M, Persson L-Å, Ewald U. Causes of neonatal death: results from NeoKIP community-based trial in Quang Ninh province, Vietnam. Acta Paediatr. 2012; 101(4): 368-73

Ngo AD, Rao C, Hoa NP, Adair T, Chuc NTK. Mortality patterns in Vietnam, 2006: Findings from a national verbal autopsy survey. BMC Res Notes. 2010; 3: 78

Nicaragua Vital Registration - Deaths 1988 ICD9 as it appears in World Health Organization (WHO). WHO Mortality Database Version September 2016. Geneva, Switzerland: World Health Organization (WHO), 2016

Nicaragua Vital Registration - Deaths 1989 ICD9 as it appears in World Health Organization (WHO). WHO Mortality Database Version September 2016. Geneva, Switzerland: World Health Organization (WHO), 2016

Nicaragua Vital Registration - Deaths 1990 ICD9 as it appears in World Health Organization (WHO). WHO Mortality Database Version September 2016. Geneva, Switzerland: World Health Organization (WHO), 2016

Nicaragua Vital Registration - Deaths 1991 ICD9 as it appears in World Health Organization (WHO). WHO Mortality Database Version September 2016. Geneva, Switzerland: World Health Organization (WHO), 2016
Nicaragua Vital Registration - Deaths 1992 ICD9 as it appears in World Health Organization (WHO). WHO Mortality Database Version September 2016. Geneva, Switzerland: World Health Organization (WHO), 2016
Nicaragua Vital Registration - Deaths 1993 ICD9 as it appears in World Health Organization (WHO). WHO Mortality Database Version September 2016. Geneva, Switzerland: World Health Organization (WHO), 2016
Nicaragua Vital Registration - Deaths 1994 ICD9 as it appears in World Health Organization (WHO). WHO Mortality Database Version September 2016. Geneva, Switzerland: World Health Organization (WHO), 2016
Nicaragua Vital Registration - Deaths 1996 ICD9 as it appears in World Health Organization (WHO). WHO Mortality Database Version September 2016. Geneva, Switzerland: World Health Organization (WHO), 2016
Nicaragua Vital Registration - Deaths 1997 ICD10 as it appears in World Health Organization (WHO). WHO Mortality Database Version October 2017. Geneva, Switzerland: World Health Organization (WHO), 2017
Nicaragua Vital Registration - Deaths 1998 ICD10 as it appears in World Health Organization (WHO). WHO Mortality Database Version October 2017. Geneva, Switzerland: World Health Organization (WHO), 2017
Nicaragua Vital Registration - Deaths 1999 ICD10 as it appears in World Health Organization (WHO). WHO Mortality Database Version October 2017. Geneva, Switzerland: World Health Organization (WHO), 2017
Nicaragua Vital Registration - Deaths 2000 ICD10 as it appears in World Health Organization (WHO). WHO Mortality Database Version October 2017. Geneva, Switzerland: World Health Organization (WHO), 2017
Nicaragua Vital Registration - Deaths 2001 ICD10 as it appears in World Health Organization (WHO). WHO Mortality Database Version October 2017. Geneva, Switzerland: World Health Organization (WHO), 2017
Nicaragua Vital Registration - Deaths 2002 ICD10 as it appears in World Health Organization (WHO). WHO Mortality Database Version October 2017. Geneva, Switzerland: World Health Organization (WHO), 2017
Nicaragua Vital Registration - Deaths 2003 ICD10 as it appears in World Health Organization (WHO). WHO Mortality Database Version October 2017. Geneva, Switzerland: World Health Organization (WHO), 2017
Nicaragua Vital Registration - Deaths 2004 ICD10 as it appears in World Health Organization (WHO). WHO Mortality Database Version October 2017. Geneva, Switzerland: World Health Organization (WHO), 2017
Nicaragua Vital Registration - Deaths 2005 ICD10 as it appears in World Health Organization (WHO). WHO Mortality Database Version October 2017. Geneva, Switzerland: World Health Organization (WHO), 2017
Nicaragua Vital Registration - Deaths 2006 ICD10 as it appears in World Health Organization (WHO). WHO Mortality Database Version October 2017. Geneva, Switzerland: World Health Organization (WHO), 2017
Nicaragua Vital Registration - Deaths 2007 ICD10 as it appears in World Health Organization (WHO). WHO Mortality Database Version October 2017. Geneva, Switzerland: World Health Organization (WHO), 2017
Nicaragua Vital Registration - Deaths 2008 ICD10 as it appears in World Health Organization (WHO). WHO Mortality Database Version October 2017. Geneva, Switzerland: World Health Organization (WHO), 2017
Nicaragua Vital Registration - Deaths 2009 ICD10 as it appears in World Health Organization (WHO). WHO Mortality Database Version October 2017. Geneva, Switzerland: World Health Organization (WHO), 2017

Nicaragua Vital Registration - Deaths 2010 ICD10 as it appears in World Health Organization (WHO). WHO Mortality Database Version October 2017. Geneva, Switzerland: World Health Organization (WHO), 2017

Nicaragua Vital Registration - Deaths 2011 ICD10 as it appears in World Health Organization (WHO). WHO Mortality Database Version October 2017. Geneva, Switzerland: World Health Organization (WHO), 2017

Nicaragua Vital Registration - Deaths 2012 ICD10 as it appears in World Health Organization (WHO). WHO Mortality Database Version October 2017. Geneva, Switzerland: World Health Organization (WHO), 2017

Nicaragua Vital Registration - Deaths 2013 ICD10 as it appears in World Health Organization (WHO). WHO Mortality Database Version October 2017. Geneva, Switzerland: World Health Organization (WHO), 2017

Nicaragua Vital Registration - Deaths 2014 ICD10 as it appears in World Health Organization (WHO). WHO Mortality Database Version October 2017. Geneva, Switzerland: World Health Organization (WHO), 2017

Nicaragua Vital Registration - Deaths 2015 ICD10 as it appears in World Health Organization (WHO). WHO Mortality Database Version October 2017. Geneva, Switzerland: World Health Organization (WHO), 2017

Nisar MI, Ilyas M, Naeem K, Fatima U, Jehan F. Cause of Death in under 5 Children in a Demographic Surveillance Site in Pakistan. Online J Public Health Inform. 2017; 9(1): e174

Norwegian Institute of Public Health. Norway Cause of Death Registry 1980. Oslo, Norway: Norwegian Institute of Public Health

Norwegian Institute of Public Health. Norway Cause of Death Registry 1981. Oslo, Norway: Norwegian Institute of Public Health

Norwegian Institute of Public Health. Norway Cause of Death Registry 1982. Oslo, Norway: Norwegian Institute of Public Health

Norwegian Institute of Public Health. Norway Cause of Death Registry 1983. Oslo, Norway: Norwegian Institute of Public Health

Norwegian Institute of Public Health. Norway Cause of Death Registry 1984. Oslo, Norway: Norwegian Institute of Public Health

Norwegian Institute of Public Health. Norway Cause of Death Registry 1985. Oslo, Norway: Norwegian Institute of Public Health

Norwegian Institute of Public Health. Norway Cause of Death Registry 1986. Oslo, Norway: Norwegian Institute of Public Health

Norwegian Institute of Public Health. Norway Cause of Death Registry 1987. Oslo, Norway: Norwegian Institute of Public Health

Norwegian Institute of Public Health. Norway Cause of Death Registry 1988. Oslo, Norway: Norwegian Institute of Public Health

Norwegian Institute of Public Health. Norway Cause of Death Registry 1989. Oslo, Norway: Norwegian Institute of Public Health

Norwegian Institute of Public Health. Norway Cause of Death Registry 1990. Oslo, Norway: Norwegian Institute of Public Health

Norwegian Institute of Public Health. Norway Cause of Death Registry 1991. Oslo, Norway: Norwegian Institute of Public Health

Norwegian Institute of Public Health. Norway Cause of Death Registry 1992. Oslo, Norway: Norwegian Institute of Public Health

Norwegian Institute of Public Health. Norway Cause of Death Registry 1993. Oslo, Norway: Norwegian Institute of Public Health

Norwegian Institute of Public Health. Norway Cause of Death Registry 1994. Oslo, Norway: Norwegian Institute of Public Health

Norwegian Institute of Public Health. Norway Cause of Death Registry 1995. Oslo, Norway: Norwegian Institute of Public Health

Norwegian Institute of Public Health. Norway Cause of Death Registry 1996. Oslo, Norway: Norwegian Institute of Public Health

Norwegian Institute of Public Health. Norway Cause of Death Registry 1997. Oslo, Norway: Norwegian Institute of Public Health

Norwegian Institute of Public Health. Norway Cause of Death Registry 1998. Oslo, Norway: Norwegian Institute of Public Health

Norwegian Institute of Public Health. Norway Cause of Death Registry 1999. Oslo, Norway: Norwegian Institute of Public Health

Norwegian Institute of Public Health. Norway Cause of Death Registry 2000. Oslo, Norway: Norwegian Institute of Public Health

Norwegian Institute of Public Health. Norway Cause of Death Registry 2001. Oslo, Norway: Norwegian Institute of Public Health
Norwegian Institute of Public Health. Norway Cause of Death Registry 2002. Oslo, Norway: Norwegian Institute of Public Health
Norwegian Institute of Public Health. Norway Cause of Death Registry 2003. Oslo, Norway: Norwegian Institute of Public Health
Norwegian Institute of Public Health. Norway Cause of Death Registry 2004. Oslo, Norway: Norwegian Institute of Public Health
Norwegian Institute of Public Health. Norway Cause of Death Registry 2005. Oslo, Norway: Norwegian Institute of Public Health
Norwegian Institute of Public Health. Norway Cause of Death Registry 2006. Oslo, Norway: Norwegian Institute of Public Health
Norwegian Institute of Public Health. Norway Cause of Death Registry 2007. Oslo, Norway: Norwegian Institute of Public Health
Norwegian Institute of Public Health. Norway Cause of Death Registry 2008. Oslo, Norway: Norwegian Institute of Public Health
Norwegian Institute of Public Health. Norway Cause of Death Registry 2009. Oslo, Norway: Norwegian Institute of Public Health
Norwegian Institute of Public Health. Norway Cause of Death Registry 2010. Oslo, Norway: Norwegian Institute of Public Health
Norwegian Institute of Public Health. Norway Cause of Death Registry 2011. Oslo, Norway: Norwegian Institute of Public Health
Norwegian Institute of Public Health. Norway Cause of Death Registry 2012. Oslo, Norway: Norwegian Institute of Public Health
Norwegian Institute of Public Health. Norway Cause of Death Registry 2013. Oslo, Norway: Norwegian Institute of Public Health
Norwegian Institute of Public Health. Norway Cause of Death Registry 2014. Oslo, Norway: Norwegian Institute of Public Health
Norwegian Institute of Public Health. Norway Cause of Death Registry 2015. Oslo, Norway: Norwegian Institute of Public Health
Office for National Statistics (ONS) (United Kingdom). United Kingdom - England and Wales Mortality Statistics 2014
Office for National Statistics (ONS) (United Kingdom). United Kingdom - England and Wales Mortality Statistics 2015
Office for National Statistics (ONS) (United Kingdom). United Kingdom - England and Wales Mortality Statistics 2016
Office for National Statistics (United Kingdom). United Kingdom - England and Wales Mortality Data 1981-1994
Office for National Statistics (United Kingdom). United Kingdom - England and Wales Mortality Data 1995-2000
Office for National Statistics (United Kingdom). United Kingdom - England and Wales Mortality Data 2001-2012
Office for National Statistics (United Kingdom). United Kingdom - England and Wales Mortality Statistics 2013
Office for National Statistics (United Kingdom). United Kingdom - England Mortality Statistics 1981
Office for National Statistics (United Kingdom). United Kingdom - England Mortality Statistics 1982
Office for National Statistics (United Kingdom). United Kingdom - England Mortality Statistics 1983
Office for National Statistics (United Kingdom). United Kingdom - England Mortality Statistics 1984
Office for National Statistics (United Kingdom). United Kingdom - England Mortality Statistics 1985
Office for National Statistics (United Kingdom). United Kingdom - England Mortality Statistics 1986
Office for National Statistics (United Kingdom). United Kingdom - England Mortality Statistics 1987
Office for National Statistics (United Kingdom). United Kingdom - England Mortality Statistics 1988
Office for National Statistics (United Kingdom). United Kingdom - England Mortality Statistics 1989
Office for National Statistics (United Kingdom). United Kingdom - England Mortality Statistics 1990
Office for National Statistics (United Kingdom). United Kingdom - England Mortality Statistics 1991
Office for National Statistics (United Kingdom). United Kingdom - England Mortality Statistics 1992
Office for National Statistics (United Kingdom). United Kingdom - England Mortality Statistics 1993

Office for National Statistics (United Kingdom). United Kingdom - England Mortality Statistics 1994
Office for National Statistics (United Kingdom). United Kingdom - England Mortality Statistics 1995
Office for National Statistics (United Kingdom). United Kingdom - England Mortality Statistics 1996
Office for National Statistics (United Kingdom). United Kingdom - England Mortality Statistics 1997
Office for National Statistics (United Kingdom). United Kingdom - England Mortality Statistics 1998
Office for National Statistics (United Kingdom). United Kingdom - England Mortality Statistics 1999
Office for National Statistics (United Kingdom). United Kingdom - England Mortality Statistics 2000
Office for National Statistics (United Kingdom). United Kingdom - England Mortality Statistics 2001
Office for National Statistics (United Kingdom). United Kingdom - England Mortality Statistics 2002
Office for National Statistics (United Kingdom). United Kingdom - England Mortality Statistics 2003
Office for National Statistics (United Kingdom). United Kingdom - England Mortality Statistics 2004
Office for National Statistics (United Kingdom). United Kingdom - England Mortality Statistics 2005
Office for National Statistics (United Kingdom). United Kingdom - England Mortality Statistics 2006
Office for National Statistics (United Kingdom). United Kingdom - England Mortality Statistics 2007
Office for National Statistics (United Kingdom). United Kingdom - England Mortality Statistics 2008
Office for National Statistics (United Kingdom). United Kingdom - England Mortality Statistics 2009
Office for National Statistics (United Kingdom). United Kingdom - England Mortality Statistics 2010
Office for National Statistics (United Kingdom). United Kingdom - England Mortality Statistics 2011
Office for National Statistics (United Kingdom). United Kingdom - England Mortality Statistics 2012
Office of the Registrar General and Census Commissioner (India). India Medical Certification of Cause of Death Report 2005. New Delhi, India: Office of the Registrar General and Census Commissioner (India)
Office of the Registrar General and Census Commissioner (India). India Medical Certification of Cause of Death Report 2006. New Delhi, India: Office of the Registrar General and Census Commissioner (India)
Office of the Registrar General and Census Commissioner (India). India Medical Certification of Cause of Death Report 2008. New Delhi, India: Office of the Registrar General and Census Commissioner (India)
Office of the Registrar General and Census Commissioner (India). India Medical Certification of Cause of Death Report 2009. New Delhi, India: Office of the Registrar General and Census Commissioner (India), 2014
Office of the Registrar General and Census Commissioner (India). India Medical Certification of Cause of Death Report 2010. New Delhi, India: Office of the Registrar General and Census Commissioner (India), 2014
Office of the Registrar General and Census Commissioner (India). India Medical Certification of Cause of Death Report 2012. New Delhi, India: Office of the Registrar General and Census Commissioner (India), 2015
Office of the Registrar General and Census Commissioner (India). India Medical Certification of Cause of Death State-Level Tabulations 1990
Office of the Registrar General and Census Commissioner (India). India Medical Certification of Cause of Death State-Level Tabulations 1993

Office of the Registrar General and Census Commissioner (India). India Medical Certification of Cause of Death State-Level Tabulations 1999
Office of the Registrar General and Census Commissioner (India). India Medical Certification of Cause of Death State-Level Tabulations 2000
Office of the Registrar General and Census Commissioner (India). India Medical Certification of Cause of Death State-Level Tabulations 2001
Office of the Registrar General and Census Commissioner (India). India Medical Certification of Cause of Death State-Level Tabulations 2002
Office of the Registrar General and Census Commissioner (India). India Medical Certification of Cause of Death State-Level Tabulations 2003
Office of the Registrar General and Census Commissioner (India). India Medical Certification of Cause of Death State-Level Tabulations 2004
Office of the Registrar General and Census Commissioner (India). India Vital Statistics 1980. New Delhi, India: Office of the Registrar General and Census Commissioner (India)
Office of the Registrar General and Census Commissioner (India). India Vital Statistics 1981. New Delhi, India: Office of the Registrar General and Census Commissioner (India)
Office of the Registrar General and Census Commissioner (India). India Vital Statistics 1989. New Delhi, India: Office of the Registrar General and Census Commissioner (India)
Oman Vital Registration - Deaths 2009 ICD10 as it appears in World Health Organization (WHO). WHO Mortality Database Version November 2015. Geneva, Switzerland: World Health Organization (WHO), 2015
Palestine - West Bank and Gaza Strip Vital Registration - Deaths 2010 ICD10 as it appears in World Health Organization (WHO). WHO Mortality Database Version October 2017. Geneva, Switzerland: World Health Organization (WHO), 2017
Palestine - West Bank and Gaza Strip Vital Registration - Deaths 2011 ICD10 as it appears in World Health Organization (WHO). WHO Mortality Database Version October 2017. Geneva, Switzerland: World Health Organization (WHO), 2017
Palestine Vital Registration - Deaths 2008 ICD10 as it appears in World Health Organization (WHO). WHO Mortality Database Version October 2017. Geneva, Switzerland: World Health Organization (WHO), 2017
Palestine Vital Registration - Deaths 2009 ICD10 as it appears in World Health Organization (WHO). WHO Mortality Database Version October 2017. Geneva, Switzerland: World Health Organization (WHO), 2017
Palestinian Central Bureau of Statistics. Palestine - West Bank Vital Registration - Deaths 2004
Palestinian Central Bureau of Statistics. Palestine - West Bank Vital Registration - Deaths 2005
Palestinian Central Bureau of Statistics. Palestine - West Bank Vital Registration - Deaths 2007
Panama Vital Registration - Deaths 1980 ICD9 as it appears in World Health Organization (WHO). WHO Mortality Database Version September 2016. Geneva, Switzerland: World Health Organization (WHO), 2016
Panama Vital Registration - Deaths 1981 ICD9 as it appears in World Health Organization (WHO). WHO Mortality Database Version September 2016. Geneva, Switzerland: World Health Organization (WHO), 2016

Panama Vital Registration - Deaths 1982 ICD9 as it appears in World Health Organization (WHO). WHO Mortality Database Version September 2016. Geneva, Switzerland: World Health Organization (WHO), 2016
Panama Vital Registration - Deaths 1983 ICD9 as it appears in World Health Organization (WHO). WHO Mortality Database Version September 2016. Geneva, Switzerland: World Health Organization (WHO), 2016
Panama Vital Registration - Deaths 1984 ICD9 as it appears in World Health Organization (WHO). WHO Mortality Database Version September 2016. Geneva, Switzerland: World Health Organization (WHO), 2016
Panama Vital Registration - Deaths 1985 ICD9 as it appears in World Health Organization (WHO). WHO Mortality Database Version September 2016. Geneva, Switzerland: World Health Organization (WHO), 2016
Panama Vital Registration - Deaths 1986 ICD9 as it appears in World Health Organization (WHO). WHO Mortality Database Version September 2016. Geneva, Switzerland: World Health Organization (WHO), 2016
Panama Vital Registration - Deaths 1987 ICD9 as it appears in World Health Organization (WHO). WHO Mortality Database Version September 2016. Geneva, Switzerland: World Health Organization (WHO), 2016
Panama Vital Registration - Deaths 1988 ICD9 as it appears in World Health Organization (WHO). WHO Mortality Database Version September 2016. Geneva, Switzerland: World Health Organization (WHO), 2016
Panama Vital Registration - Deaths 1989 ICD9 as it appears in World Health Organization (WHO). WHO Mortality Database Version September 2016. Geneva, Switzerland: World Health Organization (WHO), 2016
Panama Vital Registration - Deaths 1996 ICD9 as it appears in World Health Organization (WHO). WHO Mortality Database Version September 2016. Geneva, Switzerland: World Health Organization (WHO), 2016
Panama Vital Registration - Deaths 1997 ICD9 as it appears in World Health Organization (WHO). WHO Mortality Database Version September 2016. Geneva, Switzerland: World Health Organization (WHO), 2016
Panama Vital Registration - Deaths 1998 ICD10 as it appears in World Health Organization (WHO). WHO Mortality Database Version October 2017. Geneva, Switzerland: World Health Organization (WHO), 2017
Panama Vital Registration - Deaths 1999 ICD10 as it appears in World Health Organization (WHO). WHO Mortality Database Version October 2017. Geneva, Switzerland: World Health Organization (WHO), 2017
Panama Vital Registration - Deaths 2000 ICD10 as it appears in World Health Organization (WHO). WHO Mortality Database Version October 2017. Geneva, Switzerland: World Health Organization (WHO), 2017
Panama Vital Registration - Deaths 2001 ICD10 as it appears in World Health Organization (WHO). WHO Mortality Database Version October 2017. Geneva, Switzerland: World Health Organization (WHO), 2017
Panama Vital Registration - Deaths 2002 ICD10 as it appears in World Health Organization (WHO). WHO Mortality Database Version October 2017. Geneva, Switzerland: World Health Organization (WHO), 2017
Panama Vital Registration - Deaths 2003 ICD10 as it appears in World Health Organization (WHO). WHO Mortality Database Version October 2017. Geneva, Switzerland: World Health Organization (WHO), 2017
Panama Vital Registration - Deaths 2004 ICD10 as it appears in World Health Organization (WHO). WHO Mortality Database Version October 2017. Geneva, Switzerland: World Health Organization (WHO), 2017
Panama Vital Registration - Deaths 2006 ICD10 as it appears in World Health Organization (WHO). WHO Mortality Database Version October 2017. Geneva, Switzerland: World Health Organization (WHO), 2017

Panama Vital Registration - Deaths 2005 ICD10 as it appears in World Health Organization (WHO). WHO Mortality Database Version February 2014. Geneva, Switzerland: World Health Organization (WHO), 2014
Panama Vital Registration - Deaths 2007 ICD10 as it appears in World Health Organization (WHO). WHO Mortality Database Version October 2017. Geneva, Switzerland: World Health Organization (WHO), 2017
Panama Vital Registration - Deaths 2008 ICD10 as it appears in World Health Organization (WHO). WHO Mortality Database Version October 2017. Geneva, Switzerland: World Health Organization (WHO), 2017
Panama Vital Registration - Deaths 2009 ICD10 as it appears in World Health Organization (WHO). WHO Mortality Database Version October 2017. Geneva, Switzerland: World Health Organization (WHO), 2017
Panama Vital Registration - Deaths 2010 ICD10 as it appears in World Health Organization (WHO). WHO Mortality Database Version October 2017. Geneva, Switzerland: World Health Organization (WHO), 2017
Panama Vital Registration - Deaths 2011 ICD10 as it appears in World Health Organization (WHO). WHO Mortality Database Version October 2017. Geneva, Switzerland: World Health Organization (WHO), 2017
Panama Vital Registration - Deaths 2012 ICD10 as it appears in World Health Organization (WHO). WHO Mortality Database Version October 2017. Geneva, Switzerland: World Health Organization (WHO), 2017
Panama Vital Registration - Deaths 2013 ICD10 as it appears in World Health Organization (WHO). WHO Mortality Database Version October 2017. Geneva, Switzerland: World Health Organization (WHO), 2017
Panama Vital Registration - Deaths 2014 ICD10 as it appears in World Health Organization (WHO). WHO Mortality Database Version October 2017. Geneva, Switzerland: World Health Organization (WHO), 2017
Panama Vital Registration - Deaths 2015 ICD10 as it appears in World Health Organization (WHO). WHO Mortality Database Version October 2017. Geneva, Switzerland: World Health Organization (WHO), 2017
Paraguay Vital Registration - Deaths 1980 ICD9 as it appears in World Health Organization (WHO). WHO Mortality Database Version September 2016. Geneva, Switzerland: World Health Organization (WHO), 2016
Paraguay Vital Registration - Deaths 1981 ICD9 as it appears in World Health Organization (WHO). WHO Mortality Database Version September 2016. Geneva, Switzerland: World Health Organization (WHO), 2016
Paraguay Vital Registration - Deaths 1982 ICD9 as it appears in World Health Organization (WHO). WHO Mortality Database Version September 2016. Geneva, Switzerland: World Health Organization (WHO), 2016
Paraguay Vital Registration - Deaths 1983 ICD9 as it appears in World Health Organization (WHO). WHO Mortality Database Version September 2016. Geneva, Switzerland: World Health Organization (WHO), 2016
Paraguay Vital Registration - Deaths 1984 ICD9 as it appears in World Health Organization (WHO). WHO Mortality Database Version September 2016. Geneva, Switzerland: World Health Organization (WHO), 2016
Paraguay Vital Registration - Deaths 1985 ICD9 as it appears in World Health Organization (WHO). WHO Mortality Database Version September 2016. Geneva, Switzerland: World Health Organization (WHO), 2016
Paraguay Vital Registration - Deaths 1986 ICD9 as it appears in World Health Organization (WHO). WHO Mortality Database Version September 2016. Geneva, Switzerland: World Health Organization (WHO), 2016
Paraguay Vital Registration - Deaths 1987 ICD9 as it appears in World Health Organization (WHO). WHO Mortality Database Version September 2016. Geneva, Switzerland: World Health Organization (WHO), 2016

Paraguay Vital Registration - Deaths 1988 ICD9 as it appears in World Health Organization (WHO). WHO Mortality Database Version September 2016. Geneva, Switzerland: World Health Organization (WHO), 2016
Paraguay Vital Registration - Deaths 1989 ICD9 as it appears in World Health Organization (WHO). WHO Mortality Database Version September 2016. Geneva, Switzerland: World Health Organization (WHO), 2016
Paraguay Vital Registration - Deaths 1990 ICD9 as it appears in World Health Organization (WHO). WHO Mortality Database Version September 2016. Geneva, Switzerland: World Health Organization (WHO), 2016
Paraguay Vital Registration - Deaths 1991 ICD9 as it appears in World Health Organization (WHO). WHO Mortality Database Version September 2016. Geneva, Switzerland: World Health Organization (WHO), 2016
Paraguay Vital Registration - Deaths 1994 ICD9 as it appears in World Health Organization (WHO). WHO Mortality Database Version September 2016. Geneva, Switzerland: World Health Organization (WHO), 2016
Paraguay Vital Registration - Deaths 1995 ICD9 as it appears in World Health Organization (WHO). WHO Mortality Database Version September 2016. Geneva, Switzerland: World Health Organization (WHO), 2016
Paraguay Vital Registration - Deaths 1996 ICD10 as it appears in World Health Organization (WHO). WHO Mortality Database Version October 2017. Geneva, Switzerland: World Health Organization (WHO), 2017
Paraguay Vital Registration - Deaths 1997 ICD10 as it appears in World Health Organization (WHO). WHO Mortality Database Version October 2017. Geneva, Switzerland: World Health Organization (WHO), 2017
Paraguay Vital Registration - Deaths 1998 ICD10 as it appears in World Health Organization (WHO). WHO Mortality Database Version October 2017. Geneva, Switzerland: World Health Organization (WHO), 2017
Paraguay Vital Registration - Deaths 1999 ICD10 as it appears in World Health Organization (WHO). WHO Mortality Database Version October 2017. Geneva, Switzerland: World Health Organization (WHO), 2017
Paraguay Vital Registration - Deaths 2000 ICD10 as it appears in World Health Organization (WHO). WHO Mortality Database Version October 2017. Geneva, Switzerland: World Health Organization (WHO), 2017
Paraguay Vital Registration - Deaths 2001 ICD10 as it appears in World Health Organization (WHO). WHO Mortality Database Version October 2017. Geneva, Switzerland: World Health Organization (WHO), 2017
Paraguay Vital Registration - Deaths 2002 ICD10 as it appears in World Health Organization (WHO). WHO Mortality Database Version October 2017. Geneva, Switzerland: World Health Organization (WHO), 2017
Paraguay Vital Registration - Deaths 2003 ICD10 as it appears in World Health Organization (WHO). WHO Mortality Database Version October 2017. Geneva, Switzerland: World Health Organization (WHO), 2017
Paraguay Vital Registration - Deaths 2004 ICD10 as it appears in World Health Organization (WHO). WHO Mortality Database Version October 2017. Geneva, Switzerland: World Health Organization (WHO), 2017
Paraguay Vital Registration - Deaths 2005 ICD10 as it appears in World Health Organization (WHO). WHO Mortality Database Version October 2017. Geneva, Switzerland: World Health Organization (WHO), 2017
Paraguay Vital Registration - Deaths 2006 ICD10 as it appears in World Health Organization (WHO). WHO Mortality Database Version October 2017. Geneva, Switzerland: World Health Organization (WHO), 2017
Paraguay Vital Registration - Deaths 2007 ICD10 as it appears in World Health Organization (WHO). WHO Mortality Database Version October 2017. Geneva, Switzerland: World Health Organization (WHO), 2017

Paraguay Vital Registration - Deaths 2008 ICD10 as it appears in World Health Organization (WHO). WHO Mortality Database Version October 2017. Geneva, Switzerland: World Health Organization (WHO), 2017

Paraguay Vital Registration - Deaths 2009 ICD10 as it appears in World Health Organization (WHO). WHO Mortality Database Version October 2017. Geneva, Switzerland: World Health Organization (WHO), 2017

Paraguay Vital Registration - Deaths 2010 ICD10 as it appears in World Health Organization (WHO). WHO Mortality Database Version October 2017. Geneva, Switzerland: World Health Organization (WHO), 2017

Paraguay Vital Registration - Deaths 2011 ICD10 as it appears in World Health Organization (WHO). WHO Mortality Database Version October 2017. Geneva, Switzerland: World Health Organization (WHO), 2017

Paraguay Vital Registration - Deaths 2012 ICD10 as it appears in World Health Organization (WHO). WHO Mortality Database Version October 2017. Geneva, Switzerland: World Health Organization (WHO), 2017

Paraguay Vital Registration - Deaths 2013 ICD10 as it appears in World Health Organization (WHO). WHO Mortality Database Version October 2017. Geneva, Switzerland: World Health Organization (WHO), 2017

Paraguay Vital Registration - Deaths 2014 ICD10 as it appears in World Health Organization (WHO). WHO Mortality Database Version October 2017. Geneva, Switzerland: World Health Organization (WHO), 2017

Pérez W, Eriksson L, Blandón EZ, Persson L-Å, Källestål C, Peña R. Comparing progress toward the millennium development goal for under-five mortality in León and Cuatro Santos, Nicaragua, 1990-2008. BMC Pediatr. 2014; 14(1): 9

Perry HB, Ross AG, Fernand F. Assessing the causes of under-five mortality in the Albert Schweitzer Hospital service area of rural Haiti. Rev Panam Salud Publica. 2005; 18(3): 178-86

Peru Vital Registration - Deaths 1980 ICD9 as it appears in World Health Organization (WHO). WHO Mortality Database Version September 2016. Geneva, Switzerland: World Health Organization (WHO), 2016

Peru Vital Registration - Deaths 1981 ICD9 as it appears in World Health Organization (WHO). WHO Mortality Database Version September 2016. Geneva, Switzerland: World Health Organization (WHO), 2016

Peru Vital Registration - Deaths 1982 ICD9 as it appears in World Health Organization (WHO). WHO Mortality Database Version September 2016. Geneva, Switzerland: World Health Organization (WHO), 2016

Peru Vital Registration - Deaths 1983 ICD9 as it appears in World Health Organization (WHO). WHO Mortality Database Version September 2016. Geneva, Switzerland: World Health Organization (WHO), 2016

Peru Vital Registration - Deaths 1986 ICD9 as it appears in World Health Organization (WHO). WHO Mortality Database Version September 2016. Geneva, Switzerland: World Health Organization (WHO), 2016

Peru Vital Registration - Deaths 1988 ICD9 as it appears in World Health Organization (WHO). WHO Mortality Database Version September 2016. Geneva, Switzerland: World Health Organization (WHO), 2016

Peru Vital Registration - Deaths 1989 ICD9 as it appears in World Health Organization (WHO). WHO Mortality Database Version September 2016. Geneva, Switzerland: World Health Organization (WHO), 2016

Peru Vital Registration - Deaths 1990 ICD9 as it appears in World Health Organization (WHO). WHO Mortality Database Version September 2016. Geneva, Switzerland: World Health Organization (WHO), 2016

Peru Vital Registration - Deaths 1992 ICD9 as it appears in World Health Organization (WHO). WHO Mortality Database Version September 2016. Geneva, Switzerland: World Health Organization (WHO), 2016

Peru Vital Registration - Deaths 1994 ICD9 as it appears in World Health Organization (WHO). WHO Mortality Database Version September 2016. Geneva, Switzerland: World Health Organization (WHO), 2016

Peru Vital Registration - Deaths 1995 ICD9 as it appears in World Health Organization (WHO). WHO Mortality Database Version September 2016. Geneva, Switzerland: World Health Organization (WHO), 2016

Peru Vital Registration - Deaths 1996 ICD9 as it appears in World Health Organization (WHO). WHO Mortality Database Version September 2016. Geneva, Switzerland: World Health Organization (WHO), 2016

Peru Vital Registration - Deaths 1997 ICD9 as it appears in World Health Organization (WHO). WHO Mortality Database Version September 2016. Geneva, Switzerland: World Health Organization (WHO), 2016

Peru Vital Registration - Deaths 1998 ICD9 as it appears in World Health Organization (WHO). WHO Mortality Database Version September 2016. Geneva, Switzerland: World Health Organization (WHO), 2016

Peru Vital Registration - Deaths 1999 ICD10 as it appears in World Health Organization (WHO). WHO Mortality Database Version October 2017. Geneva, Switzerland: World Health Organization (WHO), 2017

Peru Vital Registration - Deaths 2000 ICD10 as it appears in World Health Organization (WHO). WHO Mortality Database Version October 2017. Geneva, Switzerland: World Health Organization (WHO), 2017

Peru Vital Registration - Deaths 2007 ICD10 as it appears in World Health Organization (WHO). WHO Mortality Database Version October 2017. Geneva, Switzerland: World Health Organization (WHO), 2017

Peru Vital Registration - Deaths 2008 ICD10 as it appears in World Health Organization (WHO). WHO Mortality Database Version October 2017. Geneva, Switzerland: World Health Organization (WHO), 2017

Peru Vital Registration - Deaths 2009 ICD10 as it appears in World Health Organization (WHO). WHO Mortality Database Version October 2017. Geneva, Switzerland: World Health Organization (WHO), 2017

Peru Vital Registration - Deaths 2010 ICD10 as it appears in World Health Organization (WHO). WHO Mortality Database Version October 2017. Geneva, Switzerland: World Health Organization (WHO), 2017

Peru Vital Registration - Deaths 2011 ICD10 as it appears in World Health Organization (WHO). WHO Mortality Database Version October 2017. Geneva, Switzerland: World Health Organization (WHO), 2017

Peru Vital Registration - Deaths 2012 ICD10 as it appears in World Health Organization (WHO). WHO Mortality Database Version October 2017. Geneva, Switzerland: World Health Organization (WHO), 2017

Peru Vital Registration - Deaths 2013 ICD10 as it appears in World Health Organization (WHO). WHO Mortality Database Version October 2017. Geneva, Switzerland: World Health Organization (WHO), 2017

Peru Vital Registration - Deaths 2014 ICD10 as it appears in World Health Organization (WHO). WHO Mortality Database Version October 2017. Geneva, Switzerland: World Health Organization (WHO), 2017

Peru Vital Registration - Deaths 2015 ICD10 as it appears in World Health Organization (WHO). WHO Mortality Database Version October 2017. Geneva, Switzerland: World Health Organization (WHO), 2017

Philippines Statistics Authority. Philippines Vital Registration - Deaths 2013

Philippines Statistics Authority. Philippines Vital Registration - Deaths 2014

Philippines Statistics Authority. Philippines Vital Registration - Deaths 2015

Philippines Vital Registration - Deaths 1981 ICD8 as it appears in World Health Organization (WHO). WHO Mortality Database Version July 2012. Geneva, Switzerland: World Health Organization (WHO), 2012
Philippines Vital Registration - Deaths 1992 ICD9 as it appears in World Health Organization (WHO). WHO Mortality Database Version September 2016. Geneva, Switzerland: World Health Organization (WHO), 2016
Philippines Vital Registration - Deaths 1993 ICD9 as it appears in World Health Organization (WHO). WHO Mortality Database Version September 2016. Geneva, Switzerland: World Health Organization (WHO), 2016
Philippines Vital Registration - Deaths 1994 ICD9 as it appears in World Health Organization (WHO). WHO Mortality Database Version September 2016. Geneva, Switzerland: World Health Organization (WHO), 2016
Philippines Vital Registration - Deaths 1995 ICD9 as it appears in World Health Organization (WHO). WHO Mortality Database Version September 2016. Geneva, Switzerland: World Health Organization (WHO), 2016
Philippines Vital Registration - Deaths 1996 ICD9 as it appears in World Health Organization (WHO). WHO Mortality Database Version September 2016. Geneva, Switzerland: World Health Organization (WHO), 2016
Philippines Vital Registration - Deaths 1999 ICD10 as it appears in World Health Organization (WHO). WHO Mortality Database Version October 2017. Geneva, Switzerland: World Health Organization (WHO), 2017
Philippines Vital Registration - Deaths 2000 ICD10 as it appears in World Health Organization (WHO). WHO Mortality Database Version October 2017. Geneva, Switzerland: World Health Organization (WHO), 2017
Philippines Vital Registration - Deaths 2001 ICD10 as it appears in World Health Organization (WHO). WHO Mortality Database Version October 2017. Geneva, Switzerland: World Health Organization (WHO), 2017
Philippines Vital Registration - Deaths 2002 ICD10 as it appears in World Health Organization (WHO). WHO Mortality Database Version October 2017. Geneva, Switzerland: World Health Organization (WHO), 2017
Philippines Vital Registration - Deaths 2003 ICD10 as it appears in World Health Organization (WHO). WHO Mortality Database Version October 2017. Geneva, Switzerland: World Health Organization (WHO), 2017
Philippines Vital Registration - Deaths 1997 ICD9 as it appears in World Health Organization (WHO). WHO Mortality Database Version November 2015. Geneva, Switzerland: World Health Organization (WHO), 2015
Philippines Vital Registration - Deaths 1998 ICD9 as it appears in World Health Organization (WHO). WHO Mortality Database Version November 2015. Geneva, Switzerland: World Health Organization (WHO), 2015
Philippines Vital Registration - Deaths 2006 ICD10 as it appears in World Health Organization (WHO). WHO Mortality Database Version October 2017. Geneva, Switzerland: World Health Organization (WHO), 2017
Philippines Vital Registration - Deaths 2007 ICD10 as it appears in World Health Organization (WHO). WHO Mortality Database Version October 2017. Geneva, Switzerland: World Health Organization (WHO), 2017
Philippines Vital Registration - Deaths 2008 ICD10 as it appears in World Health Organization (WHO). WHO Mortality Database Version October 2017. Geneva, Switzerland: World Health Organization (WHO), 2017
Philippines Vital Registration - Deaths 2009 ICD10 as it appears in World Health Organization (WHO). WHO Mortality Database Version October 2017. Geneva, Switzerland: World Health Organization (WHO), 2017
Philippines Vital Registration - Deaths 2010 ICD10 as it appears in World Health Organization (WHO). WHO Mortality Database Version October 2017. Geneva, Switzerland: World Health Organization (WHO), 2017

Philippines Vital Registration - Deaths 2011 ICD10 as it appears in World Health Organization (WHO). WHO Mortality Database Version October 2017. Geneva, Switzerland: World Health Organization (WHO), 2017
Poland Vital Registration - Deaths 1980 ICD9 as it appears in World Health Organization (WHO). WHO Mortality Database Version September 2016. Geneva, Switzerland: World Health Organization (WHO), 2016
Poland Vital Registration - Deaths 1981 ICD9 as it appears in World Health Organization (WHO). WHO Mortality Database Version September 2016. Geneva, Switzerland: World Health Organization (WHO), 2016
Poland Vital Registration - Deaths 1982 ICD9 as it appears in World Health Organization (WHO). WHO Mortality Database Version September 2016. Geneva, Switzerland: World Health Organization (WHO), 2016
Poland Vital Registration - Deaths 1983 ICD9 as it appears in World Health Organization (WHO). WHO Mortality Database Version September 2016. Geneva, Switzerland: World Health Organization (WHO), 2016
Poland Vital Registration - Deaths 1984 ICD9 as it appears in World Health Organization (WHO). WHO Mortality Database Version September 2016. Geneva, Switzerland: World Health Organization (WHO), 2016
Poland Vital Registration - Deaths 1985 ICD9 as it appears in World Health Organization (WHO). WHO Mortality Database Version September 2016. Geneva, Switzerland: World Health Organization (WHO), 2016
Poland Vital Registration - Deaths 1986 ICD9 as it appears in World Health Organization (WHO). WHO Mortality Database Version September 2016. Geneva, Switzerland: World Health Organization (WHO), 2016
Poland Vital Registration - Deaths 1987 ICD9 as it appears in World Health Organization (WHO). WHO Mortality Database Version September 2016. Geneva, Switzerland: World Health Organization (WHO), 2016
Poland Vital Registration - Deaths 1988 ICD9 as it appears in World Health Organization (WHO). WHO Mortality Database Version September 2016. Geneva, Switzerland: World Health Organization (WHO), 2016
Poland Vital Registration - Deaths 1989 ICD9 as it appears in World Health Organization (WHO). WHO Mortality Database Version September 2016. Geneva, Switzerland: World Health Organization (WHO), 2016
Poland Vital Registration - Deaths 1990 ICD9 as it appears in World Health Organization (WHO). WHO Mortality Database Version September 2016. Geneva, Switzerland: World Health Organization (WHO), 2016
Poland Vital Registration - Deaths 1991 ICD9 as it appears in World Health Organization (WHO). WHO Mortality Database Version September 2016. Geneva, Switzerland: World Health Organization (WHO), 2016
Poland Vital Registration - Deaths 1992 ICD9 as it appears in World Health Organization (WHO). WHO Mortality Database Version September 2016. Geneva, Switzerland: World Health Organization (WHO), 2016
Poland Vital Registration - Deaths 1993 ICD9 as it appears in World Health Organization (WHO). WHO Mortality Database Version September 2016. Geneva, Switzerland: World Health Organization (WHO), 2016
Poland Vital Registration - Deaths 1994 ICD9 as it appears in World Health Organization (WHO). WHO Mortality Database Version September 2016. Geneva, Switzerland: World Health Organization (WHO), 2016
Poland Vital Registration - Deaths 1995 ICD9 as it appears in World Health Organization (WHO). WHO Mortality Database Version September 2016. Geneva, Switzerland: World Health Organization (WHO), 2016
Poland Vital Registration - Deaths 1996 ICD9 as it appears in World Health Organization (WHO). WHO Mortality Database Version September 2016. Geneva, Switzerland: World Health Organization (WHO), 2016

Poland Vital Registration - Deaths 1999 ICD10 as it appears in World Health Organization (WHO). WHO Mortality Database Version October 2017. Geneva, Switzerland: World Health Organization (WHO), 2017

Poland Vital Registration - Deaths 2000 ICD10 as it appears in World Health Organization (WHO). WHO Mortality Database Version October 2017. Geneva, Switzerland: World Health Organization (WHO), 2017

Poland Vital Registration - Deaths 2001 ICD10 as it appears in World Health Organization (WHO). WHO Mortality Database Version October 2017. Geneva, Switzerland: World Health Organization (WHO), 2017

Poland Vital Registration - Deaths 2002 ICD10 as it appears in World Health Organization (WHO). WHO Mortality Database Version October 2017. Geneva, Switzerland: World Health Organization (WHO), 2017

Poland Vital Registration - Deaths 2003 ICD10 as it appears in World Health Organization (WHO). WHO Mortality Database Version October 2017. Geneva, Switzerland: World Health Organization (WHO), 2017

Poland Vital Registration - Deaths 2004 ICD10 as it appears in World Health Organization (WHO). WHO Mortality Database Version October 2017. Geneva, Switzerland: World Health Organization (WHO), 2017

Poland Vital Registration - Deaths 2005 ICD10 as it appears in World Health Organization (WHO). WHO Mortality Database Version October 2017. Geneva, Switzerland: World Health Organization (WHO), 2017

Poland Vital Registration - Deaths 2006 ICD10 as it appears in World Health Organization (WHO). WHO Mortality Database Version October 2017. Geneva, Switzerland: World Health Organization (WHO), 2017

Poland Vital Registration - Deaths 2007 ICD10 as it appears in World Health Organization (WHO). WHO Mortality Database Version October 2017. Geneva, Switzerland: World Health Organization (WHO), 2017

Poland Vital Registration - Deaths 2008 ICD10 as it appears in World Health Organization (WHO). WHO Mortality Database Version October 2017. Geneva, Switzerland: World Health Organization (WHO), 2017

Poland Vital Registration - Deaths 2009 ICD10 as it appears in World Health Organization (WHO). WHO Mortality Database Version October 2017. Geneva, Switzerland: World Health Organization (WHO), 2017

Poland Vital Registration - Deaths 2010 ICD10 as it appears in World Health Organization (WHO). WHO Mortality Database Version October 2017. Geneva, Switzerland: World Health Organization (WHO), 2017

Poland Vital Registration - Deaths 2011 ICD10 as it appears in World Health Organization (WHO). WHO Mortality Database Version October 2017. Geneva, Switzerland: World Health Organization (WHO), 2017

Poland Vital Registration - Deaths 2012 ICD10 as it appears in World Health Organization (WHO). WHO Mortality Database Version October 2017. Geneva, Switzerland: World Health Organization (WHO), 2017

Poland Vital Registration - Deaths 2013 ICD10 as it appears in World Health Organization (WHO). WHO Mortality Database Version October 2017. Geneva, Switzerland: World Health Organization (WHO), 2017

Poland Vital Registration - Deaths 2014 ICD10 as it appears in World Health Organization (WHO). WHO Mortality Database Version October 2017. Geneva, Switzerland: World Health Organization (WHO), 2017

Poland Vital Registration - Deaths 2015 ICD10 as it appears in World Health Organization (WHO). WHO Mortality Database Version October 2017. Geneva, Switzerland: World Health Organization (WHO), 2017

Porapakkham Y, Rao C, Pattaraarchachai J, Polprasert W, Vos T, Adair T, Lopez AD. Estimated causes of death in Thailand, 2005: implications for health policy. Popul Health Metr. 2010; 8:14

Portugal Vital Registration - Deaths 1984 ICD9 as it appears in World Health Organization (WHO). WHO Mortality Database Version November 2015. Geneva, Switzerland: World Health Organization (WHO), 2015
Portugal Vital Registration - Deaths 1985 ICD9 as it appears in World Health Organization (WHO). WHO Mortality Database Version November 2015. Geneva, Switzerland: World Health Organization (WHO), 2015
Portugal Vital Registration - Deaths 1986 ICD9 as it appears in World Health Organization (WHO). WHO Mortality Database Version November 2015. Geneva, Switzerland: World Health Organization (WHO), 2015
Portugal Vital Registration - Deaths 1987 ICD9 as it appears in World Health Organization (WHO). WHO Mortality Database Version November 2015. Geneva, Switzerland: World Health Organization (WHO), 2015
Portugal Vital Registration - Deaths 1988 ICD9 as it appears in World Health Organization (WHO). WHO Mortality Database Version November 2015. Geneva, Switzerland: World Health Organization (WHO), 2015
Portugal Vital Registration - Deaths 1989 ICD9 as it appears in World Health Organization (WHO). WHO Mortality Database Version November 2015. Geneva, Switzerland: World Health Organization (WHO), 2015
Portugal Vital Registration - Deaths 1990 ICD9 as it appears in World Health Organization (WHO). WHO Mortality Database Version November 2015. Geneva, Switzerland: World Health Organization (WHO), 2015
Portugal Vital Registration - Deaths 1991 ICD9 as it appears in World Health Organization (WHO). WHO Mortality Database Version November 2015. Geneva, Switzerland: World Health Organization (WHO), 2015
Portugal Vital Registration - Deaths 1992 ICD9 as it appears in World Health Organization (WHO). WHO Mortality Database Version November 2015. Geneva, Switzerland: World Health Organization (WHO), 2015
Portugal Vital Registration - Deaths 1993 ICD9 as it appears in World Health Organization (WHO). WHO Mortality Database Version November 2015. Geneva, Switzerland: World Health Organization (WHO), 2015
Portugal Vital Registration - Deaths 1994 ICD9 as it appears in World Health Organization (WHO). WHO Mortality Database Version November 2015. Geneva, Switzerland: World Health Organization (WHO), 2015
Portugal Vital Registration - Deaths 1995 ICD9 as it appears in World Health Organization (WHO). WHO Mortality Database Version November 2015. Geneva, Switzerland: World Health Organization (WHO), 2015
Portugal Vital Registration - Deaths 1996 ICD9 as it appears in World Health Organization (WHO). WHO Mortality Database Version November 2015. Geneva, Switzerland: World Health Organization (WHO), 2015
Portugal Vital Registration - Deaths 1997 ICD9 as it appears in World Health Organization (WHO). WHO Mortality Database Version November 2015. Geneva, Switzerland: World Health Organization (WHO), 2015
Portugal Vital Registration - Deaths 1998 ICD9 as it appears in World Health Organization (WHO). WHO Mortality Database Version November 2015. Geneva, Switzerland: World Health Organization (WHO), 2015
Portugal Vital Registration - Deaths 1999 ICD9 as it appears in World Health Organization (WHO). WHO Mortality Database Version November 2015. Geneva, Switzerland: World Health Organization (WHO), 2015
Portugal Vital Registration - Deaths 2000 ICD9 as it appears in World Health Organization (WHO). WHO Mortality Database Version November 2015. Geneva, Switzerland: World Health Organization (WHO), 2015
Portugal Vital Registration - Deaths 2001 ICD9 as it appears in World Health Organization (WHO). WHO Mortality Database Version November 2015. Geneva, Switzerland: World Health Organization (WHO), 2015

Portugal Vital Registration - Deaths 2002 ICD10 as it appears in World Health Organization (WHO). WHO Mortality Database Version October 2017. Geneva, Switzerland: World Health Organization (WHO), 2017
Portugal Vital Registration - Deaths 2003 ICD10 as it appears in World Health Organization (WHO). WHO Mortality Database Version October 2017. Geneva, Switzerland: World Health Organization (WHO), 2017
Portugal Vital Registration - Deaths 2007 ICD10 as it appears in World Health Organization (WHO). WHO Mortality Database Version October 2017. Geneva, Switzerland: World Health Organization (WHO), 2017
Portugal Vital Registration - Deaths 2008 ICD10 as it appears in World Health Organization (WHO). WHO Mortality Database Version October 2017. Geneva, Switzerland: World Health Organization (WHO), 2017
Portugal Vital Registration - Deaths 2009 ICD10 as it appears in World Health Organization (WHO). WHO Mortality Database Version October 2017. Geneva, Switzerland: World Health Organization (WHO), 2017
Portugal Vital Registration - Deaths 2010 ICD10 as it appears in World Health Organization (WHO). WHO Mortality Database Version October 2017. Geneva, Switzerland: World Health Organization (WHO), 2017
Portugal Vital Registration - Deaths 2011 ICD10 as it appears in World Health Organization (WHO). WHO Mortality Database Version October 2017. Geneva, Switzerland: World Health Organization (WHO), 2017
Portugal Vital Registration - Deaths 2012 ICD10 as it appears in World Health Organization (WHO). WHO Mortality Database Version October 2017. Geneva, Switzerland: World Health Organization (WHO), 2017
Portugal Vital Registration - Deaths 2013 ICD10 as it appears in World Health Organization (WHO). WHO Mortality Database Version October 2017. Geneva, Switzerland: World Health Organization (WHO), 2017
Portugal Vital Registration - Deaths 2014 ICD10 as it appears in World Health Organization (WHO). WHO Mortality Database Version October 2017. Geneva, Switzerland: World Health Organization (WHO), 2017
Public Health Foundation of India. India Cause of Death Estimation Study in Bihar 2011-2014
Puerto Rico Vital Registration - Deaths 1980 ICD9 as it appears in World Health Organization (WHO). WHO Mortality Database Version September 2016. Geneva, Switzerland: World Health Organization (WHO), 2016
Puerto Rico Vital Registration - Deaths 1981 ICD9 as it appears in World Health Organization (WHO). WHO Mortality Database Version September 2016. Geneva, Switzerland: World Health Organization (WHO), 2016
Puerto Rico Vital Registration - Deaths 1982 ICD9 as it appears in World Health Organization (WHO). WHO Mortality Database Version September 2016. Geneva, Switzerland: World Health Organization (WHO), 2016
Puerto Rico Vital Registration - Deaths 1983 ICD9 as it appears in World Health Organization (WHO). WHO Mortality Database Version September 2016. Geneva, Switzerland: World Health Organization (WHO), 2016
Puerto Rico Vital Registration - Deaths 1984 ICD9 as it appears in World Health Organization (WHO). WHO Mortality Database Version September 2016. Geneva, Switzerland: World Health Organization (WHO), 2016
Puerto Rico Vital Registration - Deaths 1985 ICD9 as it appears in World Health Organization (WHO). WHO Mortality Database Version September 2016. Geneva, Switzerland: World Health Organization (WHO), 2016
Puerto Rico Vital Registration - Deaths 1986 ICD9 as it appears in World Health Organization (WHO). WHO Mortality Database Version September 2016. Geneva, Switzerland: World Health Organization (WHO), 2016

Puerto Rico Vital Registration - Deaths 1987 ICD9 as it appears in World Health Organization (WHO). WHO Mortality Database Version September 2016. Geneva, Switzerland: World Health Organization (WHO), 2016
Puerto Rico Vital Registration - Deaths 1988 ICD9 as it appears in World Health Organization (WHO). WHO Mortality Database Version September 2016. Geneva, Switzerland: World Health Organization (WHO), 2016
Puerto Rico Vital Registration - Deaths 1989 ICD9 as it appears in World Health Organization (WHO). WHO Mortality Database Version September 2016. Geneva, Switzerland: World Health Organization (WHO), 2016
Puerto Rico Vital Registration - Deaths 1990 ICD9 as it appears in World Health Organization (WHO). WHO Mortality Database Version September 2016. Geneva, Switzerland: World Health Organization (WHO), 2016
Puerto Rico Vital Registration - Deaths 1991 ICD9 as it appears in World Health Organization (WHO). WHO Mortality Database Version September 2016. Geneva, Switzerland: World Health Organization (WHO), 2016
Puerto Rico Vital Registration - Deaths 1992 ICD9 as it appears in World Health Organization (WHO). WHO Mortality Database Version September 2016. Geneva, Switzerland: World Health Organization (WHO), 2016
Puerto Rico Vital Registration - Deaths 1993 ICD9 as it appears in World Health Organization (WHO). WHO Mortality Database Version September 2016. Geneva, Switzerland: World Health Organization (WHO), 2016
Qatar Vital Registration - Deaths 1995 ICD10 as it appears in World Health Organization (WHO). WHO Mortality Database Version October 2017. Geneva, Switzerland: World Health Organization (WHO), 2017
Qatar Vital Registration - Deaths 2001 ICD10 as it appears in World Health Organization (WHO). WHO Mortality Database Version October 2017. Geneva, Switzerland: World Health Organization (WHO), 2017
Qatar Vital Registration - Deaths 2004 ICD10 as it appears in World Health Organization (WHO). WHO Mortality Database Version October 2017. Geneva, Switzerland: World Health Organization (WHO), 2017
Qatar Vital Registration - Deaths 2005 ICD10 as it appears in World Health Organization (WHO). WHO Mortality Database Version October 2017. Geneva, Switzerland: World Health Organization (WHO), 2017
Qatar Vital Registration - Deaths 2006 ICD10 as it appears in World Health Organization (WHO). WHO Mortality Database Version October 2017. Geneva, Switzerland: World Health Organization (WHO), 2017
Qatar Vital Registration - Deaths 2007 ICD10 as it appears in World Health Organization (WHO). WHO Mortality Database Version October 2017. Geneva, Switzerland: World Health Organization (WHO), 2017
Qatar Vital Registration - Deaths 2008 ICD10 as it appears in World Health Organization (WHO). WHO Mortality Database Version October 2017. Geneva, Switzerland: World Health Organization (WHO), 2017
Qatar Vital Registration - Deaths 2009 ICD10 as it appears in World Health Organization (WHO). WHO Mortality Database Version October 2017. Geneva, Switzerland: World Health Organization (WHO), 2017
Qatar Vital Registration - Deaths 2010 ICD10 as it appears in World Health Organization (WHO). WHO Mortality Database Version October 2017. Geneva, Switzerland: World Health Organization (WHO), 2017
Qatar Vital Registration - Deaths 2011 ICD10 as it appears in World Health Organization (WHO). WHO Mortality Database Version October 2017. Geneva, Switzerland: World Health Organization (WHO), 2017
Qatar Vital Registration - Deaths 2012 ICD10 as it appears in World Health Organization (WHO). WHO Mortality Database Version October 2017. Geneva, Switzerland: World Health Organization (WHO), 2017

Qatar Vital Registration - Deaths 2013 ICD10 as it appears in World Health Organization (WHO). WHO Mortality Database Version March 2017. Geneva, Switzerland: World Health Organization (WHO), 2017

Qatar Vital Registration - Deaths 2014 ICD10 as it appears in World Health Organization (WHO). WHO Mortality Database Version March 2017. Geneva, Switzerland: World Health Organization (WHO), 2017

Qatar Vital Registration - Deaths 2015 ICD10 as it appears in World Health Organization (WHO). WHO Mortality Database Version October 2017. Geneva, Switzerland: World Health Organization (WHO), 2017

Qatar Vital Registration - Deaths 2016 ICD10 as it appears in World Health Organization (WHO). WHO Mortality Database Version April 2018. Geneva, Switzerland: World Health Organization (WHO), 2018

Quyen BTT, Nhung NT, Cuong PV. The causes of deaths in Chililab between 2008-2010 based on verbal autopsy method. Vietnam J Public Health. 2012; 1(1): 24-31

Rahman SM, Åkesson A, Kippler M, Grandér M, Hamadani JD, Streatfield PK, Persson L-Å, Arifeen SE, Vahter M. Elevated Manganese Concentrations in Drinking Water May Be Beneficial for Fetal Survival. PLoS One. 2013; 8(9): e74119

Rai SK, Kant S, Srivastava R, Gupta P, Misra P, Pandav CS, Singh AK. Causes of and contributors to infant mortality in a rural community of North India: evidence from verbal and social autopsy. BMJ Open. 2017; 7(8): e012856

Registrar General's Department (Sri Lanka). Sri Lanka Vital Registration - Deaths 2009

Registrar General's Department (Sri Lanka). Sri Lanka Vital Registration - Deaths 2010

Registrar General's Department (Zimbabwe), Zimbabwe National Statistics Agency. Zimbabwe Mortality Report 2007

Republic of Moldova Vital Registration - Deaths 1981 ICD9 as it appears in World Health Organization (WHO). WHO Mortality Database Version September 2016. Geneva, Switzerland: World Health Organization (WHO), 2016

Republic of Moldova Vital Registration - Deaths 1982 ICD9 as it appears in World Health Organization (WHO). WHO Mortality Database Version September 2016. Geneva, Switzerland: World Health Organization (WHO), 2016

Republic of Moldova Vital Registration - Deaths 1985 ICD9 as it appears in World Health Organization (WHO). WHO Mortality Database Version September 2016. Geneva, Switzerland: World Health Organization (WHO), 2016

Republic of Moldova Vital Registration - Deaths 1986 ICD9 as it appears in World Health Organization (WHO). WHO Mortality Database Version September 2016. Geneva, Switzerland: World Health Organization (WHO), 2016

Republic of Moldova Vital Registration - Deaths 1987 ICD9 as it appears in World Health Organization (WHO). WHO Mortality Database Version September 2016. Geneva, Switzerland: World Health Organization (WHO), 2016

Republic of Moldova Vital Registration - Deaths 1988 ICD9 as it appears in World Health Organization (WHO). WHO Mortality Database Version September 2016. Geneva, Switzerland: World Health Organization (WHO), 2016

Republic of Moldova Vital Registration - Deaths 1989 ICD9 as it appears in World Health Organization (WHO). WHO Mortality Database Version September 2016. Geneva, Switzerland: World Health Organization (WHO), 2016

Republic of Moldova Vital Registration - Deaths 1990 ICD9 as it appears in World Health Organization (WHO). WHO Mortality Database Version September 2016. Geneva, Switzerland: World Health Organization (WHO), 2016

Republic of Moldova Vital Registration - Deaths 1991 ICD9 as it appears in World Health Organization (WHO). WHO Mortality Database Version September 2016. Geneva, Switzerland: World Health Organization (WHO), 2016

Republic of Moldova Vital Registration - Deaths 1992 ICD9 as it appears in World Health Organization (WHO). WHO Mortality Database Version September 2016. Geneva, Switzerland: World Health Organization (WHO), 2016
Republic of Moldova Vital Registration - Deaths 1993 ICD9 as it appears in World Health Organization (WHO). WHO Mortality Database Version September 2016. Geneva, Switzerland: World Health Organization (WHO), 2016
Republic of Moldova Vital Registration - Deaths 1994 ICD9 as it appears in World Health Organization (WHO). WHO Mortality Database Version September 2016. Geneva, Switzerland: World Health Organization (WHO), 2016
Republic of Moldova Vital Registration - Deaths 1995 ICD9 as it appears in World Health Organization (WHO). WHO Mortality Database Version September 2016. Geneva, Switzerland: World Health Organization (WHO), 2016
Republic of Moldova Vital Registration - Deaths 1996 ICD10 as it appears in World Health Organization (WHO). WHO Mortality Database Version October 2017. Geneva, Switzerland: World Health Organization (WHO), 2017
Republic of Moldova Vital Registration - Deaths 1997 ICD10 as it appears in World Health Organization (WHO). WHO Mortality Database Version October 2017. Geneva, Switzerland: World Health Organization (WHO), 2017
Republic of Moldova Vital Registration - Deaths 1998 ICD10 as it appears in World Health Organization (WHO). WHO Mortality Database Version October 2017. Geneva, Switzerland: World Health Organization (WHO), 2017
Republic of Moldova Vital Registration - Deaths 1999 ICD10 as it appears in World Health Organization (WHO). WHO Mortality Database Version October 2017. Geneva, Switzerland: World Health Organization (WHO), 2017
Republic of Moldova Vital Registration - Deaths 2000 ICD10 as it appears in World Health Organization (WHO). WHO Mortality Database Version October 2017. Geneva, Switzerland: World Health Organization (WHO), 2017
Republic of Moldova Vital Registration - Deaths 2001 ICD10 as it appears in World Health Organization (WHO). WHO Mortality Database Version October 2017. Geneva, Switzerland: World Health Organization (WHO), 2017
Republic of Moldova Vital Registration - Deaths 2002 ICD10 as it appears in World Health Organization (WHO). WHO Mortality Database Version October 2017. Geneva, Switzerland: World Health Organization (WHO), 2017
Republic of Moldova Vital Registration - Deaths 2003 ICD10 as it appears in World Health Organization (WHO). WHO Mortality Database Version October 2017. Geneva, Switzerland: World Health Organization (WHO), 2017
Republic of Moldova Vital Registration - Deaths 2004 ICD10 as it appears in World Health Organization (WHO). WHO Mortality Database Version October 2017. Geneva, Switzerland: World Health Organization (WHO), 2017
Republic of Moldova Vital Registration - Deaths 2005 ICD10 as it appears in World Health Organization (WHO). WHO Mortality Database Version October 2017. Geneva, Switzerland: World Health Organization (WHO), 2017
Republic of Moldova Vital Registration - Deaths 2006 ICD10 as it appears in World Health Organization (WHO). WHO Mortality Database Version October 2017. Geneva, Switzerland: World Health Organization (WHO), 2017
Republic of Moldova Vital Registration - Deaths 2007 ICD10 as it appears in World Health Organization (WHO). WHO Mortality Database Version October 2017. Geneva, Switzerland: World Health Organization (WHO), 2017
Republic of Moldova Vital Registration - Deaths 2008 ICD10 as it appears in World Health Organization (WHO). WHO Mortality Database Version October 2017. Geneva, Switzerland: World Health Organization (WHO), 2017
Republic of Moldova Vital Registration - Deaths 2009 ICD10 as it appears in World Health Organization (WHO). WHO Mortality Database Version October 2017. Geneva, Switzerland: World Health Organization (WHO), 2017

Romania Vital Registration - Deaths 1980 ICD9 as it appears in World Health Organization (WHO). WHO Mortality Database Version September 2016. Geneva, Switzerland: World Health Organization (WHO), 2016
Romania Vital Registration - Deaths 1981 ICD9 as it appears in World Health Organization (WHO). WHO Mortality Database Version September 2016. Geneva, Switzerland: World Health Organization (WHO), 2016
Romania Vital Registration - Deaths 1982 ICD9 as it appears in World Health Organization (WHO). WHO Mortality Database Version September 2016. Geneva, Switzerland: World Health Organization (WHO), 2016
Romania Vital Registration - Deaths 1983 ICD9 as it appears in World Health Organization (WHO). WHO Mortality Database Version September 2016. Geneva, Switzerland: World Health Organization (WHO), 2016
Romania Vital Registration - Deaths 1984 ICD9 as it appears in World Health Organization (WHO). WHO Mortality Database Version September 2016. Geneva, Switzerland: World Health Organization (WHO), 2016
Romania Vital Registration - Deaths 1985 ICD9 as it appears in World Health Organization (WHO). WHO Mortality Database Version September 2016. Geneva, Switzerland: World Health Organization (WHO), 2016
Romania Vital Registration - Deaths 1986 ICD9 as it appears in World Health Organization (WHO). WHO Mortality Database Version September 2016. Geneva, Switzerland: World Health Organization (WHO), 2016
Romania Vital Registration - Deaths 1987 ICD9 as it appears in World Health Organization (WHO). WHO Mortality Database Version September 2016. Geneva, Switzerland: World Health Organization (WHO), 2016
Romania Vital Registration - Deaths 1988 ICD9 as it appears in World Health Organization (WHO). WHO Mortality Database Version September 2016. Geneva, Switzerland: World Health Organization (WHO), 2016
Romania Vital Registration - Deaths 1989 ICD9 as it appears in World Health Organization (WHO). WHO Mortality Database Version September 2016. Geneva, Switzerland: World Health Organization (WHO), 2016
Romania Vital Registration - Deaths 1990 ICD9 as it appears in World Health Organization (WHO). WHO Mortality Database Version September 2016. Geneva, Switzerland: World Health Organization (WHO), 2016
Romania Vital Registration - Deaths 1991 ICD9 as it appears in World Health Organization (WHO). WHO Mortality Database Version September 2016. Geneva, Switzerland: World Health Organization (WHO), 2016
Romania Vital Registration - Deaths 1992 ICD9 as it appears in World Health Organization (WHO). WHO Mortality Database Version September 2016. Geneva, Switzerland: World Health Organization (WHO), 2016
Romania Vital Registration - Deaths 1993 ICD9 as it appears in World Health Organization (WHO). WHO Mortality Database Version September 2016. Geneva, Switzerland: World Health Organization (WHO), 2016
Romania Vital Registration - Deaths 1994 ICD9 as it appears in World Health Organization (WHO). WHO Mortality Database Version September 2016. Geneva, Switzerland: World Health Organization (WHO), 2016
Romania Vital Registration - Deaths 1995 ICD9 as it appears in World Health Organization (WHO). WHO Mortality Database Version September 2016. Geneva, Switzerland: World Health Organization (WHO), 2016
Romania Vital Registration - Deaths 1996 ICD9 as it appears in World Health Organization (WHO). WHO Mortality Database Version September 2016. Geneva, Switzerland: World Health Organization (WHO), 2016
Romania Vital Registration - Deaths 1997 ICD9 as it appears in World Health Organization (WHO). WHO Mortality Database Version September 2016. Geneva, Switzerland: World Health Organization (WHO), 2016

Romania Vital Registration - Deaths 1998 ICD9 as it appears in World Health Organization (WHO). WHO Mortality Database Version September 2016. Geneva, Switzerland: World Health Organization (WHO), 2016
Romania Vital Registration - Deaths 1999 ICD10 as it appears in World Health Organization (WHO). WHO Mortality Database Version October 2017. Geneva, Switzerland: World Health Organization (WHO), 2017
Romania Vital Registration - Deaths 2000 ICD10 as it appears in World Health Organization (WHO). WHO Mortality Database Version October 2017. Geneva, Switzerland: World Health Organization (WHO), 2017
Romania Vital Registration - Deaths 2001 ICD10 as it appears in World Health Organization (WHO). WHO Mortality Database Version October 2017. Geneva, Switzerland: World Health Organization (WHO), 2017
Romania Vital Registration - Deaths 2002 ICD10 as it appears in World Health Organization (WHO). WHO Mortality Database Version October 2017. Geneva, Switzerland: World Health Organization (WHO), 2017
Romania Vital Registration - Deaths 2003 ICD10 as it appears in World Health Organization (WHO). WHO Mortality Database Version October 2017. Geneva, Switzerland: World Health Organization (WHO), 2017
Romania Vital Registration - Deaths 2004 ICD10 as it appears in World Health Organization (WHO). WHO Mortality Database Version October 2017. Geneva, Switzerland: World Health Organization (WHO), 2017
Romania Vital Registration - Deaths 2005 ICD10 as it appears in World Health Organization (WHO). WHO Mortality Database Version October 2017. Geneva, Switzerland: World Health Organization (WHO), 2017
Romania Vital Registration - Deaths 2006 ICD10 as it appears in World Health Organization (WHO). WHO Mortality Database Version October 2017. Geneva, Switzerland: World Health Organization (WHO), 2017
Romania Vital Registration - Deaths 2007 ICD10 as it appears in World Health Organization (WHO). WHO Mortality Database Version October 2017. Geneva, Switzerland: World Health Organization (WHO), 2017
Romania Vital Registration - Deaths 2008 ICD10 as it appears in World Health Organization (WHO). WHO Mortality Database Version October 2017. Geneva, Switzerland: World Health Organization (WHO), 2017
Romania Vital Registration - Deaths 2009 ICD10 as it appears in World Health Organization (WHO). WHO Mortality Database Version October 2017. Geneva, Switzerland: World Health Organization (WHO), 2017
Romania Vital Registration - Deaths 2010 ICD10 as it appears in World Health Organization (WHO). WHO Mortality Database Version October 2017. Geneva, Switzerland: World Health Organization (WHO), 2017
Romania Vital Registration - Deaths 2011 ICD10 as it appears in World Health Organization (WHO). WHO Mortality Database Version October 2017. Geneva, Switzerland: World Health Organization (WHO), 2017
Romania Vital Registration - Deaths 2012 ICD10 as it appears in World Health Organization (WHO). WHO Mortality Database Version October 2017. Geneva, Switzerland: World Health Organization (WHO), 2017
Romania Vital Registration - Deaths 2013 ICD10 as it appears in World Health Organization (WHO). WHO Mortality Database Version October 2017. Geneva, Switzerland: World Health Organization (WHO), 2017
Romania Vital Registration - Deaths 2014 ICD10 as it appears in World Health Organization (WHO). WHO Mortality Database Version October 2017. Geneva, Switzerland: World Health Organization (WHO), 2017
Romania Vital Registration - Deaths 2015 ICD10 as it appears in World Health Organization (WHO). WHO Mortality Database Version March 2017. Geneva, Switzerland: World Health Organization (WHO), 2017

Romania Vital Registration - Deaths 2016 ICD10 as it appears in World Health Organization (WHO). WHO Mortality Database Version April 2018. Geneva, Switzerland: World Health Organization (WHO), 2018
Russia Mortality by Region, Age, Sex, and Cause of Death 1989 as it appears in Center for Demographic Research, New Economic School (Russia). Russia Mortality Rates by Region, Age, Sex, and Cause of Death 1989-1998. Moscow, Russia: Center for Demographic Research, New Economic School (Russia). http://demogr.nes.ru/index.php/ru/demogr_indicat/data
Russia Mortality by Region, Age, Sex, and Cause of Death 1990 as it appears in Center for Demographic Research, New Economic School (Russia). Russia Mortality Rates by Region, Age, Sex, and Cause of Death 1989-1998. Moscow, Russia: Center for Demographic Research, New Economic School (Russia). http://demogr.nes.ru/index.php/ru/demogr_indicat/data
Russia Mortality by Region, Age, Sex, and Cause of Death 1991 as it appears in Center for Demographic Research, New Economic School (Russia). Russia Mortality Rates by Region, Age, Sex, and Cause of Death 1989-1998. Moscow, Russia: Center for Demographic Research, New Economic School (Russia). http://demogr.nes.ru/index.php/ru/demogr_indicat/data
Russia Mortality by Region, Age, Sex, and Cause of Death 1992 as it appears in Center for Demographic Research, New Economic School (Russia). Russia Mortality Rates by Region, Age, Sex, and Cause of Death 1989-1998. Moscow, Russia: Center for Demographic Research, New Economic School (Russia). http://demogr.nes.ru/index.php/ru/demogr_indicat/data
Russia Mortality by Region, Age, Sex, and Cause of Death 1993 as it appears in Center for Demographic Research, New Economic School (Russia). Russia Mortality Rates by Region, Age, Sex, and Cause of Death 1989-1998. Moscow, Russia: Center for Demographic Research, New Economic School (Russia). http://demogr.nes.ru/index.php/ru/demogr_indicat/data
Russia Mortality by Region, Age, Sex, and Cause of Death 1994 as it appears in Center for Demographic Research, New Economic School (Russia). Russia Mortality Rates by Region, Age, Sex, and Cause of Death 1989-1998. Moscow, Russia: Center for Demographic Research, New Economic School (Russia). http://demogr.nes.ru/index.php/ru/demogr_indicat/data
Russia Mortality by Region, Age, Sex, and Cause of Death 1995 as it appears in Center for Demographic Research, New Economic School (Russia). Russia Mortality Rates by Region, Age, Sex, and Cause of Death 1989-1998. Moscow, Russia: Center for Demographic Research, New Economic School (Russia). http://demogr.nes.ru/index.php/ru/demogr_indicat/data
Russia Mortality by Region, Age, Sex, and Cause of Death 1996 as it appears in Center for Demographic Research, New Economic School (Russia). Russia Mortality Rates by Region, Age, Sex, and Cause of Death 1989-1998. Moscow, Russia: Center for Demographic Research, New Economic School (Russia). http://demogr.nes.ru/index.php/ru/demogr_indicat/data
Russia Mortality by Region, Age, Sex, and Cause of Death 1997 as it appears in Center for Demographic Research, New Economic School (Russia). Russia Mortality Rates by Region, Age, Sex, and Cause of Death 1989-1998. Moscow, Russia: Center for Demographic Research, New Economic School (Russia). http://demogr.nes.ru/index.php/ru/demogr_indicat/data
Russia Mortality by Region, Age, Sex, and Cause of Death 1998 as it appears in Center for Demographic Research, New Economic School (Russia). Russia Mortality Rates by Region, Age, Sex, and Cause of Death 1989-1998. Moscow, Russia: Center for Demographic Research, New Economic School (Russia). http://demogr.nes.ru/index.php/ru/demogr_indicat/data
Russia Mortality by Region, Age, Sex, and Cause of Death 1999 as it appears in Center for Demographic Research, New Economic School (Russia). Russia Mortality Rates by Region, Age, Sex, and Cause of Death 1999-2005. Moscow, Russia: Center for Demographic Research, New Economic School (Russia). http://demogr.nes.ru/index.php/ru/demogr_indicat/data

Russia Mortality by Region, Age, Sex, and Cause of Death 2000 as it appears in Center for Demographic Research, New Economic School (Russia). Russia Mortality Rates by Region, Age, Sex, and Cause of Death 1999-2005. Moscow, Russia: Center for Demographic Research, New Economic School (Russia). http://demogr.nes.ru/index.php/ru/demogr_indicat/data
Russia Mortality by Region, Age, Sex, and Cause of Death 2001 as it appears in Center for Demographic Research, New Economic School (Russia). Russia Mortality Rates by Region, Age, Sex, and Cause of Death 1999-2005. Moscow, Russia: Center for Demographic Research, New Economic School (Russia). http://demogr.nes.ru/index.php/ru/demogr_indicat/data
Russia Mortality by Region, Age, Sex, and Cause of Death 2002 as it appears in Center for Demographic Research, New Economic School (Russia). Russia Mortality Rates by Region, Age, Sex, and Cause of Death 1999-2005. Moscow, Russia: Center for Demographic Research, New Economic School (Russia). http://demogr.nes.ru/index.php/ru/demogr_indicat/data
Russia Mortality by Region, Age, Sex, and Cause of Death 2003 as it appears in Center for Demographic Research, New Economic School (Russia). Russia Mortality Rates by Region, Age, Sex, and Cause of Death 1999-2005. Moscow, Russia: Center for Demographic Research, New Economic School (Russia). http://demogr.nes.ru/index.php/ru/demogr_indicat/data
Russia Mortality by Region, Age, Sex, and Cause of Death 2004 as it appears in Center for Demographic Research, New Economic School (Russia). Russia Mortality Rates by Region, Age, Sex, and Cause of Death 1999-2005. Moscow, Russia: Center for Demographic Research, New Economic School (Russia). http://demogr.nes.ru/index.php/ru/demogr_indicat/data
Russia Mortality by Region, Age, Sex, and Cause of Death 2005 as it appears in Center for Demographic Research, New Economic School (Russia). Russia Mortality Rates by Region, Age, Sex, and Cause of Death 1999-2005. Moscow, Russia: Center for Demographic Research, New Economic School (Russia). http://demogr.nes.ru/index.php/ru/demogr_indicat/data
Russia Mortality by Region, Age, Sex, and Cause of Death 2006 as it appears in Center for Demographic Research, New Economic School (Russia). Russia Mortality Rates by Region, Age, Sex, and Cause of Death 2006-2012. Moscow, Russia: Center for Demographic Research, New Economic School (Russia). http://demogr.nes.ru/index.php/ru/demogr_indicat/data
Russia Mortality by Region, Age, Sex, and Cause of Death 2007 as it appears in Center for Demographic Research, New Economic School (Russia). Russia Mortality Rates by Region, Age, Sex, and Cause of Death 2006-2012. Moscow, Russia: Center for Demographic Research, New Economic School (Russia). http://demogr.nes.ru/index.php/ru/demogr_indicat/data
Russia Mortality by Region, Age, Sex, and Cause of Death 2008 as it appears in Center for Demographic Research, New Economic School (Russia). Russia Mortality Rates by Region, Age, Sex, and Cause of Death 2006-2012. Moscow, Russia: Center for Demographic Research, New Economic School (Russia). http://demogr.nes.ru/index.php/ru/demogr_indicat/data
Russia Mortality by Region, Age, Sex, and Cause of Death 2009 as it appears in Center for Demographic Research, New Economic School (Russia). Russia Mortality Rates by Region, Age, Sex, and Cause of Death 2006-2012. Moscow, Russia: Center for Demographic Research, New Economic School (Russia). http://demogr.nes.ru/index.php/ru/demogr_indicat/data
Russia Mortality by Region, Age, Sex, and Cause of Death 2010 as it appears in Center for Demographic Research, New Economic School (Russia). Russia Mortality Rates by Region, Age, Sex, and Cause of Death 2006-2012. Moscow, Russia: Center for Demographic Research, New Economic School (Russia). http://demogr.nes.ru/index.php/ru/demogr_indicat/data
Russia Mortality by Region, Age, Sex, and Cause of Death 2011 as it appears in Center for Demographic Research, New Economic School (Russia). Russia Mortality Rates by Region, Cause of Death, 5-Year Age Groups, and Sex, 2011-2014. Moscow, Russia: Center for Demographic Research, New Economic School (Russia). http://demogr.nes.ru/index.php/ru/demogr_indicat/data

Russia Mortality by Region, Age, Sex, and Cause of Death 2012 as it appears in Center for Demographic Research, New Economic School (Russia). Russia Mortality Rates by Region, Cause of Death, 5-Year Age Groups, and Sex, 2011-2014. Moscow, Russia: Center for Demographic Research, New Economic School (Russia). http://demogr.nes.ru/index.php/ru/demogr_indicat/data
Russia Mortality by Region, Age, Sex, and Cause of Death 2013 as it appears in Center for Demographic Research, New Economic School (Russia). Russia Mortality Rates by Region, Cause of Death, 5-Year Age Groups, and Sex, 2011-2014. Moscow, Russia: Center for Demographic Research, New Economic School (Russia). http://demogr.nes.ru/index.php/ru/demogr_indicat/data
Russia Mortality by Region, Age, Sex, and Cause of Death 2014 as it appears in Center for Demographic Research, New Economic School (Russia). Russia Mortality Rates by Region, Cause of Death, 5-Year Age Groups, and Sex, 2011-2014. Moscow, Russia: Center for Demographic Research, New Economic School (Russia). http://demogr.nes.ru/index.php/ru/demogr_indicat/data
Russia Mortality by Region, Age, Sex, and Cause of Death 2015 as it appears in Center for Demographic Research, New Economic School (Russia). Russia Mortality Rates by Region, 1-Year Age Groups, and Sex, 2015-2016. Moscow, Russia: Center for Demographic Research, New Economic School (Russia). http://demogr.nes.ru/index.php/ru/demogr_indicat/data
Russia Mortality by Region, Age, Sex, and Cause of Death 2016 as it appears in Center for Demographic Research, New Economic School (Russia). Russia Mortality Rates by Region, 1-Year Age Groups, and Sex, 2015-2016. Moscow, Russia: Center for Demographic Research, New Economic School (Russia). http://demogr.nes.ru/index.php/ru/demogr_indicat/data
Saint Lucia Vital Registration - Deaths 1980 ICD9 as it appears in World Health Organization (WHO). WHO Mortality Database Version September 2016. Geneva, Switzerland: World Health Organization (WHO), 2016
Saint Lucia Vital Registration - Deaths 1981 ICD9 as it appears in World Health Organization (WHO). WHO Mortality Database Version September 2016. Geneva, Switzerland: World Health Organization (WHO), 2016
Saint Lucia Vital Registration - Deaths 1983 ICD9 as it appears in World Health Organization (WHO). WHO Mortality Database Version September 2016. Geneva, Switzerland: World Health Organization (WHO), 2016
Saint Lucia Vital Registration - Deaths 1986 ICD9 as it appears in World Health Organization (WHO). WHO Mortality Database Version September 2016. Geneva, Switzerland: World Health Organization (WHO), 2016
Saint Lucia Vital Registration - Deaths 1987 ICD9 as it appears in World Health Organization (WHO). WHO Mortality Database Version September 2016. Geneva, Switzerland: World Health Organization (WHO), 2016
Saint Lucia Vital Registration - Deaths 1988 ICD9 as it appears in World Health Organization (WHO). WHO Mortality Database Version September 2016. Geneva, Switzerland: World Health Organization (WHO), 2016
Saint Lucia Vital Registration - Deaths 1989 ICD9 as it appears in World Health Organization (WHO). WHO Mortality Database Version September 2016. Geneva, Switzerland: World Health Organization (WHO), 2016
Saint Lucia Vital Registration - Deaths 1990 ICD9 as it appears in World Health Organization (WHO). WHO Mortality Database Version September 2016. Geneva, Switzerland: World Health Organization (WHO), 2016
Saint Lucia Vital Registration - Deaths 1991 ICD9 as it appears in World Health Organization (WHO). WHO Mortality Database Version September 2016. Geneva, Switzerland: World Health Organization (WHO), 2016
Saint Lucia Vital Registration - Deaths 1992 ICD9 as it appears in World Health Organization (WHO). WHO Mortality Database Version September 2016. Geneva, Switzerland: World Health Organization (WHO), 2016

Saint Lucia Vital Registration - Deaths 1993 ICD9 as it appears in World Health Organization (WHO). WHO Mortality Database Version September 2016. Geneva, Switzerland: World Health Organization (WHO), 2016
Saint Lucia Vital Registration - Deaths 1994 ICD9 as it appears in World Health Organization (WHO). WHO Mortality Database Version September 2016. Geneva, Switzerland: World Health Organization (WHO), 2016
Saint Lucia Vital Registration - Deaths 1995 ICD9 as it appears in World Health Organization (WHO). WHO Mortality Database Version September 2016. Geneva, Switzerland: World Health Organization (WHO), 2016
Saint Lucia Vital Registration - Deaths 1996 ICD10 as it appears in World Health Organization (WHO). WHO Mortality Database Version October 2017. Geneva, Switzerland: World Health Organization (WHO), 2017
Saint Lucia Vital Registration - Deaths 1997 ICD10 as it appears in World Health Organization (WHO). WHO Mortality Database Version October 2017. Geneva, Switzerland: World Health Organization (WHO), 2017
Saint Lucia Vital Registration - Deaths 1998 ICD10 as it appears in World Health Organization (WHO). WHO Mortality Database Version October 2017. Geneva, Switzerland: World Health Organization (WHO), 2017
Saint Lucia Vital Registration - Deaths 1999 ICD10 as it appears in World Health Organization (WHO). WHO Mortality Database Version October 2017. Geneva, Switzerland: World Health Organization (WHO), 2017
Saint Lucia Vital Registration - Deaths 2000 ICD10 as it appears in World Health Organization (WHO). WHO Mortality Database Version October 2017. Geneva, Switzerland: World Health Organization (WHO), 2017
Saint Lucia Vital Registration - Deaths 2001 ICD10 as it appears in World Health Organization (WHO). WHO Mortality Database Version October 2017. Geneva, Switzerland: World Health Organization (WHO), 2017
Saint Lucia Vital Registration - Deaths 2002 ICD10 as it appears in World Health Organization (WHO). WHO Mortality Database Version October 2017. Geneva, Switzerland: World Health Organization (WHO), 2017
Saint Lucia Vital Registration - Deaths 2003 ICD10 as it appears in World Health Organization (WHO). WHO Mortality Database Version October 2017. Geneva, Switzerland: World Health Organization (WHO), 2017
Saint Lucia Vital Registration - Deaths 2004 ICD10 as it appears in World Health Organization (WHO). WHO Mortality Database Version October 2017. Geneva, Switzerland: World Health Organization (WHO), 2017
Saint Lucia Vital Registration - Deaths 2005 ICD10 as it appears in World Health Organization (WHO). WHO Mortality Database Version October 2017. Geneva, Switzerland: World Health Organization (WHO), 2017
Saint Lucia Vital Registration - Deaths 2006 ICD10 as it appears in World Health Organization (WHO). WHO Mortality Database Version October 2017. Geneva, Switzerland: World Health Organization (WHO), 2017
Saint Lucia Vital Registration - Deaths 2008 ICD10 as it appears in World Health Organization (WHO). WHO Mortality Database Version October 2017. Geneva, Switzerland: World Health Organization (WHO), 2017
Saint Lucia Vital Registration - Deaths 2009 ICD10 as it appears in World Health Organization (WHO). WHO Mortality Database Version October 2017. Geneva, Switzerland: World Health Organization (WHO), 2017
Saint Lucia Vital Registration - Deaths 2010 ICD10 as it appears in World Health Organization (WHO). WHO Mortality Database Version October 2017. Geneva, Switzerland: World Health Organization (WHO), 2017
Saint Lucia Vital Registration - Deaths 2011 ICD10 as it appears in World Health Organization (WHO). WHO Mortality Database Version October 2017. Geneva, Switzerland: World Health Organization (WHO), 2017

Saint Lucia Vital Registration - Deaths 2012 ICD10 as it appears in World Health Organization (WHO). WHO Mortality Database Version October 2017. Geneva, Switzerland: World Health Organization (WHO), 2017

Saint Lucia Vital Registration - Deaths 2013 ICD10 as it appears in World Health Organization (WHO). WHO Mortality Database Version October 2017. Geneva, Switzerland: World Health Organization (WHO), 2017

Saint Lucia Vital Registration - Deaths 2014 ICD10 as it appears in World Health Organization (WHO). WHO Mortality Database Version October 2017. Geneva, Switzerland: World Health Organization (WHO), 2017

Saint Vincent and the Grenadines Vital Registration - Deaths 1982 ICD9 as it appears in World Health Organization (WHO). WHO Mortality Database Version September 2016. Geneva, Switzerland: World Health Organization (WHO), 2016

Saint Vincent and the Grenadines Vital Registration - Deaths 1983 ICD9 as it appears in World Health Organization (WHO). WHO Mortality Database Version September 2016. Geneva, Switzerland: World Health Organization (WHO), 2016

Saint Vincent and the Grenadines Vital Registration - Deaths 1984 ICD9 as it appears in World Health Organization (WHO). WHO Mortality Database Version September 2016. Geneva, Switzerland: World Health Organization (WHO), 2016

Saint Vincent and the Grenadines Vital Registration - Deaths 1985 ICD9 as it appears in World Health Organization (WHO). WHO Mortality Database Version September 2016. Geneva, Switzerland: World Health Organization (WHO), 2016

Saint Vincent and the Grenadines Vital Registration - Deaths 1986 ICD9 as it appears in World Health Organization (WHO). WHO Mortality Database Version September 2016. Geneva, Switzerland: World Health Organization (WHO), 2016

Saint Vincent and the Grenadines Vital Registration - Deaths 1987 ICD9 as it appears in World Health Organization (WHO). WHO Mortality Database Version September 2016. Geneva, Switzerland: World Health Organization (WHO), 2016

Saint Vincent and the Grenadines Vital Registration - Deaths 1990 ICD9 as it appears in World Health Organization (WHO). WHO Mortality Database Version November 2015. Geneva, Switzerland: World Health Organization (WHO), 2015

Saint Vincent and the Grenadines Vital Registration - Deaths 1995 ICD9 as it appears in World Health Organization (WHO). WHO Mortality Database Version September 2016. Geneva, Switzerland: World Health Organization (WHO), 2016

Saint Vincent and the Grenadines Vital Registration - Deaths 1996 ICD9 as it appears in World Health Organization (WHO). WHO Mortality Database Version September 2016. Geneva, Switzerland: World Health Organization (WHO), 2016

Saint Vincent and the Grenadines Vital Registration - Deaths 1997 ICD9 as it appears in World Health Organization (WHO). WHO Mortality Database Version September 2016. Geneva, Switzerland: World Health Organization (WHO), 2016

Saint Vincent and the Grenadines Vital Registration - Deaths 1998 ICD9 as it appears in World Health Organization (WHO). WHO Mortality Database Version September 2016. Geneva, Switzerland: World Health Organization (WHO), 2016

Saint Vincent and the Grenadines Vital Registration - Deaths 1999 ICD9 as it appears in World Health Organization (WHO). WHO Mortality Database Version September 2016. Geneva, Switzerland: World Health Organization (WHO), 2016

Saint Vincent and the Grenadines Vital Registration - Deaths 2000 ICD10 as it appears in World Health Organization (WHO). WHO Mortality Database Version October 2017. Geneva, Switzerland: World Health Organization (WHO), 2017

Saint Vincent and the Grenadines Vital Registration - Deaths 2001 ICD10 as it appears in World Health Organization (WHO). WHO Mortality Database Version October 2017. Geneva, Switzerland: World Health Organization (WHO), 2017

Saint Vincent and the Grenadines Vital Registration - Deaths 2002 ICD10 as it appears in World Health Organization (WHO). WHO Mortality Database Version October 2017. Geneva, Switzerland: World Health Organization (WHO), 2017

Saint Vincent and the Grenadines Vital Registration - Deaths 2003 ICD10 as it appears in World Health Organization (WHO). WHO Mortality Database Version October 2017. Geneva, Switzerland: World Health Organization (WHO), 2017

Saint Vincent and the Grenadines Vital Registration - Deaths 2004 ICD10 as it appears in World Health Organization (WHO). WHO Mortality Database Version October 2017. Geneva, Switzerland: World Health Organization (WHO), 2017

Saint Vincent and the Grenadines Vital Registration - Deaths 2005 ICD10 as it appears in World Health Organization (WHO). WHO Mortality Database Version October 2017. Geneva, Switzerland: World Health Organization (WHO), 2017

Saint Vincent and the Grenadines Vital Registration - Deaths 2006 ICD10 as it appears in World Health Organization (WHO). WHO Mortality Database Version October 2017. Geneva, Switzerland: World Health Organization (WHO), 2017

Saint Vincent and the Grenadines Vital Registration - Deaths 2007 ICD10 as it appears in World Health Organization (WHO). WHO Mortality Database Version October 2017. Geneva, Switzerland: World Health Organization (WHO), 2017

Saint Vincent and the Grenadines Vital Registration - Deaths 2008 ICD10 as it appears in World Health Organization (WHO). WHO Mortality Database Version October 2017. Geneva, Switzerland: World Health Organization (WHO), 2017

Saint Vincent and the Grenadines Vital Registration - Deaths 2009 ICD10 as it appears in World Health Organization (WHO). WHO Mortality Database Version October 2017. Geneva, Switzerland: World Health Organization (WHO), 2017

Saint Vincent and the Grenadines Vital Registration - Deaths 2010 ICD10 as it appears in World Health Organization (WHO). WHO Mortality Database Version October 2017. Geneva, Switzerland: World Health Organization (WHO), 2017

Saint Vincent and the Grenadines Vital Registration - Deaths 2011 ICD10 as it appears in World Health Organization (WHO). WHO Mortality Database Version October 2017. Geneva, Switzerland: World Health Organization (WHO), 2017

Saint Vincent and the Grenadines Vital Registration - Deaths 2012 ICD10 as it appears in World Health Organization (WHO). WHO Mortality Database Version October 2017. Geneva, Switzerland: World Health Organization (WHO), 2017

Saint Vincent and the Grenadines Vital Registration - Deaths 2013 ICD10 as it appears in World Health Organization (WHO). WHO Mortality Database Version October 2017. Geneva, Switzerland: World Health Organization (WHO), 2017

Saint Vincent and the Grenadines Vital Registration - Deaths 2014 ICD10 as it appears in World Health Organization (WHO). WHO Mortality Database Version October 2017. Geneva, Switzerland: World Health Organization (WHO), 2017

Saint Vincent and the Grenadines Vital Registration - Deaths 2015 ICD10 as it appears in World Health Organization (WHO). WHO Mortality Database Version October 2017. Geneva, Switzerland: World Health Organization (WHO), 2017

Samms-Vaughan ME, McCaw-Binns AM, Ashley DC, Foster-Williams K. Neonatal mortality determinants in Jamaica. J Trop Pediatr. 1990; 36(4): 171-5

Sao Tome and Principe Vital Registration - Deaths 1985 ICD9 as it appears in World Health Organization (WHO). WHO Mortality Database Version September 2016. Geneva, Switzerland: World Health Organization (WHO), 2016

Serbia and Montenegro - Montenegro Vital Registration - Deaths 2003 ICD10 as it appears in World Health Organization (WHO). WHO Mortality Database Version October 2017. Geneva, Switzerland: World Health Organization (WHO), 2017

Serbia and Montenegro - Montenegro Vital Registration - Deaths 2004 ICD10 as it appears in World Health Organization (WHO). WHO Mortality Database Version October 2017. Geneva, Switzerland: World Health Organization (WHO), 2017

Serbia and Montenegro - Serbia Vital Registration - Deaths 2003 ICD10 as it appears in World Health Organization (WHO). WHO Mortality Database Version October 2017. Geneva, Switzerland: World Health Organization (WHO), 2017

Serbia and Montenegro - Serbia Vital Registration - Deaths 2004 ICD10 as it appears in World Health Organization (WHO). WHO Mortality Database Version October 2017. Geneva, Switzerland: World Health Organization (WHO), 2017
Serbia and Montenegro - Serbia Vital Registration - Deaths 2005 ICD10 as it appears in World Health Organization (WHO). WHO Mortality Database Version October 2017. Geneva, Switzerland: World Health Organization (WHO), 2017
Serbia and Montenegro - Serbia Vital Registration - Deaths 2006 ICD10 as it appears in World Health Organization (WHO). WHO Mortality Database Version October 2017. Geneva, Switzerland: World Health Organization (WHO), 2017
Serbia Vital Registration - Deaths 2007 ICD10 as it appears in World Health Organization (WHO). WHO Mortality Database Version October 2017. Geneva, Switzerland: World Health Organization (WHO), 2017
Serbia Vital Registration - Deaths 2008 ICD10 as it appears in World Health Organization (WHO). WHO Mortality Database Version October 2017. Geneva, Switzerland: World Health Organization (WHO), 2017
Serbia Vital Registration - Deaths 2009 ICD10 as it appears in World Health Organization (WHO). WHO Mortality Database Version October 2017. Geneva, Switzerland: World Health Organization (WHO), 2017
Serbia Vital Registration - Deaths 2010 ICD10 as it appears in World Health Organization (WHO). WHO Mortality Database Version October 2017. Geneva, Switzerland: World Health Organization (WHO), 2017
Serbia Vital Registration - Deaths 2011 ICD10 as it appears in World Health Organization (WHO). WHO Mortality Database Version October 2017. Geneva, Switzerland: World Health Organization (WHO), 2017
Serbia Vital Registration - Deaths 2012 ICD10 as it appears in World Health Organization (WHO). WHO Mortality Database Version October 2017. Geneva, Switzerland: World Health Organization (WHO), 2017
Serbia Vital Registration - Deaths 2013 ICD10 as it appears in World Health Organization (WHO). WHO Mortality Database Version October 2017. Geneva, Switzerland: World Health Organization (WHO), 2017
Serbia Vital Registration - Deaths 2014 ICD10 as it appears in World Health Organization (WHO). WHO Mortality Database Version October 2017. Geneva, Switzerland: World Health Organization (WHO), 2017
Serbia Vital Registration - Deaths 2015 ICD10 as it appears in World Health Organization (WHO). WHO Mortality Database Version March 2017. Geneva, Switzerland: World Health Organization (WHO), 2017
Seychelles Vital Registration - Deaths 2008 ICD10 as it appears in World Health Organization (WHO). WHO Mortality Database Version November 2015. Geneva, Switzerland: World Health Organization (WHO), 2015
Seychelles Vital Registration - Deaths 1981 ICD9 as it appears in World Health Organization (WHO). WHO Mortality Database Version September 2016. Geneva, Switzerland: World Health Organization (WHO), 2016
Seychelles Vital Registration - Deaths 1982 ICD9 as it appears in World Health Organization (WHO). WHO Mortality Database Version September 2016. Geneva, Switzerland: World Health Organization (WHO), 2016
Seychelles Vital Registration - Deaths 1985 ICD9 as it appears in World Health Organization (WHO). WHO Mortality Database Version September 2016. Geneva, Switzerland: World Health Organization (WHO), 2016
Seychelles Vital Registration - Deaths 1986 ICD9 as it appears in World Health Organization (WHO). WHO Mortality Database Version September 2016. Geneva, Switzerland: World Health Organization (WHO), 2016
Seychelles Vital Registration - Deaths 1987 ICD9 as it appears in World Health Organization (WHO). WHO Mortality Database Version September 2016. Geneva, Switzerland: World Health Organization (WHO), 2016

Seychelles Vital Registration - Deaths 2001 ICD10 as it appears in World Health Organization (WHO). WHO Mortality Database Version November 2015. Geneva, Switzerland: World Health Organization (WHO), 2015

Seychelles Vital Registration - Deaths 2002 ICD10 as it appears in World Health Organization (WHO). WHO Mortality Database Version November 2015. Geneva, Switzerland: World Health Organization (WHO), 2015

Seychelles Vital Registration - Deaths 2003 ICD10 as it appears in World Health Organization (WHO). WHO Mortality Database Version November 2015. Geneva, Switzerland: World Health Organization (WHO), 2015

Seychelles Vital Registration - Deaths 2004 ICD10 as it appears in World Health Organization (WHO). WHO Mortality Database Version November 2015. Geneva, Switzerland: World Health Organization (WHO), 2015

Seychelles Vital Registration - Deaths 2005 ICD10 as it appears in World Health Organization (WHO). WHO Mortality Database Version November 2015. Geneva, Switzerland: World Health Organization (WHO), 2015

Seychelles Vital Registration - Deaths 2006 ICD10 as it appears in World Health Organization (WHO). WHO Mortality Database Version November 2015. Geneva, Switzerland: World Health Organization (WHO), 2015

Seychelles Vital Registration - Deaths 2007 ICD10 as it appears in World Health Organization (WHO). WHO Mortality Database Version November 2015. Geneva, Switzerland: World Health Organization (WHO), 2015

Seychelles Vital Registration - Deaths 2009 ICD10 as it appears in World Health Organization (WHO). WHO Mortality Database Version November 2015. Geneva, Switzerland: World Health Organization (WHO), 2015

Seychelles Vital Registration - Deaths 2010 ICD10 as it appears in World Health Organization (WHO). WHO Mortality Database Version November 2015. Geneva, Switzerland: World Health Organization (WHO), 2015

Seychelles Vital Registration - Deaths 2011 ICD10 as it appears in World Health Organization (WHO). WHO Mortality Database Version November 2015. Geneva, Switzerland: World Health Organization (WHO), 2015

Seychelles Vital Registration - Deaths 2012 ICD10 as it appears in World Health Organization (WHO). WHO Mortality Database Version November 2015. Geneva, Switzerland: World Health Organization (WHO), 2015

Seychelles Vital Registration - Deaths 2013 ICD10 as it appears in World Health Organization (WHO). WHO Mortality Database Version November 2015. Geneva, Switzerland: World Health Organization (WHO), 2015

Seychelles Vital Registration - Deaths 2014 ICD10 as it appears in World Health Organization (WHO). WHO Mortality Database Version November 2015. Geneva, Switzerland: World Health Organization (WHO), 2015

Seychelles Vital Registration - Deaths 2015 ICD10 as it appears in World Health Organization (WHO). WHO Mortality Database Version April 2018. Geneva, Switzerland: World Health Organization (WHO), 2018

Shah BD, Dwivedi LK. Causes of neonatal deaths among tribal women in Gujarat, India. Popul Res Policy Rev. 2011; 30(4): 517-36

Shah MS, Khalique N, Khan Z. Determinants of childhood mortality. Indian J Prev Soc Med. 2011; 42(2): 118-22

Sharifzadeh G, Namakin K, Mehrjoofard H. An Epidemiological Study on Infant Mortality and Factors Affecting it in Rural Areas of Birjand, Iran. Iran J Pediatr. 2008; 18(4): 335-42

Shikha B, Harsh S, Narayan G. Infant deaths' audit: Contextual factors contributing to Infant deaths in tribal district-Valsad, Gujarat (India). J Res Med Den Sci. 2015; 3(3): 171-5

Singhal PK, Mathur GP, Mathur S, Singh YD. Neonatal morbidity and mortality in ICDS Urban Slums. Indian Pediatr. 1990; 27(5): 485-8

Slovakia Vital Registration - Deaths 1992 ICD9 as it appears in World Health Organization (WHO). WHO Mortality Database Version November 2015. Geneva, Switzerland: World Health Organization (WHO), 2015
Slovakia Vital Registration - Deaths 1993 ICD9 as it appears in World Health Organization (WHO). WHO Mortality Database Version November 2015. Geneva, Switzerland: World Health Organization (WHO), 2015
Slovakia Vital Registration - Deaths 1994 ICD10 as it appears in World Health Organization (WHO). WHO Mortality Database Version October 2017. Geneva, Switzerland: World Health Organization (WHO), 2017
Slovakia Vital Registration - Deaths 1995 ICD10 as it appears in World Health Organization (WHO). WHO Mortality Database Version October 2017. Geneva, Switzerland: World Health Organization (WHO), 2017
Slovakia Vital Registration - Deaths 1996 ICD10 as it appears in World Health Organization (WHO). WHO Mortality Database Version October 2017. Geneva, Switzerland: World Health Organization (WHO), 2017
Slovakia Vital Registration - Deaths 1997 ICD10 as it appears in World Health Organization (WHO). WHO Mortality Database Version October 2017. Geneva, Switzerland: World Health Organization (WHO), 2017
Slovakia Vital Registration - Deaths 1998 ICD10 as it appears in World Health Organization (WHO). WHO Mortality Database Version October 2017. Geneva, Switzerland: World Health Organization (WHO), 2017
Slovakia Vital Registration - Deaths 1999 ICD10 as it appears in World Health Organization (WHO). WHO Mortality Database Version October 2017. Geneva, Switzerland: World Health Organization (WHO), 2017
Slovakia Vital Registration - Deaths 2000 ICD10 as it appears in World Health Organization (WHO). WHO Mortality Database Version October 2017. Geneva, Switzerland: World Health Organization (WHO), 2017
Slovakia Vital Registration - Deaths 2001 ICD10 as it appears in World Health Organization (WHO). WHO Mortality Database Version October 2017. Geneva, Switzerland: World Health Organization (WHO), 2017
Slovakia Vital Registration - Deaths 2002 ICD10 as it appears in World Health Organization (WHO). WHO Mortality Database Version October 2017. Geneva, Switzerland: World Health Organization (WHO), 2017
Slovakia Vital Registration - Deaths 2003 ICD10 as it appears in World Health Organization (WHO). WHO Mortality Database Version October 2017. Geneva, Switzerland: World Health Organization (WHO), 2017
Slovakia Vital Registration - Deaths 2004 ICD10 as it appears in World Health Organization (WHO). WHO Mortality Database Version October 2017. Geneva, Switzerland: World Health Organization (WHO), 2017
Slovakia Vital Registration - Deaths 2005 ICD10 as it appears in World Health Organization (WHO). WHO Mortality Database Version October 2017. Geneva, Switzerland: World Health Organization (WHO), 2017
Slovakia Vital Registration - Deaths 2006 ICD10 as it appears in World Health Organization (WHO). WHO Mortality Database Version October 2017. Geneva, Switzerland: World Health Organization (WHO), 2017
Slovakia Vital Registration - Deaths 2007 ICD10 as it appears in World Health Organization (WHO). WHO Mortality Database Version October 2017. Geneva, Switzerland: World Health Organization (WHO), 2017
Slovakia Vital Registration - Deaths 2008 ICD10 as it appears in World Health Organization (WHO). WHO Mortality Database Version October 2017. Geneva, Switzerland: World Health Organization (WHO), 2017
Slovakia Vital Registration - Deaths 2009 ICD10 as it appears in World Health Organization (WHO). WHO Mortality Database Version October 2017. Geneva, Switzerland: World Health Organization (WHO), 2017

Slovakia Vital Registration - Deaths 2010 ICD10 as it appears in World Health Organization (WHO). WHO Mortality Database Version October 2017. Geneva, Switzerland: World Health Organization (WHO), 2017

Slovakia Vital Registration - Deaths 2012 ICD10 as it appears in World Health Organization (WHO). WHO Mortality Database Version October 2017. Geneva, Switzerland: World Health Organization (WHO), 2017

Slovakia Vital Registration - Deaths 2013 ICD10 as it appears in World Health Organization (WHO). WHO Mortality Database Version October 2017. Geneva, Switzerland: World Health Organization (WHO), 2017

Slovakia Vital Registration - Deaths 2014 ICD10 as it appears in World Health Organization (WHO). WHO Mortality Database Version October 2017. Geneva, Switzerland: World Health Organization (WHO), 2017

Slovenia Vital Registration - Deaths 1989 ICD9 as it appears in World Health Organization (WHO). WHO Mortality Database Version November 2015. Geneva, Switzerland: World Health Organization (WHO), 2015

Slovenia Vital Registration - Deaths 1991 ICD9 as it appears in World Health Organization (WHO). WHO Mortality Database Version November 2015. Geneva, Switzerland: World Health Organization (WHO), 2015

Slovenia Vital Registration - Deaths 1992 ICD9 as it appears in World Health Organization (WHO). WHO Mortality Database Version November 2015. Geneva, Switzerland: World Health Organization (WHO), 2015

Slovenia Vital Registration - Deaths 1993 ICD9 as it appears in World Health Organization (WHO). WHO Mortality Database Version November 2015. Geneva, Switzerland: World Health Organization (WHO), 2015

Slovenia Vital Registration - Deaths 1994 ICD9 as it appears in World Health Organization (WHO). WHO Mortality Database Version November 2015. Geneva, Switzerland: World Health Organization (WHO), 2015

Slovenia Vital Registration - Deaths 1995 ICD9 as it appears in World Health Organization (WHO). WHO Mortality Database Version November 2015. Geneva, Switzerland: World Health Organization (WHO), 2015

Slovenia Vital Registration - Deaths 1996 ICD9 as it appears in World Health Organization (WHO). WHO Mortality Database Version November 2015. Geneva, Switzerland: World Health Organization (WHO), 2015

Slovenia Vital Registration - Deaths 1997 ICD10 as it appears in World Health Organization (WHO). WHO Mortality Database Version October 2017. Geneva, Switzerland: World Health Organization (WHO), 2017

Slovenia Vital Registration - Deaths 1998 ICD10 as it appears in World Health Organization (WHO). WHO Mortality Database Version October 2017. Geneva, Switzerland: World Health Organization (WHO), 2017

Slovenia Vital Registration - Deaths 1999 ICD10 as it appears in World Health Organization (WHO). WHO Mortality Database Version October 2017. Geneva, Switzerland: World Health Organization (WHO), 2017

Slovenia Vital Registration - Deaths 2000 ICD10 as it appears in World Health Organization (WHO). WHO Mortality Database Version October 2017. Geneva, Switzerland: World Health Organization (WHO), 2017

Slovenia Vital Registration - Deaths 2001 ICD10 as it appears in World Health Organization (WHO). WHO Mortality Database Version October 2017. Geneva, Switzerland: World Health Organization (WHO), 2017

Slovenia Vital Registration - Deaths 2002 ICD10 as it appears in World Health Organization (WHO). WHO Mortality Database Version October 2017. Geneva, Switzerland: World Health Organization (WHO), 2017

Slovenia Vital Registration - Deaths 2003 ICD10 as it appears in World Health Organization (WHO). WHO Mortality Database Version October 2017. Geneva, Switzerland: World Health Organization (WHO), 2017

Slovenia Vital Registration - Deaths 2004 ICD10 as it appears in World Health Organization (WHO). WHO Mortality Database Version October 2017. Geneva, Switzerland: World Health Organization (WHO), 2017

Slovenia Vital Registration - Deaths 2005 ICD10 as it appears in World Health Organization (WHO). WHO Mortality Database Version October 2017. Geneva, Switzerland: World Health Organization (WHO), 2017

Slovenia Vital Registration - Deaths 2006 ICD10 as it appears in World Health Organization (WHO). WHO Mortality Database Version October 2017. Geneva, Switzerland: World Health Organization (WHO), 2017

Slovenia Vital Registration - Deaths 2007 ICD10 as it appears in World Health Organization (WHO). WHO Mortality Database Version October 2017. Geneva, Switzerland: World Health Organization (WHO), 2017

Slovenia Vital Registration - Deaths 2008 ICD10 as it appears in World Health Organization (WHO). WHO Mortality Database Version October 2017. Geneva, Switzerland: World Health Organization (WHO), 2017

Slovenia Vital Registration - Deaths 2009 ICD10 as it appears in World Health Organization (WHO). WHO Mortality Database Version October 2017. Geneva, Switzerland: World Health Organization (WHO), 2017

Slovenia Vital Registration - Deaths 2010 ICD10 as it appears in World Health Organization (WHO). WHO Mortality Database Version October 2017. Geneva, Switzerland: World Health Organization (WHO), 2017

Slovenia Vital Registration - Deaths 2011 ICD10 as it appears in World Health Organization (WHO). WHO Mortality Database Version March 2017. Geneva, Switzerland: World Health Organization (WHO), 2017

Slovenia Vital Registration - Deaths 2012 ICD10 as it appears in World Health Organization (WHO). WHO Mortality Database Version March 2017. Geneva, Switzerland: World Health Organization (WHO), 2017

Slovenia Vital Registration - Deaths 2013 ICD10 as it appears in World Health Organization (WHO). WHO Mortality Database Version March 2017. Geneva, Switzerland: World Health Organization (WHO), 2017

Slovenia Vital Registration - Deaths 2014 ICD10 as it appears in World Health Organization (WHO). WHO Mortality Database Version March 2017. Geneva, Switzerland: World Health Organization (WHO), 2017

Slovenia Vital Registration - Deaths 2015 ICD10 as it appears in World Health Organization (WHO). WHO Mortality Database Version March 2017. Geneva, Switzerland: World Health Organization (WHO), 2017

South Korea Vital Registration - Deaths 1986 ICD9 as it appears in World Health Organization (WHO). WHO Mortality Database Version September 2016. Geneva, Switzerland: World Health Organization (WHO), 2016

South Korea Vital Registration - Deaths 1987 ICD9 as it appears in World Health Organization (WHO). WHO Mortality Database Version September 2016. Geneva, Switzerland: World Health Organization (WHO), 2016

South Korea Vital Registration - Deaths 1988 ICD9 as it appears in World Health Organization (WHO). WHO Mortality Database Version September 2016. Geneva, Switzerland: World Health Organization (WHO), 2016

South Korea Vital Registration - Deaths 1989 ICD9 as it appears in World Health Organization (WHO). WHO Mortality Database Version September 2016. Geneva, Switzerland: World Health Organization (WHO), 2016

South Korea Vital Registration - Deaths 1990 ICD9 as it appears in World Health Organization (WHO). WHO Mortality Database Version September 2016. Geneva, Switzerland: World Health Organization (WHO), 2016

South Korea Vital Registration - Deaths 1994 ICD9 as it appears in World Health Organization (WHO). WHO Mortality Database Version September 2016. Geneva, Switzerland: World Health Organization (WHO), 2016

South Korea Vital Registration - Deaths 1995 ICD10 as it appears in World Health Organization (WHO). WHO Mortality Database Version October 2017. Geneva, Switzerland: World Health Organization (WHO), 2017
South Korea Vital Registration - Deaths 1996 ICD10 as it appears in World Health Organization (WHO). WHO Mortality Database Version October 2017. Geneva, Switzerland: World Health Organization (WHO), 2017
South Korea Vital Registration - Deaths 1997 ICD10 as it appears in World Health Organization (WHO). WHO Mortality Database Version October 2017. Geneva, Switzerland: World Health Organization (WHO), 2017
South Korea Vital Registration - Deaths 1998 ICD10 as it appears in World Health Organization (WHO). WHO Mortality Database Version October 2017. Geneva, Switzerland: World Health Organization (WHO), 2017
South Korea Vital Registration - Deaths 1999 ICD10 as it appears in World Health Organization (WHO). WHO Mortality Database Version October 2017. Geneva, Switzerland: World Health Organization (WHO), 2017
South Korea Vital Registration - Deaths 1985 ICD9 as it appears in World Health Organization (WHO). WHO Mortality Database Version September 2016. Geneva, Switzerland: World Health Organization (WHO), 2016
South Korea Vital Registration - Deaths 1991 ICD9 as it appears in World Health Organization (WHO). WHO Mortality Database Version September 2016. Geneva, Switzerland: World Health Organization (WHO), 2016
South Korea Vital Registration - Deaths 1992 ICD9 as it appears in World Health Organization (WHO). WHO Mortality Database Version September 2016. Geneva, Switzerland: World Health Organization (WHO), 2016
South Korea Vital Registration - Deaths 1993 ICD9 as it appears in World Health Organization (WHO). WHO Mortality Database Version September 2016. Geneva, Switzerland: World Health Organization (WHO), 2016
South Korea Vital Registration - Deaths 2000 ICD10 as it appears in World Health Organization (WHO). WHO Mortality Database Version October 2017. Geneva, Switzerland: World Health Organization (WHO), 2017
South Korea Vital Registration - Deaths 2001 ICD10 as it appears in World Health Organization (WHO). WHO Mortality Database Version October 2017. Geneva, Switzerland: World Health Organization (WHO), 2017
South Korea Vital Registration - Deaths 2002 ICD10 as it appears in World Health Organization (WHO). WHO Mortality Database Version October 2017. Geneva, Switzerland: World Health Organization (WHO), 2017
South Korea Vital Registration - Deaths 2003 ICD10 as it appears in World Health Organization (WHO). WHO Mortality Database Version October 2017. Geneva, Switzerland: World Health Organization (WHO), 2017
South Korea Vital Registration - Deaths 2004 ICD10 as it appears in World Health Organization (WHO). WHO Mortality Database Version October 2017. Geneva, Switzerland: World Health Organization (WHO), 2017
South Korea Vital Registration - Deaths 2005 ICD10 as it appears in World Health Organization (WHO). WHO Mortality Database Version October 2017. Geneva, Switzerland: World Health Organization (WHO), 2017
South Korea Vital Registration - Deaths 2006 ICD10 as it appears in World Health Organization (WHO). WHO Mortality Database Version October 2017. Geneva, Switzerland: World Health Organization (WHO), 2017
South Korea Vital Registration - Deaths 2007 ICD10 as it appears in World Health Organization (WHO). WHO Mortality Database Version October 2017. Geneva, Switzerland: World Health Organization (WHO), 2017
South Korea Vital Registration - Deaths 2008 ICD10 as it appears in World Health Organization (WHO). WHO Mortality Database Version October 2017. Geneva, Switzerland: World Health Organization (WHO), 2017

South Korea Vital Registration - Deaths 2009 ICD10 as it appears in World Health Organization (WHO). WHO Mortality Database Version October 2017. Geneva, Switzerland: World Health Organization (WHO), 2017
South Korea Vital Registration - Deaths 2010 ICD10 as it appears in World Health Organization (WHO). WHO Mortality Database Version October 2017. Geneva, Switzerland: World Health Organization (WHO), 2017
South Korea Vital Registration - Deaths 2011 ICD10 as it appears in World Health Organization (WHO). WHO Mortality Database Version October 2017. Geneva, Switzerland: World Health Organization (WHO), 2017
South Korea Vital Registration - Deaths 2012 ICD10 as it appears in World Health Organization (WHO). WHO Mortality Database Version October 2017. Geneva, Switzerland: World Health Organization (WHO), 2017
South Korea Vital Registration - Deaths 2013 ICD10 as it appears in World Health Organization (WHO). WHO Mortality Database Version October 2017. Geneva, Switzerland: World Health Organization (WHO), 2017
South Korea Vital Registration - Deaths 2014 ICD10 as it appears in World Health Organization (WHO). WHO Mortality Database Version October 2017. Geneva, Switzerland: World Health Organization (WHO), 2017
South Korea Vital Registration - Deaths 2015 ICD10 as it appears in World Health Organization (WHO). WHO Mortality Database Version October 2017. Geneva, Switzerland: World Health Organization (WHO), 2017
Spain Vital Registration - Deaths 1980 ICD9 as it appears in World Health Organization (WHO). WHO Mortality Database Version November 2015. Geneva, Switzerland: World Health Organization (WHO), 2015
Spain Vital Registration - Deaths 1981 ICD9 as it appears in World Health Organization (WHO). WHO Mortality Database Version November 2015. Geneva, Switzerland: World Health Organization (WHO), 2015
Spain Vital Registration - Deaths 1982 ICD9 as it appears in World Health Organization (WHO). WHO Mortality Database Version November 2015. Geneva, Switzerland: World Health Organization (WHO), 2015
Spain Vital Registration - Deaths 1983 ICD9 as it appears in World Health Organization (WHO). WHO Mortality Database Version November 2015. Geneva, Switzerland: World Health Organization (WHO), 2015
Spain Vital Registration - Deaths 1984 ICD9 as it appears in World Health Organization (WHO). WHO Mortality Database Version November 2015. Geneva, Switzerland: World Health Organization (WHO), 2015
Spain Vital Registration - Deaths 1985 ICD9 as it appears in World Health Organization (WHO). WHO Mortality Database Version November 2015. Geneva, Switzerland: World Health Organization (WHO), 2015
Spain Vital Registration - Deaths 1986 ICD9 as it appears in World Health Organization (WHO). WHO Mortality Database Version November 2015. Geneva, Switzerland: World Health Organization (WHO), 2015
Spain Vital Registration - Deaths 1987 ICD9 as it appears in World Health Organization (WHO). WHO Mortality Database Version November 2015. Geneva, Switzerland: World Health Organization (WHO), 2015
Spain Vital Registration - Deaths 1988 ICD9 as it appears in World Health Organization (WHO). WHO Mortality Database Version November 2015. Geneva, Switzerland: World Health Organization (WHO), 2015
Spain Vital Registration - Deaths 1989 ICD9 as it appears in World Health Organization (WHO). WHO Mortality Database Version November 2015. Geneva, Switzerland: World Health Organization (WHO), 2015
Spain Vital Registration - Deaths 1990 ICD9 as it appears in World Health Organization (WHO). WHO Mortality Database Version November 2015. Geneva, Switzerland: World Health Organization (WHO), 2015

Spain Vital Registration - Deaths 1991 ICD9 as it appears in World Health Organization (WHO). WHO Mortality Database Version November 2015. Geneva, Switzerland: World Health Organization (WHO), 2015
Spain Vital Registration - Deaths 1992 ICD9 as it appears in World Health Organization (WHO). WHO Mortality Database Version November 2015. Geneva, Switzerland: World Health Organization (WHO), 2015
Spain Vital Registration - Deaths 1993 ICD9 as it appears in World Health Organization (WHO). WHO Mortality Database Version November 2015. Geneva, Switzerland: World Health Organization (WHO), 2015
Spain Vital Registration - Deaths 1994 ICD9 as it appears in World Health Organization (WHO). WHO Mortality Database Version November 2015. Geneva, Switzerland: World Health Organization (WHO), 2015
Spain Vital Registration - Deaths 1995 ICD9 as it appears in World Health Organization (WHO). WHO Mortality Database Version November 2015. Geneva, Switzerland: World Health Organization (WHO), 2015
Spain Vital Registration - Deaths 1996 ICD9 as it appears in World Health Organization (WHO). WHO Mortality Database Version November 2015. Geneva, Switzerland: World Health Organization (WHO), 2015
Spain Vital Registration - Deaths 1997 ICD9 as it appears in World Health Organization (WHO). WHO Mortality Database Version November 2015. Geneva, Switzerland: World Health Organization (WHO), 2015
Spain Vital Registration - Deaths 1998 ICD9 as it appears in World Health Organization (WHO). WHO Mortality Database Version November 2015. Geneva, Switzerland: World Health Organization (WHO), 2015
Spain Vital Registration - Deaths 1999 ICD10 as it appears in World Health Organization (WHO). WHO Mortality Database Version October 2017. Geneva, Switzerland: World Health Organization (WHO), 2017
Spain Vital Registration - Deaths 2000 ICD10 as it appears in World Health Organization (WHO). WHO Mortality Database Version October 2017. Geneva, Switzerland: World Health Organization (WHO), 2017
Spain Vital Registration - Deaths 2001 ICD10 as it appears in World Health Organization (WHO). WHO Mortality Database Version October 2017. Geneva, Switzerland: World Health Organization (WHO), 2017
Spain Vital Registration - Deaths 2002 ICD10 as it appears in World Health Organization (WHO). WHO Mortality Database Version October 2017. Geneva, Switzerland: World Health Organization (WHO), 2017
Spain Vital Registration - Deaths 2003 ICD10 as it appears in World Health Organization (WHO). WHO Mortality Database Version October 2017. Geneva, Switzerland: World Health Organization (WHO), 2017
Spain Vital Registration - Deaths 2004 ICD10 as it appears in World Health Organization (WHO). WHO Mortality Database Version October 2017. Geneva, Switzerland: World Health Organization (WHO), 2017
Spain Vital Registration - Deaths 2005 ICD10 as it appears in World Health Organization (WHO). WHO Mortality Database Version October 2017. Geneva, Switzerland: World Health Organization (WHO), 2017
Spain Vital Registration - Deaths 2006 ICD10 as it appears in World Health Organization (WHO). WHO Mortality Database Version October 2017. Geneva, Switzerland: World Health Organization (WHO), 2017
Spain Vital Registration - Deaths 2007 ICD10 as it appears in World Health Organization (WHO). WHO Mortality Database Version October 2017. Geneva, Switzerland: World Health Organization (WHO), 2017
Spain Vital Registration - Deaths 2008 ICD10 as it appears in World Health Organization (WHO). WHO Mortality Database Version October 2017. Geneva, Switzerland: World Health Organization (WHO), 2017

Spain Vital Registration - Deaths 2009 ICD10 as it appears in World Health Organization (WHO). WHO Mortality Database Version October 2017. Geneva, Switzerland: World Health Organization (WHO), 2017
Spain Vital Registration - Deaths 2010 ICD10 as it appears in World Health Organization (WHO). WHO Mortality Database Version October 2017. Geneva, Switzerland: World Health Organization (WHO), 2017
Spain Vital Registration - Deaths 2011 ICD10 as it appears in World Health Organization (WHO). WHO Mortality Database Version October 2017. Geneva, Switzerland: World Health Organization (WHO), 2017
Spain Vital Registration - Deaths 2012 ICD10 as it appears in World Health Organization (WHO). WHO Mortality Database Version October 2017. Geneva, Switzerland: World Health Organization (WHO), 2017
Spain Vital Registration - Deaths 2013 ICD10 as it appears in World Health Organization (WHO). WHO Mortality Database Version October 2017. Geneva, Switzerland: World Health Organization (WHO), 2017
Spain Vital Registration - Deaths 2014 ICD10 as it appears in World Health Organization (WHO). WHO Mortality Database Version October 2017. Geneva, Switzerland: World Health Organization (WHO), 2017
Spain Vital Registration - Deaths 2015 ICD10 as it appears in World Health Organization (WHO). WHO Mortality Database Version October 2017. Geneva, Switzerland: World Health Organization (WHO), 2017
Sri Lanka Vital Registration - Deaths 2006 ICD10 as it appears in World Health Organization (WHO). WHO Mortality Database Version October 2017. Geneva, Switzerland: World Health Organization (WHO), 2017
State Statistics Service (Ukraine). Ukraine Vital Registration - Deaths 2013
State Statistics Service (Ukraine). Ukraine Vital Registration - Deaths 2015
State Statistics Service (Ukraine). Ukraine Vital Registration - Deaths 2016
Statistics Portugal. Portugal Vital Registration - Deaths 1980
Statistics Portugal. Portugal Vital Registration - Deaths 1981
Statistics Portugal. Portugal Vital Registration - Deaths 1982
Statistics Portugal. Portugal Vital Registration - Deaths 1983
Statistics Portugal. Portugal Vital Registration - Deaths 2004
Suriname Vital Registration - Deaths 1980 ICD9 as it appears in World Health Organization (WHO). WHO Mortality Database Version September 2016. Geneva, Switzerland: World Health Organization (WHO), 2016
Suriname Vital Registration - Deaths 1981 ICD9 as it appears in World Health Organization (WHO). WHO Mortality Database Version September 2016. Geneva, Switzerland: World Health Organization (WHO), 2016
Suriname Vital Registration - Deaths 1984 ICD9 as it appears in World Health Organization (WHO). WHO Mortality Database Version September 2016. Geneva, Switzerland: World Health Organization (WHO), 2016
Suriname Vital Registration - Deaths 1986 ICD9 as it appears in World Health Organization (WHO). WHO Mortality Database Version September 2016. Geneva, Switzerland: World Health Organization (WHO), 2016
Suriname Vital Registration - Deaths 1987 ICD9 as it appears in World Health Organization (WHO). WHO Mortality Database Version September 2016. Geneva, Switzerland: World Health Organization (WHO), 2016
Suriname Vital Registration - Deaths 1988 ICD9 as it appears in World Health Organization (WHO). WHO Mortality Database Version September 2016. Geneva, Switzerland: World Health Organization (WHO), 2016

Suriname Vital Registration - Deaths 1989 ICD9 as it appears in World Health Organization (WHO). WHO Mortality Database Version September 2016. Geneva, Switzerland: World Health Organization (WHO), 2016

Suriname Vital Registration - Deaths 1990 ICD9 as it appears in World Health Organization (WHO). WHO Mortality Database Version September 2016. Geneva, Switzerland: World Health Organization (WHO), 2016

Suriname Vital Registration - Deaths 1991 ICD9 as it appears in World Health Organization (WHO). WHO Mortality Database Version September 2016. Geneva, Switzerland: World Health Organization (WHO), 2016

Suriname Vital Registration - Deaths 1992 ICD9 as it appears in World Health Organization (WHO). WHO Mortality Database Version September 2016. Geneva, Switzerland: World Health Organization (WHO), 2016

Suriname Vital Registration - Deaths 1995 ICD10 as it appears in World Health Organization (WHO). WHO Mortality Database Version October 2017. Geneva, Switzerland: World Health Organization (WHO), 2017

Suriname Vital Registration - Deaths 1996 ICD10 as it appears in World Health Organization (WHO). WHO Mortality Database Version October 2017. Geneva, Switzerland: World Health Organization (WHO), 2017

Suriname Vital Registration - Deaths 1997 ICD10 as it appears in World Health Organization (WHO). WHO Mortality Database Version October 2017. Geneva, Switzerland: World Health Organization (WHO), 2017

Suriname Vital Registration - Deaths 1998 ICD10 as it appears in World Health Organization (WHO). WHO Mortality Database Version October 2017. Geneva, Switzerland: World Health Organization (WHO), 2017

Suriname Vital Registration - Deaths 1999 ICD10 as it appears in World Health Organization (WHO). WHO Mortality Database Version October 2017. Geneva, Switzerland: World Health Organization (WHO), 2017

Suriname Vital Registration - Deaths 2000 ICD10 as it appears in World Health Organization (WHO). WHO Mortality Database Version October 2017. Geneva, Switzerland: World Health Organization (WHO), 2017

Suriname Vital Registration - Deaths 2004 ICD10 as it appears in World Health Organization (WHO). WHO Mortality Database Version October 2017. Geneva, Switzerland: World Health Organization (WHO), 2017

Suriname Vital Registration - Deaths 2005 ICD10 as it appears in World Health Organization (WHO). WHO Mortality Database Version October 2017. Geneva, Switzerland: World Health Organization (WHO), 2017

Suriname Vital Registration - Deaths 1982 ICD9 as it appears in World Health Organization (WHO). WHO Mortality Database Version September 2016. Geneva, Switzerland: World Health Organization (WHO), 2016

Suriname Vital Registration - Deaths 1985 ICD9 as it appears in World Health Organization (WHO). WHO Mortality Database Version September 2016. Geneva, Switzerland: World Health Organization (WHO), 2016

Suriname Vital Registration - Deaths 2001 ICD10 as it appears in World Health Organization (WHO). WHO Mortality Database Version October 2017. Geneva, Switzerland: World Health Organization (WHO), 2017

Suriname Vital Registration - Deaths 2002 ICD10 as it appears in World Health Organization (WHO). WHO Mortality Database Version October 2017. Geneva, Switzerland: World Health Organization (WHO), 2017

Suriname Vital Registration - Deaths 2003 ICD10 as it appears in World Health Organization (WHO). WHO Mortality Database Version October 2017. Geneva, Switzerland: World Health Organization (WHO), 2017

Suriname Vital Registration - Deaths 2006 ICD10 as it appears in World Health Organization (WHO). WHO Mortality Database Version October 2017. Geneva, Switzerland: World Health Organization (WHO), 2017

Suriname Vital Registration - Deaths 2008 ICD10 as it appears in World Health Organization (WHO). WHO Mortality Database Version October 2017. Geneva, Switzerland: World Health Organization (WHO), 2017
Suriname Vital Registration - Deaths 2009 ICD10 as it appears in World Health Organization (WHO). WHO Mortality Database Version October 2017. Geneva, Switzerland: World Health Organization (WHO), 2017
Suriname Vital Registration - Deaths 2010 ICD10 as it appears in World Health Organization (WHO). WHO Mortality Database Version October 2017. Geneva, Switzerland: World Health Organization (WHO), 2017
Suriname Vital Registration - Deaths 2011 ICD10 as it appears in World Health Organization (WHO). WHO Mortality Database Version October 2017. Geneva, Switzerland: World Health Organization (WHO), 2017
Suriname Vital Registration - Deaths 2012 ICD10 as it appears in World Health Organization (WHO). WHO Mortality Database Version October 2017. Geneva, Switzerland: World Health Organization (WHO), 2017
Suriname Vital Registration - Deaths 2013 ICD10 as it appears in World Health Organization (WHO). WHO Mortality Database Version October 2017. Geneva, Switzerland: World Health Organization (WHO), 2017
Suriname Vital Registration - Deaths 2014 ICD10 as it appears in World Health Organization (WHO). WHO Mortality Database Version October 2017. Geneva, Switzerland: World Health Organization (WHO), 2017
Switzerland Vital Registration - Deaths 1980 ICD8 as it appears in World Health Organization (WHO). WHO Mortality Database Version July 2012. Geneva, Switzerland: World Health Organization (WHO), 2012
Switzerland Vital Registration - Deaths 1981 ICD8 as it appears in World Health Organization (WHO). WHO Mortality Database Version July 2012. Geneva, Switzerland: World Health Organization (WHO), 2012
Switzerland Vital Registration - Deaths 1982 ICD8 as it appears in World Health Organization (WHO). WHO Mortality Database Version July 2012. Geneva, Switzerland: World Health Organization (WHO), 2012
Switzerland Vital Registration - Deaths 1983 ICD8 as it appears in World Health Organization (WHO). WHO Mortality Database Version July 2012. Geneva, Switzerland: World Health Organization (WHO), 2012
Switzerland Vital Registration - Deaths 1984 ICD8 as it appears in World Health Organization (WHO). WHO Mortality Database Version July 2012. Geneva, Switzerland: World Health Organization (WHO), 2012
Switzerland Vital Registration - Deaths 1985 ICD8 as it appears in World Health Organization (WHO). WHO Mortality Database Version July 2012. Geneva, Switzerland: World Health Organization (WHO), 2012
Switzerland Vital Registration - Deaths 1986 ICD8 as it appears in World Health Organization (WHO). WHO Mortality Database Version July 2012. Geneva, Switzerland: World Health Organization (WHO), 2012
Switzerland Vital Registration - Deaths 1987 ICD8 as it appears in World Health Organization (WHO). WHO Mortality Database Version July 2012. Geneva, Switzerland: World Health Organization (WHO), 2012
Switzerland Vital Registration - Deaths 1988 ICD8 as it appears in World Health Organization (WHO). WHO Mortality Database Version July 2012. Geneva, Switzerland: World Health Organization (WHO), 2012
Switzerland Vital Registration - Deaths 1989 ICD8 as it appears in World Health Organization (WHO). WHO Mortality Database Version July 2012. Geneva, Switzerland: World Health Organization (WHO), 2012
Switzerland Vital Registration - Deaths 1990 ICD8 as it appears in World Health Organization (WHO). WHO Mortality Database Version July 2012. Geneva, Switzerland: World Health Organization (WHO), 2012

Switzerland Vital Registration - Deaths 1991 ICD8 as it appears in World Health Organization (WHO). WHO Mortality Database Version July 2012. Geneva, Switzerland: World Health Organization (WHO), 2012
Switzerland Vital Registration - Deaths 1992 ICD8 as it appears in World Health Organization (WHO). WHO Mortality Database Version July 2012. Geneva, Switzerland: World Health Organization (WHO), 2012
Switzerland Vital Registration - Deaths 1993 ICD8 as it appears in World Health Organization (WHO). WHO Mortality Database Version July 2012. Geneva, Switzerland: World Health Organization (WHO), 2012
Switzerland Vital Registration - Deaths 1994 ICD8 as it appears in World Health Organization (WHO). WHO Mortality Database Version July 2012. Geneva, Switzerland: World Health Organization (WHO), 2012
Switzerland Vital Registration - Deaths 1995 ICD10 as it appears in World Health Organization (WHO). WHO Mortality Database Version October 2017. Geneva, Switzerland: World Health Organization (WHO), 2017
Switzerland Vital Registration - Deaths 1996 ICD10 as it appears in World Health Organization (WHO). WHO Mortality Database Version October 2017. Geneva, Switzerland: World Health Organization (WHO), 2017
Switzerland Vital Registration - Deaths 1997 ICD10 as it appears in World Health Organization (WHO). WHO Mortality Database Version October 2017. Geneva, Switzerland: World Health Organization (WHO), 2017
Switzerland Vital Registration - Deaths 1998 ICD10 as it appears in World Health Organization (WHO). WHO Mortality Database Version October 2017. Geneva, Switzerland: World Health Organization (WHO), 2017
Switzerland Vital Registration - Deaths 1999 ICD10 as it appears in World Health Organization (WHO). WHO Mortality Database Version October 2017. Geneva, Switzerland: World Health Organization (WHO), 2017
Switzerland Vital Registration - Deaths 2000 ICD10 as it appears in World Health Organization (WHO). WHO Mortality Database Version October 2017. Geneva, Switzerland: World Health Organization (WHO), 2017
Switzerland Vital Registration - Deaths 2001 ICD10 as it appears in World Health Organization (WHO). WHO Mortality Database Version October 2017. Geneva, Switzerland: World Health Organization (WHO), 2017
Switzerland Vital Registration - Deaths 2002 ICD10 as it appears in World Health Organization (WHO). WHO Mortality Database Version October 2017. Geneva, Switzerland: World Health Organization (WHO), 2017
Switzerland Vital Registration - Deaths 2003 ICD10 as it appears in World Health Organization (WHO). WHO Mortality Database Version October 2017. Geneva, Switzerland: World Health Organization (WHO), 2017
Switzerland Vital Registration - Deaths 2004 ICD10 as it appears in World Health Organization (WHO). WHO Mortality Database Version October 2017. Geneva, Switzerland: World Health Organization (WHO), 2017
Switzerland Vital Registration - Deaths 2005 ICD10 as it appears in World Health Organization (WHO). WHO Mortality Database Version October 2017. Geneva, Switzerland: World Health Organization (WHO), 2017
Switzerland Vital Registration - Deaths 2006 ICD10 as it appears in World Health Organization (WHO). WHO Mortality Database Version October 2017. Geneva, Switzerland: World Health Organization (WHO), 2017
Switzerland Vital Registration - Deaths 2007 ICD10 as it appears in World Health Organization (WHO). WHO Mortality Database Version October 2017. Geneva, Switzerland: World Health Organization (WHO), 2017
Switzerland Vital Registration - Deaths 2008 ICD10 as it appears in World Health Organization (WHO). WHO Mortality Database Version October 2017. Geneva, Switzerland: World Health Organization (WHO), 2017

Switzerland Vital Registration - Deaths 2009 ICD10 as it appears in World Health Organization (WHO). WHO Mortality Database Version October 2017. Geneva, Switzerland: World Health Organization (WHO), 2017
Switzerland Vital Registration - Deaths 2010 ICD10 as it appears in World Health Organization (WHO). WHO Mortality Database Version October 2017. Geneva, Switzerland: World Health Organization (WHO), 2017
Switzerland Vital Registration - Deaths 2011 ICD10 as it appears in World Health Organization (WHO). WHO Mortality Database Version October 2017. Geneva, Switzerland: World Health Organization (WHO), 2017
Switzerland Vital Registration - Deaths 2012 ICD10 as it appears in World Health Organization (WHO). WHO Mortality Database Version October 2017. Geneva, Switzerland: World Health Organization (WHO), 2017
Switzerland Vital Registration - Deaths 2013 ICD10 as it appears in World Health Organization (WHO). WHO Mortality Database Version October 2017. Geneva, Switzerland: World Health Organization (WHO), 2017
Switzerland Vital Registration - Deaths 2014 ICD10 as it appears in World Health Organization (WHO). WHO Mortality Database Version October 2017. Geneva, Switzerland: World Health Organization (WHO), 2017
Switzerland Vital Registration - Deaths 2015 ICD10 as it appears in World Health Organization (WHO). WHO Mortality Database Version April 2018. Geneva, Switzerland: World Health Organization (WHO), 2018
Syria Vital Registration - Deaths 2010 ICD10 as it appears in World Health Organization (WHO). WHO Mortality Database Version October 2017. Geneva, Switzerland: World Health Organization (WHO), 2017
Syrian Arab Republic Vital Registration - Deaths 1999 ICD10 as it appears in World Health Organization (WHO). WHO Mortality Database Version November 2015. Geneva, Switzerland: World Health Organization (WHO), 2015
Syrian Arab Republic Vital Registration - Deaths 2000 ICD10 as it appears in World Health Organization (WHO). WHO Mortality Database Version November 2015. Geneva, Switzerland: World Health Organization (WHO), 2015
Syrian Arab Republic Vital Registration - Deaths 2001 ICD10 as it appears in World Health Organization (WHO). WHO Mortality Database Version November 2015. Geneva, Switzerland: World Health Organization (WHO), 2015
Syrian Arab Republic Vital Registration - Deaths 2002 ICD10 as it appears in World Health Organization (WHO). WHO Mortality Database Version November 2015. Geneva, Switzerland: World Health Organization (WHO), 2015
Syrian Arab Republic Vital Registration - Deaths 2003 ICD10 as it appears in World Health Organization (WHO). WHO Mortality Database Version November 2015. Geneva, Switzerland: World Health Organization (WHO), 2015
Syrian Arab Republic Vital Registration - Deaths 2004 ICD10 as it appears in World Health Organization (WHO). WHO Mortality Database Version November 2015. Geneva, Switzerland: World Health Organization (WHO), 2015
Syrian Arab Republic Vital Registration - Deaths 2005 ICD10 as it appears in World Health Organization (WHO). WHO Mortality Database Version November 2015. Geneva, Switzerland: World Health Organization (WHO), 2015
Syrian Arab Republic Vital Registration - Deaths 2006 ICD10 as it appears in World Health Organization (WHO). WHO Mortality Database Version November 2015. Geneva, Switzerland: World Health Organization (WHO), 2015
Syrian Arab Republic Vital Registration - Deaths 2007 ICD10 as it appears in World Health Organization (WHO). WHO Mortality Database Version November 2015. Geneva, Switzerland: World Health Organization (WHO), 2015
Syrian Arab Republic Vital Registration - Deaths 2008 ICD10 as it appears in World Health Organization (WHO). WHO Mortality Database Version November 2015. Geneva, Switzerland: World Health Organization (WHO), 2015

Syrian Arab Republic Vital Registration - Deaths 2009 ICD10 as it appears in World Health Organization (WHO). WHO Mortality Database Version November 2015. Geneva, Switzerland: World Health Organization (WHO), 2015
Tajikistan Vital Registration - Deaths 1981 ICD9 as it appears in World Health Organization (WHO). WHO Mortality Database Version September 2016. Geneva, Switzerland: World Health Organization (WHO), 2016
Tajikistan Vital Registration - Deaths 1982 ICD9 as it appears in World Health Organization (WHO). WHO Mortality Database Version September 2016. Geneva, Switzerland: World Health Organization (WHO), 2016
Tajikistan Vital Registration - Deaths 1985 ICD9 as it appears in World Health Organization (WHO). WHO Mortality Database Version September 2016. Geneva, Switzerland: World Health Organization (WHO), 2016
Tajikistan Vital Registration - Deaths 1986 ICD9 as it appears in World Health Organization (WHO). WHO Mortality Database Version September 2016. Geneva, Switzerland: World Health Organization (WHO), 2016
Tajikistan Vital Registration - Deaths 1987 ICD9 as it appears in World Health Organization (WHO). WHO Mortality Database Version September 2016. Geneva, Switzerland: World Health Organization (WHO), 2016
Tajikistan Vital Registration - Deaths 1988 ICD9 as it appears in World Health Organization (WHO). WHO Mortality Database Version September 2016. Geneva, Switzerland: World Health Organization (WHO), 2016
Tajikistan Vital Registration - Deaths 1989 ICD9 as it appears in World Health Organization (WHO). WHO Mortality Database Version September 2016. Geneva, Switzerland: World Health Organization (WHO), 2016
Tajikistan Vital Registration - Deaths 1990 ICD9 as it appears in World Health Organization (WHO). WHO Mortality Database Version September 2016. Geneva, Switzerland: World Health Organization (WHO), 2016
Tajikistan Vital Registration - Deaths 1991 ICD9 as it appears in World Health Organization (WHO). WHO Mortality Database Version September 2016. Geneva, Switzerland: World Health Organization (WHO), 2016
Tajikistan Vital Registration - Deaths 1992 ICD9 as it appears in World Health Organization (WHO). WHO Mortality Database Version September 2016. Geneva, Switzerland: World Health Organization (WHO), 2016
Tajikistan Vital Registration - Deaths 1993 ICD9 as it appears in World Health Organization (WHO). WHO Mortality Database Version September 2016. Geneva, Switzerland: World Health Organization (WHO), 2016
Tajikistan Vital Registration - Deaths 1994 ICD9 as it appears in World Health Organization (WHO). WHO Mortality Database Version September 2016. Geneva, Switzerland: World Health Organization (WHO), 2016
Tajikistan Vital Registration - Deaths 1995 ICD9 as it appears in World Health Organization (WHO). WHO Mortality Database Version September 2016. Geneva, Switzerland: World Health Organization (WHO), 2016
Tajikistan Vital Registration - Deaths 1996 ICD9 as it appears in World Health Organization (WHO). WHO Mortality Database Version September 2016. Geneva, Switzerland: World Health Organization (WHO), 2016
Tajikistan Vital Registration - Deaths 1997 ICD9 as it appears in World Health Organization (WHO). WHO Mortality Database Version September 2016. Geneva, Switzerland: World Health Organization (WHO), 2016
Tajikistan Vital Registration - Deaths 1998 ICD9 as it appears in World Health Organization (WHO). WHO Mortality Database Version September 2016. Geneva, Switzerland: World Health Organization (WHO), 2016
Tajikistan Vital Registration - Deaths 1999 ICD9 as it appears in World Health Organization (WHO). WHO Mortality Database Version September 2016. Geneva, Switzerland: World Health Organization (WHO), 2016

Tajikistan Vital Registration - Deaths 2000 ICD9 as it appears in World Health Organization (WHO). WHO Mortality Database Version September 2016. Geneva, Switzerland: World Health Organization (WHO), 2016
Tajikistan Vital Registration - Deaths 2001 ICD9 as it appears in World Health Organization (WHO). WHO Mortality Database Version September 2016. Geneva, Switzerland: World Health Organization (WHO), 2016
Tajikistan Vital Registration - Deaths 2002 ICD9 as it appears in World Health Organization (WHO). WHO Mortality Database Version September 2016. Geneva, Switzerland: World Health Organization (WHO), 2016
Tajikistan Vital Registration - Deaths 2003 ICD9 as it appears in World Health Organization (WHO). WHO Mortality Database Version September 2016. Geneva, Switzerland: World Health Organization (WHO), 2016
Tajikistan Vital Registration - Deaths 2004 ICD9 as it appears in World Health Organization (WHO). WHO Mortality Database Version September 2016. Geneva, Switzerland: World Health Organization (WHO), 2016
Tajikistan Vital Registration - Deaths 2005 ICD9 as it appears in World Health Organization (WHO). WHO Mortality Database Version September 2016. Geneva, Switzerland: World Health Organization (WHO), 2016
Tajikistan Vital Registration - Deaths 2016 ICD10 as it appears in World Health Organization (WHO). WHO Mortality Database Version April 2018. Geneva, Switzerland: World Health Organization (WHO), 2018
Thailand Vital Registration - Deaths 2003 ICD10 as it appears in World Health Organization (WHO). WHO Mortality Database Version October 2017. Geneva, Switzerland: World Health Organization (WHO), 2017
Thailand Vital Registration - Deaths 2011 ICD10 as it appears in World Health Organization (WHO). WHO Mortality Database Version October 2017. Geneva, Switzerland: World Health Organization (WHO), 2017
Thailand Vital Registration - Deaths 2012 ICD10 as it appears in World Health Organization (WHO). WHO Mortality Database Version October 2017. Geneva, Switzerland: World Health Organization (WHO), 2017
Thailand Vital Registration - Deaths 2013 ICD10 as it appears in World Health Organization (WHO). WHO Mortality Database Version October 2017. Geneva, Switzerland: World Health Organization (WHO), 2017
Thailand Vital Registration - Deaths 2014 ICD10 as it appears in World Health Organization (WHO). WHO Mortality Database Version October 2017. Geneva, Switzerland: World Health Organization (WHO), 2017
Thailand Vital Registration - Deaths 2015 ICD10 as it appears in World Health Organization (WHO). WHO Mortality Database Version October 2017. Geneva, Switzerland: World Health Organization (WHO), 2017
Thailand Vital Registration - Deaths 2016 ICD10 as it appears in World Health Organization (WHO). WHO Mortality Database Version April 2018. Geneva, Switzerland: World Health Organization (WHO), 2018
Tielsch JM, Khatry SK, Stoltzfus RJ, Katz J, LeClerq SC, Adhikari R, Mullany LC, Black R, Shresta S. Effect of daily zinc supplementation on child mortality in southern Nepal: a community-based, cluster randomised, placebo-controlled trial. Lancet. 2007; 370(9594): 1230-9
Trinidad and Tobago Vital Registration - Deaths 1980 ICD9 as it appears in World Health Organization (WHO). WHO Mortality Database Version September 2016. Geneva, Switzerland: World Health Organization (WHO), 2016
Trinidad and Tobago Vital Registration - Deaths 1981 ICD9 as it appears in World Health Organization (WHO). WHO Mortality Database Version September 2016. Geneva, Switzerland: World Health Organization (WHO), 2016
Trinidad and Tobago Vital Registration - Deaths 1982 ICD9 as it appears in World Health Organization (WHO). WHO Mortality Database Version September 2016. Geneva, Switzerland: World Health Organization (WHO), 2016

Trinidad and Tobago Vital Registration - Deaths 1983 ICD9 as it appears in World Health Organization (WHO). WHO Mortality Database Version September 2016. Geneva, Switzerland: World Health Organization (WHO), 2016
Trinidad and Tobago Vital Registration - Deaths 1984 ICD9 as it appears in World Health Organization (WHO). WHO Mortality Database Version September 2016. Geneva, Switzerland: World Health Organization (WHO), 2016
Trinidad and Tobago Vital Registration - Deaths 1985 ICD9 as it appears in World Health Organization (WHO). WHO Mortality Database Version September 2016. Geneva, Switzerland: World Health Organization (WHO), 2016
Trinidad and Tobago Vital Registration - Deaths 1986 ICD9 as it appears in World Health Organization (WHO). WHO Mortality Database Version September 2016. Geneva, Switzerland: World Health Organization (WHO), 2016
Trinidad and Tobago Vital Registration - Deaths 1987 ICD9 as it appears in World Health Organization (WHO). WHO Mortality Database Version September 2016. Geneva, Switzerland: World Health Organization (WHO), 2016
Trinidad and Tobago Vital Registration - Deaths 1988 ICD9 as it appears in World Health Organization (WHO). WHO Mortality Database Version September 2016. Geneva, Switzerland: World Health Organization (WHO), 2016
Trinidad and Tobago Vital Registration - Deaths 1989 ICD9 as it appears in World Health Organization (WHO). WHO Mortality Database Version September 2016. Geneva, Switzerland: World Health Organization (WHO), 2016
Trinidad and Tobago Vital Registration - Deaths 1990 ICD9 as it appears in World Health Organization (WHO). WHO Mortality Database Version September 2016. Geneva, Switzerland: World Health Organization (WHO), 2016
Trinidad and Tobago Vital Registration - Deaths 1991 ICD9 as it appears in World Health Organization (WHO). WHO Mortality Database Version September 2016. Geneva, Switzerland: World Health Organization (WHO), 2016
Trinidad and Tobago Vital Registration - Deaths 1992 ICD9 as it appears in World Health Organization (WHO). WHO Mortality Database Version September 2016. Geneva, Switzerland: World Health Organization (WHO), 2016
Trinidad and Tobago Vital Registration - Deaths 1993 ICD9 as it appears in World Health Organization (WHO). WHO Mortality Database Version September 2016. Geneva, Switzerland: World Health Organization (WHO), 2016
Trinidad and Tobago Vital Registration - Deaths 1994 ICD9 as it appears in World Health Organization (WHO). WHO Mortality Database Version September 2016. Geneva, Switzerland: World Health Organization (WHO), 2016
Trinidad and Tobago Vital Registration - Deaths 1995 ICD9 as it appears in World Health Organization (WHO). WHO Mortality Database Version September 2016. Geneva, Switzerland: World Health Organization (WHO), 2016
Trinidad and Tobago Vital Registration - Deaths 1996 ICD9 as it appears in World Health Organization (WHO). WHO Mortality Database Version September 2016. Geneva, Switzerland: World Health Organization (WHO), 2016
Trinidad and Tobago Vital Registration - Deaths 1997 ICD9 as it appears in World Health Organization (WHO). WHO Mortality Database Version September 2016. Geneva, Switzerland: World Health Organization (WHO), 2016
Trinidad and Tobago Vital Registration - Deaths 1998 ICD9 as it appears in World Health Organization (WHO). WHO Mortality Database Version September 2016. Geneva, Switzerland: World Health Organization (WHO), 2016
Trinidad and Tobago Vital Registration - Deaths 1999 ICD10 as it appears in World Health Organization (WHO). WHO Mortality Database Version October 2017. Geneva, Switzerland: World Health Organization (WHO), 2017
Trinidad and Tobago Vital Registration - Deaths 2000 ICD10 as it appears in World Health Organization (WHO). WHO Mortality Database Version October 2017. Geneva, Switzerland: World Health Organization (WHO), 2017

Trinidad and Tobago Vital Registration - Deaths 2001 ICD10 as it appears in World Health Organization (WHO). WHO Mortality Database Version October 2017. Geneva, Switzerland: World Health Organization (WHO), 2017

Trinidad and Tobago Vital Registration - Deaths 2002 ICD10 as it appears in World Health Organization (WHO). WHO Mortality Database Version October 2017. Geneva, Switzerland: World Health Organization (WHO), 2017

Trinidad and Tobago Vital Registration - Deaths 2003 ICD10 as it appears in World Health Organization (WHO). WHO Mortality Database Version October 2017. Geneva, Switzerland: World Health Organization (WHO), 2017

Trinidad and Tobago Vital Registration - Deaths 2004 ICD10 as it appears in World Health Organization (WHO). WHO Mortality Database Version October 2017. Geneva, Switzerland: World Health Organization (WHO), 2017

Trinidad and Tobago Vital Registration - Deaths 2005 ICD10 as it appears in World Health Organization (WHO). WHO Mortality Database Version October 2017. Geneva, Switzerland: World Health Organization (WHO), 2017

Trinidad and Tobago Vital Registration - Deaths 2006 ICD10 as it appears in World Health Organization (WHO). WHO Mortality Database Version October 2017. Geneva, Switzerland: World Health Organization (WHO), 2017

Trinidad and Tobago Vital Registration - Deaths 2007 ICD10 as it appears in World Health Organization (WHO). WHO Mortality Database Version October 2017. Geneva, Switzerland: World Health Organization (WHO), 2017

Trinidad and Tobago Vital Registration - Deaths 2008 ICD10 as it appears in World Health Organization (WHO). WHO Mortality Database Version October 2017. Geneva, Switzerland: World Health Organization (WHO), 2017

Trinidad and Tobago Vital Registration - Deaths 2009 ICD10 as it appears in World Health Organization (WHO). WHO Mortality Database Version October 2017. Geneva, Switzerland: World Health Organization (WHO), 2017

Trinidad and Tobago Vital Registration - Deaths 2010 ICD10 as it appears in World Health Organization (WHO). WHO Mortality Database Version October 2017. Geneva, Switzerland: World Health Organization (WHO), 2017

Trinidad and Tobago Vital Registration - Deaths 2011 ICD10 as it appears in World Health Organization (WHO). WHO Mortality Database Version October 2017. Geneva, Switzerland: World Health Organization (WHO), 2017

Turkey Vital Registration - Deaths 2009 ICD10 as it appears in World Health Organization (WHO). WHO Mortality Database Version October 2017. Geneva, Switzerland: World Health Organization (WHO), 2017

Turkey Vital Registration - Deaths 2010 ICD10 as it appears in World Health Organization (WHO). WHO Mortality Database Version October 2017. Geneva, Switzerland: World Health Organization (WHO), 2017

Turkey Vital Registration - Deaths 2011 ICD10 as it appears in World Health Organization (WHO). WHO Mortality Database Version October 2017. Geneva, Switzerland: World Health Organization (WHO), 2017

Turkey Vital Registration - Deaths 2012 ICD10 as it appears in World Health Organization (WHO). WHO Mortality Database Version October 2017. Geneva, Switzerland: World Health Organization (WHO), 2017

Turkey Vital Registration - Deaths 2013 ICD10 as it appears in World Health Organization (WHO). WHO Mortality Database Version October 2017. Geneva, Switzerland: World Health Organization (WHO), 2017

Turkey Vital Registration - Deaths 2014 ICD10 as it appears in World Health Organization (WHO). WHO Mortality Database Version April 2018. Geneva, Switzerland: World Health Organization (WHO), 2018

Turkey Vital Registration - Deaths 2015 ICD10 as it appears in World Health Organization (WHO). WHO Mortality Database Version April 2018. Geneva, Switzerland: World Health Organization (WHO), 2018

Turkmenistan Vital Registration - Deaths 1981 ICD9 as it appears in World Health Organization (WHO). WHO Mortality Database Version September 2016. Geneva, Switzerland: World Health Organization (WHO), 2016
Turkmenistan Vital Registration - Deaths 1982 ICD9 as it appears in World Health Organization (WHO). WHO Mortality Database Version September 2016. Geneva, Switzerland: World Health Organization (WHO), 2016
Turkmenistan Vital Registration - Deaths 1985 ICD9 as it appears in World Health Organization (WHO). WHO Mortality Database Version September 2016. Geneva, Switzerland: World Health Organization (WHO), 2016
Turkmenistan Vital Registration - Deaths 1986 ICD9 as it appears in World Health Organization (WHO). WHO Mortality Database Version September 2016. Geneva, Switzerland: World Health Organization (WHO), 2016
Turkmenistan Vital Registration - Deaths 1987 ICD9 as it appears in World Health Organization (WHO). WHO Mortality Database Version September 2016. Geneva, Switzerland: World Health Organization (WHO), 2016
Turkmenistan Vital Registration - Deaths 1988 ICD9 as it appears in World Health Organization (WHO). WHO Mortality Database Version September 2016. Geneva, Switzerland: World Health Organization (WHO), 2016
Turkmenistan Vital Registration - Deaths 1989 ICD9 as it appears in World Health Organization (WHO). WHO Mortality Database Version September 2016. Geneva, Switzerland: World Health Organization (WHO), 2016
Turkmenistan Vital Registration - Deaths 1991 ICD9 as it appears in World Health Organization (WHO). WHO Mortality Database Version September 2016. Geneva, Switzerland: World Health Organization (WHO), 2016
Turkmenistan Vital Registration - Deaths 1992 ICD9 as it appears in World Health Organization (WHO). WHO Mortality Database Version September 2016. Geneva, Switzerland: World Health Organization (WHO), 2016
Turkmenistan Vital Registration - Deaths 1994 ICD9 as it appears in World Health Organization (WHO). WHO Mortality Database Version September 2016. Geneva, Switzerland: World Health Organization (WHO), 2016
Turkmenistan Vital Registration - Deaths 1995 ICD9 as it appears in World Health Organization (WHO). WHO Mortality Database Version September 2016. Geneva, Switzerland: World Health Organization (WHO), 2016
Turkmenistan Vital Registration - Deaths 1996 ICD9 as it appears in World Health Organization (WHO). WHO Mortality Database Version September 2016. Geneva, Switzerland: World Health Organization (WHO), 2016
Turkmenistan Vital Registration - Deaths 1997 ICD9 as it appears in World Health Organization (WHO). WHO Mortality Database Version September 2016. Geneva, Switzerland: World Health Organization (WHO), 2016
Turkmenistan Vital Registration - Deaths 1998 ICD9 as it appears in World Health Organization (WHO). WHO Mortality Database Version September 2016. Geneva, Switzerland: World Health Organization (WHO), 2016
Turkmenistan Vital Registration - Deaths 1999 ICD10 as it appears in World Health Organization (WHO). WHO Mortality Database Version April 2018. Geneva, Switzerland: World Health Organization (WHO), 2018
Turkmenistan Vital Registration - Deaths 1990 ICD9 as it appears in World Health Organization (WHO). WHO Mortality Database Version September 2016. Geneva, Switzerland: World Health Organization (WHO), 2016
Turkmenistan Vital Registration - Deaths 1993 ICD9 as it appears in World Health Organization (WHO). WHO Mortality Database Version September 2016. Geneva, Switzerland: World Health Organization (WHO), 2016
Turkmenistan Vital Registration - Deaths 2000 ICD10 as it appears in World Health Organization (WHO). WHO Mortality Database Version November 2015. Geneva, Switzerland: World Health Organization (WHO), 2015

Turkmenistan Vital Registration - Deaths 2001 ICD10 as it appears in World Health Organization (WHO). WHO Mortality Database Version November 2015. Geneva, Switzerland: World Health Organization (WHO), 2015

Turkmenistan Vital Registration - Deaths 2002 ICD10 as it appears in World Health Organization (WHO). WHO Mortality Database Version November 2015. Geneva, Switzerland: World Health Organization (WHO), 2015

Turkmenistan Vital Registration - Deaths 2003 ICD10 as it appears in World Health Organization (WHO). WHO Mortality Database Version November 2015. Geneva, Switzerland: World Health Organization (WHO), 2015

Turkmenistan Vital Registration - Deaths 2004 ICD10 as it appears in World Health Organization (WHO). WHO Mortality Database Version November 2015. Geneva, Switzerland: World Health Organization (WHO), 2015

Turkmenistan Vital Registration - Deaths 2005 ICD10 as it appears in World Health Organization (WHO). WHO Mortality Database Version November 2015. Geneva, Switzerland: World Health Organization (WHO), 2015

Turkmenistan Vital Registration - Deaths 2006 ICD10 as it appears in World Health Organization (WHO). WHO Mortality Database Version November 2015. Geneva, Switzerland: World Health Organization (WHO), 2015

Turkmenistan Vital Registration - Deaths 2007 ICD10 as it appears in World Health Organization (WHO). WHO Mortality Database Version November 2015. Geneva, Switzerland: World Health Organization (WHO), 2015

Turkmenistan Vital Registration - Deaths 2008 ICD10 as it appears in World Health Organization (WHO). WHO Mortality Database Version November 2015. Geneva, Switzerland: World Health Organization (WHO), 2015

Turkmenistan Vital Registration - Deaths 2009 ICD10 as it appears in World Health Organization (WHO). WHO Mortality Database Version November 2015. Geneva, Switzerland: World Health Organization (WHO), 2015

Turkmenistan Vital Registration - Deaths 2010 ICD10 as it appears in World Health Organization (WHO). WHO Mortality Database Version November 2015. Geneva, Switzerland: World Health Organization (WHO), 2015

Turkmenistan Vital Registration - Deaths 2011 ICD10 as it appears in World Health Organization (WHO). WHO Mortality Database Version November 2015. Geneva, Switzerland: World Health Organization (WHO), 2015

Turkmenistan Vital Registration - Deaths 2012 ICD10 as it appears in World Health Organization (WHO). WHO Mortality Database Version November 2015. Geneva, Switzerland: World Health Organization (WHO), 2015

Turkmenistan Vital Registration - Deaths 2013 ICD10 as it appears in World Health Organization (WHO). WHO Mortality Database Version November 2015. Geneva, Switzerland: World Health Organization (WHO), 2015

Turkmenistan Vital Registration - Deaths 2014 ICD10 as it appears in World Health Organization (WHO). WHO Mortality Database Version November 2015. Geneva, Switzerland: World Health Organization (WHO), 2015

Turkmenistan Vital Registration - Deaths 2015 ICD10 as it appears in World Health Organization (WHO). WHO Mortality Database Version April 2018. Geneva, Switzerland: World Health Organization (WHO), 2018

Ukraine Mortality by Region, Age, Sex, and Cause of Death 2015 as it appears in Center for Demographic Research, New Economic School (Russia). Russia Mortality Rates by Region, 1-Year Age Groups, and Sex, 2015-2016. Moscow, Russia: Center for Demographic Research, New Economic School (Russia). http://demogr.nes.ru/index.php/ru/demogr_indicat/data

Ukraine Mortality by Region, Age, Sex, and Cause of Death 2016 as it appears in Center for Demographic Research, New Economic School (Russia). Russia Mortality Rates by Region, 1-Year Age Groups, and Sex, 2015-2016. Moscow, Russia: Center for Demographic Research, New Economic School (Russia). http://demogr.nes.ru/index.php/ru/demogr_indicat/data

Ukraine Vital Registration - Deaths 1981 ICD9 as it appears in World Health Organization (WHO). WHO Mortality Database Version September 2016. Geneva, Switzerland: World Health Organization (WHO), 2016
Ukraine Vital Registration - Deaths 1982 ICD9 as it appears in World Health Organization (WHO). WHO Mortality Database Version September 2016. Geneva, Switzerland: World Health Organization (WHO), 2016
Ukraine Vital Registration - Deaths 1985 ICD9 as it appears in World Health Organization (WHO). WHO Mortality Database Version September 2016. Geneva, Switzerland: World Health Organization (WHO), 2016
Ukraine Vital Registration - Deaths 1986 ICD9 as it appears in World Health Organization (WHO). WHO Mortality Database Version September 2016. Geneva, Switzerland: World Health Organization (WHO), 2016
Ukraine Vital Registration - Deaths 1987 ICD9 as it appears in World Health Organization (WHO). WHO Mortality Database Version September 2016. Geneva, Switzerland: World Health Organization (WHO), 2016
Ukraine Vital Registration - Deaths 1988 ICD9 as it appears in World Health Organization (WHO). WHO Mortality Database Version September 2016. Geneva, Switzerland: World Health Organization (WHO), 2016
Ukraine Vital Registration - Deaths 1989 ICD9 as it appears in World Health Organization (WHO). WHO Mortality Database Version September 2016. Geneva, Switzerland: World Health Organization (WHO), 2016
Ukraine Vital Registration - Deaths 1990 ICD9 as it appears in World Health Organization (WHO). WHO Mortality Database Version September 2016. Geneva, Switzerland: World Health Organization (WHO), 2016
Ukraine Vital Registration - Deaths 1991 ICD9 as it appears in World Health Organization (WHO). WHO Mortality Database Version September 2016. Geneva, Switzerland: World Health Organization (WHO), 2016
Ukraine Vital Registration - Deaths 1992 ICD9 as it appears in World Health Organization (WHO). WHO Mortality Database Version September 2016. Geneva, Switzerland: World Health Organization (WHO), 2016
Ukraine Vital Registration - Deaths 1993 ICD9 as it appears in World Health Organization (WHO). WHO Mortality Database Version September 2016. Geneva, Switzerland: World Health Organization (WHO), 2016
Ukraine Vital Registration - Deaths 1994 ICD9 as it appears in World Health Organization (WHO). WHO Mortality Database Version September 2016. Geneva, Switzerland: World Health Organization (WHO), 2016
Ukraine Vital Registration - Deaths 1995 ICD9 as it appears in World Health Organization (WHO). WHO Mortality Database Version September 2016. Geneva, Switzerland: World Health Organization (WHO), 2016
Ukraine Vital Registration - Deaths 1996 ICD9 as it appears in World Health Organization (WHO). WHO Mortality Database Version September 2016. Geneva, Switzerland: World Health Organization (WHO), 2016
Ukraine Vital Registration - Deaths 1997 ICD9 as it appears in World Health Organization (WHO). WHO Mortality Database Version September 2016. Geneva, Switzerland: World Health Organization (WHO), 2016
Ukraine Vital Registration - Deaths 1998 ICD9 as it appears in World Health Organization (WHO). WHO Mortality Database Version September 2016. Geneva, Switzerland: World Health Organization (WHO), 2016
Ukraine Vital Registration - Deaths 1999 ICD9 as it appears in World Health Organization (WHO). WHO Mortality Database Version September 2016. Geneva, Switzerland: World Health Organization (WHO), 2016
Ukraine Vital Registration - Deaths 2000 ICD9 as it appears in World Health Organization (WHO). WHO Mortality Database Version September 2016. Geneva, Switzerland: World Health Organization (WHO), 2016

Ukraine Vital Registration - Deaths 2001 ICD9 as it appears in World Health Organization (WHO). WHO Mortality Database Version September 2016. Geneva, Switzerland: World Health Organization (WHO), 2016
Ukraine Vital Registration - Deaths 2002 ICD9 as it appears in World Health Organization (WHO). WHO Mortality Database Version September 2016. Geneva, Switzerland: World Health Organization (WHO), 2016
Ukraine Vital Registration - Deaths 2003 ICD9 as it appears in World Health Organization (WHO). WHO Mortality Database Version September 2016. Geneva, Switzerland: World Health Organization (WHO), 2016
Ukraine Vital Registration - Deaths 2004 ICD9 as it appears in World Health Organization (WHO). WHO Mortality Database Version September 2016. Geneva, Switzerland: World Health Organization (WHO), 2016
Ukraine Vital Registration - Deaths 2005 ICD10 as it appears in World Health Organization (WHO). WHO Mortality Database Version November 2015. Geneva, Switzerland: World Health Organization (WHO), 2015
Ukraine Vital Registration - Deaths 2006 ICD10 as it appears in World Health Organization (WHO). WHO Mortality Database Version November 2015. Geneva, Switzerland: World Health Organization (WHO), 2015
Ukraine Vital Registration - Deaths 2007 ICD10 as it appears in World Health Organization (WHO). WHO Mortality Database Version November 2015. Geneva, Switzerland: World Health Organization (WHO), 2015
Ukraine Vital Registration - Deaths 2008 ICD10 as it appears in World Health Organization (WHO). WHO Mortality Database Version November 2015. Geneva, Switzerland: World Health Organization (WHO), 2015
Ukraine Vital Registration - Deaths 2009 ICD10 as it appears in World Health Organization (WHO). WHO Mortality Database Version November 2015. Geneva, Switzerland: World Health Organization (WHO), 2015
Ukraine Vital Registration - Deaths 2010 ICD10 as it appears in World Health Organization (WHO). WHO Mortality Database Version November 2015. Geneva, Switzerland: World Health Organization (WHO), 2015
Ukraine Vital Registration - Deaths 2011 ICD10 as it appears in World Health Organization (WHO). WHO Mortality Database Version November 2015. Geneva, Switzerland: World Health Organization (WHO), 2015
Ukraine Vital Registration - Deaths 2012 ICD10 as it appears in World Health Organization (WHO). WHO Mortality Database Version November 2015. Geneva, Switzerland: World Health Organization (WHO), 2015
Ukraine Vital Registration - Deaths 2014 ICD10 as it appears in World Health Organization (WHO). WHO Mortality Database Version November 2015. Geneva, Switzerland: World Health Organization (WHO), 2015
United Kingdom - Northern Ireland Vital Registration - Deaths 1980 ICD9 as it appears in World Health Organization (WHO). WHO Mortality Database Version November 2015. Geneva, Switzerland: World Health Organization (WHO), 2015
United Kingdom - Northern Ireland Vital Registration - Deaths 1981 ICD9 as it appears in World Health Organization (WHO). WHO Mortality Database Version November 2015. Geneva, Switzerland: World Health Organization (WHO), 2015
United Kingdom - Northern Ireland Vital Registration - Deaths 1982 ICD9 as it appears in World Health Organization (WHO). WHO Mortality Database Version November 2015. Geneva, Switzerland: World Health Organization (WHO), 2015
United Kingdom - Northern Ireland Vital Registration - Deaths 1983 ICD9 as it appears in World Health Organization (WHO). WHO Mortality Database Version November 2015. Geneva, Switzerland: World Health Organization (WHO), 2015
United Kingdom - Northern Ireland Vital Registration - Deaths 1984 ICD9 as it appears in World Health Organization (WHO). WHO Mortality Database Version November 2015. Geneva, Switzerland: World Health Organization (WHO), 2015

United Kingdom - Northern Ireland Vital Registration - Deaths 1985 ICD9 as it appears in World Health Organization (WHO). WHO Mortality Database Version November 2015. Geneva, Switzerland: World Health Organization (WHO), 2015
United Kingdom - Northern Ireland Vital Registration - Deaths 1986 ICD9 as it appears in World Health Organization (WHO). WHO Mortality Database Version November 2015. Geneva, Switzerland: World Health Organization (WHO), 2015
United Kingdom - Northern Ireland Vital Registration - Deaths 1987 ICD9 as it appears in World Health Organization (WHO). WHO Mortality Database Version November 2015. Geneva, Switzerland: World Health Organization (WHO), 2015
United Kingdom - Northern Ireland Vital Registration - Deaths 1988 ICD9 as it appears in World Health Organization (WHO). WHO Mortality Database Version November 2015. Geneva, Switzerland: World Health Organization (WHO), 2015
United Kingdom - Northern Ireland Vital Registration - Deaths 1993 ICD9 as it appears in World Health Organization (WHO). WHO Mortality Database Version November 2015. Geneva, Switzerland: World Health Organization (WHO), 2015
United Kingdom - Northern Ireland Vital Registration - Deaths 1994 ICD9 as it appears in World Health Organization (WHO). WHO Mortality Database Version November 2015. Geneva, Switzerland: World Health Organization (WHO), 2015
United Kingdom - Northern Ireland Vital Registration - Deaths 1995 ICD9 as it appears in World Health Organization (WHO). WHO Mortality Database Version November 2015. Geneva, Switzerland: World Health Organization (WHO), 2015
United Kingdom - Northern Ireland Vital Registration - Deaths 1996 ICD9 as it appears in World Health Organization (WHO). WHO Mortality Database Version November 2015. Geneva, Switzerland: World Health Organization (WHO), 2015
United Kingdom - Northern Ireland Vital Registration - Deaths 1997 ICD9 as it appears in World Health Organization (WHO). WHO Mortality Database Version November 2015. Geneva, Switzerland: World Health Organization (WHO), 2015
United Kingdom - Northern Ireland Vital Registration - Deaths 1998 ICD9 as it appears in World Health Organization (WHO). WHO Mortality Database Version November 2015. Geneva, Switzerland: World Health Organization (WHO), 2015
United Kingdom - Northern Ireland Vital Registration - Deaths 1999 ICD9 as it appears in World Health Organization (WHO). WHO Mortality Database Version November 2015. Geneva, Switzerland: World Health Organization (WHO), 2015
United Kingdom - Northern Ireland Vital Registration - Deaths 2001 ICD10 as it appears in World Health Organization (WHO). WHO Mortality Database Version October 2017. Geneva, Switzerland: World Health Organization (WHO), 2017
United Kingdom - Northern Ireland Vital Registration - Deaths 2002 ICD10 as it appears in World Health Organization (WHO). WHO Mortality Database Version October 2017. Geneva, Switzerland: World Health Organization (WHO), 2017
United Kingdom - Northern Ireland Vital Registration - Deaths 2003 ICD10 as it appears in World Health Organization (WHO). WHO Mortality Database Version October 2017. Geneva, Switzerland: World Health Organization (WHO), 2017
United Kingdom - Northern Ireland Vital Registration - Deaths 2004 ICD10 as it appears in World Health Organization (WHO). WHO Mortality Database Version October 2017. Geneva, Switzerland: World Health Organization (WHO), 2017
United Kingdom - Northern Ireland Vital Registration - Deaths 2005 ICD10 as it appears in World Health Organization (WHO). WHO Mortality Database Version October 2017. Geneva, Switzerland: World Health Organization (WHO), 2017
United Kingdom - Northern Ireland Vital Registration - Deaths 2006 ICD10 as it appears in World Health Organization (WHO). WHO Mortality Database Version October 2017. Geneva, Switzerland: World Health Organization (WHO), 2017
United Kingdom - Northern Ireland Vital Registration - Deaths 2007 ICD10 as it appears in World Health Organization (WHO). WHO Mortality Database Version October 2017. Geneva, Switzerland: World Health Organization (WHO), 2017

United Kingdom - Northern Ireland Vital Registration - Deaths 1989 ICD9 as it appears in World Health Organization (WHO). WHO Mortality Database Version November 2015. Geneva, Switzerland: World Health Organization (WHO), 2015
United Kingdom - Northern Ireland Vital Registration - Deaths 1990 ICD9 as it appears in World Health Organization (WHO). WHO Mortality Database Version November 2015. Geneva, Switzerland: World Health Organization (WHO), 2015
United Kingdom - Northern Ireland Vital Registration - Deaths 1991 ICD9 as it appears in World Health Organization (WHO). WHO Mortality Database Version November 2015. Geneva, Switzerland: World Health Organization (WHO), 2015
United Kingdom - Northern Ireland Vital Registration - Deaths 1992 ICD9 as it appears in World Health Organization (WHO). WHO Mortality Database Version November 2015. Geneva, Switzerland: World Health Organization (WHO), 2015
United Kingdom - Northern Ireland Vital Registration - Deaths 2000 ICD9 as it appears in World Health Organization (WHO). WHO Mortality Database Version November 2015. Geneva, Switzerland: World Health Organization (WHO), 2015
United Kingdom - Northern Ireland Vital Registration - Deaths 2008 ICD10 as it appears in World Health Organization (WHO). WHO Mortality Database Version October 2017. Geneva, Switzerland: World Health Organization (WHO), 2017
United Kingdom - Northern Ireland Vital Registration - Deaths 2009 ICD10 as it appears in World Health Organization (WHO). WHO Mortality Database Version October 2017. Geneva, Switzerland: World Health Organization (WHO), 2017
United Kingdom - Northern Ireland Vital Registration - Deaths 2010 ICD10 as it appears in World Health Organization (WHO). WHO Mortality Database Version October 2017. Geneva, Switzerland: World Health Organization (WHO), 2017
United Kingdom - Northern Ireland Vital Registration - Deaths 2011 ICD10 as it appears in World Health Organization (WHO). WHO Mortality Database Version October 2017. Geneva, Switzerland: World Health Organization (WHO), 2017
United Kingdom - Northern Ireland Vital Registration - Deaths 2012 ICD10 as it appears in World Health Organization (WHO). WHO Mortality Database Version October 2017. Geneva, Switzerland: World Health Organization (WHO), 2017
United Kingdom - Northern Ireland Vital Registration - Deaths 2013 ICD10 as it appears in World Health Organization (WHO). WHO Mortality Database Version October 2017. Geneva, Switzerland: World Health Organization (WHO), 2017
United Kingdom - Northern Ireland Vital Registration - Deaths 2014 ICD10 as it appears in World Health Organization (WHO). WHO Mortality Database Version April 2018. Geneva, Switzerland: World Health Organization (WHO), 2018
United Kingdom - Northern Ireland Vital Registration - Deaths 2015 ICD10 as it appears in World Health Organization (WHO). WHO Mortality Database Version April 2018. Geneva, Switzerland: World Health Organization (WHO), 2018
United Kingdom - Scotland Vital Registration - Deaths 1980 ICD9 as it appears in World Health Organization (WHO). WHO Mortality Database Version November 2015. Geneva, Switzerland: World Health Organization (WHO), 2015
United Kingdom - Scotland Vital Registration - Deaths 1981 ICD9 as it appears in World Health Organization (WHO). WHO Mortality Database Version November 2015. Geneva, Switzerland: World Health Organization (WHO), 2015
United Kingdom - Scotland Vital Registration - Deaths 1982 ICD9 as it appears in World Health Organization (WHO). WHO Mortality Database Version November 2015. Geneva, Switzerland: World Health Organization (WHO), 2015
United Kingdom - Scotland Vital Registration - Deaths 1983 ICD9 as it appears in World Health Organization (WHO). WHO Mortality Database Version November 2015. Geneva, Switzerland: World Health Organization (WHO), 2015
United Kingdom - Scotland Vital Registration - Deaths 1984 ICD9 as it appears in World Health Organization (WHO). WHO Mortality Database Version November 2015. Geneva, Switzerland: World Health Organization (WHO), 2015

United Kingdom - Scotland Vital Registration - Deaths 1985 ICD9 as it appears in World Health Organization (WHO). WHO Mortality Database Version November 2015. Geneva, Switzerland: World Health Organization (WHO), 2015
United Kingdom - Scotland Vital Registration - Deaths 1986 ICD9 as it appears in World Health Organization (WHO). WHO Mortality Database Version November 2015. Geneva, Switzerland: World Health Organization (WHO), 2015
United Kingdom - Scotland Vital Registration - Deaths 1987 ICD9 as it appears in World Health Organization (WHO). WHO Mortality Database Version November 2015. Geneva, Switzerland: World Health Organization (WHO), 2015
United Kingdom - Scotland Vital Registration - Deaths 1988 ICD9 as it appears in World Health Organization (WHO). WHO Mortality Database Version November 2015. Geneva, Switzerland: World Health Organization (WHO), 2015
United Kingdom - Scotland Vital Registration - Deaths 1989 ICD9 as it appears in World Health Organization (WHO). WHO Mortality Database Version November 2015. Geneva, Switzerland: World Health Organization (WHO), 2015
United Kingdom - Scotland Vital Registration - Deaths 1990 ICD9 as it appears in World Health Organization (WHO). WHO Mortality Database Version November 2015. Geneva, Switzerland: World Health Organization (WHO), 2015
United Kingdom - Scotland Vital Registration - Deaths 1991 ICD9 as it appears in World Health Organization (WHO). WHO Mortality Database Version November 2015. Geneva, Switzerland: World Health Organization (WHO), 2015
United Kingdom - Scotland Vital Registration - Deaths 1992 ICD9 as it appears in World Health Organization (WHO). WHO Mortality Database Version November 2015. Geneva, Switzerland: World Health Organization (WHO), 2015
United Kingdom - Scotland Vital Registration - Deaths 1993 ICD9 as it appears in World Health Organization (WHO). WHO Mortality Database Version November 2015. Geneva, Switzerland: World Health Organization (WHO), 2015
United Kingdom - Scotland Vital Registration - Deaths 1994 ICD9 as it appears in World Health Organization (WHO). WHO Mortality Database Version November 2015. Geneva, Switzerland: World Health Organization (WHO), 2015
United Kingdom - Scotland Vital Registration - Deaths 1995 ICD9 as it appears in World Health Organization (WHO). WHO Mortality Database Version November 2015. Geneva, Switzerland: World Health Organization (WHO), 2015
United Kingdom - Scotland Vital Registration - Deaths 1996 ICD9 as it appears in World Health Organization (WHO). WHO Mortality Database Version November 2015. Geneva, Switzerland: World Health Organization (WHO), 2015
United Kingdom - Scotland Vital Registration - Deaths 1997 ICD9 as it appears in World Health Organization (WHO). WHO Mortality Database Version November 2015. Geneva, Switzerland: World Health Organization (WHO), 2015
United Kingdom - Scotland Vital Registration - Deaths 1998 ICD9 as it appears in World Health Organization (WHO). WHO Mortality Database Version November 2015. Geneva, Switzerland: World Health Organization (WHO), 2015
United Kingdom - Scotland Vital Registration - Deaths 1999 ICD9 as it appears in World Health Organization (WHO). WHO Mortality Database Version November 2015. Geneva, Switzerland: World Health Organization (WHO), 2015
United Kingdom - Scotland Vital Registration - Deaths 2000 ICD10 as it appears in World Health Organization (WHO). WHO Mortality Database Version October 2017. Geneva, Switzerland: World Health Organization (WHO), 2017
United Kingdom - Scotland Vital Registration - Deaths 2001 ICD10 as it appears in World Health Organization (WHO). WHO Mortality Database Version October 2017. Geneva, Switzerland: World Health Organization (WHO), 2017
United Kingdom - Scotland Vital Registration - Deaths 2002 ICD10 as it appears in World Health Organization (WHO). WHO Mortality Database Version October 2017. Geneva, Switzerland: World Health Organization (WHO), 2017

United Kingdom - Scotland Vital Registration - Deaths 2003 ICD10 as it appears in World Health Organization (WHO). WHO Mortality Database Version October 2017. Geneva, Switzerland: World Health Organization (WHO), 2017

United Kingdom - Scotland Vital Registration - Deaths 2004 ICD10 as it appears in World Health Organization (WHO). WHO Mortality Database Version October 2017. Geneva, Switzerland: World Health Organization (WHO), 2017

United Kingdom - Scotland Vital Registration - Deaths 2005 ICD10 as it appears in World Health Organization (WHO). WHO Mortality Database Version October 2017. Geneva, Switzerland: World Health Organization (WHO), 2017

United Kingdom - Scotland Vital Registration - Deaths 2006 ICD10 as it appears in World Health Organization (WHO). WHO Mortality Database Version October 2017. Geneva, Switzerland: World Health Organization (WHO), 2017

United Kingdom - Scotland Vital Registration - Deaths 2007 ICD10 as it appears in World Health Organization (WHO). WHO Mortality Database Version October 2017. Geneva, Switzerland: World Health Organization (WHO), 2017

United Kingdom - Scotland Vital Registration - Deaths 2008 ICD10 as it appears in World Health Organization (WHO). WHO Mortality Database Version October 2017. Geneva, Switzerland: World Health Organization (WHO), 2017

United Kingdom - Scotland Vital Registration - Deaths 2009 ICD10 as it appears in World Health Organization (WHO). WHO Mortality Database Version October 2017. Geneva, Switzerland: World Health Organization (WHO), 2017

United Kingdom - Scotland Vital Registration - Deaths 2010 ICD10 as it appears in World Health Organization (WHO). WHO Mortality Database Version October 2017. Geneva, Switzerland: World Health Organization (WHO), 2017

United Kingdom - Scotland Vital Registration - Deaths 2011 ICD10 as it appears in World Health Organization (WHO). WHO Mortality Database Version October 2017. Geneva, Switzerland: World Health Organization (WHO), 2017

United Kingdom - Scotland Vital Registration - Deaths 2012 ICD10 as it appears in World Health Organization (WHO). WHO Mortality Database Version October 2017. Geneva, Switzerland: World Health Organization (WHO), 2017

United Kingdom - Scotland Vital Registration - Deaths 2013 ICD10 as it appears in World Health Organization (WHO). WHO Mortality Database Version October 2017. Geneva, Switzerland: World Health Organization (WHO), 2017

United Kingdom - Scotland Vital Registration - Deaths 2014 ICD10 as it appears in World Health Organization (WHO). WHO Mortality Database Version October 2017. Geneva, Switzerland: World Health Organization (WHO), 2017

United Nations Children's Fund (UNICEF). Infant Mortality in Tajikistan: Two Studies Look at Risk Factors. New York, United States: United Nations Children's Fund (UNICEF). (Child Research Digest, No. 4)

United States Virgin Islands Vital Registration - Deaths 1980 ICD9 as it appears in World Health Organization (WHO). WHO Mortality Database Version September 2016. Geneva, Switzerland: World Health Organization (WHO), 2016

Upadhyay RP, Rai SK, Krishnan A. Using three delays model to understand the social factors responsible for neonatal deaths in rural Haryana, India. J Trop Pediatr. 2013; 59(2): 100-5

Upadhyaya S, Shettyb S, Kumarc SS, Dongred A, Deshmukhe P. Institutionalizing district level infant death review: an experience from southern India. Southeast Asia J Public Health. 2012; 1(4): 446-56

Uruguay Vital Registration - Deaths 1980 ICD9 as it appears in World Health Organization (WHO). WHO Mortality Database Version September 2016. Geneva, Switzerland: World Health Organization (WHO), 2016

Uruguay Vital Registration - Deaths 1981 ICD9 as it appears in World Health Organization (WHO). WHO Mortality Database Version September 2016. Geneva, Switzerland: World Health Organization (WHO), 2016

Uruguay Vital Registration - Deaths 1982 ICD9 as it appears in World Health Organization (WHO). WHO Mortality Database Version September 2016. Geneva, Switzerland: World Health Organization (WHO), 2016
Uruguay Vital Registration - Deaths 1983 ICD9 as it appears in World Health Organization (WHO). WHO Mortality Database Version September 2016. Geneva, Switzerland: World Health Organization (WHO), 2016
Uruguay Vital Registration - Deaths 1984 ICD9 as it appears in World Health Organization (WHO). WHO Mortality Database Version September 2016. Geneva, Switzerland: World Health Organization (WHO), 2016
Uruguay Vital Registration - Deaths 1985 ICD9 as it appears in World Health Organization (WHO). WHO Mortality Database Version September 2016. Geneva, Switzerland: World Health Organization (WHO), 2016
Uruguay Vital Registration - Deaths 1986 ICD9 as it appears in World Health Organization (WHO). WHO Mortality Database Version September 2016. Geneva, Switzerland: World Health Organization (WHO), 2016
Uruguay Vital Registration - Deaths 1987 ICD9 as it appears in World Health Organization (WHO). WHO Mortality Database Version September 2016. Geneva, Switzerland: World Health Organization (WHO), 2016
Uruguay Vital Registration - Deaths 1989 ICD9 as it appears in World Health Organization (WHO). WHO Mortality Database Version September 2016. Geneva, Switzerland: World Health Organization (WHO), 2016
Uruguay Vital Registration - Deaths 1990 ICD9 as it appears in World Health Organization (WHO). WHO Mortality Database Version September 2016. Geneva, Switzerland: World Health Organization (WHO), 2016
Uruguay Vital Registration - Deaths 1993 ICD9 as it appears in World Health Organization (WHO). WHO Mortality Database Version September 2016. Geneva, Switzerland: World Health Organization (WHO), 2016
Uruguay Vital Registration - Deaths 1994 ICD9 as it appears in World Health Organization (WHO). WHO Mortality Database Version September 2016. Geneva, Switzerland: World Health Organization (WHO), 2016
Uruguay Vital Registration - Deaths 1995 ICD9 as it appears in World Health Organization (WHO). WHO Mortality Database Version September 2016. Geneva, Switzerland: World Health Organization (WHO), 2016
Uruguay Vital Registration - Deaths 1996 ICD9 as it appears in World Health Organization (WHO). WHO Mortality Database Version September 2016. Geneva, Switzerland: World Health Organization (WHO), 2016
Uruguay Vital Registration - Deaths 1997 ICD10 as it appears in World Health Organization (WHO). WHO Mortality Database Version October 2017. Geneva, Switzerland: World Health Organization (WHO), 2017
Uruguay Vital Registration - Deaths 1998 ICD10 as it appears in World Health Organization (WHO). WHO Mortality Database Version October 2017. Geneva, Switzerland: World Health Organization (WHO), 2017
Uruguay Vital Registration - Deaths 1999 ICD10 as it appears in World Health Organization (WHO). WHO Mortality Database Version October 2017. Geneva, Switzerland: World Health Organization (WHO), 2017
Uruguay Vital Registration - Deaths 2000 ICD10 as it appears in World Health Organization (WHO). WHO Mortality Database Version October 2017. Geneva, Switzerland: World Health Organization (WHO), 2017
Uruguay Vital Registration - Deaths 2001 ICD10 as it appears in World Health Organization (WHO). WHO Mortality Database Version October 2017. Geneva, Switzerland: World Health Organization (WHO), 2017
Uruguay Vital Registration - Deaths 2004 ICD10 as it appears in World Health Organization (WHO). WHO Mortality Database Version October 2017. Geneva, Switzerland: World Health Organization (WHO), 2017

Uruguay Vital Registration - Deaths 1988 ICD9 as it appears in World Health Organization (WHO). WHO Mortality Database Version September 2016. Geneva, Switzerland: World Health Organization (WHO), 2016

Uruguay Vital Registration - Deaths 2002 ICD10 as it appears in World Health Organization (WHO). WHO Mortality Database Version October 2017. Geneva, Switzerland: World Health Organization (WHO), 2017

Uruguay Vital Registration - Deaths 2003 ICD10 as it appears in World Health Organization (WHO). WHO Mortality Database Version October 2017. Geneva, Switzerland: World Health Organization (WHO), 2017

Uruguay Vital Registration - Deaths 2005 ICD10 as it appears in World Health Organization (WHO). WHO Mortality Database Version October 2017. Geneva, Switzerland: World Health Organization (WHO), 2017

Uruguay Vital Registration - Deaths 2006 ICD10 as it appears in World Health Organization (WHO). WHO Mortality Database Version October 2017. Geneva, Switzerland: World Health Organization (WHO), 2017

Uruguay Vital Registration - Deaths 2007 ICD10 as it appears in World Health Organization (WHO). WHO Mortality Database Version October 2017. Geneva, Switzerland: World Health Organization (WHO), 2017

Uruguay Vital Registration - Deaths 2008 ICD10 as it appears in World Health Organization (WHO). WHO Mortality Database Version October 2017. Geneva, Switzerland: World Health Organization (WHO), 2017

Uruguay Vital Registration - Deaths 2009 ICD10 as it appears in World Health Organization (WHO). WHO Mortality Database Version October 2017. Geneva, Switzerland: World Health Organization (WHO), 2017

Uruguay Vital Registration - Deaths 2010 ICD10 as it appears in World Health Organization (WHO). WHO Mortality Database Version October 2017. Geneva, Switzerland: World Health Organization (WHO), 2017

Uruguay Vital Registration - Deaths 2012 ICD10 as it appears in World Health Organization (WHO). WHO Mortality Database Version October 2017. Geneva, Switzerland: World Health Organization (WHO), 2017

Uruguay Vital Registration - Deaths 2013 ICD10 as it appears in World Health Organization (WHO). WHO Mortality Database Version October 2017. Geneva, Switzerland: World Health Organization (WHO), 2017

Uruguay Vital Registration - Deaths 2014 ICD10 as it appears in World Health Organization (WHO). WHO Mortality Database Version October 2017. Geneva, Switzerland: World Health Organization (WHO), 2017

Uruguay Vital Registration - Deaths 2015 ICD10 as it appears in World Health Organization (WHO). WHO Mortality Database Version October 2017. Geneva, Switzerland: World Health Organization (WHO), 2017

USSR - Russia Mortality by Region, Age, Sex, and Cause of Death 1980 as it appears in Center for Demographic Research, New Economic School (Russia). USSR - Russia Mortality Rates by Region, Age, Sex, and Cause of Death 1969-1989. Moscow, Russia: Center for Demographic Research, New Economic School (Russia). http://demogr.nes.ru/index.php/ru/demogr_indicat/data

Uzbekistan Vital Registration - Deaths 1981 ICD9 as it appears in World Health Organization (WHO). WHO Mortality Database Version September 2016. Geneva, Switzerland: World Health Organization (WHO), 2016

Uzbekistan Vital Registration - Deaths 1982 ICD9 as it appears in World Health Organization (WHO). WHO Mortality Database Version September 2016. Geneva, Switzerland: World Health Organization (WHO), 2016

Uzbekistan Vital Registration - Deaths 1985 ICD9 as it appears in World Health Organization (WHO). WHO Mortality Database Version September 2016. Geneva, Switzerland: World Health Organization (WHO), 2016

Uzbekistan Vital Registration - Deaths 1986 ICD9 as it appears in World Health Organization (WHO). WHO Mortality Database Version September 2016. Geneva, Switzerland: World Health Organization (WHO), 2016
Uzbekistan Vital Registration - Deaths 1987 ICD9 as it appears in World Health Organization (WHO). WHO Mortality Database Version September 2016. Geneva, Switzerland: World Health Organization (WHO), 2016
Uzbekistan Vital Registration - Deaths 1988 ICD9 as it appears in World Health Organization (WHO). WHO Mortality Database Version September 2016. Geneva, Switzerland: World Health Organization (WHO), 2016
Uzbekistan Vital Registration - Deaths 1989 ICD9 as it appears in World Health Organization (WHO). WHO Mortality Database Version September 2016. Geneva, Switzerland: World Health Organization (WHO), 2016
Uzbekistan Vital Registration - Deaths 1990 ICD9 as it appears in World Health Organization (WHO). WHO Mortality Database Version September 2016. Geneva, Switzerland: World Health Organization (WHO), 2016
Uzbekistan Vital Registration - Deaths 1991 ICD9 as it appears in World Health Organization (WHO). WHO Mortality Database Version September 2016. Geneva, Switzerland: World Health Organization (WHO), 2016
Uzbekistan Vital Registration - Deaths 1992 ICD9 as it appears in World Health Organization (WHO). WHO Mortality Database Version September 2016. Geneva, Switzerland: World Health Organization (WHO), 2016
Uzbekistan Vital Registration - Deaths 1993 ICD9 as it appears in World Health Organization (WHO). WHO Mortality Database Version September 2016. Geneva, Switzerland: World Health Organization (WHO), 2016
Uzbekistan Vital Registration - Deaths 1994 ICD9 as it appears in World Health Organization (WHO). WHO Mortality Database Version September 2016. Geneva, Switzerland: World Health Organization (WHO), 2016
Uzbekistan Vital Registration - Deaths 1995 ICD9 as it appears in World Health Organization (WHO). WHO Mortality Database Version September 2016. Geneva, Switzerland: World Health Organization (WHO), 2016
Uzbekistan Vital Registration - Deaths 1996 ICD9 as it appears in World Health Organization (WHO). WHO Mortality Database Version September 2016. Geneva, Switzerland: World Health Organization (WHO), 2016
Uzbekistan Vital Registration - Deaths 1997 ICD9 as it appears in World Health Organization (WHO). WHO Mortality Database Version September 2016. Geneva, Switzerland: World Health Organization (WHO), 2016
Uzbekistan Vital Registration - Deaths 1998 ICD9 as it appears in World Health Organization (WHO). WHO Mortality Database Version September 2016. Geneva, Switzerland: World Health Organization (WHO), 2016
Uzbekistan Vital Registration - Deaths 1999 ICD9 as it appears in World Health Organization (WHO). WHO Mortality Database Version September 2016. Geneva, Switzerland: World Health Organization (WHO), 2016
Uzbekistan Vital Registration - Deaths 2000 ICD9 as it appears in World Health Organization (WHO). WHO Mortality Database Version September 2016. Geneva, Switzerland: World Health Organization (WHO), 2016
Uzbekistan Vital Registration - Deaths 2001 ICD9 as it appears in World Health Organization (WHO). WHO Mortality Database Version September 2016. Geneva, Switzerland: World Health Organization (WHO), 2016
Uzbekistan Vital Registration - Deaths 2002 ICD9 as it appears in World Health Organization (WHO). WHO Mortality Database Version September 2016. Geneva, Switzerland: World Health Organization (WHO), 2016
Uzbekistan Vital Registration - Deaths 2003 ICD9 as it appears in World Health Organization (WHO). WHO Mortality Database Version September 2016. Geneva, Switzerland: World Health Organization (WHO), 2016

Uzbekistan Vital Registration - Deaths 2004 ICD10 as it appears in World Health Organization (WHO). WHO Mortality Database Version October 2017. Geneva, Switzerland: World Health Organization (WHO), 2017

Uzbekistan Vital Registration - Deaths 2005 ICD10 as it appears in World Health Organization (WHO). WHO Mortality Database Version October 2017. Geneva, Switzerland: World Health Organization (WHO), 2017

Uzbekistan Vital Registration - Deaths 2009 ICD10 as it appears in World Health Organization (WHO). WHO Mortality Database Version March 2017. Geneva, Switzerland: World Health Organization (WHO), 2017

Uzbekistan Vital Registration - Deaths 2010 ICD10 as it appears in World Health Organization (WHO). WHO Mortality Database Version March 2017. Geneva, Switzerland: World Health Organization (WHO), 2017

Uzbekistan Vital Registration - Deaths 2011 ICD10 as it appears in World Health Organization (WHO). WHO Mortality Database Version March 2017. Geneva, Switzerland: World Health Organization (WHO), 2017

Uzbekistan Vital Registration - Deaths 2012 ICD10 as it appears in World Health Organization (WHO). WHO Mortality Database Version March 2017. Geneva, Switzerland: World Health Organization (WHO), 2017

Uzbekistan Vital Registration - Deaths 2013 ICD10 as it appears in World Health Organization (WHO). WHO Mortality Database Version March 2017. Geneva, Switzerland: World Health Organization (WHO), 2017

Uzbekistan Vital Registration - Deaths 2014 ICD10 as it appears in World Health Organization (WHO). WHO Mortality Database Version October 2017. Geneva, Switzerland: World Health Organization (WHO), 2017

Vaid A, Mammen A, Primrose B, Kang G. Infant mortality in an urban slum. Indian J Pediatr. 2007; 74(5): 449-53

Venezuela Vital Registration - Deaths 1980 ICD9 as it appears in World Health Organization (WHO). WHO Mortality Database Version September 2016. Geneva, Switzerland: World Health Organization (WHO), 2016

Venezuela Vital Registration - Deaths 1981 ICD9 as it appears in World Health Organization (WHO). WHO Mortality Database Version September 2016. Geneva, Switzerland: World Health Organization (WHO), 2016

Venezuela Vital Registration - Deaths 1982 ICD9 as it appears in World Health Organization (WHO). WHO Mortality Database Version September 2016. Geneva, Switzerland: World Health Organization (WHO), 2016

Venezuela Vital Registration - Deaths 1983 ICD9 as it appears in World Health Organization (WHO). WHO Mortality Database Version September 2016. Geneva, Switzerland: World Health Organization (WHO), 2016

Venezuela Vital Registration - Deaths 1985 ICD9 as it appears in World Health Organization (WHO). WHO Mortality Database Version September 2016. Geneva, Switzerland: World Health Organization (WHO), 2016

Venezuela Vital Registration - Deaths 1986 ICD9 as it appears in World Health Organization (WHO). WHO Mortality Database Version September 2016. Geneva, Switzerland: World Health Organization (WHO), 2016

Venezuela Vital Registration - Deaths 1987 ICD9 as it appears in World Health Organization (WHO). WHO Mortality Database Version September 2016. Geneva, Switzerland: World Health Organization (WHO), 2016

Venezuela Vital Registration - Deaths 1988 ICD9 as it appears in World Health Organization (WHO). WHO Mortality Database Version September 2016. Geneva, Switzerland: World Health Organization (WHO), 2016

Venezuela Vital Registration - Deaths 1989 ICD9 as it appears in World Health Organization (WHO). WHO Mortality Database Version September 2016. Geneva, Switzerland: World Health Organization (WHO), 2016

Venezuela Vital Registration - Deaths 1990 ICD9 as it appears in World Health Organization (WHO). WHO Mortality Database Version September 2016. Geneva, Switzerland: World Health Organization (WHO), 2016
Venezuela Vital Registration - Deaths 1992 ICD9 as it appears in World Health Organization (WHO). WHO Mortality Database Version September 2016. Geneva, Switzerland: World Health Organization (WHO), 2016
Venezuela Vital Registration - Deaths 1993 ICD9 as it appears in World Health Organization (WHO). WHO Mortality Database Version September 2016. Geneva, Switzerland: World Health Organization (WHO), 2016
Venezuela Vital Registration - Deaths 1994 ICD9 as it appears in World Health Organization (WHO). WHO Mortality Database Version September 2016. Geneva, Switzerland: World Health Organization (WHO), 2016
Venezuela Vital Registration - Deaths 1996 ICD10 as it appears in World Health Organization (WHO). WHO Mortality Database Version October 2017. Geneva, Switzerland: World Health Organization (WHO), 2017
Venezuela Vital Registration - Deaths 1997 ICD10 as it appears in World Health Organization (WHO). WHO Mortality Database Version October 2017. Geneva, Switzerland: World Health Organization (WHO), 2017
Venezuela Vital Registration - Deaths 1998 ICD10 as it appears in World Health Organization (WHO). WHO Mortality Database Version October 2017. Geneva, Switzerland: World Health Organization (WHO), 2017
Venezuela Vital Registration - Deaths 1999 ICD10 as it appears in World Health Organization (WHO). WHO Mortality Database Version October 2017. Geneva, Switzerland: World Health Organization (WHO), 2017
Venezuela Vital Registration - Deaths 2000 ICD10 as it appears in World Health Organization (WHO). WHO Mortality Database Version October 2017. Geneva, Switzerland: World Health Organization (WHO), 2017
Venezuela Vital Registration - Deaths 2001 ICD10 as it appears in World Health Organization (WHO). WHO Mortality Database Version October 2017. Geneva, Switzerland: World Health Organization (WHO), 2017
Venezuela Vital Registration - Deaths 2002 ICD10 as it appears in World Health Organization (WHO). WHO Mortality Database Version October 2017. Geneva, Switzerland: World Health Organization (WHO), 2017
Venezuela Vital Registration - Deaths 2003 ICD10 as it appears in World Health Organization (WHO). WHO Mortality Database Version October 2017. Geneva, Switzerland: World Health Organization (WHO), 2017
Venezuela Vital Registration - Deaths 2004 ICD10 as it appears in World Health Organization (WHO). WHO Mortality Database Version October 2017. Geneva, Switzerland: World Health Organization (WHO), 2017
Venezuela Vital Registration - Deaths 2005 ICD10 as it appears in World Health Organization (WHO). WHO Mortality Database Version October 2017. Geneva, Switzerland: World Health Organization (WHO), 2017
Venezuela Vital Registration - Deaths 2006 ICD10 as it appears in World Health Organization (WHO). WHO Mortality Database Version October 2017. Geneva, Switzerland: World Health Organization (WHO), 2017
Venezuela Vital Registration - Deaths 2007 ICD10 as it appears in World Health Organization (WHO). WHO Mortality Database Version October 2017. Geneva, Switzerland: World Health Organization (WHO), 2017
Venezuela Vital Registration - Deaths 2008 ICD10 as it appears in World Health Organization (WHO). WHO Mortality Database Version October 2017. Geneva, Switzerland: World Health Organization (WHO), 2017
Venezuela Vital Registration - Deaths 2009 ICD10 as it appears in World Health Organization (WHO). WHO Mortality Database Version October 2017. Geneva, Switzerland: World Health Organization (WHO), 2017

Venezuela Vital Registration - Deaths 2010 ICD10 as it appears in World Health Organization (WHO). WHO Mortality Database Version October 2017. Geneva, Switzerland: World Health Organization (WHO), 2017

Venezuela Vital Registration - Deaths 2011 ICD10 as it appears in World Health Organization (WHO). WHO Mortality Database Version October 2017. Geneva, Switzerland: World Health Organization (WHO), 2017

Venezuela Vital Registration - Deaths 2012 ICD10 as it appears in World Health Organization (WHO). WHO Mortality Database Version October 2017. Geneva, Switzerland: World Health Organization (WHO), 2017

Venezuela Vital Registration - Deaths 2013 ICD10 as it appears in World Health Organization (WHO). WHO Mortality Database Version October 2017. Geneva, Switzerland: World Health Organization (WHO), 2017

Victora CG, Barros FC, Huttly SR, Teixeira AM, Vaughan JP. Early childhood mortality in a Brazilian cohort: the roles of birthweight and socioeconomic status. Int J Epidemiol. 1992; 21(5): 911-5

Waltisperger D, Cantrelle P, Ralijaona J, Population and Development Research Center (CEPED) (France). Madagascar - Antananorivo Mortality Report 1984-1995. Paris, France: Population and Development Research Center (CEPED) (France), 1998

West KP Jr, Christian P, Labrique AB, Rashid M, Shamim AA, Klemm RD, Massie AB, Mehra S, Schulze KJ, Ali H, Ullah B, Wu LS, Katz J, Banu H, Akhter HH, Sommer A. Effects of vitamin A or beta carotene supplementation on pregnancy-related mortality and infant mortality in rural Bangladesh: a cluster randomized trial. JAMA. 2011; 305(19): 1986-95

Würthwein R, Gbangou A, Sauerborn R, Schmidt CM. Measuring the local burden of disease. A study of years of life lost in sub-Saharan Africa. Int J Epidemiol. 2001; 30(3): 501-8

Yugoslavia - Bosnia and Herzegovina Vital Registration - Deaths 1985 ICD9 as it appears in World Health Organization (WHO). WHO Mortality Database Version September 2016. Geneva, Switzerland: World Health Organization (WHO), 2016

Yugoslavia - Bosnia and Herzegovina Vital Registration - Deaths 1986 ICD9 as it appears in World Health Organization (WHO). WHO Mortality Database Version September 2016. Geneva, Switzerland: World Health Organization (WHO), 2016

Yugoslavia - Bosnia and Herzegovina Vital Registration - Deaths 1987 ICD9 as it appears in World Health Organization (WHO). WHO Mortality Database Version September 2016. Geneva, Switzerland: World Health Organization (WHO), 2016

Yugoslavia - Bosnia and Herzegovina Vital Registration - Deaths 1988 ICD9 as it appears in World Health Organization (WHO). WHO Mortality Database Version September 2016. Geneva, Switzerland: World Health Organization (WHO), 2016

Yugoslavia - Bosnia and Herzegovina Vital Registration - Deaths 1989 ICD9 as it appears in World Health Organization (WHO). WHO Mortality Database Version September 2016. Geneva, Switzerland: World Health Organization (WHO), 2016

Yugoslavia - Bosnia and Herzegovina Vital Registration - Deaths 1990 ICD9 as it appears in World Health Organization (WHO). WHO Mortality Database Version September 2016. Geneva, Switzerland: World Health Organization (WHO), 2016

Yugoslavia - Croatia Vital Registration - Deaths 1985 ICD9 as it appears in World Health Organization (WHO). WHO Mortality Database Version November 2015. Geneva, Switzerland: World Health Organization (WHO), 2015

Yugoslavia - Croatia Vital Registration - Deaths 1986 ICD9 as it appears in World Health Organization (WHO). WHO Mortality Database Version November 2015. Geneva, Switzerland: World Health Organization (WHO), 2015

Yugoslavia - Croatia Vital Registration - Deaths 1987 ICD9 as it appears in World Health Organization (WHO). WHO Mortality Database Version November 2015. Geneva, Switzerland: World Health Organization (WHO), 2015

Yugoslavia - Croatia Vital Registration - Deaths 1988 ICD9 as it appears in World Health Organization (WHO). WHO Mortality Database Version November 2015. Geneva, Switzerland: World Health Organization (WHO), 2015
Yugoslavia - Croatia Vital Registration - Deaths 1989 ICD9 as it appears in World Health Organization (WHO). WHO Mortality Database Version November 2015. Geneva, Switzerland: World Health Organization (WHO), 2015
Yugoslavia - Croatia Vital Registration - Deaths 1990 ICD9 as it appears in World Health Organization (WHO). WHO Mortality Database Version November 2015. Geneva, Switzerland: World Health Organization (WHO), 2015
Yugoslavia - Slovenia Vital Registration - Deaths 1985 ICD9 as it appears in World Health Organization (WHO). WHO Mortality Database Version November 2015. Geneva, Switzerland: World Health Organization (WHO), 2015
Yugoslavia - Slovenia Vital Registration - Deaths 1986 ICD9 as it appears in World Health Organization (WHO). WHO Mortality Database Version November 2015. Geneva, Switzerland: World Health Organization (WHO), 2015
Yugoslavia - Slovenia Vital Registration - Deaths 1987 ICD9 as it appears in World Health Organization (WHO). WHO Mortality Database Version November 2015. Geneva, Switzerland: World Health Organization (WHO), 2015
Yugoslavia - Slovenia Vital Registration - Deaths 1988 ICD9 as it appears in World Health Organization (WHO). WHO Mortality Database Version November 2015. Geneva, Switzerland: World Health Organization (WHO), 2015
Yugoslavia - Slovenia Vital Registration - Deaths 1990 ICD9 as it appears in World Health Organization (WHO). WHO Mortality Database Version November 2015. Geneva, Switzerland: World Health Organization (WHO), 2015
Yugoslavia, Federal Republic - Montenegro Vital Registration - Deaths 2000 ICD10 as it appears in World Health Organization (WHO). WHO Mortality Database Version October 2017. Geneva, Switzerland: World Health Organization (WHO), 2017
Yugoslavia, Federal Republic - Serbia Vital Registration - Deaths 1998 ICD10 as it appears in World Health Organization (WHO). WHO Mortality Database Version October 2017. Geneva, Switzerland: World Health Organization (WHO), 2017
Yugoslavia, Federal Republic - Serbia Vital Registration - Deaths 1999 ICD10 as it appears in World Health Organization (WHO). WHO Mortality Database Version October 2017. Geneva, Switzerland: World Health Organization (WHO), 2017
Yugoslavia, Federal Republic - Serbia Vital Registration - Deaths 2000 ICD10 as it appears in World Health Organization (WHO). WHO Mortality Database Version October 2017. Geneva, Switzerland: World Health Organization (WHO), 2017
Yugoslavia, Federal Republic - Serbia Vital Registration - Deaths 2001 ICD10 as it appears in World Health Organization (WHO). WHO Mortality Database Version October 2017. Geneva, Switzerland: World Health Organization (WHO), 2017
Yugoslavia, Federal Republic - Serbia Vital Registration - Deaths 2002 ICD10 as it appears in World Health Organization (WHO). WHO Mortality Database Version October 2017. Geneva, Switzerland: World Health Organization (WHO), 2017
Zimbabwe Vital Registration - Deaths 1990 ICD9 as it appears in World Health Organization (WHO). WHO Mortality Database Version September 2016. Geneva, Switzerland: World Health Organization (WHO), 2016

Table S14: Alphabetical listing of all data sources used in estimating cause-specific mortality due to congenital heart anomalies for GBD 2017

Citation from Global Health Data Exchange (http://ghdx.healthdata.org/gbd-2017/data-input-sources)
Albania Vital Registration - Deaths 1987 ICD9 as it appears in World Health Organization (WHO). WHO Mortality Database Version November 2015. Geneva, Switzerland: World Health Organization (WHO), 2015
Albania Vital Registration - Deaths 1988 ICD9 as it appears in World Health Organization (WHO). WHO Mortality Database Version November 2015. Geneva, Switzerland: World Health Organization (WHO), 2015
Albania Vital Registration - Deaths 1989 ICD9 as it appears in World Health Organization (WHO). WHO Mortality Database Version November 2015. Geneva, Switzerland: World Health Organization (WHO), 2015
Albania Vital Registration - Deaths 1992 ICD9 as it appears in World Health Organization (WHO). WHO Mortality Database Version November 2015. Geneva, Switzerland: World Health Organization (WHO), 2015
Albania Vital Registration - Deaths 1993 ICD9 as it appears in World Health Organization (WHO). WHO Mortality Database Version November 2015. Geneva, Switzerland: World Health Organization (WHO), 2015
Albania Vital Registration - Deaths 1994 ICD9 as it appears in World Health Organization (WHO). WHO Mortality Database Version November 2015. Geneva, Switzerland: World Health Organization (WHO), 2015
Albania Vital Registration - Deaths 1995 ICD9 as it appears in World Health Organization (WHO). WHO Mortality Database Version November 2015. Geneva, Switzerland: World Health Organization (WHO), 2015
Albania Vital Registration - Deaths 1996 ICD9 as it appears in World Health Organization (WHO). WHO Mortality Database Version November 2015. Geneva, Switzerland: World Health Organization (WHO), 2015
Albania Vital Registration - Deaths 1997 ICD9 as it appears in World Health Organization (WHO). WHO Mortality Database Version November 2015. Geneva, Switzerland: World Health Organization (WHO), 2015
Albania Vital Registration - Deaths 1998 ICD9 as it appears in World Health Organization (WHO). WHO Mortality Database Version November 2015. Geneva, Switzerland: World Health Organization (WHO), 2015
Albania Vital Registration - Deaths 1999 ICD9 as it appears in World Health Organization (WHO). WHO Mortality Database Version November 2015. Geneva, Switzerland: World Health Organization (WHO), 2015
Albania Vital Registration - Deaths 2000 ICD9 as it appears in World Health Organization (WHO). WHO Mortality Database Version November 2015. Geneva, Switzerland: World Health Organization (WHO), 2015
Albania Vital Registration - Deaths 2001 ICD9 as it appears in World Health Organization (WHO). WHO Mortality Database Version November 2015. Geneva, Switzerland: World Health Organization (WHO), 2015
Albania Vital Registration - Deaths 2002 ICD9 as it appears in World Health Organization (WHO). WHO Mortality Database Version November 2015. Geneva, Switzerland: World Health Organization (WHO), 2015
Albania Vital Registration - Deaths 2003 ICD9 as it appears in World Health Organization (WHO). WHO Mortality Database Version November 2015. Geneva, Switzerland: World Health Organization (WHO), 2015
Albania Vital Registration - Deaths 2004 ICD9 as it appears in World Health Organization (WHO). WHO Mortality Database Version November 2015. Geneva, Switzerland: World Health Organization (WHO), 2015
Albania Vital Registration - Deaths 2005 ICD9 as it appears in World Health Organization (WHO). WHO Mortality Database Version September 2016. Geneva, Switzerland: World Health Organization (WHO), 2016
Albania Vital Registration - Deaths 2006 ICD9 as it appears in World Health Organization (WHO). WHO Mortality Database Version September 2016. Geneva, Switzerland: World Health Organization (WHO), 2016

Albania Vital Registration - Deaths 2007 ICD9 as it appears in World Health Organization (WHO). WHO Mortality Database Version September 2016. Geneva, Switzerland: World Health Organization (WHO), 2016
Albania Vital Registration - Deaths 2008 ICD9 as it appears in World Health Organization (WHO). WHO Mortality Database Version September 2016. Geneva, Switzerland: World Health Organization (WHO), 2016
Albania Vital Registration - Deaths 2009 ICD9 as it appears in World Health Organization (WHO). WHO Mortality Database Version September 2016. Geneva, Switzerland: World Health Organization (WHO), 2016
Albania Vital Registration - Deaths 2010 ICD9 as it appears in World Health Organization (WHO). WHO Mortality Database Version March 2017. Geneva, Switzerland: World Health Organization (WHO), 2017
Antigua and Barbuda Vital Registration - Deaths 1983 ICD9 as it appears in World Health Organization (WHO). WHO Mortality Database Version September 2016. Geneva, Switzerland: World Health Organization (WHO), 2016
Antigua and Barbuda Vital Registration - Deaths 1986 ICD9 as it appears in World Health Organization (WHO). WHO Mortality Database Version September 2016. Geneva, Switzerland: World Health Organization (WHO), 2016
Antigua and Barbuda Vital Registration - Deaths 1987 ICD9 as it appears in World Health Organization (WHO). WHO Mortality Database Version September 2016. Geneva, Switzerland: World Health Organization (WHO), 2016
Antigua and Barbuda Vital Registration - Deaths 1988 ICD9 as it appears in World Health Organization (WHO). WHO Mortality Database Version September 2016. Geneva, Switzerland: World Health Organization (WHO), 2016
Antigua and Barbuda Vital Registration - Deaths 1989 ICD9 as it appears in World Health Organization (WHO). WHO Mortality Database Version September 2016. Geneva, Switzerland: World Health Organization (WHO), 2016
Antigua and Barbuda Vital Registration - Deaths 1990 ICD9 as it appears in World Health Organization (WHO). WHO Mortality Database Version September 2016. Geneva, Switzerland: World Health Organization (WHO), 2016
Antigua and Barbuda Vital Registration - Deaths 1991 ICD9 as it appears in World Health Organization (WHO). WHO Mortality Database Version September 2016. Geneva, Switzerland: World Health Organization (WHO), 2016
Antigua and Barbuda Vital Registration - Deaths 1992 ICD9 as it appears in World Health Organization (WHO). WHO Mortality Database Version September 2016. Geneva, Switzerland: World Health Organization (WHO), 2016
Antigua and Barbuda Vital Registration - Deaths 1993 ICD9 as it appears in World Health Organization (WHO). WHO Mortality Database Version September 2016. Geneva, Switzerland: World Health Organization (WHO), 2016
Antigua and Barbuda Vital Registration - Deaths 1994 ICD9 as it appears in World Health Organization (WHO). WHO Mortality Database Version September 2016. Geneva, Switzerland: World Health Organization (WHO), 2016
Antigua and Barbuda Vital Registration - Deaths 1995 ICD9 as it appears in World Health Organization (WHO). WHO Mortality Database Version September 2016. Geneva, Switzerland: World Health Organization (WHO), 2016
Antigua and Barbuda Vital Registration - Deaths 2000 ICD10 as it appears in World Health Organization (WHO). WHO Mortality Database Version October 2017. Geneva, Switzerland: World Health Organization (WHO), 2017
Antigua and Barbuda Vital Registration - Deaths 2001 ICD10 as it appears in World Health Organization (WHO). WHO Mortality Database Version October 2017. Geneva, Switzerland: World Health Organization (WHO), 2017
Antigua and Barbuda Vital Registration - Deaths 2002 ICD10 as it appears in World Health Organization (WHO). WHO Mortality Database Version October 2017. Geneva, Switzerland: World Health Organization (WHO), 2017
Antigua and Barbuda Vital Registration - Deaths 2003 ICD10 as it appears in World Health Organization (WHO). WHO Mortality Database Version October 2017. Geneva, Switzerland: World Health Organization (WHO), 2017
Antigua and Barbuda Vital Registration - Deaths 2004 ICD10 as it appears in World Health Organization (WHO). WHO Mortality Database Version October 2017. Geneva, Switzerland: World Health Organization (WHO), 2017

Antigua and Barbuda Vital Registration - Deaths 2005 ICD10 as it appears in World Health Organization (WHO). WHO Mortality Database Version October 2017. Geneva, Switzerland: World Health Organization (WHO), 2017
Antigua and Barbuda Vital Registration - Deaths 2006 ICD10 as it appears in World Health Organization (WHO). WHO Mortality Database Version October 2017. Geneva, Switzerland: World Health Organization (WHO), 2017
Antigua and Barbuda Vital Registration - Deaths 1985 ICD9 as it appears in World Health Organization (WHO). WHO Mortality Database Version September 2016. Geneva, Switzerland: World Health Organization (WHO), 2016
Antigua and Barbuda Vital Registration - Deaths 2007 ICD10 as it appears in World Health Organization (WHO). WHO Mortality Database Version October 2017. Geneva, Switzerland: World Health Organization (WHO), 2017
Antigua and Barbuda Vital Registration - Deaths 2008 ICD10 as it appears in World Health Organization (WHO). WHO Mortality Database Version October 2017. Geneva, Switzerland: World Health Organization (WHO), 2017
Antigua and Barbuda Vital Registration - Deaths 2009 ICD10 as it appears in World Health Organization (WHO). WHO Mortality Database Version October 2017. Geneva, Switzerland: World Health Organization (WHO), 2017
Antigua and Barbuda Vital Registration - Deaths 2012 ICD10 as it appears in World Health Organization (WHO). WHO Mortality Database Version October 2017. Geneva, Switzerland: World Health Organization (WHO), 2017
Antigua and Barbuda Vital Registration - Deaths 2013 ICD10 as it appears in World Health Organization (WHO). WHO Mortality Database Version October 2017. Geneva, Switzerland: World Health Organization (WHO), 2017
Antigua and Barbuda Vital Registration - Deaths 2014 ICD10 as it appears in World Health Organization (WHO). WHO Mortality Database Version October 2017. Geneva, Switzerland: World Health Organization (WHO), 2017
Antigua and Barbuda Vital Registration - Deaths 2015 ICD10 as it appears in World Health Organization (WHO). WHO Mortality Database Version October 2017. Geneva, Switzerland: World Health Organization (WHO), 2017
Argentina Vital Registration - Deaths 1980 ICD9 as it appears in World Health Organization (WHO). WHO Mortality Database Version September 2016. Geneva, Switzerland: World Health Organization (WHO), 2016
Argentina Vital Registration - Deaths 1981 ICD9 as it appears in World Health Organization (WHO). WHO Mortality Database Version September 2016. Geneva, Switzerland: World Health Organization (WHO), 2016
Argentina Vital Registration - Deaths 1982 ICD9 as it appears in World Health Organization (WHO). WHO Mortality Database Version September 2016. Geneva, Switzerland: World Health Organization (WHO), 2016
Argentina Vital Registration - Deaths 1983 ICD9 as it appears in World Health Organization (WHO). WHO Mortality Database Version September 2016. Geneva, Switzerland: World Health Organization (WHO), 2016
Argentina Vital Registration - Deaths 1984 ICD9 as it appears in World Health Organization (WHO). WHO Mortality Database Version September 2016. Geneva, Switzerland: World Health Organization (WHO), 2016
Argentina Vital Registration - Deaths 1985 ICD9 as it appears in World Health Organization (WHO). WHO Mortality Database Version September 2016. Geneva, Switzerland: World Health Organization (WHO), 2016
Argentina Vital Registration - Deaths 1986 ICD9 as it appears in World Health Organization (WHO). WHO Mortality Database Version September 2016. Geneva, Switzerland: World Health Organization (WHO), 2016
Argentina Vital Registration - Deaths 1987 ICD9 as it appears in World Health Organization (WHO). WHO Mortality Database Version September 2016. Geneva, Switzerland: World Health Organization (WHO), 2016
Argentina Vital Registration - Deaths 1988 ICD9 as it appears in World Health Organization (WHO). WHO Mortality Database Version September 2016. Geneva, Switzerland: World Health Organization (WHO), 2016
Argentina Vital Registration - Deaths 1989 ICD9 as it appears in World Health Organization (WHO). WHO Mortality Database Version September 2016. Geneva, Switzerland: World Health Organization (WHO), 2016

Argentina Vital Registration - Deaths 1990 ICD9 as it appears in World Health Organization (WHO). WHO Mortality Database Version September 2016. Geneva, Switzerland: World Health Organization (WHO), 2016
Argentina Vital Registration - Deaths 1991 ICD9 as it appears in World Health Organization (WHO). WHO Mortality Database Version September 2016. Geneva, Switzerland: World Health Organization (WHO), 2016
Argentina Vital Registration - Deaths 1992 ICD9 as it appears in World Health Organization (WHO). WHO Mortality Database Version September 2016. Geneva, Switzerland: World Health Organization (WHO), 2016
Argentina Vital Registration - Deaths 1993 ICD9 as it appears in World Health Organization (WHO). WHO Mortality Database Version September 2016. Geneva, Switzerland: World Health Organization (WHO), 2016
Argentina Vital Registration - Deaths 1994 ICD9 as it appears in World Health Organization (WHO). WHO Mortality Database Version September 2016. Geneva, Switzerland: World Health Organization (WHO), 2016
Argentina Vital Registration - Deaths 1995 ICD9 as it appears in World Health Organization (WHO). WHO Mortality Database Version September 2016. Geneva, Switzerland: World Health Organization (WHO), 2016
Argentina Vital Registration - Deaths 1996 ICD9 as it appears in World Health Organization (WHO). WHO Mortality Database Version September 2016. Geneva, Switzerland: World Health Organization (WHO), 2016
Argentina Vital Registration - Deaths 1997 ICD10 as it appears in World Health Organization (WHO). WHO Mortality Database Version October 2017. Geneva, Switzerland: World Health Organization (WHO), 2017
Argentina Vital Registration - Deaths 1998 ICD10 as it appears in World Health Organization (WHO). WHO Mortality Database Version October 2017. Geneva, Switzerland: World Health Organization (WHO), 2017
Argentina Vital Registration - Deaths 1999 ICD10 as it appears in World Health Organization (WHO). WHO Mortality Database Version October 2017. Geneva, Switzerland: World Health Organization (WHO), 2017
Argentina Vital Registration - Deaths 2000 ICD10 as it appears in World Health Organization (WHO). WHO Mortality Database Version October 2017. Geneva, Switzerland: World Health Organization (WHO), 2017
Argentina Vital Registration - Deaths 2001 ICD10 as it appears in World Health Organization (WHO). WHO Mortality Database Version October 2017. Geneva, Switzerland: World Health Organization (WHO), 2017
Argentina Vital Registration - Deaths 2002 ICD10 as it appears in World Health Organization (WHO). WHO Mortality Database Version October 2017. Geneva, Switzerland: World Health Organization (WHO), 2017
Argentina Vital Registration - Deaths 2003 ICD10 as it appears in World Health Organization (WHO). WHO Mortality Database Version October 2017. Geneva, Switzerland: World Health Organization (WHO), 2017
Argentina Vital Registration - Deaths 2004 ICD10 as it appears in World Health Organization (WHO). WHO Mortality Database Version October 2017. Geneva, Switzerland: World Health Organization (WHO), 2017
Argentina Vital Registration - Deaths 2005 ICD10 as it appears in World Health Organization (WHO). WHO Mortality Database Version October 2017. Geneva, Switzerland: World Health Organization (WHO), 2017
Argentina Vital Registration - Deaths 2006 ICD10 as it appears in World Health Organization (WHO). WHO Mortality Database Version October 2017. Geneva, Switzerland: World Health Organization (WHO), 2017
Argentina Vital Registration - Deaths 2007 ICD10 as it appears in World Health Organization (WHO). WHO Mortality Database Version October 2017. Geneva, Switzerland: World Health Organization (WHO), 2017
Argentina Vital Registration - Deaths 2008 ICD10 as it appears in World Health Organization (WHO). WHO Mortality Database Version October 2017. Geneva, Switzerland: World Health Organization (WHO), 2017
Argentina Vital Registration - Deaths 2009 ICD10 as it appears in World Health Organization (WHO). WHO Mortality Database Version October 2017. Geneva, Switzerland: World Health Organization (WHO), 2017

Argentina Vital Registration - Deaths 2010 ICD10 as it appears in World Health Organization (WHO). WHO Mortality Database Version October 2017. Geneva, Switzerland: World Health Organization (WHO), 2017
Argentina Vital Registration - Deaths 2011 ICD10 as it appears in World Health Organization (WHO). WHO Mortality Database Version October 2017. Geneva, Switzerland: World Health Organization (WHO), 2017
Argentina Vital Registration - Deaths 2012 ICD10 as it appears in World Health Organization (WHO). WHO Mortality Database Version October 2017. Geneva, Switzerland: World Health Organization (WHO), 2017
Argentina Vital Registration - Deaths 2013 ICD10 as it appears in World Health Organization (WHO). WHO Mortality Database Version October 2017. Geneva, Switzerland: World Health Organization (WHO), 2017
Argentina Vital Registration - Deaths 2014 ICD10 as it appears in World Health Organization (WHO). WHO Mortality Database Version October 2017. Geneva, Switzerland: World Health Organization (WHO), 2017
Argentina Vital Registration - Deaths 2015 ICD10 as it appears in World Health Organization (WHO). WHO Mortality Database Version April 2018. Geneva, Switzerland: World Health Organization (WHO), 2018
Armenia Vital Registration - Deaths 1981 ICD9 as it appears in World Health Organization (WHO). WHO Mortality Database Version September 2016. Geneva, Switzerland: World Health Organization (WHO), 2016
Armenia Vital Registration - Deaths 1982 ICD9 as it appears in World Health Organization (WHO). WHO Mortality Database Version September 2016. Geneva, Switzerland: World Health Organization (WHO), 2016
Armenia Vital Registration - Deaths 1985 ICD9 as it appears in World Health Organization (WHO). WHO Mortality Database Version September 2016. Geneva, Switzerland: World Health Organization (WHO), 2016
Armenia Vital Registration - Deaths 1986 ICD9 as it appears in World Health Organization (WHO). WHO Mortality Database Version September 2016. Geneva, Switzerland: World Health Organization (WHO), 2016
Armenia Vital Registration - Deaths 1987 ICD9 as it appears in World Health Organization (WHO). WHO Mortality Database Version September 2016. Geneva, Switzerland: World Health Organization (WHO), 2016
Armenia Vital Registration - Deaths 1989 ICD9 as it appears in World Health Organization (WHO). WHO Mortality Database Version September 2016. Geneva, Switzerland: World Health Organization (WHO), 2016
Armenia Vital Registration - Deaths 1990 ICD9 as it appears in World Health Organization (WHO). WHO Mortality Database Version September 2016. Geneva, Switzerland: World Health Organization (WHO), 2016
Armenia Vital Registration - Deaths 1991 ICD9 as it appears in World Health Organization (WHO). WHO Mortality Database Version September 2016. Geneva, Switzerland: World Health Organization (WHO), 2016
Armenia Vital Registration - Deaths 1992 ICD9 as it appears in World Health Organization (WHO). WHO Mortality Database Version September 2016. Geneva, Switzerland: World Health Organization (WHO), 2016
Armenia Vital Registration - Deaths 1993 ICD9 as it appears in World Health Organization (WHO). WHO Mortality Database Version September 2016. Geneva, Switzerland: World Health Organization (WHO), 2016
Armenia Vital Registration - Deaths 1994 ICD9 as it appears in World Health Organization (WHO). WHO Mortality Database Version September 2016. Geneva, Switzerland: World Health Organization (WHO), 2016
Armenia Vital Registration - Deaths 1995 ICD9 as it appears in World Health Organization (WHO). WHO Mortality Database Version September 2016. Geneva, Switzerland: World Health Organization (WHO), 2016
Armenia Vital Registration - Deaths 1996 ICD9 as it appears in World Health Organization (WHO). WHO Mortality Database Version September 2016. Geneva, Switzerland: World Health Organization (WHO), 2016
Armenia Vital Registration - Deaths 1997 ICD9 as it appears in World Health Organization (WHO). WHO Mortality Database Version September 2016. Geneva, Switzerland: World Health Organization (WHO), 2016

Armenia Vital Registration - Deaths 1998 ICD9 as it appears in World Health Organization (WHO). WHO Mortality Database Version September 2016. Geneva, Switzerland: World Health Organization (WHO), 2016
Armenia Vital Registration - Deaths 1999 ICD9 as it appears in World Health Organization (WHO). WHO Mortality Database Version September 2016. Geneva, Switzerland: World Health Organization (WHO), 2016
Armenia Vital Registration - Deaths 2000 ICD9 as it appears in World Health Organization (WHO). WHO Mortality Database Version September 2016. Geneva, Switzerland: World Health Organization (WHO), 2016
Armenia Vital Registration - Deaths 2001 ICD9 as it appears in World Health Organization (WHO). WHO Mortality Database Version September 2016. Geneva, Switzerland: World Health Organization (WHO), 2016
Armenia Vital Registration - Deaths 2002 ICD9 as it appears in World Health Organization (WHO). WHO Mortality Database Version September 2016. Geneva, Switzerland: World Health Organization (WHO), 2016
Armenia Vital Registration - Deaths 2003 ICD9 as it appears in World Health Organization (WHO). WHO Mortality Database Version September 2016. Geneva, Switzerland: World Health Organization (WHO), 2016
Armenia Vital Registration - Deaths 2012 ICD10 as it appears in World Health Organization (WHO). WHO Mortality Database Version October 2017. Geneva, Switzerland: World Health Organization (WHO), 2017
Armenia Vital Registration - Deaths 2013 ICD10 as it appears in World Health Organization (WHO). WHO Mortality Database Version March 2017. Geneva, Switzerland: World Health Organization (WHO), 2017
Armenia Vital Registration - Deaths 2014 ICD10 as it appears in World Health Organization (WHO). WHO Mortality Database Version October 2017. Geneva, Switzerland: World Health Organization (WHO), 2017
Armenia Vital Registration - Deaths 2015 ICD10 as it appears in World Health Organization (WHO). WHO Mortality Database Version March 2017. Geneva, Switzerland: World Health Organization (WHO), 2017
Armenia Vital Registration - Deaths 2016 ICD10 as it appears in World Health Organization (WHO). WHO Mortality Database Version April 2018. Geneva, Switzerland: World Health Organization (WHO), 2018
Australia Vital Registration - Deaths 1980 ICD9 as it appears in World Health Organization (WHO). WHO Mortality Database Version November 2015. Geneva, Switzerland: World Health Organization (WHO), 2015
Australia Vital Registration - Deaths 1981 ICD9 as it appears in World Health Organization (WHO). WHO Mortality Database Version November 2015. Geneva, Switzerland: World Health Organization (WHO), 2015
Australia Vital Registration - Deaths 1982 ICD9 as it appears in World Health Organization (WHO). WHO Mortality Database Version November 2015. Geneva, Switzerland: World Health Organization (WHO), 2015
Australia Vital Registration - Deaths 1983 ICD9 as it appears in World Health Organization (WHO). WHO Mortality Database Version November 2015. Geneva, Switzerland: World Health Organization (WHO), 2015
Australia Vital Registration - Deaths 1984 ICD9 as it appears in World Health Organization (WHO). WHO Mortality Database Version November 2015. Geneva, Switzerland: World Health Organization (WHO), 2015
Australia Vital Registration - Deaths 1985 ICD9 as it appears in World Health Organization (WHO). WHO Mortality Database Version November 2015. Geneva, Switzerland: World Health Organization (WHO), 2015
Australia Vital Registration - Deaths 1986 ICD9 as it appears in World Health Organization (WHO). WHO Mortality Database Version November 2015. Geneva, Switzerland: World Health Organization (WHO), 2015
Australia Vital Registration - Deaths 1987 ICD9 as it appears in World Health Organization (WHO). WHO Mortality Database Version November 2015. Geneva, Switzerland: World Health Organization (WHO), 2015
Australia Vital Registration - Deaths 1988 ICD9 as it appears in World Health Organization (WHO). WHO Mortality Database Version November 2015. Geneva, Switzerland: World Health Organization (WHO), 2015

Australia Vital Registration - Deaths 1989 ICD9 as it appears in World Health Organization (WHO). WHO Mortality Database Version November 2015. Geneva, Switzerland: World Health Organization (WHO), 2015
Australia Vital Registration - Deaths 1990 ICD9 as it appears in World Health Organization (WHO). WHO Mortality Database Version November 2015. Geneva, Switzerland: World Health Organization (WHO), 2015
Australia Vital Registration - Deaths 1991 ICD9 as it appears in World Health Organization (WHO). WHO Mortality Database Version November 2015. Geneva, Switzerland: World Health Organization (WHO), 2015
Australia Vital Registration - Deaths 1992 ICD9 as it appears in World Health Organization (WHO). WHO Mortality Database Version November 2015. Geneva, Switzerland: World Health Organization (WHO), 2015
Australia Vital Registration - Deaths 1993 ICD9 as it appears in World Health Organization (WHO). WHO Mortality Database Version November 2015. Geneva, Switzerland: World Health Organization (WHO), 2015
Australia Vital Registration - Deaths 1994 ICD9 as it appears in World Health Organization (WHO). WHO Mortality Database Version November 2015. Geneva, Switzerland: World Health Organization (WHO), 2015
Australia Vital Registration - Deaths 1995 ICD9 as it appears in World Health Organization (WHO). WHO Mortality Database Version November 2015. Geneva, Switzerland: World Health Organization (WHO), 2015
Australia Vital Registration - Deaths 1996 ICD9 as it appears in World Health Organization (WHO). WHO Mortality Database Version November 2015. Geneva, Switzerland: World Health Organization (WHO), 2015
Australia Vital Registration - Deaths 1997 ICD9 as it appears in World Health Organization (WHO). WHO Mortality Database Version November 2015. Geneva, Switzerland: World Health Organization (WHO), 2015
Australia Vital Registration - Deaths 1998 ICD10 as it appears in World Health Organization (WHO). WHO Mortality Database Version October 2017. Geneva, Switzerland: World Health Organization (WHO), 2017
Australia Vital Registration - Deaths 1999 ICD10 as it appears in World Health Organization (WHO). WHO Mortality Database Version October 2017. Geneva, Switzerland: World Health Organization (WHO), 2017
Australia Vital Registration - Deaths 2000 ICD10 as it appears in World Health Organization (WHO). WHO Mortality Database Version October 2017. Geneva, Switzerland: World Health Organization (WHO), 2017
Australia Vital Registration - Deaths 2001 ICD10 as it appears in World Health Organization (WHO). WHO Mortality Database Version October 2017. Geneva, Switzerland: World Health Organization (WHO), 2017
Australia Vital Registration - Deaths 2002 ICD10 as it appears in World Health Organization (WHO). WHO Mortality Database Version October 2017. Geneva, Switzerland: World Health Organization (WHO), 2017
Australia Vital Registration - Deaths 2003 ICD10 as it appears in World Health Organization (WHO). WHO Mortality Database Version October 2017. Geneva, Switzerland: World Health Organization (WHO), 2017
Australia Vital Registration - Deaths 2004 ICD10 as it appears in World Health Organization (WHO). WHO Mortality Database Version October 2017. Geneva, Switzerland: World Health Organization (WHO), 2017
Australia Vital Registration - Deaths 2006 ICD10 as it appears in World Health Organization (WHO). WHO Mortality Database Version October 2017. Geneva, Switzerland: World Health Organization (WHO), 2017
Australia Vital Registration - Deaths 2007 ICD10 as it appears in World Health Organization (WHO). WHO Mortality Database Version October 2017. Geneva, Switzerland: World Health Organization (WHO), 2017
Australia Vital Registration - Deaths 2008 ICD10 as it appears in World Health Organization (WHO). WHO Mortality Database Version October 2017. Geneva, Switzerland: World Health Organization (WHO), 2017
Australia Vital Registration - Deaths 2009 ICD10 as it appears in World Health Organization (WHO). WHO Mortality Database Version October 2017. Geneva, Switzerland: World Health Organization (WHO), 2017

Australia Vital Registration - Deaths 2010 ICD10 as it appears in World Health Organization (WHO). WHO Mortality Database Version October 2017. Geneva, Switzerland: World Health Organization (WHO), 2017
Australia Vital Registration - Deaths 2011 ICD10 as it appears in World Health Organization (WHO). WHO Mortality Database Version October 2017. Geneva, Switzerland: World Health Organization (WHO), 2017
Australia Vital Registration - Deaths 2012 ICD10 as it appears in World Health Organization (WHO). WHO Mortality Database Version October 2017. Geneva, Switzerland: World Health Organization (WHO), 2017
Australia Vital Registration - Deaths 2013 ICD10 as it appears in World Health Organization (WHO). WHO Mortality Database Version October 2017. Geneva, Switzerland: World Health Organization (WHO), 2017
Australia Vital Registration - Deaths 2014 ICD10 as it appears in World Health Organization (WHO). WHO Mortality Database Version October 2017. Geneva, Switzerland: World Health Organization (WHO), 2017
Australia Vital Registration - Deaths 2015 ICD10 as it appears in World Health Organization (WHO). WHO Mortality Database Version October 2017. Geneva, Switzerland: World Health Organization (WHO), 2017
Australian Bureau of Statistics. Australia Vital Registration - Deaths 2005
Austria Vital Registration - Deaths 1980 ICD9 as it appears in World Health Organization (WHO). WHO Mortality Database Version November 2015. Geneva, Switzerland: World Health Organization (WHO), 2015
Austria Vital Registration - Deaths 1981 ICD9 as it appears in World Health Organization (WHO). WHO Mortality Database Version November 2015. Geneva, Switzerland: World Health Organization (WHO), 2015
Austria Vital Registration - Deaths 1982 ICD9 as it appears in World Health Organization (WHO). WHO Mortality Database Version November 2015. Geneva, Switzerland: World Health Organization (WHO), 2015
Austria Vital Registration - Deaths 1983 ICD9 as it appears in World Health Organization (WHO). WHO Mortality Database Version September 2016. Geneva, Switzerland: World Health Organization (WHO), 2016
Austria Vital Registration - Deaths 1984 ICD9 as it appears in World Health Organization (WHO). WHO Mortality Database Version November 2015. Geneva, Switzerland: World Health Organization (WHO), 2015
Austria Vital Registration - Deaths 1985 ICD9 as it appears in World Health Organization (WHO). WHO Mortality Database Version November 2015. Geneva, Switzerland: World Health Organization (WHO), 2015
Austria Vital Registration - Deaths 1986 ICD9 as it appears in World Health Organization (WHO). WHO Mortality Database Version November 2015. Geneva, Switzerland: World Health Organization (WHO), 2015
Austria Vital Registration - Deaths 1987 ICD9 as it appears in World Health Organization (WHO). WHO Mortality Database Version November 2015. Geneva, Switzerland: World Health Organization (WHO), 2015
Austria Vital Registration - Deaths 1988 ICD9 as it appears in World Health Organization (WHO). WHO Mortality Database Version November 2015. Geneva, Switzerland: World Health Organization (WHO), 2015
Austria Vital Registration - Deaths 1989 ICD9 as it appears in World Health Organization (WHO). WHO Mortality Database Version November 2015. Geneva, Switzerland: World Health Organization (WHO), 2015
Austria Vital Registration - Deaths 1990 ICD9 as it appears in World Health Organization (WHO). WHO Mortality Database Version November 2015. Geneva, Switzerland: World Health Organization (WHO), 2015
Austria Vital Registration - Deaths 1991 ICD9 as it appears in World Health Organization (WHO). WHO Mortality Database Version November 2015. Geneva, Switzerland: World Health Organization (WHO), 2015
Austria Vital Registration - Deaths 1992 ICD9 as it appears in World Health Organization (WHO). WHO Mortality Database Version November 2015. Geneva, Switzerland: World Health Organization (WHO), 2015
Austria Vital Registration - Deaths 1993 ICD9 as it appears in World Health Organization (WHO). WHO Mortality Database Version November 2015. Geneva, Switzerland: World Health Organization (WHO), 2015

Austria Vital Registration - Deaths 1994 ICD9 as it appears in World Health Organization (WHO). WHO Mortality Database Version November 2015. Geneva, Switzerland: World Health Organization (WHO), 2015
Austria Vital Registration - Deaths 1995 ICD9 as it appears in World Health Organization (WHO). WHO Mortality Database Version November 2015. Geneva, Switzerland: World Health Organization (WHO), 2015
Austria Vital Registration - Deaths 1996 ICD9 as it appears in World Health Organization (WHO). WHO Mortality Database Version November 2015. Geneva, Switzerland: World Health Organization (WHO), 2015
Austria Vital Registration - Deaths 2002 ICD10 as it appears in World Health Organization (WHO). WHO Mortality Database Version October 2017. Geneva, Switzerland: World Health Organization (WHO), 2017
Austria Vital Registration - Deaths 2003 ICD10 as it appears in World Health Organization (WHO). WHO Mortality Database Version October 2017. Geneva, Switzerland: World Health Organization (WHO), 2017
Austria Vital Registration - Deaths 2004 ICD10 as it appears in World Health Organization (WHO). WHO Mortality Database Version October 2017. Geneva, Switzerland: World Health Organization (WHO), 2017
Austria Vital Registration - Deaths 2005 ICD10 as it appears in World Health Organization (WHO). WHO Mortality Database Version October 2017. Geneva, Switzerland: World Health Organization (WHO), 2017
Austria Vital Registration - Deaths 2006 ICD10 as it appears in World Health Organization (WHO). WHO Mortality Database Version October 2017. Geneva, Switzerland: World Health Organization (WHO), 2017
Austria Vital Registration - Deaths 2007 ICD10 as it appears in World Health Organization (WHO). WHO Mortality Database Version October 2017. Geneva, Switzerland: World Health Organization (WHO), 2017
Austria Vital Registration - Deaths 2008 ICD10 as it appears in World Health Organization (WHO). WHO Mortality Database Version October 2017. Geneva, Switzerland: World Health Organization (WHO), 2017
Austria Vital Registration - Deaths 1997 ICD9 as it appears in World Health Organization (WHO). WHO Mortality Database Version November 2015. Geneva, Switzerland: World Health Organization (WHO), 2015
Austria Vital Registration - Deaths 1998 ICD9 as it appears in World Health Organization (WHO). WHO Mortality Database Version November 2015. Geneva, Switzerland: World Health Organization (WHO), 2015
Austria Vital Registration - Deaths 1999 ICD9 as it appears in World Health Organization (WHO). WHO Mortality Database Version November 2015. Geneva, Switzerland: World Health Organization (WHO), 2015
Austria Vital Registration - Deaths 2000 ICD9 as it appears in World Health Organization (WHO). WHO Mortality Database Version November 2015. Geneva, Switzerland: World Health Organization (WHO), 2015
Austria Vital Registration - Deaths 2001 ICD9 as it appears in World Health Organization (WHO). WHO Mortality Database Version November 2015. Geneva, Switzerland: World Health Organization (WHO), 2015
Austria Vital Registration - Deaths 2009 ICD10 as it appears in World Health Organization (WHO). WHO Mortality Database Version October 2017. Geneva, Switzerland: World Health Organization (WHO), 2017
Austria Vital Registration - Deaths 2010 ICD10 as it appears in World Health Organization (WHO). WHO Mortality Database Version October 2017. Geneva, Switzerland: World Health Organization (WHO), 2017
Austria Vital Registration - Deaths 2011 ICD10 as it appears in World Health Organization (WHO). WHO Mortality Database Version October 2017. Geneva, Switzerland: World Health Organization (WHO), 2017
Austria Vital Registration - Deaths 2012 ICD10 as it appears in World Health Organization (WHO). WHO Mortality Database Version October 2017. Geneva, Switzerland: World Health Organization (WHO), 2017
Austria Vital Registration - Deaths 2013 ICD10 as it appears in World Health Organization (WHO). WHO Mortality Database Version October 2017. Geneva, Switzerland: World Health Organization (WHO), 2017

Austria Vital Registration - Deaths 2014 ICD10 as it appears in World Health Organization (WHO). WHO Mortality Database Version October 2017. Geneva, Switzerland: World Health Organization (WHO), 2017
Austria Vital Registration - Deaths 2015 ICD10 as it appears in World Health Organization (WHO). WHO Mortality Database Version October 2017. Geneva, Switzerland: World Health Organization (WHO), 2017
Austria Vital Registration - Deaths 2016 ICD10 as it appears in World Health Organization (WHO). WHO Mortality Database Version October 2017. Geneva, Switzerland: World Health Organization (WHO), 2017
Azerbaijan Vital Registration - Deaths 1992 ICD9 as it appears in World Health Organization (WHO). WHO Mortality Database Version September 2016. Geneva, Switzerland: World Health Organization (WHO), 2016
Azerbaijan Vital Registration - Deaths 1993 ICD9 as it appears in World Health Organization (WHO). WHO Mortality Database Version September 2016. Geneva, Switzerland: World Health Organization (WHO), 2016
Azerbaijan Vital Registration - Deaths 1994 ICD9 as it appears in World Health Organization (WHO). WHO Mortality Database Version September 2016. Geneva, Switzerland: World Health Organization (WHO), 2016
Azerbaijan Vital Registration - Deaths 1995 ICD9 as it appears in World Health Organization (WHO). WHO Mortality Database Version September 2016. Geneva, Switzerland: World Health Organization (WHO), 2016
Azerbaijan Vital Registration - Deaths 1996 ICD9 as it appears in World Health Organization (WHO). WHO Mortality Database Version September 2016. Geneva, Switzerland: World Health Organization (WHO), 2016
Azerbaijan Vital Registration - Deaths 1997 ICD9 as it appears in World Health Organization (WHO). WHO Mortality Database Version September 2016. Geneva, Switzerland: World Health Organization (WHO), 2016
Azerbaijan Vital Registration - Deaths 1998 ICD9 as it appears in World Health Organization (WHO). WHO Mortality Database Version September 2016. Geneva, Switzerland: World Health Organization (WHO), 2016
Azerbaijan Vital Registration - Deaths 1999 ICD9 as it appears in World Health Organization (WHO). WHO Mortality Database Version September 2016. Geneva, Switzerland: World Health Organization (WHO), 2016
Azerbaijan Vital Registration - Deaths 2000 ICD9 as it appears in World Health Organization (WHO). WHO Mortality Database Version September 2016. Geneva, Switzerland: World Health Organization (WHO), 2016
Azerbaijan Vital Registration - Deaths 2007 ICD10 as it appears in World Health Organization (WHO). WHO Mortality Database Version October 2017. Geneva, Switzerland: World Health Organization (WHO), 2017
Azerbaijan Vital Registration - Deaths 1981 ICD9 as it appears in World Health Organization (WHO). WHO Mortality Database Version September 2016. Geneva, Switzerland: World Health Organization (WHO), 2016
Azerbaijan Vital Registration - Deaths 1982 ICD9 as it appears in World Health Organization (WHO). WHO Mortality Database Version September 2016. Geneva, Switzerland: World Health Organization (WHO), 2016
Azerbaijan Vital Registration - Deaths 1985 ICD9 as it appears in World Health Organization (WHO). WHO Mortality Database Version September 2016. Geneva, Switzerland: World Health Organization (WHO), 2016
Azerbaijan Vital Registration - Deaths 1986 ICD9 as it appears in World Health Organization (WHO). WHO Mortality Database Version September 2016. Geneva, Switzerland: World Health Organization (WHO), 2016
Azerbaijan Vital Registration - Deaths 1987 ICD9 as it appears in World Health Organization (WHO). WHO Mortality Database Version September 2016. Geneva, Switzerland: World Health Organization (WHO), 2016
Azerbaijan Vital Registration - Deaths 1988 ICD9 as it appears in World Health Organization (WHO). WHO Mortality Database Version September 2016. Geneva, Switzerland: World Health Organization (WHO), 2016
Azerbaijan Vital Registration - Deaths 1989 ICD9 as it appears in World Health Organization (WHO). WHO Mortality Database Version September 2016. Geneva, Switzerland: World Health Organization (WHO), 2016

Azerbaijan Vital Registration - Deaths 1990 ICD9 as it appears in World Health Organization (WHO). WHO Mortality Database Version September 2016. Geneva, Switzerland: World Health Organization (WHO), 2016
Azerbaijan Vital Registration - Deaths 1991 ICD9 as it appears in World Health Organization (WHO). WHO Mortality Database Version September 2016. Geneva, Switzerland: World Health Organization (WHO), 2016
Bahamas Vital Registration - Deaths 1980 ICD9 as it appears in World Health Organization (WHO). WHO Mortality Database Version September 2016. Geneva, Switzerland: World Health Organization (WHO), 2016
Bahamas Vital Registration - Deaths 1981 ICD9 as it appears in World Health Organization (WHO). WHO Mortality Database Version September 2016. Geneva, Switzerland: World Health Organization (WHO), 2016
Bahamas Vital Registration - Deaths 1984 ICD9 as it appears in World Health Organization (WHO). WHO Mortality Database Version September 2016. Geneva, Switzerland: World Health Organization (WHO), 2016
Bahamas Vital Registration - Deaths 1985 ICD9 as it appears in World Health Organization (WHO). WHO Mortality Database Version September 2016. Geneva, Switzerland: World Health Organization (WHO), 2016
Bahamas Vital Registration - Deaths 1987 ICD9 as it appears in World Health Organization (WHO). WHO Mortality Database Version September 2016. Geneva, Switzerland: World Health Organization (WHO), 2016
Bahamas Vital Registration - Deaths 1993 ICD9 as it appears in World Health Organization (WHO). WHO Mortality Database Version September 2016. Geneva, Switzerland: World Health Organization (WHO), 2016
Bahamas Vital Registration - Deaths 1994 ICD9 as it appears in World Health Organization (WHO). WHO Mortality Database Version September 2016. Geneva, Switzerland: World Health Organization (WHO), 2016
Bahamas Vital Registration - Deaths 1995 ICD9 as it appears in World Health Organization (WHO). WHO Mortality Database Version September 2016. Geneva, Switzerland: World Health Organization (WHO), 2016
Bahamas Vital Registration - Deaths 1996 ICD9 as it appears in World Health Organization (WHO). WHO Mortality Database Version September 2016. Geneva, Switzerland: World Health Organization (WHO), 2016
Bahamas Vital Registration - Deaths 1997 ICD9 as it appears in World Health Organization (WHO). WHO Mortality Database Version September 2016. Geneva, Switzerland: World Health Organization (WHO), 2016
Bahamas Vital Registration - Deaths 1998 ICD9 as it appears in World Health Organization (WHO). WHO Mortality Database Version September 2016. Geneva, Switzerland: World Health Organization (WHO), 2016
Bahamas Vital Registration - Deaths 1999 ICD10 as it appears in World Health Organization (WHO). WHO Mortality Database Version October 2017. Geneva, Switzerland: World Health Organization (WHO), 2017
Bahamas Vital Registration - Deaths 2000 ICD10 as it appears in World Health Organization (WHO). WHO Mortality Database Version October 2017. Geneva, Switzerland: World Health Organization (WHO), 2017
Bahamas Vital Registration - Deaths 2001 ICD10 as it appears in World Health Organization (WHO). WHO Mortality Database Version October 2017. Geneva, Switzerland: World Health Organization (WHO), 2017
Bahamas Vital Registration - Deaths 2002 ICD10 as it appears in World Health Organization (WHO). WHO Mortality Database Version October 2017. Geneva, Switzerland: World Health Organization (WHO), 2017
Bahamas Vital Registration - Deaths 2003 ICD10 as it appears in World Health Organization (WHO). WHO Mortality Database Version October 2017. Geneva, Switzerland: World Health Organization (WHO), 2017
Bahamas Vital Registration - Deaths 2005 ICD10 as it appears in World Health Organization (WHO). WHO Mortality Database Version October 2017. Geneva, Switzerland: World Health Organization (WHO), 2017
Bahamas Vital Registration - Deaths 2004 ICD10 as it appears in World Health Organization (WHO). WHO Mortality Database Version October 2017. Geneva, Switzerland: World Health Organization (WHO), 2017

Bahamas Vital Registration - Deaths 2006 ICD10 as it appears in World Health Organization (WHO). WHO Mortality Database Version October 2017. Geneva, Switzerland: World Health Organization (WHO), 2017
Bahamas Vital Registration - Deaths 2007 ICD10 as it appears in World Health Organization (WHO). WHO Mortality Database Version October 2017. Geneva, Switzerland: World Health Organization (WHO), 2017
Bahamas Vital Registration - Deaths 2008 ICD10 as it appears in World Health Organization (WHO). WHO Mortality Database Version October 2017. Geneva, Switzerland: World Health Organization (WHO), 2017
Bahamas Vital Registration - Deaths 2009 ICD10 as it appears in World Health Organization (WHO). WHO Mortality Database Version October 2017. Geneva, Switzerland: World Health Organization (WHO), 2017
Bahamas Vital Registration - Deaths 2010 ICD10 as it appears in World Health Organization (WHO). WHO Mortality Database Version October 2017. Geneva, Switzerland: World Health Organization (WHO), 2017
Bahamas Vital Registration - Deaths 2011 ICD10 as it appears in World Health Organization (WHO). WHO Mortality Database Version October 2017. Geneva, Switzerland: World Health Organization (WHO), 2017
Bahamas Vital Registration - Deaths 2012 ICD10 as it appears in World Health Organization (WHO). WHO Mortality Database Version October 2017. Geneva, Switzerland: World Health Organization (WHO), 2017
Bahamas Vital Registration - Deaths 2013 ICD10 as it appears in World Health Organization (WHO). WHO Mortality Database Version October 2017. Geneva, Switzerland: World Health Organization (WHO), 2017
Bahrain Vital Registration - Deaths 1997 ICD10 as it appears in World Health Organization (WHO). WHO Mortality Database Version April 2018. Geneva, Switzerland: World Health Organization (WHO), 2018
Bahrain Vital Registration - Deaths 1998 ICD10 as it appears in World Health Organization (WHO). WHO Mortality Database Version April 2018. Geneva, Switzerland: World Health Organization (WHO), 2018
Bahrain Vital Registration - Deaths 1999 ICD10 as it appears in World Health Organization (WHO). WHO Mortality Database Version April 2018. Geneva, Switzerland: World Health Organization (WHO), 2018
Bahrain Vital Registration - Deaths 2000 ICD10 as it appears in World Health Organization (WHO). WHO Mortality Database Version October 2017. Geneva, Switzerland: World Health Organization (WHO), 2017
Bahrain Vital Registration - Deaths 2001 ICD10 as it appears in World Health Organization (WHO). WHO Mortality Database Version October 2017. Geneva, Switzerland: World Health Organization (WHO), 2017
Bahrain Vital Registration - Deaths 1986 as it appears in World Health Organization (WHO). WHO Mortality Database Version September 2016. Geneva, Switzerland: World Health Organization (WHO), 2016
Bahrain Vital Registration - Deaths 2002 ICD10 as it appears in World Health Organization (WHO). WHO Mortality Database Version October 2017. Geneva, Switzerland: World Health Organization (WHO), 2017
Bahrain Vital Registration - Deaths 2003 ICD10 as it appears in World Health Organization (WHO). WHO Mortality Database Version October 2017. Geneva, Switzerland: World Health Organization (WHO), 2017
Bahrain Vital Registration - Deaths 2004 ICD10 as it appears in World Health Organization (WHO). WHO Mortality Database Version October 2017. Geneva, Switzerland: World Health Organization (WHO), 2017
Bahrain Vital Registration - Deaths 2005 ICD10 as it appears in World Health Organization (WHO). WHO Mortality Database Version October 2017. Geneva, Switzerland: World Health Organization (WHO), 2017
Bahrain Vital Registration - Deaths 2006 ICD10 as it appears in World Health Organization (WHO). WHO Mortality Database Version October 2017. Geneva, Switzerland: World Health Organization (WHO), 2017
Bahrain Vital Registration - Deaths 2007 ICD10 as it appears in World Health Organization (WHO). WHO Mortality Database Version October 2017. Geneva, Switzerland: World Health Organization (WHO), 2017

Bahrain Vital Registration - Deaths 2008 ICD10 as it appears in World Health Organization (WHO). WHO Mortality Database Version October 2017. Geneva, Switzerland: World Health Organization (WHO), 2017

Bahrain Vital Registration - Deaths 2009 ICD10 as it appears in World Health Organization (WHO). WHO Mortality Database Version October 2017. Geneva, Switzerland: World Health Organization (WHO), 2017

Bahrain Vital Registration - Deaths 2010 ICD10 as it appears in World Health Organization (WHO). WHO Mortality Database Version October 2017. Geneva, Switzerland: World Health Organization (WHO), 2017

Bahrain Vital Registration - Deaths 2011 ICD10 as it appears in World Health Organization (WHO). WHO Mortality Database Version October 2017. Geneva, Switzerland: World Health Organization (WHO), 2017

Bahrain Vital Registration - Deaths 2012 ICD10 as it appears in World Health Organization (WHO). WHO Mortality Database Version October 2017. Geneva, Switzerland: World Health Organization (WHO), 2017

Bahrain Vital Registration - Deaths 2013 ICD10 as it appears in World Health Organization (WHO). WHO Mortality Database Version October 2017. Geneva, Switzerland: World Health Organization (WHO), 2017

Bahrain Vital Registration - Deaths 2014 ICD10 as it appears in World Health Organization (WHO). WHO Mortality Database Version October 2017. Geneva, Switzerland: World Health Organization (WHO), 2017

Barbados Vital Registration - Deaths 1980 ICD9 as it appears in World Health Organization (WHO). WHO Mortality Database Version September 2016. Geneva, Switzerland: World Health Organization (WHO), 2016

Barbados Vital Registration - Deaths 1981 ICD9 as it appears in World Health Organization (WHO). WHO Mortality Database Version September 2016. Geneva, Switzerland: World Health Organization (WHO), 2016

Barbados Vital Registration - Deaths 1982 ICD9 as it appears in World Health Organization (WHO). WHO Mortality Database Version September 2016. Geneva, Switzerland: World Health Organization (WHO), 2016

Barbados Vital Registration - Deaths 1983 ICD9 as it appears in World Health Organization (WHO). WHO Mortality Database Version September 2016. Geneva, Switzerland: World Health Organization (WHO), 2016

Barbados Vital Registration - Deaths 1984 ICD9 as it appears in World Health Organization (WHO). WHO Mortality Database Version September 2016. Geneva, Switzerland: World Health Organization (WHO), 2016

Barbados Vital Registration - Deaths 1985 ICD9 as it appears in World Health Organization (WHO). WHO Mortality Database Version September 2016. Geneva, Switzerland: World Health Organization (WHO), 2016

Barbados Vital Registration - Deaths 1986 ICD9 as it appears in World Health Organization (WHO). WHO Mortality Database Version September 2016. Geneva, Switzerland: World Health Organization (WHO), 2016

Barbados Vital Registration - Deaths 1987 ICD9 as it appears in World Health Organization (WHO). WHO Mortality Database Version September 2016. Geneva, Switzerland: World Health Organization (WHO), 2016

Barbados Vital Registration - Deaths 1988 ICD9 as it appears in World Health Organization (WHO). WHO Mortality Database Version September 2016. Geneva, Switzerland: World Health Organization (WHO), 2016

Barbados Vital Registration - Deaths 1989 ICD9 as it appears in World Health Organization (WHO). WHO Mortality Database Version September 2016. Geneva, Switzerland: World Health Organization (WHO), 2016

Barbados Vital Registration - Deaths 1990 ICD9 as it appears in World Health Organization (WHO). WHO Mortality Database Version September 2016. Geneva, Switzerland: World Health Organization (WHO), 2016

Barbados Vital Registration - Deaths 1991 ICD9 as it appears in World Health Organization (WHO). WHO Mortality Database Version September 2016. Geneva, Switzerland: World Health Organization (WHO), 2016

Barbados Vital Registration - Deaths 1992 ICD9 as it appears in World Health Organization (WHO). WHO Mortality Database Version September 2016. Geneva, Switzerland: World Health Organization (WHO), 2016

Barbados Vital Registration - Deaths 1993 ICD9 as it appears in World Health Organization (WHO). WHO Mortality Database Version September 2016. Geneva, Switzerland: World Health Organization (WHO), 2016

Barbados Vital Registration - Deaths 1994 ICD9 as it appears in World Health Organization (WHO). WHO Mortality Database Version September 2016. Geneva, Switzerland: World Health Organization (WHO), 2016

Barbados Vital Registration - Deaths 1995 ICD9 as it appears in World Health Organization (WHO). WHO Mortality Database Version September 2016. Geneva, Switzerland: World Health Organization (WHO), 2016

Barbados Vital Registration - Deaths 2000 ICD10 as it appears in World Health Organization (WHO). WHO Mortality Database Version October 2017. Geneva, Switzerland: World Health Organization (WHO), 2017

Barbados Vital Registration - Deaths 2001 ICD10 as it appears in World Health Organization (WHO). WHO Mortality Database Version October 2017. Geneva, Switzerland: World Health Organization (WHO), 2017

Barbados Vital Registration - Deaths 2002 ICD10 as it appears in World Health Organization (WHO). WHO Mortality Database Version October 2017. Geneva, Switzerland: World Health Organization (WHO), 2017

Barbados Vital Registration - Deaths 2003 ICD10 as it appears in World Health Organization (WHO). WHO Mortality Database Version October 2017. Geneva, Switzerland: World Health Organization (WHO), 2017

Barbados Vital Registration - Deaths 2004 ICD10 as it appears in World Health Organization (WHO). WHO Mortality Database Version October 2017. Geneva, Switzerland: World Health Organization (WHO), 2017

Barbados Vital Registration - Deaths 2005 ICD10 as it appears in World Health Organization (WHO). WHO Mortality Database Version October 2017. Geneva, Switzerland: World Health Organization (WHO), 2017

Barbados Vital Registration - Deaths 2006 ICD10 as it appears in World Health Organization (WHO). WHO Mortality Database Version October 2017. Geneva, Switzerland: World Health Organization (WHO), 2017

Barbados Vital Registration - Deaths 2007 ICD10 as it appears in World Health Organization (WHO). WHO Mortality Database Version October 2017. Geneva, Switzerland: World Health Organization (WHO), 2017

Barbados Vital Registration - Deaths 2008 ICD10 as it appears in World Health Organization (WHO). WHO Mortality Database Version October 2017. Geneva, Switzerland: World Health Organization (WHO), 2017

Barbados Vital Registration - Deaths 2009 ICD10 as it appears in World Health Organization (WHO). WHO Mortality Database Version October 2017. Geneva, Switzerland: World Health Organization (WHO), 2017

Barbados Vital Registration - Deaths 2010 ICD10 as it appears in World Health Organization (WHO). WHO Mortality Database Version October 2017. Geneva, Switzerland: World Health Organization (WHO), 2017

Barbados Vital Registration - Deaths 2011 ICD10 as it appears in World Health Organization (WHO). WHO Mortality Database Version October 2017. Geneva, Switzerland: World Health Organization (WHO), 2017

Barbados Vital Registration - Deaths 2012 ICD10 as it appears in World Health Organization (WHO). WHO Mortality Database Version October 2017. Geneva, Switzerland: World Health Organization (WHO), 2017

Barbados Vital Registration - Deaths 2013 ICD10 as it appears in World Health Organization (WHO). WHO Mortality Database Version October 2017. Geneva, Switzerland: World Health Organization (WHO), 2017

Barrêto IC, Kerr Pontes L, Corrêa L. Surveillance of infant deaths in local health systems: assessment of verbal autopsy reports and of information gathered from health agents. Rev Panam Salud Publica. 2000; 79(5): 303-12

Baskent University, Ministry of Health (Turkey), State Institute of Statistics (Turkey). Turkey Verbal Autopsy Survey 2003

Belarus Vital Registration - Deaths 1981 ICD9 as it appears in World Health Organization (WHO). WHO Mortality Database Version September 2016. Geneva, Switzerland: World Health Organization (WHO), 2016

Belarus Vital Registration - Deaths 1982 ICD9 as it appears in World Health Organization (WHO). WHO Mortality Database Version September 2016. Geneva, Switzerland: World Health Organization (WHO), 2016

Belarus Vital Registration - Deaths 1985 ICD9 as it appears in World Health Organization (WHO). WHO Mortality Database Version September 2016. Geneva, Switzerland: World Health Organization (WHO), 2016
Belarus Vital Registration - Deaths 1986 ICD9 as it appears in World Health Organization (WHO). WHO Mortality Database Version September 2016. Geneva, Switzerland: World Health Organization (WHO), 2016
Belarus Vital Registration - Deaths 1987 ICD9 as it appears in World Health Organization (WHO). WHO Mortality Database Version September 2016. Geneva, Switzerland: World Health Organization (WHO), 2016
Belarus Vital Registration - Deaths 1988 ICD9 as it appears in World Health Organization (WHO). WHO Mortality Database Version September 2016. Geneva, Switzerland: World Health Organization (WHO), 2016
Belarus Vital Registration - Deaths 1989 ICD9 as it appears in World Health Organization (WHO). WHO Mortality Database Version September 2016. Geneva, Switzerland: World Health Organization (WHO), 2016
Belarus Vital Registration - Deaths 1990 ICD9 as it appears in World Health Organization (WHO). WHO Mortality Database Version September 2016. Geneva, Switzerland: World Health Organization (WHO), 2016
Belarus Vital Registration - Deaths 1991 ICD9 as it appears in World Health Organization (WHO). WHO Mortality Database Version September 2016. Geneva, Switzerland: World Health Organization (WHO), 2016
Belarus Vital Registration - Deaths 1992 ICD9 as it appears in World Health Organization (WHO). WHO Mortality Database Version September 2016. Geneva, Switzerland: World Health Organization (WHO), 2016
Belarus Vital Registration - Deaths 1993 ICD9 as it appears in World Health Organization (WHO). WHO Mortality Database Version September 2016. Geneva, Switzerland: World Health Organization (WHO), 2016
Belarus Vital Registration - Deaths 1994 ICD9 as it appears in World Health Organization (WHO). WHO Mortality Database Version September 2016. Geneva, Switzerland: World Health Organization (WHO), 2016
Belarus Vital Registration - Deaths 1995 ICD9 as it appears in World Health Organization (WHO). WHO Mortality Database Version September 2016. Geneva, Switzerland: World Health Organization (WHO), 2016
Belarus Vital Registration - Deaths 1997 ICD9 as it appears in World Health Organization (WHO). WHO Mortality Database Version September 2016. Geneva, Switzerland: World Health Organization (WHO), 2016
Belarus Vital Registration - Deaths 1998 ICD9 as it appears in World Health Organization (WHO). WHO Mortality Database Version September 2016. Geneva, Switzerland: World Health Organization (WHO), 2016
Belarus Vital Registration - Deaths 1999 ICD9 as it appears in World Health Organization (WHO). WHO Mortality Database Version September 2016. Geneva, Switzerland: World Health Organization (WHO), 2016
Belarus Vital Registration - Deaths 2000 ICD9 as it appears in World Health Organization (WHO). WHO Mortality Database Version September 2016. Geneva, Switzerland: World Health Organization (WHO), 2016
Belarus Vital Registration - Deaths 2001 ICD9 as it appears in World Health Organization (WHO). WHO Mortality Database Version September 2016. Geneva, Switzerland: World Health Organization (WHO), 2016
Belgium Vital Registration - Deaths 1980 ICD9 as it appears in World Health Organization (WHO). WHO Mortality Database Version November 2015. Geneva, Switzerland: World Health Organization (WHO), 2015
Belgium Vital Registration - Deaths 1981 ICD9 as it appears in World Health Organization (WHO). WHO Mortality Database Version November 2015. Geneva, Switzerland: World Health Organization (WHO), 2015
Belgium Vital Registration - Deaths 1982 ICD9 as it appears in World Health Organization (WHO). WHO Mortality Database Version November 2015. Geneva, Switzerland: World Health Organization (WHO), 2015
Belgium Vital Registration - Deaths 1983 ICD9 as it appears in World Health Organization (WHO). WHO Mortality Database Version November 2015. Geneva, Switzerland: World Health Organization (WHO), 2015

Belgium Vital Registration - Deaths 1984 ICD9 as it appears in World Health Organization (WHO). WHO Mortality Database Version November 2015. Geneva, Switzerland: World Health Organization (WHO), 2015
Belgium Vital Registration - Deaths 1985 ICD9 as it appears in World Health Organization (WHO). WHO Mortality Database Version September 2016. Geneva, Switzerland: World Health Organization (WHO), 2016
Belgium Vital Registration - Deaths 1986 ICD9 as it appears in World Health Organization (WHO). WHO Mortality Database Version November 2015. Geneva, Switzerland: World Health Organization (WHO), 2015
Belgium Vital Registration - Deaths 1987 ICD9 as it appears in World Health Organization (WHO). WHO Mortality Database Version November 2015. Geneva, Switzerland: World Health Organization (WHO), 2015
Belgium Vital Registration - Deaths 1988 ICD9 as it appears in World Health Organization (WHO). WHO Mortality Database Version September 2016. Geneva, Switzerland: World Health Organization (WHO), 2016
Belgium Vital Registration - Deaths 1989 ICD9 as it appears in World Health Organization (WHO). WHO Mortality Database Version September 2016. Geneva, Switzerland: World Health Organization (WHO), 2016
Belgium Vital Registration - Deaths 1990 ICD9 as it appears in World Health Organization (WHO). WHO Mortality Database Version September 2016. Geneva, Switzerland: World Health Organization (WHO), 2016
Belgium Vital Registration - Deaths 1991 ICD9 as it appears in World Health Organization (WHO). WHO Mortality Database Version September 2016. Geneva, Switzerland: World Health Organization (WHO), 2016
Belgium Vital Registration - Deaths 1992 ICD9 as it appears in World Health Organization (WHO). WHO Mortality Database Version November 2015. Geneva, Switzerland: World Health Organization (WHO), 2015
Belgium Vital Registration - Deaths 1993 ICD9 as it appears in World Health Organization (WHO). WHO Mortality Database Version November 2015. Geneva, Switzerland: World Health Organization (WHO), 2015
Belgium Vital Registration - Deaths 1994 ICD9 as it appears in World Health Organization (WHO). WHO Mortality Database Version November 2015. Geneva, Switzerland: World Health Organization (WHO), 2015
Belgium Vital Registration - Deaths 1995 ICD9 as it appears in World Health Organization (WHO). WHO Mortality Database Version November 2015. Geneva, Switzerland: World Health Organization (WHO), 2015
Belgium Vital Registration - Deaths 1996 ICD9 as it appears in World Health Organization (WHO). WHO Mortality Database Version November 2015. Geneva, Switzerland: World Health Organization (WHO), 2015
Belgium Vital Registration - Deaths 1997 ICD9 as it appears in World Health Organization (WHO). WHO Mortality Database Version November 2015. Geneva, Switzerland: World Health Organization (WHO), 2015
Belgium Vital Registration - Deaths 1998 ICD10 as it appears in World Health Organization (WHO). WHO Mortality Database Version October 2017. Geneva, Switzerland: World Health Organization (WHO), 2017
Belgium Vital Registration - Deaths 1999 ICD10 as it appears in World Health Organization (WHO). WHO Mortality Database Version October 2017. Geneva, Switzerland: World Health Organization (WHO), 2017
Belgium Vital Registration - Deaths 2004 ICD10 as it appears in World Health Organization (WHO). WHO Mortality Database Version October 2017. Geneva, Switzerland: World Health Organization (WHO), 2017
Belgium Vital Registration - Deaths 2000 ICD10 as it appears in World Health Organization (WHO). WHO Mortality Database Version October 2017. Geneva, Switzerland: World Health Organization (WHO), 2017
Belgium Vital Registration - Deaths 2001 ICD10 as it appears in World Health Organization (WHO). WHO Mortality Database Version October 2017. Geneva, Switzerland: World Health Organization (WHO), 2017
Belgium Vital Registration - Deaths 2002 ICD10 as it appears in World Health Organization (WHO). WHO Mortality Database Version October 2017. Geneva, Switzerland: World Health Organization (WHO), 2017

Belgium Vital Registration - Deaths 2003 ICD10 as it appears in World Health Organization (WHO). WHO Mortality Database Version October 2017. Geneva, Switzerland: World Health Organization (WHO), 2017

Belgium Vital Registration - Deaths 2005 ICD10 as it appears in World Health Organization (WHO). WHO Mortality Database Version October 2017. Geneva, Switzerland: World Health Organization (WHO), 2017

Belgium Vital Registration - Deaths 2006 ICD10 as it appears in World Health Organization (WHO). WHO Mortality Database Version October 2017. Geneva, Switzerland: World Health Organization (WHO), 2017

Belgium Vital Registration - Deaths 2007 ICD10 as it appears in World Health Organization (WHO). WHO Mortality Database Version October 2017. Geneva, Switzerland: World Health Organization (WHO), 2017

Belgium Vital Registration - Deaths 2008 ICD10 as it appears in World Health Organization (WHO). WHO Mortality Database Version October 2017. Geneva, Switzerland: World Health Organization (WHO), 2017

Belgium Vital Registration - Deaths 2009 ICD10 as it appears in World Health Organization (WHO). WHO Mortality Database Version October 2017. Geneva, Switzerland: World Health Organization (WHO), 2017

Belgium Vital Registration - Deaths 2010 ICD10 as it appears in World Health Organization (WHO). WHO Mortality Database Version October 2017. Geneva, Switzerland: World Health Organization (WHO), 2017

Belgium Vital Registration - Deaths 2011 ICD10 as it appears in World Health Organization (WHO). WHO Mortality Database Version October 2017. Geneva, Switzerland: World Health Organization (WHO), 2017

Belgium Vital Registration - Deaths 2012 ICD10 as it appears in World Health Organization (WHO). WHO Mortality Database Version October 2017. Geneva, Switzerland: World Health Organization (WHO), 2017

Belgium Vital Registration - Deaths 2013 ICD10 as it appears in World Health Organization (WHO). WHO Mortality Database Version October 2017. Geneva, Switzerland: World Health Organization (WHO), 2017

Belgium Vital Registration - Deaths 2014 ICD10 as it appears in World Health Organization (WHO). WHO Mortality Database Version March 2017. Geneva, Switzerland: World Health Organization (WHO), 2017

Belgium Vital Registration - Deaths 2015 ICD10 as it appears in World Health Organization (WHO). WHO Mortality Database Version April 2018. Geneva, Switzerland: World Health Organization (WHO), 2018

Belize Vital Registration - Deaths 1980 ICD9 as it appears in World Health Organization (WHO). WHO Mortality Database Version September 2016. Geneva, Switzerland: World Health Organization (WHO), 2016

Belize Vital Registration - Deaths 1981 ICD9 as it appears in World Health Organization (WHO). WHO Mortality Database Version September 2016. Geneva, Switzerland: World Health Organization (WHO), 2016

Belize Vital Registration - Deaths 1982 ICD9 as it appears in World Health Organization (WHO). WHO Mortality Database Version September 2016. Geneva, Switzerland: World Health Organization (WHO), 2016

Belize Vital Registration - Deaths 1983 ICD9 as it appears in World Health Organization (WHO). WHO Mortality Database Version September 2016. Geneva, Switzerland: World Health Organization (WHO), 2016

Belize Vital Registration - Deaths 1984 ICD9 as it appears in World Health Organization (WHO). WHO Mortality Database Version September 2016. Geneva, Switzerland: World Health Organization (WHO), 2016

Belize Vital Registration - Deaths 1986 ICD9 as it appears in World Health Organization (WHO). WHO Mortality Database Version September 2016. Geneva, Switzerland: World Health Organization (WHO), 2016

Belize Vital Registration - Deaths 1987 ICD9 as it appears in World Health Organization (WHO). WHO Mortality Database Version September 2016. Geneva, Switzerland: World Health Organization (WHO), 2016

Belize Vital Registration - Deaths 1989 ICD9 as it appears in World Health Organization (WHO). WHO Mortality Database Version September 2016. Geneva, Switzerland: World Health Organization (WHO), 2016

Belize Vital Registration - Deaths 1997 ICD10 as it appears in World Health Organization (WHO). WHO Mortality Database Version October 2017. Geneva, Switzerland:

World Health Organization (WHO), 2017

Belize Vital Registration - Deaths 1998 ICD10 as it appears in World Health Organization (WHO). WHO Mortality Database Version October 2017. Geneva, Switzerland: World Health Organization (WHO), 2017

Belize Vital Registration - Deaths 1999 ICD10 as it appears in World Health Organization (WHO). WHO Mortality Database Version October 2017. Geneva, Switzerland: World Health Organization (WHO), 2017

Belize Vital Registration - Deaths 2000 ICD10 as it appears in World Health Organization (WHO). WHO Mortality Database Version October 2017. Geneva, Switzerland: World Health Organization (WHO), 2017

Belize Vital Registration - Deaths 2001 ICD10 as it appears in World Health Organization (WHO). WHO Mortality Database Version October 2017. Geneva, Switzerland: World Health Organization (WHO), 2017

Belize Vital Registration - Deaths 2002 ICD10 as it appears in World Health Organization (WHO). WHO Mortality Database Version October 2017. Geneva, Switzerland: World Health Organization (WHO), 2017

Belize Vital Registration - Deaths 2003 ICD10 as it appears in World Health Organization (WHO). WHO Mortality Database Version October 2017. Geneva, Switzerland: World Health Organization (WHO), 2017

Belize Vital Registration - Deaths 2004 ICD10 as it appears in World Health Organization (WHO). WHO Mortality Database Version October 2017. Geneva, Switzerland: World Health Organization (WHO), 2017

Belize Vital Registration - Deaths 2005 ICD10 as it appears in World Health Organization (WHO). WHO Mortality Database Version October 2017. Geneva, Switzerland: World Health Organization (WHO), 2017

Belize Vital Registration - Deaths 1990 ICD9 as it appears in World Health Organization (WHO). WHO Mortality Database Version September 2016. Geneva, Switzerland: World Health Organization (WHO), 2016

Belize Vital Registration - Deaths 1991 ICD9 as it appears in World Health Organization (WHO). WHO Mortality Database Version September 2016. Geneva, Switzerland: World Health Organization (WHO), 2016

Belize Vital Registration - Deaths 1993 ICD9 as it appears in World Health Organization (WHO). WHO Mortality Database Version September 2016. Geneva, Switzerland: World Health Organization (WHO), 2016

Belize Vital Registration - Deaths 1994 ICD9 as it appears in World Health Organization (WHO). WHO Mortality Database Version September 2016. Geneva, Switzerland: World Health Organization (WHO), 2016

Belize Vital Registration - Deaths 1995 ICD9 as it appears in World Health Organization (WHO). WHO Mortality Database Version September 2016. Geneva, Switzerland: World Health Organization (WHO), 2016

Belize Vital Registration - Deaths 1996 ICD9 as it appears in World Health Organization (WHO). WHO Mortality Database Version September 2016. Geneva, Switzerland: World Health Organization (WHO), 2016

Belize Vital Registration - Deaths 2006 ICD10 as it appears in World Health Organization (WHO). WHO Mortality Database Version October 2017. Geneva, Switzerland: World Health Organization (WHO), 2017

Belize Vital Registration - Deaths 2007 ICD10 as it appears in World Health Organization (WHO). WHO Mortality Database Version October 2017. Geneva, Switzerland: World Health Organization (WHO), 2017

Belize Vital Registration - Deaths 2008 ICD10 as it appears in World Health Organization (WHO). WHO Mortality Database Version October 2017. Geneva, Switzerland: World Health Organization (WHO), 2017

Belize Vital Registration - Deaths 2009 ICD10 as it appears in World Health Organization (WHO). WHO Mortality Database Version October 2017. Geneva, Switzerland: World Health Organization (WHO), 2017

Belize Vital Registration - Deaths 2010 ICD10 as it appears in World Health Organization (WHO). WHO Mortality Database Version October 2017. Geneva, Switzerland: World Health Organization (WHO), 2017

Belize Vital Registration - Deaths 2011 ICD10 as it appears in World Health Organization (WHO). WHO Mortality Database Version October 2017. Geneva, Switzerland: World Health Organization (WHO), 2017

Belize Vital Registration - Deaths 2012 ICD10 as it appears in World Health Organization (WHO). WHO Mortality Database Version October 2017. Geneva, Switzerland: World Health Organization (WHO), 2017

Belize Vital Registration - Deaths 2013 ICD10 as it appears in World Health Organization (WHO). WHO Mortality Database Version October 2017. Geneva, Switzerland: World Health Organization (WHO), 2017

Belize Vital Registration - Deaths 2014 ICD10 as it appears in World Health Organization (WHO). WHO Mortality Database Version October 2017. Geneva, Switzerland: World Health Organization (WHO), 2017

Belize Vital Registration - Deaths 2015 ICD10 as it appears in World Health Organization (WHO). WHO Mortality Database Version October 2017. Geneva, Switzerland: World Health Organization (WHO), 2017

Bermuda Vital Registration - Deaths 1980 ICD9 as it appears in World Health Organization (WHO). WHO Mortality Database Version September 2016. Geneva, Switzerland: World Health Organization (WHO), 2016

Bermuda Vital Registration - Deaths 1983 ICD9 as it appears in World Health Organization (WHO). WHO Mortality Database Version September 2016. Geneva, Switzerland: World Health Organization (WHO), 2016

Bermuda Vital Registration - Deaths 1984 ICD9 as it appears in World Health Organization (WHO). WHO Mortality Database Version September 2016. Geneva, Switzerland: World Health Organization (WHO), 2016

Bermuda Vital Registration - Deaths 1985 ICD9 as it appears in World Health Organization (WHO). WHO Mortality Database Version September 2016. Geneva, Switzerland: World Health Organization (WHO), 2016

Bermuda Vital Registration - Deaths 1986 ICD9 as it appears in World Health Organization (WHO). WHO Mortality Database Version September 2016. Geneva, Switzerland: World Health Organization (WHO), 2016

Bermuda Vital Registration - Deaths 1987 ICD9 as it appears in World Health Organization (WHO). WHO Mortality Database Version September 2016. Geneva, Switzerland: World Health Organization (WHO), 2016

Bermuda Vital Registration - Deaths 1988 ICD9 as it appears in World Health Organization (WHO). WHO Mortality Database Version September 2016. Geneva, Switzerland: World Health Organization (WHO), 2016

Bermuda Vital Registration - Deaths 1989 ICD9 as it appears in World Health Organization (WHO). WHO Mortality Database Version September 2016. Geneva, Switzerland: World Health Organization (WHO), 2016

Bermuda Vital Registration - Deaths 1990 ICD9 as it appears in World Health Organization (WHO). WHO Mortality Database Version September 2016. Geneva, Switzerland: World Health Organization (WHO), 2016

Bermuda Vital Registration - Deaths 1991 ICD9 as it appears in World Health Organization (WHO). WHO Mortality Database Version September 2016. Geneva, Switzerland: World Health Organization (WHO), 2016

Bermuda Vital Registration - Deaths 1992 ICD9 as it appears in World Health Organization (WHO). WHO Mortality Database Version September 2016. Geneva, Switzerland: World Health Organization (WHO), 2016

Bermuda Vital Registration - Deaths 1993 ICD9 as it appears in World Health Organization (WHO). WHO Mortality Database Version September 2016. Geneva, Switzerland: World Health Organization (WHO), 2016

Bermuda Vital Registration - Deaths 1994 ICD9 as it appears in World Health Organization (WHO). WHO Mortality Database Version September 2016. Geneva, Switzerland: World Health Organization (WHO), 2016

Bermuda Vital Registration - Deaths 1995 ICD9 as it appears in World Health Organization (WHO). WHO Mortality Database Version September 2016. Geneva, Switzerland: World Health Organization (WHO), 2016

Bermuda Vital Registration - Deaths 1996 ICD10 as it appears in World Health Organization (WHO). WHO Mortality Database Version October 2017. Geneva, Switzerland: World Health Organization (WHO), 2017

Bermuda Vital Registration - Deaths 1997 ICD10 as it appears in World Health Organization (WHO). WHO Mortality Database Version October 2017. Geneva, Switzerland: World Health Organization (WHO), 2017

Bermuda Vital Registration - Deaths 1998 ICD10 as it appears in World Health Organization (WHO). WHO Mortality Database Version October 2017. Geneva, Switzerland: World Health Organization (WHO), 2017

Bermuda Vital Registration - Deaths 1999 ICD10 as it appears in World Health Organization (WHO). WHO Mortality Database Version October 2017. Geneva, Switzerland: World Health Organization (WHO), 2017

Bermuda Vital Registration - Deaths 2000 ICD10 as it appears in World Health Organization (WHO). WHO Mortality Database Version October 2017. Geneva, Switzerland: World Health Organization (WHO), 2017

Bermuda Vital Registration - Deaths 2001 ICD10 as it appears in World Health Organization (WHO). WHO Mortality Database Version October 2017. Geneva, Switzerland: World Health Organization (WHO), 2017

Bermuda Vital Registration - Deaths 2002 ICD10 as it appears in World Health Organization (WHO). WHO Mortality Database Version October 2017. Geneva, Switzerland: World Health Organization (WHO), 2017

Bermuda Vital Registration - Deaths 2003 ICD10 as it appears in World Health Organization (WHO). WHO Mortality Database Version October 2017. Geneva, Switzerland: World Health Organization (WHO), 2017

Bermuda Vital Registration - Deaths 2004 ICD10 as it appears in World Health Organization (WHO). WHO Mortality Database Version October 2017. Geneva, Switzerland: World Health Organization (WHO), 2017

Bermuda Vital Registration - Deaths 2005 ICD10 as it appears in World Health Organization (WHO). WHO Mortality Database Version October 2017. Geneva, Switzerland: World Health Organization (WHO), 2017

Bermuda Vital Registration - Deaths 2006 ICD10 as it appears in World Health Organization (WHO). WHO Mortality Database Version October 2017. Geneva, Switzerland: World Health Organization (WHO), 2017

Bermuda Vital Registration - Deaths 2007 ICD10 as it appears in World Health Organization (WHO). WHO Mortality Database Version October 2017. Geneva, Switzerland: World Health Organization (WHO), 2017

Bermuda Vital Registration - Deaths 2008 ICD10 as it appears in World Health Organization (WHO). WHO Mortality Database Version October 2017. Geneva, Switzerland: World Health Organization (WHO), 2017

Bermuda Vital Registration - Deaths 2009 ICD10 as it appears in World Health Organization (WHO). WHO Mortality Database Version October 2017. Geneva, Switzerland: World Health Organization (WHO), 2017

Bermuda Vital Registration - Deaths 2010 ICD10 as it appears in World Health Organization (WHO). WHO Mortality Database Version October 2017. Geneva, Switzerland: World Health Organization (WHO), 2017

Bermuda Vital Registration - Deaths 2011 ICD10 as it appears in World Health Organization (WHO). WHO Mortality Database Version October 2017. Geneva, Switzerland: World Health Organization (WHO), 2017

Bermuda Vital Registration - Deaths 2012 ICD10 as it appears in World Health Organization (WHO). WHO Mortality Database Version October 2017. Geneva, Switzerland: World Health Organization (WHO), 2017

Bermuda Vital Registration - Deaths 2013 ICD10 as it appears in World Health Organization (WHO). WHO Mortality Database Version October 2017. Geneva, Switzerland: World Health Organization (WHO), 2017

Bermuda Vital Registration - Deaths 2014 ICD10 as it appears in World Health Organization (WHO). WHO Mortality Database Version October 2017. Geneva, Switzerland: World Health Organization (WHO), 2017

Bermuda Vital Registration - Deaths 2015 ICD10 as it appears in World Health Organization (WHO). WHO Mortality Database Version October 2017. Geneva, Switzerland: World Health Organization (WHO), 2017

Births and Deaths Registry (Ghana). Ghana - Accra Births and Deaths Registry - Deaths 2000-2007

Bosnia and Herzegovina Vital Registration - Deaths 1991 ICD9 as it appears in World Health Organization (WHO). WHO Mortality Database Version September 2016. Geneva, Switzerland: World Health Organization (WHO), 2016

Bosnia and Herzegovina Vital Registration - Deaths 2011 ICD10 as it appears in World Health Organization (WHO). WHO Mortality Database Version October 2017. Geneva, Switzerland: World Health Organization (WHO), 2017

Bosnia and Herzegovina Vital Registration - Deaths 2014 ICD10 as it appears in World Health Organization (WHO). WHO Mortality Database Version October 2017. Geneva, Switzerland: World Health Organization (WHO), 2017
Brunei Vital Registration - Deaths 2011 ICD10 as it appears in World Health Organization (WHO). WHO Mortality Database Version October 2017. Geneva, Switzerland: World Health Organization (WHO), 2017
Brunei Vital Registration - Deaths 2012 ICD10 as it appears in World Health Organization (WHO). WHO Mortality Database Version October 2017. Geneva, Switzerland: World Health Organization (WHO), 2017
Brunei Vital Registration - Deaths 2013 ICD10 as it appears in World Health Organization (WHO). WHO Mortality Database Version October 2017. Geneva, Switzerland: World Health Organization (WHO), 2017
Brunei Vital Registration - Deaths 2014 ICD10 as it appears in World Health Organization (WHO). WHO Mortality Database Version October 2017. Geneva, Switzerland: World Health Organization (WHO), 2017
Brunei Vital Registration - Deaths 2015 ICD10 as it appears in World Health Organization (WHO). WHO Mortality Database Version October 2017. Geneva, Switzerland: World Health Organization (WHO), 2017
Bulgaria Vital Registration - Deaths 1980 ICD9 as it appears in World Health Organization (WHO). WHO Mortality Database Version September 2016. Geneva, Switzerland: World Health Organization (WHO), 2016
Bulgaria Vital Registration - Deaths 1981 ICD9 as it appears in World Health Organization (WHO). WHO Mortality Database Version September 2016. Geneva, Switzerland: World Health Organization (WHO), 2016
Bulgaria Vital Registration - Deaths 1982 ICD9 as it appears in World Health Organization (WHO). WHO Mortality Database Version September 2016. Geneva, Switzerland: World Health Organization (WHO), 2016
Bulgaria Vital Registration - Deaths 1983 ICD9 as it appears in World Health Organization (WHO). WHO Mortality Database Version September 2016. Geneva, Switzerland: World Health Organization (WHO), 2016
Bulgaria Vital Registration - Deaths 1984 ICD9 as it appears in World Health Organization (WHO). WHO Mortality Database Version September 2016. Geneva, Switzerland: World Health Organization (WHO), 2016
Bulgaria Vital Registration - Deaths 1985 ICD9 as it appears in World Health Organization (WHO). WHO Mortality Database Version September 2016. Geneva, Switzerland: World Health Organization (WHO), 2016
Bulgaria Vital Registration - Deaths 1986 ICD9 as it appears in World Health Organization (WHO). WHO Mortality Database Version September 2016. Geneva, Switzerland: World Health Organization (WHO), 2016
Bulgaria Vital Registration - Deaths 1987 ICD9 as it appears in World Health Organization (WHO). WHO Mortality Database Version September 2016. Geneva, Switzerland: World Health Organization (WHO), 2016
Bulgaria Vital Registration - Deaths 1988 ICD9 as it appears in World Health Organization (WHO). WHO Mortality Database Version September 2016. Geneva, Switzerland: World Health Organization (WHO), 2016
Bulgaria Vital Registration - Deaths 1989 ICD9 as it appears in World Health Organization (WHO). WHO Mortality Database Version September 2016. Geneva, Switzerland: World Health Organization (WHO), 2016
Bulgaria Vital Registration - Deaths 1990 ICD9 as it appears in World Health Organization (WHO). WHO Mortality Database Version November 2015. Geneva, Switzerland: World Health Organization (WHO), 2015
Bulgaria Vital Registration - Deaths 1991 ICD9 as it appears in World Health Organization (WHO). WHO Mortality Database Version November 2015. Geneva, Switzerland: World Health Organization (WHO), 2015
Bulgaria Vital Registration - Deaths 1992 ICD9 as it appears in World Health Organization (WHO). WHO Mortality Database Version November 2015. Geneva, Switzerland: World Health Organization (WHO), 2015
Bulgaria Vital Registration - Deaths 1993 ICD9 as it appears in World Health Organization (WHO). WHO Mortality Database Version November 2015. Geneva, Switzerland: World Health Organization (WHO), 2015

Bulgaria Vital Registration - Deaths 1994 ICD9 as it appears in World Health Organization (WHO). WHO Mortality Database Version November 2015. Geneva, Switzerland: World Health Organization (WHO), 2015

Bulgaria Vital Registration - Deaths 1995 ICD9 as it appears in World Health Organization (WHO). WHO Mortality Database Version November 2015. Geneva, Switzerland: World Health Organization (WHO), 2015

Bulgaria Vital Registration - Deaths 1996 ICD9 as it appears in World Health Organization (WHO). WHO Mortality Database Version November 2015. Geneva, Switzerland: World Health Organization (WHO), 2015

Bulgaria Vital Registration - Deaths 1997 ICD9 as it appears in World Health Organization (WHO). WHO Mortality Database Version November 2015. Geneva, Switzerland: World Health Organization (WHO), 2015

Bulgaria Vital Registration - Deaths 1998 ICD9 as it appears in World Health Organization (WHO). WHO Mortality Database Version November 2015. Geneva, Switzerland: World Health Organization (WHO), 2015

Bulgaria Vital Registration - Deaths 1999 ICD9 as it appears in World Health Organization (WHO). WHO Mortality Database Version November 2015. Geneva, Switzerland: World Health Organization (WHO), 2015

Bulgaria Vital Registration - Deaths 2000 ICD9 as it appears in World Health Organization (WHO). WHO Mortality Database Version November 2015. Geneva, Switzerland: World Health Organization (WHO), 2015

Bulgaria Vital Registration - Deaths 2001 ICD9 as it appears in World Health Organization (WHO). WHO Mortality Database Version November 2015. Geneva, Switzerland: World Health Organization (WHO), 2015

Bulgaria Vital Registration - Deaths 2002 ICD9 as it appears in World Health Organization (WHO). WHO Mortality Database Version November 2015. Geneva, Switzerland: World Health Organization (WHO), 2015

Bulgaria Vital Registration - Deaths 2003 ICD9 as it appears in World Health Organization (WHO). WHO Mortality Database Version November 2015. Geneva, Switzerland: World Health Organization (WHO), 2015

Bulgaria Vital Registration - Deaths 2004 ICD9 as it appears in World Health Organization (WHO). WHO Mortality Database Version November 2015. Geneva, Switzerland: World Health Organization (WHO), 2015

Bulgaria Vital Registration - Deaths 2005 ICD10 as it appears in World Health Organization (WHO). WHO Mortality Database Version October 2017. Geneva, Switzerland: World Health Organization (WHO), 2017

Bulgaria Vital Registration - Deaths 2006 ICD10 as it appears in World Health Organization (WHO). WHO Mortality Database Version October 2017. Geneva, Switzerland: World Health Organization (WHO), 2017

Bulgaria Vital Registration - Deaths 2007 ICD10 as it appears in World Health Organization (WHO). WHO Mortality Database Version October 2017. Geneva, Switzerland: World Health Organization (WHO), 2017

Bulgaria Vital Registration - Deaths 2008 ICD10 as it appears in World Health Organization (WHO). WHO Mortality Database Version October 2017. Geneva, Switzerland: World Health Organization (WHO), 2017

Bulgaria Vital Registration - Deaths 2009 ICD10 as it appears in World Health Organization (WHO). WHO Mortality Database Version October 2017. Geneva, Switzerland: World Health Organization (WHO), 2017

Bulgaria Vital Registration - Deaths 2010 ICD10 as it appears in World Health Organization (WHO). WHO Mortality Database Version October 2017. Geneva, Switzerland: World Health Organization (WHO), 2017

Bulgaria Vital Registration - Deaths 2011 ICD10 as it appears in World Health Organization (WHO). WHO Mortality Database Version October 2017. Geneva, Switzerland: World Health Organization (WHO), 2017

Bulgaria Vital Registration - Deaths 2012 ICD10 as it appears in World Health Organization (WHO). WHO Mortality Database Version October 2017. Geneva, Switzerland: World Health Organization (WHO), 2017

Bulgaria Vital Registration - Deaths 2013 ICD10 as it appears in World Health Organization (WHO). WHO Mortality Database Version October 2017. Geneva, Switzerland: World Health Organization (WHO), 2017

Bulgaria Vital Registration - Deaths 2014 ICD10 as it appears in World Health Organization (WHO). WHO Mortality Database Version October 2017. Geneva, Switzerland: World Health Organization (WHO), 2017
Canada Vital Registration - Deaths 1980 ICD9 as it appears in World Health Organization (WHO). WHO Mortality Database Version September 2016. Geneva, Switzerland: World Health Organization (WHO), 2016
Canada Vital Registration - Deaths 1981 ICD9 as it appears in World Health Organization (WHO). WHO Mortality Database Version September 2016. Geneva, Switzerland: World Health Organization (WHO), 2016
Canada Vital Registration - Deaths 1982 ICD9 as it appears in World Health Organization (WHO). WHO Mortality Database Version September 2016. Geneva, Switzerland: World Health Organization (WHO), 2016
Canada Vital Registration - Deaths 1983 ICD9 as it appears in World Health Organization (WHO). WHO Mortality Database Version September 2016. Geneva, Switzerland: World Health Organization (WHO), 2016
Canada Vital Registration - Deaths 1984 ICD9 as it appears in World Health Organization (WHO). WHO Mortality Database Version September 2016. Geneva, Switzerland: World Health Organization (WHO), 2016
Canada Vital Registration - Deaths 1985 ICD9 as it appears in World Health Organization (WHO). WHO Mortality Database Version September 2016. Geneva, Switzerland: World Health Organization (WHO), 2016
Canada Vital Registration - Deaths 1986 ICD9 as it appears in World Health Organization (WHO). WHO Mortality Database Version November 2015. Geneva, Switzerland: World Health Organization (WHO), 2015
Canada Vital Registration - Deaths 1987 ICD9 as it appears in World Health Organization (WHO). WHO Mortality Database Version November 2015. Geneva, Switzerland: World Health Organization (WHO), 2015
Canada Vital Registration - Deaths 1988 ICD9 as it appears in World Health Organization (WHO). WHO Mortality Database Version November 2015. Geneva, Switzerland: World Health Organization (WHO), 2015
Canada Vital Registration - Deaths 1989 ICD9 as it appears in World Health Organization (WHO). WHO Mortality Database Version November 2015. Geneva, Switzerland: World Health Organization (WHO), 2015
Canada Vital Registration - Deaths 1990 ICD9 as it appears in World Health Organization (WHO). WHO Mortality Database Version November 2015. Geneva, Switzerland: World Health Organization (WHO), 2015
Canada Vital Registration - Deaths 1991 ICD9 as it appears in World Health Organization (WHO). WHO Mortality Database Version November 2015. Geneva, Switzerland: World Health Organization (WHO), 2015
Canada Vital Registration - Deaths 1992 ICD9 as it appears in World Health Organization (WHO). WHO Mortality Database Version November 2015. Geneva, Switzerland: World Health Organization (WHO), 2015
Canada Vital Registration - Deaths 1993 ICD9 as it appears in World Health Organization (WHO). WHO Mortality Database Version November 2015. Geneva, Switzerland: World Health Organization (WHO), 2015
Canada Vital Registration - Deaths 1994 ICD9 as it appears in World Health Organization (WHO). WHO Mortality Database Version November 2015. Geneva, Switzerland: World Health Organization (WHO), 2015
Canada Vital Registration - Deaths 1995 ICD9 as it appears in World Health Organization (WHO). WHO Mortality Database Version November 2015. Geneva, Switzerland: World Health Organization (WHO), 2015
Canada Vital Registration - Deaths 1996 ICD9 as it appears in World Health Organization (WHO). WHO Mortality Database Version November 2015. Geneva, Switzerland: World Health Organization (WHO), 2015
Canada Vital Registration - Deaths 1997 ICD9 as it appears in World Health Organization (WHO). WHO Mortality Database Version November 2015. Geneva, Switzerland: World Health Organization (WHO), 2015
Canada Vital Registration - Deaths 1998 ICD9 as it appears in World Health Organization (WHO). WHO Mortality Database Version November 2015. Geneva, Switzerland: World Health Organization (WHO), 2015

Canada Vital Registration - Deaths 1999 ICD9 as it appears in World Health Organization (WHO). WHO Mortality Database Version November 2015. Geneva, Switzerland: World Health Organization (WHO), 2015
Canada Vital Registration - Deaths 2000 ICD10 as it appears in World Health Organization (WHO). WHO Mortality Database Version October 2017. Geneva, Switzerland: World Health Organization (WHO), 2017
Canada Vital Registration - Deaths 2001 ICD10 as it appears in World Health Organization (WHO). WHO Mortality Database Version October 2017. Geneva, Switzerland: World Health Organization (WHO), 2017
Canada Vital Registration - Deaths 2002 ICD10 as it appears in World Health Organization (WHO). WHO Mortality Database Version October 2017. Geneva, Switzerland: World Health Organization (WHO), 2017
Canada Vital Registration - Deaths 2003 ICD10 as it appears in World Health Organization (WHO). WHO Mortality Database Version October 2017. Geneva, Switzerland: World Health Organization (WHO), 2017
Canada Vital Registration - Deaths 2004 ICD10 as it appears in World Health Organization (WHO). WHO Mortality Database Version October 2017. Geneva, Switzerland: World Health Organization (WHO), 2017
Canada Vital Registration - Deaths 2005 ICD10 as it appears in World Health Organization (WHO). WHO Mortality Database Version October 2017. Geneva, Switzerland: World Health Organization (WHO), 2017
Canada Vital Registration - Deaths 2006 ICD10 as it appears in World Health Organization (WHO). WHO Mortality Database Version October 2017. Geneva, Switzerland: World Health Organization (WHO), 2017
Canada Vital Registration - Deaths 2007 ICD10 as it appears in World Health Organization (WHO). WHO Mortality Database Version October 2017. Geneva, Switzerland: World Health Organization (WHO), 2017
Canada Vital Registration - Deaths 2008 ICD10 as it appears in World Health Organization (WHO). WHO Mortality Database Version October 2017. Geneva, Switzerland: World Health Organization (WHO), 2017
Canada Vital Registration - Deaths 2009 ICD10 as it appears in World Health Organization (WHO). WHO Mortality Database Version October 2017. Geneva, Switzerland: World Health Organization (WHO), 2017
Canada Vital Registration - Deaths 2010 ICD10 as it appears in World Health Organization (WHO). WHO Mortality Database Version October 2017. Geneva, Switzerland: World Health Organization (WHO), 2017
Canada Vital Registration - Deaths 2011 ICD10 as it appears in World Health Organization (WHO). WHO Mortality Database Version October 2017. Geneva, Switzerland: World Health Organization (WHO), 2017
Canada Vital Registration - Deaths 2012 ICD10 as it appears in World Health Organization (WHO). WHO Mortality Database Version March 2017. Geneva, Switzerland: World Health Organization (WHO), 2017
Canada Vital Registration - Deaths 2013 ICD10 as it appears in World Health Organization (WHO). WHO Mortality Database Version October 2017. Geneva, Switzerland: World Health Organization (WHO), 2017
Cape Verde Vital Registration - Deaths 1980 ICD8 as it appears in World Health Organization (WHO). WHO Mortality Database Version July 2012. Geneva, Switzerland: World Health Organization (WHO), 2012
Cape Verde Vital Registration - Deaths 2011 ICD10 as it appears in World Health Organization (WHO). WHO Mortality Database Version October 2017. Geneva, Switzerland: World Health Organization (WHO), 2017
Cape Verde Vital Registration - Deaths 2012 ICD10 as it appears in World Health Organization (WHO). WHO Mortality Database Version October 2017. Geneva, Switzerland: World Health Organization (WHO), 2017
Centers for Disease Control and Prevention (CDC), INDEPTH, International Vaccine Institute. Bangladesh - Abhoynagar, Mirsarai, and Kamalapur Health and Demographic Surveillance System
Chief Medical Office of Greenland. Greenland Vital Registration - Deaths 1995
Chief Medical Office of Greenland. Greenland Vital Registration - Deaths 1996
Chief Medical Office of Greenland. Greenland Vital Registration - Deaths 1997

Chief Medical Office of Greenland. Greenland Vital Registration - Deaths 1998
Chief Medical Office of Greenland. Greenland Vital Registration - Deaths 1999
Chief Medical Office of Greenland. Greenland Vital Registration - Deaths 2000
Chief Medical Office of Greenland. Greenland Vital Registration - Deaths 2001
Chief Medical Office of Greenland. Greenland Vital Registration - Deaths 2002
Chief Medical Office of Greenland. Greenland Vital Registration - Deaths 2003
Chief Medical Office of Greenland. Greenland Vital Registration - Deaths 2004
Chief Medical Office of Greenland. Greenland Vital Registration - Deaths 2005
Chief Medical Office of Greenland. Greenland Vital Registration - Deaths 2006
Chief Medical Office of Greenland. Greenland Vital Registration - Deaths 2007
Chief Medical Office of Greenland. Greenland Vital Registration - Deaths 2008
Chief Medical Office of Greenland. Greenland Vital Registration - Deaths 2009
Chief Medical Office of Greenland. Greenland Vital Registration - Deaths 2010
Chief Medical Office of Greenland. Greenland Vital Registration - Deaths 2011
Chief Medical Office of Greenland. Greenland Vital Registration - Deaths 2012
Chief Medical Office of Greenland. Greenland Vital Registration - Deaths 2013
Chief Medical Office of Greenland. Greenland Vital Registration - Deaths 2014
Chief Medical Office of Greenland. Greenland Vital Registration - Deaths 2015
Chile Vital Registration - Deaths 1980 ICD9 as it appears in World Health Organization (WHO). WHO Mortality Database Version September 2016. Geneva, Switzerland: World Health Organization (WHO), 2016
Chile Vital Registration - Deaths 1981 ICD9 as it appears in World Health Organization (WHO). WHO Mortality Database Version September 2016. Geneva, Switzerland: World Health Organization (WHO), 2016
Chile Vital Registration - Deaths 1982 ICD9 as it appears in World Health Organization (WHO). WHO Mortality Database Version September 2016. Geneva, Switzerland: World Health Organization (WHO), 2016
Chile Vital Registration - Deaths 1983 ICD9 as it appears in World Health Organization (WHO). WHO Mortality Database Version September 2016. Geneva, Switzerland: World Health Organization (WHO), 2016
Chile Vital Registration - Deaths 1984 ICD9 as it appears in World Health Organization (WHO). WHO Mortality Database Version September 2016. Geneva, Switzerland: World Health Organization (WHO), 2016
Chile Vital Registration - Deaths 1986 ICD9 as it appears in World Health Organization (WHO). WHO Mortality Database Version September 2016. Geneva, Switzerland: World Health Organization (WHO), 2016
Chile Vital Registration - Deaths 1987 ICD9 as it appears in World Health Organization (WHO). WHO Mortality Database Version September 2016. Geneva, Switzerland: World Health Organization (WHO), 2016
Chile Vital Registration - Deaths 1988 ICD9 as it appears in World Health Organization (WHO). WHO Mortality Database Version September 2016. Geneva, Switzerland: World Health Organization (WHO), 2016
Chile Vital Registration - Deaths 1989 ICD9 as it appears in World Health Organization (WHO). WHO Mortality Database Version September 2016. Geneva, Switzerland: World Health Organization (WHO), 2016
Chile Vital Registration - Deaths 1997 ICD10 as it appears in World Health Organization (WHO). WHO Mortality Database Version October 2017. Geneva, Switzerland: World Health Organization (WHO), 2017

Chile Vital Registration - Deaths 1998 ICD10 as it appears in World Health Organization (WHO). WHO Mortality Database Version October 2017. Geneva, Switzerland: World Health Organization (WHO), 2017
Chile Vital Registration - Deaths 1999 ICD10 as it appears in World Health Organization (WHO). WHO Mortality Database Version October 2017. Geneva, Switzerland: World Health Organization (WHO), 2017
Chile Vital Registration - Deaths 2000 ICD10 as it appears in World Health Organization (WHO). WHO Mortality Database Version October 2017. Geneva, Switzerland: World Health Organization (WHO), 2017
Chile Vital Registration - Deaths 2001 ICD10 as it appears in World Health Organization (WHO). WHO Mortality Database Version October 2017. Geneva, Switzerland: World Health Organization (WHO), 2017
Chile Vital Registration - Deaths 2002 ICD10 as it appears in World Health Organization (WHO). WHO Mortality Database Version October 2017. Geneva, Switzerland: World Health Organization (WHO), 2017
Chile Vital Registration - Deaths 2003 ICD10 as it appears in World Health Organization (WHO). WHO Mortality Database Version October 2017. Geneva, Switzerland: World Health Organization (WHO), 2017
Chile Vital Registration - Deaths 2004 ICD10 as it appears in World Health Organization (WHO). WHO Mortality Database Version October 2017. Geneva, Switzerland: World Health Organization (WHO), 2017
Chile Vital Registration - Deaths 2005 ICD10 as it appears in World Health Organization (WHO). WHO Mortality Database Version October 2017. Geneva, Switzerland: World Health Organization (WHO), 2017
Chile Vital Registration - Deaths 2006 ICD10 as it appears in World Health Organization (WHO). WHO Mortality Database Version October 2017. Geneva, Switzerland: World Health Organization (WHO), 2017
Chile Vital Registration - Deaths 2007 ICD10 as it appears in World Health Organization (WHO). WHO Mortality Database Version October 2017. Geneva, Switzerland: World Health Organization (WHO), 2017
Chile Vital Registration - Deaths 2008 ICD10 as it appears in World Health Organization (WHO). WHO Mortality Database Version October 2017. Geneva, Switzerland: World Health Organization (WHO), 2017
Chile Vital Registration - Deaths 2009 ICD10 as it appears in World Health Organization (WHO). WHO Mortality Database Version October 2017. Geneva, Switzerland: World Health Organization (WHO), 2017
Chile Vital Registration - Deaths 2010 ICD10 as it appears in World Health Organization (WHO). WHO Mortality Database Version October 2017. Geneva, Switzerland: World Health Organization (WHO), 2017
Chile Vital Registration - Deaths 2011 ICD10 as it appears in World Health Organization (WHO). WHO Mortality Database Version October 2017. Geneva, Switzerland: World Health Organization (WHO), 2017
Chile Vital Registration - Deaths 2012 ICD10 as it appears in World Health Organization (WHO). WHO Mortality Database Version October 2017. Geneva, Switzerland: World Health Organization (WHO), 2017
Chile Vital Registration - Deaths 2013 ICD10 as it appears in World Health Organization (WHO). WHO Mortality Database Version October 2017. Geneva, Switzerland: World Health Organization (WHO), 2017
Chile Vital Registration - Deaths 2014 ICD10 as it appears in World Health Organization (WHO). WHO Mortality Database Version October 2017. Geneva, Switzerland: World Health Organization (WHO), 2017
Chile Vital Registration - Deaths 2015 ICD10 as it appears in World Health Organization (WHO). WHO Mortality Database Version October 2017. Geneva, Switzerland: World Health Organization (WHO), 2017
Chinese Center for Disease Control and Prevention (CCDC). China Disease Surveillance Points 1991 - China CDC
Chinese Center for Disease Control and Prevention (CCDC). China Disease Surveillance Points 1992 - China CDC
Chinese Center for Disease Control and Prevention (CCDC). China Disease Surveillance Points 1993 - China CDC
Chinese Center for Disease Control and Prevention (CCDC). China Disease Surveillance Points 1994 - China CDC

Chinese Center for Disease Control and Prevention (CCDC). China Disease Surveillance Points 1995 - China CDC
Chinese Center for Disease Control and Prevention (CCDC). China Disease Surveillance Points 1996 - China CDC
Chinese Center for Disease Control and Prevention (CCDC). China Disease Surveillance Points 1997 - China CDC
Chinese Center for Disease Control and Prevention (CCDC). China Disease Surveillance Points 1998 - China CDC
Chinese Center for Disease Control and Prevention (CCDC). China Disease Surveillance Points 1999 - China CDC
Chinese Center for Disease Control and Prevention (CCDC). China Disease Surveillance Points 2000 - China CDC
Chinese Center for Disease Control and Prevention (CCDC). China Disease Surveillance Points 2001 - China CDC
Chinese Center for Disease Control and Prevention (CCDC). China Disease Surveillance Points 2002 - China CDC
Chinese Center for Disease Control and Prevention (CCDC). China Disease Surveillance Points 2004 - China CDC
Chinese Center for Disease Control and Prevention (CCDC). China Disease Surveillance Points 2005 - China CDC
Chinese Center for Disease Control and Prevention (CCDC). China Disease Surveillance Points 2006 - China CDC
Chinese Center for Disease Control and Prevention (CCDC). China Disease Surveillance Points 2007 - China CDC
Chinese Center for Disease Control and Prevention (CCDC). China Disease Surveillance Points 2013 - China CDC
Chinese Center for Disease Control and Prevention (CCDC). China Disease Surveillance Points 2014 - China CDC
Chinese Center for Disease Control and Prevention (CCDC). China Disease Surveillance Points 2015 - China CDC
Chinese Center for Disease Control and Prevention (CCDC). China Disease Surveillance Points 2016 - China CDC
Chinese Center for Disease Control and Prevention (CCDC). China Disease Surveillance Points and Death Registration 2008 - China CDC
Chinese Center for Disease Control and Prevention (CCDC). China Disease Surveillance Points and Death Registration 2009 - China CDC
Chinese Center for Disease Control and Prevention (CCDC). China Disease Surveillance Points and Death Registration 2010 - China CDC
Chinese Center for Disease Control and Prevention (CCDC). China Disease Surveillance Points and Death Registration 2011 - China CDC
Chinese Center for Disease Control and Prevention (CCDC). China Disease Surveillance Points and Death Registration 2012 - China CDC
Colombia Vital Registration - Deaths 1997 ICD10 as it appears in World Health Organization (WHO). WHO Mortality Database Version October 2017. Geneva, Switzerland: World Health Organization (WHO), 2017
Colombia Vital Registration - Deaths 1998 ICD10 as it appears in World Health Organization (WHO). WHO Mortality Database Version October 2017. Geneva, Switzerland: World Health Organization (WHO), 2017
Colombia Vital Registration - Deaths 1999 ICD10 as it appears in World Health Organization (WHO). WHO Mortality Database Version October 2017. Geneva, Switzerland: World Health Organization (WHO), 2017
Colombia Vital Registration - Deaths 2000 ICD10 as it appears in World Health Organization (WHO). WHO Mortality Database Version October 2017. Geneva, Switzerland: World Health Organization (WHO), 2017
Colombia Vital Registration - Deaths 2001 ICD10 as it appears in World Health Organization (WHO). WHO Mortality Database Version October 2017. Geneva, Switzerland: World Health Organization (WHO), 2017
Colombia Vital Registration - Deaths 2002 ICD10 as it appears in World Health Organization (WHO). WHO Mortality Database Version October 2017. Geneva, Switzerland: World Health Organization (WHO), 2017
Colombia Vital Registration - Deaths 2003 ICD10 as it appears in World Health Organization (WHO). WHO Mortality Database Version April 2018. Geneva, Switzerland: World Health Organization (WHO), 2018
Colombia Vital Registration - Deaths 2004 ICD10 as it appears in World Health Organization (WHO). WHO Mortality Database Version October 2017. Geneva, Switzerland: World Health Organization (WHO), 2017

Colombia Vital Registration - Deaths 2005 ICD10 as it appears in World Health Organization (WHO). WHO Mortality Database Version October 2017. Geneva, Switzerland: World Health Organization (WHO), 2017
Colombia Vital Registration - Deaths 2006 ICD10 as it appears in World Health Organization (WHO). WHO Mortality Database Version October 2017. Geneva, Switzerland: World Health Organization (WHO), 2017
Colombia Vital Registration - Deaths 2007 ICD10 as it appears in World Health Organization (WHO). WHO Mortality Database Version October 2017. Geneva, Switzerland: World Health Organization (WHO), 2017
Colombia Vital Registration - Deaths 2008 ICD10 as it appears in World Health Organization (WHO). WHO Mortality Database Version October 2017. Geneva, Switzerland: World Health Organization (WHO), 2017
Colombia Vital Registration - Deaths 2009 ICD10 as it appears in World Health Organization (WHO). WHO Mortality Database Version October 2017. Geneva, Switzerland: World Health Organization (WHO), 2017
Colombia Vital Registration - Deaths 2010 ICD10 as it appears in World Health Organization (WHO). WHO Mortality Database Version October 2017. Geneva, Switzerland: World Health Organization (WHO), 2017
Colombia Vital Registration - Deaths 2011 ICD10 as it appears in World Health Organization (WHO). WHO Mortality Database Version October 2017. Geneva, Switzerland: World Health Organization (WHO), 2017
Colombia Vital Registration - Deaths 2012 ICD10 as it appears in World Health Organization (WHO). WHO Mortality Database Version October 2017. Geneva, Switzerland: World Health Organization (WHO), 2017
Colombia Vital Registration - Deaths 2013 ICD10 as it appears in World Health Organization (WHO). WHO Mortality Database Version October 2017. Geneva, Switzerland: World Health Organization (WHO), 2017
Colombia Vital Registration - Deaths 2014 ICD10 as it appears in World Health Organization (WHO). WHO Mortality Database Version April 2018. Geneva, Switzerland: World Health Organization (WHO), 2018
Colombia Vital Registration - Deaths 2015 ICD10 as it appears in World Health Organization (WHO). WHO Mortality Database Version April 2018. Geneva, Switzerland: World Health Organization (WHO), 2018
Costa Rica Vital Registration - Deaths 1980 ICD9 as it appears in World Health Organization (WHO). WHO Mortality Database Version September 2016. Geneva, Switzerland: World Health Organization (WHO), 2016
Costa Rica Vital Registration - Deaths 1981 ICD9 as it appears in World Health Organization (WHO). WHO Mortality Database Version September 2016. Geneva, Switzerland: World Health Organization (WHO), 2016
Costa Rica Vital Registration - Deaths 1982 ICD9 as it appears in World Health Organization (WHO). WHO Mortality Database Version September 2016. Geneva, Switzerland: World Health Organization (WHO), 2016
Costa Rica Vital Registration - Deaths 1983 ICD9 as it appears in World Health Organization (WHO). WHO Mortality Database Version September 2016. Geneva, Switzerland: World Health Organization (WHO), 2016
Costa Rica Vital Registration - Deaths 1984 ICD9 as it appears in World Health Organization (WHO). WHO Mortality Database Version September 2016. Geneva, Switzerland: World Health Organization (WHO), 2016
Costa Rica Vital Registration - Deaths 1985 ICD9 as it appears in World Health Organization (WHO). WHO Mortality Database Version September 2016. Geneva, Switzerland: World Health Organization (WHO), 2016
Costa Rica Vital Registration - Deaths 1986 ICD9 as it appears in World Health Organization (WHO). WHO Mortality Database Version September 2016. Geneva, Switzerland: World Health Organization (WHO), 2016
Costa Rica Vital Registration - Deaths 1987 ICD9 as it appears in World Health Organization (WHO). WHO Mortality Database Version September 2016. Geneva, Switzerland: World Health Organization (WHO), 2016
Costa Rica Vital Registration - Deaths 1988 ICD9 as it appears in World Health Organization (WHO). WHO Mortality Database Version September 2016. Geneva, Switzerland: World Health Organization (WHO), 2016

Costa Rica Vital Registration - Deaths 1989 ICD9 as it appears in World Health Organization (WHO). WHO Mortality Database Version September 2016. Geneva, Switzerland: World Health Organization (WHO), 2016
Costa Rica Vital Registration - Deaths 1990 ICD9 as it appears in World Health Organization (WHO). WHO Mortality Database Version September 2016. Geneva, Switzerland: World Health Organization (WHO), 2016
Costa Rica Vital Registration - Deaths 1991 ICD9 as it appears in World Health Organization (WHO). WHO Mortality Database Version September 2016. Geneva, Switzerland: World Health Organization (WHO), 2016
Costa Rica Vital Registration - Deaths 1992 ICD9 as it appears in World Health Organization (WHO). WHO Mortality Database Version September 2016. Geneva, Switzerland: World Health Organization (WHO), 2016
Costa Rica Vital Registration - Deaths 1993 ICD9 as it appears in World Health Organization (WHO). WHO Mortality Database Version September 2016. Geneva, Switzerland: World Health Organization (WHO), 2016
Costa Rica Vital Registration - Deaths 1994 ICD9 as it appears in World Health Organization (WHO). WHO Mortality Database Version September 2016. Geneva, Switzerland: World Health Organization (WHO), 2016
Costa Rica Vital Registration - Deaths 1995 ICD9 as it appears in World Health Organization (WHO). WHO Mortality Database Version September 2016. Geneva, Switzerland: World Health Organization (WHO), 2016
Costa Rica Vital Registration - Deaths 1996 ICD9 as it appears in World Health Organization (WHO). WHO Mortality Database Version September 2016. Geneva, Switzerland: World Health Organization (WHO), 2016
Costa Rica Vital Registration - Deaths 1997 ICD10 as it appears in World Health Organization (WHO). WHO Mortality Database Version October 2017. Geneva, Switzerland: World Health Organization (WHO), 2017
Costa Rica Vital Registration - Deaths 1998 ICD10 as it appears in World Health Organization (WHO). WHO Mortality Database Version October 2017. Geneva, Switzerland: World Health Organization (WHO), 2017
Costa Rica Vital Registration - Deaths 1999 ICD10 as it appears in World Health Organization (WHO). WHO Mortality Database Version October 2017. Geneva, Switzerland: World Health Organization (WHO), 2017
Costa Rica Vital Registration - Deaths 2000 ICD10 as it appears in World Health Organization (WHO). WHO Mortality Database Version October 2017. Geneva, Switzerland: World Health Organization (WHO), 2017
Costa Rica Vital Registration - Deaths 2001 ICD10 as it appears in World Health Organization (WHO). WHO Mortality Database Version October 2017. Geneva, Switzerland: World Health Organization (WHO), 2017
Costa Rica Vital Registration - Deaths 2002 ICD10 as it appears in World Health Organization (WHO). WHO Mortality Database Version October 2017. Geneva, Switzerland: World Health Organization (WHO), 2017
Costa Rica Vital Registration - Deaths 2003 ICD10 as it appears in World Health Organization (WHO). WHO Mortality Database Version October 2017. Geneva, Switzerland: World Health Organization (WHO), 2017
Costa Rica Vital Registration - Deaths 2004 ICD10 as it appears in World Health Organization (WHO). WHO Mortality Database Version October 2017. Geneva, Switzerland: World Health Organization (WHO), 2017
Costa Rica Vital Registration - Deaths 2005 ICD10 as it appears in World Health Organization (WHO). WHO Mortality Database Version October 2017. Geneva, Switzerland: World Health Organization (WHO), 2017
Costa Rica Vital Registration - Deaths 2006 ICD10 as it appears in World Health Organization (WHO). WHO Mortality Database Version October 2017. Geneva, Switzerland: World Health Organization (WHO), 2017
Costa Rica Vital Registration - Deaths 2007 ICD10 as it appears in World Health Organization (WHO). WHO Mortality Database Version October 2017. Geneva, Switzerland: World Health Organization (WHO), 2017
Costa Rica Vital Registration - Deaths 2008 ICD10 as it appears in World Health Organization (WHO). WHO Mortality Database Version October 2017. Geneva, Switzerland: World Health Organization (WHO), 2017

Costa Rica Vital Registration - Deaths 2009 ICD10 as it appears in World Health Organization (WHO). WHO Mortality Database Version October 2017. Geneva, Switzerland: World Health Organization (WHO), 2017
Costa Rica Vital Registration - Deaths 2010 ICD10 as it appears in World Health Organization (WHO). WHO Mortality Database Version October 2017. Geneva, Switzerland: World Health Organization (WHO), 2017
Costa Rica Vital Registration - Deaths 2011 ICD10 as it appears in World Health Organization (WHO). WHO Mortality Database Version October 2017. Geneva, Switzerland: World Health Organization (WHO), 2017
Costa Rica Vital Registration - Deaths 2012 ICD10 as it appears in World Health Organization (WHO). WHO Mortality Database Version October 2017. Geneva, Switzerland: World Health Organization (WHO), 2017
Costa Rica Vital Registration - Deaths 2013 ICD10 as it appears in World Health Organization (WHO). WHO Mortality Database Version October 2017. Geneva, Switzerland: World Health Organization (WHO), 2017
Costa Rica Vital Registration - Deaths 2014 ICD10 as it appears in World Health Organization (WHO). WHO Mortality Database Version October 2017. Geneva, Switzerland: World Health Organization (WHO), 2017
Croatia Vital Registration - Deaths 1991 ICD9 as it appears in World Health Organization (WHO). WHO Mortality Database Version November 2015. Geneva, Switzerland: World Health Organization (WHO), 2015
Croatia Vital Registration - Deaths 1992 ICD9 as it appears in World Health Organization (WHO). WHO Mortality Database Version November 2015. Geneva, Switzerland: World Health Organization (WHO), 2015
Croatia Vital Registration - Deaths 1993 ICD9 as it appears in World Health Organization (WHO). WHO Mortality Database Version November 2015. Geneva, Switzerland: World Health Organization (WHO), 2015
Croatia Vital Registration - Deaths 1994 ICD9 as it appears in World Health Organization (WHO). WHO Mortality Database Version November 2015. Geneva, Switzerland: World Health Organization (WHO), 2015
Croatia Vital Registration - Deaths 1995 ICD10 as it appears in World Health Organization (WHO). WHO Mortality Database Version October 2017. Geneva, Switzerland: World Health Organization (WHO), 2017
Croatia Vital Registration - Deaths 1996 ICD10 as it appears in World Health Organization (WHO). WHO Mortality Database Version October 2017. Geneva, Switzerland: World Health Organization (WHO), 2017
Croatia Vital Registration - Deaths 1997 ICD10 as it appears in World Health Organization (WHO). WHO Mortality Database Version October 2017. Geneva, Switzerland: World Health Organization (WHO), 2017
Croatia Vital Registration - Deaths 1998 ICD10 as it appears in World Health Organization (WHO). WHO Mortality Database Version October 2017. Geneva, Switzerland: World Health Organization (WHO), 2017
Croatia Vital Registration - Deaths 1999 ICD10 as it appears in World Health Organization (WHO). WHO Mortality Database Version October 2017. Geneva, Switzerland: World Health Organization (WHO), 2017
Croatia Vital Registration - Deaths 2000 ICD10 as it appears in World Health Organization (WHO). WHO Mortality Database Version October 2017. Geneva, Switzerland: World Health Organization (WHO), 2017
Croatia Vital Registration - Deaths 2001 ICD10 as it appears in World Health Organization (WHO). WHO Mortality Database Version October 2017. Geneva, Switzerland: World Health Organization (WHO), 2017
Croatia Vital Registration - Deaths 2002 ICD10 as it appears in World Health Organization (WHO). WHO Mortality Database Version October 2017. Geneva, Switzerland: World Health Organization (WHO), 2017
Croatia Vital Registration - Deaths 2003 ICD10 as it appears in World Health Organization (WHO). WHO Mortality Database Version October 2017. Geneva, Switzerland: World Health Organization (WHO), 2017
Croatia Vital Registration - Deaths 2004 ICD10 as it appears in World Health Organization (WHO). WHO Mortality Database Version October 2017. Geneva, Switzerland: World Health Organization (WHO), 2017

Croatia Vital Registration - Deaths 2005 ICD10 as it appears in World Health Organization (WHO). WHO Mortality Database Version October 2017. Geneva, Switzerland: World Health Organization (WHO), 2017

Croatia Vital Registration - Deaths 2006 ICD10 as it appears in World Health Organization (WHO). WHO Mortality Database Version October 2017. Geneva, Switzerland: World Health Organization (WHO), 2017

Croatia Vital Registration - Deaths 2007 ICD10 as it appears in World Health Organization (WHO). WHO Mortality Database Version October 2017. Geneva, Switzerland: World Health Organization (WHO), 2017

Croatia Vital Registration - Deaths 2008 ICD10 as it appears in World Health Organization (WHO). WHO Mortality Database Version October 2017. Geneva, Switzerland: World Health Organization (WHO), 2017

Croatia Vital Registration - Deaths 2009 ICD10 as it appears in World Health Organization (WHO). WHO Mortality Database Version October 2017. Geneva, Switzerland: World Health Organization (WHO), 2017

Croatia Vital Registration - Deaths 2010 ICD10 as it appears in World Health Organization (WHO). WHO Mortality Database Version October 2017. Geneva, Switzerland: World Health Organization (WHO), 2017

Croatia Vital Registration - Deaths 2011 ICD10 as it appears in World Health Organization (WHO). WHO Mortality Database Version October 2017. Geneva, Switzerland: World Health Organization (WHO), 2017

Croatia Vital Registration - Deaths 2012 ICD10 as it appears in World Health Organization (WHO). WHO Mortality Database Version October 2017. Geneva, Switzerland: World Health Organization (WHO), 2017

Croatia Vital Registration - Deaths 2013 ICD10 as it appears in World Health Organization (WHO). WHO Mortality Database Version October 2017. Geneva, Switzerland: World Health Organization (WHO), 2017

Croatia Vital Registration - Deaths 2014 ICD10 as it appears in World Health Organization (WHO). WHO Mortality Database Version October 2017. Geneva, Switzerland: World Health Organization (WHO), 2017

Croatia Vital Registration - Deaths 2015 ICD10 as it appears in World Health Organization (WHO). WHO Mortality Database Version April 2018. Geneva, Switzerland: World Health Organization (WHO), 2018

Croatia Vital Registration - Deaths 2016 ICD10 as it appears in World Health Organization (WHO). WHO Mortality Database Version April 2018. Geneva, Switzerland: World Health Organization (WHO), 2018

Cuba Vital Registration - Deaths 1980 ICD9 as it appears in World Health Organization (WHO). WHO Mortality Database Version September 2016. Geneva, Switzerland: World Health Organization (WHO), 2016

Cuba Vital Registration - Deaths 1981 ICD9 as it appears in World Health Organization (WHO). WHO Mortality Database Version September 2016. Geneva, Switzerland: World Health Organization (WHO), 2016

Cuba Vital Registration - Deaths 1982 ICD9 as it appears in World Health Organization (WHO). WHO Mortality Database Version September 2016. Geneva, Switzerland: World Health Organization (WHO), 2016

Cuba Vital Registration - Deaths 1983 ICD9 as it appears in World Health Organization (WHO). WHO Mortality Database Version September 2016. Geneva, Switzerland: World Health Organization (WHO), 2016

Cuba Vital Registration - Deaths 1984 ICD9 as it appears in World Health Organization (WHO). WHO Mortality Database Version September 2016. Geneva, Switzerland: World Health Organization (WHO), 2016

Cuba Vital Registration - Deaths 1985 ICD9 as it appears in World Health Organization (WHO). WHO Mortality Database Version September 2016. Geneva, Switzerland: World Health Organization (WHO), 2016

Cuba Vital Registration - Deaths 1986 ICD9 as it appears in World Health Organization (WHO). WHO Mortality Database Version September 2016. Geneva, Switzerland: World Health Organization (WHO), 2016

Cuba Vital Registration - Deaths 1987 ICD9 as it appears in World Health Organization (WHO). WHO Mortality Database Version September 2016. Geneva, Switzerland: World Health Organization (WHO), 2016

Cuba Vital Registration - Deaths 1988 ICD9 as it appears in World Health Organization (WHO). WHO Mortality Database Version September 2016. Geneva, Switzerland:

World Health Organization (WHO), 2016
Cuba Vital Registration - Deaths 1989 ICD9 as it appears in World Health Organization (WHO). WHO Mortality Database Version September 2016. Geneva, Switzerland: World Health Organization (WHO), 2016
Cuba Vital Registration - Deaths 1990 ICD9 as it appears in World Health Organization (WHO). WHO Mortality Database Version September 2016. Geneva, Switzerland: World Health Organization (WHO), 2016
Cuba Vital Registration - Deaths 1991 ICD9 as it appears in World Health Organization (WHO). WHO Mortality Database Version September 2016. Geneva, Switzerland: World Health Organization (WHO), 2016
Cuba Vital Registration - Deaths 1992 ICD9 as it appears in World Health Organization (WHO). WHO Mortality Database Version September 2016. Geneva, Switzerland: World Health Organization (WHO), 2016
Cuba Vital Registration - Deaths 1993 ICD9 as it appears in World Health Organization (WHO). WHO Mortality Database Version September 2016. Geneva, Switzerland: World Health Organization (WHO), 2016
Cuba Vital Registration - Deaths 1994 ICD9 as it appears in World Health Organization (WHO). WHO Mortality Database Version September 2016. Geneva, Switzerland: World Health Organization (WHO), 2016
Cuba Vital Registration - Deaths 1995 ICD9 as it appears in World Health Organization (WHO). WHO Mortality Database Version September 2016. Geneva, Switzerland: World Health Organization (WHO), 2016
Cuba Vital Registration - Deaths 1996 ICD9 as it appears in World Health Organization (WHO). WHO Mortality Database Version September 2016. Geneva, Switzerland: World Health Organization (WHO), 2016
Cuba Vital Registration - Deaths 1997 ICD9 as it appears in World Health Organization (WHO). WHO Mortality Database Version November 2015. Geneva, Switzerland: World Health Organization (WHO), 2015
Cuba Vital Registration - Deaths 1998 ICD9 as it appears in World Health Organization (WHO). WHO Mortality Database Version November 2015. Geneva, Switzerland: World Health Organization (WHO), 2015
Cuba Vital Registration - Deaths 1999 ICD9 as it appears in World Health Organization (WHO). WHO Mortality Database Version November 2015. Geneva, Switzerland: World Health Organization (WHO), 2015
Cuba Vital Registration - Deaths 2000 ICD9 as it appears in World Health Organization (WHO). WHO Mortality Database Version November 2015. Geneva, Switzerland: World Health Organization (WHO), 2015
Cuba Vital Registration - Deaths 2001 ICD10 as it appears in World Health Organization (WHO). WHO Mortality Database Version October 2017. Geneva, Switzerland: World Health Organization (WHO), 2017
Cuba Vital Registration - Deaths 2002 ICD10 as it appears in World Health Organization (WHO). WHO Mortality Database Version October 2017. Geneva, Switzerland: World Health Organization (WHO), 2017
Cuba Vital Registration - Deaths 2003 ICD10 as it appears in World Health Organization (WHO). WHO Mortality Database Version October 2017. Geneva, Switzerland: World Health Organization (WHO), 2017
Cuba Vital Registration - Deaths 2004 ICD10 as it appears in World Health Organization (WHO). WHO Mortality Database Version October 2017. Geneva, Switzerland: World Health Organization (WHO), 2017
Cuba Vital Registration - Deaths 2005 ICD10 as it appears in World Health Organization (WHO). WHO Mortality Database Version October 2017. Geneva, Switzerland: World Health Organization (WHO), 2017
Cuba Vital Registration - Deaths 2006 ICD10 as it appears in World Health Organization (WHO). WHO Mortality Database Version October 2017. Geneva, Switzerland: World Health Organization (WHO), 2017
Cuba Vital Registration - Deaths 2007 ICD10 as it appears in World Health Organization (WHO). WHO Mortality Database Version October 2017. Geneva, Switzerland: World Health Organization (WHO), 2017
Cuba Vital Registration - Deaths 2008 ICD10 as it appears in World Health Organization (WHO). WHO Mortality Database Version October 2017. Geneva, Switzerland: World Health Organization (WHO), 2017

Cuba Vital Registration - Deaths 2009 ICD10 as it appears in World Health Organization (WHO). WHO Mortality Database Version October 2017. Geneva, Switzerland: World Health Organization (WHO), 2017
Cuba Vital Registration - Deaths 2010 ICD10 as it appears in World Health Organization (WHO). WHO Mortality Database Version October 2017. Geneva, Switzerland: World Health Organization (WHO), 2017
Cuba Vital Registration - Deaths 2011 ICD10 as it appears in World Health Organization (WHO). WHO Mortality Database Version October 2017. Geneva, Switzerland: World Health Organization (WHO), 2017
Cuba Vital Registration - Deaths 2012 ICD10 as it appears in World Health Organization (WHO). WHO Mortality Database Version October 2017. Geneva, Switzerland: World Health Organization (WHO), 2017
Cuba Vital Registration - Deaths 2013 ICD10 as it appears in World Health Organization (WHO). WHO Mortality Database Version October 2017. Geneva, Switzerland: World Health Organization (WHO), 2017
Cuba Vital Registration - Deaths 2014 ICD10 as it appears in World Health Organization (WHO). WHO Mortality Database Version October 2017. Geneva, Switzerland: World Health Organization (WHO), 2017
Cuba Vital Registration - Deaths 2015 ICD10 as it appears in World Health Organization (WHO). WHO Mortality Database Version October 2017. Geneva, Switzerland: World Health Organization (WHO), 2017
Cyprus Vital Registration - Deaths 2004 ICD10 as it appears in World Health Organization (WHO). WHO Mortality Database Version October 2017. Geneva, Switzerland: World Health Organization (WHO), 2017
Cyprus Vital Registration - Deaths 2005 ICD10 as it appears in World Health Organization (WHO). WHO Mortality Database Version October 2017. Geneva, Switzerland: World Health Organization (WHO), 2017
Cyprus Vital Registration - Deaths 2006 ICD10 as it appears in World Health Organization (WHO). WHO Mortality Database Version October 2017. Geneva, Switzerland: World Health Organization (WHO), 2017
Cyprus Vital Registration - Deaths 2007 ICD10 as it appears in World Health Organization (WHO). WHO Mortality Database Version October 2017. Geneva, Switzerland: World Health Organization (WHO), 2017
Cyprus Vital Registration - Deaths 2008 ICD10 as it appears in World Health Organization (WHO). WHO Mortality Database Version October 2017. Geneva, Switzerland: World Health Organization (WHO), 2017
Cyprus Vital Registration - Deaths 2009 ICD10 as it appears in World Health Organization (WHO). WHO Mortality Database Version October 2017. Geneva, Switzerland: World Health Organization (WHO), 2017
Cyprus Vital Registration - Deaths 2010 ICD10 as it appears in World Health Organization (WHO). WHO Mortality Database Version October 2017. Geneva, Switzerland: World Health Organization (WHO), 2017
Cyprus Vital Registration - Deaths 2011 ICD10 as it appears in World Health Organization (WHO). WHO Mortality Database Version October 2017. Geneva, Switzerland: World Health Organization (WHO), 2017
Cyprus Vital Registration - Deaths 2012 ICD10 as it appears in World Health Organization (WHO). WHO Mortality Database Version October 2017. Geneva, Switzerland: World Health Organization (WHO), 2017
Cyprus Vital Registration - Deaths 2013 ICD10 as it appears in World Health Organization (WHO). WHO Mortality Database Version October 2017. Geneva, Switzerland: World Health Organization (WHO), 2017
Cyprus Vital Registration - Deaths 2014 ICD10 as it appears in World Health Organization (WHO). WHO Mortality Database Version March 2017. Geneva, Switzerland: World Health Organization (WHO), 2017
Cyprus Vital Registration - Deaths 2015 ICD10 as it appears in World Health Organization (WHO). WHO Mortality Database Version October 2017. Geneva, Switzerland: World Health Organization (WHO), 2017
Cyprus Vital Registration - Deaths 2016 ICD10 as it appears in World Health Organization (WHO). WHO Mortality Database Version April 2018. Geneva, Switzerland: World Health Organization (WHO), 2018

Czech Republic Vital Registration - Deaths 1993 ICD9 as it appears in World Health Organization (WHO). WHO Mortality Database Version November 2015. Geneva, Switzerland: World Health Organization (WHO), 2015
Czech Republic Vital Registration - Deaths 1994 ICD10 as it appears in World Health Organization (WHO). WHO Mortality Database Version October 2017. Geneva, Switzerland: World Health Organization (WHO), 2017
Czech Republic Vital Registration - Deaths 1995 ICD10 as it appears in World Health Organization (WHO). WHO Mortality Database Version October 2017. Geneva, Switzerland: World Health Organization (WHO), 2017
Czech Republic Vital Registration - Deaths 1996 ICD10 as it appears in World Health Organization (WHO). WHO Mortality Database Version October 2017. Geneva, Switzerland: World Health Organization (WHO), 2017
Czech Republic Vital Registration - Deaths 1997 ICD10 as it appears in World Health Organization (WHO). WHO Mortality Database Version October 2017. Geneva, Switzerland: World Health Organization (WHO), 2017
Czech Republic Vital Registration - Deaths 1998 ICD10 as it appears in World Health Organization (WHO). WHO Mortality Database Version October 2017. Geneva, Switzerland: World Health Organization (WHO), 2017
Czech Republic Vital Registration - Deaths 1999 ICD10 as it appears in World Health Organization (WHO). WHO Mortality Database Version October 2017. Geneva, Switzerland: World Health Organization (WHO), 2017
Czech Republic Vital Registration - Deaths 2000 ICD10 as it appears in World Health Organization (WHO). WHO Mortality Database Version October 2017. Geneva, Switzerland: World Health Organization (WHO), 2017
Czech Republic Vital Registration - Deaths 2001 ICD10 as it appears in World Health Organization (WHO). WHO Mortality Database Version October 2017. Geneva, Switzerland: World Health Organization (WHO), 2017
Czech Republic Vital Registration - Deaths 2002 ICD10 as it appears in World Health Organization (WHO). WHO Mortality Database Version October 2017. Geneva, Switzerland: World Health Organization (WHO), 2017
Czech Republic Vital Registration - Deaths 2003 ICD10 as it appears in World Health Organization (WHO). WHO Mortality Database Version October 2017. Geneva, Switzerland: World Health Organization (WHO), 2017
Czech Republic Vital Registration - Deaths 2004 ICD10 as it appears in World Health Organization (WHO). WHO Mortality Database Version October 2017. Geneva, Switzerland: World Health Organization (WHO), 2017
Czech Republic Vital Registration - Deaths 2005 ICD10 as it appears in World Health Organization (WHO). WHO Mortality Database Version October 2017. Geneva, Switzerland: World Health Organization (WHO), 2017
Czech Republic Vital Registration - Deaths 2006 ICD10 as it appears in World Health Organization (WHO). WHO Mortality Database Version October 2017. Geneva, Switzerland: World Health Organization (WHO), 2017
Czech Republic Vital Registration - Deaths 2007 ICD10 as it appears in World Health Organization (WHO). WHO Mortality Database Version October 2017. Geneva, Switzerland: World Health Organization (WHO), 2017
Czech Republic Vital Registration - Deaths 2008 ICD10 as it appears in World Health Organization (WHO). WHO Mortality Database Version October 2017. Geneva, Switzerland: World Health Organization (WHO), 2017
Czech Republic Vital Registration - Deaths 2009 ICD10 as it appears in World Health Organization (WHO). WHO Mortality Database Version October 2017. Geneva, Switzerland: World Health Organization (WHO), 2017
Czech Republic Vital Registration - Deaths 2010 ICD10 as it appears in World Health Organization (WHO). WHO Mortality Database Version October 2017. Geneva, Switzerland: World Health Organization (WHO), 2017
Czech Republic Vital Registration - Deaths 2011 ICD10 as it appears in World Health Organization (WHO). WHO Mortality Database Version October 2017. Geneva, Switzerland: World Health Organization (WHO), 2017
Czech Republic Vital Registration - Deaths 2012 ICD10 as it appears in World Health Organization (WHO). WHO Mortality Database Version October 2017. Geneva, Switzerland: World Health Organization (WHO), 2017

Czech Republic Vital Registration - Deaths 2013 ICD10 as it appears in World Health Organization (WHO). WHO Mortality Database Version October 2017. Geneva, Switzerland: World Health Organization (WHO), 2017
Czech Republic Vital Registration - Deaths 2014 ICD10 as it appears in World Health Organization (WHO). WHO Mortality Database Version October 2017. Geneva, Switzerland: World Health Organization (WHO), 2017
Czech Republic Vital Registration - Deaths 2015 ICD10 as it appears in World Health Organization (WHO). WHO Mortality Database Version March 2017. Geneva, Switzerland: World Health Organization (WHO), 2017
Czech Republic Vital Registration - Deaths 2016 ICD10 as it appears in World Health Organization (WHO). WHO Mortality Database Version April 2018. Geneva, Switzerland: World Health Organization (WHO), 2018
Czechoslovakia - Czech Republic Vital Registration - Deaths 1986 ICD9 as it appears in World Health Organization (WHO). WHO Mortality Database Version November 2015. Geneva, Switzerland: World Health Organization (WHO), 2015
Czechoslovakia - Czech Republic Vital Registration - Deaths 1987 ICD9 as it appears in World Health Organization (WHO). WHO Mortality Database Version November 2015. Geneva, Switzerland: World Health Organization (WHO), 2015
Czechoslovakia - Czech Republic Vital Registration - Deaths 1988 ICD9 as it appears in World Health Organization (WHO). WHO Mortality Database Version November 2015. Geneva, Switzerland: World Health Organization (WHO), 2015
Czechoslovakia - Czech Republic Vital Registration - Deaths 1989 ICD9 as it appears in World Health Organization (WHO). WHO Mortality Database Version November 2015. Geneva, Switzerland: World Health Organization (WHO), 2015
Czechoslovakia - Czech Republic Vital Registration - Deaths 1990 ICD9 as it appears in World Health Organization (WHO). WHO Mortality Database Version November 2015. Geneva, Switzerland: World Health Organization (WHO), 2015
Czechoslovakia - Czech Republic Vital Registration - Deaths 1991 ICD9 as it appears in World Health Organization (WHO). WHO Mortality Database Version November 2015. Geneva, Switzerland: World Health Organization (WHO), 2015
Czechoslovakia - Czech Republic Vital Registration - Deaths 1992 ICD9 as it appears in World Health Organization (WHO). WHO Mortality Database Version November 2015. Geneva, Switzerland: World Health Organization (WHO), 2015
Denmark Vital Registration - Deaths 1980 ICD8 as it appears in World Health Organization (WHO). WHO Mortality Database Version July 2012. Geneva, Switzerland: World Health Organization (WHO), 2012
Denmark Vital Registration - Deaths 1981 ICD8 as it appears in World Health Organization (WHO). WHO Mortality Database Version July 2012. Geneva, Switzerland: World Health Organization (WHO), 2012
Denmark Vital Registration - Deaths 1982 ICD8 as it appears in World Health Organization (WHO). WHO Mortality Database Version July 2012. Geneva, Switzerland: World Health Organization (WHO), 2012
Denmark Vital Registration - Deaths 1983 ICD8 as it appears in World Health Organization (WHO). WHO Mortality Database Version July 2012. Geneva, Switzerland: World Health Organization (WHO), 2012
Denmark Vital Registration - Deaths 1984 ICD8 as it appears in World Health Organization (WHO). WHO Mortality Database Version July 2012. Geneva, Switzerland: World Health Organization (WHO), 2012
Denmark Vital Registration - Deaths 1985 ICD8 as it appears in World Health Organization (WHO). WHO Mortality Database Version July 2012. Geneva, Switzerland: World Health Organization (WHO), 2012
Denmark Vital Registration - Deaths 1986 ICD8 as it appears in World Health Organization (WHO). WHO Mortality Database Version July 2012. Geneva, Switzerland: World Health Organization (WHO), 2012
Denmark Vital Registration - Deaths 1987 ICD8 as it appears in World Health Organization (WHO). WHO Mortality Database Version July 2012. Geneva, Switzerland: World Health Organization (WHO), 2012
Denmark Vital Registration - Deaths 1988 ICD8 as it appears in World Health Organization (WHO). WHO Mortality Database Version July 2012. Geneva, Switzerland: World Health Organization (WHO), 2012
Denmark Vital Registration - Deaths 1989 ICD8 as it appears in World Health Organization (WHO). WHO Mortality Database Version July 2012. Geneva, Switzerland: World

Health Organization (WHO), 2012
Denmark Vital Registration - Deaths 1990 ICD8 as it appears in World Health Organization (WHO). WHO Mortality Database Version July 2012. Geneva, Switzerland: World Health Organization (WHO), 2012
Denmark Vital Registration - Deaths 1991 ICD8 as it appears in World Health Organization (WHO). WHO Mortality Database Version July 2012. Geneva, Switzerland: World Health Organization (WHO), 2012
Denmark Vital Registration - Deaths 1992 ICD8 as it appears in World Health Organization (WHO). WHO Mortality Database Version July 2012. Geneva, Switzerland: World Health Organization (WHO), 2012
Denmark Vital Registration - Deaths 1993 ICD8 as it appears in World Health Organization (WHO). WHO Mortality Database Version July 2012. Geneva, Switzerland: World Health Organization (WHO), 2012
Denmark Vital Registration - Deaths 1994 ICD10 as it appears in World Health Organization (WHO). WHO Mortality Database Version October 2017. Geneva, Switzerland: World Health Organization (WHO), 2017
Denmark Vital Registration - Deaths 1995 ICD10 as it appears in World Health Organization (WHO). WHO Mortality Database Version October 2017. Geneva, Switzerland: World Health Organization (WHO), 2017
Denmark Vital Registration - Deaths 1996 ICD10 as it appears in World Health Organization (WHO). WHO Mortality Database Version October 2017. Geneva, Switzerland: World Health Organization (WHO), 2017
Denmark Vital Registration - Deaths 1997 ICD10 as it appears in World Health Organization (WHO). WHO Mortality Database Version October 2017. Geneva, Switzerland: World Health Organization (WHO), 2017
Denmark Vital Registration - Deaths 1998 ICD10 as it appears in World Health Organization (WHO). WHO Mortality Database Version October 2017. Geneva, Switzerland: World Health Organization (WHO), 2017
Denmark Vital Registration - Deaths 1999 ICD10 as it appears in World Health Organization (WHO). WHO Mortality Database Version October 2017. Geneva, Switzerland: World Health Organization (WHO), 2017
Denmark Vital Registration - Deaths 2000 ICD10 as it appears in World Health Organization (WHO). WHO Mortality Database Version October 2017. Geneva, Switzerland: World Health Organization (WHO), 2017
Denmark Vital Registration - Deaths 2001 ICD10 as it appears in World Health Organization (WHO). WHO Mortality Database Version October 2017. Geneva, Switzerland: World Health Organization (WHO), 2017
Denmark Vital Registration - Deaths 2002 ICD10 as it appears in World Health Organization (WHO). WHO Mortality Database Version October 2017. Geneva, Switzerland: World Health Organization (WHO), 2017
Denmark Vital Registration - Deaths 2003 ICD10 as it appears in World Health Organization (WHO). WHO Mortality Database Version October 2017. Geneva, Switzerland: World Health Organization (WHO), 2017
Denmark Vital Registration - Deaths 2004 ICD10 as it appears in World Health Organization (WHO). WHO Mortality Database Version October 2017. Geneva, Switzerland: World Health Organization (WHO), 2017
Denmark Vital Registration - Deaths 2005 ICD10 as it appears in World Health Organization (WHO). WHO Mortality Database Version October 2017. Geneva, Switzerland: World Health Organization (WHO), 2017
Denmark Vital Registration - Deaths 2006 ICD10 as it appears in World Health Organization (WHO). WHO Mortality Database Version October 2017. Geneva, Switzerland: World Health Organization (WHO), 2017
Denmark Vital Registration - Deaths 2007 ICD10 as it appears in World Health Organization (WHO). WHO Mortality Database Version October 2017. Geneva, Switzerland: World Health Organization (WHO), 2017
Denmark Vital Registration - Deaths 2008 ICD10 as it appears in World Health Organization (WHO). WHO Mortality Database Version October 2017. Geneva, Switzerland: World Health Organization (WHO), 2017
Denmark Vital Registration - Deaths 2009 ICD10 as it appears in World Health Organization (WHO). WHO Mortality Database Version October 2017. Geneva, Switzerland: World Health Organization (WHO), 2017

Denmark Vital Registration - Deaths 2010 ICD10 as it appears in World Health Organization (WHO). WHO Mortality Database Version October 2017. Geneva, Switzerland: World Health Organization (WHO), 2017

Denmark Vital Registration - Deaths 2011 ICD10 as it appears in World Health Organization (WHO). WHO Mortality Database Version October 2017. Geneva, Switzerland: World Health Organization (WHO), 2017

Denmark Vital Registration - Deaths 2012 ICD10 as it appears in World Health Organization (WHO). WHO Mortality Database Version October 2017. Geneva, Switzerland: World Health Organization (WHO), 2017

Denmark Vital Registration - Deaths 2013 ICD10 as it appears in World Health Organization (WHO). WHO Mortality Database Version March 2017. Geneva, Switzerland: World Health Organization (WHO), 2017

Denmark Vital Registration - Deaths 2014 ICD10 as it appears in World Health Organization (WHO). WHO Mortality Database Version March 2017. Geneva, Switzerland: World Health Organization (WHO), 2017

Denmark Vital Registration - Deaths 2015 ICD10 as it appears in World Health Organization (WHO). WHO Mortality Database Version October 2017. Geneva, Switzerland: World Health Organization (WHO), 2017

Department of Health (Taiwan). Taiwan Vital Registration - Deaths 1980

Department of Health (Taiwan). Taiwan Vital Registration - Deaths 1981

Department of Health (Taiwan). Taiwan Vital Registration - Deaths 1982

Department of Health (Taiwan). Taiwan Vital Registration - Deaths 1983

Department of Health (Taiwan). Taiwan Vital Registration - Deaths 1984

Department of Health (Taiwan). Taiwan Vital Registration - Deaths 1985

Department of Health (Taiwan). Taiwan Vital Registration - Deaths 1986

Department of Health (Taiwan). Taiwan Vital Registration - Deaths 1987

Department of Health (Taiwan). Taiwan Vital Registration - Deaths 1988

Department of Health (Taiwan). Taiwan Vital Registration - Deaths 1989

Department of Health (Taiwan). Taiwan Vital Registration - Deaths 1990

Department of Health (Taiwan). Taiwan Vital Registration - Deaths 1991

Department of Health (Taiwan). Taiwan Vital Registration - Deaths 1992. Taipei City, Taiwan: Ministry of Health and Welfare (Taiwan)

Department of Health (Taiwan). Taiwan Vital Registration - Deaths 1993. Taipei City, Taiwan: Ministry of Health and Welfare (Taiwan)

Department of Health (Taiwan). Taiwan Vital Registration - Deaths 1994. Taipei City, Taiwan: Ministry of Health and Welfare (Taiwan)

Department of Health (Taiwan). Taiwan Vital Registration - Deaths 1995. Taipei City, Taiwan: Ministry of Health and Welfare (Taiwan)

Department of Health (Taiwan). Taiwan Vital Registration - Deaths 1996. Taipei City, Taiwan: Ministry of Health and Welfare (Taiwan)

Department of Health (Taiwan). Taiwan Vital Registration - Deaths 1997. Taipei City, Taiwan: Ministry of Health and Welfare (Taiwan)

Department of Health (Taiwan). Taiwan Vital Registration - Deaths 1998. Taipei City, Taiwan: Ministry of Health and Welfare (Taiwan)

Department of Health (Taiwan). Taiwan Vital Registration - Deaths 1999. Taipei City, Taiwan: Ministry of Health and Welfare (Taiwan)

Department of Health (Taiwan). Taiwan Vital Registration - Deaths 2000. Taipei City, Taiwan: Ministry of Health and Welfare (Taiwan)

Department of Health (Taiwan). Taiwan Vital Registration - Deaths 2001. Taipei City, Taiwan: Ministry of Health and Welfare (Taiwan)

Department of Health (Taiwan). Taiwan Vital Registration - Deaths 2002. Taipei City, Taiwan: Ministry of Health and Welfare (Taiwan)

Department of Health (Taiwan). Taiwan Vital Registration - Deaths 2003. Taipei City, Taiwan: Ministry of Health and Welfare (Taiwan)

Department of Health (Taiwan). Taiwan Vital Registration - Deaths 2004. Taipei City, Taiwan: Ministry of Health and Welfare (Taiwan)
Department of Health (Taiwan). Taiwan Vital Registration - Deaths 2005. Taipei City, Taiwan: Ministry of Health and Welfare (Taiwan)
Department of Health (Taiwan). Taiwan Vital Registration - Deaths 2006. Taipei City, Taiwan: Ministry of Health and Welfare (Taiwan)
Department of Health (Taiwan). Taiwan Vital Registration - Deaths 2007. Taipei City, Taiwan: Ministry of Health and Welfare (Taiwan)
Department of Health (Taiwan). Taiwan Vital Registration - Deaths 2008. Taipei City, Taiwan: Ministry of Health and Welfare (Taiwan)
Department of Health (Taiwan). Taiwan Vital Registration - Deaths 2009. Taipei City, Taiwan: Ministry of Health and Welfare (Taiwan)
Department of Health (Taiwan). Taiwan Vital Registration - Deaths 2010. Taipei City, Taiwan: Ministry of Health and Welfare (Taiwan)
Department of Health (Taiwan). Taiwan Vital Registration - Deaths 2011. Taipei City, Taiwan: Ministry of Health and Welfare (Taiwan)
Department of Health (Taiwan). Taiwan Vital Registration - Deaths 2012. Taipei City, Taiwan: Ministry of Health and Welfare (Taiwan)
Department of Home Affairs (South Africa), Statistics South Africa. South Africa Vital Registration - Causes of Death 1997-2005. Pretoria, South Africa: Statistics South Africa
Department of Home Affairs (South Africa), Statistics South Africa. South Africa Vital Registration - Causes of Death 2006. Pretoria, South Africa: Statistics South Africa
Department of Home Affairs (South Africa), Statistics South Africa. South Africa Vital Registration - Causes of Death 2007. Pretoria, South Africa: Statistics South Africa
Department of Home Affairs (South Africa), Statistics South Africa. South Africa Vital Registration - Causes of Death 2008. Pretoria, South Africa: Statistics South Africa
Department of Home Affairs (South Africa), Statistics South Africa. South Africa Vital Registration - Causes of Death 2009. Pretoria, South Africa: Statistics South Africa
Department of Home Affairs (South Africa), Statistics South Africa. South Africa Vital Registration - Causes of Death 2010. Pretoria, South Africa: Statistics South Africa
Department of Home Affairs (South Africa), Statistics South Africa. South Africa Vital Registration - Causes of Death 2011. Pretoria, South Africa: Statistics South Africa
Department of Home Affairs (South Africa), Statistics South Africa. South Africa Vital Registration - Causes of Death 2012. Pretoria, South Africa: Statistics South Africa
Department of Home Affairs (South Africa), Statistics South Africa. South Africa Vital Registration - Causes of Death 2013. Pretoria, South Africa: Statistics South Africa
Department of Home Affairs (South Africa), Statistics South Africa. South Africa Vital Registration - Causes of Death 2014. Pretoria, South Africa: Statistics South Africa
Department of Home Affairs (South Africa), Statistics South Africa. South Africa Vital Registration - Causes of Death 2015. Pretoria, South Africa: Statistics South Africa
Department of Statistics and Health Information, Ministry of Health (Chile), National Institute of Statistics (Chile). Chile Vital Statistics - Deaths 1990. Santiago, Chile: Department of Statistics and Health Information, Ministry of Health (Chile)
Department of Statistics and Health Information, Ministry of Health (Chile), National Institute of Statistics (Chile). Chile Vital Statistics - Deaths 1991. Santiago, Chile: Department of Statistics and Health Information, Ministry of Health (Chile)
Department of Statistics and Health Information, Ministry of Health (Chile), National Institute of Statistics (Chile). Chile Vital Statistics - Deaths 1992. Santiago, Chile: Department of Statistics and Health Information, Ministry of Health (Chile)
Department of Statistics and Health Information, Ministry of Health (Chile), National Institute of Statistics (Chile). Chile Vital Statistics - Deaths 1993. Santiago, Chile: Department of Statistics and Health Information, Ministry of Health (Chile)
Department of Statistics and Health Information, Ministry of Health (Chile), National Institute of Statistics (Chile). Chile Vital Statistics - Deaths 1994. Santiago, Chile: Department of Statistics and Health Information, Ministry of Health (Chile)
Department of Statistics and Health Information, Ministry of Health (Chile), National Institute of Statistics (Chile). Chile Vital Statistics - Deaths 1995. Santiago, Chile: Department of Statistics and Health Information, Ministry of Health (Chile)
Department of Statistics and Health Information, Ministry of Health (Chile), National Institute of Statistics (Chile). Chile Vital Statistics - Deaths 1996. Santiago, Chile: Department of Statistics and Health Information, Ministry of Health (Chile)
Directorate of Economics and Statistics and Office of Chief Registrar Births and Deaths (Delhi Territory, India). India - Delhi Medical Certification of Cause of Death Report 2013
Directorate of Economics and Statistics, Government of Karnataka, Office of the Chief Registrar of Births and Deaths, Government of Karnataka. India - Karnataka Medical

Certification of Cause of Death Report 2014. Bangalore, India: Directorate of Economics and Statistics, Government of Karnataka

Directorate of Economics and Statistics, Government of Karnataka, Office of the Chief Registrar of Births and Deaths, Government of Karnataka. India - Karnataka Medical Certification of Cause of Death Report 2015. Bangalore, India: Directorate of Economics and Statistics, Government of Karnataka

Directorate of Health (Iceland). Iceland Causes of Death Register 2017

Dominica Vital Registration - Deaths 1980 ICD9 as it appears in World Health Organization (WHO). WHO Mortality Database Version September 2016. Geneva, Switzerland: World Health Organization (WHO), 2016

Dominica Vital Registration - Deaths 1981 ICD9 as it appears in World Health Organization (WHO). WHO Mortality Database Version September 2016. Geneva, Switzerland: World Health Organization (WHO), 2016

Dominica Vital Registration - Deaths 1982 ICD9 as it appears in World Health Organization (WHO). WHO Mortality Database Version September 2016. Geneva, Switzerland: World Health Organization (WHO), 2016

Dominica Vital Registration - Deaths 1983 ICD9 as it appears in World Health Organization (WHO). WHO Mortality Database Version September 2016. Geneva, Switzerland: World Health Organization (WHO), 2016

Dominica Vital Registration - Deaths 1984 ICD9 as it appears in World Health Organization (WHO). WHO Mortality Database Version September 2016. Geneva, Switzerland: World Health Organization (WHO), 2016

Dominica Vital Registration - Deaths 1985 ICD9 as it appears in World Health Organization (WHO). WHO Mortality Database Version September 2016. Geneva, Switzerland: World Health Organization (WHO), 2016

Dominica Vital Registration - Deaths 1986 ICD9 as it appears in World Health Organization (WHO). WHO Mortality Database Version September 2016. Geneva, Switzerland: World Health Organization (WHO), 2016

Dominica Vital Registration - Deaths 1987 ICD9 as it appears in World Health Organization (WHO). WHO Mortality Database Version September 2016. Geneva, Switzerland: World Health Organization (WHO), 2016

Dominica Vital Registration - Deaths 1988 ICD9 as it appears in World Health Organization (WHO). WHO Mortality Database Version September 2016. Geneva, Switzerland: World Health Organization (WHO), 2016

Dominica Vital Registration - Deaths 1989 ICD9 as it appears in World Health Organization (WHO). WHO Mortality Database Version September 2016. Geneva, Switzerland: World Health Organization (WHO), 2016

Dominica Vital Registration - Deaths 1990 ICD9 as it appears in World Health Organization (WHO). WHO Mortality Database Version September 2016. Geneva, Switzerland: World Health Organization (WHO), 2016

Dominica Vital Registration - Deaths 1991 ICD9 as it appears in World Health Organization (WHO). WHO Mortality Database Version September 2016. Geneva, Switzerland: World Health Organization (WHO), 2016

Dominica Vital Registration - Deaths 1992 ICD9 as it appears in World Health Organization (WHO). WHO Mortality Database Version September 2016. Geneva, Switzerland: World Health Organization (WHO), 2016

Dominica Vital Registration - Deaths 1993 ICD9 as it appears in World Health Organization (WHO). WHO Mortality Database Version September 2016. Geneva, Switzerland: World Health Organization (WHO), 2016

Dominica Vital Registration - Deaths 1994 ICD9 as it appears in World Health Organization (WHO). WHO Mortality Database Version September 2016. Geneva, Switzerland: World Health Organization (WHO), 2016

Dominica Vital Registration - Deaths 1995 ICD9 as it appears in World Health Organization (WHO). WHO Mortality Database Version September 2016. Geneva, Switzerland: World Health Organization (WHO), 2016

Dominica Vital Registration - Deaths 1996 ICD9 as it appears in World Health Organization (WHO). WHO Mortality Database Version September 2016. Geneva, Switzerland: World Health Organization (WHO), 2016

Dominica Vital Registration - Deaths 1997 ICD9 as it appears in World Health Organization (WHO). WHO Mortality Database Version September 2016. Geneva, Switzerland: World Health Organization (WHO), 2016

Dominica Vital Registration - Deaths 1998 ICD9 as it appears in World Health Organization (WHO). WHO Mortality Database Version September 2016. Geneva, Switzerland: World Health Organization (WHO), 2016
Dominica Vital Registration - Deaths 1999 ICD9 as it appears in World Health Organization (WHO). WHO Mortality Database Version September 2016. Geneva, Switzerland: World Health Organization (WHO), 2016
Dominica Vital Registration - Deaths 2000 ICD9 as it appears in World Health Organization (WHO). WHO Mortality Database Version November 2015. Geneva, Switzerland: World Health Organization (WHO), 2015
Dominica Vital Registration - Deaths 2001 ICD10 as it appears in World Health Organization (WHO). WHO Mortality Database Version October 2017. Geneva, Switzerland: World Health Organization (WHO), 2017
Dominica Vital Registration - Deaths 2002 ICD10 as it appears in World Health Organization (WHO). WHO Mortality Database Version October 2017. Geneva, Switzerland: World Health Organization (WHO), 2017
Dominica Vital Registration - Deaths 2003 ICD10 as it appears in World Health Organization (WHO). WHO Mortality Database Version October 2017. Geneva, Switzerland: World Health Organization (WHO), 2017
Dominica Vital Registration - Deaths 2004 ICD10 as it appears in World Health Organization (WHO). WHO Mortality Database Version October 2017. Geneva, Switzerland: World Health Organization (WHO), 2017
Dominica Vital Registration - Deaths 2005 ICD10 as it appears in World Health Organization (WHO). WHO Mortality Database Version October 2017. Geneva, Switzerland: World Health Organization (WHO), 2017
Dominica Vital Registration - Deaths 2006 ICD10 as it appears in World Health Organization (WHO). WHO Mortality Database Version October 2017. Geneva, Switzerland: World Health Organization (WHO), 2017
Dominica Vital Registration - Deaths 2007 ICD10 as it appears in World Health Organization (WHO). WHO Mortality Database Version October 2017. Geneva, Switzerland: World Health Organization (WHO), 2017
Dominica Vital Registration - Deaths 2008 ICD10 as it appears in World Health Organization (WHO). WHO Mortality Database Version October 2017. Geneva, Switzerland: World Health Organization (WHO), 2017
Dominica Vital Registration - Deaths 2009 ICD10 as it appears in World Health Organization (WHO). WHO Mortality Database Version October 2017. Geneva, Switzerland: World Health Organization (WHO), 2017
Dominica Vital Registration - Deaths 2010 ICD10 as it appears in World Health Organization (WHO). WHO Mortality Database Version October 2017. Geneva, Switzerland: World Health Organization (WHO), 2017
Dominica Vital Registration - Deaths 2011 ICD10 as it appears in World Health Organization (WHO). WHO Mortality Database Version October 2017. Geneva, Switzerland: World Health Organization (WHO), 2017
Dominica Vital Registration - Deaths 2012 ICD10 as it appears in World Health Organization (WHO). WHO Mortality Database Version October 2017. Geneva, Switzerland: World Health Organization (WHO), 2017
Dominica Vital Registration - Deaths 2013 ICD10 as it appears in World Health Organization (WHO). WHO Mortality Database Version October 2017. Geneva, Switzerland: World Health Organization (WHO), 2017
Dominica Vital Registration - Deaths 2014 ICD10 as it appears in World Health Organization (WHO). WHO Mortality Database Version October 2017. Geneva, Switzerland: World Health Organization (WHO), 2017
Dominica Vital Registration - Deaths 2015 ICD10 as it appears in World Health Organization (WHO). WHO Mortality Database Version October 2017. Geneva, Switzerland: World Health Organization (WHO), 2017
Dominican Republic Vital Registration - Deaths 1980 ICD9 as it appears in World Health Organization (WHO). WHO Mortality Database Version September 2016. Geneva, Switzerland: World Health Organization (WHO), 2016
Dominican Republic Vital Registration - Deaths 1981 ICD9 as it appears in World Health Organization (WHO). WHO Mortality Database Version September 2016. Geneva, Switzerland: World Health Organization (WHO), 2016

Dominican Republic Vital Registration - Deaths 1982 ICD9 as it appears in World Health Organization (WHO). WHO Mortality Database Version September 2016. Geneva, Switzerland: World Health Organization (WHO), 2016
Dominican Republic Vital Registration - Deaths 1983 ICD9 as it appears in World Health Organization (WHO). WHO Mortality Database Version September 2016. Geneva, Switzerland: World Health Organization (WHO), 2016
Dominican Republic Vital Registration - Deaths 1984 ICD9 as it appears in World Health Organization (WHO). WHO Mortality Database Version September 2016. Geneva, Switzerland: World Health Organization (WHO), 2016
Dominican Republic Vital Registration - Deaths 1985 ICD9 as it appears in World Health Organization (WHO). WHO Mortality Database Version September 2016. Geneva, Switzerland: World Health Organization (WHO), 2016
Dominican Republic Vital Registration - Deaths 1986 ICD9 as it appears in World Health Organization (WHO). WHO Mortality Database Version September 2016. Geneva, Switzerland: World Health Organization (WHO), 2016
Dominican Republic Vital Registration - Deaths 1987 ICD9 as it appears in World Health Organization (WHO). WHO Mortality Database Version September 2016. Geneva, Switzerland: World Health Organization (WHO), 2016
Dominican Republic Vital Registration - Deaths 1988 ICD9 as it appears in World Health Organization (WHO). WHO Mortality Database Version September 2016. Geneva, Switzerland: World Health Organization (WHO), 2016
Dominican Republic Vital Registration - Deaths 1989 ICD9 as it appears in World Health Organization (WHO). WHO Mortality Database Version September 2016. Geneva, Switzerland: World Health Organization (WHO), 2016
Dominican Republic Vital Registration - Deaths 1990 ICD9 as it appears in World Health Organization (WHO). WHO Mortality Database Version November 2015. Geneva, Switzerland: World Health Organization (WHO), 2015
Dominican Republic Vital Registration - Deaths 1991 ICD9 as it appears in World Health Organization (WHO). WHO Mortality Database Version September 2016. Geneva, Switzerland: World Health Organization (WHO), 2016
Dominican Republic Vital Registration - Deaths 1992 ICD9 as it appears in World Health Organization (WHO). WHO Mortality Database Version September 2016. Geneva, Switzerland: World Health Organization (WHO), 2016
Dominican Republic Vital Registration - Deaths 1994 ICD9 as it appears in World Health Organization (WHO). WHO Mortality Database Version September 2016. Geneva, Switzerland: World Health Organization (WHO), 2016
Dominican Republic Vital Registration - Deaths 1995 ICD9 as it appears in World Health Organization (WHO). WHO Mortality Database Version September 2016. Geneva, Switzerland: World Health Organization (WHO), 2016
Dominican Republic Vital Registration - Deaths 1996 ICD10 as it appears in World Health Organization (WHO). WHO Mortality Database Version October 2017. Geneva, Switzerland: World Health Organization (WHO), 2017
Dominican Republic Vital Registration - Deaths 1997 ICD10 as it appears in World Health Organization (WHO). WHO Mortality Database Version October 2017. Geneva, Switzerland: World Health Organization (WHO), 2017
Dominican Republic Vital Registration - Deaths 1998 ICD10 as it appears in World Health Organization (WHO). WHO Mortality Database Version October 2017. Geneva, Switzerland: World Health Organization (WHO), 2017
Dominican Republic Vital Registration - Deaths 1999 ICD10 as it appears in World Health Organization (WHO). WHO Mortality Database Version October 2017. Geneva, Switzerland: World Health Organization (WHO), 2017
Dominican Republic Vital Registration - Deaths 2000 ICD10 as it appears in World Health Organization (WHO). WHO Mortality Database Version October 2017. Geneva, Switzerland: World Health Organization (WHO), 2017
Dominican Republic Vital Registration - Deaths 2001 ICD10 as it appears in World Health Organization (WHO). WHO Mortality Database Version October 2017. Geneva, Switzerland: World Health Organization (WHO), 2017
Dominican Republic Vital Registration - Deaths 2003 ICD10 as it appears in World Health Organization (WHO). WHO Mortality Database Version October 2017. Geneva, Switzerland: World Health Organization (WHO), 2017

Dominican Republic Vital Registration - Deaths 2004 ICD10 as it appears in World Health Organization (WHO). WHO Mortality Database Version October 2017. Geneva, Switzerland: World Health Organization (WHO), 2017
Dominican Republic Vital Registration - Deaths 2005 ICD10 as it appears in World Health Organization (WHO). WHO Mortality Database Version October 2017. Geneva, Switzerland: World Health Organization (WHO), 2017
Dominican Republic Vital Registration - Deaths 2006 ICD10 as it appears in World Health Organization (WHO). WHO Mortality Database Version October 2017. Geneva, Switzerland: World Health Organization (WHO), 2017
Dominican Republic Vital Registration - Deaths 2007 ICD10 as it appears in World Health Organization (WHO). WHO Mortality Database Version October 2017. Geneva, Switzerland: World Health Organization (WHO), 2017
Dominican Republic Vital Registration - Deaths 2008 ICD10 as it appears in World Health Organization (WHO). WHO Mortality Database Version October 2017. Geneva, Switzerland: World Health Organization (WHO), 2017
Dominican Republic Vital Registration - Deaths 2009 ICD10 as it appears in World Health Organization (WHO). WHO Mortality Database Version October 2017. Geneva, Switzerland: World Health Organization (WHO), 2017
Dominican Republic Vital Registration - Deaths 2010 ICD10 as it appears in World Health Organization (WHO). WHO Mortality Database Version October 2017. Geneva, Switzerland: World Health Organization (WHO), 2017
Dominican Republic Vital Registration - Deaths 2011 ICD10 as it appears in World Health Organization (WHO). WHO Mortality Database Version October 2017. Geneva, Switzerland: World Health Organization (WHO), 2017
Dominican Republic Vital Registration - Deaths 2012 ICD10 as it appears in World Health Organization (WHO). WHO Mortality Database Version October 2017. Geneva, Switzerland: World Health Organization (WHO), 2017
Dominican Republic Vital Registration - Deaths 2013 ICD10 as it appears in World Health Organization (WHO). WHO Mortality Database Version October 2017. Geneva, Switzerland: World Health Organization (WHO), 2017
Ecuador Vital Registration - Deaths 1980 ICD9 as it appears in World Health Organization (WHO). WHO Mortality Database Version September 2016. Geneva, Switzerland: World Health Organization (WHO), 2016
Ecuador Vital Registration - Deaths 1981 ICD9 as it appears in World Health Organization (WHO). WHO Mortality Database Version September 2016. Geneva, Switzerland: World Health Organization (WHO), 2016
Ecuador Vital Registration - Deaths 1982 ICD9 as it appears in World Health Organization (WHO). WHO Mortality Database Version September 2016. Geneva, Switzerland: World Health Organization (WHO), 2016
Ecuador Vital Registration - Deaths 1983 ICD9 as it appears in World Health Organization (WHO). WHO Mortality Database Version September 2016. Geneva, Switzerland: World Health Organization (WHO), 2016
Ecuador Vital Registration - Deaths 1984 ICD9 as it appears in World Health Organization (WHO). WHO Mortality Database Version September 2016. Geneva, Switzerland: World Health Organization (WHO), 2016
Ecuador Vital Registration - Deaths 1985 ICD9 as it appears in World Health Organization (WHO). WHO Mortality Database Version September 2016. Geneva, Switzerland: World Health Organization (WHO), 2016
Ecuador Vital Registration - Deaths 1986 ICD9 as it appears in World Health Organization (WHO). WHO Mortality Database Version September 2016. Geneva, Switzerland: World Health Organization (WHO), 2016
Ecuador Vital Registration - Deaths 1988 ICD9 as it appears in World Health Organization (WHO). WHO Mortality Database Version September 2016. Geneva, Switzerland: World Health Organization (WHO), 2016
Ecuador Vital Registration - Deaths 1989 ICD9 as it appears in World Health Organization (WHO). WHO Mortality Database Version September 2016. Geneva, Switzerland: World Health Organization (WHO), 2016
Ecuador Vital Registration - Deaths 1990 ICD9 as it appears in World Health Organization (WHO). WHO Mortality Database Version September 2016. Geneva, Switzerland: World Health Organization (WHO), 2016

Ecuador Vital Registration - Deaths 1991 ICD9 as it appears in World Health Organization (WHO). WHO Mortality Database Version September 2016. Geneva, Switzerland: World Health Organization (WHO), 2016
Ecuador Vital Registration - Deaths 1992 ICD9 as it appears in World Health Organization (WHO). WHO Mortality Database Version September 2016. Geneva, Switzerland: World Health Organization (WHO), 2016
Ecuador Vital Registration - Deaths 1993 ICD9 as it appears in World Health Organization (WHO). WHO Mortality Database Version September 2016. Geneva, Switzerland: World Health Organization (WHO), 2016
Ecuador Vital Registration - Deaths 1994 ICD9 as it appears in World Health Organization (WHO). WHO Mortality Database Version September 2016. Geneva, Switzerland: World Health Organization (WHO), 2016
Ecuador Vital Registration - Deaths 1995 ICD9 as it appears in World Health Organization (WHO). WHO Mortality Database Version September 2016. Geneva, Switzerland: World Health Organization (WHO), 2016
Ecuador Vital Registration - Deaths 1996 ICD9 as it appears in World Health Organization (WHO). WHO Mortality Database Version November 2015. Geneva, Switzerland: World Health Organization (WHO), 2015
Ecuador Vital Registration - Deaths 1997 ICD10 as it appears in World Health Organization (WHO). WHO Mortality Database Version October 2017. Geneva, Switzerland: World Health Organization (WHO), 2017
Ecuador Vital Registration - Deaths 1998 ICD10 as it appears in World Health Organization (WHO). WHO Mortality Database Version October 2017. Geneva, Switzerland: World Health Organization (WHO), 2017
Ecuador Vital Registration - Deaths 1999 ICD10 as it appears in World Health Organization (WHO). WHO Mortality Database Version October 2017. Geneva, Switzerland: World Health Organization (WHO), 2017
Ecuador Vital Registration - Deaths 2000 ICD10 as it appears in World Health Organization (WHO). WHO Mortality Database Version October 2017. Geneva, Switzerland: World Health Organization (WHO), 2017
Ecuador Vital Registration - Deaths 2001 ICD10 as it appears in World Health Organization (WHO). WHO Mortality Database Version October 2017. Geneva, Switzerland: World Health Organization (WHO), 2017
Ecuador Vital Registration - Deaths 2002 ICD10 as it appears in World Health Organization (WHO). WHO Mortality Database Version October 2017. Geneva, Switzerland: World Health Organization (WHO), 2017
Ecuador Vital Registration - Deaths 2003 ICD10 as it appears in World Health Organization (WHO). WHO Mortality Database Version October 2017. Geneva, Switzerland: World Health Organization (WHO), 2017
Ecuador Vital Registration - Deaths 2004 ICD10 as it appears in World Health Organization (WHO). WHO Mortality Database Version October 2017. Geneva, Switzerland: World Health Organization (WHO), 2017
Ecuador Vital Registration - Deaths 2005 ICD10 as it appears in World Health Organization (WHO). WHO Mortality Database Version October 2017. Geneva, Switzerland: World Health Organization (WHO), 2017
Ecuador Vital Registration - Deaths 2006 ICD10 as it appears in World Health Organization (WHO). WHO Mortality Database Version October 2017. Geneva, Switzerland: World Health Organization (WHO), 2017
Ecuador Vital Registration - Deaths 1987 ICD9 as it appears in World Health Organization (WHO). WHO Mortality Database Version September 2016. Geneva, Switzerland: World Health Organization (WHO), 2016
Ecuador Vital Registration - Deaths 2007 ICD10 as it appears in World Health Organization (WHO). WHO Mortality Database Version October 2017. Geneva, Switzerland: World Health Organization (WHO), 2017
Ecuador Vital Registration - Deaths 2008 ICD10 as it appears in World Health Organization (WHO). WHO Mortality Database Version October 2017. Geneva, Switzerland: World Health Organization (WHO), 2017
Ecuador Vital Registration - Deaths 2009 ICD10 as it appears in World Health Organization (WHO). WHO Mortality Database Version October 2017. Geneva, Switzerland: World Health Organization (WHO), 2017

Ecuador Vital Registration - Deaths 2010 ICD10 as it appears in World Health Organization (WHO). WHO Mortality Database Version October 2017. Geneva, Switzerland: World Health Organization (WHO), 2017

Ecuador Vital Registration - Deaths 2011 ICD10 as it appears in World Health Organization (WHO). WHO Mortality Database Version October 2017. Geneva, Switzerland: World Health Organization (WHO), 2017

Ecuador Vital Registration - Deaths 2012 ICD10 as it appears in World Health Organization (WHO). WHO Mortality Database Version October 2017. Geneva, Switzerland: World Health Organization (WHO), 2017

Ecuador Vital Registration - Deaths 2013 ICD10 as it appears in World Health Organization (WHO). WHO Mortality Database Version October 2017. Geneva, Switzerland: World Health Organization (WHO), 2017

Ecuador Vital Registration - Deaths 2014 ICD10 as it appears in World Health Organization (WHO). WHO Mortality Database Version October 2017. Geneva, Switzerland: World Health Organization (WHO), 2017

Ecuador Vital Registration - Deaths 2015 ICD10 as it appears in World Health Organization (WHO). WHO Mortality Database Version October 2017. Geneva, Switzerland: World Health Organization (WHO), 2017

Egypt Vital Registration - Deaths 1991 ICD9 as it appears in World Health Organization (WHO). WHO Mortality Database Version September 2016. Geneva, Switzerland: World Health Organization (WHO), 2016

El Salvador Vital Registration - Deaths 1981 ICD9 as it appears in World Health Organization (WHO). WHO Mortality Database Version September 2016. Geneva, Switzerland: World Health Organization (WHO), 2016

El Salvador Vital Registration - Deaths 1982 ICD9 as it appears in World Health Organization (WHO). WHO Mortality Database Version September 2016. Geneva, Switzerland: World Health Organization (WHO), 2016

El Salvador Vital Registration - Deaths 1983 ICD9 as it appears in World Health Organization (WHO). WHO Mortality Database Version September 2016. Geneva, Switzerland: World Health Organization (WHO), 2016

El Salvador Vital Registration - Deaths 1984 ICD9 as it appears in World Health Organization (WHO). WHO Mortality Database Version September 2016. Geneva, Switzerland: World Health Organization (WHO), 2016

El Salvador Vital Registration - Deaths 1990 ICD9 as it appears in World Health Organization (WHO). WHO Mortality Database Version September 2016. Geneva, Switzerland: World Health Organization (WHO), 2016

El Salvador Vital Registration - Deaths 1991 ICD9 as it appears in World Health Organization (WHO). WHO Mortality Database Version September 2016. Geneva, Switzerland: World Health Organization (WHO), 2016

El Salvador Vital Registration - Deaths 1992 ICD9 as it appears in World Health Organization (WHO). WHO Mortality Database Version September 2016. Geneva, Switzerland: World Health Organization (WHO), 2016

El Salvador Vital Registration - Deaths 1993 ICD9 as it appears in World Health Organization (WHO). WHO Mortality Database Version September 2016. Geneva, Switzerland: World Health Organization (WHO), 2016

El Salvador Vital Registration - Deaths 1995 ICD9 as it appears in World Health Organization (WHO). WHO Mortality Database Version September 2016. Geneva, Switzerland: World Health Organization (WHO), 2016

El Salvador Vital Registration - Deaths 1996 ICD9 as it appears in World Health Organization (WHO). WHO Mortality Database Version September 2016. Geneva, Switzerland: World Health Organization (WHO), 2016

El Salvador Vital Registration - Deaths 1997 ICD10 as it appears in World Health Organization (WHO). WHO Mortality Database Version October 2017. Geneva, Switzerland: World Health Organization (WHO), 2017

El Salvador Vital Registration - Deaths 1998 ICD10 as it appears in World Health Organization (WHO). WHO Mortality Database Version October 2017. Geneva, Switzerland: World Health Organization (WHO), 2017

El Salvador Vital Registration - Deaths 1999 ICD10 as it appears in World Health Organization (WHO). WHO Mortality Database Version October 2017. Geneva, Switzerland: World Health Organization (WHO), 2017

El Salvador Vital Registration - Deaths 2000 ICD10 as it appears in World Health Organization (WHO). WHO Mortality Database Version October 2017. Geneva, Switzerland: World Health Organization (WHO), 2017

El Salvador Vital Registration - Deaths 2001 ICD10 as it appears in World Health Organization (WHO). WHO Mortality Database Version October 2017. Geneva, Switzerland: World Health Organization (WHO), 2017

El Salvador Vital Registration - Deaths 2002 ICD10 as it appears in World Health Organization (WHO). WHO Mortality Database Version October 2017. Geneva, Switzerland: World Health Organization (WHO), 2017

El Salvador Vital Registration - Deaths 2003 ICD10 as it appears in World Health Organization (WHO). WHO Mortality Database Version October 2017. Geneva, Switzerland: World Health Organization (WHO), 2017

El Salvador Vital Registration - Deaths 2004 ICD10 as it appears in World Health Organization (WHO). WHO Mortality Database Version October 2017. Geneva, Switzerland: World Health Organization (WHO), 2017

El Salvador Vital Registration - Deaths 2005 ICD10 as it appears in World Health Organization (WHO). WHO Mortality Database Version October 2017. Geneva, Switzerland: World Health Organization (WHO), 2017

El Salvador Vital Registration - Deaths 2006 ICD10 as it appears in World Health Organization (WHO). WHO Mortality Database Version October 2017. Geneva, Switzerland: World Health Organization (WHO), 2017

El Salvador Vital Registration - Deaths 2007 ICD10 as it appears in World Health Organization (WHO). WHO Mortality Database Version October 2017. Geneva, Switzerland: World Health Organization (WHO), 2017

El Salvador Vital Registration - Deaths 2008 ICD10 as it appears in World Health Organization (WHO). WHO Mortality Database Version October 2017. Geneva, Switzerland: World Health Organization (WHO), 2017

El Salvador Vital Registration - Deaths 2009 ICD10 as it appears in World Health Organization (WHO). WHO Mortality Database Version October 2017. Geneva, Switzerland: World Health Organization (WHO), 2017

El Salvador Vital Registration - Deaths 2010 ICD10 as it appears in World Health Organization (WHO). WHO Mortality Database Version October 2017. Geneva, Switzerland: World Health Organization (WHO), 2017

El Salvador Vital Registration - Deaths 2011 ICD10 as it appears in World Health Organization (WHO). WHO Mortality Database Version October 2017. Geneva, Switzerland: World Health Organization (WHO), 2017

El Salvador Vital Registration - Deaths 2012 ICD10 as it appears in World Health Organization (WHO). WHO Mortality Database Version October 2017. Geneva, Switzerland: World Health Organization (WHO), 2017

El Salvador Vital Registration - Deaths 2013 ICD10 as it appears in World Health Organization (WHO). WHO Mortality Database Version October 2017. Geneva, Switzerland: World Health Organization (WHO), 2017

El Salvador Vital Registration - Deaths 2014 ICD10 as it appears in World Health Organization (WHO). WHO Mortality Database Version October 2017. Geneva, Switzerland: World Health Organization (WHO), 2017

Estonia Vital Registration - Deaths 1981 ICD9 as it appears in World Health Organization (WHO). WHO Mortality Database Version September 2016. Geneva, Switzerland: World Health Organization (WHO), 2016

Estonia Vital Registration - Deaths 1982 ICD9 as it appears in World Health Organization (WHO). WHO Mortality Database Version September 2016. Geneva, Switzerland: World Health Organization (WHO), 2016

Estonia Vital Registration - Deaths 1985 ICD9 as it appears in World Health Organization (WHO). WHO Mortality Database Version September 2016. Geneva, Switzerland: World Health Organization (WHO), 2016

Estonia Vital Registration - Deaths 1986 ICD9 as it appears in World Health Organization (WHO). WHO Mortality Database Version September 2016. Geneva, Switzerland: World Health Organization (WHO), 2016

Estonia Vital Registration - Deaths 1987 ICD9 as it appears in World Health Organization (WHO). WHO Mortality Database Version September 2016. Geneva, Switzerland: World Health Organization (WHO), 2016

Estonia Vital Registration - Deaths 1988 ICD9 as it appears in World Health Organization (WHO). WHO Mortality Database Version September 2016. Geneva, Switzerland: World Health Organization (WHO), 2016
Estonia Vital Registration - Deaths 1989 ICD9 as it appears in World Health Organization (WHO). WHO Mortality Database Version September 2016. Geneva, Switzerland: World Health Organization (WHO), 2016
Estonia Vital Registration - Deaths 1990 ICD9 as it appears in World Health Organization (WHO). WHO Mortality Database Version September 2016. Geneva, Switzerland: World Health Organization (WHO), 2016
Estonia Vital Registration - Deaths 1991 ICD9 as it appears in World Health Organization (WHO). WHO Mortality Database Version September 2016. Geneva, Switzerland: World Health Organization (WHO), 2016
Estonia Vital Registration - Deaths 1992 ICD9 as it appears in World Health Organization (WHO). WHO Mortality Database Version September 2016. Geneva, Switzerland: World Health Organization (WHO), 2016
Estonia Vital Registration - Deaths 1993 ICD9 as it appears in World Health Organization (WHO). WHO Mortality Database Version September 2016. Geneva, Switzerland: World Health Organization (WHO), 2016
Estonia Vital Registration - Deaths 1994 ICD9 as it appears in World Health Organization (WHO). WHO Mortality Database Version November 2015. Geneva, Switzerland: World Health Organization (WHO), 2015
Estonia Vital Registration - Deaths 1995 ICD9 as it appears in World Health Organization (WHO). WHO Mortality Database Version November 2015. Geneva, Switzerland: World Health Organization (WHO), 2015
Estonia Vital Registration - Deaths 1996 ICD9 as it appears in World Health Organization (WHO). WHO Mortality Database Version November 2015. Geneva, Switzerland: World Health Organization (WHO), 2015
Estonia Vital Registration - Deaths 1997 ICD10 as it appears in World Health Organization (WHO). WHO Mortality Database Version October 2017. Geneva, Switzerland: World Health Organization (WHO), 2017
Estonia Vital Registration - Deaths 1998 ICD10 as it appears in World Health Organization (WHO). WHO Mortality Database Version October 2017. Geneva, Switzerland: World Health Organization (WHO), 2017
Estonia Vital Registration - Deaths 1999 ICD10 as it appears in World Health Organization (WHO). WHO Mortality Database Version October 2017. Geneva, Switzerland: World Health Organization (WHO), 2017
Estonia Vital Registration - Deaths 2000 ICD10 as it appears in World Health Organization (WHO). WHO Mortality Database Version October 2017. Geneva, Switzerland: World Health Organization (WHO), 2017
Estonia Vital Registration - Deaths 2001 ICD10 as it appears in World Health Organization (WHO). WHO Mortality Database Version October 2017. Geneva, Switzerland: World Health Organization (WHO), 2017
Estonia Vital Registration - Deaths 2002 ICD10 as it appears in World Health Organization (WHO). WHO Mortality Database Version October 2017. Geneva, Switzerland: World Health Organization (WHO), 2017
Estonia Vital Registration - Deaths 2003 ICD10 as it appears in World Health Organization (WHO). WHO Mortality Database Version October 2017. Geneva, Switzerland: World Health Organization (WHO), 2017
Estonia Vital Registration - Deaths 2004 ICD10 as it appears in World Health Organization (WHO). WHO Mortality Database Version October 2017. Geneva, Switzerland: World Health Organization (WHO), 2017
Estonia Vital Registration - Deaths 2005 ICD10 as it appears in World Health Organization (WHO). WHO Mortality Database Version October 2017. Geneva, Switzerland: World Health Organization (WHO), 2017
Estonia Vital Registration - Deaths 2006 ICD10 as it appears in World Health Organization (WHO). WHO Mortality Database Version October 2017. Geneva, Switzerland: World Health Organization (WHO), 2017
Estonia Vital Registration - Deaths 2007 ICD10 as it appears in World Health Organization (WHO). WHO Mortality Database Version October 2017. Geneva, Switzerland: World Health Organization (WHO), 2017

Estonia Vital Registration - Deaths 2008 ICD10 as it appears in World Health Organization (WHO). WHO Mortality Database Version October 2017. Geneva, Switzerland: World Health Organization (WHO), 2017
Estonia Vital Registration - Deaths 2009 ICD10 as it appears in World Health Organization (WHO). WHO Mortality Database Version October 2017. Geneva, Switzerland: World Health Organization (WHO), 2017
Estonia Vital Registration - Deaths 2010 ICD10 as it appears in World Health Organization (WHO). WHO Mortality Database Version October 2017. Geneva, Switzerland: World Health Organization (WHO), 2017
Estonia Vital Registration - Deaths 2011 ICD10 as it appears in World Health Organization (WHO). WHO Mortality Database Version October 2017. Geneva, Switzerland: World Health Organization (WHO), 2017
Estonia Vital Registration - Deaths 2012 ICD10 as it appears in World Health Organization (WHO). WHO Mortality Database Version October 2017. Geneva, Switzerland: World Health Organization (WHO), 2017
Estonia Vital Registration - Deaths 2013 ICD10 as it appears in World Health Organization (WHO). WHO Mortality Database Version October 2017. Geneva, Switzerland: World Health Organization (WHO), 2017
Estonia Vital Registration - Deaths 2014 ICD10 as it appears in World Health Organization (WHO). WHO Mortality Database Version October 2017. Geneva, Switzerland: World Health Organization (WHO), 2017
Estonia Vital Registration - Deaths 2015 ICD10 as it appears in World Health Organization (WHO). WHO Mortality Database Version October 2017. Geneva, Switzerland: World Health Organization (WHO), 2017
Fiji Vital Registration - Deaths 1999 ICD9 as it appears in World Health Organization (WHO). WHO Mortality Database Version September 2016. Geneva, Switzerland: World Health Organization (WHO), 2016
Fiji Vital Registration - Deaths 2002 ICD10 as it appears in World Health Organization (WHO). WHO Mortality Database Version October 2017. Geneva, Switzerland: World Health Organization (WHO), 2017
Fiji Vital Registration - Deaths 2003 ICD10 as it appears in World Health Organization (WHO). WHO Mortality Database Version October 2017. Geneva, Switzerland: World Health Organization (WHO), 2017
Fiji Vital Registration - Deaths 2004 ICD10 as it appears in World Health Organization (WHO). WHO Mortality Database Version October 2017. Geneva, Switzerland: World Health Organization (WHO), 2017
Fiji Vital Registration - Deaths 2005 ICD10 as it appears in World Health Organization (WHO). WHO Mortality Database Version October 2017. Geneva, Switzerland: World Health Organization (WHO), 2017
Fiji Vital Registration - Deaths 2006 ICD10 as it appears in World Health Organization (WHO). WHO Mortality Database Version October 2017. Geneva, Switzerland: World Health Organization (WHO), 2017
Fiji Vital Registration - Deaths 2007 ICD10 as it appears in World Health Organization (WHO). WHO Mortality Database Version October 2017. Geneva, Switzerland: World Health Organization (WHO), 2017
Fiji Vital Registration - Deaths 2008 ICD10 as it appears in World Health Organization (WHO). WHO Mortality Database Version October 2017. Geneva, Switzerland: World Health Organization (WHO), 2017
Fiji Vital Registration - Deaths 2009 ICD10 as it appears in World Health Organization (WHO). WHO Mortality Database Version October 2017. Geneva, Switzerland: World Health Organization (WHO), 2017
Fiji Vital Registration - Deaths 2011 ICD10 as it appears in World Health Organization (WHO). WHO Mortality Database Version October 2017. Geneva, Switzerland: World Health Organization (WHO), 2017
Fiji Vital Registration - Deaths 2012 ICD10 as it appears in World Health Organization (WHO). WHO Mortality Database Version October 2017. Geneva, Switzerland: World Health Organization (WHO), 2017
Finland Vital Registration - Deaths 1980 ICD8 as it appears in World Health Organization (WHO). WHO Mortality Database Version July 2012. Geneva, Switzerland: World Health Organization (WHO), 2012
Finland Vital Registration - Deaths 1982 ICD8 as it appears in World Health Organization (WHO). WHO Mortality Database Version July 2012. Geneva, Switzerland: World

Health Organization (WHO), 2012

Finland Vital Registration - Deaths 1983 ICD8 as it appears in World Health Organization (WHO). WHO Mortality Database Version July 2012. Geneva, Switzerland: World Health Organization (WHO), 2012

Finland Vital Registration - Deaths 1984 ICD8 as it appears in World Health Organization (WHO). WHO Mortality Database Version July 2012. Geneva, Switzerland: World Health Organization (WHO), 2012

Finland Vital Registration - Deaths 1985 ICD8 as it appears in World Health Organization (WHO). WHO Mortality Database Version July 2012. Geneva, Switzerland: World Health Organization (WHO), 2012

Finland Vital Registration - Deaths 1986 ICD8 as it appears in World Health Organization (WHO). WHO Mortality Database Version July 2012. Geneva, Switzerland: World Health Organization (WHO), 2012

Finland Vital Registration - Deaths 1987 ICD9 as it appears in World Health Organization (WHO). WHO Mortality Database Version September 2016. Geneva, Switzerland: World Health Organization (WHO), 2016

Finland Vital Registration - Deaths 1988 ICD9 as it appears in World Health Organization (WHO). WHO Mortality Database Version September 2016. Geneva, Switzerland: World Health Organization (WHO), 2016

Finland Vital Registration - Deaths 1989 ICD9 as it appears in World Health Organization (WHO). WHO Mortality Database Version September 2016. Geneva, Switzerland: World Health Organization (WHO), 2016

Finland Vital Registration - Deaths 1990 ICD9 as it appears in World Health Organization (WHO). WHO Mortality Database Version September 2016. Geneva, Switzerland: World Health Organization (WHO), 2016

Finland Vital Registration - Deaths 1991 ICD9 as it appears in World Health Organization (WHO). WHO Mortality Database Version September 2016. Geneva, Switzerland: World Health Organization (WHO), 2016

Finland Vital Registration - Deaths 1992 ICD9 as it appears in World Health Organization (WHO). WHO Mortality Database Version September 2016. Geneva, Switzerland: World Health Organization (WHO), 2016

Finland Vital Registration - Deaths 1993 ICD9 as it appears in World Health Organization (WHO). WHO Mortality Database Version September 2016. Geneva, Switzerland: World Health Organization (WHO), 2016

Finland Vital Registration - Deaths 1994 ICD9 as it appears in World Health Organization (WHO). WHO Mortality Database Version September 2016. Geneva, Switzerland: World Health Organization (WHO), 2016

Finland Vital Registration - Deaths 1995 ICD9 as it appears in World Health Organization (WHO). WHO Mortality Database Version September 2016. Geneva, Switzerland: World Health Organization (WHO), 2016

Finland Vital Registration - Deaths 1996 ICD10 as it appears in World Health Organization (WHO). WHO Mortality Database Version October 2017. Geneva, Switzerland: World Health Organization (WHO), 2017

Finland Vital Registration - Deaths 1997 ICD10 as it appears in World Health Organization (WHO). WHO Mortality Database Version October 2017. Geneva, Switzerland: World Health Organization (WHO), 2017

Finland Vital Registration - Deaths 1998 ICD10 as it appears in World Health Organization (WHO). WHO Mortality Database Version October 2017. Geneva, Switzerland: World Health Organization (WHO), 2017

Finland Vital Registration - Deaths 1999 ICD10 as it appears in World Health Organization (WHO). WHO Mortality Database Version October 2017. Geneva, Switzerland: World Health Organization (WHO), 2017

Finland Vital Registration - Deaths 2000 ICD10 as it appears in World Health Organization (WHO). WHO Mortality Database Version October 2017. Geneva, Switzerland: World Health Organization (WHO), 2017

Finland Vital Registration - Deaths 2001 ICD10 as it appears in World Health Organization (WHO). WHO Mortality Database Version October 2017. Geneva, Switzerland: World Health Organization (WHO), 2017

Finland Vital Registration - Deaths 2002 ICD10 as it appears in World Health Organization (WHO). WHO Mortality Database Version October 2017. Geneva, Switzerland: World Health Organization (WHO), 2017

Finland Vital Registration - Deaths 2003 ICD10 as it appears in World Health Organization (WHO). WHO Mortality Database Version October 2017. Geneva, Switzerland: World Health Organization (WHO), 2017
Finland Vital Registration - Deaths 2004 ICD10 as it appears in World Health Organization (WHO). WHO Mortality Database Version October 2017. Geneva, Switzerland: World Health Organization (WHO), 2017
Finland Vital Registration - Deaths 2005 ICD10 as it appears in World Health Organization (WHO). WHO Mortality Database Version October 2017. Geneva, Switzerland: World Health Organization (WHO), 2017
Finland Vital Registration - Deaths 2006 ICD10 as it appears in World Health Organization (WHO). WHO Mortality Database Version October 2017. Geneva, Switzerland: World Health Organization (WHO), 2017
Finland Vital Registration - Deaths 2007 ICD10 as it appears in World Health Organization (WHO). WHO Mortality Database Version October 2017. Geneva, Switzerland: World Health Organization (WHO), 2017
Finland Vital Registration - Deaths 2008 ICD10 as it appears in World Health Organization (WHO). WHO Mortality Database Version October 2017. Geneva, Switzerland: World Health Organization (WHO), 2017
Finland Vital Registration - Deaths 1981 ICD8 as it appears in World Health Organization (WHO). WHO Mortality Database Version July 2012. Geneva, Switzerland: World Health Organization (WHO), 2012
Finland Vital Registration - Deaths 2009 ICD10 as it appears in World Health Organization (WHO). WHO Mortality Database Version October 2017. Geneva, Switzerland: World Health Organization (WHO), 2017
Finland Vital Registration - Deaths 2010 ICD10 as it appears in World Health Organization (WHO). WHO Mortality Database Version October 2017. Geneva, Switzerland: World Health Organization (WHO), 2017
Finland Vital Registration - Deaths 2011 ICD10 as it appears in World Health Organization (WHO). WHO Mortality Database Version October 2017. Geneva, Switzerland: World Health Organization (WHO), 2017
Finland Vital Registration - Deaths 2012 ICD10 as it appears in World Health Organization (WHO). WHO Mortality Database Version October 2017. Geneva, Switzerland: World Health Organization (WHO), 2017
Finland Vital Registration - Deaths 2013 ICD10 as it appears in World Health Organization (WHO). WHO Mortality Database Version October 2017. Geneva, Switzerland: World Health Organization (WHO), 2017
Finland Vital Registration - Deaths 2014 ICD10 as it appears in World Health Organization (WHO). WHO Mortality Database Version October 2017. Geneva, Switzerland: World Health Organization (WHO), 2017
Finland Vital Registration - Deaths 2015 ICD10 as it appears in World Health Organization (WHO). WHO Mortality Database Version October 2017. Geneva, Switzerland: World Health Organization (WHO), 2017
France Vital Registration - Deaths 1980 ICD9 as it appears in World Health Organization (WHO). WHO Mortality Database Version November 2015. Geneva, Switzerland: World Health Organization (WHO), 2015
France Vital Registration - Deaths 1981 ICD9 as it appears in World Health Organization (WHO). WHO Mortality Database Version November 2015. Geneva, Switzerland: World Health Organization (WHO), 2015
France Vital Registration - Deaths 1982 ICD9 as it appears in World Health Organization (WHO). WHO Mortality Database Version November 2015. Geneva, Switzerland: World Health Organization (WHO), 2015
France Vital Registration - Deaths 1983 ICD9 as it appears in World Health Organization (WHO). WHO Mortality Database Version November 2015. Geneva, Switzerland: World Health Organization (WHO), 2015
France Vital Registration - Deaths 1984 ICD9 as it appears in World Health Organization (WHO). WHO Mortality Database Version November 2015. Geneva, Switzerland: World Health Organization (WHO), 2015
France Vital Registration - Deaths 1985 ICD9 as it appears in World Health Organization (WHO). WHO Mortality Database Version November 2015. Geneva, Switzerland: World Health Organization (WHO), 2015

France Vital Registration - Deaths 1986 ICD9 as it appears in World Health Organization (WHO). WHO Mortality Database Version November 2015. Geneva, Switzerland: World Health Organization (WHO), 2015
France Vital Registration - Deaths 1987 ICD9 as it appears in World Health Organization (WHO). WHO Mortality Database Version November 2015. Geneva, Switzerland: World Health Organization (WHO), 2015
France Vital Registration - Deaths 1988 ICD9 as it appears in World Health Organization (WHO). WHO Mortality Database Version November 2015. Geneva, Switzerland: World Health Organization (WHO), 2015
France Vital Registration - Deaths 1989 ICD9 as it appears in World Health Organization (WHO). WHO Mortality Database Version November 2015. Geneva, Switzerland: World Health Organization (WHO), 2015
France Vital Registration - Deaths 1990 ICD9 as it appears in World Health Organization (WHO). WHO Mortality Database Version November 2015. Geneva, Switzerland: World Health Organization (WHO), 2015
France Vital Registration - Deaths 1991 ICD9 as it appears in World Health Organization (WHO). WHO Mortality Database Version November 2015. Geneva, Switzerland: World Health Organization (WHO), 2015
France Vital Registration - Deaths 1992 ICD9 as it appears in World Health Organization (WHO). WHO Mortality Database Version November 2015. Geneva, Switzerland: World Health Organization (WHO), 2015
France Vital Registration - Deaths 1993 ICD9 as it appears in World Health Organization (WHO). WHO Mortality Database Version November 2015. Geneva, Switzerland: World Health Organization (WHO), 2015
France Vital Registration - Deaths 1994 ICD9 as it appears in World Health Organization (WHO). WHO Mortality Database Version November 2015. Geneva, Switzerland: World Health Organization (WHO), 2015
France Vital Registration - Deaths 1995 ICD9 as it appears in World Health Organization (WHO). WHO Mortality Database Version November 2015. Geneva, Switzerland: World Health Organization (WHO), 2015
France Vital Registration - Deaths 1996 ICD9 as it appears in World Health Organization (WHO). WHO Mortality Database Version November 2015. Geneva, Switzerland: World Health Organization (WHO), 2015
France Vital Registration - Deaths 1997 ICD9 as it appears in World Health Organization (WHO). WHO Mortality Database Version November 2015. Geneva, Switzerland: World Health Organization (WHO), 2015
France Vital Registration - Deaths 1998 ICD9 as it appears in World Health Organization (WHO). WHO Mortality Database Version November 2015. Geneva, Switzerland: World Health Organization (WHO), 2015
France Vital Registration - Deaths 1999 ICD9 as it appears in World Health Organization (WHO). WHO Mortality Database Version November 2015. Geneva, Switzerland: World Health Organization (WHO), 2015
France Vital Registration - Deaths 2000 ICD10 as it appears in World Health Organization (WHO). WHO Mortality Database Version October 2017. Geneva, Switzerland: World Health Organization (WHO), 2017
France Vital Registration - Deaths 2001 ICD10 as it appears in World Health Organization (WHO). WHO Mortality Database Version October 2017. Geneva, Switzerland: World Health Organization (WHO), 2017
France Vital Registration - Deaths 2002 ICD10 as it appears in World Health Organization (WHO). WHO Mortality Database Version October 2017. Geneva, Switzerland: World Health Organization (WHO), 2017
France Vital Registration - Deaths 2003 ICD10 as it appears in World Health Organization (WHO). WHO Mortality Database Version October 2017. Geneva, Switzerland: World Health Organization (WHO), 2017
France Vital Registration - Deaths 2004 ICD10 as it appears in World Health Organization (WHO). WHO Mortality Database Version October 2017. Geneva, Switzerland: World Health Organization (WHO), 2017
France Vital Registration - Deaths 2005 ICD10 as it appears in World Health Organization (WHO). WHO Mortality Database Version October 2017. Geneva, Switzerland: World Health Organization (WHO), 2017

France Vital Registration - Deaths 2006 ICD10 as it appears in World Health Organization (WHO). WHO Mortality Database Version October 2017. Geneva, Switzerland: World Health Organization (WHO), 2017
France Vital Registration - Deaths 2007 ICD10 as it appears in World Health Organization (WHO). WHO Mortality Database Version October 2017. Geneva, Switzerland: World Health Organization (WHO), 2017
France Vital Registration - Deaths 2008 ICD10 as it appears in World Health Organization (WHO). WHO Mortality Database Version October 2017. Geneva, Switzerland: World Health Organization (WHO), 2017
France Vital Registration - Deaths 2009 ICD10 as it appears in World Health Organization (WHO). WHO Mortality Database Version October 2017. Geneva, Switzerland: World Health Organization (WHO), 2017
France Vital Registration - Deaths 2010 ICD10 as it appears in World Health Organization (WHO). WHO Mortality Database Version October 2017. Geneva, Switzerland: World Health Organization (WHO), 2017
France Vital Registration - Deaths 2011 ICD10 as it appears in World Health Organization (WHO). WHO Mortality Database Version October 2017. Geneva, Switzerland: World Health Organization (WHO), 2017
France Vital Registration - Deaths 2012 ICD10 as it appears in World Health Organization (WHO). WHO Mortality Database Version October 2017. Geneva, Switzerland: World Health Organization (WHO), 2017
France Vital Registration - Deaths 2013 ICD10 as it appears in World Health Organization (WHO). WHO Mortality Database Version October 2017. Geneva, Switzerland: World Health Organization (WHO), 2017
France Vital Registration - Deaths 2014 ICD10 as it appears in World Health Organization (WHO). WHO Mortality Database Version October 2017. Geneva, Switzerland: World Health Organization (WHO), 2017
Georgia Vital Registration - Deaths 1981 ICD9 as it appears in World Health Organization (WHO). WHO Mortality Database Version September 2016. Geneva, Switzerland: World Health Organization (WHO), 2016
Georgia Vital Registration - Deaths 1982 ICD9 as it appears in World Health Organization (WHO). WHO Mortality Database Version September 2016. Geneva, Switzerland: World Health Organization (WHO), 2016
Georgia Vital Registration - Deaths 1985 ICD9 as it appears in World Health Organization (WHO). WHO Mortality Database Version September 2016. Geneva, Switzerland: World Health Organization (WHO), 2016
Georgia Vital Registration - Deaths 1986 ICD9 as it appears in World Health Organization (WHO). WHO Mortality Database Version September 2016. Geneva, Switzerland: World Health Organization (WHO), 2016
Georgia Vital Registration - Deaths 1987 ICD9 as it appears in World Health Organization (WHO). WHO Mortality Database Version September 2016. Geneva, Switzerland: World Health Organization (WHO), 2016
Georgia Vital Registration - Deaths 1988 ICD9 as it appears in World Health Organization (WHO). WHO Mortality Database Version September 2016. Geneva, Switzerland: World Health Organization (WHO), 2016
Georgia Vital Registration - Deaths 1989 ICD9 as it appears in World Health Organization (WHO). WHO Mortality Database Version September 2016. Geneva, Switzerland: World Health Organization (WHO), 2016
Georgia Vital Registration - Deaths 1990 ICD9 as it appears in World Health Organization (WHO). WHO Mortality Database Version September 2016. Geneva, Switzerland: World Health Organization (WHO), 2016
Georgia Vital Registration - Deaths 1991 ICD9 as it appears in World Health Organization (WHO). WHO Mortality Database Version September 2016. Geneva, Switzerland: World Health Organization (WHO), 2016
Georgia Vital Registration - Deaths 1992 ICD9 as it appears in World Health Organization (WHO). WHO Mortality Database Version September 2016. Geneva, Switzerland: World Health Organization (WHO), 2016
Georgia Vital Registration - Deaths 1994 ICD9 as it appears in World Health Organization (WHO). WHO Mortality Database Version September 2016. Geneva, Switzerland: World Health Organization (WHO), 2016

Georgia Vital Registration - Deaths 1995 ICD9 as it appears in World Health Organization (WHO). WHO Mortality Database Version September 2016. Geneva, Switzerland: World Health Organization (WHO), 2016
Georgia Vital Registration - Deaths 1996 ICD9 as it appears in World Health Organization (WHO). WHO Mortality Database Version September 2016. Geneva, Switzerland: World Health Organization (WHO), 2016
Georgia Vital Registration - Deaths 1997 ICD9 as it appears in World Health Organization (WHO). WHO Mortality Database Version September 2016. Geneva, Switzerland: World Health Organization (WHO), 2016
Georgia Vital Registration - Deaths 1998 ICD10 as it appears in World Health Organization (WHO). WHO Mortality Database Version October 2017. Geneva, Switzerland: World Health Organization (WHO), 2017
Georgia Vital Registration - Deaths 1999 ICD10 as it appears in World Health Organization (WHO). WHO Mortality Database Version October 2017. Geneva, Switzerland: World Health Organization (WHO), 2017
Georgia Vital Registration - Deaths 2000 ICD10 as it appears in World Health Organization (WHO). WHO Mortality Database Version October 2017. Geneva, Switzerland: World Health Organization (WHO), 2017
Georgia Vital Registration - Deaths 2001 ICD10 as it appears in World Health Organization (WHO). WHO Mortality Database Version October 2017. Geneva, Switzerland: World Health Organization (WHO), 2017
Georgia Vital Registration - Deaths 2004 ICD10 as it appears in World Health Organization (WHO). WHO Mortality Database Version October 2017. Geneva, Switzerland: World Health Organization (WHO), 2017
Georgia Vital Registration - Deaths 2005 ICD10 as it appears in World Health Organization (WHO). WHO Mortality Database Version October 2017. Geneva, Switzerland: World Health Organization (WHO), 2017
Georgia Vital Registration - Deaths 2006 ICD10 as it appears in World Health Organization (WHO). WHO Mortality Database Version October 2017. Geneva, Switzerland: World Health Organization (WHO), 2017
Georgia Vital Registration - Deaths 2007 ICD10 as it appears in World Health Organization (WHO). WHO Mortality Database Version October 2017. Geneva, Switzerland: World Health Organization (WHO), 2017
Georgia Vital Registration - Deaths 2009 ICD10 as it appears in World Health Organization (WHO). WHO Mortality Database Version October 2017. Geneva, Switzerland: World Health Organization (WHO), 2017
Georgia Vital Registration - Deaths 2012 ICD10 as it appears in World Health Organization (WHO). WHO Mortality Database Version October 2017. Geneva, Switzerland: World Health Organization (WHO), 2017
Georgia Vital Registration - Deaths 2014 ICD10 as it appears in World Health Organization (WHO). WHO Mortality Database Version October 2017. Geneva, Switzerland: World Health Organization (WHO), 2017
Georgia Vital Registration - Deaths 2015 ICD10 as it appears in World Health Organization (WHO). WHO Mortality Database Version October 2017. Geneva, Switzerland: World Health Organization (WHO), 2017
Germany Vital Registration - Deaths 1991 ICD9 as it appears in World Health Organization (WHO). WHO Mortality Database Version September 2016. Geneva, Switzerland: World Health Organization (WHO), 2016
Germany Vital Registration - Deaths 1992 ICD9 as it appears in World Health Organization (WHO). WHO Mortality Database Version September 2016. Geneva, Switzerland: World Health Organization (WHO), 2016
Germany Vital Registration - Deaths 1993 ICD9 as it appears in World Health Organization (WHO). WHO Mortality Database Version September 2016. Geneva, Switzerland: World Health Organization (WHO), 2016
Germany Vital Registration - Deaths 1994 ICD9 as it appears in World Health Organization (WHO). WHO Mortality Database Version September 2016. Geneva, Switzerland: World Health Organization (WHO), 2016
Germany Vital Registration - Deaths 1995 ICD9 as it appears in World Health Organization (WHO). WHO Mortality Database Version November 2015. Geneva, Switzerland: World Health Organization (WHO), 2015

Germany Vital Registration - Deaths 1996 ICD9 as it appears in World Health Organization (WHO). WHO Mortality Database Version November 2015. Geneva, Switzerland: World Health Organization (WHO), 2015
Germany Vital Registration - Deaths 1997 ICD9 as it appears in World Health Organization (WHO). WHO Mortality Database Version November 2015. Geneva, Switzerland: World Health Organization (WHO), 2015
Germany Vital Registration - Deaths 1998 ICD10 as it appears in World Health Organization (WHO). WHO Mortality Database Version October 2017. Geneva, Switzerland: World Health Organization (WHO), 2017
Germany Vital Registration - Deaths 1999 ICD10 as it appears in World Health Organization (WHO). WHO Mortality Database Version October 2017. Geneva, Switzerland: World Health Organization (WHO), 2017
Germany Vital Registration - Deaths 2000 ICD10 as it appears in World Health Organization (WHO). WHO Mortality Database Version October 2017. Geneva, Switzerland: World Health Organization (WHO), 2017
Germany Vital Registration - Deaths 2001 ICD10 as it appears in World Health Organization (WHO). WHO Mortality Database Version October 2017. Geneva, Switzerland: World Health Organization (WHO), 2017
Germany Vital Registration - Deaths 2002 ICD10 as it appears in World Health Organization (WHO). WHO Mortality Database Version October 2017. Geneva, Switzerland: World Health Organization (WHO), 2017
Germany Vital Registration - Deaths 2003 ICD10 as it appears in World Health Organization (WHO). WHO Mortality Database Version October 2017. Geneva, Switzerland: World Health Organization (WHO), 2017
Germany Vital Registration - Deaths 2004 ICD10 as it appears in World Health Organization (WHO). WHO Mortality Database Version October 2017. Geneva, Switzerland: World Health Organization (WHO), 2017
Germany Vital Registration - Deaths 2005 ICD10 as it appears in World Health Organization (WHO). WHO Mortality Database Version October 2017. Geneva, Switzerland: World Health Organization (WHO), 2017
Germany Vital Registration - Deaths 2006 ICD10 as it appears in World Health Organization (WHO). WHO Mortality Database Version October 2017. Geneva, Switzerland: World Health Organization (WHO), 2017
Germany Vital Registration - Deaths 1980 ICD9 as it appears in World Health Organization (WHO). WHO Mortality Database Version September 2016. Geneva, Switzerland: World Health Organization (WHO), 2016
Germany Vital Registration - Deaths 1981 ICD9 as it appears in World Health Organization (WHO). WHO Mortality Database Version September 2016. Geneva, Switzerland: World Health Organization (WHO), 2016
Germany Vital Registration - Deaths 1982 ICD9 as it appears in World Health Organization (WHO). WHO Mortality Database Version September 2016. Geneva, Switzerland: World Health Organization (WHO), 2016
Germany Vital Registration - Deaths 1983 ICD9 as it appears in World Health Organization (WHO). WHO Mortality Database Version September 2016. Geneva, Switzerland: World Health Organization (WHO), 2016
Germany Vital Registration - Deaths 1984 ICD9 as it appears in World Health Organization (WHO). WHO Mortality Database Version September 2016. Geneva, Switzerland: World Health Organization (WHO), 2016
Germany Vital Registration - Deaths 1985 ICD9 as it appears in World Health Organization (WHO). WHO Mortality Database Version September 2016. Geneva, Switzerland: World Health Organization (WHO), 2016
Germany Vital Registration - Deaths 1986 ICD9 as it appears in World Health Organization (WHO). WHO Mortality Database Version September 2016. Geneva, Switzerland: World Health Organization (WHO), 2016
Germany Vital Registration - Deaths 1987 ICD9 as it appears in World Health Organization (WHO). WHO Mortality Database Version September 2016. Geneva, Switzerland: World Health Organization (WHO), 2016
Germany Vital Registration - Deaths 1988 ICD9 as it appears in World Health Organization (WHO). WHO Mortality Database Version September 2016. Geneva, Switzerland: World Health Organization (WHO), 2016

Germany Vital Registration - Deaths 1989 ICD9 as it appears in World Health Organization (WHO). WHO Mortality Database Version September 2016. Geneva, Switzerland: World Health Organization (WHO), 2016

Germany Vital Registration - Deaths 1990 ICD9 as it appears in World Health Organization (WHO). WHO Mortality Database Version September 2016. Geneva, Switzerland: World Health Organization (WHO), 2016

Germany Vital Registration - Deaths 2007 ICD10 as it appears in World Health Organization (WHO). WHO Mortality Database Version October 2017. Geneva, Switzerland: World Health Organization (WHO), 2017

Germany Vital Registration - Deaths 2008 ICD10 as it appears in World Health Organization (WHO). WHO Mortality Database Version October 2017. Geneva, Switzerland: World Health Organization (WHO), 2017

Germany Vital Registration - Deaths 2009 ICD10 as it appears in World Health Organization (WHO). WHO Mortality Database Version October 2017. Geneva, Switzerland: World Health Organization (WHO), 2017

Germany Vital Registration - Deaths 2010 ICD10 as it appears in World Health Organization (WHO). WHO Mortality Database Version October 2017. Geneva, Switzerland: World Health Organization (WHO), 2017

Germany Vital Registration - Deaths 2011 ICD10 as it appears in World Health Organization (WHO). WHO Mortality Database Version October 2017. Geneva, Switzerland: World Health Organization (WHO), 2017

Germany Vital Registration - Deaths 2012 ICD10 as it appears in World Health Organization (WHO). WHO Mortality Database Version October 2017. Geneva, Switzerland: World Health Organization (WHO), 2017

Germany Vital Registration - Deaths 2013 ICD10 as it appears in World Health Organization (WHO). WHO Mortality Database Version October 2017. Geneva, Switzerland: World Health Organization (WHO), 2017

Germany Vital Registration - Deaths 2014 ICD10 as it appears in World Health Organization (WHO). WHO Mortality Database Version October 2017. Geneva, Switzerland: World Health Organization (WHO), 2017

Germany Vital Registration - Deaths 2015 ICD10 as it appears in World Health Organization (WHO). WHO Mortality Database Version October 2017. Geneva, Switzerland: World Health Organization (WHO), 2017

Ghana Health Service, Ghana Statistical Service, Macro International, Inc. Ghana Special Demographic and Health Survey 2007-2008. Fairfax, United States: ICF International

Greece Vital Registration - Deaths 1980 ICD9 as it appears in World Health Organization (WHO). WHO Mortality Database Version September 2016. Geneva, Switzerland: World Health Organization (WHO), 2016

Greece Vital Registration - Deaths 1981 ICD9 as it appears in World Health Organization (WHO). WHO Mortality Database Version September 2016. Geneva, Switzerland: World Health Organization (WHO), 2016

Greece Vital Registration - Deaths 1982 ICD9 as it appears in World Health Organization (WHO). WHO Mortality Database Version September 2016. Geneva, Switzerland: World Health Organization (WHO), 2016

Greece Vital Registration - Deaths 1983 ICD9 as it appears in World Health Organization (WHO). WHO Mortality Database Version September 2016. Geneva, Switzerland: World Health Organization (WHO), 2016

Greece Vital Registration - Deaths 1984 ICD9 as it appears in World Health Organization (WHO). WHO Mortality Database Version September 2016. Geneva, Switzerland: World Health Organization (WHO), 2016

Greece Vital Registration - Deaths 1985 ICD9 as it appears in World Health Organization (WHO). WHO Mortality Database Version September 2016. Geneva, Switzerland: World Health Organization (WHO), 2016

Greece Vital Registration - Deaths 1986 ICD9 as it appears in World Health Organization (WHO). WHO Mortality Database Version September 2016. Geneva, Switzerland: World Health Organization (WHO), 2016

Greece Vital Registration - Deaths 1987 ICD9 as it appears in World Health Organization (WHO). WHO Mortality Database Version November 2015. Geneva, Switzerland: World Health Organization (WHO), 2015

Greece Vital Registration - Deaths 1988 ICD9 as it appears in World Health Organization (WHO). WHO Mortality Database Version November 2015. Geneva, Switzerland: World Health Organization (WHO), 2015
Greece Vital Registration - Deaths 1989 ICD9 as it appears in World Health Organization (WHO). WHO Mortality Database Version November 2015. Geneva, Switzerland: World Health Organization (WHO), 2015
Greece Vital Registration - Deaths 1990 ICD9 as it appears in World Health Organization (WHO). WHO Mortality Database Version November 2015. Geneva, Switzerland: World Health Organization (WHO), 2015
Greece Vital Registration - Deaths 1991 ICD9 as it appears in World Health Organization (WHO). WHO Mortality Database Version November 2015. Geneva, Switzerland: World Health Organization (WHO), 2015
Greece Vital Registration - Deaths 1992 ICD9 as it appears in World Health Organization (WHO). WHO Mortality Database Version November 2015. Geneva, Switzerland: World Health Organization (WHO), 2015
Greece Vital Registration - Deaths 1993 ICD9 as it appears in World Health Organization (WHO). WHO Mortality Database Version November 2015. Geneva, Switzerland: World Health Organization (WHO), 2015
Greece Vital Registration - Deaths 1994 ICD9 as it appears in World Health Organization (WHO). WHO Mortality Database Version November 2015. Geneva, Switzerland: World Health Organization (WHO), 2015
Greece Vital Registration - Deaths 1995 ICD9 as it appears in World Health Organization (WHO). WHO Mortality Database Version November 2015. Geneva, Switzerland: World Health Organization (WHO), 2015
Greece Vital Registration - Deaths 1996 ICD9 as it appears in World Health Organization (WHO). WHO Mortality Database Version November 2015. Geneva, Switzerland: World Health Organization (WHO), 2015
Greece Vital Registration - Deaths 1997 ICD9 as it appears in World Health Organization (WHO). WHO Mortality Database Version November 2015. Geneva, Switzerland: World Health Organization (WHO), 2015
Greece Vital Registration - Deaths 1998 ICD9 as it appears in World Health Organization (WHO). WHO Mortality Database Version November 2015. Geneva, Switzerland: World Health Organization (WHO), 2015
Greece Vital Registration - Deaths 1999 ICD9 as it appears in World Health Organization (WHO). WHO Mortality Database Version November 2015. Geneva, Switzerland: World Health Organization (WHO), 2015
Greece Vital Registration - Deaths 2000 ICD9 as it appears in World Health Organization (WHO). WHO Mortality Database Version November 2015. Geneva, Switzerland: World Health Organization (WHO), 2015
Greece Vital Registration - Deaths 2001 ICD9 as it appears in World Health Organization (WHO). WHO Mortality Database Version November 2015. Geneva, Switzerland: World Health Organization (WHO), 2015
Greece Vital Registration - Deaths 2002 ICD9 as it appears in World Health Organization (WHO). WHO Mortality Database Version November 2015. Geneva, Switzerland: World Health Organization (WHO), 2015
Greece Vital Registration - Deaths 2003 ICD9 as it appears in World Health Organization (WHO). WHO Mortality Database Version November 2015. Geneva, Switzerland: World Health Organization (WHO), 2015
Greece Vital Registration - Deaths 2004 ICD9 as it appears in World Health Organization (WHO). WHO Mortality Database Version November 2015. Geneva, Switzerland: World Health Organization (WHO), 2015
Greece Vital Registration - Deaths 2005 ICD9 as it appears in World Health Organization (WHO). WHO Mortality Database Version November 2015. Geneva, Switzerland: World Health Organization (WHO), 2015
Greece Vital Registration - Deaths 2006 ICD9 as it appears in World Health Organization (WHO). WHO Mortality Database Version November 2015. Geneva, Switzerland: World Health Organization (WHO), 2015
Greece Vital Registration - Deaths 2007 ICD9 as it appears in World Health Organization (WHO). WHO Mortality Database Version September 2016. Geneva, Switzerland: World Health Organization (WHO), 2016

Greece Vital Registration - Deaths 2008 ICD9 as it appears in World Health Organization (WHO). WHO Mortality Database Version November 2015. Geneva, Switzerland: World Health Organization (WHO), 2015
Greece Vital Registration - Deaths 2009 ICD9 as it appears in World Health Organization (WHO). WHO Mortality Database Version September 2016. Geneva, Switzerland: World Health Organization (WHO), 2016
Greece Vital Registration - Deaths 2010 ICD9 as it appears in World Health Organization (WHO). WHO Mortality Database Version September 2016. Geneva, Switzerland: World Health Organization (WHO), 2016
Greece Vital Registration - Deaths 2011 ICD9 as it appears in World Health Organization (WHO). WHO Mortality Database Version September 2016. Geneva, Switzerland: World Health Organization (WHO), 2016
Greece Vital Registration - Deaths 2012 ICD9 as it appears in World Health Organization (WHO). WHO Mortality Database Version September 2016. Geneva, Switzerland: World Health Organization (WHO), 2016
Greece Vital Registration - Deaths 2013 ICD9 as it appears in World Health Organization (WHO). WHO Mortality Database Version September 2016. Geneva, Switzerland: World Health Organization (WHO), 2016
Greece Vital Registration - Deaths 2014 ICD10 as it appears in World Health Organization (WHO). WHO Mortality Database Version March 2017. Geneva, Switzerland: World Health Organization (WHO), 2017
Greece Vital Registration - Deaths 2015 ICD10 as it appears in World Health Organization (WHO). WHO Mortality Database Version April 2018. Geneva, Switzerland: World Health Organization (WHO), 2018
Grenada Vital Registration - Deaths 1984 ICD9 as it appears in World Health Organization (WHO). WHO Mortality Database Version September 2016. Geneva, Switzerland: World Health Organization (WHO), 2016
Grenada Vital Registration - Deaths 1985 ICD9 as it appears in World Health Organization (WHO). WHO Mortality Database Version September 2016. Geneva, Switzerland: World Health Organization (WHO), 2016
Grenada Vital Registration - Deaths 1988 ICD9 as it appears in World Health Organization (WHO). WHO Mortality Database Version September 2016. Geneva, Switzerland: World Health Organization (WHO), 2016
Grenada Vital Registration - Deaths 1989 ICD9 as it appears in World Health Organization (WHO). WHO Mortality Database Version September 2016. Geneva, Switzerland: World Health Organization (WHO), 2016
Grenada Vital Registration - Deaths 1990 ICD9 as it appears in World Health Organization (WHO). WHO Mortality Database Version September 2016. Geneva, Switzerland: World Health Organization (WHO), 2016
Grenada Vital Registration - Deaths 1991 ICD9 as it appears in World Health Organization (WHO). WHO Mortality Database Version September 2016. Geneva, Switzerland: World Health Organization (WHO), 2016
Grenada Vital Registration - Deaths 1992 ICD9 as it appears in World Health Organization (WHO). WHO Mortality Database Version September 2016. Geneva, Switzerland: World Health Organization (WHO), 2016
Grenada Vital Registration - Deaths 1993 ICD9 as it appears in World Health Organization (WHO). WHO Mortality Database Version September 2016. Geneva, Switzerland: World Health Organization (WHO), 2016
Grenada Vital Registration - Deaths 1994 ICD9 as it appears in World Health Organization (WHO). WHO Mortality Database Version September 2016. Geneva, Switzerland: World Health Organization (WHO), 2016
Grenada Vital Registration - Deaths 1995 ICD9 as it appears in World Health Organization (WHO). WHO Mortality Database Version September 2016. Geneva, Switzerland: World Health Organization (WHO), 2016
Grenada Vital Registration - Deaths 1996 ICD9 as it appears in World Health Organization (WHO). WHO Mortality Database Version September 2016. Geneva, Switzerland: World Health Organization (WHO), 2016
Grenada Vital Registration - Deaths 2001 ICD10 as it appears in World Health Organization (WHO). WHO Mortality Database Version October 2017. Geneva, Switzerland: World Health Organization (WHO), 2017

Grenada Vital Registration - Deaths 2002 ICD10 as it appears in World Health Organization (WHO). WHO Mortality Database Version October 2017. Geneva, Switzerland: World Health Organization (WHO), 2017
Grenada Vital Registration - Deaths 2003 ICD10 as it appears in World Health Organization (WHO). WHO Mortality Database Version October 2017. Geneva, Switzerland: World Health Organization (WHO), 2017
Grenada Vital Registration - Deaths 2004 ICD10 as it appears in World Health Organization (WHO). WHO Mortality Database Version October 2017. Geneva, Switzerland: World Health Organization (WHO), 2017
Grenada Vital Registration - Deaths 2005 ICD10 as it appears in World Health Organization (WHO). WHO Mortality Database Version October 2017. Geneva, Switzerland: World Health Organization (WHO), 2017
Grenada Vital Registration - Deaths 2007 ICD10 as it appears in World Health Organization (WHO). WHO Mortality Database Version October 2017. Geneva, Switzerland: World Health Organization (WHO), 2017
Grenada Vital Registration - Deaths 2006 ICD10 as it appears in World Health Organization (WHO). WHO Mortality Database Version October 2017. Geneva, Switzerland: World Health Organization (WHO), 2017
Grenada Vital Registration - Deaths 2008 ICD10 as it appears in World Health Organization (WHO). WHO Mortality Database Version October 2017. Geneva, Switzerland: World Health Organization (WHO), 2017
Grenada Vital Registration - Deaths 2009 ICD10 as it appears in World Health Organization (WHO). WHO Mortality Database Version October 2017. Geneva, Switzerland: World Health Organization (WHO), 2017
Grenada Vital Registration - Deaths 2010 ICD10 as it appears in World Health Organization (WHO). WHO Mortality Database Version October 2017. Geneva, Switzerland: World Health Organization (WHO), 2017
Grenada Vital Registration - Deaths 2011 ICD10 as it appears in World Health Organization (WHO). WHO Mortality Database Version October 2017. Geneva, Switzerland: World Health Organization (WHO), 2017
Grenada Vital Registration - Deaths 2012 ICD10 as it appears in World Health Organization (WHO). WHO Mortality Database Version October 2017. Geneva, Switzerland: World Health Organization (WHO), 2017
Grenada Vital Registration - Deaths 2013 ICD10 as it appears in World Health Organization (WHO). WHO Mortality Database Version October 2017. Geneva, Switzerland: World Health Organization (WHO), 2017
Grenada Vital Registration - Deaths 2014 ICD10 as it appears in World Health Organization (WHO). WHO Mortality Database Version October 2017. Geneva, Switzerland: World Health Organization (WHO), 2017
Grenada Vital Registration - Deaths 2015 ICD10 as it appears in World Health Organization (WHO). WHO Mortality Database Version October 2017. Geneva, Switzerland: World Health Organization (WHO), 2017
Grenada Vital Registration - Deaths 2016 ICD10 as it appears in World Health Organization (WHO). WHO Mortality Database Version October 2017. Geneva, Switzerland: World Health Organization (WHO), 2017
Guatemala Vital Registration - Deaths 1980 ICD9 as it appears in World Health Organization (WHO). WHO Mortality Database Version September 2016. Geneva, Switzerland: World Health Organization (WHO), 2016
Guatemala Vital Registration - Deaths 1981 ICD9 as it appears in World Health Organization (WHO). WHO Mortality Database Version September 2016. Geneva, Switzerland: World Health Organization (WHO), 2016
Guatemala Vital Registration - Deaths 1984 ICD9 as it appears in World Health Organization (WHO). WHO Mortality Database Version September 2016. Geneva, Switzerland: World Health Organization (WHO), 2016
Guatemala Vital Registration - Deaths 1986 ICD9 as it appears in World Health Organization (WHO). WHO Mortality Database Version September 2016. Geneva, Switzerland: World Health Organization (WHO), 2016
Guatemala Vital Registration - Deaths 1987 ICD9 as it appears in World Health Organization (WHO). WHO Mortality Database Version September 2016. Geneva, Switzerland: World Health Organization (WHO), 2016

Guatemala Vital Registration - Deaths 1988 ICD9 as it appears in World Health Organization (WHO). WHO Mortality Database Version September 2016. Geneva, Switzerland: World Health Organization (WHO), 2016
Guatemala Vital Registration - Deaths 1989 ICD9 as it appears in World Health Organization (WHO). WHO Mortality Database Version September 2016. Geneva, Switzerland: World Health Organization (WHO), 2016
Guatemala Vital Registration - Deaths 1990 ICD9 as it appears in World Health Organization (WHO). WHO Mortality Database Version September 2016. Geneva, Switzerland: World Health Organization (WHO), 2016
Guatemala Vital Registration - Deaths 1991 ICD9 as it appears in World Health Organization (WHO). WHO Mortality Database Version September 2016. Geneva, Switzerland: World Health Organization (WHO), 2016
Guatemala Vital Registration - Deaths 1992 ICD9 as it appears in World Health Organization (WHO). WHO Mortality Database Version September 2016. Geneva, Switzerland: World Health Organization (WHO), 2016
Guatemala Vital Registration - Deaths 1993 ICD9 as it appears in World Health Organization (WHO). WHO Mortality Database Version September 2016. Geneva, Switzerland: World Health Organization (WHO), 2016
Guatemala Vital Registration - Deaths 1994 ICD9 as it appears in World Health Organization (WHO). WHO Mortality Database Version September 2016. Geneva, Switzerland: World Health Organization (WHO), 2016
Guatemala Vital Registration - Deaths 1995 ICD9 as it appears in World Health Organization (WHO). WHO Mortality Database Version September 2016. Geneva, Switzerland: World Health Organization (WHO), 2016
Guatemala Vital Registration - Deaths 1996 ICD9 as it appears in World Health Organization (WHO). WHO Mortality Database Version September 2016. Geneva, Switzerland: World Health Organization (WHO), 2016
Guatemala Vital Registration - Deaths 1997 ICD9 as it appears in World Health Organization (WHO). WHO Mortality Database Version September 2016. Geneva, Switzerland: World Health Organization (WHO), 2016
Guatemala Vital Registration - Deaths 1998 ICD9 as it appears in World Health Organization (WHO). WHO Mortality Database Version September 2016. Geneva, Switzerland: World Health Organization (WHO), 2016
Guatemala Vital Registration - Deaths 1999 ICD9 as it appears in World Health Organization (WHO). WHO Mortality Database Version September 2016. Geneva, Switzerland: World Health Organization (WHO), 2016
Guatemala Vital Registration - Deaths 2000 ICD9 as it appears in World Health Organization (WHO). WHO Mortality Database Version November 2015. Geneva, Switzerland: World Health Organization (WHO), 2015
Guatemala Vital Registration - Deaths 2001 ICD9 as it appears in World Health Organization (WHO). WHO Mortality Database Version November 2015. Geneva, Switzerland: World Health Organization (WHO), 2015
Guatemala Vital Registration - Deaths 2002 ICD9 as it appears in World Health Organization (WHO). WHO Mortality Database Version November 2015. Geneva, Switzerland: World Health Organization (WHO), 2015
Guatemala Vital Registration - Deaths 2003 ICD9 as it appears in World Health Organization (WHO). WHO Mortality Database Version November 2015. Geneva, Switzerland: World Health Organization (WHO), 2015
Guatemala Vital Registration - Deaths 2004 ICD9 as it appears in World Health Organization (WHO). WHO Mortality Database Version November 2015. Geneva, Switzerland: World Health Organization (WHO), 2015
Guatemala Vital Registration - Deaths 2005 ICD10 as it appears in World Health Organization (WHO). WHO Mortality Database Version October 2017. Geneva, Switzerland: World Health Organization (WHO), 2017
Guatemala Vital Registration - Deaths 2006 ICD10 as it appears in World Health Organization (WHO). WHO Mortality Database Version October 2017. Geneva, Switzerland: World Health Organization (WHO), 2017
Guatemala Vital Registration - Deaths 2007 ICD10 as it appears in World Health Organization (WHO). WHO Mortality Database Version October 2017. Geneva, Switzerland: World Health Organization (WHO), 2017

Guatemala Vital Registration - Deaths 2008 ICD10 as it appears in World Health Organization (WHO). WHO Mortality Database Version October 2017. Geneva, Switzerland: World Health Organization (WHO), 2017
Guatemala Vital Registration - Deaths 2009 ICD10 as it appears in World Health Organization (WHO). WHO Mortality Database Version October 2017. Geneva, Switzerland: World Health Organization (WHO), 2017
Guatemala Vital Registration - Deaths 2010 ICD10 as it appears in World Health Organization (WHO). WHO Mortality Database Version October 2017. Geneva, Switzerland: World Health Organization (WHO), 2017
Guatemala Vital Registration - Deaths 2011 ICD10 as it appears in World Health Organization (WHO). WHO Mortality Database Version October 2017. Geneva, Switzerland: World Health Organization (WHO), 2017
Guatemala Vital Registration - Deaths 2012 ICD10 as it appears in World Health Organization (WHO). WHO Mortality Database Version October 2017. Geneva, Switzerland: World Health Organization (WHO), 2017
Guatemala Vital Registration - Deaths 2013 ICD10 as it appears in World Health Organization (WHO). WHO Mortality Database Version October 2017. Geneva, Switzerland: World Health Organization (WHO), 2017
Guatemala Vital Registration - Deaths 2014 ICD10 as it appears in World Health Organization (WHO). WHO Mortality Database Version October 2017. Geneva, Switzerland: World Health Organization (WHO), 2017
Guatemala Vital Registration - Deaths 2015 ICD10 as it appears in World Health Organization (WHO). WHO Mortality Database Version October 2017. Geneva, Switzerland: World Health Organization (WHO), 2017
Guyana Vital Registration - Deaths 1984 ICD9 as it appears in World Health Organization (WHO). WHO Mortality Database Version September 2016. Geneva, Switzerland: World Health Organization (WHO), 2016
Guyana Vital Registration - Deaths 1988 ICD9 as it appears in World Health Organization (WHO). WHO Mortality Database Version September 2016. Geneva, Switzerland: World Health Organization (WHO), 2016
Guyana Vital Registration - Deaths 1989 ICD9 as it appears in World Health Organization (WHO). WHO Mortality Database Version September 2016. Geneva, Switzerland: World Health Organization (WHO), 2016
Guyana Vital Registration - Deaths 1990 ICD9 as it appears in World Health Organization (WHO). WHO Mortality Database Version September 2016. Geneva, Switzerland: World Health Organization (WHO), 2016
Guyana Vital Registration - Deaths 1993 ICD9 as it appears in World Health Organization (WHO). WHO Mortality Database Version September 2016. Geneva, Switzerland: World Health Organization (WHO), 2016
Guyana Vital Registration - Deaths 1994 ICD9 as it appears in World Health Organization (WHO). WHO Mortality Database Version September 2016. Geneva, Switzerland: World Health Organization (WHO), 2016
Guyana Vital Registration - Deaths 1995 ICD9 as it appears in World Health Organization (WHO). WHO Mortality Database Version September 2016. Geneva, Switzerland: World Health Organization (WHO), 2016
Guyana Vital Registration - Deaths 1996 ICD9 as it appears in World Health Organization (WHO). WHO Mortality Database Version September 2016. Geneva, Switzerland: World Health Organization (WHO), 2016
Guyana Vital Registration - Deaths 1997 ICD9 as it appears in World Health Organization (WHO). WHO Mortality Database Version September 2016. Geneva, Switzerland: World Health Organization (WHO), 2016
Guyana Vital Registration - Deaths 1998 ICD9 as it appears in World Health Organization (WHO). WHO Mortality Database Version November 2015. Geneva, Switzerland: World Health Organization (WHO), 2015
Guyana Vital Registration - Deaths 1999 ICD9 as it appears in World Health Organization (WHO). WHO Mortality Database Version November 2015. Geneva, Switzerland: World Health Organization (WHO), 2015
Guyana Vital Registration - Deaths 2001 ICD10 as it appears in World Health Organization (WHO). WHO Mortality Database Version October 2017. Geneva, Switzerland: World Health Organization (WHO), 2017

Guyana Vital Registration - Deaths 2002 ICD10 as it appears in World Health Organization (WHO). WHO Mortality Database Version October 2017. Geneva, Switzerland: World Health Organization (WHO), 2017

Guyana Vital Registration - Deaths 2003 ICD10 as it appears in World Health Organization (WHO). WHO Mortality Database Version October 2017. Geneva, Switzerland: World Health Organization (WHO), 2017

Guyana Vital Registration - Deaths 2004 ICD10 as it appears in World Health Organization (WHO). WHO Mortality Database Version October 2017. Geneva, Switzerland: World Health Organization (WHO), 2017

Guyana Vital Registration - Deaths 2005 ICD10 as it appears in World Health Organization (WHO). WHO Mortality Database Version October 2017. Geneva, Switzerland: World Health Organization (WHO), 2017

Guyana Vital Registration - Deaths 2000 ICD10 - PAHO

Guyana Vital Registration - Deaths 2006 ICD10 as it appears in World Health Organization (WHO). WHO Mortality Database Version October 2017. Geneva, Switzerland: World Health Organization (WHO), 2017

Guyana Vital Registration - Deaths 2007 ICD10 as it appears in World Health Organization (WHO). WHO Mortality Database Version October 2017. Geneva, Switzerland: World Health Organization (WHO), 2017

Guyana Vital Registration - Deaths 2008 ICD10 as it appears in World Health Organization (WHO). WHO Mortality Database Version October 2017. Geneva, Switzerland: World Health Organization (WHO), 2017

Guyana Vital Registration - Deaths 2009 ICD10 as it appears in World Health Organization (WHO). WHO Mortality Database Version October 2017. Geneva, Switzerland: World Health Organization (WHO), 2017

Guyana Vital Registration - Deaths 2010 ICD10 as it appears in World Health Organization (WHO). WHO Mortality Database Version October 2017. Geneva, Switzerland: World Health Organization (WHO), 2017

Guyana Vital Registration - Deaths 2011 ICD10 as it appears in World Health Organization (WHO). WHO Mortality Database Version October 2017. Geneva, Switzerland: World Health Organization (WHO), 2017

Guyana Vital Registration - Deaths 2012 ICD10 as it appears in World Health Organization (WHO). WHO Mortality Database Version October 2017. Geneva, Switzerland: World Health Organization (WHO), 2017

Guyana Vital Registration - Deaths 2013 ICD10 as it appears in World Health Organization (WHO). WHO Mortality Database Version October 2017. Geneva, Switzerland: World Health Organization (WHO), 2017

Hanoi School of Public Health, Ministry of Health (Vietnam), School of Population Health, University of Queensland (Australia). Vietnam Burden of Disease and Injury Study 2008

Hoa NP, Rao C, Hoy DG, Hinh ND, Chuc NT, Ngo DA. Mortality measures from sample-based surveillance: evidence of the epidemiological transition in Viet Nam. Bull World Health Organ. 2012; 90(10): 764-72

Hoa NP, Rao C, Hoy DG, Hinh ND, Chuc NT, Ngo DA. Mortality measures from sample-based surveillance: evidence of the epidemiological transition in Viet Nam. Bull World Health Organ. 2012; 90(10): 764-72. [Unpublished data]

Honduras Vital Registration - Deaths 1981 ICD9 as it appears in World Health Organization (WHO). WHO Mortality Database Version September 2016. Geneva, Switzerland: World Health Organization (WHO), 2016

Honduras Vital Registration - Deaths 1982 ICD9 as it appears in World Health Organization (WHO). WHO Mortality Database Version September 2016. Geneva, Switzerland: World Health Organization (WHO), 2016

Honduras Vital Registration - Deaths 1987 ICD9 as it appears in World Health Organization (WHO). WHO Mortality Database Version September 2016. Geneva, Switzerland: World Health Organization (WHO), 2016

Honduras Vital Registration - Deaths 1988 ICD9 as it appears in World Health Organization (WHO). WHO Mortality Database Version September 2016. Geneva, Switzerland: World Health Organization (WHO), 2016

Honduras Vital Registration - Deaths 1990 ICD9 as it appears in World Health Organization (WHO). WHO Mortality Database Version September 2016. Geneva, Switzerland: World Health Organization (WHO), 2016

Hong Kong Vital Registration - Deaths 1980 ICD9 as it appears in World Health Organization (WHO). WHO Mortality Database Version September 2016. Geneva, Switzerland: World Health Organization (WHO), 2016
Hong Kong Vital Registration - Deaths 1981 ICD9 as it appears in World Health Organization (WHO). WHO Mortality Database Version September 2016. Geneva, Switzerland: World Health Organization (WHO), 2016
Hong Kong Vital Registration - Deaths 1982 ICD9 as it appears in World Health Organization (WHO). WHO Mortality Database Version September 2016. Geneva, Switzerland: World Health Organization (WHO), 2016
Hong Kong Vital Registration - Deaths 1983 ICD9 as it appears in World Health Organization (WHO). WHO Mortality Database Version September 2016. Geneva, Switzerland: World Health Organization (WHO), 2016
Hong Kong Vital Registration - Deaths 1984 ICD9 as it appears in World Health Organization (WHO). WHO Mortality Database Version September 2016. Geneva, Switzerland: World Health Organization (WHO), 2016
Hong Kong Vital Registration - Deaths 1985 ICD9 as it appears in World Health Organization (WHO). WHO Mortality Database Version September 2016. Geneva, Switzerland: World Health Organization (WHO), 2016
Hong Kong Vital Registration - Deaths 1986 ICD9 as it appears in World Health Organization (WHO). WHO Mortality Database Version September 2016. Geneva, Switzerland: World Health Organization (WHO), 2016
Hong Kong Vital Registration - Deaths 1987 ICD9 as it appears in World Health Organization (WHO). WHO Mortality Database Version September 2016. Geneva, Switzerland: World Health Organization (WHO), 2016
Hong Kong Vital Registration - Deaths 1988 ICD9 as it appears in World Health Organization (WHO). WHO Mortality Database Version September 2016. Geneva, Switzerland: World Health Organization (WHO), 2016
Hong Kong Vital Registration - Deaths 1989 ICD9 as it appears in World Health Organization (WHO). WHO Mortality Database Version September 2016. Geneva, Switzerland: World Health Organization (WHO), 2016
Hong Kong Vital Registration - Deaths 1990 ICD9 as it appears in World Health Organization (WHO). WHO Mortality Database Version September 2016. Geneva, Switzerland: World Health Organization (WHO), 2016
Hong Kong Vital Registration - Deaths 1991 ICD9 as it appears in World Health Organization (WHO). WHO Mortality Database Version September 2016. Geneva, Switzerland: World Health Organization (WHO), 2016
Hong Kong Vital Registration - Deaths 1992 ICD9 as it appears in World Health Organization (WHO). WHO Mortality Database Version September 2016. Geneva, Switzerland: World Health Organization (WHO), 2016
Hong Kong Vital Registration - Deaths 1993 ICD9 as it appears in World Health Organization (WHO). WHO Mortality Database Version September 2016. Geneva, Switzerland: World Health Organization (WHO), 2016
Hong Kong Vital Registration - Deaths 1994 ICD9 as it appears in World Health Organization (WHO). WHO Mortality Database Version November 2015. Geneva, Switzerland: World Health Organization (WHO), 2015
Hong Kong Vital Registration - Deaths 1995 ICD9 as it appears in World Health Organization (WHO). WHO Mortality Database Version November 2015. Geneva, Switzerland: World Health Organization (WHO), 2015
Hong Kong Vital Registration - Deaths 1996 ICD9 as it appears in World Health Organization (WHO). WHO Mortality Database Version November 2015. Geneva, Switzerland: World Health Organization (WHO), 2015
Hong Kong Vital Registration - Deaths 1997 ICD9 as it appears in World Health Organization (WHO). WHO Mortality Database Version November 2015. Geneva, Switzerland: World Health Organization (WHO), 2015
Hong Kong Vital Registration - Deaths 1998 ICD9 as it appears in World Health Organization (WHO). WHO Mortality Database Version November 2015. Geneva, Switzerland: World Health Organization (WHO), 2015
Hong Kong Vital Registration - Deaths 1999 ICD9 as it appears in World Health Organization (WHO). WHO Mortality Database Version November 2015. Geneva, Switzerland: World Health Organization (WHO), 2015

Hong Kong Vital Registration - Deaths 2000 ICD9 as it appears in World Health Organization (WHO). WHO Mortality Database Version November 2015. Geneva, Switzerland: World Health Organization (WHO), 2015
Hong Kong Vital Registration - Deaths 2001 ICD10 as it appears in World Health Organization (WHO). WHO Mortality Database Version October 2017. Geneva, Switzerland: World Health Organization (WHO), 2017
Hong Kong Vital Registration - Deaths 2002 ICD10 as it appears in World Health Organization (WHO). WHO Mortality Database Version October 2017. Geneva, Switzerland: World Health Organization (WHO), 2017
Hong Kong Vital Registration - Deaths 2003 ICD10 as it appears in World Health Organization (WHO). WHO Mortality Database Version October 2017. Geneva, Switzerland: World Health Organization (WHO), 2017
Hong Kong Vital Registration - Deaths 2004 ICD10 as it appears in World Health Organization (WHO). WHO Mortality Database Version October 2017. Geneva, Switzerland: World Health Organization (WHO), 2017
Hong Kong Vital Registration - Deaths 2005 ICD10 as it appears in World Health Organization (WHO). WHO Mortality Database Version October 2017. Geneva, Switzerland: World Health Organization (WHO), 2017
Hong Kong Vital Registration - Deaths 2006 ICD10 as it appears in World Health Organization (WHO). WHO Mortality Database Version October 2017. Geneva, Switzerland: World Health Organization (WHO), 2017
Hong Kong Vital Registration - Deaths 2007 ICD10 as it appears in World Health Organization (WHO). WHO Mortality Database Version October 2017. Geneva, Switzerland: World Health Organization (WHO), 2017
Hong Kong Vital Registration - Deaths 2008 ICD10 as it appears in World Health Organization (WHO). WHO Mortality Database Version October 2017. Geneva, Switzerland: World Health Organization (WHO), 2017
Hong Kong Vital Registration - Deaths 2009 ICD10 as it appears in World Health Organization (WHO). WHO Mortality Database Version October 2017. Geneva, Switzerland: World Health Organization (WHO), 2017
Hong Kong Vital Registration - Deaths 2010 ICD10 as it appears in World Health Organization (WHO). WHO Mortality Database Version October 2017. Geneva, Switzerland: World Health Organization (WHO), 2017
Hong Kong Vital Registration - Deaths 2011 ICD10 as it appears in World Health Organization (WHO). WHO Mortality Database Version October 2017. Geneva, Switzerland: World Health Organization (WHO), 2017
Hong Kong Vital Registration - Deaths 2012 ICD10 as it appears in World Health Organization (WHO). WHO Mortality Database Version October 2017. Geneva, Switzerland: World Health Organization (WHO), 2017
Hong Kong Vital Registration - Deaths 2013 ICD10 as it appears in World Health Organization (WHO). WHO Mortality Database Version October 2017. Geneva, Switzerland: World Health Organization (WHO), 2017
Hong Kong Vital Registration - Deaths 2014 ICD10 as it appears in World Health Organization (WHO). WHO Mortality Database Version April 2018. Geneva, Switzerland: World Health Organization (WHO), 2018
Hong Kong Vital Registration - Deaths 2015 ICD10 as it appears in World Health Organization (WHO). WHO Mortality Database Version April 2018. Geneva, Switzerland: World Health Organization (WHO), 2018
Hungary Vital Registration - Deaths 1980 ICD9 as it appears in World Health Organization (WHO). WHO Mortality Database Version November 2015. Geneva, Switzerland: World Health Organization (WHO), 2015
Hungary Vital Registration - Deaths 1981 ICD9 as it appears in World Health Organization (WHO). WHO Mortality Database Version November 2015. Geneva, Switzerland: World Health Organization (WHO), 2015
Hungary Vital Registration - Deaths 1982 ICD9 as it appears in World Health Organization (WHO). WHO Mortality Database Version November 2015. Geneva, Switzerland: World Health Organization (WHO), 2015
Hungary Vital Registration - Deaths 1983 ICD9 as it appears in World Health Organization (WHO). WHO Mortality Database Version November 2015. Geneva, Switzerland: World Health Organization (WHO), 2015

Hungary Vital Registration - Deaths 1984 ICD9 as it appears in World Health Organization (WHO). WHO Mortality Database Version November 2015. Geneva, Switzerland: World Health Organization (WHO), 2015
Hungary Vital Registration - Deaths 1985 ICD9 as it appears in World Health Organization (WHO). WHO Mortality Database Version November 2015. Geneva, Switzerland: World Health Organization (WHO), 2015
Hungary Vital Registration - Deaths 1986 ICD9 as it appears in World Health Organization (WHO). WHO Mortality Database Version November 2015. Geneva, Switzerland: World Health Organization (WHO), 2015
Hungary Vital Registration - Deaths 1987 ICD9 as it appears in World Health Organization (WHO). WHO Mortality Database Version November 2015. Geneva, Switzerland: World Health Organization (WHO), 2015
Hungary Vital Registration - Deaths 1988 ICD9 as it appears in World Health Organization (WHO). WHO Mortality Database Version November 2015. Geneva, Switzerland: World Health Organization (WHO), 2015
Hungary Vital Registration - Deaths 1989 ICD9 as it appears in World Health Organization (WHO). WHO Mortality Database Version November 2015. Geneva, Switzerland: World Health Organization (WHO), 2015
Hungary Vital Registration - Deaths 1990 ICD9 as it appears in World Health Organization (WHO). WHO Mortality Database Version November 2015. Geneva, Switzerland: World Health Organization (WHO), 2015
Hungary Vital Registration - Deaths 1991 ICD9 as it appears in World Health Organization (WHO). WHO Mortality Database Version November 2015. Geneva, Switzerland: World Health Organization (WHO), 2015
Hungary Vital Registration - Deaths 1992 ICD9 as it appears in World Health Organization (WHO). WHO Mortality Database Version November 2015. Geneva, Switzerland: World Health Organization (WHO), 2015
Hungary Vital Registration - Deaths 1993 ICD9 as it appears in World Health Organization (WHO). WHO Mortality Database Version November 2015. Geneva, Switzerland: World Health Organization (WHO), 2015
Hungary Vital Registration - Deaths 1994 ICD9 as it appears in World Health Organization (WHO). WHO Mortality Database Version November 2015. Geneva, Switzerland: World Health Organization (WHO), 2015
Hungary Vital Registration - Deaths 1995 ICD9 as it appears in World Health Organization (WHO). WHO Mortality Database Version November 2015. Geneva, Switzerland: World Health Organization (WHO), 2015
Hungary Vital Registration - Deaths 1996 ICD10 as it appears in World Health Organization (WHO). WHO Mortality Database Version October 2017. Geneva, Switzerland: World Health Organization (WHO), 2017
Hungary Vital Registration - Deaths 1997 ICD10 as it appears in World Health Organization (WHO). WHO Mortality Database Version October 2017. Geneva, Switzerland: World Health Organization (WHO), 2017
Hungary Vital Registration - Deaths 1998 ICD10 as it appears in World Health Organization (WHO). WHO Mortality Database Version October 2017. Geneva, Switzerland: World Health Organization (WHO), 2017
Hungary Vital Registration - Deaths 1999 ICD10 as it appears in World Health Organization (WHO). WHO Mortality Database Version October 2017. Geneva, Switzerland: World Health Organization (WHO), 2017
Hungary Vital Registration - Deaths 2000 ICD10 as it appears in World Health Organization (WHO). WHO Mortality Database Version October 2017. Geneva, Switzerland: World Health Organization (WHO), 2017
Hungary Vital Registration - Deaths 2001 ICD10 as it appears in World Health Organization (WHO). WHO Mortality Database Version October 2017. Geneva, Switzerland: World Health Organization (WHO), 2017
Hungary Vital Registration - Deaths 2002 ICD10 as it appears in World Health Organization (WHO). WHO Mortality Database Version October 2017. Geneva, Switzerland: World Health Organization (WHO), 2017
Hungary Vital Registration - Deaths 2003 ICD10 as it appears in World Health Organization (WHO). WHO Mortality Database Version October 2017. Geneva, Switzerland: World Health Organization (WHO), 2017

Hungary Vital Registration - Deaths 2004 ICD10 as it appears in World Health Organization (WHO). WHO Mortality Database Version October 2017. Geneva, Switzerland: World Health Organization (WHO), 2017
Hungary Vital Registration - Deaths 2005 ICD10 as it appears in World Health Organization (WHO). WHO Mortality Database Version October 2017. Geneva, Switzerland: World Health Organization (WHO), 2017
Hungary Vital Registration - Deaths 2006 ICD10 as it appears in World Health Organization (WHO). WHO Mortality Database Version October 2017. Geneva, Switzerland: World Health Organization (WHO), 2017
Hungary Vital Registration - Deaths 2007 ICD10 as it appears in World Health Organization (WHO). WHO Mortality Database Version October 2017. Geneva, Switzerland: World Health Organization (WHO), 2017
Hungary Vital Registration - Deaths 2008 ICD10 as it appears in World Health Organization (WHO). WHO Mortality Database Version October 2017. Geneva, Switzerland: World Health Organization (WHO), 2017
Hungary Vital Registration - Deaths 2009 ICD10 as it appears in World Health Organization (WHO). WHO Mortality Database Version October 2017. Geneva, Switzerland: World Health Organization (WHO), 2017
Hungary Vital Registration - Deaths 2010 ICD10 as it appears in World Health Organization (WHO). WHO Mortality Database Version October 2017. Geneva, Switzerland: World Health Organization (WHO), 2017
Hungary Vital Registration - Deaths 2011 ICD10 as it appears in World Health Organization (WHO). WHO Mortality Database Version October 2017. Geneva, Switzerland: World Health Organization (WHO), 2017
Hungary Vital Registration - Deaths 2012 ICD10 as it appears in World Health Organization (WHO). WHO Mortality Database Version October 2017. Geneva, Switzerland: World Health Organization (WHO), 2017
Hungary Vital Registration - Deaths 2013 ICD10 as it appears in World Health Organization (WHO). WHO Mortality Database Version October 2017. Geneva, Switzerland: World Health Organization (WHO), 2017
Hungary Vital Registration - Deaths 2014 ICD10 as it appears in World Health Organization (WHO). WHO Mortality Database Version October 2017. Geneva, Switzerland: World Health Organization (WHO), 2017
Hungary Vital Registration - Deaths 2015 ICD10 as it appears in World Health Organization (WHO). WHO Mortality Database Version March 2017. Geneva, Switzerland: World Health Organization (WHO), 2017
Hungary Vital Registration - Deaths 2016 ICD10 as it appears in World Health Organization (WHO). WHO Mortality Database Version April 2018. Geneva, Switzerland: World Health Organization (WHO), 2018
Iceland Vital Registration - Deaths 1980 ICD8 as it appears in World Health Organization (WHO). WHO Mortality Database Version July 2012. Geneva, Switzerland: World Health Organization (WHO), 2012
Iceland Vital Registration - Deaths 1981 ICD9 as it appears in World Health Organization (WHO). WHO Mortality Database Version September 2016. Geneva, Switzerland: World Health Organization (WHO), 2016
Iceland Vital Registration - Deaths 1982 ICD9 as it appears in World Health Organization (WHO). WHO Mortality Database Version September 2016. Geneva, Switzerland: World Health Organization (WHO), 2016
Iceland Vital Registration - Deaths 1983 ICD9 as it appears in World Health Organization (WHO). WHO Mortality Database Version September 2016. Geneva, Switzerland: World Health Organization (WHO), 2016
Iceland Vital Registration - Deaths 1984 ICD9 as it appears in World Health Organization (WHO). WHO Mortality Database Version September 2016. Geneva, Switzerland: World Health Organization (WHO), 2016
Iceland Vital Registration - Deaths 1985 ICD9 as it appears in World Health Organization (WHO). WHO Mortality Database Version September 2016. Geneva, Switzerland: World Health Organization (WHO), 2016
Iceland Vital Registration - Deaths 1986 ICD9 as it appears in World Health Organization (WHO). WHO Mortality Database Version September 2016. Geneva, Switzerland: World Health Organization (WHO), 2016

Iceland Vital Registration - Deaths 1987 ICD9 as it appears in World Health Organization (WHO). WHO Mortality Database Version September 2016. Geneva, Switzerland: World Health Organization (WHO), 2016
Iceland Vital Registration - Deaths 1988 ICD9 as it appears in World Health Organization (WHO). WHO Mortality Database Version September 2016. Geneva, Switzerland: World Health Organization (WHO), 2016
Iceland Vital Registration - Deaths 1989 ICD9 as it appears in World Health Organization (WHO). WHO Mortality Database Version November 2015. Geneva, Switzerland: World Health Organization (WHO), 2015
Iceland Vital Registration - Deaths 1990 ICD9 as it appears in World Health Organization (WHO). WHO Mortality Database Version November 2015. Geneva, Switzerland: World Health Organization (WHO), 2015
Iceland Vital Registration - Deaths 1991 ICD9 as it appears in World Health Organization (WHO). WHO Mortality Database Version November 2015. Geneva, Switzerland: World Health Organization (WHO), 2015
Iceland Vital Registration - Deaths 1992 ICD9 as it appears in World Health Organization (WHO). WHO Mortality Database Version November 2015. Geneva, Switzerland: World Health Organization (WHO), 2015
Iceland Vital Registration - Deaths 1993 ICD9 as it appears in World Health Organization (WHO). WHO Mortality Database Version November 2015. Geneva, Switzerland: World Health Organization (WHO), 2015
Iceland Vital Registration - Deaths 1994 ICD9 as it appears in World Health Organization (WHO). WHO Mortality Database Version November 2015. Geneva, Switzerland: World Health Organization (WHO), 2015
Iceland Vital Registration - Deaths 1995 ICD9 as it appears in World Health Organization (WHO). WHO Mortality Database Version November 2015. Geneva, Switzerland: World Health Organization (WHO), 2015
Iceland Vital Registration - Deaths 1996 ICD10 as it appears in World Health Organization (WHO). WHO Mortality Database Version October 2017. Geneva, Switzerland: World Health Organization (WHO), 2017
Iceland Vital Registration - Deaths 1997 ICD10 as it appears in World Health Organization (WHO). WHO Mortality Database Version October 2017. Geneva, Switzerland: World Health Organization (WHO), 2017
Iceland Vital Registration - Deaths 1998 ICD10 as it appears in World Health Organization (WHO). WHO Mortality Database Version October 2017. Geneva, Switzerland: World Health Organization (WHO), 2017
Iceland Vital Registration - Deaths 1999 ICD10 as it appears in World Health Organization (WHO). WHO Mortality Database Version October 2017. Geneva, Switzerland: World Health Organization (WHO), 2017
Iceland Vital Registration - Deaths 2000 ICD10 as it appears in World Health Organization (WHO). WHO Mortality Database Version October 2017. Geneva, Switzerland: World Health Organization (WHO), 2017
Iceland Vital Registration - Deaths 2001 ICD10 as it appears in World Health Organization (WHO). WHO Mortality Database Version October 2017. Geneva, Switzerland: World Health Organization (WHO), 2017
Iceland Vital Registration - Deaths 2002 ICD10 as it appears in World Health Organization (WHO). WHO Mortality Database Version October 2017. Geneva, Switzerland: World Health Organization (WHO), 2017
Iceland Vital Registration - Deaths 2003 ICD10 as it appears in World Health Organization (WHO). WHO Mortality Database Version October 2017. Geneva, Switzerland: World Health Organization (WHO), 2017
Iceland Vital Registration - Deaths 2004 ICD10 as it appears in World Health Organization (WHO). WHO Mortality Database Version October 2017. Geneva, Switzerland: World Health Organization (WHO), 2017
Iceland Vital Registration - Deaths 2005 ICD10 as it appears in World Health Organization (WHO). WHO Mortality Database Version October 2017. Geneva, Switzerland: World Health Organization (WHO), 2017
Iceland Vital Registration - Deaths 2006 ICD10 as it appears in World Health Organization (WHO). WHO Mortality Database Version October 2017. Geneva, Switzerland: World Health Organization (WHO), 2017

Iceland Vital Registration - Deaths 2007 ICD10 as it appears in World Health Organization (WHO). WHO Mortality Database Version October 2017. Geneva, Switzerland: World Health Organization (WHO), 2017
Iceland Vital Registration - Deaths 2008 ICD10 as it appears in World Health Organization (WHO). WHO Mortality Database Version October 2017. Geneva, Switzerland: World Health Organization (WHO), 2017
Iceland Vital Registration - Deaths 2009 ICD10 as it appears in World Health Organization (WHO). WHO Mortality Database Version October 2017. Geneva, Switzerland: World Health Organization (WHO), 2017
Iceland Vital Registration - Deaths 2010 ICD10 as it appears in World Health Organization (WHO). WHO Mortality Database Version March 2017. Geneva, Switzerland: World Health Organization (WHO), 2017
Iceland Vital Registration - Deaths 2011 ICD10 as it appears in World Health Organization (WHO). WHO Mortality Database Version March 2017. Geneva, Switzerland: World Health Organization (WHO), 2017
Iceland Vital Registration - Deaths 2012 ICD10 as it appears in World Health Organization (WHO). WHO Mortality Database Version March 2017. Geneva, Switzerland: World Health Organization (WHO), 2017
Iceland Vital Registration - Deaths 2013 ICD10 as it appears in World Health Organization (WHO). WHO Mortality Database Version March 2017. Geneva, Switzerland: World Health Organization (WHO), 2017
Iceland Vital Registration - Deaths 2014 ICD10 as it appears in World Health Organization (WHO). WHO Mortality Database Version March 2017. Geneva, Switzerland: World Health Organization (WHO), 2017
Iceland Vital Registration - Deaths 2015 ICD10 as it appears in World Health Organization (WHO). WHO Mortality Database Version March 2017. Geneva, Switzerland: World Health Organization (WHO), 2017
Iceland Vital Registration - Deaths 2016 ICD10 as it appears in World Health Organization (WHO). WHO Mortality Database Version October 2017. Geneva, Switzerland: World Health Organization (WHO), 2017
INDEPTH, International Centre for Diarrhoeal Disease Research, Bangladesh (ICDDR,B). Bangladesh - Matlab Health and Demographic Surveillance System
INDEPTH, Nouna Health Research Center (CRSN)(Burkina Faso). Burkina Faso - Nouna Health and Demographic Surveillance System
Indian Council of Medical Research (ICMR). India Study on Causes of Death by Verbal Autopsy 2003
International Centre for Diarrhoeal Disease Research, Bangladesh (ICDDR,B). Bangladesh - Chandpur and Comilla District Verbal Autopsy Study 2011-2014
Iraq Vital Registration - Deaths 2008 ICD10 as it appears in World Health Organization (WHO). WHO Mortality Database Version October 2017. Geneva, Switzerland: World Health Organization (WHO), 2017
Ireland Vital Registration - Deaths 1980 ICD9 as it appears in World Health Organization (WHO). WHO Mortality Database Version November 2015. Geneva, Switzerland: World Health Organization (WHO), 2015
Ireland Vital Registration - Deaths 1981 ICD9 as it appears in World Health Organization (WHO). WHO Mortality Database Version November 2015. Geneva, Switzerland: World Health Organization (WHO), 2015
Ireland Vital Registration - Deaths 1982 ICD9 as it appears in World Health Organization (WHO). WHO Mortality Database Version November 2015. Geneva, Switzerland: World Health Organization (WHO), 2015
Ireland Vital Registration - Deaths 1983 ICD9 as it appears in World Health Organization (WHO). WHO Mortality Database Version November 2015. Geneva, Switzerland: World Health Organization (WHO), 2015
Ireland Vital Registration - Deaths 1984 ICD9 as it appears in World Health Organization (WHO). WHO Mortality Database Version November 2015. Geneva, Switzerland: World Health Organization (WHO), 2015
Ireland Vital Registration - Deaths 1985 ICD9 as it appears in World Health Organization (WHO). WHO Mortality Database Version November 2015. Geneva, Switzerland: World Health Organization (WHO), 2015
Ireland Vital Registration - Deaths 1986 ICD9 as it appears in World Health Organization (WHO). WHO Mortality Database Version November 2015. Geneva, Switzerland: World Health Organization (WHO), 2015

Ireland Vital Registration - Deaths 1987 ICD9 as it appears in World Health Organization (WHO). WHO Mortality Database Version November 2015. Geneva, Switzerland: World Health Organization (WHO), 2015
Ireland Vital Registration - Deaths 1988 ICD9 as it appears in World Health Organization (WHO). WHO Mortality Database Version November 2015. Geneva, Switzerland: World Health Organization (WHO), 2015
Ireland Vital Registration - Deaths 1989 ICD9 as it appears in World Health Organization (WHO). WHO Mortality Database Version November 2015. Geneva, Switzerland: World Health Organization (WHO), 2015
Ireland Vital Registration - Deaths 1990 ICD9 as it appears in World Health Organization (WHO). WHO Mortality Database Version November 2015. Geneva, Switzerland: World Health Organization (WHO), 2015
Ireland Vital Registration - Deaths 1991 ICD9 as it appears in World Health Organization (WHO). WHO Mortality Database Version November 2015. Geneva, Switzerland: World Health Organization (WHO), 2015
Ireland Vital Registration - Deaths 1992 ICD9 as it appears in World Health Organization (WHO). WHO Mortality Database Version November 2015. Geneva, Switzerland: World Health Organization (WHO), 2015
Ireland Vital Registration - Deaths 1993 ICD9 as it appears in World Health Organization (WHO). WHO Mortality Database Version November 2015. Geneva, Switzerland: World Health Organization (WHO), 2015
Ireland Vital Registration - Deaths 1994 ICD9 as it appears in World Health Organization (WHO). WHO Mortality Database Version November 2015. Geneva, Switzerland: World Health Organization (WHO), 2015
Ireland Vital Registration - Deaths 1995 ICD9 as it appears in World Health Organization (WHO). WHO Mortality Database Version November 2015. Geneva, Switzerland: World Health Organization (WHO), 2015
Ireland Vital Registration - Deaths 1996 ICD9 as it appears in World Health Organization (WHO). WHO Mortality Database Version November 2015. Geneva, Switzerland: World Health Organization (WHO), 2015
Ireland Vital Registration - Deaths 1997 ICD9 as it appears in World Health Organization (WHO). WHO Mortality Database Version November 2015. Geneva, Switzerland: World Health Organization (WHO), 2015
Ireland Vital Registration - Deaths 1998 ICD9 as it appears in World Health Organization (WHO). WHO Mortality Database Version November 2015. Geneva, Switzerland: World Health Organization (WHO), 2015
Ireland Vital Registration - Deaths 1999 ICD9 as it appears in World Health Organization (WHO). WHO Mortality Database Version November 2015. Geneva, Switzerland: World Health Organization (WHO), 2015
Ireland Vital Registration - Deaths 2000 ICD9 as it appears in World Health Organization (WHO). WHO Mortality Database Version November 2015. Geneva, Switzerland: World Health Organization (WHO), 2015
Ireland Vital Registration - Deaths 2001 ICD9 as it appears in World Health Organization (WHO). WHO Mortality Database Version November 2015. Geneva, Switzerland: World Health Organization (WHO), 2015
Ireland Vital Registration - Deaths 2002 ICD9 as it appears in World Health Organization (WHO). WHO Mortality Database Version November 2015. Geneva, Switzerland: World Health Organization (WHO), 2015
Ireland Vital Registration - Deaths 2003 ICD9 as it appears in World Health Organization (WHO). WHO Mortality Database Version November 2015. Geneva, Switzerland: World Health Organization (WHO), 2015
Ireland Vital Registration - Deaths 2004 ICD9 as it appears in World Health Organization (WHO). WHO Mortality Database Version November 2015. Geneva, Switzerland: World Health Organization (WHO), 2015
Ireland Vital Registration - Deaths 2005 ICD9 as it appears in World Health Organization (WHO). WHO Mortality Database Version November 2015. Geneva, Switzerland: World Health Organization (WHO), 2015
Ireland Vital Registration - Deaths 2006 ICD9 as it appears in World Health Organization (WHO). WHO Mortality Database Version September 2016. Geneva, Switzerland: World Health Organization (WHO), 2016

Ireland Vital Registration - Deaths 2007 ICD10 as it appears in World Health Organization (WHO). WHO Mortality Database Version October 2017. Geneva, Switzerland: World Health Organization (WHO), 2017
Ireland Vital Registration - Deaths 2008 ICD10 as it appears in World Health Organization (WHO). WHO Mortality Database Version October 2017. Geneva, Switzerland: World Health Organization (WHO), 2017
Ireland Vital Registration - Deaths 2009 ICD10 as it appears in World Health Organization (WHO). WHO Mortality Database Version October 2017. Geneva, Switzerland: World Health Organization (WHO), 2017
Ireland Vital Registration - Deaths 2010 ICD10 as it appears in World Health Organization (WHO). WHO Mortality Database Version October 2017. Geneva, Switzerland: World Health Organization (WHO), 2017
Ireland Vital Registration - Deaths 2011 ICD10 as it appears in World Health Organization (WHO). WHO Mortality Database Version October 2017. Geneva, Switzerland: World Health Organization (WHO), 2017
Ireland Vital Registration - Deaths 2012 ICD10 as it appears in World Health Organization (WHO). WHO Mortality Database Version October 2017. Geneva, Switzerland: World Health Organization (WHO), 2017
Ireland Vital Registration - Deaths 2013 ICD10 as it appears in World Health Organization (WHO). WHO Mortality Database Version October 2017. Geneva, Switzerland: World Health Organization (WHO), 2017
Ireland Vital Registration - Deaths 2014 ICD10 as it appears in World Health Organization (WHO). WHO Mortality Database Version October 2017. Geneva, Switzerland: World Health Organization (WHO), 2017
Israel Vital Registration - Deaths 1980 ICD9 as it appears in World Health Organization (WHO). WHO Mortality Database Version September 2016. Geneva, Switzerland: World Health Organization (WHO), 2016
Israel Vital Registration - Deaths 1981 ICD9 as it appears in World Health Organization (WHO). WHO Mortality Database Version September 2016. Geneva, Switzerland: World Health Organization (WHO), 2016
Israel Vital Registration - Deaths 1982 ICD9 as it appears in World Health Organization (WHO). WHO Mortality Database Version September 2016. Geneva, Switzerland: World Health Organization (WHO), 2016
Israel Vital Registration - Deaths 1983 ICD9 as it appears in World Health Organization (WHO). WHO Mortality Database Version September 2016. Geneva, Switzerland: World Health Organization (WHO), 2016
Israel Vital Registration - Deaths 1984 ICD9 as it appears in World Health Organization (WHO). WHO Mortality Database Version September 2016. Geneva, Switzerland: World Health Organization (WHO), 2016
Israel Vital Registration - Deaths 1985 ICD9 as it appears in World Health Organization (WHO). WHO Mortality Database Version September 2016. Geneva, Switzerland: World Health Organization (WHO), 2016
Israel Vital Registration - Deaths 1986 ICD9 as it appears in World Health Organization (WHO). WHO Mortality Database Version September 2016. Geneva, Switzerland: World Health Organization (WHO), 2016
Israel Vital Registration - Deaths 1987 ICD9 as it appears in World Health Organization (WHO). WHO Mortality Database Version September 2016. Geneva, Switzerland: World Health Organization (WHO), 2016
Israel Vital Registration - Deaths 1988 ICD9 as it appears in World Health Organization (WHO). WHO Mortality Database Version September 2016. Geneva, Switzerland: World Health Organization (WHO), 2016
Israel Vital Registration - Deaths 1989 ICD9 as it appears in World Health Organization (WHO). WHO Mortality Database Version September 2016. Geneva, Switzerland: World Health Organization (WHO), 2016
Israel Vital Registration - Deaths 1990 ICD9 as it appears in World Health Organization (WHO). WHO Mortality Database Version September 2016. Geneva, Switzerland: World Health Organization (WHO), 2016
Israel Vital Registration - Deaths 1991 ICD9 as it appears in World Health Organization (WHO). WHO Mortality Database Version September 2016. Geneva, Switzerland: World Health Organization (WHO), 2016

Israel Vital Registration - Deaths 1992 ICD9 as it appears in World Health Organization (WHO). WHO Mortality Database Version September 2016. Geneva, Switzerland: World Health Organization (WHO), 2016
Israel Vital Registration - Deaths 1993 ICD9 as it appears in World Health Organization (WHO). WHO Mortality Database Version September 2016. Geneva, Switzerland: World Health Organization (WHO), 2016
Israel Vital Registration - Deaths 1994 ICD9 as it appears in World Health Organization (WHO). WHO Mortality Database Version September 2016. Geneva, Switzerland: World Health Organization (WHO), 2016
Israel Vital Registration - Deaths 1995 ICD9 as it appears in World Health Organization (WHO). WHO Mortality Database Version November 2015. Geneva, Switzerland: World Health Organization (WHO), 2015
Israel Vital Registration - Deaths 1996 ICD9 as it appears in World Health Organization (WHO). WHO Mortality Database Version November 2015. Geneva, Switzerland: World Health Organization (WHO), 2015
Israel Vital Registration - Deaths 1997 ICD9 as it appears in World Health Organization (WHO). WHO Mortality Database Version November 2015. Geneva, Switzerland: World Health Organization (WHO), 2015
Israel Vital Registration - Deaths 1998 ICD10 as it appears in World Health Organization (WHO). WHO Mortality Database Version October 2017. Geneva, Switzerland: World Health Organization (WHO), 2017
Israel Vital Registration - Deaths 1999 ICD10 as it appears in World Health Organization (WHO). WHO Mortality Database Version October 2017. Geneva, Switzerland: World Health Organization (WHO), 2017
Israel Vital Registration - Deaths 2000 ICD10 as it appears in World Health Organization (WHO). WHO Mortality Database Version October 2017. Geneva, Switzerland: World Health Organization (WHO), 2017
Israel Vital Registration - Deaths 2001 ICD10 as it appears in World Health Organization (WHO). WHO Mortality Database Version October 2017. Geneva, Switzerland: World Health Organization (WHO), 2017
Israel Vital Registration - Deaths 2002 ICD10 as it appears in World Health Organization (WHO). WHO Mortality Database Version October 2017. Geneva, Switzerland: World Health Organization (WHO), 2017
Israel Vital Registration - Deaths 2003 ICD10 as it appears in World Health Organization (WHO). WHO Mortality Database Version October 2017. Geneva, Switzerland: World Health Organization (WHO), 2017
Israel Vital Registration - Deaths 2004 ICD10 as it appears in World Health Organization (WHO). WHO Mortality Database Version October 2017. Geneva, Switzerland: World Health Organization (WHO), 2017
Israel Vital Registration - Deaths 2005 ICD10 as it appears in World Health Organization (WHO). WHO Mortality Database Version October 2017. Geneva, Switzerland: World Health Organization (WHO), 2017
Israel Vital Registration - Deaths 2006 ICD10 as it appears in World Health Organization (WHO). WHO Mortality Database Version October 2017. Geneva, Switzerland: World Health Organization (WHO), 2017
Israel Vital Registration - Deaths 2007 ICD10 as it appears in World Health Organization (WHO). WHO Mortality Database Version October 2017. Geneva, Switzerland: World Health Organization (WHO), 2017
Israel Vital Registration - Deaths 2008 ICD10 as it appears in World Health Organization (WHO). WHO Mortality Database Version October 2017. Geneva, Switzerland: World Health Organization (WHO), 2017
Israel Vital Registration - Deaths 2009 ICD10 as it appears in World Health Organization (WHO). WHO Mortality Database Version October 2017. Geneva, Switzerland: World Health Organization (WHO), 2017
Israel Vital Registration - Deaths 2010 ICD10 as it appears in World Health Organization (WHO). WHO Mortality Database Version October 2017. Geneva, Switzerland: World Health Organization (WHO), 2017
Israel Vital Registration - Deaths 2011 ICD10 as it appears in World Health Organization (WHO). WHO Mortality Database Version October 2017. Geneva, Switzerland: World Health Organization (WHO), 2017
Israel Vital Registration - Deaths 2012 ICD10 as it appears in World Health Organization (WHO). WHO Mortality Database Version October 2017. Geneva, Switzerland:

World Health Organization (WHO), 2017
Israel Vital Registration - Deaths 2013 ICD10 as it appears in World Health Organization (WHO). WHO Mortality Database Version October 2017. Geneva, Switzerland: World Health Organization (WHO), 2017
Israel Vital Registration - Deaths 2014 ICD10 as it appears in World Health Organization (WHO). WHO Mortality Database Version March 2017. Geneva, Switzerland: World Health Organization (WHO), 2017
Israel Vital Registration - Deaths 2015 ICD10 as it appears in World Health Organization (WHO). WHO Mortality Database Version April 2018. Geneva, Switzerland: World Health Organization (WHO), 2018
Italy Vital Registration - Deaths 1980 ICD9 as it appears in World Health Organization (WHO). WHO Mortality Database Version November 2015. Geneva, Switzerland: World Health Organization (WHO), 2015
Italy Vital Registration - Deaths 1981 ICD9 as it appears in World Health Organization (WHO). WHO Mortality Database Version November 2015. Geneva, Switzerland: World Health Organization (WHO), 2015
Italy Vital Registration - Deaths 1982 ICD9 as it appears in World Health Organization (WHO). WHO Mortality Database Version November 2015. Geneva, Switzerland: World Health Organization (WHO), 2015
Italy Vital Registration - Deaths 1983 ICD9 as it appears in World Health Organization (WHO). WHO Mortality Database Version November 2015. Geneva, Switzerland: World Health Organization (WHO), 2015
Italy Vital Registration - Deaths 1984 ICD9 as it appears in World Health Organization (WHO). WHO Mortality Database Version November 2015. Geneva, Switzerland: World Health Organization (WHO), 2015
Italy Vital Registration - Deaths 1985 ICD9 as it appears in World Health Organization (WHO). WHO Mortality Database Version November 2015. Geneva, Switzerland: World Health Organization (WHO), 2015
Italy Vital Registration - Deaths 1986 ICD9 as it appears in World Health Organization (WHO). WHO Mortality Database Version November 2015. Geneva, Switzerland: World Health Organization (WHO), 2015
Italy Vital Registration - Deaths 1987 ICD9 as it appears in World Health Organization (WHO). WHO Mortality Database Version November 2015. Geneva, Switzerland: World Health Organization (WHO), 2015
Italy Vital Registration - Deaths 1988 ICD9 as it appears in World Health Organization (WHO). WHO Mortality Database Version November 2015. Geneva, Switzerland: World Health Organization (WHO), 2015
Italy Vital Registration - Deaths 1989 ICD9 as it appears in World Health Organization (WHO). WHO Mortality Database Version November 2015. Geneva, Switzerland: World Health Organization (WHO), 2015
Italy Vital Registration - Deaths 1990 ICD9 as it appears in World Health Organization (WHO). WHO Mortality Database Version November 2015. Geneva, Switzerland: World Health Organization (WHO), 2015
Italy Vital Registration - Deaths 1991 ICD9 as it appears in World Health Organization (WHO). WHO Mortality Database Version November 2015. Geneva, Switzerland: World Health Organization (WHO), 2015
Italy Vital Registration - Deaths 1992 ICD9 as it appears in World Health Organization (WHO). WHO Mortality Database Version November 2015. Geneva, Switzerland: World Health Organization (WHO), 2015
Italy Vital Registration - Deaths 1993 ICD9 as it appears in World Health Organization (WHO). WHO Mortality Database Version November 2015. Geneva, Switzerland: World Health Organization (WHO), 2015
Italy Vital Registration - Deaths 1994 ICD9 as it appears in World Health Organization (WHO). WHO Mortality Database Version November 2015. Geneva, Switzerland: World Health Organization (WHO), 2015
Italy Vital Registration - Deaths 1995 ICD9 as it appears in World Health Organization (WHO). WHO Mortality Database Version November 2015. Geneva, Switzerland: World Health Organization (WHO), 2015
Italy Vital Registration - Deaths 1996 ICD9 as it appears in World Health Organization (WHO). WHO Mortality Database Version November 2015. Geneva, Switzerland: World Health Organization (WHO), 2015

Italy Vital Registration - Deaths 1997 ICD9 as it appears in World Health Organization (WHO). WHO Mortality Database Version November 2015. Geneva, Switzerland: World Health Organization (WHO), 2015
Italy Vital Registration - Deaths 1998 ICD9 as it appears in World Health Organization (WHO). WHO Mortality Database Version November 2015. Geneva, Switzerland: World Health Organization (WHO), 2015
Italy Vital Registration - Deaths 1999 ICD9 as it appears in World Health Organization (WHO). WHO Mortality Database Version November 2015. Geneva, Switzerland: World Health Organization (WHO), 2015
Italy Vital Registration - Deaths 2000 ICD9 as it appears in World Health Organization (WHO). WHO Mortality Database Version November 2015. Geneva, Switzerland: World Health Organization (WHO), 2015
Italy Vital Registration - Deaths 2001 ICD9 as it appears in World Health Organization (WHO). WHO Mortality Database Version November 2015. Geneva, Switzerland: World Health Organization (WHO), 2015
Italy Vital Registration - Deaths 2002 ICD9 as it appears in World Health Organization (WHO). WHO Mortality Database Version November 2015. Geneva, Switzerland: World Health Organization (WHO), 2015
Italy Vital Registration - Deaths 2003 ICD10 as it appears in World Health Organization (WHO). WHO Mortality Database Version October 2017. Geneva, Switzerland: World Health Organization (WHO), 2017
Italy Vital Registration - Deaths 2006 ICD10 as it appears in World Health Organization (WHO). WHO Mortality Database Version October 2017. Geneva, Switzerland: World Health Organization (WHO), 2017
Italy Vital Registration - Deaths 2007 ICD10 as it appears in World Health Organization (WHO). WHO Mortality Database Version October 2017. Geneva, Switzerland: World Health Organization (WHO), 2017
Italy Vital Registration - Deaths 2004 ICD10 as it appears in World Health Organization (WHO). WHO Mortality Database Version October 2017. Geneva, Switzerland: World Health Organization (WHO), 2017
Italy Vital Registration - Deaths 2005 ICD10 as it appears in World Health Organization (WHO). WHO Mortality Database Version October 2017. Geneva, Switzerland: World Health Organization (WHO), 2017
Italy Vital Registration - Deaths 2008 ICD10 as it appears in World Health Organization (WHO). WHO Mortality Database Version October 2017. Geneva, Switzerland: World Health Organization (WHO), 2017
Italy Vital Registration - Deaths 2009 ICD10 as it appears in World Health Organization (WHO). WHO Mortality Database Version October 2017. Geneva, Switzerland: World Health Organization (WHO), 2017
Italy Vital Registration - Deaths 2010 ICD10 as it appears in World Health Organization (WHO). WHO Mortality Database Version October 2017. Geneva, Switzerland: World Health Organization (WHO), 2017
Italy Vital Registration - Deaths 2011 ICD10 as it appears in World Health Organization (WHO). WHO Mortality Database Version October 2017. Geneva, Switzerland: World Health Organization (WHO), 2017
Italy Vital Registration - Deaths 2012 ICD10 as it appears in World Health Organization (WHO). WHO Mortality Database Version October 2017. Geneva, Switzerland: World Health Organization (WHO), 2017
Italy Vital Registration - Deaths 2013 ICD10 as it appears in World Health Organization (WHO). WHO Mortality Database Version October 2017. Geneva, Switzerland: World Health Organization (WHO), 2017
Italy Vital Registration - Deaths 2014 ICD10 as it appears in World Health Organization (WHO). WHO Mortality Database Version October 2017. Geneva, Switzerland: World Health Organization (WHO), 2017
Jamaica Vital Registration - Deaths 1980 ICD9 as it appears in World Health Organization (WHO). WHO Mortality Database Version September 2016. Geneva, Switzerland: World Health Organization (WHO), 2016
Jamaica Vital Registration - Deaths 1981 ICD9 as it appears in World Health Organization (WHO). WHO Mortality Database Version September 2016. Geneva, Switzerland: World Health Organization (WHO), 2016

Jamaica Vital Registration - Deaths 1982 ICD9 as it appears in World Health Organization (WHO). WHO Mortality Database Version September 2016. Geneva, Switzerland: World Health Organization (WHO), 2016

Jamaica Vital Registration - Deaths 1983 ICD9 as it appears in World Health Organization (WHO). WHO Mortality Database Version September 2016. Geneva, Switzerland: World Health Organization (WHO), 2016

Jamaica Vital Registration - Deaths 1984 ICD9 as it appears in World Health Organization (WHO). WHO Mortality Database Version September 2016. Geneva, Switzerland: World Health Organization (WHO), 2016

Jamaica Vital Registration - Deaths 1985 ICD9 as it appears in World Health Organization (WHO). WHO Mortality Database Version September 2016. Geneva, Switzerland: World Health Organization (WHO), 2016

Jamaica Vital Registration - Deaths 1986 ICD9 as it appears in World Health Organization (WHO). WHO Mortality Database Version September 2016. Geneva, Switzerland: World Health Organization (WHO), 2016

Jamaica Vital Registration - Deaths 1987 ICD9 as it appears in World Health Organization (WHO). WHO Mortality Database Version September 2016. Geneva, Switzerland: World Health Organization (WHO), 2016

Jamaica Vital Registration - Deaths 1988 ICD9 as it appears in World Health Organization (WHO). WHO Mortality Database Version September 2016. Geneva, Switzerland: World Health Organization (WHO), 2016

Jamaica Vital Registration - Deaths 1989 ICD9 as it appears in World Health Organization (WHO). WHO Mortality Database Version September 2016. Geneva, Switzerland: World Health Organization (WHO), 2016

Jamaica Vital Registration - Deaths 1990 ICD9 as it appears in World Health Organization (WHO). WHO Mortality Database Version September 2016. Geneva, Switzerland: World Health Organization (WHO), 2016

Jamaica Vital Registration - Deaths 1991 ICD9 as it appears in World Health Organization (WHO). WHO Mortality Database Version September 2016. Geneva, Switzerland: World Health Organization (WHO), 2016

Jamaica Vital Registration - Deaths 2000 ICD10 as it appears in World Health Organization (WHO). WHO Mortality Database Version October 2017. Geneva, Switzerland: World Health Organization (WHO), 2017

Jamaica Vital Registration - Deaths 2001 ICD10 as it appears in World Health Organization (WHO). WHO Mortality Database Version October 2017. Geneva, Switzerland: World Health Organization (WHO), 2017

Jamaica Vital Registration - Deaths 2002 ICD10 as it appears in World Health Organization (WHO). WHO Mortality Database Version October 2017. Geneva, Switzerland: World Health Organization (WHO), 2017

Jamaica Vital Registration - Deaths 2003 ICD10 as it appears in World Health Organization (WHO). WHO Mortality Database Version October 2017. Geneva, Switzerland: World Health Organization (WHO), 2017

Jamaica Vital Registration - Deaths 2004 ICD10 as it appears in World Health Organization (WHO). WHO Mortality Database Version October 2017. Geneva, Switzerland: World Health Organization (WHO), 2017

Jamaica Vital Registration - Deaths 2005 ICD10 as it appears in World Health Organization (WHO). WHO Mortality Database Version October 2017. Geneva, Switzerland: World Health Organization (WHO), 2017

Jamaica Vital Registration - Deaths 2006 ICD10 as it appears in World Health Organization (WHO). WHO Mortality Database Version October 2017. Geneva, Switzerland: World Health Organization (WHO), 2017

Jamaica Vital Registration - Deaths 2009 ICD10 as it appears in World Health Organization (WHO). WHO Mortality Database Version October 2017. Geneva, Switzerland: World Health Organization (WHO), 2017

Jamaica Vital Registration - Deaths 2010 ICD10 as it appears in World Health Organization (WHO). WHO Mortality Database Version October 2017. Geneva, Switzerland: World Health Organization (WHO), 2017

Jamaica Vital Registration - Deaths 2011 ICD10 as it appears in World Health Organization (WHO). WHO Mortality Database Version October 2017. Geneva, Switzerland: World Health Organization (WHO), 2017

Jordan Vital Registration - Deaths 2008 ICD10 as it appears in World Health Organization (WHO). WHO Mortality Database Version October 2017. Geneva, Switzerland:

World Health Organization (WHO), 2017

Jordan Vital Registration - Deaths 2009 ICD10 as it appears in World Health Organization (WHO). WHO Mortality Database Version October 2017. Geneva, Switzerland: World Health Organization (WHO), 2017

Jordan Vital Registration - Deaths 2010 ICD10 as it appears in World Health Organization (WHO). WHO Mortality Database Version October 2017. Geneva, Switzerland: World Health Organization (WHO), 2017

Jordan Vital Registration - Deaths 2011 ICD10 as it appears in World Health Organization (WHO). WHO Mortality Database Version October 2017. Geneva, Switzerland: World Health Organization (WHO), 2017

Jordan Vital Registration - Deaths 2012 ICD10 as it appears in World Health Organization (WHO). WHO Mortality Database Version March 2017. Geneva, Switzerland: World Health Organization (WHO), 2017

Joshi R, Cardona M, Iyengar S, Sukumar A, Raju CR, Raju KR, Raju K, Reddy KS, Lopez A, Neal B. Chronic diseases now a leading cause of death in rural India – mortality data from the Andhra Pradesh Rural Health Initiative. Int J Epidemiol. 2006; 35(6): 1522-9

Kazakhstan Vital Registration - Deaths 1981 ICD9 as it appears in World Health Organization (WHO). WHO Mortality Database Version September 2016. Geneva, Switzerland: World Health Organization (WHO), 2016

Kazakhstan Vital Registration - Deaths 1982 ICD9 as it appears in World Health Organization (WHO). WHO Mortality Database Version September 2016. Geneva, Switzerland: World Health Organization (WHO), 2016

Kazakhstan Vital Registration - Deaths 1985 ICD9 as it appears in World Health Organization (WHO). WHO Mortality Database Version September 2016. Geneva, Switzerland: World Health Organization (WHO), 2016

Kazakhstan Vital Registration - Deaths 1986 ICD9 as it appears in World Health Organization (WHO). WHO Mortality Database Version September 2016. Geneva, Switzerland: World Health Organization (WHO), 2016

Kazakhstan Vital Registration - Deaths 1987 ICD9 as it appears in World Health Organization (WHO). WHO Mortality Database Version September 2016. Geneva, Switzerland: World Health Organization (WHO), 2016

Kazakhstan Vital Registration - Deaths 1988 ICD9 as it appears in World Health Organization (WHO). WHO Mortality Database Version September 2016. Geneva, Switzerland: World Health Organization (WHO), 2016

Kazakhstan Vital Registration - Deaths 1989 ICD9 as it appears in World Health Organization (WHO). WHO Mortality Database Version September 2016. Geneva, Switzerland: World Health Organization (WHO), 2016

Kazakhstan Vital Registration - Deaths 1990 ICD9 as it appears in World Health Organization (WHO). WHO Mortality Database Version September 2016. Geneva, Switzerland: World Health Organization (WHO), 2016

Kazakhstan Vital Registration - Deaths 1991 ICD9 as it appears in World Health Organization (WHO). WHO Mortality Database Version September 2016. Geneva, Switzerland: World Health Organization (WHO), 2016

Kazakhstan Vital Registration - Deaths 1992 ICD9 as it appears in World Health Organization (WHO). WHO Mortality Database Version September 2016. Geneva, Switzerland: World Health Organization (WHO), 2016

Kazakhstan Vital Registration - Deaths 1993 ICD9 as it appears in World Health Organization (WHO). WHO Mortality Database Version September 2016. Geneva, Switzerland: World Health Organization (WHO), 2016

Kazakhstan Vital Registration - Deaths 1994 ICD9 as it appears in World Health Organization (WHO). WHO Mortality Database Version September 2016. Geneva, Switzerland: World Health Organization (WHO), 2016

Kazakhstan Vital Registration - Deaths 1995 ICD9 as it appears in World Health Organization (WHO). WHO Mortality Database Version September 2016. Geneva, Switzerland: World Health Organization (WHO), 2016

Kazakhstan Vital Registration - Deaths 1996 ICD9 as it appears in World Health Organization (WHO). WHO Mortality Database Version September 2016. Geneva, Switzerland: World Health Organization (WHO), 2016

Kazakhstan Vital Registration - Deaths 1997 ICD9 as it appears in World Health Organization (WHO). WHO Mortality Database Version September 2016. Geneva, Switzerland: World Health Organization (WHO), 2016

Kazakhstan Vital Registration - Deaths 1998 ICD9 as it appears in World Health Organization (WHO). WHO Mortality Database Version September 2016. Geneva, Switzerland: World Health Organization (WHO), 2016
Kazakhstan Vital Registration - Deaths 1999 ICD9 as it appears in World Health Organization (WHO). WHO Mortality Database Version September 2016. Geneva, Switzerland: World Health Organization (WHO), 2016
Kazakhstan Vital Registration - Deaths 2000 ICD9 as it appears in World Health Organization (WHO). WHO Mortality Database Version September 2016. Geneva, Switzerland: World Health Organization (WHO), 2016
Kazakhstan Vital Registration - Deaths 2001 ICD9 as it appears in World Health Organization (WHO). WHO Mortality Database Version September 2016. Geneva, Switzerland: World Health Organization (WHO), 2016
Kazakhstan Vital Registration - Deaths 2002 ICD9 as it appears in World Health Organization (WHO). WHO Mortality Database Version September 2016. Geneva, Switzerland: World Health Organization (WHO), 2016
Kazakhstan Vital Registration - Deaths 2003 ICD9 as it appears in World Health Organization (WHO). WHO Mortality Database Version September 2016. Geneva, Switzerland: World Health Organization (WHO), 2016
Kazakhstan Vital Registration - Deaths 2013 ICD10 as it appears in World Health Organization (WHO). WHO Mortality Database Version October 2017. Geneva, Switzerland: World Health Organization (WHO), 2017
Kazakhstan Vital Registration - Deaths 2014 ICD10 as it appears in World Health Organization (WHO). WHO Mortality Database Version October 2017. Geneva, Switzerland: World Health Organization (WHO), 2017
Kazakhstan Vital Registration - Deaths 2015 ICD10 as it appears in World Health Organization (WHO). WHO Mortality Database Version October 2017. Geneva, Switzerland: World Health Organization (WHO), 2017
Kuwait Vital Registration - Deaths 1995 ICD10 as it appears in World Health Organization (WHO). WHO Mortality Database Version October 2017. Geneva, Switzerland: World Health Organization (WHO), 2017
Kuwait Vital Registration - Deaths 1996 ICD10 as it appears in World Health Organization (WHO). WHO Mortality Database Version October 2017. Geneva, Switzerland: World Health Organization (WHO), 2017
Kuwait Vital Registration - Deaths 1997 ICD10 as it appears in World Health Organization (WHO). WHO Mortality Database Version October 2017. Geneva, Switzerland: World Health Organization (WHO), 2017
Kuwait Vital Registration - Deaths 1999 ICD10 as it appears in World Health Organization (WHO). WHO Mortality Database Version October 2017. Geneva, Switzerland: World Health Organization (WHO), 2017
Kuwait Vital Registration - Deaths 2000 ICD10 as it appears in World Health Organization (WHO). WHO Mortality Database Version October 2017. Geneva, Switzerland: World Health Organization (WHO), 2017
Kuwait Vital Registration - Deaths 2001 ICD10 as it appears in World Health Organization (WHO). WHO Mortality Database Version October 2017. Geneva, Switzerland: World Health Organization (WHO), 2017
Kuwait Vital Registration - Deaths 2002 ICD10 as it appears in World Health Organization (WHO). WHO Mortality Database Version October 2017. Geneva, Switzerland: World Health Organization (WHO), 2017
Kuwait Vital Registration - Deaths 2008 ICD10 as it appears in World Health Organization (WHO). WHO Mortality Database Version October 2017. Geneva, Switzerland: World Health Organization (WHO), 2017
Kuwait Vital Registration - Deaths 1998 ICD10 as it appears in World Health Organization (WHO). WHO Mortality Database Version October 2017. Geneva, Switzerland: World Health Organization (WHO), 2017
Kuwait Vital Registration - Deaths 2003 ICD10 as it appears in World Health Organization (WHO). WHO Mortality Database Version October 2017. Geneva, Switzerland: World Health Organization (WHO), 2017
Kuwait Vital Registration - Deaths 2004 ICD10 as it appears in World Health Organization (WHO). WHO Mortality Database Version October 2017. Geneva, Switzerland: World Health Organization (WHO), 2017
Kuwait Vital Registration - Deaths 2005 ICD10 as it appears in World Health Organization (WHO). WHO Mortality Database Version October 2017. Geneva, Switzerland:

World Health Organization (WHO), 2017
Kuwait Vital Registration - Deaths 2006 ICD10 as it appears in World Health Organization (WHO). WHO Mortality Database Version October 2017. Geneva, Switzerland: World Health Organization (WHO), 2017
Kuwait Vital Registration - Deaths 2007 ICD10 as it appears in World Health Organization (WHO). WHO Mortality Database Version October 2017. Geneva, Switzerland: World Health Organization (WHO), 2017
Kuwait Vital Registration - Deaths 2009 ICD10 as it appears in World Health Organization (WHO). WHO Mortality Database Version October 2017. Geneva, Switzerland: World Health Organization (WHO), 2017
Kuwait Vital Registration - Deaths 2010 ICD10 as it appears in World Health Organization (WHO). WHO Mortality Database Version October 2017. Geneva, Switzerland: World Health Organization (WHO), 2017
Kuwait Vital Registration - Deaths 2011 ICD10 as it appears in World Health Organization (WHO). WHO Mortality Database Version October 2017. Geneva, Switzerland: World Health Organization (WHO), 2017
Kuwait Vital Registration - Deaths 2012 ICD10 as it appears in World Health Organization (WHO). WHO Mortality Database Version October 2017. Geneva, Switzerland: World Health Organization (WHO), 2017
Kuwait Vital Registration - Deaths 2013 ICD10 as it appears in World Health Organization (WHO). WHO Mortality Database Version October 2017. Geneva, Switzerland: World Health Organization (WHO), 2017
Kuwait Vital Registration - Deaths 2014 ICD10 as it appears in World Health Organization (WHO). WHO Mortality Database Version October 2017. Geneva, Switzerland: World Health Organization (WHO), 2017
Kyrgyzstan Vital Registration - Deaths 1981 ICD9 as it appears in World Health Organization (WHO). WHO Mortality Database Version September 2016. Geneva, Switzerland: World Health Organization (WHO), 2016
Kyrgyzstan Vital Registration - Deaths 1982 ICD9 as it appears in World Health Organization (WHO). WHO Mortality Database Version September 2016. Geneva, Switzerland: World Health Organization (WHO), 2016
Kyrgyzstan Vital Registration - Deaths 1985 ICD9 as it appears in World Health Organization (WHO). WHO Mortality Database Version September 2016. Geneva, Switzerland: World Health Organization (WHO), 2016
Kyrgyzstan Vital Registration - Deaths 1986 ICD9 as it appears in World Health Organization (WHO). WHO Mortality Database Version September 2016. Geneva, Switzerland: World Health Organization (WHO), 2016
Kyrgyzstan Vital Registration - Deaths 1987 ICD9 as it appears in World Health Organization (WHO). WHO Mortality Database Version September 2016. Geneva, Switzerland: World Health Organization (WHO), 2016
Kyrgyzstan Vital Registration - Deaths 1988 ICD9 as it appears in World Health Organization (WHO). WHO Mortality Database Version September 2016. Geneva, Switzerland: World Health Organization (WHO), 2016
Kyrgyzstan Vital Registration - Deaths 1989 ICD9 as it appears in World Health Organization (WHO). WHO Mortality Database Version September 2016. Geneva, Switzerland: World Health Organization (WHO), 2016
Kyrgyzstan Vital Registration - Deaths 1990 ICD9 as it appears in World Health Organization (WHO). WHO Mortality Database Version September 2016. Geneva, Switzerland: World Health Organization (WHO), 2016
Kyrgyzstan Vital Registration - Deaths 1991 ICD9 as it appears in World Health Organization (WHO). WHO Mortality Database Version September 2016. Geneva, Switzerland: World Health Organization (WHO), 2016
Kyrgyzstan Vital Registration - Deaths 1992 ICD9 as it appears in World Health Organization (WHO). WHO Mortality Database Version September 2016. Geneva, Switzerland: World Health Organization (WHO), 2016
Kyrgyzstan Vital Registration - Deaths 1993 ICD9 as it appears in World Health Organization (WHO). WHO Mortality Database Version September 2016. Geneva, Switzerland: World Health Organization (WHO), 2016
Kyrgyzstan Vital Registration - Deaths 1994 ICD9 as it appears in World Health Organization (WHO). WHO Mortality Database Version September 2016. Geneva, Switzerland: World Health Organization (WHO), 2016

Kyrgyzstan Vital Registration - Deaths 1995 ICD9 as it appears in World Health Organization (WHO). WHO Mortality Database Version September 2016. Geneva, Switzerland: World Health Organization (WHO), 2016
Kyrgyzstan Vital Registration - Deaths 1996 ICD9 as it appears in World Health Organization (WHO). WHO Mortality Database Version September 2016. Geneva, Switzerland: World Health Organization (WHO), 2016
Kyrgyzstan Vital Registration - Deaths 1997 ICD9 as it appears in World Health Organization (WHO). WHO Mortality Database Version September 2016. Geneva, Switzerland: World Health Organization (WHO), 2016
Kyrgyzstan Vital Registration - Deaths 1998 ICD9 as it appears in World Health Organization (WHO). WHO Mortality Database Version September 2016. Geneva, Switzerland: World Health Organization (WHO), 2016
Kyrgyzstan Vital Registration - Deaths 1999 ICD9 as it appears in World Health Organization (WHO). WHO Mortality Database Version September 2016. Geneva, Switzerland: World Health Organization (WHO), 2016
Kyrgyzstan Vital Registration - Deaths 2000 ICD10 as it appears in World Health Organization (WHO). WHO Mortality Database Version October 2017. Geneva, Switzerland: World Health Organization (WHO), 2017
Kyrgyzstan Vital Registration - Deaths 2001 ICD10 as it appears in World Health Organization (WHO). WHO Mortality Database Version October 2017. Geneva, Switzerland: World Health Organization (WHO), 2017
Kyrgyzstan Vital Registration - Deaths 2002 ICD10 as it appears in World Health Organization (WHO). WHO Mortality Database Version October 2017. Geneva, Switzerland: World Health Organization (WHO), 2017
Kyrgyzstan Vital Registration - Deaths 2003 ICD10 as it appears in World Health Organization (WHO). WHO Mortality Database Version October 2017. Geneva, Switzerland: World Health Organization (WHO), 2017
Kyrgyzstan Vital Registration - Deaths 2004 ICD10 as it appears in World Health Organization (WHO). WHO Mortality Database Version October 2017. Geneva, Switzerland: World Health Organization (WHO), 2017
Kyrgyzstan Vital Registration - Deaths 2005 ICD10 as it appears in World Health Organization (WHO). WHO Mortality Database Version October 2017. Geneva, Switzerland: World Health Organization (WHO), 2017
Kyrgyzstan Vital Registration - Deaths 2006 ICD10 as it appears in World Health Organization (WHO). WHO Mortality Database Version October 2017. Geneva, Switzerland: World Health Organization (WHO), 2017
Kyrgyzstan Vital Registration - Deaths 2007 ICD10 as it appears in World Health Organization (WHO). WHO Mortality Database Version October 2017. Geneva, Switzerland: World Health Organization (WHO), 2017
Kyrgyzstan Vital Registration - Deaths 2008 ICD10 as it appears in World Health Organization (WHO). WHO Mortality Database Version October 2017. Geneva, Switzerland: World Health Organization (WHO), 2017
Kyrgyzstan Vital Registration - Deaths 2009 ICD10 as it appears in World Health Organization (WHO). WHO Mortality Database Version October 2017. Geneva, Switzerland: World Health Organization (WHO), 2017
Kyrgyzstan Vital Registration - Deaths 2010 ICD10 as it appears in World Health Organization (WHO). WHO Mortality Database Version October 2017. Geneva, Switzerland: World Health Organization (WHO), 2017
Kyrgyzstan Vital Registration - Deaths 2011 ICD10 as it appears in World Health Organization (WHO). WHO Mortality Database Version October 2017. Geneva, Switzerland: World Health Organization (WHO), 2017
Kyrgyzstan Vital Registration - Deaths 2012 ICD10 as it appears in World Health Organization (WHO). WHO Mortality Database Version October 2017. Geneva, Switzerland: World Health Organization (WHO), 2017
Kyrgyzstan Vital Registration - Deaths 2013 ICD10 as it appears in World Health Organization (WHO). WHO Mortality Database Version October 2017. Geneva, Switzerland: World Health Organization (WHO), 2017
Kyrgyzstan Vital Registration - Deaths 2014 ICD10 as it appears in World Health Organization (WHO). WHO Mortality Database Version March 2017. Geneva, Switzerland: World Health Organization (WHO), 2017

Kyrgyzstan Vital Registration - Deaths 2015 ICD10 as it appears in World Health Organization (WHO). WHO Mortality Database Version March 2017. Geneva, Switzerland: World Health Organization (WHO), 2017
Latvia Vital Registration - Deaths 1980 ICD9 as it appears in World Health Organization (WHO). WHO Mortality Database Version September 2016. Geneva, Switzerland: World Health Organization (WHO), 2016
Latvia Vital Registration - Deaths 1981 ICD9 as it appears in World Health Organization (WHO). WHO Mortality Database Version September 2016. Geneva, Switzerland: World Health Organization (WHO), 2016
Latvia Vital Registration - Deaths 1982 ICD9 as it appears in World Health Organization (WHO). WHO Mortality Database Version September 2016. Geneva, Switzerland: World Health Organization (WHO), 2016
Latvia Vital Registration - Deaths 1983 ICD9 as it appears in World Health Organization (WHO). WHO Mortality Database Version September 2016. Geneva, Switzerland: World Health Organization (WHO), 2016
Latvia Vital Registration - Deaths 1984 ICD9 as it appears in World Health Organization (WHO). WHO Mortality Database Version September 2016. Geneva, Switzerland: World Health Organization (WHO), 2016
Latvia Vital Registration - Deaths 1985 ICD9 as it appears in World Health Organization (WHO). WHO Mortality Database Version September 2016. Geneva, Switzerland: World Health Organization (WHO), 2016
Latvia Vital Registration - Deaths 1986 ICD9 as it appears in World Health Organization (WHO). WHO Mortality Database Version September 2016. Geneva, Switzerland: World Health Organization (WHO), 2016
Latvia Vital Registration - Deaths 1987 ICD9 as it appears in World Health Organization (WHO). WHO Mortality Database Version September 2016. Geneva, Switzerland: World Health Organization (WHO), 2016
Latvia Vital Registration - Deaths 1988 ICD9 as it appears in World Health Organization (WHO). WHO Mortality Database Version September 2016. Geneva, Switzerland: World Health Organization (WHO), 2016
Latvia Vital Registration - Deaths 1989 ICD9 as it appears in World Health Organization (WHO). WHO Mortality Database Version September 2016. Geneva, Switzerland: World Health Organization (WHO), 2016
Latvia Vital Registration - Deaths 1990 ICD9 as it appears in World Health Organization (WHO). WHO Mortality Database Version September 2016. Geneva, Switzerland: World Health Organization (WHO), 2016
Latvia Vital Registration - Deaths 1991 ICD9 as it appears in World Health Organization (WHO). WHO Mortality Database Version September 2016. Geneva, Switzerland: World Health Organization (WHO), 2016
Latvia Vital Registration - Deaths 1992 ICD9 as it appears in World Health Organization (WHO). WHO Mortality Database Version September 2016. Geneva, Switzerland: World Health Organization (WHO), 2016
Latvia Vital Registration - Deaths 1993 ICD9 as it appears in World Health Organization (WHO). WHO Mortality Database Version September 2016. Geneva, Switzerland: World Health Organization (WHO), 2016
Latvia Vital Registration - Deaths 1994 ICD9 as it appears in World Health Organization (WHO). WHO Mortality Database Version September 2016. Geneva, Switzerland: World Health Organization (WHO), 2016
Latvia Vital Registration - Deaths 1995 ICD9 as it appears in World Health Organization (WHO). WHO Mortality Database Version September 2016. Geneva, Switzerland: World Health Organization (WHO), 2016
Latvia Vital Registration - Deaths 1996 ICD10 as it appears in World Health Organization (WHO). WHO Mortality Database Version October 2017. Geneva, Switzerland: World Health Organization (WHO), 2017
Latvia Vital Registration - Deaths 1997 ICD10 as it appears in World Health Organization (WHO). WHO Mortality Database Version October 2017. Geneva, Switzerland: World Health Organization (WHO), 2017
Latvia Vital Registration - Deaths 1998 ICD10 as it appears in World Health Organization (WHO). WHO Mortality Database Version October 2017. Geneva, Switzerland: World Health Organization (WHO), 2017
Latvia Vital Registration - Deaths 1999 ICD10 as it appears in World Health Organization (WHO). WHO Mortality Database Version October 2017. Geneva, Switzerland:

World Health Organization (WHO), 2017
Latvia Vital Registration - Deaths 2000 ICD10 as it appears in World Health Organization (WHO). WHO Mortality Database Version October 2017. Geneva, Switzerland: World Health Organization (WHO), 2017
Latvia Vital Registration - Deaths 2001 ICD10 as it appears in World Health Organization (WHO). WHO Mortality Database Version October 2017. Geneva, Switzerland: World Health Organization (WHO), 2017
Latvia Vital Registration - Deaths 2002 ICD10 as it appears in World Health Organization (WHO). WHO Mortality Database Version October 2017. Geneva, Switzerland: World Health Organization (WHO), 2017
Latvia Vital Registration - Deaths 2003 ICD10 as it appears in World Health Organization (WHO). WHO Mortality Database Version October 2017. Geneva, Switzerland: World Health Organization (WHO), 2017
Latvia Vital Registration - Deaths 2004 ICD10 as it appears in World Health Organization (WHO). WHO Mortality Database Version October 2017. Geneva, Switzerland: World Health Organization (WHO), 2017
Latvia Vital Registration - Deaths 2005 ICD10 as it appears in World Health Organization (WHO). WHO Mortality Database Version October 2017. Geneva, Switzerland: World Health Organization (WHO), 2017
Latvia Vital Registration - Deaths 2006 ICD10 as it appears in World Health Organization (WHO). WHO Mortality Database Version October 2017. Geneva, Switzerland: World Health Organization (WHO), 2017
Latvia Vital Registration - Deaths 2007 ICD10 as it appears in World Health Organization (WHO). WHO Mortality Database Version October 2017. Geneva, Switzerland: World Health Organization (WHO), 2017
Latvia Vital Registration - Deaths 2008 ICD10 as it appears in World Health Organization (WHO). WHO Mortality Database Version October 2017. Geneva, Switzerland: World Health Organization (WHO), 2017
Latvia Vital Registration - Deaths 2009 ICD10 as it appears in World Health Organization (WHO). WHO Mortality Database Version October 2017. Geneva, Switzerland: World Health Organization (WHO), 2017
Latvia Vital Registration - Deaths 2010 ICD10 as it appears in World Health Organization (WHO). WHO Mortality Database Version October 2017. Geneva, Switzerland: World Health Organization (WHO), 2017
Latvia Vital Registration - Deaths 2011 ICD10 as it appears in World Health Organization (WHO). WHO Mortality Database Version October 2017. Geneva, Switzerland: World Health Organization (WHO), 2017
Latvia Vital Registration - Deaths 2012 ICD10 as it appears in World Health Organization (WHO). WHO Mortality Database Version October 2017. Geneva, Switzerland: World Health Organization (WHO), 2017
Latvia Vital Registration - Deaths 2013 ICD10 as it appears in World Health Organization (WHO). WHO Mortality Database Version October 2017. Geneva, Switzerland: World Health Organization (WHO), 2017
Latvia Vital Registration - Deaths 2014 ICD10 as it appears in World Health Organization (WHO). WHO Mortality Database Version October 2017. Geneva, Switzerland: World Health Organization (WHO), 2017
Latvia Vital Registration - Deaths 2015 ICD10 as it appears in World Health Organization (WHO). WHO Mortality Database Version October 2017. Geneva, Switzerland: World Health Organization (WHO), 2017
Lithuania Vital Registration - Deaths 1981 ICD9 as it appears in World Health Organization (WHO). WHO Mortality Database Version September 2016. Geneva, Switzerland: World Health Organization (WHO), 2016
Lithuania Vital Registration - Deaths 1982 ICD9 as it appears in World Health Organization (WHO). WHO Mortality Database Version September 2016. Geneva, Switzerland: World Health Organization (WHO), 2016
Lithuania Vital Registration - Deaths 1985 ICD9 as it appears in World Health Organization (WHO). WHO Mortality Database Version September 2016. Geneva, Switzerland: World Health Organization (WHO), 2016
Lithuania Vital Registration - Deaths 1986 ICD9 as it appears in World Health Organization (WHO). WHO Mortality Database Version September 2016. Geneva, Switzerland: World Health Organization (WHO), 2016

Lithuania Vital Registration - Deaths 1987 ICD9 as it appears in World Health Organization (WHO). WHO Mortality Database Version September 2016. Geneva, Switzerland: World Health Organization (WHO), 2016
Lithuania Vital Registration - Deaths 1988 ICD9 as it appears in World Health Organization (WHO). WHO Mortality Database Version September 2016. Geneva, Switzerland: World Health Organization (WHO), 2016
Lithuania Vital Registration - Deaths 1989 ICD9 as it appears in World Health Organization (WHO). WHO Mortality Database Version September 2016. Geneva, Switzerland: World Health Organization (WHO), 2016
Lithuania Vital Registration - Deaths 1990 ICD9 as it appears in World Health Organization (WHO). WHO Mortality Database Version September 2016. Geneva, Switzerland: World Health Organization (WHO), 2016
Lithuania Vital Registration - Deaths 1991 ICD9 as it appears in World Health Organization (WHO). WHO Mortality Database Version September 2016. Geneva, Switzerland: World Health Organization (WHO), 2016
Lithuania Vital Registration - Deaths 1992 ICD9 as it appears in World Health Organization (WHO). WHO Mortality Database Version September 2016. Geneva, Switzerland: World Health Organization (WHO), 2016
Lithuania Vital Registration - Deaths 1993 ICD9 as it appears in World Health Organization (WHO). WHO Mortality Database Version September 2016. Geneva, Switzerland: World Health Organization (WHO), 2016
Lithuania Vital Registration - Deaths 1994 ICD9 as it appears in World Health Organization (WHO). WHO Mortality Database Version November 2015. Geneva, Switzerland: World Health Organization (WHO), 2015
Lithuania Vital Registration - Deaths 1995 ICD9 as it appears in World Health Organization (WHO). WHO Mortality Database Version November 2015. Geneva, Switzerland: World Health Organization (WHO), 2015
Lithuania Vital Registration - Deaths 1996 ICD9 as it appears in World Health Organization (WHO). WHO Mortality Database Version November 2015. Geneva, Switzerland: World Health Organization (WHO), 2015
Lithuania Vital Registration - Deaths 1998 ICD10 as it appears in World Health Organization (WHO). WHO Mortality Database Version October 2017. Geneva, Switzerland: World Health Organization (WHO), 2017
Lithuania Vital Registration - Deaths 1999 ICD10 as it appears in World Health Organization (WHO). WHO Mortality Database Version October 2017. Geneva, Switzerland: World Health Organization (WHO), 2017
Lithuania Vital Registration - Deaths 2000 ICD10 as it appears in World Health Organization (WHO). WHO Mortality Database Version October 2017. Geneva, Switzerland: World Health Organization (WHO), 2017
Lithuania Vital Registration - Deaths 2001 ICD10 as it appears in World Health Organization (WHO). WHO Mortality Database Version October 2017. Geneva, Switzerland: World Health Organization (WHO), 2017
Lithuania Vital Registration - Deaths 2002 ICD10 as it appears in World Health Organization (WHO). WHO Mortality Database Version October 2017. Geneva, Switzerland: World Health Organization (WHO), 2017
Lithuania Vital Registration - Deaths 2003 ICD10 as it appears in World Health Organization (WHO). WHO Mortality Database Version October 2017. Geneva, Switzerland: World Health Organization (WHO), 2017
Lithuania Vital Registration - Deaths 2004 ICD10 as it appears in World Health Organization (WHO). WHO Mortality Database Version October 2017. Geneva, Switzerland: World Health Organization (WHO), 2017
Lithuania Vital Registration - Deaths 2005 ICD10 as it appears in World Health Organization (WHO). WHO Mortality Database Version October 2017. Geneva, Switzerland: World Health Organization (WHO), 2017
Lithuania Vital Registration - Deaths 2006 ICD10 as it appears in World Health Organization (WHO). WHO Mortality Database Version October 2017. Geneva, Switzerland: World Health Organization (WHO), 2017
Lithuania Vital Registration - Deaths 2007 ICD10 as it appears in World Health Organization (WHO). WHO Mortality Database Version October 2017. Geneva, Switzerland: World Health Organization (WHO), 2017

Lithuania Vital Registration - Deaths 2008 ICD10 as it appears in World Health Organization (WHO). WHO Mortality Database Version October 2017. Geneva, Switzerland: World Health Organization (WHO), 2017
Lithuania Vital Registration - Deaths 1997 ICD9 as it appears in World Health Organization (WHO). WHO Mortality Database Version November 2015. Geneva, Switzerland: World Health Organization (WHO), 2015
Lithuania Vital Registration - Deaths 2009 ICD10 as it appears in World Health Organization (WHO). WHO Mortality Database Version October 2017. Geneva, Switzerland: World Health Organization (WHO), 2017
Lithuania Vital Registration - Deaths 2010 ICD10 as it appears in World Health Organization (WHO). WHO Mortality Database Version October 2017. Geneva, Switzerland: World Health Organization (WHO), 2017
Lithuania Vital Registration - Deaths 2011 ICD10 as it appears in World Health Organization (WHO). WHO Mortality Database Version October 2017. Geneva, Switzerland: World Health Organization (WHO), 2017
Lithuania Vital Registration - Deaths 2012 ICD10 as it appears in World Health Organization (WHO). WHO Mortality Database Version October 2017. Geneva, Switzerland: World Health Organization (WHO), 2017
Lithuania Vital Registration - Deaths 2013 ICD10 as it appears in World Health Organization (WHO). WHO Mortality Database Version October 2017. Geneva, Switzerland: World Health Organization (WHO), 2017
Lithuania Vital Registration - Deaths 2014 ICD10 as it appears in World Health Organization (WHO). WHO Mortality Database Version October 2017. Geneva, Switzerland: World Health Organization (WHO), 2017
Lithuania Vital Registration - Deaths 2015 ICD10 as it appears in World Health Organization (WHO). WHO Mortality Database Version March 2017. Geneva, Switzerland: World Health Organization (WHO), 2017
Lithuania Vital Registration - Deaths 2016 ICD10 as it appears in World Health Organization (WHO). WHO Mortality Database Version April 2018. Geneva, Switzerland: World Health Organization (WHO), 2018
Luxembourg Vital Registration - Deaths 1980 ICD9 as it appears in World Health Organization (WHO). WHO Mortality Database Version November 2015. Geneva, Switzerland: World Health Organization (WHO), 2015
Luxembourg Vital Registration - Deaths 1981 ICD9 as it appears in World Health Organization (WHO). WHO Mortality Database Version November 2015. Geneva, Switzerland: World Health Organization (WHO), 2015
Luxembourg Vital Registration - Deaths 1982 ICD9 as it appears in World Health Organization (WHO). WHO Mortality Database Version November 2015. Geneva, Switzerland: World Health Organization (WHO), 2015
Luxembourg Vital Registration - Deaths 1983 ICD9 as it appears in World Health Organization (WHO). WHO Mortality Database Version November 2015. Geneva, Switzerland: World Health Organization (WHO), 2015
Luxembourg Vital Registration - Deaths 1984 ICD9 as it appears in World Health Organization (WHO). WHO Mortality Database Version November 2015. Geneva, Switzerland: World Health Organization (WHO), 2015
Luxembourg Vital Registration - Deaths 1985 ICD9 as it appears in World Health Organization (WHO). WHO Mortality Database Version November 2015. Geneva, Switzerland: World Health Organization (WHO), 2015
Luxembourg Vital Registration - Deaths 1986 ICD9 as it appears in World Health Organization (WHO). WHO Mortality Database Version November 2015. Geneva, Switzerland: World Health Organization (WHO), 2015
Luxembourg Vital Registration - Deaths 1987 ICD9 as it appears in World Health Organization (WHO). WHO Mortality Database Version November 2015. Geneva, Switzerland: World Health Organization (WHO), 2015
Luxembourg Vital Registration - Deaths 1988 ICD9 as it appears in World Health Organization (WHO). WHO Mortality Database Version November 2015. Geneva, Switzerland: World Health Organization (WHO), 2015
Luxembourg Vital Registration - Deaths 1989 ICD9 as it appears in World Health Organization (WHO). WHO Mortality Database Version November 2015. Geneva, Switzerland: World Health Organization (WHO), 2015

Luxembourg Vital Registration - Deaths 1990 ICD9 as it appears in World Health Organization (WHO). WHO Mortality Database Version November 2015. Geneva, Switzerland: World Health Organization (WHO), 2015
Luxembourg Vital Registration - Deaths 1991 ICD9 as it appears in World Health Organization (WHO). WHO Mortality Database Version September 2016. Geneva, Switzerland: World Health Organization (WHO), 2016
Luxembourg Vital Registration - Deaths 1992 ICD9 as it appears in World Health Organization (WHO). WHO Mortality Database Version November 2015. Geneva, Switzerland: World Health Organization (WHO), 2015
Luxembourg Vital Registration - Deaths 1993 ICD9 as it appears in World Health Organization (WHO). WHO Mortality Database Version November 2015. Geneva, Switzerland: World Health Organization (WHO), 2015
Luxembourg Vital Registration - Deaths 1994 ICD9 as it appears in World Health Organization (WHO). WHO Mortality Database Version November 2015. Geneva, Switzerland: World Health Organization (WHO), 2015
Luxembourg Vital Registration - Deaths 1995 ICD9 as it appears in World Health Organization (WHO). WHO Mortality Database Version November 2015. Geneva, Switzerland: World Health Organization (WHO), 2015
Luxembourg Vital Registration - Deaths 1996 ICD9 as it appears in World Health Organization (WHO). WHO Mortality Database Version November 2015. Geneva, Switzerland: World Health Organization (WHO), 2015
Luxembourg Vital Registration - Deaths 1997 ICD9 as it appears in World Health Organization (WHO). WHO Mortality Database Version November 2015. Geneva, Switzerland: World Health Organization (WHO), 2015
Luxembourg Vital Registration - Deaths 1998 ICD10 as it appears in World Health Organization (WHO). WHO Mortality Database Version October 2017. Geneva, Switzerland: World Health Organization (WHO), 2017
Luxembourg Vital Registration - Deaths 1999 ICD10 as it appears in World Health Organization (WHO). WHO Mortality Database Version October 2017. Geneva, Switzerland: World Health Organization (WHO), 2017
Luxembourg Vital Registration - Deaths 2000 ICD10 as it appears in World Health Organization (WHO). WHO Mortality Database Version October 2017. Geneva, Switzerland: World Health Organization (WHO), 2017
Luxembourg Vital Registration - Deaths 2001 ICD10 as it appears in World Health Organization (WHO). WHO Mortality Database Version October 2017. Geneva, Switzerland: World Health Organization (WHO), 2017
Luxembourg Vital Registration - Deaths 2002 ICD10 as it appears in World Health Organization (WHO). WHO Mortality Database Version October 2017. Geneva, Switzerland: World Health Organization (WHO), 2017
Luxembourg Vital Registration - Deaths 2003 ICD10 as it appears in World Health Organization (WHO). WHO Mortality Database Version October 2017. Geneva, Switzerland: World Health Organization (WHO), 2017
Luxembourg Vital Registration - Deaths 2004 ICD10 as it appears in World Health Organization (WHO). WHO Mortality Database Version October 2017. Geneva, Switzerland: World Health Organization (WHO), 2017
Luxembourg Vital Registration - Deaths 2005 ICD10 as it appears in World Health Organization (WHO). WHO Mortality Database Version October 2017. Geneva, Switzerland: World Health Organization (WHO), 2017
Luxembourg Vital Registration - Deaths 2006 ICD10 as it appears in World Health Organization (WHO). WHO Mortality Database Version October 2017. Geneva, Switzerland: World Health Organization (WHO), 2017
Luxembourg Vital Registration - Deaths 2007 ICD10 as it appears in World Health Organization (WHO). WHO Mortality Database Version October 2017. Geneva, Switzerland: World Health Organization (WHO), 2017
Luxembourg Vital Registration - Deaths 2008 ICD10 as it appears in World Health Organization (WHO). WHO Mortality Database Version October 2017. Geneva, Switzerland: World Health Organization (WHO), 2017
Luxembourg Vital Registration - Deaths 2009 ICD10 as it appears in World Health Organization (WHO). WHO Mortality Database Version October 2017. Geneva, Switzerland: World Health Organization (WHO), 2017

Luxembourg Vital Registration - Deaths 2010 ICD10 as it appears in World Health Organization (WHO). WHO Mortality Database Version October 2017. Geneva, Switzerland: World Health Organization (WHO), 2017
Luxembourg Vital Registration - Deaths 2011 ICD10 as it appears in World Health Organization (WHO). WHO Mortality Database Version October 2017. Geneva, Switzerland: World Health Organization (WHO), 2017
Luxembourg Vital Registration - Deaths 2012 ICD10 as it appears in World Health Organization (WHO). WHO Mortality Database Version October 2017. Geneva, Switzerland: World Health Organization (WHO), 2017
Luxembourg Vital Registration - Deaths 2013 ICD10 as it appears in World Health Organization (WHO). WHO Mortality Database Version October 2017. Geneva, Switzerland: World Health Organization (WHO), 2017
Luxembourg Vital Registration - Deaths 2014 ICD10 as it appears in World Health Organization (WHO). WHO Mortality Database Version October 2017. Geneva, Switzerland: World Health Organization (WHO), 2017
Luxembourg Vital Registration - Deaths 2015 ICD10 as it appears in World Health Organization (WHO). WHO Mortality Database Version October 2017. Geneva, Switzerland: World Health Organization (WHO), 2017
Macau Vital Registration - Deaths 1994 ICD9 as it appears in World Health Organization (WHO). WHO Mortality Database Version November 2015. Geneva, Switzerland: World Health Organization (WHO), 2015
Macedonia Vital Registration - Deaths 1991 ICD9 as it appears in World Health Organization (WHO). WHO Mortality Database Version November 2015. Geneva, Switzerland: World Health Organization (WHO), 2015
Macedonia Vital Registration - Deaths 1992 ICD9 as it appears in World Health Organization (WHO). WHO Mortality Database Version November 2015. Geneva, Switzerland: World Health Organization (WHO), 2015
Macedonia Vital Registration - Deaths 1993 ICD9 as it appears in World Health Organization (WHO). WHO Mortality Database Version November 2015. Geneva, Switzerland: World Health Organization (WHO), 2015
Macedonia Vital Registration - Deaths 1994 ICD9 as it appears in World Health Organization (WHO). WHO Mortality Database Version November 2015. Geneva, Switzerland: World Health Organization (WHO), 2015
Macedonia Vital Registration - Deaths 1995 ICD9 as it appears in World Health Organization (WHO). WHO Mortality Database Version November 2015. Geneva, Switzerland: World Health Organization (WHO), 2015
Macedonia Vital Registration - Deaths 1996 ICD9 as it appears in World Health Organization (WHO). WHO Mortality Database Version November 2015. Geneva, Switzerland: World Health Organization (WHO), 2015
Macedonia Vital Registration - Deaths 1997 ICD9 as it appears in World Health Organization (WHO). WHO Mortality Database Version November 2015. Geneva, Switzerland: World Health Organization (WHO), 2015
Macedonia Vital Registration - Deaths 1998 ICD9 as it appears in World Health Organization (WHO). WHO Mortality Database Version November 2015. Geneva, Switzerland: World Health Organization (WHO), 2015
Macedonia Vital Registration - Deaths 1999 ICD9 as it appears in World Health Organization (WHO). WHO Mortality Database Version November 2015. Geneva, Switzerland: World Health Organization (WHO), 2015
Macedonia Vital Registration - Deaths 2000 ICD9 as it appears in World Health Organization (WHO). WHO Mortality Database Version November 2015. Geneva, Switzerland: World Health Organization (WHO), 2015
Macedonia Vital Registration - Deaths 2001 ICD9 as it appears in World Health Organization (WHO). WHO Mortality Database Version November 2015. Geneva, Switzerland: World Health Organization (WHO), 2015
Macedonia Vital Registration - Deaths 2002 ICD9 as it appears in World Health Organization (WHO). WHO Mortality Database Version November 2015. Geneva, Switzerland: World Health Organization (WHO), 2015
Macedonia Vital Registration - Deaths 2003 ICD9 as it appears in World Health Organization (WHO). WHO Mortality Database Version November 2015. Geneva, Switzerland: World Health Organization (WHO), 2015

Macedonia Vital Registration - Deaths 2004 ICD9 as it appears in World Health Organization (WHO). WHO Mortality Database Version September 2016. Geneva, Switzerland: World Health Organization (WHO), 2016
Macedonia Vital Registration - Deaths 2005 ICD9 as it appears in World Health Organization (WHO). WHO Mortality Database Version September 2016. Geneva, Switzerland: World Health Organization (WHO), 2016
Macedonia Vital Registration - Deaths 2006 ICD10 as it appears in World Health Organization (WHO). WHO Mortality Database Version October 2017. Geneva, Switzerland: World Health Organization (WHO), 2017
Macedonia Vital Registration - Deaths 2007 ICD10 as it appears in World Health Organization (WHO). WHO Mortality Database Version October 2017. Geneva, Switzerland: World Health Organization (WHO), 2017
Macedonia Vital Registration - Deaths 2008 ICD10 as it appears in World Health Organization (WHO). WHO Mortality Database Version October 2017. Geneva, Switzerland: World Health Organization (WHO), 2017
Macedonia Vital Registration - Deaths 2009 ICD10 as it appears in World Health Organization (WHO). WHO Mortality Database Version October 2017. Geneva, Switzerland: World Health Organization (WHO), 2017
Macedonia Vital Registration - Deaths 2010 ICD10 as it appears in World Health Organization (WHO). WHO Mortality Database Version October 2017. Geneva, Switzerland: World Health Organization (WHO), 2017
Macedonia Vital Registration - Deaths 2011 ICD10 as it appears in World Health Organization (WHO). WHO Mortality Database Version March 2017. Geneva, Switzerland: World Health Organization (WHO), 2017
Macedonia Vital Registration - Deaths 2012 ICD10 as it appears in World Health Organization (WHO). WHO Mortality Database Version March 2017. Geneva, Switzerland: World Health Organization (WHO), 2017
Macedonia Vital Registration - Deaths 2013 ICD10 as it appears in World Health Organization (WHO). WHO Mortality Database Version March 2017. Geneva, Switzerland: World Health Organization (WHO), 2017
Malaysia Vital Registration - Deaths 2000 ICD10 as it appears in World Health Organization (WHO). WHO Mortality Database Version October 2017. Geneva, Switzerland: World Health Organization (WHO), 2017
Malaysia Vital Registration - Deaths 2001 ICD10 as it appears in World Health Organization (WHO). WHO Mortality Database Version October 2017. Geneva, Switzerland: World Health Organization (WHO), 2017
Malaysia Vital Registration - Deaths 2002 ICD10 as it appears in World Health Organization (WHO). WHO Mortality Database Version October 2017. Geneva, Switzerland: World Health Organization (WHO), 2017
Malaysia Vital Registration - Deaths 2003 ICD10 as it appears in World Health Organization (WHO). WHO Mortality Database Version October 2017. Geneva, Switzerland: World Health Organization (WHO), 2017
Malaysia Vital Registration - Deaths 2004 ICD10 as it appears in World Health Organization (WHO). WHO Mortality Database Version October 2017. Geneva, Switzerland: World Health Organization (WHO), 2017
Malaysia Vital Registration - Deaths 2005 ICD10 as it appears in World Health Organization (WHO). WHO Mortality Database Version October 2017. Geneva, Switzerland: World Health Organization (WHO), 2017
Malaysia Vital Registration - Deaths 2006 ICD10 as it appears in World Health Organization (WHO). WHO Mortality Database Version October 2017. Geneva, Switzerland: World Health Organization (WHO), 2017
Malaysia Vital Registration - Deaths 2007 ICD10 as it appears in World Health Organization (WHO). WHO Mortality Database Version October 2017. Geneva, Switzerland: World Health Organization (WHO), 2017
Malaysia Vital Registration - Deaths 2008 ICD10 as it appears in World Health Organization (WHO). WHO Mortality Database Version October 2017. Geneva, Switzerland: World Health Organization (WHO), 2017
Malaysia Vital Registration - Deaths 2009 ICD10 as it appears in World Health Organization (WHO). WHO Mortality Database Version October 2017. Geneva, Switzerland: World Health Organization (WHO), 2017

Malaysia Vital Registration - Deaths 2010 ICD10 as it appears in World Health Organization (WHO). WHO Mortality Database Version October 2017. Geneva, Switzerland: World Health Organization (WHO), 2017
Malaysia Vital Registration - Deaths 2011 ICD10 as it appears in World Health Organization (WHO). WHO Mortality Database Version October 2017. Geneva, Switzerland: World Health Organization (WHO), 2017
Malaysia Vital Registration - Deaths 2012 ICD10 as it appears in World Health Organization (WHO). WHO Mortality Database Version October 2017. Geneva, Switzerland: World Health Organization (WHO), 2017
Malaysia Vital Registration - Deaths 2013 ICD10 as it appears in World Health Organization (WHO). WHO Mortality Database Version October 2017. Geneva, Switzerland: World Health Organization (WHO), 2017
Malaysia Vital Registration - Deaths 2014 ICD10 as it appears in World Health Organization (WHO). WHO Mortality Database Version October 2017. Geneva, Switzerland: World Health Organization (WHO), 2017
Maldives Vital Registration - Deaths 2010 ICD10 as it appears in World Health Organization (WHO). WHO Mortality Database Version October 2017. Geneva, Switzerland: World Health Organization (WHO), 2017
Maldives Vital Registration - Deaths 2011 ICD10 as it appears in World Health Organization (WHO). WHO Mortality Database Version October 2017. Geneva, Switzerland: World Health Organization (WHO), 2017
Malta Vital Registration - Deaths 1980 ICD9 as it appears in World Health Organization (WHO). WHO Mortality Database Version November 2015. Geneva, Switzerland: World Health Organization (WHO), 2015
Malta Vital Registration - Deaths 1981 ICD9 as it appears in World Health Organization (WHO). WHO Mortality Database Version November 2015. Geneva, Switzerland: World Health Organization (WHO), 2015
Malta Vital Registration - Deaths 1982 ICD9 as it appears in World Health Organization (WHO). WHO Mortality Database Version November 2015. Geneva, Switzerland: World Health Organization (WHO), 2015
Malta Vital Registration - Deaths 1983 ICD9 as it appears in World Health Organization (WHO). WHO Mortality Database Version November 2015. Geneva, Switzerland: World Health Organization (WHO), 2015
Malta Vital Registration - Deaths 1984 ICD9 as it appears in World Health Organization (WHO). WHO Mortality Database Version November 2015. Geneva, Switzerland: World Health Organization (WHO), 2015
Malta Vital Registration - Deaths 1985 ICD9 as it appears in World Health Organization (WHO). WHO Mortality Database Version November 2015. Geneva, Switzerland: World Health Organization (WHO), 2015
Malta Vital Registration - Deaths 1986 ICD9 as it appears in World Health Organization (WHO). WHO Mortality Database Version November 2015. Geneva, Switzerland: World Health Organization (WHO), 2015
Malta Vital Registration - Deaths 1987 ICD9 as it appears in World Health Organization (WHO). WHO Mortality Database Version November 2015. Geneva, Switzerland: World Health Organization (WHO), 2015
Malta Vital Registration - Deaths 1988 ICD9 as it appears in World Health Organization (WHO). WHO Mortality Database Version November 2015. Geneva, Switzerland: World Health Organization (WHO), 2015
Malta Vital Registration - Deaths 1989 ICD9 as it appears in World Health Organization (WHO). WHO Mortality Database Version November 2015. Geneva, Switzerland: World Health Organization (WHO), 2015
Malta Vital Registration - Deaths 1990 ICD9 as it appears in World Health Organization (WHO). WHO Mortality Database Version November 2015. Geneva, Switzerland: World Health Organization (WHO), 2015
Malta Vital Registration - Deaths 1991 ICD9 as it appears in World Health Organization (WHO). WHO Mortality Database Version November 2015. Geneva, Switzerland: World Health Organization (WHO), 2015
Malta Vital Registration - Deaths 1992 ICD9 as it appears in World Health Organization (WHO). WHO Mortality Database Version November 2015. Geneva, Switzerland: World Health Organization (WHO), 2015

Malta Vital Registration - Deaths 1994 ICD9 as it appears in World Health Organization (WHO). WHO Mortality Database Version November 2015. Geneva, Switzerland: World Health Organization (WHO), 2015

Malta Vital Registration - Deaths 1995 ICD10 as it appears in World Health Organization (WHO). WHO Mortality Database Version October 2017. Geneva, Switzerland: World Health Organization (WHO), 2017

Malta Vital Registration - Deaths 1996 ICD10 as it appears in World Health Organization (WHO). WHO Mortality Database Version October 2017. Geneva, Switzerland: World Health Organization (WHO), 2017

Malta Vital Registration - Deaths 1998 ICD10 as it appears in World Health Organization (WHO). WHO Mortality Database Version October 2017. Geneva, Switzerland: World Health Organization (WHO), 2017

Malta Vital Registration - Deaths 1999 ICD10 as it appears in World Health Organization (WHO). WHO Mortality Database Version October 2017. Geneva, Switzerland: World Health Organization (WHO), 2017

Malta Vital Registration - Deaths 2000 ICD10 as it appears in World Health Organization (WHO). WHO Mortality Database Version October 2017. Geneva, Switzerland: World Health Organization (WHO), 2017

Malta Vital Registration - Deaths 2001 ICD10 as it appears in World Health Organization (WHO). WHO Mortality Database Version October 2017. Geneva, Switzerland: World Health Organization (WHO), 2017

Malta Vital Registration - Deaths 2002 ICD10 as it appears in World Health Organization (WHO). WHO Mortality Database Version October 2017. Geneva, Switzerland: World Health Organization (WHO), 2017

Malta Vital Registration - Deaths 2004 ICD10 as it appears in World Health Organization (WHO). WHO Mortality Database Version October 2017. Geneva, Switzerland: World Health Organization (WHO), 2017

Malta Vital Registration - Deaths 2005 ICD10 as it appears in World Health Organization (WHO). WHO Mortality Database Version October 2017. Geneva, Switzerland: World Health Organization (WHO), 2017

Malta Vital Registration - Deaths 2006 ICD10 as it appears in World Health Organization (WHO). WHO Mortality Database Version October 2017. Geneva, Switzerland: World Health Organization (WHO), 2017

Malta Vital Registration - Deaths 2007 ICD10 as it appears in World Health Organization (WHO). WHO Mortality Database Version October 2017. Geneva, Switzerland: World Health Organization (WHO), 2017

Malta Vital Registration - Deaths 2008 ICD10 as it appears in World Health Organization (WHO). WHO Mortality Database Version October 2017. Geneva, Switzerland: World Health Organization (WHO), 2017

Malta Vital Registration - Deaths 1993 ICD9 as it appears in World Health Organization (WHO). WHO Mortality Database Version November 2015. Geneva, Switzerland: World Health Organization (WHO), 2015

Malta Vital Registration - Deaths 1997 ICD10 as it appears in World Health Organization (WHO). WHO Mortality Database Version October 2017. Geneva, Switzerland: World Health Organization (WHO), 2017

Malta Vital Registration - Deaths 2003 ICD10 as it appears in World Health Organization (WHO). WHO Mortality Database Version October 2017. Geneva, Switzerland: World Health Organization (WHO), 2017

Malta Vital Registration - Deaths 2009 ICD10 as it appears in World Health Organization (WHO). WHO Mortality Database Version October 2017. Geneva, Switzerland: World Health Organization (WHO), 2017

Malta Vital Registration - Deaths 2010 ICD10 as it appears in World Health Organization (WHO). WHO Mortality Database Version October 2017. Geneva, Switzerland: World Health Organization (WHO), 2017

Malta Vital Registration - Deaths 2011 ICD10 as it appears in World Health Organization (WHO). WHO Mortality Database Version October 2017. Geneva, Switzerland: World Health Organization (WHO), 2017

Malta Vital Registration - Deaths 2012 ICD10 as it appears in World Health Organization (WHO). WHO Mortality Database Version October 2017. Geneva, Switzerland: World Health Organization (WHO), 2017

Malta Vital Registration - Deaths 2013 ICD10 as it appears in World Health Organization (WHO). WHO Mortality Database Version October 2017. Geneva, Switzerland:

World Health Organization (WHO), 2017
Malta Vital Registration - Deaths 2014 ICD10 as it appears in World Health Organization (WHO). WHO Mortality Database Version October 2017. Geneva, Switzerland: World Health Organization (WHO), 2017
Malta Vital Registration - Deaths 2015 ICD10 as it appears in World Health Organization (WHO). WHO Mortality Database Version April 2018. Geneva, Switzerland: World Health Organization (WHO), 2018
Mauritius Vital Registration - Deaths 1980 ICD8 as it appears in World Health Organization (WHO). WHO Mortality Database Version July 2012. Geneva, Switzerland: World Health Organization (WHO), 2012
Mauritius Vital Registration - Deaths 1981 ICD9 as it appears in World Health Organization (WHO). WHO Mortality Database Version September 2016. Geneva, Switzerland: World Health Organization (WHO), 2016
Mauritius Vital Registration - Deaths 1982 ICD9 as it appears in World Health Organization (WHO). WHO Mortality Database Version September 2016. Geneva, Switzerland: World Health Organization (WHO), 2016
Mauritius Vital Registration - Deaths 1983 ICD9 as it appears in World Health Organization (WHO). WHO Mortality Database Version September 2016. Geneva, Switzerland: World Health Organization (WHO), 2016
Mauritius Vital Registration - Deaths 1984 ICD9 as it appears in World Health Organization (WHO). WHO Mortality Database Version September 2016. Geneva, Switzerland: World Health Organization (WHO), 2016
Mauritius Vital Registration - Deaths 1985 ICD9 as it appears in World Health Organization (WHO). WHO Mortality Database Version September 2016. Geneva, Switzerland: World Health Organization (WHO), 2016
Mauritius Vital Registration - Deaths 1986 ICD9 as it appears in World Health Organization (WHO). WHO Mortality Database Version September 2016. Geneva, Switzerland: World Health Organization (WHO), 2016
Mauritius Vital Registration - Deaths 1987 ICD9 as it appears in World Health Organization (WHO). WHO Mortality Database Version September 2016. Geneva, Switzerland: World Health Organization (WHO), 2016
Mauritius Vital Registration - Deaths 1988 ICD9 as it appears in World Health Organization (WHO). WHO Mortality Database Version September 2016. Geneva, Switzerland: World Health Organization (WHO), 2016
Mauritius Vital Registration - Deaths 1989 ICD9 as it appears in World Health Organization (WHO). WHO Mortality Database Version September 2016. Geneva, Switzerland: World Health Organization (WHO), 2016
Mauritius Vital Registration - Deaths 1990 ICD9 as it appears in World Health Organization (WHO). WHO Mortality Database Version September 2016. Geneva, Switzerland: World Health Organization (WHO), 2016
Mauritius Vital Registration - Deaths 1991 ICD9 as it appears in World Health Organization (WHO). WHO Mortality Database Version November 2015. Geneva, Switzerland: World Health Organization (WHO), 2015
Mauritius Vital Registration - Deaths 1992 ICD9 as it appears in World Health Organization (WHO). WHO Mortality Database Version November 2015. Geneva, Switzerland: World Health Organization (WHO), 2015
Mauritius Vital Registration - Deaths 1993 ICD9 as it appears in World Health Organization (WHO). WHO Mortality Database Version November 2015. Geneva, Switzerland: World Health Organization (WHO), 2015
Mauritius Vital Registration - Deaths 1994 ICD9 as it appears in World Health Organization (WHO). WHO Mortality Database Version November 2015. Geneva, Switzerland: World Health Organization (WHO), 2015
Mauritius Vital Registration - Deaths 1995 ICD9 as it appears in World Health Organization (WHO). WHO Mortality Database Version November 2015. Geneva, Switzerland: World Health Organization (WHO), 2015
Mauritius Vital Registration - Deaths 1996 ICD9 as it appears in World Health Organization (WHO). WHO Mortality Database Version November 2015. Geneva, Switzerland: World Health Organization (WHO), 2015
Mauritius Vital Registration - Deaths 1997 ICD9 as it appears in World Health Organization (WHO). WHO Mortality Database Version November 2015. Geneva, Switzerland: World Health Organization (WHO), 2015

Mauritius Vital Registration - Deaths 1998 ICD9 as it appears in World Health Organization (WHO). WHO Mortality Database Version November 2015. Geneva, Switzerland: World Health Organization (WHO), 2015
Mauritius Vital Registration - Deaths 1999 ICD9 as it appears in World Health Organization (WHO). WHO Mortality Database Version November 2015. Geneva, Switzerland: World Health Organization (WHO), 2015
Mauritius Vital Registration - Deaths 2000 ICD9 as it appears in World Health Organization (WHO). WHO Mortality Database Version November 2015. Geneva, Switzerland: World Health Organization (WHO), 2015
Mauritius Vital Registration - Deaths 2001 ICD9 as it appears in World Health Organization (WHO). WHO Mortality Database Version November 2015. Geneva, Switzerland: World Health Organization (WHO), 2015
Mauritius Vital Registration - Deaths 2003 ICD9 as it appears in World Health Organization (WHO). WHO Mortality Database Version November 2015. Geneva, Switzerland: World Health Organization (WHO), 2015
Mauritius Vital Registration - Deaths 2004 ICD9 as it appears in World Health Organization (WHO). WHO Mortality Database Version November 2015. Geneva, Switzerland: World Health Organization (WHO), 2015
Mauritius Vital Registration - Deaths 2005 ICD10 as it appears in World Health Organization (WHO). WHO Mortality Database Version October 2017. Geneva, Switzerland: World Health Organization (WHO), 2017
Mauritius Vital Registration - Deaths 2006 ICD10 as it appears in World Health Organization (WHO). WHO Mortality Database Version October 2017. Geneva, Switzerland: World Health Organization (WHO), 2017
Mauritius Vital Registration - Deaths 2007 ICD10 as it appears in World Health Organization (WHO). WHO Mortality Database Version October 2017. Geneva, Switzerland: World Health Organization (WHO), 2017
Mauritius Vital Registration - Deaths 2008 ICD10 as it appears in World Health Organization (WHO). WHO Mortality Database Version October 2017. Geneva, Switzerland: World Health Organization (WHO), 2017
Mauritius Vital Registration - Deaths 2002 ICD9 as it appears in World Health Organization (WHO). WHO Mortality Database Version November 2015. Geneva, Switzerland: World Health Organization (WHO), 2015
Mauritius Vital Registration - Deaths 2009 ICD10 as it appears in World Health Organization (WHO). WHO Mortality Database Version October 2017. Geneva, Switzerland: World Health Organization (WHO), 2017
Mauritius Vital Registration - Deaths 2010 ICD10 as it appears in World Health Organization (WHO). WHO Mortality Database Version October 2017. Geneva, Switzerland: World Health Organization (WHO), 2017
Mauritius Vital Registration - Deaths 2011 ICD10 as it appears in World Health Organization (WHO). WHO Mortality Database Version October 2017. Geneva, Switzerland: World Health Organization (WHO), 2017
Mauritius Vital Registration - Deaths 2012 ICD10 as it appears in World Health Organization (WHO). WHO Mortality Database Version October 2017. Geneva, Switzerland: World Health Organization (WHO), 2017
Mauritius Vital Registration - Deaths 2013 ICD10 as it appears in World Health Organization (WHO). WHO Mortality Database Version October 2017. Geneva, Switzerland: World Health Organization (WHO), 2017
Mauritius Vital Registration - Deaths 2014 ICD10 as it appears in World Health Organization (WHO). WHO Mortality Database Version October 2017. Geneva, Switzerland: World Health Organization (WHO), 2017
Mauritius Vital Registration - Deaths 2015 ICD10 as it appears in World Health Organization (WHO). WHO Mortality Database Version October 2017. Geneva, Switzerland: World Health Organization (WHO), 2017
Mauritius Vital Registration - Deaths 2016 ICD10 as it appears in World Health Organization (WHO). WHO Mortality Database Version October 2017. Geneva, Switzerland: World Health Organization (WHO), 2017
Ministry of Health (Brazil). Brazil Mortality Information System - Deaths 1980. Rio de Janeiro, Brazil: Ministry of Health (Brazil)
Ministry of Health (Brazil). Brazil Mortality Information System - Deaths 1981. Rio de Janeiro, Brazil: Ministry of Health (Brazil)
Ministry of Health (Brazil). Brazil Mortality Information System - Deaths 1982. Rio de Janeiro, Brazil: Ministry of Health (Brazil)

Ministry of Health (Brazil). Brazil Mortality Information System - Deaths 1983. Rio de Janeiro, Brazil: Ministry of Health (Brazil)
Ministry of Health (Brazil). Brazil Mortality Information System - Deaths 1984. Rio de Janeiro, Brazil: Ministry of Health (Brazil)
Ministry of Health (Brazil). Brazil Mortality Information System - Deaths 1985. Rio de Janeiro, Brazil: Ministry of Health (Brazil)
Ministry of Health (Brazil). Brazil Mortality Information System - Deaths 1986. Rio de Janeiro, Brazil: Ministry of Health (Brazil)
Ministry of Health (Brazil). Brazil Mortality Information System - Deaths 1987. Rio de Janeiro, Brazil: Ministry of Health (Brazil)
Ministry of Health (Brazil). Brazil Mortality Information System - Deaths 1988. Rio de Janeiro, Brazil: Ministry of Health (Brazil)
Ministry of Health (Brazil). Brazil Mortality Information System - Deaths 1989. Rio de Janeiro, Brazil: Ministry of Health (Brazil)
Ministry of Health (Brazil). Brazil Mortality Information System - Deaths 1990. Rio de Janeiro, Brazil: Ministry of Health (Brazil)
Ministry of Health (Brazil). Brazil Mortality Information System - Deaths 1991. Rio de Janeiro, Brazil: Ministry of Health (Brazil)
Ministry of Health (Brazil). Brazil Mortality Information System - Deaths 1992. Rio de Janeiro, Brazil: Ministry of Health (Brazil)
Ministry of Health (Brazil). Brazil Mortality Information System - Deaths 1993. Rio de Janeiro, Brazil: Ministry of Health (Brazil)
Ministry of Health (Brazil). Brazil Mortality Information System - Deaths 1994. Rio de Janeiro, Brazil: Ministry of Health (Brazil)
Ministry of Health (Brazil). Brazil Mortality Information System - Deaths 1995. Rio de Janeiro, Brazil: Ministry of Health (Brazil)
Ministry of Health (Brazil). Brazil Mortality Information System - Deaths 1996. Rio de Janeiro, Brazil: Ministry of Health (Brazil)
Ministry of Health (Brazil). Brazil Mortality Information System - Deaths 1997. Rio de Janeiro, Brazil: Ministry of Health (Brazil)
Ministry of Health (Brazil). Brazil Mortality Information System - Deaths 1998. Rio de Janeiro, Brazil: Ministry of Health (Brazil)
Ministry of Health (Brazil). Brazil Mortality Information System - Deaths 1999. Rio de Janeiro, Brazil: Ministry of Health (Brazil)
Ministry of Health (Brazil). Brazil Mortality Information System - Deaths 2000. Rio de Janeiro, Brazil: Ministry of Health (Brazil)
Ministry of Health (Brazil). Brazil Mortality Information System - Deaths 2001. Rio de Janeiro, Brazil: Ministry of Health (Brazil)
Ministry of Health (Brazil). Brazil Mortality Information System - Deaths 2002. Rio de Janeiro, Brazil: Ministry of Health (Brazil)
Ministry of Health (Brazil). Brazil Mortality Information System - Deaths 2003. Rio de Janeiro, Brazil: Ministry of Health (Brazil)
Ministry of Health (Brazil). Brazil Mortality Information System - Deaths 2004. Rio de Janeiro, Brazil: Ministry of Health (Brazil)
Ministry of Health (Brazil). Brazil Mortality Information System - Deaths 2005. Rio de Janeiro, Brazil: Ministry of Health (Brazil)
Ministry of Health (Brazil). Brazil Mortality Information System - Deaths 2006. Rio de Janeiro, Brazil: Ministry of Health (Brazil)
Ministry of Health (Brazil). Brazil Mortality Information System - Deaths 2007. Rio de Janeiro, Brazil: Ministry of Health (Brazil)
Ministry of Health (Brazil). Brazil Mortality Information System - Deaths 2008. Rio de Janeiro, Brazil: Ministry of Health (Brazil)
Ministry of Health (Brazil). Brazil Mortality Information System - Deaths 2009. Rio de Janeiro, Brazil: Ministry of Health (Brazil)
Ministry of Health (Brazil). Brazil Mortality Information System - Deaths 2010. Rio de Janeiro, Brazil: Ministry of Health (Brazil)
Ministry of Health (Brazil). Brazil Mortality Information System - Deaths 2011. Rio de Janeiro, Brazil: Ministry of Health (Brazil)
Ministry of Health (Brazil). Brazil Mortality Information System - Deaths 2012. Rio de Janeiro, Brazil: Ministry of Health (Brazil)
Ministry of Health (Brazil). Brazil Mortality Information System - Deaths 2013. Rio de Janeiro, Brazil: Ministry of Health (Brazil)
Ministry of Health (Brazil). Brazil Mortality Information System - Deaths 2014. Rio de Janeiro, Brazil: Ministry of Health (Brazil)
Ministry of Health (Brazil). Brazil Mortality Information System - Deaths 2015. Rio de Janeiro, Brazil: Ministry of Health (Brazil)
Ministry of Health (Brazil). Brazil Mortality Information System - Deaths 2016. Rio de Janeiro, Brazil: Ministry of Health (Brazil)
Ministry of Health (China). China National Maternal and Child Health Surveillance System Child Mortality By Cause 2013 - MCHS

Ministry of Health (China). China National Maternal and Child Health Surveillance System Child Mortality Data By Cause 1996-2012 - MCHS
Ministry of Health (Jordan). Jordan Causes of Death and Population 2013
Ministry of Health (Jordan). Jordan Vital Registration - Deaths 2004-2006
Ministry of Health (Myanmar). Myanmar National Mortality Survey 2016
Ministry of Health (New Zealand). New Zealand Mortality Collection 1988. Wellington, New Zealand: Ministry of Health (New Zealand)
Ministry of Health (New Zealand). New Zealand Mortality Collection 1989. Wellington, New Zealand: Ministry of Health (New Zealand)
Ministry of Health (New Zealand). New Zealand Mortality Collection 1990. Wellington, New Zealand: Ministry of Health (New Zealand)
Ministry of Health (New Zealand). New Zealand Mortality Collection 1991. Wellington, New Zealand: Ministry of Health (New Zealand)
Ministry of Health (New Zealand). New Zealand Mortality Collection 1992. Wellington, New Zealand: Ministry of Health (New Zealand)
Ministry of Health (New Zealand). New Zealand Mortality Collection 1993. Wellington, New Zealand: Ministry of Health (New Zealand)
Ministry of Health (New Zealand). New Zealand Mortality Collection 1994. Wellington, New Zealand: Ministry of Health (New Zealand)
Ministry of Health (New Zealand). New Zealand Mortality Collection 1996. Wellington, New Zealand: Ministry of Health (New Zealand)
Ministry of Health (New Zealand). New Zealand Mortality Collection 1997. Wellington, New Zealand: Ministry of Health (New Zealand)
Ministry of Health (New Zealand). New Zealand Mortality Collection 1998. Wellington, New Zealand: Ministry of Health (New Zealand)
Ministry of Health (New Zealand). New Zealand Mortality Collection 1999. Wellington, New Zealand: Ministry of Health (New Zealand)
Ministry of Health (New Zealand). New Zealand Mortality Collection 2000. Wellington, New Zealand: Ministry of Health (New Zealand)
Ministry of Health (New Zealand). New Zealand Mortality Collection 2001. Wellington, New Zealand: Ministry of Health (New Zealand)
Ministry of Health (New Zealand). New Zealand Mortality Collection 2002. Wellington, New Zealand: Ministry of Health (New Zealand)
Ministry of Health (New Zealand). New Zealand Mortality Collection 2003. Wellington, New Zealand: Ministry of Health (New Zealand)
Ministry of Health (New Zealand). New Zealand Mortality Collection 2004. Wellington, New Zealand: Ministry of Health (New Zealand)
Ministry of Health (New Zealand). New Zealand Mortality Collection 2005. Wellington, New Zealand: Ministry of Health (New Zealand)
Ministry of Health (New Zealand). New Zealand Mortality Collection 2006. Wellington, New Zealand: Ministry of Health (New Zealand)
Ministry of Health (New Zealand). New Zealand Mortality Collection 2007. Wellington, New Zealand: Ministry of Health (New Zealand)
Ministry of Health (New Zealand). New Zealand Mortality Collection 2008. Wellington, New Zealand: Ministry of Health (New Zealand)
Ministry of Health (New Zealand). New Zealand Mortality Collection 2009. Wellington, New Zealand: Ministry of Health (New Zealand)
Ministry of Health (New Zealand). New Zealand Mortality Collection 2010. Wellington, New Zealand: Ministry of Health (New Zealand)
Ministry of Health (New Zealand). New Zealand Mortality Collection 2011. Wellington, New Zealand: Ministry of Health (New Zealand)
Ministry of Health (New Zealand). New Zealand Mortality Collection 2012. Wellington, New Zealand: Ministry of Health (New Zealand)
Ministry of Health (New Zealand). New Zealand Mortality Collection 2013. Wellington, New Zealand: Ministry of Health (New Zealand)
Ministry of Health (New Zealand). New Zealand Mortality Collection 2014. Wellington, New Zealand: Ministry of Health (New Zealand)
Ministry of Health (New Zealand). New Zealand Mortality Collection 2015. Wellington, New Zealand: Ministry of Health (New Zealand)
Ministry of Health (New Zealand). New Zealand Mortality Data 1980. Wellington, New Zealand: Ministry of Health (New Zealand)
Ministry of Health (New Zealand). New Zealand Mortality Data 1981. Wellington, New Zealand: Ministry of Health (New Zealand)
Ministry of Health (New Zealand). New Zealand Mortality Data 1982. Wellington, New Zealand: Ministry of Health (New Zealand)
Ministry of Health (New Zealand). New Zealand Mortality Data 1983. Wellington, New Zealand: Ministry of Health (New Zealand)

Ministry of Health (New Zealand). New Zealand Mortality Data 1984. Wellington, New Zealand: Ministry of Health (New Zealand)
Ministry of Health (New Zealand). New Zealand Mortality Data 1985. Wellington, New Zealand: Ministry of Health (New Zealand)
Ministry of Health (New Zealand). New Zealand Mortality Data 1986. Wellington, New Zealand: Ministry of Health (New Zealand)
Ministry of Health (New Zealand). New Zealand Mortality Data 1987. Wellington, New Zealand: Ministry of Health (New Zealand)
Ministry of Health (Palestine). Palestine - Gaza Strip and West Bank Mortality by Sex, Age, and Cause 2012
Ministry of Health (Palestine). Palestine - Gaza Strip and West Bank Mortality by Sex, Age, and Cause 2013
Ministry of Health (Palestine). Palestine - Gaza Strip and West Bank Mortality by Sex, Age, and Cause 2014
Ministry of Health (Palestine). Palestine - Gaza Strip and West Bank Mortality by Sex, Age, and Cause 2015
Ministry of Health (Palestine). Palestine - Gaza Strip and West Bank Mortality by Sex, Age, and Cause 2016
Ministry of Health (Saudi Arabia). Saudi Arabia Vital Registration - Deaths 1996-2011
Ministry of Health (Singapore). Singapore Causes of Death 1980
Ministry of Health (Singapore). Singapore Causes of Death 1981
Ministry of Health (Singapore). Singapore Causes of Death 1982
Ministry of Health (Singapore). Singapore Causes of Death 1983
Ministry of Health (Singapore). Singapore Causes of Death 1984
Ministry of Health (Singapore). Singapore Causes of Death 1985
Ministry of Health (Singapore). Singapore Causes of Death 1986
Ministry of Health (Singapore). Singapore Causes of Death 1987
Ministry of Health (Singapore). Singapore Causes of Death 1988
Ministry of Health (Singapore). Singapore Causes of Death 1989
Ministry of Health (Singapore). Singapore Causes of Death 1990
Ministry of Health (Singapore). Singapore Causes of Death 1991
Ministry of Health (Singapore). Singapore Causes of Death 1992
Ministry of Health (Singapore). Singapore Causes of Death 1993
Ministry of Health (Singapore). Singapore Causes of Death 1994
Ministry of Health (Singapore). Singapore Causes of Death 1995
Ministry of Health (Singapore). Singapore Causes of Death 1996
Ministry of Health (Singapore). Singapore Causes of Death 1997
Ministry of Health (Singapore). Singapore Causes of Death 1998
Ministry of Health (Singapore). Singapore Causes of Death 1999
Ministry of Health (Singapore). Singapore Causes of Death 2000
Ministry of Health (Singapore). Singapore Causes of Death 2001
Ministry of Health (Singapore). Singapore Causes of Death 2002
Ministry of Health (Singapore). Singapore Causes of Death 2003
Ministry of Health (Singapore). Singapore Causes of Death 2004

Ministry of Health (Singapore). Singapore Causes of Death 2005
Ministry of Health (Singapore). Singapore Causes of Death 2006
Ministry of Health (Singapore). Singapore Causes of Death 2007
Ministry of Health (Singapore). Singapore Causes of Death 2008
Ministry of Health (Singapore). Singapore Causes of Death 2009
Ministry of Health (Singapore). Singapore Causes of Death 2010
Ministry of Health (Singapore). Singapore Causes of Death 2011
Ministry of Health (Singapore). Singapore Causes of Death 2012
Ministry of Health (Singapore). Singapore Causes of Death 2013
Ministry of Health (Singapore). Singapore Causes of Death 2014
Ministry of Health (Singapore). Singapore Causes of Death 2015
Ministry of Health (Singapore). Singapore Causes of Death 2016
Ministry of Health and Medical Education (Iran). Iran Death Registration System 2001
Ministry of Health and Medical Education (Iran). Iran Death Registration System 2002
Ministry of Health and Medical Education (Iran). Iran Death Registration System 2003
Ministry of Health and Medical Education (Iran). Iran Death Registration System 2004
Ministry of Health and Medical Education (Iran). Iran Death Registration System 2005
Ministry of Health and Medical Education (Iran). Iran Death Registration System 2006
Ministry of Health and Medical Education (Iran). Iran Death Registration System 2007
Ministry of Health and Medical Education (Iran). Iran Death Registration System 2008
Ministry of Health and Medical Education (Iran). Iran Death Registration System 2009
Ministry of Health and Medical Education (Iran). Iran Death Registration System 2010
Ministry of Health and Medical Education (Iran). Iran Death Registration System 2011
Ministry of Health and Medical Education (Iran). Iran Death Registration System 2012
Ministry of Health and Medical Education (Iran). Iran Death Registration System 2013
Ministry of Health and Medical Education (Iran). Iran Death Registration System 2014
Ministry of Health and Medical Education (Iran). Iran Death Registration System 2015
Ministry of Health and Welfare (Taiwan). Taiwan Vital Registration - Deaths 2013. Taipei City, Taiwan: Ministry of Health and Welfare (Taiwan)
Ministry of Health and Welfare (Taiwan). Taiwan Vital Registration - Deaths 2014. Taipei City, Taiwan: Ministry of Health and Welfare (Taiwan)
Ministry of Health and Welfare (Taiwan). Taiwan Vital Registration - Deaths 2015. Taipei City, Taiwan: Ministry of Health and Welfare (Taiwan)
Ministry of Health and Welfare (Taiwan). Taiwan Vital Registration - Deaths 2016. Taipei City, Taiwan: Ministry of Health and Welfare (Taiwan)
Ministry of Health, Labour and Welfare (Japan). Japan Vital Registration - Deaths 1980
Ministry of Health, Labour and Welfare (Japan). Japan Vital Registration - Deaths 1981
Ministry of Health, Labour and Welfare (Japan). Japan Vital Registration - Deaths 1982
Ministry of Health, Labour and Welfare (Japan). Japan Vital Registration - Deaths 1983

Ministry of Health, Labour and Welfare (Japan). Japan Vital Registration - Deaths 1984
Ministry of Health, Labour and Welfare (Japan). Japan Vital Registration - Deaths 1985
Ministry of Health, Labour and Welfare (Japan). Japan Vital Registration - Deaths 1986
Ministry of Health, Labour and Welfare (Japan). Japan Vital Registration - Deaths 1987
Ministry of Health, Labour and Welfare (Japan). Japan Vital Registration - Deaths 1988
Ministry of Health, Labour and Welfare (Japan). Japan Vital Registration - Deaths 1989
Ministry of Health, Labour and Welfare (Japan). Japan Vital Registration - Deaths 1990
Ministry of Health, Labour and Welfare (Japan). Japan Vital Registration - Deaths 1991
Ministry of Health, Labour and Welfare (Japan). Japan Vital Registration - Deaths 1992
Ministry of Health, Labour and Welfare (Japan). Japan Vital Registration - Deaths 1993
Ministry of Health, Labour and Welfare (Japan). Japan Vital Registration - Deaths 1994
Ministry of Health, Labour and Welfare (Japan). Japan Vital Registration - Deaths 1995
Ministry of Health, Labour and Welfare (Japan). Japan Vital Registration - Deaths 1996
Ministry of Health, Labour and Welfare (Japan). Japan Vital Registration - Deaths 1997
Ministry of Health, Labour and Welfare (Japan). Japan Vital Registration - Deaths 1998
Ministry of Health, Labour and Welfare (Japan). Japan Vital Registration - Deaths 1999
Ministry of Health, Labour and Welfare (Japan). Japan Vital Registration - Deaths 2000
Ministry of Health, Labour and Welfare (Japan). Japan Vital Registration - Deaths 2001
Ministry of Health, Labour and Welfare (Japan). Japan Vital Registration - Deaths 2002
Ministry of Health, Labour and Welfare (Japan). Japan Vital Registration - Deaths 2003
Ministry of Health, Labour and Welfare (Japan). Japan Vital Registration - Deaths 2004
Ministry of Health, Labour and Welfare (Japan). Japan Vital Registration - Deaths 2005
Ministry of Health, Labour and Welfare (Japan). Japan Vital Registration - Deaths 2006
Ministry of Health, Labour and Welfare (Japan). Japan Vital Registration - Deaths 2007
Ministry of Health, Labour and Welfare (Japan). Japan Vital Registration - Deaths 2008
Ministry of Health, Labour and Welfare (Japan). Japan Vital Registration - Deaths 2009
Ministry of Health, Labour and Welfare (Japan). Japan Vital Registration - Deaths 2010
Ministry of Health, Labour and Welfare (Japan). Japan Vital Registration - Deaths 2011
Ministry of Health, Labour and Welfare (Japan). Japan Vital Registration - Deaths 2012
Ministry of Health, Labour and Welfare (Japan). Japan Vital Registration - Deaths 2013
Ministry of Health, Labour and Welfare (Japan). Japan Vital Registration - Deaths 2014
Ministry of Health, Labour and Welfare (Japan). Japan Vital Registration - Deaths 2015
Ministry of Public Health (Thailand). Thailand Burden of Disease and Injuries 1998-1999
Ministry of Public Health (Uruguay). Uruguay Vital Registration - Deaths 1991

Moldova Vital Registration - Deaths 2010 ICD10 as it appears in World Health Organization (WHO). WHO Mortality Database Version October 2017. Geneva, Switzerland: World Health Organization (WHO), 2017

Moldova Vital Registration - Deaths 2011 ICD10 as it appears in World Health Organization (WHO). WHO Mortality Database Version October 2017. Geneva, Switzerland: World Health Organization (WHO), 2017

Moldova Vital Registration - Deaths 2012 ICD10 as it appears in World Health Organization (WHO). WHO Mortality Database Version October 2017. Geneva, Switzerland: World Health Organization (WHO), 2017

Moldova Vital Registration - Deaths 2013 ICD10 as it appears in World Health Organization (WHO). WHO Mortality Database Version October 2017. Geneva, Switzerland: World Health Organization (WHO), 2017

Moldova Vital Registration - Deaths 2014 ICD10 as it appears in World Health Organization (WHO). WHO Mortality Database Version October 2017. Geneva, Switzerland: World Health Organization (WHO), 2017

Moldova Vital Registration - Deaths 2015 ICD10 as it appears in World Health Organization (WHO). WHO Mortality Database Version March 2017. Geneva, Switzerland: World Health Organization (WHO), 2017

Moldova Vital Registration - Deaths 2016 ICD10 as it appears in World Health Organization (WHO). WHO Mortality Database Version April 2018. Geneva, Switzerland: World Health Organization (WHO), 2018

Mongolia Vital Registration - Deaths 1994 ICD9 as it appears in World Health Organization (WHO). WHO Mortality Database Version September 2016. Geneva, Switzerland: World Health Organization (WHO), 2016

Mongolia Vital Registration - Deaths 2016 ICD10 as it appears in World Health Organization (WHO). WHO Mortality Database Version April 2018. Geneva, Switzerland: World Health Organization (WHO), 2018

Montenegro Vital Registration - Deaths 2001 ICD10 as it appears in World Health Organization (WHO). WHO Mortality Database Version October 2017. Geneva, Switzerland: World Health Organization (WHO), 2017

Montenegro Vital Registration - Deaths 2002 ICD10 as it appears in World Health Organization (WHO). WHO Mortality Database Version October 2017. Geneva, Switzerland: World Health Organization (WHO), 2017

Montenegro Vital Registration - Deaths 2005 ICD10 as it appears in World Health Organization (WHO). WHO Mortality Database Version April 2018. Geneva, Switzerland: World Health Organization (WHO), 2018

Montenegro Vital Registration - Deaths 2006 ICD10 as it appears in World Health Organization (WHO). WHO Mortality Database Version April 2018. Geneva, Switzerland: World Health Organization (WHO), 2018

Montenegro Vital Registration - Deaths 2007 ICD10 as it appears in World Health Organization (WHO). WHO Mortality Database Version April 2018. Geneva, Switzerland: World Health Organization (WHO), 2018

Montenegro Vital Registration - Deaths 2008 ICD10 as it appears in World Health Organization (WHO). WHO Mortality Database Version April 2018. Geneva, Switzerland: World Health Organization (WHO), 2018

Montenegro Vital Registration - Deaths 2009 ICD10 as it appears in World Health Organization (WHO). WHO Mortality Database Version October 2017. Geneva, Switzerland: World Health Organization (WHO), 2017

Myint, S, Ministry of Health (Myanmar). Cause of Death Verification Study in Myanmar. Presentation at: World Health Organization Regional Office for South East Asia. Regional Consultation on Mortality Statistics; 2007; New Delhi, India

National Administrative Department of Statistics (DANE) (Colombia). Colombia Vital Statistics - Deaths 2008. Bogotá, Colombia: National Administrative Department of Statistics (DANE) (Colombia)

National Board of Health and Welfare (Sweden). Sweden - Stockholm County Vital Registration - Deaths 1980

National Board of Health and Welfare (Sweden). Sweden - Stockholm County Vital Registration - Deaths 1981

National Board of Health and Welfare (Sweden). Sweden - Stockholm County Vital Registration - Deaths 1982

National Board of Health and Welfare (Sweden). Sweden - Stockholm County Vital Registration - Deaths 1983

National Board of Health and Welfare (Sweden). Sweden - Stockholm County Vital Registration - Deaths 1984
National Board of Health and Welfare (Sweden). Sweden - Stockholm County Vital Registration - Deaths 1985
National Board of Health and Welfare (Sweden). Sweden - Stockholm County Vital Registration - Deaths 1986
National Board of Health and Welfare (Sweden). Sweden Cause of Death Register 1987
National Board of Health and Welfare (Sweden). Sweden Cause of Death Register 1988
National Board of Health and Welfare (Sweden). Sweden Cause of Death Register 1989
National Board of Health and Welfare (Sweden). Sweden Cause of Death Register 1990
National Board of Health and Welfare (Sweden). Sweden Cause of Death Register 1991
National Board of Health and Welfare (Sweden). Sweden Cause of Death Register 1992
National Board of Health and Welfare (Sweden). Sweden Cause of Death Register 1993
National Board of Health and Welfare (Sweden). Sweden Cause of Death Register 1994
National Board of Health and Welfare (Sweden). Sweden Cause of Death Register 1995
National Board of Health and Welfare (Sweden). Sweden Cause of Death Register 1996
National Board of Health and Welfare (Sweden). Sweden Cause of Death Register 1997
National Board of Health and Welfare (Sweden). Sweden Cause of Death Register 1998
National Board of Health and Welfare (Sweden). Sweden Cause of Death Register 1999
National Board of Health and Welfare (Sweden). Sweden Cause of Death Register 2000
National Board of Health and Welfare (Sweden). Sweden Cause of Death Register 2001
National Board of Health and Welfare (Sweden). Sweden Cause of Death Register 2002
National Board of Health and Welfare (Sweden). Sweden Cause of Death Register 2003
National Board of Health and Welfare (Sweden). Sweden Cause of Death Register 2004
National Board of Health and Welfare (Sweden). Sweden Cause of Death Register 2005
National Board of Health and Welfare (Sweden). Sweden Cause of Death Register 2006
National Board of Health and Welfare (Sweden). Sweden Cause of Death Register 2007
National Board of Health and Welfare (Sweden). Sweden Cause of Death Register 2008
National Board of Health and Welfare (Sweden). Sweden Cause of Death Register 2009
National Board of Health and Welfare (Sweden). Sweden Cause of Death Register 2010
National Board of Health and Welfare (Sweden). Sweden Cause of Death Register 2011
National Board of Health and Welfare (Sweden). Sweden Cause of Death Register 2012. Stockholm, Sweden: National Board of Health and Welfare (Sweden)
National Board of Health and Welfare (Sweden). Sweden Cause of Death Register 2013. Stockholm, Sweden: National Board of Health and Welfare (Sweden)
National Board of Health and Welfare (Sweden). Sweden Cause of Death Register 2014. Stockholm, Sweden: National Board of Health and Welfare (Sweden)
National Board of Health and Welfare (Sweden). Sweden Cause of Death Register 2015. Stockholm, Sweden: National Board of Health and Welfare (Sweden)
National Board of Health and Welfare (Sweden). Sweden Cause of Death Register 2016. Stockholm, Sweden: National Board of Health and Welfare (Sweden)
National Board of Health and Welfare (Sweden). Sweden Vital Registration - Deaths 1980
National Board of Health and Welfare (Sweden). Sweden Vital Registration - Deaths 1981

National Board of Health and Welfare (Sweden). Sweden Vital Registration - Deaths 1982
National Board of Health and Welfare (Sweden). Sweden Vital Registration - Deaths 1983
National Board of Health and Welfare (Sweden). Sweden Vital Registration - Deaths 1984
National Board of Health and Welfare (Sweden). Sweden Vital Registration - Deaths 1985
National Board of Health and Welfare (Sweden). Sweden Vital Registration - Deaths 1986
National Center for Health Statistics (NCHS), Centers for Disease Control and Prevention (CDC), U.S. Department of Defense. United States NVSS Custom Mortality Data 2005 and United States Military Deaths 1980-2014
National Center for Health Statistics (NCHS), Centers for Disease Control and Prevention (CDC), U.S. Department of Defense. United States NVSS Custom Mortality Data 2006 and United States Military Deaths 1980-2014
National Center for Health Statistics (NCHS), Centers for Disease Control and Prevention (CDC), U.S. Department of Defense. United States NVSS Custom Mortality Data 2007 and United States Military Deaths 1980-2014
National Center for Health Statistics (NCHS), Centers for Disease Control and Prevention (CDC), U.S. Department of Defense. United States NVSS Custom Mortality Data 2008 and United States Military Deaths 1980-2014
National Center for Health Statistics (NCHS), Centers for Disease Control and Prevention (CDC), U.S. Department of Defense. United States NVSS Custom Mortality Data 2009 and United States Military Deaths 1980-2014
National Center for Health Statistics (NCHS), Centers for Disease Control and Prevention (CDC), U.S. Department of Defense. United States NVSS Custom Mortality Data 2010 and United States Military Deaths 1980-2014
National Center for Health Statistics (NCHS), Centers for Disease Control and Prevention (CDC), U.S. Department of Defense. United States NVSS Custom Mortality Data 2011 and United States Military Deaths 1980-2014
National Center for Health Statistics (NCHS), Centers for Disease Control and Prevention (CDC), U.S. Department of Defense. United States NVSS Custom Mortality Data 2012 and United States Military Deaths 1980-2014
National Center for Health Statistics (NCHS), Centers for Disease Control and Prevention (CDC), U.S. Department of Defense. United States NVSS Custom Mortality Data 2013 and United States Military Deaths 1980-2014
National Center for Health Statistics (NCHS), Centers for Disease Control and Prevention (CDC), U.S. Department of Defense. United States NVSS Custom Mortality Data 2014 and United States Military Deaths 1980-2014
National Center for Health Statistics (NCHS), Centers for Disease Control and Prevention (CDC), U.S. Department of Defense. United States NVSS Mortality Data 1980 - NBER and United States Military Deaths 1980-2014
National Center for Health Statistics (NCHS), Centers for Disease Control and Prevention (CDC), U.S. Department of Defense. United States NVSS Mortality Data 1981 - NBER and United States Military Deaths 1980-2014
National Center for Health Statistics (NCHS), Centers for Disease Control and Prevention (CDC), U.S. Department of Defense. United States NVSS Mortality Data 1982 - NBER and United States Military Deaths 1980-2014
National Center for Health Statistics (NCHS), Centers for Disease Control and Prevention (CDC), U.S. Department of Defense. United States NVSS Mortality Data 1983 - NBER and United States Military Deaths 1980-2014
National Center for Health Statistics (NCHS), Centers for Disease Control and Prevention (CDC), U.S. Department of Defense. United States NVSS Mortality Data 1984 - NBER and United States Military Deaths 1980-2014
National Center for Health Statistics (NCHS), Centers for Disease Control and Prevention (CDC), U.S. Department of Defense. United States NVSS Mortality Data 1985 - NBER and United States Military Deaths 1980-2014
National Center for Health Statistics (NCHS), Centers for Disease Control and Prevention (CDC), U.S. Department of Defense. United States NVSS Mortality Data 1986 - NBER and United States Military Deaths 1980-2014

National Center for Health Statistics (NCHS), Centers for Disease Control and Prevention (CDC), U.S. Department of Defense. United States NVSS Mortality Data 1987 - NBER and United States Military Deaths 1980-2014
National Center for Health Statistics (NCHS), Centers for Disease Control and Prevention (CDC), U.S. Department of Defense. United States NVSS Mortality Data 1988 - NBER and United States Military Deaths 1980-2014
National Center for Health Statistics (NCHS), Centers for Disease Control and Prevention (CDC), U.S. Department of Defense. United States NVSS Mortality Data 1989 - NBER and United States Military Deaths 1980-2014
National Center for Health Statistics (NCHS), Centers for Disease Control and Prevention (CDC), U.S. Department of Defense. United States NVSS Mortality Data 1990 - NBER and United States Military Deaths 1980-2014
National Center for Health Statistics (NCHS), Centers for Disease Control and Prevention (CDC), U.S. Department of Defense. United States NVSS Mortality Data 1991 - NBER and United States Military Deaths 1980-2014
National Center for Health Statistics (NCHS), Centers for Disease Control and Prevention (CDC), U.S. Department of Defense. United States NVSS Mortality Data 1992 - NBER and United States Military Deaths 1980-2014
National Center for Health Statistics (NCHS), Centers for Disease Control and Prevention (CDC), U.S. Department of Defense. United States NVSS Mortality Data 1993 - NBER and United States Military Deaths 1980-2014
National Center for Health Statistics (NCHS), Centers for Disease Control and Prevention (CDC), U.S. Department of Defense. United States NVSS Mortality Data 1994 - NBER and United States Military Deaths 1980-2014
National Center for Health Statistics (NCHS), Centers for Disease Control and Prevention (CDC), U.S. Department of Defense. United States NVSS Mortality Data 1995 - NBER and United States Military Deaths 1980-2014
National Center for Health Statistics (NCHS), Centers for Disease Control and Prevention (CDC), U.S. Department of Defense. United States NVSS Mortality Data 1996 - NBER and United States Military Deaths 1980-2014
National Center for Health Statistics (NCHS), Centers for Disease Control and Prevention (CDC), U.S. Department of Defense. United States NVSS Mortality Data 1997 - NBER and United States Military Deaths 1980-2014
National Center for Health Statistics (NCHS), Centers for Disease Control and Prevention (CDC), U.S. Department of Defense. United States NVSS Mortality Data 1998 - NBER and United States Military Deaths 1980-2014
National Center for Health Statistics (NCHS), Centers for Disease Control and Prevention (CDC), U.S. Department of Defense. United States NVSS Mortality Data 1999 - NBER and United States Military Deaths 1980-2014
National Center for Health Statistics (NCHS), Centers for Disease Control and Prevention (CDC), U.S. Department of Defense. United States NVSS Mortality Data 2000 - NBER and United States Military Deaths 1980-2014
National Center for Health Statistics (NCHS), Centers for Disease Control and Prevention (CDC), U.S. Department of Defense. United States NVSS Mortality Data 2001 - NBER and United States Military Deaths 1980-2014
National Center for Health Statistics (NCHS), Centers for Disease Control and Prevention (CDC), U.S. Department of Defense. United States NVSS Mortality Data 2002 - NBER and United States Military Deaths 1980-2014
National Center for Health Statistics (NCHS), Centers for Disease Control and Prevention (CDC), U.S. Department of Defense. United States NVSS Mortality Data 2003 - NBER and United States Military Deaths 1980-2014
National Center for Health Statistics (NCHS), Centers for Disease Control and Prevention (CDC), U.S. Department of Defense. United States NVSS Mortality Data 2004 - NBER and United States Military Deaths 1980-2014
National Center for Health Statistics, Centers for Disease Control and Prevention. American Samoa NVSS Custom Mortality Data 2005. Hyattsville, United States: National Center for Health Statistics, Centers for Disease Control and Prevention
National Center for Health Statistics, Centers for Disease Control and Prevention. American Samoa NVSS Custom Mortality Data 2006. Hyattsville, United States: National Center for Health Statistics, Centers for Disease Control and Prevention

National Center for Health Statistics, Centers for Disease Control and Prevention. American Samoa NVSS Custom Mortality Data 2007. Hyattsville, United States: National Center for Health Statistics, Centers for Disease Control and Prevention

National Center for Health Statistics, Centers for Disease Control and Prevention. American Samoa NVSS Custom Mortality Data 2008. Hyattsville, United States: National Center for Health Statistics, Centers for Disease Control and Prevention

National Center for Health Statistics, Centers for Disease Control and Prevention. American Samoa NVSS Custom Mortality Data 2009. Hyattsville, United States: National Center for Health Statistics, Centers for Disease Control and Prevention

National Center for Health Statistics, Centers for Disease Control and Prevention. American Samoa NVSS Custom Mortality Data 2010. Hyattsville, United States: National Center for Health Statistics, Centers for Disease Control and Prevention

National Center for Health Statistics, Centers for Disease Control and Prevention. American Samoa NVSS Custom Mortality Data 2011. Hyattsville, United States: National Center for Health Statistics, Centers for Disease Control and Prevention

National Center for Health Statistics, Centers for Disease Control and Prevention. American Samoa NVSS Custom Mortality Data 2012. Hyattsville, United States: National Center for Health Statistics, Centers for Disease Control and Prevention

National Center for Health Statistics, Centers for Disease Control and Prevention. American Samoa NVSS Custom Mortality Data 2013. Hyattsville, United States: National Center for Health Statistics, Centers for Disease Control and Prevention

National Center for Health Statistics, Centers for Disease Control and Prevention. American Samoa NVSS Custom Mortality Data 2014. Hyattsville, United States: National Center for Health Statistics, Centers for Disease Control and Prevention

National Center for Health Statistics, Centers for Disease Control and Prevention. American Samoa NVSS Custom Mortality Data 2015. Hyattsville, United States: National Center for Health Statistics, Centers for Disease Control and Prevention

National Center for Health Statistics, Centers for Disease Control and Prevention. American Samoa NVSS Mortality Data 1997 - NBER. Hyattsville, United States: National Center for Health Statistics, Centers for Disease Control and Prevention

National Center for Health Statistics, Centers for Disease Control and Prevention. American Samoa NVSS Mortality Data 1998 - NBER. Hyattsville, United States: National Center for Health Statistics, Centers for Disease Control and Prevention

National Center for Health Statistics, Centers for Disease Control and Prevention. American Samoa NVSS Mortality Data 1999 - NBER. Hyattsville, United States: National Center for Health Statistics, Centers for Disease Control and Prevention

National Center for Health Statistics, Centers for Disease Control and Prevention. American Samoa NVSS Mortality Data 2000 - NBER. Hyattsville, United States: National Center for Health Statistics, Centers for Disease Control and Prevention

National Center for Health Statistics, Centers for Disease Control and Prevention. American Samoa NVSS Mortality Data 2001 - NBER. Hyattsville, United States: National Center for Health Statistics, Centers for Disease Control and Prevention

National Center for Health Statistics, Centers for Disease Control and Prevention. American Samoa NVSS Mortality Data 2002 - NBER. Hyattsville, United States: National Center for Health Statistics, Centers for Disease Control and Prevention

National Center for Health Statistics, Centers for Disease Control and Prevention. American Samoa NVSS Mortality Data 2003 - NBER. Hyattsville, United States: National Center for Health Statistics, Centers for Disease Control and Prevention

National Center for Health Statistics, Centers for Disease Control and Prevention. American Samoa NVSS Mortality Data 2004 - NBER. Hyattsville, United States: National Center for Health Statistics, Centers for Disease Control and Prevention

National Center for Health Statistics, Centers for Disease Control and Prevention. Guam NVSS Custom Mortality Data 2005. Hyattsville, United States: National Center for Health Statistics, Centers for Disease Control and Prevention

National Center for Health Statistics, Centers for Disease Control and Prevention. Guam NVSS Custom Mortality Data 2006. Hyattsville, United States: National Center for Health Statistics, Centers for Disease Control and Prevention

National Center for Health Statistics, Centers for Disease Control and Prevention. Guam NVSS Custom Mortality Data 2007. Hyattsville, United States: National Center for Health Statistics, Centers for Disease Control and Prevention

National Center for Health Statistics, Centers for Disease Control and Prevention. Guam NVSS Custom Mortality Data 2008. Hyattsville, United States: National Center for Health Statistics, Centers for Disease Control and Prevention

National Center for Health Statistics, Centers for Disease Control and Prevention. Guam NVSS Custom Mortality Data 2009. Hyattsville, United States: National Center for Health Statistics, Centers for Disease Control and Prevention

National Center for Health Statistics, Centers for Disease Control and Prevention. Guam NVSS Custom Mortality Data 2010. Hyattsville, United States: National Center for Health Statistics, Centers for Disease Control and Prevention

National Center for Health Statistics, Centers for Disease Control and Prevention. Guam NVSS Custom Mortality Data 2011. Hyattsville, United States: National Center for Health Statistics, Centers for Disease Control and Prevention

National Center for Health Statistics, Centers for Disease Control and Prevention. Guam NVSS Custom Mortality Data 2012. Hyattsville, United States: National Center for Health Statistics, Centers for Disease Control and Prevention

National Center for Health Statistics, Centers for Disease Control and Prevention. Guam NVSS Custom Mortality Data 2013. Hyattsville, United States: National Center for Health Statistics, Centers for Disease Control and Prevention

National Center for Health Statistics, Centers for Disease Control and Prevention. Guam NVSS Custom Mortality Data 2014. Hyattsville, United States: National Center for Health Statistics, Centers for Disease Control and Prevention

National Center for Health Statistics, Centers for Disease Control and Prevention. Guam NVSS Custom Mortality Data 2015. Hyattsville, United States: National Center for Health Statistics, Centers for Disease Control and Prevention

National Center for Health Statistics, Centers for Disease Control and Prevention. Guam NVSS Mortality Data 1994 - NBER. Hyattsville, United States: National Center for Health Statistics, Centers for Disease Control and Prevention

National Center for Health Statistics, Centers for Disease Control and Prevention. Guam NVSS Mortality Data 1995 - NBER. Hyattsville, United States: National Center for Health Statistics, Centers for Disease Control and Prevention

National Center for Health Statistics, Centers for Disease Control and Prevention. Guam NVSS Mortality Data 1996 - NBER. Hyattsville, United States: National Center for Health Statistics, Centers for Disease Control and Prevention

National Center for Health Statistics, Centers for Disease Control and Prevention. Guam NVSS Mortality Data 1997 - NBER. Hyattsville, United States: National Center for Health Statistics, Centers for Disease Control and Prevention

National Center for Health Statistics, Centers for Disease Control and Prevention. Guam NVSS Mortality Data 1998 - NBER. Hyattsville, United States: National Center for Health Statistics, Centers for Disease Control and Prevention

National Center for Health Statistics, Centers for Disease Control and Prevention. Guam NVSS Mortality Data 1999 - NBER. Hyattsville, United States: National Center for Health Statistics, Centers for Disease Control and Prevention

National Center for Health Statistics, Centers for Disease Control and Prevention. Guam NVSS Mortality Data 2000 - NBER. Hyattsville, United States: National Center for Health Statistics, Centers for Disease Control and Prevention

National Center for Health Statistics, Centers for Disease Control and Prevention. Guam NVSS Mortality Data 2001 - NBER. Hyattsville, United States: National Center for Health Statistics, Centers for Disease Control and Prevention

National Center for Health Statistics, Centers for Disease Control and Prevention. Guam NVSS Mortality Data 2002 - NBER. Hyattsville, United States: National Center for Health Statistics, Centers for Disease Control and Prevention

National Center for Health Statistics, Centers for Disease Control and Prevention. Guam NVSS Mortality Data 2003 - NBER. Hyattsville, United States: National Center for Health Statistics, Centers for Disease Control and Prevention

National Center for Health Statistics, Centers for Disease Control and Prevention. Guam NVSS Mortality Data 2004 - NBER. Hyattsville, United States: National Center for Health Statistics, Centers for Disease Control and Prevention

National Center for Health Statistics, Centers for Disease Control and Prevention. Northern Mariana Islands NVSS Custom Mortality Data 2005. Hyattsville, United States: National Center for Health Statistics, Centers for Disease Control and Prevention

National Center for Health Statistics, Centers for Disease Control and Prevention. Northern Mariana Islands NVSS Custom Mortality Data 2006. Hyattsville, United States: National Center for Health Statistics, Centers for Disease Control and Prevention

National Center for Health Statistics, Centers for Disease Control and Prevention. Northern Mariana Islands NVSS Custom Mortality Data 2007. Hyattsville, United States: National Center for Health Statistics, Centers for Disease Control and Prevention

National Center for Health Statistics, Centers for Disease Control and Prevention. Northern Mariana Islands NVSS Custom Mortality Data 2008. Hyattsville, United States: National Center for Health Statistics, Centers for Disease Control and Prevention

National Center for Health Statistics, Centers for Disease Control and Prevention. Northern Mariana Islands NVSS Custom Mortality Data 2009. Hyattsville, United States: National Center for Health Statistics, Centers for Disease Control and Prevention

National Center for Health Statistics, Centers for Disease Control and Prevention. Northern Mariana Islands NVSS Custom Mortality Data 2010. Hyattsville, United States: National Center for Health Statistics, Centers for Disease Control and Prevention

National Center for Health Statistics, Centers for Disease Control and Prevention. Northern Mariana Islands NVSS Custom Mortality Data 2011. Hyattsville, United States: National Center for Health Statistics, Centers for Disease Control and Prevention

National Center for Health Statistics, Centers for Disease Control and Prevention. Northern Mariana Islands NVSS Custom Mortality Data 2012. Hyattsville, United States: National Center for Health Statistics, Centers for Disease Control and Prevention

National Center for Health Statistics, Centers for Disease Control and Prevention. Northern Mariana Islands NVSS Custom Mortality Data 2013. Hyattsville, United States: National Center for Health Statistics, Centers for Disease Control and Prevention

National Center for Health Statistics, Centers for Disease Control and Prevention. Northern Mariana Islands NVSS Custom Mortality Data 2014. Hyattsville, United States: National Center for Health Statistics, Centers for Disease Control and Prevention

National Center for Health Statistics, Centers for Disease Control and Prevention. Northern Mariana Islands NVSS Custom Mortality Data 2015. Hyattsville, United States: National Center for Health Statistics, Centers for Disease Control and Prevention

National Center for Health Statistics, Centers for Disease Control and Prevention. Northern Mariana Islands NVSS Mortality Data 1998 - NBER. Hyattsville, United States: National Center for Health Statistics, Centers for Disease Control and Prevention

National Center for Health Statistics, Centers for Disease Control and Prevention. Northern Mariana Islands NVSS Mortality Data 1999 - NBER. Hyattsville, United States: National Center for Health Statistics, Centers for Disease Control and Prevention

National Center for Health Statistics, Centers for Disease Control and Prevention. Northern Mariana Islands NVSS Mortality Data 2000 - NBER. Hyattsville, United States: National Center for Health Statistics, Centers for Disease Control and Prevention

National Center for Health Statistics, Centers for Disease Control and Prevention. Northern Mariana Islands NVSS Mortality Data 2001 - NBER. Hyattsville, United States: National Center for Health Statistics, Centers for Disease Control and Prevention

National Center for Health Statistics, Centers for Disease Control and Prevention. Northern Mariana Islands NVSS Mortality Data 2002 - NBER. Hyattsville, United States: National Center for Health Statistics, Centers for Disease Control and Prevention

National Center for Health Statistics, Centers for Disease Control and Prevention. Northern Mariana Islands NVSS Mortality Data 2003 - NBER. Hyattsville, United States: National Center for Health Statistics, Centers for Disease Control and Prevention

National Center for Health Statistics, Centers for Disease Control and Prevention. Northern Mariana Islands NVSS Mortality Data 2004 - NBER. Hyattsville, United States: National Center for Health Statistics, Centers for Disease Control and Prevention

National Center for Health Statistics, Centers for Disease Control and Prevention. Puerto Rico NVSS Custom Mortality Data 2005. Hyattsville, United States: National Center for Health Statistics, Centers for Disease Control and Prevention

National Center for Health Statistics, Centers for Disease Control and Prevention. Puerto Rico NVSS Custom Mortality Data 2006. Hyattsville, United States: National Center for Health Statistics, Centers for Disease Control and Prevention

National Center for Health Statistics, Centers for Disease Control and Prevention. Puerto Rico NVSS Custom Mortality Data 2007. Hyattsville, United States: National Center for Health Statistics, Centers for Disease Control and Prevention

National Center for Health Statistics, Centers for Disease Control and Prevention. Puerto Rico NVSS Custom Mortality Data 2008. Hyattsville, United States: National Center for Health Statistics, Centers for Disease Control and Prevention
National Center for Health Statistics, Centers for Disease Control and Prevention. Puerto Rico NVSS Custom Mortality Data 2009. Hyattsville, United States: National Center for Health Statistics, Centers for Disease Control and Prevention
National Center for Health Statistics, Centers for Disease Control and Prevention. Puerto Rico NVSS Custom Mortality Data 2010. Hyattsville, United States: National Center for Health Statistics, Centers for Disease Control and Prevention
National Center for Health Statistics, Centers for Disease Control and Prevention. Puerto Rico NVSS Custom Mortality Data 2011. Hyattsville, United States: National Center for Health Statistics, Centers for Disease Control and Prevention
National Center for Health Statistics, Centers for Disease Control and Prevention. Puerto Rico NVSS Custom Mortality Data 2012. Hyattsville, United States: National Center for Health Statistics, Centers for Disease Control and Prevention
National Center for Health Statistics, Centers for Disease Control and Prevention. Puerto Rico NVSS Custom Mortality Data 2013. Hyattsville, United States: National Center for Health Statistics, Centers for Disease Control and Prevention
National Center for Health Statistics, Centers for Disease Control and Prevention. Puerto Rico NVSS Custom Mortality Data 2014. Hyattsville, United States: National Center for Health Statistics, Centers for Disease Control and Prevention
National Center for Health Statistics, Centers for Disease Control and Prevention. Puerto Rico NVSS Custom Mortality Data 2015. Hyattsville, United States: National Center for Health Statistics, Centers for Disease Control and Prevention
National Center for Health Statistics, Centers for Disease Control and Prevention. Puerto Rico NVSS Mortality Data 1994 - NBER. Hyattsville, United States: National Center for Health Statistics, Centers for Disease Control and Prevention
National Center for Health Statistics, Centers for Disease Control and Prevention. Puerto Rico NVSS Mortality Data 1995 - NBER. Hyattsville, United States: National Center for Health Statistics, Centers for Disease Control and Prevention
National Center for Health Statistics, Centers for Disease Control and Prevention. Puerto Rico NVSS Mortality Data 1996 - NBER. Hyattsville, United States: National Center for Health Statistics, Centers for Disease Control and Prevention
National Center for Health Statistics, Centers for Disease Control and Prevention. Puerto Rico NVSS Mortality Data 1997 - NBER. Hyattsville, United States: National Center for Health Statistics, Centers for Disease Control and Prevention
National Center for Health Statistics, Centers for Disease Control and Prevention. Puerto Rico NVSS Mortality Data 1998 - NBER. Hyattsville, United States: National Center for Health Statistics, Centers for Disease Control and Prevention
National Center for Health Statistics, Centers for Disease Control and Prevention. Puerto Rico NVSS Mortality Data 1999 - NBER. Hyattsville, United States: National Center for Health Statistics, Centers for Disease Control and Prevention
National Center for Health Statistics, Centers for Disease Control and Prevention. Puerto Rico NVSS Mortality Data 2000 - NBER. Hyattsville, United States: National Center for Health Statistics, Centers for Disease Control and Prevention
National Center for Health Statistics, Centers for Disease Control and Prevention. Puerto Rico NVSS Mortality Data 2001 - NBER. Hyattsville, United States: National Center for Health Statistics, Centers for Disease Control and Prevention
National Center for Health Statistics, Centers for Disease Control and Prevention. Puerto Rico NVSS Mortality Data 2002 - NBER. Hyattsville, United States: National Center for Health Statistics, Centers for Disease Control and Prevention
National Center for Health Statistics, Centers for Disease Control and Prevention. Puerto Rico NVSS Mortality Data 2003 - NBER. Hyattsville, United States: National Center for Health Statistics, Centers for Disease Control and Prevention
National Center for Health Statistics, Centers for Disease Control and Prevention. Puerto Rico NVSS Mortality Data 2004 - NBER. Hyattsville, United States: National Center for Health Statistics, Centers for Disease Control and Prevention
National Center for Health Statistics, Centers for Disease Control and Prevention. United States NVSS Custom Mortality Data 2015. Hyattsville, United States: National Center for Health Statistics, Centers for Disease Control and Prevention

National Center for Health Statistics, Centers for Disease Control and Prevention. United States Virgin Islands NVSS Custom Mortality Data 2005. Hyattsville, United States: National Center for Health Statistics, Centers for Disease Control and Prevention

National Center for Health Statistics, Centers for Disease Control and Prevention. United States Virgin Islands NVSS Custom Mortality Data 2006. Hyattsville, United States: National Center for Health Statistics, Centers for Disease Control and Prevention

National Center for Health Statistics, Centers for Disease Control and Prevention. United States Virgin Islands NVSS Custom Mortality Data 2007. Hyattsville, United States: National Center for Health Statistics, Centers for Disease Control and Prevention

National Center for Health Statistics, Centers for Disease Control and Prevention. United States Virgin Islands NVSS Custom Mortality Data 2008. Hyattsville, United States: National Center for Health Statistics, Centers for Disease Control and Prevention

National Center for Health Statistics, Centers for Disease Control and Prevention. United States Virgin Islands NVSS Custom Mortality Data 2009. Hyattsville, United States: National Center for Health Statistics, Centers for Disease Control and Prevention

National Center for Health Statistics, Centers for Disease Control and Prevention. United States Virgin Islands NVSS Custom Mortality Data 2010. Hyattsville, United States: National Center for Health Statistics, Centers for Disease Control and Prevention

National Center for Health Statistics, Centers for Disease Control and Prevention. United States Virgin Islands NVSS Custom Mortality Data 2011. Hyattsville, United States: National Center for Health Statistics, Centers for Disease Control and Prevention

National Center for Health Statistics, Centers for Disease Control and Prevention. United States Virgin Islands NVSS Custom Mortality Data 2012. Hyattsville, United States: National Center for Health Statistics, Centers for Disease Control and Prevention

National Center for Health Statistics, Centers for Disease Control and Prevention. United States Virgin Islands NVSS Custom Mortality Data 2015. Hyattsville, United States: National Center for Health Statistics, Centers for Disease Control and Prevention

National Center for Health Statistics, Centers for Disease Control and Prevention. United States Virgin Islands NVSS Mortality Data 1994 - NBER. Hyattsville, United States: National Center for Health Statistics, Centers for Disease Control and Prevention

National Center for Health Statistics, Centers for Disease Control and Prevention. United States Virgin Islands NVSS Mortality Data 1995 - NBER. Hyattsville, United States: National Center for Health Statistics, Centers for Disease Control and Prevention

National Center for Health Statistics, Centers for Disease Control and Prevention. United States Virgin Islands NVSS Mortality Data 1996 - NBER. Hyattsville, United States: National Center for Health Statistics, Centers for Disease Control and Prevention

National Center for Health Statistics, Centers for Disease Control and Prevention. United States Virgin Islands NVSS Mortality Data 1997 - NBER. Hyattsville, United States: National Center for Health Statistics, Centers for Disease Control and Prevention

National Center for Health Statistics, Centers for Disease Control and Prevention. United States Virgin Islands NVSS Mortality Data 1998 - NBER. Hyattsville, United States: National Center for Health Statistics, Centers for Disease Control and Prevention

National Center for Health Statistics, Centers for Disease Control and Prevention. United States Virgin Islands NVSS Mortality Data 1999 - NBER. Hyattsville, United States: National Center for Health Statistics, Centers for Disease Control and Prevention

National Center for Health Statistics, Centers for Disease Control and Prevention. United States Virgin Islands NVSS Mortality Data 2000 - NBER. Hyattsville, United States: National Center for Health Statistics, Centers for Disease Control and Prevention

National Center for Health Statistics, Centers for Disease Control and Prevention. United States Virgin Islands NVSS Mortality Data 2001 - NBER. Hyattsville, United States: National Center for Health Statistics, Centers for Disease Control and Prevention

National Center for Health Statistics, Centers for Disease Control and Prevention. United States Virgin Islands NVSS Mortality Data 2002 - NBER. Hyattsville, United States: National Center for Health Statistics, Centers for Disease Control and Prevention

National Center for Health Statistics, Centers for Disease Control and Prevention. United States Virgin Islands NVSS Mortality Data 2003 - NBER. Hyattsville, United States: National Center for Health Statistics, Centers for Disease Control and Prevention

National Center for Health Statistics, Centers for Disease Control and Prevention. United States Virgin Islands NVSS Mortality Data 2004 - NBER. Hyattsville, United States: National Center for Health Statistics, Centers for Disease Control and Prevention

National Institute of Statistics (Italy). Italy Vital Registration - Deaths 2015. Rome, Italy: National Institute of Statistics (Italy)

National Institute of Statistics and Geography (INEGI) (Mexico), Secretariat of Health (Mexico). Mexico Vital Registration - Deaths 1980
National Institute of Statistics and Geography (INEGI) (Mexico), Secretariat of Health (Mexico). Mexico Vital Registration - Deaths 1981
National Institute of Statistics and Geography (INEGI) (Mexico), Secretariat of Health (Mexico). Mexico Vital Registration - Deaths 1982
National Institute of Statistics and Geography (INEGI) (Mexico), Secretariat of Health (Mexico). Mexico Vital Registration - Deaths 1983
National Institute of Statistics and Geography (INEGI) (Mexico), Secretariat of Health (Mexico). Mexico Vital Registration - Deaths 1984
National Institute of Statistics and Geography (INEGI) (Mexico), Secretariat of Health (Mexico). Mexico Vital Registration - Deaths 1985
National Institute of Statistics and Geography (INEGI) (Mexico), Secretariat of Health (Mexico). Mexico Vital Registration - Deaths 1986
National Institute of Statistics and Geography (INEGI) (Mexico), Secretariat of Health (Mexico). Mexico Vital Registration - Deaths 1987
National Institute of Statistics and Geography (INEGI) (Mexico), Secretariat of Health (Mexico). Mexico Vital Registration - Deaths 1988
National Institute of Statistics and Geography (INEGI) (Mexico), Secretariat of Health (Mexico). Mexico Vital Registration - Deaths 1989
National Institute of Statistics and Geography (INEGI) (Mexico), Secretariat of Health (Mexico). Mexico Vital Registration - Deaths 1990
National Institute of Statistics and Geography (INEGI) (Mexico), Secretariat of Health (Mexico). Mexico Vital Registration - Deaths 1991
National Institute of Statistics and Geography (INEGI) (Mexico), Secretariat of Health (Mexico). Mexico Vital Registration - Deaths 1992
National Institute of Statistics and Geography (INEGI) (Mexico), Secretariat of Health (Mexico). Mexico Vital Registration - Deaths 1993
National Institute of Statistics and Geography (INEGI) (Mexico), Secretariat of Health (Mexico). Mexico Vital Registration - Deaths 1994
National Institute of Statistics and Geography (INEGI) (Mexico), Secretariat of Health (Mexico). Mexico Vital Registration - Deaths 1995
National Institute of Statistics and Geography (INEGI) (Mexico), Secretariat of Health (Mexico). Mexico Vital Registration - Deaths 1996
National Institute of Statistics and Geography (INEGI) (Mexico), Secretariat of Health (Mexico). Mexico Vital Registration - Deaths 1997
National Institute of Statistics and Geography (INEGI) (Mexico), Secretariat of Health (Mexico). Mexico Vital Registration - Deaths 1998
National Institute of Statistics and Geography (INEGI) (Mexico), Secretariat of Health (Mexico). Mexico Vital Registration - Deaths 1999
National Institute of Statistics and Geography (INEGI) (Mexico), Secretariat of Health (Mexico). Mexico Vital Registration - Deaths 2000
National Institute of Statistics and Geography (INEGI) (Mexico), Secretariat of Health (Mexico). Mexico Vital Registration - Deaths 2001
National Institute of Statistics and Geography (INEGI) (Mexico), Secretariat of Health (Mexico). Mexico Vital Registration - Deaths 2002
National Institute of Statistics and Geography (INEGI) (Mexico), Secretariat of Health (Mexico). Mexico Vital Registration - Deaths 2003
National Institute of Statistics and Geography (INEGI) (Mexico), Secretariat of Health (Mexico). Mexico Vital Registration - Deaths 2004
National Institute of Statistics and Geography (INEGI) (Mexico), Secretariat of Health (Mexico). Mexico Vital Registration - Deaths 2005
National Institute of Statistics and Geography (INEGI) (Mexico), Secretariat of Health (Mexico). Mexico Vital Registration - Deaths 2006
National Institute of Statistics and Geography (INEGI) (Mexico), Secretariat of Health (Mexico). Mexico Vital Registration - Deaths 2007
National Institute of Statistics and Geography (INEGI) (Mexico), Secretariat of Health (Mexico). Mexico Vital Registration - Deaths 2008
National Institute of Statistics and Geography (INEGI) (Mexico), Secretariat of Health (Mexico). Mexico Vital Registration - Deaths 2010
National Institute of Statistics and Geography (INEGI) (Mexico), Secretariat of Health (Mexico). Mexico Vital Registration - Deaths 2011
National Institute of Statistics and Geography (INEGI) (Mexico). Mexico Vital Registration - Deaths 2012
National Institute of Statistics and Geography (INEGI) (Mexico). Mexico Vital Registration - Deaths 2013
National Institute of Statistics and Geography (INEGI) (Mexico). Mexico Vital Registration - Deaths 2014. Mexico City, Mexico: National Institute of Statistics and Geography (INEGI) (Mexico)

National Institute of Statistics and Geography (INEGI) (Mexico). Mexico Vital Registration - Deaths 2015. Mexico City, Mexico: National Institute of Statistics and Geography (INEGI) (Mexico)

National Institute of Statistics and Geography (INEGI) (Mexico). Mexico Vital Registration - Deaths 2016. Mexico City, Mexico: National Institute of Statistics and Geography (INEGI) (Mexico)

National Institute of Statistics and Geography (INEGI) (Mexico). Mexico Vital Statistics - Deaths 2009

National Records of Scotland. United Kingdom - Scotland Vital Events Reference Tables 2015. Edinburgh, Scotland: National Records of Scotland, 2016

National Records of Scotland. United Kingdom - Scotland Vital Events Reference Tables 2016. Edinburgh, Scotland: National Records of Scotland, 2017

National Statistics Office (Philippines). Philippines Vital Registration - Deaths 2012

Navrongo Health Research Centre. Ghana - Navrongo Health and Demographic Surveillance System

Netherlands Vital Registration - Deaths 1980 ICD9 as it appears in World Health Organization (WHO). WHO Mortality Database Version November 2015. Geneva, Switzerland: World Health Organization (WHO), 2015

Netherlands Vital Registration - Deaths 1981 ICD9 as it appears in World Health Organization (WHO). WHO Mortality Database Version November 2015. Geneva, Switzerland: World Health Organization (WHO), 2015

Netherlands Vital Registration - Deaths 1982 ICD9 as it appears in World Health Organization (WHO). WHO Mortality Database Version November 2015. Geneva, Switzerland: World Health Organization (WHO), 2015

Netherlands Vital Registration - Deaths 1983 ICD9 as it appears in World Health Organization (WHO). WHO Mortality Database Version November 2015. Geneva, Switzerland: World Health Organization (WHO), 2015

Netherlands Vital Registration - Deaths 1984 ICD9 as it appears in World Health Organization (WHO). WHO Mortality Database Version November 2015. Geneva, Switzerland: World Health Organization (WHO), 2015

Netherlands Vital Registration - Deaths 1986 ICD9 as it appears in World Health Organization (WHO). WHO Mortality Database Version November 2015. Geneva, Switzerland: World Health Organization (WHO), 2015

Netherlands Vital Registration - Deaths 1987 ICD9 as it appears in World Health Organization (WHO). WHO Mortality Database Version November 2015. Geneva, Switzerland: World Health Organization (WHO), 2015

Netherlands Vital Registration - Deaths 1989 ICD9 as it appears in World Health Organization (WHO). WHO Mortality Database Version November 2015. Geneva, Switzerland: World Health Organization (WHO), 2015

Netherlands Vital Registration - Deaths 1990 ICD9 as it appears in World Health Organization (WHO). WHO Mortality Database Version November 2015. Geneva, Switzerland: World Health Organization (WHO), 2015

Netherlands Vital Registration - Deaths 1991 ICD9 as it appears in World Health Organization (WHO). WHO Mortality Database Version November 2015. Geneva, Switzerland: World Health Organization (WHO), 2015

Netherlands Vital Registration - Deaths 1992 ICD9 as it appears in World Health Organization (WHO). WHO Mortality Database Version November 2015. Geneva, Switzerland: World Health Organization (WHO), 2015

Netherlands Vital Registration - Deaths 1993 ICD9 as it appears in World Health Organization (WHO). WHO Mortality Database Version November 2015. Geneva, Switzerland: World Health Organization (WHO), 2015

Netherlands Vital Registration - Deaths 1994 ICD9 as it appears in World Health Organization (WHO). WHO Mortality Database Version November 2015. Geneva, Switzerland: World Health Organization (WHO), 2015

Netherlands Vital Registration - Deaths 1995 ICD9 as it appears in World Health Organization (WHO). WHO Mortality Database Version November 2015. Geneva, Switzerland: World Health Organization (WHO), 2015

Netherlands Vital Registration - Deaths 1996 ICD10 as it appears in World Health Organization (WHO). WHO Mortality Database Version October 2017. Geneva, Switzerland: World Health Organization (WHO), 2017

Netherlands Vital Registration - Deaths 1997 ICD10 as it appears in World Health Organization (WHO). WHO Mortality Database Version October 2017. Geneva, Switzerland: World Health Organization (WHO), 2017
Netherlands Vital Registration - Deaths 1998 ICD10 as it appears in World Health Organization (WHO). WHO Mortality Database Version October 2017. Geneva, Switzerland: World Health Organization (WHO), 2017
Netherlands Vital Registration - Deaths 1999 ICD10 as it appears in World Health Organization (WHO). WHO Mortality Database Version October 2017. Geneva, Switzerland: World Health Organization (WHO), 2017
Netherlands Vital Registration - Deaths 2000 ICD10 as it appears in World Health Organization (WHO). WHO Mortality Database Version October 2017. Geneva, Switzerland: World Health Organization (WHO), 2017
Netherlands Vital Registration - Deaths 2001 ICD10 as it appears in World Health Organization (WHO). WHO Mortality Database Version October 2017. Geneva, Switzerland: World Health Organization (WHO), 2017
Netherlands Vital Registration - Deaths 2002 ICD10 as it appears in World Health Organization (WHO). WHO Mortality Database Version October 2017. Geneva, Switzerland: World Health Organization (WHO), 2017
Netherlands Vital Registration - Deaths 2003 ICD10 as it appears in World Health Organization (WHO). WHO Mortality Database Version October 2017. Geneva, Switzerland: World Health Organization (WHO), 2017
Netherlands Vital Registration - Deaths 2004 ICD10 as it appears in World Health Organization (WHO). WHO Mortality Database Version October 2017. Geneva, Switzerland: World Health Organization (WHO), 2017
Netherlands Vital Registration - Deaths 2005 ICD10 as it appears in World Health Organization (WHO). WHO Mortality Database Version October 2017. Geneva, Switzerland: World Health Organization (WHO), 2017
Netherlands Vital Registration - Deaths 2006 ICD10 as it appears in World Health Organization (WHO). WHO Mortality Database Version October 2017. Geneva, Switzerland: World Health Organization (WHO), 2017
Netherlands Vital Registration - Deaths 2007 ICD10 as it appears in World Health Organization (WHO). WHO Mortality Database Version October 2017. Geneva, Switzerland: World Health Organization (WHO), 2017
Netherlands Vital Registration - Deaths 2008 ICD10 as it appears in World Health Organization (WHO). WHO Mortality Database Version October 2017. Geneva, Switzerland: World Health Organization (WHO), 2017
Netherlands Vital Registration - Deaths 1985 ICD9 as it appears in World Health Organization (WHO). WHO Mortality Database Version November 2015. Geneva, Switzerland: World Health Organization (WHO), 2015
Netherlands Vital Registration - Deaths 1988 ICD9 as it appears in World Health Organization (WHO). WHO Mortality Database Version November 2015. Geneva, Switzerland: World Health Organization (WHO), 2015
Netherlands Vital Registration - Deaths 2009 ICD10 as it appears in World Health Organization (WHO). WHO Mortality Database Version October 2017. Geneva, Switzerland: World Health Organization (WHO), 2017
Netherlands Vital Registration - Deaths 2010 ICD10 as it appears in World Health Organization (WHO). WHO Mortality Database Version October 2017. Geneva, Switzerland: World Health Organization (WHO), 2017
Netherlands Vital Registration - Deaths 2011 ICD10 as it appears in World Health Organization (WHO). WHO Mortality Database Version October 2017. Geneva, Switzerland: World Health Organization (WHO), 2017
Netherlands Vital Registration - Deaths 2012 ICD10 as it appears in World Health Organization (WHO). WHO Mortality Database Version October 2017. Geneva, Switzerland: World Health Organization (WHO), 2017
Netherlands Vital Registration - Deaths 2013 ICD10 as it appears in World Health Organization (WHO). WHO Mortality Database Version October 2017. Geneva, Switzerland: World Health Organization (WHO), 2017
Netherlands Vital Registration - Deaths 2014 ICD10 as it appears in World Health Organization (WHO). WHO Mortality Database Version March 2017. Geneva, Switzerland: World Health Organization (WHO), 2017

Netherlands Vital Registration - Deaths 2015 ICD10 as it appears in World Health Organization (WHO). WHO Mortality Database Version March 2017. Geneva, Switzerland: World Health Organization (WHO), 2017

Netherlands Vital Registration - Deaths 2016 ICD10 as it appears in World Health Organization (WHO). WHO Mortality Database Version April 2018. Geneva, Switzerland: World Health Organization (WHO), 2018

Ngo AD, Rao C, Hoa NP, Adair T, Chuc NTK. Mortality patterns in Vietnam, 2006: Findings from a national verbal autopsy survey. BMC Res Notes. 2010; 3: 78

Nicaragua Vital Registration - Deaths 1988 ICD9 as it appears in World Health Organization (WHO). WHO Mortality Database Version September 2016. Geneva, Switzerland: World Health Organization (WHO), 2016

Nicaragua Vital Registration - Deaths 1989 ICD9 as it appears in World Health Organization (WHO). WHO Mortality Database Version September 2016. Geneva, Switzerland: World Health Organization (WHO), 2016

Nicaragua Vital Registration - Deaths 1990 ICD9 as it appears in World Health Organization (WHO). WHO Mortality Database Version September 2016. Geneva, Switzerland: World Health Organization (WHO), 2016

Nicaragua Vital Registration - Deaths 1991 ICD9 as it appears in World Health Organization (WHO). WHO Mortality Database Version September 2016. Geneva, Switzerland: World Health Organization (WHO), 2016

Nicaragua Vital Registration - Deaths 1992 ICD9 as it appears in World Health Organization (WHO). WHO Mortality Database Version September 2016. Geneva, Switzerland: World Health Organization (WHO), 2016

Nicaragua Vital Registration - Deaths 1993 ICD9 as it appears in World Health Organization (WHO). WHO Mortality Database Version September 2016. Geneva, Switzerland: World Health Organization (WHO), 2016

Nicaragua Vital Registration - Deaths 1994 ICD9 as it appears in World Health Organization (WHO). WHO Mortality Database Version September 2016. Geneva, Switzerland: World Health Organization (WHO), 2016

Nicaragua Vital Registration - Deaths 1996 ICD9 as it appears in World Health Organization (WHO). WHO Mortality Database Version September 2016. Geneva, Switzerland: World Health Organization (WHO), 2016

Nicaragua Vital Registration - Deaths 1997 ICD10 as it appears in World Health Organization (WHO). WHO Mortality Database Version October 2017. Geneva, Switzerland: World Health Organization (WHO), 2017

Nicaragua Vital Registration - Deaths 1998 ICD10 as it appears in World Health Organization (WHO). WHO Mortality Database Version October 2017. Geneva, Switzerland: World Health Organization (WHO), 2017

Nicaragua Vital Registration - Deaths 1999 ICD10 as it appears in World Health Organization (WHO). WHO Mortality Database Version October 2017. Geneva, Switzerland: World Health Organization (WHO), 2017

Nicaragua Vital Registration - Deaths 2000 ICD10 as it appears in World Health Organization (WHO). WHO Mortality Database Version October 2017. Geneva, Switzerland: World Health Organization (WHO), 2017

Nicaragua Vital Registration - Deaths 2001 ICD10 as it appears in World Health Organization (WHO). WHO Mortality Database Version October 2017. Geneva, Switzerland: World Health Organization (WHO), 2017

Nicaragua Vital Registration - Deaths 2002 ICD10 as it appears in World Health Organization (WHO). WHO Mortality Database Version October 2017. Geneva, Switzerland: World Health Organization (WHO), 2017

Nicaragua Vital Registration - Deaths 2003 ICD10 as it appears in World Health Organization (WHO). WHO Mortality Database Version October 2017. Geneva, Switzerland: World Health Organization (WHO), 2017

Nicaragua Vital Registration - Deaths 2004 ICD10 as it appears in World Health Organization (WHO). WHO Mortality Database Version October 2017. Geneva, Switzerland: World Health Organization (WHO), 2017

Nicaragua Vital Registration - Deaths 2005 ICD10 as it appears in World Health Organization (WHO). WHO Mortality Database Version October 2017. Geneva, Switzerland: World Health Organization (WHO), 2017

Nicaragua Vital Registration - Deaths 2006 ICD10 as it appears in World Health Organization (WHO). WHO Mortality Database Version October 2017. Geneva, Switzerland: World Health Organization (WHO), 2017

Nicaragua Vital Registration - Deaths 2007 ICD10 as it appears in World Health Organization (WHO). WHO Mortality Database Version October 2017. Geneva, Switzerland: World Health Organization (WHO), 2017

Nicaragua Vital Registration - Deaths 2008 ICD10 as it appears in World Health Organization (WHO). WHO Mortality Database Version October 2017. Geneva, Switzerland: World Health Organization (WHO), 2017

Nicaragua Vital Registration - Deaths 2009 ICD10 as it appears in World Health Organization (WHO). WHO Mortality Database Version October 2017. Geneva, Switzerland: World Health Organization (WHO), 2017

Nicaragua Vital Registration - Deaths 2010 ICD10 as it appears in World Health Organization (WHO). WHO Mortality Database Version October 2017. Geneva, Switzerland: World Health Organization (WHO), 2017

Nicaragua Vital Registration - Deaths 2011 ICD10 as it appears in World Health Organization (WHO). WHO Mortality Database Version October 2017. Geneva, Switzerland: World Health Organization (WHO), 2017

Nicaragua Vital Registration - Deaths 2012 ICD10 as it appears in World Health Organization (WHO). WHO Mortality Database Version October 2017. Geneva, Switzerland: World Health Organization (WHO), 2017

Nicaragua Vital Registration - Deaths 2013 ICD10 as it appears in World Health Organization (WHO). WHO Mortality Database Version October 2017. Geneva, Switzerland: World Health Organization (WHO), 2017

Nicaragua Vital Registration - Deaths 2014 ICD10 as it appears in World Health Organization (WHO). WHO Mortality Database Version October 2017. Geneva, Switzerland: World Health Organization (WHO), 2017

Nicaragua Vital Registration - Deaths 2015 ICD10 as it appears in World Health Organization (WHO). WHO Mortality Database Version October 2017. Geneva, Switzerland: World Health Organization (WHO), 2017

Norwegian Institute of Public Health. Norway Cause of Death Registry 1980. Oslo, Norway: Norwegian Institute of Public Health

Norwegian Institute of Public Health. Norway Cause of Death Registry 1981. Oslo, Norway: Norwegian Institute of Public Health

Norwegian Institute of Public Health. Norway Cause of Death Registry 1982. Oslo, Norway: Norwegian Institute of Public Health

Norwegian Institute of Public Health. Norway Cause of Death Registry 1983. Oslo, Norway: Norwegian Institute of Public Health

Norwegian Institute of Public Health. Norway Cause of Death Registry 1984. Oslo, Norway: Norwegian Institute of Public Health

Norwegian Institute of Public Health. Norway Cause of Death Registry 1985. Oslo, Norway: Norwegian Institute of Public Health

Norwegian Institute of Public Health. Norway Cause of Death Registry 1986. Oslo, Norway: Norwegian Institute of Public Health

Norwegian Institute of Public Health. Norway Cause of Death Registry 1987. Oslo, Norway: Norwegian Institute of Public Health

Norwegian Institute of Public Health. Norway Cause of Death Registry 1988. Oslo, Norway: Norwegian Institute of Public Health

Norwegian Institute of Public Health. Norway Cause of Death Registry 1989. Oslo, Norway: Norwegian Institute of Public Health

Norwegian Institute of Public Health. Norway Cause of Death Registry 1990. Oslo, Norway: Norwegian Institute of Public Health

Norwegian Institute of Public Health. Norway Cause of Death Registry 1991. Oslo, Norway: Norwegian Institute of Public Health

Norwegian Institute of Public Health. Norway Cause of Death Registry 1992. Oslo, Norway: Norwegian Institute of Public Health

Norwegian Institute of Public Health. Norway Cause of Death Registry 1993. Oslo, Norway: Norwegian Institute of Public Health

Norwegian Institute of Public Health. Norway Cause of Death Registry 1994. Oslo, Norway: Norwegian Institute of Public Health

Norwegian Institute of Public Health. Norway Cause of Death Registry 1995. Oslo, Norway: Norwegian Institute of Public Health

Norwegian Institute of Public Health. Norway Cause of Death Registry 1996. Oslo, Norway: Norwegian Institute of Public Health

Norwegian Institute of Public Health. Norway Cause of Death Registry 1997. Oslo, Norway: Norwegian Institute of Public Health

Norwegian Institute of Public Health. Norway Cause of Death Registry 1998. Oslo, Norway: Norwegian Institute of Public Health

Norwegian Institute of Public Health. Norway Cause of Death Registry 1999. Oslo, Norway: Norwegian Institute of Public Health
Norwegian Institute of Public Health. Norway Cause of Death Registry 2000. Oslo, Norway: Norwegian Institute of Public Health
Norwegian Institute of Public Health. Norway Cause of Death Registry 2001. Oslo, Norway: Norwegian Institute of Public Health
Norwegian Institute of Public Health. Norway Cause of Death Registry 2002. Oslo, Norway: Norwegian Institute of Public Health
Norwegian Institute of Public Health. Norway Cause of Death Registry 2003. Oslo, Norway: Norwegian Institute of Public Health
Norwegian Institute of Public Health. Norway Cause of Death Registry 2004. Oslo, Norway: Norwegian Institute of Public Health
Norwegian Institute of Public Health. Norway Cause of Death Registry 2005. Oslo, Norway: Norwegian Institute of Public Health
Norwegian Institute of Public Health. Norway Cause of Death Registry 2006. Oslo, Norway: Norwegian Institute of Public Health
Norwegian Institute of Public Health. Norway Cause of Death Registry 2007. Oslo, Norway: Norwegian Institute of Public Health
Norwegian Institute of Public Health. Norway Cause of Death Registry 2008. Oslo, Norway: Norwegian Institute of Public Health
Norwegian Institute of Public Health. Norway Cause of Death Registry 2009. Oslo, Norway: Norwegian Institute of Public Health
Norwegian Institute of Public Health. Norway Cause of Death Registry 2010. Oslo, Norway: Norwegian Institute of Public Health
Norwegian Institute of Public Health. Norway Cause of Death Registry 2011. Oslo, Norway: Norwegian Institute of Public Health
Norwegian Institute of Public Health. Norway Cause of Death Registry 2012. Oslo, Norway: Norwegian Institute of Public Health
Norwegian Institute of Public Health. Norway Cause of Death Registry 2013. Oslo, Norway: Norwegian Institute of Public Health
Norwegian Institute of Public Health. Norway Cause of Death Registry 2014. Oslo, Norway: Norwegian Institute of Public Health
Norwegian Institute of Public Health. Norway Cause of Death Registry 2015. Oslo, Norway: Norwegian Institute of Public Health
Office for National Statistics (ONS) (United Kingdom). United Kingdom - England and Wales Mortality Statistics 2014
Office for National Statistics (ONS) (United Kingdom). United Kingdom - England and Wales Mortality Statistics 2015
Office for National Statistics (ONS) (United Kingdom). United Kingdom - England and Wales Mortality Statistics 2016
Office for National Statistics (United Kingdom). United Kingdom - England and Wales Mortality Data 1981-1994
Office for National Statistics (United Kingdom). United Kingdom - England and Wales Mortality Data 1995-2000
Office for National Statistics (United Kingdom). United Kingdom - England and Wales Mortality Data 2001-2012
Office for National Statistics (United Kingdom). United Kingdom - England and Wales Mortality Statistics 2013
Office for National Statistics (United Kingdom). United Kingdom - England Mortality Statistics 1981
Office for National Statistics (United Kingdom). United Kingdom - England Mortality Statistics 1982
Office for National Statistics (United Kingdom). United Kingdom - England Mortality Statistics 1983
Office for National Statistics (United Kingdom). United Kingdom - England Mortality Statistics 1984
Office for National Statistics (United Kingdom). United Kingdom - England Mortality Statistics 1985
Office for National Statistics (United Kingdom). United Kingdom - England Mortality Statistics 1986
Office for National Statistics (United Kingdom). United Kingdom - England Mortality Statistics 1987
Office for National Statistics (United Kingdom). United Kingdom - England Mortality Statistics 1988
Office for National Statistics (United Kingdom). United Kingdom - England Mortality Statistics 1989
Office for National Statistics (United Kingdom). United Kingdom - England Mortality Statistics 1990
Office for National Statistics (United Kingdom). United Kingdom - England Mortality Statistics 1991

Office for National Statistics (United Kingdom). United Kingdom - England Mortality Statistics 1992
Office for National Statistics (United Kingdom). United Kingdom - England Mortality Statistics 1993
Office for National Statistics (United Kingdom). United Kingdom - England Mortality Statistics 1994
Office for National Statistics (United Kingdom). United Kingdom - England Mortality Statistics 1995
Office for National Statistics (United Kingdom). United Kingdom - England Mortality Statistics 1996
Office for National Statistics (United Kingdom). United Kingdom - England Mortality Statistics 1997
Office for National Statistics (United Kingdom). United Kingdom - England Mortality Statistics 1998
Office for National Statistics (United Kingdom). United Kingdom - England Mortality Statistics 1999
Office for National Statistics (United Kingdom). United Kingdom - England Mortality Statistics 2000
Office for National Statistics (United Kingdom). United Kingdom - England Mortality Statistics 2001
Office for National Statistics (United Kingdom). United Kingdom - England Mortality Statistics 2002
Office for National Statistics (United Kingdom). United Kingdom - England Mortality Statistics 2003
Office for National Statistics (United Kingdom). United Kingdom - England Mortality Statistics 2004
Office for National Statistics (United Kingdom). United Kingdom - England Mortality Statistics 2005
Office for National Statistics (United Kingdom). United Kingdom - England Mortality Statistics 2006
Office for National Statistics (United Kingdom). United Kingdom - England Mortality Statistics 2007
Office for National Statistics (United Kingdom). United Kingdom - England Mortality Statistics 2008
Office for National Statistics (United Kingdom). United Kingdom - England Mortality Statistics 2009
Office for National Statistics (United Kingdom). United Kingdom - England Mortality Statistics 2010
Office for National Statistics (United Kingdom). United Kingdom - England Mortality Statistics 2011
Office for National Statistics (United Kingdom). United Kingdom - England Mortality Statistics 2012
Office of the Registrar General and Census Commissioner (India). India Medical Certification of Cause of Death Report 2005. New Delhi, India: Office of the Registrar General and Census Commissioner (India)
Office of the Registrar General and Census Commissioner (India). India Medical Certification of Cause of Death Report 2006. New Delhi, India: Office of the Registrar General and Census Commissioner (India)
Office of the Registrar General and Census Commissioner (India). India Medical Certification of Cause of Death Report 2008. New Delhi, India: Office of the Registrar General and Census Commissioner (India)
Office of the Registrar General and Census Commissioner (India). India Medical Certification of Cause of Death Report 2009. New Delhi, India: Office of the Registrar General and Census Commissioner (India), 2014
Office of the Registrar General and Census Commissioner (India). India Medical Certification of Cause of Death Report 2010. New Delhi, India: Office of the Registrar General and Census Commissioner (India), 2014
Office of the Registrar General and Census Commissioner (India). India Medical Certification of Cause of Death Report 2012. New Delhi, India: Office of the Registrar General and Census Commissioner (India), 2015
Office of the Registrar General and Census Commissioner (India). India Medical Certification of Cause of Death State-Level Tabulations 1999
Office of the Registrar General and Census Commissioner (India). India Medical Certification of Cause of Death State-Level Tabulations 2000
Office of the Registrar General and Census Commissioner (India). India Medical Certification of Cause of Death State-Level Tabulations 2001

Office of the Registrar General and Census Commissioner (India). India Medical Certification of Cause of Death State-Level Tabulations 2002
Office of the Registrar General and Census Commissioner (India). India Medical Certification of Cause of Death State-Level Tabulations 2003
Office of the Registrar General and Census Commissioner (India). India Medical Certification of Cause of Death State-Level Tabulations 2004
Palestine - West Bank and Gaza Strip Vital Registration - Deaths 2010 ICD10 as it appears in World Health Organization (WHO). WHO Mortality Database Version October 2017. Geneva, Switzerland: World Health Organization (WHO), 2017
Palestine - West Bank and Gaza Strip Vital Registration - Deaths 2011 ICD10 as it appears in World Health Organization (WHO). WHO Mortality Database Version October 2017. Geneva, Switzerland: World Health Organization (WHO), 2017
Palestine Vital Registration - Deaths 2008 ICD10 as it appears in World Health Organization (WHO). WHO Mortality Database Version October 2017. Geneva, Switzerland: World Health Organization (WHO), 2017
Palestine Vital Registration - Deaths 2009 ICD10 as it appears in World Health Organization (WHO). WHO Mortality Database Version October 2017. Geneva, Switzerland: World Health Organization (WHO), 2017
Palestinian Central Bureau of Statistics. Palestine - West Bank Vital Registration - Deaths 2004
Palestinian Central Bureau of Statistics. Palestine - West Bank Vital Registration - Deaths 2005
Palestinian Central Bureau of Statistics. Palestine - West Bank Vital Registration - Deaths 2007
Panama Vital Registration - Deaths 1980 ICD9 as it appears in World Health Organization (WHO). WHO Mortality Database Version September 2016. Geneva, Switzerland: World Health Organization (WHO), 2016
Panama Vital Registration - Deaths 1981 ICD9 as it appears in World Health Organization (WHO). WHO Mortality Database Version September 2016. Geneva, Switzerland: World Health Organization (WHO), 2016
Panama Vital Registration - Deaths 1982 ICD9 as it appears in World Health Organization (WHO). WHO Mortality Database Version September 2016. Geneva, Switzerland: World Health Organization (WHO), 2016
Panama Vital Registration - Deaths 1983 ICD9 as it appears in World Health Organization (WHO). WHO Mortality Database Version September 2016. Geneva, Switzerland: World Health Organization (WHO), 2016
Panama Vital Registration - Deaths 1984 ICD9 as it appears in World Health Organization (WHO). WHO Mortality Database Version September 2016. Geneva, Switzerland: World Health Organization (WHO), 2016
Panama Vital Registration - Deaths 1985 ICD9 as it appears in World Health Organization (WHO). WHO Mortality Database Version September 2016. Geneva, Switzerland: World Health Organization (WHO), 2016
Panama Vital Registration - Deaths 1986 ICD9 as it appears in World Health Organization (WHO). WHO Mortality Database Version September 2016. Geneva, Switzerland: World Health Organization (WHO), 2016
Panama Vital Registration - Deaths 1987 ICD9 as it appears in World Health Organization (WHO). WHO Mortality Database Version September 2016. Geneva, Switzerland: World Health Organization (WHO), 2016
Panama Vital Registration - Deaths 1988 ICD9 as it appears in World Health Organization (WHO). WHO Mortality Database Version September 2016. Geneva, Switzerland: World Health Organization (WHO), 2016
Panama Vital Registration - Deaths 1989 ICD9 as it appears in World Health Organization (WHO). WHO Mortality Database Version September 2016. Geneva, Switzerland: World Health Organization (WHO), 2016
Panama Vital Registration - Deaths 1996 ICD9 as it appears in World Health Organization (WHO). WHO Mortality Database Version September 2016. Geneva, Switzerland: World Health Organization (WHO), 2016
Panama Vital Registration - Deaths 1997 ICD9 as it appears in World Health Organization (WHO). WHO Mortality Database Version September 2016. Geneva, Switzerland: World Health Organization (WHO), 2016
Panama Vital Registration - Deaths 1998 ICD10 as it appears in World Health Organization (WHO). WHO Mortality Database Version October 2017. Geneva, Switzerland: World Health Organization (WHO), 2017

Panama Vital Registration - Deaths 1999 ICD10 as it appears in World Health Organization (WHO). WHO Mortality Database Version October 2017. Geneva, Switzerland: World Health Organization (WHO), 2017
Panama Vital Registration - Deaths 2000 ICD10 as it appears in World Health Organization (WHO). WHO Mortality Database Version October 2017. Geneva, Switzerland: World Health Organization (WHO), 2017
Panama Vital Registration - Deaths 2001 ICD10 as it appears in World Health Organization (WHO). WHO Mortality Database Version October 2017. Geneva, Switzerland: World Health Organization (WHO), 2017
Panama Vital Registration - Deaths 2002 ICD10 as it appears in World Health Organization (WHO). WHO Mortality Database Version October 2017. Geneva, Switzerland: World Health Organization (WHO), 2017
Panama Vital Registration - Deaths 2003 ICD10 as it appears in World Health Organization (WHO). WHO Mortality Database Version October 2017. Geneva, Switzerland: World Health Organization (WHO), 2017
Panama Vital Registration - Deaths 2004 ICD10 as it appears in World Health Organization (WHO). WHO Mortality Database Version October 2017. Geneva, Switzerland: World Health Organization (WHO), 2017
Panama Vital Registration - Deaths 2006 ICD10 as it appears in World Health Organization (WHO). WHO Mortality Database Version October 2017. Geneva, Switzerland: World Health Organization (WHO), 2017
Panama Vital Registration - Deaths 2005 ICD10 as it appears in World Health Organization (WHO). WHO Mortality Database Version February 2014. Geneva, Switzerland: World Health Organization (WHO), 2014
Panama Vital Registration - Deaths 2007 ICD10 as it appears in World Health Organization (WHO). WHO Mortality Database Version October 2017. Geneva, Switzerland: World Health Organization (WHO), 2017
Panama Vital Registration - Deaths 2008 ICD10 as it appears in World Health Organization (WHO). WHO Mortality Database Version October 2017. Geneva, Switzerland: World Health Organization (WHO), 2017
Panama Vital Registration - Deaths 2009 ICD10 as it appears in World Health Organization (WHO). WHO Mortality Database Version October 2017. Geneva, Switzerland: World Health Organization (WHO), 2017
Panama Vital Registration - Deaths 2010 ICD10 as it appears in World Health Organization (WHO). WHO Mortality Database Version October 2017. Geneva, Switzerland: World Health Organization (WHO), 2017
Panama Vital Registration - Deaths 2011 ICD10 as it appears in World Health Organization (WHO). WHO Mortality Database Version October 2017. Geneva, Switzerland: World Health Organization (WHO), 2017
Panama Vital Registration - Deaths 2012 ICD10 as it appears in World Health Organization (WHO). WHO Mortality Database Version October 2017. Geneva, Switzerland: World Health Organization (WHO), 2017
Panama Vital Registration - Deaths 2013 ICD10 as it appears in World Health Organization (WHO). WHO Mortality Database Version October 2017. Geneva, Switzerland: World Health Organization (WHO), 2017
Panama Vital Registration - Deaths 2014 ICD10 as it appears in World Health Organization (WHO). WHO Mortality Database Version October 2017. Geneva, Switzerland: World Health Organization (WHO), 2017
Panama Vital Registration - Deaths 2015 ICD10 as it appears in World Health Organization (WHO). WHO Mortality Database Version October 2017. Geneva, Switzerland: World Health Organization (WHO), 2017
Paraguay Vital Registration - Deaths 1980 ICD9 as it appears in World Health Organization (WHO). WHO Mortality Database Version September 2016. Geneva, Switzerland: World Health Organization (WHO), 2016
Paraguay Vital Registration - Deaths 1981 ICD9 as it appears in World Health Organization (WHO). WHO Mortality Database Version September 2016. Geneva, Switzerland: World Health Organization (WHO), 2016
Paraguay Vital Registration - Deaths 1982 ICD9 as it appears in World Health Organization (WHO). WHO Mortality Database Version September 2016. Geneva, Switzerland: World Health Organization (WHO), 2016

Paraguay Vital Registration - Deaths 1983 ICD9 as it appears in World Health Organization (WHO). WHO Mortality Database Version September 2016. Geneva, Switzerland: World Health Organization (WHO), 2016
Paraguay Vital Registration - Deaths 1984 ICD9 as it appears in World Health Organization (WHO). WHO Mortality Database Version September 2016. Geneva, Switzerland: World Health Organization (WHO), 2016
Paraguay Vital Registration - Deaths 1985 ICD9 as it appears in World Health Organization (WHO). WHO Mortality Database Version September 2016. Geneva, Switzerland: World Health Organization (WHO), 2016
Paraguay Vital Registration - Deaths 1986 ICD9 as it appears in World Health Organization (WHO). WHO Mortality Database Version September 2016. Geneva, Switzerland: World Health Organization (WHO), 2016
Paraguay Vital Registration - Deaths 1987 ICD9 as it appears in World Health Organization (WHO). WHO Mortality Database Version September 2016. Geneva, Switzerland: World Health Organization (WHO), 2016
Paraguay Vital Registration - Deaths 1988 ICD9 as it appears in World Health Organization (WHO). WHO Mortality Database Version September 2016. Geneva, Switzerland: World Health Organization (WHO), 2016
Paraguay Vital Registration - Deaths 1989 ICD9 as it appears in World Health Organization (WHO). WHO Mortality Database Version September 2016. Geneva, Switzerland: World Health Organization (WHO), 2016
Paraguay Vital Registration - Deaths 1990 ICD9 as it appears in World Health Organization (WHO). WHO Mortality Database Version September 2016. Geneva, Switzerland: World Health Organization (WHO), 2016
Paraguay Vital Registration - Deaths 1991 ICD9 as it appears in World Health Organization (WHO). WHO Mortality Database Version September 2016. Geneva, Switzerland: World Health Organization (WHO), 2016
Paraguay Vital Registration - Deaths 1994 ICD9 as it appears in World Health Organization (WHO). WHO Mortality Database Version September 2016. Geneva, Switzerland: World Health Organization (WHO), 2016
Paraguay Vital Registration - Deaths 1995 ICD9 as it appears in World Health Organization (WHO). WHO Mortality Database Version September 2016. Geneva, Switzerland: World Health Organization (WHO), 2016
Paraguay Vital Registration - Deaths 1996 ICD10 as it appears in World Health Organization (WHO). WHO Mortality Database Version October 2017. Geneva, Switzerland: World Health Organization (WHO), 2017
Paraguay Vital Registration - Deaths 1997 ICD10 as it appears in World Health Organization (WHO). WHO Mortality Database Version October 2017. Geneva, Switzerland: World Health Organization (WHO), 2017
Paraguay Vital Registration - Deaths 1998 ICD10 as it appears in World Health Organization (WHO). WHO Mortality Database Version October 2017. Geneva, Switzerland: World Health Organization (WHO), 2017
Paraguay Vital Registration - Deaths 1999 ICD10 as it appears in World Health Organization (WHO). WHO Mortality Database Version October 2017. Geneva, Switzerland: World Health Organization (WHO), 2017
Paraguay Vital Registration - Deaths 2000 ICD10 as it appears in World Health Organization (WHO). WHO Mortality Database Version October 2017. Geneva, Switzerland: World Health Organization (WHO), 2017
Paraguay Vital Registration - Deaths 2001 ICD10 as it appears in World Health Organization (WHO). WHO Mortality Database Version October 2017. Geneva, Switzerland: World Health Organization (WHO), 2017
Paraguay Vital Registration - Deaths 2002 ICD10 as it appears in World Health Organization (WHO). WHO Mortality Database Version October 2017. Geneva, Switzerland: World Health Organization (WHO), 2017
Paraguay Vital Registration - Deaths 2003 ICD10 as it appears in World Health Organization (WHO). WHO Mortality Database Version October 2017. Geneva, Switzerland: World Health Organization (WHO), 2017
Paraguay Vital Registration - Deaths 2004 ICD10 as it appears in World Health Organization (WHO). WHO Mortality Database Version October 2017. Geneva, Switzerland: World Health Organization (WHO), 2017

Paraguay Vital Registration - Deaths 2005 ICD10 as it appears in World Health Organization (WHO). WHO Mortality Database Version October 2017. Geneva, Switzerland: World Health Organization (WHO), 2017
Paraguay Vital Registration - Deaths 2006 ICD10 as it appears in World Health Organization (WHO). WHO Mortality Database Version October 2017. Geneva, Switzerland: World Health Organization (WHO), 2017
Paraguay Vital Registration - Deaths 2007 ICD10 as it appears in World Health Organization (WHO). WHO Mortality Database Version October 2017. Geneva, Switzerland: World Health Organization (WHO), 2017
Paraguay Vital Registration - Deaths 2008 ICD10 as it appears in World Health Organization (WHO). WHO Mortality Database Version October 2017. Geneva, Switzerland: World Health Organization (WHO), 2017
Paraguay Vital Registration - Deaths 2009 ICD10 as it appears in World Health Organization (WHO). WHO Mortality Database Version October 2017. Geneva, Switzerland: World Health Organization (WHO), 2017
Paraguay Vital Registration - Deaths 2010 ICD10 as it appears in World Health Organization (WHO). WHO Mortality Database Version October 2017. Geneva, Switzerland: World Health Organization (WHO), 2017
Paraguay Vital Registration - Deaths 2011 ICD10 as it appears in World Health Organization (WHO). WHO Mortality Database Version October 2017. Geneva, Switzerland: World Health Organization (WHO), 2017
Paraguay Vital Registration - Deaths 2012 ICD10 as it appears in World Health Organization (WHO). WHO Mortality Database Version October 2017. Geneva, Switzerland: World Health Organization (WHO), 2017
Paraguay Vital Registration - Deaths 2013 ICD10 as it appears in World Health Organization (WHO). WHO Mortality Database Version October 2017. Geneva, Switzerland: World Health Organization (WHO), 2017
Paraguay Vital Registration - Deaths 2014 ICD10 as it appears in World Health Organization (WHO). WHO Mortality Database Version October 2017. Geneva, Switzerland: World Health Organization (WHO), 2017
Peru Vital Registration - Deaths 1980 ICD9 as it appears in World Health Organization (WHO). WHO Mortality Database Version September 2016. Geneva, Switzerland: World Health Organization (WHO), 2016
Peru Vital Registration - Deaths 1981 ICD9 as it appears in World Health Organization (WHO). WHO Mortality Database Version September 2016. Geneva, Switzerland: World Health Organization (WHO), 2016
Peru Vital Registration - Deaths 1982 ICD9 as it appears in World Health Organization (WHO). WHO Mortality Database Version September 2016. Geneva, Switzerland: World Health Organization (WHO), 2016
Peru Vital Registration - Deaths 1983 ICD9 as it appears in World Health Organization (WHO). WHO Mortality Database Version September 2016. Geneva, Switzerland: World Health Organization (WHO), 2016
Peru Vital Registration - Deaths 1986 ICD9 as it appears in World Health Organization (WHO). WHO Mortality Database Version September 2016. Geneva, Switzerland: World Health Organization (WHO), 2016
Peru Vital Registration - Deaths 1988 ICD9 as it appears in World Health Organization (WHO). WHO Mortality Database Version September 2016. Geneva, Switzerland: World Health Organization (WHO), 2016
Peru Vital Registration - Deaths 1989 ICD9 as it appears in World Health Organization (WHO). WHO Mortality Database Version September 2016. Geneva, Switzerland: World Health Organization (WHO), 2016
Peru Vital Registration - Deaths 1990 ICD9 as it appears in World Health Organization (WHO). WHO Mortality Database Version September 2016. Geneva, Switzerland: World Health Organization (WHO), 2016
Peru Vital Registration - Deaths 1992 ICD9 as it appears in World Health Organization (WHO). WHO Mortality Database Version September 2016. Geneva, Switzerland: World Health Organization (WHO), 2016
Peru Vital Registration - Deaths 1994 ICD9 as it appears in World Health Organization (WHO). WHO Mortality Database Version September 2016. Geneva, Switzerland: World Health Organization (WHO), 2016
Peru Vital Registration - Deaths 1995 ICD9 as it appears in World Health Organization (WHO). WHO Mortality Database Version September 2016. Geneva, Switzerland:

World Health Organization (WHO), 2016
Peru Vital Registration - Deaths 1996 ICD9 as it appears in World Health Organization (WHO). WHO Mortality Database Version September 2016. Geneva, Switzerland: World Health Organization (WHO), 2016
Peru Vital Registration - Deaths 1997 ICD9 as it appears in World Health Organization (WHO). WHO Mortality Database Version September 2016. Geneva, Switzerland: World Health Organization (WHO), 2016
Peru Vital Registration - Deaths 1998 ICD9 as it appears in World Health Organization (WHO). WHO Mortality Database Version September 2016. Geneva, Switzerland: World Health Organization (WHO), 2016
Peru Vital Registration - Deaths 1999 ICD10 as it appears in World Health Organization (WHO). WHO Mortality Database Version October 2017. Geneva, Switzerland: World Health Organization (WHO), 2017
Peru Vital Registration - Deaths 2000 ICD10 as it appears in World Health Organization (WHO). WHO Mortality Database Version October 2017. Geneva, Switzerland: World Health Organization (WHO), 2017
Peru Vital Registration - Deaths 2007 ICD10 as it appears in World Health Organization (WHO). WHO Mortality Database Version October 2017. Geneva, Switzerland: World Health Organization (WHO), 2017
Peru Vital Registration - Deaths 2008 ICD10 as it appears in World Health Organization (WHO). WHO Mortality Database Version October 2017. Geneva, Switzerland: World Health Organization (WHO), 2017
Peru Vital Registration - Deaths 2009 ICD10 as it appears in World Health Organization (WHO). WHO Mortality Database Version October 2017. Geneva, Switzerland: World Health Organization (WHO), 2017
Peru Vital Registration - Deaths 2010 ICD10 as it appears in World Health Organization (WHO). WHO Mortality Database Version October 2017. Geneva, Switzerland: World Health Organization (WHO), 2017
Peru Vital Registration - Deaths 2011 ICD10 as it appears in World Health Organization (WHO). WHO Mortality Database Version October 2017. Geneva, Switzerland: World Health Organization (WHO), 2017
Peru Vital Registration - Deaths 2012 ICD10 as it appears in World Health Organization (WHO). WHO Mortality Database Version October 2017. Geneva, Switzerland: World Health Organization (WHO), 2017
Peru Vital Registration - Deaths 2013 ICD10 as it appears in World Health Organization (WHO). WHO Mortality Database Version October 2017. Geneva, Switzerland: World Health Organization (WHO), 2017
Peru Vital Registration - Deaths 2014 ICD10 as it appears in World Health Organization (WHO). WHO Mortality Database Version October 2017. Geneva, Switzerland: World Health Organization (WHO), 2017
Peru Vital Registration - Deaths 2015 ICD10 as it appears in World Health Organization (WHO). WHO Mortality Database Version October 2017. Geneva, Switzerland: World Health Organization (WHO), 2017
Philippines Statistics Authority. Philippines Vital Registration - Deaths 2013
Philippines Statistics Authority. Philippines Vital Registration - Deaths 2014
Philippines Statistics Authority. Philippines Vital Registration - Deaths 2015
Philippines Vital Registration - Deaths 1981 ICD8 as it appears in World Health Organization (WHO). WHO Mortality Database Version July 2012. Geneva, Switzerland: World Health Organization (WHO), 2012
Philippines Vital Registration - Deaths 1992 ICD9 as it appears in World Health Organization (WHO). WHO Mortality Database Version September 2016. Geneva, Switzerland: World Health Organization (WHO), 2016
Philippines Vital Registration - Deaths 1993 ICD9 as it appears in World Health Organization (WHO). WHO Mortality Database Version September 2016. Geneva, Switzerland: World Health Organization (WHO), 2016
Philippines Vital Registration - Deaths 1994 ICD9 as it appears in World Health Organization (WHO). WHO Mortality Database Version September 2016. Geneva, Switzerland: World Health Organization (WHO), 2016

Philippines Vital Registration - Deaths 1995 ICD9 as it appears in World Health Organization (WHO). WHO Mortality Database Version September 2016. Geneva, Switzerland: World Health Organization (WHO), 2016
Philippines Vital Registration - Deaths 1996 ICD9 as it appears in World Health Organization (WHO). WHO Mortality Database Version September 2016. Geneva, Switzerland: World Health Organization (WHO), 2016
Philippines Vital Registration - Deaths 1999 ICD10 as it appears in World Health Organization (WHO). WHO Mortality Database Version October 2017. Geneva, Switzerland: World Health Organization (WHO), 2017
Philippines Vital Registration - Deaths 2000 ICD10 as it appears in World Health Organization (WHO). WHO Mortality Database Version October 2017. Geneva, Switzerland: World Health Organization (WHO), 2017
Philippines Vital Registration - Deaths 2001 ICD10 as it appears in World Health Organization (WHO). WHO Mortality Database Version October 2017. Geneva, Switzerland: World Health Organization (WHO), 2017
Philippines Vital Registration - Deaths 2002 ICD10 as it appears in World Health Organization (WHO). WHO Mortality Database Version October 2017. Geneva, Switzerland: World Health Organization (WHO), 2017
Philippines Vital Registration - Deaths 2003 ICD10 as it appears in World Health Organization (WHO). WHO Mortality Database Version October 2017. Geneva, Switzerland: World Health Organization (WHO), 2017
Philippines Vital Registration - Deaths 1997 ICD9 as it appears in World Health Organization (WHO). WHO Mortality Database Version November 2015. Geneva, Switzerland: World Health Organization (WHO), 2015
Philippines Vital Registration - Deaths 1998 ICD9 as it appears in World Health Organization (WHO). WHO Mortality Database Version November 2015. Geneva, Switzerland: World Health Organization (WHO), 2015
Philippines Vital Registration - Deaths 2006 ICD10 as it appears in World Health Organization (WHO). WHO Mortality Database Version October 2017. Geneva, Switzerland: World Health Organization (WHO), 2017
Philippines Vital Registration - Deaths 2007 ICD10 as it appears in World Health Organization (WHO). WHO Mortality Database Version October 2017. Geneva, Switzerland: World Health Organization (WHO), 2017
Philippines Vital Registration - Deaths 2008 ICD10 as it appears in World Health Organization (WHO). WHO Mortality Database Version October 2017. Geneva, Switzerland: World Health Organization (WHO), 2017
Philippines Vital Registration - Deaths 2009 ICD10 as it appears in World Health Organization (WHO). WHO Mortality Database Version October 2017. Geneva, Switzerland: World Health Organization (WHO), 2017
Philippines Vital Registration - Deaths 2010 ICD10 as it appears in World Health Organization (WHO). WHO Mortality Database Version October 2017. Geneva, Switzerland: World Health Organization (WHO), 2017
Philippines Vital Registration - Deaths 2011 ICD10 as it appears in World Health Organization (WHO). WHO Mortality Database Version October 2017. Geneva, Switzerland: World Health Organization (WHO), 2017
Poland Vital Registration - Deaths 1980 ICD9 as it appears in World Health Organization (WHO). WHO Mortality Database Version September 2016. Geneva, Switzerland: World Health Organization (WHO), 2016
Poland Vital Registration - Deaths 1981 ICD9 as it appears in World Health Organization (WHO). WHO Mortality Database Version September 2016. Geneva, Switzerland: World Health Organization (WHO), 2016
Poland Vital Registration - Deaths 1982 ICD9 as it appears in World Health Organization (WHO). WHO Mortality Database Version September 2016. Geneva, Switzerland: World Health Organization (WHO), 2016
Poland Vital Registration - Deaths 1983 ICD9 as it appears in World Health Organization (WHO). WHO Mortality Database Version September 2016. Geneva, Switzerland: World Health Organization (WHO), 2016
Poland Vital Registration - Deaths 1984 ICD9 as it appears in World Health Organization (WHO). WHO Mortality Database Version September 2016. Geneva, Switzerland: World Health Organization (WHO), 2016

Poland Vital Registration - Deaths 1985 ICD9 as it appears in World Health Organization (WHO). WHO Mortality Database Version September 2016. Geneva, Switzerland: World Health Organization (WHO), 2016
Poland Vital Registration - Deaths 1986 ICD9 as it appears in World Health Organization (WHO). WHO Mortality Database Version September 2016. Geneva, Switzerland: World Health Organization (WHO), 2016
Poland Vital Registration - Deaths 1987 ICD9 as it appears in World Health Organization (WHO). WHO Mortality Database Version September 2016. Geneva, Switzerland: World Health Organization (WHO), 2016
Poland Vital Registration - Deaths 1988 ICD9 as it appears in World Health Organization (WHO). WHO Mortality Database Version September 2016. Geneva, Switzerland: World Health Organization (WHO), 2016
Poland Vital Registration - Deaths 1989 ICD9 as it appears in World Health Organization (WHO). WHO Mortality Database Version September 2016. Geneva, Switzerland: World Health Organization (WHO), 2016
Poland Vital Registration - Deaths 1990 ICD9 as it appears in World Health Organization (WHO). WHO Mortality Database Version September 2016. Geneva, Switzerland: World Health Organization (WHO), 2016
Poland Vital Registration - Deaths 1991 ICD9 as it appears in World Health Organization (WHO). WHO Mortality Database Version September 2016. Geneva, Switzerland: World Health Organization (WHO), 2016
Poland Vital Registration - Deaths 1992 ICD9 as it appears in World Health Organization (WHO). WHO Mortality Database Version September 2016. Geneva, Switzerland: World Health Organization (WHO), 2016
Poland Vital Registration - Deaths 1993 ICD9 as it appears in World Health Organization (WHO). WHO Mortality Database Version September 2016. Geneva, Switzerland: World Health Organization (WHO), 2016
Poland Vital Registration - Deaths 1994 ICD9 as it appears in World Health Organization (WHO). WHO Mortality Database Version September 2016. Geneva, Switzerland: World Health Organization (WHO), 2016
Poland Vital Registration - Deaths 1995 ICD9 as it appears in World Health Organization (WHO). WHO Mortality Database Version September 2016. Geneva, Switzerland: World Health Organization (WHO), 2016
Poland Vital Registration - Deaths 1996 ICD9 as it appears in World Health Organization (WHO). WHO Mortality Database Version September 2016. Geneva, Switzerland: World Health Organization (WHO), 2016
Poland Vital Registration - Deaths 1999 ICD10 as it appears in World Health Organization (WHO). WHO Mortality Database Version October 2017. Geneva, Switzerland: World Health Organization (WHO), 2017
Poland Vital Registration - Deaths 2000 ICD10 as it appears in World Health Organization (WHO). WHO Mortality Database Version October 2017. Geneva, Switzerland: World Health Organization (WHO), 2017
Poland Vital Registration - Deaths 2001 ICD10 as it appears in World Health Organization (WHO). WHO Mortality Database Version October 2017. Geneva, Switzerland: World Health Organization (WHO), 2017
Poland Vital Registration - Deaths 2002 ICD10 as it appears in World Health Organization (WHO). WHO Mortality Database Version October 2017. Geneva, Switzerland: World Health Organization (WHO), 2017
Poland Vital Registration - Deaths 2003 ICD10 as it appears in World Health Organization (WHO). WHO Mortality Database Version October 2017. Geneva, Switzerland: World Health Organization (WHO), 2017
Poland Vital Registration - Deaths 2004 ICD10 as it appears in World Health Organization (WHO). WHO Mortality Database Version October 2017. Geneva, Switzerland: World Health Organization (WHO), 2017
Poland Vital Registration - Deaths 2005 ICD10 as it appears in World Health Organization (WHO). WHO Mortality Database Version October 2017. Geneva, Switzerland: World Health Organization (WHO), 2017
Poland Vital Registration - Deaths 2006 ICD10 as it appears in World Health Organization (WHO). WHO Mortality Database Version October 2017. Geneva, Switzerland: World Health Organization (WHO), 2017

Poland Vital Registration - Deaths 2007 ICD10 as it appears in World Health Organization (WHO). WHO Mortality Database Version October 2017. Geneva, Switzerland: World Health Organization (WHO), 2017

Poland Vital Registration - Deaths 2008 ICD10 as it appears in World Health Organization (WHO). WHO Mortality Database Version October 2017. Geneva, Switzerland: World Health Organization (WHO), 2017

Poland Vital Registration - Deaths 2009 ICD10 as it appears in World Health Organization (WHO). WHO Mortality Database Version October 2017. Geneva, Switzerland: World Health Organization (WHO), 2017

Poland Vital Registration - Deaths 2010 ICD10 as it appears in World Health Organization (WHO). WHO Mortality Database Version October 2017. Geneva, Switzerland: World Health Organization (WHO), 2017

Poland Vital Registration - Deaths 2011 ICD10 as it appears in World Health Organization (WHO). WHO Mortality Database Version October 2017. Geneva, Switzerland: World Health Organization (WHO), 2017

Poland Vital Registration - Deaths 2012 ICD10 as it appears in World Health Organization (WHO). WHO Mortality Database Version October 2017. Geneva, Switzerland: World Health Organization (WHO), 2017

Poland Vital Registration - Deaths 2013 ICD10 as it appears in World Health Organization (WHO). WHO Mortality Database Version October 2017. Geneva, Switzerland: World Health Organization (WHO), 2017

Poland Vital Registration - Deaths 2014 ICD10 as it appears in World Health Organization (WHO). WHO Mortality Database Version October 2017. Geneva, Switzerland: World Health Organization (WHO), 2017

Poland Vital Registration - Deaths 2015 ICD10 as it appears in World Health Organization (WHO). WHO Mortality Database Version October 2017. Geneva, Switzerland: World Health Organization (WHO), 2017

Porapakkham Y, Rao C, Pattaraarchachai J, Polprasert W, Vos T, Adair T, Lopez AD. Estimated causes of death in Thailand, 2005: implications for health policy. Popul Health Metr. 2010; 8:14

Portugal Vital Registration - Deaths 1984 ICD9 as it appears in World Health Organization (WHO). WHO Mortality Database Version November 2015. Geneva, Switzerland: World Health Organization (WHO), 2015

Portugal Vital Registration - Deaths 1985 ICD9 as it appears in World Health Organization (WHO). WHO Mortality Database Version November 2015. Geneva, Switzerland: World Health Organization (WHO), 2015

Portugal Vital Registration - Deaths 1986 ICD9 as it appears in World Health Organization (WHO). WHO Mortality Database Version November 2015. Geneva, Switzerland: World Health Organization (WHO), 2015

Portugal Vital Registration - Deaths 1987 ICD9 as it appears in World Health Organization (WHO). WHO Mortality Database Version November 2015. Geneva, Switzerland: World Health Organization (WHO), 2015

Portugal Vital Registration - Deaths 1988 ICD9 as it appears in World Health Organization (WHO). WHO Mortality Database Version November 2015. Geneva, Switzerland: World Health Organization (WHO), 2015

Portugal Vital Registration - Deaths 1989 ICD9 as it appears in World Health Organization (WHO). WHO Mortality Database Version November 2015. Geneva, Switzerland: World Health Organization (WHO), 2015

Portugal Vital Registration - Deaths 1990 ICD9 as it appears in World Health Organization (WHO). WHO Mortality Database Version November 2015. Geneva, Switzerland: World Health Organization (WHO), 2015

Portugal Vital Registration - Deaths 1991 ICD9 as it appears in World Health Organization (WHO). WHO Mortality Database Version November 2015. Geneva, Switzerland: World Health Organization (WHO), 2015

Portugal Vital Registration - Deaths 1992 ICD9 as it appears in World Health Organization (WHO). WHO Mortality Database Version November 2015. Geneva, Switzerland: World Health Organization (WHO), 2015

Portugal Vital Registration - Deaths 1993 ICD9 as it appears in World Health Organization (WHO). WHO Mortality Database Version November 2015. Geneva, Switzerland: World Health Organization (WHO), 2015

Portugal Vital Registration - Deaths 1994 ICD9 as it appears in World Health Organization (WHO). WHO Mortality Database Version November 2015. Geneva, Switzerland: World Health Organization (WHO), 2015
Portugal Vital Registration - Deaths 1995 ICD9 as it appears in World Health Organization (WHO). WHO Mortality Database Version November 2015. Geneva, Switzerland: World Health Organization (WHO), 2015
Portugal Vital Registration - Deaths 1996 ICD9 as it appears in World Health Organization (WHO). WHO Mortality Database Version November 2015. Geneva, Switzerland: World Health Organization (WHO), 2015
Portugal Vital Registration - Deaths 1997 ICD9 as it appears in World Health Organization (WHO). WHO Mortality Database Version November 2015. Geneva, Switzerland: World Health Organization (WHO), 2015
Portugal Vital Registration - Deaths 1998 ICD9 as it appears in World Health Organization (WHO). WHO Mortality Database Version November 2015. Geneva, Switzerland: World Health Organization (WHO), 2015
Portugal Vital Registration - Deaths 1999 ICD9 as it appears in World Health Organization (WHO). WHO Mortality Database Version November 2015. Geneva, Switzerland: World Health Organization (WHO), 2015
Portugal Vital Registration - Deaths 2000 ICD9 as it appears in World Health Organization (WHO). WHO Mortality Database Version November 2015. Geneva, Switzerland: World Health Organization (WHO), 2015
Portugal Vital Registration - Deaths 2001 ICD9 as it appears in World Health Organization (WHO). WHO Mortality Database Version November 2015. Geneva, Switzerland: World Health Organization (WHO), 2015
Portugal Vital Registration - Deaths 2002 ICD10 as it appears in World Health Organization (WHO). WHO Mortality Database Version October 2017. Geneva, Switzerland: World Health Organization (WHO), 2017
Portugal Vital Registration - Deaths 2003 ICD10 as it appears in World Health Organization (WHO). WHO Mortality Database Version October 2017. Geneva, Switzerland: World Health Organization (WHO), 2017
Portugal Vital Registration - Deaths 2007 ICD10 as it appears in World Health Organization (WHO). WHO Mortality Database Version October 2017. Geneva, Switzerland: World Health Organization (WHO), 2017
Portugal Vital Registration - Deaths 2008 ICD10 as it appears in World Health Organization (WHO). WHO Mortality Database Version October 2017. Geneva, Switzerland: World Health Organization (WHO), 2017
Portugal Vital Registration - Deaths 2009 ICD10 as it appears in World Health Organization (WHO). WHO Mortality Database Version October 2017. Geneva, Switzerland: World Health Organization (WHO), 2017
Portugal Vital Registration - Deaths 2010 ICD10 as it appears in World Health Organization (WHO). WHO Mortality Database Version October 2017. Geneva, Switzerland: World Health Organization (WHO), 2017
Portugal Vital Registration - Deaths 2011 ICD10 as it appears in World Health Organization (WHO). WHO Mortality Database Version October 2017. Geneva, Switzerland: World Health Organization (WHO), 2017
Portugal Vital Registration - Deaths 2012 ICD10 as it appears in World Health Organization (WHO). WHO Mortality Database Version October 2017. Geneva, Switzerland: World Health Organization (WHO), 2017
Portugal Vital Registration - Deaths 2013 ICD10 as it appears in World Health Organization (WHO). WHO Mortality Database Version October 2017. Geneva, Switzerland: World Health Organization (WHO), 2017
Portugal Vital Registration - Deaths 2014 ICD10 as it appears in World Health Organization (WHO). WHO Mortality Database Version October 2017. Geneva, Switzerland: World Health Organization (WHO), 2017
Puerto Rico Vital Registration - Deaths 1980 ICD9 as it appears in World Health Organization (WHO). WHO Mortality Database Version September 2016. Geneva, Switzerland: World Health Organization (WHO), 2016
Puerto Rico Vital Registration - Deaths 1981 ICD9 as it appears in World Health Organization (WHO). WHO Mortality Database Version September 2016. Geneva, Switzerland: World Health Organization (WHO), 2016

Puerto Rico Vital Registration - Deaths 1982 ICD9 as it appears in World Health Organization (WHO). WHO Mortality Database Version September 2016. Geneva, Switzerland: World Health Organization (WHO), 2016
Puerto Rico Vital Registration - Deaths 1983 ICD9 as it appears in World Health Organization (WHO). WHO Mortality Database Version September 2016. Geneva, Switzerland: World Health Organization (WHO), 2016
Puerto Rico Vital Registration - Deaths 1984 ICD9 as it appears in World Health Organization (WHO). WHO Mortality Database Version September 2016. Geneva, Switzerland: World Health Organization (WHO), 2016
Puerto Rico Vital Registration - Deaths 1985 ICD9 as it appears in World Health Organization (WHO). WHO Mortality Database Version September 2016. Geneva, Switzerland: World Health Organization (WHO), 2016
Puerto Rico Vital Registration - Deaths 1986 ICD9 as it appears in World Health Organization (WHO). WHO Mortality Database Version September 2016. Geneva, Switzerland: World Health Organization (WHO), 2016
Puerto Rico Vital Registration - Deaths 1987 ICD9 as it appears in World Health Organization (WHO). WHO Mortality Database Version September 2016. Geneva, Switzerland: World Health Organization (WHO), 2016
Puerto Rico Vital Registration - Deaths 1988 ICD9 as it appears in World Health Organization (WHO). WHO Mortality Database Version September 2016. Geneva, Switzerland: World Health Organization (WHO), 2016
Puerto Rico Vital Registration - Deaths 1989 ICD9 as it appears in World Health Organization (WHO). WHO Mortality Database Version September 2016. Geneva, Switzerland: World Health Organization (WHO), 2016
Puerto Rico Vital Registration - Deaths 1990 ICD9 as it appears in World Health Organization (WHO). WHO Mortality Database Version September 2016. Geneva, Switzerland: World Health Organization (WHO), 2016
Puerto Rico Vital Registration - Deaths 1991 ICD9 as it appears in World Health Organization (WHO). WHO Mortality Database Version September 2016. Geneva, Switzerland: World Health Organization (WHO), 2016
Puerto Rico Vital Registration - Deaths 1992 ICD9 as it appears in World Health Organization (WHO). WHO Mortality Database Version September 2016. Geneva, Switzerland: World Health Organization (WHO), 2016
Puerto Rico Vital Registration - Deaths 1993 ICD9 as it appears in World Health Organization (WHO). WHO Mortality Database Version September 2016. Geneva, Switzerland: World Health Organization (WHO), 2016
Qatar Vital Registration - Deaths 1995 ICD10 as it appears in World Health Organization (WHO). WHO Mortality Database Version October 2017. Geneva, Switzerland: World Health Organization (WHO), 2017
Qatar Vital Registration - Deaths 2001 ICD10 as it appears in World Health Organization (WHO). WHO Mortality Database Version October 2017. Geneva, Switzerland: World Health Organization (WHO), 2017
Qatar Vital Registration - Deaths 2004 ICD10 as it appears in World Health Organization (WHO). WHO Mortality Database Version October 2017. Geneva, Switzerland: World Health Organization (WHO), 2017
Qatar Vital Registration - Deaths 2005 ICD10 as it appears in World Health Organization (WHO). WHO Mortality Database Version October 2017. Geneva, Switzerland: World Health Organization (WHO), 2017
Qatar Vital Registration - Deaths 2006 ICD10 as it appears in World Health Organization (WHO). WHO Mortality Database Version October 2017. Geneva, Switzerland: World Health Organization (WHO), 2017
Qatar Vital Registration - Deaths 2007 ICD10 as it appears in World Health Organization (WHO). WHO Mortality Database Version October 2017. Geneva, Switzerland: World Health Organization (WHO), 2017
Qatar Vital Registration - Deaths 2008 ICD10 as it appears in World Health Organization (WHO). WHO Mortality Database Version October 2017. Geneva, Switzerland: World Health Organization (WHO), 2017
Qatar Vital Registration - Deaths 2009 ICD10 as it appears in World Health Organization (WHO). WHO Mortality Database Version October 2017. Geneva, Switzerland: World Health Organization (WHO), 2017
Qatar Vital Registration - Deaths 2010 ICD10 as it appears in World Health Organization (WHO). WHO Mortality Database Version October 2017. Geneva, Switzerland:

World Health Organization (WHO), 2017

Qatar Vital Registration - Deaths 2011 ICD10 as it appears in World Health Organization (WHO). WHO Mortality Database Version October 2017. Geneva, Switzerland: World Health Organization (WHO), 2017

Qatar Vital Registration - Deaths 2012 ICD10 as it appears in World Health Organization (WHO). WHO Mortality Database Version October 2017. Geneva, Switzerland: World Health Organization (WHO), 2017

Qatar Vital Registration - Deaths 2013 ICD10 as it appears in World Health Organization (WHO). WHO Mortality Database Version March 2017. Geneva, Switzerland: World Health Organization (WHO), 2017

Qatar Vital Registration - Deaths 2014 ICD10 as it appears in World Health Organization (WHO). WHO Mortality Database Version March 2017. Geneva, Switzerland: World Health Organization (WHO), 2017

Qatar Vital Registration - Deaths 2015 ICD10 as it appears in World Health Organization (WHO). WHO Mortality Database Version October 2017. Geneva, Switzerland: World Health Organization (WHO), 2017

Qatar Vital Registration - Deaths 2016 ICD10 as it appears in World Health Organization (WHO). WHO Mortality Database Version April 2018. Geneva, Switzerland: World Health Organization (WHO), 2018

Registrar General’s Department (Sri Lanka). Sri Lanka Vital Registration - Deaths 2009

Registrar General’s Department (Sri Lanka). Sri Lanka Vital Registration - Deaths 2010

Registrar General's Department (Zimbabwe), Zimbabwe National Statistics Agency. Zimbabwe Mortality Report 2007

Republic of Moldova Vital Registration - Deaths 1981 ICD9 as it appears in World Health Organization (WHO). WHO Mortality Database Version September 2016. Geneva, Switzerland: World Health Organization (WHO), 2016

Republic of Moldova Vital Registration - Deaths 1982 ICD9 as it appears in World Health Organization (WHO). WHO Mortality Database Version September 2016. Geneva, Switzerland: World Health Organization (WHO), 2016

Republic of Moldova Vital Registration - Deaths 1985 ICD9 as it appears in World Health Organization (WHO). WHO Mortality Database Version September 2016. Geneva, Switzerland: World Health Organization (WHO), 2016

Republic of Moldova Vital Registration - Deaths 1986 ICD9 as it appears in World Health Organization (WHO). WHO Mortality Database Version September 2016. Geneva, Switzerland: World Health Organization (WHO), 2016

Republic of Moldova Vital Registration - Deaths 1987 ICD9 as it appears in World Health Organization (WHO). WHO Mortality Database Version September 2016. Geneva, Switzerland: World Health Organization (WHO), 2016

Republic of Moldova Vital Registration - Deaths 1988 ICD9 as it appears in World Health Organization (WHO). WHO Mortality Database Version September 2016. Geneva, Switzerland: World Health Organization (WHO), 2016

Republic of Moldova Vital Registration - Deaths 1989 ICD9 as it appears in World Health Organization (WHO). WHO Mortality Database Version September 2016. Geneva, Switzerland: World Health Organization (WHO), 2016

Republic of Moldova Vital Registration - Deaths 1990 ICD9 as it appears in World Health Organization (WHO). WHO Mortality Database Version September 2016. Geneva, Switzerland: World Health Organization (WHO), 2016

Republic of Moldova Vital Registration - Deaths 1991 ICD9 as it appears in World Health Organization (WHO). WHO Mortality Database Version September 2016. Geneva, Switzerland: World Health Organization (WHO), 2016

Republic of Moldova Vital Registration - Deaths 1992 ICD9 as it appears in World Health Organization (WHO). WHO Mortality Database Version September 2016. Geneva, Switzerland: World Health Organization (WHO), 2016

Republic of Moldova Vital Registration - Deaths 1993 ICD9 as it appears in World Health Organization (WHO). WHO Mortality Database Version September 2016. Geneva, Switzerland: World Health Organization (WHO), 2016

Republic of Moldova Vital Registration - Deaths 1994 ICD9 as it appears in World Health Organization (WHO). WHO Mortality Database Version September 2016. Geneva, Switzerland: World Health Organization (WHO), 2016

Republic of Moldova Vital Registration - Deaths 1995 ICD9 as it appears in World Health Organization (WHO). WHO Mortality Database Version September 2016. Geneva, Switzerland: World Health Organization (WHO), 2016
Republic of Moldova Vital Registration - Deaths 1996 ICD10 as it appears in World Health Organization (WHO). WHO Mortality Database Version October 2017. Geneva, Switzerland: World Health Organization (WHO), 2017
Republic of Moldova Vital Registration - Deaths 1997 ICD10 as it appears in World Health Organization (WHO). WHO Mortality Database Version October 2017. Geneva, Switzerland: World Health Organization (WHO), 2017
Republic of Moldova Vital Registration - Deaths 1998 ICD10 as it appears in World Health Organization (WHO). WHO Mortality Database Version October 2017. Geneva, Switzerland: World Health Organization (WHO), 2017
Republic of Moldova Vital Registration - Deaths 1999 ICD10 as it appears in World Health Organization (WHO). WHO Mortality Database Version October 2017. Geneva, Switzerland: World Health Organization (WHO), 2017
Republic of Moldova Vital Registration - Deaths 2000 ICD10 as it appears in World Health Organization (WHO). WHO Mortality Database Version October 2017. Geneva, Switzerland: World Health Organization (WHO), 2017
Republic of Moldova Vital Registration - Deaths 2001 ICD10 as it appears in World Health Organization (WHO). WHO Mortality Database Version October 2017. Geneva, Switzerland: World Health Organization (WHO), 2017
Republic of Moldova Vital Registration - Deaths 2002 ICD10 as it appears in World Health Organization (WHO). WHO Mortality Database Version October 2017. Geneva, Switzerland: World Health Organization (WHO), 2017
Republic of Moldova Vital Registration - Deaths 2003 ICD10 as it appears in World Health Organization (WHO). WHO Mortality Database Version October 2017. Geneva, Switzerland: World Health Organization (WHO), 2017
Republic of Moldova Vital Registration - Deaths 2004 ICD10 as it appears in World Health Organization (WHO). WHO Mortality Database Version October 2017. Geneva, Switzerland: World Health Organization (WHO), 2017
Republic of Moldova Vital Registration - Deaths 2005 ICD10 as it appears in World Health Organization (WHO). WHO Mortality Database Version October 2017. Geneva, Switzerland: World Health Organization (WHO), 2017
Republic of Moldova Vital Registration - Deaths 2006 ICD10 as it appears in World Health Organization (WHO). WHO Mortality Database Version October 2017. Geneva, Switzerland: World Health Organization (WHO), 2017
Republic of Moldova Vital Registration - Deaths 2007 ICD10 as it appears in World Health Organization (WHO). WHO Mortality Database Version October 2017. Geneva, Switzerland: World Health Organization (WHO), 2017
Republic of Moldova Vital Registration - Deaths 2008 ICD10 as it appears in World Health Organization (WHO). WHO Mortality Database Version October 2017. Geneva, Switzerland: World Health Organization (WHO), 2017
Republic of Moldova Vital Registration - Deaths 2009 ICD10 as it appears in World Health Organization (WHO). WHO Mortality Database Version October 2017. Geneva, Switzerland: World Health Organization (WHO), 2017
Romania Vital Registration - Deaths 1980 ICD9 as it appears in World Health Organization (WHO). WHO Mortality Database Version September 2016. Geneva, Switzerland: World Health Organization (WHO), 2016
Romania Vital Registration - Deaths 1981 ICD9 as it appears in World Health Organization (WHO). WHO Mortality Database Version September 2016. Geneva, Switzerland: World Health Organization (WHO), 2016
Romania Vital Registration - Deaths 1982 ICD9 as it appears in World Health Organization (WHO). WHO Mortality Database Version September 2016. Geneva, Switzerland: World Health Organization (WHO), 2016
Romania Vital Registration - Deaths 1983 ICD9 as it appears in World Health Organization (WHO). WHO Mortality Database Version September 2016. Geneva, Switzerland: World Health Organization (WHO), 2016
Romania Vital Registration - Deaths 1984 ICD9 as it appears in World Health Organization (WHO). WHO Mortality Database Version September 2016. Geneva, Switzerland: World Health Organization (WHO), 2016

Romania Vital Registration - Deaths 1985 ICD9 as it appears in World Health Organization (WHO). WHO Mortality Database Version September 2016. Geneva, Switzerland: World Health Organization (WHO), 2016
Romania Vital Registration - Deaths 1986 ICD9 as it appears in World Health Organization (WHO). WHO Mortality Database Version September 2016. Geneva, Switzerland: World Health Organization (WHO), 2016
Romania Vital Registration - Deaths 1987 ICD9 as it appears in World Health Organization (WHO). WHO Mortality Database Version September 2016. Geneva, Switzerland: World Health Organization (WHO), 2016
Romania Vital Registration - Deaths 1988 ICD9 as it appears in World Health Organization (WHO). WHO Mortality Database Version September 2016. Geneva, Switzerland: World Health Organization (WHO), 2016
Romania Vital Registration - Deaths 1989 ICD9 as it appears in World Health Organization (WHO). WHO Mortality Database Version September 2016. Geneva, Switzerland: World Health Organization (WHO), 2016
Romania Vital Registration - Deaths 1990 ICD9 as it appears in World Health Organization (WHO). WHO Mortality Database Version September 2016. Geneva, Switzerland: World Health Organization (WHO), 2016
Romania Vital Registration - Deaths 1991 ICD9 as it appears in World Health Organization (WHO). WHO Mortality Database Version September 2016. Geneva, Switzerland: World Health Organization (WHO), 2016
Romania Vital Registration - Deaths 1992 ICD9 as it appears in World Health Organization (WHO). WHO Mortality Database Version September 2016. Geneva, Switzerland: World Health Organization (WHO), 2016
Romania Vital Registration - Deaths 1993 ICD9 as it appears in World Health Organization (WHO). WHO Mortality Database Version September 2016. Geneva, Switzerland: World Health Organization (WHO), 2016
Romania Vital Registration - Deaths 1994 ICD9 as it appears in World Health Organization (WHO). WHO Mortality Database Version September 2016. Geneva, Switzerland: World Health Organization (WHO), 2016
Romania Vital Registration - Deaths 1995 ICD9 as it appears in World Health Organization (WHO). WHO Mortality Database Version September 2016. Geneva, Switzerland: World Health Organization (WHO), 2016
Romania Vital Registration - Deaths 1996 ICD9 as it appears in World Health Organization (WHO). WHO Mortality Database Version September 2016. Geneva, Switzerland: World Health Organization (WHO), 2016
Romania Vital Registration - Deaths 1997 ICD9 as it appears in World Health Organization (WHO). WHO Mortality Database Version September 2016. Geneva, Switzerland: World Health Organization (WHO), 2016
Romania Vital Registration - Deaths 1998 ICD9 as it appears in World Health Organization (WHO). WHO Mortality Database Version September 2016. Geneva, Switzerland: World Health Organization (WHO), 2016
Romania Vital Registration - Deaths 1999 ICD10 as it appears in World Health Organization (WHO). WHO Mortality Database Version October 2017. Geneva, Switzerland: World Health Organization (WHO), 2017
Romania Vital Registration - Deaths 2000 ICD10 as it appears in World Health Organization (WHO). WHO Mortality Database Version October 2017. Geneva, Switzerland: World Health Organization (WHO), 2017
Romania Vital Registration - Deaths 2001 ICD10 as it appears in World Health Organization (WHO). WHO Mortality Database Version October 2017. Geneva, Switzerland: World Health Organization (WHO), 2017
Romania Vital Registration - Deaths 2002 ICD10 as it appears in World Health Organization (WHO). WHO Mortality Database Version October 2017. Geneva, Switzerland: World Health Organization (WHO), 2017
Romania Vital Registration - Deaths 2003 ICD10 as it appears in World Health Organization (WHO). WHO Mortality Database Version October 2017. Geneva, Switzerland: World Health Organization (WHO), 2017
Romania Vital Registration - Deaths 2004 ICD10 as it appears in World Health Organization (WHO). WHO Mortality Database Version October 2017. Geneva, Switzerland: World Health Organization (WHO), 2017

Romania Vital Registration - Deaths 2005 ICD10 as it appears in World Health Organization (WHO). WHO Mortality Database Version October 2017. Geneva, Switzerland: World Health Organization (WHO), 2017
Romania Vital Registration - Deaths 2006 ICD10 as it appears in World Health Organization (WHO). WHO Mortality Database Version October 2017. Geneva, Switzerland: World Health Organization (WHO), 2017
Romania Vital Registration - Deaths 2007 ICD10 as it appears in World Health Organization (WHO). WHO Mortality Database Version October 2017. Geneva, Switzerland: World Health Organization (WHO), 2017
Romania Vital Registration - Deaths 2008 ICD10 as it appears in World Health Organization (WHO). WHO Mortality Database Version October 2017. Geneva, Switzerland: World Health Organization (WHO), 2017
Romania Vital Registration - Deaths 2009 ICD10 as it appears in World Health Organization (WHO). WHO Mortality Database Version October 2017. Geneva, Switzerland: World Health Organization (WHO), 2017
Romania Vital Registration - Deaths 2010 ICD10 as it appears in World Health Organization (WHO). WHO Mortality Database Version October 2017. Geneva, Switzerland: World Health Organization (WHO), 2017
Romania Vital Registration - Deaths 2011 ICD10 as it appears in World Health Organization (WHO). WHO Mortality Database Version October 2017. Geneva, Switzerland: World Health Organization (WHO), 2017
Romania Vital Registration - Deaths 2012 ICD10 as it appears in World Health Organization (WHO). WHO Mortality Database Version October 2017. Geneva, Switzerland: World Health Organization (WHO), 2017
Romania Vital Registration - Deaths 2013 ICD10 as it appears in World Health Organization (WHO). WHO Mortality Database Version October 2017. Geneva, Switzerland: World Health Organization (WHO), 2017
Romania Vital Registration - Deaths 2014 ICD10 as it appears in World Health Organization (WHO). WHO Mortality Database Version October 2017. Geneva, Switzerland: World Health Organization (WHO), 2017
Romania Vital Registration - Deaths 2015 ICD10 as it appears in World Health Organization (WHO). WHO Mortality Database Version March 2017. Geneva, Switzerland: World Health Organization (WHO), 2017
Romania Vital Registration - Deaths 2016 ICD10 as it appears in World Health Organization (WHO). WHO Mortality Database Version April 2018. Geneva, Switzerland: World Health Organization (WHO), 2018
Russia Mortality by Region, Age, Sex, and Cause of Death 1989 as it appears in Center for Demographic Research, New Economic School (Russia). Russia Mortality Rates by Region, Age, Sex, and Cause of Death 1989-1998. Moscow, Russia: Center for Demographic Research, New Economic School (Russia). http://demogr.nes.ru/index.php/ru/demogr_indicat/data
Russia Mortality by Region, Age, Sex, and Cause of Death 1990 as it appears in Center for Demographic Research, New Economic School (Russia). Russia Mortality Rates by Region, Age, Sex, and Cause of Death 1989-1998. Moscow, Russia: Center for Demographic Research, New Economic School (Russia). http://demogr.nes.ru/index.php/ru/demogr_indicat/data
Russia Mortality by Region, Age, Sex, and Cause of Death 1991 as it appears in Center for Demographic Research, New Economic School (Russia). Russia Mortality Rates by Region, Age, Sex, and Cause of Death 1989-1998. Moscow, Russia: Center for Demographic Research, New Economic School (Russia). http://demogr.nes.ru/index.php/ru/demogr_indicat/data
Russia Mortality by Region, Age, Sex, and Cause of Death 1992 as it appears in Center for Demographic Research, New Economic School (Russia). Russia Mortality Rates by Region, Age, Sex, and Cause of Death 1989-1998. Moscow, Russia: Center for Demographic Research, New Economic School (Russia). http://demogr.nes.ru/index.php/ru/demogr_indicat/data
Russia Mortality by Region, Age, Sex, and Cause of Death 1993 as it appears in Center for Demographic Research, New Economic School (Russia). Russia Mortality Rates by Region, Age, Sex, and Cause of Death 1989-1998. Moscow, Russia: Center for Demographic Research, New Economic School (Russia). http://demogr.nes.ru/index.php/ru/demogr_indicat/data
Russia Mortality by Region, Age, Sex, and Cause of Death 1994 as it appears in Center for Demographic Research, New Economic School (Russia). Russia Mortality Rates by Region, Age, Sex, and Cause of Death 1989-1998. Moscow, Russia: Center for Demographic Research, New Economic School (Russia).

http://demogr.nes.ru/index.php/ru/demogr_indicat/data

Russia Mortality by Region, Age, Sex, and Cause of Death 1995 as it appears in Center for Demographic Research, New Economic School (Russia). Russia Mortality Rates by Region, Age, Sex, and Cause of Death 1989-1998. Moscow, Russia: Center for Demographic Research, New Economic School (Russia). http://demogr.nes.ru/index.php/ru/demogr_indicat/data

Russia Mortality by Region, Age, Sex, and Cause of Death 1996 as it appears in Center for Demographic Research, New Economic School (Russia). Russia Mortality Rates by Region, Age, Sex, and Cause of Death 1989-1998. Moscow, Russia: Center for Demographic Research, New Economic School (Russia). http://demogr.nes.ru/index.php/ru/demogr_indicat/data

Russia Mortality by Region, Age, Sex, and Cause of Death 1997 as it appears in Center for Demographic Research, New Economic School (Russia). Russia Mortality Rates by Region, Age, Sex, and Cause of Death 1989-1998. Moscow, Russia: Center for Demographic Research, New Economic School (Russia). http://demogr.nes.ru/index.php/ru/demogr_indicat/data

Russia Mortality by Region, Age, Sex, and Cause of Death 1998 as it appears in Center for Demographic Research, New Economic School (Russia). Russia Mortality Rates by Region, Age, Sex, and Cause of Death 1989-1998. Moscow, Russia: Center for Demographic Research, New Economic School (Russia). http://demogr.nes.ru/index.php/ru/demogr_indicat/data

Russia Mortality by Region, Age, Sex, and Cause of Death 1999 as it appears in Center for Demographic Research, New Economic School (Russia). Russia Mortality Rates by Region, Age, Sex, and Cause of Death 1999-2005. Moscow, Russia: Center for Demographic Research, New Economic School (Russia). http://demogr.nes.ru/index.php/ru/demogr_indicat/data

Russia Mortality by Region, Age, Sex, and Cause of Death 2000 as it appears in Center for Demographic Research, New Economic School (Russia). Russia Mortality Rates by Region, Age, Sex, and Cause of Death 1999-2005. Moscow, Russia: Center for Demographic Research, New Economic School (Russia). http://demogr.nes.ru/index.php/ru/demogr_indicat/data

Russia Mortality by Region, Age, Sex, and Cause of Death 2001 as it appears in Center for Demographic Research, New Economic School (Russia). Russia Mortality Rates by Region, Age, Sex, and Cause of Death 1999-2005. Moscow, Russia: Center for Demographic Research, New Economic School (Russia). http://demogr.nes.ru/index.php/ru/demogr_indicat/data

Russia Mortality by Region, Age, Sex, and Cause of Death 2002 as it appears in Center for Demographic Research, New Economic School (Russia). Russia Mortality Rates by Region, Age, Sex, and Cause of Death 1999-2005. Moscow, Russia: Center for Demographic Research, New Economic School (Russia). http://demogr.nes.ru/index.php/ru/demogr_indicat/data

Russia Mortality by Region, Age, Sex, and Cause of Death 2003 as it appears in Center for Demographic Research, New Economic School (Russia). Russia Mortality Rates by Region, Age, Sex, and Cause of Death 1999-2005. Moscow, Russia: Center for Demographic Research, New Economic School (Russia). http://demogr.nes.ru/index.php/ru/demogr_indicat/data

Russia Mortality by Region, Age, Sex, and Cause of Death 2004 as it appears in Center for Demographic Research, New Economic School (Russia). Russia Mortality Rates by Region, Age, Sex, and Cause of Death 1999-2005. Moscow, Russia: Center for Demographic Research, New Economic School (Russia). http://demogr.nes.ru/index.php/ru/demogr_indicat/data

Russia Mortality by Region, Age, Sex, and Cause of Death 2005 as it appears in Center for Demographic Research, New Economic School (Russia). Russia Mortality Rates by Region, Age, Sex, and Cause of Death 1999-2005. Moscow, Russia: Center for Demographic Research, New Economic School (Russia). http://demogr.nes.ru/index.php/ru/demogr_indicat/data

Russia Mortality by Region, Age, Sex, and Cause of Death 2006 as it appears in Center for Demographic Research, New Economic School (Russia). Russia Mortality Rates by Region, Age, Sex, and Cause of Death 2006-2012. Moscow, Russia: Center for Demographic Research, New Economic School (Russia). http://demogr.nes.ru/index.php/ru/demogr_indicat/data

Russia Mortality by Region, Age, Sex, and Cause of Death 2007 as it appears in Center for Demographic Research, New Economic School (Russia). Russia Mortality Rates by Region, Age, Sex, and Cause of Death 2006-2012. Moscow, Russia: Center for Demographic Research, New Economic School (Russia). http://demogr.nes.ru/index.php/ru/demogr_indicat/data

Russia Mortality by Region, Age, Sex, and Cause of Death 2008 as it appears in Center for Demographic Research, New Economic School (Russia). Russia Mortality Rates by Region, Age, Sex, and Cause of Death 2006-2012. Moscow, Russia: Center for Demographic Research, New Economic School (Russia). http://demogr.nes.ru/index.php/ru/demogr_indicat/data
Russia Mortality by Region, Age, Sex, and Cause of Death 2009 as it appears in Center for Demographic Research, New Economic School (Russia). Russia Mortality Rates by Region, Age, Sex, and Cause of Death 2006-2012. Moscow, Russia: Center for Demographic Research, New Economic School (Russia). http://demogr.nes.ru/index.php/ru/demogr_indicat/data
Russia Mortality by Region, Age, Sex, and Cause of Death 2010 as it appears in Center for Demographic Research, New Economic School (Russia). Russia Mortality Rates by Region, Age, Sex, and Cause of Death 2006-2012. Moscow, Russia: Center for Demographic Research, New Economic School (Russia). http://demogr.nes.ru/index.php/ru/demogr_indicat/data
Russia Mortality by Region, Age, Sex, and Cause of Death 2011 as it appears in Center for Demographic Research, New Economic School (Russia). Russia Mortality Rates by Region, Cause of Death, 5-Year Age Groups, and Sex, 2011-2014. Moscow, Russia: Center for Demographic Research, New Economic School (Russia). http://demogr.nes.ru/index.php/ru/demogr_indicat/data
Russia Mortality by Region, Age, Sex, and Cause of Death 2012 as it appears in Center for Demographic Research, New Economic School (Russia). Russia Mortality Rates by Region, Cause of Death, 5-Year Age Groups, and Sex, 2011-2014. Moscow, Russia: Center for Demographic Research, New Economic School (Russia). http://demogr.nes.ru/index.php/ru/demogr_indicat/data
Russia Mortality by Region, Age, Sex, and Cause of Death 2013 as it appears in Center for Demographic Research, New Economic School (Russia). Russia Mortality Rates by Region, Cause of Death, 5-Year Age Groups, and Sex, 2011-2014. Moscow, Russia: Center for Demographic Research, New Economic School (Russia). http://demogr.nes.ru/index.php/ru/demogr_indicat/data
Russia Mortality by Region, Age, Sex, and Cause of Death 2014 as it appears in Center for Demographic Research, New Economic School (Russia). Russia Mortality Rates by Region, Cause of Death, 5-Year Age Groups, and Sex, 2011-2014. Moscow, Russia: Center for Demographic Research, New Economic School (Russia). http://demogr.nes.ru/index.php/ru/demogr_indicat/data
Russia Mortality by Region, Age, Sex, and Cause of Death 2015 as it appears in Center for Demographic Research, New Economic School (Russia). Russia Mortality Rates by Region, 1-Year Age Groups, and Sex, 2015-2016. Moscow, Russia: Center for Demographic Research, New Economic School (Russia). http://demogr.nes.ru/index.php/ru/demogr_indicat/data
Russia Mortality by Region, Age, Sex, and Cause of Death 2016 as it appears in Center for Demographic Research, New Economic School (Russia). Russia Mortality Rates by Region, 1-Year Age Groups, and Sex, 2015-2016. Moscow, Russia: Center for Demographic Research, New Economic School (Russia). http://demogr.nes.ru/index.php/ru/demogr_indicat/data
Saint Lucia Vital Registration - Deaths 1980 ICD9 as it appears in World Health Organization (WHO). WHO Mortality Database Version September 2016. Geneva, Switzerland: World Health Organization (WHO), 2016
Saint Lucia Vital Registration - Deaths 1981 ICD9 as it appears in World Health Organization (WHO). WHO Mortality Database Version September 2016. Geneva, Switzerland: World Health Organization (WHO), 2016
Saint Lucia Vital Registration - Deaths 1983 ICD9 as it appears in World Health Organization (WHO). WHO Mortality Database Version September 2016. Geneva, Switzerland: World Health Organization (WHO), 2016
Saint Lucia Vital Registration - Deaths 1986 ICD9 as it appears in World Health Organization (WHO). WHO Mortality Database Version September 2016. Geneva, Switzerland: World Health Organization (WHO), 2016
Saint Lucia Vital Registration - Deaths 1987 ICD9 as it appears in World Health Organization (WHO). WHO Mortality Database Version September 2016. Geneva, Switzerland: World Health Organization (WHO), 2016
Saint Lucia Vital Registration - Deaths 1988 ICD9 as it appears in World Health Organization (WHO). WHO Mortality Database Version September 2016. Geneva, Switzerland: World Health Organization (WHO), 2016
Saint Lucia Vital Registration - Deaths 1989 ICD9 as it appears in World Health Organization (WHO). WHO Mortality Database Version September 2016. Geneva, Switzerland: World Health Organization (WHO), 2016

Saint Lucia Vital Registration - Deaths 1990 ICD9 as it appears in World Health Organization (WHO). WHO Mortality Database Version September 2016. Geneva, Switzerland: World Health Organization (WHO), 2016
Saint Lucia Vital Registration - Deaths 1991 ICD9 as it appears in World Health Organization (WHO). WHO Mortality Database Version September 2016. Geneva, Switzerland: World Health Organization (WHO), 2016
Saint Lucia Vital Registration - Deaths 1992 ICD9 as it appears in World Health Organization (WHO). WHO Mortality Database Version September 2016. Geneva, Switzerland: World Health Organization (WHO), 2016
Saint Lucia Vital Registration - Deaths 1993 ICD9 as it appears in World Health Organization (WHO). WHO Mortality Database Version September 2016. Geneva, Switzerland: World Health Organization (WHO), 2016
Saint Lucia Vital Registration - Deaths 1994 ICD9 as it appears in World Health Organization (WHO). WHO Mortality Database Version September 2016. Geneva, Switzerland: World Health Organization (WHO), 2016
Saint Lucia Vital Registration - Deaths 1995 ICD9 as it appears in World Health Organization (WHO). WHO Mortality Database Version September 2016. Geneva, Switzerland: World Health Organization (WHO), 2016
Saint Lucia Vital Registration - Deaths 1996 ICD10 as it appears in World Health Organization (WHO). WHO Mortality Database Version October 2017. Geneva, Switzerland: World Health Organization (WHO), 2017
Saint Lucia Vital Registration - Deaths 1997 ICD10 as it appears in World Health Organization (WHO). WHO Mortality Database Version October 2017. Geneva, Switzerland: World Health Organization (WHO), 2017
Saint Lucia Vital Registration - Deaths 1998 ICD10 as it appears in World Health Organization (WHO). WHO Mortality Database Version October 2017. Geneva, Switzerland: World Health Organization (WHO), 2017
Saint Lucia Vital Registration - Deaths 1999 ICD10 as it appears in World Health Organization (WHO). WHO Mortality Database Version October 2017. Geneva, Switzerland: World Health Organization (WHO), 2017
Saint Lucia Vital Registration - Deaths 2000 ICD10 as it appears in World Health Organization (WHO). WHO Mortality Database Version October 2017. Geneva, Switzerland: World Health Organization (WHO), 2017
Saint Lucia Vital Registration - Deaths 2001 ICD10 as it appears in World Health Organization (WHO). WHO Mortality Database Version October 2017. Geneva, Switzerland: World Health Organization (WHO), 2017
Saint Lucia Vital Registration - Deaths 2002 ICD10 as it appears in World Health Organization (WHO). WHO Mortality Database Version October 2017. Geneva, Switzerland: World Health Organization (WHO), 2017
Saint Lucia Vital Registration - Deaths 2003 ICD10 as it appears in World Health Organization (WHO). WHO Mortality Database Version October 2017. Geneva, Switzerland: World Health Organization (WHO), 2017
Saint Lucia Vital Registration - Deaths 2004 ICD10 as it appears in World Health Organization (WHO). WHO Mortality Database Version October 2017. Geneva, Switzerland: World Health Organization (WHO), 2017
Saint Lucia Vital Registration - Deaths 2005 ICD10 as it appears in World Health Organization (WHO). WHO Mortality Database Version October 2017. Geneva, Switzerland: World Health Organization (WHO), 2017
Saint Lucia Vital Registration - Deaths 2006 ICD10 as it appears in World Health Organization (WHO). WHO Mortality Database Version October 2017. Geneva, Switzerland: World Health Organization (WHO), 2017
Saint Lucia Vital Registration - Deaths 2008 ICD10 as it appears in World Health Organization (WHO). WHO Mortality Database Version October 2017. Geneva, Switzerland: World Health Organization (WHO), 2017
Saint Lucia Vital Registration - Deaths 2009 ICD10 as it appears in World Health Organization (WHO). WHO Mortality Database Version October 2017. Geneva, Switzerland: World Health Organization (WHO), 2017
Saint Lucia Vital Registration - Deaths 2010 ICD10 as it appears in World Health Organization (WHO). WHO Mortality Database Version October 2017. Geneva, Switzerland: World Health Organization (WHO), 2017

Saint Lucia Vital Registration - Deaths 2011 ICD10 as it appears in World Health Organization (WHO). WHO Mortality Database Version October 2017. Geneva, Switzerland: World Health Organization (WHO), 2017
Saint Lucia Vital Registration - Deaths 2012 ICD10 as it appears in World Health Organization (WHO). WHO Mortality Database Version October 2017. Geneva, Switzerland: World Health Organization (WHO), 2017
Saint Lucia Vital Registration - Deaths 2013 ICD10 as it appears in World Health Organization (WHO). WHO Mortality Database Version October 2017. Geneva, Switzerland: World Health Organization (WHO), 2017
Saint Lucia Vital Registration - Deaths 2014 ICD10 as it appears in World Health Organization (WHO). WHO Mortality Database Version October 2017. Geneva, Switzerland: World Health Organization (WHO), 2017
Saint Vincent and the Grenadines Vital Registration - Deaths 1982 ICD9 as it appears in World Health Organization (WHO). WHO Mortality Database Version September 2016. Geneva, Switzerland: World Health Organization (WHO), 2016
Saint Vincent and the Grenadines Vital Registration - Deaths 1983 ICD9 as it appears in World Health Organization (WHO). WHO Mortality Database Version September 2016. Geneva, Switzerland: World Health Organization (WHO), 2016
Saint Vincent and the Grenadines Vital Registration - Deaths 1984 ICD9 as it appears in World Health Organization (WHO). WHO Mortality Database Version September 2016. Geneva, Switzerland: World Health Organization (WHO), 2016
Saint Vincent and the Grenadines Vital Registration - Deaths 1985 ICD9 as it appears in World Health Organization (WHO). WHO Mortality Database Version September 2016. Geneva, Switzerland: World Health Organization (WHO), 2016
Saint Vincent and the Grenadines Vital Registration - Deaths 1986 ICD9 as it appears in World Health Organization (WHO). WHO Mortality Database Version September 2016. Geneva, Switzerland: World Health Organization (WHO), 2016
Saint Vincent and the Grenadines Vital Registration - Deaths 1987 ICD9 as it appears in World Health Organization (WHO). WHO Mortality Database Version September 2016. Geneva, Switzerland: World Health Organization (WHO), 2016
Saint Vincent and the Grenadines Vital Registration - Deaths 1990 ICD9 as it appears in World Health Organization (WHO). WHO Mortality Database Version November 2015. Geneva, Switzerland: World Health Organization (WHO), 2015
Saint Vincent and the Grenadines Vital Registration - Deaths 1995 ICD9 as it appears in World Health Organization (WHO). WHO Mortality Database Version September 2016. Geneva, Switzerland: World Health Organization (WHO), 2016
Saint Vincent and the Grenadines Vital Registration - Deaths 1996 ICD9 as it appears in World Health Organization (WHO). WHO Mortality Database Version September 2016. Geneva, Switzerland: World Health Organization (WHO), 2016
Saint Vincent and the Grenadines Vital Registration - Deaths 1997 ICD9 as it appears in World Health Organization (WHO). WHO Mortality Database Version September 2016. Geneva, Switzerland: World Health Organization (WHO), 2016
Saint Vincent and the Grenadines Vital Registration - Deaths 1998 ICD9 as it appears in World Health Organization (WHO). WHO Mortality Database Version September 2016. Geneva, Switzerland: World Health Organization (WHO), 2016
Saint Vincent and the Grenadines Vital Registration - Deaths 1999 ICD9 as it appears in World Health Organization (WHO). WHO Mortality Database Version September 2016. Geneva, Switzerland: World Health Organization (WHO), 2016
Saint Vincent and the Grenadines Vital Registration - Deaths 2000 ICD10 as it appears in World Health Organization (WHO). WHO Mortality Database Version October 2017. Geneva, Switzerland: World Health Organization (WHO), 2017
Saint Vincent and the Grenadines Vital Registration - Deaths 2001 ICD10 as it appears in World Health Organization (WHO). WHO Mortality Database Version October 2017. Geneva, Switzerland: World Health Organization (WHO), 2017
Saint Vincent and the Grenadines Vital Registration - Deaths 2002 ICD10 as it appears in World Health Organization (WHO). WHO Mortality Database Version October 2017. Geneva, Switzerland: World Health Organization (WHO), 2017
Saint Vincent and the Grenadines Vital Registration - Deaths 2003 ICD10 as it appears in World Health Organization (WHO). WHO Mortality Database Version October 2017. Geneva, Switzerland: World Health Organization (WHO), 2017

Saint Vincent and the Grenadines Vital Registration - Deaths 2004 ICD10 as it appears in World Health Organization (WHO). WHO Mortality Database Version October 2017. Geneva, Switzerland: World Health Organization (WHO), 2017
Saint Vincent and the Grenadines Vital Registration - Deaths 2005 ICD10 as it appears in World Health Organization (WHO). WHO Mortality Database Version October 2017. Geneva, Switzerland: World Health Organization (WHO), 2017
Saint Vincent and the Grenadines Vital Registration - Deaths 2006 ICD10 as it appears in World Health Organization (WHO). WHO Mortality Database Version October 2017. Geneva, Switzerland: World Health Organization (WHO), 2017
Saint Vincent and the Grenadines Vital Registration - Deaths 2007 ICD10 as it appears in World Health Organization (WHO). WHO Mortality Database Version October 2017. Geneva, Switzerland: World Health Organization (WHO), 2017
Saint Vincent and the Grenadines Vital Registration - Deaths 2008 ICD10 as it appears in World Health Organization (WHO). WHO Mortality Database Version October 2017. Geneva, Switzerland: World Health Organization (WHO), 2017
Saint Vincent and the Grenadines Vital Registration - Deaths 2009 ICD10 as it appears in World Health Organization (WHO). WHO Mortality Database Version October 2017. Geneva, Switzerland: World Health Organization (WHO), 2017
Saint Vincent and the Grenadines Vital Registration - Deaths 2010 ICD10 as it appears in World Health Organization (WHO). WHO Mortality Database Version October 2017. Geneva, Switzerland: World Health Organization (WHO), 2017
Saint Vincent and the Grenadines Vital Registration - Deaths 2011 ICD10 as it appears in World Health Organization (WHO). WHO Mortality Database Version October 2017. Geneva, Switzerland: World Health Organization (WHO), 2017
Saint Vincent and the Grenadines Vital Registration - Deaths 2012 ICD10 as it appears in World Health Organization (WHO). WHO Mortality Database Version October 2017. Geneva, Switzerland: World Health Organization (WHO), 2017
Saint Vincent and the Grenadines Vital Registration - Deaths 2013 ICD10 as it appears in World Health Organization (WHO). WHO Mortality Database Version October 2017. Geneva, Switzerland: World Health Organization (WHO), 2017
Saint Vincent and the Grenadines Vital Registration - Deaths 2014 ICD10 as it appears in World Health Organization (WHO). WHO Mortality Database Version October 2017. Geneva, Switzerland: World Health Organization (WHO), 2017
Saint Vincent and the Grenadines Vital Registration - Deaths 2015 ICD10 as it appears in World Health Organization (WHO). WHO Mortality Database Version October 2017. Geneva, Switzerland: World Health Organization (WHO), 2017
Sao Tome and Principe Vital Registration - Deaths 1985 ICD9 as it appears in World Health Organization (WHO). WHO Mortality Database Version September 2016. Geneva, Switzerland: World Health Organization (WHO), 2016
Serbia and Montenegro - Montenegro Vital Registration - Deaths 2003 ICD10 as it appears in World Health Organization (WHO). WHO Mortality Database Version October 2017. Geneva, Switzerland: World Health Organization (WHO), 2017
Serbia and Montenegro - Montenegro Vital Registration - Deaths 2004 ICD10 as it appears in World Health Organization (WHO). WHO Mortality Database Version October 2017. Geneva, Switzerland: World Health Organization (WHO), 2017
Serbia and Montenegro - Serbia Vital Registration - Deaths 2003 ICD10 as it appears in World Health Organization (WHO). WHO Mortality Database Version October 2017. Geneva, Switzerland: World Health Organization (WHO), 2017
Serbia and Montenegro - Serbia Vital Registration - Deaths 2004 ICD10 as it appears in World Health Organization (WHO). WHO Mortality Database Version October 2017. Geneva, Switzerland: World Health Organization (WHO), 2017
Serbia and Montenegro - Serbia Vital Registration - Deaths 2005 ICD10 as it appears in World Health Organization (WHO). WHO Mortality Database Version October 2017. Geneva, Switzerland: World Health Organization (WHO), 2017
Serbia and Montenegro - Serbia Vital Registration - Deaths 2006 ICD10 as it appears in World Health Organization (WHO). WHO Mortality Database Version October 2017. Geneva, Switzerland: World Health Organization (WHO), 2017
Serbia Vital Registration - Deaths 2007 ICD10 as it appears in World Health Organization (WHO). WHO Mortality Database Version October 2017. Geneva, Switzerland: World Health Organization (WHO), 2017
Serbia Vital Registration - Deaths 2008 ICD10 as it appears in World Health Organization (WHO). WHO Mortality Database Version October 2017. Geneva, Switzerland:

World Health Organization (WHO), 2017
Serbia Vital Registration - Deaths 2009 ICD10 as it appears in World Health Organization (WHO). WHO Mortality Database Version October 2017. Geneva, Switzerland: World Health Organization (WHO), 2017
Serbia Vital Registration - Deaths 2010 ICD10 as it appears in World Health Organization (WHO). WHO Mortality Database Version October 2017. Geneva, Switzerland: World Health Organization (WHO), 2017
Serbia Vital Registration - Deaths 2011 ICD10 as it appears in World Health Organization (WHO). WHO Mortality Database Version October 2017. Geneva, Switzerland: World Health Organization (WHO), 2017
Serbia Vital Registration - Deaths 2012 ICD10 as it appears in World Health Organization (WHO). WHO Mortality Database Version October 2017. Geneva, Switzerland: World Health Organization (WHO), 2017
Serbia Vital Registration - Deaths 2013 ICD10 as it appears in World Health Organization (WHO). WHO Mortality Database Version October 2017. Geneva, Switzerland: World Health Organization (WHO), 2017
Serbia Vital Registration - Deaths 2014 ICD10 as it appears in World Health Organization (WHO). WHO Mortality Database Version October 2017. Geneva, Switzerland: World Health Organization (WHO), 2017
Serbia Vital Registration - Deaths 2015 ICD10 as it appears in World Health Organization (WHO). WHO Mortality Database Version March 2017. Geneva, Switzerland: World Health Organization (WHO), 2017
Seychelles Vital Registration - Deaths 1981 ICD9 as it appears in World Health Organization (WHO). WHO Mortality Database Version September 2016. Geneva, Switzerland: World Health Organization (WHO), 2016
Seychelles Vital Registration - Deaths 1982 ICD9 as it appears in World Health Organization (WHO). WHO Mortality Database Version September 2016. Geneva, Switzerland: World Health Organization (WHO), 2016
Seychelles Vital Registration - Deaths 1985 ICD9 as it appears in World Health Organization (WHO). WHO Mortality Database Version September 2016. Geneva, Switzerland: World Health Organization (WHO), 2016
Seychelles Vital Registration - Deaths 1986 ICD9 as it appears in World Health Organization (WHO). WHO Mortality Database Version September 2016. Geneva, Switzerland: World Health Organization (WHO), 2016
Seychelles Vital Registration - Deaths 1987 ICD9 as it appears in World Health Organization (WHO). WHO Mortality Database Version September 2016. Geneva, Switzerland: World Health Organization (WHO), 2016
Slovakia Vital Registration - Deaths 1992 ICD9 as it appears in World Health Organization (WHO). WHO Mortality Database Version November 2015. Geneva, Switzerland: World Health Organization (WHO), 2015
Slovakia Vital Registration - Deaths 1993 ICD9 as it appears in World Health Organization (WHO). WHO Mortality Database Version November 2015. Geneva, Switzerland: World Health Organization (WHO), 2015
Slovakia Vital Registration - Deaths 1994 ICD10 as it appears in World Health Organization (WHO). WHO Mortality Database Version October 2017. Geneva, Switzerland: World Health Organization (WHO), 2017
Slovakia Vital Registration - Deaths 1995 ICD10 as it appears in World Health Organization (WHO). WHO Mortality Database Version October 2017. Geneva, Switzerland: World Health Organization (WHO), 2017
Slovakia Vital Registration - Deaths 1996 ICD10 as it appears in World Health Organization (WHO). WHO Mortality Database Version October 2017. Geneva, Switzerland: World Health Organization (WHO), 2017
Slovakia Vital Registration - Deaths 1997 ICD10 as it appears in World Health Organization (WHO). WHO Mortality Database Version October 2017. Geneva, Switzerland: World Health Organization (WHO), 2017
Slovakia Vital Registration - Deaths 1998 ICD10 as it appears in World Health Organization (WHO). WHO Mortality Database Version October 2017. Geneva, Switzerland: World Health Organization (WHO), 2017
Slovakia Vital Registration - Deaths 1999 ICD10 as it appears in World Health Organization (WHO). WHO Mortality Database Version October 2017. Geneva, Switzerland: World Health Organization (WHO), 2017

Slovakia Vital Registration - Deaths 2000 ICD10 as it appears in World Health Organization (WHO). WHO Mortality Database Version October 2017. Geneva, Switzerland: World Health Organization (WHO), 2017
Slovakia Vital Registration - Deaths 2001 ICD10 as it appears in World Health Organization (WHO). WHO Mortality Database Version October 2017. Geneva, Switzerland: World Health Organization (WHO), 2017
Slovakia Vital Registration - Deaths 2002 ICD10 as it appears in World Health Organization (WHO). WHO Mortality Database Version October 2017. Geneva, Switzerland: World Health Organization (WHO), 2017
Slovakia Vital Registration - Deaths 2003 ICD10 as it appears in World Health Organization (WHO). WHO Mortality Database Version October 2017. Geneva, Switzerland: World Health Organization (WHO), 2017
Slovakia Vital Registration - Deaths 2004 ICD10 as it appears in World Health Organization (WHO). WHO Mortality Database Version October 2017. Geneva, Switzerland: World Health Organization (WHO), 2017
Slovakia Vital Registration - Deaths 2005 ICD10 as it appears in World Health Organization (WHO). WHO Mortality Database Version October 2017. Geneva, Switzerland: World Health Organization (WHO), 2017
Slovakia Vital Registration - Deaths 2006 ICD10 as it appears in World Health Organization (WHO). WHO Mortality Database Version October 2017. Geneva, Switzerland: World Health Organization (WHO), 2017
Slovakia Vital Registration - Deaths 2007 ICD10 as it appears in World Health Organization (WHO). WHO Mortality Database Version October 2017. Geneva, Switzerland: World Health Organization (WHO), 2017
Slovakia Vital Registration - Deaths 2008 ICD10 as it appears in World Health Organization (WHO). WHO Mortality Database Version October 2017. Geneva, Switzerland: World Health Organization (WHO), 2017
Slovakia Vital Registration - Deaths 2009 ICD10 as it appears in World Health Organization (WHO). WHO Mortality Database Version October 2017. Geneva, Switzerland: World Health Organization (WHO), 2017
Slovakia Vital Registration - Deaths 2010 ICD10 as it appears in World Health Organization (WHO). WHO Mortality Database Version October 2017. Geneva, Switzerland: World Health Organization (WHO), 2017
Slovakia Vital Registration - Deaths 2012 ICD10 as it appears in World Health Organization (WHO). WHO Mortality Database Version October 2017. Geneva, Switzerland: World Health Organization (WHO), 2017
Slovakia Vital Registration - Deaths 2013 ICD10 as it appears in World Health Organization (WHO). WHO Mortality Database Version October 2017. Geneva, Switzerland: World Health Organization (WHO), 2017
Slovakia Vital Registration - Deaths 2014 ICD10 as it appears in World Health Organization (WHO). WHO Mortality Database Version October 2017. Geneva, Switzerland: World Health Organization (WHO), 2017
Slovenia Vital Registration - Deaths 1989 ICD9 as it appears in World Health Organization (WHO). WHO Mortality Database Version November 2015. Geneva, Switzerland: World Health Organization (WHO), 2015
Slovenia Vital Registration - Deaths 1991 ICD9 as it appears in World Health Organization (WHO). WHO Mortality Database Version November 2015. Geneva, Switzerland: World Health Organization (WHO), 2015
Slovenia Vital Registration - Deaths 1992 ICD9 as it appears in World Health Organization (WHO). WHO Mortality Database Version November 2015. Geneva, Switzerland: World Health Organization (WHO), 2015
Slovenia Vital Registration - Deaths 1993 ICD9 as it appears in World Health Organization (WHO). WHO Mortality Database Version November 2015. Geneva, Switzerland: World Health Organization (WHO), 2015
Slovenia Vital Registration - Deaths 1994 ICD9 as it appears in World Health Organization (WHO). WHO Mortality Database Version November 2015. Geneva, Switzerland: World Health Organization (WHO), 2015
Slovenia Vital Registration - Deaths 1995 ICD9 as it appears in World Health Organization (WHO). WHO Mortality Database Version November 2015. Geneva, Switzerland: World Health Organization (WHO), 2015

Slovenia Vital Registration - Deaths 1996 ICD9 as it appears in World Health Organization (WHO). WHO Mortality Database Version November 2015. Geneva, Switzerland: World Health Organization (WHO), 2015
Slovenia Vital Registration - Deaths 1997 ICD10 as it appears in World Health Organization (WHO). WHO Mortality Database Version October 2017. Geneva, Switzerland: World Health Organization (WHO), 2017
Slovenia Vital Registration - Deaths 1998 ICD10 as it appears in World Health Organization (WHO). WHO Mortality Database Version October 2017. Geneva, Switzerland: World Health Organization (WHO), 2017
Slovenia Vital Registration - Deaths 1999 ICD10 as it appears in World Health Organization (WHO). WHO Mortality Database Version October 2017. Geneva, Switzerland: World Health Organization (WHO), 2017
Slovenia Vital Registration - Deaths 2000 ICD10 as it appears in World Health Organization (WHO). WHO Mortality Database Version October 2017. Geneva, Switzerland: World Health Organization (WHO), 2017
Slovenia Vital Registration - Deaths 2001 ICD10 as it appears in World Health Organization (WHO). WHO Mortality Database Version October 2017. Geneva, Switzerland: World Health Organization (WHO), 2017
Slovenia Vital Registration - Deaths 2002 ICD10 as it appears in World Health Organization (WHO). WHO Mortality Database Version October 2017. Geneva, Switzerland: World Health Organization (WHO), 2017
Slovenia Vital Registration - Deaths 2003 ICD10 as it appears in World Health Organization (WHO). WHO Mortality Database Version October 2017. Geneva, Switzerland: World Health Organization (WHO), 2017
Slovenia Vital Registration - Deaths 2004 ICD10 as it appears in World Health Organization (WHO). WHO Mortality Database Version October 2017. Geneva, Switzerland: World Health Organization (WHO), 2017
Slovenia Vital Registration - Deaths 2005 ICD10 as it appears in World Health Organization (WHO). WHO Mortality Database Version October 2017. Geneva, Switzerland: World Health Organization (WHO), 2017
Slovenia Vital Registration - Deaths 2006 ICD10 as it appears in World Health Organization (WHO). WHO Mortality Database Version October 2017. Geneva, Switzerland: World Health Organization (WHO), 2017
Slovenia Vital Registration - Deaths 2007 ICD10 as it appears in World Health Organization (WHO). WHO Mortality Database Version October 2017. Geneva, Switzerland: World Health Organization (WHO), 2017
Slovenia Vital Registration - Deaths 2008 ICD10 as it appears in World Health Organization (WHO). WHO Mortality Database Version October 2017. Geneva, Switzerland: World Health Organization (WHO), 2017
Slovenia Vital Registration - Deaths 2009 ICD10 as it appears in World Health Organization (WHO). WHO Mortality Database Version October 2017. Geneva, Switzerland: World Health Organization (WHO), 2017
Slovenia Vital Registration - Deaths 2010 ICD10 as it appears in World Health Organization (WHO). WHO Mortality Database Version October 2017. Geneva, Switzerland: World Health Organization (WHO), 2017
Slovenia Vital Registration - Deaths 2011 ICD10 as it appears in World Health Organization (WHO). WHO Mortality Database Version March 2017. Geneva, Switzerland: World Health Organization (WHO), 2017
Slovenia Vital Registration - Deaths 2012 ICD10 as it appears in World Health Organization (WHO). WHO Mortality Database Version March 2017. Geneva, Switzerland: World Health Organization (WHO), 2017
Slovenia Vital Registration - Deaths 2013 ICD10 as it appears in World Health Organization (WHO). WHO Mortality Database Version March 2017. Geneva, Switzerland: World Health Organization (WHO), 2017
Slovenia Vital Registration - Deaths 2014 ICD10 as it appears in World Health Organization (WHO). WHO Mortality Database Version March 2017. Geneva, Switzerland: World Health Organization (WHO), 2017
Slovenia Vital Registration - Deaths 2015 ICD10 as it appears in World Health Organization (WHO). WHO Mortality Database Version March 2017. Geneva, Switzerland: World Health Organization (WHO), 2017

South Korea Vital Registration - Deaths 1986 ICD9 as it appears in World Health Organization (WHO). WHO Mortality Database Version September 2016. Geneva, Switzerland: World Health Organization (WHO), 2016
South Korea Vital Registration - Deaths 1987 ICD9 as it appears in World Health Organization (WHO). WHO Mortality Database Version September 2016. Geneva, Switzerland: World Health Organization (WHO), 2016
South Korea Vital Registration - Deaths 1988 ICD9 as it appears in World Health Organization (WHO). WHO Mortality Database Version September 2016. Geneva, Switzerland: World Health Organization (WHO), 2016
South Korea Vital Registration - Deaths 1989 ICD9 as it appears in World Health Organization (WHO). WHO Mortality Database Version September 2016. Geneva, Switzerland: World Health Organization (WHO), 2016
South Korea Vital Registration - Deaths 1990 ICD9 as it appears in World Health Organization (WHO). WHO Mortality Database Version September 2016. Geneva, Switzerland: World Health Organization (WHO), 2016
South Korea Vital Registration - Deaths 1994 ICD9 as it appears in World Health Organization (WHO). WHO Mortality Database Version September 2016. Geneva, Switzerland: World Health Organization (WHO), 2016
South Korea Vital Registration - Deaths 1995 ICD10 as it appears in World Health Organization (WHO). WHO Mortality Database Version October 2017. Geneva, Switzerland: World Health Organization (WHO), 2017
South Korea Vital Registration - Deaths 1996 ICD10 as it appears in World Health Organization (WHO). WHO Mortality Database Version October 2017. Geneva, Switzerland: World Health Organization (WHO), 2017
South Korea Vital Registration - Deaths 1997 ICD10 as it appears in World Health Organization (WHO). WHO Mortality Database Version October 2017. Geneva, Switzerland: World Health Organization (WHO), 2017
South Korea Vital Registration - Deaths 1998 ICD10 as it appears in World Health Organization (WHO). WHO Mortality Database Version October 2017. Geneva, Switzerland: World Health Organization (WHO), 2017
South Korea Vital Registration - Deaths 1999 ICD10 as it appears in World Health Organization (WHO). WHO Mortality Database Version October 2017. Geneva, Switzerland: World Health Organization (WHO), 2017
South Korea Vital Registration - Deaths 1985 ICD9 as it appears in World Health Organization (WHO). WHO Mortality Database Version September 2016. Geneva, Switzerland: World Health Organization (WHO), 2016
South Korea Vital Registration - Deaths 1991 ICD9 as it appears in World Health Organization (WHO). WHO Mortality Database Version September 2016. Geneva, Switzerland: World Health Organization (WHO), 2016
South Korea Vital Registration - Deaths 1992 ICD9 as it appears in World Health Organization (WHO). WHO Mortality Database Version September 2016. Geneva, Switzerland: World Health Organization (WHO), 2016
South Korea Vital Registration - Deaths 1993 ICD9 as it appears in World Health Organization (WHO). WHO Mortality Database Version September 2016. Geneva, Switzerland: World Health Organization (WHO), 2016
South Korea Vital Registration - Deaths 2000 ICD10 as it appears in World Health Organization (WHO). WHO Mortality Database Version October 2017. Geneva, Switzerland: World Health Organization (WHO), 2017
South Korea Vital Registration - Deaths 2001 ICD10 as it appears in World Health Organization (WHO). WHO Mortality Database Version October 2017. Geneva, Switzerland: World Health Organization (WHO), 2017
South Korea Vital Registration - Deaths 2002 ICD10 as it appears in World Health Organization (WHO). WHO Mortality Database Version October 2017. Geneva, Switzerland: World Health Organization (WHO), 2017
South Korea Vital Registration - Deaths 2003 ICD10 as it appears in World Health Organization (WHO). WHO Mortality Database Version October 2017. Geneva, Switzerland: World Health Organization (WHO), 2017
South Korea Vital Registration - Deaths 2004 ICD10 as it appears in World Health Organization (WHO). WHO Mortality Database Version October 2017. Geneva, Switzerland: World Health Organization (WHO), 2017

South Korea Vital Registration - Deaths 2005 ICD10 as it appears in World Health Organization (WHO). WHO Mortality Database Version October 2017. Geneva, Switzerland: World Health Organization (WHO), 2017
South Korea Vital Registration - Deaths 2006 ICD10 as it appears in World Health Organization (WHO). WHO Mortality Database Version October 2017. Geneva, Switzerland: World Health Organization (WHO), 2017
South Korea Vital Registration - Deaths 2007 ICD10 as it appears in World Health Organization (WHO). WHO Mortality Database Version October 2017. Geneva, Switzerland: World Health Organization (WHO), 2017
South Korea Vital Registration - Deaths 2008 ICD10 as it appears in World Health Organization (WHO). WHO Mortality Database Version October 2017. Geneva, Switzerland: World Health Organization (WHO), 2017
South Korea Vital Registration - Deaths 2009 ICD10 as it appears in World Health Organization (WHO). WHO Mortality Database Version October 2017. Geneva, Switzerland: World Health Organization (WHO), 2017
South Korea Vital Registration - Deaths 2010 ICD10 as it appears in World Health Organization (WHO). WHO Mortality Database Version October 2017. Geneva, Switzerland: World Health Organization (WHO), 2017
South Korea Vital Registration - Deaths 2011 ICD10 as it appears in World Health Organization (WHO). WHO Mortality Database Version October 2017. Geneva, Switzerland: World Health Organization (WHO), 2017
South Korea Vital Registration - Deaths 2012 ICD10 as it appears in World Health Organization (WHO). WHO Mortality Database Version October 2017. Geneva, Switzerland: World Health Organization (WHO), 2017
South Korea Vital Registration - Deaths 2013 ICD10 as it appears in World Health Organization (WHO). WHO Mortality Database Version October 2017. Geneva, Switzerland: World Health Organization (WHO), 2017
South Korea Vital Registration - Deaths 2014 ICD10 as it appears in World Health Organization (WHO). WHO Mortality Database Version October 2017. Geneva, Switzerland: World Health Organization (WHO), 2017
South Korea Vital Registration - Deaths 2015 ICD10 as it appears in World Health Organization (WHO). WHO Mortality Database Version October 2017. Geneva, Switzerland: World Health Organization (WHO), 2017
Spain Vital Registration - Deaths 1980 ICD9 as it appears in World Health Organization (WHO). WHO Mortality Database Version November 2015. Geneva, Switzerland: World Health Organization (WHO), 2015
Spain Vital Registration - Deaths 1981 ICD9 as it appears in World Health Organization (WHO). WHO Mortality Database Version November 2015. Geneva, Switzerland: World Health Organization (WHO), 2015
Spain Vital Registration - Deaths 1982 ICD9 as it appears in World Health Organization (WHO). WHO Mortality Database Version November 2015. Geneva, Switzerland: World Health Organization (WHO), 2015
Spain Vital Registration - Deaths 1983 ICD9 as it appears in World Health Organization (WHO). WHO Mortality Database Version November 2015. Geneva, Switzerland: World Health Organization (WHO), 2015
Spain Vital Registration - Deaths 1984 ICD9 as it appears in World Health Organization (WHO). WHO Mortality Database Version November 2015. Geneva, Switzerland: World Health Organization (WHO), 2015
Spain Vital Registration - Deaths 1985 ICD9 as it appears in World Health Organization (WHO). WHO Mortality Database Version November 2015. Geneva, Switzerland: World Health Organization (WHO), 2015
Spain Vital Registration - Deaths 1986 ICD9 as it appears in World Health Organization (WHO). WHO Mortality Database Version November 2015. Geneva, Switzerland: World Health Organization (WHO), 2015
Spain Vital Registration - Deaths 1987 ICD9 as it appears in World Health Organization (WHO). WHO Mortality Database Version November 2015. Geneva, Switzerland: World Health Organization (WHO), 2015
Spain Vital Registration - Deaths 1988 ICD9 as it appears in World Health Organization (WHO). WHO Mortality Database Version November 2015. Geneva, Switzerland: World Health Organization (WHO), 2015
Spain Vital Registration - Deaths 1989 ICD9 as it appears in World Health Organization (WHO). WHO Mortality Database Version November 2015. Geneva, Switzerland:

World Health Organization (WHO), 2015
Spain Vital Registration - Deaths 1990 ICD9 as it appears in World Health Organization (WHO). WHO Mortality Database Version November 2015. Geneva, Switzerland: World Health Organization (WHO), 2015
Spain Vital Registration - Deaths 1991 ICD9 as it appears in World Health Organization (WHO). WHO Mortality Database Version November 2015. Geneva, Switzerland: World Health Organization (WHO), 2015
Spain Vital Registration - Deaths 1992 ICD9 as it appears in World Health Organization (WHO). WHO Mortality Database Version November 2015. Geneva, Switzerland: World Health Organization (WHO), 2015
Spain Vital Registration - Deaths 1993 ICD9 as it appears in World Health Organization (WHO). WHO Mortality Database Version November 2015. Geneva, Switzerland: World Health Organization (WHO), 2015
Spain Vital Registration - Deaths 1994 ICD9 as it appears in World Health Organization (WHO). WHO Mortality Database Version November 2015. Geneva, Switzerland: World Health Organization (WHO), 2015
Spain Vital Registration - Deaths 1995 ICD9 as it appears in World Health Organization (WHO). WHO Mortality Database Version November 2015. Geneva, Switzerland: World Health Organization (WHO), 2015
Spain Vital Registration - Deaths 1996 ICD9 as it appears in World Health Organization (WHO). WHO Mortality Database Version November 2015. Geneva, Switzerland: World Health Organization (WHO), 2015
Spain Vital Registration - Deaths 1997 ICD9 as it appears in World Health Organization (WHO). WHO Mortality Database Version November 2015. Geneva, Switzerland: World Health Organization (WHO), 2015
Spain Vital Registration - Deaths 1998 ICD9 as it appears in World Health Organization (WHO). WHO Mortality Database Version November 2015. Geneva, Switzerland: World Health Organization (WHO), 2015
Spain Vital Registration - Deaths 1999 ICD10 as it appears in World Health Organization (WHO). WHO Mortality Database Version October 2017. Geneva, Switzerland: World Health Organization (WHO), 2017
Spain Vital Registration - Deaths 2000 ICD10 as it appears in World Health Organization (WHO). WHO Mortality Database Version October 2017. Geneva, Switzerland: World Health Organization (WHO), 2017
Spain Vital Registration - Deaths 2001 ICD10 as it appears in World Health Organization (WHO). WHO Mortality Database Version October 2017. Geneva, Switzerland: World Health Organization (WHO), 2017
Spain Vital Registration - Deaths 2002 ICD10 as it appears in World Health Organization (WHO). WHO Mortality Database Version October 2017. Geneva, Switzerland: World Health Organization (WHO), 2017
Spain Vital Registration - Deaths 2003 ICD10 as it appears in World Health Organization (WHO). WHO Mortality Database Version October 2017. Geneva, Switzerland: World Health Organization (WHO), 2017
Spain Vital Registration - Deaths 2004 ICD10 as it appears in World Health Organization (WHO). WHO Mortality Database Version October 2017. Geneva, Switzerland: World Health Organization (WHO), 2017
Spain Vital Registration - Deaths 2005 ICD10 as it appears in World Health Organization (WHO). WHO Mortality Database Version October 2017. Geneva, Switzerland: World Health Organization (WHO), 2017
Spain Vital Registration - Deaths 2006 ICD10 as it appears in World Health Organization (WHO). WHO Mortality Database Version October 2017. Geneva, Switzerland: World Health Organization (WHO), 2017
Spain Vital Registration - Deaths 2007 ICD10 as it appears in World Health Organization (WHO). WHO Mortality Database Version October 2017. Geneva, Switzerland: World Health Organization (WHO), 2017
Spain Vital Registration - Deaths 2008 ICD10 as it appears in World Health Organization (WHO). WHO Mortality Database Version October 2017. Geneva, Switzerland: World Health Organization (WHO), 2017
Spain Vital Registration - Deaths 2009 ICD10 as it appears in World Health Organization (WHO). WHO Mortality Database Version October 2017. Geneva, Switzerland: World Health Organization (WHO), 2017

Spain Vital Registration - Deaths 2010 ICD10 as it appears in World Health Organization (WHO). WHO Mortality Database Version October 2017. Geneva, Switzerland: World Health Organization (WHO), 2017

Spain Vital Registration - Deaths 2011 ICD10 as it appears in World Health Organization (WHO). WHO Mortality Database Version October 2017. Geneva, Switzerland: World Health Organization (WHO), 2017

Spain Vital Registration - Deaths 2012 ICD10 as it appears in World Health Organization (WHO). WHO Mortality Database Version October 2017. Geneva, Switzerland: World Health Organization (WHO), 2017

Spain Vital Registration - Deaths 2013 ICD10 as it appears in World Health Organization (WHO). WHO Mortality Database Version October 2017. Geneva, Switzerland: World Health Organization (WHO), 2017

Spain Vital Registration - Deaths 2014 ICD10 as it appears in World Health Organization (WHO). WHO Mortality Database Version October 2017. Geneva, Switzerland: World Health Organization (WHO), 2017

Spain Vital Registration - Deaths 2015 ICD10 as it appears in World Health Organization (WHO). WHO Mortality Database Version October 2017. Geneva, Switzerland: World Health Organization (WHO), 2017

State Statistics Service (Ukraine). Ukraine Vital Registration - Deaths 2013

State Statistics Service (Ukraine). Ukraine Vital Registration - Deaths 2015

State Statistics Service (Ukraine). Ukraine Vital Registration - Deaths 2016

Statistics Portugal. Portugal Vital Registration - Deaths 1980

Statistics Portugal. Portugal Vital Registration - Deaths 1981

Statistics Portugal. Portugal Vital Registration - Deaths 1982

Statistics Portugal. Portugal Vital Registration - Deaths 1983

Statistics Portugal. Portugal Vital Registration - Deaths 2004

Suriname Vital Registration - Deaths 1980 ICD9 as it appears in World Health Organization (WHO). WHO Mortality Database Version September 2016. Geneva, Switzerland: World Health Organization (WHO), 2016

Suriname Vital Registration - Deaths 1981 ICD9 as it appears in World Health Organization (WHO). WHO Mortality Database Version September 2016. Geneva, Switzerland: World Health Organization (WHO), 2016

Suriname Vital Registration - Deaths 1984 ICD9 as it appears in World Health Organization (WHO). WHO Mortality Database Version September 2016. Geneva, Switzerland: World Health Organization (WHO), 2016

Suriname Vital Registration - Deaths 1986 ICD9 as it appears in World Health Organization (WHO). WHO Mortality Database Version September 2016. Geneva, Switzerland: World Health Organization (WHO), 2016

Suriname Vital Registration - Deaths 1987 ICD9 as it appears in World Health Organization (WHO). WHO Mortality Database Version September 2016. Geneva, Switzerland: World Health Organization (WHO), 2016

Suriname Vital Registration - Deaths 1988 ICD9 as it appears in World Health Organization (WHO). WHO Mortality Database Version September 2016. Geneva, Switzerland: World Health Organization (WHO), 2016

Suriname Vital Registration - Deaths 1989 ICD9 as it appears in World Health Organization (WHO). WHO Mortality Database Version September 2016. Geneva, Switzerland: World Health Organization (WHO), 2016

Suriname Vital Registration - Deaths 1990 ICD9 as it appears in World Health Organization (WHO). WHO Mortality Database Version September 2016. Geneva, Switzerland: World Health Organization (WHO), 2016

Suriname Vital Registration - Deaths 1991 ICD9 as it appears in World Health Organization (WHO). WHO Mortality Database Version September 2016. Geneva, Switzerland: World Health Organization (WHO), 2016

Suriname Vital Registration - Deaths 1992 ICD9 as it appears in World Health Organization (WHO). WHO Mortality Database Version September 2016. Geneva, Switzerland: World Health Organization (WHO), 2016

Suriname Vital Registration - Deaths 1995 ICD10 as it appears in World Health Organization (WHO). WHO Mortality Database Version October 2017. Geneva, Switzerland: World Health Organization (WHO), 2017
Suriname Vital Registration - Deaths 1996 ICD10 as it appears in World Health Organization (WHO). WHO Mortality Database Version October 2017. Geneva, Switzerland: World Health Organization (WHO), 2017
Suriname Vital Registration - Deaths 1997 ICD10 as it appears in World Health Organization (WHO). WHO Mortality Database Version October 2017. Geneva, Switzerland: World Health Organization (WHO), 2017
Suriname Vital Registration - Deaths 1998 ICD10 as it appears in World Health Organization (WHO). WHO Mortality Database Version October 2017. Geneva, Switzerland: World Health Organization (WHO), 2017
Suriname Vital Registration - Deaths 1999 ICD10 as it appears in World Health Organization (WHO). WHO Mortality Database Version October 2017. Geneva, Switzerland: World Health Organization (WHO), 2017
Suriname Vital Registration - Deaths 2000 ICD10 as it appears in World Health Organization (WHO). WHO Mortality Database Version October 2017. Geneva, Switzerland: World Health Organization (WHO), 2017
Suriname Vital Registration - Deaths 2004 ICD10 as it appears in World Health Organization (WHO). WHO Mortality Database Version October 2017. Geneva, Switzerland: World Health Organization (WHO), 2017
Suriname Vital Registration - Deaths 2005 ICD10 as it appears in World Health Organization (WHO). WHO Mortality Database Version October 2017. Geneva, Switzerland: World Health Organization (WHO), 2017
Suriname Vital Registration - Deaths 1982 ICD9 as it appears in World Health Organization (WHO). WHO Mortality Database Version September 2016. Geneva, Switzerland: World Health Organization (WHO), 2016
Suriname Vital Registration - Deaths 1985 ICD9 as it appears in World Health Organization (WHO). WHO Mortality Database Version September 2016. Geneva, Switzerland: World Health Organization (WHO), 2016
Suriname Vital Registration - Deaths 2001 ICD10 as it appears in World Health Organization (WHO). WHO Mortality Database Version October 2017. Geneva, Switzerland: World Health Organization (WHO), 2017
Suriname Vital Registration - Deaths 2002 ICD10 as it appears in World Health Organization (WHO). WHO Mortality Database Version October 2017. Geneva, Switzerland: World Health Organization (WHO), 2017
Suriname Vital Registration - Deaths 2003 ICD10 as it appears in World Health Organization (WHO). WHO Mortality Database Version October 2017. Geneva, Switzerland: World Health Organization (WHO), 2017
Suriname Vital Registration - Deaths 2006 ICD10 as it appears in World Health Organization (WHO). WHO Mortality Database Version October 2017. Geneva, Switzerland: World Health Organization (WHO), 2017
Suriname Vital Registration - Deaths 2008 ICD10 as it appears in World Health Organization (WHO). WHO Mortality Database Version October 2017. Geneva, Switzerland: World Health Organization (WHO), 2017
Suriname Vital Registration - Deaths 2009 ICD10 as it appears in World Health Organization (WHO). WHO Mortality Database Version October 2017. Geneva, Switzerland: World Health Organization (WHO), 2017
Suriname Vital Registration - Deaths 2010 ICD10 as it appears in World Health Organization (WHO). WHO Mortality Database Version October 2017. Geneva, Switzerland: World Health Organization (WHO), 2017
Suriname Vital Registration - Deaths 2011 ICD10 as it appears in World Health Organization (WHO). WHO Mortality Database Version October 2017. Geneva, Switzerland: World Health Organization (WHO), 2017
Suriname Vital Registration - Deaths 2012 ICD10 as it appears in World Health Organization (WHO). WHO Mortality Database Version October 2017. Geneva, Switzerland: World Health Organization (WHO), 2017
Suriname Vital Registration - Deaths 2013 ICD10 as it appears in World Health Organization (WHO). WHO Mortality Database Version October 2017. Geneva, Switzerland: World Health Organization (WHO), 2017

Suriname Vital Registration - Deaths 2014 ICD10 as it appears in World Health Organization (WHO). WHO Mortality Database Version October 2017. Geneva, Switzerland: World Health Organization (WHO), 2017

Switzerland Vital Registration - Deaths 1980 ICD8 as it appears in World Health Organization (WHO). WHO Mortality Database Version July 2012. Geneva, Switzerland: World Health Organization (WHO), 2012

Switzerland Vital Registration - Deaths 1981 ICD8 as it appears in World Health Organization (WHO). WHO Mortality Database Version July 2012. Geneva, Switzerland: World Health Organization (WHO), 2012

Switzerland Vital Registration - Deaths 1982 ICD8 as it appears in World Health Organization (WHO). WHO Mortality Database Version July 2012. Geneva, Switzerland: World Health Organization (WHO), 2012

Switzerland Vital Registration - Deaths 1983 ICD8 as it appears in World Health Organization (WHO). WHO Mortality Database Version July 2012. Geneva, Switzerland: World Health Organization (WHO), 2012

Switzerland Vital Registration - Deaths 1984 ICD8 as it appears in World Health Organization (WHO). WHO Mortality Database Version July 2012. Geneva, Switzerland: World Health Organization (WHO), 2012

Switzerland Vital Registration - Deaths 1985 ICD8 as it appears in World Health Organization (WHO). WHO Mortality Database Version July 2012. Geneva, Switzerland: World Health Organization (WHO), 2012

Switzerland Vital Registration - Deaths 1986 ICD8 as it appears in World Health Organization (WHO). WHO Mortality Database Version July 2012. Geneva, Switzerland: World Health Organization (WHO), 2012

Switzerland Vital Registration - Deaths 1987 ICD8 as it appears in World Health Organization (WHO). WHO Mortality Database Version July 2012. Geneva, Switzerland: World Health Organization (WHO), 2012

Switzerland Vital Registration - Deaths 1988 ICD8 as it appears in World Health Organization (WHO). WHO Mortality Database Version July 2012. Geneva, Switzerland: World Health Organization (WHO), 2012

Switzerland Vital Registration - Deaths 1989 ICD8 as it appears in World Health Organization (WHO). WHO Mortality Database Version July 2012. Geneva, Switzerland: World Health Organization (WHO), 2012

Switzerland Vital Registration - Deaths 1990 ICD8 as it appears in World Health Organization (WHO). WHO Mortality Database Version July 2012. Geneva, Switzerland: World Health Organization (WHO), 2012

Switzerland Vital Registration - Deaths 1991 ICD8 as it appears in World Health Organization (WHO). WHO Mortality Database Version July 2012. Geneva, Switzerland: World Health Organization (WHO), 2012

Switzerland Vital Registration - Deaths 1992 ICD8 as it appears in World Health Organization (WHO). WHO Mortality Database Version July 2012. Geneva, Switzerland: World Health Organization (WHO), 2012

Switzerland Vital Registration - Deaths 1993 ICD8 as it appears in World Health Organization (WHO). WHO Mortality Database Version July 2012. Geneva, Switzerland: World Health Organization (WHO), 2012

Switzerland Vital Registration - Deaths 1994 ICD8 as it appears in World Health Organization (WHO). WHO Mortality Database Version July 2012. Geneva, Switzerland: World Health Organization (WHO), 2012

Switzerland Vital Registration - Deaths 1995 ICD10 as it appears in World Health Organization (WHO). WHO Mortality Database Version October 2017. Geneva, Switzerland: World Health Organization (WHO), 2017

Switzerland Vital Registration - Deaths 1996 ICD10 as it appears in World Health Organization (WHO). WHO Mortality Database Version October 2017. Geneva, Switzerland: World Health Organization (WHO), 2017

Switzerland Vital Registration - Deaths 1997 ICD10 as it appears in World Health Organization (WHO). WHO Mortality Database Version October 2017. Geneva, Switzerland: World Health Organization (WHO), 2017

Switzerland Vital Registration - Deaths 1998 ICD10 as it appears in World Health Organization (WHO). WHO Mortality Database Version October 2017. Geneva, Switzerland: World Health Organization (WHO), 2017

Switzerland Vital Registration - Deaths 1999 ICD10 as it appears in World Health Organization (WHO). WHO Mortality Database Version October 2017. Geneva, Switzerland: World Health Organization (WHO), 2017
Switzerland Vital Registration - Deaths 2000 ICD10 as it appears in World Health Organization (WHO). WHO Mortality Database Version October 2017. Geneva, Switzerland: World Health Organization (WHO), 2017
Switzerland Vital Registration - Deaths 2001 ICD10 as it appears in World Health Organization (WHO). WHO Mortality Database Version October 2017. Geneva, Switzerland: World Health Organization (WHO), 2017
Switzerland Vital Registration - Deaths 2002 ICD10 as it appears in World Health Organization (WHO). WHO Mortality Database Version October 2017. Geneva, Switzerland: World Health Organization (WHO), 2017
Switzerland Vital Registration - Deaths 2003 ICD10 as it appears in World Health Organization (WHO). WHO Mortality Database Version October 2017. Geneva, Switzerland: World Health Organization (WHO), 2017
Switzerland Vital Registration - Deaths 2004 ICD10 as it appears in World Health Organization (WHO). WHO Mortality Database Version October 2017. Geneva, Switzerland: World Health Organization (WHO), 2017
Switzerland Vital Registration - Deaths 2005 ICD10 as it appears in World Health Organization (WHO). WHO Mortality Database Version October 2017. Geneva, Switzerland: World Health Organization (WHO), 2017
Switzerland Vital Registration - Deaths 2006 ICD10 as it appears in World Health Organization (WHO). WHO Mortality Database Version October 2017. Geneva, Switzerland: World Health Organization (WHO), 2017
Switzerland Vital Registration - Deaths 2007 ICD10 as it appears in World Health Organization (WHO). WHO Mortality Database Version October 2017. Geneva, Switzerland: World Health Organization (WHO), 2017
Switzerland Vital Registration - Deaths 2008 ICD10 as it appears in World Health Organization (WHO). WHO Mortality Database Version October 2017. Geneva, Switzerland: World Health Organization (WHO), 2017
Switzerland Vital Registration - Deaths 2009 ICD10 as it appears in World Health Organization (WHO). WHO Mortality Database Version October 2017. Geneva, Switzerland: World Health Organization (WHO), 2017
Switzerland Vital Registration - Deaths 2010 ICD10 as it appears in World Health Organization (WHO). WHO Mortality Database Version October 2017. Geneva, Switzerland: World Health Organization (WHO), 2017
Switzerland Vital Registration - Deaths 2011 ICD10 as it appears in World Health Organization (WHO). WHO Mortality Database Version October 2017. Geneva, Switzerland: World Health Organization (WHO), 2017
Switzerland Vital Registration - Deaths 2012 ICD10 as it appears in World Health Organization (WHO). WHO Mortality Database Version October 2017. Geneva, Switzerland: World Health Organization (WHO), 2017
Switzerland Vital Registration - Deaths 2013 ICD10 as it appears in World Health Organization (WHO). WHO Mortality Database Version October 2017. Geneva, Switzerland: World Health Organization (WHO), 2017
Switzerland Vital Registration - Deaths 2014 ICD10 as it appears in World Health Organization (WHO). WHO Mortality Database Version October 2017. Geneva, Switzerland: World Health Organization (WHO), 2017
Switzerland Vital Registration - Deaths 2015 ICD10 as it appears in World Health Organization (WHO). WHO Mortality Database Version April 2018. Geneva, Switzerland: World Health Organization (WHO), 2018
Tajikistan Vital Registration - Deaths 1981 ICD9 as it appears in World Health Organization (WHO). WHO Mortality Database Version September 2016. Geneva, Switzerland: World Health Organization (WHO), 2016
Tajikistan Vital Registration - Deaths 1982 ICD9 as it appears in World Health Organization (WHO). WHO Mortality Database Version September 2016. Geneva, Switzerland: World Health Organization (WHO), 2016
Tajikistan Vital Registration - Deaths 1985 ICD9 as it appears in World Health Organization (WHO). WHO Mortality Database Version September 2016. Geneva, Switzerland: World Health Organization (WHO), 2016

Tajikistan Vital Registration - Deaths 1986 ICD9 as it appears in World Health Organization (WHO). WHO Mortality Database Version September 2016. Geneva, Switzerland: World Health Organization (WHO), 2016
Tajikistan Vital Registration - Deaths 1987 ICD9 as it appears in World Health Organization (WHO). WHO Mortality Database Version September 2016. Geneva, Switzerland: World Health Organization (WHO), 2016
Tajikistan Vital Registration - Deaths 1988 ICD9 as it appears in World Health Organization (WHO). WHO Mortality Database Version September 2016. Geneva, Switzerland: World Health Organization (WHO), 2016
Tajikistan Vital Registration - Deaths 1989 ICD9 as it appears in World Health Organization (WHO). WHO Mortality Database Version September 2016. Geneva, Switzerland: World Health Organization (WHO), 2016
Tajikistan Vital Registration - Deaths 1990 ICD9 as it appears in World Health Organization (WHO). WHO Mortality Database Version September 2016. Geneva, Switzerland: World Health Organization (WHO), 2016
Tajikistan Vital Registration - Deaths 1991 ICD9 as it appears in World Health Organization (WHO). WHO Mortality Database Version September 2016. Geneva, Switzerland: World Health Organization (WHO), 2016
Tajikistan Vital Registration - Deaths 1992 ICD9 as it appears in World Health Organization (WHO). WHO Mortality Database Version September 2016. Geneva, Switzerland: World Health Organization (WHO), 2016
Tajikistan Vital Registration - Deaths 1993 ICD9 as it appears in World Health Organization (WHO). WHO Mortality Database Version September 2016. Geneva, Switzerland: World Health Organization (WHO), 2016
Tajikistan Vital Registration - Deaths 1994 ICD9 as it appears in World Health Organization (WHO). WHO Mortality Database Version September 2016. Geneva, Switzerland: World Health Organization (WHO), 2016
Tajikistan Vital Registration - Deaths 1995 ICD9 as it appears in World Health Organization (WHO). WHO Mortality Database Version September 2016. Geneva, Switzerland: World Health Organization (WHO), 2016
Tajikistan Vital Registration - Deaths 1996 ICD9 as it appears in World Health Organization (WHO). WHO Mortality Database Version September 2016. Geneva, Switzerland: World Health Organization (WHO), 2016
Tajikistan Vital Registration - Deaths 1997 ICD9 as it appears in World Health Organization (WHO). WHO Mortality Database Version September 2016. Geneva, Switzerland: World Health Organization (WHO), 2016
Tajikistan Vital Registration - Deaths 1998 ICD9 as it appears in World Health Organization (WHO). WHO Mortality Database Version September 2016. Geneva, Switzerland: World Health Organization (WHO), 2016
Tajikistan Vital Registration - Deaths 1999 ICD9 as it appears in World Health Organization (WHO). WHO Mortality Database Version September 2016. Geneva, Switzerland: World Health Organization (WHO), 2016
Tajikistan Vital Registration - Deaths 2000 ICD9 as it appears in World Health Organization (WHO). WHO Mortality Database Version September 2016. Geneva, Switzerland: World Health Organization (WHO), 2016
Tajikistan Vital Registration - Deaths 2001 ICD9 as it appears in World Health Organization (WHO). WHO Mortality Database Version September 2016. Geneva, Switzerland: World Health Organization (WHO), 2016
Tajikistan Vital Registration - Deaths 2002 ICD9 as it appears in World Health Organization (WHO). WHO Mortality Database Version September 2016. Geneva, Switzerland: World Health Organization (WHO), 2016
Tajikistan Vital Registration - Deaths 2003 ICD9 as it appears in World Health Organization (WHO). WHO Mortality Database Version September 2016. Geneva, Switzerland: World Health Organization (WHO), 2016
Tajikistan Vital Registration - Deaths 2004 ICD9 as it appears in World Health Organization (WHO). WHO Mortality Database Version September 2016. Geneva, Switzerland: World Health Organization (WHO), 2016
Tajikistan Vital Registration - Deaths 2005 ICD9 as it appears in World Health Organization (WHO). WHO Mortality Database Version September 2016. Geneva, Switzerland: World Health Organization (WHO), 2016
Tajikistan Vital Registration - Deaths 2016 ICD10 as it appears in World Health Organization (WHO). WHO Mortality Database Version April 2018. Geneva, Switzerland:

World Health Organization (WHO), 2018
Thailand Vital Registration - Deaths 2003 ICD10 as it appears in World Health Organization (WHO). WHO Mortality Database Version October 2017. Geneva, Switzerland: World Health Organization (WHO), 2017
Thailand Vital Registration - Deaths 2011 ICD10 as it appears in World Health Organization (WHO). WHO Mortality Database Version October 2017. Geneva, Switzerland: World Health Organization (WHO), 2017
Thailand Vital Registration - Deaths 2012 ICD10 as it appears in World Health Organization (WHO). WHO Mortality Database Version October 2017. Geneva, Switzerland: World Health Organization (WHO), 2017
Thailand Vital Registration - Deaths 2013 ICD10 as it appears in World Health Organization (WHO). WHO Mortality Database Version October 2017. Geneva, Switzerland: World Health Organization (WHO), 2017
Thailand Vital Registration - Deaths 2014 ICD10 as it appears in World Health Organization (WHO). WHO Mortality Database Version October 2017. Geneva, Switzerland: World Health Organization (WHO), 2017
Thailand Vital Registration - Deaths 2015 ICD10 as it appears in World Health Organization (WHO). WHO Mortality Database Version October 2017. Geneva, Switzerland: World Health Organization (WHO), 2017
Thailand Vital Registration - Deaths 2016 ICD10 as it appears in World Health Organization (WHO). WHO Mortality Database Version April 2018. Geneva, Switzerland: World Health Organization (WHO), 2018
Trinidad and Tobago Vital Registration - Deaths 1980 ICD9 as it appears in World Health Organization (WHO). WHO Mortality Database Version September 2016. Geneva, Switzerland: World Health Organization (WHO), 2016
Trinidad and Tobago Vital Registration - Deaths 1981 ICD9 as it appears in World Health Organization (WHO). WHO Mortality Database Version September 2016. Geneva, Switzerland: World Health Organization (WHO), 2016
Trinidad and Tobago Vital Registration - Deaths 1982 ICD9 as it appears in World Health Organization (WHO). WHO Mortality Database Version September 2016. Geneva, Switzerland: World Health Organization (WHO), 2016
Trinidad and Tobago Vital Registration - Deaths 1983 ICD9 as it appears in World Health Organization (WHO). WHO Mortality Database Version September 2016. Geneva, Switzerland: World Health Organization (WHO), 2016
Trinidad and Tobago Vital Registration - Deaths 1984 ICD9 as it appears in World Health Organization (WHO). WHO Mortality Database Version September 2016. Geneva, Switzerland: World Health Organization (WHO), 2016
Trinidad and Tobago Vital Registration - Deaths 1985 ICD9 as it appears in World Health Organization (WHO). WHO Mortality Database Version September 2016. Geneva, Switzerland: World Health Organization (WHO), 2016
Trinidad and Tobago Vital Registration - Deaths 1986 ICD9 as it appears in World Health Organization (WHO). WHO Mortality Database Version September 2016. Geneva, Switzerland: World Health Organization (WHO), 2016
Trinidad and Tobago Vital Registration - Deaths 1987 ICD9 as it appears in World Health Organization (WHO). WHO Mortality Database Version September 2016. Geneva, Switzerland: World Health Organization (WHO), 2016
Trinidad and Tobago Vital Registration - Deaths 1988 ICD9 as it appears in World Health Organization (WHO). WHO Mortality Database Version September 2016. Geneva, Switzerland: World Health Organization (WHO), 2016
Trinidad and Tobago Vital Registration - Deaths 1989 ICD9 as it appears in World Health Organization (WHO). WHO Mortality Database Version September 2016. Geneva, Switzerland: World Health Organization (WHO), 2016
Trinidad and Tobago Vital Registration - Deaths 1990 ICD9 as it appears in World Health Organization (WHO). WHO Mortality Database Version September 2016. Geneva, Switzerland: World Health Organization (WHO), 2016
Trinidad and Tobago Vital Registration - Deaths 1991 ICD9 as it appears in World Health Organization (WHO). WHO Mortality Database Version September 2016. Geneva, Switzerland: World Health Organization (WHO), 2016
Trinidad and Tobago Vital Registration - Deaths 1992 ICD9 as it appears in World Health Organization (WHO). WHO Mortality Database Version September 2016. Geneva, Switzerland: World Health Organization (WHO), 2016

Trinidad and Tobago Vital Registration - Deaths 1993 ICD9 as it appears in World Health Organization (WHO). WHO Mortality Database Version September 2016. Geneva, Switzerland: World Health Organization (WHO), 2016
Trinidad and Tobago Vital Registration - Deaths 1994 ICD9 as it appears in World Health Organization (WHO). WHO Mortality Database Version September 2016. Geneva, Switzerland: World Health Organization (WHO), 2016
Trinidad and Tobago Vital Registration - Deaths 1995 ICD9 as it appears in World Health Organization (WHO). WHO Mortality Database Version September 2016. Geneva, Switzerland: World Health Organization (WHO), 2016
Trinidad and Tobago Vital Registration - Deaths 1996 ICD9 as it appears in World Health Organization (WHO). WHO Mortality Database Version September 2016. Geneva, Switzerland: World Health Organization (WHO), 2016
Trinidad and Tobago Vital Registration - Deaths 1997 ICD9 as it appears in World Health Organization (WHO). WHO Mortality Database Version September 2016. Geneva, Switzerland: World Health Organization (WHO), 2016
Trinidad and Tobago Vital Registration - Deaths 1998 ICD9 as it appears in World Health Organization (WHO). WHO Mortality Database Version September 2016. Geneva, Switzerland: World Health Organization (WHO), 2016
Trinidad and Tobago Vital Registration - Deaths 1999 ICD10 as it appears in World Health Organization (WHO). WHO Mortality Database Version October 2017. Geneva, Switzerland: World Health Organization (WHO), 2017
Trinidad and Tobago Vital Registration - Deaths 2000 ICD10 as it appears in World Health Organization (WHO). WHO Mortality Database Version October 2017. Geneva, Switzerland: World Health Organization (WHO), 2017
Trinidad and Tobago Vital Registration - Deaths 2001 ICD10 as it appears in World Health Organization (WHO). WHO Mortality Database Version October 2017. Geneva, Switzerland: World Health Organization (WHO), 2017
Trinidad and Tobago Vital Registration - Deaths 2002 ICD10 as it appears in World Health Organization (WHO). WHO Mortality Database Version October 2017. Geneva, Switzerland: World Health Organization (WHO), 2017
Trinidad and Tobago Vital Registration - Deaths 2003 ICD10 as it appears in World Health Organization (WHO). WHO Mortality Database Version October 2017. Geneva, Switzerland: World Health Organization (WHO), 2017
Trinidad and Tobago Vital Registration - Deaths 2004 ICD10 as it appears in World Health Organization (WHO). WHO Mortality Database Version October 2017. Geneva, Switzerland: World Health Organization (WHO), 2017
Trinidad and Tobago Vital Registration - Deaths 2005 ICD10 as it appears in World Health Organization (WHO). WHO Mortality Database Version October 2017. Geneva, Switzerland: World Health Organization (WHO), 2017
Trinidad and Tobago Vital Registration - Deaths 2006 ICD10 as it appears in World Health Organization (WHO). WHO Mortality Database Version October 2017. Geneva, Switzerland: World Health Organization (WHO), 2017
Trinidad and Tobago Vital Registration - Deaths 2007 ICD10 as it appears in World Health Organization (WHO). WHO Mortality Database Version October 2017. Geneva, Switzerland: World Health Organization (WHO), 2017
Trinidad and Tobago Vital Registration - Deaths 2008 ICD10 as it appears in World Health Organization (WHO). WHO Mortality Database Version October 2017. Geneva, Switzerland: World Health Organization (WHO), 2017
Trinidad and Tobago Vital Registration - Deaths 2009 ICD10 as it appears in World Health Organization (WHO). WHO Mortality Database Version October 2017. Geneva, Switzerland: World Health Organization (WHO), 2017
Trinidad and Tobago Vital Registration - Deaths 2010 ICD10 as it appears in World Health Organization (WHO). WHO Mortality Database Version October 2017. Geneva, Switzerland: World Health Organization (WHO), 2017
Trinidad and Tobago Vital Registration - Deaths 2011 ICD10 as it appears in World Health Organization (WHO). WHO Mortality Database Version October 2017. Geneva, Switzerland: World Health Organization (WHO), 2017
Turkey Vital Registration - Deaths 2009 ICD10 as it appears in World Health Organization (WHO). WHO Mortality Database Version October 2017. Geneva, Switzerland: World Health Organization (WHO), 2017
Turkey Vital Registration - Deaths 2010 ICD10 as it appears in World Health Organization (WHO). WHO Mortality Database Version October 2017. Geneva, Switzerland:

World Health Organization (WHO), 2017

Turkey Vital Registration - Deaths 2011 ICD10 as it appears in World Health Organization (WHO). WHO Mortality Database Version October 2017. Geneva, Switzerland: World Health Organization (WHO), 2017

Turkey Vital Registration - Deaths 2012 ICD10 as it appears in World Health Organization (WHO). WHO Mortality Database Version October 2017. Geneva, Switzerland: World Health Organization (WHO), 2017

Turkey Vital Registration - Deaths 2013 ICD10 as it appears in World Health Organization (WHO). WHO Mortality Database Version October 2017. Geneva, Switzerland: World Health Organization (WHO), 2017

Turkey Vital Registration - Deaths 2014 ICD10 as it appears in World Health Organization (WHO). WHO Mortality Database Version April 2018. Geneva, Switzerland: World Health Organization (WHO), 2018

Turkey Vital Registration - Deaths 2015 ICD10 as it appears in World Health Organization (WHO). WHO Mortality Database Version April 2018. Geneva, Switzerland: World Health Organization (WHO), 2018

Turkmenistan Vital Registration - Deaths 1981 ICD9 as it appears in World Health Organization (WHO). WHO Mortality Database Version September 2016. Geneva, Switzerland: World Health Organization (WHO), 2016

Turkmenistan Vital Registration - Deaths 1982 ICD9 as it appears in World Health Organization (WHO). WHO Mortality Database Version September 2016. Geneva, Switzerland: World Health Organization (WHO), 2016

Turkmenistan Vital Registration - Deaths 1985 ICD9 as it appears in World Health Organization (WHO). WHO Mortality Database Version September 2016. Geneva, Switzerland: World Health Organization (WHO), 2016

Turkmenistan Vital Registration - Deaths 1986 ICD9 as it appears in World Health Organization (WHO). WHO Mortality Database Version September 2016. Geneva, Switzerland: World Health Organization (WHO), 2016

Turkmenistan Vital Registration - Deaths 1987 ICD9 as it appears in World Health Organization (WHO). WHO Mortality Database Version September 2016. Geneva, Switzerland: World Health Organization (WHO), 2016

Turkmenistan Vital Registration - Deaths 1988 ICD9 as it appears in World Health Organization (WHO). WHO Mortality Database Version September 2016. Geneva, Switzerland: World Health Organization (WHO), 2016

Turkmenistan Vital Registration - Deaths 1989 ICD9 as it appears in World Health Organization (WHO). WHO Mortality Database Version September 2016. Geneva, Switzerland: World Health Organization (WHO), 2016

Turkmenistan Vital Registration - Deaths 1991 ICD9 as it appears in World Health Organization (WHO). WHO Mortality Database Version September 2016. Geneva, Switzerland: World Health Organization (WHO), 2016

Turkmenistan Vital Registration - Deaths 1992 ICD9 as it appears in World Health Organization (WHO). WHO Mortality Database Version September 2016. Geneva, Switzerland: World Health Organization (WHO), 2016

Turkmenistan Vital Registration - Deaths 1994 ICD9 as it appears in World Health Organization (WHO). WHO Mortality Database Version September 2016. Geneva, Switzerland: World Health Organization (WHO), 2016

Turkmenistan Vital Registration - Deaths 1995 ICD9 as it appears in World Health Organization (WHO). WHO Mortality Database Version September 2016. Geneva, Switzerland: World Health Organization (WHO), 2016

Turkmenistan Vital Registration - Deaths 1996 ICD9 as it appears in World Health Organization (WHO). WHO Mortality Database Version September 2016. Geneva, Switzerland: World Health Organization (WHO), 2016

Turkmenistan Vital Registration - Deaths 1997 ICD9 as it appears in World Health Organization (WHO). WHO Mortality Database Version September 2016. Geneva, Switzerland: World Health Organization (WHO), 2016

Turkmenistan Vital Registration - Deaths 1998 ICD9 as it appears in World Health Organization (WHO). WHO Mortality Database Version September 2016. Geneva, Switzerland: World Health Organization (WHO), 2016

Turkmenistan Vital Registration - Deaths 1990 ICD9 as it appears in World Health Organization (WHO). WHO Mortality Database Version September 2016. Geneva, Switzerland: World Health Organization (WHO), 2016

Turkmenistan Vital Registration - Deaths 1993 ICD9 as it appears in World Health Organization (WHO). WHO Mortality Database Version September 2016. Geneva, Switzerland: World Health Organization (WHO), 2016
Ukraine Mortality by Region, Age, Sex, and Cause of Death 2015 as it appears in Center for Demographic Research, New Economic School (Russia). Russia Mortality Rates by Region, 1-Year Age Groups, and Sex, 2015-2016. Moscow, Russia: Center for Demographic Research, New Economic School (Russia). http://demogr.nes.ru/index.php/ru/demogr_indicat/data
Ukraine Mortality by Region, Age, Sex, and Cause of Death 2016 as it appears in Center for Demographic Research, New Economic School (Russia). Russia Mortality Rates by Region, 1-Year Age Groups, and Sex, 2015-2016. Moscow, Russia: Center for Demographic Research, New Economic School (Russia). http://demogr.nes.ru/index.php/ru/demogr_indicat/data
Ukraine Vital Registration - Deaths 1981 ICD9 as it appears in World Health Organization (WHO). WHO Mortality Database Version September 2016. Geneva, Switzerland: World Health Organization (WHO), 2016
Ukraine Vital Registration - Deaths 1982 ICD9 as it appears in World Health Organization (WHO). WHO Mortality Database Version September 2016. Geneva, Switzerland: World Health Organization (WHO), 2016
Ukraine Vital Registration - Deaths 1985 ICD9 as it appears in World Health Organization (WHO). WHO Mortality Database Version September 2016. Geneva, Switzerland: World Health Organization (WHO), 2016
Ukraine Vital Registration - Deaths 1986 ICD9 as it appears in World Health Organization (WHO). WHO Mortality Database Version September 2016. Geneva, Switzerland: World Health Organization (WHO), 2016
Ukraine Vital Registration - Deaths 1987 ICD9 as it appears in World Health Organization (WHO). WHO Mortality Database Version September 2016. Geneva, Switzerland: World Health Organization (WHO), 2016
Ukraine Vital Registration - Deaths 1988 ICD9 as it appears in World Health Organization (WHO). WHO Mortality Database Version September 2016. Geneva, Switzerland: World Health Organization (WHO), 2016
Ukraine Vital Registration - Deaths 1989 ICD9 as it appears in World Health Organization (WHO). WHO Mortality Database Version September 2016. Geneva, Switzerland: World Health Organization (WHO), 2016
Ukraine Vital Registration - Deaths 1990 ICD9 as it appears in World Health Organization (WHO). WHO Mortality Database Version September 2016. Geneva, Switzerland: World Health Organization (WHO), 2016
Ukraine Vital Registration - Deaths 1991 ICD9 as it appears in World Health Organization (WHO). WHO Mortality Database Version September 2016. Geneva, Switzerland: World Health Organization (WHO), 2016
Ukraine Vital Registration - Deaths 1992 ICD9 as it appears in World Health Organization (WHO). WHO Mortality Database Version September 2016. Geneva, Switzerland: World Health Organization (WHO), 2016
Ukraine Vital Registration - Deaths 1993 ICD9 as it appears in World Health Organization (WHO). WHO Mortality Database Version September 2016. Geneva, Switzerland: World Health Organization (WHO), 2016
Ukraine Vital Registration - Deaths 1994 ICD9 as it appears in World Health Organization (WHO). WHO Mortality Database Version September 2016. Geneva, Switzerland: World Health Organization (WHO), 2016
Ukraine Vital Registration - Deaths 1995 ICD9 as it appears in World Health Organization (WHO). WHO Mortality Database Version September 2016. Geneva, Switzerland: World Health Organization (WHO), 2016
Ukraine Vital Registration - Deaths 1996 ICD9 as it appears in World Health Organization (WHO). WHO Mortality Database Version September 2016. Geneva, Switzerland: World Health Organization (WHO), 2016
Ukraine Vital Registration - Deaths 1997 ICD9 as it appears in World Health Organization (WHO). WHO Mortality Database Version September 2016. Geneva, Switzerland: World Health Organization (WHO), 2016
Ukraine Vital Registration - Deaths 1998 ICD9 as it appears in World Health Organization (WHO). WHO Mortality Database Version September 2016. Geneva, Switzerland: World Health Organization (WHO), 2016

Ukraine Vital Registration - Deaths 1999 ICD9 as it appears in World Health Organization (WHO). WHO Mortality Database Version September 2016. Geneva, Switzerland: World Health Organization (WHO), 2016
Ukraine Vital Registration - Deaths 2000 ICD9 as it appears in World Health Organization (WHO). WHO Mortality Database Version September 2016. Geneva, Switzerland: World Health Organization (WHO), 2016
Ukraine Vital Registration - Deaths 2001 ICD9 as it appears in World Health Organization (WHO). WHO Mortality Database Version September 2016. Geneva, Switzerland: World Health Organization (WHO), 2016
Ukraine Vital Registration - Deaths 2002 ICD9 as it appears in World Health Organization (WHO). WHO Mortality Database Version September 2016. Geneva, Switzerland: World Health Organization (WHO), 2016
Ukraine Vital Registration - Deaths 2003 ICD9 as it appears in World Health Organization (WHO). WHO Mortality Database Version September 2016. Geneva, Switzerland: World Health Organization (WHO), 2016
Ukraine Vital Registration - Deaths 2004 ICD9 as it appears in World Health Organization (WHO). WHO Mortality Database Version September 2016. Geneva, Switzerland: World Health Organization (WHO), 2016
United Kingdom - Northern Ireland Vital Registration - Deaths 1980 ICD9 as it appears in World Health Organization (WHO). WHO Mortality Database Version November 2015. Geneva, Switzerland: World Health Organization (WHO), 2015
United Kingdom - Northern Ireland Vital Registration - Deaths 1981 ICD9 as it appears in World Health Organization (WHO). WHO Mortality Database Version November 2015. Geneva, Switzerland: World Health Organization (WHO), 2015
United Kingdom - Northern Ireland Vital Registration - Deaths 1982 ICD9 as it appears in World Health Organization (WHO). WHO Mortality Database Version November 2015. Geneva, Switzerland: World Health Organization (WHO), 2015
United Kingdom - Northern Ireland Vital Registration - Deaths 1983 ICD9 as it appears in World Health Organization (WHO). WHO Mortality Database Version November 2015. Geneva, Switzerland: World Health Organization (WHO), 2015
United Kingdom - Northern Ireland Vital Registration - Deaths 1984 ICD9 as it appears in World Health Organization (WHO). WHO Mortality Database Version November 2015. Geneva, Switzerland: World Health Organization (WHO), 2015
United Kingdom - Northern Ireland Vital Registration - Deaths 1985 ICD9 as it appears in World Health Organization (WHO). WHO Mortality Database Version November 2015. Geneva, Switzerland: World Health Organization (WHO), 2015
United Kingdom - Northern Ireland Vital Registration - Deaths 1986 ICD9 as it appears in World Health Organization (WHO). WHO Mortality Database Version November 2015. Geneva, Switzerland: World Health Organization (WHO), 2015
United Kingdom - Northern Ireland Vital Registration - Deaths 1987 ICD9 as it appears in World Health Organization (WHO). WHO Mortality Database Version November 2015. Geneva, Switzerland: World Health Organization (WHO), 2015
United Kingdom - Northern Ireland Vital Registration - Deaths 1988 ICD9 as it appears in World Health Organization (WHO). WHO Mortality Database Version November 2015. Geneva, Switzerland: World Health Organization (WHO), 2015
United Kingdom - Northern Ireland Vital Registration - Deaths 1993 ICD9 as it appears in World Health Organization (WHO). WHO Mortality Database Version November 2015. Geneva, Switzerland: World Health Organization (WHO), 2015
United Kingdom - Northern Ireland Vital Registration - Deaths 1994 ICD9 as it appears in World Health Organization (WHO). WHO Mortality Database Version November 2015. Geneva, Switzerland: World Health Organization (WHO), 2015
United Kingdom - Northern Ireland Vital Registration - Deaths 1995 ICD9 as it appears in World Health Organization (WHO). WHO Mortality Database Version November 2015. Geneva, Switzerland: World Health Organization (WHO), 2015
United Kingdom - Northern Ireland Vital Registration - Deaths 1996 ICD9 as it appears in World Health Organization (WHO). WHO Mortality Database Version November 2015. Geneva, Switzerland: World Health Organization (WHO), 2015
United Kingdom - Northern Ireland Vital Registration - Deaths 1997 ICD9 as it appears in World Health Organization (WHO). WHO Mortality Database Version November 2015. Geneva, Switzerland: World Health Organization (WHO), 2015

United Kingdom - Northern Ireland Vital Registration - Deaths 1998 ICD9 as it appears in World Health Organization (WHO). WHO Mortality Database Version November 2015. Geneva, Switzerland: World Health Organization (WHO), 2015
United Kingdom - Northern Ireland Vital Registration - Deaths 1999 ICD9 as it appears in World Health Organization (WHO). WHO Mortality Database Version November 2015. Geneva, Switzerland: World Health Organization (WHO), 2015
United Kingdom - Northern Ireland Vital Registration - Deaths 2001 ICD10 as it appears in World Health Organization (WHO). WHO Mortality Database Version October 2017. Geneva, Switzerland: World Health Organization (WHO), 2017
United Kingdom - Northern Ireland Vital Registration - Deaths 2002 ICD10 as it appears in World Health Organization (WHO). WHO Mortality Database Version October 2017. Geneva, Switzerland: World Health Organization (WHO), 2017
United Kingdom - Northern Ireland Vital Registration - Deaths 2003 ICD10 as it appears in World Health Organization (WHO). WHO Mortality Database Version October 2017. Geneva, Switzerland: World Health Organization (WHO), 2017
United Kingdom - Northern Ireland Vital Registration - Deaths 2004 ICD10 as it appears in World Health Organization (WHO). WHO Mortality Database Version October 2017. Geneva, Switzerland: World Health Organization (WHO), 2017
United Kingdom - Northern Ireland Vital Registration - Deaths 2005 ICD10 as it appears in World Health Organization (WHO). WHO Mortality Database Version October 2017. Geneva, Switzerland: World Health Organization (WHO), 2017
United Kingdom - Northern Ireland Vital Registration - Deaths 2006 ICD10 as it appears in World Health Organization (WHO). WHO Mortality Database Version October 2017. Geneva, Switzerland: World Health Organization (WHO), 2017
United Kingdom - Northern Ireland Vital Registration - Deaths 2007 ICD10 as it appears in World Health Organization (WHO). WHO Mortality Database Version October 2017. Geneva, Switzerland: World Health Organization (WHO), 2017
United Kingdom - Northern Ireland Vital Registration - Deaths 1989 ICD9 as it appears in World Health Organization (WHO). WHO Mortality Database Version November 2015. Geneva, Switzerland: World Health Organization (WHO), 2015
United Kingdom - Northern Ireland Vital Registration - Deaths 1990 ICD9 as it appears in World Health Organization (WHO). WHO Mortality Database Version November 2015. Geneva, Switzerland: World Health Organization (WHO), 2015
United Kingdom - Northern Ireland Vital Registration - Deaths 1991 ICD9 as it appears in World Health Organization (WHO). WHO Mortality Database Version November 2015. Geneva, Switzerland: World Health Organization (WHO), 2015
United Kingdom - Northern Ireland Vital Registration - Deaths 1992 ICD9 as it appears in World Health Organization (WHO). WHO Mortality Database Version November 2015. Geneva, Switzerland: World Health Organization (WHO), 2015
United Kingdom - Northern Ireland Vital Registration - Deaths 2000 ICD9 as it appears in World Health Organization (WHO). WHO Mortality Database Version November 2015. Geneva, Switzerland: World Health Organization (WHO), 2015
United Kingdom - Northern Ireland Vital Registration - Deaths 2008 ICD10 as it appears in World Health Organization (WHO). WHO Mortality Database Version October 2017. Geneva, Switzerland: World Health Organization (WHO), 2017
United Kingdom - Northern Ireland Vital Registration - Deaths 2009 ICD10 as it appears in World Health Organization (WHO). WHO Mortality Database Version October 2017. Geneva, Switzerland: World Health Organization (WHO), 2017
United Kingdom - Northern Ireland Vital Registration - Deaths 2010 ICD10 as it appears in World Health Organization (WHO). WHO Mortality Database Version October 2017. Geneva, Switzerland: World Health Organization (WHO), 2017
United Kingdom - Northern Ireland Vital Registration - Deaths 2011 ICD10 as it appears in World Health Organization (WHO). WHO Mortality Database Version October 2017. Geneva, Switzerland: World Health Organization (WHO), 2017
United Kingdom - Northern Ireland Vital Registration - Deaths 2012 ICD10 as it appears in World Health Organization (WHO). WHO Mortality Database Version October 2017. Geneva, Switzerland: World Health Organization (WHO), 2017
United Kingdom - Northern Ireland Vital Registration - Deaths 2013 ICD10 as it appears in World Health Organization (WHO). WHO Mortality Database Version October 2017. Geneva, Switzerland: World Health Organization (WHO), 2017

United Kingdom - Northern Ireland Vital Registration - Deaths 2014 ICD10 as it appears in World Health Organization (WHO). WHO Mortality Database Version April 2018. Geneva, Switzerland: World Health Organization (WHO), 2018
United Kingdom - Northern Ireland Vital Registration - Deaths 2015 ICD10 as it appears in World Health Organization (WHO). WHO Mortality Database Version April 2018. Geneva, Switzerland: World Health Organization (WHO), 2018
United Kingdom - Scotland Vital Registration - Deaths 1980 ICD9 as it appears in World Health Organization (WHO). WHO Mortality Database Version November 2015. Geneva, Switzerland: World Health Organization (WHO), 2015
United Kingdom - Scotland Vital Registration - Deaths 1981 ICD9 as it appears in World Health Organization (WHO). WHO Mortality Database Version November 2015. Geneva, Switzerland: World Health Organization (WHO), 2015
United Kingdom - Scotland Vital Registration - Deaths 1982 ICD9 as it appears in World Health Organization (WHO). WHO Mortality Database Version November 2015. Geneva, Switzerland: World Health Organization (WHO), 2015
United Kingdom - Scotland Vital Registration - Deaths 1983 ICD9 as it appears in World Health Organization (WHO). WHO Mortality Database Version November 2015. Geneva, Switzerland: World Health Organization (WHO), 2015
United Kingdom - Scotland Vital Registration - Deaths 1984 ICD9 as it appears in World Health Organization (WHO). WHO Mortality Database Version November 2015. Geneva, Switzerland: World Health Organization (WHO), 2015
United Kingdom - Scotland Vital Registration - Deaths 1985 ICD9 as it appears in World Health Organization (WHO). WHO Mortality Database Version November 2015. Geneva, Switzerland: World Health Organization (WHO), 2015
United Kingdom - Scotland Vital Registration - Deaths 1986 ICD9 as it appears in World Health Organization (WHO). WHO Mortality Database Version November 2015. Geneva, Switzerland: World Health Organization (WHO), 2015
United Kingdom - Scotland Vital Registration - Deaths 1987 ICD9 as it appears in World Health Organization (WHO). WHO Mortality Database Version November 2015. Geneva, Switzerland: World Health Organization (WHO), 2015
United Kingdom - Scotland Vital Registration - Deaths 1988 ICD9 as it appears in World Health Organization (WHO). WHO Mortality Database Version November 2015. Geneva, Switzerland: World Health Organization (WHO), 2015
United Kingdom - Scotland Vital Registration - Deaths 1989 ICD9 as it appears in World Health Organization (WHO). WHO Mortality Database Version November 2015. Geneva, Switzerland: World Health Organization (WHO), 2015
United Kingdom - Scotland Vital Registration - Deaths 1990 ICD9 as it appears in World Health Organization (WHO). WHO Mortality Database Version November 2015. Geneva, Switzerland: World Health Organization (WHO), 2015
United Kingdom - Scotland Vital Registration - Deaths 1991 ICD9 as it appears in World Health Organization (WHO). WHO Mortality Database Version November 2015. Geneva, Switzerland: World Health Organization (WHO), 2015
United Kingdom - Scotland Vital Registration - Deaths 1992 ICD9 as it appears in World Health Organization (WHO). WHO Mortality Database Version November 2015. Geneva, Switzerland: World Health Organization (WHO), 2015
United Kingdom - Scotland Vital Registration - Deaths 1993 ICD9 as it appears in World Health Organization (WHO). WHO Mortality Database Version November 2015. Geneva, Switzerland: World Health Organization (WHO), 2015
United Kingdom - Scotland Vital Registration - Deaths 1994 ICD9 as it appears in World Health Organization (WHO). WHO Mortality Database Version November 2015. Geneva, Switzerland: World Health Organization (WHO), 2015
United Kingdom - Scotland Vital Registration - Deaths 1995 ICD9 as it appears in World Health Organization (WHO). WHO Mortality Database Version November 2015. Geneva, Switzerland: World Health Organization (WHO), 2015
United Kingdom - Scotland Vital Registration - Deaths 1996 ICD9 as it appears in World Health Organization (WHO). WHO Mortality Database Version November 2015. Geneva, Switzerland: World Health Organization (WHO), 2015
United Kingdom - Scotland Vital Registration - Deaths 1997 ICD9 as it appears in World Health Organization (WHO). WHO Mortality Database Version November 2015. Geneva, Switzerland: World Health Organization (WHO), 2015

United Kingdom - Scotland Vital Registration - Deaths 1998 ICD9 as it appears in World Health Organization (WHO). WHO Mortality Database Version November 2015. Geneva, Switzerland: World Health Organization (WHO), 2015
United Kingdom - Scotland Vital Registration - Deaths 1999 ICD9 as it appears in World Health Organization (WHO). WHO Mortality Database Version November 2015. Geneva, Switzerland: World Health Organization (WHO), 2015
United Kingdom - Scotland Vital Registration - Deaths 2000 ICD10 as it appears in World Health Organization (WHO). WHO Mortality Database Version October 2017. Geneva, Switzerland: World Health Organization (WHO), 2017
United Kingdom - Scotland Vital Registration - Deaths 2001 ICD10 as it appears in World Health Organization (WHO). WHO Mortality Database Version October 2017. Geneva, Switzerland: World Health Organization (WHO), 2017
United Kingdom - Scotland Vital Registration - Deaths 2002 ICD10 as it appears in World Health Organization (WHO). WHO Mortality Database Version October 2017. Geneva, Switzerland: World Health Organization (WHO), 2017
United Kingdom - Scotland Vital Registration - Deaths 2003 ICD10 as it appears in World Health Organization (WHO). WHO Mortality Database Version October 2017. Geneva, Switzerland: World Health Organization (WHO), 2017
United Kingdom - Scotland Vital Registration - Deaths 2004 ICD10 as it appears in World Health Organization (WHO). WHO Mortality Database Version October 2017. Geneva, Switzerland: World Health Organization (WHO), 2017
United Kingdom - Scotland Vital Registration - Deaths 2005 ICD10 as it appears in World Health Organization (WHO). WHO Mortality Database Version October 2017. Geneva, Switzerland: World Health Organization (WHO), 2017
United Kingdom - Scotland Vital Registration - Deaths 2006 ICD10 as it appears in World Health Organization (WHO). WHO Mortality Database Version October 2017. Geneva, Switzerland: World Health Organization (WHO), 2017
United Kingdom - Scotland Vital Registration - Deaths 2007 ICD10 as it appears in World Health Organization (WHO). WHO Mortality Database Version October 2017. Geneva, Switzerland: World Health Organization (WHO), 2017
United Kingdom - Scotland Vital Registration - Deaths 2008 ICD10 as it appears in World Health Organization (WHO). WHO Mortality Database Version October 2017. Geneva, Switzerland: World Health Organization (WHO), 2017
United Kingdom - Scotland Vital Registration - Deaths 2009 ICD10 as it appears in World Health Organization (WHO). WHO Mortality Database Version October 2017. Geneva, Switzerland: World Health Organization (WHO), 2017
United Kingdom - Scotland Vital Registration - Deaths 2010 ICD10 as it appears in World Health Organization (WHO). WHO Mortality Database Version October 2017. Geneva, Switzerland: World Health Organization (WHO), 2017
United Kingdom - Scotland Vital Registration - Deaths 2011 ICD10 as it appears in World Health Organization (WHO). WHO Mortality Database Version October 2017. Geneva, Switzerland: World Health Organization (WHO), 2017
United Kingdom - Scotland Vital Registration - Deaths 2012 ICD10 as it appears in World Health Organization (WHO). WHO Mortality Database Version October 2017. Geneva, Switzerland: World Health Organization (WHO), 2017
United Kingdom - Scotland Vital Registration - Deaths 2013 ICD10 as it appears in World Health Organization (WHO). WHO Mortality Database Version October 2017. Geneva, Switzerland: World Health Organization (WHO), 2017
United Kingdom - Scotland Vital Registration - Deaths 2014 ICD10 as it appears in World Health Organization (WHO). WHO Mortality Database Version October 2017. Geneva, Switzerland: World Health Organization (WHO), 2017
United States Virgin Islands Vital Registration - Deaths 1980 ICD9 as it appears in World Health Organization (WHO). WHO Mortality Database Version September 2016. Geneva, Switzerland: World Health Organization (WHO), 2016
Uruguay Vital Registration - Deaths 1980 ICD9 as it appears in World Health Organization (WHO). WHO Mortality Database Version September 2016. Geneva, Switzerland: World Health Organization (WHO), 2016
Uruguay Vital Registration - Deaths 1981 ICD9 as it appears in World Health Organization (WHO). WHO Mortality Database Version September 2016. Geneva, Switzerland: World Health Organization (WHO), 2016

Uruguay Vital Registration - Deaths 1982 ICD9 as it appears in World Health Organization (WHO). WHO Mortality Database Version September 2016. Geneva, Switzerland: World Health Organization (WHO), 2016
Uruguay Vital Registration - Deaths 1983 ICD9 as it appears in World Health Organization (WHO). WHO Mortality Database Version September 2016. Geneva, Switzerland: World Health Organization (WHO), 2016
Uruguay Vital Registration - Deaths 1984 ICD9 as it appears in World Health Organization (WHO). WHO Mortality Database Version September 2016. Geneva, Switzerland: World Health Organization (WHO), 2016
Uruguay Vital Registration - Deaths 1985 ICD9 as it appears in World Health Organization (WHO). WHO Mortality Database Version September 2016. Geneva, Switzerland: World Health Organization (WHO), 2016
Uruguay Vital Registration - Deaths 1986 ICD9 as it appears in World Health Organization (WHO). WHO Mortality Database Version September 2016. Geneva, Switzerland: World Health Organization (WHO), 2016
Uruguay Vital Registration - Deaths 1987 ICD9 as it appears in World Health Organization (WHO). WHO Mortality Database Version September 2016. Geneva, Switzerland: World Health Organization (WHO), 2016
Uruguay Vital Registration - Deaths 1989 ICD9 as it appears in World Health Organization (WHO). WHO Mortality Database Version September 2016. Geneva, Switzerland: World Health Organization (WHO), 2016
Uruguay Vital Registration - Deaths 1990 ICD9 as it appears in World Health Organization (WHO). WHO Mortality Database Version September 2016. Geneva, Switzerland: World Health Organization (WHO), 2016
Uruguay Vital Registration - Deaths 1993 ICD9 as it appears in World Health Organization (WHO). WHO Mortality Database Version September 2016. Geneva, Switzerland: World Health Organization (WHO), 2016
Uruguay Vital Registration - Deaths 1994 ICD9 as it appears in World Health Organization (WHO). WHO Mortality Database Version September 2016. Geneva, Switzerland: World Health Organization (WHO), 2016
Uruguay Vital Registration - Deaths 1995 ICD9 as it appears in World Health Organization (WHO). WHO Mortality Database Version September 2016. Geneva, Switzerland: World Health Organization (WHO), 2016
Uruguay Vital Registration - Deaths 1996 ICD9 as it appears in World Health Organization (WHO). WHO Mortality Database Version September 2016. Geneva, Switzerland: World Health Organization (WHO), 2016
Uruguay Vital Registration - Deaths 1997 ICD10 as it appears in World Health Organization (WHO). WHO Mortality Database Version October 2017. Geneva, Switzerland: World Health Organization (WHO), 2017
Uruguay Vital Registration - Deaths 1998 ICD10 as it appears in World Health Organization (WHO). WHO Mortality Database Version October 2017. Geneva, Switzerland: World Health Organization (WHO), 2017
Uruguay Vital Registration - Deaths 1999 ICD10 as it appears in World Health Organization (WHO). WHO Mortality Database Version October 2017. Geneva, Switzerland: World Health Organization (WHO), 2017
Uruguay Vital Registration - Deaths 2000 ICD10 as it appears in World Health Organization (WHO). WHO Mortality Database Version October 2017. Geneva, Switzerland: World Health Organization (WHO), 2017
Uruguay Vital Registration - Deaths 2001 ICD10 as it appears in World Health Organization (WHO). WHO Mortality Database Version October 2017. Geneva, Switzerland: World Health Organization (WHO), 2017
Uruguay Vital Registration - Deaths 2004 ICD10 as it appears in World Health Organization (WHO). WHO Mortality Database Version October 2017. Geneva, Switzerland: World Health Organization (WHO), 2017
Uruguay Vital Registration - Deaths 1988 ICD9 as it appears in World Health Organization (WHO). WHO Mortality Database Version September 2016. Geneva, Switzerland: World Health Organization (WHO), 2016
Uruguay Vital Registration - Deaths 2002 ICD10 as it appears in World Health Organization (WHO). WHO Mortality Database Version October 2017. Geneva, Switzerland: World Health Organization (WHO), 2017

Uruguay Vital Registration - Deaths 2003 ICD10 as it appears in World Health Organization (WHO). WHO Mortality Database Version October 2017. Geneva, Switzerland: World Health Organization (WHO), 2017

Uruguay Vital Registration - Deaths 2005 ICD10 as it appears in World Health Organization (WHO). WHO Mortality Database Version October 2017. Geneva, Switzerland: World Health Organization (WHO), 2017

Uruguay Vital Registration - Deaths 2006 ICD10 as it appears in World Health Organization (WHO). WHO Mortality Database Version October 2017. Geneva, Switzerland: World Health Organization (WHO), 2017

Uruguay Vital Registration - Deaths 2007 ICD10 as it appears in World Health Organization (WHO). WHO Mortality Database Version October 2017. Geneva, Switzerland: World Health Organization (WHO), 2017

Uruguay Vital Registration - Deaths 2008 ICD10 as it appears in World Health Organization (WHO). WHO Mortality Database Version October 2017. Geneva, Switzerland: World Health Organization (WHO), 2017

Uruguay Vital Registration - Deaths 2009 ICD10 as it appears in World Health Organization (WHO). WHO Mortality Database Version October 2017. Geneva, Switzerland: World Health Organization (WHO), 2017

Uruguay Vital Registration - Deaths 2010 ICD10 as it appears in World Health Organization (WHO). WHO Mortality Database Version October 2017. Geneva, Switzerland: World Health Organization (WHO), 2017

Uruguay Vital Registration - Deaths 2012 ICD10 as it appears in World Health Organization (WHO). WHO Mortality Database Version October 2017. Geneva, Switzerland: World Health Organization (WHO), 2017

Uruguay Vital Registration - Deaths 2013 ICD10 as it appears in World Health Organization (WHO). WHO Mortality Database Version October 2017. Geneva, Switzerland: World Health Organization (WHO), 2017

Uruguay Vital Registration - Deaths 2014 ICD10 as it appears in World Health Organization (WHO). WHO Mortality Database Version October 2017. Geneva, Switzerland: World Health Organization (WHO), 2017

Uruguay Vital Registration - Deaths 2015 ICD10 as it appears in World Health Organization (WHO). WHO Mortality Database Version October 2017. Geneva, Switzerland: World Health Organization (WHO), 2017

USSR - Russia Mortality by Region, Age, Sex, and Cause of Death 1980 as it appears in Center for Demographic Research, New Economic School (Russia). USSR - Russia Mortality Rates by Region, Age, Sex, and Cause of Death 1969-1989. Moscow, Russia: Center for Demographic Research, New Economic School (Russia). http://demogr.nes.ru/index.php/ru/demogr_indicat/data

Uzbekistan Vital Registration - Deaths 1981 ICD9 as it appears in World Health Organization (WHO). WHO Mortality Database Version September 2016. Geneva, Switzerland: World Health Organization (WHO), 2016

Uzbekistan Vital Registration - Deaths 1982 ICD9 as it appears in World Health Organization (WHO). WHO Mortality Database Version September 2016. Geneva, Switzerland: World Health Organization (WHO), 2016

Uzbekistan Vital Registration - Deaths 1985 ICD9 as it appears in World Health Organization (WHO). WHO Mortality Database Version September 2016. Geneva, Switzerland: World Health Organization (WHO), 2016

Uzbekistan Vital Registration - Deaths 1986 ICD9 as it appears in World Health Organization (WHO). WHO Mortality Database Version September 2016. Geneva, Switzerland: World Health Organization (WHO), 2016

Uzbekistan Vital Registration - Deaths 1987 ICD9 as it appears in World Health Organization (WHO). WHO Mortality Database Version September 2016. Geneva, Switzerland: World Health Organization (WHO), 2016

Uzbekistan Vital Registration - Deaths 1988 ICD9 as it appears in World Health Organization (WHO). WHO Mortality Database Version September 2016. Geneva, Switzerland: World Health Organization (WHO), 2016

Uzbekistan Vital Registration - Deaths 1989 ICD9 as it appears in World Health Organization (WHO). WHO Mortality Database Version September 2016. Geneva, Switzerland: World Health Organization (WHO), 2016

Uzbekistan Vital Registration - Deaths 1990 ICD9 as it appears in World Health Organization (WHO). WHO Mortality Database Version September 2016. Geneva, Switzerland: World Health Organization (WHO), 2016

Uzbekistan Vital Registration - Deaths 1991 ICD9 as it appears in World Health Organization (WHO). WHO Mortality Database Version September 2016. Geneva, Switzerland: World Health Organization (WHO), 2016
Uzbekistan Vital Registration - Deaths 1992 ICD9 as it appears in World Health Organization (WHO). WHO Mortality Database Version September 2016. Geneva, Switzerland: World Health Organization (WHO), 2016
Uzbekistan Vital Registration - Deaths 1993 ICD9 as it appears in World Health Organization (WHO). WHO Mortality Database Version September 2016. Geneva, Switzerland: World Health Organization (WHO), 2016
Uzbekistan Vital Registration - Deaths 1994 ICD9 as it appears in World Health Organization (WHO). WHO Mortality Database Version September 2016. Geneva, Switzerland: World Health Organization (WHO), 2016
Uzbekistan Vital Registration - Deaths 1995 ICD9 as it appears in World Health Organization (WHO). WHO Mortality Database Version September 2016. Geneva, Switzerland: World Health Organization (WHO), 2016
Uzbekistan Vital Registration - Deaths 1996 ICD9 as it appears in World Health Organization (WHO). WHO Mortality Database Version September 2016. Geneva, Switzerland: World Health Organization (WHO), 2016
Uzbekistan Vital Registration - Deaths 1997 ICD9 as it appears in World Health Organization (WHO). WHO Mortality Database Version September 2016. Geneva, Switzerland: World Health Organization (WHO), 2016
Uzbekistan Vital Registration - Deaths 1998 ICD9 as it appears in World Health Organization (WHO). WHO Mortality Database Version September 2016. Geneva, Switzerland: World Health Organization (WHO), 2016
Uzbekistan Vital Registration - Deaths 1999 ICD9 as it appears in World Health Organization (WHO). WHO Mortality Database Version September 2016. Geneva, Switzerland: World Health Organization (WHO), 2016
Uzbekistan Vital Registration - Deaths 2000 ICD9 as it appears in World Health Organization (WHO). WHO Mortality Database Version September 2016. Geneva, Switzerland: World Health Organization (WHO), 2016
Uzbekistan Vital Registration - Deaths 2001 ICD9 as it appears in World Health Organization (WHO). WHO Mortality Database Version September 2016. Geneva, Switzerland: World Health Organization (WHO), 2016
Uzbekistan Vital Registration - Deaths 2002 ICD9 as it appears in World Health Organization (WHO). WHO Mortality Database Version September 2016. Geneva, Switzerland: World Health Organization (WHO), 2016
Uzbekistan Vital Registration - Deaths 2003 ICD9 as it appears in World Health Organization (WHO). WHO Mortality Database Version September 2016. Geneva, Switzerland: World Health Organization (WHO), 2016
Uzbekistan Vital Registration - Deaths 2004 ICD10 as it appears in World Health Organization (WHO). WHO Mortality Database Version October 2017. Geneva, Switzerland: World Health Organization (WHO), 2017
Uzbekistan Vital Registration - Deaths 2005 ICD10 as it appears in World Health Organization (WHO). WHO Mortality Database Version October 2017. Geneva, Switzerland: World Health Organization (WHO), 2017
Uzbekistan Vital Registration - Deaths 2009 ICD10 as it appears in World Health Organization (WHO). WHO Mortality Database Version March 2017. Geneva, Switzerland: World Health Organization (WHO), 2017
Uzbekistan Vital Registration - Deaths 2010 ICD10 as it appears in World Health Organization (WHO). WHO Mortality Database Version March 2017. Geneva, Switzerland: World Health Organization (WHO), 2017
Uzbekistan Vital Registration - Deaths 2011 ICD10 as it appears in World Health Organization (WHO). WHO Mortality Database Version March 2017. Geneva, Switzerland: World Health Organization (WHO), 2017
Uzbekistan Vital Registration - Deaths 2012 ICD10 as it appears in World Health Organization (WHO). WHO Mortality Database Version March 2017. Geneva, Switzerland: World Health Organization (WHO), 2017
Uzbekistan Vital Registration - Deaths 2013 ICD10 as it appears in World Health Organization (WHO). WHO Mortality Database Version March 2017. Geneva, Switzerland: World Health Organization (WHO), 2017

Uzbekistan Vital Registration - Deaths 2014 ICD10 as it appears in World Health Organization (WHO). WHO Mortality Database Version October 2017. Geneva, Switzerland: World Health Organization (WHO), 2017
Venezuela Vital Registration - Deaths 1980 ICD9 as it appears in World Health Organization (WHO). WHO Mortality Database Version September 2016. Geneva, Switzerland: World Health Organization (WHO), 2016
Venezuela Vital Registration - Deaths 1981 ICD9 as it appears in World Health Organization (WHO). WHO Mortality Database Version September 2016. Geneva, Switzerland: World Health Organization (WHO), 2016
Venezuela Vital Registration - Deaths 1982 ICD9 as it appears in World Health Organization (WHO). WHO Mortality Database Version September 2016. Geneva, Switzerland: World Health Organization (WHO), 2016
Venezuela Vital Registration - Deaths 1983 ICD9 as it appears in World Health Organization (WHO). WHO Mortality Database Version September 2016. Geneva, Switzerland: World Health Organization (WHO), 2016
Venezuela Vital Registration - Deaths 1985 ICD9 as it appears in World Health Organization (WHO). WHO Mortality Database Version September 2016. Geneva, Switzerland: World Health Organization (WHO), 2016
Venezuela Vital Registration - Deaths 1986 ICD9 as it appears in World Health Organization (WHO). WHO Mortality Database Version September 2016. Geneva, Switzerland: World Health Organization (WHO), 2016
Venezuela Vital Registration - Deaths 1987 ICD9 as it appears in World Health Organization (WHO). WHO Mortality Database Version September 2016. Geneva, Switzerland: World Health Organization (WHO), 2016
Venezuela Vital Registration - Deaths 1988 ICD9 as it appears in World Health Organization (WHO). WHO Mortality Database Version September 2016. Geneva, Switzerland: World Health Organization (WHO), 2016
Venezuela Vital Registration - Deaths 1989 ICD9 as it appears in World Health Organization (WHO). WHO Mortality Database Version September 2016. Geneva, Switzerland: World Health Organization (WHO), 2016
Venezuela Vital Registration - Deaths 1990 ICD9 as it appears in World Health Organization (WHO). WHO Mortality Database Version September 2016. Geneva, Switzerland: World Health Organization (WHO), 2016
Venezuela Vital Registration - Deaths 1992 ICD9 as it appears in World Health Organization (WHO). WHO Mortality Database Version September 2016. Geneva, Switzerland: World Health Organization (WHO), 2016
Venezuela Vital Registration - Deaths 1993 ICD9 as it appears in World Health Organization (WHO). WHO Mortality Database Version September 2016. Geneva, Switzerland: World Health Organization (WHO), 2016
Venezuela Vital Registration - Deaths 1994 ICD9 as it appears in World Health Organization (WHO). WHO Mortality Database Version September 2016. Geneva, Switzerland: World Health Organization (WHO), 2016
Venezuela Vital Registration - Deaths 1996 ICD10 as it appears in World Health Organization (WHO). WHO Mortality Database Version October 2017. Geneva, Switzerland: World Health Organization (WHO), 2017
Venezuela Vital Registration - Deaths 1997 ICD10 as it appears in World Health Organization (WHO). WHO Mortality Database Version October 2017. Geneva, Switzerland: World Health Organization (WHO), 2017
Venezuela Vital Registration - Deaths 1998 ICD10 as it appears in World Health Organization (WHO). WHO Mortality Database Version October 2017. Geneva, Switzerland: World Health Organization (WHO), 2017
Venezuela Vital Registration - Deaths 1999 ICD10 as it appears in World Health Organization (WHO). WHO Mortality Database Version October 2017. Geneva, Switzerland: World Health Organization (WHO), 2017
Venezuela Vital Registration - Deaths 2000 ICD10 as it appears in World Health Organization (WHO). WHO Mortality Database Version October 2017. Geneva, Switzerland: World Health Organization (WHO), 2017
Venezuela Vital Registration - Deaths 2001 ICD10 as it appears in World Health Organization (WHO). WHO Mortality Database Version October 2017. Geneva, Switzerland: World Health Organization (WHO), 2017

Venezuela Vital Registration - Deaths 2002 ICD10 as it appears in World Health Organization (WHO). WHO Mortality Database Version October 2017. Geneva, Switzerland: World Health Organization (WHO), 2017
Venezuela Vital Registration - Deaths 2003 ICD10 as it appears in World Health Organization (WHO). WHO Mortality Database Version October 2017. Geneva, Switzerland: World Health Organization (WHO), 2017
Venezuela Vital Registration - Deaths 2004 ICD10 as it appears in World Health Organization (WHO). WHO Mortality Database Version October 2017. Geneva, Switzerland: World Health Organization (WHO), 2017
Venezuela Vital Registration - Deaths 2005 ICD10 as it appears in World Health Organization (WHO). WHO Mortality Database Version October 2017. Geneva, Switzerland: World Health Organization (WHO), 2017
Venezuela Vital Registration - Deaths 2006 ICD10 as it appears in World Health Organization (WHO). WHO Mortality Database Version October 2017. Geneva, Switzerland: World Health Organization (WHO), 2017
Venezuela Vital Registration - Deaths 2007 ICD10 as it appears in World Health Organization (WHO). WHO Mortality Database Version October 2017. Geneva, Switzerland: World Health Organization (WHO), 2017
Venezuela Vital Registration - Deaths 2008 ICD10 as it appears in World Health Organization (WHO). WHO Mortality Database Version October 2017. Geneva, Switzerland: World Health Organization (WHO), 2017
Venezuela Vital Registration - Deaths 2009 ICD10 as it appears in World Health Organization (WHO). WHO Mortality Database Version October 2017. Geneva, Switzerland: World Health Organization (WHO), 2017
Venezuela Vital Registration - Deaths 2010 ICD10 as it appears in World Health Organization (WHO). WHO Mortality Database Version October 2017. Geneva, Switzerland: World Health Organization (WHO), 2017
Venezuela Vital Registration - Deaths 2011 ICD10 as it appears in World Health Organization (WHO). WHO Mortality Database Version October 2017. Geneva, Switzerland: World Health Organization (WHO), 2017
Venezuela Vital Registration - Deaths 2012 ICD10 as it appears in World Health Organization (WHO). WHO Mortality Database Version October 2017. Geneva, Switzerland: World Health Organization (WHO), 2017
Venezuela Vital Registration - Deaths 2013 ICD10 as it appears in World Health Organization (WHO). WHO Mortality Database Version October 2017. Geneva, Switzerland: World Health Organization (WHO), 2017
Würthwein R, Gbangou A, Sauerborn R, Schmidt CM. Measuring the local burden of disease. A study of years of life lost in sub-Saharan Africa. Int J Epidemiol. 2001; 30(3): 501-8
Yugoslavia - Bosnia and Herzegovina Vital Registration - Deaths 1985 ICD9 as it appears in World Health Organization (WHO). WHO Mortality Database Version September 2016. Geneva, Switzerland: World Health Organization (WHO), 2016
Yugoslavia - Bosnia and Herzegovina Vital Registration - Deaths 1986 ICD9 as it appears in World Health Organization (WHO). WHO Mortality Database Version September 2016. Geneva, Switzerland: World Health Organization (WHO), 2016
Yugoslavia - Bosnia and Herzegovina Vital Registration - Deaths 1987 ICD9 as it appears in World Health Organization (WHO). WHO Mortality Database Version September 2016. Geneva, Switzerland: World Health Organization (WHO), 2016
Yugoslavia - Bosnia and Herzegovina Vital Registration - Deaths 1988 ICD9 as it appears in World Health Organization (WHO). WHO Mortality Database Version September 2016. Geneva, Switzerland: World Health Organization (WHO), 2016
Yugoslavia - Bosnia and Herzegovina Vital Registration - Deaths 1989 ICD9 as it appears in World Health Organization (WHO). WHO Mortality Database Version September 2016. Geneva, Switzerland: World Health Organization (WHO), 2016
Yugoslavia - Bosnia and Herzegovina Vital Registration - Deaths 1990 ICD9 as it appears in World Health Organization (WHO). WHO Mortality Database Version September 2016. Geneva, Switzerland: World Health Organization (WHO), 2016
Yugoslavia - Croatia Vital Registration - Deaths 1985 ICD9 as it appears in World Health Organization (WHO). WHO Mortality Database Version November 2015. Geneva, Switzerland: World Health Organization (WHO), 2015

Yugoslavia - Croatia Vital Registration - Deaths 1986 ICD9 as it appears in World Health Organization (WHO). WHO Mortality Database Version November 2015. Geneva, Switzerland: World Health Organization (WHO), 2015
Yugoslavia - Croatia Vital Registration - Deaths 1987 ICD9 as it appears in World Health Organization (WHO). WHO Mortality Database Version November 2015. Geneva, Switzerland: World Health Organization (WHO), 2015
Yugoslavia - Croatia Vital Registration - Deaths 1988 ICD9 as it appears in World Health Organization (WHO). WHO Mortality Database Version November 2015. Geneva, Switzerland: World Health Organization (WHO), 2015
Yugoslavia - Croatia Vital Registration - Deaths 1989 ICD9 as it appears in World Health Organization (WHO). WHO Mortality Database Version November 2015. Geneva, Switzerland: World Health Organization (WHO), 2015
Yugoslavia - Croatia Vital Registration - Deaths 1990 ICD9 as it appears in World Health Organization (WHO). WHO Mortality Database Version November 2015. Geneva, Switzerland: World Health Organization (WHO), 2015
Yugoslavia - Slovenia Vital Registration - Deaths 1985 ICD9 as it appears in World Health Organization (WHO). WHO Mortality Database Version November 2015. Geneva, Switzerland: World Health Organization (WHO), 2015
Yugoslavia - Slovenia Vital Registration - Deaths 1986 ICD9 as it appears in World Health Organization (WHO). WHO Mortality Database Version November 2015. Geneva, Switzerland: World Health Organization (WHO), 2015
Yugoslavia - Slovenia Vital Registration - Deaths 1987 ICD9 as it appears in World Health Organization (WHO). WHO Mortality Database Version November 2015. Geneva, Switzerland: World Health Organization (WHO), 2015
Yugoslavia - Slovenia Vital Registration - Deaths 1988 ICD9 as it appears in World Health Organization (WHO). WHO Mortality Database Version November 2015. Geneva, Switzerland: World Health Organization (WHO), 2015
Yugoslavia - Slovenia Vital Registration - Deaths 1990 ICD9 as it appears in World Health Organization (WHO). WHO Mortality Database Version November 2015. Geneva, Switzerland: World Health Organization (WHO), 2015
Yugoslavia, Federal Republic - Montenegro Vital Registration - Deaths 2000 ICD10 as it appears in World Health Organization (WHO). WHO Mortality Database Version October 2017. Geneva, Switzerland: World Health Organization (WHO), 2017
Yugoslavia, Federal Republic - Serbia Vital Registration - Deaths 1998 ICD10 as it appears in World Health Organization (WHO). WHO Mortality Database Version October 2017. Geneva, Switzerland: World Health Organization (WHO), 2017
Yugoslavia, Federal Republic - Serbia Vital Registration - Deaths 1999 ICD10 as it appears in World Health Organization (WHO). WHO Mortality Database Version October 2017. Geneva, Switzerland: World Health Organization (WHO), 2017
Yugoslavia, Federal Republic - Serbia Vital Registration - Deaths 2000 ICD10 as it appears in World Health Organization (WHO). WHO Mortality Database Version October 2017. Geneva, Switzerland: World Health Organization (WHO), 2017
Yugoslavia, Federal Republic - Serbia Vital Registration - Deaths 2001 ICD10 as it appears in World Health Organization (WHO). WHO Mortality Database Version October 2017. Geneva, Switzerland: World Health Organization (WHO), 2017
Yugoslavia, Federal Republic - Serbia Vital Registration - Deaths 2002 ICD10 as it appears in World Health Organization (WHO). WHO Mortality Database Version October 2017. Geneva, Switzerland: World Health Organization (WHO), 2017
Zimbabwe Vital Registration - Deaths 1990 ICD9 as it appears in World Health Organization (WHO). WHO Mortality Database Version September 2016. Geneva, Switzerland: World Health Organization (WHO), 2016

Table S15: Alphabetical listing of all data sources used in estimating nonfatal health outcomes (incidence, prevalence, YLDs) due to congenital heart anomalies for GBD 2017

Citation from Global Health Data Exchange (http://ghdx.healthdata.org/gbd-2017/data-input-sources)
Abt Associates Inc., Kenya National Bureau of Statistics, Ministry of Health (Kenya). Kenya Household Health Expenditure and Utilization Survey 2007. Nairobi, Kenya: Kenya National Bureau of Statistics
Abudu OO, Uguru V, Olude O. Contribution of congenital malformation to perinatal mortality in Lagos, Nigeria. Int J Gynaecol Obstet. 1988; 27(1): 63-7
Action Africa Help International (AAH-I), Institute for Health Metrics and Evaluation (IHME), Ministry of Medical Services (Kenya), Ministry of Public Health and Sanitation (Kenya). Access, Bottlenecks, Costs, and Equity (ABCE) project in Kenya, 2012. Seattle, United States: Institute for Health Metrics and Evaluation (IHME), 2015
Administrative Department of Science, Technology, and Innovation (Colombia), Center for Development Projects, Pontifical Xavierian University, Ministry of Social Protection (Colombia), Specialized Information Systems. Colombia National Health Survey 2007-2008
Agarwal AK, Venugopalan P, de Bono D. Prevalence and aetiology of heart failure in an Arab population. Eur J Heart Fail. 2001; 3(3): 301-5
Agency for Statistics (Bosnia and Herzegovina), Birks Sinclair and Associates, LTD, Federal Office of Statistics (Federation of Bosnia and Herzegovina), Independent Bureau for Humanitarian Issues (IBHI), Institute for Social and Economic Research, University of Essex, Institute of Statistics (Republic of Srpska). Bosnia and Herzegovina Living Standards Measurement Survey 2004-2005
Agency for Statistics (Bosnia and Herzegovina), Institute of Statistics (Republic of Srpska), Federal Office of Statistics (Bosnia and Herzegovina), Independent Bureau for Humanitarian Issues (IBHI), Birks Sinclair and Associates, LTD, Institute for Social and Economic Research, University of Essex. Bosnia and Herzegovina Living Standards Measurement Survey 2002. Washington, DC, United States: World Bank (WB)
Agency for Statistics (Bosnia and Herzegovina), Institute of Statistics (Republic of Srpska), Federal Office of Statistics (Bosnia and Herzegovina), Swedish International Development Agency (SIDA), UK Department for International Development (DFID), United Nations Development Programme (UNDP), European Commission (EC), Government of Japan, World Bank (WB). Bosnia and Herzegovina Living Standards Measurement Survey 2001. Washington, DC, United States: World Bank (WB)
Agency of the Republic of Kazakhstan on Statistics, World Bank. Kazakhstan Living Standards Measurement Survey 1996. Washington DC, United States: World Bank
Alqurashi M, El Mouzan M, Al Herbish A, Al Salloum A, Al Omer A. Symptomatic congenital heart disease in the Saudi Children and Adolescents Project. Ann Saudi Med. 2007; 27(6): 442-4
AMATEM (Turkey), Plaza Ltd. Research, World Health Organization (WHO). Turkey WHO Multi-country Survey Study on Health and Health System Responsiveness 2000-2001. Geneva, Switzerland: World Health Organization (WHO)
Amel-Shahbaz S, Behjati-Ardakani M, Namayandeh SM, Vafaeenasab M, Andishmand A, Moghimi S, Negahdary M, Sarebanhassanabadi M. The epidemiological aspects of congenital heart disease in central and southern district of Iran. Adv Biomed Res. 2014; 3: 233
Amoah AG, Kallen C. Aetiology of heart failure as seen from a National Cardiac Referral Centre in Africa. Cardiology. 2000; 93(1-2): 11-8
Amorim LFP, Pires CAB, Lana AMA, Campos AS, Aguiar RALP, Tibúrcio JD, Siqueira AL, Mota CCC, Aguiar MJB. Presentation of congenital heart disease diagnosed at birth: analysis of 29,770 newborn infants. J Pediatr (Rio J). 2008; 84(1): 83-90
Analytical and Information Center of the Ministry of Health of Uzbekistan, Macro International, Inc, Ministry of Macroeconomics and Statistics (Uzbekistan). Uzbekistan Special Demographic and Health Survey 2002. Fairfax, United States: ICF International

Argentina Latin American Collaborative Study of Congenital Malformations Data 1993-1998 - WHO as it appears in European Surveillance of Congenital Anomalies (EUROCAT), International Centre on Birth Defects, World Health Organization (WHO). World Atlas of Birth Defects. 2nd ed. Geneva, Switzerland: World Health Organization (WHO), 2003

Argentina National Registry of Congenital Anomalies Data 2010 - ICBDSR as it appears in International Clearinghouse for Birth Defects Surveillance and Research. International Clearinghouse for Birth Defects Surveillance and Research Annual Report 2012. Rome, Italy: International Clearinghouse for Birth Defects Surveillance and Research, 2013

Argentina National Registry of Congenital Anomalies Data 2012 - ICBDSR as it appears in International Clearinghouse for Birth Defects Surveillance and Research. International Clearinghouse for Birth Defects Surveillance and Research Annual Report 2014. Rome, Italy: International Clearinghouse for Birth Defects Surveillance and Research, 2016

Armenian Sociological Association, Concluzia-Prim Center for Survey Methodology (Moldova), Institute for Advanced Studies (Austria), London School of Hygiene and Tropical Medicine, University of Aberdeen. Armenia Health in Times of Transition Household Survey 2010

Ashraf M, Chowdhary J, Khajuria K, Reyaz AM. Spectrum of congenital heart diseases in Kashmir, India. Indian Pediatr. 2009; 46(12): 1107-8

Attie F, Rosas M, Granados N, Zabal C, Buendía A, Calderón J. Surgical treatment for secundum atrial septal defects in patients >40 years old. A randomized clinical trial. J Am Coll Cardiol. 2001; 38(7): 2035-42

Australia - Victoria Birth Defects Registry Data 2001 - ICBDMS as it appears in International Clearinghouse for Birth Defects Monitoring Systems. International Clearinghouse for Birth Defects Monitoring Systems Annual Report 2003. Rome, Italy: International Centre on Birth Defects

Australia - Victoria Birth Defects Registry Data 2002 - ICBDMS as it appears in International Clearinghouse for Birth Defects Monitoring Systems. International Clearinghouse for Birth Defects Monitoring Systems Annual Report 2004. Rome, Italy: International Centre on Birth Defects, 2006

Australia - Victoria Birth Defects Registry Data 2003 - ICBDSR as it appears in International Clearinghouse for Birth Defects Surveillance and Research. International Clearinghouse for Birth Defects Surveillance and Research Annual Report 2005. Rome, Italy: International Clearinghouse for Birth Defects Surveillance and Research, 2007

Australia - Victoria Birth Defects Registry Data 2004 - ICBDSR as it appears in International Clearinghouse for Birth Defects Surveillance and Research. International Clearinghouse for Birth Defects Surveillance and Research Annual Report 2006. Rome, Italy: International Clearinghouse for Birth Defects Surveillance and Research, 2007

Australia - Victoria Birth Defects Registry Data 2005 - ICBDSR as it appears in International Clearinghouse for Birth Defects Surveillance and Research. International Clearinghouse for Birth Defects Surveillance and Research Annual Report 2007. Rome, Italy: International Clearinghouse for Birth Defects Surveillance and Research, 2008

Australia - Victoria Birth Defects Registry Data 2007 - ICBDSR as it appears in International Clearinghouse for Birth Defects Surveillance and Research. International Clearinghouse for Birth Defects Surveillance and Research Annual Report 2009. Rome, Italy: International Clearinghouse for Birth Defects Surveillance and Research

Australia - Victoria Birth Defects Registry Data 2008 - ICBDSR as it appears in International Clearinghouse for Birth Defects Surveillance and Research. International Clearinghouse for Birth Defects Surveillance and Research Annual Report 2010. Rome, Italy: International Clearinghouse for Birth Defects Surveillance and Research, 2011

Australia - Western Australian Birth Defects Registry Data 2002 - ICBDMS as it appears in International Clearinghouse for Birth Defects Monitoring Systems. International Clearinghouse for Birth Defects Monitoring Systems Annual Report 2004. Rome, Italy: International Centre on Birth Defects, 2006

Australia - Western Australian Birth Defects Registry Data 2003 - ICBDSR as it appears in International Clearinghouse for Birth Defects Surveillance and Research. International Clearinghouse for Birth Defects Surveillance and Research Annual Report 2005. Rome, Italy: International Clearinghouse for Birth Defects Surveillance and Research, 2007

Australia - Western Australian Birth Defects Registry Data 2004 - ICBDSR as it appears in International Clearinghouse for Birth Defects Surveillance and Research. International Clearinghouse for Birth Defects Surveillance and Research Annual Report 2006. Rome, Italy: International Clearinghouse for Birth Defects Surveillance and Research, 2007

Australia - Western Australian Birth Defects Registry Data 2005 - ICBDSR as it appears in International Clearinghouse for Birth Defects Surveillance and Research. International Clearinghouse for Birth Defects Surveillance and Research Annual Report 2007. Rome, Italy: International Clearinghouse for Birth Defects Surveillance and Research, 2008

Australia - Western Australian Birth Defects Registry Data 2007 - ICBDSR as it appears in International Clearinghouse for Birth Defects Surveillance and Research. International Clearinghouse for Birth Defects Surveillance and Research Annual Report 2009. Rome, Italy: International Clearinghouse for Birth Defects Surveillance and Research

Australia - Western Australian Register of Developmental Anomalies Data 2008 - ICBDSR as it appears in International Clearinghouse for Birth Defects Surveillance and Research. International Clearinghouse for Birth Defects Surveillance and Research Annual Report 2010. Rome, Italy: International Clearinghouse for Birth Defects Surveillance and Research, 2011

Australia - Western Australian Register of Developmental Anomalies Data 2009 - ICBDSR as it appears in International Clearinghouse for Birth Defects Surveillance and Research. International Clearinghouse for Birth Defects Surveillance and Research Annual Report 2011. Rome, Italy: International Clearinghouse for Birth Defects Surveillance and Research, 2012

Australia - Western Australian Register of Developmental Anomalies Data 2010 - ICBDSR as it appears in International Clearinghouse for Birth Defects Surveillance and Research. International Clearinghouse for Birth Defects Surveillance and Research Annual Report 2012. Rome, Italy: International Clearinghouse for Birth Defects Surveillance and Research, 2013

Australia - Western Australian Register of Developmental Anomalies Data 2012 - ICBDSR as it appears in International Clearinghouse for Birth Defects Surveillance and Research. International Clearinghouse for Birth Defects Surveillance and Research Annual Report 2014. Rome, Italy: International Clearinghouse for Birth Defects Surveillance and Research, 2016

Australian Bureau of Statistics. Australia National Health Survey 1995. Canberra, Australia: Australian Bureau of Statistics

Australian Congenital Malformation Monitoring System Data 1993-1997 - WHO as it appears in European Surveillance of Congenital Anomalies (EUROCAT), International Centre on Birth Defects, World Health Organization (WHO). World Atlas of Birth Defects. 2nd ed. Geneva, Switzerland: World Health Organization (WHO), 2003

Austria - Styrian Malformation Registry Data 1993-1998 - WHO as it appears in European Surveillance of Congenital Anomalies (EUROCAT), International Centre on Birth Defects, World Health Organization (WHO). World Atlas of Birth Defects. 2nd ed. Geneva, Switzerland: World Health Organization (WHO), 2003

Bahamas Department of Statistics, Ministry of Health (Bahamas). Bahamas Living Conditions Survey 2001. Nassau, The Bahamas: Bahamas Department of Statistics

Bannerman CH, Mahalu W. Congenital heart disease in Zimbabwean children. Ann Trop Paediatr. 1998; 18(1): 5-12

Baruah J, Kusre G, Bora R. Pattern of Gross Congenital Malformations in a Tertiary Referral Hospital in Northeast India. Indian J Pediatr. 2015; 82(10): 917–22

Başpinar O, Karaaslan S, Oran B, Baysal T, Elmaci AM, Yorulmaz A. Prevalence and distribution of children with congenital heart diseases in the central Anatolian region, Turkey. Turk J Pediatr. 2006; 48(3): 237-43

Bedard T, Lowry RB, Sibbald B, Harder JR, Trevenen C, Horobec V, Dyck JD. Congenital heart defect case ascertainment by the Alberta Congenital Anomalies Surveillance System. Birth Defects Res A Clin Mol Teratol. 2012; 94(6): 449-58

Begić H, Tahirović H, Mesihović-Dinarević S, Ferković V, Atić N, Latifagić A. Epidemiological and clinical aspects of congenital heart disease in children in Tuzla Canton, Bosnia-Herzegovina. Eur J Pediatr. 2003; 162(3): 191–3

Belarusian State University, Concluzia-Prim Center for Survey Methodology (Moldova), Institute for Advanced Studies (Austria), London School of Hygiene and Tropical Medicine, University of Aberdeen. Belarus Health in Times of Transition Household Survey 2010

Belgium - Antwerp Congenital Anomaly Data 1993-1998 - WHO as it appears in European Surveillance of Congenital Anomalies (EUROCAT), International Centre on Birth Defects, World Health Organization (WHO). World Atlas of Birth Defects. 2nd ed. Geneva, Switzerland: World Health Organization (WHO), 2003

Belgium - Hainaut-Namur Congenital Anomaly Registry Data 1993-1998 - WHO as it appears in European Surveillance of Congenital Anomalies (EUROCAT), International Centre on Birth Defects, World Health Organization (WHO). World Atlas of Birth Defects. 2nd ed. Geneva, Switzerland: World Health Organization (WHO), 2003

Bellizzi S, Ali MM, Abalos E, Betran AP, Kapila J, Pileggi-Castro C, Vogel JP, Merialdi M. Are hypertensive disorders in pregnancy associated with congenital malformations in offspring? Evidence from the WHO Multicountry cross sectional survey on maternal and newborn health. BMC Pregnancy Childbirth. 2016; 16(1): 198

Berul CI, Hill SL, Geggel RL, Hijazi ZM, Marx GR, Rhodes J, Walsh KA, Fulton DR. Electrocardiographic markers of late sudden death risk in postoperative tetralogy of Fallot children. J Cardiovasc Electrophysiol. 1997; 8(12): 1349-56

Bhardwaj R, Kandoria A, Marwah R, Vaidya P, Singh B, Dhiman P, Sood A, Sharma A. Prevalence of congenital heart disease in rural population of Himachal - A population-based study. Indian Heart J. 2016; 68(1): 48-51

Biomedical Engineering Institute, Kaunas University of Technology, Statistics Lithuania, World Health Organization (WHO). Lithuania WHO Multi-country Survey Study on Health and Health System Responsiveness 2000-2001. Geneva, Switzerland: World Health Organization (WHO)

Bolisetty S, Daftary A, Ewald D, Knight B, Wheaton G. Congenital heart defects in Central Australia. Med J Aust. 2004; 180(12): 614-7

Bolivia Latin American Collaborative Study of Congenital Malformations Data 1993-1998 - WHO as it appears in European Surveillance of Congenital Anomalies (EUROCAT), International Centre on Birth Defects, World Health Organization (WHO). World Atlas of Birth Defects. 2nd ed. Geneva, Switzerland: World Health Organization (WHO), 2003

Boneva RS, Botto LD, Moore CA, Yang Q, Correa A, Erickson JD. Mortality associated with congenital heart defects in the United States: trends and racial disparities, 1979-1997. Circulation. 2001; 103(19): 2376-81

Börsch-Supan, A. (2013). Survey of Health, Ageing and Retirement in Europe (SHARE) Wave 1. Release version: 2.6.0. SHARE-ERIC. Data set. DOI: 10.6103/SHARE.w1.260

Börsch-Supan, A. (2013). Survey of Health, Ageing and Retirement in Europe (SHARE) Wave 2. Release version: 2.6.0. SHARE-ERIC. Data set. DOI: 10.6103/SHARE.w2.260

Börsch-Supan, A. (2013). Survey of Health, Ageing and Retirement in Europe (SHARE) Wave 4. Release version: 1.1.1. SHARE-ERIC. Data set. DOI: 10.6103/SHARE.w4.111

Börsch-Supan, A. (2015). Survey of Health, Ageing and Retirement in Europe (SHARE) Wave 5. Release version: 1.0.0. SHARE-ERIC. Data set. DOI: 10.6103/SHARE.w5.100

Börsch-Supan, A. (2015). Survey of Health, Ageing and Retirement in Europe (SHARE) Wave 5. Release version: 1.0.0. SHARE-ERIC. Data set. DOI: 10.6103/SHARE.w5.100

Börsch-Supan, A. (2015). Survey of Health, Ageing and Retirement in Europe (SHARE) Wave 5. Release version: 1.0.0. SHARE-ERIC. Data set. DOI: 10.6103/SHARE.w5.100

Börsch-Supan, A. (2015). Survey of Health, Ageing and Retirement in Europe (SHARE) Wave 5. Release version: 1.0.0. SHARE-ERIC. Data set. DOI: 10.6103/SHARE.w5.100

Börsch-Supan, A. (2015). Survey of Health, Ageing and Retirement in Europe (SHARE) Wave 5. Release version: 1.0.0. SHARE-ERIC. Data set. DOI: 10.6103/SHARE.w5.100

Börsch-Supan, A. (2015). Survey of Health, Ageing and Retirement in Europe (SHARE) Wave 5. Release version: 1.0.0. SHARE-ERIC. Data set. DOI: 10.6103/SHARE.w5.100

Börsch-Supan, A. (2015). Survey of Health, Ageing and Retirement in Europe (SHARE) Wave 5. Release version: 1.0.0. SHARE-ERIC. Data set. DOI: 10.6103/SHARE.w5.100

Börsch-Supan, A. (2015). Survey of Health, Ageing and Retirement in Europe (SHARE) Wave 5. Release version: 1.0.0. SHARE-ERIC. Data set. DOI: 10.6103/SHARE.w5.100

Börsch-Supan, A. (2015). Survey of Health, Ageing and Retirement in Europe (SHARE) Wave 5. Release version: 1.0.0. SHARE-ERIC. Data set. DOI: 10.6103/SHARE.w5.100

Börsch-Supan, A. (2015). Survey of Health, Ageing and Retirement in Europe (SHARE) Wave 5. Release version: 1.0.0. SHARE-ERIC. Data set. DOI: 10.6103/SHARE.w5.100

Börsch-Supan, A. (2015). Survey of Health, Ageing and Retirement in Europe (SHARE) Wave 5. Release version: 1.0.0. SHARE-ERIC. Data set. DOI: 10.6103/SHARE.w5.100

Börsch-Supan, A. (2015). Survey of Health, Ageing and Retirement in Europe (SHARE) Wave 5. Release version: 1.0.0. SHARE-ERIC. Data set. DOI: 10.6103/SHARE.w5.100

Börsch-Supan, A. (2015). Survey of Health, Ageing and Retirement in Europe (SHARE) Wave 5. Release version: 1.0.0. SHARE-ERIC. Data set. DOI: 10.6103/SHARE.w5.100

Börsch-Supan, A. (2015). Survey of Health, Ageing and Retirement in Europe (SHARE) Wave 5. Release version: 1.0.0. SHARE-ERIC. Data set. DOI: 10.6103/SHARE.w5.100

Börsch-Supan, A. (2015). Survey of Health, Ageing and Retirement in Europe (SHARE) Wave 5. Release version: 1.0.0. SHARE-ERIC. Data set. DOI: 10.6103/SHARE.w5.100

Bove FJ, Fulcomer MC, Klotz JB, Esmart J, Dufficy EM, Savrin JE. Public drinking water contamination and birth outcomes. Am J Epidemiol. 1995; 141(9): 850-62

Bower C, Ramsay JM. Congenital heart disease: a 10 year cohort. J Paediatr Child Health. 1994; 30(5): 414-8

Bravo Tobar I, Parra F, Nello Pérez C, Rodríguez-Bonfante C, Useche F, Bonfante-Cabarcas R. Prevalence of Trypanosoma cruzi antibodies and inflammatory markers in uncompensated heart failure. Rev Soc Bras Med Trop. 2011; 44(6): 691-6

Brazil Latin American Collaborative Study of Congenital Malformations Data 1993-1998 - WHO as it appears in European Surveillance of Congenital Anomalies (EUROCAT), International Centre on Birth Defects, World Health Organization (WHO). World Atlas of Birth Defects. 2nd ed. Geneva, Switzerland: World Health Organization (WHO), 2003

Brazilian Institute of Geography and Statistics (IBGE), Ministry of Health (Brazil), Ministry of Planning, Budget, and Management (Brazil). Brazil National Health Survey 2013. Rio de Janeiro, Brazil: Brazilian Institute of Geography and Statistics (IBGE)

Brick DH, Allan LD. Outcome of prenatally diagnosed congenital heart disease: an update. Pediatr Cardiol. 2002; 23(4): 449-53
Budd JL, Draper ES, Lotto RR, Berry LE, Smith LK. Socioeconomic inequalities in pregnancy outcome associated with Down syndrome: a population-based study. Arch Dis Child Fetal Neonatal Ed. 2015; 100(5): F400-4
Bulgaria - Sofia Registry of Congenital Anomalies Data 1996-1997 - WHO as it appears in European Surveillance of Congenital Anomalies (EUROCAT), International Centre on Birth Defects, World Health Organization (WHO). World Atlas of Birth Defects. 2nd ed. Geneva, Switzerland: World Health Organization (WHO), 2003
Bureau of Statistics (Guyana), World Bank. Guyana Living Standards Measurement Survey 1992-1993
California Center for Population Research (CCPR), University of California Los Angeles (UCLA), Center for Research and Teaching in Economics (CIDE) (Mexico), Ibero-American University, National Institute of Public Health (Mexico). Mexico Family Life Survey 2005-2006
Canada - Alberta Congenital Anomalies Surveillance System Data 1993-1998 - WHO as it appears in European Surveillance of Congenital Anomalies (EUROCAT), International Centre on Birth Defects, World Health Organization (WHO). World Atlas of Birth Defects. 2nd ed. Geneva, Switzerland: World Health Organization (WHO), 2003
Canada - Alberta Congenital Anomalies Surveillance System Data 2001 - ICBDMS as it appears in International Clearinghouse for Birth Defects Monitoring Systems. International Clearinghouse for Birth Defects Monitoring Systems Annual Report 2003. Rome, Italy: International Centre on Birth Defects
Canada - Alberta Congenital Anomalies Surveillance System Data 2002 - ICBDMS as it appears in International Clearinghouse for Birth Defects Monitoring Systems. International Clearinghouse for Birth Defects Monitoring Systems Annual Report 2004. Rome, Italy: International Centre on Birth Defects, 2006
Canada - Alberta Congenital Anomalies Surveillance System Data 2003 - ICBDSR as it appears in International Clearinghouse for Birth Defects Surveillance and Research. International Clearinghouse for Birth Defects Surveillance and Research Annual Report 2005. Rome, Italy: International Clearinghouse for Birth Defects Surveillance and Research, 2007
Canada - Alberta Congenital Anomalies Surveillance System Data 2004 - ICBDSR as it appears in International Clearinghouse for Birth Defects Surveillance and Research. International Clearinghouse for Birth Defects Surveillance and Research Annual Report 2006. Rome, Italy: International Clearinghouse for Birth Defects Surveillance and Research, 2007
Canada - Alberta Congenital Anomalies Surveillance System Data 2005 - ICBDSR as it appears in International Clearinghouse for Birth Defects Surveillance and Research. International Clearinghouse for Birth Defects Surveillance and Research Annual Report 2007. Rome, Italy: International Clearinghouse for Birth Defects Surveillance and Research, 2008
Canada - Alberta Congenital Anomalies Surveillance System Data 2007 - ICBDSR as it appears in International Clearinghouse for Birth Defects Surveillance and Research. International Clearinghouse for Birth Defects Surveillance and Research Annual Report 2009. Rome, Italy: International Clearinghouse for Birth Defects Surveillance and Research
Canada - Alberta Congenital Anomalies Surveillance System Data 2008 - ICBDSR as it appears in International Clearinghouse for Birth Defects Surveillance and Research. International Clearinghouse for Birth Defects Surveillance and Research Annual Report 2010. Rome, Italy: International Clearinghouse for Birth Defects Surveillance and Research, 2011
Canada - Alberta Congenital Anomalies Surveillance System Data 2009 - ICBDSR as it appears in International Clearinghouse for Birth Defects Surveillance and Research. International Clearinghouse for Birth Defects Surveillance and Research Annual Report 2011. Rome, Italy: International Clearinghouse for Birth Defects Surveillance and Research, 2012

Canada - Alberta Congenital Anomalies Surveillance System Data 2010 - ICBDSR as it appears in International Clearinghouse for Birth Defects Surveillance and Research. International Clearinghouse for Birth Defects Surveillance and Research Annual Report 2012. Rome, Italy: International Clearinghouse for Birth Defects Surveillance and Research, 2013

Canada - Alberta Congenital Anomalies Surveillance System Data 2012 - ICBDSR as it appears in International Clearinghouse for Birth Defects Surveillance and Research. International Clearinghouse for Birth Defects Surveillance and Research Annual Report 2014. Rome, Italy: International Clearinghouse for Birth Defects Surveillance and Research, 2016

Canada - British Columbia Health Status Registry Congenital Anomalies Surveillance Program Data 2001 - ICBDMS as it appears in International Clearinghouse for Birth Defects Monitoring Systems. International Clearinghouse for Birth Defects Monitoring Systems Annual Report 2003. Rome, Italy: International Centre on Birth Defects

Canada - British Columbia Health Status Registry Congenital Anomalies Surveillance Program Data 2002 - ICBDMS as it appears in International Clearinghouse for Birth Defects Monitoring Systems. International Clearinghouse for Birth Defects Monitoring Systems Annual Report 2004. Rome, Italy: International Centre on Birth Defects, 2006

Canada - British Columbia Health Status Registry Congenital Anomalies Surveillance Program Data 2003 - ICBDSR as it appears in International Clearinghouse for Birth Defects Surveillance and Research. International Clearinghouse for Birth Defects Surveillance and Research Annual Report 2005. Rome, Italy: International Clearinghouse for Birth Defects Surveillance and Research, 2007

Canada - British Columbia Health Status Registry Congenital Anomalies Surveillance Program Data 2004 - ICBDSR as it appears in International Clearinghouse for Birth Defects Surveillance and Research. International Clearinghouse for Birth Defects Surveillance and Research Annual Report 2006. Rome, Italy: International Clearinghouse for Birth Defects Surveillance and Research, 2007

Canada - British Columbia Health Status Registry Congenital Anomalies Surveillance Program Data 2005 - ICBDSR as it appears in International Clearinghouse for Birth Defects Surveillance and Research. International Clearinghouse for Birth Defects Surveillance and Research Annual Report 2007. Rome, Italy: International Clearinghouse for Birth Defects Surveillance and Research, 2008

Canada - British Columbia Health Status Registry Congenital Anomalies Surveillance Program Data 2007 - ICBDSR as it appears in International Clearinghouse for Birth Defects Surveillance and Research. International Clearinghouse for Birth Defects Surveillance and Research Annual Report 2009. Rome, Italy: International Clearinghouse for Birth Defects Surveillance and Research

Canada - British Columbia Health Status Registry Congenital Anomalies Surveillance Program Data 2008 - ICBDSR as it appears in International Clearinghouse for Birth Defects Surveillance and Research. International Clearinghouse for Birth Defects Surveillance and Research Annual Report 2010. Rome, Italy: International Clearinghouse for Birth Defects Surveillance and Research, 2011

Canada Congenital Anomalies Surveillance System Data 2012 - ICBDSR as it appears in International Clearinghouse for Birth Defects Surveillance and Research. International Clearinghouse for Birth Defects Surveillance and Research Annual Report 2014. Rome, Italy: International Clearinghouse for Birth Defects Surveillance and Research, 2016

Canadian Congenital Anomalies Surveillance Network Data 2002 - ICBDMS as it appears in International Clearinghouse for Birth Defects Monitoring Systems. International Clearinghouse for Birth Defects Monitoring Systems Annual Report 2004. Rome, Italy: International Centre on Birth Defects, 2006

Canadian Congenital Anomalies Surveillance Network Data 2004 - ICBDSR as it appears in International Clearinghouse for Birth Defects Surveillance and Research. International Clearinghouse for Birth Defects Surveillance and Research Annual Report 2006. Rome, Italy: International Clearinghouse for Birth Defects Surveillance and Research, 2007

Canadian Congenital Anomalies Surveillance Network Data 2005 - ICBDSR as it appears in International Clearinghouse for Birth Defects Surveillance and Research. International Clearinghouse for Birth Defects Surveillance and Research Annual Report 2007. Rome, Italy: International Clearinghouse for Birth Defects Surveillance and Research, 2008

Canadian Congenital Anomalies Surveillance Network Data 2006 - ICBDSR as it appears in International Clearinghouse for Birth Defects Surveillance and Research. International Clearinghouse for Birth Defects Surveillance and Research Annual Report 2009. Rome, Italy: International Clearinghouse for Birth Defects Surveillance and Research

Canadian Congenital Anomalies Surveillance Network Data 2007 - ICBDSR as it appears in International Clearinghouse for Birth Defects Surveillance and Research. International Clearinghouse for Birth Defects Surveillance and Research Annual Report 2010. Rome, Italy: International Clearinghouse for Birth Defects Surveillance and Research, 2011

Canadian Congenital Anomalies Surveillance System Data 1993-1997 - WHO as it appears in European Surveillance of Congenital Anomalies (EUROCAT), International Centre on Birth Defects, World Health Organization (WHO). World Atlas of Birth Defects. 2nd ed. Geneva, Switzerland: World Health Organization (WHO), 2003

Canadian Congenital Anomalies Surveillance System Data 2009 - ICBDSR as it appears in International Clearinghouse for Birth Defects Surveillance and Research. International Clearinghouse for Birth Defects Surveillance and Research Annual Report 2011. Rome, Italy: International Clearinghouse for Birth Defects Surveillance and Research, 2012

Canadian Congenital Anomalies Surveillance System Data 2010 - ICBDSR as it appears in International Clearinghouse for Birth Defects Surveillance and Research. International Clearinghouse for Birth Defects Surveillance and Research Annual Report 2012. Rome, Italy: International Clearinghouse for Birth Defects Surveillance and Research, 2013

Canaku D, Toci E, Roshi E, Burazeri G. Prevalence and factors associated with congenital malformations in Tirana, Albania, during 2011-2013. Mater Sociomed. 2014; 26(3): 158-62

Canfield MA, Collins JS, Botto LD, Williams LJ, Mai CT, Kirby RS, Pearson K, Devine O, Mulinare J, National Birth Defects Preventin Network. Changes in the birth prevalence of selected birth defects after grain fortification with folic acid in the United States: findings from a multi-state population-based study. Birth Defects Res A Clin Mol Teratol. 2005; 73(10): 679-89

Carolina Population Center, University of North Carolina at Chapel Hill, Chinese Center for Disease Control and Prevention (CCDC). China Health and Nutrition Survey. Chapel Hill, United States: Carolina Population Center, University of North Carolina at Chapel Hill

Carolina Population Center, University of North Carolina at Chapel Hill, Institute of Sociology, Russian Academy of Sciences, National Research University Higher School of Economics (Russia), ZAO Demoscope. Russia Longitudinal Monitoring Survey of HSE, Round II 1992-1993

Carolina Population Center, University of North Carolina at Chapel Hill, Institute of Sociology, Russian Academy of Sciences, National Research University Higher School of Economics (Russia), ZAO Demoscope. Russia Longitudinal Monitoring Survey of HSE, Round IV 1993-1994

Carr JA, Amato JJ, Higgins RSD. Long-term results of surgical coarctectomy in the adolescent and young adult with 18-year follow-up. Ann Thorac Surg. 2005; 79(6): 1950-56

Census and Statistics Directorate (Panama), Ministry of Economy and Finance (Panama), World Bank. Panama Living Standard Measurement Survey 2003. Washington DC, United States: World Bank

Center for Health Care Information (GYOGYINFOK) (Hungary). Hungary Hospital Inpatient Discharges 1990

Center for Health Care Information (GYOGYINFOK) (Hungary). Hungary Hospital Inpatient Discharges 1991

Center for Health Care Information (GYOGYINFOK) (Hungary). Hungary Hospital Inpatient Discharges 1992

Center for Health Care Information (GYOGYINFOK) (Hungary). Hungary Hospital Inpatient Discharges 1993
Center for Health Care Information (GYOGYINFOK) (Hungary). Hungary Hospital Inpatient Discharges 1994
Center for Health Care Information (GYOGYINFOK) (Hungary). Hungary Hospital Inpatient Discharges 1995
Center for Health Care Information (GYOGYINFOK) (Hungary). Hungary Hospital Inpatient Discharges 1996
Center for Health Care Information (GYOGYINFOK) (Hungary). Hungary Hospital Inpatient Discharges 1997
Center for Health Care Information (GYOGYINFOK) (Hungary). Hungary Hospital Inpatient Discharges 1998
Center for Health Care Information (GYOGYINFOK) (Hungary). Hungary Hospital Inpatient Discharges 1999
Center for Health Care Information (GYOGYINFOK) (Hungary). Hungary Hospital Inpatient Discharges 2000
Center for Health Care Information (GYOGYINFOK) (Hungary). Hungary Hospital Inpatient Discharges 2001
Center for Health Care Information (GYOGYINFOK) (Hungary). Hungary Hospital Inpatient Discharges 2002
Center for Health Care Information (GYOGYINFOK) (Hungary). Hungary Hospital Inpatient Discharges 2003
Center for Health Statistics and Information, National Health and Family Planning Commission (China), Shanghai Municipal Center for Disease Control and Prevention (Shanghai CDC), Shanghai Health Information Center. China Hospital Inpatient Data 2013-2016
Center for Health Statistics, Ministry of Health (Ukraine). Ukraine Inpatient Care Discharges per 100 1980
Center for Health Statistics, Ministry of Health (Ukraine). Ukraine Inpatient Care Discharges per 100 1981
Center for Health Statistics, Ministry of Health (Ukraine). Ukraine Inpatient Care Discharges per 100 1982
Center for Health Statistics, Ministry of Health (Ukraine). Ukraine Inpatient Care Discharges per 100 1983
Center for Health Statistics, Ministry of Health (Ukraine). Ukraine Inpatient Care Discharges per 100 1984
Center for Health Statistics, Ministry of Health (Ukraine). Ukraine Inpatient Care Discharges per 100 1985
Center for Health Statistics, Ministry of Health (Ukraine). Ukraine Inpatient Care Discharges per 100 1986
Center for Health Statistics, Ministry of Health (Ukraine). Ukraine Inpatient Care Discharges per 100 1987
Center for Health Statistics, Ministry of Health (Ukraine). Ukraine Inpatient Care Discharges per 100 1988
Center for Health Statistics, Ministry of Health (Ukraine). Ukraine Inpatient Care Discharges per 100 1989
Center for Health Statistics, Ministry of Health (Ukraine). Ukraine Inpatient Care Discharges per 100 1990
Center for Health Statistics, Ministry of Health (Ukraine). Ukraine Inpatient Care Discharges per 100 1991
Center for Health Statistics, Ministry of Health (Ukraine). Ukraine Inpatient Care Discharges per 100 1992
Center for Health Statistics, Ministry of Health (Ukraine). Ukraine Inpatient Care Discharges per 100 1993
Center for Health Statistics, Ministry of Health (Ukraine). Ukraine Inpatient Care Discharges per 100 1994
Center for Health Statistics, Ministry of Health (Ukraine). Ukraine Inpatient Care Discharges per 100 1995
Center for Health Statistics, Ministry of Health (Ukraine). Ukraine Inpatient Care Discharges per 100 1996
Center for Health Statistics, Ministry of Health (Ukraine). Ukraine Inpatient Care Discharges per 100 1997
Center for Health Statistics, Ministry of Health (Ukraine). Ukraine Inpatient Care Discharges per 100 1998
Center for Health Statistics, Ministry of Health (Ukraine). Ukraine Inpatient Care Discharges per 100 1999
Center for Health Statistics, Ministry of Health (Ukraine). Ukraine Inpatient Care Discharges per 100 2000
Center for Health Statistics, Ministry of Health (Ukraine). Ukraine Inpatient Care Discharges per 100 2001

Center for Health Statistics, Ministry of Health (Ukraine). Ukraine Inpatient Care Discharges per 100 2002
Center for Health Statistics, Ministry of Health (Ukraine). Ukraine Inpatient Care Discharges per 100 2003
Center for Health Statistics, Ministry of Health (Ukraine). Ukraine Inpatient Care Discharges per 100 2004
Center for Health Statistics, Ministry of Health (Ukraine). Ukraine Inpatient Care Discharges per 100 2005
Center for Health Statistics, Ministry of Health (Ukraine). Ukraine Inpatient Care Discharges per 100 2006
Center for Health Statistics, Ministry of Health (Ukraine). Ukraine Inpatient Care Discharges per 100 2007
Center for Health Statistics, Ministry of Health (Ukraine). Ukraine Inpatient Care Discharges per 100 2008
Center for Health Statistics, Ministry of Health (Ukraine). Ukraine Inpatient Care Discharges per 100 2009
Center for Health Statistics, Ministry of Health (Ukraine). Ukraine Inpatient Care Discharges per 100 2010
Center for Health Statistics, Ministry of Health (Ukraine). Ukraine Inpatient Care Discharges per 100 2011
Center for Health Statistics, Ministry of Health (Ukraine). Ukraine Inpatient Care Discharges per 100 2012
Center for Health Statistics, Ministry of Health (Ukraine). Ukraine Inpatient Care Discharges per 100 2013
Center for Research and Teaching in Economics (CIDE) (Mexico), Duke University, Ibero-American University, National Institute of Public Health (Mexico), University of California, Los Angeles (UCLA). Mexico Family Life Survey 2008-2013
Center for Research and Teaching in Economics (CIDE) (Mexico), Ibero-American University, National Institute of Perinatology (Mexico), National Institute of Statistics and Geography (INEGI) (Mexico). Mexico Family Life Survey 2002
Center for Scientific and Technological Information, Oswaldo Cruz Foundation and World Health Organization (WHO). Brazil World Health Survey 2003. Geneva, Switzerland: World Health Organization (WHO), 2005
Center for Sociological Studies, Lomonosov Moscow State University, Concluzia-Prim Center for Survey Methodology (Moldova), Institute for Advanced Studies (Austria), London School of Hygiene and Tropical Medicine, University of Aberdeen. Russia Health in Times of Transition Household Survey 2010
Center for Study of Public Opinion (Kazakhstan), Concluzia-Prim Center for Survey Methodology (Moldova), Institute for Advanced Studies (Austria), London School of Hygiene and Tropical Medicine, University of Aberdeen. Kazakhstan Health in Times of Transition Household Survey 2010
Centers for Disease Control and Prevention (CDC), Kenya Medical Research Institute (KEMRI), Kenya National Bureau of Statistics, Ministry of Public Health and Sanitation (Kenya), National AIDS Control Council (Kenya), National AIDS and STI Control Program (Kenya), National Coordinating Agency for Population and Development (Kenya), National Public Health Laboratory Services, Ministry of Public Health and Sanitation (Kenya), United States Agency for International Development (USAID). Kenya AIDS Indicator Survey 2007. Nairobi, Kenya: Kenya National Bureau of Statistics
Centers for Disease Control and Prevention (CDC), Ministry of Health (Jordan), World Health Organization (WHO). Jordan STEPS Noncommunicable Disease Risk Factors Survey 2007
Central American Population Center, University of Costa Rica. Costa Rica Survey of Family Health Services and Expenses 2008. San José, Costa Rica: Central American Population Center, University of Costa Rica
Central Bureau of Statistics (Indonesia), Ministry of Health (Indonesia), United Nations Children's Fund (UNICEF). Indonesia National Socioeconomic Survey 1995
Central Bureau of Statistics (Indonesia), Ministry of Health (Indonesia), United Nations Children's Fund (UNICEF). Indonesia National Socioeconomic Survey 1996
Central Bureau of Statistics (Indonesia), Ministry of Health (Indonesia), United Nations Children's Fund (UNICEF). Indonesia National Socioeconomic Survey 1997
Central Bureau of Statistics (Indonesia), Ministry of Health (Indonesia), World Bank. Indonesia National Socioeconomic Survey 2000
Central Bureau of Statistics (Indonesia), Ministry of Health (Indonesia), World Bank. Indonesia National Socioeconomic Survey 2001

Central Bureau of Statistics (Indonesia). Indonesia National Socioeconomic Survey 1992

Central Bureau of Statistics (Indonesia). Indonesia National Socioeconomic Survey 1993

Central Bureau of Statistics (Indonesia). Indonesia National Socioeconomic Survey 1994

Central Statistical Office (Zambia), Churches Health Association of Zambia (CHAZ), Clinton Health Access Initiative (CHAI), Institute for Health Metrics and Evaluation (IHME), Ministry of Health (Zambia), University of Zambia. Access, Bottlenecks, Costs, and Equity (ABCE) project in Zambia, 2011-2012. Seattle, United States: Institute for Health Metrics and Evaluation (IHME), 2015

Central Statistical Office (Zambia). Zambia Living Conditions Monitoring Survey 2002-2003. Lusaka, Zambia: Central Statistical Office (Zambia)

Central Statistical Office (Zambia). Zambia Living Conditions Monitoring Survey 2004-2005. Lusaka, Zambia: Central Statistical Office (Zambia)

Central Statistical Service (South Africa). South Africa October Household Survey 1994

Central Statistical Service (South Africa). South Africa October Household Survey 1996

Central Statistics Organization (Afghanistan), ICF Macro, Indian Institute of Health Management Research (IIHMR), Ministry of Public Health (Afghanistan), World Health Organization Regional Office for the Eastern Mediterranean (EMRO-WHO). Afghanistan Special Demographic and Health Survey 2010. Fairfax, United States: ICF International

Centre for Disease Prevention and Control (Latvia), Riga Stradiņš University. Latvia Health Behavior Among the Adult Population 2014

Centre for Health Promotion Studies, National University of Ireland, Galway, Health Promotion Unit, Department of Health and Children (Ireland). Ireland Survey of Lifestyle Attitudes and Nutrition 1998. Dublin, Ireland: Health Promotion Unit, Department of Health and Children (Ireland)

Chadha SL, Singh N, Shukla DK. Epidemiological study of congenital heart disease. Indian J Pediatr. 2001; 68(6): 507-10

Chang J-K, Jien W-Y, Chen H-L, Hsieh K-S. Color Doppler echocardiographic study on the incidence and natural history of early-infancy muscular ventricular septal defect. Pediatr Neonatol. 2011; 52(5): 256-60

Changlani TD, Jose A, Sudhakar A, Rojal R, Kunjikutty R, Vaidyanathan B. Outcomes of Infants with Prenatally Diagnosed Congenital Heart Disease Delivered in a Tertiary-care Pediatric Cardiac Facility. Indian Pediatr. 2015; 52(10): 852-6

Chew C, Halliday JL, Riley MM, Penny DJ. Population-based study of antenatal detection of congenital heart disease by ultrasound examination. Ultrasound Obstet Gynecol. 2007; 29(6): 619-24

Chile - Maule Registry of Congenital Malformations, Maule Health Service Data 2002 - ICBDMS as it appears in International Clearinghouse for Birth Defects Monitoring Systems. International Clearinghouse for Birth Defects Monitoring Systems Annual Report 2004. Rome, Italy: International Centre on Birth Defects, 2006

Chile - Maule Registry of Congenital Malformations, Maule Health Service Data 2003 - ICBDSR as it appears in International Clearinghouse for Birth Defects Surveillance and Research. International Clearinghouse for Birth Defects Surveillance and Research Annual Report 2005. Rome, Italy: International Clearinghouse for Birth Defects Surveillance and Research, 2007

Chile - Maule Registry of Congenital Malformations, Maule Health Service Data 2004 - ICBDSR as it appears in International Clearinghouse for Birth Defects Surveillance and Research. International Clearinghouse for Birth Defects Surveillance and Research Annual Report 2006. Rome, Italy: International Clearinghouse for Birth Defects Surveillance and Research, 2007

Chile - Maule Registry of Congenital Malformations, Maule Health Service Data 2005 - ICBDSR as it appears in International Clearinghouse for Birth Defects Surveillance and Research. International Clearinghouse for Birth Defects Surveillance and Research Annual Report 2007. Rome, Italy: International Clearinghouse for Birth Defects Surveillance and Research, 2008

Chile - Maule Registry of Congenital Malformations, Maule Health Service Data 2007 - ICBDSR as it appears in International Clearinghouse for Birth Defects Surveillance and Research. International Clearinghouse for Birth Defects Surveillance and Research Annual Report 2009. Rome, Italy: International Clearinghouse for Birth Defects Surveillance and Research

Chile - Maule Registry of Congenital Malformations, Maule Health Service Data 2008 - ICBDSR as it appears in International Clearinghouse for Birth Defects Surveillance and Research. International Clearinghouse for Birth Defects Surveillance and Research Annual Report 2010. Rome, Italy: International Clearinghouse for Birth Defects Surveillance and Research, 2011

Chile - Maule Registry of Congenital Malformations, Maule Health Service Data 2009 - ICBDSR as it appears in International Clearinghouse for Birth Defects Surveillance and Research. International Clearinghouse for Birth Defects Surveillance and Research Annual Report 2011. Rome, Italy: International Clearinghouse for Birth Defects Surveillance and Research, 2012

Chile - Maule Registry of Congenital Malformations, Maule Health Service Data 2010 - ICBDSR as it appears in International Clearinghouse for Birth Defects Surveillance and Research. International Clearinghouse for Birth Defects Surveillance and Research Annual Report 2012. Rome, Italy: International Clearinghouse for Birth Defects Surveillance and Research, 2013

Chile Latin American Collaborative Study of Congenital Malformations Data 1993-1998 - WHO as it appears in European Surveillance of Congenital Anomalies (EUROCAT), International Centre on Birth Defects, World Health Organization (WHO). World Atlas of Birth Defects. 2nd ed. Geneva, Switzerland: World Health Organization (WHO), 2003

China Beijing Birth Defect Surveillance System in Thirty Counties of Four Provinces Data 2001 - ICBDMS as it appears in International Clearinghouse for Birth Defects Monitoring Systems. International Clearinghouse for Birth Defects Monitoring Systems Annual Report 2003. Rome, Italy: International Centre on Birth Defects

China Beijing Birth Defect Surveillance System in Thirty Counties of Four Provinces Data 2003 - ICBDSR as it appears in International Clearinghouse for Birth Defects Surveillance and Research. International Clearinghouse for Birth Defects Surveillance and Research Annual Report 2005. Rome, Italy: International Clearinghouse for Birth Defects Surveillance and Research, 2007

China Beijing Birth Defect Surveillance System in Thirty Counties of Four Provinces Data 2004 - ICBDSR as it appears in International Clearinghouse for Birth Defects Surveillance and Research. International Clearinghouse for Birth Defects Surveillance and Research Annual Report 2006. Rome, Italy: International Clearinghouse for Birth Defects Surveillance and Research, 2007

China Center for Economic Research, Peking University. China Health and Retirement Longitudinal Study Pilot Resurvey 2012. Beijing, China: China Center for Economic Research, Peking University

China National Maternal and Child Health Surveillance System Congenital Anomalies 1996-2012 - MCHS

Chinese Birth Defects Monitoring Network Data 2001 - ICBDMS as it appears in International Clearinghouse for Birth Defects Monitoring Systems. International Clearinghouse for Birth Defects Monitoring Systems Annual Report 2003. Rome, Italy: International Centre on Birth Defects

Chinese Birth Defects Monitoring Network Data 2003 - ICBDSR as it appears in International Clearinghouse for Birth Defects Surveillance and Research. International Clearinghouse for Birth Defects Surveillance and Research Annual Report 2005. Rome, Italy: International Clearinghouse for Birth Defects Surveillance and Research, 2007

Chinese Birth Defects Monitoring Network Data 2004 - ICBDSR as it appears in International Clearinghouse for Birth Defects Surveillance and Research. International Clearinghouse for Birth Defects Surveillance and Research Annual Report 2006. Rome, Italy: International Clearinghouse for Birth Defects Surveillance and Research, 2007

Chiu S-N, Wang J-K, Chen H-C, Lin M-T, Wu E-T, Chen C-A, Huang S-C, Chang C-I, Chen Y-S, Chiu I-S, Chen C-L, Wu M-H. Long-term survival and unnatural deaths of patients with repaired tetralogy of Fallot in an Asian cohort. Circ Cardiovasc Qual Outcomes. 2012; 5(1): 120-5

Chong A-Y, Rajaratnam R, Hussein NR, Lip GYH. Heart failure in a multiethnic population in Kuala Lumpur, Malaysia. Eur J Heart Fail. 2003; 5(4): 569-74

Chung ML, Lee BS, Kim EA-R, Kim K-S, Pi S-Y, Oh YM, Park IS, Seo DM, Won HS. Impact of fetal echocardiography on trends in disease patterns and outcomes of congenital heart disease in a neonatal intensive care unit. Neonatology. 2010; 98(1): 41-6

Cleves MA, Ghaffar S, Zhao W, Mosley BS, Hobbs CA. First-year survival of infants born with congenital heart defects in Arkansas (1993-1998): a survival analysis using registry data. Birth Defects Res A Clin Mol Teratol. 2003; 67(9): 662-8

Colombia - Bogota Congenital Malformations Surveillance Program Data 2009 - ICBDSR as it appears in International Clearinghouse for Birth Defects Surveillance and Research. International Clearinghouse for Birth Defects Surveillance and Research Annual Report 2011. Rome, Italy: International Clearinghouse for Birth Defects Surveillance and Research, 2012

Colombia - Bogota Congenital Malformations Surveillance Program Data 2010 - ICBDSR as it appears in International Clearinghouse for Birth Defects Surveillance and Research. International Clearinghouse for Birth Defects Surveillance and Research Annual Report 2012. Rome, Italy: International Clearinghouse for Birth Defects Surveillance and Research, 2013

Colombia Latin American Collaborative Study of Congenital Malformations Data 1993-1994 - WHO as it appears in European Surveillance of Congenital Anomalies (EUROCAT), International Centre on Birth Defects, World Health Organization (WHO). World Atlas of Birth Defects. 2nd ed. Geneva, Switzerland: World Health Organization (WHO), 2003

Concluzia-Prim Center for Survey Methodology (Moldova), East-Ukranian Foundation For Social Research, Institute for Advanced Studies (Austria), London School of Hygiene and Tropical Medicine, University of Aberdeen. Ukraine Health in Times of Transition Household Survey 2010

Concluzia-Prim Center for Survey Methodology (Moldova), Georgia Opinion Research Business International (GORBI), Institute for Advanced Studies (Austria), London School of Hygiene and Tropical Medicine, University of Aberdeen. Georgia Health in Times of Transition Household Survey 2010

Concluzia-Prim Center for Survey Methodology (Moldova), Independent Sociology and Information Service (OPINIA) (Moldova), Institute for Advanced Studies (Austria), London School of Hygiene and Tropical Medicine, University of Aberdeen. Moldova Health in Times of Transition Household Survey 2010

Concluzia-Prim Center for Survey Methodology (Moldova), Institute for Advanced Studies (Austria), International Centre for Sociological, Political and Social Psychological Research (Kyrgyzstan), London School of Hygiene and Tropical Medicine, University of Aberdeen. Kyrgyzstan Health in Times of Transition Household Survey 2011

Concluzia-Prim Center for Survey Methodology (Moldova), Institute for Advanced Studies (Austria), London School of Hygiene and Tropical Medicine, SIAR Research and Consulting (Azerbaijan), University of Aberdeen. Azerbaijan Health in Times of Transition Household Survey 2010

Copel JA, Tan AS, Kleinman CS. Does a prenatal diagnosis of congenital heart disease alter short-term outcome?. Ultrasound Obstet Gynecol. 1997; 10(4): 237-41

Costa Rica Birth Defects Register Center Data 2012 - ICBDSR as it appears in International Clearinghouse for Birth Defects Surveillance and Research. International Clearinghouse for Birth Defects Surveillance and Research Annual Report 2014. Rome, Italy: International Clearinghouse for Birth Defects Surveillance and Research, 2016

Costa Rican Birth Defects Register Center Data 2002 - ICBDMS as it appears in International Clearinghouse for Birth Defects Monitoring Systems. International Clearinghouse for Birth Defects Monitoring Systems Annual Report 2004. Rome, Italy: International Centre on Birth Defects, 2006

Costa Rican Birth Defects Register Center Data 2004 - ICBDSR as it appears in International Clearinghouse for Birth Defects Surveillance and Research. International Clearinghouse for Birth Defects Surveillance and Research Annual Report 2006. Rome, Italy: International Clearinghouse for Birth Defects Surveillance and Research, 2007

Costa Rican Birth Defects Register Center Data 2005 - ICBDSR as it appears in International Clearinghouse for Birth Defects Surveillance and Research. International Clearinghouse for Birth Defects Surveillance and Research Annual Report 2007. Rome, Italy: International Clearinghouse for Birth Defects Surveillance and Research, 2008

Costa Rican Birth Defects Register Center Data 2009 - ICBDSR as it appears in International Clearinghouse for Birth Defects Surveillance and Research. International Clearinghouse for Birth Defects Surveillance and Research Annual Report 2011. Rome, Italy: International Clearinghouse for Birth Defects Surveillance and Research, 2012

Costa Rican Birth Defects Register Center Data 2010 - ICBDSR as it appears in International Clearinghouse for Birth Defects Surveillance and Research. International Clearinghouse for Birth Defects Surveillance and Research Annual Report 2012. Rome, Italy: International Clearinghouse for Birth Defects Surveillance and Research, 2013

Costa Rican Register of Congenital Malformation Data 2008 - ICBDSR as it appears in International Clearinghouse for Birth Defects Surveillance and Research. International Clearinghouse for Birth Defects Surveillance and Research Annual Report 2010. Rome, Italy: International Clearinghouse for Birth Defects Surveillance and Research, 2011

Croatia - Zagreb Congenital Anomaly Data 1993-1997 - WHO as it appears in European Surveillance of Congenital Anomalies (EUROCAT), International Centre on Birth Defects, World Health Organization (WHO). World Atlas of Birth Defects. 2nd ed. Geneva, Switzerland: World Health Organization (WHO), 2003

Cuban Register of Congenital Malformation Data 1993-1998 - WHO as it appears in European Surveillance of Congenital Anomalies (EUROCAT), International Centre on Birth Defects, World Health Organization (WHO). World Atlas of Birth Defects. 2nd ed. Geneva, Switzerland: World Health Organization (WHO), 2003

Cuban Register of Congenital Malformation Data 2002 - ICBDMS as it appears in International Clearinghouse for Birth Defects Monitoring Systems. International Clearinghouse for Birth Defects Monitoring Systems Annual Report 2004. Rome, Italy: International Centre on Birth Defects, 2006

Cuban Register of Congenital Malformation Data 2003 - ICBDSR as it appears in International Clearinghouse for Birth Defects Surveillance and Research. International Clearinghouse for Birth Defects Surveillance and Research Annual Report 2005. Rome, Italy: International Clearinghouse for Birth Defects Surveillance and Research, 2007

Cuban Register of Congenital Malformation Data 2004 - ICBDSR as it appears in International Clearinghouse for Birth Defects Surveillance and Research. International Clearinghouse for Birth Defects Surveillance and Research Annual Report 2006. Rome, Italy: International Clearinghouse for Birth Defects Surveillance and Research, 2007

Cuban Register of Congenital Malformation Data 2005 - ICBDSR as it appears in International Clearinghouse for Birth Defects Surveillance and Research. International Clearinghouse for Birth Defects Surveillance and Research Annual Report 2007. Rome, Italy: International Clearinghouse for Birth Defects Surveillance and Research, 2008

Cuban Register of Congenital Malformation Data 2007 - ICBDSR as it appears in International Clearinghouse for Birth Defects Surveillance and Research. International Clearinghouse for Birth Defects Surveillance and Research Annual Report 2009. Rome, Italy: International Clearinghouse for Birth Defects Surveillance and Research

Cuban Register of Congenital Malformation Data 2008 - ICBDSR as it appears in International Clearinghouse for Birth Defects Surveillance and Research. International Clearinghouse for Birth Defects Surveillance and Research Annual Report 2010. Rome, Italy: International Clearinghouse for Birth Defects Surveillance and Research, 2011

Cuban Register of Congenital Malformation Data 2009 - ICBDSR as it appears in International Clearinghouse for Birth Defects Surveillance and Research. International Clearinghouse for Birth Defects Surveillance and Research Annual Report 2011. Rome, Italy: International Clearinghouse for Birth Defects Surveillance and Research, 2012
Cuban Register of Congenital Malformation Data 2010 - ICBDSR as it appears in International Clearinghouse for Birth Defects Surveillance and Research. International Clearinghouse for Birth Defects Surveillance and Research Annual Report 2012. Rome, Italy: International Clearinghouse for Birth Defects Surveillance and Research, 2013
Czech Republic Congenital Malformations Monitoring Program Data 1993-1998 - WHO as it appears in European Surveillance of Congenital Anomalies (EUROCAT), International Centre on Birth Defects, World Health Organization (WHO). World Atlas of Birth Defects. 2nd ed. Geneva, Switzerland: World Health Organization (WHO), 2003
Czech Republic Congenital Malformations Monitoring Program Data 2001 - ICBDMS as it appears in International Clearinghouse for Birth Defects Monitoring Systems. International Clearinghouse for Birth Defects Monitoring Systems Annual Report 2003. Rome, Italy: International Centre on Birth Defects
Czech Republic Congenital Malformations Monitoring Program Data 2002 - ICBDMS as it appears in International Clearinghouse for Birth Defects Monitoring Systems. International Clearinghouse for Birth Defects Monitoring Systems Annual Report 2004. Rome, Italy: International Centre on Birth Defects, 2006
Czech Republic Congenital Malformations Monitoring Program Data 2003 - ICBDSR as it appears in International Clearinghouse for Birth Defects Surveillance and Research. International Clearinghouse for Birth Defects Surveillance and Research Annual Report 2005. Rome, Italy: International Clearinghouse for Birth Defects Surveillance and Research, 2007
Czech Republic Congenital Malformations Monitoring Program Data 2004 - ICBDSR as it appears in International Clearinghouse for Birth Defects Surveillance and Research. International Clearinghouse for Birth Defects Surveillance and Research Annual Report 2006. Rome, Italy: International Clearinghouse for Birth Defects Surveillance and Research, 2007
Czech Republic Congenital Malformations Monitoring Program Data 2005 - ICBDSR as it appears in International Clearinghouse for Birth Defects Surveillance and Research. International Clearinghouse for Birth Defects Surveillance and Research Annual Report 2007. Rome, Italy: International Clearinghouse for Birth Defects Surveillance and Research, 2008
Czech Republic Congenital Malformations Monitoring Program Data 2007 - ICBDSR as it appears in International Clearinghouse for Birth Defects Surveillance and Research. International Clearinghouse for Birth Defects Surveillance and Research Annual Report 2009. Rome, Italy: International Clearinghouse for Birth Defects Surveillance and Research
Czech Republic Congenital Malformations Monitoring Program Data 2008 - ICBDSR as it appears in International Clearinghouse for Birth Defects Surveillance and Research. International Clearinghouse for Birth Defects Surveillance and Research Annual Report 2010. Rome, Italy: International Clearinghouse for Birth Defects Surveillance and Research, 2011
Czech Republic National Registry of Congenital Anomalies Data 2009 - ICBDSR as it appears in International Clearinghouse for Birth Defects Surveillance and Research. International Clearinghouse for Birth Defects Surveillance and Research Annual Report 2011. Rome, Italy: International Clearinghouse for Birth Defects Surveillance and Research, 2012
Czech Republic National Registry of Congenital Anomalies Data 2010 - ICBDSR as it appears in International Clearinghouse for Birth Defects Surveillance and Research. International Clearinghouse for Birth Defects Surveillance and Research Annual Report 2012. Rome, Italy: International Clearinghouse for Birth Defects Surveillance and Research, 2013

Czech Republic National Registry of Congenital Anomalies Data 2012 - ICBDSR as it appears in International Clearinghouse for Birth Defects Surveillance and Research. International Clearinghouse for Birth Defects Surveillance and Research Annual Report 2014. Rome, Italy: International Clearinghouse for Birth Defects Surveillance and Research, 2016

Damasceno A, Mayosi BM, Sani M, Ogah OS, Mondo C, Ojji D, Dzudie A, Kouam CK, Suliman A, Schrueder N, Yonga G, Ba SA, Maru F, Alemayehu B, Edwards C, Davison BA, Cotter G, Sliwa K. The causes, treatment, and outcome of acute heart failure in 1006 Africans from 9 countries. Arch Intern Med. 2012; 172(18): 1386-94

Danish Health and Medicines Authority. Denmark National Patient Registry 1979

Danish Health and Medicines Authority. Denmark National Patient Registry 1980

Danish Health and Medicines Authority. Denmark National Patient Registry 1981

Danish Health and Medicines Authority. Denmark National Patient Registry 1982

Danish Health and Medicines Authority. Denmark National Patient Registry 1983

Danish Health and Medicines Authority. Denmark National Patient Registry 1984

Danish Health and Medicines Authority. Denmark National Patient Registry 1985

Danish Health and Medicines Authority. Denmark National Patient Registry 1986

Danish Health and Medicines Authority. Denmark National Patient Registry 1987

Danish Health and Medicines Authority. Denmark National Patient Registry 1988

Danish Health and Medicines Authority. Denmark National Patient Registry 1989

Danish Health and Medicines Authority. Denmark National Patient Registry 1990

Danish Health and Medicines Authority. Denmark National Patient Registry 1991

Danish Health and Medicines Authority. Denmark National Patient Registry 1992

Danish Health and Medicines Authority. Denmark National Patient Registry 1993

Danish Health and Medicines Authority. Denmark National Patient Registry 1994

Danish Health and Medicines Authority. Denmark National Patient Registry 1995

Danish Health and Medicines Authority. Denmark National Patient Registry 1996

Danish Health and Medicines Authority. Denmark National Patient Registry 1997

Danish Health and Medicines Authority. Denmark National Patient Registry 1998

Danish Health and Medicines Authority. Denmark National Patient Registry 1999

Danish Health and Medicines Authority. Denmark National Patient Registry 2000

Danish Health and Medicines Authority. Denmark National Patient Registry 2001

Danish Health and Medicines Authority. Denmark National Patient Registry 2002

Danish Health and Medicines Authority. Denmark National Patient Registry 2003

Danish Health and Medicines Authority. Denmark National Patient Registry 2004

Danish Health and Medicines Authority. Denmark National Patient Registry 2005

Danish Health and Medicines Authority. Denmark National Patient Registry 2006

Danish Health and Medicines Authority. Denmark National Patient Registry 2007

Danish Health and Medicines Authority. Denmark National Patient Registry 2008

Danish Health and Medicines Authority. Denmark National Patient Registry 2009

Danish Health and Medicines Authority. Denmark National Patient Registry 2010

Danish Health and Medicines Authority. Denmark National Patient Registry 2011

Danish Health and Medicines Authority. Denmark National Patient Registry 2012

Danish Health and Medicines Authority. Denmark National Patient Registry 2013

Dastgiri S, Imani S, Kalankesh L, Barzegar M, Heidarzadeh M. Congenital anomalies in Iran: a cross-sectional study on 1574 cases in the North-West of country. Child Care Health Dev. 2007; 33(3): 257-61

Dearani JA, Danielson GK, Puga FJ, Schaff HV, Warnes CW, Driscoll DJ, Schleck CD, Ilstrup DM. Late follow-up of 1095 patients undergoing operation for complex congenital heart disease utilizing pulmonary ventricle to pulmonary artery conduits. Ann Thorac Surg. 2003; 75(2): 399-411

Debost-Legrand A, Ouchchane L, Francannet C, Goumy C, Perthus I, Beaufrere AM, Gallot D, Lemery D, Lusson JR, Laurichesse-Delmas H. Impact of prenatal diagnosis on the outcome of patients with a transposition of great arteries: A 24-year population-based study. Birth Defects Res A Clin Mol Teratol. 2016; 106(3): 178-84

Denmark - Funen Registry of Funen County Congenital Anomaly Data 1993-1998 - WHO as it appears in European Surveillance of Congenital Anomalies (EUROCAT), International Centre on Birth Defects, World Health Organization (WHO). World Atlas of Birth Defects. 2nd ed. Geneva, Switzerland: World Health Organization (WHO), 2003

Department of Economics, University of Chile, Ministry of Planning (Chile). Chile National Socioeconomic Characterization Survey 1992. Santiago, Chile: Ministry of Social Development (Chile)

Department of Economics, University of Chile, Ministry of Planning (Chile). Chile National Socioeconomic Characterization Survey 1994. Santiago, Chile: Ministry of Social Development (Chile)

Department of Economics, University of Chile, Ministry of Planning (Chile). Chile National Socioeconomic Characterization Survey 1996. Santiago, Chile: Ministry of Social Development (Chile)

Department of Economics, University of Chile, Ministry of Planning (Chile). Chile National Socioeconomic Characterization Survey 1998. Santiago, Chile: Ministry of Social Development (Chile)

Department of Economics, University of Chile, Ministry of Planning (Chile). Chile National Socioeconomic Characterization Survey 2000. Santiago, Chile: Ministry of Social Development (Chile)

Department of Economics, University of Chile, Ministry of Planning (Chile). Chile National Socioeconomic Characterization Survey 2003. Santiago, Chile: Ministry of Social Development (Chile)

Department of Economics, University of Chile, Ministry of Planning (Chile). Chile National Socioeconomic Characterization Survey 2006. Santiago, Chile: Ministry of Social Development (Chile)

Department of Health (Ireland), Economic and Social Research Institute (ESRI) (Ireland). Ireland Hospital Inpatient Enquiry 1980

Department of Health (Ireland), Economic and Social Research Institute (ESRI) (Ireland). Ireland Hospital Inpatient Enquiry 1981

Department of Health (Ireland), Economic and Social Research Institute (ESRI) (Ireland). Ireland Hospital Inpatient Enquiry 1982

Department of Health (Ireland), Economic and Social Research Institute (ESRI) (Ireland). Ireland Hospital Inpatient Enquiry 1983

Department of Health (Ireland), Economic and Social Research Institute (ESRI) (Ireland). Ireland Hospital Inpatient Enquiry 1984

Department of Health (Ireland), Economic and Social Research Institute (ESRI) (Ireland). Ireland Hospital Inpatient Enquiry 1985

Department of Health (Ireland), Economic and Social Research Institute (ESRI) (Ireland). Ireland Hospital Inpatient Enquiry 1986

Department of Health (Ireland), Economic and Social Research Institute (ESRI) (Ireland). Ireland Hospital Inpatient Enquiry 1987

Department of Health (Ireland), Economic and Social Research Institute (ESRI) (Ireland). Ireland Hospital Inpatient Enquiry 1988

Department of Health (Ireland), Economic and Social Research Institute (ESRI) (Ireland). Ireland Hospital Inpatient Enquiry 1989

Department of Health (Ireland), Economic and Social Research Institute (ESRI) (Ireland). Ireland Hospital Inpatient Enquiry 1990

Department of Health (Ireland), Economic and Social Research Institute (ESRI) (Ireland). Ireland Hospital Inpatient Enquiry 1991

Department of Health (Ireland), Economic and Social Research Institute (ESRI) (Ireland). Ireland Hospital Inpatient Enquiry 1992

Department of Health (Ireland), Economic and Social Research Institute (ESRI) (Ireland). Ireland Hospital Inpatient Enquiry 1993

Department of Health (Ireland), Economic and Social Research Institute (ESRI) (Ireland). Ireland Hospital Inpatient Enquiry 1994

Department of Health (Ireland), Economic and Social Research Institute (ESRI) (Ireland). Ireland Hospital Inpatient Enquiry 1995

Department of Health (Ireland), Economic and Social Research Institute (ESRI) (Ireland). Ireland Hospital Inpatient Enquiry 1996

Department of Health and Children (Ireland), Economic and Social Research Institute (ESRI) (Ireland). Ireland Hospital Inpatient Enquiry 1997

Department of Health and Children (Ireland), Economic and Social Research Institute (ESRI) (Ireland). Ireland Hospital Inpatient Enquiry 1998

Department of Health and Children (Ireland), Economic and Social Research Institute (ESRI) (Ireland). Ireland Hospital Inpatient Enquiry 1999

Department of Health and Children (Ireland), Economic and Social Research Institute (ESRI) (Ireland). Ireland Hospital Inpatient Enquiry 2000

Department of Health Services, Ministry of Health and Population (Nepal). Nepal Hospital Inpatient Discharges 2010-2012

Department of Health Services, Ministry of Health and Population (Nepal). Nepal Hospital Inpatient Discharges 2013-2014

Department of Health, Social Services and Public Safety (Northern Ireland), Information Centre for Health and Social Care, NHS, NHS England, NHS Health Scotland, NHS Wales. United Kingdom Hospital Patient and Discharge Data 2006

Department of Health, Social Services and Public Safety (Northern Ireland), Information Centre for Health and Social Care, NHS, NHS England, NHS Health Scotland, NHS Wales. United Kingdom Hospital Patient and Discharge Data 2012

Department of Health, Social Services and Public Safety (Northern Ireland), Information Centre for Health and Social Care, NHS, NHS England, NHS Health Scotland, NHS Wales. United Kingdom Hospital Patient and Discharge Data 2013

Department of Health, Social Services and Public Safety (Northern Ireland), Information Centre for Health and Social Care, NHS, NHS England, NHS Health Scotland, NHS Wales. United Kingdom Hospital Patient and Discharge Data 2014

Dhanardhono T, Thia E, Wei X, Saktini F, Dewi PK, Yeo GSH. Incidence and outcome of prenatally diagnosed, chromosomally normal congenital heart defects in Singapore. Singapore Med J. 2012; 53(10): 643-7

Directorate of Health (Iceland). Iceland Hospital Data Registry 1988

Directorate of Health (Iceland). Iceland Hospital Data Registry 1989

Directorate of Health (Iceland). Iceland Hospital Data Registry 1990

Directorate of Health (Iceland). Iceland Hospital Data Registry 1991

Directorate of Health (Iceland). Iceland Hospital Data Registry 1992

Directorate of Health (Iceland). Iceland Hospital Data Registry 1993

Directorate of Health (Iceland). Iceland Hospital Data Registry 1994

Directorate of Health (Iceland). Iceland Hospital Data Registry 1995

Directorate of Health (Iceland). Iceland Hospital Data Registry 1999

Directorate of Health (Iceland). Iceland Hospital Data Registry 2008
Directorate of Health (Iceland). Iceland Hospital Data Registry 2010
Directorate of Health (Iceland). Iceland Hospital Data Registry 2011
Directorate of Health (Iceland). Iceland Hospital Data Registry 2012
Directorate of Health (Iceland). Iceland Hospital Data Registry 2013
Directorate of Health (Iceland). Iceland Hospital Data Registry 2014
Directorate of Statistics of the High Commission for Planning (Morocco), World Bank. Morocco Living Standards Measurement Survey 1990-1991
Dutch Hospital Data (DHD). Netherlands National Medical Registry 1990
Dutch Hospital Data (DHD). Netherlands National Medical Registry 1991
Dutch Hospital Data (DHD). Netherlands National Medical Registry 1992
Dutch Hospital Data (DHD). Netherlands National Medical Registry 1993
Dutch Hospital Data (DHD). Netherlands National Medical Registry 1994
Dutch Hospital Data (DHD). Netherlands National Medical Registry 1995
Dutch Hospital Data (DHD). Netherlands National Medical Registry 1996
Dutch Hospital Data (DHD). Netherlands National Medical Registry 1997
Dutch Hospital Data (DHD). Netherlands National Medical Registry 1998
Dutch Hospital Data (DHD). Netherlands National Medical Registry 1999
Dutch Hospital Data (DHD). Netherlands National Medical Registry 2000
Dutch Hospital Data (DHD). Netherlands National Medical Registry 2001
Dutch Hospital Data (DHD). Netherlands National Medical Registry 2002
Dutch Hospital Data (DHD). Netherlands National Medical Registry 2003
Dutch Hospital Data (DHD). Netherlands National Medical Registry 2004
Dutch Hospital Data (DHD). Netherlands National Medical Registry 2006
Dutch Hospital Data (DHD). Netherlands National Medical Registry 2007
Dutch Hospital Data (DHD). Netherlands National Medical Registry 2010
Economic and Social Research Institute (ESRI) (Ireland), Health Service Executive (HSE) (Ireland). Ireland Hospital Inpatient Enquiry 2013
Economic and Social Research Institute (ESRI) (Ireland), Health Service Executive (HSE) (Ireland). Ireland Hospital Inpatient Enquiry 2014
Ecuador Congenital Anomaly Data 1993-1998 - WHO as it appears in European Surveillance of Congenital Anomalies (EUROCAT), International Centre on Birth Defects, World Health Organization (WHO). World Atlas of Birth Defects. 2nd ed. Geneva, Switzerland: World Health Organization (WHO), 2003
Egbe A, Lee S, Ho D, Uppu S, Srivastava S. Racial/ethnic differences in the birth prevalence of congenital anomalies in the United States. J Perinat Med. 2015; 43(1): 111-7
Environics Research Group, World Health Organization (WHO). Canada WHO Multi-country Survey Study on Health and Health System Responsiveness 2000-2001. Geneva, Switzerland: World Health Organization (WHO)
Erik Consulting, International Research Associates (INRA) Europe, World Health Organization (WHO). France WHO Multi-country Survey Study on Health and Health System Responsiveness 2000-2001. Geneva, Switzerland: World Health Organization (WHO)

European Surveillance of Congenital Anomalies (EUROCAT), International Centre on Birth Defects, World Health Organization (WHO). World Atlas of Birth Defects. 2nd ed. Geneva, Switzerland: World Health Organization (WHO), 2003
European Surveillance of Congenital Anomalies (EUROCAT), University of Ulster. Belgium EUROCAT Prevalence Tables, Individual Registries and Countries. Newtownabbey, Northern Ireland: European Surveillance of Congenital Anomalies (EUROCAT)
European Surveillance of Congenital Anomalies (EUROCAT). Austria EUROCAT Prevalence Tables, Individual Registries and Countries. Newtownabbey, Northern Ireland: European Surveillance of Congenital Anomalies (EUROCAT)
European Surveillance of Congenital Anomalies (EUROCAT). Bulgaria EUROCAT Prevalence Tables, Individual Registries and Countries. Newtownabbey, Northern Ireland: European Surveillance of Congenital Anomalies (EUROCAT)
European Surveillance of Congenital Anomalies (EUROCAT). Croatia EUROCAT Prevalence Tables, Individual Registries and Countries. Newtownabbey, Northern Ireland: European Surveillance of Congenital Anomalies (EUROCAT)
European Surveillance of Congenital Anomalies (EUROCAT). Czech Republic EUROCAT Prevalence Tables 2000-2010. Ispra, Italy: European Surveillance of Congenital Anomalies (EUROCAT)
European Surveillance of Congenital Anomalies (EUROCAT). Denmark EUROCAT Prevalence Tables, Individual Registries and Countries. Newtownabbey, Northern Ireland: European Surveillance of Congenital Anomalies (EUROCAT)
European Surveillance of Congenital Anomalies (EUROCAT). Finland EUROCAT Prevalence Tables. Ispra, Italy: European Surveillance of Congenital Anomalies (EUROCAT)
European Surveillance of Congenital Anomalies (EUROCAT). France EUROCAT Prevalence Tables, Individual Registries and Countries. Newtownabbey, Northern Ireland: European Surveillance of Congenital Anomalies (EUROCAT)
European Surveillance of Congenital Anomalies (EUROCAT). Germany EUROCAT Prevalence Tables, Individual Registries and Countries. Newtownabbey, Northern Ireland: European Surveillance of Congenital Anomalies (EUROCAT)
European Surveillance of Congenital Anomalies (EUROCAT). Hungary EUROCAT Prevalence Tables, Individual Registries and Countries. Newtownabbey, Northern Ireland: European Surveillance of Congenital Anomalies (EUROCAT)
European Surveillance of Congenital Anomalies (EUROCAT). Ireland EUROCAT Prevalence Tables, Individual Registries and Countries. Newtownabbey, Northern Ireland: European Surveillance of Congenital Anomalies (EUROCAT)
European Surveillance of Congenital Anomalies (EUROCAT). Italy EUROCAT Prevalence Tables, Individual Registries and Countries. Newtownabbey, Northern Ireland: European Surveillance of Congenital Anomalies (EUROCAT)
European Surveillance of Congenital Anomalies (EUROCAT). Malta EUROCAT Prevalence Tables, Individual Registries and Countries. Newtownabbey, Northern Ireland: European Surveillance of Congenital Anomalies (EUROCAT)
European Surveillance of Congenital Anomalies (EUROCAT). Netherlands EUROCAT Prevalence Tables, Individual Registries and Countries. Newtownabbey, Northern Ireland: European Surveillance of Congenital Anomalies (EUROCAT)
European Surveillance of Congenital Anomalies (EUROCAT). Norway EUROCAT Prevalence Tables, Individual Registries and Countries. Newtownabbey, Northern Ireland: European Surveillance of Congenital Anomalies (EUROCAT)
European Surveillance of Congenital Anomalies (EUROCAT). Poland EUROCAT Prevalence Tables, Individual Registries and Countries. Newtownabbey, Northern Ireland: European Surveillance of Congenital Anomalies (EUROCAT)

European Surveillance of Congenital Anomalies (EUROCAT). Portugal EUROCAT Prevalence Tables, Individual Registries and Countries. Newtownabbey, Northern Ireland: European Surveillance of Congenital Anomalies (EUROCAT)

European Surveillance of Congenital Anomalies (EUROCAT). Spain EUROCAT Prevalence Tables, Individual Registries and Countries. Newtownabbey, Northern Ireland: European Surveillance of Congenital Anomalies (EUROCAT)

European Surveillance of Congenital Anomalies (EUROCAT). Sweden EUROCAT Prevalence Tables. Ispra, Italy: European Surveillance of Congenital Anomalies (EUROCAT)

European Surveillance of Congenital Anomalies (EUROCAT). Switzerland EUROCAT Prevalence Tables, Individual Registries and Countries. Newtownabbey, Northern Ireland: European Surveillance of Congenital Anomalies (EUROCAT)

European Surveillance of Congenital Anomalies (EUROCAT). Ukraine EUROCAT Prevalence Tables, Individual Registries and Countries. Newtownabbey, Northern Ireland: European Surveillance of Congenital Anomalies (EUROCAT)

European Surveillance of Congenital Anomalies (EUROCAT). United Kingdom EUROCAT Prevalence Tables, Individual Registries and Countries. Newtownabbey, Northern Ireland: European Surveillance of Congenital Anomalies (EUROCAT)

Family Health International, Ministry of Health (Indonesia), National AIDS Commission (KPA), Statistics Indonesia. Indonesia Behavioral Surveillance Survey 2007

Federal Environment Agency (Germany), Federal Institute for Drugs and Medical Devices (Germany), Max Planck Institute of Psychiatry, Robert Koch Institute. Germany National Health Interview and Examination Survey 1997-1999. Berlin, Germany: Robert Koch Institute, 2000

Federal Ministry of Health (Austria), Statistics Austria. Austria Hospital Inpatient Discharges 1989. Vienna, Austria: Statistics Austria

Federal Ministry of Health (Austria), Statistics Austria. Austria Hospital Inpatient Discharges 1989-1992

Federal Ministry of Health (Austria), Statistics Austria. Austria Hospital Inpatient Discharges 1993-1997

Federal Ministry of Health (Austria), Statistics Austria. Austria Hospital Inpatient Discharges 1998-2002

Federal Ministry of Health (Austria), Statistics Austria. Austria Hospital Inpatient Discharges 2003-2007

Federal Ministry of Health (Austria), Statistics Austria. Austria Hospital Inpatient Discharges 2008-2012

Federal Ministry of Health (Austria), Statistics Austria. Austria Hospital Inpatient Discharges 2013. Vienna, Austria: Statistics Austria

Federal Ministry of Health (Austria), Statistics Austria. Austria Hospital Inpatient Discharges 2013-2014

Federal Ministry of Health (Austria), Statistics Austria. Austria Hospital Inpatient Discharges 2014. Vienna, Austria: Statistics Austria

Federal Public Service Health, Food Chain Safety, and Environment (Belgium). Belgium Minimum Clinical Summary 2000

Federal Public Service Health, Food Chain Safety, and Environment (Belgium). Belgium Minimum Clinical Summary 2001

Federal Public Service Health, Food Chain Safety, and Environment (Belgium). Belgium Minimum Clinical Summary 2002

Federal Public Service Health, Food Chain Safety, and Environment (Belgium). Belgium Minimum Clinical Summary 2005

Federal Public Service Health, Food Chain Safety, and Environment (Belgium). Belgium Minimum Clinical Summary 2006

Federal Public Service Health, Food Chain Safety, and Environment (Belgium). Belgium Minimum Clinical Summary 2007

Federal Public Service Health, Food Chain Safety, and Environment (Belgium). Belgium Minimum Hospital Summary 2010

Federal Public Service Health, Food Chain Safety, and Environment (Belgium). Belgium Minimum Hospital Summary 2011

Federal Public Service Health, Food Chain Safety, and Environment (Belgium). Belgium Minimum Hospital Summary 2012

Federal Public Service Health, Food Chain Safety, and Environment (Belgium). Belgium Minimum Hospital Summary 2013

Federal Statistical Office (Germany). Germany Federal Health Reporting Hospital Discharges 1990

Federal Statistical Office (Germany). Germany Federal Health Reporting Hospital Discharges 1991
Federal Statistical Office (Germany). Germany Federal Health Reporting Hospital Discharges 1992
Federal Statistical Office (Germany). Germany Federal Health Reporting Hospital Discharges 1993
Federal Statistical Office (Germany). Germany Federal Health Reporting Hospital Discharges 1994
Federal Statistical Office (Germany). Germany Federal Health Reporting Hospital Discharges 1995
Federal Statistical Office (Germany). Germany Federal Health Reporting Hospital Discharges 1996
Federal Statistical Office (Germany). Germany Federal Health Reporting Hospital Discharges 1997
Federal Statistical Office (Germany). Germany Federal Health Reporting Hospital Discharges 1998
Federal Statistical Office (Germany). Germany Federal Health Reporting Hospital Discharges 1999
Federal Statistical Office (Germany). Germany Federal Health Reporting Hospital Discharges 2000
Federal Statistical Office (Germany). Germany Federal Health Reporting Hospital Discharges 2001
Federal Statistical Office (Germany). Germany Federal Health Reporting Hospital Discharges 2002
Federal Statistical Office (Germany). Germany Federal Health Reporting Hospital Discharges 2013
Federal Statistical Office (Germany). Germany Federal Health Reporting Hospital Discharges 2014
Federal Statistical Office (Germany). Germany Hospital Discharges by Diagnosis 2009. Wiesbaden, Germany: Federal Statistical Office (Germany), 2011
Federal Statistical Office (Switzerland). Switzerland Medical Statistics of Hospitals 1997
Federal Statistical Office (Switzerland). Switzerland Medical Statistics of Hospitals 1998
Federal Statistical Office (Switzerland). Switzerland Medical Statistics of Hospitals 2013
Federal Statistical Office (Switzerland). Switzerland Medical Statistics of Hospitals 2014
Finnish Congenital Anomaly Data 1993-1998 - WHO as it appears in European Surveillance of Congenital Anomalies (EUROCAT), International Centre on Birth Defects, World Health Organization (WHO). World Atlas of Birth Defects. 2nd ed. Geneva, Switzerland: World Health Organization (WHO), 2003
Finnish Register of Congenital Malformations Data 2001 - ICBDMS as it appears in International Clearinghouse for Birth Defects Monitoring Systems. International Clearinghouse for Birth Defects Monitoring Systems Annual Report 2003. Rome, Italy: International Centre on Birth Defects
Finnish Register of Congenital Malformations Data 2002 - ICBDMS as it appears in International Clearinghouse for Birth Defects Monitoring Systems. International Clearinghouse for Birth Defects Monitoring Systems Annual Report 2004. Rome, Italy: International Centre on Birth Defects, 2006
Finnish Register of Congenital Malformations Data 2003 - ICBDSR as it appears in International Clearinghouse for Birth Defects Surveillance and Research. International Clearinghouse for Birth Defects Surveillance and Research Annual Report 2005. Rome, Italy: International Clearinghouse for Birth Defects Surveillance and Research, 2007
Finnish Register of Congenital Malformations Data 2004 - ICBDSR as it appears in International Clearinghouse for Birth Defects Surveillance and Research. International Clearinghouse for Birth Defects Surveillance and Research Annual Report 2006. Rome, Italy: International Clearinghouse for Birth Defects Surveillance and Research, 2007
Finnish Register of Congenital Malformations Data 2005 - ICBDSR as it appears in International Clearinghouse for Birth Defects Surveillance and Research. International Clearinghouse for Birth Defects Surveillance and Research Annual Report 2007. Rome, Italy: International Clearinghouse for Birth Defects Surveillance and Research, 2008

Finnish Register of Congenital Malformations Data 2007 - ICBDSR as it appears in International Clearinghouse for Birth Defects Surveillance and Research. International Clearinghouse for Birth Defects Surveillance and Research Annual Report 2009. Rome, Italy: International Clearinghouse for Birth Defects Surveillance and Research

Finnish Register of Congenital Malformations Data 2008 - ICBDSR as it appears in International Clearinghouse for Birth Defects Surveillance and Research. International Clearinghouse for Birth Defects Surveillance and Research Annual Report 2010. Rome, Italy: International Clearinghouse for Birth Defects Surveillance and Research, 2011

Finnish Register of Congenital Malformations Data 2009 - ICBDSR as it appears in International Clearinghouse for Birth Defects Surveillance and Research. International Clearinghouse for Birth Defects Surveillance and Research Annual Report 2011. Rome, Italy: International Clearinghouse for Birth Defects Surveillance and Research, 2012

Finnish Register of Congenital Malformations Data 2010 - ICBDSR as it appears in International Clearinghouse for Birth Defects Surveillance and Research. International Clearinghouse for Birth Defects Surveillance and Research Annual Report 2012. Rome, Italy: International Clearinghouse for Birth Defects Surveillance and Research, 2013

Fischer H, Sonnweber N, Sailer M, Fink C, Trawöger R, Hammerer I. Incidence of congenital heart disease in Tyrol, Austria 1979-1983. Padiatr Padol. 1991; 26(1): 57-60

Fixler DE, Nembhard WN, Xu P, Ethen MK, Canfield MA. Effect of acculturation and distance from cardiac center on congenital heart disease mortality. Pediatrics. 2012; 129(6): 1118-24

Fofana M, Touré S, Dadhi Balde M, Sow T, Yassima Camara A, Damby Balde O, Toure A, Conde A. Etiologic and nosologic considerations apropos of 574 cases of cardiac decompensation in Conakry. Ann Cardiol Angeiol (Paris). 1988; 37(8): 419-24

Forrester MB, Merz RD. First-year mortality rates for selected birth defects, Hawaii, 1986-1999. Am J Med Genet A. 2003; 119A(3): 311-8

France - Central-East France Register of Congenital Malformations Data 1993-1998 - WHO as it appears in European Surveillance of Congenital Anomalies (EUROCAT), International Centre on Birth Defects, World Health Organization (WHO). World Atlas of Birth Defects. 2nd ed. Geneva, Switzerland: World Health Organization (WHO), 2003

France - Central-East France Register of Congenital Malformations Data 2001 - ICBDMS as it appears in International Clearinghouse for Birth Defects Monitoring Systems. International Clearinghouse for Birth Defects Monitoring Systems Annual Report 2003. Rome, Italy: International Centre on Birth Defects

France - Central-East France Register of Congenital Malformations Data 2002 - ICBDMS as it appears in International Clearinghouse for Birth Defects Monitoring Systems. International Clearinghouse for Birth Defects Monitoring Systems Annual Report 2004. Rome, Italy: International Centre on Birth Defects, 2006

France - Central-East France Register of Congenital Malformations Data 2003 - ICBDSR as it appears in International Clearinghouse for Birth Defects Surveillance and Research. International Clearinghouse for Birth Defects Surveillance and Research Annual Report 2005. Rome, Italy: International Clearinghouse for Birth Defects Surveillance and Research, 2007

France - Central-East France Register of Congenital Malformations Data 2004 - ICBDSR as it appears in International Clearinghouse for Birth Defects Surveillance and Research. International Clearinghouse for Birth Defects Surveillance and Research Annual Report 2006. Rome, Italy: International Clearinghouse for Birth Defects Surveillance and Research, 2007

France - Central-East France Register of Congenital Malformations Data 2005 - ICBDSR as it appears in International Clearinghouse for Birth Defects Surveillance and Research. International Clearinghouse for Birth Defects Surveillance and Research Annual Report 2007. Rome, Italy: International Clearinghouse for Birth Defects Surveillance and Research, 2008

France - Paris Congenital Anomaly Data 1993-1998 - WHO as it appears in European Surveillance of Congenital Anomalies (EUROCAT), International Centre on Birth Defects, World Health Organization (WHO). World Atlas of Birth Defects. 2nd ed. Geneva, Switzerland: World Health Organization (WHO), 2003

France - Paris Congenital Anomaly Data 2003 - ICBDSR as it appears in International Clearinghouse for Birth Defects Surveillance and Research. International Clearinghouse for Birth Defects Surveillance and Research Annual Report 2005. Rome, Italy: International Clearinghouse for Birth Defects Surveillance and Research, 2007

France - Paris Congenital Anomaly Data 2004 - ICBDSR as it appears in International Clearinghouse for Birth Defects Surveillance and Research. International Clearinghouse for Birth Defects Surveillance and Research Annual Report 2006. Rome, Italy: International Clearinghouse for Birth Defects Surveillance and Research, 2007

France - Paris Congenital Anomaly Data 2007 - ICBDSR as it appears in International Clearinghouse for Birth Defects Surveillance and Research. International Clearinghouse for Birth Defects Surveillance and Research Annual Report 2009. Rome, Italy: International Clearinghouse for Birth Defects Surveillance and Research

France - Paris Congenital Anomaly Data 2008 - ICBDSR as it appears in International Clearinghouse for Birth Defects Surveillance and Research. International Clearinghouse for Birth Defects Surveillance and Research Annual Report 2010. Rome, Italy: International Clearinghouse for Birth Defects Surveillance and Research, 2011

France - Paris Congenital Anomaly Data 2009 - ICBDSR as it appears in International Clearinghouse for Birth Defects Surveillance and Research. International Clearinghouse for Birth Defects Surveillance and Research Annual Report 2011. Rome, Italy: International Clearinghouse for Birth Defects Surveillance and Research, 2012

France - Paris Congenital Anomaly Data 2010 - ICBDSR as it appears in International Clearinghouse for Birth Defects Surveillance and Research. International Clearinghouse for Birth Defects Surveillance and Research Annual Report 2012. Rome, Italy: International Clearinghouse for Birth Defects Surveillance and Research, 2013

France - Paris Registry of Congenital Malformations Data 2001 - ICBDMS as it appears in International Clearinghouse for Birth Defects Monitoring Systems. International Clearinghouse for Birth Defects Monitoring Systems Annual Report 2003. Rome, Italy: International Centre on Birth Defects

France - Paris Registry of Congenital Malformations Data 2002 - ICBDMS as it appears in International Clearinghouse for Birth Defects Monitoring Systems. International Clearinghouse for Birth Defects Monitoring Systems Annual Report 2004. Rome, Italy: International Centre on Birth Defects, 2006

France - Paris Registry of Congenital Malformations Data 2005 - ICBDSR as it appears in International Clearinghouse for Birth Defects Surveillance and Research. International Clearinghouse for Birth Defects Surveillance and Research Annual Report 2007. Rome, Italy: International Clearinghouse for Birth Defects Surveillance and Research, 2008

France - Paris Registry of Congenital Malformations Data 2012 - ICBDSR as it appears in International Clearinghouse for Birth Defects Surveillance and Research. International Clearinghouse for Birth Defects Surveillance and Research Annual Report 2014. Rome, Italy: International Clearinghouse for Birth Defects Surveillance and Research, 2016

France - Rhone Alps Registry of Malformations Data 2007 - ICBDSR as it appears in International Clearinghouse for Birth Defects Surveillance and Research. International Clearinghouse for Birth Defects Surveillance and Research Annual Report 2009. Rome, Italy: International Clearinghouse for Birth Defects Surveillance and Research

France - Rhone Alps Registry of Malformations Data 2008 - ICBDSR as it appears in International Clearinghouse for Birth Defects Surveillance and Research. International Clearinghouse for Birth Defects Surveillance and Research Annual Report 2010. Rome, Italy: International Clearinghouse for Birth Defects Surveillance and Research, 2011

France - Rhone Alps Registry of Malformations Data 2009 - ICBDSR as it appears in International Clearinghouse for Birth Defects Surveillance and Research. International Clearinghouse for Birth Defects Surveillance and Research Annual Report 2011. Rome, Italy: International Clearinghouse for Birth Defects Surveillance and Research, 2012

France - Rhone Alps Registry of Malformations Data 2010 - ICBDSR as it appears in International Clearinghouse for Birth Defects Surveillance and Research. International Clearinghouse for Birth Defects Surveillance and Research Annual Report 2012. Rome, Italy: International Clearinghouse for Birth Defects Surveillance and Research, 2013

France - Rhone Alps Registry of Malformations Data 2012 - ICBDSR as it appears in International Clearinghouse for Birth Defects Surveillance and Research. International Clearinghouse for Birth Defects Surveillance and Research Annual Report 2014. Rome, Italy: International Clearinghouse for Birth Defects Surveillance and Research, 2016

France - Strasbourg Prospective Study of Congenital Malformations Data 1993-1998 - WHO as it appears in European Surveillance of Congenital Anomalies (EUROCAT), International Centre on Birth Defects, World Health Organization (WHO). World Atlas of Birth Defects. 2nd ed. Geneva, Switzerland: World Health Organization (WHO), 2003

France - Strasbourg Prospective Study of Congenital Malformations Data 2001 - ICBDMS as it appears in International Clearinghouse for Birth Defects Monitoring Systems. International Clearinghouse for Birth Defects Monitoring Systems Annual Report 2003. Rome, Italy: International Centre on Birth Defects

France - Strasbourg Prospective Study of Congenital Malformations Data 2002 - ICBDMS as it appears in International Clearinghouse for Birth Defects Monitoring Systems. International Clearinghouse for Birth Defects Monitoring Systems Annual Report 2004. Rome, Italy: International Centre on Birth Defects, 2006

France - Strasbourg Prospective Study of Congenital Malformations Data 2003 - ICBDSR as it appears in International Clearinghouse for Birth Defects Surveillance and Research. International Clearinghouse for Birth Defects Surveillance and Research Annual Report 2005. Rome, Italy: International Clearinghouse for Birth Defects Surveillance and Research, 2007

France - Strasbourg Prospective Study of Congenital Malformations Data 2004 - ICBDSR as it appears in International Clearinghouse for Birth Defects Surveillance and Research. International Clearinghouse for Birth Defects Surveillance and Research Annual Report 2006. Rome, Italy: International Clearinghouse for Birth Defects Surveillance and Research, 2007

France - Strasbourg Prospective Study of Congenital Malformations Data 2005 - ICBDSR as it appears in International Clearinghouse for Birth Defects Surveillance and Research. International Clearinghouse for Birth Defects Surveillance and Research Annual Report 2007. Rome, Italy: International Clearinghouse for Birth Defects Surveillance and Research, 2008

France - Strasbourg Prospective Study of Congenital Malformations Data 2007 - ICBDSR as it appears in International Clearinghouse for Birth Defects Surveillance and Research. International Clearinghouse for Birth Defects Surveillance and Research Annual Report 2009. Rome, Italy: International Clearinghouse for Birth Defects Surveillance and Research

France - Strasbourg Registry of Congenital Malformations Data 2009 - ICBDSR as it appears in International Clearinghouse for Birth Defects Surveillance and Research. International Clearinghouse for Birth Defects Surveillance and Research Annual Report 2011. Rome, Italy: International Clearinghouse for Birth Defects Surveillance and Research, 2012

Frid C, Björkhem G, Jonzon A, Sunnegårdh J, Annerén G, Lundell B. Long-term survival in children with atrioventricular septal defect and common atrioventricular valvar orifice in Sweden. Cardiol Young. 2004; 14(1): 24-31

Fu C-M, Wang J-K, Lu C-W, Chiu S-N, Lin M-T, Chen C-A, Chang C-I, Chen Y-S, Chiu I-S, Wu M-H. Total anomalous pulmonary venous connection: 15 years' experience of a tertiary care center in Taiwan. Pediatr Neonatol. 2012; 53(3): 164-70

Gallivan S, Stark J, Pagel C, Williams G, Williams WG. Dead reckoning: can we trust estimates of mortality rates in clinical databases?. Eur J Cardiothorac Surg. 2008; 33(3): 334-40

Gallup, Health Promotion Research Institute (Hungary). Hungary National Population Health Survey 2000

Gallup, World Health Organization (WHO). Argentina WHO Multi-country Survey Study on Health and Health System Responsiveness 2000-2001. Geneva, Switzerland: World Health Organization (WHO)

Gallup, World Health Organization (WHO). Bahrain WHO Multi-country Survey Study on Health and Health System Responsiveness 2000-2001. Geneva, Switzerland: World Health Organization (WHO)

Gallup, World Health Organization (WHO). Costa Rica WHO Multi-country Survey Study on Health and Health System Responsiveness 2000-2001. Geneva, Switzerland: World Health Organization (WHO)

Gallup, World Health Organization (WHO). Jordan WHO Multi-country Survey Study on Health and Health System Responsiveness 2000-2001. Geneva, Switzerland: World Health Organization (WHO)

Gallup, World Health Organization (WHO). Latvia WHO Multi-country Survey Study on Health and Health System Responsiveness 2000-2001. Geneva, Switzerland: World Health Organization (WHO)

Gallup, World Health Organization (WHO). Morocco WHO Multi-country Survey Study on Health and Health System Responsiveness 2000-2001. Geneva, Switzerland: World Health Organization (WHO)

Gallup, World Health Organization (WHO). Oman WHO Multi-country Survey Study on Health and Health System Responsiveness 2000-2001. Geneva, Switzerland: World Health Organization (WHO)

Gallup, World Health Organization (WHO). United Arab Emirates WHO Multi-country Survey Study on Health and Health System Responsiveness 2000-2001. Geneva, Switzerland: World Health Organization (WHO)

Gallup, World Health Organization (WHO). Venezuela WHO Multi-country Survey Study on Health and Health System Responsiveness 2000-2001. Geneva, Switzerland: World Health Organization (WHO)

Garne E, Nielsen G, Hansen OK, Emmertsen K. Tetralogy of Fallot. A population-based study of epidemiology, associated malformations and survival in western Denmark 1984-1992. Scand Cardiovasc J. 1999; 33(1): 45-8

Garne E, Stoll C, Clementi M, Euroscan Group. Evaluation of prenatal diagnosis of congenital heart diseases by ultrasound: experience from 20 European registries. Ultrasound Obstet Gynecol. 2001; 17(5): 386-91

Gatzoulis MA, Freeman MA, Siu SC, Webb GD, Harris L. Atrial arrhythmia after surgical closure of atrial septal defects in adults. N Engl J Med. 1999; 340(11): 839-46

Gatzoulis MA, Munk MD, Williams WG, Webb GD. Definitive palliation with cavopulmonary or aortopulmonary shunts for adults with single ventricle physiology. Heart. 2000; 83(1): 51-7

Gatzoulis MA, Walters J, McLaughlin PR, Merchant N, Webb GD, Liu P. Late arrhythmia in adults with the mustard procedure for transposition of great arteries: a surrogate marker for right ventricular dysfunction?. Heart. 2000; 84(4): 409-15

Gedikbaşı A, Oztarhan K, Yıldırım G, Gül A, Ceylan Y. Counseling and outcomes of antenatally diagnosed congenital heart anomalies in Turkey. Anatol J Cardiol. 2011; 11(2): 137-45

Gelatt M, Hamilton RM, McCrindle BW, Connelly M, Davis A, Harris L, Gow RM, Williams WG, Trusler GA, Freedom RM. Arrhythmia and mortality after the Mustard procedure: a 30-year single-center experience. J Am Coll Cardiol. 1997; 29(1): 194-201

General Administration of Statistics and Censuses (El Salvador), Ministry of Economy (El Salvador). El Salvador Multipurpose Household Survey 2013. San Salvador, El Salvador: General Administration of Statistics and Censuses (El Salvador)
General Administration of Statistics and Censuses (El Salvador), Ministry of Economy (El Salvador). El Salvador Multipurpose Household Survey 2014. San Salvador, El Salvador: General Administration of Statistics and Censuses (El Salvador)
General Directorate of Curative Services, Ministry of Health (Turkey). Turkey Hospital Inpatient Discharges 2007
General Directorate of Curative Services, Ministry of Health (Turkey). Turkey Hospital Inpatient Discharges 2008
General Directorate of Curative Services, Ministry of Health (Turkey). Turkey Hospital Inpatient Discharges 2009
General Directorate of Curative Services, Ministry of Health (Turkey). Turkey Hospital Inpatient Discharges 2010
General Directorate of Curative Services, Ministry of Health (Turkey). Turkey Hospital Inpatient Discharges 2011
General Directorate of Curative Services, Ministry of Health (Turkey). Turkey Inpatient Care Discharges per 100 1980
General Directorate of Curative Services, Ministry of Health (Turkey). Turkey Inpatient Care Discharges per 100 1981
General Directorate of Curative Services, Ministry of Health (Turkey). Turkey Inpatient Care Discharges per 100 1982
General Directorate of Curative Services, Ministry of Health (Turkey). Turkey Inpatient Care Discharges per 100 1983
General Directorate of Curative Services, Ministry of Health (Turkey). Turkey Inpatient Care Discharges per 100 1984
General Directorate of Curative Services, Ministry of Health (Turkey). Turkey Inpatient Care Discharges per 100 1985
General Directorate of Curative Services, Ministry of Health (Turkey). Turkey Inpatient Care Discharges per 100 1986
General Directorate of Curative Services, Ministry of Health (Turkey). Turkey Inpatient Care Discharges per 100 1987
General Directorate of Curative Services, Ministry of Health (Turkey). Turkey Inpatient Care Discharges per 100 1988
General Directorate of Curative Services, Ministry of Health (Turkey). Turkey Inpatient Care Discharges per 100 1989
General Directorate of Curative Services, Ministry of Health (Turkey). Turkey Inpatient Care Discharges per 100 1990
General Directorate of Curative Services, Ministry of Health (Turkey). Turkey Inpatient Care Discharges per 100 1991
General Directorate of Curative Services, Ministry of Health (Turkey). Turkey Inpatient Care Discharges per 100 1992
General Directorate of Curative Services, Ministry of Health (Turkey). Turkey Inpatient Care Discharges per 100 1993
General Directorate of Curative Services, Ministry of Health (Turkey). Turkey Inpatient Care Discharges per 100 1994
General Directorate of Curative Services, Ministry of Health (Turkey). Turkey Inpatient Care Discharges per 100 1995
General Directorate of Curative Services, Ministry of Health (Turkey). Turkey Inpatient Care Discharges per 100 1996
General Directorate of Curative Services, Ministry of Health (Turkey). Turkey Inpatient Care Discharges per 100 1997
General Directorate of Curative Services, Ministry of Health (Turkey). Turkey Inpatient Care Discharges per 100 1998
General Directorate of Curative Services, Ministry of Health (Turkey). Turkey Inpatient Care Discharges per 100 1999
General Directorate of Curative Services, Ministry of Health (Turkey). Turkey Inpatient Care Discharges per 100 2000
General Directorate of Curative Services, Ministry of Health (Turkey). Turkey Inpatient Care Discharges per 100 2001
General Directorate of Curative Services, Ministry of Health (Turkey). Turkey Inpatient Care Discharges per 100 2002
General Directorate of Curative Services, Ministry of Health (Turkey). Turkey Inpatient Care Discharges per 100 2003
General Directorate of Curative Services, Ministry of Health (Turkey). Turkey Inpatient Care Discharges per 100 2004
General Directorate of Curative Services, Ministry of Health (Turkey). Turkey Inpatient Care Discharges per 100 2005

General Directorate of Curative Services, Ministry of Health (Turkey). Turkey Inpatient Care Discharges per 100 2006

General Directorate of Curative Services, Ministry of Health (Turkey). Turkey Inpatient Care Discharges per 100 2012

General Directorate of Curative Services, Ministry of Health (Turkey). Turkey Inpatient Care Discharges per 100 2013

General Directorate of Curative Services, Ministry of Health (Turkey). Turkey Inpatient Care Discharges per 100 2014

General Statistics Office (Viet Nam), United Nations Development Programme (UNDP), World Bank (WB). Viet Nam Living Standards Measurement Survey 2008. Ha N[illegible]i, Viet Nam: General Statistics Office (Viet Nam)

General Statistics Office (Vietnam), United Nations Development Programme (UNDP), World Bank. Vietnam Living Standards Measurement Survey 2006

Germany - Saxony-Anhalt Malformation Monitoring Center Data 2007 - ICBDSR as it appears in International Clearinghouse for Birth Defects Surveillance and Research. International Clearinghouse for Birth Defects Surveillance and Research Annual Report 2009. Rome, Italy: International Clearinghouse for Birth Defects Surveillance and Research

Germany - Saxony-Anhalt Malformation Monitoring Center Data 2008 - ICBDSR as it appears in International Clearinghouse for Birth Defects Surveillance and Research. International Clearinghouse for Birth Defects Surveillance and Research Annual Report 2010. Rome, Italy: International Clearinghouse for Birth Defects Surveillance and Research, 2011

Germany - Saxony-Anhalt Malformation Monitoring Center Data 2009 - ICBDSR as it appears in International Clearinghouse for Birth Defects Surveillance and Research. International Clearinghouse for Birth Defects Surveillance and Research Annual Report 2011. Rome, Italy: International Clearinghouse for Birth Defects Surveillance and Research, 2012

Germany - Saxony-Anhalt Malformation Monitoring Center Data 2010 - ICBDSR as it appears in International Clearinghouse for Birth Defects Surveillance and Research. International Clearinghouse for Birth Defects Surveillance and Research Annual Report 2012. Rome, Italy: International Clearinghouse for Birth Defects Surveillance and Research, 2013

Germany - Saxony-Anhalt Malformation Monitoring Center Data 2012 - ICBDSR as it appears in International Clearinghouse for Birth Defects Surveillance and Research. International Clearinghouse for Birth Defects Surveillance and Research Annual Report 2014. Rome, Italy: International Clearinghouse for Birth Defects Surveillance and Research, 2016

Germany - Saxony-Anhalt Malformation Monitoring Data 1993-1998 - WHO as it appears in European Surveillance of Congenital Anomalies (EUROCAT), International Centre on Birth Defects, World Health Organization (WHO). World Atlas of Birth Defects. 2nd ed. Geneva, Switzerland: World Health Organization (WHO), 2003

Germany - Saxony-Anhalt Malformation Monitoring Data 2001 - ICBDMS as it appears in International Clearinghouse for Birth Defects Monitoring Systems. International Clearinghouse for Birth Defects Monitoring Systems Annual Report 2003. Rome, Italy: International Centre on Birth Defects

Germany - Saxony-Anhalt Malformation Monitoring Data 2002 - ICBDMS as it appears in International Clearinghouse for Birth Defects Monitoring Systems. International Clearinghouse for Birth Defects Monitoring Systems Annual Report 2004. Rome, Italy: International Centre on Birth Defects, 2006

Germany - Saxony-Anhalt Malformation Monitoring Data 2003 - ICBDSR as it appears in International Clearinghouse for Birth Defects Surveillance and Research. International Clearinghouse for Birth Defects Surveillance and Research Annual Report 2005. Rome, Italy: International Clearinghouse for Birth Defects Surveillance and Research, 2007

Germany - Saxony-Anhalt Malformation Monitoring Data 2004 - ICBDSR as it appears in International Clearinghouse for Birth Defects Surveillance and Research. International Clearinghouse for Birth Defects Surveillance and Research Annual Report 2006. Rome, Italy: International Clearinghouse for Birth Defects Surveillance and Research, 2007

Germany - Saxony-Anhalt Malformation Monitoring Data 2005 - ICBDSR as it appears in International Clearinghouse for Birth Defects Surveillance and Research. International Clearinghouse for Birth Defects Surveillance and Research Annual Report 2007. Rome, Italy: International Clearinghouse for Birth Defects Surveillance and Research, 2008

Gev D, Roguin N, Freundlich E. Consanguinity and congenital heart disease in the rural Arab population in northern Israel. Hum Hered. 1986; 36(4): 213-7

Ghana Statistical Service, World Bank. Ghana Living Standards Measurement Survey 2012-2013. Accra, Ghana: Ghana Statistical Service

Ghana Statistical Service. Ghana Living Standards Measurement Survey 1991-1992. Accra, Ghana: Ghana Statistical Service

Ghana Statistical Service. Ghana Living Standards Measurement Survey 2005-2006. Accra, Ghana: Ghana Statistical Service

Ghana Statistical Service. Ghana Living Standards Survey 1998-1999

Gnanappa GK, Ganigara M, Prabhu A, Varma SK, Murmu U, Varghese R, Valliatu J, Kumar RNS. Outcome of complex adult congenital heart surgery in the developing world. Congenit Heart Dis. 2011; 6(1): 2-8

Gottschalk I, Gottschalk L, Stressig R, Ritgen J, Herberg U, Breuer J, Oberhoffer R, Willruth A, Strizek B, Geipel A, Gembruch U, Berg C. Ebstein's Anomaly of the Tricuspid Valve in the Fetus - A Multicenter Experience. Ultraschall Med. 2016; nan

Government of India, Ministry of Statistics and Programme Implementation (India). India National Sample Survey Round 71 2014. New Delhi, India: Ministry of Statistics and Programme Implementation (India)

Graduate School of Public Health, Seoul National University, World Health Organization (WHO). South Korea WHO Multi-country Survey Study on Health and Health System Responsiveness 2000-2001. Geneva, Switzerland: World Health Organization (WHO)

Graham TP Jr, Bernard Y, Arbogast P, Thapa S, Cetta F, Child J, Chugh R, Davidson W, Hurwitz R, Kay J, Sanders S, Schaufelberger M. Outcome of pulmonary valve replacements in adults after tetralogy repair: a multi-institutional study. Congenit Heart Dis. 2008; 3(3): 162-7

Grech V. Trends in presentation of congenital heart disease in a population-based study in Malta. Eur J Epidemiol. 1999; 15(10): 881-7

Guitti JC. Epidemiological characteristics of congenital heart diseases in Londrina, Paraná south Brazil. Arq Bras Cardiol. 2000; 74(5): 395-404

Hamad Medical Corporation (Qatar). Qatar - Annual Inpatients Discharge Abstract: Hamad General Hospital 2002. Doha, Qatar: Hamad Medical Corporation (Qatar)

Hamad Medical Corporation (Qatar). Qatar - Annual Inpatients Discharge Abstract: Hamad General Hospital and Women's Hospital 2003. Doha, Qatar: Hamad Medical Corporation (Qatar)

Hamada H, Terai M, Jibiki T, Nakamura T, Gatzoulis MA, Niwa K. Influence of early repair of tetralogy of fallot without an outflow patch on late arrhythmias and sudden death: a 27-year follow-up study following a uniform surgical approach. Cardiol Young. 2002; 12(4): 345-51

Hannoush H, Tamim H, Younes H, Arnaout S, Gharzeddine W, Dakik H, Obeid M, Bitar FF. Patterns of congenital heart disease in unoperated adults: a 20-year experience in a developing country. Clin Cardiol. 2004; 27(4): 236-40

Harrison DA, Harris L, Siu SC, MacLoghlin CJ, Connelly MS, Webb GD, Downar E, McLaughlin PR, Williams WG. Sustained ventricular tachycardia in adult patients late after repair of tetralogy of Fallot. J Am Coll Cardiol. 1997; 30(5): 1368-73

Harrison DA, Siu SC, Hussain F, MacLoghlin CJ, Webb GD, Harris L. Sustained atrial arrhythmias in adults late after repair of tetralogy of fallot. Am J Cardiol. 2001; 87(5): 584-8

Hassan I, Haleem AA, Bhutta ZA. Profile and risk factors for congenital heart disease. J Pak Med Assoc. 1997; 47(3): 78-81

Health Care International, World Health Organization (WHO). Egypt WHO Multi-country Survey Study on Health and Health System Responsiveness 2000-2001. Geneva, Switzerland: World Health Organization (WHO)

Health Institute (São Paulo, Brazil), State University of Campinas, São Paulo Municipal Health Department, São Paulo State University, University of São Paulo. Brazil - São Paulo Health Survey 2008-2009

Healthcare Cost and Utilization Project (HCUP), Agency for Healthcare Research and Quality (AHRQ). United States Nationwide Inpatient Sample 2013. Rockville, United States: Healthcare Cost and Utilization Project (HCUP), Agency for Healthcare Research and Quality (AHRQ)

Healthcare Cost and Utilization Project (HCUP), Agency for Healthcare Research and Quality (AHRQ). United States Nationwide Inpatient Sample 2014. Rockville, United States: Healthcare Cost and Utilization Project (HCUP), Agency for Healthcare Research and Quality (AHRQ)

Healthcare Cost and Utilization Project (HCUP), Agency for Healthcare Research and Quality (AHRQ). United States Nationwide Inpatient Sample 2015. Rockville, United States: Healthcare Cost and Utilization Project (HCUP), Agency for Healthcare Research and Quality (AHRQ)

Healthcare Cost and Utilization Project (HCUP), Agency for Healthcare Research and Quality (AHRQ). United States State Inpatient Databases 2003. Rockville, United States: Healthcare Cost and Utilization Project (HCUP), Agency for Healthcare Research and Quality (AHRQ)

Healthcare Cost and Utilization Project (HCUP), Agency for Healthcare Research and Quality (AHRQ). United States State Inpatient Databases 2003-2007

Healthcare Cost and Utilization Project (HCUP), Agency for Healthcare Research and Quality (AHRQ). United States State Inpatient Databases 2004. Rockville, United States: Healthcare Cost and Utilization Project (HCUP), Agency for Healthcare Research and Quality (AHRQ)

Healthcare Cost and Utilization Project (HCUP), Agency for Healthcare Research and Quality (AHRQ). United States State Inpatient Databases 2005. Rockville, United States: Healthcare Cost and Utilization Project (HCUP), Agency for Healthcare Research and Quality (AHRQ)

Healthcare Cost and Utilization Project (HCUP), Agency for Healthcare Research and Quality (AHRQ). United States State Inpatient Databases 2006. Rockville, United States: Healthcare Cost and Utilization Project (HCUP), Agency for Healthcare Research and Quality (AHRQ)

Healthcare Cost and Utilization Project (HCUP), Agency for Healthcare Research and Quality (AHRQ). United States State Inpatient Databases 2007. Rockville, United States: Healthcare Cost and Utilization Project (HCUP), Agency for Healthcare Research and Quality (AHRQ)

Healthcare Cost and Utilization Project (HCUP), Agency for Healthcare Research and Quality (AHRQ). United States State Inpatient Databases 2008. Rockville, United States: Healthcare Cost and Utilization Project (HCUP), Agency for Healthcare Research and Quality (AHRQ)

Healthcare Cost and Utilization Project (HCUP), Agency for Healthcare Research and Quality (AHRQ). United States State Inpatient Databases 2008-2009

Healthcare Cost and Utilization Project (HCUP), Agency for Healthcare Research and Quality (AHRQ). United States State Inpatient Databases 2009. Rockville, United States: Healthcare Cost and Utilization Project (HCUP), Agency for Healthcare Research and Quality (AHRQ)

Healthcare Cost and Utilization Project (HCUP), Agency for Healthcare Research and Quality (AHRQ). United States State Inpatient Databases 2011. Rockville, United States: Healthcare Cost and Utilization Project (HCUP), Agency for Healthcare Research and Quality (AHRQ)

Healthcare Cost and Utilization Project (HCUP), Agency for Healthcare Research and Quality (AHRQ). United States State Inpatient Databases 2012. Rockville, United States: Healthcare Cost and Utilization Project (HCUP), Agency for Healthcare Research and Quality (AHRQ)

Healthcare Cost and Utilization Project (HCUP), Agency for Healthcare Research and Quality (AHRQ). United States State Inpatient Databases 2013. Rockville, United States: Healthcare Cost and Utilization Project (HCUP), Agency for Healthcare Research and Quality (AHRQ)

Healthcare Cost and Utilization Project (HCUP), Agency for Healthcare Research and Quality (AHRQ). United States State Inpatient Databases 2014. Rockville, United States: Healthcare Cost and Utilization Project (HCUP), Agency for Healthcare Research and Quality (AHRQ)

Hegazy IS, Al-Beyari TH, Al-Amri AH, Qureshi NA, Abdelgadir MH. Congenital malformations in primary health care in Al-Qassim region. Ann Saudi Med. 1995; 15(1): 48-53

Helgason H, Jonsdottir G. Spontaneous Closure of Atrial Septal Defects. Pediatr Cardiol. 1999; 20(3): 195-9

Henry G, Alexander D, Brann S, Sammy I. Paediatric open heart surgery in Trinidad and Tobago: an example of collaborative care. West Indian Med J. 2005; 54(1): 9-13

Hickey EJ, Nosikova Y, Zhang H, Caldarone CA, Benson L, Redington A, Van Arsdell GS. Very low-birth-weight infants with congenital cardiac lesions: is there merit in delaying intervention to permit growth and maturation?. J Thorac Cardiovasc Surg. 2012; 143(1): 126-36

Hickey EJ, Veldtman G, Bradley TJ, Gengsakul A, Manlhiot C, Williams WG, Webb GD, McCrindle BW. Late risk of outcomes for adults with repaired tetralogy of Fallot from an inception cohort spanning four decades. Eur J Cardiothorac Surg. 2009; 35(1): 156-164

Himmetoglu O, Tiras MB, Gursoy R, Karabacak O, Sahin I, Onan A. The incidence of congenital malformations in a Turkish population. Int J Gynaecol Obstet. 1996; 55(2): 117-21

Hiraishi S, Agata Y, Nowatari M, Oguchi K, Misawa H, Hirota H, Fujino N, Horiguchi Y, Yashiro K, Nakae S. Incidence and natural course of trabecular ventricular septal defect: two-dimensional echocardiography and color Doppler flow imaging study. J Pediatr. 1992; 120(3): 409-15

Hisatomi K, Isomura T, Kosuga K, Sato T, Nishimi M, Mizoguchi T, Ishii H, Aoyagi S, Ohishi K, Kato H. An evaluation of long-term results over 10 years after intracardiac repair of tetralogy of Fallot. Kurume Med J. 1991; 38(3): 149-57

Hisatomi K, Isomura T, Sato T, Hirano A, Aoyagi S, Kosuga K, Ohishi K, Katoh H. Long-term results after conservative aortic valve repair for aortic regurgitation with ventricular septal defect. J Cardiovasc Surg (Torino). 1995; 36(6): 541-4

Ho NK. Congenital malformations in Toa Payoh hospital--a 18 year experience (1972-1989). Ann Acad Med Singapore. 1991; 20(2): 183-9

Ho TC, Ouyang H, Lu Y, Young AH, Chintala K, Detrano RC. Postprocedural outcomes of rural children undergoing correction of congenital heart lesions in Yunnan Province, China. Pediatr Cardiol. 2011; 32(6): 811-4

Hokanson JS, Moller JH. Significance of early transient complete heart block as a predictor of sudden death late after operative correction of tetralogy of Fallot. Am J Cardiol. 2001; 87(11): 1271-7

Horvath KA, Burke RP, Collins JJ Jr, Cohn LH. Surgical treatment of adult atrial septal defect: early and long-term results. J Am Coll Cardiol. 1992; 20(5): 1156-9

Hoshino K, Ogawa K, Hishitani T, Kitazawa R, Uehara R. Hypoplastic left heart syndrome: duration of survival without surgical intervention. Am Heart J. 1999; 137(3): 535-42

Hsiao S-M, Wu M-H, Jou H-J, Lee C-N, Shyu M-K, Shih J-C, Hsieh F-J. Outcome for fetuses with prenatally detected congenital heart disease and cardiac arrhythmias in Taiwan. J Formos Med Assoc. 2007; 106(6): 423-31

Huang CJ, Chiu IS, Lin FY, Chen WJ, Lin JL, Lo HM, Wu MH, Chu SH. Role of electrophysiological studies and arrhythmia intervention in repairing Ebstein's anomaly. Thorac Cardiovasc Surg. 2000; 48(6): 347-50

Human Sciences Research Council, South African Medical Research Council. South Africa National Health and Nutrition Examination Survey 2012

Hungarian Congenital Abnormality Registry Data 1993-1998 - WHO as it appears in European Surveillance of Congenital Anomalies (EUROCAT), International Centre on Birth Defects, World Health Organization (WHO). World Atlas of Birth Defects. 2nd ed. Geneva, Switzerland: World Health Organization (WHO), 2003

Hungarian Congenital Abnormality Registry Data 2001 - ICBDMS as it appears in International Clearinghouse for Birth Defects Monitoring Systems. International Clearinghouse for Birth Defects Monitoring Systems Annual Report 2003. Rome, Italy: International Centre on Birth Defects

Hungarian Congenital Abnormality Registry Data 2002 - ICBDMS as it appears in International Clearinghouse for Birth Defects Monitoring Systems. International Clearinghouse for Birth Defects Monitoring Systems Annual Report 2004. Rome, Italy: International Centre on Birth Defects, 2006

Hungarian Congenital Abnormality Registry Data 2003 - ICBDSR as it appears in International Clearinghouse for Birth Defects Surveillance and Research. International Clearinghouse for Birth Defects Surveillance and Research Annual Report 2005. Rome, Italy: International Clearinghouse for Birth Defects Surveillance and Research, 2007

Hungarian Congenital Abnormality Registry Data 2004 - ICBDSR as it appears in International Clearinghouse for Birth Defects Surveillance and Research. International Clearinghouse for Birth Defects Surveillance and Research Annual Report 2006. Rome, Italy: International Clearinghouse for Birth Defects Surveillance and Research, 2007

Hungarian Congenital Abnormality Registry Data 2005 - ICBDSR as it appears in International Clearinghouse for Birth Defects Surveillance and Research. International Clearinghouse for Birth Defects Surveillance and Research Annual Report 2007. Rome, Italy: International Clearinghouse for Birth Defects Surveillance and Research, 2008

Hungarian Congenital Abnormality Registry Data 2008 - ICBDSR as it appears in International Clearinghouse for Birth Defects Surveillance and Research. International Clearinghouse for Birth Defects Surveillance and Research Annual Report 2010. Rome, Italy: International Clearinghouse for Birth Defects Surveillance and Research, 2011

Hungarian Congenital Abnormality Registry Data 2009 - ICBDSR as it appears in International Clearinghouse for Birth Defects Surveillance and Research. International Clearinghouse for Birth Defects Surveillance and Research Annual Report 2011. Rome, Italy: International Clearinghouse for Birth Defects Surveillance and Research, 2012

Hungarian Congenital Abnormality Registry Data 2010 - ICBDSR as it appears in International Clearinghouse for Birth Defects Surveillance and Research. International Clearinghouse for Birth Defects Surveillance and Research Annual Report 2012. Rome, Italy: International Clearinghouse for Birth Defects Surveillance and Research, 2013

Hungary Congenital Abnormality Registry Data 2012 - ICBDSR as it appears in International Clearinghouse for Birth Defects Surveillance and Research. International Clearinghouse for Birth Defects Surveillance and Research Annual Report 2014. Rome, Italy: International Clearinghouse for Birth Defects Surveillance and Research, 2016

I O George, A I Frank-Briggs. Pattern and clinical presentation of congenital heart diseases in Port-Harcourt. Niger J Med. 2008; 18(2): 211-4

India Birth Defects Registry Data 2008 - ICBDSR as it appears in International Clearinghouse for Birth Defects Surveillance and Research. International Clearinghouse for Birth Defects Surveillance and Research Annual Report 2010. Rome, Italy: International Clearinghouse for Birth Defects Surveillance and Research, 2011

India Birth Defects Registry Data 2009 - ICBDSR as it appears in International Clearinghouse for Birth Defects Surveillance and Research. International Clearinghouse for Birth Defects Surveillance and Research Annual Report 2011. Rome, Italy: International Clearinghouse for Birth Defects Surveillance and Research, 2012

India Birth Defects Registry Data 2010 - ICBDSR as it appears in International Clearinghouse for Birth Defects Surveillance and Research. International Clearinghouse for Birth Defects Surveillance and Research Annual Report 2012. Rome, Italy: International Clearinghouse for Birth Defects Surveillance and Research, 2013

Infectious Diseases Research Collaboration (IDRC), Institute for Health Metrics and Evaluation (IHME), Makerere University, Ministry of Health (Uganda). Access, Bottlenecks, Costs, and Equity (ABCE) project in Uganda, 2012. Seattle, United States: Institute for Health Metrics and Evaluation (IHME), 2015

Institute for Polling and Marketing (Georgia), World Health Organization (WHO). Georgia WHO Multi-country Survey Study on Health and Health System Responsiveness 2000-2001

Institute for Public Health, Ministry of Health (Malaysia). Malaysia National Health And Morbidity Survey 2006. Kuala Lumpur, Malaysia: Institute for Public Health, Ministry of Health (Malaysia)

Institute for Public Health, Ministry of Health (Malaysia). Malaysia National Health and Morbidity Survey 2011

Institute of Experimental and Clinical Medicine (Estonia). Estonia Hospital Inpatient Discharges 1980

Institute of Experimental and Clinical Medicine (Estonia). Estonia Hospital Inpatient Discharges 1985

Institute of Experimental and Clinical Medicine (Estonia). Estonia Hospital Inpatient Discharges 1986

Institute of Experimental and Clinical Medicine (Estonia). Estonia Hospital Inpatient Discharges 1987
Institute of Experimental and Clinical Medicine (Estonia). Estonia Hospital Inpatient Discharges 1988
Institute of Experimental and Clinical Medicine (Estonia). Estonia Hospital Inpatient Discharges 1989
Institute of Experimental and Clinical Medicine (Estonia). Estonia Hospital Inpatient Discharges 1990
Institute of Experimental and Clinical Medicine (Estonia). Estonia Hospital Inpatient Discharges 1991
Institute of Experimental and Clinical Medicine (Estonia). Estonia Hospital Inpatient Discharges 1992
Institute of Experimental and Clinical Medicine (Estonia). Estonia Hospital Inpatient Discharges 1993
Institute of Experimental and Clinical Medicine (Estonia). Estonia Hospital Inpatient Discharges 1994
Institute of Experimental and Clinical Medicine (Estonia). Estonia Hospital Inpatient Discharges 1995
Institute of Experimental and Clinical Medicine (Estonia). Estonia Hospital Inpatient Discharges 1996
Institute of Experimental and Clinical Medicine (Estonia). Estonia Hospital Inpatient Discharges 1997
Institute of Experimental and Clinical Medicine (Estonia). Estonia Hospital Inpatient Discharges 1998
Institute of Experimental and Clinical Medicine (Estonia). Estonia Hospital Inpatient Discharges 1999
Institute of Health Information and Statistics of the Czech Republic, International Research Associates (INRA) Europe, World Health Organization (WHO). Czech Republic WHO Multi-country Survey Study on Health and Health System Responsiveness 2000-2001. Geneva, Switzerland: World Health Organization (WHO)
Institute of Health Systems (India), World Health Organization (WHO). India - Andhra Pradesh WHO Multi-country Survey Study on Health and Health System Responsiveness 2000-2001
Institute of Public Health (Macedonia). Macedonia Hospital Inpatient Discharges 1980
Institute of Public Health (Macedonia). Macedonia Hospital Inpatient Discharges 1982
Institute of Public Health (Macedonia). Macedonia Hospital Inpatient Discharges 1984
Institute of Public Health (Macedonia). Macedonia Hospital Inpatient Discharges 1985
Institute of Public Health (Macedonia). Macedonia Hospital Inpatient Discharges 1986
Institute of Public Health (Macedonia). Macedonia Hospital Inpatient Discharges 1987
Institute of Public Health (Macedonia). Macedonia Hospital Inpatient Discharges 1989
Institute of Public Health (Macedonia). Macedonia Hospital Inpatient Discharges 1990
Institute of Public Health (Macedonia). Macedonia Hospital Inpatient Discharges 1991
Institute of Public Health (Macedonia). Macedonia Hospital Inpatient Discharges 1992
Institute of Public Health (Macedonia). Macedonia Hospital Inpatient Discharges 1993
Institute of Public Health (Macedonia). Macedonia Hospital Inpatient Discharges 1995
Institute of Public Health (Macedonia). Macedonia Hospital Inpatient Discharges 1996
Institute of Public Health (Macedonia). Macedonia Hospital Inpatient Discharges 1997
Institute of Public Health (Macedonia). Macedonia Hospital Inpatient Discharges 2000
Institute of Public Health (Macedonia). Macedonia Hospital Inpatient Discharges 2001
Institute of Public Health (Macedonia). Macedonia Hospital Inpatient Discharges 2002
Institute of Public Health (Macedonia). Macedonia Hospital Inpatient Discharges 2004

Institute of Public Health (Macedonia). Macedonia Hospital Inpatient Discharges 2012

Institute of Public Health of Serbia. Serbia Inpatient Care Discharges Per 100 2010

Institute of Public Health of Serbia. Serbia Inpatient Care Discharges Per 100 2011

Institute of Public Health of Serbia. Serbia Inpatient Care Discharges Per 100 2013

Institute of Public Health of Serbia. Serbia Inpatient Care Discharges Per 100 2014

Institute of Public Health of Serbia. Serbia National Hospital Discharge Database 2012

Institute of Social Medicine and Health Policy, Shandong University, Shandong University School of Medicine, World Health Organization (WHO). China WHO Multi-country Survey Study on Health and Health System Responsiveness 2000-2001

Institute of Sociology, Russian Academy of Sciences, Paragon Research, University of North Carolina, World Bank. Kyrgyzstan Living Standards Measurement Survey 1993. Washington DC, United States: World Bank

International Clearinghouse for Birth Defects Monitoring Systems. International Clearinghouse for Birth Defects Monitoring Systems Annual Report 2003. Rome, Italy: International Centre on Birth Defects

International Clearinghouse for Birth Defects Monitoring Systems. International Clearinghouse for Birth Defects Monitoring Systems Annual Report 2004. Rome, Italy: International Centre on Birth Defects, 2006

International Clearinghouse for Birth Defects Surveillance and Research. International Clearinghouse for Birth Defects Surveillance and Research Annual Report 2005. Rome, Italy: International Clearinghouse for Birth Defects Surveillance and Research, 2007

International Clearinghouse for Birth Defects Surveillance and Research. International Clearinghouse for Birth Defects Surveillance and Research Annual Report 2006. Rome, Italy: International Clearinghouse for Birth Defects Surveillance and Research, 2007

International Clearinghouse for Birth Defects Surveillance and Research. International Clearinghouse for Birth Defects Surveillance and Research Annual Report 2007. Rome, Italy: International Clearinghouse for Birth Defects Surveillance and Research, 2008

International Clearinghouse for Birth Defects Surveillance and Research. International Clearinghouse for Birth Defects Surveillance and Research Annual Report 2009. Rome, Italy: International Clearinghouse for Birth Defects Surveillance and Research

International Clearinghouse for Birth Defects Surveillance and Research. International Clearinghouse for Birth Defects Surveillance and Research Annual Report 2010. Rome, Italy: International Clearinghouse for Birth Defects Surveillance and Research, 2011

International Clearinghouse for Birth Defects Surveillance and Research. International Clearinghouse for Birth Defects Surveillance and Research Annual Report 2011. Rome, Italy: International Clearinghouse for Birth Defects Surveillance and Research, 2012

International Clearinghouse for Birth Defects Surveillance and Research. International Clearinghouse for Birth Defects Surveillance and Research Annual Report 2012. Rome, Italy: International Clearinghouse for Birth Defects Surveillance and Research, 2013

International Clearinghouse for Birth Defects Surveillance and Research. International Clearinghouse for Birth Defects Surveillance and Research Annual Report 2014. Rome, Italy: International Clearinghouse for Birth Defects Surveillance and Research, 2016

International Institute for Population Sciences (India), World Health Organization (WHO). India WHO Study on Global Ageing and Adult Health 2007. Geneva, Switzerland: World Health Organization (WHO), 2007

International Institute for Population Sciences (India), World Health Organization (WHO). India World Health Survey 2003. Geneva, Switzerland: World Health Organization (WHO), 2005

International Research Associates (INRA) Europe, National Research and Development Center for Welfare and Health (STAKES) (Finland), World Health Organization (WHO). Finland WHO Multi-country Survey Study on Health and Health System Responsiveness 2000-2001. Geneva, Switzerland: World Health Organization (WHO)

International Research Associates (INRA) Europe, Netherlands Organisation for Applied Scientific Research (TNO), World Health Organization (WHO). Netherlands WHO Multi-country Survey Study on Health and Health System Responsiveness 2000-2001. Geneva, Switzerland: World Health Organization (WHO)

International Research Associates (INRA) Europe, World Health Organization (WHO). Belgium WHO Multi-country Survey Study on Health and Health System Responsiveness 2000-2001. Geneva, Switzerland: World Health Organization (WHO)

International Research Associates (INRA) Europe, World Health Organization (WHO). Bulgaria WHO Multi-country Survey Study on Health and Health System Responsiveness 2000-2001. Geneva, Switzerland: World Health Organization (WHO)

International Research Associates (INRA) Europe, World Health Organization (WHO). Estonia WHO Multi-country Survey Study on Health and Health System Responsiveness 2000-2001. Geneva, Switzerland: World Health Organization (WHO)

International Research Associates (INRA) Europe, World Health Organization (WHO). Germany WHO Multi-country Survey Study on Health and Health System Responsiveness 2000-2001. Geneva, Switzerland: World Health Organization (WHO)

International Research Associates (INRA) Europe, World Health Organization (WHO). Iceland WHO Multi-country Survey Study on Health and Health System Responsiveness 2000-2001. Geneva, Switzerland: World Health Organization (WHO)

International Research Associates (INRA) Europe, World Health Organization (WHO). Ireland WHO Multi-country Survey Study on Health and Health System Responsiveness 2000-2001. Geneva, Switzerland: World Health Organization (WHO)

International Research Associates (INRA) Europe, World Health Organization (WHO). Italy WHO Multi-country Survey Study on Health and Health System Responsiveness 2000-2001. Geneva, Switzerland: World Health Organization (WHO)

International Research Associates (INRA) Europe, World Health Organization (WHO). Luxembourg WHO Multi-country Survey Study on Health and Health System Responsiveness 2000-2001. Geneva, Switzerland: World Health Organization (WHO)

International Research Associates (INRA) Europe, World Health Organization (WHO). Malta WHO Multi-country Survey Study on Health and Health System Responsiveness 2000-2001. Geneva, Switzerland: World Health Organization (WHO)

International Research Associates (INRA) Europe, World Health Organization (WHO). Portugal WHO Multi-country Survey Study on Health and Health System Responsiveness 2000-2001. Geneva, Switzerland: World Health Organization (WHO)

International Research Associates (INRA) Europe, World Health Organization (WHO). Romania WHO Multi-country Survey Study on Health and Health System Responsiveness 2000-2001. Geneva, Switzerland: World Health Organization (WHO)

International Research Associates (INRA) Europe, World Health Organization (WHO). Russia WHO Multi-country Survey Study on Health and Health System Responsiveness 2000-2001. Geneva, Switzerland: World Health Organization (WHO)

International Research Associates (INRA) Europe, World Health Organization (WHO). Spain WHO Multi-country Survey Study on Health and Health System Responsiveness 2000-2001. Geneva, Switzerland: World Health Organization (WHO)

International Research Associates (INRA) Europe, World Health Organization (WHO). Sweden WHO Multi-country Survey Study on Health and Health System Responsiveness 2000-2001. Geneva, Switzerland: World Health Organization (WHO)

Iran - Tabriz Registry of Congenital Anomalies Data 2004 - ICBDSR as it appears in International Clearinghouse for Birth Defects Surveillance and Research. International Clearinghouse for Birth Defects Surveillance and Research Annual Report 2006. Rome, Italy: International Clearinghouse for Birth Defects Surveillance and Research, 2007

Iran - Tabriz Registry of Congenital Anomalies Data 2005 - ICBDSR as it appears in International Clearinghouse for Birth Defects Surveillance and Research. International Clearinghouse for Birth Defects Surveillance and Research Annual Report 2007. Rome, Italy: International Clearinghouse for Birth Defects Surveillance and Research, 2008

Iran - Tabriz Registry of Congenital Anomalies Data 2007 - ICBDSR as it appears in International Clearinghouse for Birth Defects Surveillance and Research. International Clearinghouse for Birth Defects Surveillance and Research Annual Report 2009. Rome, Italy: International Clearinghouse for Birth Defects Surveillance and Research

Iran - Tabriz Registry of Congenital Anomalies Data 2008 - ICBDSR as it appears in International Clearinghouse for Birth Defects Surveillance and Research. International Clearinghouse for Birth Defects Surveillance and Research Annual Report 2010. Rome, Italy: International Clearinghouse for Birth Defects Surveillance and Research, 2011

Iran - Tabriz Registry of Congenital Anomalies Data 2009 - ICBDSR as it appears in International Clearinghouse for Birth Defects Surveillance and Research. International Clearinghouse for Birth Defects Surveillance and Research Annual Report 2011. Rome, Italy: International Clearinghouse for Birth Defects Surveillance and Research, 2012

Iran - Tabriz Registry of Congenital Anomalies Data 2010 - ICBDSR as it appears in International Clearinghouse for Birth Defects Surveillance and Research. International Clearinghouse for Birth Defects Surveillance and Research Annual Report 2012. Rome, Italy: International Clearinghouse for Birth Defects Surveillance and Research, 2013

Iran - Tabriz Registry of Congenital Anomalies Data 2012 - ICBDSR as it appears in International Clearinghouse for Birth Defects Surveillance and Research. International Clearinghouse for Birth Defects Surveillance and Research Annual Report 2014. Rome, Italy: International Clearinghouse for Birth Defects Surveillance and Research, 2016

Ireland EUROCAT Data 1993-1998 - WHO as it appears in European Surveillance of Congenital Anomalies (EUROCAT), International Centre on Birth Defects, World Health Organization (WHO). World Atlas of Birth Defects. 2nd ed. Geneva, Switzerland: World Health Organization (WHO), 2003

Ireland EUROCAT Data 2001 - ICBDMS as it appears in International Clearinghouse for Birth Defects Monitoring Systems. International Clearinghouse for Birth Defects Monitoring Systems Annual Report 2003. Rome, Italy: International Centre on Birth Defects

Ireland EUROCAT Data 2002 - ICBDMS as it appears in International Clearinghouse for Birth Defects Monitoring Systems. International Clearinghouse for Birth Defects Monitoring Systems Annual Report 2004. Rome, Italy: International Centre on Birth Defects, 2006

Ireland EUROCAT Data 2003 - ICBDSR as it appears in International Clearinghouse for Birth Defects Surveillance and Research. International Clearinghouse for Birth Defects Surveillance and Research Annual Report 2005. Rome, Italy: International Clearinghouse for Birth Defects Surveillance and Research, 2007

Ireland EUROCAT Data 2004 - ICBDSR as it appears in International Clearinghouse for Birth Defects Surveillance and Research. International Clearinghouse for Birth Defects Surveillance and Research Annual Report 2006. Rome, Italy: International Clearinghouse for Birth Defects Surveillance and Research, 2007

Ireland EUROCAT Data 2005 - ICBDSR as it appears in International Clearinghouse for Birth Defects Surveillance and Research. International Clearinghouse for Birth Defects Surveillance and Research Annual Report 2007. Rome, Italy: International Clearinghouse for Birth Defects Surveillance and Research, 2008

Ireland EUROCAT Data 2007 - ICBDSR as it appears in International Clearinghouse for Birth Defects Surveillance and Research. International Clearinghouse for Birth Defects Surveillance and Research Annual Report 2009. Rome, Italy: International Clearinghouse for Birth Defects Surveillance and Research

Ireland EUROCAT Data 2008 - ICBDSR as it appears in International Clearinghouse for Birth Defects Surveillance and Research. International Clearinghouse for Birth Defects Surveillance and Research Annual Report 2010. Rome, Italy: International Clearinghouse for Birth Defects Surveillance and Research, 2011

Ireland EUROCAT Data 2009 - ICBDSR as it appears in International Clearinghouse for Birth Defects Surveillance and Research. International Clearinghouse for Birth Defects Surveillance and Research Annual Report 2011. Rome, Italy: International Clearinghouse for Birth Defects Surveillance and Research, 2012

Ireland EUROCAT Data 2010 - ICBDSR as it appears in International Clearinghouse for Birth Defects Surveillance and Research. International Clearinghouse for Birth Defects Surveillance and Research Annual Report 2012. Rome, Italy: International Clearinghouse for Birth Defects Surveillance and Research, 2013

Israel Birth Defects Monitoring System Data 1993-1998 - WHO as it appears in European Surveillance of Congenital Anomalies (EUROCAT), International Centre on Birth Defects, World Health Organization (WHO). World Atlas of Birth Defects. 2nd ed. Geneva, Switzerland: World Health Organization (WHO), 2003

Israel Birth Defects Monitoring System Data 2001 - ICBDMS as it appears in International Clearinghouse for Birth Defects Monitoring Systems. International Clearinghouse for Birth Defects Monitoring Systems Annual Report 2003. Rome, Italy: International Centre on Birth Defects

Israel Birth Defects Monitoring System Data 2002 - ICBDMS as it appears in International Clearinghouse for Birth Defects Monitoring Systems. International Clearinghouse for Birth Defects Monitoring Systems Annual Report 2004. Rome, Italy: International Centre on Birth Defects, 2006

Israel Birth Defects Surveillance Program Data 2003 - ICBDSR as it appears in International Clearinghouse for Birth Defects Surveillance and Research. International Clearinghouse for Birth Defects Surveillance and Research Annual Report 2005. Rome, Italy: International Clearinghouse for Birth Defects Surveillance and Research, 2007

Israel Birth Defects Surveillance Program Data 2004 - ICBDSR as it appears in International Clearinghouse for Birth Defects Surveillance and Research. International Clearinghouse for Birth Defects Surveillance and Research Annual Report 2006. Rome, Italy: International Clearinghouse for Birth Defects Surveillance and Research, 2007

Israel Birth Defects Surveillance Program Data 2005 - ICBDSR as it appears in International Clearinghouse for Birth Defects Surveillance and Research. International Clearinghouse for Birth Defects Surveillance and Research Annual Report 2007. Rome, Italy: International Clearinghouse for Birth Defects Surveillance and Research, 2008

Israel Birth Defects Surveillance Program Data 2007 - ICBDSR as it appears in International Clearinghouse for Birth Defects Surveillance and Research. International Clearinghouse for Birth Defects Surveillance and Research Annual Report 2009. Rome, Italy: International Clearinghouse for Birth Defects Surveillance and Research

Israel Birth Defects Surveillance Program Data 2008 - ICBDSR as it appears in International Clearinghouse for Birth Defects Surveillance and Research. International Clearinghouse for Birth Defects Surveillance and Research Annual Report 2010. Rome, Italy: International Clearinghouse for Birth Defects Surveillance and Research, 2011

Israel Birth Defects Surveillance Program Data 2009 - ICBDSR as it appears in International Clearinghouse for Birth Defects Surveillance and Research. International Clearinghouse for Birth Defects Surveillance and Research Annual Report 2011. Rome, Italy: International Clearinghouse for Birth Defects Surveillance and Research, 2012

Israel Birth Defects Surveillance Program Data 2010 - ICBDSR as it appears in International Clearinghouse for Birth Defects Surveillance and Research. International Clearinghouse for Birth Defects Surveillance and Research Annual Report 2012. Rome, Italy: International Clearinghouse for Birth Defects Surveillance and Research, 2013

Italy - Campania Birth Defects Registry Data 1993-1998 - WHO as it appears in European Surveillance of Congenital Anomalies (EUROCAT), International Centre on Birth Defects, World Health Organization (WHO). World Atlas of Birth Defects. 2nd ed. Geneva, Switzerland: World Health Organization (WHO), 2003

Italy - Campania Birth Defects Registry Data 2001 - ICBDMS as it appears in International Clearinghouse for Birth Defects Monitoring Systems. International Clearinghouse for Birth Defects Monitoring Systems Annual Report 2003. Rome, Italy: International Centre on Birth Defects

Italy - Campania Birth Defects Registry Data 2002 - ICBDMS as it appears in International Clearinghouse for Birth Defects Monitoring Systems. International Clearinghouse for Birth Defects Monitoring Systems Annual Report 2004. Rome, Italy: International Centre on Birth Defects, 2006

Italy - Campania Birth Defects Registry Data 2003 - ICBDSR as it appears in International Clearinghouse for Birth Defects Surveillance and Research. International Clearinghouse for Birth Defects Surveillance and Research Annual Report 2005. Rome, Italy: International Clearinghouse for Birth Defects Surveillance and Research, 2007

Italy - Campania Birth Defects Registry Data 2004 - ICBDSR as it appears in International Clearinghouse for Birth Defects Surveillance and Research. International Clearinghouse for Birth Defects Surveillance and Research Annual Report 2006. Rome, Italy: International Clearinghouse for Birth Defects Surveillance and Research, 2007

Italy - Campania Birth Defects Registry Data 2005 - ICBDSR as it appears in International Clearinghouse for Birth Defects Surveillance and Research. International Clearinghouse for Birth Defects Surveillance and Research Annual Report 2007. Rome, Italy: International Clearinghouse for Birth Defects Surveillance and Research, 2008

Italy - Campania Birth Defects Registry Data 2007 - ICBDSR as it appears in International Clearinghouse for Birth Defects Surveillance and Research. International Clearinghouse for Birth Defects Surveillance and Research Annual Report 2009. Rome, Italy: International Clearinghouse for Birth Defects Surveillance and Research

Italy - Campania Birth Defects Registry Data 2008 - ICBDSR as it appears in International Clearinghouse for Birth Defects Surveillance and Research. International Clearinghouse for Birth Defects Surveillance and Research Annual Report 2010. Rome, Italy: International Clearinghouse for Birth Defects Surveillance and Research, 2011

Italy - Campania Birth Defects Registry Data 2009 - ICBDSR as it appears in International Clearinghouse for Birth Defects Surveillance and Research. International Clearinghouse for Birth Defects Surveillance and Research Annual Report 2011. Rome, Italy: International Clearinghouse for Birth Defects Surveillance and Research, 2012

Italy - Campania Birth Defects Registry Data 2010 - ICBDSR as it appears in International Clearinghouse for Birth Defects Surveillance and Research. International Clearinghouse for Birth Defects Surveillance and Research Annual Report 2012. Rome, Italy: International Clearinghouse for Birth Defects Surveillance and Research, 2013

Italy - Emilia-Romagna Registry of Congenital Malformations Data 1993-1998 - WHO as it appears in European Surveillance of Congenital Anomalies (EUROCAT), International Centre on Birth Defects, World Health Organization (WHO). World Atlas of Birth Defects. 2nd ed. Geneva, Switzerland: World Health Organization (WHO), 2003

Italy - Emilia-Romagna Registry of Congenital Malformations Data 2001 - ICBDMS as it appears in International Clearinghouse for Birth Defects Monitoring Systems. International Clearinghouse for Birth Defects Monitoring Systems Annual Report 2003. Rome, Italy: International Centre on Birth Defects

Italy - Emilia-Romagna Registry of Congenital Malformations Data 2002 - ICBDMS as it appears in International Clearinghouse for Birth Defects Monitoring Systems. International Clearinghouse for Birth Defects Monitoring Systems Annual Report 2004. Rome, Italy: International Centre on Birth Defects, 2006

Italy - Emilia-Romagna Registry of Congenital Malformations Data 2003 - ICBDSR as it appears in International Clearinghouse for Birth Defects Surveillance and Research. International Clearinghouse for Birth Defects Surveillance and Research Annual Report 2005. Rome, Italy: International Clearinghouse for Birth Defects Surveillance and Research, 2007

Italy - Emilia-Romagna Registry of Congenital Malformations Data 2004 - ICBDSR as it appears in International Clearinghouse for Birth Defects Surveillance and Research. International Clearinghouse for Birth Defects Surveillance and Research Annual Report 2006. Rome, Italy: International Clearinghouse for Birth Defects Surveillance and Research, 2007

Italy - Emilia-Romagna Registry of Congenital Malformations Data 2005 - ICBDSR as it appears in International Clearinghouse for Birth Defects Surveillance and Research. International Clearinghouse for Birth Defects Surveillance and Research Annual Report 2007. Rome, Italy: International Clearinghouse for Birth Defects Surveillance and Research, 2008
Italy - Emilia-Romagna Registry of Congenital Malformations Data 2007 - ICBDSR as it appears in International Clearinghouse for Birth Defects Surveillance and Research. International Clearinghouse for Birth Defects Surveillance and Research Annual Report 2009. Rome, Italy: International Clearinghouse for Birth Defects Surveillance and Research
Italy - Emilia-Romagna Registry of Congenital Malformations Data 2008 - ICBDSR as it appears in International Clearinghouse for Birth Defects Surveillance and Research. International Clearinghouse for Birth Defects Surveillance and Research Annual Report 2010. Rome, Italy: International Clearinghouse for Birth Defects Surveillance and Research, 2011
Italy - Emilia-Romagna Registry of Congenital Malformations Data 2009 - ICBDSR as it appears in International Clearinghouse for Birth Defects Surveillance and Research. International Clearinghouse for Birth Defects Surveillance and Research Annual Report 2011. Rome, Italy: International Clearinghouse for Birth Defects Surveillance and Research, 2012
Italy - Emilia-Romagna Registry of Congenital Malformations Data 2010 - ICBDSR as it appears in International Clearinghouse for Birth Defects Surveillance and Research. International Clearinghouse for Birth Defects Surveillance and Research Annual Report 2012. Rome, Italy: International Clearinghouse for Birth Defects Surveillance and Research, 2013
Italy - Lombardy Birth Defects Registry Data 2012 - ICBDSR as it appears in International Clearinghouse for Birth Defects Surveillance and Research. International Clearinghouse for Birth Defects Surveillance and Research Annual Report 2014. Rome, Italy: International Clearinghouse for Birth Defects Surveillance and Research, 2016
Italy - Lombardy Registry of Congenital Malformations Data 2008 - ICBDSR as it appears in International Clearinghouse for Birth Defects Surveillance and Research. International Clearinghouse for Birth Defects Surveillance and Research Annual Report 2010. Rome, Italy: International Clearinghouse for Birth Defects Surveillance and Research, 2011
Italy - North East Italy Registry of Congenital Malformations Data 1993-1998 - WHO as it appears in European Surveillance of Congenital Anomalies (EUROCAT), International Centre on Birth Defects, World Health Organization (WHO). World Atlas of Birth Defects. 2nd ed. Geneva, Switzerland: World Health Organization (WHO), 2003
Italy - North East Italy Registry of Congenital Malformations Data 2001 - ICBDMS as it appears in International Clearinghouse for Birth Defects Monitoring Systems. International Clearinghouse for Birth Defects Monitoring Systems Annual Report 2003. Rome, Italy: International Centre on Birth Defects
Italy - North East Italy Registry of Congenital Malformations Data 2002 - ICBDMS as it appears in International Clearinghouse for Birth Defects Monitoring Systems. International Clearinghouse for Birth Defects Monitoring Systems Annual Report 2004. Rome, Italy: International Centre on Birth Defects, 2006
Italy - North East Italy Registry of Congenital Malformations Data 2004 - ICBDSR as it appears in International Clearinghouse for Birth Defects Surveillance and Research. International Clearinghouse for Birth Defects Surveillance and Research Annual Report 2006. Rome, Italy: International Clearinghouse for Birth Defects Surveillance and Research, 2007
Italy - North East Italy Registry of Congenital Malformations Data 2005 - ICBDSR as it appears in International Clearinghouse for Birth Defects Surveillance and Research. International Clearinghouse for Birth Defects Surveillance and Research Annual Report 2007. Rome, Italy: International Clearinghouse for Birth Defects Surveillance and Research, 2008

Italy - North East Italy Registry of Congenital Malformations Data 2007 - ICBDSR as it appears in International Clearinghouse for Birth Defects Surveillance and Research. International Clearinghouse for Birth Defects Surveillance and Research Annual Report 2009. Rome, Italy: International Clearinghouse for Birth Defects Surveillance and Research
Italy - North East Italy Registry of Congenital Malformations Data 2009 - ICBDSR as it appears in International Clearinghouse for Birth Defects Surveillance and Research. International Clearinghouse for Birth Defects Surveillance and Research Annual Report 2011. Rome, Italy: International Clearinghouse for Birth Defects Surveillance and Research, 2012
Italy - Northeast Italy Registry of Congenital Defects Data 2003 - ICBDSR as it appears in International Clearinghouse for Birth Defects Surveillance and Research. International Clearinghouse for Birth Defects Surveillance and Research Annual Report 2005. Rome, Italy: International Clearinghouse for Birth Defects Surveillance and Research, 2007
Italy - Northern Lombardy Congenital Malformation Registry Data 2009 - ICBDSR as it appears in International Clearinghouse for Birth Defects Surveillance and Research. International Clearinghouse for Birth Defects Surveillance and Research Annual Report 2011. Rome, Italy: International Clearinghouse for Birth Defects Surveillance and Research, 2012
Italy - Northern Lombardy Congenital Malformation Registry Data 2010 - ICBDSR as it appears in International Clearinghouse for Birth Defects Surveillance and Research. International Clearinghouse for Birth Defects Surveillance and Research Annual Report 2012. Rome, Italy: International Clearinghouse for Birth Defects Surveillance and Research, 2013
Italy - Sicilian Registry of Congenital Malformations Data 1993-1998 - WHO as it appears in European Surveillance of Congenital Anomalies (EUROCAT), International Centre on Birth Defects, World Health Organization (WHO). World Atlas of Birth Defects. 2nd ed. Geneva, Switzerland: World Health Organization (WHO), 2003
Italy - Sicilian Registry of Congenital Malformations Data 2001 - ICBDMS as it appears in International Clearinghouse for Birth Defects Monitoring Systems. International Clearinghouse for Birth Defects Monitoring Systems Annual Report 2003. Rome, Italy: International Centre on Birth Defects
Italy - Sicilian Registry of Congenital Malformations Data 2002 - ICBDMS as it appears in International Clearinghouse for Birth Defects Monitoring Systems. International Clearinghouse for Birth Defects Monitoring Systems Annual Report 2004. Rome, Italy: International Centre on Birth Defects, 2006
Italy - Sicilian Registry of Congenital Malformations Data 2003 - ICBDSR as it appears in International Clearinghouse for Birth Defects Surveillance and Research. International Clearinghouse for Birth Defects Surveillance and Research Annual Report 2005. Rome, Italy: International Clearinghouse for Birth Defects Surveillance and Research, 2007
Italy - Sicilian Registry of Congenital Malformations Data 2004 - ICBDSR as it appears in International Clearinghouse for Birth Defects Surveillance and Research. International Clearinghouse for Birth Defects Surveillance and Research Annual Report 2006. Rome, Italy: International Clearinghouse for Birth Defects Surveillance and Research, 2007
Italy - Sicilian Registry of Congenital Malformations Data 2005 - ICBDSR as it appears in International Clearinghouse for Birth Defects Surveillance and Research. International Clearinghouse for Birth Defects Surveillance and Research Annual Report 2007. Rome, Italy: International Clearinghouse for Birth Defects Surveillance and Research, 2008
Italy - Tuscany Registry of Congenital Defects Data 1993-1998 - WHO as it appears in European Surveillance of Congenital Anomalies (EUROCAT), International Centre on Birth Defects, World Health Organization (WHO). World Atlas of Birth Defects. 2nd ed. Geneva, Switzerland: World Health Organization (WHO), 2003
Italy - Tuscany Registry of Congenital Defects Data 2001 - ICBDMS as it appears in International Clearinghouse for Birth Defects Monitoring Systems. International Clearinghouse for Birth Defects Monitoring Systems Annual Report 2003. Rome, Italy: International Centre on Birth Defects

Italy - Tuscany Registry of Congenital Defects Data 2002 - ICBDMS as it appears in International Clearinghouse for Birth Defects Monitoring Systems. International Clearinghouse for Birth Defects Monitoring Systems Annual Report 2004. Rome, Italy: International Centre on Birth Defects, 2006

Italy - Tuscany Registry of Congenital Defects Data 2003 - ICBDSR as it appears in International Clearinghouse for Birth Defects Surveillance and Research. International Clearinghouse for Birth Defects Surveillance and Research Annual Report 2005. Rome, Italy: International Clearinghouse for Birth Defects Surveillance and Research, 2007

Italy - Tuscany Registry of Congenital Defects Data 2004 - ICBDSR as it appears in International Clearinghouse for Birth Defects Surveillance and Research. International Clearinghouse for Birth Defects Surveillance and Research Annual Report 2006. Rome, Italy: International Clearinghouse for Birth Defects Surveillance and Research, 2007

Italy - Tuscany Registry of Congenital Defects Data 2005 - ICBDSR as it appears in International Clearinghouse for Birth Defects Surveillance and Research. International Clearinghouse for Birth Defects Surveillance and Research Annual Report 2007. Rome, Italy: International Clearinghouse for Birth Defects Surveillance and Research, 2008

Italy - Tuscany Registry of Congenital Defects Data 2007 - ICBDSR as it appears in International Clearinghouse for Birth Defects Surveillance and Research. International Clearinghouse for Birth Defects Surveillance and Research Annual Report 2009. Rome, Italy: International Clearinghouse for Birth Defects Surveillance and Research

Italy - Tuscany Registry of Congenital Defects Data 2008 - ICBDSR as it appears in International Clearinghouse for Birth Defects Surveillance and Research. International Clearinghouse for Birth Defects Surveillance and Research Annual Report 2010. Rome, Italy: International Clearinghouse for Birth Defects Surveillance and Research, 2011

Italy - Tuscany Registry of Congenital Defects Data 2009 - ICBDSR as it appears in International Clearinghouse for Birth Defects Surveillance and Research. International Clearinghouse for Birth Defects Surveillance and Research Annual Report 2011. Rome, Italy: International Clearinghouse for Birth Defects Surveillance and Research, 2012

Italy - Tuscany Registry of Congenital Defects Data 2010 - ICBDSR as it appears in International Clearinghouse for Birth Defects Surveillance and Research. International Clearinghouse for Birth Defects Surveillance and Research Annual Report 2012. Rome, Italy: International Clearinghouse for Birth Defects Surveillance and Research, 2013

Italy - Tuscany Registry of Congenital Defects Data 2012 - ICBDSR as it appears in International Clearinghouse for Birth Defects Surveillance and Research. International Clearinghouse for Birth Defects Surveillance and Research Annual Report 2014. Rome, Italy: International Clearinghouse for Birth Defects Surveillance and Research, 2016

Itoh H, Koike A, Taniguchi K, Marumo F. Severity and pathophysiology of heart failure on the basis of anaerobic threshold (AT) and related parameters. Jpn Circ J. 1989; 53(2): 146-54

Jacobs EG, Leung MP, Karlberg J. Distribution of symptomatic congenital heart disease in Hong Kong. Pediatr Cardiol. 2000; 21(2): 148-57

Japan Association of Obstetricians and Gynaecologists Congenital Anomaly Data 2001 - ICBDMS as it appears in International Clearinghouse for Birth Defects Monitoring Systems. International Clearinghouse for Birth Defects Monitoring Systems Annual Report 2003. Rome, Italy: International Centre on Birth Defects

Japan Association of Obstetricians and Gynaecologists Congenital Anomaly Data 2002 - ICBDMS as it appears in International Clearinghouse for Birth Defects Monitoring Systems. International Clearinghouse for Birth Defects Monitoring Systems Annual Report 2004. Rome, Italy: International Centre on Birth Defects, 2006

Japan Association of Obstetricians and Gynaecologists Congenital Anomaly Data 2003 - ICBDSR as it appears in International Clearinghouse for Birth Defects Surveillance and Research. International Clearinghouse for Birth Defects Surveillance and Research Annual Report 2005. Rome, Italy: International Clearinghouse for Birth Defects Surveillance and Research, 2007

Japan Association of Obstetricians and Gynaecologists Congenital Anomaly Data 2004 - ICBDSR as it appears in International Clearinghouse for Birth Defects Surveillance and Research. International Clearinghouse for Birth Defects Surveillance and Research Annual Report 2006. Rome, Italy: International Clearinghouse for Birth Defects Surveillance and Research, 2007

Japan Association of Obstetricians and Gynaecologists Congenital Anomaly Data 2005 - ICBDSR as it appears in International Clearinghouse for Birth Defects Surveillance and Research. International Clearinghouse for Birth Defects Surveillance and Research Annual Report 2007. Rome, Italy: International Clearinghouse for Birth Defects Surveillance and Research, 2008

Japan Association of Obstetricians and Gynaecologists Congenital Anomaly Data 2007 - ICBDSR as it appears in International Clearinghouse for Birth Defects Surveillance and Research. International Clearinghouse for Birth Defects Surveillance and Research Annual Report 2009. Rome, Italy: International Clearinghouse for Birth Defects Surveillance and Research

Japan Association of Obstetricians and Gynaecologists Congenital Anomaly Data 2008 - ICBDSR as it appears in International Clearinghouse for Birth Defects Surveillance and Research. International Clearinghouse for Birth Defects Surveillance and Research Annual Report 2010. Rome, Italy: International Clearinghouse for Birth Defects Surveillance and Research, 2011

Japan Association of Obstetricians and Gynaecologists Congenital Anomaly Data 2009 - ICBDSR as it appears in International Clearinghouse for Birth Defects Surveillance and Research. International Clearinghouse for Birth Defects Surveillance and Research Annual Report 2011. Rome, Italy: International Clearinghouse for Birth Defects Surveillance and Research, 2012

Japan Association of Obstetricians and Gynaecologists Congenital Anomaly Data 2010 - ICBDSR as it appears in International Clearinghouse for Birth Defects Surveillance and Research. International Clearinghouse for Birth Defects Surveillance and Research Annual Report 2012. Rome, Italy: International Clearinghouse for Birth Defects Surveillance and Research, 2013

Japan Association of Obstetricians and Gynaecologists Congenital Anomaly Data 2012 - ICBDSR as it appears in International Clearinghouse for Birth Defects Surveillance and Research. International Clearinghouse for Birth Defects Surveillance and Research Annual Report 2014. Rome, Italy: International Clearinghouse for Birth Defects Surveillance and Research, 2016

Johnson KC, Rouleau J. Temporal trends in Canadian birth defects birth prevalences, 1979-1993. Can J Public Health. 1997; 88(3): 169-76

Kang G, Xiao J, Wang J, Chen J, Li W, Wang Y, Liu Q, Wang Z, Xia J, Huang J, Cheng L, Chen Y, Chen Q, Yang F. Congenital Heart Disease in Local and Migrant Elementary Schoolchildren in Dongguan, China. Am J Cardiol. 2016; 117(3): 461-4

Kang GS, Soh YF, Kofidis T, Lee CN. Five-year experience with congenital cardiac surgery at National University Heart Centre, Singapore. Singapore Med J. 2010; 51(7): 570-5

Kapoor R, Gupta S. Prevalence of congenital heart disease, Kanpur, India. Indian Pediatr. 2008; 45(4): 309-11

Karaye KM, Sani MU. Factors associated with poor prognosis among patients admitted with heart failure in a Nigerian tertiary medical centre: a cross-sectional study. BMC Cardiovasc Disord. 2008; 8(1): 16

Khairy P, Ionescu-Ittu R, Mackie AS, Abrahamowicz M, Pilote L, Marelli AJ. Changing mortality in congenital heart disease. J Am Coll Cardiol. 2010; 56(14): 1149-57

Khalil A, Aggarwal R, Thirupuram S, Arora R. Incidence of congenital heart disease among hospital live births in India. Indian Pediatr. 1994; 31(5): 519-27

Kidd SA, Lancaster PA, McCredie RM. The incidence of congenital heart defects in the first year of life. J Paediatr Child Health. 1993; 29(5): 344-9

Kiev International Institute of Sociology, World Health Organization (WHO). Ukraine WHO Multi-country Survey Study on Health and Health System Responsiveness 2000-2001. Geneva, Switzerland: World Health Organization (WHO)

Kingue S, Dzudie A, Menanga A, Akono M, Ouankou M, Muna W. Nouveau regard sur l'insuffisance cardiaque chronique de l'adulte en Afrique à l'ère de l'échocardiographie Doppler : expérience du service de médecine de l'Hôpital Général de Yaoundé. Ann Cardiol Angeiol (Paris). 2005; 54(5): 276-83

Kirklin JW, Pacifico AD, Blackstone EH, Kirklin JK, Bargeron LM Jr. Current risks and protocols for operations for double-outlet right ventricle. Derivation from an 18 year experience. J Thorac Cardiovasc Surg. 1986; 92(5): 913-30

Knauth AL, Lock JE, Perry SB, McElhinney DB, Gauvreau K, Landzberg MJ, Rome JJ, Hellenbrand WE, Ruiz CE, Jenkins KJ. Transcatheter device closure of congenital and postoperative residual ventricular septal defects. Circulation. 2004; 110(5): 501-7

Kuehl KS, Loffredo CA, Ferencz C. Failure to diagnose congenital heart disease in infancy. Pediatrics. 1999; 103(4 Pt 1): 743-7

Kumar SP, Rubinstein CS, Simsic JM, Taylor AB, Saul JP, Bradley SM. Lateral tunnel versus extracardiac conduit Fontan procedure: a concurrent comparison. Ann Thorac Surg. 2003; 76(5): 1389-97

Lalljie GR, Lalljie SE. Characteristics, treatment and short-term survival of patients with heart failure in a cardiology private practice in Jamaica. West Indian Med J. 2007; 56(2): 139-43

Larrazabal LA, Jenkins KJ, Gauvreau K, Vida VL, Benavidez OJ, Gaitán GA, Garcia F, Castañeda AR. Improvement in congenital heart surgery in a developing country: the Guatemalan experience. Circulation. 2007; 116(17): 1882-7

Latin American Collaborative Study of Congenital Malformations Data 2001 - ICBDMS as it appears in International Clearinghouse for Birth Defects Monitoring Systems. International Clearinghouse for Birth Defects Monitoring Systems Annual Report 2003. Rome, Italy: International Centre on Birth Defects

Latin American Collaborative Study of Congenital Malformations Data 2002 - ICBDMS as it appears in International Clearinghouse for Birth Defects Monitoring Systems. International Clearinghouse for Birth Defects Monitoring Systems Annual Report 2004. Rome, Italy: International Centre on Birth Defects, 2006

Latin American Collaborative Study of Congenital Malformations Data 2003 - ICBDSR as it appears in International Clearinghouse for Birth Defects Surveillance and Research. International Clearinghouse for Birth Defects Surveillance and Research Annual Report 2005. Rome, Italy: International Clearinghouse for Birth Defects Surveillance and Research, 2007

Latin American Collaborative Study of Congenital Malformations Data 2004 - ICBDSR as it appears in International Clearinghouse for Birth Defects Surveillance and Research. International Clearinghouse for Birth Defects Surveillance and Research Annual Report 2006. Rome, Italy: International Clearinghouse for Birth Defects Surveillance and Research, 2007

Latin American Collaborative Study of Congenital Malformations Data 2005 - ICBDSR as it appears in International Clearinghouse for Birth Defects Surveillance and Research. International Clearinghouse for Birth Defects Surveillance and Research Annual Report 2007. Rome, Italy: International Clearinghouse for Birth Defects Surveillance and Research, 2008

Latin American Collaborative Study of Congenital Malformations Data 2007 - ICBDSR as it appears in International Clearinghouse for Birth Defects Surveillance and Research. International Clearinghouse for Birth Defects Surveillance and Research Annual Report 2009. Rome, Italy: International Clearinghouse for Birth Defects Surveillance and Research

Latin American Collaborative Study of Congenital Malformations Data 2008 - ICBDSR as it appears in International Clearinghouse for Birth Defects Surveillance and Research. International Clearinghouse for Birth Defects Surveillance and Research Annual Report 2010. Rome, Italy: International Clearinghouse for Birth Defects Surveillance and Research, 2011

Latin American Collaborative Study of Congenital Malformations Data 2009 - ICBDSR as it appears in International Clearinghouse for Birth Defects Surveillance and Research. International Clearinghouse for Birth Defects Surveillance and Research Annual Report 2011. Rome, Italy: International Clearinghouse for Birth Defects Surveillance and Research, 2012

Latin American Collaborative Study of Congenital Malformations Data 2010 - ICBDSR as it appears in International Clearinghouse for Birth Defects Surveillance and Research. International Clearinghouse for Birth Defects Surveillance and Research Annual Report 2012. Rome, Italy: International Clearinghouse for Birth Defects Surveillance and Research, 2013

Latin American Collaborative Study of Congenital Malformations Data 2012 - ICBDSR as it appears in International Clearinghouse for Birth Defects Surveillance and Research. International Clearinghouse for Birth Defects Surveillance and Research Annual Report 2014. Rome, Italy: International Clearinghouse for Birth Defects Surveillance and Research, 2016

Lee JE, Jung K-L, Kim S-E, Nam S-H, Choi S-J, Oh S-Y, Roh C-R, Kim J-H. Prenatal diagnosis of congenital heart disease: trends in pregnancy termination rate, and perinatal and 1-year infant mortalities in Korea between 1994 and 2005. J Obstet Gynaecol Res. 2010; 36(3): 474-8

Lee MG, Brizard CP, Galati JC, Iyengar AJ, Rakhra SS, Konstantinov IE, Pflaumer A, d'Udekem Y. Outcomes of patients born with single-ventricle physiology and aortic arch obstruction: the 26-year Melbourne experience. J Thorac Cardiovasc Surg. 2014; 148(1): 194-201

Leite DCF, de Mendonça JT, Cipolotti R, de Melo EV. Heart defects treatment in Sergipe: propose of resources' rationalization to improve care. Rev Bras Cir Cardiovasc. 2012; 27(2): 224-30

Lim C, Lee JY, Kim W-H, Kim S-C, Song J-Y, Kim S-J, Choh J-H, Kim CW. Early replacement of pulmonary valve after repair of tetralogy: is it really beneficial?. Eur J Cardiothorac Surg. 2004; 25(5): 728-34

LINK Institute for Market and Social Research (Switzerland), World Health Organization (WHO). Switzerland WHO Multi-country Survey Study on Health and Health System Responsiveness 2000-2001. Geneva, Switzerland: World Health Organization (WHO)

Liu QG, Sun J, Xiao XW, Song GR. Birth defects data from surveillance hospitals in Dalian city, China, 2006-2010. J Matern Fetal Neonatal Med. 2016; 29(22): 3615-21

Lu X, Wu S, Gu X, Li L, Zhang G, Sun W, Yu J. Long-term results of surgical treatment of tetralogy of Fallot in adults. Thorac Cardiovasc Surg. 2006; 54(5): 295-9

Mahidol University, World Health Organization (WHO). Thailand WHO Multi-country Survey Study on Health and Health System Responsiveness 2000-2001. Geneva, Switzerland: World Health Organization (WHO)

Mai CT, Isenburg J, Langlois PH, Alverson CJ, Gilboa SM, Rickard R, Canfield MA, Anjohrin SB, Lupo PJ, Jackson DR, Stallings EB, Scheuerle AE, Kirby RS, National Birth Defects Prevention Network. Population-Based Birth Defects Data in the United States, 2008 to 2012: Presentation of State-Specific Data and Descriptive Brief on Variability of Prevalence. Birth Defects Res A Clin Mol Teratol. 2015; 103(11): 972-93

Malta Congenital Anomalies Register Data 1993-1998 - WHO as it appears in European Surveillance of Congenital Anomalies (EUROCAT), International Centre on Birth Defects, World Health Organization (WHO). World Atlas of Birth Defects. 2nd ed. Geneva, Switzerland: World Health Organization (WHO), 2003

Malta Congenital Anomalies Register Data 2001 - ICBDMS as it appears in International Clearinghouse for Birth Defects Monitoring Systems. International Clearinghouse for Birth Defects Monitoring Systems Annual Report 2003. Rome, Italy: International Centre on Birth Defects

Malta Congenital Anomalies Register Data 2002 - ICBDMS as it appears in International Clearinghouse for Birth Defects Monitoring Systems. International Clearinghouse for Birth Defects Monitoring Systems Annual Report 2004. Rome, Italy: International Centre on Birth Defects, 2006

Malta Congenital Anomalies Register Data 2003 - ICBDSR as it appears in International Clearinghouse for Birth Defects Surveillance and Research. International Clearinghouse for Birth Defects Surveillance and Research Annual Report 2005. Rome, Italy: International Clearinghouse for Birth Defects Surveillance and Research, 2007

Malta Congenital Anomalies Register Data 2004 - ICBDSR as it appears in International Clearinghouse for Birth Defects Surveillance and Research. International Clearinghouse for Birth Defects Surveillance and Research Annual Report 2006. Rome, Italy: International Clearinghouse for Birth Defects Surveillance and Research, 2007

Malta Congenital Anomalies Register Data 2005 - ICBDSR as it appears in International Clearinghouse for Birth Defects Surveillance and Research. International Clearinghouse for Birth Defects Surveillance and Research Annual Report 2007. Rome, Italy: International Clearinghouse for Birth Defects Surveillance and Research, 2008

Malta Congenital Anomalies Register Data 2007 - ICBDSR as it appears in International Clearinghouse for Birth Defects Surveillance and Research. International Clearinghouse for Birth Defects Surveillance and Research Annual Report 2009. Rome, Italy: International Clearinghouse for Birth Defects Surveillance and Research

Malta Congenital Anomalies Register Data 2008 - ICBDSR as it appears in International Clearinghouse for Birth Defects Surveillance and Research. International Clearinghouse for Birth Defects Surveillance and Research Annual Report 2010. Rome, Italy: International Clearinghouse for Birth Defects Surveillance and Research, 2011

Malta Congenital Anomalies Register Data 2009 - ICBDSR as it appears in International Clearinghouse for Birth Defects Surveillance and Research. International Clearinghouse for Birth Defects Surveillance and Research Annual Report 2011. Rome, Italy: International Clearinghouse for Birth Defects Surveillance and Research, 2012

Malta Congenital Anomalies Register Data 2010 - ICBDSR as it appears in International Clearinghouse for Birth Defects Surveillance and Research. International Clearinghouse for Birth Defects Surveillance and Research Annual Report 2012. Rome, Italy: International Clearinghouse for Birth Defects Surveillance and Research, 2013

Market, Media, and Public Opinion Research (Croatia), World Health Organization (WHO). Croatia WHO Multi-country Survey Study on Health and Health System Responsiveness 2000-2001. Geneva, Switzerland: World Health Organization (WHO)

Masura J, Gavora P, Podnar T. Long-term outcome of transcatheter secundum-type atrial septal defect closure using Amplatzer septal occluders. J Am Coll Cardiol. 2005; 45(4): 505-7

Mavroudis C, Backer CL, Gevitz M. Forty-six years of patient ductus arteriosus division at Children's Memorial Hospital of Chicago. Standards for comparison. Ann Surg. 1994; 220(3): 402-10

Mayosi BM. Contemporary trends in the epidemiology and management of cardiomyopathy and pericarditis in sub-Saharan Africa. Heart. 2007; 93(10): 1176 -1183

McBride KL, Marengo L, Canfield M, Langlois P, Fixler D, Belmont JW. Epidemiology of noncomplex left ventricular outflow tract obstruction malformations (aortic valve stenosis, coarctation of the aorta, hypoplastic left heart syndrome) in Texas, 1999-2001. Birth Defects Res A Clin Mol Teratol. 2005; 73(8): 555-61

McGrath LB, Gonzalez-Lavin L. Actuarial survival, freedom from reoperation, and other events after repair of atrioventricular septal defects. J Thorac Cardiovasc Surg. 1987; 94(4): 582-90

McSwain M, Martin TC, Amaraswamy R. The prevalence, aetiology and treatment of congestive cardiac failure in Antigua and Barbuda. West Indian Med J. 1999; 48(3): 137-40

Medeiros A, Biagi DG, Sobreira TJP, de Oliveira PSL, Negrão CE, Mansur AJ, Krieger JE, Brum PC, Pereira AC. Mutations in the human phospholamban gene in patients with heart failure. Am Heart J. 2011; 162(6): 1088-1095

MEMRB International, World Health Organization (WHO). Cyprus WHO Multi-country Survey Study on Health and Health System Responsiveness 2000-2001. Geneva, Switzerland: World Health Organization (WHO)

Mexican Registry and Epidemiological Surveillance of External Congenital Malformations Data 1993-1998 - WHO as it appears in European Surveillance of Congenital Anomalies (EUROCAT), International Centre on Birth Defects, World Health Organization (WHO). World Atlas of Birth Defects. 2nd ed. Geneva, Switzerland: World Health Organization (WHO), 2003
Mexican Registry and Epidemiological Surveillance of External Congenital Malformations Data 2001 - ICBDMS as it appears in International Clearinghouse for Birth Defects Monitoring Systems. International Clearinghouse for Birth Defects Monitoring Systems Annual Report 2003. Rome, Italy: International Centre on Birth Defects
Mexican Registry and Epidemiological Surveillance of External Congenital Malformations Data 2002 - ICBDMS as it appears in International Clearinghouse for Birth Defects Monitoring Systems. International Clearinghouse for Birth Defects Monitoring Systems Annual Report 2004. Rome, Italy: International Centre on Birth Defects, 2006
Mexican Registry and Epidemiological Surveillance of External Congenital Malformations Data 2003 - ICBDSR as it appears in International Clearinghouse for Birth Defects Surveillance and Research. International Clearinghouse for Birth Defects Surveillance and Research Annual Report 2005. Rome, Italy: International Clearinghouse for Birth Defects Surveillance and Research, 2007
Mexican Registry and Epidemiological Surveillance of External Congenital Malformations Data 2004 - ICBDSR as it appears in International Clearinghouse for Birth Defects Surveillance and Research. International Clearinghouse for Birth Defects Surveillance and Research Annual Report 2006. Rome, Italy: International Clearinghouse for Birth Defects Surveillance and Research, 2007
Mexican Registry and Epidemiological Surveillance of External Congenital Malformations Data 2005 - ICBDSR as it appears in International Clearinghouse for Birth Defects Surveillance and Research. International Clearinghouse for Birth Defects Surveillance and Research Annual Report 2007. Rome, Italy: International Clearinghouse for Birth Defects Surveillance and Research, 2008
Mexican Registry and Epidemiological Surveillance of External Congenital Malformations Data 2007 - ICBDSR as it appears in International Clearinghouse for Birth Defects Surveillance and Research. International Clearinghouse for Birth Defects Surveillance and Research Annual Report 2009. Rome, Italy: International Clearinghouse for Birth Defects Surveillance and Research
Mexican Registry and Epidemiological Surveillance of External Congenital Malformations Data 2008 - ICBDSR as it appears in International Clearinghouse for Birth Defects Surveillance and Research. International Clearinghouse for Birth Defects Surveillance and Research Annual Report 2010. Rome, Italy: International Clearinghouse for Birth Defects Surveillance and Research, 2011
Mexican Registry and Epidemiological Surveillance of External Congenital Malformations Data 2009 - ICBDSR as it appears in International Clearinghouse for Birth Defects Surveillance and Research. International Clearinghouse for Birth Defects Surveillance and Research Annual Report 2011. Rome, Italy: International Clearinghouse for Birth Defects Surveillance and Research, 2012
Mexican Registry and Epidemiological Surveillance of External Congenital Malformations Data 2010 - ICBDSR as it appears in International Clearinghouse for Birth Defects Surveillance and Research. International Clearinghouse for Birth Defects Surveillance and Research Annual Report 2012. Rome, Italy: International Clearinghouse for Birth Defects Surveillance and Research, 2013
Mexico - Monterrey Birth Defects Surveillance Program Data 2012 - ICBDSR as it appears in International Clearinghouse for Birth Defects Surveillance and Research. International Clearinghouse for Birth Defects Surveillance and Research Annual Report 2014. Rome, Italy: International Clearinghouse for Birth Defects Surveillance and Research, 2016
Mexico Registry and Epidemiological Surveillance of External Congenital Malformations Data 2012 - ICBDSR as it appears in International Clearinghouse for Birth Defects Surveillance and Research. International Clearinghouse for Birth Defects Surveillance and Research Annual Report 2014. Rome, Italy: International Clearinghouse for Birth Defects Surveillance and Research, 2016

Miller A, Siffel C, Lu C, Riehle-Colarusso T, Frías JL, Correa A. Long-term survival of infants with atrioventricular septal defects. J Pediatr. 2010; 156(6): 994-1000
Ministry of Health (Albania). Albania Inpatient Care Discharges per 100 1993
Ministry of Health (Albania). Albania Inpatient Care Discharges per 100 1994
Ministry of Health (Albania). Albania Inpatient Care Discharges per 100 1995
Ministry of Health (Albania). Albania Inpatient Care Discharges per 100 1996
Ministry of Health (Albania). Albania Inpatient Care Discharges per 100 1997
Ministry of Health (Albania). Albania Inpatient Care Discharges per 100 1998
Ministry of Health (Albania). Albania Inpatient Care Discharges per 100 1999
Ministry of Health (Albania). Albania Inpatient Care Discharges per 100 2000
Ministry of Health (Albania). Albania Inpatient Care Discharges per 100 2001
Ministry of Health (Albania). Albania Inpatient Care Discharges per 100 2002
Ministry of Health (Albania). Albania Inpatient Care Discharges per 100 2003
Ministry of Health (Albania). Albania Inpatient Care Discharges per 100 2004
Ministry of Health (Albania). Albania Inpatient Care Discharges per 100 2005
Ministry of Health (Albania). Albania Inpatient Care Discharges per 100 2006
Ministry of Health (Albania). Albania Inpatient Care Discharges per 100 2007
Ministry of Health (Albania). Albania Inpatient Care Discharges per 100 2008
Ministry of Health (Albania). Albania Inpatient Care Discharges per 100 2009
Ministry of Health (Albania). Albania Inpatient Care Discharges per 100 2010
Ministry of Health (Albania). Albania Inpatient Care Discharges per 100 2011
Ministry of Health (Albania). Albania Inpatient Care Discharges per 100 2012
Ministry of Health (Albania). Albania Inpatient Care Discharges per 100 2013
Ministry of Health (Armenia). Armenia Inpatient Care Discharges per 100 1980
Ministry of Health (Armenia). Armenia Inpatient Care Discharges per 100 1981
Ministry of Health (Armenia). Armenia Inpatient Care Discharges per 100 1982
Ministry of Health (Armenia). Armenia Inpatient Care Discharges per 100 1983
Ministry of Health (Armenia). Armenia Inpatient Care Discharges per 100 1984
Ministry of Health (Armenia). Armenia Inpatient Care Discharges per 100 1985
Ministry of Health (Armenia). Armenia Inpatient Care Discharges per 100 1986
Ministry of Health (Armenia). Armenia Inpatient Care Discharges per 100 1987
Ministry of Health (Armenia). Armenia Inpatient Care Discharges per 100 1988
Ministry of Health (Armenia). Armenia Inpatient Care Discharges per 100 1989
Ministry of Health (Armenia). Armenia Inpatient Care Discharges per 100 1990
Ministry of Health (Armenia). Armenia Inpatient Care Discharges per 100 1991
Ministry of Health (Armenia). Armenia Inpatient Care Discharges per 100 1992

Ministry of Health (Armenia). Armenia Inpatient Care Discharges per 100 1993
Ministry of Health (Armenia). Armenia Inpatient Care Discharges per 100 1994
Ministry of Health (Armenia). Armenia Inpatient Care Discharges per 100 1995
Ministry of Health (Armenia). Armenia Inpatient Care Discharges per 100 1996
Ministry of Health (Armenia). Armenia Inpatient Care Discharges per 100 1997
Ministry of Health (Armenia). Armenia Inpatient Care Discharges per 100 1998
Ministry of Health (Armenia). Armenia Inpatient Care Discharges per 100 1999
Ministry of Health (Armenia). Armenia Inpatient Care Discharges per 100 2000
Ministry of Health (Armenia). Armenia Inpatient Care Discharges per 100 2001
Ministry of Health (Armenia). Armenia Inpatient Care Discharges per 100 2002
Ministry of Health (Armenia). Armenia Inpatient Care Discharges per 100 2003
Ministry of Health (Armenia). Armenia Inpatient Care Discharges per 100 2004
Ministry of Health (Armenia). Armenia Inpatient Care Discharges per 100 2005
Ministry of Health (Armenia). Armenia Inpatient Care Discharges per 100 2006
Ministry of Health (Armenia). Armenia Inpatient Care Discharges per 100 2007
Ministry of Health (Armenia). Armenia Inpatient Care Discharges per 100 2008
Ministry of Health (Armenia). Armenia Inpatient Care Discharges per 100 2009
Ministry of Health (Armenia). Armenia Inpatient Care Discharges per 100 2010
Ministry of Health (Armenia). Armenia Inpatient Care Discharges per 100 2011
Ministry of Health (Armenia). Armenia Inpatient Care Discharges per 100 2012
Ministry of Health (Armenia). Armenia Inpatient Care Discharges per 100 2013
Ministry of Health (Armenia). Armenia Inpatient Care Discharges per 100 2014
Ministry of Health (Brazil). Brazil Hospital Information System 1997. Rio de Janeiro, Brazil: Ministry of Health (Brazil)
Ministry of Health (Brazil). Brazil Hospital Information System 1998-2002
Ministry of Health (Brazil). Brazil Hospital Information System 2003-2007
Ministry of Health (Brazil). Brazil Hospital Information System 2008-2012
Ministry of Health (Brazil). Brazil Hospital Information System 2013-2014
Ministry of Health (Chile). Chile Hospital Discharges 2001. Santiago, Chile: Ministry of Health (Chile)
Ministry of Health (Chile). Chile Hospital Discharges 2001-2002
Ministry of Health (Chile). Chile Hospital Discharges 2002. Santiago, Chile: Ministry of Health (Chile)
Ministry of Health (Chile). Chile Hospital Discharges 2003. Santiago, Chile: Ministry of Health (Chile)
Ministry of Health (Chile). Chile Hospital Discharges 2003-2007
Ministry of Health (Chile). Chile Hospital Discharges 2004. Santiago, Chile: Ministry of Health (Chile)
Ministry of Health (Chile). Chile Hospital Discharges 2005. Santiago, Chile: Ministry of Health (Chile)
Ministry of Health (Chile). Chile Hospital Discharges 2006. Santiago, Chile: Ministry of Health (Chile)

Ministry of Health (Chile). Chile Hospital Discharges 2007. Santiago, Chile: Ministry of Health (Chile)
Ministry of Health (Chile). Chile Hospital Discharges 2008. Santiago, Chile: Ministry of Health (Chile)
Ministry of Health (Chile). Chile Hospital Discharges 2008-2012
Ministry of Health (Chile). Chile Hospital Discharges 2009. Santiago, Chile: Ministry of Health (Chile)
Ministry of Health (Chile). Chile Hospital Discharges 2010. Santiago, Chile: Ministry of Health (Chile)
Ministry of Health (Chile). Chile Hospital Discharges 2011. Santiago, Chile: Ministry of Health (Chile)
Ministry of Health (Chile). Chile Hospital Discharges 2012. Santiago, Chile: Ministry of Health (Chile)
Ministry of Health (Israel). Israel National Hospital Discharge Database 2011
Ministry of Health (Israel). Israel National Hospital Discharge Database 2012
Ministry of Health (Israel). Israel National Hospital Discharge Database 2013
Ministry of Health (Israel). Israel National Hospital Discharge Database 2014
Ministry of Health (Italy). Italy Hospital Inpatient Discharges 2005-2007
Ministry of Health (Italy). Italy Hospital Inpatient Discharges 2008-2012
Ministry of Health (Italy). Italy Hospital Inpatient Discharges 2013-2016
Ministry of Health (Italy). Italy National Hospital Discharge Database 1970
Ministry of Health (Italy). Italy National Hospital Discharge Database 1971
Ministry of Health (Italy). Italy National Hospital Discharge Database 1972
Ministry of Health (Italy). Italy National Hospital Discharge Database 1973
Ministry of Health (Italy). Italy National Hospital Discharge Database 1974
Ministry of Health (Italy). Italy National Hospital Discharge Database 1975
Ministry of Health (Italy). Italy National Hospital Discharge Database 1976
Ministry of Health (Italy). Italy National Hospital Discharge Database 1977
Ministry of Health (Italy). Italy National Hospital Discharge Database 1978
Ministry of Health (Italy). Italy National Hospital Discharge Database 1979
Ministry of Health (Italy). Italy National Hospital Discharge Database 1980
Ministry of Health (Italy). Italy National Hospital Discharge Database 1981
Ministry of Health (Italy). Italy National Hospital Discharge Database 1982
Ministry of Health (Italy). Italy National Hospital Discharge Database 1983
Ministry of Health (Italy). Italy National Hospital Discharge Database 1984
Ministry of Health (Italy). Italy National Hospital Discharge Database 1985
Ministry of Health (Italy). Italy National Hospital Discharge Database 1986
Ministry of Health (Italy). Italy National Hospital Discharge Database 1987
Ministry of Health (Italy). Italy National Hospital Discharge Database 1988
Ministry of Health (Italy). Italy National Hospital Discharge Database 1989
Ministry of Health (Italy). Italy National Hospital Discharge Database 1990

Ministry of Health (Italy). Italy National Hospital Discharge Database 1991
Ministry of Health (Italy). Italy National Hospital Discharge Database 1992
Ministry of Health (Italy). Italy National Hospital Discharge Database 1993
Ministry of Health (Italy). Italy National Hospital Discharge Database 1994
Ministry of Health (Italy). Italy National Hospital Discharge Database 1995
Ministry of Health (Italy). Italy National Hospital Discharge Database 1996
Ministry of Health (Italy). Italy National Hospital Discharge Database 1997
Ministry of Health (Italy). Italy National Hospital Discharge Database 1998
Ministry of Health (Italy). Italy National Hospital Discharge Database 1999
Ministry of Health (Italy). Italy National Hospital Discharge Database 2000
Ministry of Health (Italy). Italy National Hospital Discharge Database 2012
Ministry of Health (Italy). Italy National Hospital Discharge Database 2013
Ministry of Health (Italy). Italy National Hospital Discharge Database 2014
Ministry of Health (Jordan). Jordan Al-Bashir Hospital Discharges 2016
Ministry of Health (Kenya). Kenya National Inpatient Morbidity and Mortality Statistics 1999
Ministry of Health (Mexico). Mexico Ministry of Health Hospital Discharges 2000-2002
Ministry of Health (Mexico). Mexico Ministry of Health Hospital Discharges 2003-2007
Ministry of Health (Mexico). Mexico Ministry of Health Hospital Discharges 2008-2012
Ministry of Health (New Zealand). New Zealand National Minimum Dataset 2000
Ministry of Health (New Zealand). New Zealand National Minimum Dataset 2000-2002
Ministry of Health (New Zealand). New Zealand National Minimum Dataset 2001
Ministry of Health (New Zealand). New Zealand National Minimum Dataset 2002
Ministry of Health (New Zealand). New Zealand National Minimum Dataset 2003
Ministry of Health (New Zealand). New Zealand National Minimum Dataset 2003-2007
Ministry of Health (New Zealand). New Zealand National Minimum Dataset 2004
Ministry of Health (New Zealand). New Zealand National Minimum Dataset 2005
Ministry of Health (New Zealand). New Zealand National Minimum Dataset 2006
Ministry of Health (New Zealand). New Zealand National Minimum Dataset 2007. Wellington, New Zealand: Ministry of Health (New Zealand)
Ministry of Health (New Zealand). New Zealand National Minimum Dataset 2008. Wellington, New Zealand: Ministry of Health (New Zealand)
Ministry of Health (New Zealand). New Zealand National Minimum Dataset 2008-2012
Ministry of Health (New Zealand). New Zealand National Minimum Dataset 2009. Wellington, New Zealand: Ministry of Health (New Zealand)
Ministry of Health (New Zealand). New Zealand National Minimum Dataset 2010. Wellington, New Zealand: Ministry of Health (New Zealand)
Ministry of Health (New Zealand). New Zealand National Minimum Dataset 2011. Wellington, New Zealand: Ministry of Health (New Zealand)
Ministry of Health (New Zealand). New Zealand National Minimum Dataset 2012. Wellington, New Zealand: Ministry of Health (New Zealand)
Ministry of Health (New Zealand). New Zealand National Minimum Dataset 2013. Wellington, New Zealand: Ministry of Health (New Zealand)

Ministry of Health (New Zealand). New Zealand National Minimum Dataset 2013-2014
Ministry of Health (New Zealand). New Zealand National Minimum Dataset 2014. Wellington, New Zealand: Ministry of Health (New Zealand)
Ministry of Health (New Zealand). New Zealand National Minimum Dataset 2015. Wellington, New Zealand: Ministry of Health (New Zealand)
Ministry of Health (Nicaragua), National Institute for Development Information (Nicaragua). Nicaragua National Demographic and Health Survey 2011-2012. Managua, Nicaragua: National Institute for Development Information (Nicaragua)
Ministry of Health (Poland), Ministry of Health (Poland). Poland Hospital Inpatient Discharges 2013
Ministry of Health (Poland), National Institute of Public Health-National Institute of Hygiene (NIPH-NIH) (Poland). Poland Hospital Inpatient Discharges 2003
Ministry of Health (Poland), National Institute of Public Health-National Institute of Hygiene (NIPH-NIH) (Poland). Poland Hospital Inpatient Discharges 2004
Ministry of Health (Poland). Poland Hospital Inpatient Discharges 1980
Ministry of Health (Poland). Poland Hospital Inpatient Discharges 1981
Ministry of Health (Poland). Poland Hospital Inpatient Discharges 1982
Ministry of Health (Poland). Poland Hospital Inpatient Discharges 1983
Ministry of Health (Poland). Poland Hospital Inpatient Discharges 1984
Ministry of Health (Poland). Poland Hospital Inpatient Discharges 1985
Ministry of Health (Poland). Poland Hospital Inpatient Discharges 1986
Ministry of Health (Poland). Poland Hospital Inpatient Discharges 1987
Ministry of Health (Poland). Poland Hospital Inpatient Discharges 1988
Ministry of Health (Poland). Poland Hospital Inpatient Discharges 1989
Ministry of Health (Poland). Poland Hospital Inpatient Discharges 1990
Ministry of Health (Poland). Poland Hospital Inpatient Discharges 1991
Ministry of Health (Poland). Poland Hospital Inpatient Discharges 1992
Ministry of Health (Poland). Poland Hospital Inpatient Discharges 1993
Ministry of Health (Poland). Poland Hospital Inpatient Discharges 1994
Ministry of Health (Poland). Poland Hospital Inpatient Discharges 1995
Ministry of Health (Poland). Poland Hospital Inpatient Discharges 1996
Ministry of Health (Poland). Poland Hospital Inpatient Discharges 1997
Ministry of Health (Poland). Poland Hospital Inpatient Discharges 1998
Ministry of Health (Poland). Poland Hospital Inpatient Discharges 1999
Ministry of Health (Poland). Poland Hospital Inpatient Discharges 2000
Ministry of Health (Poland). Poland Hospital Inpatient Discharges 2001
Ministry of Health (Poland). Poland Hospital Inpatient Discharges 2002
Ministry of Health (Poland). Poland Hospital Inpatient Discharges 2014
Ministry of Health (Portugal). Portugal Hospital Inpatient Discharges 2015
Ministry of Health (Romania). Romania Hospital Inpatient Discharges 1980
Ministry of Health (Romania). Romania Hospital Inpatient Discharges 1985

Ministry of Health (Romania). Romania Hospital Inpatient Discharges 1986
Ministry of Health (Romania). Romania Hospital Inpatient Discharges 1987
Ministry of Health (Romania). Romania Hospital Inpatient Discharges 1988
Ministry of Health (Romania). Romania Hospital Inpatient Discharges 1989
Ministry of Health (Romania). Romania Hospital Inpatient Discharges 1990
Ministry of Health (Romania). Romania Hospital Inpatient Discharges 1991
Ministry of Health (Romania). Romania Hospital Inpatient Discharges 1992
Ministry of Health (Romania). Romania Hospital Inpatient Discharges 1993
Ministry of Health (Romania). Romania Hospital Inpatient Discharges 1994
Ministry of Health (Romania). Romania Hospital Inpatient Discharges 1995
Ministry of Health (Romania). Romania Hospital Inpatient Discharges 1996
Ministry of Health (Romania). Romania Hospital Inpatient Discharges 1997
Ministry of Health (Romania). Romania Hospital Inpatient Discharges 1998
Ministry of Health (Romania). Romania Hospital Inpatient Discharges 1999
Ministry of Health (Romania). Romania Hospital Inpatient Discharges 2000
Ministry of Health (Romania). Romania Hospital Inpatient Discharges 2001
Ministry of Health (Romania). Romania Hospital Inpatient Discharges 2002
Ministry of Health (Romania). Romania Hospital Inpatient Discharges 2003
Ministry of Health (Romania). Romania Hospital Inpatient Discharges 2004
Ministry of Health (Romania). Romania Hospital Inpatient Discharges 2005
Ministry of Health (Romania). Romania Hospital Inpatient Discharges 2013
Ministry of Health (Syria), World Health Organization (WHO). Syria WHO Multi-country Survey Study on Health and Health System Responsiveness 2000-2001
Ministry of Health (Turkey). Turkey Diagnosis-Related Group Hospital Inpatient Database 2011-2012
Ministry of Health (Vietnam). Vietnam Hospital Data 2013
Ministry of Health and Consumer Affairs (Spain), National Statistics Institute (Spain). Spain Statistics on Health Establishments Providing Inpatient Care 1982
Ministry of Health and Consumer Affairs (Spain), National Statistics Institute (Spain). Spain Statistics on Health Establishments Providing Inpatient Care 1983
Ministry of Health and Consumer Affairs (Spain), National Statistics Institute (Spain). Spain Statistics on Health Establishments Providing Inpatient Care 1984
Ministry of Health and Consumer Affairs (Spain), National Statistics Institute (Spain). Spain Statistics on Health Establishments Providing Inpatient Care 1985
Ministry of Health and Consumer Affairs (Spain), National Statistics Institute (Spain). Spain Statistics on Health Establishments Providing Inpatient Care 1986
Ministry of Health and Consumer Affairs (Spain), National Statistics Institute (Spain). Spain Statistics on Health Establishments Providing Inpatient Care 1987
Ministry of Health and Consumer Affairs (Spain), National Statistics Institute (Spain). Spain Statistics on Health Establishments Providing Inpatient Care 1988
Ministry of Health and Consumer Affairs (Spain), National Statistics Institute (Spain). Spain Statistics on Health Establishments Providing Inpatient Care 1989
Ministry of Health and Consumer Affairs (Spain), National Statistics Institute (Spain). Spain Statistics on Health Establishments Providing Inpatient Care 1990
Ministry of Health and Consumer Affairs (Spain), National Statistics Institute (Spain). Spain Statistics on Health Establishments Providing Inpatient Care 1991
Ministry of Health and Consumer Affairs (Spain), National Statistics Institute (Spain). Spain Statistics on Health Establishments Providing Inpatient Care 1992

Ministry of Health and Consumer Affairs (Spain), National Statistics Institute (Spain). Spain Statistics on Health Establishments Providing Inpatient Care 1993
Ministry of Health and Consumer Affairs (Spain), National Statistics Institute (Spain). Spain Statistics on Health Establishments Providing Inpatient Care 1994
Ministry of Health and Consumer Affairs (Spain), National Statistics Institute (Spain). Spain Statistics on Health Establishments Providing Inpatient Care 1995
Ministry of Health and Consumer Affairs (Spain), National Statistics Institute (Spain). Spain Statistics on Health Establishments Providing Inpatient Care 1996
Ministry of Health and Consumer Affairs (Spain). Spain Statistics on Health Establishments Providing Inpatient Care 1997. Madrid, Spain: Ministry of Health, Social Services and Equality (Spain)
Ministry of Health and Consumer Affairs (Spain). Spain Statistics on Health Establishments Providing Inpatient Care 1998. Madrid, Spain: Ministry of Health, Social Services and Equality (Spain)
Ministry of Health and Consumer Affairs (Spain). Spain Statistics on Health Establishments Providing Inpatient Care 1999. Madrid, Spain: Ministry of Health, Social Services and Equality (Spain)
Ministry of Health and Consumer Affairs (Spain). Spain Statistics on Health Establishments Providing Inpatient Care 2000. Madrid, Spain: Ministry of Health, Social Services and Equality (Spain)
Ministry of Health and Consumer Affairs (Spain). Spain Statistics on Health Establishments Providing Inpatient Care 2001. Madrid, Spain: Ministry of Health, Social Services and Equality (Spain)
Ministry of Health and Consumer Affairs (Spain). Spain Statistics on Health Establishments Providing Inpatient Care 2002. Madrid, Spain: Ministry of Health, Social Services and Equality (Spain)
Ministry of Health and Consumer Affairs (Spain). Spain Statistics on Health Establishments Providing Inpatient Care 2003. Madrid, Spain: Ministry of Health, Social Services and Equality (Spain)
Ministry of Health and Consumer Affairs (Spain). Spain Statistics on Health Establishments Providing Inpatient Care 2004. Madrid, Spain: Ministry of Health, Social Services and Equality (Spain)
Ministry of Health and Consumer Affairs (Spain). Spain Statistics on Health Establishments Providing Inpatient Care 2005. Madrid, Spain: Ministry of Health, Social Services and Equality (Spain)
Ministry of Health and Consumer Affairs (Spain). Spain Statistics on Health Establishments Providing Inpatient Care 2006. Madrid, Spain: Ministry of Health, Social Services and Equality (Spain)
Ministry of Health and Consumer Affairs (Spain). Spain Statistics on Health Establishments Providing Inpatient Care 2007. Madrid, Spain: Ministry of Health, Social Services and Equality (Spain)
Ministry of Health and Consumer Affairs (Spain). Spain Statistics on Health Establishments Providing Inpatient Care 2008. Madrid, Spain: Ministry of Health, Social Services and Equality (Spain)
Ministry of Health and Consumer Affairs (Spain). Spain Statistics on Health Establishments Providing Inpatient Care 2009. Madrid, Spain: Ministry of Health, Social Services and Equality (Spain)
Ministry of Health and Medical Education (Iran), World Health Organization (WHO). Iran WHO Multi-country Survey Study on Health and Health System Responsiveness 2000-2001. Geneva, Switzerland: World Health Organization (WHO)
Ministry of Health and Medical Education (Iran). Iran Hospital Data 2001-2010
Ministry of Health and Social Security (Spain), National Statistics Institute (Spain). Spain Statistics on Health Establishments Providing Inpatient Care 1972
Ministry of Health and Social Security (Spain), National Statistics Institute (Spain). Spain Statistics on Health Establishments Providing Inpatient Care 1973

Ministry of Health and Social Security (Spain), National Statistics Institute (Spain). Spain Statistics on Health Establishments Providing Inpatient Care 1974
Ministry of Health and Social Security (Spain), National Statistics Institute (Spain). Spain Statistics on Health Establishments Providing Inpatient Care 1975
Ministry of Health and Social Security (Spain), National Statistics Institute (Spain). Spain Statistics on Health Establishments Providing Inpatient Care 1976
Ministry of Health and Social Security (Spain), National Statistics Institute (Spain). Spain Statistics on Health Establishments Providing Inpatient Care 1977
Ministry of Health and Social Security (Spain), National Statistics Institute (Spain). Spain Statistics on Health Establishments Providing Inpatient Care 1978
Ministry of Health and Social Security (Spain), National Statistics Institute (Spain). Spain Statistics on Health Establishments Providing Inpatient Care 1979
Ministry of Health and Social Security (Spain), National Statistics Institute (Spain). Spain Statistics on Health Establishments Providing Inpatient Care 1980
Ministry of Health and Social Security (Spain), National Statistics Institute (Spain). Spain Statistics on Health Establishments Providing Inpatient Care 1981
Ministry of Health and Welfare (Taiwan). Taiwan National Health Insurance Claims Data 2016
Ministry of Health of the Republic of Latvia, National Health Service (Latvia). Latvia Hospital Inpatient Discharges 2011
Ministry of Health of the Republic of Latvia, National Health Service (Latvia). Latvia Hospital Inpatient Discharges 2012
Ministry of Health of the Republic of Latvia, National Health Service (Latvia). Latvia Hospital Inpatient Discharges 2013
Ministry of Health of the Republic of Latvia, National Health Service (Latvia). Latvia Hospital Inpatient Discharges 2014
Ministry of Health of the Republic of Latvia. Latvia Hospital Inpatient Discharges 1980
Ministry of Health of the Republic of Latvia. Latvia Hospital Inpatient Discharges 1981
Ministry of Health of the Republic of Latvia. Latvia Hospital Inpatient Discharges 1982
Ministry of Health of the Republic of Latvia. Latvia Hospital Inpatient Discharges 1983
Ministry of Health of the Republic of Latvia. Latvia Hospital Inpatient Discharges 1984
Ministry of Health of the Republic of Latvia. Latvia Hospital Inpatient Discharges 1985
Ministry of Health of the Republic of Latvia. Latvia Hospital Inpatient Discharges 1986
Ministry of Health of the Republic of Latvia. Latvia Hospital Inpatient Discharges 1987
Ministry of Health of the Republic of Latvia. Latvia Hospital Inpatient Discharges 1988
Ministry of Health of the Republic of Latvia. Latvia Hospital Inpatient Discharges 1989
Ministry of Health of the Republic of Latvia. Latvia Hospital Inpatient Discharges 1990
Ministry of Health of the Republic of Latvia. Latvia Hospital Inpatient Discharges 1991
Ministry of Health of the Republic of Latvia. Latvia Hospital Inpatient Discharges 1992
Ministry of Health of the Republic of Latvia. Latvia Hospital Inpatient Discharges 1993
Ministry of Health of the Republic of Latvia. Latvia Hospital Inpatient Discharges 1994
Ministry of Health of the Republic of Latvia. Latvia Hospital Inpatient Discharges 1995
Ministry of Health of the Republic of Latvia. Latvia Hospital Inpatient Discharges 1996
Ministry of Health of the Republic of Latvia. Latvia Hospital Inpatient Discharges 1997
Ministry of Health of the Republic of Latvia. Latvia Hospital Inpatient Discharges 1998
Ministry of Health of the Republic of Latvia. Latvia Hospital Inpatient Discharges 1999
Ministry of Health of the Republic of Latvia. Latvia Hospital Inpatient Discharges 2000
Ministry of Health of the Republic of Latvia. Latvia Hospital Inpatient Discharges 2001

Ministry of Health of the Republic of Latvia. Latvia Hospital Inpatient Discharges 2002
Ministry of Health of the Republic of Latvia. Latvia Hospital Inpatient Discharges 2003
Ministry of Health of the Republic of Latvia. Latvia Hospital Inpatient Discharges 2004
Ministry of Health of the Republic of Latvia. Latvia Hospital Inpatient Discharges 2006
Ministry of Health of the Republic of Latvia. Latvia Hospital Inpatient Discharges 2007
Ministry of Health of the Republic of Latvia. Latvia Hospital Inpatient Discharges 2008
Ministry of Health of the Republic of Latvia. Latvia Hospital Inpatient Discharges 2010
Ministry of Health, Labour and Welfare (Japan). Japan Diagnosis Procedure Combination Database 2010-2012
Ministry of Health, Labour and Welfare (Japan). Japan Diagnosis Procedure Combination Database 2013-2015
Ministry of Health, Social Services and Equality (Spain), National Statistics Institute (Spain). Spain National Health Survey 2006-2007
Ministry of Health, Social Services and Equality (Spain), Sociological Research Center (Spain). Spain Health Barometer Survey 2004. Madrid, Spain: Ministry of Health, Social Services and Equality (Spain), 2004
Ministry of Health, Social Services and Equality (Spain). Spain Statistics on Specialized Healthcare Centers 2010. Madrid, Spain: Ministry of Health, Social Services and Equality (Spain)
Ministry of Health, Social Services and Equality (Spain). Spain Statistics on Specialized Healthcare Centers 2011. Madrid, Spain: Ministry of Health, Social Services and Equality (Spain)
Ministry of Health, Social Services and Equality (Spain). Spain Statistics on Specialized Healthcare Centers 2012. Madrid, Spain: Ministry of Health, Social Services and Equality (Spain)
Ministry of Health, Social Services and Equality (Spain). Spain Statistics on Specialized Healthcare Centers 2013. Madrid, Spain: Ministry of Health, Social Services and Equality (Spain)
Ministry of Health, Social Services and Equality (Spain). Spain Statistics on Specialized Healthcare Centers 2014. Madrid, Spain: Ministry of Health, Social Services and Equality (Spain)
Ministry of Labor and Social Policy (Bulgaria), National Statistical Institute of Bulgaria, TNS Gallup, World Bank. Bulgaria Multitopic Household Survey 2007. Washington DC, United States: World Bank
Ministry of Planning (Chile), Social Observatory, Alberto Hurtado University. Chile National Socioeconomic Characterization Survey 2009. Santiago, Chile: Ministry of Social Development (Chile)
Ministry of Public Health (Lebanon), World Health Organization (WHO). Lebanon WHO Multi-country Survey Study on Health and Health System Responsiveness 2000-2001
Ministry of Public Health (Thailand). Thailand National Health and Examination Survey 2003-2004
Ministry of Rural Development (Mali), National Institute of Statistics (INSTAT) (Mali), World Bank. Mali Agricultural Integrated Economic Survey 2014-2015. Washington DC, United States: World Bank
Ministry of Social Affairs (Estonia), National Institute for Health Development (Estonia). Estonia Hospital Inpatient Discharges 2003
Ministry of Social Affairs (Estonia), National Institute for Health Development (Estonia). Estonia Hospital Inpatient Discharges 2004
Ministry of Social Affairs (Estonia), National Institute for Health Development (Estonia). Estonia Hospital Inpatient Discharges 2005
Ministry of Social Affairs (Estonia), National Institute for Health Development (Estonia). Estonia Hospital Inpatient Discharges 2006

Ministry of Social Affairs (Estonia), National Institute for Health Development (Estonia). Estonia Hospital Inpatient Discharges 2007

Ministry of Social Affairs (Estonia), National Institute for Health Development (Estonia). Estonia Hospital Inpatient Discharges 2008

Ministry of Social Affairs (Estonia), National Institute for Health Development (Estonia). Estonia Hospital Inpatient Discharges 2009

Ministry of Social Affairs (Estonia), National Institute for Health Development (Estonia). Estonia Hospital Inpatient Discharges 2010

Ministry of Social Affairs (Estonia), National Institute for Health Development (Estonia). Estonia Hospital Inpatient Discharges 2011

Ministry of Social Affairs (Serbia), World Bank. Serbia and Montenegro - Serbia Living Standards Measurement Survey 2003. Washington DC, United States: World Bank

Ministry of Social Affairs (Serbia), World Bank. Serbia Living Standards Measurement Survey 2007. Washington DC, United States: World Bank

Ministry of Social Affairs (Serbia), World Bank. Yugoslavia, Federal Republic - Serbia Living Standards Measurement Survey 2002. Washington DC, United States: World Bank

Ministry of Statistics and Programme Implementation (India). India National Sample Survey Round 52 1995-1996. New Delhi, India: Ministry of Statistics and Programme Implementation (India)

Ministry of Statistics and Programme Implementation (India). India National Sample Survey Round 60 2004. New Delhi, India: Ministry of Statistics and Programme Implementation (India)

Morris CD, Menashe VD. 25-year mortality after surgical repair of congenital heart defect in childhood. A population-based cohort study. JAMA. 1991; 266(24): 3447-52

Morris SA, Ethen MK, Penny DJ, Canfield MA, Minard CG, Fixler DE, Nembhard WN. Prenatal diagnosis, birth location, surgical center, and neonatal mortality in infants with hypoplastic left heart syndrome. Circulation. 2014; 129(3): 285-92

Mosayebi Z, Movahedian AH. Pattern of congenital malformations in consanguineous versus nonconsanguineous marriages in Kashan, Islamic Republic of Iran. East Mediterr Health J. 2007; 13(4): 868-75

Murphy JG, Gersh BJ, Mair DD, Fuster V, McGoon MD, Ilstrup DM, McGoon DC, Kirklin JW, Danielson GK. Long-term outcome in patients undergoing surgical repair of tetralogy of Fallot. N Engl J Med. 1993; 329(9): 593-9

National Administrative Department of Statistics (Colombia). Colombia National Quality of Life Survey 1997. Bogotá, Colombia: National Administrative Department of Statistics (Colombia)

National Administrative Department of Statistics (Colombia). Colombia National Quality of Life Survey 2008. Bogotá, Colombia: National Administrative Department of Statistics (Colombia)

National Administrative Department of Statistics (DANE) (Colombia). Colombia National Quality of Life Survey 2010. Bogotá, Colombia: National Administrative Department of Statistics (DANE) (Colombia), 2012

National Birth Defects Prevention Network, National Center on Birth Defects and Developmental Disabilities, Centers for Disease Control and Prevention (NCBDDD). Selected Birth Defects Data from Population-based Birth Defects Surveillance Programs in the United States, 2003-2007. Birth Defects Res A Clin Mol Teratol. 2010; 88(12): 1062-1174

National Birth Defects Prevention Network. Birth Defect Surveillance Data from Selected States 1989-1996. Birth Defects Res A Clin Mol Teratol. 2000; 61(1-2): 86-160

National Birth Defects Prevention Network. Birth Defects Surveillance Data from Selected States 1989-1998. Birth Defects Res A Clin Mol Teratol. 2001; 64(S1): S117-73

National Birth Defects Prevention Network. Birth Defects Surveillance Data from Selected States 1998-2002. Birth Defects Res A Clin Mol Teratol. 2005; 73(10): 758-853

National Board of Health and Welfare (Sweden). Sweden National Patient Register 1987. Stockholm, Sweden: National Board of Health and Welfare (Sweden)
National Board of Health and Welfare (Sweden). Sweden National Patient Register 1988. Stockholm, Sweden: National Board of Health and Welfare (Sweden)
National Board of Health and Welfare (Sweden). Sweden National Patient Register 1989. Stockholm, Sweden: National Board of Health and Welfare (Sweden)
National Board of Health and Welfare (Sweden). Sweden National Patient Register 1990. Stockholm, Sweden: National Board of Health and Welfare (Sweden)
National Board of Health and Welfare (Sweden). Sweden National Patient Register 1991. Stockholm, Sweden: National Board of Health and Welfare (Sweden)
National Board of Health and Welfare (Sweden). Sweden National Patient Register 1992. Stockholm, Sweden: National Board of Health and Welfare (Sweden)
National Board of Health and Welfare (Sweden). Sweden National Patient Register 1993. Stockholm, Sweden: National Board of Health and Welfare (Sweden)
National Board of Health and Welfare (Sweden). Sweden National Patient Register 1994. Stockholm, Sweden: National Board of Health and Welfare (Sweden)
National Board of Health and Welfare (Sweden). Sweden National Patient Register 1995. Stockholm, Sweden: National Board of Health and Welfare (Sweden)
National Board of Health and Welfare (Sweden). Sweden National Patient Register 1996. Stockholm, Sweden: National Board of Health and Welfare (Sweden)
National Board of Health and Welfare (Sweden). Sweden National Patient Register 1997. Stockholm, Sweden: National Board of Health and Welfare (Sweden)
National Board of Health and Welfare (Sweden). Sweden National Patient Register 1998. Stockholm, Sweden: National Board of Health and Welfare (Sweden)
National Board of Health and Welfare (Sweden). Sweden National Patient Register 1998-2002
National Board of Health and Welfare (Sweden). Sweden National Patient Register 1999. Stockholm, Sweden: National Board of Health and Welfare (Sweden)
National Board of Health and Welfare (Sweden). Sweden National Patient Register 2003-2007
National Board of Health and Welfare (Sweden). Sweden National Patient Register 2008-2012
National Board of Health and Welfare (Sweden). Sweden National Patient Register 2013. Stockholm, Sweden: National Board of Health and Welfare (Sweden)
National Board of Health and Welfare (Sweden). Sweden National Patient Register 2014. Stockholm, Sweden: National Board of Health and Welfare (Sweden)
National Bureau of Statistics (Nigeria). Nigeria Living Standards Survey 2008-2010. Abuja, Nigeria: National Bureau of Statistics (Nigeria)
National Bureau of Statistics (Tanzania). Tanzania Living Standards Measurement Study - Integrated Surveys on Agriculture 2010-2011. Dar es Salaam, Tanzania: National Bureau of Statistics (Tanzania)
National Bureau of Statistics of China. China Statistical Yearbook 2015. Beijing, China: National Bureau of Statistics of China
National Center for Disease Control and Public Health (Georgia). Georgia Hospital Data 2013-2014
National Center for Disease Control and Public Health (Georgia). Georgia Inpatient Care Discharges per 100 1980
National Center for Disease Control and Public Health (Georgia). Georgia Inpatient Care Discharges per 100 1985
National Center for Disease Control and Public Health (Georgia). Georgia Inpatient Care Discharges per 100 1986
National Center for Disease Control and Public Health (Georgia). Georgia Inpatient Care Discharges per 100 1987
National Center for Disease Control and Public Health (Georgia). Georgia Inpatient Care Discharges per 100 1988
National Center for Disease Control and Public Health (Georgia). Georgia Inpatient Care Discharges per 100 1989
National Center for Disease Control and Public Health (Georgia). Georgia Inpatient Care Discharges per 100 1990
National Center for Disease Control and Public Health (Georgia). Georgia Inpatient Care Discharges per 100 1991
National Center for Disease Control and Public Health (Georgia). Georgia Inpatient Care Discharges per 100 1992
National Center for Disease Control and Public Health (Georgia). Georgia Inpatient Care Discharges per 100 1993
National Center for Disease Control and Public Health (Georgia). Georgia Inpatient Care Discharges per 100 1994
National Center for Disease Control and Public Health (Georgia). Georgia Inpatient Care Discharges per 100 1995

National Center for Disease Control and Public Health (Georgia). Georgia Inpatient Care Discharges per 100 1996
National Center for Disease Control and Public Health (Georgia). Georgia Inpatient Care Discharges per 100 1997
National Center for Disease Control and Public Health (Georgia). Georgia Inpatient Care Discharges per 100 1998
National Center for Disease Control and Public Health (Georgia). Georgia Inpatient Care Discharges per 100 1999
National Center for Disease Control and Public Health (Georgia). Georgia Inpatient Care Discharges per 100 2000
National Center for Disease Control and Public Health (Georgia). Georgia Inpatient Care Discharges per 100 2001
National Center for Disease Control and Public Health (Georgia). Georgia Inpatient Care Discharges per 100 2002
National Center for Disease Control and Public Health (Georgia). Georgia Inpatient Care Discharges per 100 2003
National Center for Disease Control and Public Health (Georgia). Georgia Inpatient Care Discharges per 100 2004
National Center for Disease Control and Public Health (Georgia). Georgia Inpatient Care Discharges per 100 2005
National Center for Disease Control and Public Health (Georgia). Georgia Inpatient Care Discharges per 100 2006
National Center for Disease Control and Public Health (Georgia). Georgia Inpatient Care Discharges per 100 2007
National Center for Disease Control and Public Health (Georgia). Georgia Inpatient Care Discharges per 100 2008
National Center for Disease Control and Public Health (Georgia). Georgia Inpatient Care Discharges per 100 2009
National Center for Disease Control and Public Health (Georgia). Georgia Inpatient Care Discharges per 100 2010
National Center for Disease Control and Public Health (Georgia). Georgia Inpatient Care Discharges per 100 2011
National Center for Disease Control and Public Health (Georgia). Georgia Inpatient Care Discharges per 100 2012
National Center for Disease Control and Public Health (Georgia). Georgia Inpatient Care Discharges per 100 2013
National Center for Disease Control and Public Health (Georgia). Georgia Inpatient Care Discharges per 100 2014
National Center for Health Statistics (NCHS), Centers for Disease Control and Prevention (CDC), US Census Bureau. United States National Health Interview Survey 1990. Hyattsville, United States: National Center for Health Statistics (NCHS), Centers for Disease Control and Prevention (CDC)
National Center for Health Statistics (NCHS), Centers for Disease Control and Prevention (CDC), US Census Bureau. United States National Health Interview Survey 1991. Hyattsville, United States: National Center for Health Statistics (NCHS), Centers for Disease Control and Prevention (CDC)
National Center for Health Statistics (NCHS), Centers for Disease Control and Prevention (CDC), US Census Bureau. United States National Health Interview Survey 1992. Hyattsville, United States: National Center for Health Statistics (NCHS), Centers for Disease Control and Prevention (CDC)
National Center for Health Statistics (NCHS), Centers for Disease Control and Prevention (CDC), US Census Bureau. United States National Health Interview Survey 1993. Hyattville, United States: National Center for Health Statistics (NCHS), Centers for Disease Control and Prevention (CDC)
National Center for Health Statistics (NCHS), Centers for Disease Control and Prevention (CDC), US Census Bureau. United States National Health Interview Survey 1997. Hyattsville, United States: National Center for Health Statistics (NCHS), Centers for Disease Control and Prevention (CDC)
National Center for Health Statistics (NCHS), Centers for Disease Control and Prevention (CDC), US Census Bureau. United States National Health Interview Survey 1998. Hyattsville, United States: National Center for Health Statistics (NCHS), Centers for Disease Control and Prevention (CDC)
National Center for Health Statistics (NCHS), Centers for Disease Control and Prevention (CDC), US Census Bureau. United States National Health Interview Survey 1999. Hyattsville, United States: National Center for Health Statistics (NCHS), Centers for Disease Control and Prevention (CDC)
National Center for Health Statistics (NCHS), Centers for Disease Control and Prevention (CDC), US Census Bureau. United States National Health Interview Survey 2000. Hyattsville, United States: National Center for Health Statistics (NCHS), Centers for Disease Control and Prevention (CDC)

National Center for Health Statistics (NCHS), Centers for Disease Control and Prevention (CDC), US Census Bureau. United States National Health Interview Survey 2001. Hyattsville, United States: National Center for Health Statistics (NCHS), Centers for Disease Control and Prevention (CDC)

National Center for Health Statistics (NCHS), Centers for Disease Control and Prevention (CDC), US Census Bureau. United States National Health Interview Survey 2002. Hyattsville, United States: National Center for Health Statistics (NCHS), Centers for Disease Control and Prevention (CDC)

National Center for Health Statistics (NCHS), Centers for Disease Control and Prevention (CDC), US Census Bureau. United States National Health Interview Survey 2003. Hyattsville, United States: National Center for Health Statistics (NCHS), Centers for Disease Control and Prevention (CDC)

National Center for Health Statistics (NCHS), Centers for Disease Control and Prevention (CDC), US Census Bureau. United States National Health Interview Survey 2004. Hyattsville, United States: National Center for Health Statistics (NCHS), Centers for Disease Control and Prevention (CDC)

National Center for Health Statistics (NCHS), Centers for Disease Control and Prevention (CDC), US Census Bureau. United States National Health Interview Survey 2006. Hyattsville, United States: National Center for Health Statistics (NCHS), Centers for Disease Control and Prevention (CDC)

National Center for Health Statistics (NCHS), Centers for Disease Control and Prevention (CDC), US Census Bureau. United States National Health Interview Survey 2007. Hyattsville, United States: National Center for Health Statistics (NCHS), Centers for Disease Control and Prevention (CDC)

National Center for Health Statistics (NCHS), Centers for Disease Control and Prevention (CDC), US Census Bureau. United States National Health Interview Survey 2008. Hyattsville, United States: National Center for Health Statistics (NCHS), Centers for Disease Control and Prevention (CDC)

National Center for Health Statistics (NCHS), Centers for Disease Control and Prevention (CDC), US Census Bureau. United States National Health Interview Survey 2009. Hyattsville, United States: National Center for Health Statistics (NCHS), Centers for Disease Control and Prevention (CDC)

National Center for Health Statistics (NCHS), Centers for Disease Control and Prevention (CDC), US Census Bureau. United States National Health Interview Survey 2010. Hyattsville, United States: National Center for Health Statistics (NCHS), Centers for Disease Control and Prevention (CDC)

National Center for Health Statistics (NCHS), Centers for Disease Control and Prevention (CDC), US Census Bureau. United States National Health Interview Survey 2011. Hyattsville, United States: National Center for Health Statistics (NCHS), Centers for Disease Control and Prevention (CDC)

National Center for Health Statistics (NCHS), Centers for Disease Control and Prevention (CDC). United States National Health and Nutrition Examination Survey 1988-1994. Hyattsville, United States: National Center for Health Statistics (NCHS), Centers for Disease Control and Prevention (CDC)

National Center for Health Statistics (NCHS), Centers for Disease Control and Prevention (CDC). United States National Health and Nutrition Examination Survey 1999-2000. Hyattsville, United States: National Center for Health Statistics (NCHS), Centers for Disease Control and Prevention (CDC)

National Center for Health Statistics (NCHS), Centers for Disease Control and Prevention (CDC). United States National Health and Nutrition Examination Survey 2001-2002. Hyattsville, United States: National Center for Health Statistics (NCHS), Centers for Disease Control and Prevention (CDC)

National Center for Health Statistics (NCHS), Centers for Disease Control and Prevention (CDC). United States National Health and Nutrition Examination Survey 2003-2004. Hyattsville, United States: National Center for Health Statistics (NCHS), Centers for Disease Control and Prevention (CDC)

National Center for Health Statistics (NCHS), Centers for Disease Control and Prevention (CDC). United States National Health and Nutrition Examination Survey 2005-2006. Hyattsville, United States: National Center for Health Statistics (NCHS), Centers for Disease Control and Prevention (CDC), 2007

National Center for Health Statistics (NCHS), Centers for Disease Control and Prevention (CDC). United States National Health and Nutrition Examination Survey 2007-2008. Hyattsville, United States: National Center for Health Statistics (NCHS), Centers for Disease Control and Prevention (CDC), 2009

National Center for Health Statistics (NCHS), Centers for Disease Control and Prevention (CDC). United States National Health and Nutrition Examination Survey 2009-2010. Hyattsville, United States: National Center for Health Statistics (NCHS), Centers for Disease Control and Prevention (CDC), 2011

National Center for Health Statistics (NCHS), Centers for Disease Control and Prevention (CDC). United States National Hospital Discharge Survey 1988-1992

National Center for Health Statistics (NCHS), Centers for Disease Control and Prevention (CDC). United States National Hospital Discharge Survey 1993-1997
National Center for Health Statistics (NCHS), Centers for Disease Control and Prevention (CDC). United States National Hospital Discharge Survey 1998-2002
National Center for Health Statistics (NCHS), Centers for Disease Control and Prevention (CDC). United States National Hospital Discharge Survey 2003-2007
National Center for Health Statistics (NCHS), Centers for Disease Control and Prevention (CDC). United States National Hospital Discharge Survey 2008-2010
National Center for Health Statistics, Centers for Disease Control and Prevention, United States Census Bureau. United States National Health Interview Survey 2012. Hyattsville, United States: National Center for Health Statistics, Centers for Disease Control and Prevention, 2013
National Center for Health Statistics, Centers for Disease Control and Prevention, United States Census Bureau. United States National Health Interview Survey 2013. Hyattsville, United States: National Center for Health Statistics, Centers for Disease Control and Prevention, 2014
National Center for Health Statistics, Centers for Disease Control and Prevention, United States Census Bureau. United States National Hospital Discharge Survey 1979. Hyattsville, United States: National Center for Health Statistics, Centers for Disease Control and Prevention
National Center for Health Statistics, Centers for Disease Control and Prevention, United States Census Bureau. United States National Hospital Discharge Survey 1980. Hyattsville, United States: National Center for Health Statistics, Centers for Disease Control and Prevention
National Center for Health Statistics, Centers for Disease Control and Prevention, United States Census Bureau. United States National Hospital Discharge Survey 1981. Hyattsville, United States: National Center for Health Statistics, Centers for Disease Control and Prevention
National Center for Health Statistics, Centers for Disease Control and Prevention, United States Census Bureau. United States National Hospital Discharge Survey 1982. Hyattsville, United States: National Center for Health Statistics, Centers for Disease Control and Prevention
National Center for Health Statistics, Centers for Disease Control and Prevention, United States Census Bureau. United States National Hospital Discharge Survey 1983. Hyattsville, United States: National Center for Health Statistics, Centers for Disease Control and Prevention
National Center for Health Statistics, Centers for Disease Control and Prevention, United States Census Bureau. United States National Hospital Discharge Survey 1984. Hyattsville, United States: National Center for Health Statistics, Centers for Disease Control and Prevention
National Center for Health Statistics, Centers for Disease Control and Prevention, United States Census Bureau. United States National Hospital Discharge Survey 1985. Hyattsville, United States: National Center for Health Statistics, Centers for Disease Control and Prevention
National Center for Health Statistics, Centers for Disease Control and Prevention, United States Census Bureau. United States National Hospital Discharge Survey 1986. Hyattsville, United States: National Center for Health Statistics, Centers for Disease Control and Prevention
National Center for Health Statistics, Centers for Disease Control and Prevention, United States Census Bureau. United States National Hospital Discharge Survey 1987. Hyattsville, United States: National Center for Health Statistics, Centers for Disease Control and Prevention
National Center for Health Statistics, Centers for Disease Control and Prevention, United States Census Bureau. United States National Hospital Discharge Survey 1988. Hyattsville, United States: National Center for Health Statistics, Centers for Disease Control and Prevention
National Center for Health Statistics, Centers for Disease Control and Prevention, United States Census Bureau. United States National Hospital Discharge Survey 1989. Hyattsville, United States: National Center for Health Statistics, Centers for Disease Control and Prevention
National Center for Health Statistics, Centers for Disease Control and Prevention, United States Census Bureau. United States National Hospital Discharge Survey 1990. Hyattsville, United States: National Center for Health Statistics, Centers for Disease Control and Prevention
National Center for Health Statistics, Centers for Disease Control and Prevention, United States Census Bureau. United States National Hospital Discharge Survey 1991. Hyattsville, United States: National Center for Health Statistics, Centers for Disease Control and Prevention

National Center for Health Statistics, Centers for Disease Control and Prevention, United States Census Bureau. United States National Hospital Discharge Survey 1992. Hyattsville, United States: National Center for Health Statistics, Centers for Disease Control and Prevention

National Center for Health Statistics, Centers for Disease Control and Prevention, United States Census Bureau. United States National Hospital Discharge Survey 1993. Hyattsville, United States: National Center for Health Statistics, Centers for Disease Control and Prevention

National Center for Health Statistics, Centers for Disease Control and Prevention, United States Census Bureau. United States National Hospital Discharge Survey 1994. Hyattsville, United States: National Center for Health Statistics, Centers for Disease Control and Prevention

National Center for Health Statistics, Centers for Disease Control and Prevention, United States Census Bureau. United States National Hospital Discharge Survey 1995. Hyattsville, United States: National Center for Health Statistics, Centers for Disease Control and Prevention

National Center for Health Statistics, Centers for Disease Control and Prevention, United States Census Bureau. United States National Hospital Discharge Survey 1996. Hyattsville, United States: National Center for Health Statistics, Centers for Disease Control and Prevention

National Center for Health Statistics, Centers for Disease Control and Prevention, United States Census Bureau. United States National Hospital Discharge Survey 1997. Hyattsville, United States: National Center for Health Statistics, Centers for Disease Control and Prevention

National Center for Health Statistics, Centers for Disease Control and Prevention, United States Census Bureau. United States National Hospital Discharge Survey 1998. Hyattsville, United States: National Center for Health Statistics, Centers for Disease Control and Prevention

National Center for Health Statistics, Centers for Disease Control and Prevention, United States Census Bureau. United States National Hospital Discharge Survey 1999. Hyattsville, United States: National Center for Health Statistics, Centers for Disease Control and Prevention

National Center for Health Statistics, Centers for Disease Control and Prevention, United States Census Bureau. United States National Hospital Discharge Survey 2000. Hyattsville, United States: National Center for Health Statistics, Centers for Disease Control and Prevention

National Center for Health Statistics, Centers for Disease Control and Prevention, United States Census Bureau. United States National Hospital Discharge Survey 2001. Hyattsville, United States: National Center for Health Statistics, Centers for Disease Control and Prevention

National Center for Health Statistics, Centers for Disease Control and Prevention, United States Census Bureau. United States National Hospital Discharge Survey 2002. Hyattsville, United States: National Center for Health Statistics, Centers for Disease Control and Prevention

National Center for Health Statistics, Centers for Disease Control and Prevention, United States Census Bureau. United States National Hospital Discharge Survey 2003. Hyattsville, United States: National Center for Health Statistics, Centers for Disease Control and Prevention

National Center for Health Statistics, Centers for Disease Control and Prevention, United States Census Bureau. United States National Hospital Discharge Survey 2004. Hyattsville, United States: National Center for Health Statistics, Centers for Disease Control and Prevention

National Center for Health Statistics, Centers for Disease Control and Prevention, United States Census Bureau. United States National Hospital Discharge Survey 2005. Hyattsville, United States: National Center for Health Statistics, Centers for Disease Control and Prevention

National Center for Health Statistics, Centers for Disease Control and Prevention, United States Census Bureau. United States National Hospital Discharge Survey 2006. Hyattsville, United States: National Center for Health Statistics, Centers for Disease Control and Prevention

National Center for Health Statistics, Centers for Disease Control and Prevention, United States Census Bureau. United States National Hospital Discharge Survey 2007. Hyattsville, United States: National Center for Health Statistics, Centers for Disease Control and Prevention

National Center for Health Statistics, Centers for Disease Control and Prevention, United States Census Bureau. United States National Hospital Discharge Survey 2008. Hyattsville, United States: National Center for Health Statistics, Centers for Disease Control and Prevention

National Center for Health Statistics, Centers for Disease Control and Prevention, United States Census Bureau. United States National Hospital Discharge Survey 2009. Hyattsville, United States: National Center for Health Statistics, Centers for Disease Control and Prevention

National Center for Health Statistics, Centers for Disease Control and Prevention, United States Census Bureau. United States National Hospital Discharge Survey 2010. Hyattsville, United States: National Center for Health Statistics, Centers for Disease Control and Prevention

National Center for Health Statistics, Centers for Disease Control and Prevention. United States National Health and Nutrition Examination Survey 2011-2012. Hyattsville, United States: National Center for Health Statistics, Centers for Disease Control and Prevention, 2013

National Center for Health Statistics, Centers for Disease Control and Prevention. United States National Health and Nutrition Examination Survey 2013-2014. Hyattsville, United States: National Center for Health Statistics, Centers for Disease Control and Prevention

National Center for Health Statistics, Centers for Disease Control and Prevention. United States National Health Interview Survey 2014. Hyattsville, United States: National Center for Health Statistics, Centers for Disease Control and Prevention, 2015

National Centre for Social Research (NatCen), World Health Organization (WHO). United Kingdom WHO Multi-country Survey Study on Health and Health System Responsiveness 2000-2001. Geneva, Switzerland: World Health Organization (WHO)

National Institute for Health and Welfare (THL) (Finland). Finland Hospital Discharge Register 2013

National Institute for Health and Welfare (THL) (Finland). Finland Hospital Discharge Register 2014

National Institute for Health Development (Estonia). Estonia Hospital Inpatient Discharges 2012

National Institute for Health Development (Estonia). Estonia Hospital Inpatient Discharges 2013

National Institute for Health Development (Estonia). Estonia Hospital Inpatient Discharges 2014

National Institute for Strategic Health Research (ESKI) (Hungary). Hungary Hospital Inpatient Discharges 2013

National Institute for Strategic Health Research (ESKI) (Hungary). Hungary Hospital Inpatient Discharges 2014

National Institute of Public Health (Mexico), World Health Organization (WHO). Mexico WHO Multi-country Survey Study on Health and Health System Responsiveness 2000-2001. Geneva, Switzerland: World Health Organization (WHO)

National Institute of Public Health (Mexico). Mexico National Health Survey 1999-2000

National Institute of Public Health (Mexico). Mexico National Survey of Health and Nutrition 2005-2006. Cuernavaca, Mexico: National Institute of Public Health (Mexico)

National Institute of Public Health (Mexico). Mexico National Survey of Health and Nutrition 2011-2012. Cuernavaca, Mexico: National Institute of Public Health (Mexico)

National Institute of Public Health (Slovenia). Slovenia National Hospital Health Care Statistics Database 1989

National Institute of Public Health (Slovenia). Slovenia National Hospital Health Care Statistics Database 1991

National Institute of Public Health (Slovenia). Slovenia National Hospital Health Care Statistics Database 1992

National Institute of Public Health (Slovenia). Slovenia National Hospital Health Care Statistics Database 1993

National Institute of Public Health (Slovenia). Slovenia National Hospital Health Care Statistics Database 1994

National Institute of Public Health (Slovenia). Slovenia National Hospital Health Care Statistics Database 1995

National Institute of Public Health (Slovenia). Slovenia National Hospital Health Care Statistics Database 1996

National Institute of Public Health (Slovenia). Slovenia National Hospital Health Care Statistics Database 1997

National Institute of Public Health (Slovenia). Slovenia National Hospital Health Care Statistics Database 1998

National Institute of Public Health (Slovenia). Slovenia National Hospital Health Care Statistics Database 1999
National Institute of Public Health (Slovenia). Slovenia National Hospital Health Care Statistics Database 2000
National Institute of Public Health (Slovenia). Slovenia National Hospital Health Care Statistics Database 2001
National Institute of Public Health (Slovenia). Slovenia National Hospital Health Care Statistics Database 2002
National Institute of Public Health (Slovenia). Slovenia National Hospital Health Care Statistics Database 2003
National Institute of Public Health (Slovenia). Slovenia National Hospital Health Care Statistics Database 2004
National Institute of Public Health (Slovenia). Slovenia National Hospital Health Care Statistics Database 2005
National Institute of Public Health (Slovenia). Slovenia National Hospital Health Care Statistics Database 2006
National Institute of Public Health (Slovenia). Slovenia National Hospital Health Care Statistics Database 2007
National Institute of Public Health (Slovenia). Slovenia National Hospital Health Care Statistics Database 2008
National Institute of Public Health (Slovenia). Slovenia National Hospital Health Care Statistics Database 2013
National Institute of Public Health (Slovenia). Slovenia National Hospital Health Care Statistics Database 2014
National Institute of Public Health (Slovenia). Yugoslavia - Slovenia National Hospital Health Care Statistics Database 1980
National Institute of Public Health (Slovenia). Yugoslavia - Slovenia National Hospital Health Care Statistics Database 1985
National Institute of Public Health (Slovenia). Yugoslavia - Slovenia National Hospital Health Care Statistics Database 1986
National Institute of Public Health (Slovenia). Yugoslavia - Slovenia National Hospital Health Care Statistics Database 1987
National Institute of Public Health (Slovenia). Yugoslavia - Slovenia National Hospital Health Care Statistics Database 1988
National Institute of Public Health (Slovenia). Yugoslavia - Slovenia National Hospital Health Care Statistics Database 1990
National Institute of Statistics (Albania), World Bank (WB). Albania Living Standards Measurement Survey 2002. Washington DC, United States: World Bank (WB)
National Institute of Statistics (Albania), World Bank (WB). Albania Living Standards Measurement Survey 2005. Washington DC, United States: World Bank (WB)
National Institute of Statistics (Cambodia), Statistics Sweden. Cambodia Socio-Economic Survey 2003-2005. Phnom Penh, Cambodia: National Institute of Statistics (Cambodia)
National Institute of Statistics (Cambodia), Statistics Sweden. Cambodia Socio-Economic Survey 2006-2007. Phnom Penh, Cambodia: National Institute of Statistics (Cambodia)
National Institute of Statistics (Cambodia), Statistics Sweden. Cambodia Socio-Economic Survey 2007-2008. Phnom Penh, Cambodia: National Institute of Statistics (Cambodia)
National Institute of Statistics (Niger), World Bank. Niger National Survey on Household Living Conditions and Agriculture 2011-2012
National Institute of Statistics and Censuses (Ecuador). Ecuador Hospital Inpatient Discharges 1993-1997
National Institute of Statistics and Censuses (Ecuador). Ecuador Hospital Inpatient Discharges 1997. Quito, Ecuador: National Institute of Statistics and Censuses (Ecuador)
National Institute of Statistics and Censuses (Ecuador). Ecuador Hospital Inpatient Discharges 1998. Quito, Ecuador: National Institute of Statistics and Censuses (Ecuador)
National Institute of Statistics and Censuses (Ecuador). Ecuador Hospital Inpatient Discharges 1998-2002
National Institute of Statistics and Censuses (Ecuador). Ecuador Hospital Inpatient Discharges 1999. Quito, Ecuador: National Institute of Statistics and Censuses (Ecuador)

National Institute of Statistics and Censuses (Ecuador). Ecuador Hospital Inpatient Discharges 2000. Quito, Ecuador: National Institute of Statistics and Censuses (Ecuador)

National Institute of Statistics and Censuses (Ecuador). Ecuador Hospital Inpatient Discharges 2001. Quito, Ecuador: National Institute of Statistics and Censuses (Ecuador)

National Institute of Statistics and Censuses (Ecuador). Ecuador Hospital Inpatient Discharges 2002. Quito, Ecuador: National Institute of Statistics and Censuses (Ecuador)

National Institute of Statistics and Censuses (Ecuador). Ecuador Hospital Inpatient Discharges 2003. Quito, Ecuador: National Institute of Statistics and Censuses (Ecuador)

National Institute of Statistics and Censuses (Ecuador). Ecuador Hospital Inpatient Discharges 2003-2007

National Institute of Statistics and Censuses (Ecuador). Ecuador Hospital Inpatient Discharges 2004. Quito, Ecuador: National Institute of Statistics and Censuses (Ecuador)

National Institute of Statistics and Censuses (Ecuador). Ecuador Hospital Inpatient Discharges 2005. Quito, Ecuador: National Institute of Statistics and Censuses (Ecuador)

National Institute of Statistics and Censuses (Ecuador). Ecuador Hospital Inpatient Discharges 2006. Quito, Ecuador: National Institute of Statistics and Censuses (Ecuador)

National Institute of Statistics and Censuses (Ecuador). Ecuador Hospital Inpatient Discharges 2007. Quito, Ecuador: National Institute of Statistics and Censuses (Ecuador)

National Institute of Statistics and Censuses (Ecuador). Ecuador Hospital Inpatient Discharges 2008. Quito, Ecuador: National Institute of Statistics and Censuses (Ecuador)

National Institute of Statistics and Censuses (Ecuador). Ecuador Hospital Inpatient Discharges 2008-2012

National Institute of Statistics and Censuses (Ecuador). Ecuador Hospital Inpatient Discharges 2009. Quito, Ecuador: National Institute of Statistics and Censuses (Ecuador)

National Institute of Statistics and Censuses (Ecuador). Ecuador Hospital Inpatient Discharges 2010. Quito, Ecuador: National Institute of Statistics and Censuses (Ecuador)

National Institute of Statistics and Censuses (Ecuador). Ecuador Hospital Inpatient Discharges 2011. Quito, Ecuador: National Institute of Statistics and Censuses (Ecuador)

National Institute of Statistics and Censuses (Ecuador). Ecuador Hospital Inpatient Discharges 2012. Quito, Ecuador: National Institute of Statistics and Censuses (Ecuador), 2013

National Institute of Statistics and Censuses (Ecuador). Ecuador Hospital Inpatient Discharges 2013. Quito, Ecuador: National Institute of Statistics and Censuses (Ecuador)

National Institute of Statistics and Censuses (Ecuador). Ecuador Hospital Inpatient Discharges 2013-2014

National Institute of Statistics and Censuses (Ecuador). Ecuador Hospital Inpatient Discharges 2014. Quito, Ecuador: National Institute of Statistics and Censuses (Ecuador)

National Institute of Statistics and Censuses (Nicaragua), World Bank. Nicaragua Living Standards Measurement Survey 1993

National Institute of Statistics and Censuses (Nicaragua), World Bank. Nicaragua Living Standards Measurement Survey 1998-1999

National Institute of Statistics and Informatics (INEI) (Peru), United Nations Economic Commission for Latin America and the Caribbean (CEPAL), Institute of Research for Development (France). Peru National Household Survey 2007. Lima, Peru: National Institute of Statistics and Informatics (INEI) (Peru)

National Institute of Statistics and Informatics (INEI) (Peru), United Nations Economic Commission for Latin America and the Caribbean (CEPAL), Institute of Research for Development (France). Peru National Household Survey 2008. Lima, Peru: National Institute of Statistics and Informatics (INEI) (Peru)

National Institute of Statistics and Informatics (INEI) (Peru). Peru National Household Survey 2005. Lima, Peru: National Institute of Statistics and Informatics (INEI) (Peru)

National Institute of Statistics and Informatics (INEI) (Peru). Peru National Household Survey 2006. Lima, Peru: National Institute of Statistics and Informatics (INEI) (Peru)

National Institute of Statistics and Informatics (INEI) (Peru). Peru National Household Survey 2009. Lima, Peru: National Institute of Statistics and Informatics (INEI) (Peru)

National Institute of Statistics and Informatics (INEI) (Peru). Peru National Household Survey 2010. Lima, Peru: National Institute of Statistics and Informatics (INEI) (Peru)

National Institute of Statistics and Informatics (INEI) (Peru). Peru National Household Survey, Second Quarter 1998. Lima, Peru: National Institute of Statistics and Informatics (INEI) (Peru)

National Institute of Statistics and Informatics (INEI) (Peru). Peru National Household Survey, Second Quarter 1999. Lima, Peru: National Institute of Statistics and Informatics (INEI) (Peru)

National Institute of Statistics and Informatics (INEI) (Peru). Peru National Household Survey, Second Quarter 2000. Lima, Peru: National Institute of Statistics and Informatics (INEI) (Peru)

National Institute of Statistics and Informatics (Peru), World Bank (WB). Peru Living Standards Measurement Survey 1990

National Institute of Statistics and Informatics (Peru), World Bank (WB). Peru Living Standards Measurement Survey 1994

National Institute of Statistics and Informatics (Peru), World Bank. Peru Living Standards Measurement Survey 1991. Washington DC, United States: World Bank

National Institute of Statistics of Rwanda. Rwanda Integrated Household Living Conditions Survey 2010-2011. Kigali, Rwanda: National Institute of Statistics of Rwanda

National Office of Statistics (Cuba). Cuba Statistical Yearbook 2012. Havana, Cuba: National Office of Statistics (Cuba)

National Public Health Institute (Finland). Finland Hospital Discharge Register 1988

National Public Health Institute (Finland). Finland Hospital Discharge Register 1989

National Public Health Institute (Finland). Finland Hospital Discharge Register 1990

National Public Health Institute (Finland). Finland Hospital Discharge Register 1991

National Public Health Institute (Finland). Finland Hospital Discharge Register 1992

National Public Health Institute (Finland). Finland Hospital Discharge Register 1993

National Public Health Institute (Finland). Finland Hospital Discharge Register 1994

National Public Health Institute (Finland). Finland Hospital Discharge Register 1995

National Public Health Institute (Finland). Finland Hospital Discharge Register 1996

National Public Health Institute (Finland). Finland Hospital Discharge Register 1997

National Public Health Institute (Finland). Finland Hospital Discharge Register 1998

National Public Health Institute (Finland). Finland Hospital Discharge Register 1999

National School of Public Health (Greece), World Health Organization (WHO). Greece WHO Multi-country Survey Study on Health and Health System Responsiveness 2000-2001

National State Statistical Agency (Tajikistan), World Bank. Tajikistan Living Standards Measurement Survey 2003

National State Statistical Agency (Tajikistan), World Bank. Tajikistan Living Standards Measurement Survey 2007

National State Statistical Agency (Tajikistan), World Bank. Tajikistan Living Standards Measurement Survey 2009

National Statistical Committee of the Kyrgyz Republic, Research Triangle Institute, Inc. (RTI), World Bank. Kyrgyzstan Living Standards Measurement Survey 1997. Washington DC, United States: World Bank

National Statistical Committee of the Kyrgyz Republic, Research Triangle Institute, Inc. (RTI), World Bank. Kyrgyzstan Living Standards Measurement Survey 1998. Washington DC, United States: World Bank

National Statistical Committee of the Kyrgyz Republic, SIAR Research and Consulting (Kyrgyzstan), World Health Organization (WHO). Kyrgyzstan WHO Multi-country Survey Study on Health and Health System Responsiveness 2000-2001. Geneva, Switzerland: World Health Organization (WHO)

National Statistical Institute of Bulgaria. Bulgaria Living Standards Measurement Survey 2003. Washington DC, United States: World Bank

National Statistical Office of Malawi, World Bank. Malawi Living Standards Measurement Survey 2004-2005

National Statistical Office of Malawi. Malawi Integrated Household Survey 2010 (IHS3 - Year: 1). Dataset downloaded from [http://go.worldbank.org/NOXNI9YDS0] on [September 19, 2015]

National Statistical Office of Malawi. Malawi Integrated Household Survey 2013 (IHS3 - Year: 2). Dataset downloaded from [http://go.worldbank.org/NOXNI9YDS0] on [September 15, 2015]

National Statistics Directorate (Timor-Leste), World Bank. Timor-Leste Living Standards and Measurement Survey 2001. Washington DC, United States: World Bank

National Statistics Directorate (Timor-Leste), World Bank. Timor-Leste Living Standards and Measurement Survey 2007-2008. Washington DC, United States: World Bank

Nazareth Hospital, Shillong, JSS Hospital, Mysore, King George's Medical University (India). India Hospital Inpatient Data 2014-2017.

Nazareth Hospital, Shillong. India - Shillong Nazareth Hospital Inpatient Discharges 2014

Ndibazza J, Lule S, Nampijja M, Mpairwe H, Oduru G, Kiggundu M, Akello M, Muhangi L, Elliott AM. A description of congenital anomalies among infants in Entebbe, Uganda. Birth Defects Res A Clin Mol Teratol. 2011; 91(9): 857-61

Nembhard WN, Pathak EB, Schocken DD. Racial/ethnic disparities in mortality related to congenital heart defects among children and adults in the United States. Ethn Dis. 2008; 18(4): 442-9

Nembhard WN, Waller DK, Sever LE, Canfield MA. Patterns of first-year survival among infants with selected congenital anomalies in Texas, 1995-1997. Teratology. 2001; 64(5): 267-75

Netherlands - Northern Netherlands EUROCAT Data 1993-1998 - WHO as it appears in European Surveillance of Congenital Anomalies (EUROCAT), International Centre on Birth Defects, World Health Organization (WHO). World Atlas of Birth Defects. 2nd ed. Geneva, Switzerland: World Health Organization (WHO), 2003

Netherlands - Northern Netherlands EUROCAT Data 2001 - ICBDMS as it appears in International Clearinghouse for Birth Defects Monitoring Systems. International Clearinghouse for Birth Defects Monitoring Systems Annual Report 2003. Rome, Italy: International Centre on Birth Defects

Netherlands - Northern Netherlands EUROCAT Data 2002 - ICBDMS as it appears in International Clearinghouse for Birth Defects Monitoring Systems. International Clearinghouse for Birth Defects Monitoring Systems Annual Report 2004. Rome, Italy: International Centre on Birth Defects, 2006

Netherlands - Northern Netherlands EUROCAT Data 2003 - ICBDSR as it appears in International Clearinghouse for Birth Defects Surveillance and Research. International Clearinghouse for Birth Defects Surveillance and Research Annual Report 2005. Rome, Italy: International Clearinghouse for Birth Defects Surveillance and Research, 2007

Netherlands - Northern Netherlands EUROCAT Data 2004 - ICBDSR as it appears in International Clearinghouse for Birth Defects Surveillance and Research. International Clearinghouse for Birth Defects Surveillance and Research Annual Report 2006. Rome, Italy: International Clearinghouse for Birth Defects Surveillance and Research, 2007

Netherlands - Northern Netherlands EUROCAT Data 2005 - ICBDSR as it appears in International Clearinghouse for Birth Defects Surveillance and Research. International Clearinghouse for Birth Defects Surveillance and Research Annual Report 2007. Rome, Italy: International Clearinghouse for Birth Defects Surveillance and Research, 2008

Netherlands - Northern Netherlands EUROCAT Data 2007 - ICBDSR as it appears in International Clearinghouse for Birth Defects Surveillance and Research. International Clearinghouse for Birth Defects Surveillance and Research Annual Report 2009. Rome, Italy: International Clearinghouse for Birth Defects Surveillance and Research

Netherlands - Northern Netherlands EUROCAT Data 2008 - ICBDSR as it appears in International Clearinghouse for Birth Defects Surveillance and Research. International Clearinghouse for Birth Defects Surveillance and Research Annual Report 2010. Rome, Italy: International Clearinghouse for Birth Defects Surveillance and Research, 2011

Netherlands - Northern Netherlands EUROCAT Data 2009 - ICBDSR as it appears in International Clearinghouse for Birth Defects Surveillance and Research. International Clearinghouse for Birth Defects Surveillance and Research Annual Report 2011. Rome, Italy: International Clearinghouse for Birth Defects Surveillance and Research, 2012

Netherlands - Northern Netherlands EUROCAT Data 2010 - ICBDSR as it appears in International Clearinghouse for Birth Defects Surveillance and Research. International Clearinghouse for Birth Defects Surveillance and Research Annual Report 2012. Rome, Italy: International Clearinghouse for Birth Defects Surveillance and Research, 2013

Netherlands - Northern Netherlands EUROCAT Data 2012 - ICBDSR as it appears in International Clearinghouse for Birth Defects Surveillance and Research. International Clearinghouse for Birth Defects Surveillance and Research Annual Report 2014. Rome, Italy: International Clearinghouse for Birth Defects Surveillance and Research, 2016

New Zealand Birth Defects Monitoring Program Data 2001 - ICBDMS as it appears in International Clearinghouse for Birth Defects Monitoring Systems. International Clearinghouse for Birth Defects Monitoring Systems Annual Report 2003. Rome, Italy: International Centre on Birth Defects

New Zealand Birth Defects Monitoring Program Data 2002 - ICBDMS as it appears in International Clearinghouse for Birth Defects Monitoring Systems. International Clearinghouse for Birth Defects Monitoring Systems Annual Report 2004. Rome, Italy: International Centre on Birth Defects, 2006

New Zealand Birth Defects Monitoring Program Data 2003 - ICBDSR as it appears in International Clearinghouse for Birth Defects Surveillance and Research. International Clearinghouse for Birth Defects Surveillance and Research Annual Report 2005. Rome, Italy: International Clearinghouse for Birth Defects Surveillance and Research, 2007

New Zealand Birth Defects Monitoring Program Data 2005 - ICBDSR as it appears in International Clearinghouse for Birth Defects Surveillance and Research. International Clearinghouse for Birth Defects Surveillance and Research Annual Report 2007. Rome, Italy: International Clearinghouse for Birth Defects Surveillance and Research, 2008

New Zealand Birth Defects Monitoring Programme Data 1993-1998 - WHO as it appears in European Surveillance of Congenital Anomalies (EUROCAT), International Centre on Birth Defects, World Health Organization (WHO). World Atlas of Birth Defects. 2nd ed. Geneva, Switzerland: World Health Organization (WHO), 2003

New Zealand Birth Defects Monitoring Programme Data 2007 - ICBDSR as it appears in International Clearinghouse for Birth Defects Surveillance and Research. International Clearinghouse for Birth Defects Surveillance and Research Annual Report 2009. Rome, Italy: International Clearinghouse for Birth Defects Surveillance and Research

New Zealand Birth Defects Registry Data 2008 - ICBDSR as it appears in International Clearinghouse for Birth Defects Surveillance and Research. International Clearinghouse for Birth Defects Surveillance and Research Annual Report 2010. Rome, Italy: International Clearinghouse for Birth Defects Surveillance and Research, 2011

New Zealand Birth Defects Registry Data 2009 - ICBDSR as it appears in International Clearinghouse for Birth Defects Surveillance and Research. International Clearinghouse for Birth Defects Surveillance and Research Annual Report 2011. Rome, Italy: International Clearinghouse for Birth Defects Surveillance and Research, 2012

New Zealand Birth Defects Registry Data 2010 - ICBDSR as it appears in International Clearinghouse for Birth Defects Surveillance and Research. International Clearinghouse for Birth Defects Surveillance and Research Annual Report 2012. Rome, Italy: International Clearinghouse for Birth Defects Surveillance and Research, 2013

New Zealand Birth Defects Registry Data 2012 - ICBDSR as it appears in International Clearinghouse for Birth Defects Surveillance and Research. International Clearinghouse for Birth Defects Surveillance and Research Annual Report 2014. Rome, Italy: International Clearinghouse for Birth Defects Surveillance and Research, 2016

New Zealand Congenital Anomalies Monitoring Programme Data 2004 - ICBDSR as it appears in International Clearinghouse for Birth Defects Surveillance and Research. International Clearinghouse for Birth Defects Surveillance and Research Annual Report 2006. Rome, Italy: International Clearinghouse for Birth Defects Surveillance and Research, 2007

Ng B, Hokanson J. Missed congenital heart disease in neonates. Congenit Heart Dis. 2010; 5(3): 292-6

NHS England. United Kingdom - England Hospital Episode Statistics 2001-2002

NHS England. United Kingdom - England Hospital Episode Statistics 2003-2007

NHS England. United Kingdom - England Hospital Episode Statistics 2008-2012

NHS England. United Kingdom - England Hospital Episode Statistics 2013-2014

Niwa K, Hamada H, Nakazawa M, Terai M, Tateno S, Sugimoto S, Watanabe H, Murakami A, Ohta M, Ishizawa A, Katoki T, Mori K, Yasui S, Kawahira Y, Akagi T, Haraguchi N, Gatzoulis MA, Japanese Multi-center Study Group. Mortality and risk factors for late deaths in tetralogy of Fallot: the Japanese Nationwide Multicentric Survey. Cardiol Young. 2002; 12(5): 453-60

Nogueira PR, Rassi S, Corrêa K de S. Epidemiological, clinical e therapeutic profile of heart failure in a tertiary hospital. Arq Bras Cardiol. 2010; 95(3): 392-8

Norway Medical Birth Registry Congenital Anomaly Data 1993-1998 - WHO as it appears in European Surveillance of Congenital Anomalies (EUROCAT), International Centre on Birth Defects, World Health Organization (WHO). World Atlas of Birth Defects. 2nd ed. Geneva, Switzerland: World Health Organization (WHO), 2003

Norway Medical Birth Registry Congenital Anomaly Data 2001 - ICBDMS as it appears in International Clearinghouse for Birth Defects Monitoring Systems. International Clearinghouse for Birth Defects Monitoring Systems Annual Report 2003. Rome, Italy: International Centre on Birth Defects

Norway Medical Birth Registry Congenital Anomaly Data 2002 - ICBDMS as it appears in International Clearinghouse for Birth Defects Monitoring Systems. International Clearinghouse for Birth Defects Monitoring Systems Annual Report 2004. Rome, Italy: International Centre on Birth Defects, 2006

Norway Medical Birth Registry Congenital Anomaly Data 2003 - ICBDSR as it appears in International Clearinghouse for Birth Defects Surveillance and Research. International Clearinghouse for Birth Defects Surveillance and Research Annual Report 2005. Rome, Italy: International Clearinghouse for Birth Defects Surveillance and Research, 2007

Norway Medical Birth Registry Congenital Anomaly Data 2004 - ICBDSR as it appears in International Clearinghouse for Birth Defects Surveillance and Research. International Clearinghouse for Birth Defects Surveillance and Research Annual Report 2006. Rome, Italy: International Clearinghouse for Birth Defects Surveillance and Research, 2007

Norway Medical Birth Registry Congenital Anomaly Data 2005 - ICBDSR as it appears in International Clearinghouse for Birth Defects Surveillance and Research. International Clearinghouse for Birth Defects Surveillance and Research Annual Report 2007. Rome, Italy: International Clearinghouse for Birth Defects Surveillance and Research, 2008

Norway Medical Birth Registry Congenital Anomaly Data 2007 - ICBDSR as it appears in International Clearinghouse for Birth Defects Surveillance and Research. International Clearinghouse for Birth Defects Surveillance and Research Annual Report 2009. Rome, Italy: International Clearinghouse for Birth Defects Surveillance and Research

Norway Medical Birth Registry Congenital Anomaly Data 2008 - ICBDSR as it appears in International Clearinghouse for Birth Defects Surveillance and Research. International Clearinghouse for Birth Defects Surveillance and Research Annual Report 2010. Rome, Italy: International Clearinghouse for Birth Defects Surveillance and Research, 2011

Norway Medical Birth Registry Congenital Anomaly Data 2009 - ICBDSR as it appears in International Clearinghouse for Birth Defects Surveillance and Research. International Clearinghouse for Birth Defects Surveillance and Research Annual Report 2011. Rome, Italy: International Clearinghouse for Birth Defects Surveillance and Research, 2012

Norway Medical Birth Registry Congenital Anomaly Data 2010 - ICBDSR as it appears in International Clearinghouse for Birth Defects Surveillance and Research. International Clearinghouse for Birth Defects Surveillance and Research Annual Report 2012. Rome, Italy: International Clearinghouse for Birth Defects Surveillance and Research, 2013

Norwegian Directorate of Health. Norway Patient Register 1973

Norwegian Directorate of Health. Norway Patient Register 1975

Norwegian Directorate of Health. Norway Patient Register 1977

Norwegian Directorate of Health. Norway Patient Register 1978

Norwegian Directorate of Health. Norway Patient Register 1979

Norwegian Directorate of Health. Norway Patient Register 1980

Norwegian Directorate of Health. Norway Patient Register 1981

Norwegian Directorate of Health. Norway Patient Register 1982

Norwegian Directorate of Health. Norway Patient Register 1983

Norwegian Directorate of Health. Norway Patient Register 1984

Norwegian Directorate of Health. Norway Patient Register 1985

Norwegian Directorate of Health. Norway Patient Register 1986

Norwegian Directorate of Health. Norway Patient Register 1987

Norwegian Directorate of Health. Norway Patient Register 1988

Norwegian Directorate of Health. Norway Patient Register 1989

Norwegian Directorate of Health. Norway Patient Register 1990

Norwegian Directorate of Health. Norway Patient Register 1991

Norwegian Directorate of Health. Norway Patient Register 1992

Norwegian Directorate of Health. Norway Patient Register 1993

Norwegian Directorate of Health. Norway Patient Register 1994

Norwegian Directorate of Health. Norway Patient Register 1995

Norwegian Directorate of Health. Norway Patient Register 1996

Norwegian Directorate of Health. Norway Patient Register 1997

Norwegian Directorate of Health. Norway Patient Register 1998

Norwegian Directorate of Health. Norway Patient Register 1999

Norwegian Directorate of Health. Norway Patient Register 2000

Norwegian Directorate of Health. Norway Patient Register 2001

Norwegian Directorate of Health. Norway Patient Register 2002

Norwegian Directorate of Health. Norway Patient Register 2003

Norwegian Directorate of Health. Norway Patient Register 2008-2012

Norwegian Directorate of Health. Norway Patient Register 2009

Norwegian Directorate of Health. Norway Patient Register 2010

Norwegian Directorate of Health. Norway Patient Register 2011

Norwegian Directorate of Health. Norway Patient Register 2012

Norwegian Directorate of Health. Norway Patient Register 2013

Norwegian Directorate of Health. Norway Patient Register 2014

Ola BA, Adewuya AO, Ajayi OE, Akintomide AO, Oginni OO, Ologun YA. Relationship between depression and quality of life in Nigerian outpatients with heart failure. J Psychosom Res. 2006; 61(6): 797-800

O'Malley CD, Shaw GM, Wasserman CR, Lammer EJ. Epidemiologic characteristics of conotruncal heart defects in California, 1987-1988. Teratology. 1996; 53(6): 374-7

Onan IS, Erek E, Haydin S, Onan B, Kocyigit OI, Topuz U, Odemis E, Yeniterzi M, Bakir I. Clinical outcome of patients in a start-up congenital heart surgery program in Turkey. Artif Organs. 2013; 37(1): E18-23

Ooshima A, Fukushige J, Ueda K. Incidence of structural cardiac disorders in neonates: an evaluation by color Doppler echocardiography and the results of a 1-year follow-up. Cardiology. 1995; 86(5): 402-6

Organization for Economic Co-operation and Development (OECD). OECD Health Statistics. Paris, France: Organization for Economic Co-operation and Development (OECD)

Orün UA, Bilici M, Demirçeken FG, Tosun M, Ocal B, Cavuşoğlu YH, Erdoğan D, Senocak F, Karademir S. Gastrointestinal system malformations in children are associated with congenital heart defects. Anatol J Cardiol. 2011; 11(2): 146-9

Osiovich H, Phillipos E, Byrne P, Robertson M. Hypoplastic left heart syndrome: "to treat or not to treat.". J Perinatol. 2000; 20(6): 363-5

Oztarhan K, Gedikbasi A, Yildirim D, Arslan O, Adal E, Kavuncuoglu S, Ozbek S, Ceylan Y. Prevalence and distribution of congenital abnormalities in Turkey: differences between the prenatal and postnatal periods. Congenit Anom (Kyoto). 2010; 50(4): 221-5

Palestinian Central Bureau of Statistics. Palestine Demographic and Health Survey 2004

Pan American Health Organization (PAHO), Center for Demography and Ecology, University of Wisconsin-Madison, Inter-University Consortium for Political and Social Research (ICPSR), Chronic Disease Research Centre (CDRC), University of the West Indies. Barbados - Bridgetown Survey on Health, Well-Being, and Aging in Latin America and the Caribbean 1999-2000. Ann Arbor, United States: Inter-University Consortium for Political and Social Research (ICPSR)

Pan American Health Organization (PAHO), Center for Demography and Ecology, University of Wisconsin-Madison, Inter-University Consortium for Political and Social Research (ICPSR), College of the Northern Border (COLEF), Research in Health and Demographics (INSAD), National Institute of Medical Sciences and Nutrition Salvador Zubirán. Mexico - Mexico City Survey on Health, Well-Being, and Aging in Latin America and the Caribbean 1999-2000. Ann Arbor, United States: Inter-University Consortium for Political and Social Research (ICPSR)

Pan American Health Organization (PAHO), Center for Demography and Ecology, University of Wisconsin-Madison, Inter-University Consortium for Political and Social Research (ICPSR), Institute of Nutrition and Food Technology (INTA), University of Chile, Center for Geriatrics and Gerontology, Pontifical Catholic University of Chile. Chile - Santiago Survey on Health, Well-Being, and Aging in Latin America and the Caribbean 1999-2000. Ann Arbor, United States: Inter-University Consortium for Political and Social Research (ICPSR)

Panel Study of Income Dynamics, 2005 public use dataset. Produced and distributed by the University of Michigan with primary funding from the National Science Foundation, the National Institute of Aging, and the National Institute of Child Health and Human Development. Ann Arbor, MI, (2011)

Panel Study of Income Dynamics, 2007 public use dataset. Produced and distributed by the University of Michigan with primary funding from the National Science Foundation, the National Institute of Aging, and the National Institute of Child Health and Human Development. Ann Arbor, MI, (2011)

Pangkanon S, Sawasdivorn S, Kuptanon C, Chotigeat U, Vandepitte W. Establishing of National Birth Defects Registry in Thailand. J Med Assoc Thai. 2014; 97 Suppl 6: S182-8

Paraguay Latin American Collaborative Study of Congenital Malformations Data 1993-1998 - WHO as it appears in European Surveillance of Congenital Anomalies (EUROCAT), International Centre on Birth Defects, World Health Organization (WHO). World Atlas of Birth Defects. 2nd ed. Geneva, Switzerland: World Health Organization (WHO), 2003

Philippine Health Insurance Corporation. Philippine Health Insurance Corporation Claims 2013-2016

Planning Commission (Tanzania), University of Dar es Salaam, World Bank. Tanzania Living Standards Measurement Study 1993-1994. Washington DC, United States: World Bank

Planning Institute of Jamaica, Statistical Institute of Jamaica. Jamaica Survey of Living Conditions 1988. Kingston, Jamaica: Planning Institute of Jamaica

Planning Institute of Jamaica, Statistical Institute of Jamaica. Jamaica Survey of Living Conditions 1991

Planning Institute of Jamaica, Statistical Institute of Jamaica. Jamaica Survey of Living Conditions 1992

Planning Institute of Jamaica, Statistical Institute of Jamaica. Jamaica Survey of Living Conditions 1993

Planning Institute of Jamaica, Statistical Institute of Jamaica. Jamaica Survey of Living Conditions 1994

Planning Institute of Jamaica, Statistical Institute of Jamaica. Jamaica Survey of Living Conditions 1995

Planning Institute of Jamaica, Statistical Institute of Jamaica. Jamaica Survey of Living Conditions 1996

Planning Institute of Jamaica, Statistical Institute of Jamaica. Jamaica Survey of Living Conditions 1997

Planning Institute of Jamaica, Statistical Institute of Jamaica. Jamaica Survey of Living Conditions 1999

Planning Institute of Jamaica, Statistical Institute of Jamaica. Jamaica Survey of Living Conditions 2000

Pontifical Xavierian University, World Health Organization (WHO). Colombia WHO Multi-country Survey Study on Health and Health System Responsiveness 2000-2001. Geneva, Switzerland: World Health Organization (WHO)

Portugal - Southern Portugal Congenital Anomaly Data 1993-1998 - WHO as it appears in European Surveillance of Congenital Anomalies (EUROCAT), International Centre on Birth Defects, World Health Organization (WHO). World Atlas of Birth Defects. 2nd ed. Geneva, Switzerland: World Health Organization (WHO), 2003

Postoev VA, Nieboer E, Grjibovski AM, Odland JO. Prevalence of birth defects in an Arctic Russian setting from 1973 to 2011: a register-based study. Reprod Health. 2015; 12: 37

Pradat P, Francannet C, Harris JA, Robert E. The epidemiology of cardiovascular defects, part I: a study based on data from three large registries of congenital malformations. Pediatr Cardiol. 2003; 24(3): 195-221

Public Health Agency of Sweden, Statistics Sweden. Sweden National Survey of Public Health 2014

Public Health Authority of the Slovak Republic, World Health Organization (WHO). Slovakia WHO Multi-country Survey Study on Health and Health System Responsiveness 2000

Public Opinion Research Center (CBOS) (Poland), World Health Organization (WHO). Poland WHO Multi-country Survey Study on Health and Health System Responsiveness 2000-2001. Geneva, Switzerland: World Health Organization (WHO)

RAND Corporation, University of Indonesia. Indonesia Family Life Survey 1993-1994. Santa Monica, United States: RAND Corporation

Reich JD, Auld D, Hulse E, Sullivan K, Campbell R. The Pediatric Radiofrequency Ablation Registry's experience with Ebstein's anomaly. Pediatric Electrophysiology Society. J Cardiovasc Electrophysiol. 1998; 9(12): 1370-7

Ristivojevic A, Djokic PL, Katanic D, Dobanovacki D, Privrodski JJ. Epidemiology and structure of congenital anomalies of the newborns in the region of Novi Sad (Vojvodina, Serbia) in 1996 and 2006. Vojnosanit Pregl. 2016; 73(5): 442-8

Rizvi SF, Mustafa G, Kundi A, Khan MA. PREVALENCE OF CONGENITAL HEART DISEASE IN RURAL COMMUNITIES OF PAKISTAN. J Ayub Med Coll Abbottabad. 2015; 27(1): 124-7

Robert Koch Institute. Germany Health Update 2009-2010. Berlin, Germany: Robert Koch Institute

Robida A, Folger GM, Hajar HA. Incidence of congenital heart disease in Qatari children. Int J Cardiol. 1997; 60(1): 19-22

Roos-Hesselink J, Perlroth MG, McGhie J, Spitaels S. Atrial arrhythmias in adults after repair of tetralogy of Fallot. Correlations with clinical, exercise, and echocardiographic findings. Circulation. 1995; 91(8): 2214-9

Russell HM, Pasquali SK, Jacobs JP, Jacobs ML, O'Brien SM, Mavroudis C, Backer CL. Outcomes of repair of common arterial trunk with truncal valve surgery: a review of the society of thoracic surgeons congenital heart surgery database. Ann Thorac Surg. 2012; 93(1): 164-9

Russia - Moscow Regional Registry of Congenital Malformation Data 2001 - ICBDMS as it appears in International Clearinghouse for Birth Defects Monitoring Systems. International Clearinghouse for Birth Defects Monitoring Systems Annual Report 2003. Rome, Italy: International Centre on Birth Defects

Russia - Moscow Regional Registry of Congenital Malformation Data 2002 - ICBDMS as it appears in International Clearinghouse for Birth Defects Monitoring Systems. International Clearinghouse for Birth Defects Monitoring Systems Annual Report 2004. Rome, Italy: International Centre on Birth Defects, 2006

Russia - Moscow Regional Registry of Congenital Malformation Data 2003 - ICBDSR as it appears in International Clearinghouse for Birth Defects Surveillance and Research. International Clearinghouse for Birth Defects Surveillance and Research Annual Report 2005. Rome, Italy: International Clearinghouse for Birth Defects Surveillance and Research, 2007

Russia - Moscow Regional Registry of Congenital Malformation Data 2004 - ICBDSR as it appears in International Clearinghouse for Birth Defects Surveillance and Research. International Clearinghouse for Birth Defects Surveillance and Research Annual Report 2006. Rome, Italy: International Clearinghouse for Birth Defects Surveillance and Research, 2007
Russia - Moscow Regional Registry of Congenital Malformation Data 2005 - ICBDSR as it appears in International Clearinghouse for Birth Defects Surveillance and Research. International Clearinghouse for Birth Defects Surveillance and Research Annual Report 2007. Rome, Italy: International Clearinghouse for Birth Defects Surveillance and Research, 2008
Russia - Moscow Regional Registry of Congenital Malformation Data 2007 - ICBDSR as it appears in International Clearinghouse for Birth Defects Surveillance and Research. International Clearinghouse for Birth Defects Surveillance and Research Annual Report 2009. Rome, Italy: International Clearinghouse for Birth Defects Surveillance and Research
Russia - Moscow Regional Registry of Congenital Malformation Data 2008 - ICBDSR as it appears in International Clearinghouse for Birth Defects Surveillance and Research. International Clearinghouse for Birth Defects Surveillance and Research Annual Report 2010. Rome, Italy: International Clearinghouse for Birth Defects Surveillance and Research, 2011
Russia - Moscow Regional Registry of Congenital Malformation Data 2009 - ICBDSR as it appears in International Clearinghouse for Birth Defects Surveillance and Research. International Clearinghouse for Birth Defects Surveillance and Research Annual Report 2011. Rome, Italy: International Clearinghouse for Birth Defects Surveillance and Research, 2012
Russia - Tomsk Birth Defects Monitoring Programme Data 1993-1998 - WHO as it appears in European Surveillance of Congenital Anomalies (EUROCAT), International Centre on Birth Defects, World Health Organization (WHO). World Atlas of Birth Defects. 2nd ed. Geneva, Switzerland: World Health Organization (WHO), 2003
Russia Longitudinal Monitoring Survey (RLMS-HSE), Round V 1994. National Research University Higher School of Economics, ZAO Demoscope, Carolina Population Center, Univeristy of North Carolina at Chapel Hill, Institute of Sociology, Russian Academy of Sciences
Russia Longitudinal Monitoring Survey (RLMS-HSE), Round VI 1995. National Research University Higher School of Economics, ZAO Demoscope, Carolina Population Center, Univeristy of North Carolina at Chapel Hill, Institute of Sociology, Russian Academy of Sciences
Russia Longitudinal Monitoring Survey (RLMS-HSE), Round VII 1996. National Research University Higher School of Economics, ZAO Demoscope, Carolina Population Center, Univeristy of North Carolina at Chapel Hill, Institute of Sociology, Russian Academy of Sciences
Russia Longitudinal Monitoring Survey (RLMS-HSE), Round VIII 1998-1999. National Research University Higher School of Economics, ZAO Demoscope, Carolina Population Center, Univeristy of North Carolina at Chapel Hill, Institute of Sociology, Russian Academy of Sciences
Russia Longitudinal Monitoring Survey (RLMS-HSE), Round X 2001. National Research University Higher School of Economics, ZAO Demoscope, Carolina Population Center, Univeristy of North Carolina at Chapel Hill, Institute of Sociology, Russian Academy of Sciences
Russia Longitudinal Monitoring Survey (RLMS-HSE), Round XI 2002. National Research University Higher School of Economics, ZAO Demoscope, Carolina Population Center, Univeristy of North Carolina at Chapel Hill, Institute of Sociology, Russian Academy of Sciences
Russia Longitudinal Monitoring Survey (RLMS-HSE), Round XII 2003. National Research University Higher School of Economics, ZAO Demoscope, Carolina Population Center, Univeristy of North Carolina at Chapel Hill, Institute of Sociology, Russian Academy of Sciences
Russia Longitudinal Monitoring Survey (RLMS-HSE), Round XIII 2004. National Research University Higher School of Economics, ZAO Demoscope, Carolina Population Center, Univeristy of North Carolina at Chapel Hill, Institute of Sociology, Russian Academy of Sciences
Russia Longitudinal Monitoring Survey (RLMS-HSE), Round XIV 2005. National Research University Higher School of Economics, ZAO Demoscope, Carolina Population Center, Univeristy of North Carolina at Chapel Hill, Institute of Sociology, Russian Academy of Sciences

Russia Longitudinal Monitoring Survey (RLMS-HSE), Round XV 2006. National Research University Higher School of Economics, ZAO Demoscope, Carolina Population Center, Univeristy of North Carolina at Chapel Hill, Institute of Sociology, Russian Academy of Sciences

Russia Longitudinal Monitoring Survey (RLMS-HSE), Round XVI 2007. National Research University Higher School of Economics, ZAO Demoscope, Carolina Population Center, Univeristy of North Carolina at Chapel Hill, Institute of Sociology, Russian Academy of Sciences

Russia Longitudinal Monitoring Survey (RLMS-HSE), Round XVII 2008. National Research University Higher School of Economics, ZAO Demoscope, Carolina Population Center, Univeristy of North Carolina at Chapel Hill, Institute of Sociology, Russian Academy of Sciences

Russia Longitudinal Monitoring Survey (RLMS-HSE), Round XVIII 2009. National Research University Higher School of Economics, ZAO Demoscope, Carolina Population Center, Univeristy of North Carolina at Chapel Hill, Institute of Sociology, Russian Academy of Sciences

Rutledge JM, Nihill MR, Fraser CD, Smith OE, McMahon CJ, Bezold LI. Outcome of 121 patients with congenitally corrected transposition of the great arteries. Pediatr Cardiol. 2002; 23(2): 137-45

Rywik TM, Kołodziej P, Targoński R, Fedyk-Łukasik M, Nowicka A, Zinka E, Zbyszyński B, Achremczyk P, Górski J, Muder A, Sadowski J, Leszek P, Kurjata P, Broda G, Korewicki J. Characteristics of the heart failure population in Poland: ZOPAN, a multicentre national programme. Kardiol Pol. 2011; 69(1): 24-31

Samánek M, Slavík Z, Krejcír M. Seasonal differences in the incidence of congenital heart defects. Czech Med. 1991; 14(3): 146-55

Samánek M, Slavík Z, Zborilová B, Hrobonová V, Vorísková M, Skovránek J. Prevalence, treatment, and outcome of heart disease in live-born children: a prospective analysis of 91,823 live-born children. Pediatr Cardiol. 1989; 10(4): 205-11

Samánek M. Boy:girl ratio in children born with different forms of cardiac malformation: a population-based study. Pediatr Cardiol. 1994; 15(2): 53-7

Samánek M. Children with congenital heart disease: probability of natural survival. Pediatr Cardiol. 1992; 13(3): 152-8

Samánek M. Congenital heart malformations: prevalence, severity, survival, and quality of life. Cardiol Young. 2000; 10(3): 179-85

Sarkar S, Patra C, Dasgupta MK, Nayek K, Karmakar PR. Prevalence of congenital anomalies in neonates and associated risk factors in a tertiary care hospital in eastern India. J Clin Neonatol. 2013; 2(3): 131-4

Saudi Arabia Medical Service Department Birth Defect Registry Data 2010 - ICBDSR as it appears in International Clearinghouse for Birth Defects Surveillance and Research. International Clearinghouse for Birth Defects Surveillance and Research Annual Report 2012. Rome, Italy: International Clearinghouse for Birth Defects Surveillance and Research, 2013

Scotland - Greater Glasgow NHS Board Congenital Anomalies Register Data 1993-1998 - WHO as it appears in European Surveillance of Congenital Anomalies (EUROCAT), International Centre on Birth Defects, World Health Organization (WHO). World Atlas of Birth Defects. 2nd ed. Geneva, Switzerland: World Health Organization (WHO), 2003

Scott DJ, Campbell DN, Clarke DR, Goldberg SP, Karlin DR, Mitchell MB. Twenty-year surgical experience with congenital supravalvar aortic stenosis. Ann Thorac Surg. 2009; 87(5): 1501-8

Seow S-C, Chai P, Lee Y-P, Chan Y-H, Kwok BWK, Yeo T-C, Chia B-L. Heart Failure Mortality in Southeast Asian Patients With Left Ventricular Systolic Dysfunction. J Card Fail. 2007; 13(6): 476-81

Sever LE, Hessol NA, Gilbert ES, McIntyre JM. The prevalence at birth of congenital malformations in communities near the Hanford site. Am J Epidemiol. 1988; 127(2): 243-54

Shah GS, Singh MK, Pandey TR, Kalakheti BK, Bhandari GP. Incidence of congenital heart disease in tertiary care hospital. Kathmandu Univ Med J (KUMJ). 2008; 6(1): 33-6

Sheehan A, Ward OC, Duff DF, Denham B, Neligan M, Wood A. Cardiac surgery in Down syndrome. Ir Med J. 1990; 83(2): 67-9

Siffel C, Riehle-Colarusso T, Oster ME, Correa A. Survival of Children With Hypoplastic Left Heart Syndrome. Pediatrics. 2015; 136(4): e864-70

Singh S, Chukwunyere DN, Omembelede J, Onankpa B. Foetal congenital anomalies: An experience from a tertiary health institution in north-west Nigeria (2011-2013). Niger Postgrad Med J. 2015; 22(3): 174-8

Sklansky M, Shaughnessy R, Lucas V, Kashani I, Rothman A. A comparison of fetal echocardiography in university and health maintenance organization settings. Pediatr Cardiol. 2000; 21(3): 234-9

Slovakia - Slovak Republic Congenital Malformations Monitoring Program Data 2003 - ICBDSR as it appears in International Clearinghouse for Birth Defects Surveillance and Research. International Clearinghouse for Birth Defects Surveillance and Research Annual Report 2005. Rome, Italy: International Clearinghouse for Birth Defects Surveillance and Research, 2007

Slovakia - Slovak Republic Congenital Malformations Monitoring Program Data 2004 - ICBDSR as it appears in International Clearinghouse for Birth Defects Surveillance and Research. International Clearinghouse for Birth Defects Surveillance and Research Annual Report 2006. Rome, Italy: International Clearinghouse for Birth Defects Surveillance and Research, 2007

Slovakia - Slovak Republic Congenital Malformations Monitoring Program Data 2005 - ICBDSR as it appears in International Clearinghouse for Birth Defects Surveillance and Research. International Clearinghouse for Birth Defects Surveillance and Research Annual Report 2007. Rome, Italy: International Clearinghouse for Birth Defects Surveillance and Research, 2008

Slovakia - Slovak Republic Teratologic Information Centre, Slovak Medical University Congenital Anomaly Data 2012 - ICBDSR as it appears in International Clearinghouse for Birth Defects Surveillance and Research. International Clearinghouse for Birth Defects Surveillance and Research Annual Report 2014. Rome, Italy: International Clearinghouse for Birth Defects Surveillance and Research, 2016

Slovakia - Slovak Teratologic Information Centre, Slovak Medical University Data 2007 - ICBDSR as it appears in International Clearinghouse for Birth Defects Surveillance and Research. International Clearinghouse for Birth Defects Surveillance and Research Annual Report 2009. Rome, Italy: International Clearinghouse for Birth Defects Surveillance and Research

Slovakia - Slovak Teratologic Information Centre, Slovak Medical University Data 2008 - ICBDSR as it appears in International Clearinghouse for Birth Defects Surveillance and Research. International Clearinghouse for Birth Defects Surveillance and Research Annual Report 2010. Rome, Italy: International Clearinghouse for Birth Defects Surveillance and Research, 2011

Slovakia - Slovak Teratologic Information Centre, Slovak Medical University Data 2009 - ICBDSR as it appears in International Clearinghouse for Birth Defects Surveillance and Research. International Clearinghouse for Birth Defects Surveillance and Research Annual Report 2011. Rome, Italy: International Clearinghouse for Birth Defects Surveillance and Research, 2012

Slovakia - Slovak Teratologic Information Centre, Slovak Medical University Data 2010 - ICBDSR as it appears in International Clearinghouse for Birth Defects Surveillance and Research. International Clearinghouse for Birth Defects Surveillance and Research Annual Report 2012. Rome, Italy: International Clearinghouse for Birth Defects Surveillance and Research, 2013

Song MS, Hu A, Dyamenahalli U, Dyhamenahali U, Chitayat D, Winsor EJT, Ryan G, Smallhorn J, Barrett J, Yoo S-J, Hornberger LK. Extracardiac lesions and chromosomal abnormalities associated with major fetal heart defects: comparison of intrauterine, postnatal and postmortem diagnoses. Ultrasound Obstet Gynecol. 2009; 33(5): 552-9

South African Birth Defects Surveillance Systems Data 2001 - ICBDMS as it appears in International Clearinghouse for Birth Defects Monitoring Systems. International Clearinghouse for Birth Defects Monitoring Systems Annual Report 2003. Rome, Italy: International Centre on Birth Defects

South African Birth Defects Surveillance Systems Data 2002 - ICBDMS as it appears in International Clearinghouse for Birth Defects Monitoring Systems. International Clearinghouse for Birth Defects Monitoring Systems Annual Report 2004. Rome, Italy: International Centre on Birth Defects, 2006
South African Birth Defects Surveillance Systems Data 2003 - ICBDSR as it appears in International Clearinghouse for Birth Defects Surveillance and Research. International Clearinghouse for Birth Defects Surveillance and Research Annual Report 2005. Rome, Italy: International Clearinghouse for Birth Defects Surveillance and Research, 2007
Spain - Asturias Congenital Anomaly Data 1993-1998 - WHO as it appears in European Surveillance of Congenital Anomalies (EUROCAT), International Centre on Birth Defects, World Health Organization (WHO). World Atlas of Birth Defects. 2nd ed. Geneva, Switzerland: World Health Organization (WHO), 2003
Spain - Barcelona Birth Defects Registry Data 1993-1998 - WHO as it appears in European Surveillance of Congenital Anomalies (EUROCAT), International Centre on Birth Defects, World Health Organization (WHO). World Atlas of Birth Defects. 2nd ed. Geneva, Switzerland: World Health Organization (WHO), 2003
Spain - Basque Country Registry of Congenital Anomalies Data 1993-1997 - WHO as it appears in European Surveillance of Congenital Anomalies (EUROCAT), International Centre on Birth Defects, World Health Organization (WHO). World Atlas of Birth Defects. 2nd ed. Geneva, Switzerland: World Health Organization (WHO), 2003
Spain - El Valles Congenital Anomaly Data 1993-1997 - WHO as it appears in European Surveillance of Congenital Anomalies (EUROCAT), International Centre on Birth Defects, World Health Organization (WHO). World Atlas of Birth Defects. 2nd ed. Geneva, Switzerland: World Health Organization (WHO), 2003
Spain Collaborative Study of Congenital Malformations Data 2012 - ICBDSR as it appears in International Clearinghouse for Birth Defects Surveillance and Research. International Clearinghouse for Birth Defects Surveillance and Research Annual Report 2014. Rome, Italy: International Clearinghouse for Birth Defects Surveillance and Research, 2016
Spanish Collaborative Study of Congenital Malformations Data 1993-1998 - WHO as it appears in European Surveillance of Congenital Anomalies (EUROCAT), International Centre on Birth Defects, World Health Organization (WHO). World Atlas of Birth Defects. 2nd ed. Geneva, Switzerland: World Health Organization (WHO), 2003
Spanish Collaborative Study of Congenital Malformations Data 2001 - ICBDMS as it appears in International Clearinghouse for Birth Defects Monitoring Systems. International Clearinghouse for Birth Defects Monitoring Systems Annual Report 2003. Rome, Italy: International Centre on Birth Defects
Spanish Collaborative Study of Congenital Malformations Data 2002 - ICBDMS as it appears in International Clearinghouse for Birth Defects Monitoring Systems. International Clearinghouse for Birth Defects Monitoring Systems Annual Report 2004. Rome, Italy: International Centre on Birth Defects, 2006
Spanish Collaborative Study of Congenital Malformations Data 2003 - ICBDSR as it appears in International Clearinghouse for Birth Defects Surveillance and Research. International Clearinghouse for Birth Defects Surveillance and Research Annual Report 2005. Rome, Italy: International Clearinghouse for Birth Defects Surveillance and Research, 2007
Spanish Collaborative Study of Congenital Malformations Data 2004 - ICBDSR as it appears in International Clearinghouse for Birth Defects Surveillance and Research. International Clearinghouse for Birth Defects Surveillance and Research Annual Report 2006. Rome, Italy: International Clearinghouse for Birth Defects Surveillance and Research, 2007
Spanish Collaborative Study of Congenital Malformations Data 2005 - ICBDSR as it appears in International Clearinghouse for Birth Defects Surveillance and Research. International Clearinghouse for Birth Defects Surveillance and Research Annual Report 2007. Rome, Italy: International Clearinghouse for Birth Defects Surveillance and Research, 2008

Spanish Collaborative Study of Congenital Malformations Data 2007 - ICBDSR as it appears in International Clearinghouse for Birth Defects Surveillance and Research. International Clearinghouse for Birth Defects Surveillance and Research Annual Report 2009. Rome, Italy: International Clearinghouse for Birth Defects Surveillance and Research
Spanish Collaborative Study of Congenital Malformations Data 2008 - ICBDSR as it appears in International Clearinghouse for Birth Defects Surveillance and Research. International Clearinghouse for Birth Defects Surveillance and Research Annual Report 2010. Rome, Italy: International Clearinghouse for Birth Defects Surveillance and Research, 2011
Spanish Collaborative Study of Congenital Malformations Data 2009 - ICBDSR as it appears in International Clearinghouse for Birth Defects Surveillance and Research. International Clearinghouse for Birth Defects Surveillance and Research Annual Report 2011. Rome, Italy: International Clearinghouse for Birth Defects Surveillance and Research, 2012
Spanish Collaborative Study of Congenital Malformations Data 2010 - ICBDSR as it appears in International Clearinghouse for Birth Defects Surveillance and Research. International Clearinghouse for Birth Defects Surveillance and Research Annual Report 2012. Rome, Italy: International Clearinghouse for Birth Defects Surveillance and Research, 2013
Statistical Institute of Jamaica. Jamaica Survey of Living Conditions 1990. Washington DC, United States: World Bank
Statistics Austria, World Health Organization (WHO). Austria WHO Multi-country Survey Study on Health and Health System Responsiveness 2000-2001. Geneva, Switzerland: World Health Organization (WHO)
Statistics Canada. Canada Community Health Survey 2000-2001. Ottawa, Canada: Statistics Canada, 2003
Statistics Canada. Canada Community Health Survey 2005. Ottawa, Canada: Statistics Canada
Statistics Canada. Canada Community Health Survey 2007-2008. Ottawa, Canada: Statistics Canada, 2009
Statistics Denmark, World Health Organization (WHO). Denmark WHO Multi-country Survey Study on Health and Health System Responsiveness 2000-2001. Geneva, Switzerland: World Health Organization (WHO)
Statistics Indonesia. Indonesia National Socioeconomic Survey - Poverty Program Evaluation 2006. Jakarta, Indonesia: Statistics Indonesia
Statistics Indonesia. Indonesia National Socioeconomic Survey - Poverty Program Evaluation 2008-2009. Jakarta, Indonesia: Statistics Indonesia
Statistics Indonesia. Indonesia National Socioeconomic Survey 2002
Statistics Indonesia. Indonesia National Socioeconomic Survey 2004
Statistics Indonesia. Indonesia National Socioeconomic Survey 2005
Statistics Indonesia. Indonesia National Socioeconomic Survey 2008
Statistics Indonesia. Indonesia National Socioeconomic Survey 2010
Statistics Indonesia. Indonesia National Socioeconomic Survey 2011
Statistics Portugal. Portugal Hospital Inpatient Discharges 1985
Statistics Portugal. Portugal Hospital Inpatient Discharges 1986
Statistics Portugal. Portugal Hospital Inpatient Discharges 1987
Statistics Portugal. Portugal Hospital Inpatient Discharges 1988
Statistics Portugal. Portugal Hospital Inpatient Discharges 1989
Statistics Portugal. Portugal Hospital Inpatient Discharges 1990
Statistics Portugal. Portugal Hospital Inpatient Discharges 1991

Statistics Portugal. Portugal Hospital Inpatient Discharges 1992
Statistics Portugal. Portugal Hospital Inpatient Discharges 1993
Statistics Portugal. Portugal Hospital Inpatient Discharges 1994
Statistics Portugal. Portugal Hospital Inpatient Discharges 1995
Statistics Portugal. Portugal Hospital Inpatient Discharges 1996
Statistics Portugal. Portugal Hospital Inpatient Discharges 1997
Statistics Portugal. Portugal Hospital Inpatient Discharges 1998
Statistics Portugal. Portugal Hospital Inpatient Discharges 1999
Statistics Portugal. Portugal Hospital Inpatient Discharges 2000
Statistics Portugal. Portugal Hospital Inpatient Discharges 2001
Statistics Portugal. Portugal Hospital Inpatient Discharges 2002
Statistics Portugal. Portugal Hospital Inpatient Discharges 2003
Statistics Portugal. Portugal Hospital Inpatient Discharges 2004
Statistics Portugal. Portugal Hospital Inpatient Discharges 2005
Statistics Portugal. Portugal Hospital Inpatient Discharges 2006
Statistics Portugal. Portugal Hospital Inpatient Discharges 2007
Statistics Portugal. Portugal Hospital Inpatient Discharges 2008
Statistics Portugal. Portugal Hospital Inpatient Discharges 2009
Statistics Portugal. Portugal Hospital Inpatient Discharges 2010
Statistics Portugal. Portugal Hospital Inpatient Discharges 2011
Statistics Portugal. Portugal Hospital Inpatient Discharges 2012
Statistics Portugal. Portugal Hospital Inpatient Discharges 2013
Statistics Portugal. Portugal Hospital Inpatient Discharges 2014
Statistics Sweden, Swedish National Institute of Public Health. Sweden National Survey of Public Health 2004
Statistics Sweden, Swedish National Institute of Public Health. Sweden National Survey of Public Health 2005
Statistics Sweden, Swedish National Institute of Public Health. Sweden National Survey of Public Health 2007
Statistics Sweden, Swedish National Institute of Public Health. Sweden National Survey of Public Health 2008
Statistics Sweden, Swedish National Institute of Public Health. Sweden National Survey of Public Health 2009
Statistics Sweden, Swedish National Institute of Public Health. Sweden National Survey of Public Health 2010
Statistics Sweden, Swedish National Institute of Public Health. Sweden National Survey of Public Health 2011
Statistics Sweden, Swedish National Institute of Public Health. Sweden National Survey of Public Health 2012
Statistics Sweden, Swedish National Institute of Public Health. Sweden National Survey of Public Health 2013
Stephensen SS, Sigfusson G, Eiriksson H, Sverrisson JT, Torfason B, Haraldsson A, Helgason H. Congenital cardiac malformations in Iceland from 1990 through 1999. Cardiol Young. 2004; 14(4): 396-401

Storch TG, Mannick EE. Epidemiology of congenital heart disease in Louisiana: an association between race and sex and the prevalence of specific cardiac malformations. Teratology. 1992; 46(3): 271-6

Subramanyan R, Joy J, Venugopalan P, Sapru A, al Khusaiby SM. Incidence and spectrum of congenital heart disease in Oman. Ann Trop Paediatr. 2000; 20(4): 337-41

Sumanovic-Glamuzina D, Saraga-Karacic V, Roncevic Z, Milanov A, Bozic T, Boranic M. Incidence of major congenital malformations in a region of Bosnia and Herzegovina allegedly polluted with depleted uranium. Croat Med J. 2003; 44(5): 579-84

Sun G, Xu Z-M, Liang J-F, Li L, Tang D-X. Twelve-year prevalence of common neonatal congenital malformations in Zhejiang Province, China. World J Pediatr. 2011; 7(4): 331-6

Sung RY, So LY, Ng HK, Ho JK, Fok TF. Echocardiography as a tool for determining the incidence of congenital heart disease in newborn babies: a pilot study in Hong Kong. Int J Cardiol. 1991; 30(1): 43-7

Swanson TM, Selamet Tierney ES, Tworetzky W, Pigula F, McElhinney DB. Truncus arteriosus: diagnostic accuracy, outcomes, and impact of prenatal diagnosis. Pediatr Cardiol. 2009; 30(3): 256-61

Swedish Birth Defects Register Data 2005 - ICBDSR as it appears in International Clearinghouse for Birth Defects Surveillance and Research. International Clearinghouse for Birth Defects Surveillance and Research Annual Report 2007. Rome, Italy: International Clearinghouse for Birth Defects Surveillance and Research, 2008

Swedish Registry of Congenital Malformations and Medical Birth Registry Data 2001 - ICBDMS as it appears in International Clearinghouse for Birth Defects Monitoring Systems. International Clearinghouse for Birth Defects Monitoring Systems Annual Report 2003. Rome, Italy: International Centre on Birth Defects

Swedish Registry of Congenital Malformations and Medical Birth Registry Data 2002 - ICBDMS as it appears in International Clearinghouse for Birth Defects Monitoring Systems. International Clearinghouse for Birth Defects Monitoring Systems Annual Report 2004. Rome, Italy: International Centre on Birth Defects, 2006

Swedish Registry of Congenital Malformations and Medical Birth Registry Data 2003 - ICBDSR as it appears in International Clearinghouse for Birth Defects Surveillance and Research. International Clearinghouse for Birth Defects Surveillance and Research Annual Report 2005. Rome, Italy: International Clearinghouse for Birth Defects Surveillance and Research, 2007

Swedish Registry of Congenital Malformations and Medical Birth Registry Data 2004 - ICBDSR as it appears in International Clearinghouse for Birth Defects Surveillance and Research. International Clearinghouse for Birth Defects Surveillance and Research Annual Report 2006. Rome, Italy: International Clearinghouse for Birth Defects Surveillance and Research, 2007

Swedish Registry of Congenital Malformations and Medical Birth Registry Data 2008 - ICBDSR as it appears in International Clearinghouse for Birth Defects Surveillance and Research. International Clearinghouse for Birth Defects Surveillance and Research Annual Report 2010. Rome, Italy: International Clearinghouse for Birth Defects Surveillance and Research, 2011

Swedish Registry of Congenital Malformations and Medical Birth Registry Data 2009 - ICBDSR as it appears in International Clearinghouse for Birth Defects Surveillance and Research. International Clearinghouse for Birth Defects Surveillance and Research Annual Report 2011. Rome, Italy: International Clearinghouse for Birth Defects Surveillance and Research, 2012

Swedish Registry of Congenital Malformations and Medical Birth Registry Data 2010 - ICBDSR as it appears in International Clearinghouse for Birth Defects Surveillance and Research. International Clearinghouse for Birth Defects Surveillance and Research Annual Report 2012. Rome, Italy: International Clearinghouse for Birth Defects Surveillance and Research, 2013

Swedish Registry of Congenital Malformations and Medical Birth Registry. Data 2007 - ICBDSR as it appears in International Clearinghouse for Birth Defects Surveillance and Research. International Clearinghouse for Birth Defects Surveillance and Research Annual Report 2009. Rome, Italy: International Clearinghouse for Birth Defects Surveillance and Research

Switzerland - Zurich Registry of Switzerland Data 1993-1998 - WHO as it appears in European Surveillance of Congenital Anomalies (EUROCAT), International Centre on Birth Defects, World Health Organization (WHO). World Atlas of Birth Defects. 2nd ed. Geneva, Switzerland: World Health Organization (WHO), 2003

Szonda Ipsos, World Health Organization (WHO). Hungary WHO Multi-country Survey Study on Health and Health System Responsiveness 2000-2001. Geneva, Switzerland: World Health Organization (WHO)

Tan KH, Tan TY, Tan J, Tan I, Chew SK, Yeo GS. Birth defects in Singapore: 1994-2000. Singapore Med J. 2005; 46(10): 545-52

Thein MM, Koh D, Tan KL, Lee HP, Yip YY, Tye CY, Phoon WO. Descriptive profile of birth defects among livebirths in Singapore. Teratology. 1992; 46(3): 277-84

TNS BBSS, World Bank. Bulgaria Living Standards Measurement Survey 2001. Washington DC, United States: World Bank

Tomatir AG, Demirhan H, Sorkun HC, Koksal A, Ozerdem F, Cilengir N. Major congenital anomalies: a five-year retrospective regional study in Turkey. Genet Mol Res. 2009; 8(1): 19-27

TQA Research, World Health Organization (WHO). Australia WHO Multi-country Survey Study on Health and Health System Responsiveness 2000-2001. Geneva, Switzerland: World Health Organization (WHO)

Trinity College Dublin. Ireland Longitudinal Study on Ageing 2009-2011. Dublin, Ireland: Irish Social Science Data Archive, University College Dublin

Trinity College Dublin. Ireland Longitudinal Study on Ageing 2012-2013. Dublin, Ireland: Irish Social Science Data Archive, University College Dublin

Truven Health Analytics. United States MarketScan Claims and Medicare Data - 2000. Ann Arbor, United States: Truven Health Analytics

Truven Health Analytics. United States MarketScan Claims and Medicare Data - 2010. Ann Arbor, United States: Truven Health Analytics

Truven Health Analytics. United States MarketScan Claims and Medicare Data - 2012. Ann Arbor, United States: Truven Health Analytics

Truven Health Analytics. United States MarketScan Claims and Medicare Data 2011. Ann Arbor, United States: Truven Health Analytics

Truven Health Analytics. United States MarketScan Claims and Medicare Data 2013. Ann Arbor, United States: Truven Health Analytics

Truven Health Analytics. United States MarketScan Claims and Medicare Data 2014. Ann Arbor, United States: Truven Health Analytics

Tsuchihashi M, Tsutsui H, Kodama K, Kasagi F, Takeshita A. Clinical characteristics and prognosis of hospitalized patients with congestive heart failure--a study in Fukuoka, Japan. Jpn Circ J. 2000; 64(12): 953-9

Turkish Statistical Institute. Turkey Health Interview Survey 2010. Ankara, Turkey: Turkish Statistical Institute

Ukraine - Northwest Ukraine OMNI-Net Birth Defects Program Data 2005 - ICBDSR as it appears in International Clearinghouse for Birth Defects Surveillance and Research. International Clearinghouse for Birth Defects Surveillance and Research Annual Report 2007. Rome, Italy: International Clearinghouse for Birth Defects Surveillance and Research, 2008

Ukraine - Northwest Ukraine OMNI-Net Birth Defects Program Data 2007 - ICBDSR as it appears in International Clearinghouse for Birth Defects Surveillance and Research. International Clearinghouse for Birth Defects Surveillance and Research Annual Report 2009. Rome, Italy: International Clearinghouse for Birth Defects Surveillance and Research

Ukraine - Rivne and Volyn OMNI-Net Birth Defects Program Data 2008 - ICBDSR as it appears in International Clearinghouse for Birth Defects Surveillance and Research. International Clearinghouse for Birth Defects Surveillance and Research Annual Report 2010. Rome, Italy: International Clearinghouse for Birth Defects Surveillance and Research, 2011

Ukraine - Rivne and Volyn OMNI-Net Birth Defects Program Data 2009 - ICBDSR as it appears in International Clearinghouse for Birth Defects Surveillance and Research. International Clearinghouse for Birth Defects Surveillance and Research Annual Report 2011. Rome, Italy: International Clearinghouse for Birth Defects Surveillance and Research, 2012

Ukraine - Rivne and Volyn OMNI-Net Birth Defects Program Data 2010 - ICBDSR as it appears in International Clearinghouse for Birth Defects Surveillance and Research. International Clearinghouse for Birth Defects Surveillance and Research Annual Report 2012. Rome, Italy: International Clearinghouse for Birth Defects Surveillance and Research, 2013
Ukraine - Rivne and Volyn OMNI-Net Birth Defects Program Data 2012 - ICBDSR as it appears in International Clearinghouse for Birth Defects Surveillance and Research. International Clearinghouse for Birth Defects Surveillance and Research Annual Report 2014. Rome, Italy: International Clearinghouse for Birth Defects Surveillance and Research, 2016
Ukraine - Ukrainian-American Birth Defects Program Data 2001 - ICBDMS as it appears in International Clearinghouse for Birth Defects Monitoring Systems. International Clearinghouse for Birth Defects Monitoring Systems Annual Report 2003. Rome, Italy: International Centre on Birth Defects
Ukraine - Ukrainian-American Birth Defects Program Data 2002 - ICBDMS as it appears in International Clearinghouse for Birth Defects Monitoring Systems. International Clearinghouse for Birth Defects Monitoring Systems Annual Report 2004. Rome, Italy: International Centre on Birth Defects, 2006
Ukraine - Ukrainian-American Birth Defects Program Data 2003 - ICBDSR as it appears in International Clearinghouse for Birth Defects Surveillance and Research. International Clearinghouse for Birth Defects Surveillance and Research Annual Report 2005. Rome, Italy: International Clearinghouse for Birth Defects Surveillance and Research, 2007
Ukraine - Ukrainian-American Birth Defects Program Data 2004 - ICBDSR as it appears in International Clearinghouse for Birth Defects Surveillance and Research. International Clearinghouse for Birth Defects Surveillance and Research Annual Report 2006. Rome, Italy: International Clearinghouse for Birth Defects Surveillance and Research, 2007
United Arab Emirates - Al Ain Medical District Congenital Abnormality Study Group Data 1996-1998 - WHO as it appears in European Surveillance of Congenital Anomalies (EUROCAT), International Centre on Birth Defects, World Health Organization (WHO). World Atlas of Birth Defects. 2nd ed. Geneva, Switzerland: World Health Organization (WHO), 2003
United Arab Emirates - Al Ain Medical District Congenital Abnormality Study Group Data 2001 - ICBDMS as it appears in International Clearinghouse for Birth Defects Monitoring Systems. International Clearinghouse for Birth Defects Monitoring Systems Annual Report 2003. Rome, Italy: International Centre on Birth Defects
United Arab Emirates - Al Ain Medical District Congenital Abnormality Study Group Data 2002 - ICBDMS as it appears in International Clearinghouse for Birth Defects Monitoring Systems. International Clearinghouse for Birth Defects Monitoring Systems Annual Report 2004. Rome, Italy: International Centre on Birth Defects, 2006
United Arab Emirates - Al Ain Medical District Congenital Abnormality Study Group Data 2003 - ICBDSR as it appears in International Clearinghouse for Birth Defects Surveillance and Research. International Clearinghouse for Birth Defects Surveillance and Research Annual Report 2005. Rome, Italy: International Clearinghouse for Birth Defects Surveillance and Research, 2007
United Kingdom - England and Wales National Congenital Anomaly System Data 1993-1998 - WHO as it appears in European Surveillance of Congenital Anomalies (EUROCAT), International Centre on Birth Defects, World Health Organization (WHO). World Atlas of Birth Defects. 2nd ed. Geneva, Switzerland: World Health Organization (WHO), 2003
United Kingdom - England and Wales National Congenital Anomaly System Data 2001 - ICBDMS as it appears in International Clearinghouse for Birth Defects Monitoring Systems. International Clearinghouse for Birth Defects Monitoring Systems Annual Report 2003. Rome, Italy: International Centre on Birth Defects
United Kingdom - England and Wales National Congenital Anomaly System Data 2002 - ICBDMS as it appears in International Clearinghouse for Birth Defects Monitoring Systems. International Clearinghouse for Birth Defects Monitoring Systems Annual Report 2004. Rome, Italy: International Centre on Birth Defects, 2006

United Kingdom - England and Wales National Congenital Anomaly System Data 2003 - ICBDSR as it appears in International Clearinghouse for Birth Defects Surveillance and Research. International Clearinghouse for Birth Defects Surveillance and Research Annual Report 2005. Rome, Italy: International Clearinghouse for Birth Defects Surveillance and Research, 2007

United Kingdom - England and Wales National Congenital Anomaly System Data 2005 - ICBDSR as it appears in International Clearinghouse for Birth Defects Surveillance and Research. International Clearinghouse for Birth Defects Surveillance and Research Annual Report 2007. Rome, Italy: International Clearinghouse for Birth Defects Surveillance and Research, 2008

United Kingdom - Mersey Congenital Anomaly Survey Data 1998 - WHO as it appears in European Surveillance of Congenital Anomalies (EUROCAT), International Centre on Birth Defects, World Health Organization (WHO). World Atlas of Birth Defects. 2nd ed. Geneva, Switzerland: World Health Organization (WHO), 2003

United Kingdom - North Thames (West) Congenital Malformation Register Data 1997-1998 - WHO as it appears in European Surveillance of Congenital Anomalies (EUROCAT), International Centre on Birth Defects, World Health Organization (WHO). World Atlas of Birth Defects. 2nd ed. Geneva, Switzerland: World Health Organization (WHO), 2003

United Kingdom - Wales Congenital Anomaly Register and Information Service Data 2004 - ICBDSR as it appears in International Clearinghouse for Birth Defects Surveillance and Research. International Clearinghouse for Birth Defects Surveillance and Research Annual Report 2006. Rome, Italy: International Clearinghouse for Birth Defects Surveillance and Research, 2007

United Kingdom - Wales Congenital Anomaly Register and Information Service Data 2007 - ICBDSR as it appears in International Clearinghouse for Birth Defects Surveillance and Research. International Clearinghouse for Birth Defects Surveillance and Research Annual Report 2009. Rome, Italy: International Clearinghouse for Birth Defects Surveillance and Research

United Kingdom - Wales Congenital Anomaly Register and Information Service Data 2008 - ICBDSR as it appears in International Clearinghouse for Birth Defects Surveillance and Research. International Clearinghouse for Birth Defects Surveillance and Research Annual Report 2010. Rome, Italy: International Clearinghouse for Birth Defects Surveillance and Research, 2011

United Kingdom - Wales Congenital Anomaly Register and Information System Data 2009 - ICBDSR as it appears in International Clearinghouse for Birth Defects Surveillance and Research. International Clearinghouse for Birth Defects Surveillance and Research Annual Report 2011. Rome, Italy: International Clearinghouse for Birth Defects Surveillance and Research, 2012

United Kingdom - Wales Congenital Anomaly Register and Information System Data 2010 - ICBDSR as it appears in International Clearinghouse for Birth Defects Surveillance and Research. International Clearinghouse for Birth Defects Surveillance and Research Annual Report 2012. Rome, Italy: International Clearinghouse for Birth Defects Surveillance and Research, 2013

United Kingdom - Wales Congenital Anomaly Register and Information System Data 2012 - ICBDSR as it appears in International Clearinghouse for Birth Defects Surveillance and Research. International Clearinghouse for Birth Defects Surveillance and Research Annual Report 2014. Rome, Italy: International Clearinghouse for Birth Defects Surveillance and Research, 2016

United Kingdom - Wessex Antenatally Detected Anomalies Register Data 2007 - ICBDSR as it appears in International Clearinghouse for Birth Defects Surveillance and Research. International Clearinghouse for Birth Defects Surveillance and Research Annual Report 2009. Rome, Italy: International Clearinghouse for Birth Defects Surveillance and Research

United Kingdom - Wessex Antenatally Detected Anomalies Register Data 2008 - ICBDSR as it appears in International Clearinghouse for Birth Defects Surveillance and Research. International Clearinghouse for Birth Defects Surveillance and Research Annual Report 2010. Rome, Italy: International Clearinghouse for Birth Defects Surveillance and Research, 2011

United States - Arkansas Reproductive Health Monitoring System Data 2012 - ICBDSR as it appears in International Clearinghouse for Birth Defects Surveillance and Research. International Clearinghouse for Birth Defects Surveillance and Research Annual Report 2014. Rome, Italy: International Clearinghouse for Birth Defects Surveillance and Research, 2016
United States - Georgia Metropolitan Atlanta Congenital Defects Program Data 1993-1998 - WHO as it appears in European Surveillance of Congenital Anomalies (EUROCAT), International Centre on Birth Defects, World Health Organization (WHO). World Atlas of Birth Defects. 2nd ed. Geneva, Switzerland: World Health Organization (WHO), 2003
United States - Georgia Metropolitan Atlanta Congenital Defects Program Data 2001 - ICBDMS as it appears in International Clearinghouse for Birth Defects Monitoring Systems. International Clearinghouse for Birth Defects Monitoring Systems Annual Report 2003. Rome, Italy: International Centre on Birth Defects
United States - Georgia Metropolitan Atlanta Congenital Defects Program Data 2002 - ICBDMS as it appears in International Clearinghouse for Birth Defects Monitoring Systems. International Clearinghouse for Birth Defects Monitoring Systems Annual Report 2004. Rome, Italy: International Centre on Birth Defects, 2006
United States - Georgia Metropolitan Atlanta Congenital Defects Program Data 2003 - ICBDSR as it appears in International Clearinghouse for Birth Defects Surveillance and Research. International Clearinghouse for Birth Defects Surveillance and Research Annual Report 2005. Rome, Italy: International Clearinghouse for Birth Defects Surveillance and Research, 2007
United States - Georgia Metropolitan Atlanta Congenital Defects Program Data 2005 - ICBDSR as it appears in International Clearinghouse for Birth Defects Surveillance and Research. International Clearinghouse for Birth Defects Surveillance and Research Annual Report 2007. Rome, Italy: International Clearinghouse for Birth Defects Surveillance and Research, 2008
United States - Georgia Metropolitan Atlanta Congenital Defects Program Data 2007 - ICBDSR as it appears in International Clearinghouse for Birth Defects Surveillance and Research. International Clearinghouse for Birth Defects Surveillance and Research Annual Report 2009. Rome, Italy: International Clearinghouse for Birth Defects Surveillance and Research
United States - Georgia Metropolitan Atlanta Congenital Defects Program Data 2008 - ICBDSR as it appears in International Clearinghouse for Birth Defects Surveillance and Research. International Clearinghouse for Birth Defects Surveillance and Research Annual Report 2010. Rome, Italy: International Clearinghouse for Birth Defects Surveillance and Research, 2011
United States - Georgia Metropolitan Atlanta Congenital Defects Program Data 2009 - ICBDSR as it appears in International Clearinghouse for Birth Defects Surveillance and Research. International Clearinghouse for Birth Defects Surveillance and Research Annual Report 2011. Rome, Italy: International Clearinghouse for Birth Defects Surveillance and Research, 2012
United States - Georgia Metropolitan Atlanta Congenital Defects Program Data 2010 - ICBDSR as it appears in International Clearinghouse for Birth Defects Surveillance and Research. International Clearinghouse for Birth Defects Surveillance and Research Annual Report 2012. Rome, Italy: International Clearinghouse for Birth Defects Surveillance and Research, 2013
United States - Georgia Metropolitan Atlanta Congenital Defects Program Data 2012 - ICBDSR as it appears in International Clearinghouse for Birth Defects Surveillance and Research. International Clearinghouse for Birth Defects Surveillance and Research Annual Report 2014. Rome, Italy: International Clearinghouse for Birth Defects Surveillance and Research, 2016
United States - Metropolitan Atlanta Congenital Defects Program Data 2004 - ICBDSR as it appears in International Clearinghouse for Birth Defects Surveillance and Research. International Clearinghouse for Birth Defects Surveillance and Research Annual Report 2006. Rome, Italy: International Clearinghouse for Birth Defects Surveillance and Research, 2007

United States - Texas Birth Defects Epidemiology & Surveillance Branch Data 2002 - ICBDSR as it appears in International Clearinghouse for Birth Defects Surveillance and Research. International Clearinghouse for Birth Defects Surveillance and Research Annual Report 2005. Rome, Italy: International Clearinghouse for Birth Defects Surveillance and Research, 2007

United States - Texas Birth Defects Epidemiology & Surveillance Branch Data 2008 - ICBDSR as it appears in International Clearinghouse for Birth Defects Surveillance and Research. International Clearinghouse for Birth Defects Surveillance and Research Annual Report 2010. Rome, Italy: International Clearinghouse for Birth Defects Surveillance and Research, 2011

United States - Texas Birth Defects Epidemiology & Surveillance Branch Data 2009 - ICBDSR as it appears in International Clearinghouse for Birth Defects Surveillance and Research. International Clearinghouse for Birth Defects Surveillance and Research Annual Report 2011. Rome, Italy: International Clearinghouse for Birth Defects Surveillance and Research, 2012

United States - Texas Birth Defects Epidemiology & Surveillance Branch Data 2011 - ICBDSR as it appears in International Clearinghouse for Birth Defects Surveillance and Research. International Clearinghouse for Birth Defects Surveillance and Research Annual Report 2014. Rome, Italy: International Clearinghouse for Birth Defects Surveillance and Research, 2016

United States - Texas Birth Defects Epidemiology and Surveillance Branch Data 2004 - ICBDSR as it appears in International Clearinghouse for Birth Defects Surveillance and Research. International Clearinghouse for Birth Defects Surveillance and Research Annual Report 2007. Rome, Italy: International Clearinghouse for Birth Defects Surveillance and Research, 2008

United States - Texas Birth Defects Epidemiology and Surveillance Branch Data 2006 - ICBDSR as it appears in International Clearinghouse for Birth Defects Surveillance and Research. International Clearinghouse for Birth Defects Surveillance and Research Annual Report 2009. Rome, Italy: International Clearinghouse for Birth Defects Surveillance and Research

United States - Texas Center for Birth Defects Research Data 2003 - ICBDSR as it appears in International Clearinghouse for Birth Defects Surveillance and Research. International Clearinghouse for Birth Defects Surveillance and Research Annual Report 2006. Rome, Italy: International Clearinghouse for Birth Defects Surveillance and Research, 2007

United States - Utah Birth Defects Network Data 2004 - ICBDSR as it appears in International Clearinghouse for Birth Defects Surveillance and Research. International Clearinghouse for Birth Defects Surveillance and Research Annual Report 2006. Rome, Italy: International Clearinghouse for Birth Defects Surveillance and Research, 2007

United States - Utah Birth Defects Network Data 2005 - ICBDSR as it appears in International Clearinghouse for Birth Defects Surveillance and Research. International Clearinghouse for Birth Defects Surveillance and Research Annual Report 2007. Rome, Italy: International Clearinghouse for Birth Defects Surveillance and Research, 2008

United States - Utah Birth Defects Network Data 2007 - ICBDSR as it appears in International Clearinghouse for Birth Defects Surveillance and Research. International Clearinghouse for Birth Defects Surveillance and Research Annual Report 2009. Rome, Italy: International Clearinghouse for Birth Defects Surveillance and Research

United States - Utah Birth Defects Network Data 2008 - ICBDSR as it appears in International Clearinghouse for Birth Defects Surveillance and Research. International Clearinghouse for Birth Defects Surveillance and Research Annual Report 2010. Rome, Italy: International Clearinghouse for Birth Defects Surveillance and Research, 2011

United States - Utah Birth Defects Network Data 2009 - ICBDSR as it appears in International Clearinghouse for Birth Defects Surveillance and Research. International Clearinghouse for Birth Defects Surveillance and Research Annual Report 2011. Rome, Italy: International Clearinghouse for Birth Defects Surveillance and Research, 2012

United States - Utah Birth Defects Network Data 2010 - ICBDSR as it appears in International Clearinghouse for Birth Defects Surveillance and Research. International Clearinghouse for Birth Defects Surveillance and Research Annual Report 2012. Rome, Italy: International Clearinghouse for Birth Defects Surveillance and Research, 2013

United States - Utah Birth Defects Network Data 2012 - ICBDSR as it appears in International Clearinghouse for Birth Defects Surveillance and Research. International Clearinghouse for Birth Defects Surveillance and Research Annual Report 2014. Rome, Italy: International Clearinghouse for Birth Defects Surveillance and Research, 2016

United States Department of Health and Human Services. Centers for Disease Control and Prevention. National Center for Health Statistics. National Health Interview Survey, 1994: Second Longitudinal Study on Aging, Wave 2, 1997. ICPSR03526-v2. Ann Arbor, MI: Inter-university Consortium for Political and Social Research [distributor], 2007-03-01. http://doi.org/10.3886/ICPSR03526.v2

United States Department of Health and Human Services. Centers for Disease Control and Prevention. National Center for Health Statistics. National Health Interview Survey, 1994: Second Supplement on Aging. ICPSR02563-v3. Ann Arbor, MI: Inter-university Consortium for Political and Social Research [distributor], 2007-02-12. http://doi.org/10.3886/ICPSR02563.v3

University of Concepcion (Chile), World Health Organization (WHO). Chile WHO Multi-country Survey Study on Health and Health System Responsiveness 2000-2001. Geneva, Switzerland: World Health Organization (WHO)

University of Ibadan (Nigeria), World Health Organization (WHO). Nigeria WHO Multi-country Survey Study on Health and Health System Responsiveness 2000-2001

University of Otago (New Zealand), World Health Organization (WHO). New Zealand WHO Multi-country Survey Study on Health and Health System Responsiveness 2000-2001. Geneva, Switzerland: World Health Organization (WHO)

University of the West Indies, World Health Organization (WHO). Trinidad and Tobago WHO Multi-country Survey Study on Health and Health System Responsiveness 2000-2001. Geneva, Switzerland: World Health Organization (WHO)

Uruguay Latin American Collaborative Study of Congenital Malformations Data 1993-1998 - WHO as it appears in European Surveillance of Congenital Anomalies (EUROCAT), International Centre on Birth Defects, World Health Organization (WHO). World Atlas of Birth Defects. 2nd ed. Geneva, Switzerland: World Health Organization (WHO), 2003

V M Vashishtha, A Kalra, K Kalra, V K Jain. Prevalence of congenital heart disease in school children. Indian Pediatr. 1993; 30(11): 1337-40

Venezuela Latin American Collaborative Study of Congenital Malformations Data 1993-1998 - WHO as it appears in European Surveillance of Congenital Anomalies (EUROCAT), International Centre on Birth Defects, World Health Organization (WHO). World Atlas of Birth Defects. 2nd ed. Geneva, Switzerland: World Health Organization (WHO), 2003

Vobecky SJ, Williams WG, Trusler GA, Coles JG, Rebeyka IM, Smallhorn J, Burrows P, Gow R, Freedom RM. Survival analysis of infants under age 18 months presenting with tetralogy of Fallot. Ann Thorac Surg. 1993; 56(4): 944-50

Wang Y, Liu G, Canfield MA, Mai CT, Gilboa SM, Meyer RE, Anderka M, Copeland GE, Kucik JE, Nembhard WN, Kirby RS. Racial/ethnic differences in survival of United States children with birth defects: a population-based study. J Pediatr. 2015; 166(4): 819-26e1-2

Wang Y, Hu J, Druschel CM, Kirby RS. Twenty-five-year survival of children with birth defects in New York State: a population-based study. Birth Defects Res A Clin Mol Teratol. 2011; 91(12): 995-1003

Ward KE, Pryor RW, Matson JR, Razook JD, Thompson WM, Elkins RC. Delayed detection of coarctation in infancy: implications for timing of newborn follow-up. Pediatrics. 1990; 86(6): 972-6

Washington State University, World Health Organization (WHO). United States WHO Multi-country Survey Study on Health and Health System Responsiveness 2000-2001. Geneva, Switzerland: World Health Organization (WHO)

Welke KF, Shen I, Ungerleider RM. Current assessment of mortality rates in congenital cardiac surgery. Ann Thorac Surg. 2006; 82(1): 164-71

Wertaschnigg D, Manlhiot C, Jaeggi M, Seed M, Dragulescu A, Schwartz SM, van Arsdell G, Jaeggi ET. Contemporary Outcomes and Factors Associated With Mortality After a Fetal or Neonatal Diagnosis of Ebstein Anomaly and Tricuspid Valve Disease. Can J Cardiol. 2016; nan

Wilson NJ, Clarkson PM, Barratt-Boyes BG, Calder AL, Whitlock RM, Easthope RN, Neutze JM. Long-term outcome after the mustard repair for simple transposition of the great arteries. 28-year follow-up. J Am Coll Cardiol. 1998; 32(3): 758-65

World Health Organization (WHO). Austria World Health Survey 2003. Geneva, Switzerland: World Health Organization (WHO), 2005

World Health Organization (WHO). Bangladesh World Health Survey 2003. Geneva, Switzerland: World Health Organization (WHO), 2005

World Health Organization (WHO). Belgium World Health Survey 2002. Geneva, Switzerland: World Health Organization (WHO), 2005

World Health Organization (WHO). Bosnia and Herzegovina World Health Survey 2003. Geneva, Switzerland: World Health Organization (WHO), 2005

World Health Organization (WHO). Burkina Faso World Health Survey 2002-2003. Geneva, Switzerland: World Health Organization (WHO), 2005

World Health Organization (WHO). Chad World Health Survey 2003. Geneva, Switzerland: World Health Organization (WHO), 2005

World Health Organization (WHO). China World Health Survey 2002. Geneva, Switzerland: World Health Organization (WHO), 2005

World Health Organization (WHO). Comoros World Health Survey 2003. Geneva, Switzerland: World Health Organization (WHO), 2005

World Health Organization (WHO). Congo World Health Survey 2003. Geneva, Switzerland: World Health Organization (WHO), 2005

World Health Organization (WHO). Côte d'Ivoire World Health Survey 2003. Geneva, Switzerland: World Health Organization (WHO), 2005

World Health Organization (WHO). Croatia World Health Survey 2003. Geneva, Switzerland: World Health Organization (WHO), 2005

World Health Organization (WHO). Czech Republic World Health Survey 2002-2003. Geneva, Switzerland: World Health Organization (WHO), 2005

World Health Organization (WHO). Denmark World Health Survey 2003. Geneva, Switzerland: World Health Organization (WHO), 2005

World Health Organization (WHO). Dominican Republic World Health Survey 2003. Geneva, Switzerland: World Health Organization (WHO), 2005

World Health Organization (WHO). Ecuador World Health Survey 2003. Geneva, Switzerland: World Health Organization (WHO), 2005

World Health Organization (WHO). Estonia World Health Survey 2003. Geneva, Switzerland: World Health Organization (WHO), 2005

World Health Organization (WHO). Ethiopia World Health Survey 2003. Geneva, Switzerland: World Health Organization (WHO), 2005

World Health Organization (WHO). Finland World Health Survey 2004. Geneva, Switzerland: World Health Organization (WHO), 2005

World Health Organization (WHO). France World Health Survey 2003. Geneva, Switzerland: World Health Organization (WHO), 2005

World Health Organization (WHO). Georgia World Health Survey 2003. Geneva, Switzerland: World Health Organization (WHO), 2005

World Health Organization (WHO). Germany World Health Survey 2004. Geneva, Switzerland: World Health Organization (WHO), 2005

World Health Organization (WHO). Ghana World Health Survey 2003. Geneva, Switzerland: World Health Organization (WHO), 2005

World Health Organization (WHO). Greece World Health Survey 2003. Geneva, Switzerland: World Health Organization (WHO), 2005

World Health Organization (WHO). Guatemala World Health Survey 2003. Geneva, Switzerland: World Health Organization (WHO), 2005

World Health Organization (WHO). Hungary World Health Survey 2003. Geneva, Switzerland: World Health Organization (WHO), 2005

World Health Organization (WHO). Ireland World Health Survey 2003. Geneva, Switzerland: World Health Organization (WHO), 2005

World Health Organization (WHO). Israel World Health Survey 2003. Geneva, Switzerland: World Health Organization (WHO), 2005

World Health Organization (WHO). Italy World Health Survey 2003. Geneva, Switzerland: World Health Organization (WHO), 2005

World Health Organization (WHO). Kazakhstan World Health Survey 2002-2003. Geneva, Switzerland: World Health Organization (WHO), 2005
World Health Organization (WHO). Kenya World Health Survey 2004. Geneva, Switzerland: World Health Organization (WHO), 2005
World Health Organization (WHO). Laos World Health Survey 2003
World Health Organization (WHO). Latvia World Health Survey 2003. Geneva, Switzerland: World Health Organization (WHO), 2005
World Health Organization (WHO). Luxembourg World Health Survey 2003. Geneva, Switzerland: World Health Organization (WHO), 2005
World Health Organization (WHO). Malawi World Health Survey 2003. Geneva, Switzerland: World Health Organization (WHO), 2005
World Health Organization (WHO). Malaysia World Health Survey 2003. Geneva, Switzerland: World Health Organization (WHO), 2005
World Health Organization (WHO). Mali World Health Survey 2003. Geneva, Switzerland: World Health Organization (WHO), 2005
World Health Organization (WHO). Mauritania World Health Survey 2003. Geneva, Switzerland: World Health Organization (WHO), 2005
World Health Organization (WHO). Mauritius World Health Survey 2003. Geneva, Switzerland: World Health Organization (WHO), 2005
World Health Organization (WHO). Mexico World Health Survey 2002-2003. Geneva, Switzerland: World Health Organization (WHO), 2005
World Health Organization (WHO). Morocco World Health Survey 2003. Geneva, Switzerland: World Health Organization (WHO), 2005
World Health Organization (WHO). Myanmar World Health Survey 2003. Geneva, Switzerland: World Health Organization (WHO), 2005
World Health Organization (WHO). Namibia World Health Survey 2003. Geneva, Switzerland: World Health Organization (WHO), 2005
World Health Organization (WHO). Nepal World Health Survey 2003. Geneva, Switzerland: World Health Organization (WHO), 2005
World Health Organization (WHO). Netherlands World Health Survey 2004. Geneva, Switzerland: World Health Organization (WHO), 2005
World Health Organization (WHO). Norway World Health Survey 2003. Geneva, Switzerland: World Health Organization (WHO), 2005
World Health Organization (WHO). Pakistan World Health Survey 2003-2004. Geneva, Switzerland: World Health Organization (WHO), 2005
World Health Organization (WHO). Paraguay World Health Survey 2002-2003. Geneva, Switzerland: World Health Organization (WHO), 2005
World Health Organization (WHO). Philippines World Health Survey 2003. Geneva, Switzerland: World Health Organization (WHO), 2005
World Health Organization (WHO). Portugal World Health Survey 2003. Geneva, Switzerland: World Health Organization (WHO), 2006
World Health Organization (WHO). Russia World Health Survey 2003. Geneva, Switzerland: World Health Organization (WHO), 2005
World Health Organization (WHO). Senegal World Health Survey 2003. Geneva, Switzerland: World Health Organization (WHO), 2005
World Health Organization (WHO). Slovakia World Health Survey 2003. Geneva, Switzerland: World Health Organization (WHO), 2005
World Health Organization (WHO). Slovenia World Health Survey 2003. Geneva, Switzerland: World Health Organization (WHO), 2005
World Health Organization (WHO). Spain World Health Survey 2002-2003. Geneva, Switzerland: World Health Organization (WHO), 2005
World Health Organization (WHO). Sri Lanka World Health Survey 2003. Geneva, Switzerland: World Health Organization (WHO), 2005
World Health Organization (WHO). Swaziland World Health Survey 2003. Geneva, Switzerland: World Health Organization (WHO), 2005
World Health Organization (WHO). Sweden World Health Survey 2003. Geneva, Switzerland: World Health Organization (WHO), 2005
World Health Organization (WHO). Tunisia World Health Survey 2003. Geneva, Switzerland: World Health Organization (WHO), 2005
World Health Organization (WHO). Ukraine World Health Survey 2002-2003. Geneva, Switzerland: World Health Organization (WHO), 2005
World Health Organization (WHO). United Arab Emirates World Health Survey 2003. Geneva, Switzerland: World Health Organization (WHO), 2005
World Health Organization (WHO). United Kingdom World Health Survey 2004. Geneva, Switzerland: World Health Organization (WHO), 2005
World Health Organization (WHO). Uruguay World Health Survey 2002-2003. Geneva, Switzerland: World Health Organization (WHO), 2005
World Health Organization (WHO). Vietnam World Health Survey 2002-2003. Geneva, Switzerland: World Health Organization (WHO), 2005

World Health Organization (WHO). Zambia World Health Survey 2003. Geneva, Switzerland: World Health Organization (WHO), 2005

World Health Organization (WHO). Zimbabwe World Health Survey 2003. Geneva, Switzerland: World Health Organization (WHO), 2005

World Health Organization Regional Office for Europe (WHO/Europe). European Health for All Database - Inpatient Care Discharges Per 100. Copenhagen, Denmark: World Health Organization Regional Office for Europe (WHO/Europe)

World Health Organization Regional Office for Europe (WHO/Europe). European Hospital Morbidity Database, 2008. Copenhagen, Denmark: World Health Organization Regional Office for Europe (WHO/Europe)

Wu M-H, Chen H-C, Lu C-W, Wang J-K, Huang S-C, Huang S-K. Prevalence of Congenital Heart Disease at Live Birth in Taiwan. J Pediatr. 2010; 156(5): 782-5

Wu MH, Lu CW, Chen HC, Chiu SN, Kao FY, Huang SK. Arrhythmic burdens in patients with tetralogy of Fallot: a national database study. Heart Rhythm. 2015; 12(3): 604-9

Yagel S, Weissman A, Rotstein Z, Manor M, Hegesh J, Anteby E, Lipitz S, Achiron R. Congenital heart defects: natural course and in utero development. Circulation. 1997; 96(2): 550-5

Yeh SJ, Chen HC, Lu CW, Wang JK, Huang LM, Huang SC, Huang SK, Wu MH. National database study of survival of pediatric congenital heart disease patients in Taiwan. J Formos Med Assoc. 2015; 114(2): 159-63

Yu M, Ping Z, Zhang S, He Y, Dong R, Guo X. The survey of birth defects rate based on birth registration system. Chin Med J (Engl). 2015; 128(1): 7-14

Yu Z, Xi Y, Ding W, Han S, Cao L, Zhu C, Wang X, Guo X. Congenital heart disease in a Chinese hospital: pre- and postnatal detection, incidence, clinical characteristics and outcomes. Pediatr Int. 2011; 53(6): 1059-65

Zhao Q-M, Ma X-J, Jia B, Huang G-Y. Prevalence of congenital heart disease at live birth: an accurate assessment by echocardiographic screening. Acta Paediatr. 2013; 102(4): 397-402

Zheng J-Y, Tian H-T, Zhu Z-M, Li B, Han L, Jiang S-L, Chen Y, Li D-T, He J-C, Zhao Z, Cao Y, Qiu Y-G, Li T-C. Prevalence of symptomatic congenital heart disease in Tibetan school children. Am J Cardiol. 2013; 112(9): 1468-70

Zucker N, Rozin I, Levitas A, Zalzstein E. Clinical presentation, natural history, and outcome of patients with the absent pulmonary valve syndrome. Cardiol Young. 2004; 14(4): 402-8
